# Specific changes in mitochondrial lipidome alter mitochondrial proteome and increase the geroprotective efficiency of lithocholic acid in chronologically aging yeast

**DOI:** 10.18632/oncotarget.16766

**Published:** 2017-03-31

**Authors:** Anna Leonov, Anthony Arlia-Ciommo, Simon D. Bourque, Olivia Koupaki, Pavlo Kyryakov, Pam鼳la Dakik, M鼳lissa McAuley, Younes Medkour, Karamat Mohammad, Tamara Di Maulo, Vladimir I. Titorenko

**Affiliations:** ^1^ Department of Biology, Concordia University, Montreal, Quebec, Canada

**Keywords:** yeast, aging, mitochondria, mitochondrial lipidome, mitochondrial proteome, Gerotarget

## Abstract

We have previously found that exogenously added lithocholic acid delays yeast chronological aging. We demonstrated that lithocholic acid enters the yeast cell, is sorted to mitochondria, resides in both mitochondrial membranes, changes the relative concentrations of different membrane phospholipids, triggers changes in the concentrations of many mitochondrial proteins, and alters some key aspects of mitochondrial functionality. We hypothesized that the lithocholic acid-driven changes in mitochondrial lipidome may have a causal role in the remodeling of mitochondrial proteome, which may in turn alter the functional state of mitochondria to create a mitochondrial pattern that delays yeast chronological aging. Here, we test this hypothesis by investigating how the *ups1?*, *ups2?* and *psd1?* mutations that eliminate enzymes involved in mitochondrial phospholipid metabolism influence the mitochondrial lipidome. We also assessed how these mutations affect the mitochondrial proteome, influence mitochondrial functionality and impinge on the efficiency of aging delay by lithocholic acid. Our findings provide evidence that 1) lithocholic acid initially creates a distinct pro-longevity pattern of mitochondrial lipidome by proportionally decreasing phosphatidylethanolamine and cardiolipin concentrations to maintain equimolar concentrations of these phospholipids, and by increasing phosphatidic acid concentration; 2) this pattern of mitochondrial lipidome allows to establish a specific, aging-delaying pattern of mitochondrial proteome; and 3) this pattern of mitochondrial proteome plays an essential role in creating a distinctive, geroprotective pattern of mitochondrial functionality.

## INTRODUCTION

An age-related deterioration of mitochondrial functionality is the universal hallmark of aging in eukaryotes across phyla [1]. This age-related deterioration is manifested as a progressive decline of the efficiencies with which mitochondria create the bulk of cellular ATP and generate biosynthetic intermediates for amino acids, nucleotides and lipids [1–10].

The functional state of mitochondria defines the pace of the replicative and chronological modes of aging in the yeast *Saccharomyces cerevisiae* [11–15]. Some traits of mitochondrial functionality are well known for their essential roles in yeast replicative and chronological aging. These mitochondrial traits include the following: 1) electron transport chain, oxidative phosphorylation and membrane potential [11–22]; 2) reactive oxygen species (ROS) homeostasis [11–13, 17, 23–34]; 3) protein synthesis and proteostasis [11, 27, 35–47]; 4) synthesis and assembly of iron-sulfur clusters [19, 27, 48–50]; 5) amino acid synthesis [12, 24, 27, 34, 51–56]; and 6) NADPH synthesis [12, 27, 34, 57, 58].

It remains unknown, however, if such an important trait of mitochondrial functionality as the composition of mitochondrial membrane lipids can play a role in yeast aging. We have previously found that exogenous lithocholic bile acid (LCA) delays the onset and decreases the rate of yeast chronological aging [20, 59]. We showed that the aging-delaying efficiency of LCA under caloric restriction (CR) on 0.2% glucose greatly exceeds its aging-delaying efficiency under non-CR conditions on 2% glucose [20]. We demonstrated that exogenously added LCA enters the yeast cell, accumulates in mitochondria, resides mainly in the inner mitochondrial membrane (IMM), and also associates with the outer mitochondrial membrane (OMM) [60]. We provided evidence that mitochondria-associated LCA alters the abundance and relative concentrations of different classes of membrane phospholipids [60], elicits age-related changes in the concentrations of many mitochondrial proteins [61], and modifies the age-related chronology of some key aspects of mitochondrial functionality [60]. Based on these findings, we proposed the following hypotheses: 1) LCA delays yeast chronological aging because it initially causes specific changes in the composition of mitochondrial membrane lipids; 2) the LCA-driven changes in mitochondrial lipidome then alter mitochondrial proteome; and 3) these changes in protein composition of mitochondria lead to specific alterations in mitochondrial functionality, thus creating a mitochondrial pattern that delays the onset and slows down the progression of yeast chronological aging [61, 62].

In our hypotheses, the LCA-dependent specific changes in the composition of mitochondrial membrane lipids may have a causal role in the age-related remodeling of mitochondrial proteome. The hypotheses further posit that certain mitochondrial proteins whose abundance is altered in response to LCA-driven changes in mitochondrial lipidome may play essential roles in creating a distinct, aging-delaying pattern of mitochondrial functionality. To test our hypotheses, in this study we investigated how the *ups1Δ*, *ups2Δ* and *psd1Δ* mutations eliminating different enzymes involved in mitochondrial phospholipid metabolism influence the following: 1) mitochondrial lipidome and proteome; 2) mitochondrial functionality; and 3) the geroprotective (aging-delaying) efficiency of LCA in chronologically aging yeast. These analyses revealed a distinct LCA-dependent pro-longevity pattern of mitochondrial lipidome, which consists in a proportional decrease of phosphatidylethanolamine and cardiolipin concentrations to maintain equimolar concentrations of these phospholipids, and in an increase of phosphatidic acid concentration. In turn, this pattern of mitochondrial lipidome enables a sustained specific aging-delaying pattern of mitochondrial proteome. Therefore, we also investigated how single-gene-deletion mutations eliminating the key proteins of such mitochondrial proteome pattern affect the efficiency of aging delay by LCA. In all these experiments yeast cells were cultured under CR in medium initially containing 0.2% glucose.

## RESULTS

### The relative concentrations of mitochondrial membrane phospholipids define the geroprotective (aging-delaying) efficiency of LCA

Our first objective was to test a hypothesis that the composition of mitochondrial membrane lipids may define the efficiency with which LCA delays yeast chronological aging. To attain this objective, we examined how single-gene-deletion mutations eliminating proteins involved in different aspects of phospholipid metabolism and transfer in mitochondrial membranes influence mitochondrial lipidome. We also assessed how these mutations affect the efficiency of yeast chronological aging delay by LCA.

**Figure 1:**
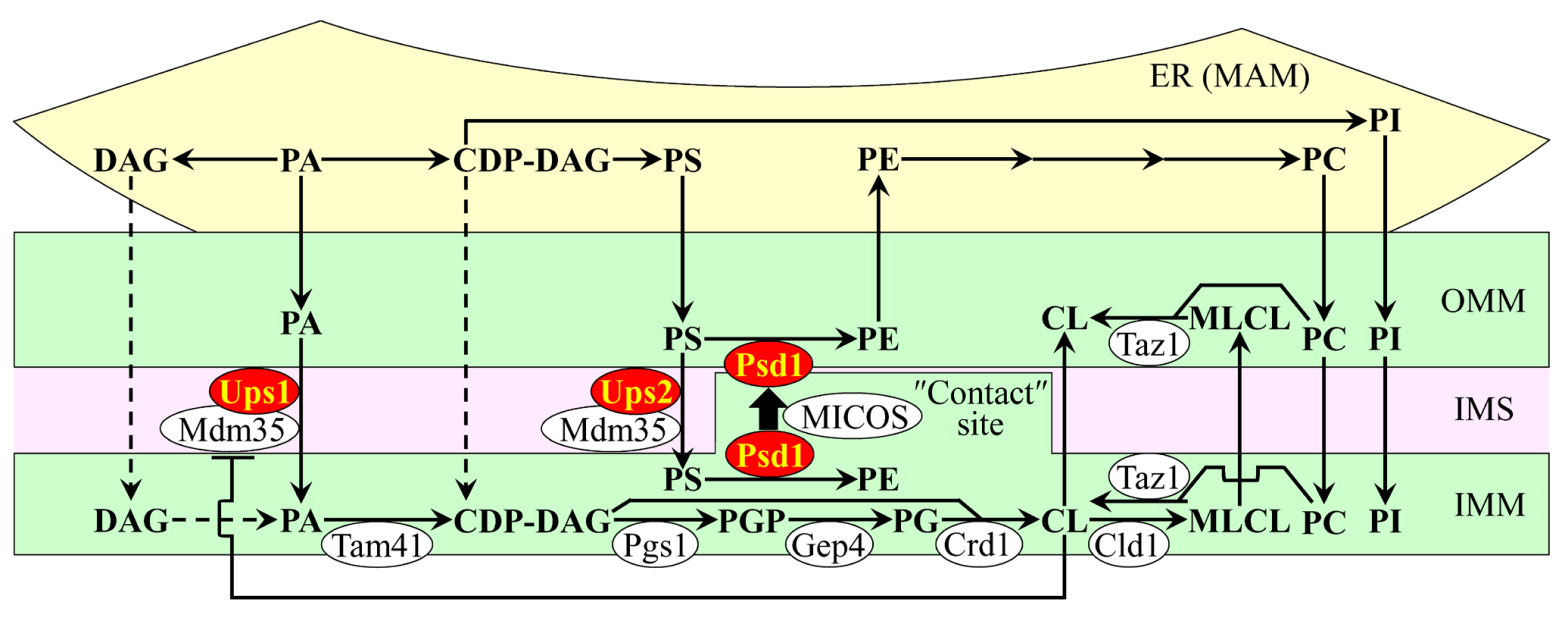
The relative concentrations of different classes of membrane phospholipids in yeast mitochondria depend on several processes of phospholipid synthesis and transfer These processes are catalyzed by enzymes that reside in the inner mitochondrial membrane (IMM), intermembrane space (IMS), outer mitochondrial membrane (OMM) and endoplasmic reticulum (ER). Only enzymes catalyzing these processes in the IMM, IMS and OMM are shown. A T bar denotes a cardiolipin (CL)-dependent inhibition of phosphatidic acid (PA) transfer from the OMM across the IMS to the IMM. In this study we investigated various effects of single-gene-deletion mutations that eliminate enzymes displayed in red color. See text for more details. Abbreviations: CDP-DAG, cytidine diphosphate-diacylglycerol; DAG, diacylglycerol; MAM, the mitochondria-associated membrane domain of the ER; MICOS, the mitochondrial contact site protein complex; MLCL, monolysocardiolipin; PC, phosphatidylcholine; PG, phosphatidylglycerol; PGP, phosphatidylglycerol-phosphate; PI, phosphatidylinositol; PS, phosphatidylserine.

A spatiotemporal dynamics of processes that define the relative concentrations of different classes of membrane phospholipids in yeast mitochondria is well-understood [63–70]. These processes are catalyzed by enzymes that reside in both mitochondria and the endoplasmic reticulum (ER). These processes include the following steps of phospholipid synthesis and transfer: 1) the synthesis of phosphatidic acid (PA), cytidine diphosphate-diacylglycerol (CDP-DAG), diacylglycerol (DAG), phosphatidylserine (PS), phosphatidylcholine (PC) and phosphatidylinositol (PI) in the ER; 2) the movement of PA from the ER to the OMM through mitochondria-ER contact sites at zones of a juxtaposition between the OMM and the mitochondria-associated membrane (MAM) domain of the ER; 3) the transfer of PA from the OMM across the intermembrane space (IMS) to the IMM, which is catalyzed by the Ups1/Mdm35 protein complex and inhibited by CL; 4) the conversion of ER-derived PA into CDP-DAG, phosphatidylglycerol-phosphate (PGP), phosphatidylglycerol (PG), cardiolipin (CL) and monolysocardiolipin (MLCL) in a series of reactions that are catalyzed by Tam41, Pgs1, Gep4, Crd1, Cld1 and Taz1 (respectively) in the IMM; 5) the transfer of PS from the ER to the OMM *via* mitochondria-ER contact sites by an unidentified mechanism; 6) the Ups2/Mdm35-dependent transport of PS from the OMM *via* the IMS to the IMM, where PS is converted into phosphatidylethanolamine (PE) in a reaction catalyzed by Psd1; 7) the synthesis of PE from PS in the OMM, which is catalyzed by Psd1, requires a close apposition between the two mitochondrial membranes and is assisted by the mitochondrial contact site (MICOS) protein complex; 8) the transfer of PC and PI from the ER to the OMM *via* mitochondria-ER contact sites, which is followed by the movement of PC and PI from the OMM to the IMM; mechanisms of both these processes remain obscure; and 9) the movement of DAG and CDP-DAG from the ER to the OMM across mitochondria-ER contact sites, and the subsequent transfer of these two phospholipids to the IMM by currently unknown mechanisms (Figure 1) [63–70].

### The *ups1Δ* mutation alters mitochondrial membrane lipidome and lowers the geroprotective efficiency of LCA

We used quantitative mass spectrometry to compare lipidomes of mitochondria purified from wild-type (WT) yeast cells cultured with or without LCA to those from *ups1Δ* cells. Both WT and *ups1Δ* cells were recovered on day 2, 4 or 7 of cell culturing, i.e. at different stages of chronological aging. The *ups1Δ* mutation eliminates a component of the Ups1/Mdm35 protein complex, which catalyzes the transfer of PA from the OMM across the IMS to the IMM [63, 71, 72]. We found that 1) akin to the effect of LCA on mitochondrial membrane lipidome in WT, LCA increases the concentrations of PS, PI, PC and PA in mitochondria of *ups1Δ* in an age-related manner; 2) similar to its effect in WT, LCA decreases the concentration of PE in mitochondrial membranes of *ups1Δ* at different stages of chronological aging; and 3) unlike an age-related decrease in the concentration of CL elicited by LCA in WT, this bile acid does not cause significant changes in CL concentration in *ups1Δ* (compare Supplementary Figures S1 and S2).

**Figure 2:**
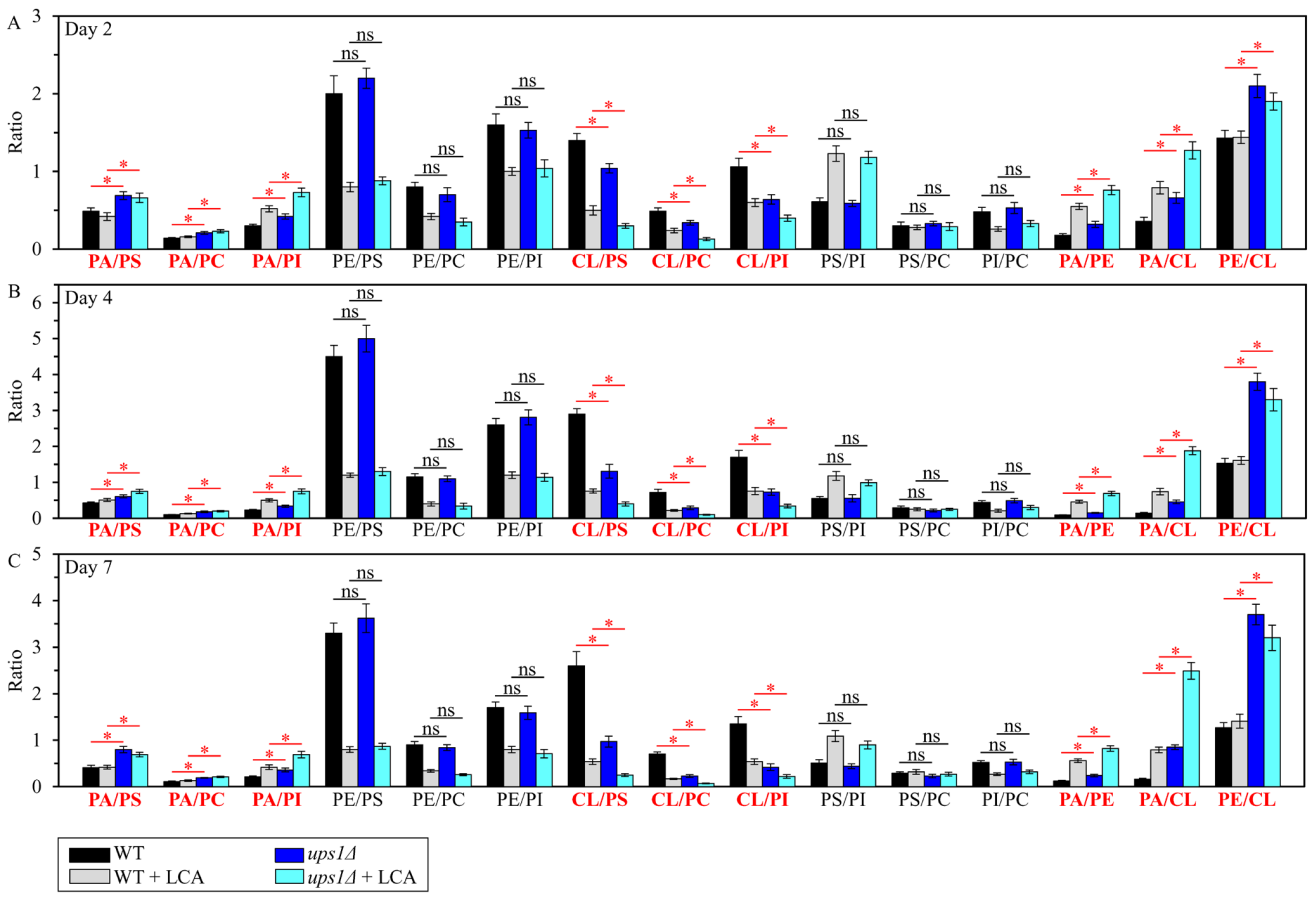
The *ups1Δ* mutation differently affects the ratios between concentrations of pairwise combinations of different phospholipid classes in mitochondrial membranes of chronologically aging yeast *ups1Δ* cells were cultured in the nutrient-rich YP medium initially containing 0.2% glucose with 50 μM LCA or without it. Mitochondria were purified from cells recovered on day 2 **A**., 4 **B**. or 7 **C**. of cell culturing. Extraction of mitochondrial membrane lipids, and mass spectrometric identification and quantitation of the extracted phospholipid classes were carried out as described in Materials and methods. Based on these data, the ratios between concentrations of all possible pairwise combinations of phospholipid classes were calculated. Data are presented as means ± SEM (*n* = 3; *p < 0.05; ns, not significant).

We then used these data to compare the ratios between concentrations of all possible pairwise combinations of phospholipid classes in mitochondrial membranes of WT and *ups1Δ* cells cultured with or without LCA. Of note, PA, PE and CL are phospholipid classes that have the non-bilayer forming shape of a cone; they increase the extent of membrane curving for the IMM, thereby raising the abundance of mitochondrial cristae (formed by the IMM) and mitochondrial contact cites (formed between the IMM and OMM) [60, 69, 70, 73–78]. In contrast, PS, PC and PI are phospholipid classes that exhibit the bilayer forming shape of a cylinder; they decrease the extent of membrane curving for the IMM, thus 1) increasing the abundance of the IMM domains having ″flat″ bilayer conformation; 2) decreasing the abundance of the IMM domains exhibiting negative curvature typical of mitochondrial contact sites; and 3) decreasing the abundance of the IMM domains displaying positive curvature characteristic of mitochondrial cristae [60, 69, 70, 73–78]. We found that, in cells cultured with or without LCA, the *ups1Δ* mutation 1) increases the PA/PS, PA/PC and PA/PI ratios for non-bilayer forming PA *vs*. bilayer forming PS, PC and PI; 2) does not alter the PE/PS, PE/PC and PE/PI ratios for non-bilayer forming PE *vs*. PS, PC and PI; 3) decreases the CL/PS, CL/PC and CL/PI ratios for non-bilayer forming CL *vs*. PS, PC and PI; 4) does not change the PS/PI, PS/PC and PI/PC ratios for all these bilayer forming phospholipid classes; and 5) increases the PA/PE, PA/CL and PE/CL ratios for all these non-bilayer forming phospholipid classes (Figure 2). Thus, while this mutation alters the relative to each other concentrations for all non-bilayer forming phospholipids, it has no effect on the relative to each other concentrations for phospholipids that exhibit the bilayer forming shape.

**Figure 3:**
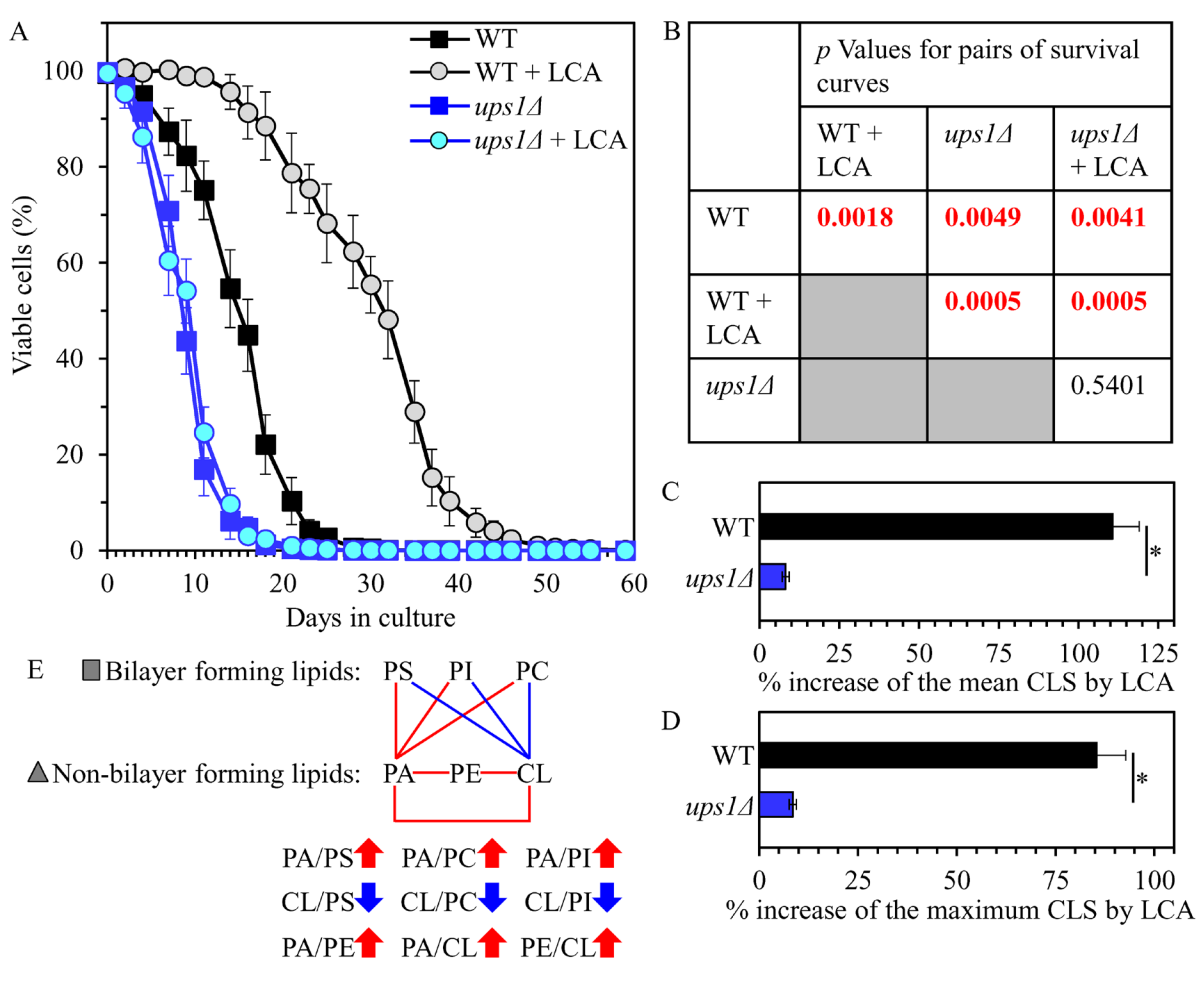
The *ups1Δ* mutation shortens yeast CLS in the absence of LCA and lowers the aging-delaying efficiency of LCA WT and *ups1Δ* cells were cultured in the nutrient-rich YP medium initially containing 0.2% glucose with 50 μM LCA or without it. **A**. Survival curves of chronologically aging WT and *ups1Δ* strains are shown. Data are presented as means ± SEM (*n* = 3). **B**. *p* Values for different pairs of survival curves of WT and *ups1Δ* strains cultured with or without LCA. Survival curves shown in **A**. were compared. Two survival curves were considered statistically different if the *p* value was less than 0.05. The *p* values for comparing pairs of survival curves using the logrank test were calculated as described in Materials and methods. **C**. and **D**. Survival curves shown in **A**. were used to calculate the percentage of increase of the mean and maximum CLS by LCA for WT and *ups1Δ* strains. Data are presented as means ± SEM (*n* = 3; *p < 0.05). **E**. The pattern of mitochondrial lipidome characteristic of the *ups1Δ* mutation is shown. Arrows next to the ratios between concentrations of different pairwise combinations of phospholipid classes indicate ratios that are increased (red arrows) or decreased (blue arrows) in *ups1Δ* cells (as compared to WT cells) cultured with or without LCA. Each colored line connects the names of two phospholipid classes whose concentration ratio is increased (red lines) or decreased (blue lines) by the *ups1Δ* mutation and cannot be restored by LCA.

We then assessed the effect of the *ups1Δ* mutation on the chronological lifespan (CLS) of yeast cultured with or without LCA. We found that *ups1Δ* 1) significantly shortens yeast CLS in the absence of LCA (Figures 3A and 3B); and 2) substantially lowers the geroprotective efficiency of LCA by almost completely eliminating the ability of LCA to increase both the mean and the maximum CLS (Figures 3A-3D).

In sum, these findings suggest that the *ups1Δ* mutation may accelerate yeast chronological aging and decrease the geroprotective efficiency of LCA because it creates a distinct pro-aging pattern of mitochondrial lipidome. This characteristic of *ups1Δ* pattern may include the following specific changes in the relative concentrations of different classes of membrane phospholipids: 1) an increase of the PA/PS, PA/PC and PA/PI ratios for non-bilayer forming PA *vs*. bilayer forming PS, PC and PI; 2) a decrease of the CL/PS, CL/PC and CL/PI ratios for non-bilayer forming CL *vs*. bilayer forming PS, PC and PI; and 3) an increase of the PA/PE, PA/CL and PE/CL ratios for all these non-bilayer forming phospholipid classes (Figure 3E).

### The *ups2Δ* mutation changes the composition of mitochondrial membrane lipids and amplifies the aging-delaying effect of LCA

Using quantitative mass spectrometry, we compared lipidomes of mitochondria purified from WT and *ups2Δ* cells. Both WT and mutant strains were cultured with or without LCA and recovered on day 2, 4 or 7 of cell culturing. The *ups2Δ* mutation eliminates a component of the Ups2/Mdm35 protein complex; Ups2/Mdm35 catalyzes the transfer of PS from the OMM across the IMS to the IMM, where PS is converted into PE in a Psd1-dependent reaction [66, 67, 70]. We found that similar to the effect of LCA on mitochondrial membrane lipidome in WT, LCA causes an age-related increase in the concentrations of PS, PI, PC and PA in mitochondria of *ups2Δ*; however, the concentration of PA in mitochondria of *ups2Δ* exceeds that in WT mitochondria of cells cultured with or without LCA (compare Supplementary Figures S1 and S3). We also observed that akin to the effect of LCA on mitochondrial membrane lipidome in WT, LCA elicits an age-related decline in the concentrations of PE and CL in mitochondria of *ups2Δ*; yet, the concentrations of both these phospholipids in mitochondria of *ups2Δ* are lower than in WT mitochondria of cells cultured with or without LCA (compare Supplementary Figures S1 and S3).

**Figure 4:**
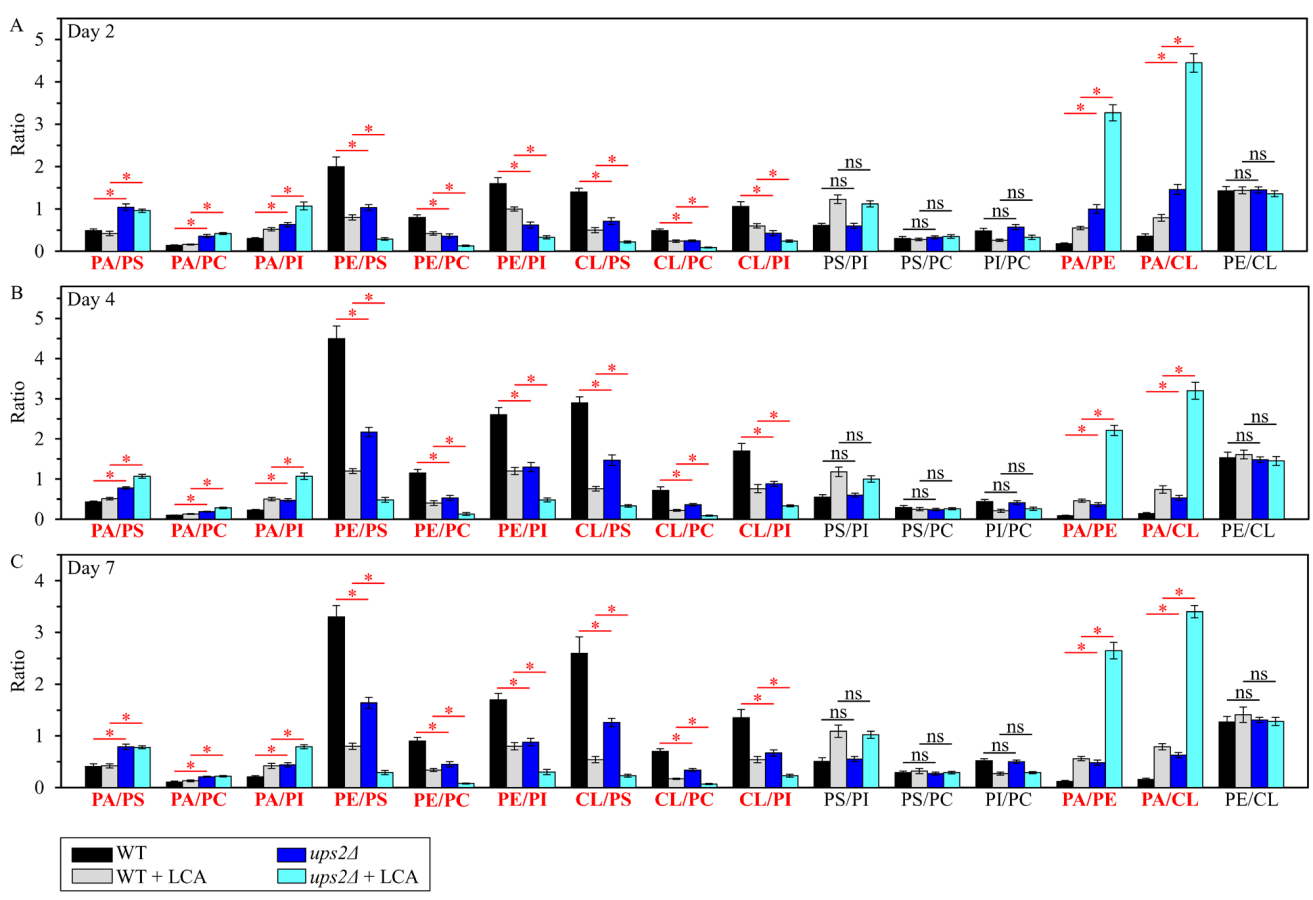
The *ups2Δ* mutation exhibits differential effects on the ratios between concentrations of different pairwise combinations of phospholipid classes in mitochondrial membranes of chronologically aging yeast *ups2Δ* cells were cultured in the nutrient-rich YP medium initially containing 0.2% glucose with 50 μM LCA or without it. Mitochondria were purified from cells recovered on day 2 **A**., 4 **B**. or 7 **C**. of cell culturing. Extraction of mitochondrial membrane lipids, and mass spectrometric identification and quantitation of the extracted phospholipid classes were carried out as described in Materials and methods. Based on these data, the ratios between concentrations of all possible pairwise combinations of phospholipid classes were calculated. Data are presented as means ± SEM (*n* = 3; *p < 0.05; ns, not significant).

Using the above data, we then compared the ratios between concentrations of all conceivable pairwise combinations of different phospholipids in membranes of mitochondria purified from WT and *ups2Δ* cells; these cells were cultured with or without LCA. We found that, in cells cultured in the presence or absence of LCA, the *ups2Δ* mutation 1) raises the PA/PS, PA/PC and PA/PI ratios for non-bilayer forming PA *vs*. bilayer forming PS, PC and PI; 2) lowers the PE/PS, PE/PC and PE/PI ratios for non-bilayer forming PE *vs*. PS, PC and PI; 3) lessens the CL/PS, CL/PC and CL/PI ratios for non-bilayer forming CL *vs*. PS, PC and PI; 4) does not alter the PS/PI, PS/PC and PI/PC ratios for all these bilayer forming classes of phospholipids; 5) rises the PA/PE and PA/CL ratios for all these non-bilayer forming phospholipid classes; and 6) does not alter the PE/CL ratio for these two non-bilayer forming phospholipids (Figure 4). Thus, unlike the *ups1Δ* mutation, *ups2Δ* allows to maintain the equimolar concentrations of two non-bilayer forming phospholipids, PE and CL, by proportionally decreasing their concentrations.

**Figure 5:**
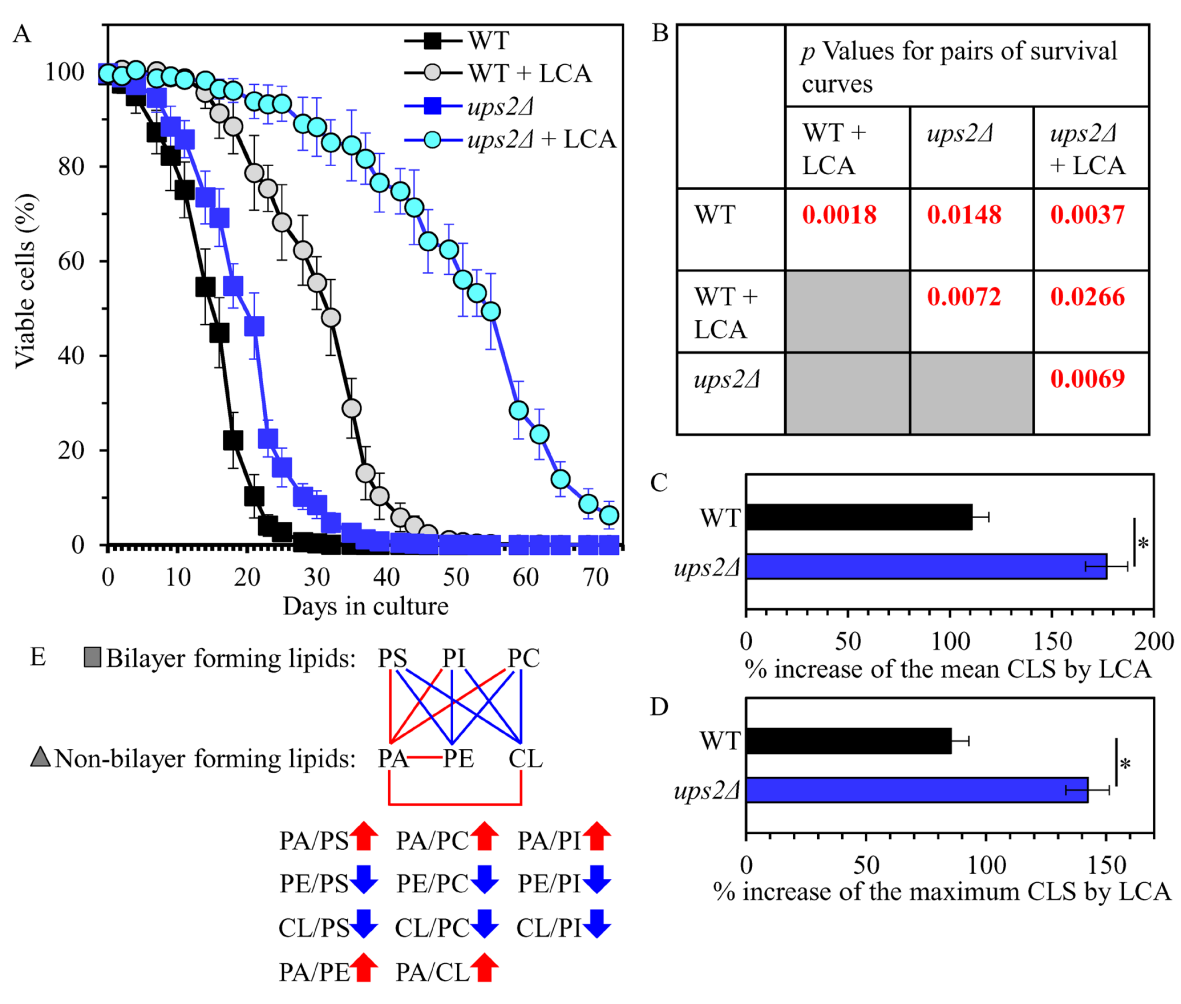
The *ups2Δ* mutation extends yeast CLS in the absence of LCA and amplifies the geroprotective efficiency of LCA WT and *ups2Δ* cells were cultured in the nutrient-rich YP medium initially containing 0.2% glucose with 50 μM LCA or without it. **A**. Survival curves of chronologically aging WT and *ups2Δ* strains are shown. Data are presented as means ± SEM (*n* = 3). **B**. *p* Values for different pairs of survival curves of WT and *ups2Δ* strains cultured with or without LCA. Survival curves shown in **A**. were compared. Two survival curves were considered statistically different if the *p* value was less than 0.05. The *p* values for comparing pairs of survival curves using the logrank test were calculated as described in Materials and methods. **C**. and **D**. Survival curves shown in **A**. were used to calculate the percentage of increase of the mean and maximum CLS by LCA for WT and *ups2Δ* strains. Data are presented as means ± SEM (*n* = 3; *p < 0.05). **E**. The pattern of mitochondrial lipidome characteristic of the *ups2Δ* mutation is shown. Arrows next to the ratios between concentrations of different pairwise combinations of phospholipid classes indicate ratios that are increased (red arrows) or decreased (blue arrows) in *ups2Δ* cells (as compared to WT cells) cultured with or without LCA. Each colored line connects the names of two phospholipid classes whose concentration ratio is increased (red lines) or decreased (blue lines) by the *ups2Δ* mutation and cannot be restored by LCA.

Our assessment of the effect of the *ups2Δ* mutation on the CLS of yeast cultured with or without LCA revealed that *ups2Δ* 1) extends yeast CLS in the absence of LCA (Figures 5A and 5B); and 2) significantly amplifies the geroprotective efficiency of LCA by enhancing the ability of this bile acid to increase both the mean and the maximum CLS (Figures 5A-5D).

Taken together, the above findings indicate that the *ups2Δ* mutation may slow down yeast chronological aging and increase the geroprotective efficiency of LCA because it establishes a distinctive aging-delaying pattern of mitochondrial lipidome. This *ups2Δ*-specific pattern may consist of the following distinct changes in the relative concentrations of different phospholipid classes: 1) an increase of the PA/PS, PA/PC and PA/PI ratios for non-bilayer forming PA *vs*. bilayer forming PS, PC and PI; 2) a decrease of the PE/PS, PE/PC and PE/PI ratios for non-bilayer forming PE *vs*. bilayer forming PS, PC and PI; 3) a decline of the CL/PS, CL/PC and CL/PI ratios for non-bilayer forming CL *vs*. bilayer forming PS, PC and PI; and 4) a rise of the PA/PE and PA/CL ratios for these non-bilayer forming classes of phospholipids (Figure 5E).

### The *psd1Δ* mutation elicits changes in mitochondrial membrane lipidome and lessens the geroprotective efficiency of LCA

We compared lipidomes of purified mitochondria from WT cells cultured with or without LCA to those from *psd1Δ* cells. These cells were recovered for purification of mitochondria on day 2, 4 or 7 of cell culturing. The *psd1Δ* mutation eliminates phosphatidylserine decarboxylase which catalyzes the conversion of PS to PE in the IMM and OMM [66, 67, 70, 79, 80]. We found that similar to the effect of LCA on mitochondrial membrane lipidome in WT, LCA causes an age-related rise in the concentrations of PS, PI, PC and PA in mitochondria of *psd1Δ*; however, the concentration of PA in mitochondria of *psd1Δ* is higher than in mitochondria of WT cultured with or without LCA (compare Supplementary Figures S1 and S4). We also observed that akin to the effect of LCA on mitochondrial membrane lipidome in WT, LCA triggers an age-related decline in the concentrations of PE and CL in mitochondria of *psd1Δ*; however, the concentrations of both PE and CL in mitochondria of *psd1Δ* are lower than in mitochondria of WT cultured with or without LCA (compare Supplementary Figures S1 and S4).

**Figure 6:**
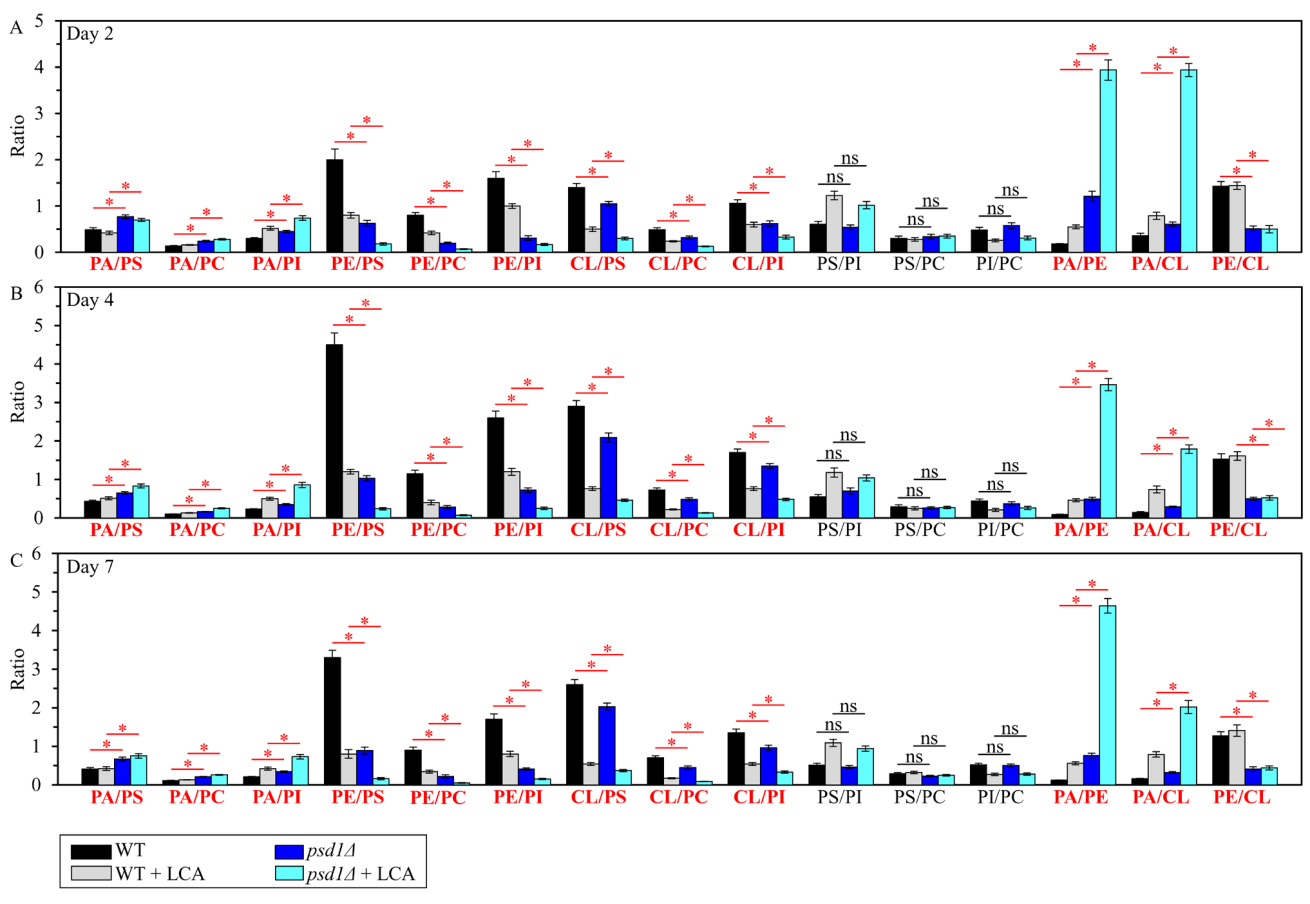
The *psd1Δ* mutation differently affects the ratios between concentrations of pairwise combinations of different phospholipid classes in mitochondrial membranes of chronologically aging yeast *psd1Δ* cells were cultured in the nutrient-rich YP medium initially containing 0.2% glucose with 50 μM LCA or without it. Mitochondria were purified from cells recovered on day 2 **A**., 4 **B**. or 7 **C**. of cell culturing. Extraction of mitochondrial membrane lipids, and mass spectrometric identification and quantitation of the extracted phospholipid classes were carried out as described in Materials and methods. Based on these data, the ratios between concentrations of all possible pairwise combinations of phospholipid classes were calculated. Data are presented as means ± SEM (*n* = 3; *p < 0.05; ns, not significant).

We then used the above data to compare the ratios between concentrations of all possible pairwise combinations of phospholipid classes in mitochondrial membranes of WT and *psd1Δ* cells cultured in the presence or absence of LCA. We found that, in cells cultured with or without LCA, the *psd1Δ* mutation 1) augments the PA/PS, PA/PC and PA/PI ratios for non-bilayer forming PA *vs*. bilayer forming PS, PC and PI; 2) reduces the PE/PS, PE/PC and PE/PI ratios for non-bilayer forming PE *vs*. PS, PC and PI; 3) lessens the CL/PS, CL/PC and CL/PI ratios for non-bilayer forming CL *vs*. PS, PC and PI; 4) does not change the PS/PI, PS/PC and PI/PC ratios for all these bilayer forming classes of phospholipids; 5) rises the PA/PE and PA/CL ratios for all these non-bilayer forming phospholipid classes; and 6) decreases the PE/CL ratio for these two non-bilayer forming phospholipids (Figure 6). Thus, unlike *ups2Δ* and similar to *ups1Δ*, the *psd1Δ* mutation does not allow to maintain the equimolar concentrations of two non-bilayer forming phospholipids, PE and CL.

**Figure 7:**
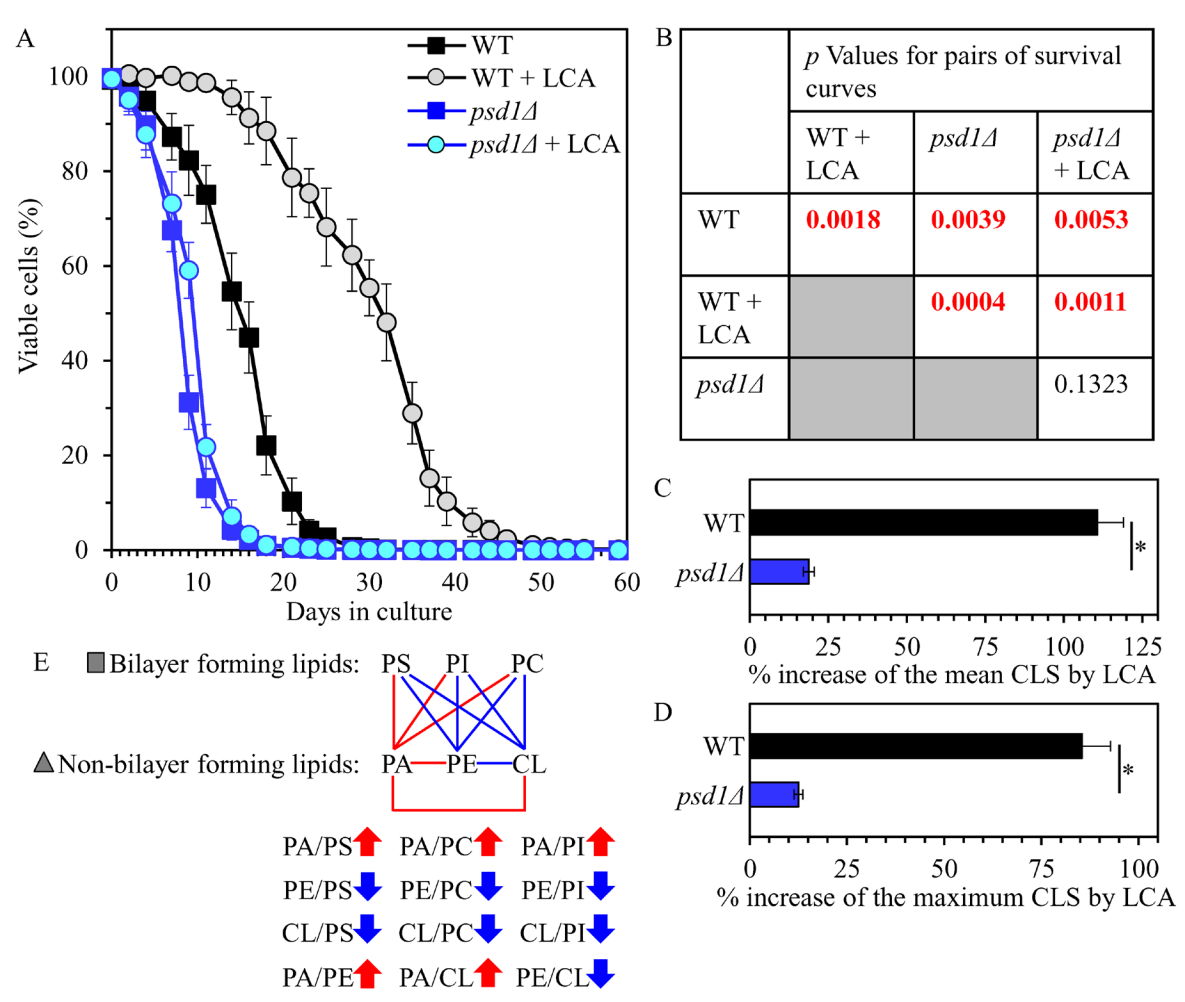
The *psd1Δ* mutation shortens yeast CLS in the absence of LCA and considerably decreases the geroprotective efficiency of LCA WT and *psd1Δ* cells were cultured in the nutrient-rich YP medium initially containing 0.2% glucose with 50 μM LCA or without it. **A**. Survival curves of chronologically aging WT and *psd1Δ* strains are shown. Data are presented as means ± SEM (*n* = 3). **B**. *p* Values for different pairs of survival curves of WT and *psd1Δ* strains cultured with or without LCA. Survival curves shown in **A**. were compared. Two survival curves were considered statistically different if the *p* value was less than 0.05. The *p* values for comparing pairs of survival curves using the logrank test were calculated as described in Materials and methods. **C**. and **D**. Survival curves shown in **A**. were used to calculate the percentage of increase of the mean and maximum CLS by LCA for WT and *psd1Δ* strains. Data are presented as means ± SEM (*n* = 3; *p < 0.05). **E**. The pattern of mitochondrial lipidome characteristic of the *psd1Δ* mutation is shown. Arrows next to the ratios between concentrations of different pairwise combinations of phospholipid classes indicate ratios that are increased (red arrows) or decreased (blue arrows) in *psd1Δ* cells (as compared to WT cells) cultured with or without LCA. Each colored line connects the names of two phospholipid classes whose concentration ratio is increased (red lines) or decreased (blue lines) by the *psd1Δ* mutation and cannot be restored by LCA.

Our assessment of how the *psd1Δ* mutation influences the CLS of yeast cultured with or without LCA showed that *psd1Δ* 1) shortens yeast CLS in the absence of LCA (Figures 7A and 7B); and 2) substantially decreases the aging-delaying efficiency of LCA by significantly lowering the ability of this bile acid to increase both the mean and the maximum CLS (Figures 7A-7D).

Collectively, these findings suggest that the *psd1Δ* mutation may accelerate yeast chronological aging and decrease the geroprotective efficiency of LCA because it establishes a distinct pro-aging pattern of mitochondrial lipidome. This characteristic for *psd1Δ* pattern may include the following specific changes in the relative concentrations of different classes of membrane phospholipids: 1) an increase of the PA/PS, PA/PC and PA/PI ratios for non-bilayer forming PA *vs*. bilayer forming PS, PC and PI; 2) a decrease of the PE/PS, PE/PC and PE/PI ratios for non-bilayer forming PE *vs*. bilayer forming PS, PC and PI; 3) a decline of the CL/PS, CL/PC and CL/PI ratios for non-bilayer forming CL *vs*. bilayer forming PS, PC and PI; 4) an increase of the PA/PE and PA/CL ratios for these non-bilayer forming phospholipid classes; and 5) a decrease in the PE/CL ratio for these two non-bilayer forming classes of phospholipids (Figure 7E).

### A distinct pro-longevity pattern of mitochondrial lipidome extends yeast CLS in the absence of LCA and amplifies the geroprotective efficiency of LCA

**Figure 8:**
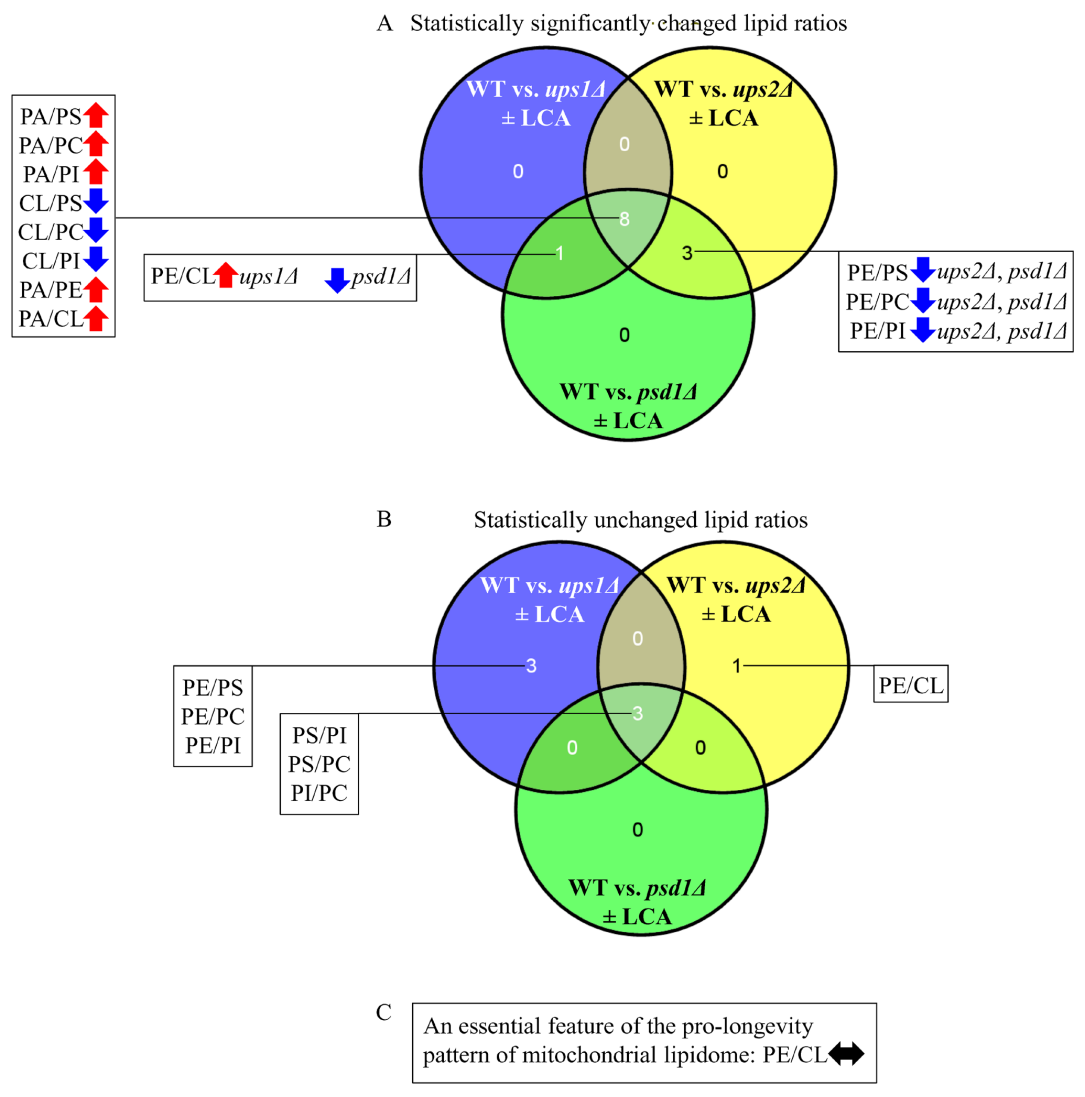
Venn diagrams showing a comparison of the effects of the *ups1Δ*, *ups2Δ* and *psd1Δ* mutations on the ratios between concentrations of various pairwise combinations of mitochondrial membrane phospholipids A comparison of the datasets for the ratios that have been statistically significantly changed **A**. or statistically unchanged **B**. by the *ups1Δ*, *ups2Δ* and *psd1Δ* mutations in yeast cultured with or without LCA are shown. **C**. The maintenance of equimolar concentrations of PE and CL is an essential feature of the pro-longevity pattern of mitochondrial lipidome. Arrows next to the ratios between concentrations of different pairwise combinations of phospholipid classes indicate ratios that are increased (red arrows) or decreased (blue arrows) in *ups1Δ*, *ups2Δ* or *psd1Δ* cells (as compared to WT cells) cultured with or without LCA.

We noticed that the *ups1Δ*, *ups2Δ* and *psd1Δ* mutations cause some similar changes to the ratios between concentrations of various pairwise combinations of mitochondrial membrane phospholipids (compare Figures 3E, 5E and 7E). We therefore compared the datasets of such ratios that have been statistically significantly changed (SSC) by the *ups1Δ*, *ups2Δ* and *psd1Δ* mutations in yeast cultured with or without LCA. We found the following for the total of 12 SSC ratios: 1) 8 SSC ratios overlap between the *ups1Δ*, *ups2Δ* and *psd1Δ* datasets; 2) 3 SSC ratios are common to the datasets of the CLS-extending *ups2Δ* mutation that amplifies the geroprotective efficiency of LCA and the CLS-shortening *psd1Δ* mutation that lessens such efficiency; and 3) 1 SSC ratio, namely PE/CL, is common to the datasets of the *ups1Δ* and *psd1Δ* mutations, both of which are CLS-shortening mutations that lower the geroprotective efficiency of LCA (*ups1Δ* increases the PE/CL ratio, whereas *psd1Δ* decreases it) (Figure 8A).

We also noticed that some ratios between concentrations of various pairwise combinations of mitochondrial membrane phospholipids are not statistically changed by the *ups1Δ*, *ups2Δ* and *psd1Δ* mutations. Our analysis of these statistically unchanged (SU) ratios revealed the following: 1) 3 SU ratios overlap between the *ups1Δ*, *ups2Δ* and *psd1Δ* datasets; 2) 3 SU ratios are unique to the dataset of *ups1Δ*, a CLS-shortening mutation that lowers the geroprotective efficiency of LCA; and 3) 1 SU ratio, namely PE/CL, is unique to the dataset of *ups2Δ*, a CLS-extending mutation that amplifies the geroprotective efficiency of LCA (Figure 8B).

In sum, these findings suggest that the maintenance of equimolar concentrations of two non-bilayer forming and cone-shaped phospholipids, PE and CL, may be critical feature of the pro-longevity pattern of mitochondrial lipidome (Figure 8C). This feature is a longevity assurance trait in the absence of LCA. This feature is also required for the delay of yeast chronological aging by exogenous LCA.

The maintenance of equimolar concentrations of PE and CL is a trait that is common to WT and *ups2Δ*, a mutant strain that not only lives longer than WT but also exhibits an amplified (as compared to WT) geroprotective efficiency of LCA (Figure 5). It is conceivable, therefore, that some traits distinguishing mitochondrial lipidome of *ups2Δ* from that of WT may represent additional features of the pro-longevity pattern of mitochondrial lipidome. These additional features are likely to be responsible for the ability of the *ups2Δ* mutation to extend longevity in the absence of LCA and also to increase the geroprotective efficiency of LCA. Among these additional features are the ones that differentiate mitochondrial lipidome of *ups2Δ* from that of WT; they include the following: 1) an increased concentration of PA, another non-bilayer forming and cone-shaped phospholipid; 2) a decreased concentration of PE; and 3) a proportionally (as compared to PE) decreased concentration of CL (compare Supplementary Figures S1 and S3). We hypothesize that the proportional decrease in the concentrations of PE and CL in mitochondrial membranes of *ups2Δ* may be responsible for the increased concentration of PA in these membranes.

### The *ups1Δ* and *ups2Δ* mutations alter the concentrations of many mitochondrial proteins in yeast cultured with or without LCA

Our hypothesis posits that the LCA-dependent establishment and maintenance of a distinct pro-longevity pattern of mitochondrial lipidome may have a causal role in the age-related remodeling of mitochondrial proteome. We therefore used quantitative mass spectrometry to compare the identities and relative concentrations of proteins that were recovered in mitochondria purified from WT, *ups1Δ* and *ups2Δ* cells cultured with or without LCA. The *ups1Δ* mutation establishes and maintains a distinct pro-aging pattern of mitochondrial lipidome (Figures 2, 3 and 8), shortens CLS (Figure 3), and lowers the geroprotective efficiency of LCA (Figure 3). In contrast, the *ups2Δ* mutation institutes and preserves a specific pro-longevity pattern of mitochondrial lipidome (Figures 4, 5 and 8), extends CLS (Figure 5), and amplifies the geroprotective efficiency of LCA (Figure 5).

We found that both mutations, *ups1Δ* and *ups2Δ*, alter the age-related chronology of changes in concentrations of numerous mitochondrial proteins in yeast cultured in the presence of LCA or in its absence; these proteins have been implicated in many essential mitochondrial functions (see Supplementary Figure S5 and Supplementary Tables S1 - S9 for *ups1Δ*, and Supplementary Figure S7 and Supplementary Tables S10 - S15 for *ups2Δ*).

We then compared the datasets of relative concentrations of mitochondrial proteins that are statistically significantly downregulated or upregulated 1) by LCA in WT; 2) by the *ups1Δ* or *ups2Δ* mutation in the absence of LCA; and 3) by LCA in *ups1Δ* or *ups2Δ* cells.

Our comparative analysis of these datasets for *ups1Δ* revealed the following: 1) many mitochondrial proteins that are downregulated or upregulated in *ups1Δ* cells cultured without LCA are unique to these datasets (i.e. these proteins are not present in the datasets of mitochondrial proteins that are downregulated or upregulated by LCA in WT or by LCA in *ups1Δ* cells) (Supplementary Figures S6A - S6F); and 2) the total number and identities of mitochondrial proteins that are downregulated or upregulated in *ups1Δ* cells cultured with or without LCA fluctuate significantly in cells recovered on day 2, 4 or 7 of cell culturing (i.e. at different stages of chronological aging) (Supplementary Figures S6H - S6L).

For *ups2Δ*, we found that: 1) a number of mitochondrial proteins that are downregulated or upregulated in *ups2Δ* cells cultured in the absence of LCA cannot be found in the datasets of mitochondrial proteins downregulated or upregulated by LCA in WT or by LCA in *ups2Δ* cells (Supplementary Figures S8A - S8F); 2) the total number and identities of mitochondrial proteins that are downregulated or upregulated in *ups2Δ* cells cultured in the absence of LCA vary substantially with the chronological age of cells (Supplementary Figures S8H and S8K); 3) numerous mitochondrial proteins that are downregulated or upregulated by LCA in *ups2Δ* cells are unique to these datasets as they cannot be found in the datasets of mitochondrial proteins downregulated or upregulated by LCA in WT or by the *ups2Δ* mutation in the absence of LCA (Supplementary Figures S8A - S8F); and 4) the total number and identities of mitochondrial proteins that are downregulated or upregulated by LCA in *ups2Δ* cells fluctuate significantly with the chronological age of cells (Supplementary Figures S8I and S8L).

Taken together, these findings indicate that in yeast cultured with or without LCA 1) the establishment and maintenance of a distinct pro-aging pattern of mitochondrial lipidome in short-lived *ups1Δ* cells (Figures 2, 3 and 8) correlate with the establishment and maintenance of a specific aging-accelerating pattern of mitochondrial proteome in these mutant cells; and 2) the institution and preservation of a specific pro-longevity pattern of mitochondrial lipidome in long-lived *ups2Δ* cells (Figures 4, 5 and 8) correlate with the institution and preservation of a distinctive aging-delaying pattern of mitochondrial proteome in these mutant cells.

### The mitochondrial proteomes of *ups1Δ* and *ups2Δ* differ substantially

Because our findings suggest that in yeast cultured with or without LCA the *ups1Δ* and *ups2Δ* mutations can establish and maintain either an aging-accelerating or an aging-delaying (respectively) pattern of mitochondrial proteome, we compared the datasets of relative concentrations of mitochondrial proteins that are statistically significantly downregulated or upregulated in *ups1Δ* or *ups2Δ* cells in the absence or presence of LCA.

**Figure 9:**
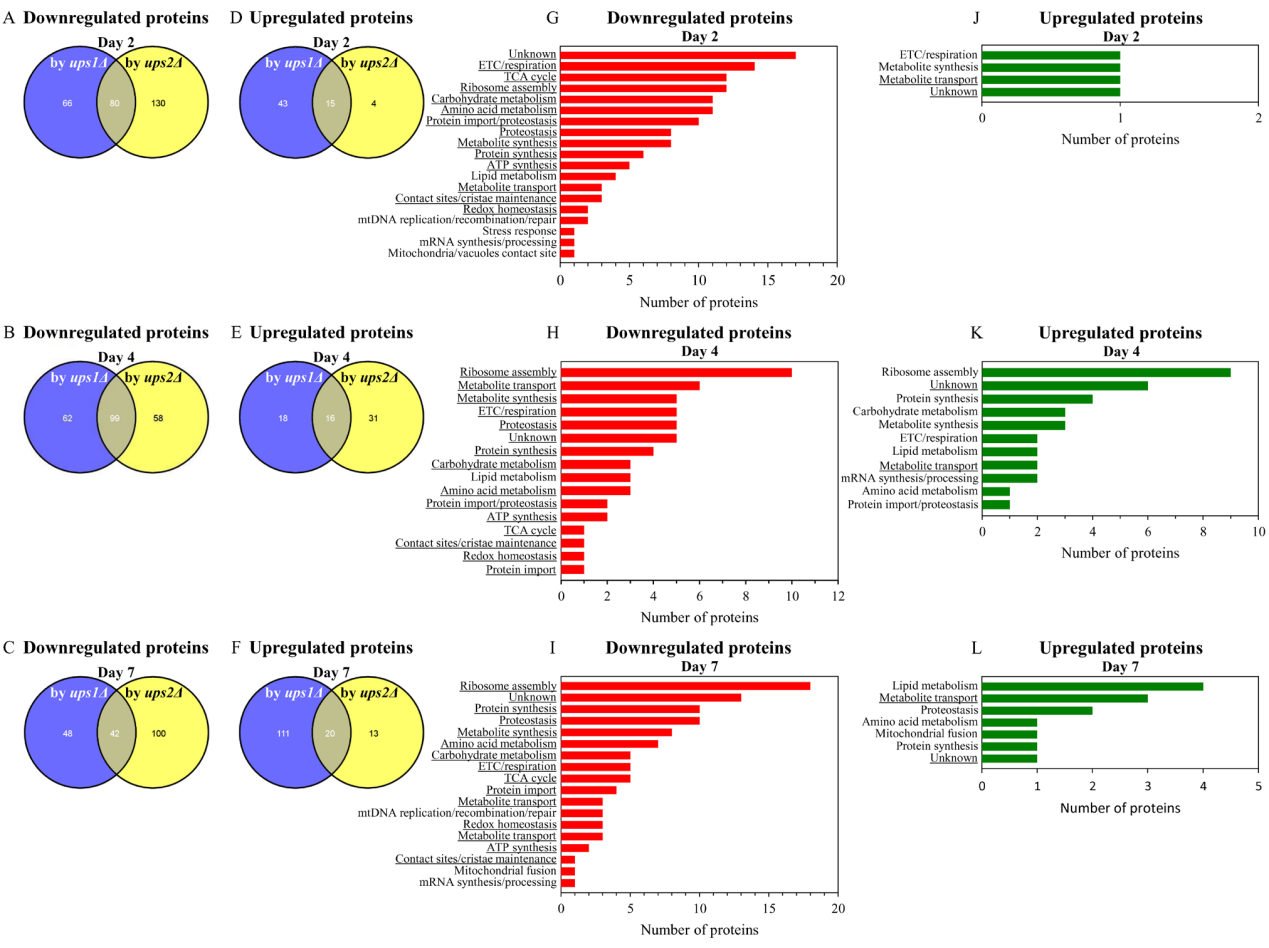
In cells cultured without LCA, many mitochondrial proteins that are downregulated or upregulated in long-lived *ups2Δ* cells are not downregulated or upregulated in short-lived *ups1Δ* cells (A - F) Venn diagrams showing a comparison of the datasets of relative concentrations of mitochondrial proteins that are statistically significantly downregulated or upregulated in *ups1Δ* or *ups2Δ* cells cultured in the absence of LCA; cells were recovered on day 2, 4 or 7 of culturing. (G - L) Mitochondrial proteins that are downregulated or upregulated only in long-lived *ups2Δ* cells belong to various functional categories. The names of functional categories whose protein representatives were downregulated or upregulated in *ups2Δ* cells recovered on every of the three days are underlined. Functions of some mitochondrial proteins that are downregulated or upregulated only in *ups2Δ* cells are currently unknown. Abbreviations: ETC, electron transport chain; mtDNA, mitochondrial DNA; TCA, the tricarboxylic acid cycle.

For *ups1Δ* and *ups2Δ* cells cultured without LCA, we found the following: 1) many mitochondrial proteins that are downregulated or upregulated in long-lived *ups2Δ* cells are not present in the datasets of mitochondrial proteins that are downregulated or upregulated in short-lived *ups1Δ* cells (Figures 9A - 9F; Supplementary Tables S16 and S17); and 2) the total number and identities of mitochondrial proteins that are downregulated or upregulated only in long-lived *ups2Δ* cells fluctuate significantly in cells recovered at different stages of chronological aging (Figures 9A - 9F; Supplementary Tables S16 and S17). Functions of some mitochondrial proteins that are downregulated or upregulated only in long-lived *ups2Δ* cells remain to be established (Figures 9G - 9L; Supplementary Tables S16 and S17). Many mitochondrial proteins that are downregulated or upregulated only in long-lived *ups2Δ* cells have been implicated in essential mitochondrial functions, including the electron transport chain (ETC), respiration, the tricarboxylic acid (TCA) cycle, ribosome assembly, amino acid metabolism, carbohydrate metabolism, protein import, proteostasis, metabolite synthesis, protein synthesis, ATP synthesis, metabolite transport, lipid metabolism, contact sites and cristae maintenance, redox homeostasis, mitochondrial DNA (mtDNA) maintenance, stress response, mRNA synthesis and processing, the maintenance of contact sites between mitochondria and vacuoles, and mitochondrial fusion (Figures 9G - 9L; Supplementary Tables S16 and S17).

**Figure 10:**
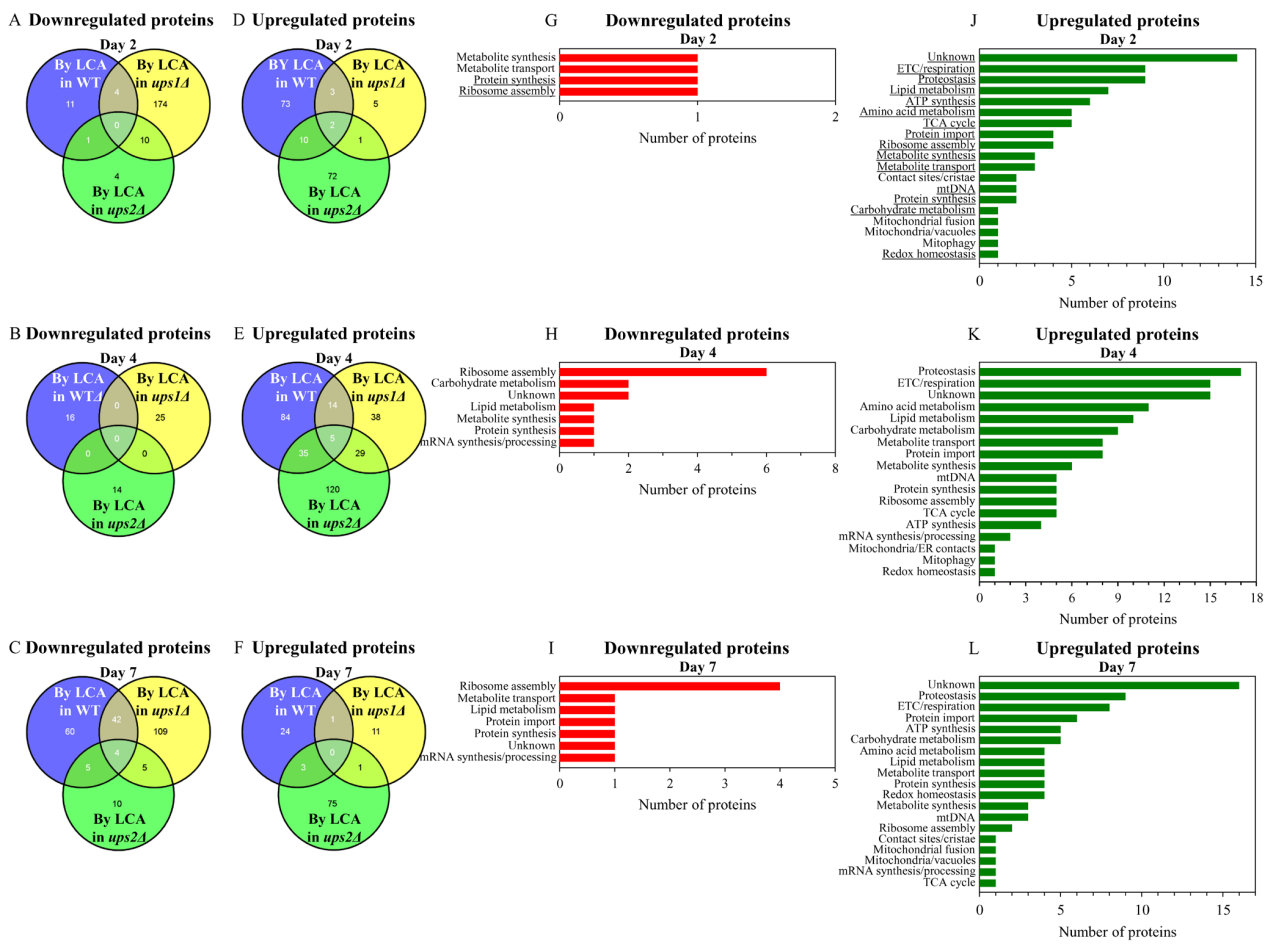
In cells cultured with LCA, many mitochondrial proteins that are downregulated or upregulated by LCA in long-lived *ups2Δ* cells are not downregulated or upregulated by LCA in WT or short-lived *ups1Δ* cells **A**. - **F**. Venn diagrams showing a comparison of the datasets of relative concentrations of mitochondrial proteins that are statistically significantly downregulated or upregulated by LCA in WT, *ups1Δ* or *ups2Δ*; cells were recovered on day 2, 4 or 7 of culturing. **G**. - **L**. Mitochondrial proteins that are downregulated or upregulated by LCA only in long-lived *ups2Δ* cells belong to many different functional categories. The names of functional categories whose protein representatives were downregulated or upregulated by LCA in *ups2Δ* cells recovered on every of the three days are underlined. Functions of some mitochondrial proteins that are downregulated or upregulated by LCA only in *ups2Δ* cells are currently unknown. Abbreviations: ETC, electron transport chain; mtDNA, mitochondrial DNA; TCA, the tricarboxylic acid cycle.

For *ups1Δ* and *ups2Δ* cells cultured with LCA, we found the following: 1) a number of mitochondrial proteins that are downregulated or upregulated by LCA in long-lived *ups2Δ* cells cannot be found in the datasets of mitochondrial proteins that are downregulated or upregulated by LCA in WT or short-lived *ups1Δ* cells (Figures 10A - 10F; Supplementary Tables S18 and S19); and 2) the total number and identities of mitochondrial proteins that are downregulated or upregulated by LCA only in long-lived *ups2Δ* cells vary notably in cells recovered at different stages of chronological aging (Figures 10A - 10F; Supplementary Tables S18 and S19). Functions of some mitochondrial proteins that are downregulated or upregulated by LCA only in long-lived *ups2Δ* cells remain unknown (Figures 10G - 10L; Supplementary Tables S18 and S19). Numerous mitochondrial proteins that are downregulated or upregulated by LCA only in long-lived *ups2Δ* cells are known for their essential roles in the same set of mitochondrial functions as the ones played by proteins that are downregulated or upregulated only in long-lived *ups2Δ* cells culture without LCA (compare Figures 9G - 9L and Supplementary Tables S16 and S17 to Figures 10G - 10L and Supplementary Tables S18 and S19).

In sum, these findings suggest that in yeast cultured with or without LCA the *ups2Δ* mutation may establish and maintain an aging-delaying pattern of mitochondrial proteome.

### Many mitochondrial proteins that are downregulated or upregulated by LCA only in long-lived *ups2Δ* cells play essential roles in aging delay by LCA

Because our findings suggest that in yeast cultured with LCA the *ups2Δ* mutation may establish and maintain a distinct pattern of mitochondrial proteome that is essential for the ability of LCA to delay aging, we investigated how single-gene-deletion mutations eliminating the key proteins constituting such pattern affect the geroprotective efficiency of LCA.

We thought that some of the proteins downregulated by LCA in *ups2Δ* may function in attenuating the geroprotective efficiency of LCA, and thus the elimination of these proteins by mutations may increase such efficiency. We also thought that some of the proteins upregulated by LCA in *ups2Δ* may act as facilitators of aging delay by LCA, and therefore the elimination of these proteins by mutations may decrease the geroprotective efficiency of LCA.

**Figure 11:**
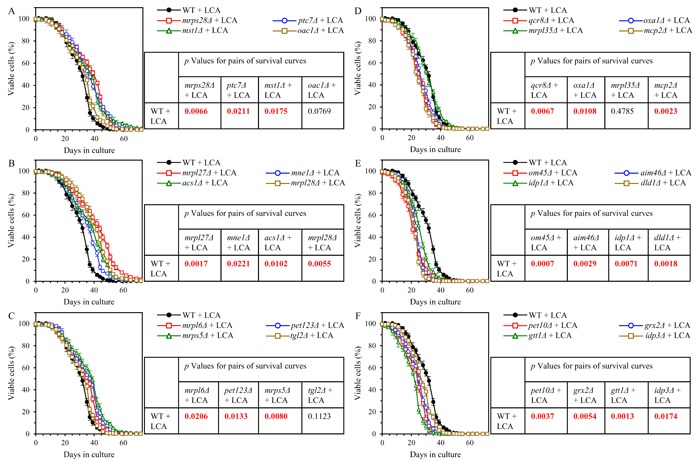
Many mutations eliminating proteins downregulated by LCA in *ups2Δ* increase the aging-delaying efficiency of LCA, while many mutations eliminating proteins upregulated by LCA in *ups2Δ* decrease such efficiency WT and mutant cells were cultured in the nutrient-rich YP medium initially containing 0.2% glucose with 50 μM LCA. **A**. - **C**. Survival curves of chronologically aging WT and mutant strains that lack proteins most highly downregulated by LCA in *ups2Δ* are shown. Data are presented as means ± SEM (*n* = 3). Also shown are *p* values for different pairs of survival curves of WT and mutant strains cultured with LCA. Survival curves shown in **A**. - **C**. were compared. Two survival curves were considered statistically different if the *p* value was less than 0.05. The *p* values for comparing pairs of survival curves using the logrank test were calculated as described in Materials and methods. **D**. - **F**. Survival curves of chronologically aging WT and mutant strains that lack proteins most substantially upregulated by LCA in *ups2Δ* are shown. Data are presented as means ± SEM (*n* = 3). Also shown are *p* values for different pairs of survival curves of WT and mutant strains cultured with LCA. Survival curves shown in **D**. -**F**. were compared. Two survival curves were considered statistically different if the *p* value was less than 0.05. The *p* values for comparing pairs of survival curves using the logrank test were calculated as described in Materials and methods.

We found that mutants that lack 10 out of 12 proteins most highly downregulated by LCA in *ups2Δ* exhibit a statistically significantly increase of the aging-delaying efficiency of LCA; a slight increase of such efficiency seen in mutants that lack 2 other downregulated by LCA proteins was not statistically significant (Figures 11A - 11C). We also found that mutants that lack 11 out of 12 proteins vastly upregulated by LCA in *ups2Δ* display a statistically significantly decrease of the geroprotective efficiency of LCA; a minor decrease of such efficiency observed in a mutant lacking one of the downregulated by LCA proteins was not statistically significant (Figures 11D - 11F). Of note, none of these mutants exhibits such robust change of the aging-delaying efficiency of LCA as the changes seen in the *ups1Δ*, *ups2Δ* and *psd1Δ* mutants (compare Figures 3, 5, 7 and 11). This finding suggests that Ups1, Ups2 and Psd1 may function as upstream regulators of various mitochondrial processes whose synergistic action defines the efficiency of aging delay by LCA.

Taken together, these findings further support the notion that the *ups2Δ* mutation may allow to sustain a distinct aging-delaying pattern of mitochondrial proteome that is essential for the ability of LCA to delay aging.

### The *ups1Δ* and *ups2Δ* mutations have different effects on some key aspects of mitochondrial functionality in yeast cultured with LCA

Our hypothesis posits that the LCA-dependent remodeling of mitochondrial lipidome and the resulting changes in mitochondrial proteome may create an aging-delaying pattern of mitochondrial functionality that is essential for the ability of LCA to delay aging. To test this hypothesis, we monitored the age-related chronology of changes in four vital cellular processes confined to and regulated by mitochondria. These processes include the following: 1) mitochondrial respiration; 2) the maintenance of electrochemical potential (ΔΨ) across the IMM; 3) the maintenance of the homeostasis of cellular ROS known to be produced primarily as by-products of mitochondrial respiration [81, 82]; and 4) the modulation of cellular concentrations of ATP, which in yeast cultured in media with low (0.2%) glucose concentration is generated mainly in mitochondria [81, 82]. These vital processes were monitored in chronologically aging WT, *ups1Δ* and *ups2Δ* cells cultured with LCA.

**Figure 12:**
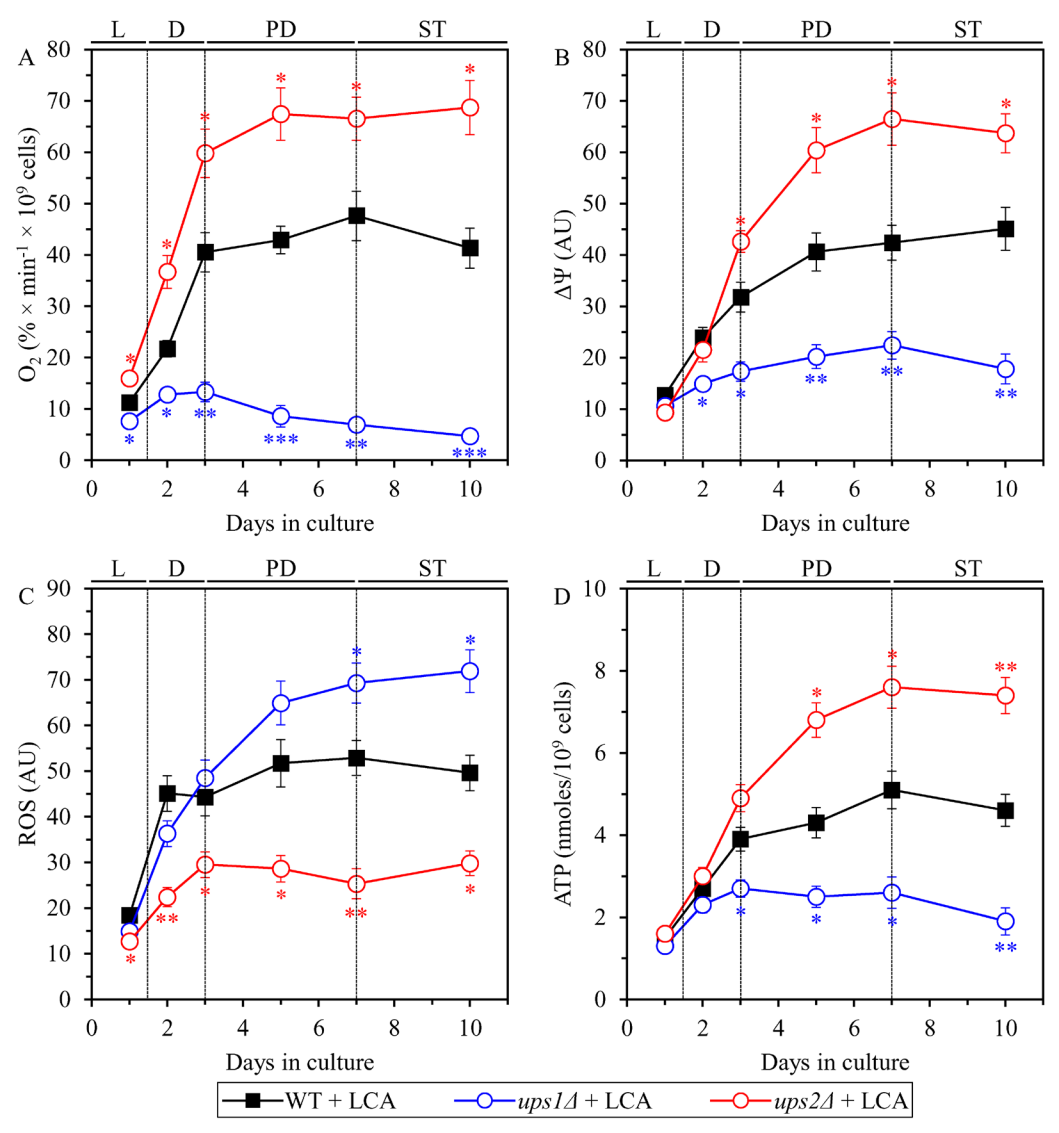
In yeast cultured with LCA, the *ups1Δ* and *ups2Δ* mutations differently affect four key aspects of mitochondrial functionality WT and mutant cells were cultured in the nutrient-rich YP medium initially containing 0.2% glucose with 50 μM LCA. The dynamics of age-related changes in the rate of oxygen consumption my cells **A**., electrochemical potential (ΔΨ) across the IMM **B**., cellular concentration of ROS **C**. and cellular concentration of ATP **D**. during chronological aging of yeast. Data are presented as means ± SEM (*n* = 3; *p < 0.05; **p < 0.01; ***p < 0.001).

We found that in WT cells cultured with LCA the rate of mitochondrial respiration 1) is increased when cells enter diauxic (D) growth phase that begins on day 2 of culturing; 2) reaches a plateau in post-diauxic (PD) growth phase that occurs between days 3 and 7 of culturing; and 3) slightly declines during the subsequent stationary (ST) growth phase that follows PD phase and begins after day 7 of culturing (Figure 12A). In *ups2Δ* cells cultured with LCA the rate of mitochondrial respiration 1) is increased to a significantly higher extent during D phase than in WT cells; and 2) reaches a plateau in PD phase and then additionally rises in ST phase to a substantially higher level than that in WT cells (Figure 12A). In *ups1Δ* cells cultured with LCA the rate of mitochondrial respiration 1) is increased to a significantly lower degree during D phase than in WT cells; and 2) substantially declines in PD and ST phases to reach a markedly lower level than that in WT cells (Figure 12A).

In WT cells cultured with LCA the value of ΔΨ 1) is increased during D phase; 2) reaches a plateau during PD phase; and 3) slightly rises during ST phase (Figure 12B). In *ups2Δ* cells cultured with LCA the value of ΔΨ 1) is increased to a similar extent during D phase as in WT cells; 2) further rises during PD phase to reach a level significantly exceeding that in WT cells; and 3) slightly declines but remains higher than that in WT cells during ST phase (Figure 12B). In *ups1Δ* cells cultured with LCA the value of ΔΨ 1) is increased to a significantly lower degree during D phase than in WT cells; and 2) slightly rises during PD phase and then declines in ST phase to reach a substantially lower level than that in WT cells (Figure 12B).

In WT cells cultured with LCA the concentration of ROS 1) is increased during D phase; 2) reaches a plateau during PD phase; and 3) remains mainly unchanged during ST phase (Figure 12C). In *ups2Δ* cells cultured with LCA the concentration of ROS 1) is increased to a lower degree during D phase than in WT cells; and 2) undergoes minor changes during PD and ST phases to reach a level that is significantly lower than that in WT cells (Figure 12C). In *ups1Δ* cells cultured with LCA the concentration of ROS 1) is increased to a similar extent during D phase as in WT cells; and 2) further rises during PD and ST phases to reach a level significantly exceeding that in WT cells (Figure 12C).

In WT cells cultured with LCA the concentration of ATP 1) is increased during D phase; 2) reaches a plateau during PD phase; and 3) slightly declines during ST phase (Figure 12D). In *ups2Δ* cells cultured with LCA the concentration of ATP 1) is increased to a similar degree during D phase as in WT cells; 2) further rises during PD phase to reach a level significantly higher than that in WT cells; and 3) slightly declines but remains higher than that in WT cells during ST phase (Figure 12D). In *ups1Δ* cells cultured with LCA the value of ΔΨ 1) is increased to a significantly lower extent during D phase than in WT cells; and 2) remains unchanged during PD phase and then declines in ST phase to reach a substantially lower level than that in WT cells (Figure 12D).

In sum, these findings support the notion that the LCA-dependent remodeling of mitochondrial lipidome and the resulting changes in mitochondrial proteome can create an aging-delaying pattern of mitochondrial functionality that is essential for the ability of LCA to delay aging.

## DISCUSSION

This study provides evidence that the mitochondrial lipidome defines not only the rate of yeast chronological aging but also the geroprotective efficiency of LCA in chronologically aging yeast. We demonstrate the existence of a distinct pro-longevity pattern of mitochondrial lipidome, which extends yeast CLS in the absence of LCA and amplifies the geroprotective efficiency of LCA. This pattern consists in a proportional decrease of PE and CL concentrations, and in a concomitant increase of PA concentration. PE, CL and PA are non-bilayer forming, cone-shaped phospholipid classes that increase the extent of membrane curving for the IMM to rise the abundance of mitochondrial cristae (formed by the IMM) and mitochondrial contact cites (formed between the IMM and OMM) [60, 69, 70, 73–78]. We also show that these LCA-driven specific changes in the composition of mitochondrial membrane lipids cause a distinct remodeling of mitochondrial proteome by decreasing and increasing concentration of many mitochondrial proteins. These proteins have been implicated in such vital mitochondrial functions as the ETC and respiration, the TCA cycle, ribosome assembly, amino acid metabolism, carbohydrate metabolism, protein import, proteostasis, metabolite synthesis, protein synthesis, ATP synthesis, metabolite transport, lipid metabolism, contact sites and cristae maintenance, redox homeostasis, mtDNA maintenance, stress response, mRNA synthesis and processing, the maintenance of contact sites between mitochondria and vacuoles, and mitochondrial fusion. We provide evidence that the LCA-dependent remodeling of mitochondrial lipidome and the resulting changes in mitochondrial proteome allow to change the age-related chronology of changes in such vital mitochondrial processes as respiration, electrochemical potential maintenance, ROS homeostasis preservation and ATP synthesis. These changes facilitate the establishment and maintenance of an aging-delaying pattern of mitochondrial functionality that is essential for the ability of LCA to delay aging.

The challenge for the future is to define molecular mechanisms underlying the LCA-driven remodeling of mitochondrial lipidome and proteome. Our previous findings suggested a hypothesis in which LCA may cause such remodeling of mitochondrial lipidome by 1) slowing down the Psd1-dependent reaction of the synthesis of PE from PS in the IMM and/or OMM; 2) decelerating the Crd1-dependent reaction of CL synthesis from PG in the IMM; and 3) attenuating a negative feedback loop that involves a CL-dependent inhibition of PA transport from the OMM across the IMS to the IMM, which is catalyzed by the Ups1/Mdm35 protein complex (Figure 1) [60]. Studies aimed at testing this hypothesis are currently in progress. Furthermore, we previously demonstrated that LCA causes major changes not only in mitochondrial membrane lipidome but also in the size, number and morphology of mitochondria [60]. One could envision that these LCA-driven changes in mitochondrial abundance and morphology may affect mitochondrial protein import, folding, assembly and other aspects of mitochondrial proteostasis, thereby altering mitochondrial proteome. Our ongoing studies address the validity of this assumption.

## MATERIALS AND METHODS

### Yeast strains and growth conditions

The WT strain BY4742 (*MAT*α *his3Δ1 leu2Δ0 lys2Δ0 ura3Δ0*) and single-gene-deletion mutant strains in the BY4742 genetic background (all from Thermo Scientific/Open Biosystems) were grown in YP medium (1% yeast extract, 2% peptone) initially containing 0.2% glucose with 50 μM LCA or without it. Cells were cultured at 30°C with rotational shaking at 200 rpm in Erlenmeyer flasks at a “flask volume/medium volume” ratio of 5:1.

### Miscellaneous procedures

Purification of mitochondria [83], SDS-PAGE [84], quantitative mass spectrometric analysis of lipids [85] and statistical analysis [86] were performed as previously described. Mass spectrometric identification and quantification of proteins were performed as previously reported [87]. The ″Proteome Discoverer″ software was used to calculate the exponentially modified protein abundance index (emPAI), a measure of the relative abundance of mitochondrial proteins in a pair of analyzed datasets.

## SUPPLEMENTARY MATERIALS FIGURES AND TABLES



## Abbreviations

CL: cardiolipin
CLS: chronological life span
CDP-DAG: cytidine diphosphate-diacylglycerol
DAG: diacylglycerol
D: diauxic
IMM: the inner mitochondrial membrane
IMS: the intermembrane space
LCA: lithocholic bile acid
MAM: mitochondria-associated membrane
MICOS: mitochondrial contact site
MLCL: monolysocardiolipin
OMM: the outer mitochondrial membrane
PA: phosphatidic acid
PC: phosphatidylcholine
PD: post-diauxic
PG: phosphatidylglycerol
PGP: phosphatidylglycerol-phosphate
PI: phosphatidylinositol
PS: phosphatidylserine
ROS: reactive oxygen species
SSC: statistically significantly changed
ST: stationary
SU: statistically unchanged
WT: wild-type.
