# Supplementary material for: Specific changes in mitochondrial lipidome alter mitochondrial proteome and increase the geroprotective efficiency of lithocholic acid in chronologically aging yeast
Source: Oncotarget. 2017 Mar 31;8(19):30672–91. doi: 10.18632/oncotarget.16766 (PMC5458158; doi:10.18632/oncotarget.16766)
Supplement: Supplementary file 1 [file oncotarget-08-30672-s001.pdf]

Specific changes in mitochondrial lipidome alter mitochondrial proteome and increase the geroprotective efficiency of lithocholic acid in chronologically aging yeast

Supplementary Material

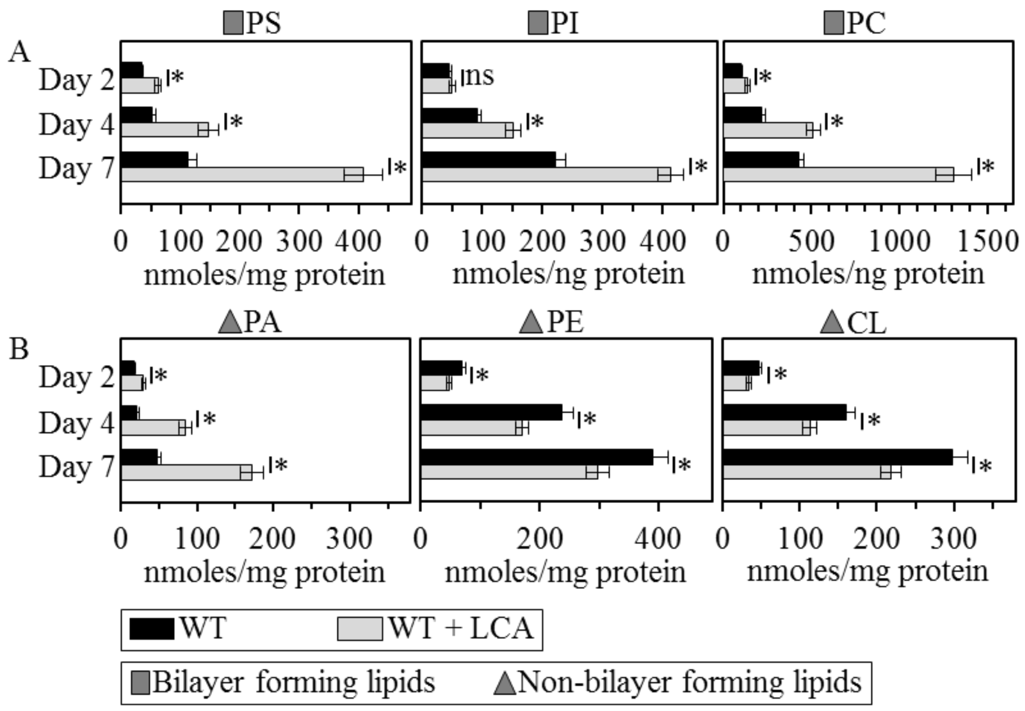

Supplemental Figure S1. LCA exhibits age-related differential effects on the concentrations of different classes of mitochondrial membrane phospholipids in chronologically aging wild-type (WT) yeast. WT cells were cultured in the nutrient-rich YP medium initially containing 0.2% glucose with 50 µM LCA or without it. Mitochondria were purified from cells recovered on day 2, 4 or 7 of cell culturing. Extraction of mitochondrial membrane lipids, and mass spectrometric identification and quantitation of the extracted phospholipid classes were carried out as described in Materials and methods. The concentrations of phospholipids were calculated as nmoles phospholipid/mg protein. (**A**) PS, PI and PC are phospholipid classes that exhibit the bilayer forming shape of a cylinder; they decrease the extent of membrane curving for the IMM. (**B**) PA, PE and CL are phospholipid classes that have the non-bilayer forming shape of a cone; they increase the extent of membrane curving for the IMM. Data are presented as means ± SEM (n = 3; *p < 0.05; ns, not significant). Abbreviations: CL, cardiolipin; PA, phosphatidic acid; PC, phosphatidylcholine; PE, phosphatidylethanolamine; PA, phosphatidic acid; PI, phosphatidylinositol; PS, phosphatidylserine.

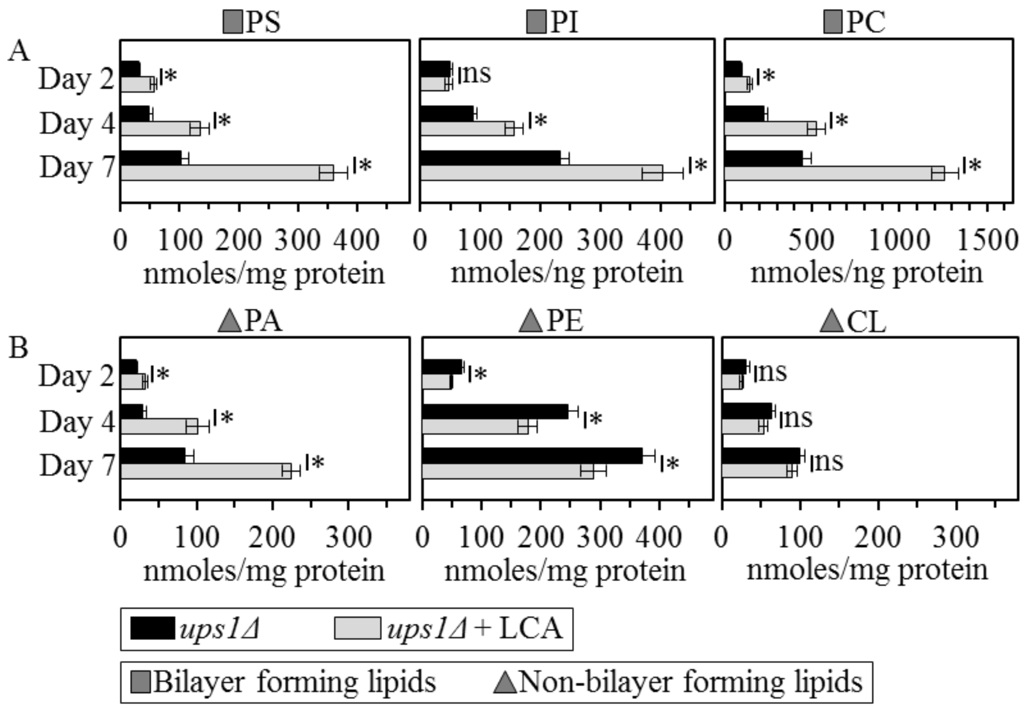

Supplemental Figure S2. LCA exhibits age-related differential effects on the concentrations of different classes of mitochondrial membrane phospholipids in chronologically aging *ups1Δ* yeast. Cells of the *ups1Δ* mutant strain were cultured in the nutrient-rich YP medium initially containing 0.2% glucose with 50 µM LCA or without it. Mitochondria were purified from cells recovered on day 2, 4 or 7 of cell culturing. Extraction of mitochondrial membrane lipids, and mass spectrometric identification and quantitation of the extracted phospholipid classes were carried out as described in Materials and methods. The concentrations of phospholipids were calculated as nmoles phospholipid/mg protein. (**A**) PS, PI and PC are phospholipid classes that exhibit the bilayer forming shape of a cylinder; they decrease the extent of membrane curving for the IMM. (**B**) PA, PE and CL are phospholipid classes that have the non-bilayer forming shape of a cone; they increase the extent of membrane curving for the IMM. Data are presented as means ± SEM (n = 3; *p < 0.05; ns, not significant). Abbreviations: CL, cardiolipin; PA, phosphatidic acid; PC, phosphatidylcholine; PE, phosphatidylethanolamine; PA, phosphatidic acid; PI, phosphatidylinositol; PS, phosphatidylserine.

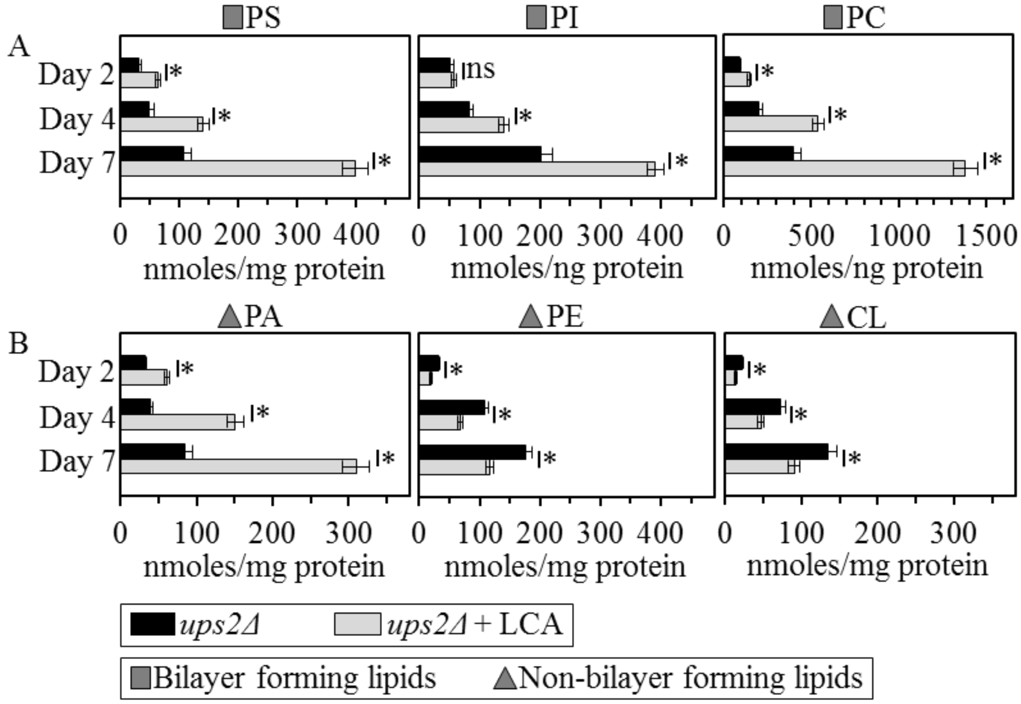

Supplemental Figure S3. LCA exhibits age-related differential effects on the concentrations of different classes of mitochondrial membrane phospholipids in chronologically aging *ups2Δ* yeast. Cells of the *ups2Δ* mutant strain were cultured in the nutrient-rich YP medium initially containing 0.2% glucose with 50 µM LCA or without it. Mitochondria were purified from cells recovered on day 2, 4 or 7 of cell culturing. Extraction of mitochondrial membrane lipids, and mass spectrometric identification and quantitation of the extracted phospholipid classes were carried out as described in Materials and methods. The concentrations of phospholipids were calculated as nmoles phospholipid/mg protein. (**A**) PS, PI and PC are phospholipid classes that exhibit the bilayer forming shape of a cylinder; they decrease the extent of membrane curving for the IMM. (**B**) PA, PE and CL are phospholipid classes that have the non-bilayer forming shape of a cone; they increase the extent of membrane curving for the IMM. Data are presented as means ± SEM (n = 3; *p < 0.05; ns, not significant). Abbreviations: CL, cardiolipin; PA, phosphatidic acid; PC, phosphatidylcholine; PE, phosphatidylethanolamine; PA, phosphatidic acid; PI, phosphatidylinositol; PS, phosphatidylserine.

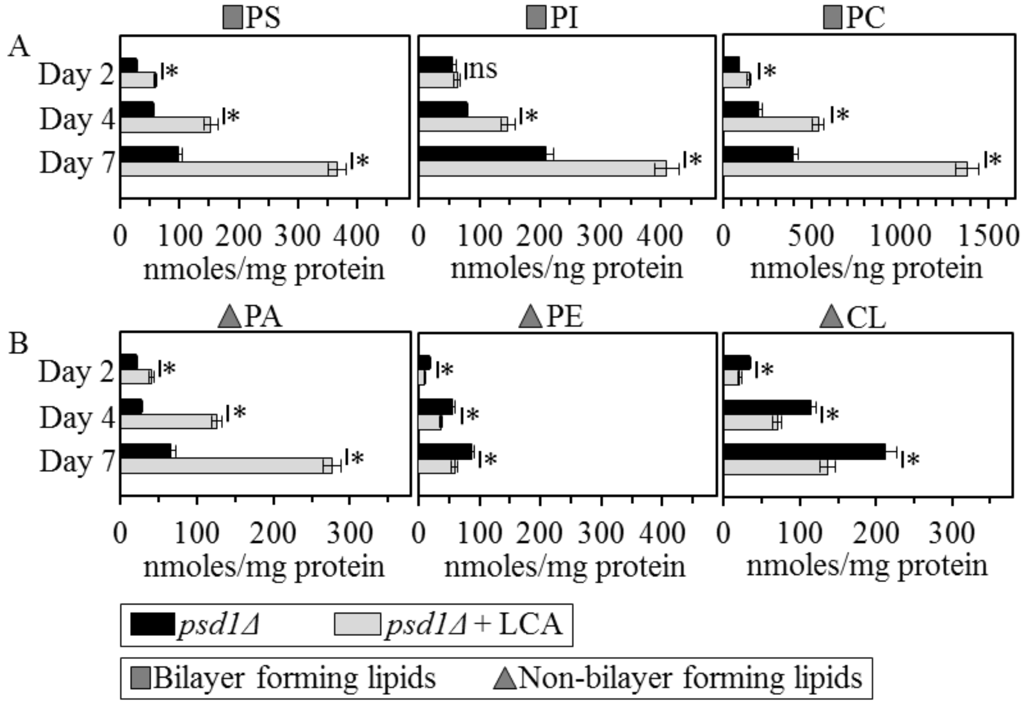

Supplemental Figure S4. LCA exhibits age-related differential effects on the concentrations of different classes of mitochondrial membrane phospholipids in chronologically aging *psd1Δ* yeast. Cells of the *psd1Δ* mutant strain were cultured in the nutrient-rich YP medium initially containing 0.2% glucose with 50 µM LCA or without it. Mitochondria were purified from cells recovered on day 2, 4 or 7 of cell culturing. Extraction of mitochondrial membrane lipids, and mass spectrometric identification and quantitation of the extracted phospholipid classes were carried out as described in Materials and methods. The concentrations of phospholipids were calculated as nmoles phospholipid/mg protein. (**A**) PS, PI and PC are phospholipid classes that exhibit the bilayer forming shape of a cylinder; they decrease the extent of membrane curving for the IMM. (**B**) PA, PE and CL are phospholipid classes that have the non-bilayer forming shape of a cone; they increase the extent of membrane curving for the IMM. Data are presented as means ± SEM (n = 3; *p < 0.05; ns, not significant). Abbreviations: CL, cardiolipin; PA, phosphatidic acid; PC, phosphatidylcholine; PE, phosphatidylethanolamine; PA, phosphatidic acid; PI, phosphatidylinositol; PS, phosphatidylserine.

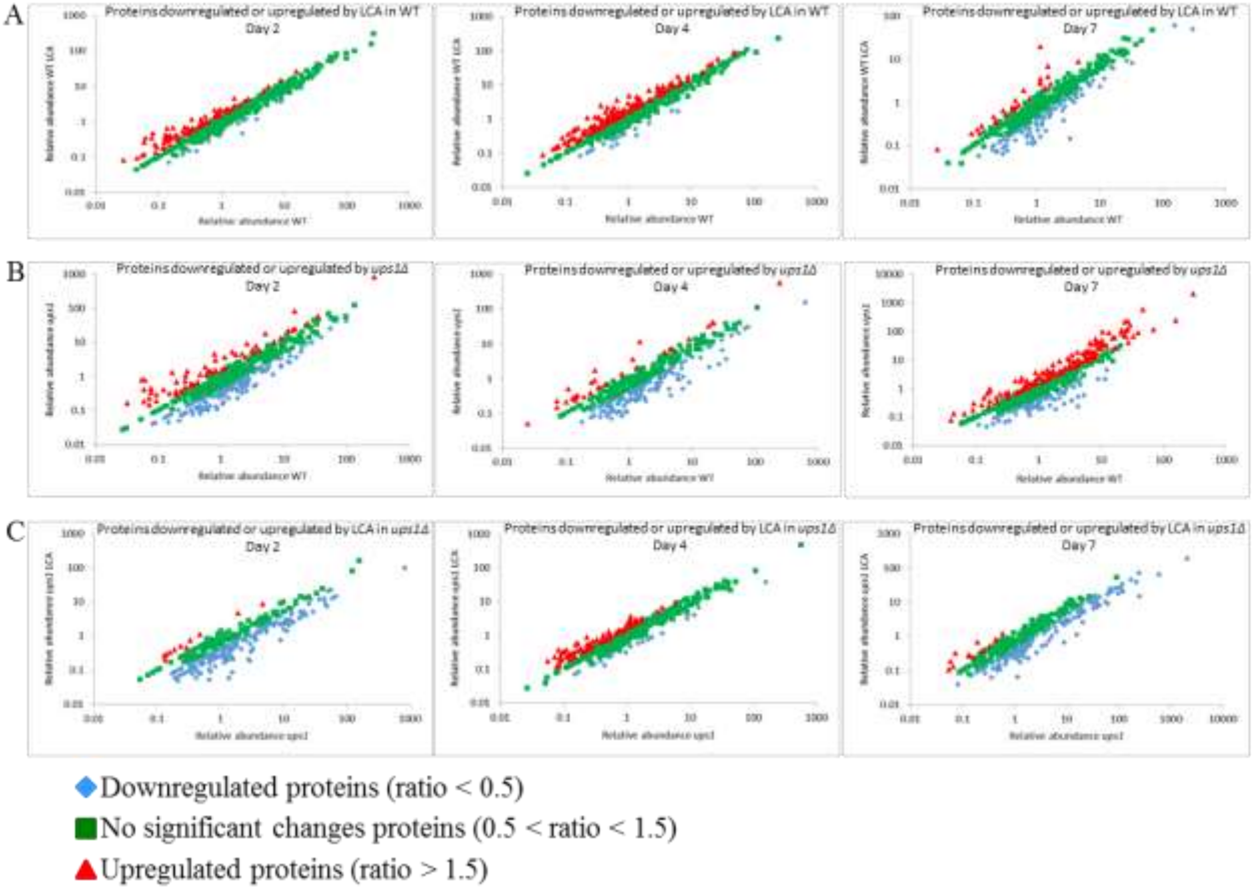

Supplemental Figure S5. Scatter plots comparing the relative concentrations of proteins in mitochondria purified from WT or *ups1Δ* (short-lived) cells cultured with or without LCA. Mitochondria were purified from WT or *ups1Δ* cells recovered on day 2, 4 or 7 of cell culturing. Mass spectrometry-based identification and quantitation of proteins recovered in purified mitochondria were performed as described in Materials and methods. Scatter plots comparing the relative abundance of mitochondrial proteins between specified datasets were plotted on a log-log scale spanning six orders of magnitude. Data on the relative abundance of mitochondrial proteins are presented as means of 2 independent experiments.

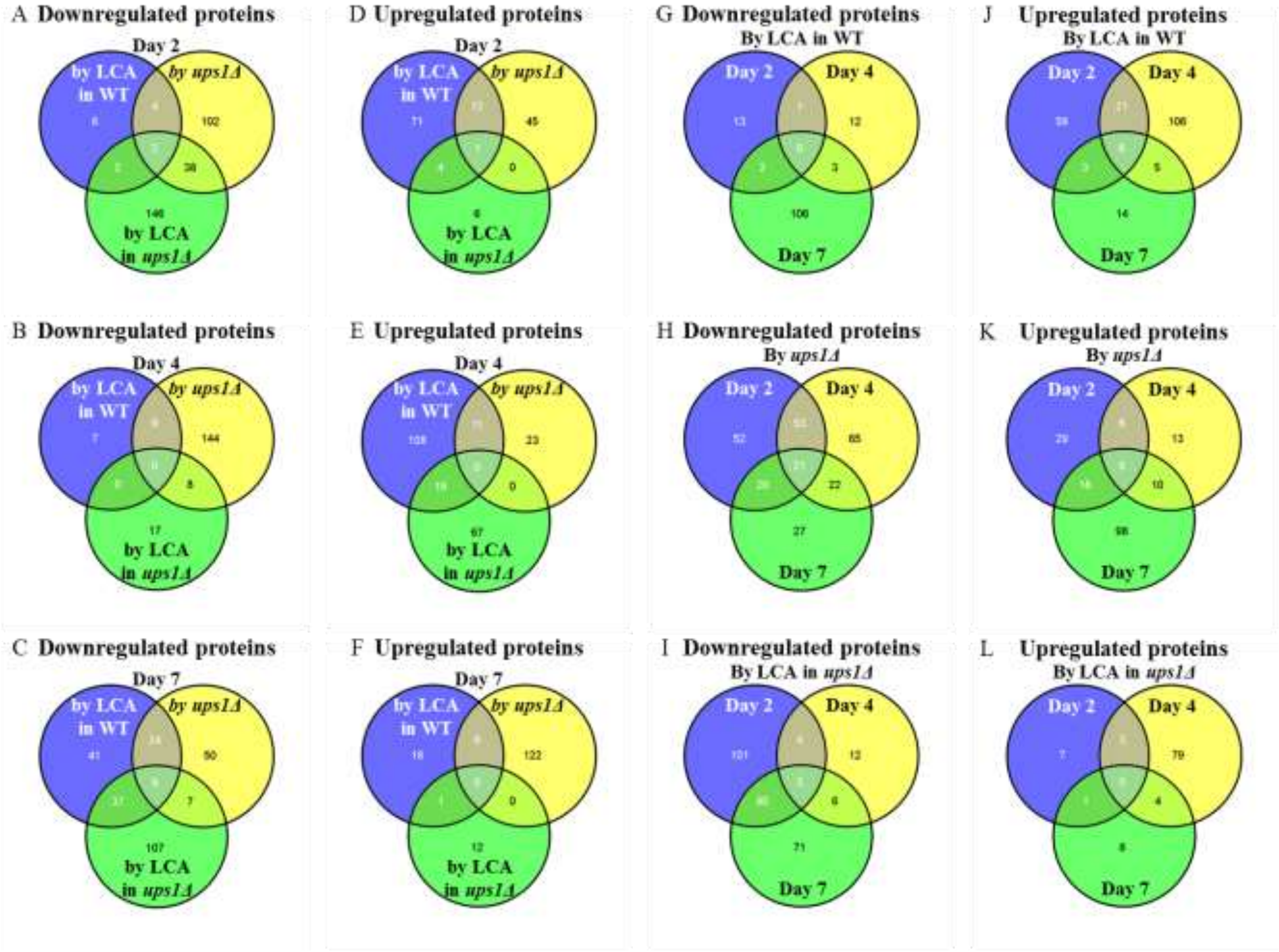

Supplemental Figure S6. The *ups1Δ* mutation alters the concentrations of many mitochondrial proteins in yeast cultured with or without LCA. (**A** – **F**) Venn diagrams showing a comparison of the datasets of relative concentrations of mitochondrial proteins that are statistically significantly downregulated or upregulated by LCA in WT, by the *ups1Δ* mutation in the absence of LCA, and by LCA in *ups1Δ* cells; WT cells were recovered on day 2, 4 or 7 of culturing. (**G** and **J**) Venn diagrams showing a comparison of the datasets of relative concentrations of mitochondrial proteins that are statistically significantly downregulated or upregulated by LCA in WT cells recovered on day 2, 4 or 7 of culturing. (**H** and **K**) Venn diagrams showing a comparison of the datasets of relative concentrations of mitochondrial proteins that are statistically significantly downregulated or upregulated by the *ups1Δ* mutation in the absence of LCA; *ups1Δ* cells were recovered on day 2, 4 or 7 of culturing. (**I** and **L**) Venn diagrams showing a comparison of the datasets of relative concentrations of mitochondrial proteins that are statistically significantly downregulated or upregulated by LCA in *ups1Δ* cells recovered on day 2, 4 or 7 of culturing.

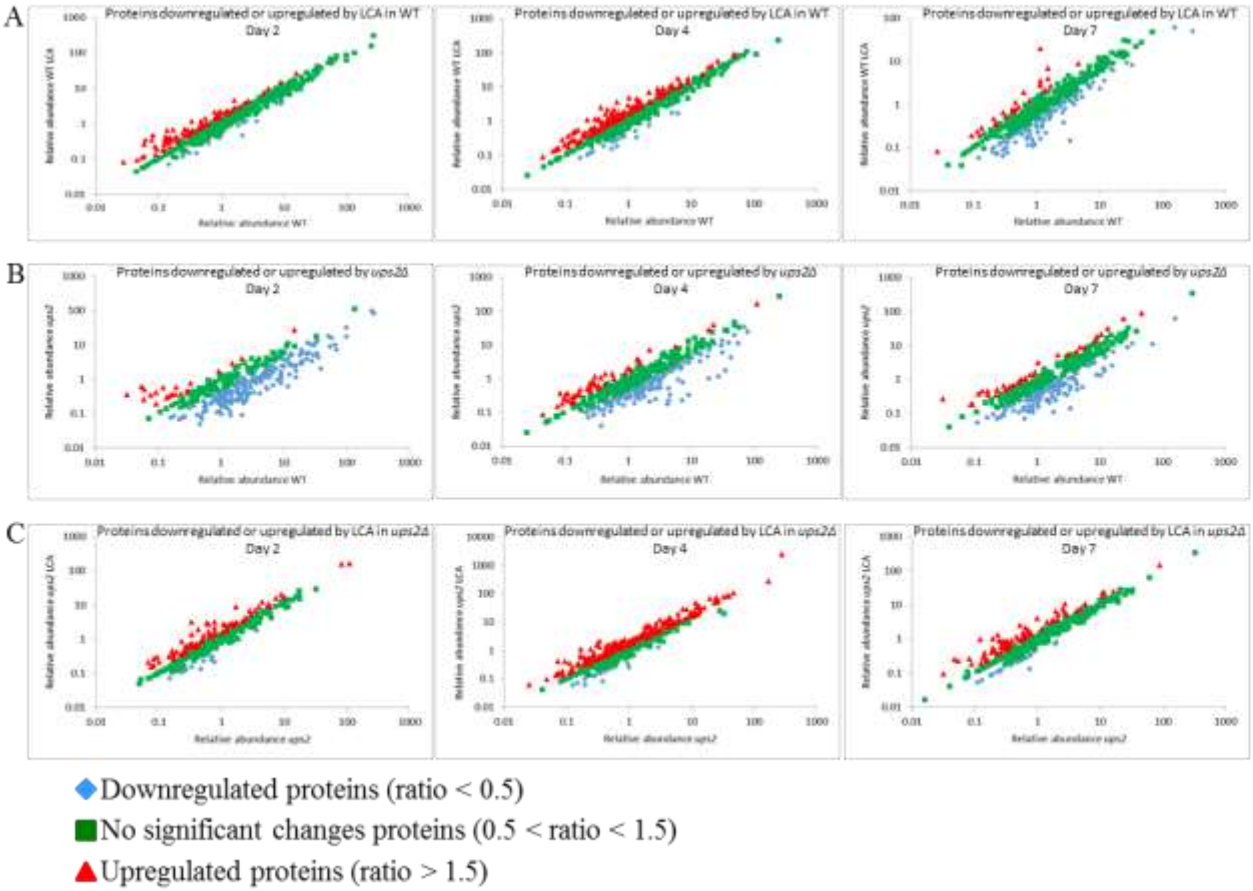

Supplemental Figure S7. Scatter plots comparing the relative concentrations of proteins in mitochondria purified from WT or *ups2Δ* (long-lived) cells cultured with or without LCA. Mitochondria were purified from WT or *ups2Δ* cells recovered on day 2, 4 or 7 of cell culturing. Mass spectrometry-based identification and quantitation of proteins recovered in purified mitochondria were performed as described in Materials and methods. Scatter plots comparing the relative abundance of mitochondrial proteins between specified datasets were plotted on a log-log scale spanning six orders of magnitude. Data on the relative abundance of mitochondrial proteins are presented as means of 2 independent experiments.

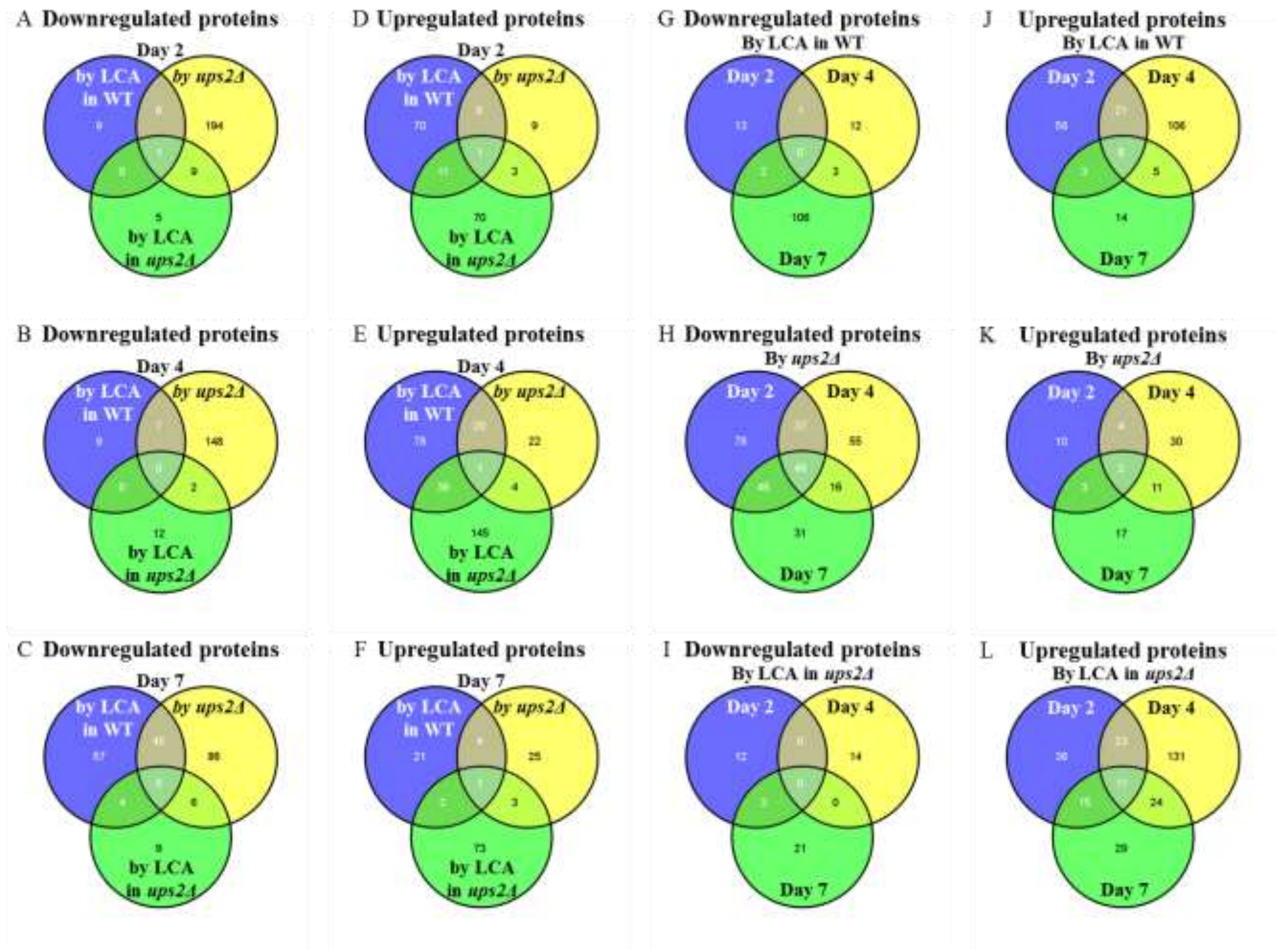

Supplemental Figure S8. The *ups2Δ* mutation alters the concentrations of many mitochondrial proteins in yeast cultured with or without LCA. (**A** – **F**) Venn diagrams showing a comparison of the datasets of relative concentrations of mitochondrial proteins that are statistically significantly downregulated or upregulated by LCA in WT, by the *ups2Δ* mutation in the absence of LCA, and by LCA in *ups2Δ* cells; WT cells were recovered on day 2, 4 or 7 of culturing. (**G** and **J**) Venn diagrams showing a comparison of the datasets of relative concentrations of mitochondrial proteins that are statistically significantly downregulated or upregulated by LCA in WT cells recovered on day 2, 4 or 7 of culturing. (**H** and **K**) Venn diagrams showing a comparison of the datasets of relative concentrations of mitochondrial proteins that are statistically significantly downregulated or upregulated by the *ups2Δ* mutation in the absence of LCA; *ups2Δ* cells were recovered on day 2, 4 or 7 of culturing. (**I** and **L**) Venn diagrams showing a comparison of the datasets of relative concentrations of mitochondrial proteins that are statistically significantly downregulated or upregulated by LCA in *ups2Δ* cells recovered on day 2, 4 or 7 of culturing.

Supplemental Table S1. The relative concentrations of proteins in mitochondria purified from WT cells cultured with or without LCA. Mitochondria were purified from WT cells recovered on day 2 of cell culturing with or without LCA. Mass spectrometry-based identification and quantitation of proteins recovered in purified mitochondria were performed as described in Materials and methods. The exponentially modified protein abundance index (emPAI) provided by the ″Proteome Discoverer″ software was used to calculate the relative abundance of mitochondrial proteins between specified datasets. Data on the relative concentrations of mitochondrial proteins are presented as means of 2 independent experiments.

Accession	Description	Entrez Gene ID	Gene ID	Biological Process	Cellular Component	Molecular Function	emPAI WT	emPAI WT + LCA	Ratio WT + LCA /WT
P01097	ATPase inhibitor, mitochondrial [OS=Saccharomyces cerevisiae S288c]	851347	INH1; YDL181W	metabolic process;regulation of biological process	mitochondrion	catalytic activity;enzyme regulator activity;protein binding	2.162	0.468	0.22
P00420	Cytochrome c oxidase subunit 3 [OS=Saccharomyces cerevisiae S288c]	854627	COX3; Q0275	metabolic process;transport	membrane;mitochondrion	catalytic activity;transporter activity	3.642	1.154	0.32
P47084	MIOREX complex component 12 [OS=Saccharomyces cerevisiae S288c]	853459	MRX12; YJR003C		mitochondrion		0.403	0.145	0.36
P39726	Glycine cleavage system H protein, mitochondrial [OS=Saccharomyces cerevisiae S288c]	851254	GCV3; YAL044C	metabolic process	mitochondrion	catalytic activity	9	3.642	0.40
P07255	Cytochrome c oxidase subunit 7A [OS=Saccharomyces cerevisiae S288c]	851492	COX9; YDL067C	metabolic process;transport	membrane;mitochondrion	catalytic activity;transporter activity	9	3.642	0.40
Q04401	Succinate dehydrogenase assembly factor 3, mitochondrial [OS=Saccharomyces cerevisiae S288c]	852123	ACN9; SDH7; YDR511 W	cell organization and biogenesis;regulation of biological process	mitochondrion;organelle lumen		0.931	0.389	0.42
P49954	probable hydrolase NIT3 [OS=Saccharomyces cerevisiae S288c]	851065	NIT3; YLR351C	metabolic process	cytoplasm;mitochondrion	catalytic activity	0.624	0.274	0.44
P23180	Probable oxidoreductase AIM17 [OS=Saccharomyces cerevisiae S288c]	856365	AIM17; YHL021C	cell organization and biogenesis;metabolic process	mitochondrion	catalytic activity;metal ion binding	9	4.012	0.45
P10507	mitochondrial-processing peptidase subunit beta [OS=Saccharomyces cerevisiae S288c]	850860	MAS1; YLR163C	metabolic process;transport	mitochondrion;organelle lumen	catalytic activity;metal ion binding;protein binding	2.481	1.154	0.47
P38884	Altered inheritance of mitochondria protein 18, mitochondrial [OS=Saccharomyces cerevisiae S288c]	856605	AIM18; YHR198C		mitochondrion	catalytic activity	0.311	0.145	0.47
Q03028	Mitochondrial 2-oxodicarboxylate carrier 1 [OS=Saccharomyces cerevisiae S288c]	855969	ODC1; YPL134C	metabolic process;transport	membrane;mitochondrion	structural molecule activity;transporter activity	7.799	3.642	0.47
P00445	Superoxide dismutase [Cu-Zn] [OS=Saccharomyces cerevisiae S288c]	853568	SOD1; YJR104C	cell organization and biogenesis;cellular homeostasis;metabolic process;regulation of biological process;response to stimulus	cytoplasm;cytosol;mitochondrion;nucleus	antioxidant activity;catalytic activity;metal ion binding;protein binding	1.848	0.874	0.47
Q06005	Octanoyltransferase, mitochondrial [OS=Saccharomyces cerevisiae S288c]	850940	LIP2; YLR239C	metabolic process	cytoplasm;mitochondrion	catalytic activity	0.245	0.116	0.47
P32799	Cytochrome c oxidase subunit 6A, mitochondrial [OS=Saccharomyces cerevisiae S288c]	852684	COX13; YGL191W	cell organization and biogenesis;metabolic process;regulation of biological process;transport	membrane;mitochondrion	catalytic activity;enzyme regulator activity;transporter activity	18.953	9	0.47
P38860	GTPase MTG2, mitochondrial [OS=Saccharomyces cerevisiae S288c]	856573	MTG2; YHR168 W	metabolic process;transport	membrane;mitochondrion	catalytic activity;metal ion binding;nucleotide binding;transporter activity	0.145	0.07	0.48
P40008	Protein FMP52, mitochondrial [OS=Saccharomyces	856721	FMP52; YER004W		endoplasmic reticulum;membrane;mitochondrion	catalytic activity	2.162	1.054	0.49

	cerevisiae S288c]								
P38172	MIOREX complex component 3 [OS=Saccharomyces cerevisiae S288c]	852181	YBL095W ; MRX3; YBL095W		membrane;mitochondrion		0.968	0.501	0.52
P04803	Tryptophan--tRNA ligase, mitochondrial [OS=Saccharomyces cerevisiae S288c]	851861	MSW1; YDR268 W	metabolic process	cytoplasm;mitochondrion;organelle lumen	catalytic activity;nucleotide binding	0.896	0.468	0.52
Q04013	Citrate/oxoglutarate carrier protein [OS=Saccharomyces cerevisiae S288c]	855282	YHM2; YMR241 W	cell organization and biogenesis;metabolic process;transport	membrane;mitochondrion	DNA binding;structural molecule activity;transporter activity	3.467	1.818	0.52
P28625	Protein YIM1 [OS=Saccharomyces cerevisiae S288c]	855183	YIM1; YMR152 W	metabolic process;response to stimulus	cytoplasm;endoplasmic reticulum;mitochondrion	catalytic activity;metal ion binding	0.833	0.438	0.53
P00044	Cytochrome c iso-1 [OS=Saccharomyces cerevisiae S288c]	853507	CYC1; YJR048W	metabolic process;transport	mitochondrion	metal ion binding;protein binding	12.335	6.499	0.53
P40364	Mitochondrial peculiar membrane protein 1 [OS=Saccharomyces cerevisiae S288c]	853379	MPM1; YJL066C		membrane;mitochondrion		3.642	1.929	0.53
P00447	Superoxide dismutase [Mn], mitochondrial [OS=Saccharomyces cerevisiae S288c]	856399	SOD2; YHR008C	metabolic process;response to stimulus	mitochondrion;organelle lumen	antioxidant activity;catalytic activity;metal ion binding	9	4.878	0.54
Q03557	Glutamyl-tRNA(Gln) amidotransferase subunit A, mitochondrial [OS=Saccharomyces cerevisiae S288c]	855338	HER2; YMR293C	cell organization and biogenesis;metabolic process	mitochondrion	catalytic activity;nucleotide binding	0.468	0.259	0.55
P08525	Cytochrome b-c1 complex subunit 8 [OS=Saccharomyces cerevisiae S288c]	853273	QCR8; YJL166W	metabolic process;transport	membrane;mitochondrion	catalytic activity;transporter activity	5.31	2.981	0.56
P19955	37S ribosomal protein YMR-31, mitochondrial [OS=Saccharomyces cerevisiae S288c]	850610	YMR31; YFR049W	cell organization and biogenesis;metabolic process	mitochondrion;ribosome	catalytic activity;protein binding;structural molecule activity	15.681	9	0.57
Q12466	tricalbin-1 [OS=Saccharomyces cerevisiae S288c]	854253	TCB1; YOR086C	cell organization and biogenesis;regulation of biological process	endoplasmic reticulum;membrane;mitochondrion	metal ion binding;protein binding	0.191	0.11	0.58
P33303	Succinate/fumarate mitochondrial transporter [OS=Saccharomyces cerevisiae S288c]	853558	SFC1; YJR095W	metabolic process;transport	membrane;mitochondrion	structural molecule activity;transporter activity	17.957	10.365	0.58
P36516	54S ribosomal protein L3, mitochondrial [OS=Saccharomyces cerevisiae S288c]	855039	MRPL3; YMR024 W	cell organization and biogenesis;metabolic process;response to stimulus	mitochondrion;nucleus;ribosome	catalytic activity;RNA binding;structural molecule activity	1.043	0.61	0.58
P00359	Glyceraldehyde-3-phosphate dehydrogenase 3 [OS=Saccharomyces cerevisiae S288c]	853106	TDH3; YGR192C	cell death;metabolic process	cytoplasm;cytosol;membrane;mitochondrion;nucleus	catalytic activity;nucleotide binding;RNA binding	3.962	2.325	0.59
P32907	Ammonia transport outward protein 2 [OS=Saccharomyces cerevisiae S288c]	855736	ATO2; YNR002C	transport	membrane;mitochondrion;vacuole	transporter activity	0.874	0.52	0.59
P0CX55	40S ribosomal protein S18-A [OS=Saccharomyces cerevisiae S288c]	854982 ; 852061	RPS18B; RPS18A; YML026C ; YDR450 W	metabolic process;transport	cytoplasm;cytosol;mitochondrion;ribosome	RNA binding;structural molecule activity	0.874	0.52	0.59
P25348	54S ribosomal protein L32, mitochondrial [OS=Saccharomyces cerevisiae S288c]	850359	MRPL32; YCR003W	cell organization and biogenesis;metabolic process	membrane;mitochondrion;ribosome	RNA binding;structural molecule activity	0.638	0.389	0.61
P53721	Respiratory supercomplex factor 2, mitochondrial [OS=Saccharomyces cerevisiae S288c]	855752	RCF2; YNR018 W	cell organization and biogenesis	membrane;mitochondrion		7.111	4.337	0.61
Q06630	Mitochondrial homologous recombination protein 1 [OS=Saccharomyces cerevisiae S288c]	851890	MHR1; YDR296 W	cell organization and biogenesis;metabolic process;regulation of biological process;response to stimulus	mitochondrion;nucleus	catalytic activity;DNA binding;structural molecule activity	0.585	0.359	0.61
Q12375	mitochondrial ornithine transporter 1 [OS=Saccharomyces cerevisiae S288c]	854297	ORT1; YOR130C	metabolic process;transport	membrane;mitochondrion	structural molecule activity;transporter activity	0.54	0.334	0.62
P53081	NGG1-interacting factor 3 [OS=Saccharomyces cerevisiae S288c]	852651	NIF3; YGL221C		cytoplasm;mitochondrion		0.501	0.311	0.62
P36139	protein PET10 [OS=Saccharomyces cerevisiae S288c]	853920	PET10; YKR046C	metabolic process	membrane		0.413	0.259	0.63
P00830	ATP synthase subunit beta,	853585	ATP2;	metabolic	cytosol;membrane;mitochondrion	catalytic	99	62.096	0.63

	mitochondrial [OS=Saccharomyces cerevisiae S288c]		YJR121W	process;transport		activity;nucleotide binding;protein binding;transporter activity			
P50088	Stationary phase gene 1 protein [OS=Saccharomyces cerevisiae S288c]	853151	SPG1; YGR236C		endoplasmic reticulum;membrane;mitochondrion		250.189	157.489	0.63
P37292	Serine hydroxymethyltransferase, mitochondrial [OS=Saccharomyces cerevisiae S288c]	852565	SHM1; YBR263W	metabolic process	mitochondrion	catalytic activity	12.219	7.697	0.63
Q01163	37S ribosomal protein S23, mitochondrial [OS=Saccharomyces cerevisiae S288c]	852748	RSM23; YGL129C	cell organization and biogenesis;metabolic process	mitochondrion;ribosome	catalytic activity;nucleotide binding;RNA binding;structural molecule activity	2.695	1.707	0.63
P04806	Hexokinase-1 [OS=Saccharomyces cerevisiae S288c]	850614	HXK1; YFR053C	cellular homeostasis;metabolic process;transport	cytoplasm;cytosol;mitochondrion	catalytic activity;nucleotide binding	0.269	0.172	0.64
P40051	Intermediate cleaving peptidase 55 [OS=Saccharomyces cerevisiae S288c]	856811	ICP55; YER078C	metabolic process;regulation of biological process	membrane;mitochondrion;nucleus	catalytic activity;metal ion binding	0.25	0.16	0.64
P47158	Putative transferase CAF17, mitochondrial [OS=Saccharomyces cerevisiae S288c]	853586	IBA57; YJR122W	cell organization and biogenesis;metabolic process	mitochondrion;organelle lumen	catalytic activity	0.241	0.155	0.64
P38714	Arginine--tRNA ligase, mitochondrial [OS=Saccharomyces cerevisiae S288c]	856491	MSR1; YHR091C	cell organization and biogenesis;metabolic process	cytoplasm;mitochondrion;organelle lumen	catalytic activity;nucleotide binding	0.174	0.113	0.65
Q96VH5	MICOS complex subunit Mic10 [OS=Saccharomyces cerevisiae S288c]	850300	MOS1; MIC10; YCL057C-A	cell organization and biogenesis	membrane;mitochondrion		4.179	2.728	0.65
Q00711	Succinate dehydrogenase [ubiquinone] flavoprotein subunit, mitochondrial [OS=Saccharomyces cerevisiae S288c]	853709	SDH1; YKL148C	metabolic process;transport	membrane;mitochondrion	catalytic activity;nucleotide binding;protein binding	18.619	12.242	0.66
P00431	cytochrome c peroxidase, mitochondrial [OS=Saccharomyces cerevisiae S288c]	853940	CCP1; YKR066C	metabolic process;response to stimulus	mitochondrion;organelle lumen	antioxidant activity;catalytic activity;metal ion binding;protein binding	2.162	1.424	0.66
P40961	prohibitin-1 [OS=Saccharomyces cerevisiae S288c]	853033	PHB1; YGR132C	cell organization and biogenesis;metabolic process;regulation of biological process	membrane;mitochondrion	protein binding	9	5.952	0.66
P39926	Protein SSO2 [OS=Saccharomyces cerevisiae S288c]	855221	SSO2; YMR183C	cell organization and biogenesis;transport	cytoplasm;endoplasmic reticulum;membrane	protein binding	3.642	2.415	0.66
P38072	Protein SCO2, mitochondrial [OS=Saccharomyces cerevisiae S288c]	852312	SCO2; YBR024W	cell organization and biogenesis;cellular homeostasis;metabolic process;transport	membrane;mitochondrion	antioxidant activity;catalytic activity;metal ion binding	1.404	0.931	0.66
P04710	ADP,ATP carrier protein 1 [OS=Saccharomyces cerevisiae S288c]	855078	AAC1; YMR056C	metabolic process;transport	cytosol;membrane;mitochondrion	structural molecule activity;transporter activity	10.288	6.848	0.67
P17695	Glutaredoxin-2, mitochondrial [OS=Saccharomyces cerevisiae S288c]	852124	GRX2; YDR513W	cellular homeostasis;metabolic process;regulation of biological process;response to stimulus;transport	cytoplasm;cytosol;mitochondrion;nucleus	antioxidant activity;catalytic activity	1.31	0.874	0.67
P14832	peptidyl-prolyl cis-trans isomerase [OS=Saccharomyces cerevisiae S288c]	851733	CPR1; YDR155C	cell differentiation;cell organization and biogenesis;metabolic process;regulation of biological process;transport	cytoplasm;mitochondrion;nucleus	catalytic activity;RNA binding	1.31	0.874	0.67
P23641	mitochondrial phosphate carrier protein [OS=Saccharomyces cerevisiae S288c]	853540	MIR1; YJR077C	metabolic process;transport	membrane;mitochondrion	protein binding;structural molecule activity;transporter activity	26.826	17.957	0.67
P25642	54S ribosomal protein IMG2, mitochondrial [OS=Saccharomyces cerevisiae S288c]	850434	IMG2; YCR071C	cell organization and biogenesis;metabolic process	mitochondrion;ribosome	structural molecule activity	1.154	0.778	0.67
P36151	Uncharacterized protein YKR070W [OS=Saccharomyces cerevisiae S288c]	853944	YKR070W; YKR070W	metabolic process	mitochondrion		1.976	1.336	0.68
Q04487	Mitochondrial inner membrane protein SHH3 [OS=Saccharomyces cerevisiae S288c]	855145	SHH3; YMR118C	metabolic process	membrane;mitochondrion	catalytic activity;metal ion binding	1.031	0.701	0.68
Q12428	Probable 2-methylcitrate dehydratase	856108	PDH1; YPR002W	metabolic process	cytoplasm;membrane;mitochondrion	catalytic activity	19.535	13.33	0.68

	[OS=Saccharomyces cerevisiae S288c]								
P10823	Guanine nucleotide-binding protein alpha-2 subunit [OS=Saccharomyces cerevisiae S288c]	856741	GPA2; YER020W	cell differentiation;cell growth;cell organization and biogenesis;cellular homeostasis;regulation of biological process;response to stimulus	cytosol;membrane;mitochondrion	catalytic activity;metal ion binding;nucleotide binding;protein binding;signal transducer activity	0.978	0.668	0.68
P07143	Cytochrome c1, heme protein, mitochondrial [OS=Saccharomyces cerevisiae S288c]	854231	CYT1; YOR065 W	metabolic process;transport	cytosol;membrane;mitochondrion	metal ion binding	19.309	13.251	0.69
P36517	54S ribosomal protein L4, mitochondrial [OS=Saccharomyces cerevisiae S288c]	851160	MRPL4; YLR439W	cell organization and biogenesis;metabolic process	mitochondrion;ribosome	structural molecule activity	1.565	1.081	0.69
P38523	GrpE protein homolog, mitochondrial [OS=Saccharomyces cerevisiae S288c]	854407	MGE1; YOR232 W	metabolic process;regulation of biological process;transport	membrane;mitochondrion;organelle lumen	enzyme regulator activity;nucleotide binding;protein binding	2.36	1.637	0.69
P32316	acetyl-CoA hydrolase [OS=Saccharomyces cerevisiae S288c]	852266	ACH1; YBL015W	metabolic process	cytoplasm;cytosol;mitochondrion	catalytic activity	29.539	20.544	0.70
P36017	Vacuolar protein sorting-associated protein 21 [OS=Saccharomyces cerevisiae S288c]	854256	VPS21; YOR089C	cell organization and biogenesis;regulation of biological process;response to stimulus;transport	cytosol;endosome;membrane;mitochondrion	catalytic activity;nucleotide binding	0.719	0.501	0.70
P32860	NifU-like protein, mitochondrial [OS=Saccharomyces cerevisiae S288c]	853826	NFU1; YKL040C	cell organization and biogenesis;cellular homeostasis;metabolic process	mitochondrion;organelle lumen	metal ion binding	5.494	3.87	0.70
P38122	3-methyl-2-oxobutanoate hydroxymethyltransferase [OS=Saccharomyces cerevisiae S288c]	852474	ECM31; YBR176W	metabolic process	mitochondrion	catalytic activity	0.585	0.413	0.71
P00425	Cytochrome c oxidase polypeptide 5B, mitochondrial [OS=Saccharomyces cerevisiae S288c]	854695	COX5B; YIL111W	metabolic process;transport	membrane;mitochondrion	catalytic activity;transporter activity	1.848	1.31	0.71
P06168	ketol-acid reductoisomerase, mitochondrial [OS=Saccharomyces cerevisiae S288c]	851069	ILV5; YLR355C	cell organization and biogenesis;metabolic process	mitochondrion	catalytic activity;DNA binding;metal ion binding	28.126	19.962	0.71
P00358	glyceraldehyde-3-phosphate dehydrogenase 2 [OS=Saccharomyces cerevisiae S288c]	853465	TDH2; YJR009C	cell death;metabolic process	cytoplasm;cytosol;membrane;mitochondrion;nucleus	catalytic activity;nucleotide binding;protein binding	1.721	1.228	0.71
P32340	Rotenone-insensitive NADH-ubiquinone oxidoreductase, mitochondrial [OS=Saccharomyces cerevisiae S288c]	854919	NDI1; YML120C	metabolic process;regulation of biological process	membrane;mitochondrion;organelle lumen	catalytic activity;nucleotide binding	22.95	16.433	0.72
P09950	5-aminolevulinate synthase, mitochondrial [OS=Saccharomyces cerevisiae S288c]	851818	HEM1; YDR232 W	metabolic process	mitochondrion;organelle lumen	catalytic activity	0.389	0.28	0.72
P81449	ATP synthase subunit e, mitochondrial [OS=Saccharomyces cerevisiae S288c]	851922	TIM11; YDR322C -A	cell organization and biogenesis;metabolic process;transport	membrane;mitochondrion	catalytic activity;structural molecule activity;transporter activity	9	6.499	0.72
P08425	phenylalanine--tRNA ligase, mitochondrial [OS=Saccharomyces cerevisiae S288c]	856160	MSF1; YPR047W	metabolic process	cytoplasm;mitochondrion;organelle lumen	catalytic activity;metal ion binding;nucleotide binding;RNA binding	0.346	0.25	0.72
P19882	heat shock protein 60, mitochondrial [OS=Saccharomyces cerevisiae S288c]	850963	HSP60; YLR259C	cell organization and biogenesis;metabolic process;regulation of biological process;transport	cytoplasm;cytosol;membrane;mitochondrion;organelle lumen	catalytic activity;DNA binding;nucleotide binding;protein binding	23.816	17.33	0.73
P18239	ADP,ATP carrier protein 2 [OS=Saccharomyces cerevisiae S288c]	852250	PET9; YBL030C	cell death;metabolic process;transport	membrane;mitochondrion	structural molecule activity;transporter activity	135.88 7	99	0.73
Q04438	Stationary phase protein 4 [OS=Saccharomyces cerevisiae S288c]	855134	SPG4; YMR107 W				4.995	3.642	0.73
P32317	Protein AFG1 [OS=Saccharomyces cerevisiae S288c]	856658	AFG1; YEL052W	cell organization and biogenesis;metabolic process;response to stimulus;transport	membrane;mitochondrion	catalytic activity;nucleotide binding	1.154	0.848	0.73
Q06892	NADH kinase POS5, mitochondrial [OS=Saccharomyces cerevisiae S288c]	855913	POS5; YPL188W	metabolic process;response to stimulus	mitochondrion;organelle lumen	catalytic activity;nucleotide binding	2.511	1.848	0.74
P32445	Single-stranded DNA-binding protein RIM1, mitochondrial [OS=Saccharomyces	850395	RIM1; YCR028C-A	cell organization and biogenesis;metabolic process;regulation of	mitochondrion	DNA binding	6.743	4.995	0.74

	cerevisiae S288c]			biological process					
Q01217	Protein ARG5,6, mitochondrial [OS=Saccharomyces cerevisiae S288c]	856800	ARG5,6; YER069W	metabolic process;regulation of biological process	cytoplasm;mitochondrion;organelle lumen	catalytic activity;nucleotide binding;protein binding	0.458	0.341	0.74
P00401	Cytochrome c oxidase subunit 1 [OS=Saccharomyces cerevisiae S288c]	854598	COX1; Q0045	metabolic process;transport	membrane;mitochondrion	catalytic activity;metal ion binding;transporter activity	2.162	1.61	0.74
P43617	Uncharacterized mitochondrial carrier YFR045W [OS=Saccharomyces cerevisiae S288c]	850606	YFR045W ; YFR045W	metabolic process;transport	membrane;mitochondrion	structural molecule activity;transporter activity	0.778	0.585	0.75
P32898	Mitochondrial presequence protease [OS=Saccharomyces cerevisiae S288c]	852041	CYM1; YDR430C	metabolic process	mitochondrion	catalytic activity;metal ion binding	2.162	1.626	0.75
P21801	Succinate dehydrogenase [ubiquinone] iron-sulfur subunit, mitochondrial [OS=Saccharomyces cerevisiae S288c]	850685	SDH2; YLL041C	metabolic process;transport	membrane;mitochondrion	catalytic activity;metal ion binding	32.839	24.809	0.76
P15179	Aspartate--tRNA ligase, mitochondrial [OS=Saccharomyces cerevisiae S288c]	856000	MSD1; YPL104W	metabolic process	cytoplasm;mitochondrion;organelle lumen	catalytic activity;nucleotide binding	0.708	0.535	0.76
Q12031	Mitochondrial 2-methylisocitrate lyase [OS=Saccharomyces cerevisiae S288c]	856114	ICL2; YPR006C	metabolic process	mitochondrion;organelle lumen	catalytic activity	7.859	5.952	0.76
P40047	Aldehyde dehydrogenase 5, mitochondrial [OS=Saccharomyces cerevisiae S288c]	856804	ALD5; YER073W	metabolic process	mitochondrion;organelle lumen	catalytic activity	3.146	2.384	0.76
P40341	Mitochondrial respiratory chain complexes assembly protein YTA12 [OS=Saccharomyces cerevisiae S288c]	855114	YTA12; YMR089C	cell organization and biogenesis;metabolic process;response to stimulus;transport	cytoplasm;membrane;mitochondrion	catalytic activity;metal ion binding;nucleotide binding;protein binding	1.795	1.371	0.76
P25605	Acetolactate synthase small subunit, mitochondrial [OS=Saccharomyces cerevisiae S288c]	850348	ILV6; YCL009C	metabolic process;regulation of biological process	mitochondrion	catalytic activity;enzyme regulator activity	11.743	9	0.77
P40185	Protein mmf1, mitochondrial [OS=Saccharomyces cerevisiae S288c]	854760	MMF1; YIL051C	cell organization and biogenesis;metabolic process	mitochondrion;organelle lumen		9	6.943	0.77
P36060	NADH-cytochrome b5 reductase 2 [OS=Saccharomyces cerevisiae S288c]	853707	MCR1; YKL150W	metabolic process;response to stimulus	membrane;mitochondrion	catalytic activity;protein binding	19.691	15.238	0.77
P37293	N-terminal acetyltransferase 2 [OS=Saccharomyces cerevisiae S288c]	853050	NAT2; YGR147C	metabolic process	cytoplasm;mitochondrion	catalytic activity	0.931	0.73	0.78
Q07651	SUR7 family protein FMP45 [OS=Saccharomyces cerevisiae S288c]	851304	FMP45; YDL222C	cell differentiation;cell organization and biogenesis	membrane;mitochondrion		3.125	2.455	0.79
P38797	Protein phosphatase 2C homolog 7, mitochondrial [OS=Saccharomyces cerevisiae S288c]	856475	PTC7; YHR076 W	metabolic process	mitochondrion	catalytic activity;metal ion binding;protein binding	0.874	0.688	0.79
P00360	glyceraldehyde-3-phosphate dehydrogenase 1 [OS=Saccharomyces cerevisiae S288c]	853395	TDH1; YJL052W	metabolic process	cytoplasm;cytosol;membrane;mitochondrion	catalytic activity;nucleotide binding	0.823	0.65	0.79
P53312	Succinyl-CoA ligase [ADP-forming] subunit beta, mitochondrial [OS=Saccharomyces cerevisiae S288c]	853159	LSC2; YGR244C	metabolic process	mitochondrion	catalytic activity;nucleotide binding	20.017	15.819	0.79
P07253	cytochrome B pre-mRNA-processing protein 6 [OS=Saccharomyces cerevisiae S288c]	852417	CBP6; YBR120C	cell organization and biogenesis;metabolic process;regulation of biological process	mitochondrion;ribosome		2.728	2.162	0.79
P00175	Cytochrome b2, mitochondrial [OS=Saccharomyces cerevisiae S288c]	854950	CYB2; YML054C	metabolic process;transport	cytosol;membrane;mitochondrion;nucleus	catalytic activity;metal ion binding;nucleotide binding	1.113	0.884	0.79
P25646	[Pyruvate dehydrogenase [acetyl-transferring]]-phosphatase 2, mitochondrial [OS=Saccharomyces cerevisiae S288c]	850441	PTC6; YCR079W	cell communication;metabolic process;regulation of biological process;response to stimulus	mitochondrion;organelle lumen	catalytic activity	0.61	0.487	0.80
P00560	Phosphoglycerate kinase [OS=Saccharomyces cerevisiae S288c]	850370	PGK1; YCR012W	metabolic process	cytoplasm;membrane;mitochondrion	catalytic activity;nucleotide binding	2.415	1.929	0.80
P38891	Branched-chain-amino-acid aminotransferase, mitochondrial [OS=Saccharomyces	856615	BAT1; YHR208 W	metabolic process;regulation of biological process;response to	mitochondrion;organelle lumen	catalytic activity	15.876	12.689	0.80

	cerevisiae S288c]			stimulus					
P47052	succinate dehydrogenase [ubiquinone] flavoprotein subunit 2, mitochondrial [OS=Saccharomyces cerevisiae S288c]	853405	YJL045W; YJL045W	metabolic process;transport	membrane;mitochondrion	catalytic activity;nucleotide binding	5.884	4.712	0.80
P32891	D-lactate dehydrogenase [cytochrome] 1, mitochondrial [OS=Saccharomyces cerevisiae S288c]	851380	DLD1; YDL174C	metabolic process;transport	membrane;mitochondrion	catalytic activity;nucleotide binding	13.03	10.45	0.80
P53736	Uncharacterized protein YNR040W [OS=Saccharomyces cerevisiae S288c]	855776	YNR040 W; YNR040 W		mitochondrion		1.154	0.931	0.81
P36059	ATP-dependent (S)-NAD(P)H-hydrate dehydratase [OS=Saccharomyces cerevisiae S288c]	853706	YKL151C; YKL151C	metabolic process	cytoplasm	catalytic activity;nucleotide binding	1.081	0.874	0.81
P23833	Protein SCO1, mitochondrial [OS=Saccharomyces cerevisiae S288c]	852325	SCO1; YBR037C	cell organization and biogenesis;cellular homeostasis;response to stimulus;transport	membrane;mitochondrion	antioxidant activity;catalytic activity;metal ion binding	1.783	1.448	0.81
P33416	Heat shock protein 78, mitochondrial [OS=Saccharomyces cerevisiae S288c]	851845	HSP78; YDR258C	cell organization and biogenesis;metabolic process;regulation of biological process;response to stimulus	mitochondrion;organelle lumen	catalytic activity;nucleotide binding;protein binding	12.525	10.199	0.81
P43635	Citrate synthase 3, mitochondrial [OS=Saccharomyces cerevisiae S288c]	856107	CIT3; YPR001W	metabolic process	mitochondrion	catalytic activity	6.11	4.995	0.82
P12686	37S ribosomal protein MRP13, mitochondrial [OS=Saccharomyces cerevisiae S288c]	852975	MRP13; YGR084C	cell organization and biogenesis;metabolic process	mitochondrion;ribosome	structural molecule activity	1.512	1.239	0.82
P07251	ATP synthase subunit alpha, mitochondrial [OS=Saccharomyces cerevisiae S288c]	852177	ATP1; YBL099W	metabolic process;transport	cytosol;membrane;mitochondrion	catalytic activity;nucleotide binding;transporter activity	34.938	28.663	0.82
P53219	Abhydrolase domain-containing protein IMO32 [OS=Saccharomyces cerevisiae S288c]	852919	IMO32; YGR031 W	metabolic process;transport	mitochondrion	catalytic activity	1.404	1.154	0.82
P00924	Enolase 1 [OS=Saccharomyces cerevisiae S288c]	853169	ENO1; YGR254 W	metabolic process;regulation of biological process	cytoplasm;cytosol;membrane;mitochondrion;vacuole	catalytic activity;metal ion binding;protein binding	0.711	0.585	0.82
P08539	Guanine nucleotide-binding protein alpha-1 subunit [OS=Saccharomyces cerevisiae S288c]	856394	GPA1; YHR005C	cell organization and biogenesis;cellular component movement;regulation of biological process;response to stimulus;transport	cytosol;endosome;membrane	catalytic activity;metal ion binding;nucleotide binding;protein binding;signal transducer activity	0.682	0.562	0.82
P32902	37S ribosomal protein MRP4, mitochondrial [OS=Saccharomyces cerevisiae S288c]	856384	MRP4; YHL004W	cell organization and biogenesis;metabolic process	mitochondrion;ribosome	structural molecule activity	1.154	0.957	0.83
P18496	Mitochondrial ATPase complex subunit ATP10 [OS=Saccharomyces cerevisiae S288c]	851109	ATP10; YLR393W	cell organization and biogenesis	cytoplasm;membrane;mitochondrion	protein binding	1.818	1.512	0.83
P21560	Protein CBP3, mitochondrial [OS=Saccharomyces cerevisiae S288c]	855886	CBP3; YPL215W	cell organization and biogenesis;regulation of biological process	membrane;mitochondrion;ribosome		1.031	0.859	0.83
P35191	DnaJ homolog 1, mitochondrial [OS=Saccharomyces cerevisiae S288c]	850530	MDJ1; YFL016C	cell organization and biogenesis;metabolic process;regulation of biological process;response to stimulus	mitochondrion;organelle lumen	enzyme regulator activity;metal ion binding;nucleotide binding;protein binding	4.722	3.977	0.84
P49095	Glycine dehydrogenase (Decarboxylating), mitochondrial [OS=Saccharomyces cerevisiae S288c]	855227	GCV2; YMR189 W	metabolic process	cytosol;mitochondrion	catalytic activity	2.884	2.433	0.84
P33310	ATP-dependent permease MDL1, mitochondrial [OS=Saccharomyces cerevisiae S288c]	850885	MDL1; YLR188W	transport	membrane;mitochondrion	catalytic activity;nucleotide binding;transporter activity	0.624	0.528	0.85
Q01574	acetyl-coenzyme A synthetase 1 [OS=Saccharomyces cerevisiae S288c]	851245	ACS1; YAL054C	cell organization and biogenesis;metabolic process	cytoplasm;cytosol;endoplasmic reticulum;mitochondrion;nucleus	catalytic activity;nucleotide binding	0.624	0.528	0.85
P53170	[Pyruvate dehydrogenase (acetyl-transferring)] kinase 2, mitochondrial [OS=Saccharomyces	852821	PKP2; YGL059W	metabolic process;regulation of biological process	mitochondrion;organelle lumen	catalytic activity;nucleotide binding;protein binding	1.096	0.931	0.85

	cerevisiae S288c]								
P23291	Casein kinase I homolog 1 [OS=Saccharomyces cerevisiae S288c]	856537	YCK1; YHR135C	cell organization and biogenesis;metabolic process;regulation of biological process;response to stimulus;transport	cytoplasm;endoplasmic reticulum;membrane;mitochondrion;nucleus	catalytic activity;nucleotide binding;protein binding	1.043	0.887	0.85
P00925	Enolase 2 [OS=Saccharomyces cerevisiae S288c]	856579	ENO2; YHR174 W	metabolic process;regulation of biological process	cytoplasm;cytosol;membrane;mitochondrion;vacuole	catalytic activity;metal ion binding;protein binding	1.043	0.887	0.85
Q06678	54S ribosomal protein L35, mitochondrial [OS=Saccharomyces cerevisiae S288c]	851921	MRPL35; YDR322 W	cell organization and biogenesis;metabolic process	mitochondrion;ribosome	structural molecule activity	1.424	1.219	0.86
P25349	Flavoprotein-like protein YCP4 [OS=Saccharomyces cerevisiae S288c]	850360	YCP4; YCR004C	metabolic process;regulation of biological process	cytoplasm;membrane;mitochondrion	catalytic activity;nucleotide binding;protein binding	6.743	5.813	0.86
P53252	sphingolipid long chain base-responsive protein PIL1 [OS=Saccharomyces cerevisiae S288c]	852977	PIL1; YGR086C	cell organization and biogenesis;regulation of biological process;response to stimulus;transport	cytoplasm;membrane;mitochondrion	protein binding	14.013	12.111	0.86
P40086	Cytochrome c oxidase assembly protein cox15 [OS=Saccharomyces cerevisiae S288c]	856884	COX15; YER141W	cell organization and biogenesis;metabolic process;transport	membrane;mitochondrion	catalytic activity;transporter activity	1.555	1.346	0.87
P09457	ATP synthase subunit 5, mitochondrial [OS=Saccharomyces cerevisiae S288c]	851892	ATP5; YDR298C	metabolic process;transport	membrane;mitochondrion	catalytic activity;transporter activity	9	7.799	0.87
P46367	Potassium-activated aldehyde dehydrogenase, mitochondrial [OS=Saccharomyces cerevisiae S288c]	854556	ALD4; YOR374 W	metabolic process	mitochondrion;organelle lumen	catalytic activity	58.078	50.795	0.87
P04840	Mitochondrial outer membrane protein porin 1 [OS=Saccharomyces cerevisiae S288c]	855669	POR1; YNL055C	cell death;cell organization and biogenesis;cellular homeostasis;regulation of biological process;transport	cytoplasm;membrane;mitochondrion	transporter activity	15.238	13.384	0.88
P28241	Isocitrate dehydrogenase [NAD] subunit 2, mitochondrial [OS=Saccharomyces cerevisiae S288c]	854303	IDH2; YOR136 W	metabolic process	mitochondrion;organelle lumen	catalytic activity;metal ion binding;nucleotide binding;protein binding;RNA binding	6.197	5.449	0.88
P50085	Prohibitin-2 [OS=Saccharomyces cerevisiae S288c]	853146	PHB2; YGR231C	cell organization and biogenesis;metabolic process;regulation of biological process	membrane;mitochondrion	protein binding	4.926	4.337	0.88
P07246	Alcohol dehydrogenase 3, mitochondrial [OS=Saccharomyces cerevisiae S288c]	855107	ADH3; YMR083 W	metabolic process	mitochondrion;organelle lumen	catalytic activity;metal ion binding	10.159	9	0.89
P46681	D-lactate dehydrogenase [cytochrome] 2, mitochondrial [OS=Saccharomyces cerevisiae S288c]	851376	DLD2; YDL178W	metabolic process	mitochondrion;organelle lumen	catalytic activity;nucleotide binding;protein binding	1.829	1.626	0.89
P40495	Homoisocitrate dehydrogenase, mitochondrial [OS=Saccharomyces cerevisiae S288c]	854714	LYS12; YIL094C	metabolic process	mitochondrion	catalytic activity;metal ion binding;nucleotide binding	6.499	5.813	0.89
P17505	Malate dehydrogenase, mitochondrial [OS=Saccharomyces cerevisiae S288c]	853777	MDH1; YKL085W	metabolic process	mitochondrion;organelle lumen	catalytic activity;protein binding;RNA binding	99	88.615	0.90
P48015	Aminomethyltransferase, mitochondrial [OS=Saccharomyces cerevisiae S288c]	851582	GCV1; YDR019C	metabolic process	mitochondrion	catalytic activity;protein binding	11.217	10.053	0.90
P53326	Uncharacterized protein YGR266W [OS=Saccharomyces cerevisiae S288c]	853182	YGR266 W; YGR266 W		membrane;mitochondrion		1.081	0.974	0.90
P33311	ATP-dependent permease MDL2, mitochondrial [OS=Saccharomyces cerevisiae S288c]	855858	MDL2; YPL270W	response to stimulus;transport	membrane;mitochondrion	catalytic activity;nucleotide binding;transporter activity	1.233	1.116	0.91
P20967	2-oxoglutarate dehydrogenase, mitochondrial [OS=Saccharomyces cerevisiae S288c]	854681	KGD1; YIL125W	metabolic process	cytosol;mitochondrion;organelle lumen	catalytic activity	13.785	12.554	0.91
P36013	NAD-dependent malic enzyme, mitochondrial [OS=Saccharomyces	853839	MAE1; YKL029C	metabolic process	mitochondrion;organelle lumen	catalytic activity;metal ion binding;nucleotide	2.35	2.162	0.92

	cerevisiae S288c]					binding			
Q12166	2-isopropylmalate synthase 2, mitochondrial [OS=Saccharomyces cerevisiae S288c]	854275	LEU9; YOR108 W	metabolic process	mitochondrion	catalytic activity;protein binding	3.739	3.453	0.92
P16387	Pyruvate dehydrogenase E1 component subunit alpha, mitochondrial [OS=Saccharomyces cerevisiae S288c]	856925	PDA1; YER178W	cell growth;metabolic process	mitochondrion;organelle lumen	catalytic activity	25.264	23.384	0.93
P00927	Threonine dehydratase, mitochondrial [OS=Saccharomyces cerevisiae S288c]	856819	ILV1; YER086W	metabolic process	cytoplasm;mitochondrion	catalytic activity	3.375	3.125	0.93
P32795	Mitochondrial inner membrane i-AAA protease supercomplex subunit YME1 [OS=Saccharomyces cerevisiae S288c]	856135	YME1; YPR024W	metabolic process;response to stimulus;transport	membrane;mitochondrion	catalytic activity;metal ion binding;nucleotide binding;protein binding	1.938	1.798	0.93
P39522	Dihydroxy-acid dehydratase, mitochondrial [OS=Saccharomyces cerevisiae S288c]	853473	ILV3; YJR016C	metabolic process	mitochondrion	catalytic activity;metal ion binding	39.37	36.649	0.93
Q08179	Mitochondrial distribution and morphology protein 38 [OS=Saccharomyces cerevisiae S288c]	854130	MDM38; YOL027C	cell organization and biogenesis;cellular homeostasis;regulation of biological process;transport	membrane;mitochondrion		5.683	5.31	0.93
P19657	plasma membrane ATPase 2 [OS=Saccharomyces cerevisiae S288c]	856071	PMA2; YPL036W	cellular homeostasis;metabolic process;transport	membrane;mitochondrion	catalytic activity;metal ion binding;nucleotide binding;transporter activity	9	8.427	0.94
P40053	Altered inheritance of mitochondria protein 9, mitochondrial [OS=Saccharomyces cerevisiae S288c]	856813	AIM9; YER080W		mitochondrion		4.289	4.036	0.94
P32191	Glycerol-3-phosphate dehydrogenase, mitochondrial [OS=Saccharomyces cerevisiae S288c]	854651	GUT2; YIL155C	metabolic process	membrane;mitochondrion	catalytic activity	9.502	9	0.95
P32843	Mitochondrial escape protein 2 [OS=Saccharomyces cerevisiae S288c]	855348	YME2; YMR302C	cell organization and biogenesis;metabolic process	membrane;mitochondrion	nucleotide binding;RNA binding	3.112	2.949	0.95
P19414	Aconitate hydratase, mitochondrial [OS=Saccharomyces cerevisiae S288c]	851013	ACO1; YLR304C	cell organization and biogenesis;metabolic process;transport	cytoplasm;cytosol;mitochondrion;organelle lumen	catalytic activity;DNA binding;metal ion binding	66.002	66.002	1.00
P0CS90	Heat shock protein SSC1, mitochondrial [OS=Saccharomyces cerevisiae S288c]	853503	SSC1; YJR045C	cell organization and biogenesis;metabolic process;regulation of biological process;transport	membrane;mitochondrion;nucleus;organelle lumen	catalytic activity;enzyme regulator activity;nucleotide binding;protein binding	51.75	51.75	1.00
P09624	Dihydrolipoyl dehydrogenase, mitochondrial [OS=Saccharomyces cerevisiae S288c]	850527	LPD1; YFL018C	cellular homeostasis;metabolic process;regulation of biological process	mitochondrion;organelle lumen	catalytic activity;nucleotide binding	30.623	30.623	1.00
Q07500	External NADH-ubiquinone oxidoreductase 2, mitochondrial [OS=Saccharomyces cerevisiae S288c]	851474	NDE2; YDL085W	metabolic process	mitochondrion	catalytic activity;nucleotide binding	12.594	12.594	1.00
P00890	citrate synthase, mitochondrial [OS=Saccharomyces cerevisiae S288c]	855732	CIT1; YNR001C	metabolic process;transport	cytosol;mitochondrion;organelle lumen	catalytic activity	11.798	11.798	1.00
P07275	Delta-1-pyrroline-5-carboxylate dehydrogenase, mitochondrial [OS=Saccharomyces cerevisiae S288c]	856432	PUT2; YHR037 W	metabolic process	membrane;mitochondrion;organelle lumen	catalytic activity	6.565	6.565	1.00
P28834	Isocitrate dehydrogenase [NAD] subunit 1, mitochondrial [OS=Saccharomyces cerevisiae S288c]	855691	IDH1; YNL037C	metabolic process;transport	cytosol;mitochondrion;organelle lumen	catalytic activity;metal ion binding;nucleotide binding;protein binding;RNA binding	15.238	15.238	1.00
Q08822	Probable electron transfer flavoprotein-ubiquinone oxidoreductase, mitochondrial [OS=Saccharomyces cerevisiae S288c]	854538	CIR2; YOR356 W	metabolic process;transport	membrane;mitochondrion	catalytic activity;metal ion binding;nucleotide binding	3.019	3.019	1.00
P32473	Pyruvate dehydrogenase E1 component subunit beta, mitochondrial [OS=Saccharomyces	852522	PDB1; YBR221C	metabolic process	mitochondrion;organelle lumen	catalytic activity	10.288	10.288	1.00

	cerevisiae S288c]								
P34227	Mitochondrial peroxiredoxin PRX1 [OS=Saccharomyces cerevisiae S288c]	852215	PRX1; YBL064C	cellular homeostasis;metabolic process;regulation of biological process;response to stimulus	mitochondrion	antioxidant activity;catalytic activity	6.848	6.848	1.00
P47127	Altered inheritance of mitochondria protein 24, mitochondrial [OS=Saccharomyces cerevisiae S288c]	853543	AIM24; YJR080C	cell organization and biogenesis	mitochondrion		3.175	3.175	1.00
P39925	Mitochondrial respiratory chain complexes assembly protein AFG3 [OS=Saccharomyces cerevisiae S288c]	856737	AFG3; YER017C	cell organization and biogenesis;metabolic process;response to stimulus;transport	membrane;mitochondrion	catalytic activity;metal ion binding;nucleotide binding;protein binding	1.228	1.228	1.00
P05626	ATP synthase subunit 4, mitochondrial [OS=Saccharomyces cerevisiae S288c]	856027	ATP4; YPL078C	cell organization and biogenesis;metabolic process;transport	membrane;mitochondrion	catalytic activity;transporter activity	3.642	3.642	1.00
P42940	Probable electron transfer flavoprotein subunit beta [OS=Saccharomyces cerevisiae S288c]	853121	CIR1; YGR207C	metabolic process;transport	mitochondrion;organelle lumen		7.66	7.66	1.00
P32332	Mitochondrial oxaloacetate transport protein [OS=Saccharomyces cerevisiae S288c]	853739	OAC1; YKL120W	metabolic process;transport	membrane;mitochondrion	structural molecule activity;transporter activity	5.813	5.813	1.00
P52893	probable alanine aminotransferase, mitochondrial [OS=Saccharomyces cerevisiae S288c]	850778	ALT1; YLR089C	metabolic process	mitochondrion;organelle lumen	catalytic activity	1.438	1.438	1.00
P25039	Elongation factor G, mitochondrial [OS=Saccharomyces cerevisiae S288c]	850758	MEF1; YLR069C	cell organization and biogenesis;metabolic process	cytosol;mitochondrion	catalytic activity;nucleotide binding;RNA binding	1.024	1.024	1.00
P32787	Mitochondrial genome maintenance protein MGM101 [OS=Saccharomyces cerevisiae S288c]	853609	MGM101 ; YJR144W	cell organization and biogenesis;metabolic process;response to stimulus	chromosome;mitochondrion	DNA binding	4.456	4.456	1.00
P25613	Accumulation of dyads protein 2 [OS=Saccharomyces cerevisiae S288c]	850368	ADY2; YCR010C	transport	membrane;mitochondrion;vacuole	transporter activity	12.895	12.895	1.00
P30902	ATP synthase subunit d, mitochondrial [OS=Saccharomyces cerevisiae S288c]	853853	ATP7; YKL016C	metabolic process;transport	membrane;mitochondrion	catalytic activity;transporter activity	5.105	5.105	1.00
P40035	Mitochondrial phosphate carrier protein 2 [OS=Saccharomyces cerevisiae S288c]	856779	PIC2; YER053C	cellular homeostasis;metabolic process;transport	membrane;mitochondrion	structural molecule activity;transporter activity	2.793	2.793	1.00
Q12207	Non-classical export protein 2 [OS=Saccharomyces cerevisiae S288c]	856272	NCE102; YPR149W	cell organization and biogenesis;regulation of biological process;transport	cytoplasm;endoplasmic reticulum;membrane;mitochondrion		12.895	12.895	1.00
P38088	Glycine--tRNA ligase 1, mitochondrial [OS=Saccharomyces cerevisiae S288c]			metabolic process	cytoplasm;mitochondrion;organelle lumen	catalytic activity;nucleotide binding;protein binding	0.468	0.468	1.00
P32897	Mitochondrial import inner membrane translocase subunit tim23 [OS=Saccharomyces cerevisiae S288c]	855751	TIM23; YNR017 W	transport	membrane;mitochondrion	protein binding;transporter activity	5.31	5.31	1.00
P40513	Mitochondrial acidic protein MAM33 [OS=Saccharomyces cerevisiae S288c]	854740	MAM33; YIL070C	metabolic process;regulation of biological process	mitochondrion;organelle lumen	translation regulator activity	3.642	3.642	1.00
P00410	Cytochrome c oxidase subunit 2 [OS=Saccharomyces cerevisiae S288c]	854622	COX2; Q0250	metabolic process;transport	membrane;mitochondrion	catalytic activity;metal ion binding;protein binding;transporter activity	3.642	3.642	1.00
Q02950	37S ribosomal protein MRP51, mitochondrial [OS=Saccharomyces cerevisiae S288c]	855985	MRP51; YPL118W	cell organization and biogenesis;metabolic process	mitochondrion;ribosome	structural molecule activity	1.154	1.154	1.00
Q07349	MIOREX complex component 9 [OS=Saccharomyces cerevisiae S288c]	851535	YDL027C; MRX9; YDL027C		endoplasmic reticulum;membrane;mitochondrion		1.913	1.913	1.00
Q06236	Mitochondrial inner membrane protein SHH4 [OS=Saccharomyces cerevisiae S288c]	850861	SHH4; YLR164W	metabolic process	membrane;mitochondrion	catalytic activity;metal ion binding	6.499	6.499	1.00
P32331	Carrier protein YMC1, mitochondrial [OS=Saccharomyces cerevisiae S288c]	856171	YMC1; YPR058W	metabolic process;transport	membrane;mitochondrion;vacuole	structural molecule activity;transporter activity	2.384	2.384	1.00

Q12029	Probable mitochondrial transport protein fsf1 [OS=Saccharomyces cerevisiae S288c]	854445	FSF1; YOR271C	transport	membrane;mitochondrion	transporter activity	2.36	2.36	1.00
P39952	Mitochondrial inner membrane protein OXA1 [OS=Saccharomyces cerevisiae S288c]	856898	OXA1; YER154W	cell organization and biogenesis;transport	membrane;mitochondrion	transporter activity	1.738	1.738	1.00
P53292	37S ribosomal protein S35, mitochondrial [OS=Saccharomyces cerevisiae S288c]	853075	MRPS35; YGR165 W	cell organization and biogenesis;metabolic process	mitochondrion;ribosome	structural molecule activity	1.512	1.512	1.00
P11325	Leucine--tRNA ligase, mitochondrial [OS=Saccharomyces cerevisiae S288c]	851098	NAM2; YLR382C	cell organization and biogenesis;metabolic process;regulation of biological process	cytosol;mitochondrion;organelle lumen	catalytic activity;nucleotide binding;RNA binding	0.353	0.353	1.00
P53889	uncharacterized mitochondrial hydrolase FMP41 [OS=Saccharomyces cerevisiae S288c]	855553	FMP41; YNL168C	metabolic process	mitochondrion	catalytic activity;metal ion binding	1.254	1.254	1.00
Q12165	ATP synthase subunit delta, mitochondrial [OS=Saccharomyces cerevisiae S288c]	851560	ATP16; YDL004W	metabolic process;transport	membrane;mitochondrion	catalytic activity;transporter activity	4.623	4.623	1.00
P36141	Putative redox protein fmp46, mitochondrial [OS=Saccharomyces cerevisiae S288c]	853923	FMP46; YKR049C	metabolic process	mitochondrion	catalytic activity;protein binding	4.337	4.337	1.00
P23644	Mitochondrial import receptor subunit TOM40 [OS=Saccharomyces cerevisiae S288c]	855243	TOM40; YMR203 W	transport	cytosol;membrane;mitochondrion	protein binding;transporter activity	1.154	1.154	1.00
Q12117	Protein MRH1 [OS=Saccharomyces cerevisiae S288c]	851597	MRH1; YDR033 W	transport	endoplasmic reticulum;membrane;mitochondrion	transporter activity	2.831	2.831	1.00
Q06493	LETM1 domain-containing protein YLH47, mitochondrial [OS=Saccharomyces cerevisiae S288c]	856243	YLH47; YPR125W	cell organization and biogenesis;transport	membrane;mitochondrion		1.043	1.043	1.00
P43594	MICOS complex subunit MIC19 [OS=Saccharomyces cerevisiae S288c]	850563	AIM13; MIC19; YFR011C	cell organization and biogenesis	cytoplasm;membrane;mitochondrion		3.329	3.329	1.00
P36534	54S ribosomal protein L40, mitochondrial [OS=Saccharomyces cerevisiae S288c]	855930	MRPL40; YPL173W	cell organization and biogenesis;metabolic process	mitochondrion;ribosome	protein binding;structural molecule activity	1.512	1.512	1.00
Q02204	54S ribosomal protein L13, mitochondrial [OS=Saccharomyces cerevisiae S288c]	853875	MRPL13; YKR006C	cell organization and biogenesis;metabolic process	mitochondrion;ribosome	structural molecule activity	1.371	1.371	1.00
P40508	Uncharacterized protein YIL077C [OS=Saccharomyces cerevisiae S288c]	854733	YIL077C; YIL077C		mitochondrion		1.448	1.448	1.00
Q05892	MIOREX complex component 2 [OS=Saccharomyces cerevisiae S288c]	850997	YLR290C; COQ11; YLR290C	metabolic process	mitochondrion	catalytic activity	3.217	3.217	1.00
P37298	Succinate dehydrogenase [ubiquinone] cytochrome b small subunit, mitochondrial [OS=Saccharomyces cerevisiae S288c]	851758	SDH4; YDR178 W	metabolic process;transport	membrane;mitochondrion	catalytic activity;metal ion binding	4.995	4.995	1.00
Q12374	Nuclear control of ATPase protein 2 [OS=Saccharomyces cerevisiae S288c]	856278	NCA2; YPR155C	metabolic process	membrane;mitochondrion		0.565	0.565	1.00
P47045	mitochondrial import inner membrane translocase subunit TIM54 [OS=Saccharomyces cerevisiae S288c]	853392	TIM54; YJL054W	cell organization and biogenesis;transport	cytosol;membrane;mitochondrion	transporter activity	0.655	0.655	1.00
P39965	probable proline--tRNA ligase, mitochondrial [OS=Saccharomyces cerevisiae S288c]	856820	AIM10; YER087W	metabolic process	cytoplasm;mitochondrion	catalytic activity;nucleotide binding;RNA binding	0.343	0.343	1.00
P40582	Glutathione S-transferase 1 [OS=Saccharomyces cerevisiae S288c]	854856	GTT1; YIR038C	metabolic process	endoplasmic reticulum;membrane;mitochondrion;nucleus	antioxidant activity;catalytic activity;protein binding;RNA binding	1.683	1.683	1.00
P38910	10 kDa heat shock protein, mitochondrial [OS=Saccharomyces cerevisiae S288c]	854185	HSP10; YOR020C	cell organization and biogenesis;metabolic process;response to stimulus;transport	cytoplasm;mitochondrion;organelle lumen	metal ion binding;nucleotide binding;protein binding	4.623	4.623	1.00
Q12482	Mitochondrial aspartate-glutamate transporter AGC1 [OS=Saccharomyces cerevisiae S288c]	856132	AGC1; YPR021C	metabolic process;transport	membrane;mitochondrion	structural molecule activity;transporter activity	0.407	0.407	1.00
Q06143	mitochondrial dicarboxylate transporter [OS=Saccharomyces cerevisiae S288c]	851063	DIC1; YLR348C	metabolic process;transport	membrane;mitochondrion	structural molecule activity;transporter activity	1.154	1.154	1.00

Q12349	ATP synthase subunit H, mitochondrial [OS=Saccharomyces cerevisiae S288c]	851002	ATP14; YLR295C	cell organization and biogenesis;metabolic process;transport	membrane;mitochondrion	catalytic activity;transporter activity	2.728	2.728	1.00
P35996	54S ribosomal protein L38, mitochondrial [OS=Saccharomyces cerevisiae S288c]	853684	MRPL38; YKL170W	cell organization and biogenesis;metabolic process	mitochondrion;ribosome	RNA binding;structural molecule activity	2.594	2.594	1.00
Q3E824	Uncharacterized protein YOR020W-A [OS=Saccharomyces cerevisiae S288c]	1466480	YOR020W-A; YOR020W-A	metabolic process;transport	membrane;mitochondrion		5.813	5.813	1.00
P32904	54S ribosomal protein L6, mitochondrial [OS=Saccharomyces cerevisiae S288c]	856552	MRPL6; YHR147C	cell organization and biogenesis;metabolic process	mitochondrion;ribosome	RNA binding;structural molecule activity	1.371	1.371	1.00
P40416	Iron-sulfur clusters transporter ATM1, mitochondrial [OS=Saccharomyces cerevisiae S288c]	855347	ATM1; YMR301C	cellular homeostasis;metabolic process;transport	membrane;mitochondrion	catalytic activity;nucleotide binding;transporter activity	0.453	0.453	1.00
P32453	Protein ATP11, mitochondrial [OS=Saccharomyces cerevisiae S288c]	855401	ATP11; YNL315C	cell organization and biogenesis	mitochondrion	protein binding	1.512	1.512	1.00
P25626	54S ribosomal protein IMG1, mitochondrial [OS=Saccharomyces cerevisiae S288c]	850413	IMG1; YCR046C	cell organization and biogenesis;metabolic process	mitochondrion;ribosome	structural molecule activity	1.424	1.424	1.00
P50945	MICOS complex subunit MIC27 [OS=Saccharomyces cerevisiae S288c]	855623	AIM37; MIC27; YNL100W	cell organization and biogenesis	membrane;mitochondrion		1.254	1.254	1.00
P10834	protein PET54 [OS=Saccharomyces cerevisiae S288c]	853137	PET54; YGR222W	metabolic process;regulation of biological process	membrane;mitochondrion;organelle lumen	nucleotide binding;RNA binding;translation regulator activity	0.874	0.874	1.00
Q08970	Mitochondrial metal transporter 2 [OS=Saccharomyces cerevisiae S288c]	855877	MMT2; YPL224C	cellular homeostasis;regulation of biological process;response to stimulus;transport	membrane;mitochondrion	transporter activity	0.532	0.532	1.00
Q03201	37S ribosomal protein S10, mitochondrial [OS=Saccharomyces cerevisiae S288c]	851611	RSM10; YDR041W	cell organization and biogenesis;metabolic process	mitochondrion;ribosome	structural molecule activity	1.424	1.424	1.00
P36521	54S ribosomal protein L11, mitochondrial [OS=Saccharomyces cerevisiae S288c]	851325	MRPL11; YDL202W	cell organization and biogenesis;metabolic process	mitochondrion;ribosome	structural molecule activity	1.069	1.069	1.00
P25372	Thioredoxin-3, mitochondrial [OS=Saccharomyces cerevisiae S288c]	850444	TRX3; YCR083W	cellular homeostasis;metabolic process;regulation of biological process;response to stimulus;transport	cytoplasm;mitochondrion	catalytic activity	2.511	2.511	1.00
P33759	37S ribosomal protein S5, mitochondrial [OS=Saccharomyces cerevisiae S288c]	852553	MRPS5; YBR251W	cell organization and biogenesis;metabolic process	mitochondrion;ribosome	RNA binding;structural molecule activity	0.995	0.995	1.00
P32387	54S ribosomal protein L41, mitochondrial [OS=Saccharomyces cerevisiae S288c]	852014	MRP20; YDR405W	cell organization and biogenesis;metabolic process	mitochondrion;ribosome	nucleotide binding;protein binding;RNA binding;structural molecule activity	1.254	1.254	1.00
Q04172	Sensitive to high expression protein 9, mitochondrial [OS=Saccharomyces cerevisiae S288c]	852002	SHE9; YDR393W	cell organization and biogenesis	membrane;mitochondrion		0.374	0.374	1.00
P27697	Atypical kinase COQ8, mitochondrial [OS=Saccharomyces cerevisiae S288c]	852758	COQ8; YGL119W	metabolic process	membrane;mitochondrion;organelle lumen	catalytic activity;nucleotide binding	0.453	0.453	1.00
Q08245	protein ZEO1 [OS=Saccharomyces cerevisiae S288c]	854040	ZEO1; YOL109W	cell organization and biogenesis	membrane;mitochondrion	protein binding	2.162	2.162	1.00
P36519	54S ribosomal protein L7, mitochondrial [OS=Saccharomyces cerevisiae S288c]	851823	MRPL7; YDR237W	cell organization and biogenesis;metabolic process	mitochondrion;ribosome	RNA binding;structural molecule activity	0.468	0.468	1.00
P53311	Mitochondrial pyruvate carrier 3 [OS=Saccharomyces cerevisiae S288c]	853158	FMP43; MPC3; YGR243W	transport	membrane;mitochondrion	transporter activity	1.154	1.154	1.00
Q12305	Thiosulfate sulfurtransferase RDL1, mitochondrial [OS=Saccharomyces cerevisiae S288c]	854459	RDL1; YOR285W		endoplasmic reticulum;membrane;mitochondrion	catalytic activity	1.154	1.154	1.00
Q12204	Probable phospholipase YOR022C, mitochondrial [OS=Saccharomyces cerevisiae S288c]	854187	YOR022C; YOR022C	metabolic process	mitochondrion	catalytic activity;metal ion binding	0.233	0.233	1.00

Q12251	Uncharacterized mitochondrial carrier YPR011C [OS=Saccharomyces cerevisiae S288c]	856121	YPR011C; YPR011C	metabolic process;transport	membrane;mitochondrion	structural molecule activity;transporter activity	0.551	0.551	1.00
P41903	Peroxisomal acyl-coenzyme A thioester hydrolase 1 [OS=Saccharomyces cerevisiae S288c]	853477	TES1; YJR019C	metabolic process	mitochondrion	catalytic activity	0.931	0.931	1.00
P47140	Altered inheritance rate of mitochondria protein 25 [OS=Saccharomyces cerevisiae S288c]	853563	AIM25; YJR100C	cell organization and biogenesis	membrane;mitochondrion	transporter activity	0.369	0.369	1.00
P32839	Mitochondrial chaperone BCS1 [OS=Saccharomyces cerevisiae S288c]	851981	BCS1; YDR375C	cell organization and biogenesis;metabolic process;response to stimulus;transport	cytosol;membrane;mitochondrion	catalytic activity;nucleotide binding;transporter activity	0.425	0.425	1.00
Q01519	Cytochrome c oxidase subunit 6b [OS=Saccharomyces cerevisiae S288c]	850727	COX12; YLR038C	cell organization and biogenesis;metabolic process;transport	mitochondrion	catalytic activity;transporter activity	3.642	3.642	1.00
Q02888	Inner membrane assembly complex subunit 17 [OS=Saccharomyces cerevisiae S288c]	856005	AIM43; INA17; YPL099C	cell organization and biogenesis	membrane;mitochondrion	protein binding	0.701	0.701	1.00
Q06567	ABC1 family protein MCP2 [OS=Saccharomyces cerevisiae S288c]	850955	YLR253W ; MCP2; YLR253W	cell organization and biogenesis	membrane;mitochondrion		0.218	0.218	1.00
P49017	2-methoxy-6-polyprenyl-1,4-benzoquinol methylase, mitochondrial [OS=Saccharomyces cerevisiae S288c]	854930	COQ5; YML110C	metabolic process	membrane;mitochondrion;organelle lumen	catalytic activity	0.551	0.551	1.00
Q12359	Ammonia transport outward protein 3 [OS=Saccharomyces cerevisiae S288c]	851992	ATO3; YDR384C	transport	membrane;mitochondrion	transporter activity	0.995	0.995	1.00
P37299	cytochrome b-c1 complex subunit 10 [OS=Saccharomyces cerevisiae S288c]	856390	QCR10; YHR001 W-A	metabolic process;transport	membrane;mitochondrion	catalytic activity;transporter activity	5.31	5.31	1.00
P38079	protein YRO2 [OS=Saccharomyces cerevisiae S288c]	852343	YRO2; YBR054W	transport	endoplasmic reticulum;membrane;mitochondrion	transporter activity	1.424	1.424	1.00
P53305	Mitochondrial 37S ribosomal protein S27 [OS=Saccharomyces cerevisiae S288c]	853129	RSM27; YGR215 W	cell organization and biogenesis;metabolic process	mitochondrion;ribosome	structural molecule activity	2.162	2.162	1.00
P46998	mitochondrial membrane protein FMP33 [OS=Saccharomyces cerevisiae S288c]	853279	FMP33; YJL161W		membrane;mitochondrion		2.162	2.162	1.00
Q06485	Autophagy-related protein 33 [OS=Saccharomyces cerevisiae S288c]	851070	ATG33; YLR356W	cell communication;metabolic process;response to stimulus	membrane;mitochondrion		2.162	2.162	1.00
Q12233	ATP synthase subunit g, mitochondrial [OS=Saccharomyces cerevisiae S288c]	856131	ATP20; YPR020W	cell organization and biogenesis;metabolic process;transport	membrane;mitochondrion	catalytic activity;transporter activity	1.371	1.371	1.00
P32611	54S ribosomal protein RML2, mitochondrial [OS=Saccharomyces cerevisiae S288c]	856660	RML2; YEL050C	cell organization and biogenesis;metabolic process	mitochondrion;ribosome	catalytic activity;RNA binding;structural molecule activity	0.389	0.389	1.00
P38300	Inner membrane mitoribosome receptor MBA1, mitochondrial [OS=Saccharomyces cerevisiae S288c]	852483	MBA1; YBR185C	cell organization and biogenesis;regulation of biological process	membrane;mitochondrion		0.995	0.995	1.00
Q03020	iron sulfur cluster assembly protein 1, mitochondrial [OS=Saccharomyces cerevisiae S288c]	855968	ISU1; YPL135W	cell organization and biogenesis;cellular homeostasis;metabolic process	cytoplasm;mitochondrion;organelle lumen	catalytic activity;metal ion binding;protein binding	0.778	0.778	1.00
P14540	fructose-bisphosphate aldolase [OS=Saccharomyces cerevisiae S288c]	853805	FBA1; YKL060C	metabolic process	cytoplasm;cytosol;membrane;mitochondrion	catalytic activity;metal ion binding	0.292	0.292	1.00
Q02608	37S ribosomal protein S16, mitochondrial [OS=Saccharomyces cerevisiae S288c]	856094	MRPS16; YPL013C	cell organization and biogenesis;metabolic process	mitochondrion;ribosome	structural molecule activity	1.683	1.683	1.00
P40159	Uncharacterized protein YNL208W [OS=Saccharomyces cerevisiae S288c]	855513	YNL208W ; YNL208W		membrane;mitochondrion;ribosome		0.668	0.668	1.00
P53732	37S ribosomal protein S12, mitochondrial [OS=Saccharomyces cerevisiae S288c]	855772	MRPS12; YNR036C	cell organization and biogenesis;metabolic process	mitochondrion;ribosome	structural molecule activity	1.154	1.154	1.00
P23369	54S ribosomal protein L25, mitochondrial [OS=Saccharomyces cerevisiae S288c]	852967	MRPL25; YGR076C	cell organization and biogenesis;metabolic process	mitochondrion;ribosome	structural molecule activity	1.31	1.31	1.00

Q6Q560	Protein isd11 [OS=Saccharomyces cerevisiae S288c]	856774	ISD11; YER048W-A	cell organization and biogenesis;metabolic process	mitochondrion;organelle lumen	catalytic activity;protein binding	1.783	1.783	1.00
P41911	Glycerol-3-phosphate dehydrogenase [NAD(+)] 2, mitochondrial [OS=Saccharomyces cerevisiae S288c]	854095	GPD2; YOL059W	metabolic process	cytoplasm;cytosol;mitochondrion	catalytic activity;nucleotide binding;protein binding	0.186	0.186	1.00
P53220	Mitochondrial import inner membrane translocase subunit TIM21 [OS=Saccharomyces cerevisiae S288c]	852921	TIM21; YGR033C	cell organization and biogenesis;transport	membrane;mitochondrion	protein binding	0.719	0.719	1.00
P33421	Succinate dehydrogenase [ubiquinone] cytochrome b subunit, mitochondrial [OS=Saccharomyces cerevisiae S288c]	853716	SDH3; YKL141W	cell organization and biogenesis;metabolic process;transport	membrane;mitochondrion	catalytic activity;metal ion binding	1.276	1.276	1.00
P53724	54S ribosomal protein L50, mitochondrial [OS=Saccharomyces cerevisiae S288c]	855756	MRPL50; YNR022C	cell organization and biogenesis;metabolic process	mitochondrion;ribosome	structural molecule activity	1.512	1.512	1.00
P38702	mitochondrial carrier protein LEU5 [OS=Saccharomyces cerevisiae S288c]	856391	LEU5; YHR002W	metabolic process;transport	membrane;mitochondrion	structural molecule activity;transporter activity	0.334	0.334	1.00
P36147	Presequence translocated-associated motor subunit pam17, mitochondrial [OS=Saccharomyces cerevisiae S288c]	853939	PAM17; YKR065C	transport	membrane;mitochondrion		1.371	1.371	1.00
Q02883	N-acyl-phosphatidylethanolamine-hydrolyzing phospholipase D, mitochondrial [OS=Saccharomyces cerevisiae S288c]	856001	FMP30; YPL103C	metabolic process	membrane;mitochondrion	catalytic activity;metal ion binding	0.425	0.425	1.00
P36526	54S ribosomal protein L27, mitochondrial [OS=Saccharomyces cerevisiae S288c]	852585	MRPL27; YBR282W	cell organization and biogenesis;metabolic process	mitochondrion;ribosome	structural molecule activity	0.995	0.995	1.00
P53163	54S ribosomal protein L12, mitochondrial [OS=Saccharomyces cerevisiae S288c]	852811	MNP1; YGL068W	cell organization and biogenesis;metabolic process	mitochondrion;ribosome	RNA binding;structural molecule activity	1.31	1.31	1.00
P53166	ATP-dependent RNA helicase mrh4, mitochondrial [OS=Saccharomyces cerevisiae S288c]	852816	MRH4; YGL064C	cell organization and biogenesis;metabolic process	mitochondrion;organelle lumen	catalytic activity;nucleotide binding;RNA binding	0.145	0.145	1.00
Q07914	mitochondrial import inner membrane translocase subunit TIM14 [OS=Saccharomyces cerevisiae S288c]	850694	PAM18; YLR008C	regulation of biological process;transport	membrane;mitochondrion	enzyme regulator activity;protein binding;transporter activity	0.778	0.778	1.00
P36531	54S ribosomal protein L36, mitochondrial [OS=Saccharomyces cerevisiae S288c]	852419	MRPL36; YBR122C	cell organization and biogenesis;metabolic process	mitochondrion;ribosome	structural molecule activity	0.668	0.668	1.00
P21306	ATP synthase subunit epsilon, mitochondrial [OS=Saccharomyces cerevisiae S288c]	855857	ATP15; YPL271W	metabolic process;transport	membrane;mitochondrion	catalytic activity;transporter activity	2.162	2.162	1.00
Q01976	ADP-ribose pyrophosphatase [OS=Saccharomyces cerevisiae S288c]	852408	YSA1; YBR111C	metabolic process	cytoplasm;mitochondrion;nucleus	catalytic activity;metal ion binding	0.425	0.425	1.00
P38169	Kynurenine 3-monooxygenase [OS=Saccharomyces cerevisiae S288c]	852179	BNA4; YBL098W	metabolic process	membrane;mitochondrion	catalytic activity;nucleotide binding	0.269	0.269	1.00
P32785	Methionyl-tRNA formyltransferase, mitochondrial [OS=Saccharomyces cerevisiae S288c]	852270	FMT1; YBL013W	metabolic process	mitochondrion	catalytic activity	0.179	0.179	1.00
P81451	ATP synthase subunit K, mitochondrial [OS=Saccharomyces cerevisiae S288c]	854077	ATP19; YOL077W-A	cell organization and biogenesis;metabolic process;transport	membrane;mitochondrion	catalytic activity;transporter activity	3.642	3.642	1.00
P81450	ATP synthase subunit J, mitochondrial [OS=Saccharomyces cerevisiae S288c]	854893	ATP18; YML081C-A	cell organization and biogenesis;metabolic process;transport	membrane;mitochondrion	catalytic activity;transporter activity	9	9	1.00
P39677	Ribosome-releasing factor 2, mitochondrial [OS=Saccharomyces cerevisiae S288c]	853342	MEF2; YJL102W	cell organization and biogenesis;metabolic process	cytosol;mitochondrion	catalytic activity;nucleotide binding;RNA binding	0.179	0.179	1.00
P25345	Asparagine--tRNA ligase, mitochondrial [OS=Saccharomyces cerevisiae S288c]	850388	SLM5; YCR024C	metabolic process	cytoplasm;mitochondrion;organelle lumen	catalytic activity;nucleotide binding	0.172	0.172	1.00
Q07534	Solute carrier family 25 member 38 homolog	851439	YDL119C; HEM25;	metabolic process;transport	membrane;mitochondrion	structural molecule activity;transporter	0.389	0.389	1.00

	[OS=Saccharomyces cerevisiae S288c]		YDL119C			activity			
P20084	54S ribosomal protein L33, mitochondrial [OS=Saccharomyces cerevisiae S288c]	855330	MRPL33; YMR286W	cell organization and biogenesis;metabolic process	mitochondrion;ribosome	structural molecule activity	2.162	2.162	1.00
P47141	37S ribosomal protein S26, mitochondrial [OS=Saccharomyces cerevisiae S288c]	853565	RSM26; YJR101W	cell organization and biogenesis;metabolic process;response to stimulus	mitochondrion;ribosome	antioxidant activity;catalytic activity;metal ion binding;structural molecule activity	0.425	0.425	1.00
P40858	54S ribosomal protein L49, mitochondrial [OS=Saccharomyces cerevisiae S288c]	853349	MRPL49; YJL096W	cell organization and biogenesis;metabolic process	mitochondrion;ribosome	RNA binding;structural molecule activity	0.468	0.468	1.00
P36523	54S ribosomal protein L15, mitochondrial [OS=Saccharomyces cerevisiae S288c]	851022	MRPL15; YLR312W-A	cell organization and biogenesis;metabolic process	mitochondrion;ribosome	catalytic activity;RNA binding;structural molecule activity	0.334	0.334	1.00
P32792	UPF0744 protein YSC83 [OS=Saccharomyces cerevisiae S288c]	856410	YSC83; YHR017W		membrane;mitochondrion		0.233	0.233	1.00
P34224	Uncharacterized protein YBL059W [OS=Saccharomyces cerevisiae S288c]	852221	YBL059W; YBL059W		membrane;mitochondrion		0.468	0.468	1.00
Q08230	Succinate dehydrogenase assembly factor 2, mitochondrial [OS=Saccharomyces cerevisiae S288c]	854083	EMI5; SDH5; YOL071W	cell differentiation;cell organization and biogenesis;metabolic process;regulation of biological process	mitochondrion;organelle lumen	protein binding	0.468	0.468	1.00
P50087	MICOS subunit MIC26 [OS=Saccharomyces cerevisiae S288c]	853150	MOS2; MIC26; YGR235C	cell organization and biogenesis	membrane;mitochondrion		0.638	0.638	1.00
P03879	Intron-encoded RNA maturase bI4 [OS=Saccharomyces cerevisiae S288c]	854582	BI4; Q0120	metabolic process	membrane;mitochondrion	catalytic activity;RNA binding	0.28	0.28	1.00
Q03429	Mitochondrial zinc maintenance protein 1, mitochondrial [OS=Saccharomyces cerevisiae S288c]	852104	MZM1; YDR493W	cell organization and biogenesis;metabolic process	mitochondrion;organelle lumen	protein binding	0.334	0.334	1.00
P53875	54S ribosomal protein L19, mitochondrial [OS=Saccharomyces cerevisiae S288c]	855536	MRPL19; YNL185C	cell organization and biogenesis;metabolic process	mitochondrion;ribosome	RNA binding;structural molecule activity	0.874	0.874	1.00
Q01802	Aspartate aminotransferase, mitochondrial [OS=Saccharomyces cerevisiae S288c]	853755	AAT1; YKL106W	metabolic process	mitochondrion;organelle lumen	catalytic activity;protein binding	0.155	0.155	1.00
P01098	ATPase-stabilizing factor 9 kDa, mitochondrial [OS=Saccharomyces cerevisiae S288c]	851426	STF1; YDL130W-A	metabolic process;regulation of biological process	mitochondrion	catalytic activity;enzyme regulator activity;protein binding	2.162	2.162	1.00
Q05779	Ubiquinone biosynthesis protein COQ9, mitochondrial [OS=Saccharomyces cerevisiae S288c]	850898	COQ9; YLR201C	metabolic process	membrane;mitochondrion		0.359	0.359	1.00
P53212	Probable transcriptional regulatory protein HAH1 [OS=Saccharomyces cerevisiae S288c]	852904	YGR021W; YGR021W		mitochondrion		0.359	0.359	1.00
P00942	Triosephosphate isomerase [OS=Saccharomyces cerevisiae S288c]	851620	TPI1; YDR050C	metabolic process	cytoplasm;cytosol;membrane;mitochondrion	catalytic activity	0.194	0.194	1.00
P32378	4-hydroxybenzoate polyprenyltransferase, mitochondrial [OS=Saccharomyces cerevisiae S288c]	855778	COQ2; YNR041C	metabolic process;transport	membrane;mitochondrion	catalytic activity	0.292	0.292	1.00
P27680	Ubiquinone biosynthesis O-methyltransferase, mitochondrial [OS=Saccharomyces cerevisiae S288c]	854057	COQ3; YOL096C	metabolic process	membrane;mitochondrion	catalytic activity	0.274	0.274	1.00
Q2V2P9	Uncharacterized protein YDR119W-A [OS=Saccharomyces cerevisiae S288c]	3799970	COX26; YDR119W-A		membrane;mitochondrion		1.154	1.154	1.00
Q06090	54S ribosomal protein L51, mitochondrial [OS=Saccharomyces cerevisiae S288c]	856214	MRPL51; YPR100W	cell organization and biogenesis;metabolic process	mitochondrion;ribosome	RNA binding;structural molecule activity	0.292	0.292	1.00
Q08686	Thiosulfate sulfurtransferase TUM1 [OS=Saccharomyces cerevisiae S288c]	854425	TUM1; YOR251C	metabolic process	cytoplasm;mitochondrion	catalytic activity;protein binding	0.212	0.212	1.00
P47131	TMEM14 protein homolog YJR085C [OS=Saccharomyces cerevisiae S288c]	853547	YJR085C; YJR085C		membrane;mitochondrion		0.778	0.778	1.00

P53320	Mitochondrial carrier protein MTM1 [OS=Saccharomyces cerevisiae S288c]	853173	MTM1; YGR257C	metabolic process;transport	membrane;mitochondrion	structural molecule activity	0.11	0.11	1.00
Q3E6R5	uncharacterized mitochondrial outer membrane protein YDR381C-A [OS=Saccharomyces cerevisiae S288c]	851989	YDR381C-A; YDR381C-A		membrane;mitochondrion		0.389	0.389	1.00
P32463	Acyl carrier protein, mitochondrial [OS=Saccharomyces cerevisiae S288c]	853642	ACP1; YKL192C	metabolic process;transport	mitochondrion		0.259	0.259	1.00
P40581	peroxiredoxin HYR1 [OS=Saccharomyces cerevisiae S288c]	854855	HYR1; YIR037W	metabolic process;response to stimulus	cytoplasm;cytosol;mitochondrion;organelle lumen	antioxidant activity;catalytic activity	0.259	0.259	1.00
Q08058	Coenzyme Q-binding protein COQ10, mitochondrial [OS=Saccharomyces cerevisiae S288c]	854154	COQ10; YOL008W	metabolic process	membrane;mitochondrion		0.194	0.194	1.00
Q07821	Iron-sulfur assembly protein 1 [OS=Saccharomyces cerevisiae S288c]	850632	ISA1; YLL027W	cell organization and biogenesis;metabolic process	mitochondrion;organelle lumen	metal ion binding;structural molecule activity	0.136	0.136	1.00
P40033	37S ribosomal protein rsm18, mitochondrial [OS=Saccharomyces cerevisiae S288c]	856776	RSM18; YER050C	cell organization and biogenesis;metabolic process	mitochondrion;ribosome	structural molecule activity	0.585	0.585	1.00
Q08645	folylpolyglutamate synthase [OS=Saccharomyces cerevisiae S288c]	854415	MET7; YOR241W	metabolic process	cytoplasm;endoplasmic reticulum;membrane;mitochondrion;organelle lumen	catalytic activity;nucleotide binding	0.15	0.15	1.00
Q12328	Mitochondrial import inner membrane translocase subunit TIM22 [OS=Saccharomyces cerevisiae S288c]	851309	TIM22; YDL217C	cell organization and biogenesis;transport	cytosol;membrane;mitochondrion	protein binding;transporter activity	0.233	0.233	1.00
P40990	Protein MSS2, mitochondrial [OS=Saccharomyces cerevisiae S288c]	851451	MSS2; YDL107W	cell organization and biogenesis	membrane;mitochondrion;organelle lumen	protein binding	0.101	0.101	1.00
P04039	Cytochrome c oxidase polypeptide VIII, mitochondrial [OS=Saccharomyces cerevisiae S288c]	851111	COX8; YLR395C	metabolic process;transport	membrane;mitochondrion	catalytic activity;transporter activity	0.778	0.778	1.00
P38812	Phosphatidylglycerophosphatase GEP4, mitochondrial [OS=Saccharomyces cerevisiae S288c]	856500	GEP4; YHR100C	metabolic process	membrane;mitochondrion;organelle lumen	catalytic activity	0.179	0.179	1.00
P10174	cytochrome c oxidase subunit 7 [OS=Saccharomyces cerevisiae S288c]	855298	COX7; YMR256C	metabolic process;transport	membrane;mitochondrion	catalytic activity;transporter activity	1.154	1.154	1.00
P39515	mitochondrial import inner membrane translocase subunit Tim17 [OS=Saccharomyces cerevisiae S288c]	853298	TIM17; YJL143W	cell organization and biogenesis;transport	membrane;mitochondrion	protein binding;transporter activity	0.778	0.778	1.00
P53733	37S ribosomal protein S19, mitochondrial [OS=Saccharomyces cerevisiae S288c]	855773	RSM19; YNR037C	cell organization and biogenesis;metabolic process	mitochondrion;ribosome	RNA binding;structural molecule activity	0.931	0.931	1.00
P07263-1	Histidine--tRNA ligase, mitochondrial [OS=Saccharomyces cerevisiae S288c]	856145	HTS1; YPR033C	cell organization and biogenesis;metabolic process	cytoplasm;mitochondrion	catalytic activity;nucleotide binding;RNA binding	0.064	0.064	1.00
P47051	putative lipoate-protein ligase A [OS=Saccharomyces cerevisiae S288c]	853401	AIM22; YJL046W	metabolic process	mitochondrion	catalytic activity;nucleotide binding	0.089	0.089	1.00
Q12487	54S ribosomal protein L23, mitochondrial [OS=Saccharomyces cerevisiae S288c]	854321	MRPL23; YOR150W	cell organization and biogenesis;metabolic process	mitochondrion;ribosome;vacuole	RNA binding;structural molecule activity	0.233	0.233	1.00
P25270	rRNA methyltransferase 1, mitochondrial [OS=Saccharomyces cerevisiae S288c]	854376	MRM1; YOR201C	metabolic process	mitochondrion	catalytic activity;RNA binding	0.129	0.129	1.00
P39940	E3 ubiquitin-protein ligase RSP5 [OS=Saccharomyces cerevisiae S288c]	856862	RSP5; YER125W	cell organization and biogenesis;metabolic process;regulation of biological process;response to stimulus;transport	cytoplasm;cytoskeleton;Golgi;membrane;mitochondrion;nucleus	catalytic activity;protein binding	0.044	0.044	1.00
Q04598	54S ribosomal protein L34, mitochondrial [OS=Saccharomyces cerevisiae S288c]	851692	YDR115W; MRX14; YDR115W	cell organization and biogenesis;metabolic process	cytoplasm;mitochondrion;nucleus;ribosome	structural molecule activity	0.468	0.468	1.00
P14693	sorting assembly machinery 35 kDa subunit [OS=Saccharomyces cerevisiae S288c]	856483	SAM35; YHR083W	cell organization and biogenesis;transport	membrane;mitochondrion	protein binding	0.334	0.334	1.00
P25578	CDP-diacylglycerol--glycerol-3-phosphate 3-phosphatidyltransferase	850352	PGS1; YCL004W	metabolic process	mitochondrion	catalytic activity;nucleotide binding	0.083	0.083	1.00

	[OS=Saccharomyces cerevisiae S288c]								
P36064	COX assembly mitochondrial protein [OS=Saccharomyces cerevisiae S288c]	853721	CMC1; YKL137W	cell organization and biogenesis	membrane;mitochondrion	metal ion binding	0.389	0.389	1.00
P39518	Long-chain-fatty-acid--CoA ligase 2 [OS=Saccharomyces cerevisiae S288c]	856734	FAA2; YER015W	metabolic process;transport	cytoplasm;mitochondrion	catalytic activity;nucleotide binding	0.055	0.055	1.00
P87275	Altered inheritance of mitochondria protein 11 [OS=Saccharomyces cerevisiae S288c]	856829	AIM11; YER093C-A		membrane;mitochondrion		0.389	0.389	1.00
Q06405	ATP synthase subunit f, mitochondrial [OS=Saccharomyces cerevisiae S288c]	851983	ATP17; YDR377W	metabolic process;transport	membrane;mitochondrion	catalytic activity;transporter activity	1.512	1.512	1.00
Q02863	ubiquitin carboxyl-terminal hydrolase 16 [OS=Saccharomyces cerevisiae S288c]	856032	UBP16; YPL072W	metabolic process	membrane;mitochondrion	catalytic activity	0.072	0.072	1.00
P00045	Cytochrome c iso-2 [OS=Saccharomyces cerevisiae S288c]	856672	CYC7; YEL039C	metabolic process;transport	mitochondrion	metal ion binding	0.259	0.259	1.00
P39722	Mitochondrial Rho GTPase 1 [OS=Saccharomyces cerevisiae S288c]	851249	GEM1; YAL048C	cell organization and biogenesis;regulation of biological process;response to stimulus;transport	membrane;mitochondrion	catalytic activity;metal ion binding;nucleotide binding	0.059	0.059	1.00
P39103	Cytochrome c oxidase assembly protein COX14 [OS=Saccharomyces cerevisiae S288c]	854910	COX14; YML129C	cell organization and biogenesis;regulation of biological process	membrane;mitochondrion;organelle lumen	protein binding	0.585	0.585	1.00
Q06011	Protein ECM19 [OS=Saccharomyces cerevisiae S288c]	851106	ECM19; YLR390W	cell organization and biogenesis	membrane;mitochondrion		0.468	0.468	1.00
P40458	Autophagy-related protein 32 [OS=Saccharomyces cerevisiae S288c]	854660	ATG32; YIL146C	metabolic process	membrane;mitochondrion;vacuole	protein binding	0.096	0.096	1.00
P39724	Altered inheritance of mitochondria protein 1 [OS=Saccharomyces cerevisiae S288c]	851251	AIM1; YAL046C				0.334	0.334	1.00
P38909	cytochrome c mitochondrial import factor CYC2 [OS=Saccharomyces cerevisiae S288c]	854202	CYC2; YOR037W	cell organization and biogenesis;metabolic process	membrane;mitochondrion	catalytic activity	0.093	0.093	1.00
P09440	C-1-tetrahydrofolate synthase, mitochondrial [OS=Saccharomyces cerevisiae S288c]	852378	MIS1; YBR084W	metabolic process	cytosol;mitochondrion	catalytic activity;nucleotide binding	1.668	1.771	1.06
P06208-1	2-isopropylmalate synthase [OS=Saccharomyces cerevisiae S288c]	855619	LEU4; YNL104C	metabolic process	cytoplasm;mitochondrion	catalytic activity;protein binding	9	9.608	1.07
P32454	Aminopeptidase 2, mitochondrial [OS=Saccharomyces cerevisiae S288c]	853699	APE2; YKL157W	metabolic process	cytoplasm;extracellular;membrane;mitochondrion;nucleus	catalytic activity;metal ion binding	1.209	1.294	1.07
P30624	Long-chain-fatty-acid--CoA ligase 1 [OS=Saccharomyces cerevisiae S288c]	854495	FAA1; YOR317W	metabolic process;transport	endoplasmic reticulum;membrane;mitochondrion	catalytic activity;nucleotide binding	3.024	3.245	1.07
P40215	External NADH-ubiquinone oxidoreductase 1, mitochondrial [OS=Saccharomyces cerevisiae S288c]	855176	NDE1; YMR145C	metabolic process	mitochondrion	catalytic activity;nucleotide binding	5.469	5.884	1.08
P07806	Valine--tRNA ligase, mitochondrial [OS=Saccharomyces cerevisiae S288c]	852986	VAS1; YGR094W	metabolic process;regulation of biological process	cytoplasm;cytosol;mitochondrion	catalytic activity;nucleotide binding	0.722	0.778	1.08
P21954	Isocitrate dehydrogenase [NADP], mitochondrial [OS=Saccharomyces cerevisiae S288c]	851493	IDP1; YDL066W	metabolic process	mitochondrion	catalytic activity;metal ion binding;nucleotide binding;protein binding	35.869	38.811	1.08
P08417	fumarate hydratase, mitochondrial [OS=Saccharomyces cerevisiae S288c]	855866	FUM1; YPL262W	cell organization and biogenesis;metabolic process	cytoplasm;cytosol;mitochondrion;organelle lumen	catalytic activity	13.874	15.103	1.09
P39987	Heat shock protein SSC3, mitochondrial [OS=Saccharomyces cerevisiae S288c]	856682	ECM10; YEL030W	cell organization and biogenesis;metabolic process;transport	mitochondrion	nucleotide binding;protein binding	2.162	2.36	1.09
P07256	Cytochrome b-c1 complex subunit 1, mitochondrial [OS=Saccharomyces cerevisiae S288c]	852235	COR1; YBL045C	metabolic process;transport	membrane;mitochondrion	catalytic activity;metal ion binding;protein binding;transporter activity	18.783	20.544	1.09
P07257	Cytochrome b-c1 complex subunit 2, mitochondrial [OS=Saccharomyces	856321	QCR2; YPR191W	metabolic process;transport	membrane;mitochondrion	catalytic activity;metal ion binding;protein	33.551	36.751	1.10

	cerevisiae S288c]					binding;transporter activity			
P16547	Mitochondrial outer membrane protein OM45 [OS=Saccharomyces cerevisiae S288c]	854670	OM45; YIL136W		membrane;mitochondrion		274.423	300.995	1.10
P54783	D-arabinono-1,4-lactone oxidase [OS=Saccharomyces cerevisiae S288c]	854888	ALO1; YML086C	metabolic process;response to stimulus	membrane;mitochondrion	catalytic activity;nucleotide binding	3.299	3.642	1.10
P16451	Pyruvate dehydrogenase complex protein X component, mitochondrial [OS=Saccharomyces cerevisiae S288c]	853107	PDX1; YGR193C	metabolic process	mitochondrion;organelle lumen	catalytic activity;structural molecule activity	4.055	4.505	1.11
P53982	isocitrate dehydrogenase [NADP] [OS=Saccharomyces cerevisiae S288c]	855723	IDP3; YNL009W	metabolic process	cytoplasm;mitochondrion	catalytic activity;metal ion binding;nucleotide binding	1.943	2.162	1.11
Q05931	Heat shock protein SSQ1, mitochondrial [OS=Saccharomyces cerevisiae S288c]	851084	SSQ1; YLR369W	cell organization and biogenesis;metabolic process	mitochondrion;organelle lumen	nucleotide binding;protein binding	0.995	1.113	1.12
P38077	ATP synthase subunit gamma, mitochondrial [OS=Saccharomyces cerevisiae S288c]	852327	ATP3; YBR039W	metabolic process;transport	membrane;mitochondrion	catalytic activity;transporter activity	11.328	12.689	1.12
P25374	Cysteine desulfurase, mitochondrial [OS=Saccharomyces cerevisiae S288c]	850343	NFS1; YCL017C	cell organization and biogenesis;cellular homeostasis;metabolic process	mitochondrion;nucleus	catalytic activity;metal ion binding;protein binding	2.594	2.914	1.12
P38071	Enoyl-[acyl-carrier protein] reductase [NADPH, B-specific], mitochondrial [OS=Saccharomyces cerevisiae S288c]	852314	ETR1; YBR026C	metabolic process	mitochondrion;organelle lumen	catalytic activity;DNA binding;metal ion binding	7.111	8.006	1.13
P01120	Ras-like protein 2 [OS=Saccharomyces cerevisiae S288c]	855625	RAS2; YNL098C	cell communication;cell differentiation;metabolic process;regulation of biological process;response to stimulus;transport	membrane;mitochondrion;nucleus	catalytic activity;nucleotide binding;protein binding	4.484	5.062	1.13
P16622	Ferrochelatase, mitochondrial [OS=Saccharomyces cerevisiae S288c]	854347	HEM15; YOR176W	metabolic process	membrane;mitochondrion	catalytic activity	5.579	6.305	1.13
P48527	Tyrosine--tRNA ligase, mitochondrial [OS=Saccharomyces cerevisiae S288c]	856007	MSY1; YPL097W	metabolic process	cytoplasm;cytosol;mitochondrion;organelle lumen	catalytic activity;nucleotide binding;RNA binding	1.154	1.31	1.14
Q04458	Fatty aldehyde dehydrogenase HFD1 [OS=Saccharomyces cerevisiae S288c]	855137	HFD1; YMR110C	metabolic process	endoplasmic reticulum;endosome;membrane;mitochondrion	catalytic activity	1.783	2.03	1.14
P53266	Cytochrome oxidase assembly protein SHY1 [OS=Saccharomyces cerevisiae S288c]	853009	SHY1; YGR112W	cell organization and biogenesis	membrane;mitochondrion	protein binding	1.783	2.03	1.14
P27929	37S ribosomal protein NAM9, mitochondrial [OS=Saccharomyces cerevisiae S288c]	855585	NAM9; YNL137C	cell organization and biogenesis;metabolic process;regulation of biological process	mitochondrion;ribosome	RNA binding;structural molecule activity	0.931	1.062	1.14
P43567	alanine--glyoxylate aminotransferase 1 [OS=Saccharomyces cerevisiae S288c]	850514	AGX1; YFL030W	metabolic process	cytosol;mitochondrion	catalytic activity	2.481	2.831	1.14
P38988	Mitochondrial GTP/GDP carrier protein 1 [OS=Saccharomyces cerevisiae S288c]	851329	GGC1; YDL198C	cell organization and biogenesis;cellular homeostasis;metabolic process;transport	membrane;mitochondrion	structural molecule activity;transporter activity	3.642	4.179	1.15
P43616	Cys-Gly metallodipeptidase dug1 [OS=Saccharomyces cerevisiae S288c]	850605	DUG1; YFR044C	metabolic process	cytoplasm;mitochondrion;ribosome	catalytic activity;metal ion binding	1.154	1.326	1.15
P28817	3-hydroxyisobutyryl-CoA hydrolase, mitochondrial [OS=Saccharomyces cerevisiae S288c]	851606	EHD3; YDR036C	metabolic process	mitochondrion	catalytic activity	1.212	1.395	1.15
Q12480	Probable electron transfer flavoprotein subunit alpha, mitochondrial [OS=Saccharomyces cerevisiae S288c]	856112	AIM45; YPR004C	metabolic process;transport	mitochondrion;organelle lumen	catalytic activity;nucleotide binding	3.467	4.012	1.16
P10662	37S ribosomal protein MRP1, mitochondrial [OS=Saccharomyces cerevisiae S288c]	851948	MRP1; YDR347W	cell organization and biogenesis;metabolic process;response to stimulus	mitochondrion;ribosome	antioxidant activity;catalytic activity;metal ion binding;structural molecule activity	2.981	3.467	1.16
P39525	3-oxoacyl-[acyl-carrier-protein] synthase homolog [OS=Saccharomyces cerevisiae S288c]	856790	CEM1; YER061C	metabolic process	mitochondrion	catalytic activity	1.848	2.162	1.17

P12687	54S ribosomal protein L2, mitochondrial [OS=Saccharomyces cerevisiae S288c]	855727	MRP7; YNL005C	cell organization and biogenesis;metabolic process	mitochondrion;ribosome	catalytic activity;RNA binding;structural molecule activity	1.848	2.162	1.17
P53881	54S ribosomal protein L22, mitochondrial [OS=Saccharomyces cerevisiae S288c]	855544	MRPL22; YNL177C	cell organization and biogenesis;metabolic process	mitochondrion;ribosome	RNA binding;structural molecule activity	1.994	2.34	1.17
Q12230	Sphingolipid long chain base-responsive protein LSP1 [OS=Saccharomyces cerevisiae S288c]	856103	LSP1; YPL004C	regulation of biological process;response to stimulus;transport	cytoplasm;membrane;mitochondrion		14.849	17.478	1.18
P12695	Dihydrolipoyllysine-residue acetyltransferase component of pyruvate dehydrogenase complex, mitochondrial [OS=Saccharomyces cerevisiae S288c]	855653	LAT1; YNL071W	metabolic process	mitochondrion;organelle lumen	catalytic activity	7.003	8.284	1.18
P36112	MICOS complex subunit Mic60 [OS=Saccharomyces cerevisiae S288c]	853886	FCJ1; MIC60; YKR016W	cell organization and biogenesis;transport	membrane;mitochondrion		12.895	15.379	1.19
Q08223	Altered inheritance of mitochondria protein 39, mitochondrial [OS=Saccharomyces cerevisiae S288c]	854103	AIM39; YOL053W		cytosol;membrane;mitochondrion		0.711	0.848	1.19
Q02486	ARS-binding factor 2, mitochondrial [OS=Saccharomyces cerevisiae S288c]	855094	ABF2; YMR072 W	cell organization and biogenesis;metabolic process;regulation of biological process	chromosome;mitochondrion;nucleus	DNA binding	69.17	82.768	1.20
P38885	Altered inheritance of mitochondria protein 46, mitochondrial [OS=Saccharomyces cerevisiae S288c]	856606	AIM46; YHR199C		mitochondrion	catalytic activity	1.404	1.683	1.20
P22136	ATPase expression protein 2, mitochondrial [OS=Saccharomyces cerevisiae S288c]	855325	AEP2; YMR282C	metabolic process;regulation of biological process	mitochondrion	RNA binding	0.359	0.431	1.20
P39002	long-chain-fatty-acid--CoA ligase 3 [OS=Saccharomyces cerevisiae S288c]	854808	FAA3; YIL009W	metabolic process		catalytic activity;nucleotide binding	0.413	0.496	1.20
Q08818	Meiotic sister-chromatid recombination protein 6, mitochondrial [OS=Saccharomyces cerevisiae S288c]	854536	MSC6; YOR354C	metabolic process	mitochondrion;organelle lumen	RNA binding	1.154	1.387	1.20
P02992	elongation factor Tu, mitochondrial [OS=Saccharomyces cerevisiae S288c]	854359	TUF1; YOR187 W	cell organization and biogenesis;metabolic process	mitochondrion	catalytic activity;nucleotide binding;RNA binding	23.245	27.943	1.20
P49367	Homoaconitase, mitochondrial [OS=Saccharomyces cerevisiae S288c]	851820	LYS4; YDR234 W	metabolic process	mitochondrion	catalytic activity;metal ion binding;protein binding	1.276	1.54	1.21
Q12320	Hydroxyacylglutathione hydrolase, mitochondrial [OS=Saccharomyces cerevisiae S288c]	854205	GLO4; YOR040 W	metabolic process	mitochondrion;organelle lumen	catalytic activity;metal ion binding	1.081	1.31	1.21
P08067	cytochrome b-c1 complex subunit Rieske, mitochondrial [OS=Saccharomyces cerevisiae S288c]	856689	RIP1; YEL024W	metabolic process;transport	membrane;mitochondrion	catalytic activity;metal ion binding;transporter activity	55.234	67.129	1.22
P25719	Peptidyl-prolyl cis-trans isomerase C, mitochondrial [OS=Saccharomyces cerevisiae S288c]	854897	CPR3; YML078 W	cell death;metabolic process	mitochondrion;organelle lumen	catalytic activity	2.981	3.642	1.22
Q03798	Altered inheritance of mitochondria protein 36, mitochondrial [OS=Saccharomyces cerevisiae S288c]	855189	AIM36; YMR157C		membrane;mitochondrion		1.336	1.637	1.23
P17558	37S ribosomal protein PET123, mitochondrial [OS=Saccharomyces cerevisiae S288c]	854329	PET123; YOR158 W	cell organization and biogenesis;metabolic process	mitochondrion;ribosome	structural molecule activity	1.448	1.783	1.23
P04037	cytochrome c oxidase subunit 4, mitochondrial [OS=Saccharomyces cerevisiae S288c]	852688	COX4; YGL187C	cell organization and biogenesis;metabolic process;transport	membrane;mitochondrion	catalytic activity;metal ion binding;transporter activity	34.112	42.288	1.24
P38705	Serine--tRNA ligase, mitochondrial [OS=Saccharomyces cerevisiae S288c]	856402	DIA4; YHR011 W	metabolic process	cytoplasm;mitochondrion;organelle lumen	catalytic activity;nucleotide binding	0.417	0.52	1.25
P48360	Probable NADPH:adrenodoxin oxidoreductase, mitochondrial [OS=Saccharomyces cerevisiae S288c]	851982	ARH1; YDR376 W	cellular homeostasis;metabolic process;transport	cytoplasm;membrane;mitochondrion;organelle lumen	catalytic activity	0.45	0.562	1.25

Q04599	54S ribosomal protein L1, mitochondrial [OS=Saccharomyces cerevisiae S288c]	851694	MRPL1; YDR116C	cell organization and biogenesis;metabolic process	mitochondrion;ribosome	RNA binding;structural molecule activity	1.069	1.336	1.25
P19516	Cytochrome c oxidase assembly protein COX11, mitochondrial [OS=Saccharomyces cerevisiae S288c]	855971	COX11; YPL132W	cell organization and biogenesis;metabolic process	membrane;mitochondrion;ribosome	metal ion binding	1.069	1.336	1.25
P39006	Phosphatidylserine decarboxylase proenzyme 1, mitochondrial [OS=Saccharomyces cerevisiae S288c]	855552	PSD1; YNL169C	metabolic process;regulation of biological process	membrane;mitochondrion	catalytic activity	0.487	0.61	1.25
P36046	Mitochondrial intermembrane space import and assembly protein 40 [OS=Saccharomyces cerevisiae S288c]	853639	MIA40; YKL195W	cellular component movement;metabolic process;response to stimulus;transport	membrane;mitochondrion	catalytic activity;protein binding;transporter activity	2.3	2.914	1.27
P37267	Assembly factor cbp4 [OS=Saccharomyces cerevisiae S288c]			cell organization and biogenesis	membrane;mitochondrion		5.579	7.111	1.27
P32335	Protein MSS51, mitochondrial [OS=Saccharomyces cerevisiae S288c]	850900	MSS51; YLR203C	metabolic process;regulation of biological process	membrane;mitochondrion	protein binding;translation regulator activity	1.512	1.929	1.28
P14908	Mitochondrial transcription factor 1 [OS=Saccharomyces cerevisiae S288c]	855268	MTF1; YMR228 W	metabolic process;regulation of biological process	mitochondrion;organelle lumen	catalytic activity;DNA binding;RNA binding	0.778	0.995	1.28
P25375	Saccharolysin [OS=Saccharomyces cerevisiae S288c]	850301	PRD1; YCL057W	metabolic process	cytoplasm;Golgi;mitochondrion;vacuole	catalytic activity;metal ion binding	0.216	0.278	1.29
P11914	Mitochondrial-processing peptidase subunit alpha [OS=Saccharomyces cerevisiae S288c]	856419	MAS2; YHR024C	metabolic process;transport	membrane;mitochondrion;organelle lumen	catalytic activity;metal ion binding;protein binding	2.311	2.981	1.29
Q04935	Cytochrome c oxidase protein 20, mitochondrial [OS=Saccharomyces cerevisiae S288c]	851817	COX20; YDR231C	cell organization and biogenesis;metabolic process	membrane;mitochondrion	protein binding	1.894	2.455	1.30
P42847	37S ribosomal protein S18, mitochondrial [OS=Saccharomyces cerevisiae S288c]	855410	MRPS18; YNL306W	cell organization and biogenesis;metabolic process	mitochondrion;ribosome	RNA binding;structural molecule activity	1.054	1.371	1.30
P53969	Sorting assembly machinery 50 kDa subunit [OS=Saccharomyces cerevisiae S288c]	855705	SAM50; YNL026W	cell organization and biogenesis;transport	membrane;mitochondrion	protein binding;transporter activity	0.374	0.487	1.30
P07213	Mitochondrial import receptor subunit TOM70 [OS=Saccharomyces cerevisiae S288c]	855602	TOM70; YNL121C	cell organization and biogenesis;transport	membrane;mitochondrion	protein binding;transporter activity	1.728	2.257	1.31
Q01852	Mitochondrial import inner membrane translocase subunit TIM44 [OS=Saccharomyces cerevisiae S288c]	854790	TIM44; YIL022W	transport	membrane;mitochondrion	nucleotide binding;protein binding	4.125	5.404	1.31
P07266	Mitochondrial RNA-splicing protein MRS1 [OS=Saccharomyces cerevisiae S288c]	854839	MRS1; YIR021W	cell organization and biogenesis;metabolic process	mitochondrion;organelle lumen	catalytic activity;DNA binding;RNA binding	0.425	0.557	1.31
Q03327	Mitochondrial fusion and transport protein Ugo1 [OS=Saccharomyces cerevisiae S288c]	852081	UGO1; YDR470C	cell organization and biogenesis;metabolic process	membrane;mitochondrion	protein binding;structural molecule activity	0.425	0.557	1.31
Q08968	UPF0061 protein FMP40 [OS=Saccharomyces cerevisiae S288c]	855879	FMP40; YPL222W		mitochondrion	protein binding	2.495	3.27	1.31
Q02776	Mitochondrial import inner membrane translocase subunit TIM50 [OS=Saccharomyces cerevisiae S288c]	856042	TIM50; YPL063W	cell organization and biogenesis;metabolic process;transport	membrane;mitochondrion	catalytic activity;protein binding;transporter activity	7.483	9.857	1.32
P36528	54S ribosomal protein L17, mitochondrial [OS=Saccharomyces cerevisiae S288c]	855469	MRPL17; YNL252C	cell organization and biogenesis;metabolic process	mitochondrion;ribosome	structural molecule activity	0.52	0.688	1.32
P36163	mitochondrial metalloendopeptidase OMA1 [OS=Saccharomyces cerevisiae S288c]	853962	OMA1; YKR087C	metabolic process	membrane;mitochondrion	catalytic activity;metal ion binding	0.551	0.73	1.32
Q03799	37S ribosomal protein S8, mitochondrial [OS=Saccharomyces cerevisiae S288c]	855190	MRPS8; YMR158 W	cell organization and biogenesis;metabolic process	mitochondrion;ribosome	structural molecule activity	1.424	1.894	1.33
Q12283	Malonyl CoA-acyl carrier protein transacylase, mitochondrial [OS=Saccharomyces cerevisiae S288c]	854396	MCT1; YOR221C	metabolic process	mitochondrion	catalytic activity	0.668	0.896	1.34

P00424	Cytochrome c oxidase polypeptide 5A, mitochondrial [OS=Saccharomyces cerevisiae S288c]	855675	COX5A; YNL052W	metabolic process;transport	membrane;mitochondrion	catalytic activity;transporter activity	1.848	2.511	1.36
Q03713	Respiratory supercomplex factor 1, mitochondrial [OS=Saccharomyces cerevisiae S288c]	854978	RCF1; YML030W	cell organization and biogenesis	membrane;mitochondrion		2.162	2.981	1.38
P07236	Threonine--tRNA ligase, mitochondrial [OS=Saccharomyces cerevisiae S288c]	853640	MST1; YKL194C	metabolic process	cytoplasm;mitochondrion;organelle lumen	catalytic activity;nucleotide binding;RNA binding	0.233	0.322	1.38
P36066	Protein MRG3-like [OS=Saccharomyces cerevisiae S288c]	853725	YKL133C; YKL133C	metabolic process	membrane;mitochondrion	protein binding	0.655	0.911	1.39
P18900	hexaprenyl pyrophosphate synthase, mitochondrial [OS=Saccharomyces cerevisiae S288c]	852288	COQ1; YBR003W	metabolic process	membrane;mitochondrion	catalytic activity;metal ion binding;protein binding	0.711	0.995	1.40
Q02784	Monothiol glutaredoxin-5, mitochondrial [OS=Saccharomyces cerevisiae S288c]	856048	GRX5; YPL059W	cell organization and biogenesis;cellular homeostasis;metabolic process;regulation of biological process;response to stimulus	mitochondrion;organelle lumen	catalytic activity;metal ion binding	2.594	3.642	1.40
P38341	MICOS complex subunit MIC12 [OS=Saccharomyces cerevisiae S288c]	852566	AIM5; MIC12; YBR262C	cell organization and biogenesis	membrane;mitochondrion		2.594	3.642	1.40
P47015	Altered inheritance of mitochondria protein 23, mitochondrial [OS=Saccharomyces cerevisiae S288c]	853310	AIM23; YJL131C	metabolic process	mitochondrion	RNA binding	0.35	0.492	1.41
P53140	Protein RMD9, mitochondrial [OS=Saccharomyces cerevisiae S288c]	852771	RMD9; YGL107C	cell differentiation;metabolic process;regulation of biological process	membrane;mitochondrion	RNA binding	1.268	1.783	1.41
P07342	Acetolactate synthase catalytic subunit, mitochondrial [OS=Saccharomyces cerevisiae S288c]	855135	ILV2; YMR108W	metabolic process	mitochondrion	catalytic activity;metal ion binding;nucleotide binding	5.236	7.377	1.41
Q04689	Altered inheritance of mitochondria protein 32 [OS=Saccharomyces cerevisiae S288c]	854955	AIM32; YML050W				0.369	0.52	1.41
P54857	lipase 2 [OS=Saccharomyces cerevisiae S288c]	851628	TGL2; YDR058C	metabolic process;transport	mitochondrion	catalytic activity	0.369	0.52	1.41
P02381	Ribosomal protein VAR1, mitochondrial [OS=Saccharomyces cerevisiae S288c]	854586	VAR1; Q0140	cell organization and biogenesis;metabolic process	mitochondrion;ribosome	structural molecule activity	1.31	1.848	1.41
O14467	multiprotein-bridging factor 1 [OS=Saccharomyces cerevisiae S288c]	854474	MBF1; YOR298C-A	metabolic process;regulation of biological process	cytoplasm;mitochondrion;nucleus	DNA binding	1.31	1.848	1.41
P36527	54S ribosomal protein L28, mitochondrial [OS=Saccharomyces cerevisiae S288c]	852073	MRPL28; YDR462W	cell organization and biogenesis;metabolic process	mitochondrion;ribosome	structural molecule activity	1.31	1.848	1.41
P38152	Tricarboxylate transport protein [OS=Saccharomyces cerevisiae S288c]	852594	CTP1; YBR291C	metabolic process;transport	membrane;mitochondrion	structural molecule activity;transporter activity	0.389	0.551	1.42
P28239	Inorganic pyrophosphatase, mitochondrial [OS=Saccharomyces cerevisiae S288c]	855309	PPA2; YMR267W	metabolic process	cytoplasm;mitochondrion	catalytic activity;metal ion binding	0.413	0.585	1.42
Q12298	uncharacterized ABC transporter ATP-binding protein YDR061W [OS=Saccharomyces cerevisiae S288c]	851633	YDR061W; YDR061W		mitochondrion	catalytic activity;nucleotide binding	0.425	0.604	1.42
Q03976	37S ribosomal protein S24, mitochondrial [OS=Saccharomyces cerevisiae S288c]	851755	RSM24; YDR175C	cell organization and biogenesis;metabolic process	mitochondrion;ribosome	structural molecule activity	0.905	1.291	1.43
P38325	Mitochondrial outer membrane protein OM14 [OS=Saccharomyces cerevisiae S288c]	852531	OM14; YBR230C	transport	membrane;mitochondrion		9	12.895	1.43
Q04728	Arginine biosynthesis bifunctional protein ArgJ, mitochondrial [OS=Saccharomyces cerevisiae S288c]	855084	ARG7; YMR062C	metabolic process	mitochondrion;organelle lumen	catalytic activity	1.154	1.656	1.44
P38323	ATP-dependent clpX-like chaperone, mitochondrial [OS=Saccharomyces cerevisiae S288c]	852528	MCX1; YBR227C	metabolic process	membrane;mitochondrion;organelle lumen	catalytic activity;nucleotide binding	0.501	0.719	1.44

P41735	5-demethoxyubiquinone hydroxylase, mitochondrial [OS=Saccharomyces cerevisiae S288c]	854292	CAT5; YOR125C	metabolic process	membrane;mitochondrion	catalytic activity;metal ion binding	0.54	0.778	1.44
Q08023	Protein FMP25, mitochondrial [OS=Saccharomyces cerevisiae S288c]	850766	FMP25; YLR077W	cell organization and biogenesis	membrane;mitochondrion		0.968	1.412	1.46
P38127	mitochondrial carrier protein RIM2 [OS=Saccharomyces cerevisiae S288c]	852491	RIM2; YBR192W	cell organization and biogenesis;metabolic process;transport	membrane;mitochondrion	structural molecule activity;transporter activity	1.239	1.818	1.47
P25573	Mitochondrial inner membrane i-AAA protease supercomplex subunit MGR1 [OS=Saccharomyces cerevisiae S288c]	850313	MGR1; YCL044C	metabolic process	membrane;mitochondrion	protein binding	0.701	1.031	1.47
P35180	mitochondrial import receptor subunit Tom20 [OS=Saccharomyces cerevisiae S288c]	852973	TOM20; YGR082 W	cell organization and biogenesis;transport	membrane;mitochondrion	protein binding;transporter activity	0.701	1.031	1.47
P08466	mitochondrial nuclease [OS=Saccharomyces cerevisiae S288c]	853222	NUC1; YJL208C	cell death;metabolic process	membrane;mitochondrion;nucleus	catalytic activity;metal ion binding	1.336	1.976	1.48
P40530	[Pyruvate dehydrogenase (acetyl-transferring)] kinase 1, mitochondrial [OS=Saccharomyces cerevisiae S288c]	854769	PKP1; YIL042C	metabolic process	mitochondrion;organelle lumen	catalytic activity;nucleotide binding;protein binding	0.931	1.404	1.51
P48237	Mitochondrial group I intron splicing factor CCM1 [OS=Saccharomyces cerevisiae S288c]	853053	CCM1; YGR150C	cell organization and biogenesis;metabolic process	mitochondrion	RNA binding	0.089	0.136	1.53
Q03153	ATPase synthesis protein 25, mitochondrial [OS=Saccharomyces cerevisiae S288c]	855123	ATP25; YMR098C	cell organization and biogenesis;regulation of biological process	membrane;mitochondrion		0.116	0.179	1.54
P36525	54S ribosomal protein L24, mitochondrial [OS=Saccharomyces cerevisiae S288c]	855231	MRPL24; YMR193 W	cell organization and biogenesis;metabolic process	mitochondrion;ribosome	RNA binding;structural molecule activity	1.929	2.981	1.55
P32048	Lysine--tRNA ligase, mitochondrial [OS=Saccharomyces cerevisiae S288c]	855651	MSK1; YNL073W	cell organization and biogenesis;metabolic process;transport	cytoplasm;mitochondrion;organelle lumen	catalytic activity;nucleotide binding;RNA binding	0.122	0.189	1.55
P33893	Glutamyl-tRNA(Gln) amidotransferase subunit B, mitochondrial [OS=Saccharomyces cerevisiae S288c]	852198	PET112; YBL080C	cell organization and biogenesis;metabolic process	mitochondrion	catalytic activity;nucleotide binding	0.15	0.233	1.55
P36101	tRNA threonylcarbamoyladenosine dehydratase 2 [OS=Saccharomyces cerevisiae S288c]	853841	TCD2; YKL027W ; YKL027W	metabolic process	cytosol;membrane;mitochondrion	catalytic activity;nucleotide binding	0.616	0.957	1.55
P47150	37S ribosomal protein S7, mitochondrial [OS=Saccharomyces cerevisiae S288c]	853578	RSM7; YJR113C	cell organization and biogenesis;metabolic process	mitochondrion;ribosome	RNA binding;structural molecule activity	0.616	0.957	1.55
P38771	Ribosome-recycling factor, mitochondrial [OS=Saccharomyces cerevisiae S288c]	856433	RRF1; YHR038 W	cell organization and biogenesis;metabolic process	mitochondrion	RNA binding	1.254	1.955	1.56
Q06510	Lysophosphatidylcholine acyltransferase [OS=Saccharomyces cerevisiae S288c]	856262	TAZ1; YPR140W	cell organization and biogenesis;metabolic process;transport	membrane;mitochondrion	catalytic activity	0.194	0.304	1.57
P00427	Cytochrome c oxidase subunit 6, mitochondrial [OS=Saccharomyces cerevisiae S288c]	856448	COX6; YHR051 W	metabolic process;transport	membrane;mitochondrion	catalytic activity;metal ion binding;transporter activity	4.623	7.254	1.57
Q12467	MIOREX complex component 4 [OS=Saccharomyces cerevisiae S288c]	855935	YPL168W ; MRX4; YPL168W		membrane;mitochondrion		0.202	0.318	1.57
P40012	protoporphyrinogen oxidase [OS=Saccharomyces cerevisiae S288c]	856733	HEM14; YER014W	metabolic process	membrane;mitochondrion	catalytic activity	1.482	2.36	1.59
P32266	Dynamin-like GTPase MGM1, mitochondrial [OS=Saccharomyces cerevisiae S288c]	854386	MGM1; YOR211C	cell organization and biogenesis	membrane;mitochondrion	catalytic activity;nucleotide binding;protein binding	0.28	0.448	1.60
P31334	54S ribosomal protein L9, mitochondrial [OS=Saccharomyces cerevisiae S288c]	853135	MRPL9; YGR220C	cell organization and biogenesis;metabolic process	mitochondrion;ribosome	structural molecule activity	0.311	0.501	1.61
P40098	uncharacterized mitochondrial membrane protein FMP10 [OS=Saccharomyces cerevisiae S288c]	856931	FMP10; YER182W		membrane;mitochondrion		2.36	3.833	1.62
P22353	54S ribosomal protein L8, mitochondrial	853382	MRPL8; YJL063C	cell organization and biogenesis;metabolic	mitochondrion;ribosome	structural molecule activity	0.359	0.585	1.63

	[OS=Saccharomyces cerevisiae S288c]			process					
P34222	Peptidyl-tRNA hydrolase 2 [OS=Saccharomyces cerevisiae S288c]	852223	PTH2; YBL057C	regulation of biological process	cytoplasm;cytosol;membrane;mitochondrion	catalytic activity;protein binding	0.425	0.701	1.65
P38175	37S ribosomal protein MRP21, mitochondrial [OS=Saccharomyces cerevisiae S288c]	852188	MRP21; YBL090W	cell organization and biogenesis;metabolic process	mitochondrion;ribosome	structural molecule activity	0.425	0.701	1.65
P22135	Protein ATP12, mitochondrial [OS=Saccharomyces cerevisiae S288c]	853259	ATP12; YJL180C	cell organization and biogenesis	mitochondrion	protein binding	0.468	0.778	1.66
P40515	Mitochondria fission 1 protein [OS=Saccharomyces cerevisiae S288c]	854745	FIS1; YIL065C	cell death;cell organization and biogenesis;regulation of biological process	membrane;mitochondrion	protein binding	0.468	0.778	1.66
Q03430	37S ribosomal protein RSM28, mitochondrial [OS=Saccharomyces cerevisiae S288c]	852105	RSM28; YDR494 W	cell organization and biogenesis;metabolic process	mitochondrion;ribosome	structural molecule activity	2.675	4.484	1.68
P42949	Mitochondrial import inner membrane translocase subunit TIM16 [OS=Saccharomyces cerevisiae S288c]	853340	PAM16; YJL104W	transport	membrane;mitochondrion	protein binding	0.52	0.874	1.68
P36520	54S ribosomal protein L10, mitochondrial [OS=Saccharomyces cerevisiae S288c]	855436	MRPL10; YNL284C	cell organization and biogenesis;metabolic process	mitochondrion;ribosome	structural molecule activity	0.551	0.931	1.69
P28321	monoglyceride lipase [OS=Saccharomyces cerevisiae S288c]	853768	YJU3; YKL094W	metabolic process;regulation of biological process	cytoplasm;endoplasmic reticulum;membrane;mitochondrion	catalytic activity	0.551	0.931	1.69
P53598	Succinyl-CoA ligase [ADP-forming] subunit alpha, mitochondrial [OS=Saccharomyces cerevisiae S288c]	854310	LSC1; YOR142 W	metabolic process	cytosol;mitochondrion	catalytic activity;nucleotide binding	9	15.238	1.69
P22354	54S ribosomal protein L20, mitochondrial [OS=Saccharomyces cerevisiae S288c]	853960	MRPL20; YKR085C	cell organization and biogenesis;metabolic process	mitochondrion;ribosome	structural molecule activity	1.276	2.162	1.69
P40471	NADPH-dependent 1-acyldihydroxyacetone phosphate reductase [OS=Saccharomyces cerevisiae S288c]	854682	AYR1; YIL124W	metabolic process	cytoplasm;endoplasmic reticulum;membrane;mitochondrion	catalytic activity	0.585	0.995	1.70
P42844	Mitochondrial protein import protein ZIM17 [OS=Saccharomyces cerevisiae S288c]	855406	ZIM17; YNL310C	cell organization and biogenesis;metabolic process;regulation of biological process;response to stimulus;transport	membrane;mitochondrion;organelle lumen	metal ion binding;protein binding	0.585	0.995	1.70
P00128	Cytochrome b-c1 complex subunit 7 [OS=Saccharomyces cerevisiae S288c]	852142	QCR7; YDR529C	cell organization and biogenesis;metabolic process;transport	membrane;mitochondrion	catalytic activity;transporter activity	15.681	26.826	1.71
P40165	NAD(P)H-hydrate epimerase [OS=Saccharomyces cerevisiae S288c]	855521	YNL200C; YNL200C	metabolic process	cytoplasm;mitochondrion	catalytic activity;metal ion binding;nucleotide binding	1.61	2.831	1.76
Q99297	Mitochondrial 2-oxodicarboxylate carrier 2 [OS=Saccharomyces cerevisiae S288c]	854397	ODC2; YOR222 W	metabolic process;transport	membrane;mitochondrion	structural molecule activity;transporter activity	0.778	1.371	1.76
P19262	Dihydrolipoyllysine-residue succinyltransferase component of 2-oxoglutarate dehydrogenase complex, mitochondrial [OS=Saccharomyces cerevisiae S288c]	851726	KGD2; YDR148C	cell organization and biogenesis;metabolic process	mitochondrion	catalytic activity;protein binding	5.7	10.053	1.76
P54003	Protein SUR7 [OS=Saccharomyces cerevisiae S288c]	854953	SUR7; YML052 W	cell differentiation;transpo rt	membrane;mitochondrion		0.848	1.512	1.78
P15424	ATP-dependent RNA helicase MSS116, mitochondrial [OS=Saccharomyces cerevisiae S288c]	851775	MSS116; YDR194C	metabolic process;regulation of biological process	mitochondrion;organelle lumen	catalytic activity;nucleotide binding;RNA binding	1.154	2.082	1.80
P10849	mitochondrial transcription factor 2 [OS=Saccharomyces cerevisiae S288c]	851517	MTF2; YDL044C	metabolic process	mitochondrion;organelle lumen	protein binding;RNA binding	0.259	0.468	1.81
Q12289	mitochondrial carnitine carrier [OS=Saccharomyces cerevisiae S288c]	854267	CRC1; YOR100C	metabolic process;transport	membrane;mitochondrion	structural molecule activity;transporter activity	0.931	1.683	1.81
P51998	54S ribosomal protein yml6, mitochondrial [OS=Saccharomyces cerevisiae S288c]	854983	YML6; YML025C	cell organization and biogenesis;metabolic process	mitochondrion;ribosome	RNA binding;structural molecule activity	0.931	1.683	1.81
P53230	Phosphatidate cytidylyltransferase, mitochondrial	852937	TAM41; YGR046 W	metabolic process	membrane;mitochondrion;organelle lumen	catalytic activity	0.292	0.532	1.82

	[OS=Saccharomyces cerevisiae S288c]								
P43122	tRNA N6-adenosine threonylcarbamoyltransferase, mitochondrial [OS=Saccharomyces cerevisiae S288c]	851454	QRI7; YDL104C	cell organization and biogenesis;metabolic process	mitochondrion	catalytic activity;metal ion binding	0.334	0.616	1.84
P38626	NADH-cytochrome b5 reductase 1 [OS=Saccharomyces cerevisiae S288c]	854768	CBR1; YIL043C	metabolic process	endoplasmic reticulum;membrane;mitochondrion;nucleus	catalytic activity	0.334	0.616	1.84
Q05648	MIOREX complex component 10 [OS=Saccharomyces cerevisiae S288c]	851876	YDR282C; MRX10; YDR282C		membrane;mitochondrion		0.35	0.65	1.86
Q02771	Protein PET117, mitochondrial [OS=Saccharomyces cerevisiae S288c]	856786	PET117; YER058W	cell organization and biogenesis	mitochondrion		1.154	2.162	1.87
P36775	Lon protease homolog, mitochondrial [OS=Saccharomyces cerevisiae S288c]	852259	PIM1; YBL022C	cell organization and biogenesis;metabolic process;regulation of biological process;response to stimulus	cytoplasm;mitochondrion;organelle lumen	catalytic activity;DNA binding;nucleotide binding;RNA binding	1.222	2.311	1.89
P38120	37S ribosomal protein S9, mitochondrial [OS=Saccharomyces cerevisiae S288c]	852443	MRPS9; YBR146W	cell organization and biogenesis;metabolic process	mitochondrion;ribosome	RNA binding;structural molecule activity	1.254	2.384	1.90
P40496	37S ribosomal protein S25, mitochondrial [OS=Saccharomyces cerevisiae S288c]	854715	RSM25; YIL093C	cell organization and biogenesis;metabolic process	mitochondrion;ribosome	structural molecule activity	0.468	0.896	1.91
Q06668	Methyltransferase OMS1, mitochondrial [OS=Saccharomyces cerevisiae S288c]	851911	OMS1; YDR316W	metabolic process	membrane;mitochondrion	catalytic activity	0.484	0.931	1.92
P21771	37S ribosomal protein S28, mitochondrial [OS=Saccharomyces cerevisiae S288c]	851937	MRPS28; YDR337W	cell organization and biogenesis;metabolic process	mitochondrion;ribosome	RNA binding;structural molecule activity	0.833	1.637	1.97
P25038	Translation initiation factor IF-2, mitochondrial [OS=Saccharomyces cerevisiae S288c]	854135	IFM1; YOL023W	cell organization and biogenesis;metabolic process	mitochondrion;nucleus	catalytic activity;nucleotide binding;RNA binding	0.054	0.11	2.04
P53848	Folic acid synthesis protein fol1 [OS=Saccharomyces cerevisiae S288c]	855465	FOL1; YNL256W	metabolic process	cytoplasm;membrane;mitochondrion	catalytic activity;metal ion binding;nucleotide binding	0.045	0.093	2.07
Q08622	Genetic interactor of prohibitins 3, mitochondrial [OS=Saccharomyces cerevisiae S288c]	854380	GEP3; YOR205C	metabolic process;transport	cytosol;membrane;mitochondrion	catalytic activity;nucleotide binding;transporter activity	0.059	0.122	2.07
P34231	Uncharacterized protein YKL187C [OS=Saccharomyces cerevisiae S288c]	853648	FAT3; YKL187C; YKL187C	cell organization and biogenesis;transport	membrane;mitochondrion		0.093	0.194	2.09
P38746	Obg-like ATPase homolog [OS=Saccharomyces cerevisiae S288c]	856372	YLF2; YHL014C		mitochondrion	nucleotide binding	0.093	0.194	2.09
P38756	tRNA threonylcarbamoyladenosine dehydratase 1 [OS=Saccharomyces cerevisiae S288c]	856392	TCD1; YHR003C; YHR003C	metabolic process	cytosol;membrane;mitochondrion	catalytic activity;nucleotide binding	0.093	0.194	2.09
P40452	Cytochrome c oxidase assembly factor 1 [OS=Saccharomyces cerevisiae S288c]	854649	COA1; YIL157C	cell organization and biogenesis	membrane;mitochondrion	protein binding	0.874	1.848	2.11
Q08742	Thiosulfate sulfurtransferase RDL2, mitochondrial [OS=Saccharomyces cerevisiae S288c]	854460	RDL2; YOR286W		mitochondrion	catalytic activity	0.874	1.848	2.11
Q12393	Genetic interactor of prohibitin 5, mitochondrial [OS=Saccharomyces cerevisiae S288c]	850780	GEP5; YLR091W	cell organization and biogenesis	mitochondrion		0.122	0.259	2.12
P28737	Protein MSP1 [OS=Saccharomyces cerevisiae S288c]	852915	MSP1; YGR028W	cell organization and biogenesis;metabolic process;regulation of biological process;response to stimulus;transport	membrane;mitochondrion;nucleus	catalytic activity;nucleotide binding;protein binding;RNA binding	0.122	0.259	2.12
Q04472	Mitochondrial inner membrane i-AAA protease supercomplex subunit MGR3 [OS=Saccharomyces cerevisiae S288c]	855142	MGR3; YMR115W	metabolic process	membrane;mitochondrion	protein binding	0.15	0.322	2.15
P38816	thioredoxin reductase 2, mitochondrial [OS=Saccharomyces cerevisiae S288c]	856506	TRR2; YHR106W	cellular homeostasis;metabolic process;regulation of biological process;response to	cytoplasm;mitochondrion	antioxidant activity;catalytic activity	0.136	0.292	2.15

				stimulus					
P21375	Fumarate reductase 2 [OS=Saccharomyces cerevisiae S288c]	853510	OSM1; YJR051W	metabolic process	endoplasmic reticulum;mitochondrion	catalytic activity;nucleotide binding	0.16	0.346	2.16
P40502	Altered inheritance of mitochondria protein 19, mitochondrial [OS=Saccharomyces cerevisiae S288c]	854722	AIM19; YIL087C		membrane;mitochondrion		0.995	2.162	2.17
Q01532-1	Cysteine proteinase 1, mitochondrial [OS=Saccharomyces cerevisiae S288c]		MCY1	metabolic process;regulation of biological process;response to stimulus	cytoplasm;mitochondrion	catalytic activity;DNA binding;RNA binding	0.179	0.389	2.17
P36056	37S ribosomal protein S22, mitochondrial [OS=Saccharomyces cerevisiae S288c]	853701	RSM22; YKL155C	cell organization and biogenesis;metabolic process	mitochondrion;ribosome	catalytic activity;structural molecule activity	0.194	0.425	2.19
P41921	glutathione reductase [OS=Saccharomyces cerevisiae S288c]	856014	GLR1; YPL091W	cellular homeostasis;metabolic process;regulation of biological process;response to stimulus	cytoplasm;cytosol;mitochondrion;nucleus	antioxidant activity;catalytic activity;nucleotide binding	0.241	0.54	2.24
P49334	Mitochondrial import receptor subunit tom22 [OS=Saccharomyces cerevisiae S288c]	855592	TOM22; YNL131W	cell organization and biogenesis;transport	membrane;mitochondrion	protein binding;transporter activity	0.292	0.668	2.29
P14063	54S ribosomal protein L31, mitochondrial [OS=Saccharomyces cerevisiae S288c]	853720	MRPL31; YKL138C	cell organization and biogenesis;metabolic process	mitochondrion;ribosome	structural molecule activity	0.292	0.668	2.29
Q03246	37S ribosomal protein S17, mitochondrial [OS=Saccharomyces cerevisiae S288c]	855226	MRPS17; YMR188C	cell differentiation;cell organization and biogenesis;metabolic process	mitochondrion;ribosome	RNA binding;structural molecule activity	0.931	2.162	2.32
P53193	J-type co-chaperone JAC1, mitochondrial [OS=Saccharomyces cerevisiae S288c]	852866	JAC1; YGL018C	cell organization and biogenesis;metabolic process;transport	membrane;mitochondrion;organelle lumen	protein binding	0.468	1.154	2.47
P53157	Mitochondrial pyruvate carrier 1 [OS=Saccharomyces cerevisiae S288c]	852800	FMP37; MPC1; YGL080W	transport	membrane;mitochondrion	transporter activity	0.468	1.154	2.47
P25087	Sterol 24-C-methyltransferase [OS=Saccharomyces cerevisiae S288c]	855003	ERG6; YML008C	metabolic process	endoplasmic reticulum;membrane;mitochondrion	catalytic activity	0.557	1.424	2.56
P35999	Mitochondrial intermediate peptidase [OS=Saccharomyces cerevisiae S288c]	853724	OCT1; YKL134C	cellular homeostasis;metabolic process;regulation of biological process	mitochondrion;organelle lumen	catalytic activity;metal ion binding	0.145	0.372	2.57
P39112	Exoribonuclease II, mitochondrial [OS=Saccharomyces cerevisiae S288c]	855331	DSS1; YMR287C	metabolic process	mitochondrion;organelle lumen	catalytic activity;RNA binding	0.087	0.233	2.68
P42900	Sigma-like sequence protein 1, mitochondrial [OS=Saccharomyces cerevisiae S288c]	850830	SLS1; YLR139C	cell organization and biogenesis;metabolic process	membrane;mitochondrion	protein binding	0.125	0.343	2.74
P38969	Pentamidine resistance factor, mitochondrial [OS=Saccharomyces cerevisiae S288c]	854440	PNT1; YOR266 W	cell organization and biogenesis;response to stimulus	membrane;mitochondrion		0.259	0.711	2.75
P22438	Methionine--tRNA ligase, mitochondrial [OS=Saccharomyces cerevisiae S288c]	853081	MSM1; YGR171C	metabolic process	cytoplasm;cytosol;mitochondrion;organelle lumen	catalytic activity;nucleotide binding	0.136	0.377	2.77
Q06089	Uncharacterized mitochondrial outer membrane protein YPR098C [OS=Saccharomyces cerevisiae S288c]	856213	YPR098C; YPR098C		membrane;mitochondrion		0.778	2.162	2.78
P32493	ATPase expression protein 1, mitochondrial [OS=Saccharomyces cerevisiae S288c]	855086	AEP1; YMR064 W	regulation of biological process	mitochondrion	translation regulator activity	0.15	0.417	2.78
Q12032	Altered inheritance of mitochondria protein 41, mitochondrial [OS=Saccharomyces cerevisiae S288c]	854390	AIM41; YOR215C		mitochondrion	catalytic activity	1.61	4.623	2.87
Q06698	Putative ATP-dependent RNA helicase YLR419W [OS=Saccharomyces cerevisiae S288c]	851137	YLR419W ; YLR419W	metabolic process	cytoplasm;mitochondrion;nucleus	catalytic activity;nucleotide binding;protein binding;RNA binding	0.027	0.082	3.04
P53234	Uncharacterized protein YGR053C [OS=Saccharomyces cerevisiae S288c]	852944	YGR053C; YGR053C				0.129	0.438	3.40
P47039	Probable kynurenine--oxoglutarate transaminase	853386	BNA3; YJL060W	metabolic process;regulation of	cytoplasm;mitochondrion	catalytic activity	0.334	1.154	3.46

	BNA3 [OS=Saccharomyces cerevisiae S288c]			biological process					
P39533	Homocitrate dehydratase, mitochondrial [OS=Saccharomyces cerevisiae S288c]	853230	ACO2; YJL200C	metabolic process	mitochondrion	catalytic activity;metal ion binding	0.17	0.688	4.05
Q02981	ABC1 family protein YPL109C, mitochondrial [OS=Saccharomyces cerevisiae S288c]	855995	YPL109C; YPL109C		mitochondrion		0.059	0.259	4.39
P53318	Ubiquinone biosynthesis monooxygenase COQ6, mitochondrial [OS=Saccharomyces cerevisiae S288c]	853170	COQ6; YGR255C	metabolic process	membrane;mitochondrion	catalytic activity;nucleotide binding	0.077	0.346	4.49
Q08926	ULP1-interacting protein 4 [OS=Saccharomyces cerevisiae S288c]	855916	UIP4; YPL186C		endoplasmic reticulum;membrane;mitochondrion;nucleus		0.136	0.668	4.91
P38825	Protein TOM71 [OS=Saccharomyces cerevisiae S288c]	856517	TOM71; YHR117 W	transport	membrane;mitochondrion	protein binding;transporter activity	0.061	0.343	5.62
P48526	Isoleucine--tRNA ligase, mitochondrial [OS=Saccharomyces cerevisiae S288c]	856067	ISM1; YPL040C	cell organization and biogenesis;metabolic process;regulation of biological process	cytoplasm;cytosol;mitochondrion;organelle lumen	catalytic activity;nucleotide binding	0.081	0.477	5.89

Supplemental Table S2. The relative concentrations of proteins in mitochondria purified from WT cells cultured with or without LCA. Mitochondria were purified from WT cells recovered on day 4 of cell culturing with or without LCA. Mass spectrometry-based identification and quantitation of proteins recovered in purified mitochondria were performed as described in Materials and methods. The exponentially modified protein abundance index (emPAI) provided by the ″Proteome Discoverer″ software was used to calculate the relative abundance of mitochondrial proteins between specified datasets. Data on the relative concentrations of mitochondrial proteins are presented as means of 2 independent experiments.

Accession	Description	Entrez Gene ID	Gene ID	Biological Process	Cellular Component	Molecular Function	emPAI WT	emPAI WT + LCA	Ratio WT + LCA / WT
P00127	Cytochrome b-c1 complex subunit 6	850593	QCR6	metabolic process; transport	membrane; mitochondrion	catalytic activity; transporter activity	1.371	0.334	0.24
Q96VH5	MICOS complex subunit Mic10	850300	MOS1	cell organization and biogenesis	membrane; mitochondrion		6.197	1.683	0.27
P53230	Phosphatidate cytidylyltransferase, mitochondrial [OS=Saccharomyces cerevisiae S288c]	852937	TAM41; YGR046W	metabolic process	membrane;mitochondrion;organelle lumen	catalytic activity	0.668	0.186	0.28
P14693	sorting assembly machinery 35 kDa subunit [OS=Saccharomyces cerevisiae S288c]	856483	SAM35; YHR083W	cell organization and biogenesis;transport	membrane;mitochondrion	protein binding	0.54	0.155	0.29
P32332	Mitochondrial oxaloacetate transport protein [OS=Saccharomyces cerevisiae S288c]	853739	OAC1; YKL120W	metabolic process;transport	membrane;mitochondrion	structural molecule activity;transporter activity	4.995	1.783	0.36
P47131	TMEM14 protein homolog YJR085C [OS=Saccharomyces cerevisiae S288c]	853547	YJR085C; YJR085C		membrane;mitochondrion		2.162	0.778	0.36
P53163	54S ribosomal protein L12, mitochondrial [OS=Saccharomyces cerevisiae S288c]	852811	MNP1; YGL068W	cell organization and biogenesis;metabolic process	mitochondrion;ribosome	RNA binding;structural molecule activity	1.31	0.52	0.40
P47140	Altered inheritance rate of mitochondria protein 25 [OS=Saccharomyces cerevisiae S288c]	853563	AIM25; YJR100C	cell organization and biogenesis	membrane;mitochondrion	transporter activity	0.874	0.369	0.42
P39515	mitochondrial import inner membrane translocase subunit Tim17 [OS=Saccharomyces cerevisiae S288c]	853298	TIM17; YJL143W	cell organization and biogenesis;transport	membrane;mitochondrion	protein binding;transporter activity	0.778	0.334	0.43
P09938	Ribonucleoside-diphosphate reductase small chain 1 [OS=Saccharomyces cerevisiae S288c]	853427	RNR2; YJL026W	metabolic process	cytoplasm;cytosol;nucleus	catalytic activity;metal ion binding;protein binding	0.668	0.292	0.44
P40581	peroxiredoxin HYR1 [OS=Saccharomyces cerevisiae S288c]	854855	HYR1; YIR037W	metabolic process;response to stimulus	cytoplasm;cytosol;mitochondrion;organelle lumen	antioxidant activity;catalytic activity	0.585	0.259	0.44
P38152	Tricarboxylate transport protein [OS=Saccharomyces cerevisiae S288c]	852594	CTP1; YBR291C	metabolic process;transport	membrane;mitochondrion	structural molecule activity;transporter activity	0.551	0.245	0.44
Q08970	Mitochondrial metal transporter 2 [OS=Saccharomyces cerevisiae S288c]	855877	MMT2; YPL224C	cellular homeostasis;regulation of biological process;response to stimulus;transport	membrane;mitochondrion	transporter activity	0.407	0.186	0.46
P25270	rRNA methyltransferase 1, mitochondrial [OS=Saccharomyces cerevisiae S288c]	854376	MRM1; YOR201C	metabolic process	mitochondrion	catalytic activity;RNA binding	0.274	0.129	0.47
P29704	squalene synthase [OS=Saccharomyces cerevisiae S288c]	856597	ERG9; YHR190W	metabolic process	endoplasmic reticulum;membrane;mitochondrion	catalytic activity	0.222	0.105	0.47
Q06005	Octanoyltransferase, mitochondrial [OS=Saccharomyces cerevisiae S288c]	850940	LIP2; YLR239C	metabolic process	cytoplasm;mitochondrion	catalytic activity	0.245	0.116	0.47
P38169	Kynurenine 3-monooxygenase [OS=Saccharomyces cerevisiae S288c]	852179	BNA4; YBL098W	metabolic process	membrane;mitochondrion	catalytic activity;nucleotide binding	0.172	0.083	0.48
P81450	ATP synthase subunit J, mitochondrial [OS=Saccharomyces cerevisiae S288c]	854893	ATP18; YML081C-A	cell organization and biogenesis;metabolic process;transport	membrane;mitochondrion	catalytic activity;transporter activity	9	4.623	0.51
P40165	NAD(P)H-hydrate epimerase [OS=Saccharomyces cerevisiae S288c]	855521	YNL200C; YNL200C	metabolic process	cytoplasm;mitochondrion	catalytic activity;metal ion binding;nucleotide binding	2.162	1.154	0.53
P01097	ATPase inhibitor, mitochondrial [OS=Saccharomyces cerevisiae S288c]	851347	INH1; YDL181W	metabolic process;regulation of biological process	mitochondrion	catalytic activity;enzyme regulator activity;protein binding	2.162	1.154	0.53

P39525	3-oxoacyl-[acyl-carrier-protein] synthase homolog [OS=Saccharomyces cerevisiae S288c]	856790	CEM1; YER061C	metabolic process	mitochondrion	catalytic activity	2.899	1.565	0.54
P54003	Protein SUR7 [OS=Saccharomyces cerevisiae S288c]	854953	SUR7; YML052W	cell differentiation;transport	membrane;mitochondrion		1.512	0.848	0.56
Q12359	Ammonia transport outward protein 3 [OS=Saccharomyces cerevisiae S288c]	851992	ATO3; YDR384C	transport	membrane;mitochondrion	transporter activity	0.995	0.585	0.59
P25642	54S ribosomal protein IMG2, mitochondrial [OS=Saccharomyces cerevisiae S288c]	850434	IMG2; YCR071C	cell organization and biogenesis;metabolic process	mitochondrion;ribosome	structural molecule activity	0.778	0.468	0.60
Q02888	Inner membrane assembly complex subunit 17 [OS=Saccharomyces cerevisiae S288c]	856005	AIM43; INA17; YPL099C	cell organization and biogenesis	membrane;mitochondrion	protein binding	0.701	0.425	0.61
P32799	Cytochrome c oxidase subunit 6A, mitochondrial [OS=Saccharomyces cerevisiae S288c]	852684	COX13; YGL191W	cell organization and biogenesis;metabolic process;regulation of biological process;transport	membrane;mitochondrion	catalytic activity;enzyme regulator activity;transporter activity	18.953	11.589	0.61
P53212	Probable transcriptional regulatory protein HAH1 [OS=Saccharomyces cerevisiae S288c]	852904	YGR021W; YGR021W		mitochondrion		0.585	0.359	0.61
Q06567	ABC1 family protein MCP2 [OS=Saccharomyces cerevisiae S288c]	850955	YLR253W; MCP2; YLR253W	cell organization and biogenesis	membrane;mitochondrion		0.484	0.301	0.62
P53234	Uncharacterized protein YGR053C [OS=Saccharomyces cerevisiae S288c]	852944	YGR053C; YGR053C				0.438	0.274	0.63
Q03028	Mitochondrial 2-oxodicarboxylate carrier 1 [OS=Saccharomyces cerevisiae S288c]	855969	ODC1; YPL134C	metabolic process;transport	membrane;mitochondrion	structural molecule activity;transporter activity	5.813	3.642	0.63
Q3E824	Uncharacterized protein YOR020W-A [OS=Saccharomyces cerevisiae S288c]	1466480	YOR020W-A; YOR020W-A	metabolic process;transport	membrane;mitochondrion		5.813	3.642	0.63
P00560	Phosphoglycerate kinase [OS=Saccharomyces cerevisiae S288c]	850370	PGK1; YCR012W	metabolic process	cytoplasm;membrane;mitochondrion	catalytic activity;nucleotide binding	6.356	4.012	0.63
P33310	ATP-dependent permease MDL1, mitochondrial [OS=Saccharomyces cerevisiae S288c]	850885	MDL1; YLR188W	transport	membrane;mitochondrion	catalytic activity;nucleotide binding;transporter activity	0.833	0.528	0.63
P15992	heat shock protein 26 [OS=Saccharomyces cerevisiae S288c]	852364	HSP26; YBR072W	metabolic process;response to stimulus	cytoplasm;mitochondrion;nucleus	protein binding;RNA binding	4.878	3.125	0.64
P07253	cytochrome B pre-mRNA-processing protein 6 [OS=Saccharomyces cerevisiae S288c]	852417	CBP6; YBR120C	cell organization and biogenesis;metabolic process;regulation of biological process	mitochondrion;ribosome		4.179	2.728	0.65
P39006	Phosphatidylserine decarboxylase proenzyme 1, mitochondrial [OS=Saccharomyces cerevisiae S288c]	855552	PSD1; YNL169C	metabolic process;regulation of biological process	membrane;mitochondrion	catalytic activity	0.743	0.487	0.66
P00045	Cytochrome c iso-2 [OS=Saccharomyces cerevisiae S288c]	856672	CYC7; YEL039C	metabolic process;transport	mitochondrion	metal ion binding	1.512	0.995	0.66
Q06143	mitochondrial dicarboxylate transporter [OS=Saccharomyces cerevisiae S288c]	851063	DIC1; YLR348C	metabolic process;transport	membrane;mitochondrion	structural molecule activity;transporter activity	1.404	0.931	0.66
P14832	peptidyl-prolyl cis-trans isomerase [OS=Saccharomyces cerevisiae S288c]	851733	CPR1; YDR155C	cell differentiation;cell organization and biogenesis;metabolic process;regulation of biological process;transport	cytoplasm;mitochondrion;nucleus	catalytic activity;RNA binding	1.31	0.874	0.67
P42949	Mitochondrial import inner membrane translocase subunit TIM16 [OS=Saccharomyces cerevisiae S288c]	853340	PAM16; YJL104W	transport	membrane;mitochondrion	protein binding	1.31	0.874	0.67
Q12298	uncharacterized ABC transporter ATP-binding protein YDR061W [OS=Saccharomyces cerevisiae S288c]	851633	YDR061W; YDR061W		mitochondrion	catalytic activity;nucleotide binding	0.512	0.343	0.67
Q03799	37S ribosomal protein S8, mitochondrial [OS=Saccharomyces cerevisiae S288c]	855190	MRPS8; YMR158W	cell organization and biogenesis;metabolic process	mitochondrion;ribosome	structural molecule activity	1.031	0.701	0.68
Q12428	Probable 2-methylcitrate dehydratase [OS=Saccharomyces cerevisiae S288c]	856108	PDH1; YPR002W	metabolic process	cytoplasm;membrane;mitochondrion	catalytic activity	18.11	12.335	0.68
P40086	Cytochrome c oxidase assembly protein cox15 [OS=Saccharomyces cerevisiae S288c]	856884	COX15; YER141W	cell organization and biogenesis;metabolic process;transport	membrane;mitochondrion	catalytic activity;transporter activity	2.594	1.783	0.69

P00445	Superoxide dismutase [Cu-Zn] [OS=Saccharomyces cerevisiae S288c]	853568	SOD1; YJR104C	cell organization and biogenesis;cellular homeostasis;metabolic process;regulation of biological process;response to stimulus	cytoplasm;cytosol;mitochondrion;nucleus	antioxidant activity;catalytic activity;metal ion binding;protein binding	1.848	1.31	0.71
P17695	Glutaredoxin-2, mitochondrial [OS=Saccharomyces cerevisiae S288c]	852124	GRX2; YDR513W	cellular homeostasis;metabolic process;regulation of biological process;response to stimulus;transport	cytoplasm;cytosol;mitochondrion;nucleus	antioxidant activity;catalytic activity	1.848	1.31	0.71
Q04689	Altered inheritance of mitochondria protein 32 [OS=Saccharomyces cerevisiae S288c]	854955	AIM32; YML050W				0.52	0.369	0.71
P00360	glyceraldehyde-3-phosphate dehydrogenase 1 [OS=Saccharomyces cerevisiae S288c]	853395	TDH1; YJL052W	metabolic process	cytoplasm;cytosol;membrane;mitochondrion	catalytic activity;nucleotide binding	1.721	1.228	0.71
P53311	Mitochondrial pyruvate carrier 3 [OS=Saccharomyces cerevisiae S288c]	853158	FMP43; MPC3; YGR243W	transport	membrane;mitochondrion	transporter activity	1.61	1.154	0.72
P53326	Uncharacterized protein YGR266W [OS=Saccharomyces cerevisiae S288c]	853182	YGR266W; YGR266W		membrane;mitochondrion		1.081	0.778	0.72
P19657	plasma membrane ATPase 2 [OS=Saccharomyces cerevisiae S288c]	856071	PMA2; YPL036W	cellular homeostasis;metabolic process;transport	membrane;mitochondrion	catalytic activity;metal ion binding;nucleotide binding;transporter activity	10.938	7.886	0.72
P48360	Probable NADPH:adrenodoxin oxidoreductase, mitochondrial [OS=Saccharomyces cerevisiae S288c]	851982	ARH1; YDR376W	cellular homeostasis;metabolic process;transport	cytoplasm;membrane;mitochondrion;organelle lumen	catalytic activity	0.346	0.25	0.72
P25087	Sterol 24-C-methyltransferase [OS=Saccharomyces cerevisiae S288c]	855003	ERG6; YML008C	metabolic process	endoplasmic reticulum;membrane;mitochondrion	catalytic activity	1.424	1.031	0.72
P39722	Mitochondrial Rho GTPase 1 [OS=Saccharomyces cerevisiae S288c]	851249	GEM1; YAL048C	cell organization and biogenesis;regulation of biological process;response to stimulus;transport	membrane;mitochondrion	catalytic activity;metal ion binding;nucleotide binding	0.259	0.189	0.73
P38885	Altered inheritance of mitochondria protein 46, mitochondrial [OS=Saccharomyces cerevisiae S288c]	856606	AIM46; YHR199C		mitochondrion	catalytic activity	2.728	1.994	0.73
P39518	Long-chain-fatty-acid--CoA ligase 2 [OS=Saccharomyces cerevisiae S288c]	856734	FAA2; YER015W	metabolic process;transport	cytoplasm;mitochondrion	catalytic activity;nucleotide binding	0.619	0.455	0.74
Q99297	Mitochondrial 2-oxodicarboxylate carrier 2 [OS=Saccharomyces cerevisiae S288c]	854397	ODC2; YOR222W	metabolic process;transport	membrane;mitochondrion	structural molecule activity;transporter activity	1.054	0.778	0.74
P28817	3-hydroxyisobutyryl-CoA hydrolase, mitochondrial [OS=Saccharomyces cerevisiae S288c]	851606	EHD3; YDR036C	metabolic process	mitochondrion	catalytic activity	1.395	1.043	0.75
P33759	37S ribosomal protein S5, mitochondrial [OS=Saccharomyces cerevisiae S288c]	852553	MRPS5; YBR251W	cell organization and biogenesis;metabolic process	mitochondrion;ribosome	RNA binding;structural molecule activity	0.778	0.585	0.75
P40012	protoporphyrinogen oxidase [OS=Saccharomyces cerevisiae S288c]	856733	HEM14; YER014W	metabolic process	membrane;mitochondrion	catalytic activity	2.162	1.637	0.76
P50087	MICOS subunit MIC26 [OS=Saccharomyces cerevisiae S288c]	853150	MOS2; MIC26; YGR235C	cell organization and biogenesis	membrane;mitochondrion		1.683	1.276	0.76
Q02883	N-acyl-phosphatidylethanolamine-hydrolyzing phospholipase D, mitochondrial [OS=Saccharomyces cerevisiae S288c]	856001	FMP30; YPL103C	metabolic process	membrane;mitochondrion	catalytic activity;metal ion binding	0.557	0.425	0.76
Q12032	Altered inheritance of mitochondria protein 41, mitochondrial [OS=Saccharomyces cerevisiae S288c]	854390	AIM41; YOR215C		mitochondrion	catalytic activity	2.831	2.162	0.76
P23641	mitochondrial phosphate carrier protein [OS=Saccharomyces cerevisiae S288c]	853540	MIR1; YJR077C	metabolic process;transport	membrane;mitochondrion	protein binding;structural molecule activity;transporter activity	30.623	23.484	0.77
P40485	Phosphatidylinositol 4,5-bisphosphate-binding protein SLM1 [OS=Saccharomyces cerevisiae S288c]	854701	SLM1; YIL105C	cell organization and biogenesis;regulation of biological process;response to stimulus;transport	cytoplasm;membrane;mitochondrion		1.371	1.054	0.77
Q07500	External NADH-ubiquinone oxidoreductase 2, mitochondrial [OS=Saccharomyces cerevisiae S288c]	851474	NDE2; YDL085W	metabolic process	mitochondrion	catalytic activity;nucleotide binding	13.678	10.659	0.78
P43617	Uncharacterized mitochondrial carrier YFR045W [OS=Saccharomyces cerevisiae S288c]	850606	YFR045W; YFR045W	metabolic process;transport	membrane;mitochondrion	structural molecule activity;transporter activity	0.995	0.778	0.78

P46681	D-lactate dehydrogenase [cytochrome] 2, mitochondrial [OS=Saccharomyces cerevisiae S288c]	851376	DLD2; YDL178W	metabolic process	mitochondrion;organelle lumen	catalytic activity;nucleotide binding;protein binding	1.829	1.438	0.79
P00410	Cytochrome c oxidase subunit 2 [OS=Saccharomyces cerevisiae S288c]	854622	COX2; Q0250	metabolic process;transport	membrane;mitochondrion	catalytic activity;metal ion binding;protein binding;transporter activity	4.623	3.642	0.79
P36101	tRNA threonylcarbamoyladenosine dehydratase 2 [OS=Saccharomyces cerevisiae S288c]	853841	TCD2; YKL027W; YKL027W	metabolic process	cytosol;membrane;mitochondrion	catalytic activity;nucleotide binding	0.778	0.616	0.79
P32387	54S ribosomal protein L41, mitochondrial [OS=Saccharomyces cerevisiae S288c]	852014	MRP20; YDR405W	cell organization and biogenesis;metabolic process	mitochondrion;ribosome	nucleotide binding;protein binding;RNA binding;structural molecule activity	1.581	1.254	0.79
P27697	Atypical kinase COQ8, mitochondrial [OS=Saccharomyces cerevisiae S288c]	852758	COQ8; YGL119W	metabolic process	membrane;mitochondrion;organelle lumen	catalytic activity;nucleotide binding	0.453	0.365	0.81
P18239	ADP,ATP carrier protein 2 [OS=Saccharomyces cerevisiae S288c]	852250	PET9; YBL030C	cell death;metabolic process;transport	membrane;mitochondrion	structural molecule activity;transporter activity	110.034	89.063	0.81
P40035	Mitochondrial phosphate carrier protein 2 [OS=Saccharomyces cerevisiae S288c]	856779	PIC2; YER053C	cellular homeostasis;metabolic process;transport	membrane;mitochondrion	structural molecule activity;transporter activity	1.637	1.336	0.82
P38127	mitochondrial carrier protein RIM2 [OS=Saccharomyces cerevisiae S288c]	852491	RIM2; YBR192W	cell organization and biogenesis;metabolic process;transport	membrane;mitochondrion	structural molecule activity;transporter activity	1.512	1.239	0.82
P38120	37S ribosomal protein S9, mitochondrial [OS=Saccharomyces cerevisiae S288c]	852443	MRPS9; YBR146W	cell organization and biogenesis;metabolic process	mitochondrion;ribosome	RNA binding;structural molecule activity	2.384	1.955	0.82
P53969	Sorting assembly machinery 50 kDa subunit [OS=Saccharomyces cerevisiae S288c]	855705	SAM50; YNL026W	cell organization and biogenesis;transport	membrane;mitochondrion	protein binding;transporter activity	0.743	0.61	0.82
Q12289	mitochondrial carnitine carrier [OS=Saccharomyces cerevisiae S288c]	854267	CRC1; YOR100C	metabolic process;transport	membrane;mitochondrion	structural molecule activity;transporter activity	1.404	1.154	0.82
Q12482	Mitochondrial aspartate-glutamate transporter AGC1 [OS=Saccharomyces cerevisiae S288c]	856132	AGC1; YPR021C	metabolic process;transport	membrane;mitochondrion	structural molecule activity;transporter activity	0.348	0.292	0.84
Q06668	Methyltransferase OMS1, mitochondrial [OS=Saccharomyces cerevisiae S288c]	851911	OMS1; YDR316W	metabolic process	membrane;mitochondrion	catalytic activity	0.693	0.585	0.84
Q04013	Citrate/oxoglutarate carrier protein [OS=Saccharomyces cerevisiae S288c]	855282	YHM2; YMR241W	cell organization and biogenesis;metabolic process;transport	membrane;mitochondrion	DNA binding;structural molecule activity;transporter activity	3.467	2.981	0.86
P00925	Enolase 2 [OS=Saccharomyces cerevisiae S288c]	856579	ENO2; YHR174W	metabolic process;regulation of biological process	cytoplasm;cytosol;membrane;mitochondrion;vacuole	catalytic activity;metal ion binding;protein binding	1.212	1.043	0.86
P36066	Protein MRG3-like [OS=Saccharomyces cerevisiae S288c]	853725	YKL133C; YKL133C	metabolic process	membrane;mitochondrion	protein binding	1.054	0.911	0.86
P40098	uncharacterized mitochondrial membrane protein FMP10 [OS=Saccharomyces cerevisiae S288c]	856931	FMP10; YER182W		membrane;mitochondrion		5.952	5.158	0.87
P32266	Dynamin-like GTPase MGM1, mitochondrial [OS=Saccharomyces cerevisiae S288c]	854386	MGM1; YOR211C	cell organization and biogenesis	membrane;mitochondrion	catalytic activity;nucleotide binding;protein binding	0.448	0.389	0.87
Q08179	Mitochondrial distribution and morphology protein 38 [OS=Saccharomyces cerevisiae S288c]	854130	MDM38; YOL027C	cell organization and biogenesis;cellular homeostasis;regulation of biological process;transport	membrane;mitochondrion		6.079	5.31	0.87
P27929	37S ribosomal protein NAM9, mitochondrial [OS=Saccharomyces cerevisiae S288c]	855585	NAM9; YNL137C	cell organization and biogenesis;metabolic process;regulation of biological process	mitochondrion;ribosome	RNA binding;structural molecule activity	1.062	0.931	0.88
P25374	Cysteine desulfurase, mitochondrial [OS=Saccharomyces cerevisiae S288c]	850343	NFS1; YCL017C	cell organization and biogenesis;cellular homeostasis;metabolic process	mitochondrion;nucleus	catalytic activity;metal ion binding;protein binding	2.594	2.3	0.89
P32335	Protein MSS51, mitochondrial [OS=Saccharomyces cerevisiae S288c]	850900	MSS51; YLR203C	metabolic process;regulation of biological process	membrane;mitochondrion	protein binding;translation regulator activity	1.929	1.712	0.89
P00359	Glyceraldehyde-3-phosphate dehydrogenase 3 [OS=Saccharomyces cerevisiae	853106	TDH3; YGR192C	cell death;metabolic process	cytoplasm;cytosol;membrane;mitochondrion;nucleus	catalytic activity;nucleotide binding;RNA	7.185	6.406	0.89

	S288c]					binding			
P32191	Glycerol-3-phosphate dehydrogenase, mitochondrial [OS=Saccharomyces cerevisiae S288c]	854651	GUT2; YIL155C	metabolic process	membrane;mitochondrion	catalytic activity	8.522	7.633	0.90
P33311	ATP-dependent permease MDL2, mitochondrial [OS=Saccharomyces cerevisiae S288c]	855858	MDL2; YPL270W	response to stimulus;transport	membrane;mitochondrion	catalytic activity;nucleotide binding;transporter activity	1.116	1.006	0.90
Q08818	Meiotic sister-chromatid recombination protein 6, mitochondrial [OS=Saccharomyces cerevisiae S288c]	854536	MSC6; YOR354C	metabolic process	mitochondrion;organelle lumen	RNA binding	1.047	0.945	0.90
P40047	Aldehyde dehydrogenase 5, mitochondrial [OS=Saccharomyces cerevisiae S288c]	856804	ALD5; YER073W	metabolic process	mitochondrion;organelle lumen	catalytic activity	2.384	2.162	0.91
P32843	Mitochondrial escape protein 2 [OS=Saccharomyces cerevisiae S288c]	855348	YME2; YMR302C	cell organization and biogenesis;metabolic process	membrane;mitochondrion	nucleotide binding;RNA binding	5.158	4.68	0.91
P16547	Mitochondrial outer membrane protein OM45 [OS=Saccharomyces cerevisiae S288c]	854670	OM45; YIL136W		membrane;mitochondrion		250.189	228.087	0.91
P07256	Cytochrome b-c1 complex subunit 1, mitochondrial [OS=Saccharomyces cerevisiae S288c]	852235	COR1; YBL045C	metabolic process;transport	membrane;mitochondrion	catalytic activity;metal ion binding;protein binding;transporter activity	18.783	17.166	0.91
P53312	Succinyl-CoA ligase [ADP-forming] subunit beta, mitochondrial [OS=Saccharomyces cerevisiae S288c]	853159	LSC2; YGR244C	metabolic process	mitochondrion	catalytic activity;nucleotide binding	20.017	18.513	0.92
P07251	ATP synthase subunit alpha, mitochondrial [OS=Saccharomyces cerevisiae S288c]	852177	ATP1; YBL099W	metabolic process;transport	cytosol;membrane;mitochondrion	catalytic activity;nucleotide binding;transporter activity	34.938	32.711	0.94
P04840	Mitochondrial outer membrane protein porin 1 [OS=Saccharomyces cerevisiae S288c]	855669	POR1; YNL055C	cell death;cell organization and biogenesis;cellular homeostasis;regulation of biological process;transport	cytoplasm;membrane;mitochondrion	transporter activity	15.238	15.238	1.00
P53252	sphingolipid long chain base-responsive protein PIL1 [OS=Saccharomyces cerevisiae S288c]	852977	PIL1; YGR086C	cell organization and biogenesis;regulation of biological process;response to stimulus;transport	cytoplasm;membrane;mitochondrion	protein binding	16.191	16.191	1.00
P40215	External NADH-ubiquinone oxidoreductase 1, mitochondrial [OS=Saccharomyces cerevisiae S288c]	855176	NDE1; YMR145C	metabolic process	mitochondrion	catalytic activity;nucleotide binding	5.469	5.469	1.00
P07213	Mitochondrial import receptor subunit TOM70 [OS=Saccharomyces cerevisiae S288c]	855602	TOM70; YNL121C	cell organization and biogenesis;transport	membrane;mitochondrion	protein binding;transporter activity	3.642	3.642	1.00
P07246	Alcohol dehydrogenase 3, mitochondrial [OS=Saccharomyces cerevisiae S288c]	855107	ADH3; YMR083W	metabolic process	mitochondrion;organelle lumen	catalytic activity;metal ion binding	10.159	10.159	1.00
P43635	Citrate synthase 3, mitochondrial [OS=Saccharomyces cerevisiae S288c]	856107	CIT3; YPR001W	metabolic process	mitochondrion	catalytic activity	5.529	5.529	1.00
P32473	Pyruvate dehydrogenase E1 component subunit beta, mitochondrial [OS=Saccharomyces cerevisiae S288c]	852522	PDB1; YBR221C	metabolic process	mitochondrion;organelle lumen	catalytic activity	9	9	1.00
P12695	Dihydrolipoyllysine-residue acetyltransferase component of pyruvate dehydrogenase complex, mitochondrial [OS=Saccharomyces cerevisiae S288c]	855653	LAT1; YNL071W	metabolic process	mitochondrion;organelle lumen	catalytic activity	6.43	6.43	1.00
P49367	Homoaconitase, mitochondrial [OS=Saccharomyces cerevisiae S288c]	851820	LYS4; YDR234W	metabolic process	mitochondrion	catalytic activity;metal ion binding;protein binding	1.54	1.54	1.00
P36013	NAD-dependent malic enzyme, mitochondrial [OS=Saccharomyces cerevisiae S288c]	853839	MAE1; YKL029C	metabolic process	mitochondrion;organelle lumen	catalytic activity;metal ion binding;nucleotide binding	1.985	1.985	1.00
P07143	Cytochrome c1, heme protein, mitochondrial [OS=Saccharomyces cerevisiae S288c]	854231	CYT1; YOR065W	metabolic process;transport	cytosol;membrane;mitochondrion	metal ion binding	23.245	23.245	1.00
P16622	Ferrochelatase, mitochondrial [OS=Saccharomyces cerevisiae S288c]	854347	HEM15; YOR176W	metabolic process	membrane;mitochondrion	catalytic activity	6.305	6.305	1.00
P43567	alanine--glyoxylate aminotransferase 1 [OS=Saccharomyces cerevisiae S288c]	850514	AGX1; YFL030W	metabolic process	cytosol;mitochondrion	catalytic activity	2.162	2.162	1.00
Q03104	Meiotic sister chromatid recombination protein 1 [OS=Saccharomyces cerevisiae S288c]	854911	MSC1; YML128C	metabolic process	endoplasmic reticulum;membrane;mitochondrion		2.415	2.415	1.00
P39925	Mitochondrial respiratory chain complexes assembly protein AFG3	856737	AFG3; YER017	cell organization and biogenesis;metabolic	membrane;mitochondrion	catalytic activity;metal ion	1.342	1.342	1.00

	[OS=Saccharomyces cerevisiae S288c]		C	process;response to stimulus;transport		binding;nucleotide binding;protein binding			
P05626	ATP synthase subunit 4, mitochondrial [OS=Saccharomyces cerevisiae S288c]	856027	ATP4; YPL078C	cell organization and biogenesis;metabolic process;transport	membrane;mitochondrion	catalytic activity;transporter activity	4.995	4.995	1.00
P53982	isocitrate dehydrogenase [NADP] [OS=Saccharomyces cerevisiae S288c]	855723	IDP3; YNL009W	metabolic process	cytoplasm;mitochondrion	catalytic activity;metal ion binding;nucleotide binding	2.652	2.652	1.00
Q07651	SUR7 family protein FMP45 [OS=Saccharomyces cerevisiae S288c]	851304	FMP45; YDL222C	cell differentiation;cell organization and biogenesis	membrane;mitochondrion		4.878	4.878	1.00
P25039	Elongation factor G, mitochondrial [OS=Saccharomyces cerevisiae S288c]	850758	MEF1; YLR069C	cell organization and biogenesis;metabolic process	cytosol;mitochondrion	catalytic activity;nucleotide binding;RNA binding	0.931	0.931	1.00
P10662	37S ribosomal protein MRP1, mitochondrial [OS=Saccharomyces cerevisiae S288c]	851948	MRP1; YDR347W	cell organization and biogenesis;metabolic process;response to stimulus	mitochondrion;ribosome	antioxidant activity;catalytic activity;metal ion binding;structural molecule activity	2.981	2.981	1.00
P42940	Probable electron transfer flavoprotein subunit beta [OS=Saccharomyces cerevisiae S288c]	853121	CIR1; YGR207C	metabolic process;transport	mitochondrion;organelle lumen		4.623	4.623	1.00
P32317	Protein AFG1 [OS=Saccharomyces cerevisiae S288c]	856658	AFG1; YEL052W	cell organization and biogenesis;metabolic process;response to stimulus;transport	membrane;mitochondrion	catalytic activity;nucleotide binding	1.326	1.326	1.00
Q04438	Stationary phase protein 4 [OS=Saccharomyces cerevisiae S288c]	855134	SPG4; YMR107W				15.681	15.681	1.00
P32787	Mitochondrial genome maintenance protein MGM101 [OS=Saccharomyces cerevisiae S288c]	853609	MGM101; YJR144W	cell organization and biogenesis;metabolic process;response to stimulus	chromosome;mitochondrion	DNA binding	3.281	3.281	1.00
Q07349	MIOREX complex component 9 [OS=Saccharomyces cerevisiae S288c]	851535	YDL027C; MRX9; YDL027C		endoplasmic reticulum;membrane;mitochondrion		1.913	1.913	1.00
P53266	Cytochrome oxidase assembly protein SHY1 [OS=Saccharomyces cerevisiae S288c]	853009	SHY1; YGR112W	cell organization and biogenesis	membrane;mitochondrion	protein binding	1.154	1.154	1.00
P81449	ATP synthase subunit e, mitochondrial [OS=Saccharomyces cerevisiae S288c]	851922	TIM11; YDR322C-A	cell organization and biogenesis;metabolic process;transport	membrane;mitochondrion	catalytic activity;structural molecule activity;transporter activity	9	9	1.00
P10834	protein PET54 [OS=Saccharomyces cerevisiae S288c]	853137	PET54; YGR222W	metabolic process;regulation of biological process	membrane;mitochondrion;organelle lumen	nucleotide binding;RNA binding;translation regulator activity	1.565	1.565	1.00
P43594	MICOS complex subunit MIC19 [OS=Saccharomyces cerevisiae S288c]	850563	AIM13; MIC19; YFR011C	cell organization and biogenesis	cytoplasm;membrane;mitochondrion		4.337	4.337	1.00
P23644	Mitochondrial import receptor subunit TOM40 [OS=Saccharomyces cerevisiae S288c]	855243	TOM40; YMR203W	transport	cytosol;membrane;mitochondrion	protein binding;transporter activity	1.154	1.154	1.00
P36141	Putative redox protein fmp46, mitochondrial [OS=Saccharomyces cerevisiae S288c]	853923	FMP46; YKR049C	metabolic process	mitochondrion	catalytic activity;protein binding	4.337	4.337	1.00
P53206	putative cysteine synthase [OS=Saccharomyces cerevisiae S288c]	852895	YGR012W; YGR012W	metabolic process		catalytic activity	0.817	0.817	1.00
P08466	mitochondrial nuclease [OS=Saccharomyces cerevisiae S288c]	853222	NUC1; YJL208C	cell death;metabolic process	membrane;mitochondrion;nucleus	catalytic activity;metal ion binding	1.637	1.637	1.00
Q12165	ATP synthase subunit delta, mitochondrial [OS=Saccharomyces cerevisiae S288c]	851560	ATP16; YDL004W	metabolic process;transport	membrane;mitochondrion	catalytic activity;transporter activity	4.623	4.623	1.00
P21560	Protein CBP3, mitochondrial [OS=Saccharomyces cerevisiae S288c]	855886	CBP3; YPL215W	cell organization and biogenesis;regulation of biological process	membrane;mitochondrion;ribosome		1.424	1.424	1.00
P04806	Hexokinase-1 [OS=Saccharomyces cerevisiae S288c]	850614	HXK1; YFR053C	cellular homeostasis;metabolic process;transport	cytoplasm;cytosol;mitochondrion	catalytic activity;nucleotide binding	1.043	1.043	1.00
Q03798	Altered inheritance of mitochondria protein 36, mitochondrial [OS=Saccharomyces cerevisiae S288c]	855189	AIM36; YMR157C		membrane;mitochondrion		1.637	1.637	1.00
P12686	37S ribosomal protein MRP13, mitochondrial [OS=Saccharomyces cerevisiae S288c]	852975	MRP13; YGR084C	cell organization and biogenesis;metabolic process	mitochondrion;ribosome	structural molecule activity	1.239	1.239	1.00
P11325	Leucine--tRNA ligase, mitochondrial [OS=Saccharomyces cerevisiae	851098	NAM2; YLR382	cell organization and biogenesis;metabolic	cytosol;mitochondrion;organelle lumen	catalytic activity;nucleotide	0.353	0.353	1.00

	S288c]		C	process;regulation of biological process		binding;RNA binding			
Q04935	Cytochrome c oxidase protein 20, mitochondrial [OS=Saccharomyces cerevisiae S288c]	851817	COX20; YDR231C	cell organization and biogenesis;metabolic process	membrane;mitochondrion	protein binding	2.455	2.455	1.00
P38825	Protein TOM71 [OS=Saccharomyces cerevisiae S288c]	856517	TOM71; YHR117W	transport	membrane;mitochondrion	protein binding;transporter activity	0.512	0.512	1.00
P37267	Assembly factor cbp4 [OS=Saccharomyces cerevisiae S288c]		CBP4	cell organization and biogenesis	membrane;mitochondrion		7.111	7.111	1.00
Q06678	54S ribosomal protein L35, mitochondrial [OS=Saccharomyces cerevisiae S288c]	851921	MRPL35; YDR322W	cell organization and biogenesis;metabolic process	mitochondrion;ribosome	structural molecule activity	1.031	1.031	1.00
P40508	Uncharacterized protein YIL077C [OS=Saccharomyces cerevisiae S288c]	854733	YIL077C; YIL077C		mitochondrion		0.896	0.896	1.00
P38072	Protein SCO2, mitochondrial [OS=Saccharomyces cerevisiae S288c]	852312	SCO2; YBR024W	cell organization and biogenesis;cellular homeostasis;metabolic process;transport	membrane;mitochondrion	antioxidant activity;catalytic activity;metal ion binding	1.154	1.154	1.00
P48569	Uncharacterized protein YDL183C [OS=Saccharomyces cerevisiae S288c]	851345	YDL183C; YDL183C	transport			1.069	1.069	1.00
P00950	Phosphoglycerate mutase 1 [OS=Saccharomyces cerevisiae S288c]	853705	GPM1; YKL152C	metabolic process;regulation of biological process	cytoplasm;cytosol;membrane;mitochondrion	catalytic activity	1.929	1.929	1.00
Q04472	Mitochondrial inner membrane i-AAA protease supercomplex subunit MGR3 [OS=Saccharomyces cerevisiae S288c]	855142	MGR3; YMR115W	metabolic process	membrane;mitochondrion	protein binding	0.52	0.52	1.00
Q12349	ATP synthase subunit H, mitochondrial [OS=Saccharomyces cerevisiae S288c]	851002	ATP14; YLR295C	cell organization and biogenesis;metabolic process;transport	membrane;mitochondrion	catalytic activity;transporter activity	2.728	2.728	1.00
P18496	Mitochondrial ATPase complex subunit ATP10 [OS=Saccharomyces cerevisiae S288c]	851109	ATP10; YLR393W	cell organization and biogenesis	cytoplasm;membrane;mitochondrion	protein binding	1.239	1.239	1.00
Q04172	Sensitive to high expression protein 9, mitochondrial [OS=Saccharomyces cerevisiae S288c]	852002	SHE9; YDR393W	cell organization and biogenesis	membrane;mitochondrion		0.374	0.374	1.00
P40416	Iron-sulfur clusters transporter ATM1, mitochondrial [OS=Saccharomyces cerevisiae S288c]	855347	ATM1; YMR301C	cellular homeostasis;metabolic process;transport	membrane;mitochondrion	catalytic activity;nucleotide binding;transporter activity	0.453	0.453	1.00
P40502	Altered inheritance of mitochondria protein 19, mitochondrial [OS=Saccharomyces cerevisiae S288c]	854722	AIM19; YIL087C		membrane;mitochondrion		1.512	1.512	1.00
P36516	54S ribosomal protein L3, mitochondrial [OS=Saccharomyces cerevisiae S288c]	855039	MRPL3; YMR024W	cell organization and biogenesis;metabolic process;response to stimulus	mitochondrion;nucleus;ribosome	catalytic activity;RNA binding;structural molecule activity	0.743	0.743	1.00
P38771	Ribosome-recycling factor, mitochondrial [OS=Saccharomyces cerevisiae S288c]	856433	RRF1; YHR038W	cell organization and biogenesis;metabolic process	mitochondrion	RNA binding	1.254	1.254	1.00
P32453	Protein ATP11, mitochondrial [OS=Saccharomyces cerevisiae S288c]	855401	ATP11; YNL315C	cell organization and biogenesis	mitochondrion	protein binding	1.239	1.239	1.00
Q02981	ABC1 family protein YPL109C, mitochondrial [OS=Saccharomyces cerevisiae S288c]	855995	YPL109C; YPL109C		mitochondrion		0.334	0.334	1.00
P39002	long-chain-fatty-acid--CoA ligase 3 [OS=Saccharomyces cerevisiae S288c]	854808	FAA3; YIL009W	metabolic process		catalytic activity;nucleotide binding	0.334	0.334	1.00
P25372	Thioredoxin-3, mitochondrial [OS=Saccharomyces cerevisiae S288c]	850444	TRX3; YCR083W	cellular homeostasis;metabolic process;regulation of biological process;response to stimulus;transport	cytoplasm;mitochondrion	catalytic activity	2.511	2.511	1.00
P38797	Protein phosphatase 2C homolog 7, mitochondrial [OS=Saccharomyces cerevisiae S288c]	856475	PTC7; YHR076W	metabolic process	mitochondrion	catalytic activity;metal ion binding;protein binding	0.688	0.688	1.00
P40159	Uncharacterized protein YNL208W [OS=Saccharomyces cerevisiae S288c]	855513	YNL208W; YNL208W		membrane;mitochondrion;ribosome		0.668	0.668	1.00
Q12283	Malonyl CoA-acyl carrier protein transacylase, mitochondrial [OS=Saccharomyces cerevisiae S288c]	854396	MCT1; YOR221C	metabolic process	mitochondrion	catalytic activity	0.896	0.896	1.00
P38702	mitochondrial carrier protein LEU5 [OS=Saccharomyces cerevisiae S288c]	856391	LEU5; YHR002W	metabolic process;transport	membrane;mitochondrion	structural molecule activity;transporter activity	0.468	0.468	1.00
P53732	37S ribosomal protein S12, mitochondrial [OS=Saccharomyces	855772	MRPS12;	cell organization and biogenesis;metabolic process	mitochondrion;ribosome	structural molecule activity	1.783	1.783	1.00

	cerevisiae S288c]		YNR036C						
P28239	Inorganic pyrophosphatase, mitochondrial [OS=Saccharomyces cerevisiae S288c]	855309	PPA2; YMR267W	metabolic process	cytoplasm;mitochondrion	catalytic activity;metal ion binding	0.585	0.585	1.00
P14908	Mitochondrial transcription factor 1 [OS=Saccharomyces cerevisiae S288c]	855268	MTF1; YMR228W	metabolic process;regulation of biological process	mitochondrion;organelle lumen	catalytic activity;DNA binding;RNA binding	0.585	0.585	1.00
P36163	mitochondrial metalloendopeptidase OMA1 [OS=Saccharomyces cerevisiae S288c]	853962	OMA1; YKR087C	metabolic process	membrane;mitochondrion	catalytic activity;metal ion binding	0.73	0.73	1.00
Q12233	ATP synthase subunit g, mitochondrial [OS=Saccharomyces cerevisiae S288c]	856131	ATP20; YPR020W	cell organization and biogenesis;metabolic process;transport	membrane;mitochondrion	catalytic activity;transporter activity	1.371	1.371	1.00
P37299	cytochrome b-c1 complex subunit 10 [OS=Saccharomyces cerevisiae S288c]	856390	QCR10; YHR001W-A	metabolic process;transport	membrane;mitochondrion	catalytic activity;transporter activity	5.31	5.31	1.00
P33421	Succinate dehydrogenase [ubiquinone] cytochrome b subunit, mitochondrial [OS=Saccharomyces cerevisiae S288c]	853716	SDH3; YKL141W	cell organization and biogenesis;metabolic process;transport	membrane;mitochondrion	catalytic activity;metal ion binding	1.276	1.276	1.00
P23369	54S ribosomal protein L25, mitochondrial [OS=Saccharomyces cerevisiae S288c]	852967	MRPL25; YGR076C	cell organization and biogenesis;metabolic process	mitochondrion;ribosome	structural molecule activity	1.31	1.31	1.00
P48526	Isoleucine--tRNA ligase, mitochondrial [OS=Saccharomyces cerevisiae S288c]	856067	ISM1; YPL040C	cell organization and biogenesis;metabolic process;regulation of biological process	cytoplasm;cytosol;mitochondrion;organelle lumen	catalytic activity;nucleotide binding	0.215	0.215	1.00
P04807	Hexokinase-2 [OS=Saccharomyces cerevisiae S288c]	852639	HXK2; YGL253W	cell communication;cell organization and biogenesis;cellular homeostasis;metabolic process;regulation of biological process;response to stimulus;transport	cytosol;mitochondrion;nucleus	catalytic activity;nucleotide binding	0.359	0.359	1.00
P21306	ATP synthase subunit epsilon, mitochondrial [OS=Saccharomyces cerevisiae S288c]	855857	ATP15; YPL271W	metabolic process;transport	membrane;mitochondrion	catalytic activity;transporter activity	2.162	2.162	1.00
Q06485	Autophagy-related protein 33 [OS=Saccharomyces cerevisiae S288c]	851070	ATG33; YLR356W	cell communication;metabolic process;response to stimulus	membrane;mitochondrion		1.371	1.371	1.00
Q08742	Thiosulfate sulfurtransferase RDL2, mitochondrial [OS=Saccharomyces cerevisiae S288c]	854460	RDL2; YOR286W		mitochondrion	catalytic activity	0.52	0.52	1.00
Q07914	mitochondrial import inner membrane translocase subunit TIM14 [OS=Saccharomyces cerevisiae S288c]	850694	PAM18; YLR008C	regulation of biological process;transport	membrane;mitochondrion	enzyme regulator activity;protein binding;transporter activity	0.778	0.778	1.00
Q03557	Glutamyl-tRNA(Gln) amidotransferase subunit A, mitochondrial [OS=Saccharomyces cerevisiae S288c]	855338	HER2; YMR293C	cell organization and biogenesis;metabolic process	mitochondrion	catalytic activity;nucleotide binding	0.259	0.259	1.00
P38172	MIOREX complex component 3 [OS=Saccharomyces cerevisiae S288c]	852181	YBL095W; MRX3; YBL095W		membrane;mitochondrion		0.501	0.501	1.00
P32785	Methionyl-tRNA formyltransferase, mitochondrial [OS=Saccharomyces cerevisiae S288c]	852270	FMT1; YBL013W	metabolic process	mitochondrion	catalytic activity	0.179	0.179	1.00
Q01976	ADP-ribose pyrophosphatase [OS=Saccharomyces cerevisiae S288c]	852408	YSA1; YBR111C	metabolic process	cytoplasm;mitochondrion;nucleus	catalytic activity;metal ion binding	0.425	0.425	1.00
P43122	tRNA N6-adenosine threonylcarbamoyltransferase, mitochondrial [OS=Saccharomyces cerevisiae S288c]	851454	QRI7; YDL104C	cell organization and biogenesis;metabolic process	mitochondrion	catalytic activity;metal ion binding	0.212	0.212	1.00
P34224	Uncharacterized protein YBL059W [OS=Saccharomyces cerevisiae S288c]	852221	YBL059W; YBL059W		membrane;mitochondrion		0.468	0.468	1.00
P21375	Fumarate reductase 2 [OS=Saccharomyces cerevisiae S288c]	853510	OSM1; YJR051W	metabolic process	endoplasmic reticulum;mitochondrion	catalytic activity;nucleotide binding	0.25	0.25	1.00
P38175	37S ribosomal protein MRP21, mitochondrial [OS=Saccharomyces cerevisiae S288c]	852188	MRP21; YBL090W	cell organization and biogenesis;metabolic process	mitochondrion;ribosome	structural molecule activity	0.701	0.701	1.00
P0CX31	40S ribosomal protein S24-A [OS=Saccharomyces cerevisiae S288c]	856805; 854741	RPS24A; RPS24B; YER074W; YIL069C	metabolic process	cytoplasm;mitochondrion;ribosome	nucleotide binding;structural molecule activity	0.585	0.585	1.00
Q03429	Mitochondrial zinc maintenance protein 1, mitochondrial [OS=Saccharomyces cerevisiae S288c]	852104	MZM1; YDR493W	cell organization and biogenesis;metabolic process	mitochondrion;organelle lumen	protein binding	0.334	0.334	1.00

O74700	Mitochondrial import inner membrane translocase subunit Tim9 [OS=Saccharomyces cerevisiae S288c]	856693	TIM9; YEL020W-A	cell organization and biogenesis;transport	membrane;mitochondrion	metal ion binding;protein binding;transporter activity	0.468	0.468	1.00
P18409	Mitochondrial distribution and morphology protein 10 [OS=Saccharomyces cerevisiae S288c]	851223	MDM10; YAL010C	cell organization and biogenesis;transport	membrane;mitochondrion	protein binding	0.16	0.16	1.00
P38876	peptidyl-tRNA hydrolase [OS=Saccharomyces cerevisiae S288c]	856596	PTH1; YHR189W	cell organization and biogenesis;metabolic process	mitochondrion	catalytic activity	0.194	0.194	1.00
P00420	Cytochrome c oxidase subunit 3 [OS=Saccharomyces cerevisiae S288c]	854627	COX3; Q0275	metabolic process;transport	membrane;mitochondrion	catalytic activity;transporter activity	3.642	3.642	1.00
P34251	Uncharacterized oxidoreductase YKL107W [OS=Saccharomyces cerevisiae S288c]	853753	YKL107W; YKL107W	metabolic process	membrane	catalytic activity	0.438	0.438	1.00
P22438	Methionine--tRNA ligase, mitochondrial [OS=Saccharomyces cerevisiae S288c]	853081	MSM1; YGR171C	metabolic process	cytoplasm;cytosol;mitochondrion;organelle lumen	catalytic activity;nucleotide binding	0.136	0.136	1.00
P41735	5-demethoxyubiquinone hydroxylase, mitochondrial [OS=Saccharomyces cerevisiae S288c]	854292	CAT5; YOR125C	metabolic process	membrane;mitochondrion	catalytic activity;metal ion binding	0.334	0.334	1.00
Q3E7A4	COX assembly mitochondrial protein 2 [OS=Saccharomyces cerevisiae S288c]	852220	CMC2; YBL059C-A	cell organization and biogenesis	cytoplasm;membrane;mitochondrion;nucleus	metal ion binding	0.931	0.931	1.00
Q12375	mitochondrial ornithine transporter 1 [OS=Saccharomyces cerevisiae S288c]	854297	ORT1; YOR130C	metabolic process;transport	membrane;mitochondrion	structural molecule activity;transporter activity	0.334	0.334	1.00
Q12328	Mitochondrial import inner membrane translocase subunit TIM22 [OS=Saccharomyces cerevisiae S288c]	851309	TIM22; YDL217C	cell organization and biogenesis;transport	cytosol;membrane;mitochondrion	protein binding;transporter activity	0.233	0.233	1.00
P01098	ATPase-stabilizing factor 9 kDa, mitochondrial [OS=Saccharomyces cerevisiae S288c]	851426	STF1; YDL130W-A	metabolic process;regulation of biological process	mitochondrion	catalytic activity;enzyme regulator activity;protein binding	2.162	2.162	1.00
P28627	mitochondrial inner membrane protease subunit 1 [OS=Saccharomyces cerevisiae S288c]	855182	IMP1; YMR150C	cell organization and biogenesis;metabolic process	membrane;mitochondrion	catalytic activity	0.425	0.425	1.00
P53081	NGG1-interacting factor 3 [OS=Saccharomyces cerevisiae S288c]	852651	NIF3; YGL221C		cytoplasm;mitochondrion		0.501	0.501	1.00
P14063	54S ribosomal protein L31, mitochondrial [OS=Saccharomyces cerevisiae S288c]	853720	MRPL31; YKL138C	cell organization and biogenesis;metabolic process	mitochondrion;ribosome	structural molecule activity	0.668	0.668	1.00
P28737	Protein MSP1 [OS=Saccharomyces cerevisiae S288c]	852915	MSP1; YGR028W	cell organization and biogenesis;metabolic process;regulation of biological process;response to stimulus;transport	membrane;mitochondrion;nucleus	catalytic activity;nucleotide binding;protein binding;RNA binding	0.259	0.259	1.00
Q3E6R5	uncharacterized mitochondrial outer membrane protein YDR381C-A [OS=Saccharomyces cerevisiae S288c]	851989	YDR381C-A; YDR381C-A		membrane;mitochondrion		0.389	0.389	1.00
P32378	4-hydroxybenzoate polyprenyltransferase, mitochondrial [OS=Saccharomyces cerevisiae S288c]	855778	COQ2; YNR041C	metabolic process;transport	membrane;mitochondrion	catalytic activity	0.292	0.292	1.00
Q07821	Iron-sulfur assembly protein 1 [OS=Saccharomyces cerevisiae S288c]	850632	ISA1; YLL027W	cell organization and biogenesis;metabolic process	mitochondrion;organelle lumen	metal ion binding;structural molecule activity	0.136	0.136	1.00
Q00402	Nuclear migration protein NUM1 [OS=Saccharomyces cerevisiae S288c]	851727	NUM1; YDR150W	cell organization and biogenesis;cellular component movement;metabolic process;regulation of biological process;transport	endoplasmic reticulum;mitochondrion	catalytic activity;motor activity;protein binding	0.025	0.025	1.00
Q01802	Aspartate aminotransferase, mitochondrial [OS=Saccharomyces cerevisiae S288c]	853755	AAT1; YKL106W	metabolic process	mitochondrion;organelle lumen	catalytic activity;protein binding	0.075	0.075	1.00
Q2V2P9	Uncharacterized protein YDR119W-A [OS=Saccharomyces cerevisiae S288c]	3799970	COX26; YDR119W-A		membrane;mitochondrion		1.154	1.154	1.00
P32463	Acyl carrier protein, mitochondrial [OS=Saccharomyces cerevisiae S288c]	853642	ACP1; YKL192C	metabolic process;transport	mitochondrion		0.259	0.259	1.00
P42844	Mitochondrial protein import protein ZIM17 [OS=Saccharomyces cerevisiae S288c]	855406	ZIM17; YNL310C	cell organization and biogenesis;metabolic process;regulation of biological process;response to stimulus;transport	membrane;mitochondrion;organelle lumen	metal ion binding;protein binding	0.585	0.585	1.00
Q3E846	Cytochrome c oxidase assembly factor 6 [OS=Saccharomyces cerevisiae S288c]	855287	COA6; YMR244C-A;	cell organization and biogenesis;cellular homeostasis;metabolic	cytoplasm;membrane;mitochondrion;nucleus	catalytic activity;metal ion binding;protein	0.931	0.931	1.00

			YMR244C-A	process;transport		binding;transporter activity			
P32048	Lysine--tRNA ligase, mitochondrial [OS=Saccharomyces cerevisiae S288c]	855651	MSK1; YNL073W	cell organization and biogenesis;metabolic process;transport	cytoplasm;mitochondrion;organelle lumen	catalytic activity;nucleotide binding;RNA binding	0.059	0.059	1.00
P04039	Cytochrome c oxidase polypeptide VIII, mitochondrial [OS=Saccharomyces cerevisiae S288c]	851111	COX8; YLR395C	metabolic process;transport	membrane;mitochondrion	catalytic activity;transporter activity	0.778	0.778	1.00
P38812	Phosphatidylglycerophosphatase GEP4, mitochondrial [OS=Saccharomyces cerevisiae S288c]	856500	GEP4; YHR100C	metabolic process	membrane;mitochondrion;organelle lumen	catalytic activity	0.179	0.179	1.00
Q06089	Uncharacterized mitochondrial outer membrane protein YPR098C [OS=Saccharomyces cerevisiae S288c]	856213	YPR098C; YPR098C		membrane;mitochondrion		2.162	2.162	1.00
P32792	UPF0744 protein YSC83 [OS=Saccharomyces cerevisiae S288c]	856410	YSC83; YHR017W		membrane;mitochondrion		0.11	0.11	1.00
Q12487	54S ribosomal protein L23, mitochondrial [OS=Saccharomyces cerevisiae S288c]	854321	MRPL23; YOR150W	cell organization and biogenesis;metabolic process	mitochondrion;ribosome;vacuole	RNA binding;structural molecule activity	0.233	0.233	1.00
P18411	Protein FUN14 [OS=Saccharomyces cerevisiae S288c]	851225	FUN14; YAL008W	metabolic process	membrane;mitochondrion		0.194	0.194	1.00
P10174	cytochrome c oxidase subunit 7 [OS=Saccharomyces cerevisiae S288c]	855298	COX7; YMR256C	metabolic process;transport	membrane;mitochondrion	catalytic activity;transporter activity	1.154	1.154	1.00
P27882	Mitochondrial FAD-linked sulfhydryl oxidase erv1 [OS=Saccharomyces cerevisiae S288c]	852916	ERV1; YGR029W	cellular homeostasis;metabolic process;response to stimulus;transport	mitochondrion	catalytic activity;protein binding	0.212	0.212	1.00
P53318	Ubiquinone biosynthesis monooxygenase COQ6, mitochondrial [OS=Saccharomyces cerevisiae S288c]	853170	COQ6; YGR255C	metabolic process	membrane;mitochondrion	catalytic activity;nucleotide binding	0.077	0.077	1.00
Q05779	Ubiquinone biosynthesis protein COQ9, mitochondrial [OS=Saccharomyces cerevisiae S288c]	850898	COQ9; YLR201C	metabolic process	membrane;mitochondrion		0.166	0.166	1.00
Q02863	ubiquitin carboxyl-terminal hydrolase 16 [OS=Saccharomyces cerevisiae S288c]	856032	UBP16; YPL072W	metabolic process	membrane;mitochondrion	catalytic activity	0.072	0.072	1.00
P39103	Cytochrome c oxidase assembly protein COX14 [OS=Saccharomyces cerevisiae S288c]	854910	COX14; YML129C	cell organization and biogenesis;regulation of biological process	membrane;mitochondrion;organelle lumen	protein binding	0.585	0.585	1.00
P25578	CDP-diacylglycerol--glycerol-3-phosphate 3-phosphatidyltransferase [OS=Saccharomyces cerevisiae S288c]	850352	PGS1; YCL004W	metabolic process	mitochondrion	catalytic activity;nucleotide binding	0.083	0.083	1.00
Q03702	Cruciform cutting endonuclease 1, mitochondrial [OS=Saccharomyces cerevisiae S288c]	853858	CCE1; YKL011C	cell organization and biogenesis;metabolic process	membrane;mitochondrion	catalytic activity;DNA binding;metal ion binding	0.105	0.105	1.00
P32388	54S ribosomal protein MRP49, mitochondrial [OS=Saccharomyces cerevisiae S288c]	853687	MRP49; YKL167C	cell organization and biogenesis;metabolic process	mitochondrion;ribosome	structural molecule activity	0.259	0.259	1.00
P53848	Folic acid synthesis protein fol1 [OS=Saccharomyces cerevisiae S288c]	855465	FOL1; YNL256W	metabolic process	cytoplasm;membrane;mitochondrion	catalytic activity;metal ion binding;nucleotide binding	0.045	0.045	1.00
P38271	Protein OPY1 [OS=Saccharomyces cerevisiae S288c]	852426	OPY1; YBR129C		cytoplasm;mitochondrion		0.129	0.129	1.00
P36775	Lon protease homolog, mitochondrial [OS=Saccharomyces cerevisiae S288c]	852259	PIM1; YBL022C	cell organization and biogenesis;metabolic process;regulation of biological process;response to stimulus	cytoplasm;mitochondrion;organelle lumen	catalytic activity;DNA binding;nucleotide binding;RNA binding	1.362	1.436	1.05
P49095	Glycine dehydrogenase (Decarboxylating), mitochondrial [OS=Saccharomyces cerevisiae S288c]	855227	GCV2; YMR189W	metabolic process	cytosol;mitochondrion	catalytic activity	2.433	2.577	1.06
P32796-1	Carnitine O-acetyltransferase, mitochondrial [OS=Saccharomyces cerevisiae S288c]	854965	CAT2; YML042W	metabolic process;transport	membrane;mitochondrion	catalytic activity	13.816	14.672	1.06
Q08822	Probable electron transfer flavoprotein-ubiquinone oxidoreductase, mitochondrial [OS=Saccharomyces cerevisiae S288c]	854538	CIR2; YOR356W	metabolic process;transport	membrane;mitochondrion	catalytic activity;metal ion binding;nucleotide binding	3.424	3.642	1.06
P21954	Isocitrate dehydrogenase [NADP], mitochondrial [OS=Saccharomyces cerevisiae S288c]	851493	IDP1; YDL066W	metabolic process	mitochondrion	catalytic activity;metal ion binding;nucleotide binding;protein binding	38.811	41.987	1.08

Q12166	2-isopropylmalate synthase 2, mitochondrial [OS=Saccharomyces cerevisiae S288c]	854275	LEU9; YOR108W	metabolic process	mitochondrion	catalytic activity;protein binding	2.472	2.695	1.09
P07257	Cytochrome b-c1 complex subunit 2, mitochondrial [OS=Saccharomyces cerevisiae S288c]	856321	QCR2; YPR191W	metabolic process;transport	membrane;mitochondrion	catalytic activity;metal ion binding;protein binding;transporter activity	36.751	40.246	1.10
Q02776	Mitochondrial import inner membrane translocase subunit TIM50 [OS=Saccharomyces cerevisiae S288c]	856042	TIM50; YPL063W	cell organization and biogenesis;metabolic process;transport	membrane;mitochondrion	catalytic activity;protein binding;transporter activity	9	9.857	1.10
P48015	Aminomethyltransferase, mitochondrial [OS=Saccharomyces cerevisiae S288c]	851582	GCV1; YDR019C	metabolic process	mitochondrion	catalytic activity;protein binding	9	10.053	1.12
P38077	ATP synthase subunit gamma, mitochondrial [OS=Saccharomyces cerevisiae S288c]	852327	ATP3; YBR039W	metabolic process;transport	membrane;mitochondrion	catalytic activity;transporter activity	12.689	14.199	1.12
Q08023	Protein FMP25, mitochondrial [OS=Saccharomyces cerevisiae S288c]	850766	FMP25; YLR077W	cell organization and biogenesis	membrane;mitochondrion		1.412	1.581	1.12
P38891	Branched-chain-amino-acid aminotransferase, mitochondrial [OS=Saccharomyces cerevisiae S288c]	856615	BAT1; YHR208W	metabolic process;regulation of biological process;response to stimulus	mitochondrion;organelle lumen	catalytic activity	9	10.103	1.12
P50085	Prohibitin-2 [OS=Saccharomyces cerevisiae S288c]	853146	PHB2; YGR231C	cell organization and biogenesis;metabolic process;regulation of biological process	membrane;mitochondrion	protein binding	4.926	5.579	1.13
P19882	heat shock protein 60, mitochondrial [OS=Saccharomyces cerevisiae S288c]	850963	HSP60; YLR259C	cell organization and biogenesis;metabolic process;regulation of biological process;transport	cytoplasm;cytosol;membrane;mitochondrion;organelle lumen	catalytic activity;DNA binding;nucleotide binding;protein binding	22.357	25.367	1.13
P36060	NADH-cytochrome b5 reductase 2 [OS=Saccharomyces cerevisiae S288c]	853707	MCR1; YKL150W	metabolic process;response to stimulus	membrane;mitochondrion	catalytic activity;protein binding	15.238	17.33	1.14
P28834	Isocitrate dehydrogenase [NAD] subunit 1, mitochondrial [OS=Saccharomyces cerevisiae S288c]	855691	IDH1; YNL037C	metabolic process;transport	cytosol;mitochondrion;organelle lumen	catalytic activity;metal ion binding;nucleotide binding;protein binding;RNA binding	15.238	17.33	1.14
P04710	ADP,ATP carrier protein 1 [OS=Saccharomyces cerevisiae S288c]	855078	AAC1; YMR056C	metabolic process;transport	cytosol;membrane;mitochondrion	structural molecule activity;transporter activity	11.743	13.384	1.14
P25573	Mitochondrial inner membrane i-AAA protease supercomplex subunit MGR1 [OS=Saccharomyces cerevisiae S288c]	850313	MGR1; YCL044C	metabolic process	membrane;mitochondrion	protein binding	1.649	1.894	1.15
P09457	ATP synthase subunit 5, mitochondrial [OS=Saccharomyces cerevisiae S288c]	851892	ATP5; YDR298C	metabolic process;transport	membrane;mitochondrion	catalytic activity;transporter activity	9	10.365	1.15
P38988	Mitochondrial GTP/GDP carrier protein 1 [OS=Saccharomyces cerevisiae S288c]	851329	GGC1; YDL198C	cell organization and biogenesis;cellular homeostasis;metabolic process;transport	membrane;mitochondrion	structural molecule activity;transporter activity	3.16	3.642	1.15
Q12031	Mitochondrial 2-methylisocitrate lyase [OS=Saccharomyces cerevisiae S288c]	856114	ICL2; YPR006C	metabolic process	mitochondrion;organelle lumen	catalytic activity	5.158	5.952	1.15
P00431	cytochrome c peroxidase, mitochondrial [OS=Saccharomyces cerevisiae S288c]	853940	CCP1; YKR066C	metabolic process;response to stimulus	mitochondrion;organelle lumen	antioxidant activity;catalytic activity;metal ion binding;protein binding	1.424	1.649	1.16
P53292	37S ribosomal protein S35, mitochondrial [OS=Saccharomyces cerevisiae S288c]	853075	MRPS35; YGR165W	cell organization and biogenesis;metabolic process	mitochondrion;ribosome	structural molecule activity	1.512	1.754	1.16
Q12230	Sphingolipid long chain base-responsive protein LSP1 [OS=Saccharomyces cerevisiae S288c]	856103	LSP1; YPL004C	regulation of biological process;response to stimulus;transport	cytoplasm;membrane;mitochondrion		24.119	28.286	1.17
P00924	Enolase 1 [OS=Saccharomyces cerevisiae S288c]	853169	ENO1; YGR254W	metabolic process;regulation of biological process	cytoplasm;cytosol;membrane;mitochondrion;vacuole	catalytic activity;metal ion binding;protein binding	0.848	0.995	1.17
P38969	Pentamidine resistance factor, mitochondrial [OS=Saccharomyces cerevisiae S288c]	854440	PNT1; YOR266W	cell organization and biogenesis;response to stimulus	membrane;mitochondrion		0.848	0.995	1.17
P15424	ATP-dependent RNA helicase MSS116, mitochondrial [OS=Saccharomyces cerevisiae S288c]	851775	MSS116; YDR194C	metabolic process;regulation of biological process	mitochondrion;organelle lumen	catalytic activity;nucleotide binding;RNA binding	1.644	1.929	1.17
P32340	Rotenone-insensitive NADH-ubiquinone oxidoreductase, mitochondrial [OS=Saccharomyces cerevisiae S288c]	854919	NDI1; YML120C	metabolic process;regulation of biological process	membrane;mitochondrion;organelle lumen	catalytic activity;nucleotide binding	24.929	29.392	1.18

P53140	Protein RMD9, mitochondrial [OS=Saccharomyces cerevisiae S288c]	852771	RMD9; YGL107C	cell differentiation;metabolic process;regulation of biological process	membrane;mitochondrion	RNA binding	1.512	1.783	1.18
P08417	fumarate hydratase, mitochondrial [OS=Saccharomyces cerevisiae S288c]	855866	FUM1; YPL262W	cell organization and biogenesis;metabolic process	cytoplasm;cytosol;mitochondrion;organelle lumen	catalytic activity	13.874	16.433	1.18
Q01217	Protein ARG5,6, mitochondrial [OS=Saccharomyces cerevisiae S288c]	856800	ARG5,6; YER069W	metabolic process;regulation of biological process	cytoplasm;mitochondrion;organelle lumen	catalytic activity;nucleotide binding;protein binding	0.286	0.341	1.19
P06168	ketol-acid reductoisomerase, mitochondrial [OS=Saccharomyces cerevisiae S288c]	851069	ILV5; YLR355C	cell organization and biogenesis;metabolic process	mitochondrion	catalytic activity;DNA binding;metal ion binding	12.895	15.379	1.19
P32860	NifU-like protein, mitochondrial [OS=Saccharomyces cerevisiae S288c]	853826	NFU1; YKL040C	cell organization and biogenesis;cellular homeostasis;metabolic process	mitochondrion;organelle lumen	metal ion binding	3.87	4.623	1.19
Q02486	ARS-binding factor 2, mitochondrial [OS=Saccharomyces cerevisiae S288c]	855094	ABF2; YMR072W	cell organization and biogenesis;metabolic process;regulation of biological process	chromosome;mitochondrion;nucleus	DNA binding	69.17	82.768	1.20
P37293	N-terminal acetyltransferase 2 [OS=Saccharomyces cerevisiae S288c]	853050	NAT2; YGR147C	metabolic process	cytoplasm;mitochondrion	catalytic activity	1.404	1.683	1.20
P02992	elongation factor Tu, mitochondrial [OS=Saccharomyces cerevisiae S288c]	854359	TUF1; YOR187W	cell organization and biogenesis;metabolic process	mitochondrion	catalytic activity;nucleotide binding;RNA binding	30.623	36.751	1.20
P40008	Protein FMP52, mitochondrial [OS=Saccharomyces cerevisiae S288c]	856721	FMP52; YER004W		endoplasmic reticulum;membrane;mitochondrion	catalytic activity	3.217	3.87	1.20
P48527	Tyrosine--tRNA ligase, mitochondrial [OS=Saccharomyces cerevisiae S288c]	856007	MSY1; YPL097W	metabolic process	cytoplasm;cytosol;mitochondrion;organelle lumen	catalytic activity;nucleotide binding;RNA binding	0.52	0.63	1.21
P30902	ATP synthase subunit d, mitochondrial [OS=Saccharomyces cerevisiae S288c]	853853	ATP7; YKL016C	metabolic process;transport	membrane;mitochondrion	catalytic activity;transporter activity	5.105	6.197	1.21
P08067	cytochrome b-c1 complex subunit Rieske, mitochondrial [OS=Saccharomyces cerevisiae S288c]	856689	RIP1; YEL024W	metabolic process;transport	membrane;mitochondrion	catalytic activity;metal ion binding;transporter activity	55.234	67.129	1.22
Q08223	Altered inheritance of mitochondria protein 39, mitochondrial [OS=Saccharomyces cerevisiae S288c]	854103	AIM39; YOL053W		cytosol;membrane;mitochondrion		0.585	0.711	1.22
P54783	D-arabinono-1,4-lactone oxidase [OS=Saccharomyces cerevisiae S288c]	854888	ALO1; YML086C	metabolic process;response to stimulus	membrane;mitochondrion	catalytic activity;nucleotide binding	3.299	4.012	1.22
P00175	Cytochrome b2, mitochondrial [OS=Saccharomyces cerevisiae S288c]	854950	CYB2; YML054C	metabolic process;transport	cytosol;membrane;mitochondrion;nucleus	catalytic activity;metal ion binding;nucleotide binding	1.239	1.512	1.22
P25719	Peptidyl-prolyl cis-trans isomerase C, mitochondrial [OS=Saccharomyces cerevisiae S288c]	854897	CPR3; YML078W	cell death;metabolic process	mitochondrion;organelle lumen	catalytic activity	2.981	3.642	1.22
P00447	Superoxide dismutase [Mn], mitochondrial [OS=Saccharomyces cerevisiae S288c]	856399	SOD2; YHR008C	metabolic process;response to stimulus	mitochondrion;organelle lumen	antioxidant activity;catalytic activity;metal ion binding	6.017	7.377	1.23
P40341	Mitochondrial respiratory chain complexes assembly protein YTA12 [OS=Saccharomyces cerevisiae S288c]	855114	YTA12; YMR089C	cell organization and biogenesis;metabolic process;response to stimulus;transport	cytoplasm;membrane;mitochondrion	catalytic activity;metal ion binding;nucleotide binding;protein binding	1.371	1.683	1.23
P23833	Protein SCO1, mitochondrial [OS=Saccharomyces cerevisiae S288c]	852325	SCO1; YBR037C	cell organization and biogenesis;cellular homeostasis;response to stimulus;transport	membrane;mitochondrion	antioxidant activity;catalytic activity;metal ion binding	1.448	1.783	1.23
P32331	Carrier protein YMC1, mitochondrial [OS=Saccharomyces cerevisiae S288c]	856171	YMC1; YPR058W	metabolic process;transport	membrane;mitochondrion;vacuole	structural molecule activity;transporter activity	1.581	1.955	1.24
Q08968	UPF0061 protein FMP40 [OS=Saccharomyces cerevisiae S288c]	855879	FMP40; YPL222W		mitochondrion	protein binding	2.162	2.675	1.24
P32891	D-lactate dehydrogenase [cytochrome] 1, mitochondrial [OS=Saccharomyces cerevisiae S288c]	851380	DLD1; YDL174C	metabolic process;transport	membrane;mitochondrion	catalytic activity;nucleotide binding	18.684	23.119	1.24
P04037	cytochrome c oxidase subunit 4, mitochondrial [OS=Saccharomyces cerevisiae S288c]	852688	COX4; YGL187C	cell organization and biogenesis;metabolic process;transport	membrane;mitochondrion	catalytic activity;metal ion binding;transporter activity	42.288	52.367	1.24
P53219	Abhydrolase domain-containing protein IMO32 [OS=Saccharomyces cerevisiae S288c]	852919	IMO32; YGR031W	metabolic process;transport	mitochondrion	catalytic activity	0.931	1.154	1.24
P00427	Cytochrome c oxidase subunit 6, mitochondrial [OS=Saccharomyces	856448	COX6; YHR051	metabolic process;transport	membrane;mitochondrion	catalytic activity;metal ion	7.254	9	1.24

	cerevisiae S288c]		W			binding;transporter activity			
P32795	Mitochondrial inner membrane i-AAA protease supercomplex subunit YME1 [OS=Saccharomyces cerevisiae S288c]	856135	YME1; YPR024W	metabolic process;response to stimulus;transport	membrane;mitochondrion	catalytic activity;metal ion binding;nucleotide binding;protein binding	1.798	2.241	1.25
P47052	succinate dehydrogenase [ubiquinone] flavoprotein subunit 2, mitochondrial [OS=Saccharomyces cerevisiae S288c]	853405	YJL045W; YJL045W	metabolic process;transport	membrane;mitochondrion	catalytic activity;nucleotide binding	4.712	5.884	1.25
P36521	54S ribosomal protein L11, mitochondrial [OS=Saccharomyces cerevisiae S288c]	851325	MRPL11; YDL202W	cell organization and biogenesis;metabolic process	mitochondrion;ribosome	structural molecule activity	1.069	1.336	1.25
P00927	Threonine dehydratase, mitochondrial [OS=Saccharomyces cerevisiae S288c]	856819	ILV1; YER086W	metabolic process	cytoplasm;mitochondrion	catalytic activity	3.125	3.924	1.26
P16451	Pyruvate dehydrogenase complex protein X component, mitochondrial [OS=Saccharomyces cerevisiae S288c]	853107	PDX1; YGR193C	metabolic process	mitochondrion;organelle lumen	catalytic activity;structural molecule activity	2.594	3.262	1.26
P38071	Enoyl-[acyl-carrier protein] reductase [NADPH, B-specific], mitochondrial [OS=Saccharomyces cerevisiae S288c]	852314	ETR1; YBR026C	metabolic process	mitochondrion;organelle lumen	catalytic activity;DNA binding;metal ion binding	9	11.328	1.26
Q05931	Heat shock protein SSQ1, mitochondrial [OS=Saccharomyces cerevisiae S288c]	851084	SSQ1; YLR369W	cell organization and biogenesis;metabolic process	mitochondrion;organelle lumen	nucleotide binding;protein binding	0.884	1.113	1.26
P40513	Mitochondrial acidic protein MAM33 [OS=Saccharomyces cerevisiae S288c]	854740	MAM33; YIL070C	metabolic process;regulation of biological process	mitochondrion;organelle lumen	translation regulator activity	3.642	4.623	1.27
P53721	Respiratory supercomplex factor 2, mitochondrial [OS=Saccharomyces cerevisiae S288c]	855752	RCF2; YNR018W	cell organization and biogenesis	membrane;mitochondrion		5.579	7.111	1.27
P33416	Heat shock protein 78, mitochondrial [OS=Saccharomyces cerevisiae S288c]	851845	HSP78; YDR258C	cell organization and biogenesis;metabolic process;regulation of biological process;response to stimulus	mitochondrion;organelle lumen	catalytic activity;nucleotide binding;protein binding	7.929	10.199	1.29
Q03640	Tricalbin-3 [OS=Saccharomyces cerevisiae S288c]	854903	TCB3; YML072C	cell organization and biogenesis;regulation of biological process	endoplasmic reticulum;membrane;mitochondrion	metal ion binding;protein binding	0.283	0.365	1.29
P39987	Heat shock protein SSC3, mitochondrial [OS=Saccharomyces cerevisiae S288c]	856682	ECM10; YEL030W	cell organization and biogenesis;metabolic process;transport	mitochondrion	nucleotide binding;protein binding	2.162	2.793	1.29
P53889	uncharacterized mitochondrial hydrolase FMP41 [OS=Saccharomyces cerevisiae S288c]	855553	FMP41; YNL168C	metabolic process	mitochondrion	catalytic activity;metal ion binding	0.968	1.254	1.30
P50945	MICOS complex subunit MIC27 [OS=Saccharomyces cerevisiae S288c]	855623	AIM37; MIC27; YNL100W	cell organization and biogenesis	membrane;mitochondrion		0.968	1.254	1.30
P0CS90	Heat shock protein SSC1, mitochondrial [OS=Saccharomyces cerevisiae S288c]	853503	SSC1; YJR045C	cell organization and biogenesis;metabolic process;regulation of biological process;transport	membrane;mitochondrion;nucleus;organelle lumen	catalytic activity;enzyme regulator activity;nucleotide binding;protein binding	48.482	62.908	1.30
P00128	Cytochrome b-c1 complex subunit 7 [OS=Saccharomyces cerevisiae S288c]	852142	QCR7; YDR529C	cell organization and biogenesis;metabolic process;transport	membrane;mitochondrion	catalytic activity;transporter activity	34.938	45.416	1.30
Q02204	54S ribosomal protein L13, mitochondrial [OS=Saccharomyces cerevisiae S288c]	853875	MRPL13; YKR006C	cell organization and biogenesis;metabolic process	mitochondrion;ribosome	structural molecule activity	1.054	1.371	1.30
P43616	Cys-Gly metallodipeptidase dug1 [OS=Saccharomyces cerevisiae S288c]	850605	DUG1; YFR044C	metabolic process	cytoplasm;mitochondrion;ribosome	catalytic activity;metal ion binding	1.154	1.512	1.31
Q03327	Mitochondrial fusion and transport protein Ugo1 [OS=Saccharomyces cerevisiae S288c]	852081	UGO1; YDR470C	cell organization and biogenesis;metabolic process	membrane;mitochondrion	protein binding;structural molecule activity	0.425	0.557	1.31
Q03246	37S ribosomal protein S17, mitochondrial [OS=Saccharomyces cerevisiae S288c]	855226	MRPS17; YMR188C	cell differentiation;cell organization and biogenesis;metabolic process	mitochondrion;ribosome	RNA binding;structural molecule activity	1.276	1.683	1.32
P00424	Cytochrome c oxidase polypeptide 5A, mitochondrial [OS=Saccharomyces cerevisiae S288c]	855675	COX5A; YNL052W	metabolic process;transport	membrane;mitochondrion	catalytic activity;transporter activity	2.511	3.329	1.33
P32454	Aminopeptidase 2, mitochondrial [OS=Saccharomyces cerevisiae S288c]	853699	APE2; YKL157W	metabolic process	cytoplasm;extracellular;membrane;mitochondrion;nucleus	catalytic activity;metal ion binding	0.973	1.294	1.33
P39522	Dihydroxy-acid dehydratase, mitochondrial [OS=Saccharomyces cerevisiae S288c]	853473	ILV3; YJR016C	metabolic process	mitochondrion	catalytic activity;metal ion binding	29.539	39.37	1.33
P00401	Cytochrome c oxidase subunit 1 [OS=Saccharomyces cerevisiae S288c]	854598	COX1; Q0045	metabolic process;transport	membrane;mitochondrion	catalytic activity;metal ion binding;transporte	1.61	2.162	1.34

						r activity			
Q12480	Probable electron transfer flavoprotein subunit alpha, mitochondrial [OS=Saccharomyces cerevisiae S288c]	85611 2	AIM45; YPR004 C	metabolic process;transport	mitochondrion;organelle lumen	catalytic activity;nucleotide binding	2.981	4.012	1.35
P32898	Mitochondrial presequence protease [OS=Saccharomyces cerevisiae S288c]	85204 1	CYM1; YDR430 C	metabolic process	mitochondrion	catalytic activity;metal ion binding	1.349	1.829	1.36
P16387	Pyruvate dehydrogenase E1 component subunit alpha, mitochondrial [OS=Saccharomyces cerevisiae S288c]	85692 5	PDA1; YER178 W	cell growth;metabolic process	mitochondrion;organelle lumen	catalytic activity	21.63 8	29.47	1.36
P00830	ATP synthase subunit beta, mitochondrial [OS=Saccharomyces cerevisiae S288c]	85358 5	ATP2; YJR121 W	metabolic process;transport	cytosol;membrane;mitochondrion	catalytic activity;nucleotide binding;protein binding;transporte r activity	78.43 3	106.9 78	1.36
P10507	mitochondrial-processing peptidase subunit beta [OS=Saccharomyces cerevisiae S288c]	85086 0	MAS1; YLR163 C	metabolic process;transport	mitochondrion;organelle lumen	catalytic activity;metal ion binding;protein binding	1.371	1.873	1.37
P00044	Cytochrome c iso-1 [OS=Saccharomyces cerevisiae S288c]	85350 7	CYC1; YJR048 W	metabolic process;transport	mitochondrion	metal ion binding;protein binding	9	12.33 5	1.37
P07275	Delta-1-pyrroline-5-carboxylate dehydrogenase, mitochondrial [OS=Saccharomyces cerevisiae S288c]	85643 2	PUT2; YHR037 W	metabolic process	membrane;mitochondrion;organelle lumen	catalytic activity	6.055	8.326	1.38
P35180	mitochondrial import receptor subunit Tom20 [OS=Saccharomyces cerevisiae S288c]	85297 3	TOM20; YGR082 W	cell organization and biogenesis;transport	membrane;mitochondrion	protein binding;transporte r activity	1.031	1.424	1.38
P38705	Serine--tRNA ligase, mitochondrial [OS=Saccharomyces cerevisiae S288c]	85640 2	DIA4; YHR011 W	metabolic process	cytoplasm;mitochondrion;organelle lumen	catalytic activity;nucleotide binding	0.233	0.322	1.38
P07236	Threonine--tRNA ligase, mitochondrial [OS=Saccharomyces cerevisiae S288c]	85364 0	MST1; YKL194 C	metabolic process	cytoplasm;mitochondrion;organelle lumen	catalytic activity;nucleotide binding;RNA binding	0.233	0.322	1.38
P20967	2-oxoglutarate dehydrogenase, mitochondrial [OS=Saccharomyces cerevisiae S288c]	85468 1	KGD1; YIL125 W	metabolic process	cytosol;mitochondrion;organelle lumen	catalytic activity	11.42 6	15.84 3	1.39
Q04728	Arginine biosynthesis bifunctional protein ArgJ, mitochondrial [OS=Saccharomyces cerevisiae S288c]	85508 4	ARG7; YMR06 2C	metabolic process	mitochondrion;organelle lumen	catalytic activity	0.63	0.874	1.39
P03879	Intron-encoded RNA maturase bI4 [OS=Saccharomyces cerevisiae S288c]	85458 2	BI4; Q0120	metabolic process	membrane;mitochondrion	catalytic activity;RNA binding	0.28	0.389	1.39
P17505	Malate dehydrogenase, mitochondrial [OS=Saccharomyces cerevisiae S288c]	85377 7	MDH1; YKL085 W	metabolic process	mitochondrion;organelle lumen	catalytic activity;protein binding;RNA binding	56.79 7	79.30 9	1.40
P07266	Mitochondrial RNA-splicing protein MRS1 [OS=Saccharomyces cerevisiae S288c]	85483 9	MRS1; YIR021 W	cell organization and biogenesis;metabolic process	mitochondrion;organelle lumen	catalytic activity;DNA binding;RNA binding	0.304	0.425	1.40
Q00055	Glycerol-3-phosphate dehydrogenase [NAD(+)] 1 [OS=Saccharomyces cerevisiae S288c]	85153 9	GPD1; YDL022 W	cell organization and biogenesis;cellular homeostasis;metabolic process;transport	cytoplasm;cytosol;mitochondrion;nucleus	catalytic activity;nucleotide binding;protein binding	0.318	0.445	1.40
P22135	Protein ATP12, mitochondrial [OS=Saccharomyces cerevisiae S288c]	85325 9	ATP12; YJL180C	cell organization and biogenesis	mitochondrion	protein binding	0.334	0.468	1.40
Q05648	MIOREX complex component 10 [OS=Saccharomyces cerevisiae S288c]	85187 6	YDR282 C; MRX10; YDR282 C		membrane;mitochondrion		0.35	0.492	1.41
P38088	Glycine--tRNA ligase 1, mitochondrial [OS=Saccharomyces cerevisiae S288c]			metabolic process	cytoplasm;mitochondrion;organelle lumen	catalytic activity;nucleotide binding;protein binding	0.616	0.866	1.41
P53170	[Pyruvate dehydrogenase (acetyl-transferring)] kinase 2, mitochondrial [OS=Saccharomyces cerevisiae S288c]	85282 1	PKP2; YGL059 W	metabolic process;regulation of biological process	mitochondrion;organelle lumen	catalytic activity;nucleotide binding;protein binding	0.778	1.096	1.41
P06208-1	2-isopropylmalate synthase [OS=Saccharomyces cerevisiae S288c]	85561 9	LEU4; YNL104 C	metabolic process	cytoplasm;mitochondrion	catalytic activity;protein binding	5.236	7.377	1.41
Q06892	NADH kinase POS5, mitochondrial [OS=Saccharomyces cerevisiae S288c]	85591 3	POS5; YPL188 W	metabolic process;response to stimulus	mitochondrion;organelle lumen	catalytic activity;nucleotide binding	1.31	1.848	1.41
P40452	Cytochrome c oxidase assembly factor 1 [OS=Saccharomyces cerevisiae S288c]	85464 9	COA1; YIL157C	cell organization and biogenesis	membrane;mitochondrion	protein binding	1.31	1.848	1.41
P40495	Homoisocitrate dehydrogenase, mitochondrial [OS=Saccharomyces cerevisiae S288c]	85471 4	LYS12; YIL094C	metabolic process	mitochondrion	catalytic activity;metal ion binding;nucleotide binding	4.109	5.813	1.41

P38122	3-methyl-2-oxobutanoate hydroxymethyltransferase [OS=Saccharomyces cerevisiae S288c]	852474	ECM31; YBR176W	metabolic process	mitochondrion	catalytic activity	0.413	0.585	1.42
Q03976	37S ribosomal protein S24, mitochondrial [OS=Saccharomyces cerevisiae S288c]	851755	RSM24; YDR175C	cell organization and biogenesis;metabolic process	mitochondrion;ribosome	structural molecule activity	0.905	1.291	1.43
Q08245	protein ZEO1 [OS=Saccharomyces cerevisiae S288c]	854040	ZEO1; YOL109W	cell organization and biogenesis	membrane;mitochondrion	protein binding	1.512	2.162	1.43
Q00711	Succinate dehydrogenase [ubiquinone] flavoprotein subunit, mitochondrial [OS=Saccharomyces cerevisiae S288c]	853709	SDH1; YKL148C	metabolic process;transport	membrane;mitochondrion	catalytic activity;nucleotide binding;protein binding	12.242	17.548	1.43
P38323	ATP-dependent clpX-like chaperone, mitochondrial [OS=Saccharomyces cerevisiae S288c]	852528	MCX1; YBR227C	metabolic process	membrane;mitochondrion;organelle lumen	catalytic activity;nucleotide binding	0.501	0.719	1.44
Q05892	MIOREX complex component 2 [OS=Saccharomyces cerevisiae S288c]	850997	YLR290C; COQ11; YLR290C	metabolic process	mitochondrion	catalytic activity	3.217	4.623	1.44
P38910	10 kDa heat shock protein, mitochondrial [OS=Saccharomyces cerevisiae S288c]	854185	HSP10; YOR020C	cell organization and biogenesis;metabolic process;response to stimulus;transport	cytoplasm;mitochondrion;organelle lumen	metal ion binding;nucleotide binding;protein binding	3.217	4.623	1.44
Q01852	Mitochondrial import inner membrane translocase subunit TIM44 [OS=Saccharomyces cerevisiae S288c]	854790	TIM44; YIL022W	transport	membrane;mitochondrion	nucleotide binding;protein binding	3.758	5.404	1.44
P32316	acetyl-CoA hydrolase [OS=Saccharomyces cerevisiae S288c]	852266	ACH1; YBL015W	metabolic process	cytoplasm;cytosol;mitochondrion	catalytic activity	15.298	22.101	1.44
Q02784	Monothiol glutaredoxin-5, mitochondrial [OS=Saccharomyces cerevisiae S288c]	856048	GRX5; YPL059W	cell organization and biogenesis;cellular homeostasis;metabolic process;regulation of biological process;response to stimulus	mitochondrion;organelle lumen	catalytic activity;metal ion binding	1.783	2.594	1.45
P38341	MICOS complex subunit MIC12 [OS=Saccharomyces cerevisiae S288c]	852566	AIM5; MIC12; YBR262C	cell organization and biogenesis	membrane;mitochondrion		1.783	2.594	1.45
P35996	54S ribosomal protein L38, mitochondrial [OS=Saccharomyces cerevisiae S288c]	853684	MRPL38; YKL170W	cell organization and biogenesis;metabolic process	mitochondrion;ribosome	RNA binding;structural molecule activity	1.783	2.594	1.45
Q6Q560	Protein isd11 [OS=Saccharomyces cerevisiae S288c]	856774	ISD11; YER048W-A	cell organization and biogenesis;metabolic process	mitochondrion;organelle lumen	catalytic activity;protein binding	1.783	2.594	1.45
P37292	Serine hydroxymethyltransferase, mitochondrial [OS=Saccharomyces cerevisiae S288c]	852565	SHM1; YBR263W	metabolic process	mitochondrion	catalytic activity	9	13.175	1.46
Q03201	37S ribosomal protein S10, mitochondrial [OS=Saccharomyces cerevisiae S288c]	851611	RSM10; YDR041W	cell organization and biogenesis;metabolic process	mitochondrion;ribosome	structural molecule activity	0.701	1.031	1.47
P52893	probable alanine aminotransferase, mitochondrial [OS=Saccharomyces cerevisiae S288c]	850778	ALT1; YLR089C	metabolic process	mitochondrion;organelle lumen	catalytic activity	1.102	1.626	1.48
P53598	Succinyl-CoA ligase [ADP-forming] subunit alpha, mitochondrial [OS=Saccharomyces cerevisiae S288c]	854310	LSC1; YOR142W	metabolic process	cytosol;mitochondrion	catalytic activity;nucleotide binding	11.743	17.33	1.48
P22136	ATPase expression protein 2, mitochondrial [OS=Saccharomyces cerevisiae S288c]	855325	AEP2; YMR282C	metabolic process;regulation of biological process	mitochondrion	RNA binding	0.292	0.431	1.48
P38523	GrpE protein homolog, mitochondrial [OS=Saccharomyces cerevisiae S288c]	854407	MGE1; YOR232W	metabolic process;regulation of biological process;transport	membrane;mitochondrion;organelle lumen	enzyme regulator activity;nucleotide binding;protein binding	1.336	1.976	1.48
Q08230	Succinate dehydrogenase assembly factor 2, mitochondrial [OS=Saccharomyces cerevisiae S288c]	854083	EMI5; SDH5; YOL071W	cell differentiation;cell organization and biogenesis;metabolic process;regulation of biological process	mitochondrion;organelle lumen	protein binding	0.778	1.154	1.48
P09440	C-1-tetrahydrofolate synthase, mitochondrial [OS=Saccharomyces cerevisiae S288c]	852378	MIS1; YBR084W	metabolic process	cytosol;mitochondrion	catalytic activity;nucleotide binding	1.049	1.569	1.50
P40053	Altered inheritance of mitochondria protein 9, mitochondrial [OS=Saccharomyces cerevisiae S288c]	856813	AIM9; YER080W		mitochondrion		4.555	6.827	1.50
P47127	Altered inheritance of mitochondria protein 24, mitochondrial [OS=Saccharomyces cerevisiae S288c]	853543	AIM24; YJR080C	cell organization and biogenesis	mitochondrion		2.857	4.298	1.50
P25349	Flavoprotein-like protein YCP4 [OS=Saccharomyces cerevisiae S288c]	850360	YCP4; YCR004C	metabolic process;regulation of biological process	cytoplasm;membrane;mitochondrion	catalytic activity;nucleotide binding;protein binding	9	13.678	1.52

P36046	Mitochondrial intermembrane space import and assembly protein 40 [OS=Saccharomyces cerevisiae S288c]	853639	MIA40; YKL195W	cellular component movement;metabolic process;response to stimulus;transport	membrane;mitochondrion	catalytic activity;protein binding;transporter activity	3.262	4.995	1.53
P19516	Cytochrome c oxidase assembly protein COX11, mitochondrial [OS=Saccharomyces cerevisiae S288c]	855971	COX11; YPL132W	cell organization and biogenesis;metabolic process	membrane;mitochondrion;ribosome	metal ion binding	1.069	1.637	1.53
P36112	MICOS complex subunit Mic60 [OS=Saccharomyces cerevisiae S288c]	853886	FCJ1; MIC60; YKR016W	cell organization and biogenesis;transport	membrane;mitochondrion		15.379	23.709	1.54
P04819	DNA ligase 1 [OS=Saccharomyces cerevisiae S288c]	851391	CDC9; YDL164C	cell division;cell organization and biogenesis;metabolic process;response to stimulus	cytoplasm;mitochondrion;nucleus	catalytic activity;DNA binding;metal ion binding;nucleotide binding	0.11	0.17	1.55
P32493	ATPase expression protein 1, mitochondrial [OS=Saccharomyces cerevisiae S288c]	855086	AEP1; YMR064W	regulation of biological process	mitochondrion	translation regulator activity	0.15	0.233	1.55
P07806	Valine--tRNA ligase, mitochondrial [OS=Saccharomyces cerevisiae S288c]	852986	VAS1; YGR094W	metabolic process;regulation of biological process	cytoplasm;cytosol;mitochondrion	catalytic activity;nucleotide binding	0.616	0.957	1.55
P41921	glutathione reductase [OS=Saccharomyces cerevisiae S288c]	856014	GLR1; YPL091W	cellular homeostasis;metabolic process;regulation of biological process;response to stimulus	cytoplasm;cytosol;mitochondrion;nucleus	antioxidant activity;catalytic activity;nucleotide binding	0.155	0.241	1.55
P25345	Asparagine--tRNA ligase, mitochondrial [OS=Saccharomyces cerevisiae S288c]	850388	SLM5; YCR024C	metabolic process	cytoplasm;mitochondrion;organelle lumen	catalytic activity;nucleotide binding	0.172	0.269	1.56
P00890	citrate synthase, mitochondrial [OS=Saccharomyces cerevisiae S288c]	855732	CIT1; YNR001C	metabolic process;transport	cytosol;mitochondrion;organelle lumen	catalytic activity	9	14.086	1.57
P19262	Dihydrolipoyllysine-residue succinyltransferase component of 2-oxoglutarate dehydrogenase complex, mitochondrial [OS=Saccharomyces cerevisiae S288c]	851726	KGD2; YDR148C	cell organization and biogenesis;metabolic process	mitochondrion	catalytic activity;protein binding	6.406	10.053	1.57
P32904	54S ribosomal protein L6, mitochondrial [OS=Saccharomyces cerevisiae S288c]	856552	MRPL6; YHR147C	cell organization and biogenesis;metabolic process	mitochondrion;ribosome	RNA binding;structural molecule activity	1.371	2.162	1.58
P36147	Presequence translocated-associated motor subunit pam17, mitochondrial [OS=Saccharomyces cerevisiae S288c]	853939	PAM17; YKR065C	transport	membrane;mitochondrion		1.371	2.162	1.58
P53736	Uncharacterized protein YNR040W [OS=Saccharomyces cerevisiae S288c]	855776	YNR040W; YNR040W		mitochondrion		0.73	1.154	1.58
Q12251	Uncharacterized mitochondrial carrier YPR011C [OS=Saccharomyces cerevisiae S288c]	856121	YPR011C; YPR011C	metabolic process;transport	membrane;mitochondrion	structural molecule activity;transporter activity	0.245	0.389	1.59
P49017	2-methoxy-6-polyprenyl-1,4-benzoquinol methylase, mitochondrial [OS=Saccharomyces cerevisiae S288c]	854930	COQ5; YML110C	metabolic process	membrane;mitochondrion;organelle lumen	catalytic activity	0.245	0.389	1.59
Q01519	Cytochrome c oxidase subunit 6b [OS=Saccharomyces cerevisiae S288c]	850727	COX12; YLR038C	cell organization and biogenesis;metabolic process;transport	mitochondrion	catalytic activity;transporter activity	3.642	5.813	1.60
P36525	54S ribosomal protein L24, mitochondrial [OS=Saccharomyces cerevisiae S288c]	855231	MRPL24; YMR193W	cell organization and biogenesis;metabolic process	mitochondrion;ribosome	RNA binding;structural molecule activity	1.512	2.415	1.60
P36519	54S ribosomal protein L7, mitochondrial [OS=Saccharomyces cerevisiae S288c]	851823	MRPL7; YDR237W	cell organization and biogenesis;metabolic process	mitochondrion;ribosome	RNA binding;structural molecule activity	0.292	0.468	1.60
P04803	Tryptophan--tRNA ligase, mitochondrial [OS=Saccharomyces cerevisiae S288c]	851861	MSW1; YDR268W	metabolic process	cytoplasm;mitochondrion;organelle lumen	catalytic activity;nucleotide binding	0.292	0.468	1.60
P28625	Protein YIM1 [OS=Saccharomyces cerevisiae S288c]	855183	YIM1; YMR152W	metabolic process;response to stimulus	cytoplasm;endoplasmic reticulum;mitochondrion	catalytic activity;metal ion binding	0.833	1.336	1.60
P47045	mitochondrial import inner membrane translocase subunit TIM54 [OS=Saccharomyces cerevisiae S288c]	853392	TIM54; YJL054W	cell organization and biogenesis;transport	cytosol;membrane;mitochondrion	transporter activity	0.655	1.054	1.61
P31334	54S ribosomal protein L9, mitochondrial [OS=Saccharomyces cerevisiae S288c]	853135	MRPL9; YGR220C	cell organization and biogenesis;metabolic process	mitochondrion;ribosome	structural molecule activity	0.311	0.501	1.61
P36523	54S ribosomal protein L15, mitochondrial [OS=Saccharomyces cerevisiae S288c]	851022	MRPL15; YLR312W-A	cell organization and biogenesis;metabolic process	mitochondrion;ribosome	catalytic activity;RNA binding;structural molecule activity	0.334	0.54	1.62
Q02608	37S ribosomal protein S16, mitochondrial [OS=Saccharomyces cerevisiae S288c]	856094	MRPS16; YPL013C	cell organization and biogenesis;metabolic process	mitochondrion;ribosome	structural molecule activity	1.683	2.728	1.62

Q12029	Probable mitochondrial transport protein fsf1 [OS=Saccharomyces cerevisiae S288c]	854445	FSF1; YOR271C	transport	membrane;mitochondrion	transporter activity	2.36	3.833	1.62
Q06630	Mitochondrial homologous recombination protein 1 [OS=Saccharomyces cerevisiae S288c]	851890	MHR1; YDR296W	cell organization and biogenesis;metabolic process;regulation of biological process;response to stimulus	mitochondrion;nucleus	catalytic activity;DNA binding;structural molecule activity	0.359	0.585	1.63
P15179	Aspartate--tRNA ligase, mitochondrial [OS=Saccharomyces cerevisiae S288c]	856000	MSD1; YPL104W	metabolic process	cytoplasm;mitochondrion;organelle lumen	catalytic activity;nucleotide binding	0.379	0.619	1.63
P32839	Mitochondrial chaperone BCS1 [OS=Saccharomyces cerevisiae S288c]	851981	BCS1; YDR375C	cell organization and biogenesis;metabolic process;response to stimulus;transport	cytosol;membrane;mitochondrion	catalytic activity;nucleotide binding;transporter activity	0.425	0.701	1.65
P34222	Peptidyl-tRNA hydrolase 2 [OS=Saccharomyces cerevisiae S288c]	852223	PTH2; YBL057C	regulation of biological process	cytoplasm;cytosol;membrane;mitochondrion	catalytic activity;protein binding	0.425	0.701	1.65
Q04487	Mitochondrial inner membrane protein SHH3 [OS=Saccharomyces cerevisiae S288c]	855145	SHH3; YMR118C	metabolic process	membrane;mitochondrion	catalytic activity;metal ion binding	0.425	0.701	1.65
P47039	Probable kynurenine--oxoglutarate transaminase BNA3 [OS=Saccharomyces cerevisiae S288c]	853386	BNA3; YJL060W	metabolic process;regulation of biological process	cytoplasm;mitochondrion	catalytic activity	0.468	0.778	1.66
P40858	54S ribosomal protein L49, mitochondrial [OS=Saccharomyces cerevisiae S288c]	853349	MRPL49; YJL096W	cell organization and biogenesis;metabolic process	mitochondrion;ribosome	RNA binding;structural molecule activity	0.468	0.778	1.66
P40515	Mitochondria fission 1 protein [OS=Saccharomyces cerevisiae S288c]	854745	FIS1; YIL065C	cell death;cell organization and biogenesis;regulation of biological process	membrane;mitochondrion	protein binding	0.468	0.778	1.66
Q03020	iron sulfur cluster assembly protein 1, mitochondrial [OS=Saccharomyces cerevisiae S288c]	855968	ISU1; YPL135W	cell organization and biogenesis;cellular homeostasis;metabolic process	cytoplasm;mitochondrion;organelle lumen	catalytic activity;metal ion binding;protein binding	0.468	0.778	1.66
P53193	J-type co-chaperone JAC1, mitochondrial [OS=Saccharomyces cerevisiae S288c]	852866	JAC1; YGL018C	cell organization and biogenesis;metabolic process;transport	membrane;mitochondrion;organelle lumen	protein binding	0.468	0.778	1.66
P36528	54S ribosomal protein L17, mitochondrial [OS=Saccharomyces cerevisiae S288c]	855469	MRPL17; YNL252C	cell organization and biogenesis;metabolic process	mitochondrion;ribosome	structural molecule activity	0.52	0.874	1.68
P53305	Mitochondrial 37S ribosomal protein S27 [OS=Saccharomyces cerevisiae S288c]	853129	RSM27; YGR215W	cell organization and biogenesis;metabolic process	mitochondrion;ribosome	structural molecule activity	2.162	3.642	1.68
P20084	54S ribosomal protein L33, mitochondrial [OS=Saccharomyces cerevisiae S288c]	855330	MRPL33; YMR286W	cell organization and biogenesis;metabolic process	mitochondrion;ribosome	structural molecule activity	2.162	3.642	1.68
P22354	54S ribosomal protein L20, mitochondrial [OS=Saccharomyces cerevisiae S288c]	853960	MRPL20; YKR085C	cell organization and biogenesis;metabolic process	mitochondrion;ribosome	structural molecule activity	1.276	2.162	1.69
P40185	Protein mmf1, mitochondrial [OS=Saccharomyces cerevisiae S288c]	854760	MMF1; YIL051C	cell organization and biogenesis;metabolic process	mitochondrion;organelle lumen		5.31	9	1.69
P36526	54S ribosomal protein L27, mitochondrial [OS=Saccharomyces cerevisiae S288c]	852585	MRPL27; YBR282W	cell organization and biogenesis;metabolic process	mitochondrion;ribosome	structural molecule activity	0.585	0.995	1.70
P40033	37S ribosomal protein rsm18, mitochondrial [OS=Saccharomyces cerevisiae S288c]	856776	RSM18; YER050C	cell organization and biogenesis;metabolic process	mitochondrion;ribosome	structural molecule activity	0.585	0.995	1.70
Q06493	LETM1 domain-containing protein YLH47, mitochondrial [OS=Saccharomyces cerevisiae S288c]	856243	YLH47; YPR125W	cell organization and biogenesis;transport	membrane;mitochondrion		0.61	1.043	1.71
P40961	prohibitin-1 [OS=Saccharomyces cerevisiae S288c]	853033	PHB1; YGR132C	cell organization and biogenesis;metabolic process;regulation of biological process	membrane;mitochondrion	protein binding	6.848	11.743	1.71
Q12305	Thiosulfate sulfurtransferase RDL1, mitochondrial [OS=Saccharomyces cerevisiae S288c]	854459	RDL1; YOR285W		endoplasmic reticulum;membrane;mitochondrion	catalytic activity	0.668	1.154	1.73
P53220	Mitochondrial import inner membrane translocase subunit TIM21 [OS=Saccharomyces cerevisiae S288c]	852921	TIM21; YGR033C	cell organization and biogenesis;transport	membrane;mitochondrion	protein binding	0.719	1.254	1.74
P34227	Mitochondrial peroxiredoxin PRX1 [OS=Saccharomyces cerevisiae S288c]	852215	PRX1; YBL064C	cellular homeostasis;metabolic process;regulation of biological process;response to stimulus	mitochondrion	antioxidant activity;catalytic activity	5.158	9	1.74
P07342	Acetolactate synthase catalytic subunit, mitochondrial [OS=Saccharomyces cerevisiae S288c]	855135	ILV2; YMR108W	metabolic process	mitochondrion	catalytic activity;metal ion binding;nucleotide binding	4.223	7.377	1.75
P42900	Sigma-like sequence protein 1, mitochondrial [OS=Saccharomyces cerevisiae S288c]	850830	SLS1; YLR139C	cell organization and biogenesis;metabolic process	membrane;mitochondrion	protein binding	0.194	0.343	1.77
P11914	Mitochondrial-processing peptidase subunit alpha [OS=Saccharomyces	856419	MAS2; YHR024	metabolic process;transport	membrane;mitochondrion;organelle lumen	catalytic activity;metal ion	1.291	2.311	1.79

	cerevisiae S288c]		C			binding;protein binding			
P17558	37S ribosomal protein PET123, mitochondrial [OS=Saccharomyces cerevisiae S288c]	854329	PET123; YOR158W	cell organization and biogenesis;metabolic process	mitochondrion;ribosome	structural molecule activity	1.448	2.594	1.79
P18900	hexaprenyl pyrophosphate synthase, mitochondrial [OS=Saccharomyces cerevisiae S288c]	852288	COQ1; YBR003W	metabolic process	membrane;mitochondrion	catalytic activity;metal ion binding;protein binding	0.468	0.848	1.81
P09950	5-aminolevulinate synthase, mitochondrial [OS=Saccharomyces cerevisiae S288c]	851818	HEM1; YDR232W	metabolic process	mitochondrion;organelle lumen	catalytic activity	0.28	0.509	1.82
Q01532-1	Cysteine proteinase 1, mitochondrial [OS=Saccharomyces cerevisiae S288c]		MCY1	metabolic process;regulation of biological process;response to stimulus	cytoplasm;mitochondrion	catalytic activity;DNA binding;RNA binding	0.28	0.509	1.82
P46367	Potassium-activated aldehyde dehydrogenase, mitochondrial [OS=Saccharomyces cerevisiae S288c]	854556	ALD4; YOR374W	metabolic process	mitochondrion;organelle lumen	catalytic activity	47.497	86.671	1.82
P35191	DnaJ homolog 1, mitochondrial [OS=Saccharomyces cerevisiae S288c]	850530	MDJ1; YFL016C	cell organization and biogenesis;metabolic process;regulation of biological process;response to stimulus	mitochondrion;organelle lumen	enzyme regulator activity;metal ion binding;nucleotide binding;protein binding	3.037	5.579	1.84
Q04599	54S ribosomal protein L1, mitochondrial [OS=Saccharomyces cerevisiae S288c]	851694	MRPL1; YDR116C	cell organization and biogenesis;metabolic process	mitochondrion;ribosome	RNA binding;structural molecule activity	1.069	1.976	1.85
P37298	Succinate dehydrogenase [ubiquinone] cytochrome b small subunit, mitochondrial [OS=Saccharomyces cerevisiae S288c]	851758	SDH4; YDR178W	metabolic process;transport	membrane;mitochondrion	catalytic activity;metal ion binding	3.642	6.743	1.85
Q12320	Hydroxyacylglutathione hydrolase, mitochondrial [OS=Saccharomyces cerevisiae S288c]	854205	GLO4; YOR040W	metabolic process	mitochondrion;organelle lumen	catalytic activity;metal ion binding	0.369	0.688	1.86
P32902	37S ribosomal protein MRP4, mitochondrial [OS=Saccharomyces cerevisiae S288c]	856384	MRP4; YHL004W	cell organization and biogenesis;metabolic process	mitochondrion;ribosome	structural molecule activity	0.616	1.154	1.87
P00425	Cytochrome c oxidase polypeptide 5B, mitochondrial [OS=Saccharomyces cerevisiae S288c]	854695	COX5B; YIL111W	metabolic process;transport	membrane;mitochondrion	catalytic activity;transporter activity	1.31	2.511	1.92
P19414	Aconitate hydratase, mitochondrial [OS=Saccharomyces cerevisiae S288c]	851013	ACO1; YLR304C	cell organization and biogenesis;metabolic process;transport	cytoplasm;cytosol;mitochondrion;organelle lumen	catalytic activity;DNA binding;metal ion binding	48.619	94.118	1.94
P36534	54S ribosomal protein L40, mitochondrial [OS=Saccharomyces cerevisiae S288c]	855930	MRPL40; YPL173W	cell organization and biogenesis;metabolic process	mitochondrion;ribosome	protein binding;structural molecule activity	0.778	1.512	1.94
P25605	Acetolactate synthase small subunit, mitochondrial [OS=Saccharomyces cerevisiae S288c]	850348	ILV6; YCL009C	metabolic process;regulation of biological process	mitochondrion	catalytic activity;enzyme regulator activity	6.848	13.384	1.95
P36151	Uncharacterized protein YKR070W [OS=Saccharomyces cerevisiae S288c]	853944	YKR070W; YKR070W	metabolic process	mitochondrion		0.833	1.637	1.97
P40364	Mitochondrial peculiar membrane protein 1 [OS=Saccharomyces cerevisiae S288c]	853379	MPM1; YJL066C		membrane;mitochondrion		1.512	2.981	1.97
P33303	Succinate/fumarate mitochondrial transporter [OS=Saccharomyces cerevisiae S288c]	853558	SFC1; YJR095W	metabolic process;transport	membrane;mitochondrion	structural molecule activity;transporter activity	9	17.957	2.00
P32611	54S ribosomal protein RML2, mitochondrial [OS=Saccharomyces cerevisiae S288c]	856660	RML2; YEL050C	cell organization and biogenesis;metabolic process	mitochondrion;ribosome	catalytic activity;RNA binding;structural molecule activity	0.638	1.276	2.00
P09624	Dihydrolipoyl dehydrogenase, mitochondrial [OS=Saccharomyces cerevisiae S288c]	850527	LPD1; YFL018C	cellular homeostasis;metabolic process;regulation of biological process	mitochondrion;organelle lumen	catalytic activity;nucleotide binding	21.539	43.367	2.01
Q02950	37S ribosomal protein MRP51, mitochondrial [OS=Saccharomyces cerevisiae S288c]	855985	MRP51; YPL118W	cell organization and biogenesis;metabolic process	mitochondrion;ribosome	structural molecule activity	0.668	1.346	2.01
Q06236	Mitochondrial inner membrane protein SHH4 [OS=Saccharomyces cerevisiae S288c]	850861	SHH4; YLR164W	metabolic process	membrane;mitochondrion	catalytic activity;metal ion binding	3.217	6.499	2.02
P21801	Succinate dehydrogenase [ubiquinone] iron-sulfur subunit, mitochondrial [OS=Saccharomyces cerevisiae S288c]	850685	SDH2; YLL041C	metabolic process;transport	membrane;mitochondrion	catalytic activity;metal ion binding	18.684	37.747	2.02
P48237	Mitochondrial group I intron splicing factor CCM1 [OS=Saccharomyces cerevisiae S288c]	853053	CCM1; YGR150C	cell organization and biogenesis;metabolic process	mitochondrion	RNA binding	0.044	0.089	2.02
P51998	54S ribosomal protein yml6, mitochondrial [OS=Saccharomyces cerevisiae S288c]	854983	YML6; YML025C	cell organization and biogenesis;metabolic process	mitochondrion;ribosome	RNA binding;structural molecule activity	1.154	2.34	2.03

P47158	Putative transferase CAF17, mitochondrial [OS=Saccharomyces cerevisiae S288c]	853586	IBA57; YJR122W	cell organization and biogenesis;metabolic process	mitochondrion;organelle lumen	catalytic activity	0.075	0.155	2.07
P38860	GTPase MTG2, mitochondrial [OS=Saccharomyces cerevisiae S288c]	856573	MTG2; YHR168W	metabolic process;transport	membrane;mitochondrion	catalytic activity;metal ion binding;nucleotide binding;transporter activity	0.07	0.145	2.07
P40051	Intermediate cleaving peptidase 55 [OS=Saccharomyces cerevisiae S288c]	856811	ICP55; YER078C	metabolic process;regulation of biological process	membrane;mitochondrion;nucleus	catalytic activity;metal ion binding	0.077	0.16	2.08
P07263-1	Histidine--tRNA ligase, mitochondrial [OS=Saccharomyces cerevisiae S288c]	856145	HTS1; YPR033C	cell organization and biogenesis;metabolic process	cytoplasm;mitochondrion	catalytic activity;nucleotide binding;RNA binding	0.064	0.133	2.08
Q08645	folylpolyglutamate synthase [OS=Saccharomyces cerevisiae S288c]	854415	MET7; YOR241W	metabolic process	cytoplasm;endoplasmic reticulum;membrane;mitochondrion;organelle lumen	catalytic activity;nucleotide binding	0.072	0.15	2.08
P40530	[Pyruvate dehydrogenase (acetyl-transferring)] kinase 1, mitochondrial [OS=Saccharomyces cerevisiae S288c]	854769	PKP1; YIL042C	metabolic process	mitochondrion;organelle lumen	catalytic activity;nucleotide binding;protein binding	0.551	1.154	2.09
P14905	cytochrome B translational activator protein CBS2 [OS=Saccharomyces cerevisiae S288c]	851778	CBS2; YDR197W	metabolic process;regulation of biological process	mitochondrion;ribosome	catalytic activity;nucleotide binding;translation regulator activity	0.096	0.202	2.10
P36527	54S ribosomal protein L28, mitochondrial [OS=Saccharomyces cerevisiae S288c]	852073	MRPL28; YDR462W	cell organization and biogenesis;metabolic process	mitochondrion;ribosome	structural molecule activity	0.874	1.848	2.11
Q12204	Probable phospholipase YOR022C, mitochondrial [OS=Saccharomyces cerevisiae S288c]	854187	YOR022C; YOR022C	metabolic process	mitochondrion	catalytic activity;metal ion binding	0.11	0.233	2.12
P24720	Protein MNE1 [OS=Saccharomyces cerevisiae S288c]	854532	MNE1; YOR350C	metabolic process	cytoplasm;mitochondrion;organelle lumen		0.125	0.266	2.13
P38816	thioredoxin reductase 2, mitochondrial [OS=Saccharomyces cerevisiae S288c]	856506	TRR2; YHR106W	cellular homeostasis;metabolic process;regulation of biological process;response to stimulus	cytoplasm;mitochondrion	antioxidant activity;catalytic activity	0.136	0.292	2.15
Q12374	Nuclear control of ATPase protein 2 [OS=Saccharomyces cerevisiae S288c]	856278	NCA2; YPR155C	metabolic process	membrane;mitochondrion		0.468	1.021	2.18
P28241	Isocitrate dehydrogenase [NAD] subunit 2, mitochondrial [OS=Saccharomyces cerevisiae S288c]	854303	IDH2; YOR136W	metabolic process	mitochondrion;organelle lumen	catalytic activity;metal ion binding;nucleotide binding;protein binding;RNA binding	3.642	7.962	2.19
P34231	Uncharacterized protein YKL187C [OS=Saccharomyces cerevisiae S288c]	853648	FAT3; YKL187C; YKL187C	cell organization and biogenesis;transport	membrane;mitochondrion		0.194	0.425	2.19
P19955	37S ribosomal protein YMR-31, mitochondrial [OS=Saccharomyces cerevisiae S288c]	850610	YMR31; YFR049W	cell organization and biogenesis;metabolic process	mitochondrion;ribosome	catalytic activity;protein binding;structural molecule activity	26.826	58.948	2.20
P40491	ATP synthase assembly factor fmc1, mitochondrial [OS=Saccharomyces cerevisiae S288c]	854709	FMC1; YIL098C	cell communication;cell organization and biogenesis;metabolic process;response to stimulus	mitochondrion;organelle lumen		0.212	0.468	2.21
P23180	Probable oxidoreductase AIM17 [OS=Saccharomyces cerevisiae S288c]	856365	AIM17; YHL021C	cell organization and biogenesis;metabolic process	mitochondrion	catalytic activity;metal ion binding	4.623	10.22	2.21
Q07534	Solute carrier family 25 member 38 homolog [OS=Saccharomyces cerevisiae S288c]	851439	YDL119C; HEM25; YDL119C	metabolic process;transport	membrane;mitochondrion	structural molecule activity;transporter activity	0.245	0.551	2.25
P10849	mitochondrial transcription factor 2 [OS=Saccharomyces cerevisiae S288c]	851517	MTF2; YDL044C	metabolic process	mitochondrion;organelle lumen	protein binding;RNA binding	0.259	0.585	2.26
P39965	probable proline--tRNA ligase, mitochondrial [OS=Saccharomyces cerevisiae S288c]	856820	AIM10; YER087W	metabolic process	cytoplasm;mitochondrion	catalytic activity;nucleotide binding;RNA binding	0.266	0.604	2.27
P22353	54S ribosomal protein L8, mitochondrial [OS=Saccharomyces cerevisiae S288c]	853382	MRPL8; YJL063C	cell organization and biogenesis;metabolic process	mitochondrion;ribosome	structural molecule activity	0.848	1.929	2.27
P49334	Mitochondrial import receptor subunit tom22 [OS=Saccharomyces cerevisiae S288c]	855592	TOM22; YNL131W	cell organization and biogenesis;transport	membrane;mitochondrion	protein binding;transporter activity	0.292	0.668	2.29
P40496	37S ribosomal protein S25, mitochondrial [OS=Saccharomyces cerevisiae S288c]	854715	RSM25; YIL093C	cell organization and biogenesis;metabolic process	mitochondrion;ribosome	structural molecule activity	0.292	0.668	2.29
P32897	Mitochondrial import inner membrane translocase subunit tim23 [OS=Saccharomyces	855751	TIM23; YNR017W	transport	membrane;mitochondrion	protein binding;transporter activity	2.981	6.943	2.33

	cerevisiae S288c]								
P39952	Mitochondrial inner membrane protein OXA1 [OS=Saccharomyces cerevisiae S288c]	856898	OXA1; YER154W	cell organization and biogenesis;transport	membrane;mitochondrion	transporter activity	1.371	3.217	2.35
P53881	54S ribosomal protein L22, mitochondrial [OS=Saccharomyces cerevisiae S288c]	855544	MRPL22; YNL177C	cell organization and biogenesis;metabolic process	mitochondrion;ribosome	RNA binding;structural molecule activity	1.154	2.728	2.36
P39726	Glycine cleavage system H protein, mitochondrial [OS=Saccharomyces cerevisiae S288c]	851254	GCV3; YAL044C	metabolic process	mitochondrion	catalytic activity	4.995	11.915	2.39
P25348	54S ribosomal protein L32, mitochondrial [OS=Saccharomyces cerevisiae S288c]	850359	MRPL32; YCR003W	cell organization and biogenesis;metabolic process	membrane;mitochondrion;ribosome	RNA binding;structural molecule activity	0.389	0.931	2.39
Q04401	Succinate dehydrogenase assembly factor 3, mitochondrial [OS=Saccharomyces cerevisiae S288c]	852123	ACN9; SDH7; YDR511W	cell organization and biogenesis;regulation of biological process	mitochondrion;organelle lumen		0.389	0.931	2.39
Q12497	Protein FMP16, mitochondrial [OS=Saccharomyces cerevisiae S288c]	851642	FMP16; YDR070C		mitochondrion		0.389	0.931	2.39
P12687	54S ribosomal protein L2, mitochondrial [OS=Saccharomyces cerevisiae S288c]	855727	MRP7; YNL005C	cell organization and biogenesis;metabolic process	mitochondrion;ribosome	catalytic activity;RNA binding;structural molecule activity	1.565	3.806	2.43
P38958	Protein PET100, mitochondrial [OS=Saccharomyces cerevisiae S288c]	851651	PET100; YDR079W	cell organization and biogenesis	membrane;mitochondrion	protein binding	0.468	1.154	2.47
Q06011	Protein ECM19 [OS=Saccharomyces cerevisiae S288c]	851106	ECM19; YLR390W	cell organization and biogenesis	membrane;mitochondrion		0.468	1.154	2.47
P32445	Single-stranded DNA-binding protein RIM1, mitochondrial [OS=Saccharomyces cerevisiae S288c]	850395	RIM1; YCR028C-A	cell organization and biogenesis;metabolic process;regulation of biological process	mitochondrion	DNA binding	3.642	9	2.47
P81451	ATP synthase subunit K, mitochondrial [OS=Saccharomyces cerevisiae S288c]	854077	ATP19; YOL077W-A	cell organization and biogenesis;metabolic process;transport	membrane;mitochondrion	catalytic activity;transporter activity	3.642	9	2.47
P07255	Cytochrome c oxidase subunit 7A [OS=Saccharomyces cerevisiae S288c]	851492	COX9; YDL067C	metabolic process;transport	membrane;mitochondrion	catalytic activity;transporter activity	3.642	9	2.47
P53875	54S ribosomal protein L19, mitochondrial [OS=Saccharomyces cerevisiae S288c]	855536	MRPL19; YNL185C	cell organization and biogenesis;metabolic process	mitochondrion;ribosome	RNA binding;structural molecule activity	0.52	1.31	2.52
P36520	54S ribosomal protein L10, mitochondrial [OS=Saccharomyces cerevisiae S288c]	855436	MRPL10; YNL284C	cell organization and biogenesis;metabolic process	mitochondrion;ribosome	structural molecule activity	0.551	1.404	2.55
P38300	Inner membrane mitoribosome receptor MBA1, mitochondrial [OS=Saccharomyces cerevisiae S288c]	852483	MBA1; YBR185C	cell organization and biogenesis;regulation of biological process	membrane;mitochondrion		0.585	1.512	2.58
P02381	Ribosomal protein VAR1, mitochondrial [OS=Saccharomyces cerevisiae S288c]	854586	VAR1; Q0140	cell organization and biogenesis;metabolic process	mitochondrion;ribosome	structural molecule activity	0.688	1.848	2.69
Q03430	37S ribosomal protein RSM28, mitochondrial [OS=Saccharomyces cerevisiae S288c]	852105	RSM28; YDR494W	cell organization and biogenesis;metabolic process	mitochondrion;ribosome	structural molecule activity	1.462	3.962	2.71
P47015	Altered inheritance of mitochondria protein 23, mitochondrial [OS=Saccharomyces cerevisiae S288c]	853310	AIM23; YJL131C	metabolic process	mitochondrion	RNA binding	0.35	1.015	2.90
P38325	Mitochondrial outer membrane protein OM14 [OS=Saccharomyces cerevisiae S288c]	852531	OM14; YBR230C	transport	membrane;mitochondrion		6.197	18.307	2.95
Q01163	37S ribosomal protein S23, mitochondrial [OS=Saccharomyces cerevisiae S288c]	852748	RSM23; YGL129C	cell organization and biogenesis;metabolic process	mitochondrion;ribosome	catalytic activity;nucleotide binding;RNA binding;structural molecule activity	0.983	2.932	2.98
P08525	Cytochrome b-c1 complex subunit 8 [OS=Saccharomyces cerevisiae S288c]	853273	QCR8; YJL166W	metabolic process;transport	membrane;mitochondrion	catalytic activity;transporter activity	2.981	9	3.02
Q03713	Respiratory supercomplex factor 1, mitochondrial [OS=Saccharomyces cerevisiae S288c]	854978	RCF1; YML030W	cell organization and biogenesis	membrane;mitochondrion		2.162	6.943	3.21
P42847	37S ribosomal protein S18, mitochondrial [OS=Saccharomyces cerevisiae S288c]	855410	MRPS18; YNL306W	cell organization and biogenesis;metabolic process	mitochondrion;ribosome	RNA binding;structural molecule activity	0.54	1.738	3.22
P41911	Glycerol-3-phosphate dehydrogenase [NAD(+)] 2, mitochondrial [OS=Saccharomyces cerevisiae S288c]	854095	GPD2; YOL059W	metabolic process	cytoplasm;cytosol;mitochondrion	catalytic activity;nucleotide binding;protein binding	0.089	0.292	3.28
P25038	Translation initiation factor IF-2, mitochondrial [OS=Saccharomyces cerevisiae S288c]	854135	IFM1; YOL023W	cell organization and biogenesis;metabolic process	mitochondrion;nucleus	catalytic activity;nucleotide binding;RNA	0.11	0.369	3.35

						binding			
P46998	mitochondrial membrane protein FMP33 [OS=Saccharomyces cerevisiae S288c]	853279	FMP33; YJL161W		membrane;mitochondrion		1.371	4.623	3.37
P49954	probable hydrolase NIT3 [OS=Saccharomyces cerevisiae S288c]	851065	NIT3; YLR351C	metabolic process	cytoplasm;mitochondrion	catalytic activity	0.129	0.438	3.40
P38297	Mitofusin FZO1 [OS=Saccharomyces cerevisiae S288c]	852477	FZO1; YBR179C	cell organization and biogenesis	membrane;mitochondrion	catalytic activity;nucleotide binding;protein binding	0.142	0.49	3.45
P47150	37S ribosomal protein S7, mitochondrial [OS=Saccharomyces cerevisiae S288c]	853578	RSM7; YJR113C	cell organization and biogenesis;metabolic process	mitochondrion;ribosome	RNA binding;structural molecule activity	0.334	1.154	3.46
P38884	Altered inheritance of mitochondria protein 18, mitochondrial [OS=Saccharomyces cerevisiae S288c]	856605	AIM18; YHR198C		mitochondrion	catalytic activity	0.145	0.501	3.46
P08425	phenylalanine--tRNA ligase, mitochondrial [OS=Saccharomyces cerevisiae S288c]	856160	MSF1; YPR047W	metabolic process	cytoplasm;mitochondrion;organelle lumen	catalytic activity;metal ion binding;nucleotide binding;RNA binding	0.16	0.562	3.51
P53724	54S ribosomal protein L50, mitochondrial [OS=Saccharomyces cerevisiae S288c]	855756	MRPL50; YNR022C	cell organization and biogenesis;metabolic process	mitochondrion;ribosome	structural molecule activity	0.585	2.162	3.70
P21771	37S ribosomal protein S28, mitochondrial [OS=Saccharomyces cerevisiae S288c]	851937	MRPS28; YDR337W	cell organization and biogenesis;metabolic process	mitochondrion;ribosome	RNA binding;structural molecule activity	0.438	1.637	3.74
Q06090	54S ribosomal protein L51, mitochondrial [OS=Saccharomyces cerevisiae S288c]	856214	MRPL51; YPR100W	cell organization and biogenesis;metabolic process	mitochondrion;ribosome	RNA binding;structural molecule activity	0.292	1.154	3.95
P53157	Mitochondrial pyruvate carrier 1 [OS=Saccharomyces cerevisiae S288c]	852800	FMP37; MPC1; YGL080W	transport	membrane;mitochondrion	transporter activity	0.468	2.162	4.62
P25626	54S ribosomal protein IMG1, mitochondrial [OS=Saccharomyces cerevisiae S288c]	850413	IMG1; YCR046C	cell organization and biogenesis;metabolic process	mitochondrion;ribosome	structural molecule activity	1.031	4.878	4.73
P53166	ATP-dependent RNA helicase mrh4, mitochondrial [OS=Saccharomyces cerevisiae S288c]	852816	MRH4; YGL064C	cell organization and biogenesis;metabolic process	mitochondrion;organelle lumen	catalytic activity;nucleotide binding;RNA binding	0.225	1.106	4.92
P36531	54S ribosomal protein L36, mitochondrial [OS=Saccharomyces cerevisiae S288c]	852419	MRPL36; YBR122C	cell organization and biogenesis;metabolic process	mitochondrion;ribosome	structural molecule activity	0.668	3.642	5.45
P36517	54S ribosomal protein L4, mitochondrial [OS=Saccharomyces cerevisiae S288c]	851160	MRPL4; YLR439W	cell organization and biogenesis;metabolic process	mitochondrion;ribosome	structural molecule activity	0.369	2.511	6.80

Supplemental Table S3. The relative concentrations of proteins in mitochondria purified from WT cells cultured with or without LCA. Mitochondria were purified from WT cells recovered on day 7 of cell culturing with or without LCA. Mass spectrometry-based identification and quantitation of proteins recovered in purified mitochondria were performed as described in Materials and methods. The exponentially modified protein abundance index (emPAI) provided by the ″Proteome Discoverer″ software was used to calculate the relative abundance of mitochondrial proteins between specified datasets. Data on the relative concentrations of mitochondrial proteins are presented as means of 2 independent experiments.

Accession	Description	Entrez Gene ID	Gene ID	Biological Process	Cellular Component	Molecular Function	emPAI WT	emPAI WT + LCA	Ratio WT + LCA/ WT
P38120	37S ribosomal protein S9, mitochondrial	852443	MRPS9	cell organization and biogenesis; metabolic process	mitochondrion; ribosome	RNA binding; structural molecule activity	3.437	0.145	0.04
P36163	mitochondrial metalloendopeptidase OMA1	853962	OMA1	metabolic process	membrane; mitochondrion	catalytic activity; metal ion binding	0.931	0.116	0.12
P47140	Altered inheritance rate of mitochondria protein 25	853563	AIM25	cell organization and biogenesis	membrane; mitochondrion	transporter activity	0.874	0.11	0.13
Q12349	ATP synthase subunit H, mitochondrial	851002	ATP14	cell organization and biogenesis; metabolic process; transport	membrane; mitochondrion	catalytic activity; transporter activity	2.728	0.389	0.14
P32317	Protein AFG1 [OS=Saccharomyces cerevisiae S288c]	856658	AFG1; YEL052W	cell organization and biogenesis;metabolic process;response to stimulus;transport	membrane;mitochondrion	catalytic activity;nucleotide binding	1.154	0.166	0.14
Q12283	Malonyl CoA-acyl carrier protein transacylase, mitochondrial [OS=Saccharomyces cerevisiae S288c]	854396	MCT1; YOR221C	metabolic process	mitochondrion	catalytic activity	0.896	0.136	0.15
Q07534	Solute carrier family 25 member 38 homolog [OS=Saccharomyces cerevisiae S288c]	851439	YDL119C; HEM25; YDL119C	metabolic process;transport	membrane;mitochondrion	structural molecule activity;transporter activity	0.73	0.116	0.16
P53166	ATP-dependent RNA helicase mrh4, mitochondrial [OS=Saccharomyces cerevisiae S288c]	852816	MRH4; YGL064C	cell organization and biogenesis;metabolic process	mitochondrion;organelle lumen	catalytic activity;nucleotide binding;RNA binding	1.412	0.225	0.16
P16547	Mitochondrial outer membrane protein OM45 [OS=Saccharomyces cerevisiae S288c]	854670	OM45; YIL136W		membrane;mitochondrion		300.995	51.481	0.17
P22353	54S ribosomal protein L8, mitochondrial [OS=Saccharomyces cerevisiae S288c]	853382	MRPL8; YJL063C	cell organization and biogenesis;metabolic process	mitochondrion;ribosome	structural molecule activity	0.848	0.166	0.20
Q08818	Meiotic sister-chromatid recombination protein 6, mitochondrial [OS=Saccharomyces cerevisiae S288c]	854536	MSC6; YOR354C	metabolic process	mitochondrion;organelle lumen	RNA binding	1.047	0.227	0.22
P54115	Magnesium-activated aldehyde dehydrogenase, cytosolic [OS=Saccharomyces cerevisiae S288c]	856044	ALD6; YPL061W	metabolic process;response to stimulus	cytoplasm;cytosol;mitochondrion	catalytic activity	0.346	0.077	0.22
P22438	Methionine--tRNA ligase, mitochondrial [OS=Saccharomyces cerevisiae S288c]	853081	MSM1; YGR171C	metabolic process	cytoplasm;cytosol;mitochondrion;organelle lumen	catalytic activity;nucleotide binding	0.292	0.066	0.23
P25605	Acetolactate synthase small subunit, mitochondrial [OS=Saccharomyces cerevisiae S288c]	850348	ILV6; YCL009C	metabolic process;regulation of biological process	mitochondrion	catalytic activity;enzyme regulator activity	10.288	2.36	0.23
P38969	Pentamidine resistance factor, mitochondrial [OS=Saccharomyces cerevisiae S288c]	854440	PNT1; YOR266W	cell organization and biogenesis;response to stimulus	membrane;mitochondrion		0.711	0.166	0.23
P15179	Aspartate--tRNA ligase, mitochondrial [OS=Saccharomyces cerevisiae S288c]	856000	MSD1; YPL104W	metabolic process	cytoplasm;mitochondrion;organelle lumen	catalytic activity;nucleotide binding	0.708	0.174	0.25
P21954	Isocitrate dehydrogenase [NADP], mitochondrial [OS=Saccharomyces cerevisiae S288c]	851493	IDP1; YDL066W	metabolic process	mitochondrion	catalytic activity;metal ion binding;nucleotide binding;protein binding	33.145	8.261	0.25
Q06678	54S ribosomal protein L35, mitochondrial [OS=Saccharomyces cerevisiae S288c]	851921	MRPL35; YDR322W	cell organization and biogenesis;metabolic process	mitochondrion;ribosome	structural molecule activity	1.219	0.304	0.25

Q02784	Monothiol glutaredoxin-5, mitochondrial [OS=Saccharomyces cerevisiae S288c]	856048	GRX5; YPL059W	cell organization and biogenesis;cellular homeostasis;metabolic process;regulation of biological process;response to stimulus	mitochondrion;organelle lumen	catalytic activity;metal ion binding	2.594	0.668	0.26
P25626	54S ribosomal protein IMG1, mitochondrial [OS=Saccharomyces cerevisiae S288c]	850413	IMG1; YCR046C	cell organization and biogenesis;metabolic process	mitochondrion;ribosome	structural molecule activity	3.924	1.031	0.26
P40858	54S ribosomal protein L49, mitochondrial [OS=Saccharomyces cerevisiae S288c]	853349	MRPL49; YJL096W	cell organization and biogenesis;metabolic process	mitochondrion;ribosome	RNA binding;structural molecule activity	0.778	0.212	0.27
P08525	Cytochrome b-c1 complex subunit 8 [OS=Saccharomyces cerevisiae S288c]	853273	QCR8; YJL166W	metabolic process;transport	membrane;mitochondrion	catalytic activity;transporter activity	5.31	1.512	0.28
P47015	Altered inheritance of mitochondria protein 23, mitochondrial [OS=Saccharomyces cerevisiae S288c]	853310	AIM23; YJL131C	metabolic process	mitochondrion	RNA binding	1.228	0.35	0.29
P36519	54S ribosomal protein L7, mitochondrial [OS=Saccharomyces cerevisiae S288c]	851823	MRPL7; YDR237W	cell organization and biogenesis;metabolic process	mitochondrion;ribosome	RNA binding;structural molecule activity	0.468	0.136	0.29
P00401	Cytochrome c oxidase subunit 1 [OS=Saccharomyces cerevisiae S288c]	854598	COX1; Q0045	metabolic process;transport	membrane;mitochondrion	catalytic activity;metal ion binding;transporter activity	1.61	0.468	0.29
P25573	Mitochondrial inner membrane i-AAA protease supercomplex subunit MGR1 [OS=Saccharomyces cerevisiae S288c]	850313	MGR1; YCL044C	metabolic process	membrane;mitochondrion	protein binding	1.031	0.304	0.29
Q12029	Probable mitochondrial transport protein fsf1 [OS=Saccharomyces cerevisiae S288c]	854445	FSF1; YOR271C	transport	membrane;mitochondrion	transporter activity	2.793	0.833	0.30
P40035	Mitochondrial phosphate carrier protein 2 [OS=Saccharomyces cerevisiae S288c]	856779	PIC2; YER053C	cellular homeostasis;metabolic process;transport	membrane;mitochondrion	structural molecule activity;transporter activity	2.793	0.833	0.30
P36527	54S ribosomal protein L28, mitochondrial [OS=Saccharomyces cerevisiae S288c]	852073	MRPL28; YDR462W	cell organization and biogenesis;metabolic process	mitochondrion;ribosome	structural molecule activity	4.337	1.31	0.30
Q08686	Thiosulfate sulfurtransferase TUM1 [OS=Saccharomyces cerevisiae S288c]	854425	TUM1; YOR251C	metabolic process	cytoplasm;mitochondrion	catalytic activity;protein binding	0.334	0.101	0.30
P40015	inositol phosphosphingolipids phospholipase C [OS=Saccharomyces cerevisiae S288c]	856739	ISC1; YER019W	metabolic process;response to stimulus	endoplasmic reticulum;membrane;mitochondrion	catalytic activity;metal ion binding	0.334	0.101	0.30
P41911	Glycerol-3-phosphate dehydrogenase [NAD(+)] 2, mitochondrial [OS=Saccharomyces cerevisiae S288c]	854095	GPD2; YOL059W	metabolic process	cytoplasm;cytosol;mitochondrion	catalytic activity;nucleotide binding;protein binding	0.292	0.089	0.30
P00128	Cytochrome b-c1 complex subunit 7 [OS=Saccharomyces cerevisiae S288c]	852142	QCR7; YDR529C	cell organization and biogenesis;metabolic process;transport	membrane;mitochondrion	catalytic activity;transporter activity	11.915	3.642	0.31
P07266	Mitochondrial RNA-splicing protein MRS1 [OS=Saccharomyces cerevisiae S288c]	854839	MRS1; YIR021W	cell organization and biogenesis;metabolic process	mitochondrion;organelle lumen	catalytic activity;DNA binding;RNA binding	0.304	0.093	0.31
P32493	ATPase expression protein 1, mitochondrial [OS=Saccharomyces cerevisiae S288c]	855086	AEP1; YMR064W	regulation of biological process	mitochondrion	translation regulator activity	0.233	0.072	0.31
P41921	glutathione reductase [OS=Saccharomyces cerevisiae S288c]	856014	GLR1; YPL091W	cellular homeostasis;metabolic process;regulation of biological process;response to stimulus	cytoplasm;cytosol;mitochondrion;nucleus	antioxidant activity;catalytic activity;nucleotide binding	0.241	0.075	0.31
P00420	Cytochrome c oxidase subunit 3 [OS=Saccharomyces cerevisiae S288c]	854627	COX3; Q0275	metabolic process;transport	membrane;mitochondrion	catalytic activity;transporter activity	3.642	1.154	0.32
P53881	54S ribosomal protein L22, mitochondrial [OS=Saccharomyces cerevisiae S288c]	855544	MRPL22; YNL177C	cell organization and biogenesis;metabolic process	mitochondrion;ribosome	RNA binding;structural molecule activity	1.683	0.551	0.33
P36151	Uncharacterized protein YKR070W [OS=Saccharomyces cerevisiae S288c]	853944	YKR070W; YKR070W	metabolic process	mitochondrion		1.336	0.438	0.33
P12686	37S ribosomal protein MRP13, mitochondrial [OS=Saccharomyces cerevisiae S288c]	852975	MRP13; YGR084C	cell organization and biogenesis;metabolic process	mitochondrion;ribosome	structural molecule activity	0.778	0.259	0.33
Q12467	MIOREX complex component 4 [OS=Saccharomyces cerevisiae S288c]	855935	YPL168W; MRX4;		membrane;mitochondrion		0.585	0.202	0.35

			YPL168W						
P32902	37S ribosomal protein MRP4, mitochondrial [OS=Saccharomyces cerevisiae S288c]	856384	MRP4; YHL004W	cell organization and biogenesis;metabolic process	mitochondrion;ribosome	structural molecule activity	0.957	0.334	0.35
P53721	Respiratory supercomplex factor 2, mitochondrial [OS=Saccharomyces cerevisiae S288c]	855752	RCF2; YNR018W	cell organization and biogenesis	membrane;mitochondrion		7.111	2.511	0.35
P07251	ATP synthase subunit alpha, mitochondrial [OS=Saccharomyces cerevisiae S288c]	852177	ATP1; YBL099W	metabolic process;transport	cytosol;membrane;mitochondrion	catalytic activity;nucleotide binding;transporter activity	26.826	9.661	0.36
P38885	Altered inheritance of mitochondria protein 46, mitochondrial [OS=Saccharomyces cerevisiae S288c]	856606	AIM46; YHR199C		mitochondrion	catalytic activity	1.994	0.73	0.37
P48360	Probable NADPH:adrenodoxin oxidoreductase, mitochondrial [OS=Saccharomyces cerevisiae S288c]	851982	ARH1; YDR376W	cellular homeostasis;metabolic process;transport	cytoplasm;membrane;mitochondrion;organelle lumen	catalytic activity	0.682	0.25	0.37
P32897	Mitochondrial import inner membrane translocase subunit tim23 [OS=Saccharomyces cerevisiae S288c]	855751	TIM23; YNR017W	transport	membrane;mitochondrion	protein binding;transporter activity	4.012	1.512	0.38
P00360	glyceraldehyde-3-phosphate dehydrogenase 1 [OS=Saccharomyces cerevisiae S288c]	853395	TDH1; YJL052W	metabolic process	cytoplasm;cytosol;membrane;mitochondrion	catalytic activity;nucleotide binding	1.721	0.65	0.38
P32340	Rotenone-insensitive NADH-ubiquinone oxidoreductase, mitochondrial [OS=Saccharomyces cerevisiae S288c]	854919	NDI1; YML120C	metabolic process;regulation of biological process	membrane;mitochondrion;organelle lumen	catalytic activity;nucleotide binding	16.433	6.279	0.38
P39726	Glycine cleavage system H protein, mitochondrial [OS=Saccharomyces cerevisiae S288c]	851254	GCV3; YAL044C	metabolic process	mitochondrion	catalytic activity	6.743	2.594	0.38
P40502	Altered inheritance of mitochondria protein 19, mitochondrial [OS=Saccharomyces cerevisiae S288c]	854722	AIM19; YIL087C		membrane;mitochondrion		1.512	0.585	0.39
P07246	Alcohol dehydrogenase 3, mitochondrial [OS=Saccharomyces cerevisiae S288c]	855107	ADH3; YMR083W	metabolic process	mitochondrion;organelle lumen	catalytic activity;metal ion binding	7.031	2.728	0.39
Q12482	Mitochondrial aspartate-glutamate transporter AGC1 [OS=Saccharomyces cerevisiae S288c]	856132	AGC1; YPR021C	metabolic process;transport	membrane;mitochondrion	structural molecule activity;transporter activity	0.348	0.136	0.39
Q01163	37S ribosomal protein S23, mitochondrial [OS=Saccharomyces cerevisiae S288c]	852748	RSM23; YGL129C	cell organization and biogenesis;metabolic process	mitochondrion;ribosome	catalytic activity;nucleotide binding;RNA binding;structural molecule activity	4.367	1.707	0.39
P11914	Mitochondrial-processing peptidase subunit alpha [OS=Saccharomyces cerevisiae S288c]	856419	MAS2; YHR024C	metabolic process;transport	membrane;mitochondrion;organelle lumen	catalytic activity;metal ion binding;protein binding	2.311	0.905	0.39
P37267	Assembly factor cbp4 [OS=Saccharomyces cerevisiae S288c]		CBP4	cell organization and biogenesis	membrane;mitochondrion		3.329	1.31	0.39
P50088	Stationary phase gene 1 protein [OS=Saccharomyces cerevisiae S288c]	853151	SPG1; YGR236C		endoplasmic reticulum;membrane;mitochondrion		157.489	62.096	0.39
P40452	Cytochrome c oxidase assembly factor 1 [OS=Saccharomyces cerevisiae S288c]	854649	COA1; YIL157C	cell organization and biogenesis	membrane;mitochondrion	protein binding	1.31	0.52	0.40
P40496	37S ribosomal protein S25, mitochondrial [OS=Saccharomyces cerevisiae S288c]	854715	RSM25; YIL093C	cell organization and biogenesis;metabolic process	mitochondrion;ribosome	structural molecule activity	1.154	0.468	0.41
P53311	Mitochondrial pyruvate carrier 3 [OS=Saccharomyces cerevisiae S288c]	853158	FMP43; MPC3; YGR243W	transport	membrane;mitochondrion	transporter activity	1.154	0.468	0.41
P40215	External NADH-ubiquinone oxidoreductase 1, mitochondrial [OS=Saccharomyces cerevisiae S288c]	855176	NDE1; YMR145C	metabolic process	mitochondrion	catalytic activity;nucleotide binding	5.078	2.065	0.41
P40513	Mitochondrial acidic protein MAM33 [OS=Saccharomyces cerevisiae S288c]	854740	MAM33; YIL070C	metabolic process;regulation of biological process	mitochondrion;organelle lumen	translation regulator activity	2.831	1.154	0.41
Q06698	Putative ATP-dependent RNA helicase YLR419W [OS=Saccharomyces cerevisiae S288c]	851137	YLR419W; YLR419W	metabolic process	cytoplasm;mitochondrion;nucleus	catalytic activity;nucleotide binding;protein binding;RNA binding	0.201	0.082	0.41

Q04599	54S ribosomal protein L1, mitochondrial [OS=Saccharomyces cerevisiae S288c]	851694	MRPL1; YDR116C	cell organization and biogenesis;metabolic process	mitochondrion;ribosome	RNA binding;structural molecule activity	1.069	0.438	0.41
P28625	Protein YIM1 [OS=Saccharomyces cerevisiae S288c]	855183	YIM1; YMR152W	metabolic process;response to stimulus	cytoplasm;endoplasmic reticulum;mitochondrion	catalytic activity;metal ion binding	1.069	0.438	0.41
P35180	mitochondrial import receptor subunit Tom20 [OS=Saccharomyces cerevisiae S288c]	852973	TOM20; YGR082W	cell organization and biogenesis;transport	membrane;mitochondrion	protein binding;transporter activity	1.031	0.425	0.41
P38175	37S ribosomal protein MRP21, mitochondrial [OS=Saccharomyces cerevisiae S288c]	852188	MRP21; YBL090W	cell organization and biogenesis;metabolic process	mitochondrion;ribosome	structural molecule activity	1.031	0.425	0.41
P28834	Isocitrate dehydrogenase [NAD] subunit 1, mitochondrial [OS=Saccharomyces cerevisiae S288c]	855691	IDH1; YNL037C	metabolic process;transport	cytosol;mitochondrion;organelle lumen	catalytic activity;metal ion binding;nucleotide binding;protein binding;RNA binding	7.859	3.281	0.42
P18496	Mitochondrial ATPase complex subunit ATP10 [OS=Saccharomyces cerevisiae S288c]	851109	ATP10; YLR393W	cell organization and biogenesis	cytoplasm;membrane;mitochondrion	protein binding	1.818	0.778	0.43
Q02204	54S ribosomal protein L13, mitochondrial [OS=Saccharomyces cerevisiae S288c]	853875	MRPL13; YKR006C	cell organization and biogenesis;metabolic process	mitochondrion;ribosome	structural molecule activity	0.778	0.334	0.43
Q99297	Mitochondrial 2-oxodicarboxylate carrier 2 [OS=Saccharomyces cerevisiae S288c]	854397	ODC2; YOR222W	metabolic process;transport	membrane;mitochondrion	structural molecule activity;transporter activity	0.778	0.334	0.43
Q07914	mitochondrial import inner membrane translocase subunit TIM14 [OS=Saccharomyces cerevisiae S288c]	850694	PAM18; YLR008C	regulation of biological process;transport	membrane;mitochondrion	enzyme regulator activity;protein binding;transporter activity	0.778	0.334	0.43
P32387	54S ribosomal protein L41, mitochondrial [OS=Saccharomyces cerevisiae S288c]	852014	MRP20; YDR405W	cell organization and biogenesis;metabolic process	mitochondrion;ribosome	nucleotide binding;protein binding;RNA binding;structural molecule activity	0.719	0.311	0.43
P38172	MIOREX complex component 3 [OS=Saccharomyces cerevisiae S288c]	852181	YBL095W; MRX3; YBL095W		membrane;mitochondrion		0.719	0.311	0.43
P07213	Mitochondrial import receptor subunit TOM70 [OS=Saccharomyces cerevisiae S288c]	855602	TOM70; YNL121C	cell organization and biogenesis;transport	membrane;mitochondrion	protein binding;transporter activity	2.665	1.154	0.43
P14063	54S ribosomal protein L31, mitochondrial [OS=Saccharomyces cerevisiae S288c]	853720	MRPL31; YKL138C	cell organization and biogenesis;metabolic process	mitochondrion;ribosome	structural molecule activity	0.668	0.292	0.44
Q06090	54S ribosomal protein L51, mitochondrial [OS=Saccharomyces cerevisiae S288c]	856214	MRPL51; YPR100W	cell organization and biogenesis;metabolic process	mitochondrion;ribosome	RNA binding;structural molecule activity	0.668	0.292	0.44
P42834	mitochondrial DnaJ homolog 2 [OS=Saccharomyces cerevisiae S288c]	855388	MDJ2; YNL328C	regulation of biological process;transport	membrane;mitochondrion	enzyme regulator activity;transporter activity	0.668	0.292	0.44
Q06892	NADH kinase POS5, mitochondrial [OS=Saccharomyces cerevisiae S288c]	855913	POS5; YPL188W	metabolic process;response to stimulus	mitochondrion;organelle lumen	catalytic activity;nucleotide binding	1.565	0.688	0.44
P38152	Tricarboxylate transport protein [OS=Saccharomyces cerevisiae S288c]	852594	CTP1; YBR291C	metabolic process;transport	membrane;mitochondrion	structural molecule activity;transporter activity	0.551	0.245	0.44
P40086	Cytochrome c oxidase assembly protein cox15 [OS=Saccharomyces cerevisiae S288c]	856884	COX15; YER141W	cell organization and biogenesis;metabolic process;transport	membrane;mitochondrion	catalytic activity;transporter activity	2.594	1.154	0.44
P36528	54S ribosomal protein L17, mitochondrial [OS=Saccharomyces cerevisiae S288c]	855469	MRPL17; YNL252C	cell organization and biogenesis;metabolic process	mitochondrion;ribosome	structural molecule activity	0.52	0.233	0.45
P36013	NAD-dependent malic enzyme, mitochondrial [OS=Saccharomyces cerevisiae S288c]	853839	MAE1; YKL029C	metabolic process	mitochondrion;organelle lumen	catalytic activity;metal ion binding;nucleotide binding	1.512	0.679	0.45
P04840	Mitochondrial outer membrane protein porin 1 [OS=Saccharomyces cerevisiae S288c]	855669	POR1; YNL055C	cell death;cell organization and biogenesis;cellular homeostasis;regulation of biological process;transport	cytoplasm;membrane;mitochondrion	transporter activity	15.238	6.848	0.45
Q07349	MIOREX complex component 9 [OS=Saccharomyces cerevisiae S288c]	851535	YDL027C; MRX9; YDL027C		endoplasmic reticulum;membrane;mitochondrion		2.433	1.096	0.45
P34224	Uncharacterized protein YBL059W [OS=Saccharomyces cerevisiae S288c]	852221	YBL059W; YBL059W		membrane;mitochondrion		0.468	0.212	0.45

P32839	Mitochondrial chaperone BCS1 [OS=Saccharomyces cerevisiae S288c]	851981	BCS1; YDR375C	cell organization and biogenesis;metabolic process;response to stimulus;transport	cytosol;membrane;mitochondrion	catalytic activity;nucleotide binding;transporter activity	0.425	0.194	0.46
Q01976	ADP-ribose pyrophosphatase [OS=Saccharomyces cerevisiae S288c]	852408	YSA1; YBR111C	metabolic process	cytoplasm;mitochondrion;nucleus	catalytic activity;metal ion binding	0.425	0.194	0.46
P25348	54S ribosomal protein L32, mitochondrial [OS=Saccharomyces cerevisiae S288c]	850359	MRPL32; YCR003W	cell organization and biogenesis;metabolic process	membrane;mitochondrion;ribosome	RNA binding;structural molecule activity	0.389	0.179	0.46
P22136	ATPase expression protein 2, mitochondrial [OS=Saccharomyces cerevisiae S288c]	855325	AEP2; YMR282C	metabolic process;regulation of biological process	mitochondrion	RNA binding	0.359	0.166	0.46
Q06630	Mitochondrial homologous recombination protein 1 [OS=Saccharomyces cerevisiae S288c]	851890	MHR1; YDR296W	cell organization and biogenesis;metabolic process;regulation of biological process;response to stimulus	mitochondrion;nucleus	catalytic activity;DNA binding;structural molecule activity	0.359	0.166	0.46
P36516	54S ribosomal protein L3, mitochondrial [OS=Saccharomyces cerevisiae S288c]	855039	MRPL3; YMR024W	cell organization and biogenesis;metabolic process;response to stimulus	mitochondrion;nucleus;ribosome	catalytic activity;RNA binding;structural molecule activity	1.043	0.487	0.47
P38297	Mitofusin FZO1 [OS=Saccharomyces cerevisiae S288c]	852477	FZO1; YBR179C	cell organization and biogenesis	membrane;mitochondrion	catalytic activity;nucleotide binding;protein binding	0.304	0.142	0.47
Q06089	Uncharacterized mitochondrial outer membrane protein YPR098C [OS=Saccharomyces cerevisiae S288c]	856213	YPR098C; YPR098C		membrane;mitochondrion		4.623	2.162	0.47
Q07500	External NADH-ubiquinone oxidoreductase 2, mitochondrial [OS=Saccharomyces cerevisiae S288c]	851474	NDE2; YDL085W	metabolic process	mitochondrion	catalytic activity;nucleotide binding	6.356	2.981	0.47
P25270	rRNA methyltransferase 1, mitochondrial [OS=Saccharomyces cerevisiae S288c]	854376	MRM1; YOR201C	metabolic process	mitochondrion	catalytic activity;RNA binding	0.274	0.129	0.47
P38122	3-methyl-2-oxobutanoate hydroxymethyltransferase [OS=Saccharomyces cerevisiae S288c]	852474	ECM31; YBR176W	metabolic process	mitochondrion	catalytic activity	0.259	0.122	0.47
P36008	Elongation factor 1-gamma 2 [OS=Saccharomyces cerevisiae S288c]	853781	TEF4; YKL081W	metabolic process;regulation of biological process	cytoplasm;mitochondrion;nucleus;ribosome	catalytic activity;protein binding;RNA binding	0.202	0.096	0.48
P47039	Probable kynurenine--oxoglutarate transaminase BNA3 [OS=Saccharomyces cerevisiae S288c]	853386	BNA3; YJL060W	metabolic process;regulation of biological process	cytoplasm;mitochondrion	catalytic activity	0.212	0.101	0.48
Q03430	37S ribosomal protein RSM28, mitochondrial [OS=Saccharomyces cerevisiae S288c]	852105	RSM28; YDR494W	cell organization and biogenesis;metabolic process	mitochondrion;ribosome	structural molecule activity	3.062	1.462	0.48
P00359	Glyceraldehyde-3-phosphate dehydrogenase 3 [OS=Saccharomyces cerevisiae S288c]	853106	TDH3; YGR192C	cell death;metabolic process	cytoplasm;cytosol;membrane;mitochondrion;nucleus	catalytic activity;nucleotide binding;RNA binding	6.406	3.062	0.48
P40165	NAD(P)H-hydrate epimerase [OS=Saccharomyces cerevisiae S288c]	855521	YNL200C; YNL200C	metabolic process	cytoplasm;mitochondrion	catalytic activity;metal ion binding;nucleotide binding	1.61	0.778	0.48
P32048	Lysine--tRNA ligase, mitochondrial [OS=Saccharomyces cerevisiae S288c]	855651	MSK1; YNL073W	cell organization and biogenesis;metabolic process;transport	cytoplasm;mitochondrion;organelle lumen	catalytic activity;nucleotide binding;RNA binding	0.122	0.059	0.48
P39925	Mitochondrial respiratory chain complexes assembly protein AFG3 [OS=Saccharomyces cerevisiae S288c]	856737	AFG3; YER017C	cell organization and biogenesis;metabolic process;response to stimulus;transport	membrane;mitochondrion	catalytic activity;metal ion binding;nucleotide binding;protein binding	1.015	0.492	0.48
Q12480	Probable electron transfer flavoprotein subunit alpha, mitochondrial [OS=Saccharomyces cerevisiae S288c]	856112	AIM45; YPR004C	metabolic process;transport	mitochondrion;organelle lumen	catalytic activity;nucleotide binding	2.548	1.239	0.49
Q04458	Fatty aldehyde dehydrogenase HFD1 [OS=Saccharomyces cerevisiae S288c]	855137	HFD1; YMR110C	metabolic process	endoplasmic reticulum;endosome;membrane;mitochondrion	catalytic activity	3.642	1.783	0.49
P06208-1	2-isopropylmalate synthase [OS=Saccharomyces cerevisiae S288c]	855619	LEU4; YNL104C	metabolic process	cytoplasm;mitochondrion	catalytic activity;protein binding	4.223	2.07	0.49
P00175	Cytochrome b2, mitochondrial [OS=Saccharomyces cerevisiae S288c]	854950	CYB2; YML054C	metabolic process;transport	cytosol;membrane;mitochondrion;nucleus	catalytic activity;metal ion binding;nucleotide binding	0.679	0.334	0.49
P00358	glyceraldehyde-3-phosphate dehydrogenase 2 [OS=Saccharomyces cerevisiae S288c]	853465	TDH2; YJR009C	cell death;metabolic process	cytoplasm;cytosol;membrane;mitochondrion;nucleus	catalytic activity;nucleotide binding;protein binding	3.489	1.721	0.49

P49095	Glycine dehydrogenase (Decarboxylating), mitochondrial [OS=Saccharomyces cerevisiae S288c]	855227	GCV2; YMR189W	metabolic process	cytosol;mitochondrion	catalytic activity	1.575	0.778	0.49
P32787	Mitochondrial genome maintenance protein MGM101 [OS=Saccharomyces cerevisiae S288c]	853609	MGM101; YJR144W	cell organization and biogenesis;metabolic process;response to stimulus	chromosome;mitochondrion	DNA binding	3.281	1.637	0.50
P07806	Valine--tRNA ligase, mitochondrial [OS=Saccharomyces cerevisiae S288c]	852986	VAS1; YGR094W	metabolic process;regulation of biological process	cytoplasm;cytosol;mitochondrion	catalytic activity;nucleotide binding	0.668	0.334	0.50
P36517	54S ribosomal protein L4, mitochondrial [OS=Saccharomyces cerevisiae S288c]	851160	MRPL4; YLR439W	cell organization and biogenesis;metabolic process	mitochondrion;ribosome	structural molecule activity	2.162	1.081	0.50
P53170	[Pyruvate dehydrogenase (acetyl-transferring)] kinase 2, mitochondrial [OS=Saccharomyces cerevisiae S288c]	852821	PKP2; YGL059W	metabolic process;regulation of biological process	mitochondrion;organelle lumen	catalytic activity;nucleotide binding;protein binding	0.778	0.389	0.50
P28817	3-hydroxyisobutyryl-CoA hydrolase, mitochondrial [OS=Saccharomyces cerevisiae S288c]	851606	EHD3; YDR036C	metabolic process	mitochondrion	catalytic activity	0.743	0.374	0.50
P23180	Probable oxidoreductase AIM17 [OS=Saccharomyces cerevisiae S288c]	856365	AIM17; YHL021C	cell organization and biogenesis;metabolic process	mitochondrion	catalytic activity;metal ion binding	2.981	1.512	0.51
Q08023	Protein FMP25, mitochondrial [OS=Saccharomyces cerevisiae S288c]	850766	FMP25; YLR077W	cell organization and biogenesis	membrane;mitochondrion		1.412	0.719	0.51
P36066	Protein MRG3-like [OS=Saccharomyces cerevisiae S288c]	853725	YKL133C; YKL133C	metabolic process	membrane;mitochondrion	protein binding	1.054	0.54	0.51
Q06668	Methyltransferase OMS1, mitochondrial [OS=Saccharomyces cerevisiae S288c]	851911	OMS1; YDR316W	metabolic process	membrane;mitochondrion	catalytic activity	0.585	0.301	0.51
P04037	cytochrome c oxidase subunit 4, mitochondrial [OS=Saccharomyces cerevisiae S288c]	852688	COX4; YGL187C	cell organization and biogenesis;metabolic process;transport	membrane;mitochondrion	catalytic activity;metal ion binding;transporter activity	27.48	14.199	0.52
P40053	Altered inheritance of mitochondria protein 9, mitochondrial [OS=Saccharomyces cerevisiae S288c]	856813	AIM9; YER080W		mitochondrion		4.036	2.086	0.52
P50945	MICOS complex subunit MIC27 [OS=Saccharomyces cerevisiae S288c]	855623	AIM37; MIC27; YNL100W	cell organization and biogenesis	membrane;mitochondrion		0.968	0.501	0.52
P51998	54S ribosomal protein yml6, mitochondrial [OS=Saccharomyces cerevisiae S288c]	854983	YML6; YML025C	cell organization and biogenesis;metabolic process	mitochondrion;ribosome	RNA binding;structural molecule activity	1.404	0.73	0.52
Q12230	Sphingolipid long chain base-responsive protein LSP1 [OS=Saccharomyces cerevisiae S288c]	856103	LSP1; YPL004C	regulation of biological process;response to stimulus;transport	cytoplasm;membrane;mitochondrion		24.119	12.594	0.52
P43617	Uncharacterized mitochondrial carrier YFR045W [OS=Saccharomyces cerevisiae S288c]	850606	YFR045W; YFR045W	metabolic process;transport	membrane;mitochondrion	structural molecule activity;transporter activity	0.778	0.413	0.53
P36520	54S ribosomal protein L10, mitochondrial [OS=Saccharomyces cerevisiae S288c]	855436	MRPL10; YNL284C	cell organization and biogenesis;metabolic process	mitochondrion;ribosome	structural molecule activity	0.73	0.389	0.53
P53736	Uncharacterized protein YNR040W [OS=Saccharomyces cerevisiae S288c]	855776	YNR040W; YNR040W		mitochondrion		0.73	0.389	0.53
P53305	Mitochondrial 37S ribosomal protein S27 [OS=Saccharomyces cerevisiae S288c]	853129	RSM27; YGR215W	cell organization and biogenesis;metabolic process	mitochondrion;ribosome	structural molecule activity	2.162	1.154	0.53
Q04935	Cytochrome c oxidase protein 20, mitochondrial [OS=Saccharomyces cerevisiae S288c]	851817	COX20; YDR231C	cell organization and biogenesis;metabolic process	membrane;mitochondrion	protein binding	1.894	1.031	0.54
P32891	D-lactate dehydrogenase [cytochrome] 1, mitochondrial [OS=Saccharomyces cerevisiae S288c]	851380	DLD1; YDL174C	metabolic process;transport	membrane;mitochondrion	catalytic activity;nucleotide binding	11.253	6.128	0.54
P0CS90	Heat shock protein SSC1, mitochondrial [OS=Saccharomyces cerevisiae S288c]	853503	SSC1; YJR045C	cell organization and biogenesis;metabolic process;regulation of biological process;transport	membrane;mitochondrion;nucleus;organelle lumen	catalytic activity;enzyme regulator activity;nucleotide binding;protein binding	26.826	14.647	0.55
P39522	Dihydroxy-acid dehydratase, mitochondrial [OS=Saccharomyces cerevisiae	853473	ILV3; YJR016C	metabolic process	mitochondrion	catalytic activity;metal ion binding	16.475	9	0.55

	S288c]								
P38071	Enoyl-[acyl-carrier protein] reductase [NADPH, B-specific], mitochondrial [OS=Saccharomyces cerevisiae S288c]	852314	ETR1; YBR026C	metabolic process	mitochondrion;organelle lumen	catalytic activity;DNA binding;metal ion binding	9	4.926	0.55
P43616	Cys-Gly metallodipeptidase dug1 [OS=Saccharomyces cerevisiae S288c]	850605	DUG1; YFR044C	metabolic process	cytoplasm;mitochondrion;ribosome	catalytic activity;metal ion binding	0.848	0.468	0.55
P39006	Phosphatidylserine decarboxylase proenzyme 1, mitochondrial [OS=Saccharomyces cerevisiae S288c]	855552	PSD1; YNL169C	metabolic process;regulation of biological process	membrane;mitochondrion	catalytic activity	0.487	0.269	0.55
P32860	NifU-like protein, mitochondrial [OS=Saccharomyces cerevisiae S288c]	853826	NFU1; YKL040C	cell organization and biogenesis;cellular homeostasis;metabolic process	mitochondrion;organelle lumen	metal ion binding	3.87	2.162	0.56
Q05892	MIOREX complex component 2 [OS=Saccharomyces cerevisiae S288c]	850997	YLR290C; COQ11; YLR290C	metabolic process	mitochondrion	catalytic activity	3.87	2.162	0.56
Q04472	Mitochondrial inner membrane i-AAA protease supercomplex subunit MGR3 [OS=Saccharomyces cerevisiae S288c]	855142	MGR3; YMR115W	metabolic process	membrane;mitochondrion	protein binding	0.417	0.233	0.56
P47052	succinate dehydrogenase [ubiquinone] flavoprotein subunit 2, mitochondrial [OS=Saccharomyces cerevisiae S288c]	853405	YJL045W; YJL045W	metabolic process;transport	membrane;mitochondrion	catalytic activity;nucleotide binding	4.043	2.262	0.56
P28241	Isocitrate dehydrogenase [NAD] subunit 2, mitochondrial [OS=Saccharomyces cerevisiae S288c]	854303	IDH2; YOR136W	metabolic process	mitochondrion;organelle lumen	catalytic activity;metal ion binding;nucleotide binding;protein binding;RNA binding	4.179	2.34	0.56
Q06567	ABC1 family protein MCP2 [OS=Saccharomyces cerevisiae S288c]	850955	YLR253W; MCP2; YLR253W	cell organization and biogenesis	membrane;mitochondrion		0.389	0.218	0.56
Q12374	Nuclear control of ATPase protein 2 [OS=Saccharomyces cerevisiae S288c]	856278	NCA2; YPR155C	metabolic process	membrane;mitochondrion		0.668	0.377	0.56
P24720	Protein MNE1 [OS=Saccharomyces cerevisiae S288c]	854532	MNE1; YOR350C	metabolic process	cytoplasm;mitochondrion;organelle lumen		0.343	0.194	0.57
Q12233	ATP synthase subunit g, mitochondrial [OS=Saccharomyces cerevisiae S288c]	856131	ATP20; YPR020W	cell organization and biogenesis;metabolic process;transport	membrane;mitochondrion	catalytic activity;transporter activity	1.371	0.778	0.57
Q12165	ATP synthase subunit delta, mitochondrial [OS=Saccharomyces cerevisiae S288c]	851560	ATP16; YDL004W	metabolic process;transport	membrane;mitochondrion	catalytic activity;transporter activity	1.371	0.778	0.57
P53326	Uncharacterized protein YGR266W [OS=Saccharomyces cerevisiae S288c]	853182	YGR266W; YGR266W		membrane;mitochondrion		0.778	0.442	0.57
P00830	ATP synthase subunit beta, mitochondrial [OS=Saccharomyces cerevisiae S288c]	853585	ATP2; YJR121W	metabolic process;transport	cytosol;membrane;mitochondrion	catalytic activity;nucleotide binding;protein binding;transporter activity	38.811	22.263	0.57
P27929	37S ribosomal protein NAM9, mitochondrial [OS=Saccharomyces cerevisiae S288c]	855585	NAM9; YNL137C	cell organization and biogenesis;metabolic process;regulation of biological process	mitochondrion;ribosome	RNA binding;structural molecule activity	1.202	0.693	0.58
P32316	acetyl-CoA hydrolase [OS=Saccharomyces cerevisiae S288c]	852266	ACH1; YBL015W	metabolic process	cytoplasm;cytosol;mitochondrion	catalytic activity	10.498	6.055	0.58
P23833	Protein SCO1, mitochondrial [OS=Saccharomyces cerevisiae S288c]	852325	SCO1; YBR037C	cell organization and biogenesis;cellular homeostasis;response to stimulus;transport	membrane;mitochondrion	antioxidant activity;catalytic activity;metal ion binding	1.154	0.668	0.58
P36531	54S ribosomal protein L36, mitochondrial [OS=Saccharomyces cerevisiae S288c]	852419	MRPL36; YBR122C	cell organization and biogenesis;metabolic process	mitochondrion;ribosome	structural molecule activity	1.154	0.668	0.58
P53732	37S ribosomal protein S12, mitochondrial [OS=Saccharomyces cerevisiae S288c]	855772	MRPS12; YNR036C	cell organization and biogenesis;metabolic process	mitochondrion;ribosome	structural molecule activity	1.154	0.668	0.58
P16622	Ferrochelatase, mitochondrial [OS=Saccharomyces cerevisiae S288c]	854347	HEM15; YOR176W	metabolic process	membrane;mitochondrion	catalytic activity	4.337	2.511	0.58
P18239	ADP,ATP carrier protein 2 [OS=Saccharomyces cerevisiae S288c]	852250	PET9; YBL030C	cell death;metabolic process;transport	membrane;mitochondrion	structural molecule activity;transporter activity	47.064	27.48	0.58

Q00402	Nuclear migration protein NUM1 [OS=Saccharomyces cerevisiae S288c]	851727	NUM1; YDR150W	cell organization and biogenesis;cellular component movement;metabolic process;regulation of biological process;transport	endoplasmic reticulum;mitochondrion	catalytic activity;motor activity;protein binding	0.065	0.038	0.58
P12687	54S ribosomal protein L2, mitochondrial [OS=Saccharomyces cerevisiae S288c]	855727	MRP7; YNL005C	cell organization and biogenesis;metabolic process	mitochondrion;ribosome	catalytic activity;RNA binding;structural molecule activity	1.848	1.081	0.58
P36112	MICOS complex subunit Mic60 [OS=Saccharomyces cerevisiae S288c]	853886	FCJ1; MIC60; YKR016W	cell organization and biogenesis;transport	membrane;mitochondrion		15.379	9	0.59
P15424	ATP-dependent RNA helicase MSS116, mitochondrial [OS=Saccharomyces cerevisiae S288c]	851775	MSS116; YDR194C	metabolic process;regulation of biological process	mitochondrion;organelle lumen	catalytic activity;nucleotide binding;RNA binding	1.783	1.047	0.59
P53724	54S ribosomal protein L50, mitochondrial [OS=Saccharomyces cerevisiae S288c]	855756	MRPL50; YNR022C	cell organization and biogenesis;metabolic process	mitochondrion;ribosome	structural molecule activity	0.995	0.585	0.59
P17695	Glutaredoxin-2, mitochondrial [OS=Saccharomyces cerevisiae S288c]	852124	GRX2; YDR513W	cellular homeostasis;metabolic process;regulation of biological process;response to stimulus;transport	cytoplasm;cytosol;mitochondrion;nucleus	antioxidant activity;catalytic activity	0.874	0.52	0.59
P14832	peptidyl-prolyl cis-trans isomerase [OS=Saccharomyces cerevisiae S288c]	851733	CPR1; YDR155C	cell differentiation;cell organization and biogenesis;metabolic process;regulation of biological process;transport	cytoplasm;mitochondrion;nucleus	catalytic activity;RNA binding	0.874	0.52	0.59
Q08742	Thiosulfate sulfurtransferase RDL2, mitochondrial [OS=Saccharomyces cerevisiae S288c]	854460	RDL2; YOR286W		mitochondrion	catalytic activity	0.874	0.52	0.59
Q00711	Succinate dehydrogenase [ubiquinone] flavoprotein subunit, mitochondrial [OS=Saccharomyces cerevisiae S288c]	853709	SDH1; YKL148C	metabolic process;transport	membrane;mitochondrion	catalytic activity;nucleotide binding;protein binding	8.454	5.032	0.60
P36101	tRNA threonylcarbamoyladenosine dehydratase 2 [OS=Saccharomyces cerevisiae S288c]	853841	TCD2; YKL027W; YKL027W	metabolic process	cytosol;membrane;mitochondrion	catalytic activity;nucleotide binding	0.778	0.468	0.60
Q03020	iron sulfur cluster assembly protein 1, mitochondrial [OS=Saccharomyces cerevisiae S288c]	855968	ISU1; YPL135W	cell organization and biogenesis;cellular homeostasis;metabolic process	cytoplasm;mitochondrion;organelle lumen	catalytic activity;metal ion binding;protein binding	0.778	0.468	0.60
P25642	54S ribosomal protein IMG2, mitochondrial [OS=Saccharomyces cerevisiae S288c]	850434	IMG2; YCR071C	cell organization and biogenesis;metabolic process	mitochondrion;ribosome	structural molecule activity	0.778	0.468	0.60
P25087	Sterol 24-C-methyltransferase [OS=Saccharomyces cerevisiae S288c]	855003	ERG6; YML008C	metabolic process	endoplasmic reticulum;membrane;mitochondrion	catalytic activity	1.424	0.859	0.60
P38825	Protein TOM71 [OS=Saccharomyces cerevisiae S288c]	856517	TOM71; YHR117W	transport	membrane;mitochondrion	protein binding;transporter activity	0.701	0.425	0.61
P53206	putative cysteine synthase [OS=Saccharomyces cerevisiae S288c]	852895	YGR012W; YGR012W	metabolic process		catalytic activity	1.346	0.817	0.61
P32843	Mitochondrial escape protein 2 [OS=Saccharomyces cerevisiae S288c]	855348	YME2; YMR302C	cell organization and biogenesis;metabolic process	membrane;mitochondrion	nucleotide binding;RNA binding	3.458	2.099	0.61
P38891	Branched-chain-amino-acid aminotransferase, mitochondrial [OS=Saccharomyces cerevisiae S288c]	856615	BAT1; YHR208W	metabolic process;regulation of biological process;response to stimulus	mitochondrion;organelle lumen	catalytic activity	7.111	4.337	0.61
P33416	Heat shock protein 78, mitochondrial [OS=Saccharomyces cerevisiae S288c]	851845	HSP78; YDR258C	cell organization and biogenesis;metabolic process;regulation of biological process;response to stimulus	mitochondrion;organelle lumen	catalytic activity;nucleotide binding;protein binding	4.264	2.609	0.61
Q02776	Mitochondrial import inner membrane translocase subunit TIM50 [OS=Saccharomyces cerevisiae S288c]	856042	TIM50; YPL063W	cell organization and biogenesis;metabolic process;transport	membrane;mitochondrion	catalytic activity;protein binding;transporter activity	7.483	4.623	0.62
Q12375	mitochondrial ornithine transporter 1 [OS=Saccharomyces cerevisiae S288c]	854297	ORT1; YOR130C	metabolic process;transport	membrane;mitochondrion	structural molecule activity;transporter activity	0.54	0.334	0.62
P14693	sorting assembly machinery 35 kDa subunit [OS=Saccharomyces cerevisiae S288c]	856483	SAM35; YHR083W	cell organization and biogenesis;transport	membrane;mitochondrion	protein binding	0.54	0.334	0.62
P31334	54S ribosomal protein L9, mitochondrial [OS=Saccharomyces cerevisiae S288c]	853135	MRPL9; YGR220C	cell organization and biogenesis;metabolic process	mitochondrion;ribosome	structural molecule activity	0.501	0.311	0.62

Q12428	Probable 2-methylcitrate dehydratase [OS=Saccharomyces cerevisiae S288c]	856108	PDH1; YPR002W	metabolic process	cytoplasm;membrane;mitochondrion	catalytic activity	12.335	7.66	0.62
P00890	citrate synthase, mitochondrial [OS=Saccharomyces cerevisiae S288c]	855732	CIT1; YNR001C	metabolic process;transport	cytosol;mitochondrion;organelle lumen	catalytic activity	8.211	5.105	0.62
P32191	Glycerol-3-phosphate dehydrogenase, mitochondrial [OS=Saccharomyces cerevisiae S288c]	854651	GUT2; YIL155C	metabolic process	membrane;mitochondrion	catalytic activity	6.097	3.796	0.62
P38271	Protein OPY1 [OS=Saccharomyces cerevisiae S288c]	852426	OPY1; YBR129C		cytoplasm;mitochondrion		0.438	0.274	0.63
P49954	probable hydrolase NIT3 [OS=Saccharomyces cerevisiae S288c]	851065	NIT3; YLR351C	metabolic process	cytoplasm;mitochondrion	catalytic activity	0.438	0.274	0.63
P42900	Sigma-like sequence protein 1, mitochondrial [OS=Saccharomyces cerevisiae S288c]	850830	SLS1; YLR139C	cell organization and biogenesis;metabolic process	membrane;mitochondrion	protein binding	0.425	0.266	0.63
P25719	Peptidyl-prolyl cis-trans isomerase C, mitochondrial [OS=Saccharomyces cerevisiae S288c]	854897	CPR3; YML078W	cell death;metabolic process	mitochondrion;organelle lumen	catalytic activity	2.415	1.512	0.63
P25349	Flavoprotein-like protein YCP4 [OS=Saccharomyces cerevisiae S288c]	850360	YCP4; YCR004C	metabolic process;regulation of biological process	cytoplasm;membrane;mitochondrion	catalytic activity;nucleotide binding;protein binding	5.813	3.642	0.63
Q02981	ABC1 family protein YPL109C, mitochondrial [OS=Saccharomyces cerevisiae S288c]	855995	YPL109C; YPL109C		mitochondrion		0.413	0.259	0.63
P28239	Inorganic pyrophosphatase, mitochondrial [OS=Saccharomyces cerevisiae S288c]	855309	PPA2; YMR267W	metabolic process	cytoplasm;mitochondrion	catalytic activity;metal ion binding	0.413	0.259	0.63
P48015	Aminomethyltransferase, mitochondrial [OS=Saccharomyces cerevisiae S288c]	851582	GCV1; YDR019C	metabolic process	mitochondrion	catalytic activity;protein binding	8.047	5.062	0.63
P49017	2-methoxy-6-polyprenyl-1,4-benzoquinol methylase, mitochondrial [OS=Saccharomyces cerevisiae S288c]	854930	COQ5; YML110C	metabolic process	membrane;mitochondrion;organelle lumen	catalytic activity	0.389	0.245	0.63
P43635	Citrate synthase 3, mitochondrial [OS=Saccharomyces cerevisiae S288c]	856107	CIT3; YPR001W	metabolic process	mitochondrion	catalytic activity	3.642	2.3	0.63
P47127	Altered inheritance of mitochondria protein 24, mitochondrial [OS=Saccharomyces cerevisiae S288c]	853543	AIM24; YJR080C	cell organization and biogenesis	mitochondrion		2.857	1.807	0.63
P08417	fumarate hydratase, mitochondrial [OS=Saccharomyces cerevisiae S288c]	855866	FUM1; YPL262W	cell organization and biogenesis;metabolic process	cytoplasm;cytosol;mitochondrion;organelle lumen	catalytic activity	8.237	5.21	0.63
P43567	alanine--glyoxylate aminotransferase 1 [OS=Saccharomyces cerevisiae S288c]	850514	AGX1; YFL030W	metabolic process	cytosol;mitochondrion	catalytic activity	2.162	1.371	0.63
P53252	sphingolipid long chain base-responsive protein PIL1 [OS=Saccharomyces cerevisiae S288c]	852977	PIL1; YGR086C	cell organization and biogenesis;regulation of biological process;response to stimulus;transport	cytoplasm;membrane;mitochondrion	protein binding	10.45	6.627	0.63
P34227	Mitochondrial peroxiredoxin PRX1 [OS=Saccharomyces cerevisiae S288c]	852215	PRX1; YBL064C	cellular homeostasis;metabolic process;regulation of biological process;response to stimulus	mitochondrion	antioxidant activity;catalytic activity	5.158	3.281	0.64
P40961	prohibitin-1 [OS=Saccharomyces cerevisiae S288c]	853033	PHB1; YGR132C	cell organization and biogenesis;metabolic process;regulation of biological process	membrane;mitochondrion	protein binding	5.158	3.281	0.64
P20967	2-oxoglutarate dehydrogenase, mitochondrial [OS=Saccharomyces cerevisiae S288c]	854681	KGD1; YIL125W	metabolic process	cytosol;mitochondrion;organelle lumen	catalytic activity	8.168	5.201	0.64
P12695	Dihydrolipoyllysine-residue acetyltransferase component of pyruvate dehydrogenase complex, mitochondrial [OS=Saccharomyces cerevisiae S288c]	855653	LAT1; YNL071W	metabolic process	mitochondrion;organelle lumen	catalytic activity	5.898	3.758	0.64
Q01532-1	Cysteine proteinase 1, mitochondrial [OS=Saccharomyces cerevisiae S288c]			metabolic process;regulation of biological process;response to stimulus	cytoplasm;mitochondrion	catalytic activity;DNA binding;RNA binding	0.28	0.179	0.64
P40047	Aldehyde dehydrogenase 5, mitochondrial [OS=Saccharomyces cerevisiae S288c]	856804	ALD5; YER073W	metabolic process	mitochondrion;organelle lumen	catalytic activity	1.955	1.254	0.64

P38771	Ribosome-recycling factor, mitochondrial [OS=Saccharomyces cerevisiae S288c]	856433	RRF1; YHR038W	cell organization and biogenesis;metabolic process	mitochondrion	RNA binding	1.955	1.254	0.64
P32445	Single-stranded DNA-binding protein RIM1, mitochondrial [OS=Saccharomyces cerevisiae S288c]	850395	RIM1; YCR028C-A	cell organization and biogenesis;metabolic process;regulation of biological process	mitochondrion	DNA binding	1.783	1.154	0.65
Q03653	protein EFR3 [OS=Saccharomyces cerevisiae S288c]	855252	EFR3; YMR212C		membrane;mitochondrion		0.148	0.096	0.65
P09624	Dihydrolipoyl dehydrogenase, mitochondrial [OS=Saccharomyces cerevisiae S288c]	850527	LPD1; YFL018C	cellular homeostasis;metabolic process;regulation of biological process	mitochondrion;organelle lumen	catalytic activity;nucleotide binding	21.539	14.013	0.65
P32473	Pyruvate dehydrogenase E1 component subunit beta, mitochondrial [OS=Saccharomyces cerevisiae S288c]	852522	PDB1; YBR221C	metabolic process	mitochondrion;organelle lumen	catalytic activity	6.848	4.456	0.65
P07275	Delta-1-pyrroline-5-carboxylate dehydrogenase, mitochondrial [OS=Saccharomyces cerevisiae S288c]	856432	PUT2; YHR037W	metabolic process	membrane;mitochondrion;organelle lumen	catalytic activity	5.579	3.642	0.65
P53140	Protein RMD9, mitochondrial [OS=Saccharomyces cerevisiae S288c]	852771	RMD9; YGL107C	cell differentiation;metabolic process;regulation of biological process	membrane;mitochondrion	RNA binding	1.154	0.756	0.66
P19414	Aconitate hydratase, mitochondrial [OS=Saccharomyces cerevisiae S288c]	851013	ACO1; YLR304C	cell organization and biogenesis;metabolic process;transport	cytoplasm;cytosol;mitochondrion;organelle lumen	catalytic activity;DNA binding;metal ion binding	24.885	16.343	0.66
P40341	Mitochondrial respiratory chain complexes assembly protein YTA12 [OS=Saccharomyces cerevisiae S288c]	855114	YTA12; YMR089C	cell organization and biogenesis;metabolic process;response to stimulus;transport	cytoplasm;membrane;mitochondrion	catalytic activity;metal ion binding;nucleotide binding;protein binding	1.184	0.778	0.66
P32453	Protein ATP11, mitochondrial [OS=Saccharomyces cerevisiae S288c]	855401	ATP11; YNL315C	cell organization and biogenesis	mitochondrion	protein binding	1.512	0.995	0.66
P40033	37S ribosomal protein rsm18, mitochondrial [OS=Saccharomyces cerevisiae S288c]	856776	RSM18; YER050C	cell organization and biogenesis;metabolic process	mitochondrion;ribosome	structural molecule activity	1.512	0.995	0.66
P36526	54S ribosomal protein L27, mitochondrial [OS=Saccharomyces cerevisiae S288c]	852585	MRPL27; YBR282W	cell organization and biogenesis;metabolic process	mitochondrion;ribosome	structural molecule activity	1.512	0.995	0.66
P18900	hexaprenyl pyrophosphate synthase, mitochondrial [OS=Saccharomyces cerevisiae S288c]	852288	COQ1; YBR003W	metabolic process	membrane;mitochondrion	catalytic activity;metal ion binding;protein binding	0.711	0.468	0.66
P50085	Prohibitin-2 [OS=Saccharomyces cerevisiae S288c]	853146	PHB2; YGR231C	cell organization and biogenesis;metabolic process;regulation of biological process	membrane;mitochondrion	protein binding	3.806	2.511	0.66
P46367	Potassium-activated aldehyde dehydrogenase, mitochondrial [OS=Saccharomyces cerevisiae S288c]	854556	ALD4; YOR374W	metabolic process	mitochondrion;organelle lumen	catalytic activity	24.119	15.927	0.66
P48527	Tyrosine--tRNA ligase, mitochondrial [OS=Saccharomyces cerevisiae S288c]	856007	MSY1; YPL097W	metabolic process	cytoplasm;cytosol;mitochondrion;organelle lumen	catalytic activity;nucleotide binding;RNA binding	0.63	0.417	0.66
P38323	ATP-dependent clpX-like chaperone, mitochondrial [OS=Saccharomyces cerevisiae S288c]	852528	MCX1; YBR227C	metabolic process	membrane;mitochondrion;organelle lumen	catalytic activity;nucleotide binding	0.607	0.403	0.66
P23641	mitochondrial phosphate carrier protein [OS=Saccharomyces cerevisiae S288c]	853540	MIR1; YJR077C	metabolic process;transport	membrane;mitochondrion	protein binding;structural molecule activity;transporter activity	20.544	13.678	0.67
P37292	Serine hydroxymethyltransferase, mitochondrial [OS=Saccharomyces cerevisiae S288c]	852565	SHM1; YBR263W	metabolic process	mitochondrion	catalytic activity	7.697	5.136	0.67
P40495	Homoisocitrate dehydrogenase, mitochondrial [OS=Saccharomyces cerevisiae S288c]	854714	LYS12; YIL094C	metabolic process	mitochondrion	catalytic activity;metal ion binding;nucleotide binding	3.217	2.162	0.67
P30902	ATP synthase subunit d, mitochondrial [OS=Saccharomyces cerevisiae S288c]	853853	ATP7; YKL016C	metabolic process;transport	membrane;mitochondrion	catalytic activity;transporter activity	6.197	4.179	0.67
P38325	Mitochondrial outer membrane protein OM14 [OS=Saccharomyces cerevisiae S288c]	852531	OM14; YBR230C	transport	membrane;mitochondrion		6.197	4.179	0.67
P07143	Cytochrome c1, heme protein, mitochondrial [OS=Saccharomyces cerevisiae	854231	CYT1; YOR065W	metabolic process;transport	cytosol;membrane;mitochondrion	metal ion binding	13.251	9	0.68

	S288c]								
P21560	Protein CBP3, mitochondrial [OS=Saccharomyces cerevisiae S288c]	855886	CBP3; YPL215W	cell organization and biogenesis;regulation of biological process	membrane;mitochondrion;ribosome		1.031	0.701	0.68
P00560	Phosphoglycerate kinase [OS=Saccharomyces cerevisiae S288c]	850370	PGK1; YCR012W	metabolic process	cytoplasm;membrane;mitochondrion	catalytic activity;nucleotide binding	4.843	3.299	0.68
Q08179	Mitochondrial distribution and morphology protein 38 [OS=Saccharomyces cerevisiae S288c]	854130	MDM38; YOL027C	cell organization and biogenesis;cellular homeostasis;regulation of biological process;transport	membrane;mitochondrion		3.732	2.548	0.68
P07342	Acetolactate synthase catalytic subunit, mitochondrial [OS=Saccharomyces cerevisiae S288c]	855135	ILV2; YMR108W	metabolic process	mitochondrion	catalytic activity;metal ion binding;nucleotide binding	4.223	2.888	0.68
P17558	37S ribosomal protein PET123, mitochondrial [OS=Saccharomyces cerevisiae S288c]	854329	PET123; YOR158W	cell organization and biogenesis;metabolic process	mitochondrion;ribosome	structural molecule activity	2.594	1.783	0.69
P38341	MICOS complex subunit MIC12 [OS=Saccharomyces cerevisiae S288c]	852566	AIM5; MIC12; YBR262C	cell organization and biogenesis	membrane;mitochondrion		2.594	1.783	0.69
Q08822	Probable electron transfer flavoprotein-ubiquinone oxidoreductase, mitochondrial [OS=Saccharomyces cerevisiae S288c]	854538	CIR2; YOR356W	metabolic process;transport	membrane;mitochondrion	catalytic activity;metal ion binding;nucleotide binding	2.162	1.488	0.69
P06168	ketol-acid reductoisomerase, mitochondrial [OS=Saccharomyces cerevisiae S288c]	851069	ILV5; YLR355C	cell organization and biogenesis;metabolic process	mitochondrion	catalytic activity;DNA binding;metal ion binding	9	6.197	0.69
P19262	Dihydrolipoyllysine-residue succinyltransferase component of 2-oxoglutarate dehydrogenase complex, mitochondrial [OS=Saccharomyces cerevisiae S288c]	851726	KGD2; YDR148C	cell organization and biogenesis;metabolic process	mitochondrion	catalytic activity;protein binding	5.7	3.962	0.70
P01120	Ras-like protein 2 [OS=Saccharomyces cerevisiae S288c]	855625	RAS2; YNL098C	cell communication;cell differentiation;metabolic process;regulation of biological process;response to stimulus;transport	membrane;mitochondrion;nucleus	catalytic activity;nucleotide binding;protein binding	5.7	3.962	0.70
Q06236	Mitochondrial inner membrane protein SHH4 [OS=Saccharomyces cerevisiae S288c]	850861	SHH4; YLR164W	metabolic process	membrane;mitochondrion	catalytic activity;metal ion binding	4.623	3.217	0.70
Q02486	ARS-binding factor 2, mitochondrial [OS=Saccharomyces cerevisiae S288c]	855094	ABF2; YMR072W	cell organization and biogenesis;metabolic process;regulation of biological process	chromosome;mitochondrion;nucleus	DNA binding	69.17	48.239	0.70
P33311	ATP-dependent permease MDL2, mitochondrial [OS=Saccharomyces cerevisiae S288c]	855858	MDL2; YPL270W	response to stimulus;transport	membrane;mitochondrion	catalytic activity;nucleotide binding;transporter activity	1.006	0.708	0.70
P53982	isocitrate dehydrogenase [NADP] [OS=Saccharomyces cerevisiae S288c]	855723	IDP3; YNL009W	metabolic process	cytoplasm;mitochondrion	catalytic activity;metal ion binding;nucleotide binding	1.943	1.371	0.71
P00445	Superoxide dismutase [Cu-Zn] [OS=Saccharomyces cerevisiae S288c]	853568	SOD1; YJR104C	cell organization and biogenesis;cellular homeostasis;metabolic process;regulation of biological process;response to stimulus	cytoplasm;cytosol;mitochondrion;nucleus	antioxidant activity;catalytic activity;metal ion binding;protein binding	1.848	1.31	0.71
P02381	Ribosomal protein VAR1, mitochondrial [OS=Saccharomyces cerevisiae S288c]	854586	VAR1; Q0140	cell organization and biogenesis;metabolic process	mitochondrion;ribosome	structural molecule activity	0.52	0.369	0.71
Q12320	Hydroxyacylglutathione hydrolase, mitochondrial [OS=Saccharomyces cerevisiae S288c]	854205	GLO4; YOR040W	metabolic process	mitochondrion;organelle lumen	catalytic activity;metal ion binding	0.52	0.369	0.71
P52893	probable alanine aminotransferase, mitochondrial [OS=Saccharomyces cerevisiae S288c]	850778	ALT1; YLR089C	metabolic process	mitochondrion;organelle lumen	catalytic activity	0.951	0.682	0.72
P32266	Dynamin-like GTPase MGM1, mitochondrial [OS=Saccharomyces cerevisiae S288c]	854386	MGM1; YOR211C	cell organization and biogenesis	membrane;mitochondrion	catalytic activity;nucleotide binding;protein binding	0.389	0.28	0.72
P03879	Intron-encoded RNA maturase bI4 [OS=Saccharomyces cerevisiae S288c]	854582	BI4; Q0120	metabolic process	membrane;mitochondrion	catalytic activity;RNA binding	0.389	0.28	0.72
P53292	37S ribosomal protein S35, mitochondrial [OS=Saccharomyces cerevisiae S288c]	853075	MRPS35; YGR165W	cell organization and biogenesis;metabolic process	mitochondrion;ribosome	structural molecule activity	1.512	1.089	0.72
Q03028	Mitochondrial 2-oxodicarboxylate carrier 1 [OS=Saccharomyces cerevisiae S288c]	855969	ODC1; YPL134C	metabolic process;transport	membrane;mitochondrion	structural molecule activity;transporter activity	4.275	3.084	0.72

P81449	ATP synthase subunit e, mitochondrial [OS=Saccharomyces cerevisiae S288c]	851922	TIM11; YDR322C-A	cell organization and biogenesis;metabolic process;transport	membrane;mitochondrion	catalytic activity;structural molecule activity;transporter activity	9	6.499	0.72
Q12031	Mitochondrial 2-methylisocitrate lyase [OS=Saccharomyces cerevisiae S288c]	856114	ICL2; YPR006C	metabolic process	mitochondrion;organelle lumen	catalytic activity	3.833	2.793	0.73
P33421	Succinate dehydrogenase [ubiquinone] cytochrome b subunit, mitochondrial [OS=Saccharomyces cerevisiae S288c]	853716	SDH3; YKL141W	cell organization and biogenesis;metabolic process;transport	membrane;mitochondrion	catalytic activity;metal ion binding	1.276	0.931	0.73
Q08968	UPF0061 protein FMP40 [OS=Saccharomyces cerevisiae S288c]	855879	FMP40; YPL222W		mitochondrion	protein binding	2.162	1.588	0.73
P32335	Protein MSS51, mitochondrial [OS=Saccharomyces cerevisiae S288c]	850900	MSS51; YLR203C	metabolic process;regulation of biological process	membrane;mitochondrion	protein binding;translation regulator activity	1.154	0.848	0.73
P54003	Protein SUR7 [OS=Saccharomyces cerevisiae S288c]	854953	SUR7; YML052W	cell differentiation;transport	membrane;mitochondrion		1.154	0.848	0.73
P40098	uncharacterized mitochondrial membrane protein FMP10 [OS=Saccharomyces cerevisiae S288c]	856931	FMP10; YER182W		membrane;mitochondrion		4.456	3.281	0.74
P42847	37S ribosomal protein S18, mitochondrial [OS=Saccharomyces cerevisiae S288c]	855410	MRPS18; YNL306W	cell organization and biogenesis;metabolic process	mitochondrion;ribosome	RNA binding;structural molecule activity	1.054	0.778	0.74
P32904	54S ribosomal protein L6, mitochondrial [OS=Saccharomyces cerevisiae S288c]	856552	MRPL6; YHR147C	cell organization and biogenesis;metabolic process	mitochondrion;ribosome	RNA binding;structural molecule activity	1.054	0.778	0.74
P00431	cytochrome c peroxidase, mitochondrial [OS=Saccharomyces cerevisiae S288c]	853940	CCP1; YKR066C	metabolic process;response to stimulus	mitochondrion;organelle lumen	antioxidant activity;catalytic activity;metal ion binding;protein binding	1.649	1.219	0.74
P38988	Mitochondrial GTP/GDP carrier protein 1 [OS=Saccharomyces cerevisiae S288c]	851329	GGC1; YDL198C	cell organization and biogenesis;cellular homeostasis;metabolic process;transport	membrane;mitochondrion	structural molecule activity;transporter activity	3.16	2.34	0.74
P23644	Mitochondrial import receptor subunit TOM40 [OS=Saccharomyces cerevisiae S288c]	855243	TOM40; YMR203W	transport	cytosol;membrane;mitochondrion	protein binding;transporter activity	0.896	0.668	0.75
P02992	elongation factor Tu, mitochondrial [OS=Saccharomyces cerevisiae S288c]	854359	TUF1; YOR187W	cell organization and biogenesis;metabolic process	mitochondrion	catalytic activity;nucleotide binding;RNA binding	12.043	9	0.75
P36521	54S ribosomal protein L11, mitochondrial [OS=Saccharomyces cerevisiae S288c]	851325	MRPL11; YDL202W	cell organization and biogenesis;metabolic process	mitochondrion;ribosome	structural molecule activity	0.833	0.624	0.75
P21801	Succinate dehydrogenase [ubiquinone] iron-sulfur subunit, mitochondrial [OS=Saccharomyces cerevisiae S288c]	850685	SDH2; YLL041C	metabolic process;transport	membrane;mitochondrion	catalytic activity;metal ion binding	18.684	14.013	0.75
Q01852	Mitochondrial import inner membrane translocase subunit TIM44 [OS=Saccharomyces cerevisiae S288c]	854790	TIM44; YIL022W	transport	membrane;mitochondrion	nucleotide binding;protein binding	4.125	3.101	0.75
P14908	Mitochondrial transcription factor 1 [OS=Saccharomyces cerevisiae S288c]	855268	MTF1; YMR228W	metabolic process;regulation of biological process	mitochondrion;organelle lumen	catalytic activity;DNA binding;RNA binding	0.778	0.585	0.75
P38300	Inner membrane mitoribosome receptor MBA1, mitochondrial [OS=Saccharomyces cerevisiae S288c]	852483	MBA1; YBR185C	cell organization and biogenesis;regulation of biological process	membrane;mitochondrion		0.778	0.585	0.75
P09457	ATP synthase subunit 5, mitochondrial [OS=Saccharomyces cerevisiae S288c]	851892	ATP5; YDR298C	metabolic process;transport	membrane;mitochondrion	catalytic activity;transporter activity	10.365	7.799	0.75
P38072	Protein SCO2, mitochondrial [OS=Saccharomyces cerevisiae S288c]	852312	SCO2; YBR024W	cell organization and biogenesis;cellular homeostasis;metabolic process;transport	membrane;mitochondrion	antioxidant activity;catalytic activity;metal ion binding	0.73	0.551	0.75
P53219	Abhydrolase domain-containing protein IMO32 [OS=Saccharomyces cerevisiae S288c]	852919	IMO32; YGR031W	metabolic process;transport	mitochondrion	catalytic activity	0.73	0.551	0.75
P53266	Cytochrome oxidase assembly protein SHY1 [OS=Saccharomyces cerevisiae S288c]	853009	SHY1; YGR112W	cell organization and biogenesis	membrane;mitochondrion	protein binding	1.783	1.346	0.75
P07257	Cytochrome b-c1 complex subunit 2, mitochondrial [OS=Saccharomyces cerevisiae S288c]	856321	QCR2; YPR191W	metabolic process;transport	membrane;mitochondrion	catalytic activity;metal ion binding;protein binding;transporter activity	21.19	16.013	0.76

Q05648	MIOREX complex component 10 [OS=Saccharomyces cerevisiae S288c]	851876	YDR282C; MRX10; YDR282C		membrane;mitochondrion		0.65	0.492	0.76
P38088	Glycine--tRNA ligase 1, mitochondrial [OS=Saccharomyces cerevisiae S288c]			metabolic process	cytoplasm;mitochondrion;organelle lumen	catalytic activity;nucleotide binding;protein binding	0.616	0.468	0.76
Q03976	37S ribosomal protein S24, mitochondrial [OS=Saccharomyces cerevisiae S288c]	851755	RSM24; YDR175C	cell organization and biogenesis;metabolic process	mitochondrion;ribosome	structural molecule activity	0.585	0.445	0.76
Q03327	Mitochondrial fusion and transport protein Ugo1 [OS=Saccharomyces cerevisiae S288c]	852081	UGO1; YDR470C	cell organization and biogenesis;metabolic process	membrane;mitochondrion	protein binding;structural molecule activity	0.557	0.425	0.76
P36060	NADH-cytochrome b5 reductase 2 [OS=Saccharomyces cerevisiae S288c]	853707	MCR1; YKL150W	metabolic process;response to stimulus	membrane;mitochondrion	catalytic activity;protein binding	10.288	7.859	0.76
P40185	Protein mmf1, mitochondrial [OS=Saccharomyces cerevisiae S288c]	854760	MMF1; YIL051C	cell organization and biogenesis;metabolic process	mitochondrion;organelle lumen		6.943	5.31	0.76
P00927	Threonine dehydratase, mitochondrial [OS=Saccharomyces cerevisiae S288c]	856819	ILV1; YER086W	metabolic process	cytoplasm;mitochondrion	catalytic activity	2.257	1.728	0.77
P36141	Putative redox protein fmp46, mitochondrial [OS=Saccharomyces cerevisiae S288c]	853923	FMP46; YKR049C	metabolic process	mitochondrion	catalytic activity;protein binding	4.337	3.329	0.77
Q05931	Heat shock protein SSQ1, mitochondrial [OS=Saccharomyces cerevisiae S288c]	851084	SSQ1; YLR369W	cell organization and biogenesis;metabolic process	mitochondrion;organelle lumen	nucleotide binding;protein binding	0.884	0.679	0.77
P53598	Succinyl-CoA ligase [ADP-forming] subunit alpha, mitochondrial [OS=Saccharomyces cerevisiae S288c]	854310	LSC1; YOR142W	metabolic process	cytosol;mitochondrion	catalytic activity;nucleotide binding	13.384	10.288	0.77
P32331	Carrier protein YMC1, mitochondrial [OS=Saccharomyces cerevisiae S288c]	856171	YMC1; YPR058W	metabolic process;transport	membrane;mitochondrion;vacuole	structural molecule activity;transporter activity	1.254	0.968	0.77
P09440	C-1-tetrahydrofolate synthase, mitochondrial [OS=Saccharomyces cerevisiae S288c]	852378	MIS1; YBR084W	metabolic process	cytosol;mitochondrion	catalytic activity;nucleotide binding	0.9	0.696	0.77
P25374	Cysteine desulfurase, mitochondrial [OS=Saccharomyces cerevisiae S288c]	850343	NFS1; YCL017C	cell organization and biogenesis;cellular homeostasis;metabolic process	mitochondrion;nucleus	catalytic activity;metal ion binding;protein binding	2.3	1.783	0.78
P27697	Atypical kinase COQ8, mitochondrial [OS=Saccharomyces cerevisiae S288c]	852758	COQ8; YGL119W	metabolic process	membrane;mitochondrion;organelle lumen	catalytic activity;nucleotide binding	0.365	0.283	0.78
Q01217	Protein ARG5,6, mitochondrial [OS=Saccharomyces cerevisiae S288c]	856800	ARG5,6; YER069W	metabolic process;regulation of biological process	cytoplasm;mitochondrion;organelle lumen	catalytic activity;nucleotide binding;protein binding	0.233	0.182	0.78
Q06143	mitochondrial dicarboxylate transporter [OS=Saccharomyces cerevisiae S288c]	851063	DIC1; YLR348C	metabolic process;transport	membrane;mitochondrion	structural molecule activity;transporter activity	0.931	0.73	0.78
P16387	Pyruvate dehydrogenase E1 component subunit alpha, mitochondrial [OS=Saccharomyces cerevisiae S288c]	856925	PDA1; YER178W	cell growth;metabolic process	mitochondrion;organelle lumen	catalytic activity	12.46	9.771	0.78
P38797	Protein phosphatase 2C homolog 7, mitochondrial [OS=Saccharomyces cerevisiae S288c]	856475	PTC7; YHR076W	metabolic process	mitochondrion	catalytic activity;metal ion binding;protein binding	0.874	0.688	0.79
P36046	Mitochondrial intermembrane space import and assembly protein 40 [OS=Saccharomyces cerevisiae S288c]	853639	MIA40; YKL195W	cellular component movement;metabolic process;response to stimulus;transport	membrane;mitochondrion	catalytic activity;protein binding;transporter activity	2.914	2.3	0.79
P00447	Superoxide dismutase [Mn], mitochondrial [OS=Saccharomyces cerevisiae S288c]	856399	SOD2; YHR008C	metabolic process;response to stimulus	mitochondrion;organelle lumen	antioxidant activity;catalytic activity;metal ion binding	3.924	3.125	0.80
P53969	Sorting assembly machinery 50 kDa subunit [OS=Saccharomyces cerevisiae S288c]	855705	SAM50; YNL026W	cell organization and biogenesis;transport	membrane;mitochondrion	protein binding;transporter activity	0.61	0.487	0.80
P08466	mitochondrial nuclease [OS=Saccharomyces cerevisiae S288c]	853222	NUC1; YJL208C	cell death;metabolic process	membrane;mitochondrion;nucleus	catalytic activity;metal ion binding	1.336	1.069	0.80
Q12466	tricalbin-1 [OS=Saccharomyces cerevisiae S288c]	854253	TCB1; YOR086C	cell organization and biogenesis;regulation of biological process	endoplasmic reticulum;membrane;mitochondrion	metal ion binding;protein binding	0.52	0.417	0.80
P07253	cytochrome B pre-mRNA-processing protein 6	852417	CBP6; YBR120C	cell organization and biogenesis;metabolic	mitochondrion;ribosome		3.394	2.728	0.80

	[OS=Saccharomyces cerevisiae S288c]			process;regulation of biological process					
P15992	heat shock protein 26 [OS=Saccharomyces cerevisiae S288c]	852364	HSP26; YBR072W	metabolic process;response to stimulus	cytoplasm;mitochondrion;nucleus	protein binding;RNA binding	4.878	3.924	0.80
P37293	N-terminal acetyltransferase 2 [OS=Saccharomyces cerevisiae S288c]	853050	NAT2; YGR147C	metabolic process	cytoplasm;mitochondrion	catalytic activity	1.154	0.931	0.81
P33310	ATP-dependent permease MDL1, mitochondrial [OS=Saccharomyces cerevisiae S288c]	850885	MDL1; YLR188W	transport	membrane;mitochondrion	catalytic activity;nucleotide binding;transporter activity	0.438	0.354	0.81
Q07651	SUR7 family protein FMP45 [OS=Saccharomyces cerevisiae S288c]	851304	FMP45; YDL222C	cell differentiation;cell organization and biogenesis	membrane;mitochondrion		6.017	4.878	0.81
P19657	plasma membrane ATPase 2 [OS=Saccharomyces cerevisiae S288c]	856071	PMA2; YPL036W	cellular homeostasis;metabolic process;transport	membrane;mitochondrion	catalytic activity;metal ion binding;nucleotide binding;transporter activity	6.897	5.615	0.81
Q12166	2-isopropylmalate synthase 2, mitochondrial [OS=Saccharomyces cerevisiae S288c]	854275	LEU9; YOR108W	metabolic process	mitochondrion	catalytic activity;protein binding	1.707	1.39	0.81
P54783	D-arabinono-1,4-lactone oxidase [OS=Saccharomyces cerevisiae S288c]	854888	ALO1; YML086C	metabolic process;response to stimulus	membrane;mitochondrion	catalytic activity;nucleotide binding	3.299	2.687	0.81
P40008	Protein FMP52, mitochondrial [OS=Saccharomyces cerevisiae S288c]	856721	FMP52; YER004W		endoplasmic reticulum;membrane;mitochondrion	catalytic activity	2.652	2.162	0.82
P38127	mitochondrial carrier protein RIM2 [OS=Saccharomyces cerevisiae S288c]	852491	RIM2; YBR192W	cell organization and biogenesis;metabolic process;transport	membrane;mitochondrion	structural molecule activity;transporter activity	1.512	1.239	0.82
P19882	heat shock protein 60, mitochondrial [OS=Saccharomyces cerevisiae S288c]	850963	HSP60; YLR259C	cell organization and biogenesis;metabolic process;regulation of biological process;transport	cytoplasm;cytosol;membrane;mitochondrion;organelle lumen	catalytic activity;DNA binding;nucleotide binding;protein binding	13.384	10.994	0.82
P35191	DnaJ homolog 1, mitochondrial [OS=Saccharomyces cerevisiae S288c]	850530	MDJ1; YFL016C	cell organization and biogenesis;metabolic process;regulation of biological process;response to stimulus	mitochondrion;organelle lumen	enzyme regulator activity;metal ion binding;nucleotide binding;protein binding	2.765	2.275	0.82
P47045	mitochondrial import inner membrane translocase subunit TIM54 [OS=Saccharomyces cerevisiae S288c]	853392	TIM54; YJL054W	cell organization and biogenesis;transport	cytosol;membrane;mitochondrion	transporter activity	0.655	0.54	0.82
P32795	Mitochondrial inner membrane i-AAA protease supercomplex subunit YME1 [OS=Saccharomyces cerevisiae S288c]	856135	YME1; YPR024W	metabolic process;response to stimulus;transport	membrane;mitochondrion	catalytic activity;metal ion binding;nucleotide binding;protein binding	1.3	1.085	0.83
P32898	Mitochondrial presequence protease [OS=Saccharomyces cerevisiae S288c]	852041	CYM1; YDR430C	metabolic process	mitochondrion	catalytic activity;metal ion binding	0.812	0.682	0.84
Q04013	Citrate/oxoglutarate carrier protein [OS=Saccharomyces cerevisiae S288c]	855282	YHM2; YMR241W	cell organization and biogenesis;metabolic process;transport	membrane;mitochondrion	DNA binding;structural molecule activity;transporter activity	2.162	1.818	0.84
P33303	Succinate/fumarate mitochondrial transporter [OS=Saccharomyces cerevisiae S288c]	853558	SFC1; YJR095W	metabolic process;transport	membrane;mitochondrion	structural molecule activity;transporter activity	6.743	5.813	0.86
P49367	Homoaconitase, mitochondrial [OS=Saccharomyces cerevisiae S288c]	851820	LYS4; YDR234W	metabolic process	mitochondrion	catalytic activity;metal ion binding;protein binding	0.638	0.551	0.86
P16451	Pyruvate dehydrogenase complex protein X component, mitochondrial [OS=Saccharomyces cerevisiae S288c]	853107	PDX1; YGR193C	metabolic process	mitochondrion;organelle lumen	catalytic activity;structural molecule activity	2.3	2.03	0.88
P38077	ATP synthase subunit gamma, mitochondrial [OS=Saccharomyces cerevisiae S288c]	852327	ATP3; YBR039W	metabolic process;transport	membrane;mitochondrion	catalytic activity;transporter activity	9	8.006	0.89
P40012	protoporphyrinogen oxidase [OS=Saccharomyces cerevisiae S288c]	856733	HEM14; YER014W	metabolic process	membrane;mitochondrion	catalytic activity	1.198	1.069	0.89
Q01574	acetyl-coenzyme A synthetase 1 [OS=Saccharomyces cerevisiae S288c]	851245	ACS1; YAL054C	cell organization and biogenesis;metabolic process	cytoplasm;cytosol;endoplasmic reticulum;mitochondrion;nucleus	catalytic activity;nucleotide binding	3.281	3.03	0.92
P17505	Malate dehydrogenase, mitochondrial [OS=Saccharomyces cerevisiae S288c]	853777	MDH1; YKL085W	metabolic process	mitochondrion;organelle lumen	catalytic activity;protein binding;RNA binding	28.936	28.936	1.00
P39987	Heat shock protein SSC3, mitochondrial [OS=Saccharomyces cerevisiae	856682	ECM10; YEL030W	cell organization and biogenesis;metabolic process;transport	mitochondrion	nucleotide binding;protein binding	1.976	1.976	1.00

	S288c]								
Q03104	Meiotic sister chromatid recombination protein 1 [OS=Saccharomyces cerevisiae S288c]	854911	MSC1; YML128C	metabolic process	endoplasmic reticulum;membrane;mitochondrion		1.929	1.929	1.00
P04710	ADP,ATP carrier protein 1 [OS=Saccharomyces cerevisiae S288c]	855078	AAC1; YMR056C	metabolic process;transport	cytosol;membrane;mitochondrion	structural molecule activity;transporter activity	10.288	10.288	1.00
P05626	ATP synthase subunit 4, mitochondrial [OS=Saccharomyces cerevisiae S288c]	856027	ATP4; YPL078C	cell organization and biogenesis;metabolic process;transport	membrane;mitochondrion	catalytic activity;transporter activity	3.084	3.084	1.00
P10823	Guanine nucleotide-binding protein alpha-2 subunit [OS=Saccharomyces cerevisiae S288c]	856741	GPA2; YER020W	cell differentiation;cell growth;cell organization and biogenesis;cellular homeostasis;regulation of biological process;response to stimulus	cytosol;membrane;mitochondrion	catalytic activity;metal ion binding;nucleotide binding;protein binding;signal transducer activity	1.346	1.346	1.00
P42940	Probable electron transfer flavoprotein subunit beta [OS=Saccharomyces cerevisiae S288c]	853121	CIR1; YGR207C	metabolic process;transport	mitochondrion;organelle lumen		3.87	3.87	1.00
P00044	Cytochrome c iso-1 [OS=Saccharomyces cerevisiae S288c]	853507	CYC1; YJR048W	metabolic process;transport	mitochondrion	metal ion binding;protein binding	12.335	12.335	1.00
P46681	D-lactate dehydrogenase [cytochrome] 2, mitochondrial [OS=Saccharomyces cerevisiae S288c]	851376	DLD2; YDL178W	metabolic process	mitochondrion;organelle lumen	catalytic activity;nucleotide binding;protein binding	1.264	1.264	1.00
P19955	37S ribosomal protein YMR-31, mitochondrial [OS=Saccharomyces cerevisiae S288c]	850610	YMR31; YFR049W	cell organization and biogenesis;metabolic process	mitochondrion;ribosome	catalytic activity;protein binding;structural molecule activity	15.681	15.681	1.00
P00410	Cytochrome c oxidase subunit 2 [OS=Saccharomyces cerevisiae S288c]	854622	COX2; Q0250	metabolic process;transport	membrane;mitochondrion	catalytic activity;metal ion binding;protein binding;transporter activity	3.642	3.642	1.00
P32332	Mitochondrial oxaloacetate transport protein [OS=Saccharomyces cerevisiae S288c]	853739	OAC1; YKL120W	metabolic process;transport	membrane;mitochondrion	structural molecule activity;transporter activity	2.594	2.594	1.00
Q04728	Arginine biosynthesis bifunctional protein ArgJ, mitochondrial [OS=Saccharomyces cerevisiae S288c]	855084	ARG7; YMR062C	metabolic process	mitochondrion;organelle lumen	catalytic activity	0.63	0.63	1.00
Q08223	Altered inheritance of mitochondria protein 39, mitochondrial [OS=Saccharomyces cerevisiae S288c]	854103	AIM39; YOL053W		cytosol;membrane;mitochondrion		0.995	0.995	1.00
Q12289	mitochondrial carnitine carrier [OS=Saccharomyces cerevisiae S288c]	854267	CRC1; YOR100C	metabolic process;transport	membrane;mitochondrion	structural molecule activity;transporter activity	0.931	0.931	1.00
P32799	Cytochrome c oxidase subunit 6A, mitochondrial [OS=Saccharomyces cerevisiae S288c]	852684	COX13; YGL191W	cell organization and biogenesis;metabolic process;regulation of biological process;transport	membrane;mitochondrion	catalytic activity;enzyme regulator activity;transporter activity	11.589	11.589	1.00
P37298	Succinate dehydrogenase [ubiquinone] cytochrome b small subunit, mitochondrial [OS=Saccharomyces cerevisiae S288c]	851758	SDH4; YDR178W	metabolic process;transport	membrane;mitochondrion	catalytic activity;metal ion binding	3.642	3.642	1.00
Q03798	Altered inheritance of mitochondria protein 36, mitochondrial [OS=Saccharomyces cerevisiae S288c]	855189	AIM36; YMR157C		membrane;mitochondrion		1.336	1.336	1.00
P38523	GrpE protein homolog, mitochondrial [OS=Saccharomyces cerevisiae S288c]	854407	MGE1; YOR232W	metabolic process;regulation of biological process;transport	membrane;mitochondrion;organelle lumen	enzyme regulator activity;nucleotide binding;protein binding	0.833	0.833	1.00
P10507	mitochondrial-processing peptidase subunit beta [OS=Saccharomyces cerevisiae S288c]	850860	MAS1; YLR163C	metabolic process;transport	mitochondrion;organelle lumen	catalytic activity;metal ion binding;protein binding	0.778	0.778	1.00
P22354	54S ribosomal protein L20, mitochondrial [OS=Saccharomyces cerevisiae S288c]	853960	MRPL20; YKR085C	cell organization and biogenesis;metabolic process	mitochondrion;ribosome	structural molecule activity	2.162	2.162	1.00
P25038	Translation initiation factor IF-2, mitochondrial [OS=Saccharomyces cerevisiae S288c]	854135	IFM1; YOL023W	cell organization and biogenesis;metabolic process	mitochondrion;nucleus	catalytic activity;nucleotide binding;RNA binding	0.299	0.299	1.00
P41903	Peroxisomal acyl-coenzyme A thioester hydrolase 1 [OS=Saccharomyces cerevisiae S288c]	853477	TES1; YJR019C	metabolic process	mitochondrion	catalytic activity	0.931	0.931	1.00

P25372	Thioredoxin-3, mitochondrial [OS=Saccharomyces cerevisiae S288c]	850444	TRX3; YCR083W	cellular homeostasis;metabolic process;regulation of biological process;response to stimulus;transport	cytoplasm;mitochondrion	catalytic activity	2.511	2.511	1.00
Q3E824	Uncharacterized protein YOR020W-A [OS=Saccharomyces cerevisiae S288c]	1466480	YOR020W-A; YOR020W-A	metabolic process;transport	membrane;mitochondrion		3.642	3.642	1.00
P32611	54S ribosomal protein RML2, mitochondrial [OS=Saccharomyces cerevisiae S288c]	856660	RML2; YEL050C	cell organization and biogenesis;metabolic process	mitochondrion;ribosome	catalytic activity;RNA binding;structural molecule activity	0.638	0.638	1.00
Q06493	LETM1 domain-containing protein YLH47, mitochondrial [OS=Saccharomyces cerevisiae S288c]	856243	YLH47; YPR125W	cell organization and biogenesis;transport	membrane;mitochondrion		0.61	0.61	1.00
P11325	Leucine--tRNA ligase, mitochondrial [OS=Saccharomyces cerevisiae S288c]	851098	NAM2; YLR382C	cell organization and biogenesis;metabolic process;regulation of biological process	cytosol;mitochondrion;organelle lumen	catalytic activity;nucleotide binding;RNA binding	0.208	0.208	1.00
P53889	uncharacterized mitochondrial hydrolase FMP41 [OS=Saccharomyces cerevisiae S288c]	855553	FMP41; YNL168C	metabolic process	mitochondrion	catalytic activity;metal ion binding	0.501	0.501	1.00
Q01519	Cytochrome c oxidase subunit 6b [OS=Saccharomyces cerevisiae S288c]	850727	COX12; YLR038C	cell organization and biogenesis;metabolic process;transport	mitochondrion	catalytic activity;transporter activity	3.642	3.642	1.00
P23369	54S ribosomal protein L25, mitochondrial [OS=Saccharomyces cerevisiae S288c]	852967	MRPL25; YGR076C	cell organization and biogenesis;metabolic process	mitochondrion;ribosome	structural molecule activity	1.31	1.31	1.00
P21375	Fumarate reductase 2 [OS=Saccharomyces cerevisiae S288c]	853510	OSM1; YJR051W	metabolic process	endoplasmic reticulum;mitochondrion	catalytic activity;nucleotide binding	0.346	0.346	1.00
P37299	cytochrome b-c1 complex subunit 10 [OS=Saccharomyces cerevisiae S288c]	856390	QCR10; YHR001W-A	metabolic process;transport	membrane;mitochondrion	catalytic activity;transporter activity	5.31	5.31	1.00
P36147	Presequence translocated-associated motor subunit pam17, mitochondrial [OS=Saccharomyces cerevisiae S288c]	853939	PAM17; YKR065C	transport	membrane;mitochondrion		1.371	1.371	1.00
Q02608	37S ribosomal protein S16, mitochondrial [OS=Saccharomyces cerevisiae S288c]	856094	MRPS16; YPL013C	cell organization and biogenesis;metabolic process	mitochondrion;ribosome	structural molecule activity	0.931	0.931	1.00
Q03246	37S ribosomal protein S17, mitochondrial [OS=Saccharomyces cerevisiae S288c]	855226	MRPS17; YMR188C	cell differentiation;cell organization and biogenesis;metabolic process	mitochondrion;ribosome	RNA binding;structural molecule activity	0.931	0.931	1.00
P32792	UPF0744 protein YSC83 [OS=Saccharomyces cerevisiae S288c]	856410	YSC83; YHR017W		membrane;mitochondrion		0.369	0.369	1.00
Q04172	Sensitive to high expression protein 9, mitochondrial [OS=Saccharomyces cerevisiae S288c]	852002	SHE9; YDR393W	cell organization and biogenesis	membrane;mitochondrion		0.269	0.269	1.00
P40530	[Pyruvate dehydrogenase (acetyl-transferring)] kinase 1, mitochondrial [OS=Saccharomyces cerevisiae S288c]	854769	PKP1; YIL042C	metabolic process	mitochondrion;organelle lumen	catalytic activity;nucleotide binding;protein binding	0.551	0.551	1.00
Q6Q560	Protein isd11 [OS=Saccharomyces cerevisiae S288c]	856774	ISD11; YER048W-A	cell organization and biogenesis;metabolic process	mitochondrion;organelle lumen	catalytic activity;protein binding	2.594	2.594	1.00
P28737	Protein MSP1 [OS=Saccharomyces cerevisiae S288c]	852915	MSP1; YGR028W	cell organization and biogenesis;metabolic process;regulation of biological process;response to stimulus;transport	membrane;mitochondrion;nucleus	catalytic activity;nucleotide binding;protein binding;RNA binding	0.585	0.585	1.00
Q02883	N-acyl-phosphatidylethanolamine-hydrolyzing phospholipase D, mitochondrial [OS=Saccharomyces cerevisiae S288c]	856001	FMP30; YPL103C	metabolic process	membrane;mitochondrion	catalytic activity;metal ion binding	0.304	0.304	1.00
P21306	ATP synthase subunit epsilon, mitochondrial [OS=Saccharomyces cerevisiae S288c]	855857	ATP15; YPL271W	metabolic process;transport	membrane;mitochondrion	catalytic activity;transporter activity	2.162	2.162	1.00
Q03799	37S ribosomal protein S8, mitochondrial [OS=Saccharomyces cerevisiae S288c]	855190	MRPS8; YMR158W	cell organization and biogenesis;metabolic process	mitochondrion;ribosome	structural molecule activity	0.701	0.701	1.00
P32785	Methionyl-tRNA formyltransferase, mitochondrial [OS=Saccharomyces cerevisiae S288c]	852270	FMT1; YBL013W	metabolic process	mitochondrion	catalytic activity	0.179	0.179	1.00
P46998	mitochondrial membrane protein FMP33 [OS=Saccharomyces	853279	FMP33; YJL161W		membrane;mitochondrion		0.778	0.778	1.00

	cerevisiae S288c]								
Q12305	Thiosulfate sulfurtransferase RDL1, mitochondrial [OS=Saccharomyces cerevisiae S288c]	854459	RDL1; YOR285W		endoplasmic reticulum;membrane;mitochondrion	catalytic activity	0.668	0.668	1.00
P18409	Mitochondrial distribution and morphology protein 10 [OS=Saccharomyces cerevisiae S288c]	851223	MDM10; YAL010C	cell organization and biogenesis;transport	membrane;mitochondrion	protein binding	0.16	0.16	1.00
P36523	54S ribosomal protein L15, mitochondrial [OS=Saccharomyces cerevisiae S288c]	851022	MRPL15; YLR312W-A	cell organization and biogenesis;metabolic process	mitochondrion;ribosome	catalytic activity;RNA binding;structural molecule activity	0.334	0.334	1.00
P0CX44	60S ribosomal protein L1-B [OS=Saccharomyces cerevisiae S288c]	852742; 855881	RPL1B; RPL1A; YGL135W; YPL220W	metabolic process	cytoplasm;ribosome	RNA binding;structural molecule activity	0.425	0.425	1.00
P20084	54S ribosomal protein L33, mitochondrial [OS=Saccharomyces cerevisiae S288c]	855330	MRPL33; YMR286W	cell organization and biogenesis;metabolic process	mitochondrion;ribosome	structural molecule activity	2.162	2.162	1.00
P01098	ATPase-stabilizing factor 9 kDa, mitochondrial [OS=Saccharomyces cerevisiae S288c]	851426	STF1; YDL130W-A	metabolic process;regulation of biological process	mitochondrion	catalytic activity;enzyme regulator activity;protein binding	2.162	2.162	1.00
Q08645	folylpolyglutamate synthase [OS=Saccharomyces cerevisiae S288c]	854415	MET7; YOR241W	metabolic process	cytoplasm;endoplasmic reticulum;membrane;mitochondrion;organelle lumen	catalytic activity;nucleotide binding	0.233	0.233	1.00
P34222	Peptidyl-tRNA hydrolase 2 [OS=Saccharomyces cerevisiae S288c]	852223	PTH2; YBL057C	regulation of biological process	cytoplasm;cytosol;membrane;mitochondrion	catalytic activity;protein binding	0.425	0.425	1.00
P40581	peroxiredoxin HYR1 [OS=Saccharomyces cerevisiae S288c]	854855	HYR1; YIR037W	metabolic process;response to stimulus	cytoplasm;cytosol;mitochondrion;organelle lumen	antioxidant activity;catalytic activity	0.995	0.995	1.00
P53875	54S ribosomal protein L19, mitochondrial [OS=Saccharomyces cerevisiae S288c]	855536	MRPL19; YNL185C	cell organization and biogenesis;metabolic process	mitochondrion;ribosome	RNA binding;structural molecule activity	0.874	0.874	1.00
P53163	54S ribosomal protein L12, mitochondrial [OS=Saccharomyces cerevisiae S288c]	852811	MNP1; YGL068W	cell organization and biogenesis;metabolic process	mitochondrion;ribosome	RNA binding;structural molecule activity	0.52	0.52	1.00
P42844	Mitochondrial protein import protein ZIM17 [OS=Saccharomyces cerevisiae S288c]	855406	ZIM17; YNL310C	cell organization and biogenesis;metabolic process;regulation of biological process;response to stimulus;transport	membrane;mitochondrion;organelle lumen	metal ion binding;protein binding	0.585	0.585	1.00
Q06510	Lysophosphatidylcholine acyltransferase [OS=Saccharomyces cerevisiae S288c]	856262	TAZ1; YPR140W	cell organization and biogenesis;metabolic process;transport	membrane;mitochondrion	catalytic activity	0.194	0.194	1.00
P38860	GTPase MTG2, mitochondrial [OS=Saccharomyces cerevisiae S288c]	856573	MTG2; YHR168W	metabolic process;transport	membrane;mitochondrion	catalytic activity;metal ion binding;nucleotide binding;transporter activity	0.07	0.07	1.00
Q06405	ATP synthase subunit f, mitochondrial [OS=Saccharomyces cerevisiae S288c]	851983	ATP17; YDR377W	metabolic process;transport	membrane;mitochondrion	catalytic activity;transporter activity	2.981	2.981	1.00
Q12204	Probable phospholipase YOR022C, mitochondrial [OS=Saccharomyces cerevisiae S288c]	854187	YOR022C; YOR022C	metabolic process	mitochondrion	catalytic activity;metal ion binding	0.11	0.11	1.00
P32606	putative mitochondrial translation system component PET127 [OS=Saccharomyces cerevisiae S288c]	854182	PET127; YOR017W	cell organization and biogenesis;metabolic process;regulation of biological process	mitochondrion		0.099	0.099	1.00
Q05779	Ubiquinone biosynthesis protein COQ9, mitochondrial [OS=Saccharomyces cerevisiae S288c]	850898	COQ9; YLR201C	metabolic process	membrane;mitochondrion		0.359	0.359	1.00
Q3E6R5	uncharacterized mitochondrial outer membrane protein YDR381C-A [OS=Saccharomyces cerevisiae S288c]	851989	YDR381C-A; YDR381C-A		membrane;mitochondrion		0.389	0.389	1.00
Q04487	Mitochondrial inner membrane protein SHH3 [OS=Saccharomyces cerevisiae S288c]	855145	SHH3; YMR118C	metabolic process	membrane;mitochondrion	catalytic activity;metal ion binding	0.425	0.425	1.00
Q07821	Iron-sulfur assembly protein 1 [OS=Saccharomyces cerevisiae S288c]	850632	ISA1; YLL027W	cell organization and biogenesis;metabolic process	mitochondrion;organelle lumen	metal ion binding;structural molecule activity	0.136	0.136	1.00
P53157	Mitochondrial pyruvate carrier 1 [OS=Saccharomyces cerevisiae S288c]	852800	FMP37; MPC1; YGL080W	transport	membrane;mitochondrion	transporter activity	1.154	1.154	1.00

Q12487	54S ribosomal protein L23, mitochondrial [OS=Saccharomyces cerevisiae S288c]	854321	MRPL23; YOR150W	cell organization and biogenesis;metabolic process	mitochondrion;ribosome;vacuole	RNA binding;structural molecule activity	0.52	0.52	1.00
Q2V2P9	Uncharacterized protein YDR119W-A [OS=Saccharomyces cerevisiae S288c]	3799970	COX26; YDR119W-A		membrane;mitochondrion		1.154	1.154	1.00
P40458	Autophagy-related protein 32 [OS=Saccharomyces cerevisiae S288c]	854660	ATG32; YIL146C	metabolic process	membrane;mitochondrion;vacuole	protein binding	0.202	0.202	1.00
P53733	37S ribosomal protein S19, mitochondrial [OS=Saccharomyces cerevisiae S288c]	855773	RSM19; YNR037C	cell organization and biogenesis;metabolic process	mitochondrion;ribosome	RNA binding;structural molecule activity	0.931	0.931	1.00
P32939	GTP-binding protein ypt7 [OS=Saccharomyces cerevisiae S288c]	855012	YPT7; YML001W	cell communication;cell organization and biogenesis;metabolic process;regulation of biological process;response to stimulus;transport	cytosol;endosome;membrane;mitochondrion;vacuole	catalytic activity;nucleotide binding;protein binding	0.166	0.166	1.00
P09950	5-aminolevulinate synthase, mitochondrial [OS=Saccharomyces cerevisiae S288c]	851818	HEM1; YDR232W	metabolic process	mitochondrion;organelle lumen	catalytic activity	0.086	0.086	1.00
P48526	Isoleucine--tRNA ligase, mitochondrial [OS=Saccharomyces cerevisiae S288c]	856067	ISM1; YPL040C	cell organization and biogenesis;metabolic process;regulation of biological process	cytoplasm;cytosol;mitochondrion;organelle lumen	catalytic activity;nucleotide binding	0.04	0.04	1.00
P53193	J-type co-chaperone JAC1, mitochondrial [OS=Saccharomyces cerevisiae S288c]	852866	JAC1; YGL018C	cell organization and biogenesis;metabolic process;transport	membrane;mitochondrion;organelle lumen	protein binding	0.212	0.212	1.00
P32378	4-hydroxybenzoate polyprenyltransferase, mitochondrial [OS=Saccharomyces cerevisiae S288c]	855778	COQ2; YNR041C	metabolic process;transport	membrane;mitochondrion	catalytic activity	0.292	0.292	1.00
P39515	mitochondrial import inner membrane translocase subunit Tim17 [OS=Saccharomyces cerevisiae S288c]	853298	TIM17; YJL143W	cell organization and biogenesis;transport	membrane;mitochondrion	protein binding;transporter activity	0.778	0.778	1.00
P36064	COX assembly mitochondrial protein [OS=Saccharomyces cerevisiae S288c]	853721	CMC1; YKL137W	cell organization and biogenesis	membrane;mitochondrion	metal ion binding	0.389	0.389	1.00
P54857	lipase 2 [OS=Saccharomyces cerevisiae S288c]	851628	TGL2; YDR058C	metabolic process;transport	mitochondrion	catalytic activity	0.233	0.233	1.00
Q06005	Octanoyltransferase, mitochondrial [OS=Saccharomyces cerevisiae S288c]	850940	LIP2; YLR239C	metabolic process	cytoplasm;mitochondrion	catalytic activity	0.116	0.116	1.00
P38812	Phosphatidylglycerophosphatase GEP4, mitochondrial [OS=Saccharomyces cerevisiae S288c]	856500	GEP4; YHR100C	metabolic process	membrane;mitochondrion;organelle lumen	catalytic activity	0.179	0.179	1.00
Q3E846	Cytochrome c oxidase assembly factor 6 [OS=Saccharomyces cerevisiae S288c]	855287	COA6; YMR244C-A; YMR244C-A	cell organization and biogenesis;cellular homeostasis;metabolic process;transport	cytoplasm;membrane;mitochondrion;nucleus	catalytic activity;metal ion binding;protein binding;transporter activity	0.931	0.931	1.00
P03876	putative COX1/OXI3 intron 2 protein [OS=Saccharomyces cerevisiae S288c]	854594	AI2; Q0055	metabolic process;transport	mitochondrion	catalytic activity;metal ion binding;transporter activity	0.086	0.086	1.00
P40491	ATP synthase assembly factor fmc1, mitochondrial [OS=Saccharomyces cerevisiae S288c]	854709	FMC1; YIL098C	cell communication;cell organization and biogenesis;metabolic process;response to stimulus	mitochondrion;organelle lumen		0.468	0.468	1.00
P10174	cytochrome c oxidase subunit 7 [OS=Saccharomyces cerevisiae S288c]	855298	COX7; YMR256C	metabolic process;transport	membrane;mitochondrion	catalytic activity;transporter activity	1.154	1.154	1.00
P22135	Protein ATP12, mitochondrial [OS=Saccharomyces cerevisiae S288c]	853259	ATP12; YJL180C	cell organization and biogenesis	mitochondrion	protein binding	0.101	0.101	1.00
P53318	Ubiquinone biosynthesis monooxygenase COQ6, mitochondrial [OS=Saccharomyces cerevisiae S288c]	853170	COQ6; YGR255C	metabolic process	membrane;mitochondrion	catalytic activity;nucleotide binding	0.077	0.077	1.00
P32463	Acyl carrier protein, mitochondrial [OS=Saccharomyces cerevisiae S288c]	853642	ACP1; YKL192C	metabolic process;transport	mitochondrion		0.259	0.259	1.00
P04039	Cytochrome c oxidase polypeptide VIII, mitochondrial [OS=Saccharomyces cerevisiae S288c]	851111	COX8; YLR395C	metabolic process;transport	membrane;mitochondrion	catalytic activity;transporter activity	0.778	0.778	1.00
P53212	Probable transcriptional regulatory protein HAH1 [OS=Saccharomyces cerevisiae S288c]	852904	YGR021W; YGR021W		mitochondrion		0.166	0.166	1.00

Q3E7A4	COX assembly mitochondrial protein 2 [OS=Saccharomyces cerevisiae S288c]	852220	CMC2; YBL059C-A	cell organization and biogenesis	cytoplasm;membrane;mitochondrion;nucleus	metal ion binding	0.389	0.389	1.00
P81451	ATP synthase subunit K, mitochondrial [OS=Saccharomyces cerevisiae S288c]	854077	ATP19; YOL077W-A	cell organization and biogenesis;metabolic process;transport	membrane;mitochondrion	catalytic activity;transporter activity	1.154	1.154	1.00
P39103	Cytochrome c oxidase assembly protein COX14 [OS=Saccharomyces cerevisiae S288c]	854910	COX14; YML129C	cell organization and biogenesis;regulation of biological process	membrane;mitochondrion;organelle lumen	protein binding	0.585	0.585	1.00
P27882	Mitochondrial FAD-linked sulfhydryl oxidase erv1 [OS=Saccharomyces cerevisiae S288c]	852916	ERV1; YGR029W	cellular homeostasis;metabolic process;response to stimulus;transport	mitochondrion	catalytic activity;protein binding	0.212	0.212	1.00
P53320	Mitochondrial carrier protein MTM1 [OS=Saccharomyces cerevisiae S288c]	853173	MTM1; YGR257C	metabolic process;transport	membrane;mitochondrion	structural molecule activity	0.11	0.11	1.00
P53312	Succinyl-CoA ligase [ADP-forming] subunit beta, mitochondrial [OS=Saccharomyces cerevisiae S288c]	853159	LSC2; YGR244C	metabolic process	mitochondrion	catalytic activity;nucleotide binding	7.003	7.62	1.09
P07256	Cytochrome b-c1 complex subunit 1, mitochondrial [OS=Saccharomyces cerevisiae S288c]	852235	COR1; YBL045C	metabolic process;transport	membrane;mitochondrion	catalytic activity;metal ion binding;protein binding;transporter activity	9.89	11.915	1.20
P08067	cytochrome b-c1 complex subunit Rieske, mitochondrial [OS=Saccharomyces cerevisiae S288c]	856689	RIP1; YEL024W	metabolic process;transport	membrane;mitochondrion	catalytic activity;metal ion binding;transporter activity	25.102	30.623	1.22
P40508	Uncharacterized protein YIL077C [OS=Saccharomyces cerevisiae S288c]	854733	YIL077C; YIL077C		mitochondrion		1.154	1.448	1.25
P10834	protein PET54 [OS=Saccharomyces cerevisiae S288c]	853137	PET54; YGR222W	metabolic process;regulation of biological process	membrane;mitochondrion;organelle lumen	nucleotide binding;RNA binding;translation regulator activity	0.688	0.874	1.27
P40364	Mitochondrial peculiar membrane protein 1 [OS=Saccharomyces cerevisiae S288c]	853379	MPM1; YJL066C		membrane;mitochondrion		1.512	1.929	1.28
P00427	Cytochrome c oxidase subunit 6, mitochondrial [OS=Saccharomyces cerevisiae S288c]	856448	COX6; YHR051W	metabolic process;transport	membrane;mitochondrion	catalytic activity;metal ion binding;transporter activity	2.831	3.642	1.29
Q12298	uncharacterized ABC transporter ATP-binding protein YDR061W [OS=Saccharomyces cerevisiae S288c]	851633	YDR061W; YDR061W		mitochondrion	catalytic activity;nucleotide binding	0.266	0.343	1.29
P39952	Mitochondrial inner membrane protein OXA1 [OS=Saccharomyces cerevisiae S288c]	856898	OXA1; YER154W	cell organization and biogenesis;transport	membrane;mitochondrion	transporter activity	1.054	1.371	1.30
Q08970	Mitochondrial metal transporter 2 [OS=Saccharomyces cerevisiae S288c]	855877	MMT2; YPL224C	cellular homeostasis;regulation of biological process;response to stimulus;transport	membrane;mitochondrion	transporter activity	0.407	0.532	1.31
P53230	Phosphatidate cytidylyltransferase, mitochondrial [OS=Saccharomyces cerevisiae S288c]	852937	TAM41; YGR046W	metabolic process	membrane;mitochondrion;organelle lumen	catalytic activity	0.407	0.532	1.31
P36775	Lon protease homolog, mitochondrial [OS=Saccharomyces cerevisiae S288c]	852259	PIM1; YBL022C	cell organization and biogenesis;metabolic process;regulation of biological process;response to stimulus	cytoplasm;mitochondrion;organelle lumen	catalytic activity;DNA binding;nucleotide binding;RNA binding	0.685	0.905	1.32
P53220	Mitochondrial import inner membrane translocase subunit TIM21 [OS=Saccharomyces cerevisiae S288c]	852921	TIM21; YGR033C	cell organization and biogenesis;transport	membrane;mitochondrion	protein binding	0.719	0.968	1.35
P39722	Mitochondrial Rho GTPase 1 [OS=Saccharomyces cerevisiae S288c]	851249	GEM1; YAL048C	cell organization and biogenesis;regulation of biological process;response to stimulus;transport	membrane;mitochondrion	catalytic activity;metal ion binding;nucleotide binding	0.189	0.259	1.37
P50087	MICOS subunit MIC26 [OS=Saccharomyces cerevisiae S288c]	853150	MOS2; MIC26; YGR235C	cell organization and biogenesis	membrane;mitochondrion		0.931	1.276	1.37
P25039	Elongation factor G, mitochondrial [OS=Saccharomyces cerevisiae S288c]	850758	MEF1; YLR069C	cell organization and biogenesis;metabolic process	cytosol;mitochondrion	catalytic activity;nucleotide binding;RNA binding	0.151	0.207	1.37
Q08245	protein ZEO1 [OS=Saccharomyces cerevisiae S288c]	854040	ZEO1; YOL109W	cell organization and biogenesis	membrane;mitochondrion	protein binding	2.162	2.981	1.38
P38705	Serine--tRNA ligase, mitochondrial [OS=Saccharomyces cerevisiae S288c]	856402	DIA4; YHR011W	metabolic process	cytoplasm;mitochondrion;organelle lumen	catalytic activity;nucleotide binding	0.233	0.322	1.38

Q03557	Glutamyl-tRNA(Gln) amidotransferase subunit A, mitochondrial [OS=Saccharomyces cerevisiae S288c]	855338	HER2; YMR293C	cell organization and biogenesis;metabolic process	mitochondrion	catalytic activity;nucleotide binding	0.259	0.359	1.39
P47150	37S ribosomal protein S7, mitochondrial [OS=Saccharomyces cerevisiae S288c]	853578	RSM7; YJR113C	cell organization and biogenesis;metabolic process	mitochondrion;ribosome	RNA binding;structural molecule activity	0.334	0.468	1.40
P00424	Cytochrome c oxidase polypeptide 5A, mitochondrial [OS=Saccharomyces cerevisiae S288c]	855675	COX5A; YNL052W	metabolic process;transport	membrane;mitochondrion	catalytic activity;transporter activity	1.31	1.848	1.41
P43594	MICOS complex subunit MIC19 [OS=Saccharomyces cerevisiae S288c]	850563	AIM13; MIC19; YFR011C	cell organization and biogenesis	cytoplasm;membrane;mitochondrion		1.31	1.848	1.41
P19516	Cytochrome c oxidase assembly protein COX11, mitochondrial [OS=Saccharomyces cerevisiae S288c]	855971	COX11; YPL132W	cell organization and biogenesis;metabolic process	membrane;mitochondrion;ribosome	metal ion binding	0.438	0.624	1.42
P38910	10 kDa heat shock protein, mitochondrial [OS=Saccharomyces cerevisiae S288c]	854185	HSP10; YOR020C	cell organization and biogenesis;metabolic process;response to stimulus;transport	cytoplasm;mitochondrion;organelle lumen	metal ion binding;nucleotide binding;protein binding	3.217	4.623	1.44
P39525	3-oxoacyl-[acyl-carrier-protein] synthase homolog [OS=Saccharomyces cerevisiae S288c]	856790	CEM1; YER061C	metabolic process	mitochondrion	catalytic activity	1.081	1.565	1.45
Q02950	37S ribosomal protein MRP51, mitochondrial [OS=Saccharomyces cerevisiae S288c]	855985	MRP51; YPL118W	cell organization and biogenesis;metabolic process	mitochondrion;ribosome	structural molecule activity	0.668	0.978	1.46
P10662	37S ribosomal protein MRP1, mitochondrial [OS=Saccharomyces cerevisiae S288c]	851948	MRP1; YDR347W	cell organization and biogenesis;metabolic process;response to stimulus	mitochondrion;ribosome	antioxidant activity;catalytic activity;metal ion binding;structural molecule activity	1.239	1.818	1.47
P39965	probable proline--tRNA ligase, mitochondrial [OS=Saccharomyces cerevisiae S288c]	856820	AIM10; YER087W	metabolic process	cytoplasm;mitochondrion	catalytic activity;nucleotide binding;RNA binding	0.125	0.194	1.55
P29704	squalene synthase [OS=Saccharomyces cerevisiae S288c]	856597	ERG9; YHR190W	metabolic process	endoplasmic reticulum;membrane;mitochondrion	catalytic activity	0.222	0.35	1.58
Q06485	Autophagy-related protein 33 [OS=Saccharomyces cerevisiae S288c]	851070	ATG33; YLR356W	cell communication;metabolic process;response to stimulus	membrane;mitochondrion		1.371	2.162	1.58
Q04689	Altered inheritance of mitochondria protein 32 [OS=Saccharomyces cerevisiae S288c]	854955	AIM32; YML050W		mitochondrion		0.233	0.369	1.58
P36534	54S ribosomal protein L40, mitochondrial [OS=Saccharomyces cerevisiae S288c]	855930	MRPL40; YPL173W	cell organization and biogenesis;metabolic process	mitochondrion;ribosome	protein binding;structural molecule activity	0.778	1.239	1.59
P41735	5-demethoxyubiquinone hydroxylase, mitochondrial [OS=Saccharomyces cerevisiae S288c]	854292	CAT5; YOR125C	metabolic process	membrane;mitochondrion	catalytic activity;metal ion binding	0.334	0.54	1.62
Q96VH5	MICOS complex subunit Mic10 [OS=Saccharomyces cerevisiae S288c]	850300	MOS1; MIC10; YCL057C-A	cell organization and biogenesis	membrane;mitochondrion		1.683	2.728	1.62
P00942	Triosephosphate isomerase [OS=Saccharomyces cerevisiae S288c]	851620	TPI1; YDR050C	metabolic process	cytoplasm;cytosol;membrane;mitochondrion	catalytic activity	0.425	0.701	1.65
Q03201	37S ribosomal protein S10, mitochondrial [OS=Saccharomyces cerevisiae S288c]	851611	RSM10; YDR041W	cell organization and biogenesis;metabolic process	mitochondrion;ribosome	structural molecule activity	0.425	0.701	1.65
P40515	Mitochondria fission 1 protein [OS=Saccharomyces cerevisiae S288c]	854745	FIS1; YIL065C	cell death;cell organization and biogenesis;regulation of biological process	membrane;mitochondrion	protein binding	0.468	0.778	1.66
P00425	Cytochrome c oxidase polypeptide 5B, mitochondrial [OS=Saccharomyces cerevisiae S288c]	854695	COX5B; YIL111W	metabolic process;transport	membrane;mitochondrion	catalytic activity;transporter activity	0.52	0.874	1.68
P42949	Mitochondrial import inner membrane translocase subunit TIM16 [OS=Saccharomyces cerevisiae S288c]	853340	PAM16; YJL104W	transport	membrane;mitochondrion	protein binding	0.52	0.874	1.68
P33759	37S ribosomal protein S5, mitochondrial [OS=Saccharomyces cerevisiae S288c]	852553	MRPS5; YBR251W	cell organization and biogenesis;metabolic process	mitochondrion;ribosome	RNA binding;structural molecule activity	0.413	0.778	1.88
P81450	ATP synthase subunit J, mitochondrial [OS=Saccharomyces cerevisiae S288c]	854893	ATP18; YML081C-A	cell organization and biogenesis;metabolic process;transport	membrane;mitochondrion	catalytic activity;transporter activity	4.623	9	1.95

P34231	Uncharacterized protein YKL187C [OS=Saccharomyces cerevisiae S288c]	853648	FAT3; YKL187C ; YKL187C	cell organization and biogenesis;transport	membrane;mitochondrion		0.093	0.194	2.09
P50110	Sorting assembly machinery 37 kDa subunit [OS=Saccharomyces cerevisiae S288c]	855082	SAM37; YMR060C	cell organization and biogenesis;transport	membrane;mitochondrion	protein binding	0.116	0.245	2.11
P38816	thioredoxin reductase 2, mitochondrial [OS=Saccharomyces cerevisiae S288c]	856506	TRR2; YHR106W	cellular homeostasis;metabolic process;regulation of biological process;response to stimulus	cytoplasm;mitochondrion	antioxidant activity;catalytic activity	0.136	0.292	2.15
P43122	tRNA N6-adenosine threonylcarbamoyltransferase, mitochondrial [OS=Saccharomyces cerevisiae S288c]	851454	QRI7; YDL104C	cell organization and biogenesis;metabolic process	mitochondrion	catalytic activity;metal ion binding	0.212	0.468	2.21
P49334	Mitochondrial import receptor subunit tom22 [OS=Saccharomyces cerevisiae S288c]	855592	TOM22; YNL131W	cell organization and biogenesis;transport	membrane;mitochondrion	protein binding;transporter activity	0.292	0.668	2.29
Q12032	Altered inheritance of mitochondria protein 41, mitochondrial [OS=Saccharomyces cerevisiae S288c]	854390	AIM41; YOR215C		mitochondrion	catalytic activity	1.154	2.831	2.45
P01097	ATPase inhibitor, mitochondrial [OS=Saccharomyces cerevisiae S288c]	851347	INH1; YDL181W	metabolic process;regulation of biological process	mitochondrion	catalytic activity;enzyme regulator activity;protein binding	0.468	1.154	2.47
P36525	54S ribosomal protein L24, mitochondrial [OS=Saccharomyces cerevisiae S288c]	855231	MRPL24; YMR193W	cell organization and biogenesis;metabolic process	mitochondrion;ribosome	RNA binding;structural molecule activity	1.154	2.981	2.58
Q03713	Respiratory supercomplex factor 1, mitochondrial [OS=Saccharomyces cerevisiae S288c]	854978	RCF1; YML030W	cell organization and biogenesis	membrane;mitochondrion		1.512	4.012	2.65
P13433	DNA-directed RNA polymerase, mitochondrial [OS=Saccharomyces cerevisiae S288c]	850507	RPO41; YFL036W	cell organization and biogenesis;metabolic process	cytoplasm;mitochondrion;organelle lumen	catalytic activity;DNA binding	0.027	0.085	3.15
P35996	54S ribosomal protein L38, mitochondrial [OS=Saccharomyces cerevisiae S288c]	853684	MRPL38; YKL170W	cell organization and biogenesis;metabolic process	mitochondrion;ribosome	RNA binding;structural molecule activity	1.154	3.642	3.16
P21771	37S ribosomal protein S28, mitochondrial [OS=Saccharomyces cerevisiae S288c]	851937	MRPS28 ; YDR337W	cell organization and biogenesis;metabolic process	mitochondrion;ribosome	RNA binding;structural molecule activity	0.624	1.976	3.17
P00045	Cytochrome c iso-2 [OS=Saccharomyces cerevisiae S288c]	856672	CYC7; YEL039C	metabolic process;transport	mitochondrion	metal ion binding	1.512	6.943	4.59
P07255	Cytochrome c oxidase subunit 7A [OS=Saccharomyces cerevisiae S288c]	851492	COX9; YDL067C	metabolic process;transport	membrane;mitochondrion	catalytic activity;transporter activity	1.154	20.544	17.80

Supplemental Table S4. The relative concentrations of proteins in mitochondria purified from WT or *ups1Δ* cells cultured without LCA. Mitochondria were purified from WT or *ups1Δ* cells recovered on day 2 of cell culturing without LCA. Mass spectrometry-based identification and quantitation of proteins recovered in purified mitochondria were performed as described in Materials and methods. The exponentially modified protein abundance index (emPAI) provided by the ″Proteome Discoverer″ software was used to calculate the relative abundance of mitochondrial proteins between specified datasets. Data on the relative concentrations of mitochondrial proteins are presented as means of 2 independent experiments.

Accession	Description	Entrez Gene ID	Gene ID	Biological Process	Cellular Component	Molecular Function	emPAI WT	emPAI *ups1*	Ratio *ups1* WT
P10834	protein PET54	853137	PET54	metabolic process; regulation of biological process	membrane; mitochondrion; organelle lumen	nucleotide binding; RNA binding; translation regulator activity	0.874	0.11	0.13
P12687	54S ribosomal protein L2, mitochondrial [OS=Saccharomyces cerevisiae S288c]	855727	MRP7; YNL005C	cell organization and biogenesis;metabolic process	mitochondrion;ribosome	catalytic activity;RNA binding;structural molecule activity	1.848	0.233	0.13
Q06892	NADH kinase POS5, mitochondrial [OS=Saccharomyces cerevisiae S288c]	855913	POS5; YPL188W	metabolic process;response to stimulus	mitochondrion;organelle lumen	catalytic activity;nucleotide binding	2.511	0.369	0.15
P42847	37S ribosomal protein S18, mitochondrial [OS=Saccharomyces cerevisiae S288c]	855410	MRPS18; YNL306W	cell organization and biogenesis;metabolic process	mitochondrion;ribosome	RNA binding;structural molecule activity	1.054	0.155	0.15
P46998	mitochondrial membrane protein FMP33 [OS=Saccharomyces cerevisiae S288c]	853279	FMP33; YJL161W		membrane;mitochondrion		2.162	0.334	0.15
P21771	37S ribosomal protein S28, mitochondrial [OS=Saccharomyces cerevisiae S288c]	851937	MRPS28; YDR337W	cell organization and biogenesis;metabolic process	mitochondrion;ribosome	RNA binding;structural molecule activity	0.833	0.129	0.15
P43594	MICOS complex subunit MIC19 [OS=Saccharomyces cerevisiae S288c]	850563	AIM13; MIC19; YFR011C	cell organization and biogenesis	cytoplasm;membrane;mitochondrion		3.329	0.52	0.16
P40364	Mitochondrial peculiar membrane protein 1 [OS=Saccharomyces cerevisiae S288c]	853379	MPM1; YJL066C		membrane;mitochondrion		3.642	0.585	0.16
P53170	[Pyruvate dehydrogenase (acetyl-transferring)] kinase 2, mitochondrial [OS=Saccharomyces cerevisiae S288c]	852821	PKP2; YGL059W	metabolic process;regulation of biological process	mitochondrion;organelle lumen	catalytic activity;nucleotide binding;protein binding	1.096	0.179	0.16
P47150	37S ribosomal protein S7, mitochondrial [OS=Saccharomyces cerevisiae S288c]	853578	RSM7; YJR113C	cell organization and biogenesis;metabolic process	mitochondrion;ribosome	RNA binding;structural molecule activity	0.616	0.101	0.16
Q03557	Glutamyl-tRNA(Gln) amidotransferase subunit A, mitochondrial [OS=Saccharomyces cerevisiae S288c]	855338	HER2; YMR293C	cell organization and biogenesis;metabolic process	mitochondrion	catalytic activity;nucleotide binding	0.468	0.08	0.17
P32445	Single-stranded DNA-binding protein RIM1, mitochondrial [OS=Saccharomyces cerevisiae S288c]	850395	RIM1; YCR028C-A	cell organization and biogenesis;metabolic process;regulation of biological process	mitochondrion	DNA binding	6.743	1.154	0.17
P02381	Ribosomal protein VAR1, mitochondrial [OS=Saccharomyces cerevisiae S288c]	854586	VAR1; Q0140	cell organization and biogenesis;metabolic process	mitochondrion;ribosome	structural molecule activity	1.31	0.233	0.18
Q01217	Protein ARG5,6, mitochondrial [OS=Saccharomyces cerevisiae S288c]	856800	ARG5,6; YER069W	metabolic process;regulation of biological process	cytoplasm;mitochondrion;organelle lumen	catalytic activity;nucleotide binding;protein binding	0.458	0.087	0.19
P39525	3-oxoacyl-[acyl-carrier-protein] synthase homolog [OS=Saccharomyces cerevisiae S288c]	856790	CEM1; YER061C	metabolic process	mitochondrion	catalytic activity	1.848	0.369	0.20
Q12283	Malonyl CoA-acyl carrier protein transacylase, mitochondrial [OS=Saccharomyces cerevisiae S288c]	854396	MCT1; YOR221C	metabolic process	mitochondrion	catalytic activity	0.668	0.136	0.20
P25372	Thioredoxin-3, mitochondrial [OS=Saccharomyces cerevisiae	850444	TRX3; YCR083	cellular homeostasis;metabolic	cytoplasm;mitochondrion	catalytic activity	2.511	0.52	0.21

	S288c]		W	process;regulation of biological process;response to stimulus;transport					
P33416	Heat shock protein 78, mitochondrial [OS=Saccharomyces cerevisiae S288c]	851845	HSP78; YDR258C	cell organization and biogenesis;metabolic process;regulation of biological process;response to stimulus	mitochondrion;organelle lumen	catalytic activity;nucleotide binding;protein binding	12.525	2.609	0.21
P38122	3-methyl-2-oxobutanoate hydroxymethyltransferase [OS=Saccharomyces cerevisiae S288c]	852474	ECM31; YBR176 W	metabolic process	mitochondrion	catalytic activity	0.585	0.122	0.21
P32331	Carrier protein YMC1, mitochondrial [OS=Saccharomyces cerevisiae S288c]	856171	YMC1; YPR058 W	metabolic process;transport	membrane;mitochondrion;vacuole	structural molecule activity;transporte r activity	2.384	0.501	0.21
P00447	Superoxide dismutase [Mn], mitochondrial [OS=Saccharomyces cerevisiae S288c]	856399	SOD2; YHR008C	metabolic process;response to stimulus	mitochondrion;organelle lumen	antioxidant activity;catalytic activity;metal ion binding	9	1.894	0.21
P49017	2-methoxy-6-polyprenyl-1,4-benzoquinol methylase, mitochondrial [OS=Saccharomyces cerevisiae S288c]	854930	COQ5; YML110C	metabolic process	membrane;mitochondrion;organelle lumen	catalytic activity	0.551	0.116	0.21
P36528	54S ribosomal protein L17, mitochondrial [OS=Saccharomyces cerevisiae S288c]	855469	MRPL17; YNL252C	cell organization and biogenesis;metabolic process	mitochondrion;ribosome	structural molecule activity	0.52	0.11	0.21
P22135	Protein ATP12, mitochondrial [OS=Saccharomyces cerevisiae S288c]	853259	ATP12; YJL180C	cell organization and biogenesis	mitochondrion	protein binding	0.468	0.101	0.22
P53305	Mitochondrial 37S ribosomal protein S27 [OS=Saccharomyces cerevisiae S288c]	853129	RSM27; YGR215 W	cell organization and biogenesis;metabolic process	mitochondrion;ribosome	structural molecule activity	2.162	0.468	0.22
P11325	Leucine--tRNA ligase, mitochondrial [OS=Saccharomyces cerevisiae S288c]	851098	NAM2; YLR382C	cell organization and biogenesis;metabolic process;regulation of biological process	cytosol;mitochondrion;organelle lumen	catalytic activity;nucleotide binding;RNA binding	0.353	0.078	0.22
P18496	Mitochondrial ATPase complex subunit ATP10 [OS=Saccharomyces cerevisiae S288c]	851109	ATP10; YLR393 W	cell organization and biogenesis	cytoplasm;membrane;mitochondrion	protein binding	1.818	0.413	0.23
P25375	Saccharolysin [OS=Saccharomyces cerevisiae S288c]	850301	PRD1; YCL057 W	metabolic process	cytoplasm;Golgi;mitochondrion;vacuole	catalytic activity;metal ion binding	0.216	0.05	0.23
P19955	37S ribosomal protein YMR-31, mitochondrial [OS=Saccharomyces cerevisiae S288c]	850610	YMR31; YFR049 W	cell organization and biogenesis;metabolic process	mitochondrion;ribosome	catalytic activity;protein binding;structural molecule activity	15.681	3.642	0.23
P32898	Mitochondrial presequence protease [OS=Saccharomyces cerevisiae S288c]	852041	CYM1; YDR430C	metabolic process	mitochondrion	catalytic activity;metal ion binding	2.162	0.505	0.23
P25605	Acetolactate synthase small subunit, mitochondrial [OS=Saccharomyces cerevisiae S288c]	850348	ILV6; YCL009C	metabolic process;regulation of biological process	mitochondrion	catalytic activity;enzyme regulator activity	11.743	2.793	0.24
P81450	ATP synthase subunit J, mitochondrial [OS=Saccharomyces cerevisiae S288c]	854893	ATP18; YML081C -A	cell organization and biogenesis;metabolic process;transport	membrane;mitochondrion	catalytic activity;transporte r activity	9	2.162	0.24
Q03430	37S ribosomal protein RSM28, mitochondrial [OS=Saccharomyces cerevisiae S288c]	852105	RSM28; YDR494 W	cell organization and biogenesis;metabolic process	mitochondrion;ribosome	structural molecule activity	2.675	0.65	0.24
Q12305	Thiosulfate sulfurtransferase RDL1, mitochondrial [OS=Saccharomyces cerevisiae S288c]	854459	RDL1; YOR285 W		endoplasmic reticulum;membrane;mitochondrion	catalytic activity	1.154	0.292	0.25
P19516	Cytochrome c oxidase assembly protein COX11, mitochondrial [OS=Saccharomyces cerevisiae S288c]	855971	COX11; YPL132 W	cell organization and biogenesis;metabolic process	membrane;mitochondrion;ribosome	metal ion binding	1.069	0.274	0.26
P35996	54S ribosomal protein L38, mitochondrial [OS=Saccharomyces cerevisiae S288c]	853684	MRPL38; YKL170 W	cell organization and biogenesis;metabolic process	mitochondrion;ribosome	RNA binding;structural molecule activity	2.594	0.668	0.26
P36516	54S ribosomal protein L3, mitochondrial [OS=Saccharomyces cerevisiae S288c]	855039	MRPL3; YMR024 W	cell organization and biogenesis;metabolic process;response to stimulus	mitochondrion;nucleus;ribosome	catalytic activity;RNA binding;structural molecule activity	1.043	0.269	0.26
P37293	N-terminal acetyltransferase 2 [OS=Saccharomyces cerevisiae S288c]	853050	NAT2; YGR147C	metabolic process	cytoplasm;mitochondrion	catalytic activity	0.931	0.245	0.26
P38523	GrpE protein homolog, mitochondrial [OS=Saccharomyces cerevisiae S288c]	854407	MGE1; YOR232 W	metabolic process;regulation of biological process;transport	membrane;mitochondrion;organelle lumen	enzyme regulator activity;nucleotide binding;protein binding	2.36	0.624	0.26

P36534	54S ribosomal protein L40, mitochondrial [OS=Saccharomyces cerevisiae S288c]	855930	MRPL40; YPL173W	cell organization and biogenesis;metabolic process	mitochondrion;ribosome	protein binding;structural molecule activity	1.512	0.413	0.27
P53881	54S ribosomal protein L22, mitochondrial [OS=Saccharomyces cerevisiae S288c]	855544	MRPL22; YNL177C	cell organization and biogenesis;metabolic process	mitochondrion;ribosome	RNA binding;structural molecule activity	1.994	0.551	0.28
P32799	Cytochrome c oxidase subunit 6A, mitochondrial [OS=Saccharomyces cerevisiae S288c]	852684	COX13; YGL191W	cell organization and biogenesis;metabolic process;regulation of biological process;transport	membrane;mitochondrion	catalytic activity;enzyme regulator activity;transporter activity	18.953	5.31	0.28
Q06630	Mitochondrial homologous recombination protein 1 [OS=Saccharomyces cerevisiae S288c]	851890	MHR1; YDR296W	cell organization and biogenesis;metabolic process;regulation of biological process;response to stimulus	mitochondrion;nucleus	catalytic activity;DNA binding;structural molecule activity	0.585	0.166	0.28
P32897	Mitochondrial import inner membrane translocase subunit tim23 [OS=Saccharomyces cerevisiae S288c]	855751	TIM23; YNR017W	transport	membrane;mitochondrion	protein binding;transporter activity	5.31	1.512	0.28
P36519	54S ribosomal protein L7, mitochondrial [OS=Saccharomyces cerevisiae S288c]	851823	MRPL7; YDR237W	cell organization and biogenesis;metabolic process	mitochondrion;ribosome	RNA binding;structural molecule activity	0.468	0.136	0.29
P40165	NAD(P)H-hydrate epimerase [OS=Saccharomyces cerevisiae S288c]	855521	NNR1; YNL200C	metabolic process	cytoplasm;mitochondrion	catalytic activity;metal ion binding;nucleotide binding	1.61	0.468	0.29
Q12032	Altered inheritance of mitochondria protein 41, mitochondrial [OS=Saccharomyces cerevisiae S288c]	854390	AIM41; YOR215C		mitochondrion	catalytic activity	1.61	0.468	0.29
P27697	Atypical kinase COQ8, mitochondrial [OS=Saccharomyces cerevisiae S288c]	852758	COQ8; YGL119W	metabolic process	membrane;mitochondrion;organelle lumen	catalytic activity;nucleotide binding	0.453	0.133	0.29
P36139	protein PET10 [OS=Saccharomyces cerevisiae S288c]	853920	PET10; YKR046C	metabolic process	membrane		0.413	0.122	0.30
Q12165	ATP synthase subunit delta, mitochondrial [OS=Saccharomyces cerevisiae S288c]	851560	ATP16; YDL004W	metabolic process;transport	membrane;mitochondrion	catalytic activity;transporter activity	4.623	1.371	0.30
Q07534	Solute carrier family 25 member 38 homolog [OS=Saccharomyces cerevisiae S288c]	851439	HEM25; YDL119C	metabolic process;transport	membrane;mitochondrion	structural molecule activity;transporter activity	0.389	0.116	0.30
P40035	Mitochondrial phosphate carrier protein 2 [OS=Saccharomyces cerevisiae S288c]	856779	PIC2; YER053C	cellular homeostasis;metabolic process;transport	membrane;mitochondrion	structural molecule activity;transporter activity	2.793	0.833	0.30
P25626	54S ribosomal protein IMG1, mitochondrial [OS=Saccharomyces cerevisiae S288c]	850413	IMG1; YCR046C	cell organization and biogenesis;metabolic process	mitochondrion;ribosome	structural molecule activity	1.424	0.425	0.30
Q03201	37S ribosomal protein S10, mitochondrial [OS=Saccharomyces cerevisiae S288c]	851611	RSM10; YDR041W	cell organization and biogenesis;metabolic process	mitochondrion;ribosome	structural molecule activity	1.424	0.425	0.30
Q03799	37S ribosomal protein S8, mitochondrial [OS=Saccharomyces cerevisiae S288c]	855190	MRPS8; YMR158W	cell organization and biogenesis;metabolic process	mitochondrion;ribosome	structural molecule activity	1.424	0.425	0.30
P47039	Probable kynurenine--oxoglutarate transaminase BNA3 [OS=Saccharomyces cerevisiae S288c]	853386	BNA3; YJL060W	metabolic process;regulation of biological process	cytoplasm;mitochondrion	catalytic activity	0.334	0.101	0.30
P22354	54S ribosomal protein L20, mitochondrial [OS=Saccharomyces cerevisiae S288c]	853960	MRPL20; YKR085C	cell organization and biogenesis;metabolic process	mitochondrion;ribosome	structural molecule activity	1.276	0.389	0.30
P32266	Dynamin-like GTPase MGM1, mitochondrial [OS=Saccharomyces cerevisiae S288c]	854386	MGM1; YOR211C	cell organization and biogenesis	membrane;mitochondrion	catalytic activity;nucleotide binding;protein binding	0.28	0.086	0.31
P40051	Intermediate cleaving peptidase 55 [OS=Saccharomyces cerevisiae S288c]	856811	ICP55; YER078C	metabolic process;regulation of biological process	membrane;mitochondrion;nucleus	catalytic activity;metal ion binding	0.25	0.077	0.31
P10849	mitochondrial transcription factor 2 [OS=Saccharomyces cerevisiae S288c]	851517	MTF2; YDL044C	metabolic process	mitochondrion;organelle lumen	protein binding;RNA binding	0.259	0.08	0.31
P07236	Threonine--tRNA ligase, mitochondrial [OS=Saccharomyces cerevisiae S288c]	853640	MST1; YKL194C	metabolic process	cytoplasm;mitochondrion;organelle lumen	catalytic activity;nucleotide binding;RNA binding	0.233	0.072	0.31
P47158	Putative transferase CAF17, mitochondrial	853586	IBA57; YJR122W	cell organization and biogenesis;metabolic	mitochondrion;organelle lumen	catalytic activity	0.241	0.075	0.31

	[OS=Saccharomyces cerevisiae S288c]			process					
P10507	mitochondrial-processing peptidase subunit beta [OS=Saccharomyces cerevisiae S288c]	850860	MAS1; YLR163C	metabolic process;transport	mitochondrion;organelle lumen	catalytic activity;metal ion binding;protein binding	2.481	0.778	0.31
P32860	NifU-like protein, mitochondrial [OS=Saccharomyces cerevisiae S288c]	853826	NFU1; YKL040C	cell organization and biogenesis;cellular homeostasis;metabolic process	mitochondrion;organelle lumen	metal ion binding	5.494	1.738	0.32
P35999	Mitochondrial intermediate peptidase [OS=Saccharomyces cerevisiae S288c]	853724	OCT1; YKL134C	cellular homeostasis;metabolic process;regulation of biological process	mitochondrion;organelle lumen	catalytic activity;metal ion binding	0.145	0.046	0.32
P25039	Elongation factor G, mitochondrial [OS=Saccharomyces cerevisiae S288c]	850758	MEF1; YLR069C	cell organization and biogenesis;metabolic process	cytosol;mitochondrion	catalytic activity;nucleotide binding;RNA binding	1.024	0.326	0.32
Q07500	External NADH-ubiquinone oxidoreductase 2, mitochondrial [OS=Saccharomyces cerevisiae S288c]	851474	NDE2; YDL085 W	metabolic process	mitochondrion	catalytic activity;nucleotide binding	12.594	4.012	0.32
P11914	Mitochondrial-processing peptidase subunit alpha [OS=Saccharomyces cerevisiae S288c]	856419	MAS2; YHR024C	metabolic process;transport	membrane;mitochondrion;organelle lumen	catalytic activity;metal ion binding;protein binding	2.311	0.738	0.32
P00431	cytochrome c peroxidase, mitochondrial [OS=Saccharomyces cerevisiae S288c]	853940	CCP1; YKR066C	metabolic process;response to stimulus	mitochondrion;organelle lumen	antioxidant activity;catalytic activity;metal ion binding;protein binding	2.162	0.701	0.32
P04803	Tryptophan--tRNA ligase, mitochondrial [OS=Saccharomyces cerevisiae S288c]	851861	MSW1; YDR268 W	metabolic process	cytoplasm;mitochondrion;organelle lumen	catalytic activity;nucleotide binding	0.896	0.292	0.33
P37267	Assembly factor cbp4 [OS=Saccharomyces cerevisiae S288c]		CBP4	cell organization and biogenesis	membrane;mitochondrion		5.579	1.848	0.33
P14908	Mitochondrial transcription factor 1 [OS=Saccharomyces cerevisiae S288c]	855268	MTF1; YMR228 W	metabolic process;regulation of biological process	mitochondrion;organelle lumen	catalytic activity;DNA binding;RNA binding	0.778	0.259	0.33
P53736	Uncharacterized protein YNR040W [OS=Saccharomyces cerevisiae S288c]	855776	YNR040 W; YNR040 W		mitochondrion		1.154	0.389	0.34
Q08970	Mitochondrial metal transporter 2 [OS=Saccharomyces cerevisiae S288c]	855877	MMT2; YPL224C	cellular homeostasis;regulation of biological process;response to stimulus;transport	membrane;mitochondrion	transporter activity	0.532	0.186	0.35
P06168	ketol-acid reductoisomerase, mitochondrial [OS=Saccharomyces cerevisiae S288c]	851069	ILV5; YLR355C	cell organization and biogenesis;metabolic process	mitochondrion	catalytic activity;DNA binding;metal ion binding	28.126	9.857	0.35
P35191	DnaJ homolog 1, mitochondrial [OS=Saccharomyces cerevisiae S288c]	850530	MDJ1; YFL016C	cell organization and biogenesis;metabolic process;regulation of biological process;response to stimulus	mitochondrion;organelle lumen	enzyme regulator activity;metal ion binding;nucleotide binding;protein binding	4.722	1.656	0.35
P36046	Mitochondrial intermembrane space import and assembly protein 40 [OS=Saccharomyces cerevisiae S288c]	853639	MIA40; YKL195 W	cellular component movement;metabolic process;response to stimulus;transport	membrane;mitochondrion	catalytic activity;protein binding;transporte r activity	2.3	0.817	0.36
Q06485	Autophagy-related protein 33 [OS=Saccharomyces cerevisiae S288c]	851070	ATG33; YLR356 W	cell communication;metabolic process;response to stimulus	membrane;mitochondrion		2.162	0.778	0.36
P39522	Dihydroxy-acid dehydratase, mitochondrial [OS=Saccharomyces cerevisiae S288c]	853473	ILV3; YJR016C	metabolic process	mitochondrion	catalytic activity;metal ion binding	39.37	14.199	0.36
Q12428	Probable 2-methylcitrate dehydratase [OS=Saccharomyces cerevisiae S288c]	856108	PDH1; YPR002 W	metabolic process	cytoplasm;membrane;mitochondrion	catalytic activity	19.535	7.058	0.36
P18900	hexaprenyl pyrophosphate synthase, mitochondrial [OS=Saccharomyces cerevisiae S288c]	852288	COQ1; YBR003 W	metabolic process	membrane;mitochondrion	catalytic activity;metal ion binding;protein binding	0.711	0.259	0.36
Q08223	Altered inheritance of mitochondria protein 39, mitochondrial [OS=Saccharomyces cerevisiae S288c]	854103	AIM39; YOL053 W		cytosol;membrane;mitochondrion		0.711	0.259	0.36
Q08968	UPF0061 protein FMP40 [OS=Saccharomyces cerevisiae S288c]	855879	FMP40; YPL222 W		mitochondrion	protein binding	2.495	0.917	0.37
P32332	Mitochondrial oxaloacetate transport protein [OS=Saccharomyces cerevisiae	853739	OAC1; YKL120 W	metabolic process;transport	membrane;mitochondrion	structural molecule activity;transporte	5.813	2.162	0.37

	S288c]					r activity			
P46681	D-lactate dehydrogenase [cytochrome] 2, mitochondrial [OS=Saccharomyces cerevisiae S288c]	851376	DLD2; YDL178W	metabolic process	mitochondrion;organelle lumen	catalytic activity;nucleotide binding;protein binding	1.829	0.682	0.37
P23833	Protein SCO1, mitochondrial [OS=Saccharomyces cerevisiae S288c]	852325	SCO1; YBR037C	cell organization and biogenesis;cellular homeostasis;response to stimulus;transport	membrane;mitochondrion	antioxidant activity;catalytic activity;metal ion binding	1.783	0.668	0.37
P00927	Threonine dehydratase, mitochondrial [OS=Saccharomyces cerevisiae S288c]	856819	ILV1; YER086W	metabolic process	cytoplasm;mitochondrion	catalytic activity	3.375	1.285	0.38
P16451	Pyruvate dehydrogenase complex protein X component, mitochondrial [OS=Saccharomyces cerevisiae S288c]	853107	PDX1; YGR193C	metabolic process	mitochondrion;organelle lumen	catalytic activity;structural molecule activity	4.055	1.555	0.38
P23180	Probable oxidoreductase AIM17 [OS=Saccharomyces cerevisiae S288c]	856365	AIM17; YHL021C	cell organization and biogenesis;metabolic process	mitochondrion	catalytic activity;metal ion binding	9	3.467	0.39
P12686	37S ribosomal protein MRP13, mitochondrial [OS=Saccharomyces cerevisiae S288c]	852975	MRP13; YGR084C	cell organization and biogenesis;metabolic process	mitochondrion;ribosome	structural molecule activity	1.512	0.585	0.39
P32453	Protein ATP11, mitochondrial [OS=Saccharomyces cerevisiae S288c]	855401	ATP11; YNL315C	cell organization and biogenesis	mitochondrion	protein binding	1.512	0.585	0.39
P02992	elongation factor Tu, mitochondrial [OS=Saccharomyces cerevisiae S288c]	854359	TUF1; YOR187W	cell organization and biogenesis;metabolic process	mitochondrion	catalytic activity;nucleotide binding;RNA binding	23.245	9	0.39
Q02204	54S ribosomal protein L13, mitochondrial [OS=Saccharomyces cerevisiae S288c]	853875	MRPL13; YKR006C	cell organization and biogenesis;metabolic process	mitochondrion;ribosome	structural molecule activity	1.371	0.54	0.39
P17695	Glutaredoxin-2, mitochondrial [OS=Saccharomyces cerevisiae S288c]	852124	GRX2; YDR513W	cellular homeostasis;metabolic process;regulation of biological process;response to stimulus;transport	cytoplasm;cytosol;mitochondrion;nucleus	antioxidant activity;catalytic activity	1.31	0.52	0.40
P36527	54S ribosomal protein L28, mitochondrial [OS=Saccharomyces cerevisiae S288c]	852073	MRPL28; YDR462W	cell organization and biogenesis;metabolic process	mitochondrion;ribosome	structural molecule activity	1.31	0.52	0.40
P23369	54S ribosomal protein L25, mitochondrial [OS=Saccharomyces cerevisiae S288c]	852967	MRPL25; YGR076C	cell organization and biogenesis;metabolic process	mitochondrion;ribosome	structural molecule activity	1.31	0.52	0.40
P38120	37S ribosomal protein S9, mitochondrial [OS=Saccharomyces cerevisiae S288c]	852443	MRPS9; YBR146W	cell organization and biogenesis;metabolic process	mitochondrion;ribosome	RNA binding;structural molecule activity	1.254	0.501	0.40
P32387	54S ribosomal protein L41, mitochondrial [OS=Saccharomyces cerevisiae S288c]	852014	MRP20; YDR405W	cell organization and biogenesis;metabolic process	mitochondrion;ribosome	nucleotide binding;protein binding;RNA binding;structural molecule activity	1.254	0.501	0.40
Q96VH5	MICOS complex subunit Mic10 [OS=Saccharomyces cerevisiae S288c]	850300	MOS1; MIC10; YCL057C-A	cell organization and biogenesis	membrane;mitochondrion		4.179	1.683	0.40
Q01163	37S ribosomal protein S23, mitochondrial [OS=Saccharomyces cerevisiae S288c]	852748	RSM23; YGL129C	cell organization and biogenesis;metabolic process	mitochondrion;ribosome	catalytic activity;nucleotide binding;RNA binding;structural molecule activity	2.695	1.11	0.41
P33759	37S ribosomal protein S5, mitochondrial [OS=Saccharomyces cerevisiae S288c]	852553	MRPS5; YBR251W	cell organization and biogenesis;metabolic process	mitochondrion;ribosome	RNA binding;structural molecule activity	0.995	0.413	0.42
Q12031	Mitochondrial 2-methylisocitrate lyase [OS=Saccharomyces cerevisiae S288c]	856114	ICL2; YPR006C	metabolic process	mitochondrion;organelle lumen	catalytic activity	7.859	3.281	0.42
P40530	[Pyruvate dehydrogenase (acetyl-transferring)] kinase 1, mitochondrial [OS=Saccharomyces cerevisiae S288c]	854769	PKP1; YIL042C	metabolic process	mitochondrion;organelle lumen	catalytic activity;nucleotide binding;protein binding	0.931	0.389	0.42
Q03246	37S ribosomal protein S17, mitochondrial [OS=Saccharomyces cerevisiae S288c]	855226	MRPS17; YMR188C	cell differentiation;cell organization and biogenesis;metabolic process	mitochondrion;ribosome	RNA binding;structural molecule activity	0.931	0.389	0.42
P36151	Uncharacterized protein YKR070W [OS=Saccharomyces cerevisiae S288c]	853944	YKR070W; YKR070W	metabolic process	mitochondrion		1.976	0.833	0.42
P38797	Protein phosphatase 2C homolog 7, mitochondrial	856475	PTC7; YHR076	metabolic process	mitochondrion	catalytic activity;metal ion	0.874	0.369	0.42

	[OS=Saccharomyces cerevisiae S288c]		W			binding;protein binding			
P00830	ATP synthase subunit beta, mitochondrial [OS=Saccharomyces cerevisiae S288c]	853585	ATP2; YJR121W	metabolic process;transport	cytosol;membrane;mitochondrion	catalytic activity;nucleotide binding;protein binding;transporter activity	99	41.987	0.42
P40086	Cytochrome c oxidase assembly protein cox15 [OS=Saccharomyces cerevisiae S288c]	856884	COX15; YER141 W	cell organization and biogenesis;metabolic process;transport	membrane;mitochondrion	catalytic activity;transporter activity	1.555	0.668	0.43
P15179	Aspartate--tRNA ligase, mitochondrial [OS=Saccharomyces cerevisiae S288c]	856000	MSD1; YPL104 W	metabolic process	cytoplasm;mitochondrion;organelle lumen	catalytic activity;nucleotide binding	0.708	0.307	0.43
Q12480	Probable electron transfer flavoprotein subunit alpha, mitochondrial [OS=Saccharomyces cerevisiae S288c]	856112	AIM45; YPR004C	metabolic process;transport	mitochondrion;organelle lumen	catalytic activity;nucleotide binding	3.467	1.512	0.44
Q08818	Meiotic sister-chromatid recombination protein 6, mitochondrial [OS=Saccharomyces cerevisiae S288c]	854536	MSC6; YOR354C	metabolic process	mitochondrion;organelle lumen	RNA binding	1.154	0.506	0.44
P48569	Uncharacterized protein YDL183C [OS=Saccharomyces cerevisiae S288c]	851345	YDL183C ; YDL183C	transport			0.624	0.274	0.44
P36775	Lon protease homolog, mitochondrial [OS=Saccharomyces cerevisiae S288c]	852259	PIM1; YBL022C	cell organization and biogenesis;metabolic process;regulation of biological process;response to stimulus	cytoplasm;mitochondrion;organelle lumen	catalytic activity;DNA binding;nucleotide binding;RNA binding	1.222	0.537	0.44
P36520	54S ribosomal protein L10, mitochondrial [OS=Saccharomyces cerevisiae S288c]	855436	MRPL10; YNL284C	cell organization and biogenesis;metabolic process	mitochondrion;ribosome	structural molecule activity	0.551	0.245	0.44
P36163	mitochondrial metalloendopeptidase OMA1 [OS=Saccharomyces cerevisiae S288c]	853962	OMA1; YKR087C	metabolic process	membrane;mitochondrion	catalytic activity;metal ion binding	0.551	0.245	0.44
Q02784	Monothiol glutaredoxin-5, mitochondrial [OS=Saccharomyces cerevisiae S288c]	856048	GRX5; YPL059 W	cell organization and biogenesis;cellular homeostasis;metabolic process;regulation of biological process;response to stimulus	mitochondrion;organelle lumen	catalytic activity;metal ion binding	2.594	1.154	0.44
P38341	MICOS complex subunit MIC12 [OS=Saccharomyces cerevisiae S288c]	852566	AIM5; MIC12; YBR262C	cell organization and biogenesis	membrane;mitochondrion		2.594	1.154	0.44
P42949	Mitochondrial import inner membrane translocase subunit TIM16 [OS=Saccharomyces cerevisiae S288c]	853340	PAM16; YJL104W	transport	membrane;mitochondrion	protein binding	0.52	0.233	0.45
P40053	Altered inheritance of mitochondria protein 9, mitochondrial [OS=Saccharomyces cerevisiae S288c]	856813	AIM9; YER080 W		mitochondrion		4.289	1.938	0.45
P38088	Glycine--tRNA ligase 1, mitochondrial [OS=Saccharomyces cerevisiae S288c]		GRS1	metabolic process	cytoplasm;mitochondrion;organelle lumen	catalytic activity;nucleotide binding;protein binding	0.468	0.212	0.45
P08067	cytochrome b-c1 complex subunit Rieske, mitochondrial [OS=Saccharomyces cerevisiae S288c]	856689	RIP1; YEL024W	metabolic process;transport	membrane;mitochondrion	catalytic activity;metal ion binding;transporter activity	55.234	25.102	0.45
P32839	Mitochondrial chaperone BCS1 [OS=Saccharomyces cerevisiae S288c]	851981	BCS1; YDR375C	cell organization and biogenesis;metabolic process;response to stimulus;transport	cytosol;membrane;mitochondrion	catalytic activity;nucleotide binding;transporter activity	0.425	0.194	0.46
Q01976	ADP-ribose pyrophosphatase [OS=Saccharomyces cerevisiae S288c]	852408	YSA1; YBR111C	metabolic process	cytoplasm;mitochondrion;nucleus	catalytic activity;metal ion binding	0.425	0.194	0.46
P38175	37S ribosomal protein MRP21, mitochondrial [OS=Saccharomyces cerevisiae S288c]	852188	MRP21; YBL090 W	cell organization and biogenesis;metabolic process	mitochondrion;ribosome	structural molecule activity	0.425	0.194	0.46
Q12166	2-isopropylmalate synthase 2, mitochondrial [OS=Saccharomyces cerevisiae S288c]	854275	LEU9; YOR108 W	metabolic process	mitochondrion	catalytic activity;protein binding	3.739	1.707	0.46
P37292	Serine hydroxymethyltransferase, mitochondrial [OS=Saccharomyces cerevisiae S288c]	852565	SHM1; YBR263 W	metabolic process	mitochondrion	catalytic activity	12.219	5.579	0.46
Q03713	Respiratory supercomplex factor 1, mitochondrial [OS=Saccharomyces cerevisiae S288c]	854978	RCF1; YML030 W	cell organization and biogenesis	membrane;mitochondrion		2.162	0.995	0.46

Q02950	37S ribosomal protein MRP51, mitochondrial [OS=Saccharomyces cerevisiae S288c]	855985	MRP51; YPL118W	cell organization and biogenesis;metabolic process	mitochondrion;ribosome	structural molecule activity	1.154	0.532	0.46
P40508	Uncharacterized protein YIL077C [OS=Saccharomyces cerevisiae S288c]	854733	YIL077C; YIL077C		mitochondrion		1.448	0.668	0.46
P17558	37S ribosomal protein PET123, mitochondrial [OS=Saccharomyces cerevisiae S288c]	854329	PET123; YOR158W	cell organization and biogenesis;metabolic process	mitochondrion;ribosome	structural molecule activity	1.448	0.668	0.46
Q05779	Ubiquinone biosynthesis protein COQ9, mitochondrial [OS=Saccharomyces cerevisiae S288c]	850898	COQ9; YLR201C	metabolic process	membrane;mitochondrion		0.359	0.166	0.46
P36523	54S ribosomal protein L15, mitochondrial [OS=Saccharomyces cerevisiae S288c]	851022	MRPL15; YLR312W-A	cell organization and biogenesis;metabolic process	mitochondrion;ribosome	catalytic activity;RNA binding;structural molecule activity	0.334	0.155	0.46
P32378	4-hydroxybenzoate polyprenyltransferase, mitochondrial [OS=Saccharomyces cerevisiae S288c]	855778	COQ2; YNR041C	metabolic process;transport	membrane;mitochondrion	catalytic activity	0.292	0.136	0.47
P38884	Altered inheritance of mitochondria protein 18, mitochondrial [OS=Saccharomyces cerevisiae S288c]	856605	AIM18; YHR198C		mitochondrion	catalytic activity	0.311	0.145	0.47
Q06493	LETM1 domain-containing protein YLH47, mitochondrial [OS=Saccharomyces cerevisiae S288c]	856243	YLH47; YPR125W	cell organization and biogenesis;transport	membrane;mitochondrion		1.043	0.487	0.47
P38127	mitochondrial carrier protein RIM2 [OS=Saccharomyces cerevisiae S288c]	852491	RIM2; YBR192W	cell organization and biogenesis;metabolic process;transport	membrane;mitochondrion	structural molecule activity;transporter activity	1.239	0.585	0.47
P00445	Superoxide dismutase [Cu-Zn] [OS=Saccharomyces cerevisiae S288c]	853568	SOD1; YJR104C	cell organization and biogenesis;cellular homeostasis;metabolic process;regulation of biological process;response to stimulus	cytoplasm;cytosol;mitochondrion;nucleus	antioxidant activity;catalytic activity;metal ion binding;protein binding	1.848	0.874	0.47
Q08686	Thiosulfate sulfurtransferase TUM1 [OS=Saccharomyces cerevisiae S288c]	854425	TUM1; YOR251C	metabolic process	cytoplasm;mitochondrion	catalytic activity;protein binding	0.212	0.101	0.48
P43635	Citrate synthase 3, mitochondrial [OS=Saccharomyces cerevisiae S288c]	856107	CIT3; YPR001W	metabolic process	mitochondrion	catalytic activity	6.11	2.914	0.48
P21375	Fumarate reductase 2 [OS=Saccharomyces cerevisiae S288c]	853510	OSM1; YJR051W	metabolic process	endoplasmic reticulum;mitochondrion	catalytic activity;nucleotide binding	0.16	0.077	0.48
P38860	GTPase MTG2, mitochondrial [OS=Saccharomyces cerevisiae S288c]	856573	MTG2; YHR168W	metabolic process;transport	membrane;mitochondrion	catalytic activity;metal ion binding;nucleotide binding;transporter activity	0.145	0.07	0.48
P53166	ATP-dependent RNA helicase mrh4, mitochondrial [OS=Saccharomyces cerevisiae S288c]	852816	MRH4; YGL064C	cell organization and biogenesis;metabolic process	mitochondrion;organelle lumen	catalytic activity;nucleotide binding;RNA binding	0.145	0.07	0.48
P42900	Sigma-like sequence protein 1, mitochondrial [OS=Saccharomyces cerevisiae S288c]	850830	SLS1; YLR139C	cell organization and biogenesis;metabolic process	membrane;mitochondrion	protein binding	0.125	0.061	0.49
P48526	Isoleucine--tRNA ligase, mitochondrial [OS=Saccharomyces cerevisiae S288c]	856067	ISM1; YPL040C	cell organization and biogenesis;metabolic process;regulation of biological process	cytoplasm;cytosol;mitochondrion;organelle lumen	catalytic activity;nucleotide binding	0.081	0.04	0.49
P48237	Mitochondrial group I intron splicing factor CCM1 [OS=Saccharomyces cerevisiae S288c]	853053	CCM1; YGR150C	cell organization and biogenesis;metabolic process	mitochondrion	RNA binding	0.089	0.044	0.49
Q06236	Mitochondrial inner membrane protein SHH4 [OS=Saccharomyces cerevisiae S288c]	850861	SHH4; YLR164W	metabolic process	membrane;mitochondrion	catalytic activity;metal ion binding	6.499	3.217	0.49
P08417	fumarate hydratase, mitochondrial [OS=Saccharomyces cerevisiae S288c]	855866	FUM1; YPL262W	cell organization and biogenesis;metabolic process	cytoplasm;cytosol;mitochondrion;organelle lumen	catalytic activity	13.874	6.88	0.50
P09440	C-1-tetrahydrofolate synthase, mitochondrial [OS=Saccharomyces cerevisiae S288c]	852378	MIS1; YBR084W	metabolic process	cytosol;mitochondrion	catalytic activity;nucleotide binding	1.668	0.829	0.50
P47052	succinate dehydrogenase [ubiquinone] flavoprotein subunit 2, mitochondrial [OS=Saccharomyces cerevisiae S288c]	853405	YJL045W; YJL045W	metabolic process;transport	membrane;mitochondrion	catalytic activity;nucleotide binding	5.884	2.932	0.50

P28817	3-hydroxyisobutyryl-CoA hydrolase, mitochondrial [OS=Saccharomyces cerevisiae S288c]	851606	EHD3; YDR036C	metabolic process	mitochondrion	catalytic activity	1.212	0.61	0.50
P42940	Probable electron transfer flavoprotein subunit beta [OS=Saccharomyces cerevisiae S288c]	853121	CIR1; YGR207C	metabolic process;transport	mitochondrion;organelle lumen		7.66	3.87	0.51
P10662	37S ribosomal protein MRP1, mitochondrial [OS=Saccharomyces cerevisiae S288c]	851948	MRP1; YDR347 W	cell organization and biogenesis;metabolic process;response to stimulus	mitochondrion;ribosome	antioxidant activity;catalytic activity;metal ion binding;structural molecule activity	2.981	1.512	0.51
P48015	Aminomethyltransferase, mitochondrial [OS=Saccharomyces cerevisiae S288c]	851582	GCV1; YDR019C	metabolic process	mitochondrion	catalytic activity;protein binding	11.217	5.7	0.51
P36066	Protein MRG3-like [OS=Saccharomyces cerevisiae S288c]	853725	YKL133C; YKL133C	metabolic process	membrane;mitochondrion	protein binding	0.655	0.334	0.51
P49095	Glycine dehydrogenase (Decarboxylating), mitochondrial [OS=Saccharomyces cerevisiae S288c]	855227	GCV2; YMR189 W	metabolic process	cytosol;mitochondrion	catalytic activity	2.884	1.471	0.51
P38172	MIOREX complex component 3 [OS=Saccharomyces cerevisiae S288c]	852181	YBL095 W; MRX3; YBL095 W		membrane;mitochondrion		0.968	0.501	0.52
P25374	Cysteine desulfurase, mitochondrial [OS=Saccharomyces cerevisiae S288c]	850343	NFS1; YCL017C	cell organization and biogenesis;cellular homeostasis;metabolic process	mitochondrion;nucleus	catalytic activity;metal ion binding;protein binding	2.594	1.346	0.52
P37298	Succinate dehydrogenase [ubiquinone] cytochrome b small subunit, mitochondrial [OS=Saccharomyces cerevisiae S288c]	851758	SDH4; YDR178 W	metabolic process;transport	membrane;mitochondrion	catalytic activity;metal ion binding	4.995	2.594	0.52
P38072	Protein SCO2, mitochondrial [OS=Saccharomyces cerevisiae S288c]	852312	SCO2; YBR024 W	cell organization and biogenesis;cellular homeostasis;metabolic process;transport	membrane;mitochondrion	antioxidant activity;catalytic activity;metal ion binding	1.404	0.73	0.52
P04037	cytochrome c oxidase subunit 4, mitochondrial [OS=Saccharomyces cerevisiae S288c]	852688	COX4; YGL187C	cell organization and biogenesis;metabolic process;transport	membrane;mitochondrion	catalytic activity;metal ion binding;transporte r activity	34.112	17.738	0.52
P07806	Valine--tRNA ligase, mitochondrial [OS=Saccharomyces cerevisiae S288c]	852986	VAS1; YGR094 W	metabolic process;regulation of biological process	cytoplasm;cytosol;mitochondrion	catalytic activity;nucleotide binding	0.722	0.377	0.52
P07256	Cytochrome b-c1 complex subunit 1, mitochondrial [OS=Saccharomyces cerevisiae S288c]	852235	COR1; YBL045C	metabolic process;transport	membrane;mitochondrion	catalytic activity;metal ion binding;protein binding;transporte r activity	18.783	9.89	0.53
P53140	Protein RMD9, mitochondrial [OS=Saccharomyces cerevisiae S288c]	852771	RMD9; YGL107C	cell differentiation;metabolic process;regulation of biological process	membrane;mitochondrion	RNA binding	1.268	0.668	0.53
P53312	Succinyl-CoA ligase [ADP-forming] subunit beta, mitochondrial [OS=Saccharomyces cerevisiae S288c]	853159	LSC2; YGR244C	metabolic process	mitochondrion	catalytic activity;nucleotide binding	20.017	10.602	0.53
P43617	Uncharacterized mitochondrial carrier YFR045W [OS=Saccharomyces cerevisiae S288c]	850606	YFR045 W; YFR045 W	metabolic process;transport	membrane;mitochondrion	structural molecule activity;transporte r activity	0.778	0.413	0.53
P01097	ATPase inhibitor, mitochondrial [OS=Saccharomyces cerevisiae S288c]	851347	INH1; YDL181 W	metabolic process;regulation of biological process	mitochondrion	catalytic activity;enzyme regulator activity;protein binding	2.162	1.154	0.53
P32902	37S ribosomal protein MRP4, mitochondrial [OS=Saccharomyces cerevisiae S288c]	856384	MRP4; YHL004 W	cell organization and biogenesis;metabolic process	mitochondrion;ribosome	structural molecule activity	1.154	0.616	0.53
P16387	Pyruvate dehydrogenase E1 component subunit alpha, mitochondrial [OS=Saccharomyces cerevisiae S288c]	856925	PDA1; YER178 W	cell growth;metabolic process	mitochondrion;organelle lumen	catalytic activity	25.264	13.497	0.53
Q05892	MIOREX complex component 2 [OS=Saccharomyces cerevisiae S288c]	850997	YLR290C; COQ11; YLR290C	metabolic process	mitochondrion	catalytic activity	3.217	1.738	0.54
P36101	tRNA threonylcarbamoyladenosine dehydratase 2 [OS=Saccharomyces cerevisiae S288c]	853841	TCD2; YKL027 W; YKL027 W	metabolic process	cytosol;membrane;mitochondrion	catalytic activity;nucleotide binding	0.616	0.334	0.54

Q04935	Cytochrome c oxidase protein 20, mitochondrial [OS=Saccharomyces cerevisiae S288c]	851817	COX20; YDR231C	cell organization and biogenesis;metabolic process	membrane;mitochondrion	protein binding	1.894	1.031	0.54
Q02608	37S ribosomal protein S16, mitochondrial [OS=Saccharomyces cerevisiae S288c]	856094	MRPS16; YPL013C	cell organization and biogenesis;metabolic process	mitochondrion;ribosome	structural molecule activity	1.683	0.931	0.55
P39726	Glycine cleavage system H protein, mitochondrial [OS=Saccharomyces cerevisiae S288c]	851254	GCV3; YAL044C	metabolic process	mitochondrion	catalytic activity	9	4.995	0.56
P48360	Probable NADPH:adrenodoxin oxidoreductase, mitochondrial [OS=Saccharomyces cerevisiae S288c]	851982	ARH1; YDR376 W	cellular homeostasis;metabolic process;transport	cytoplasm;membrane;mitochondrion;organelle lumen	catalytic activity	0.45	0.25	0.56
P53326	Uncharacterized protein YGR266W [OS=Saccharomyces cerevisiae S288c]	853182	YGR266 W; YGR266 W		membrane;mitochondrion		1.081	0.602	0.56
Q02776	Mitochondrial import inner membrane translocase subunit TIM50 [OS=Saccharomyces cerevisiae S288c]	856042	TIM50; YPL063 W	cell organization and biogenesis;metabolic process;transport	membrane;mitochondrion	catalytic activity;protein binding;transporter activity	7.483	4.179	0.56
P36517	54S ribosomal protein L4, mitochondrial [OS=Saccharomyces cerevisiae S288c]	851160	MRPL4; YLR439 W	cell organization and biogenesis;metabolic process	mitochondrion;ribosome	structural molecule activity	1.565	0.874	0.56
P32795	Mitochondrial inner membrane i-AAA protease supercomplex subunit YME1 [OS=Saccharomyces cerevisiae S288c]	856135	YME1; YPR024 W	metabolic process;response to stimulus;transport	membrane;mitochondrion	catalytic activity;metal ion binding;nucleotide binding;protein binding	1.938	1.085	0.56
P40047	Aldehyde dehydrogenase 5, mitochondrial [OS=Saccharomyces cerevisiae S288c]	856804	ALD5; YER073 W	metabolic process	mitochondrion;organelle lumen	catalytic activity	3.146	1.762	0.56
P08525	Cytochrome b-c1 complex subunit 8 [OS=Saccharomyces cerevisiae S288c]	853273	QCR8; YJL166W	metabolic process;transport	membrane;mitochondrion	catalytic activity;transporter activity	5.31	2.981	0.56
P40012	protoporphyrinogen oxidase [OS=Saccharomyces cerevisiae S288c]	856733	HEM14; YER014 W	metabolic process	membrane;mitochondrion	catalytic activity	1.482	0.833	0.56
P32904	54S ribosomal protein L6, mitochondrial [OS=Saccharomyces cerevisiae S288c]	856552	MRPL6; YHR147C	cell organization and biogenesis;metabolic process	mitochondrion;ribosome	RNA binding;structural molecule activity	1.371	0.778	0.57
P21801	Succinate dehydrogenase [ubiquinone] iron-sulfur subunit, mitochondrial [OS=Saccharomyces cerevisiae S288c]	850685	SDH2; YLL041C	metabolic process;transport	membrane;mitochondrion	catalytic activity;metal ion binding	32.839	18.684	0.57
P40961	prohibitin-1 [OS=Saccharomyces cerevisiae S288c]	853033	PHB1; YGR132C	cell organization and biogenesis;metabolic process;regulation of biological process	membrane;mitochondrion	protein binding	9	5.158	0.57
P53889	uncharacterized mitochondrial hydrolase FMP41 [OS=Saccharomyces cerevisiae S288c]	855553	FMP41; YNL168C	metabolic process	mitochondrion	catalytic activity;metal ion binding	1.254	0.719	0.57
P17505	Malate dehydrogenase, mitochondrial [OS=Saccharomyces cerevisiae S288c]	853777	MDH1; YKL085 W	metabolic process	mitochondrion;organelle lumen	catalytic activity;protein binding;RNA binding	99	56.797	0.57
P15424	ATP-dependent RNA helicase MSS116, mitochondrial [OS=Saccharomyces cerevisiae S288c]	851775	MSS116; YDR194C	metabolic process;regulation of biological process	mitochondrion;organelle lumen	catalytic activity;nucleotide binding;RNA binding	1.154	0.668	0.58
P53732	37S ribosomal protein S12, mitochondrial [OS=Saccharomyces cerevisiae S288c]	855772	MRPS12; YNR036C	cell organization and biogenesis;metabolic process	mitochondrion;ribosome	structural molecule activity	1.154	0.668	0.58
Q04599	54S ribosomal protein L1, mitochondrial [OS=Saccharomyces cerevisiae S288c]	851694	MRPL1; YDR116C	cell organization and biogenesis;metabolic process	mitochondrion;ribosome	RNA binding;structural molecule activity	1.069	0.624	0.58
P38300	Inner membrane mitoribosome receptor MBA1, mitochondrial [OS=Saccharomyces cerevisiae S288c]	852483	MBA1; YBR185C	cell organization and biogenesis;regulation of biological process	membrane;mitochondrion		0.995	0.585	0.59
P40185	Protein mmf1, mitochondrial [OS=Saccharomyces cerevisiae S288c]	854760	MMF1; YIL051C	cell organization and biogenesis;metabolic process	mitochondrion;organelle lumen		9	5.31	0.59
P40452	Cytochrome c oxidase assembly factor 1 [OS=Saccharomyces cerevisiae S288c]	854649	COA1; YIL157C	cell organization and biogenesis	membrane;mitochondrion	protein binding	0.874	0.52	0.59
P36525	54S ribosomal protein L24, mitochondrial [OS=Saccharomyces cerevisiae S288c]	855231	MRPL24; YMR193 W	cell organization and biogenesis;metabolic process	mitochondrion;ribosome	RNA binding;structural molecule activity	1.929	1.154	0.60

P43616	Cys-Gly metallodipeptidase dug1 [OS=Saccharomyces cerevisiae S288c]	850605	DUG1; YFR044C	metabolic process	cytoplasm;mitochondrion;ribosome	catalytic activity;metal ion binding	1.154	0.711	0.62
Q12349	ATP synthase subunit H, mitochondrial [OS=Saccharomyces cerevisiae S288c]	851002	ATP14; YLR295C	cell organization and biogenesis;metabolic process;transport	membrane;mitochondrion	catalytic activity;transporter activity	2.728	1.683	0.62
P38323	ATP-dependent clpX-like chaperone, mitochondrial [OS=Saccharomyces cerevisiae S288c]	852528	MCX1; YBR227C	metabolic process	membrane;mitochondrion;organelle lumen	catalytic activity;nucleotide binding	0.501	0.311	0.62
Q06668	Methyltransferase OMS1, mitochondrial [OS=Saccharomyces cerevisiae S288c]	851911	OMS1; YDR316W	metabolic process	membrane;mitochondrion	catalytic activity	0.484	0.301	0.62
P40496	37S ribosomal protein S25, mitochondrial [OS=Saccharomyces cerevisiae S288c]	854715	RSM25; YIL093C	cell organization and biogenesis;metabolic process	mitochondrion;ribosome	structural molecule activity	0.468	0.292	0.62
Q3E824	Uncharacterized protein YOR020W-A [OS=Saccharomyces cerevisiae S288c]	1466480	YOR020W-A; YOR020W-A	metabolic process;transport	membrane;mitochondrion		5.813	3.642	0.63
Q04689	Altered inheritance of mitochondria protein 32 [OS=Saccharomyces cerevisiae S288c]	854955	AIM32; YML050W				0.369	0.233	0.63
P54857	lipase 2 [OS=Saccharomyces cerevisiae S288c]	851628	TGL2; YDR058C	metabolic process;transport	mitochondrion	catalytic activity	0.369	0.233	0.63
Q05648	MIOREX complex component 10 [OS=Saccharomyces cerevisiae S288c]	851876	YDR282C; MRX10; YDR282C		membrane;mitochondrion		0.35	0.222	0.63
P38891	Branched-chain-amino-acid aminotransferase, mitochondrial [OS=Saccharomyces cerevisiae S288c]	856615	BAT1; YHR208W	metabolic process;regulation of biological process;response to stimulus	mitochondrion;organelle lumen	catalytic activity	15.876	10.103	0.64
P03879	Intron-encoded RNA maturase bI4 [OS=Saccharomyces cerevisiae S288c]	854582	BI4; Q0120	metabolic process	membrane;mitochondrion	catalytic activity;RNA binding	0.28	0.179	0.64
Q03028	Mitochondrial 2-oxodicarboxylate carrier 1 [OS=Saccharomyces cerevisiae S288c]	855969	ODC1; YPL134C	metabolic process;transport	membrane;mitochondrion	structural molecule activity;transporter activity	7.799	4.995	0.64
Q03976	37S ribosomal protein S24, mitochondrial [OS=Saccharomyces cerevisiae S288c]	851755	RSM24; YDR175C	cell organization and biogenesis;metabolic process	mitochondrion;ribosome	structural molecule activity	0.905	0.585	0.65
P39533	Homocitrate dehydratase, mitochondrial [OS=Saccharomyces cerevisiae S288c]	853230	ACO2; YJL200C	metabolic process	mitochondrion	catalytic activity;metal ion binding	0.17	0.11	0.65
P53266	Cytochrome oxidase assembly protein SHY1 [OS=Saccharomyces cerevisiae S288c]	853009	SHY1; YGR112W	cell organization and biogenesis	membrane;mitochondrion	protein binding	1.783	1.154	0.65
Q04728	Arginine biosynthesis bifunctional protein ArgJ, mitochondrial [OS=Saccharomyces cerevisiae S288c]	855084	ARG7; YMR062C	metabolic process	mitochondrion;organelle lumen	catalytic activity	1.154	0.748	0.65
P43567	alanine--glyoxylate aminotransferase 1 [OS=Saccharomyces cerevisiae S288c]	850514	AGX1; YFL030W	metabolic process	cytosol;mitochondrion	catalytic activity	2.481	1.61	0.65
P33311	ATP-dependent permease MDL2, mitochondrial [OS=Saccharomyces cerevisiae S288c]	855858	MDL2; YPL270W	response to stimulus;transport	membrane;mitochondrion	catalytic activity;nucleotide binding;transporter activity	1.233	0.802	0.65
P34227	Mitochondrial peroxiredoxin PRX1 [OS=Saccharomyces cerevisiae S288c]	852215	PRX1; YBL064C	cellular homeostasis;metabolic process;regulation of biological process;response to stimulus	mitochondrion	antioxidant activity;catalytic activity	6.848	4.456	0.65
Q08179	Mitochondrial distribution and morphology protein 38 [OS=Saccharomyces cerevisiae S288c]	854130	MDM38; YOL027C	cell organization and biogenesis;cellular homeostasis;regulation of biological process;transport	membrane;mitochondrion		5.683	3.732	0.66
Q00711	Succinate dehydrogenase [ubiquinone] flavoprotein subunit, mitochondrial [OS=Saccharomyces cerevisiae S288c]	853709	SDH1; YKL148C	metabolic process;transport	membrane;mitochondrion	catalytic activity;nucleotide binding;protein binding	18.619	12.242	0.66
P07275	Delta-1-pyrroline-5-carboxylate dehydrogenase, mitochondrial [OS=Saccharomyces cerevisiae S288c]	856432	PUT2; YHR037W	metabolic process	membrane;mitochondrion;organelle lumen	catalytic activity	6.565	4.337	0.66
P19414	Aconitate hydratase, mitochondrial [OS=Saccharomyces cerevisiae S288c]	851013	ACO1; YLR304C	cell organization and biogenesis;metabolic process;transport	cytoplasm;cytosol;mitochondrion;organelle lumen	catalytic activity;DNA binding;metal ion binding	66.002	43.893	0.67

P14832	peptidyl-prolyl cis-trans isomerase [OS=Saccharomyces cerevisiae S288c]	851733	CPR1; YDR155C	cell differentiation;cell organization and biogenesis;metabolic process;regulation of biological process;transport	cytoplasm;mitochondrion;nucleus	catalytic activity;RNA binding	1.31	0.874	0.67
P06208-1	2-isopropylmalate synthase [OS=Saccharomyces cerevisiae S288c]	855619	LEU4; YNL104C	metabolic process	cytoplasm;mitochondrion	catalytic activity;protein binding	9	6.017	0.67
Q05931	Heat shock protein SSQ1, mitochondrial [OS=Saccharomyces cerevisiae S288c]	851084	SSQ1; YLR369 W	cell organization and biogenesis;metabolic process	mitochondrion;organelle lumen	nucleotide binding;protein binding	0.995	0.679	0.68
P07143	Cytochrome c1, heme protein, mitochondrial [OS=Saccharomyces cerevisiae S288c]	854231	CYT1; YOR065 W	metabolic process;transport	cytosol;membrane;mitochondrion	metal ion binding	19.309	13.251	0.69
P40098	uncharacterized mitochondrial membrane protein FMP10 [OS=Saccharomyces cerevisiae S288c]	856931	FMP10; YER182 W		membrane;mitochondrion		2.36	1.637	0.69
Q12029	Probable mitochondrial transport protein fsf1 [OS=Saccharomyces cerevisiae S288c]	854445	FSF1; YOR271C	transport	membrane;mitochondrion	transporter activity	2.36	1.637	0.69
Q99297	Mitochondrial 2-oxodicarboxylate carrier 2 [OS=Saccharomyces cerevisiae S288c]	854397	ODC2; YOR222 W	metabolic process;transport	membrane;mitochondrion	structural molecule activity;transporte r activity	0.778	0.54	0.69
P32316	acetyl-CoA hydrolase [OS=Saccharomyces cerevisiae S288c]	852266	ACH1; YBL015 W	metabolic process	cytoplasm;cytosol;mitochondrion	catalytic activity	29.539	20.544	0.70
Q02486	ARS-binding factor 2, mitochondrial [OS=Saccharomyces cerevisiae S288c]	855094	ABF2; YMR072 W	cell organization and biogenesis;metabolic process;regulation of biological process	chromosome;mitochondrion;nucleus	DNA binding	69.17	48.239	0.70
P33310	ATP-dependent permease MDL1, mitochondrial [OS=Saccharomyces cerevisiae S288c]	850885	MDL1; YLR188 W	transport	membrane;mitochondrion	catalytic activity;nucleotide binding;transporte r activity	0.624	0.438	0.70
Q12251	Uncharacterized mitochondrial carrier YPR011C [OS=Saccharomyces cerevisiae S288c]	856121	YPR011C ; YPR011C	metabolic process;transport	membrane;mitochondrion	structural molecule activity;transporte r activity	0.551	0.389	0.71
Q12482	Mitochondrial aspartate-glutamate transporter AGC1 [OS=Saccharomyces cerevisiae S288c]	856132	AGC1; YPR021C	metabolic process;transport	membrane;mitochondrion	structural molecule activity;transporte r activity	0.407	0.292	0.72
P32191	Glycerol-3-phosphate dehydrogenase, mitochondrial [OS=Saccharomyces cerevisiae S288c]	854651	GUT2; YIL155C	metabolic process	membrane;mitochondrion	catalytic activity	9.502	6.827	0.72
Q04172	Sensitive to high expression protein 9, mitochondrial [OS=Saccharomyces cerevisiae S288c]	852002	SHE9; YDR393 W	cell organization and biogenesis	membrane;mitochondrion		0.374	0.269	0.72
P33421	Succinate dehydrogenase [ubiquinone] cytochrome b subunit, mitochondrial [OS=Saccharomyces cerevisiae S288c]	853716	SDH3; YKL141 W	cell organization and biogenesis;metabolic process;transport	membrane;mitochondrion	catalytic activity;metal ion binding	1.276	0.931	0.73
P32787	Mitochondrial genome maintenance protein MGM101 [OS=Saccharomyces cerevisiae S288c]	853609	MGM10 1; YJR144W	cell organization and biogenesis;metabolic process;response to stimulus	chromosome;mitochondrion	DNA binding	4.456	3.281	0.74
P00401	Cytochrome c oxidase subunit 1 [OS=Saccharomyces cerevisiae S288c]	854598	COX1; Q0045	metabolic process;transport	membrane;mitochondrion	catalytic activity;metal ion binding;transporte r activity	2.162	1.61	0.74
P32843	Mitochondrial escape protein 2 [OS=Saccharomyces cerevisiae S288c]	855348	YME2; YMR302 C	cell organization and biogenesis;metabolic process	membrane;mitochondrion	nucleotide binding;RNA binding	3.112	2.36	0.76
P00128	Cytochrome b-c1 complex subunit 7 [OS=Saccharomyces cerevisiae S288c]	852142	QCR7; YDR529C	cell organization and biogenesis;metabolic process;transport	membrane;mitochondrion	catalytic activity;transporte r activity	15.681	11.915	0.76
P32335	Protein MSS51, mitochondrial [OS=Saccharomyces cerevisiae S288c]	850900	MSS51; YLR203C	metabolic process;regulation of biological process	membrane;mitochondrion	protein binding;translation regulator activity	1.512	1.154	0.76
P04710	ADP,ATP carrier protein 1 [OS=Saccharomyces cerevisiae S288c]	855078	AAC1; YMR056 C	metabolic process;transport	cytosol;membrane;mitochondrion	structural molecule activity;transporte r activity	10.288	7.859	0.76
P28834	Isocitrate dehydrogenase [NAD] subunit 1, mitochondrial [OS=Saccharomyces cerevisiae S288c]	855691	IDH1; YNL037C	metabolic process;transport	cytosol;mitochondrion;organelle lumen	catalytic activity;metal ion binding;nucleotide binding;protein binding;RNA binding	15.238	11.743	0.77
P40513	Mitochondrial acidic protein MAM33 [OS=Saccharomyces cerevisiae S288c]	854740	MAM33; YIL070C	metabolic process;regulation of biological process	mitochondrion;organelle lumen	translation regulator activity	3.642	2.831	0.78

P00410	Cytochrome c oxidase subunit 2 [OS=Saccharomyces cerevisiae S288c]	854622	COX2; Q0250	metabolic process;transport	membrane;mitochondrion	catalytic activity;metal ion binding;protein binding;transporter activity	3.642	2.831	0.78
P36521	54S ribosomal protein L11, mitochondrial [OS=Saccharomyces cerevisiae S288c]	851325	MRPL11; YDL202W	cell organization and biogenesis;metabolic process	mitochondrion;ribosome	structural molecule activity	1.069	0.833	0.78
P51998	54S ribosomal protein yml6, mitochondrial [OS=Saccharomyces cerevisiae S288c]	854983	YML6; YML025C	cell organization and biogenesis;metabolic process	mitochondrion;ribosome	RNA binding;structural molecule activity	0.931	0.73	0.78
P00427	Cytochrome c oxidase subunit 6, mitochondrial [OS=Saccharomyces cerevisiae S288c]	856448	COX6; YHR051W	metabolic process;transport	membrane;mitochondrion	catalytic activity;metal ion binding;transporter activity	4.623	3.642	0.79
P39952	Mitochondrial inner membrane protein OXA1 [OS=Saccharomyces cerevisiae S288c]	856898	OXA1; YER154W	cell organization and biogenesis;transport	membrane;mitochondrion	transporter activity	1.738	1.371	0.79
P40495	Homoisocitrate dehydrogenase, mitochondrial [OS=Saccharomyces cerevisiae S288c]	854714	LYS12; YIL094C	metabolic process	mitochondrion	catalytic activity;metal ion binding;nucleotide binding	6.499	5.19	0.80
Q03798	Altered inheritance of mitochondria protein 36, mitochondrial [OS=Saccharomyces cerevisiae S288c]	855189	AIM36; YMR157C		membrane;mitochondrion		1.336	1.069	0.80
P40008	Protein FMP52, mitochondrial [OS=Saccharomyces cerevisiae S288c]	856721	FMP52; YER004W		endoplasmic reticulum;membrane;mitochondrion	catalytic activity	2.162	1.738	0.80
P07342	Acetolactate synthase catalytic subunit, mitochondrial [OS=Saccharomyces cerevisiae S288c]	855135	ILV2; YMR108W	metabolic process	mitochondrion	catalytic activity;metal ion binding;nucleotide binding	5.236	4.223	0.81
P47127	Altered inheritance of mitochondria protein 24, mitochondrial [OS=Saccharomyces cerevisiae S288c]	853543	AIM24; YJR080C	cell organization and biogenesis	mitochondrion		3.175	2.562	0.81
Q12298	uncharacterized ABC transporter ATP-binding protein YDR061W [OS=Saccharomyces cerevisiae S288c]	851633	YDR061W; YDR061W		mitochondrion	catalytic activity;nucleotide binding	0.425	0.343	0.81
P25719	Peptidyl-prolyl cis-trans isomerase C, mitochondrial [OS=Saccharomyces cerevisiae S288c]	854897	CPR3; YML078W	cell death;metabolic process	mitochondrion;organelle lumen	catalytic activity	2.981	2.415	0.81
Q08822	Probable electron transfer flavoprotein-ubiquinone oxidoreductase, mitochondrial [OS=Saccharomyces cerevisiae S288c]	854538	CIR2; YOR356W	metabolic process;transport	membrane;mitochondrion	catalytic activity;metal ion binding;nucleotide binding	3.019	2.481	0.82
P38885	Altered inheritance of mitochondria protein 46, mitochondrial [OS=Saccharomyces cerevisiae S288c]	856606	AIM46; YHR199C		mitochondrion	catalytic activity	1.404	1.154	0.82
P47045	mitochondrial import inner membrane translocase subunit TIM54 [OS=Saccharomyces cerevisiae S288c]	853392	TIM54; YJL054W	cell organization and biogenesis;transport	cytosol;membrane;mitochondrion	transporter activity	0.655	0.54	0.82
P39925	Mitochondrial respiratory chain complexes assembly protein AFG3 [OS=Saccharomyces cerevisiae S288c]	856737	AFG3; YER017C	cell organization and biogenesis;metabolic process;response to stimulus;transport	membrane;mitochondrion	catalytic activity;metal ion binding;nucleotide binding;protein binding	1.228	1.015	0.83
Q06678	54S ribosomal protein L35, mitochondrial [OS=Saccharomyces cerevisiae S288c]	851921	MRPL35; YDR322W	cell organization and biogenesis;metabolic process	mitochondrion;ribosome	structural molecule activity	1.424	1.219	0.86
P32317	Protein AFG1 [OS=Saccharomyces cerevisiae S288c]	856658	AFG1; YEL052W	cell organization and biogenesis;metabolic process;response to stimulus;transport	membrane;mitochondrion	catalytic activity;nucleotide binding	1.154	0.995	0.86
P32891	D-lactate dehydrogenase [cytochrome] 1, mitochondrial [OS=Saccharomyces cerevisiae S288c]	851380	DLD1; YDL174C	metabolic process;transport	membrane;mitochondrion	catalytic activity;nucleotide binding	13.03	11.253	0.86
P33303	Succinate/fumarate mitochondrial transporter [OS=Saccharomyces cerevisiae S288c]	853558	SFC1; YJR095W	metabolic process;transport	membrane;mitochondrion	structural molecule activity;transporter activity	17.957	15.681	0.87
P46367	Potassium-activated aldehyde dehydrogenase, mitochondrial [OS=Saccharomyces cerevisiae S288c]	854556	ALD4; YOR374W	metabolic process	mitochondrion;organelle lumen	catalytic activity	58.078	50.795	0.87

P32473	Pyruvate dehydrogenase E1 component subunit beta, mitochondrial [OS=Saccharomyces cerevisiae S288c]	852522	PDB1; YBR221C	metabolic process	mitochondrion;organelle lumen	catalytic activity	10.288	9	0.87
P40341	Mitochondrial respiratory chain complexes assembly protein YTA12 [OS=Saccharomyces cerevisiae S288c]	855114	YTA12; YMR089C	cell organization and biogenesis;metabolic process;response to stimulus;transport	cytoplasm;membrane;mitochondrion	catalytic activity;metal ion binding;nucleotide binding;protein binding	1.795	1.575	0.88
P52893	probable alanine aminotransferase, mitochondrial [OS=Saccharomyces cerevisiae S288c]	850778	ALT1; YLR089C	metabolic process	mitochondrion;organelle lumen	catalytic activity	1.438	1.264	0.88
P50085	Prohibitin-2 [OS=Saccharomyces cerevisiae S288c]	853146	PHB2; YGR231C	cell organization and biogenesis;metabolic process;regulation of biological process	membrane;mitochondrion	protein binding	4.926	4.337	0.88
P18239	ADP,ATP carrier protein 2 [OS=Saccharomyces cerevisiae S288c]	852250	PET9; YBL030C	cell death;metabolic process;transport	membrane;mitochondrion	structural molecule activity;transporter activity	135.887	122.285	0.90
P49367	Homoaconitase, mitochondrial [OS=Saccharomyces cerevisiae S288c]	851820	LYS4; YDR234W	metabolic process	mitochondrion	catalytic activity;metal ion binding;protein binding	1.276	1.154	0.90
P36013	NAD-dependent malic enzyme, mitochondrial [OS=Saccharomyces cerevisiae S288c]	853839	MAE1; YKL029C	metabolic process	mitochondrion;organelle lumen	catalytic activity;metal ion binding;nucleotide binding	2.35	2.162	0.92
P40215	External NADH-ubiquinone oxidoreductase 1, mitochondrial [OS=Saccharomyces cerevisiae S288c]	855176	NDE1; YMR145C	metabolic process	mitochondrion	catalytic activity;nucleotide binding	5.469	5.078	0.93
P07257	Cytochrome b-c1 complex subunit 2, mitochondrial [OS=Saccharomyces cerevisiae S288c]	856321	QCR2; YPR191W	metabolic process;transport	membrane;mitochondrion	catalytic activity;metal ion binding;protein binding;transporter activity	33.551	33.551	1.00
P36112	MICOS complex subunit Mic60 [OS=Saccharomyces cerevisiae S288c]	853886	FCJ1; MIC60; YKR016W	cell organization and biogenesis;transport	membrane;mitochondrion		12.895	12.895	1.00
P23641	mitochondrial phosphate carrier protein [OS=Saccharomyces cerevisiae S288c]	853540	MIR1; YJR077C	metabolic process;transport	membrane;mitochondrion	protein binding;structural molecule activity;transporter activity	26.826	26.826	1.00
P36060	NADH-cytochrome b5 reductase 2 [OS=Saccharomyces cerevisiae S288c]	853707	MCR1; YKL150W	metabolic process;response to stimulus	membrane;mitochondrion	catalytic activity;protein binding	19.691	19.691	1.00
P19262	Dihydrolipoyllysine-residue succinyltransferase component of 2-oxoglutarate dehydrogenase complex, mitochondrial [OS=Saccharomyces cerevisiae S288c]	851726	KGD2; YDR148C	cell organization and biogenesis;metabolic process	mitochondrion	catalytic activity;protein binding	5.7	5.7	1.00
P25613	Accumulation of dyads protein 2 [OS=Saccharomyces cerevisiae S288c]	850368	ADY2; YCR010C	transport	membrane;mitochondrion;vacuole	transporter activity	12.895	12.895	1.00
Q12335	Protoplast secreted protein 2 [OS=Saccharomyces cerevisiae S288c]	851596	PST2; YDR032C	metabolic process;regulation of biological process	cytoplasm;extracellular;membrane;mitochondrion	catalytic activity;nucleotide binding;protein binding	4.012	4.012	1.00
P81449	ATP synthase subunit e, mitochondrial [OS=Saccharomyces cerevisiae S288c]	851922	TIM11; YDR322C-A	cell organization and biogenesis;metabolic process;transport	membrane;mitochondrion	catalytic activity;structural molecule activity;transporter activity	9	9	1.00
Q07349	MIOREX complex component 9 [OS=Saccharomyces cerevisiae S288c]	851535	YDL027C; MRX9; YDL027C		endoplasmic reticulum;membrane;mitochondrion		1.913	1.913	1.00
P00044	Cytochrome c iso-1 [OS=Saccharomyces cerevisiae S288c]	853507	CYC1; YJR048W	metabolic process;transport	mitochondrion	metal ion binding;protein binding	12.335	12.335	1.00
P38325	Mitochondrial outer membrane protein OM14 [OS=Saccharomyces cerevisiae S288c]	852531	OM14; YBR230C	transport	membrane;mitochondrion		9	9	1.00
Q08023	Protein FMP25, mitochondrial [OS=Saccharomyces cerevisiae S288c]	850766	FMP25; YLR077W	cell organization and biogenesis	membrane;mitochondrion		0.968	0.968	1.00
P53721	Respiratory supercomplex factor 2, mitochondrial [OS=Saccharomyces cerevisiae S288c]	855752	RCF2; YNR018W	cell organization and biogenesis	membrane;mitochondrion		7.111	7.111	1.00

Q12289	mitochondrial carnitine carrier [OS=Saccharomyces cerevisiae S288c]	854267	CRC1; YOR100C	metabolic process;transport	membrane;mitochondrion	structural molecule activity;transporter activity	0.931	0.931	1.00
P10823	Guanine nucleotide-binding protein alpha-2 subunit [OS=Saccharomyces cerevisiae S288c]	856741	GPA2; YER020W	cell differentiation;cell growth;cell organization and biogenesis;cellular homeostasis;regulation of biological process;response to stimulus	cytosol;membrane;mitochondrion	catalytic activity;metal ion binding;nucleotide binding;protein binding;signal transducer activity	0.978	0.978	1.00
P39006	Phosphatidylserine decarboxylase proenzyme 1, mitochondrial [OS=Saccharomyces cerevisiae S288c]	855552	PSD1; YNL169C	metabolic process;regulation of biological process	membrane;mitochondrion	catalytic activity	0.487	0.487	1.00
Q12374	Nuclear control of ATPase protein 2 [OS=Saccharomyces cerevisiae S288c]	856278	NCA2; YPR155C	metabolic process	membrane;mitochondrion		0.565	0.565	1.00
P21560	Protein CBP3, mitochondrial [OS=Saccharomyces cerevisiae S288c]	855886	CBP3; YPL215W	cell organization and biogenesis;regulation of biological process	membrane;mitochondrion;ribosome		1.031	1.031	1.00
P07253	cytochrome B pre-mRNA-processing protein 6 [OS=Saccharomyces cerevisiae S288c]	852417	CBP6; YBR120C	cell organization and biogenesis;metabolic process;regulation of biological process	mitochondrion;ribosome		2.728	2.728	1.00
P38910	10 kDa heat shock protein, mitochondrial [OS=Saccharomyces cerevisiae S288c]	854185	HSP10; YOR020C	cell organization and biogenesis;metabolic process;response to stimulus;transport	cytoplasm;mitochondrion;organelle lumen	metal ion binding;nucleotide binding;protein binding	4.623	4.623	1.00
Q06143	mitochondrial dicarboxylate transporter [OS=Saccharomyces cerevisiae S288c]	851063	DIC1; YLR348C	metabolic process;transport	membrane;mitochondrion	structural molecule activity;transporter activity	1.154	1.154	1.00
P40416	Iron-sulfur clusters transporter ATM1, mitochondrial [OS=Saccharomyces cerevisiae S288c]	855347	ATM1; YMR301C	cellular homeostasis;metabolic process;transport	membrane;mitochondrion	catalytic activity;nucleotide binding;transporter activity	0.453	0.453	1.00
P50945	MICOS complex subunit MIC27 [OS=Saccharomyces cerevisiae S288c]	855623	AIM37; MIC27; YNL100W	cell organization and biogenesis	membrane;mitochondrion		1.254	1.254	1.00
O14467	multiprotein-bridging factor 1 [OS=Saccharomyces cerevisiae S288c]	854474	MBF1; YOR298C-A	metabolic process;regulation of biological process	cytoplasm;mitochondrion;nucleus	DNA binding	1.31	1.31	1.00
P32907	Ammonia transport outward protein 2 [OS=Saccharomyces cerevisiae S288c]	855736	ATO2; YNR002C	transport	membrane;mitochondrion;vacuole	transporter activity	0.874	0.874	1.00
P53311	Mitochondrial pyruvate carrier 3 [OS=Saccharomyces cerevisiae S288c]	853158	FMP43; MPC3; YGR243W	transport	membrane;mitochondrion	transporter activity	1.154	1.154	1.00
P40502	Altered inheritance of mitochondria protein 19, mitochondrial [OS=Saccharomyces cerevisiae S288c]	854722	AIM19; YIL087C		membrane;mitochondrion		0.995	0.995	1.00
Q01519	Cytochrome c oxidase subunit 6b [OS=Saccharomyces cerevisiae S288c]	850727	COX12; YLR038C	cell organization and biogenesis;metabolic process;transport	mitochondrion	catalytic activity;transporter activity	3.642	3.642	1.00
Q06567	ABC1 family protein MCP2 [OS=Saccharomyces cerevisiae S288c]	850955	YLR253W; MCP2; YLR253W	cell organization and biogenesis	membrane;mitochondrion		0.218	0.218	1.00
P37299	cytochrome b-c1 complex subunit 10 [OS=Saccharomyces cerevisiae S288c]	856390	QCR10; YHR001W-A	metabolic process;transport	membrane;mitochondrion	catalytic activity;transporter activity	5.31	5.31	1.00
Q12233	ATP synthase subunit g, mitochondrial [OS=Saccharomyces cerevisiae S288c]	856131	ATP20; YPR020W	cell organization and biogenesis;metabolic process;transport	membrane;mitochondrion	catalytic activity;transporter activity	1.371	1.371	1.00
P40159	Uncharacterized protein YNL208W [OS=Saccharomyces cerevisiae S288c]	855513	YNL208W; YNL208W		membrane;mitochondrion;ribosome		0.668	0.668	1.00
Q03327	Mitochondrial fusion and transport protein Ugo1 [OS=Saccharomyces cerevisiae S288c]	852081	UGO1; YDR470C	cell organization and biogenesis;metabolic process	membrane;mitochondrion	protein binding;structural molecule activity	0.425	0.425	1.00
Q6Q560	Protein isd11 [OS=Saccharomyces cerevisiae S288c]	856774	ISD11; YER048W-A	cell organization and biogenesis;metabolic process	mitochondrion;organelle lumen	catalytic activity;protein binding	1.783	1.783	1.00
Q12375	mitochondrial ornithine transporter 1 [OS=Saccharomyces cerevisiae S288c]	854297	ORT1; YOR130C	metabolic process;transport	membrane;mitochondrion	structural molecule activity;transporter activity	0.54	0.54	1.00
P41911	Glycerol-3-phosphate dehydrogenase [NAD(+)] 2, mitochondrial [OS=Saccharomyces cerevisiae S288c]	854095	GPD2; YOL059W	metabolic process	cytoplasm;cytosol;mitochondrion	catalytic activity;nucleotide binding;protein binding	0.186	0.186	1.00

P53220	Mitochondrial import inner membrane translocase subunit TIM21 [OS=Saccharomyces cerevisiae S288c]	852921	TIM21; YGR033C	cell organization and biogenesis;transport	membrane;mitochondrion	protein binding	0.719	0.719	1.00
P38702	mitochondrial carrier protein LEU5 [OS=Saccharomyces cerevisiae S288c]	856391	LEU5; YHR002 W	metabolic process;transport	membrane;mitochondrion	structural molecule activity;transporte r activity	0.334	0.334	1.00
P36147	Presequence translocated-associated motor subunit pam17, mitochondrial [OS=Saccharomyces cerevisiae S288c]	853939	PAM17; YKR065C	transport	membrane;mitochondrion		1.371	1.371	1.00
Q02883	N-acyl-phosphatidylethanolamine-hydrolyzing phospholipase D, mitochondrial [OS=Saccharomyces cerevisiae S288c]	856001	FMP30; YPL103C	metabolic process	membrane;mitochondrion	catalytic activity;metal ion binding	0.425	0.425	1.00
P36526	54S ribosomal protein L27, mitochondrial [OS=Saccharomyces cerevisiae S288c]	852585	MRPL27; YBR282 W	cell organization and biogenesis;metabolic process	mitochondrion;ribosome	structural molecule activity	0.995	0.995	1.00
P53163	54S ribosomal protein L12, mitochondrial [OS=Saccharomyces cerevisiae S288c]	852811	MNP1; YGL068 W	cell organization and biogenesis;metabolic process	mitochondrion;ribosome	RNA binding;structural molecule activity	1.31	1.31	1.00
Q01532-1	Cysteine proteinase 1, mitochondrial [OS=Saccharomyces cerevisiae S288c]			metabolic process;regulation of biological process;response to stimulus	cytoplasm;mitochondrion	catalytic activity;DNA binding;RNA binding	0.179	0.179	1.00
Q07914	mitochondrial import inner membrane translocase subunit TIM14 [OS=Saccharomyces cerevisiae S288c]	850694	PAM18; YLR008C	regulation of biological process;transport	membrane;mitochondrion	enzyme regulator activity;protein binding;transporte r activity	0.778	0.778	1.00
P38152	Tricarboxylate transport protein [OS=Saccharomyces cerevisiae S288c]	852594	CTP1; YBR291C	metabolic process;transport	membrane;mitochondrion	structural molecule activity;transporte r activity	0.389	0.389	1.00
P81451	ATP synthase subunit K, mitochondrial [OS=Saccharomyces cerevisiae S288c]	854077	ATP19; YOL077 W-A	cell organization and biogenesis;metabolic process;transport	membrane;mitochondrion	catalytic activity;transporte r activity	3.642	3.642	1.00
P25345	Asparagine--tRNA ligase, mitochondrial [OS=Saccharomyces cerevisiae S288c]	850388	SLM5; YCR024C	metabolic process	cytoplasm;mitochondrion;organelle lumen	catalytic activity;nucleotide binding	0.172	0.172	1.00
P49334	Mitochondrial import receptor subunit tom22 [OS=Saccharomyces cerevisiae S288c]	855592	TOM22; YNL131 W	cell organization and biogenesis;transport	membrane;mitochondrion	protein binding;transporte r activity	0.292	0.292	1.00
Q06005	Octanoyltransferase, mitochondrial [OS=Saccharomyces cerevisiae S288c]	850940	LIP2; YLR239C	metabolic process	cytoplasm;mitochondrion	catalytic activity	0.245	0.245	1.00
P34224	Uncharacterized protein YBL059W [OS=Saccharomyces cerevisiae S288c]	852221	YBL059 W; YBL059 W		membrane;mitochondrion		0.468	0.468	1.00
Q12467	MIOREX complex component 4 [OS=Saccharomyces cerevisiae S288c]	855935	YPL168 W; MRX4; YPL168 W		membrane;mitochondrion		0.202	0.202	1.00
P41735	5-demethoxyubiquinone hydroxylase, mitochondrial [OS=Saccharomyces cerevisiae S288c]	854292	CAT5; YOR125C	metabolic process	membrane;mitochondrion	catalytic activity;metal ion binding	0.54	0.54	1.00
P22438	Methionine--tRNA ligase, mitochondrial [OS=Saccharomyces cerevisiae S288c]	853081	MSM1; YGR171C	metabolic process	cytoplasm;cytosol;mitochondrion;organelle lumen	catalytic activity;nucleotide binding	0.136	0.136	1.00
P53320	Mitochondrial carrier protein MTM1 [OS=Saccharomyces cerevisiae S288c]	853173	MTM1; YGR257C	metabolic process;transport	membrane;mitochondrion	structural molecule activity	0.11	0.11	1.00
Q3E6R5	uncharacterized mitochondrial outer membrane protein YDR381C-A [OS=Saccharomyces cerevisiae S288c]	851989	YDR381C -A; YDR381C -A		membrane;mitochondrion		0.389	0.389	1.00
P32463	Acyl carrier protein, mitochondrial [OS=Saccharomyces cerevisiae S288c]	853642	ACP1; YKL192C	metabolic process;transport	mitochondrion		0.259	0.259	1.00
P40581	peroxiredoxin HYR1 [OS=Saccharomyces cerevisiae S288c]	854855	HYR1; YIR037W	metabolic process;response to stimulus	cytoplasm;cytosol;mitochondrion;organelle lumen	antioxidant activity;catalytic activity	0.259	0.259	1.00
Q07821	Iron-sulfur assembly protein 1 [OS=Saccharomyces cerevisiae S288c]	850632	ISA1; YLL027W	cell organization and biogenesis;metabolic process	mitochondrion;organelle lumen	metal ion binding;structural molecule activity	0.136	0.136	1.00

P04039	Cytochrome c oxidase polypeptide VIII, mitochondrial [OS=Saccharomyces cerevisiae S288c]	851111	COX8; YLR395C	metabolic process;transport	membrane;mitochondrion	catalytic activity;transporter activity	0.778	0.778	1.00
P38228	Mitochondrial chaperone TCM62 [OS=Saccharomyces cerevisiae S288c]	852332	TCM62; YBR044C	cell organization and biogenesis	membrane;mitochondrion	nucleotide binding;protein binding	0.129	0.129	1.00
P10174	cytochrome c oxidase subunit 7 [OS=Saccharomyces cerevisiae S288c]	855298	COX7; YMR256C	metabolic process;transport	membrane;mitochondrion	catalytic activity;transporter activity	1.154	1.154	1.00
P39515	mitochondrial import inner membrane translocase subunit Tim17 [OS=Saccharomyces cerevisiae S288c]	853298	TIM17; YJL143W	cell organization and biogenesis;transport	membrane;mitochondrion	protein binding;transporter activity	0.778	0.778	1.00
P38816	thioredoxin reductase 2, mitochondrial [OS=Saccharomyces cerevisiae S288c]	856506	TRR2; YHR106W	cellular homeostasis;metabolic process;regulation of biological process;response to stimulus	cytoplasm;mitochondrion	antioxidant activity;catalytic activity	0.136	0.136	1.00
P25270	rRNA methyltransferase 1, mitochondrial [OS=Saccharomyces cerevisiae S288c]	854376	MRM1; YOR201C	metabolic process	mitochondrion	catalytic activity;RNA binding	0.129	0.129	1.00
P25578	CDP-diacylglycerol--glycerol-3-phosphate 3-phosphatidyltransferase [OS=Saccharomyces cerevisiae S288c]	850352	PGS1; YCL004W	metabolic process	mitochondrion	catalytic activity;nucleotide binding	0.083	0.083	1.00
P36064	COX assembly mitochondrial protein [OS=Saccharomyces cerevisiae S288c]	853721	CMC1; YKL137W	cell organization and biogenesis	membrane;mitochondrion	metal ion binding	0.389	0.389	1.00
P33314	Inhibitory regulator protein BUD2/CLA2 [OS=Saccharomyces cerevisiae S288c]	853770	BUD2; YKL092C	cell division;regulation of biological process;reproduction;response to stimulus	cytoplasm;membrane	enzyme regulator activity;protein binding	0.031	0.031	1.00
P25038	Translation initiation factor IF-2, mitochondrial [OS=Saccharomyces cerevisiae S288c]	854135	IFM1; YOL023W	cell organization and biogenesis;metabolic process	mitochondrion;nucleus	catalytic activity;nucleotide binding;RNA binding	0.054	0.054	1.00
P87275	Altered inheritance of mitochondria protein 11 [OS=Saccharomyces cerevisiae S288c]	856829	AIM11; YER093C-A		membrane;mitochondrion		0.389	0.389	1.00
Q12497	Protein FMP16, mitochondrial [OS=Saccharomyces cerevisiae S288c]	851642	FMP16; YDR070C		mitochondrion		0.389	0.389	1.00
Q06698	Putative ATP-dependent RNA helicase YLR419W [OS=Saccharomyces cerevisiae S288c]	851137	YLR419W; YLR419W	metabolic process	cytoplasm;mitochondrion;nucleus	catalytic activity;nucleotide binding;protein binding;RNA binding	0.027	0.027	1.00
P00045	Cytochrome c iso-2 [OS=Saccharomyces cerevisiae S288c]	856672	CYC7; YEL039C	metabolic process;transport	mitochondrion	metal ion binding	0.259	0.259	1.00
P53157	Mitochondrial pyruvate carrier 1 [OS=Saccharomyces cerevisiae S288c]	852800	FMP37; MPC1; YGL080W	transport	membrane;mitochondrion	transporter activity	0.468	0.468	1.00
P39103	Cytochrome c oxidase assembly protein COX14 [OS=Saccharomyces cerevisiae S288c]	854910	COX14; YML129C	cell organization and biogenesis;regulation of biological process	membrane;mitochondrion;organelle lumen	protein binding	0.585	0.585	1.00
P38746	Obg-like ATPase homolog [OS=Saccharomyces cerevisiae S288c]	856372	YLF2; YHL014C		mitochondrion	nucleotide binding	0.093	0.093	1.00
P38071	Enoyl-[acyl-carrier protein] reductase [NADPH, B-specific], mitochondrial [OS=Saccharomyces cerevisiae S288c]	852314	ETR1; YBR026C	metabolic process	mitochondrion;organelle lumen	catalytic activity;DNA binding;metal ion binding	7.111	8.006	1.13
P07246	Alcohol dehydrogenase 3, mitochondrial [OS=Saccharomyces cerevisiae S288c]	855107	ADH3; YMR083W	metabolic process	mitochondrion;organelle lumen	catalytic activity;metal ion binding	10.159	11.452	1.13
P28241	Isocitrate dehydrogenase [NAD] subunit 2, mitochondrial [OS=Saccharomyces cerevisiae S288c]	854303	IDH2; YOR136W	metabolic process	mitochondrion;organelle lumen	catalytic activity;metal ion binding;nucleotide binding;protein binding;RNA binding	6.197	7.031	1.13
P27929	37S ribosomal protein NAM9, mitochondrial [OS=Saccharomyces cerevisiae S288c]	855585	NAM9; YNL137C	cell organization and biogenesis;metabolic process;regulation of biological process	mitochondrion;ribosome	RNA binding;structural molecule activity	0.931	1.062	1.14
P53598	Succinyl-CoA ligase [ADP-forming] subunit alpha, mitochondrial [OS=Saccharomyces cerevisiae S288c]	854310	LSC1; YOR142W	metabolic process	cytosol;mitochondrion	catalytic activity;nucleotide binding	9	10.288	1.14
P25349	Flavoprotein-like protein YCP4 [OS=Saccharomyces cerevisiae	850360	YCP4; YCR004C	metabolic process;regulation of	cytoplasm;membrane;mitochondrion	catalytic activity;nucleotide	6.743	7.799	1.16

	S288c]			biological process		binding;protein binding			
Q04013	Citrate/oxoglutarate carrier protein [OS=Saccharomyces cerevisiae S288c]	855282	YHM2; YMR241 W	cell organization and biogenesis;metabolic process;transport	membrane;mitochondrion	DNA binding;structural molecule activity;transporte r activity	3.467	4.012	1.16
P53292	37S ribosomal protein S35, mitochondrial [OS=Saccharomyces cerevisiae S288c]	853075	MRPS35; YGR165 W	cell organization and biogenesis;metabolic process	mitochondrion;ribosome	structural molecule activity	1.512	1.754	1.16
P32454	Aminopeptidase 2, mitochondrial [OS=Saccharomyces cerevisiae S288c]	853699	APE2; YKL157 W	metabolic process	cytoplasm;extracellular;membrane;mitochondrion ;nucleus	catalytic activity;metal ion binding	1.209	1.474	1.22
P00950	Phosphoglycerate mutase 1 [OS=Saccharomyces cerevisiae S288c]	853705	GPM1; YKL152C	metabolic process;regulation of biological process	cytoplasm;cytosol;membrane;mitochondrion	catalytic activity	2.981	3.642	1.22
P00175	Cytochrome b2, mitochondrial [OS=Saccharomyces cerevisiae S288c]	854950	CYB2; YML054C	metabolic process;transport	cytosol;membrane;mitochondrion;nucleus	catalytic activity;metal ion binding;nucleotide binding	1.113	1.371	1.23
P38771	Ribosome-recycling factor, mitochondrial [OS=Saccharomyces cerevisiae S288c]	856433	RRF1; YHR038 W	cell organization and biogenesis;metabolic process	mitochondrion	RNA binding	1.254	1.581	1.26
P21954	Isocitrate dehydrogenase [NADP], mitochondrial [OS=Saccharomyces cerevisiae S288c]	851493	IDP1; YDL066 W	metabolic process	mitochondrion	catalytic activity;metal ion binding;nucleotide binding;protein binding	35.869	45.416	1.27
P32340	Rotenone-insensitive NADH-ubiquinone oxidoreductase, mitochondrial [OS=Saccharomyces cerevisiae S288c]	854919	NDI1; YML120C	metabolic process;regulation of biological process	membrane;mitochondrion;organelle lumen	catalytic activity;nucleotide binding	22.95	29.392	1.28
P28625	Protein YIM1 [OS=Saccharomyces cerevisiae S288c]	855183	YIM1; YMR152 W	metabolic process;response to stimulus	cytoplasm;endoplasmic reticulum;mitochondrion	catalytic activity;metal ion binding	0.833	1.069	1.28
P40582	Glutathione S-transferase 1 [OS=Saccharomyces cerevisiae S288c]	854856	GTT1; YIR038C	metabolic process	endoplasmic reticulum;membrane;mitochondrion;nucleus	antioxidant activity;catalytic activity;protein binding;RNA binding	1.683	2.162	1.28
P0CS90	Heat shock protein SSC1, mitochondrial [OS=Saccharomyces cerevisiae S288c]	853503	SSC1; YJR045C	cell organization and biogenesis;metabolic process;regulation of biological process;transport	membrane;mitochondrion;nucleus;organelle lumen	catalytic activity;enzyme regulator activity;nucleotide binding;protein binding	51.75	67.129	1.30
P53969	Sorting assembly machinery 50 kDa subunit [OS=Saccharomyces cerevisiae S288c]	855705	SAM50; YNL026 W	cell organization and biogenesis;transport	membrane;mitochondrion	protein binding;transporte r activity	0.374	0.487	1.30
P07213	Mitochondrial import receptor subunit TOM70 [OS=Saccharomyces cerevisiae S288c]	855602	TOM70; YNL121C	cell organization and biogenesis;transport	membrane;mitochondrion	protein binding;transporte r activity	1.728	2.257	1.31
P32611	54S ribosomal protein RML2, mitochondrial [OS=Saccharomyces cerevisiae S288c]	856660	RML2; YEL050C	cell organization and biogenesis;metabolic process	mitochondrion;ribosome	catalytic activity;RNA binding;structural molecule activity	0.389	0.509	1.31
Q01852	Mitochondrial import inner membrane translocase subunit TIM44 [OS=Saccharomyces cerevisiae S288c]	854790	TIM44; YIL022W	transport	membrane;mitochondrion	nucleotide binding;protein binding	4.125	5.404	1.31
P38988	Mitochondrial GTP/GDP carrier protein 1 [OS=Saccharomyces cerevisiae S288c]	851329	GGC1; YDL198C	cell organization and biogenesis;cellular homeostasis;metabolic process;transport	membrane;mitochondrion	structural molecule activity;transporte r activity	3.642	4.78	1.31
P00424	Cytochrome c oxidase polypeptide 5A, mitochondrial [OS=Saccharomyces cerevisiae S288c]	855675	COX5A; YNL052 W	metabolic process;transport	membrane;mitochondrion	catalytic activity;transporte r activity	1.848	2.511	1.36
P54003	Protein SUR7 [OS=Saccharomyces cerevisiae S288c]	854953	SUR7; YML052 W	cell differentiation;transport	membrane;mitochondrion		0.848	1.154	1.36
P53982	isocitrate dehydrogenase [NADP] [OS=Saccharomyces cerevisiae S288c]	855723	IDP3; YNL009 W	metabolic process	cytoplasm;mitochondrion	catalytic activity;metal ion binding;nucleotide binding	1.943	2.652	1.36
P41921	glutathione reductase [OS=Saccharomyces cerevisiae S288c]	856014	GLR1; YPL091 W	cellular homeostasis;metabolic process;regulation of biological process;response to stimulus	cytoplasm;cytosol;mitochondrion;nucleus	antioxidant activity;catalytic activity;nucleotide binding	0.241	0.334	1.39
P38969	Pentamidine resistance factor, mitochondrial [OS=Saccharomyces cerevisiae S288c]	854440	PNT1; YOR266 W	cell organization and biogenesis;response to stimulus	membrane;mitochondrion		0.259	0.359	1.39

P53230	Phosphatidate cytidylyltransferase, mitochondrial [OS=Saccharomyces cerevisiae S288c]	852937	TAM41; YGR046W	metabolic process	membrane;mitochondrion;organelle lumen	catalytic activity	0.292	0.407	1.39
P38077	ATP synthase subunit gamma, mitochondrial [OS=Saccharomyces cerevisiae S288c]	852327	ATP3; YBR039W	metabolic process;transport	membrane;mitochondrion	catalytic activity;transporter activity	11.328	15.876	1.40
P47140	Altered inheritance rate of mitochondria protein 25 [OS=Saccharomyces cerevisiae S288c]	853563	AIM25; YJR100C	cell organization and biogenesis	membrane;mitochondrion	transporter activity	0.369	0.52	1.41
P22136	ATPase expression protein 2, mitochondrial [OS=Saccharomyces cerevisiae S288c]	855325	AEP2; YMR282C	metabolic process;regulation of biological process	mitochondrion	RNA binding	0.359	0.506	1.41
P09624	Dihydrolipoyl dehydrogenase, mitochondrial [OS=Saccharomyces cerevisiae S288c]	850527	LPD1; YFL018C	cellular homeostasis;metabolic process;regulation of biological process	mitochondrion;organelle lumen	catalytic activity;nucleotide binding	30.623	43.367	1.42
P16622	Ferrochelatase, mitochondrial [OS=Saccharomyces cerevisiae S288c]	854347	HEM15; YOR176W	metabolic process	membrane;mitochondrion	catalytic activity	5.579	8.006	1.44
P50087	MICOS subunit MIC26 [OS=Saccharomyces cerevisiae S288c]	853150	MOS2; MIC26; YGR235C	cell organization and biogenesis	membrane;mitochondrion		0.638	0.931	1.46
P25573	Mitochondrial inner membrane i-AAA protease supercomplex subunit MGR1 [OS=Saccharomyces cerevisiae S288c]	850313	MGR1; YCL044C	metabolic process	membrane;mitochondrion	protein binding	0.701	1.031	1.47
P08466	mitochondrial nuclease [OS=Saccharomyces cerevisiae S288c]	853222	NUC1; YJL208C	cell death;metabolic process	membrane;mitochondrion;nucleus	catalytic activity;metal ion binding	1.336	1.976	1.48
P00560	Phosphoglycerate kinase [OS=Saccharomyces cerevisiae S288c]	850370	PGK1; YCR012W	metabolic process	cytoplasm;membrane;mitochondrion	catalytic activity;nucleotide binding	2.415	3.642	1.51
P12695	Dihydrolipoyllysine-residue acetyltransferase component of pyruvate dehydrogenase complex, mitochondrial [OS=Saccharomyces cerevisiae S288c]	855653	LAT1; YNL071W	metabolic process	mitochondrion;organelle lumen	catalytic activity	7.003	10.602	1.51
P09457	ATP synthase subunit 5, mitochondrial [OS=Saccharomyces cerevisiae S288c]	851892	ATP5; YDR298C	metabolic process;transport	membrane;mitochondrion	catalytic activity;transporter activity	9	13.678	1.52
P00925	Enolase 2 [OS=Saccharomyces cerevisiae S288c]	856579	ENO2; YHR174W	metabolic process;regulation of biological process	cytoplasm;cytosol;membrane;mitochondrion;vacuole	catalytic activity;metal ion binding;protein binding	1.043	1.593	1.53
P23644	Mitochondrial import receptor subunit TOM40 [OS=Saccharomyces cerevisiae S288c]	855243	TOM40; YMR203W	transport	cytosol;membrane;mitochondrion	protein binding;transporter activity	1.154	1.783	1.55
P19882	heat shock protein 60, mitochondrial [OS=Saccharomyces cerevisiae S288c]	850963	HSP60; YLR259C	cell organization and biogenesis;metabolic process;regulation of biological process;transport	cytoplasm;cytosol;membrane;mitochondrion;organelle lumen	catalytic activity;DNA binding;nucleotide binding;protein binding	23.816	36.927	1.55
P00330	Alcohol dehydrogenase 1 [OS=Saccharomyces cerevisiae S288c]	854068	ADH1; YOL086C	metabolic process	cytoplasm;membrane	catalytic activity;metal ion binding	3.833	5.952	1.55
Q06510	Lysophosphatidylcholine acyltransferase [OS=Saccharomyces cerevisiae S288c]	856262	TAZ1; YPR140W	cell organization and biogenesis;metabolic process;transport	membrane;mitochondrion	catalytic activity	0.194	0.304	1.57
P32792	UPF0744 protein YSC83 [OS=Saccharomyces cerevisiae S288c]	856410	YSC83; YHR017W		membrane;mitochondrion		0.233	0.369	1.58
P54783	D-arabinono-1,4-lactone oxidase [OS=Saccharomyces cerevisiae S288c]	854888	ALO1; YML086C	metabolic process;response to stimulus	membrane;mitochondrion	catalytic activity;nucleotide binding	3.299	5.31	1.61
P14693	sorting assembly machinery 35 kDa subunit [OS=Saccharomyces cerevisiae S288c]	856483	SAM35; YHR083W	cell organization and biogenesis;transport	membrane;mitochondrion	protein binding	0.334	0.54	1.62
P22353	54S ribosomal protein L8, mitochondrial [OS=Saccharomyces cerevisiae S288c]	853382	MRPL8; YJL063C	cell organization and biogenesis;metabolic process	mitochondrion;ribosome	structural molecule activity	0.359	0.585	1.63
P34222	Peptidyl-tRNA hydrolase 2 [OS=Saccharomyces cerevisiae S288c]	852223	PTH2; YBL057C	regulation of biological process	cytoplasm;cytosol;membrane;mitochondrion	catalytic activity;protein binding	0.425	0.701	1.65
P20967	2-oxoglutarate dehydrogenase, mitochondrial [OS=Saccharomyces cerevisiae S288c]	854681	KGD1; YIL125W	metabolic process	cytosol;mitochondrion;organelle lumen	catalytic activity	13.785	22.843	1.66

P04840	Mitochondrial outer membrane protein porin 1 [OS=Saccharomyces cerevisiae S288c]	855669	POR1; YNL055C	cell death;cell organization and biogenesis;cellular homeostasis;regulation of biological process;transport	cytoplasm;membrane;mitochondrion	transporter activity	15.238	25.367	1.66
P07251	ATP synthase subunit alpha, mitochondrial [OS=Saccharomyces cerevisiae S288c]	852177	ATP1; YBL099 W	metabolic process;transport	cytosol;membrane;mitochondrion	catalytic activity;nucleotide binding;transporte r activity	34.938	58.948	1.69
P16603	NADPH--cytochrome P450 reductase [OS=Saccharomyces cerevisiae S288c]	856438	NCP1; YHR042 W	metabolic process	cytoplasm;cytosol;endoplasmic reticulum;membrane;mitochondrion	catalytic activity;metal ion binding;nucleotide binding	0.194	0.343	1.77
P38169	Kynurenine 3-monooxygenase [OS=Saccharomyces cerevisiae S288c]	852179	BNA4; YBL098 W	metabolic process	membrane;mitochondrion	catalytic activity;nucleotide binding	0.269	0.487	1.81
P00890	citrate synthase, mitochondrial [OS=Saccharomyces cerevisiae S288c]	855732	CIT1; YNR001C	metabolic process;transport	cytosol;mitochondrion;organelle lumen	catalytic activity	11.798	21.758	1.84
P38626	NADH-cytochrome b5 reductase 1 [OS=Saccharomyces cerevisiae S288c]	854768	CBR1; YIL043C	metabolic process	endoplasmic reticulum;membrane;mitochondrion;nucleus	catalytic activity	0.334	0.616	1.84
P00924	Enolase 1 [OS=Saccharomyces cerevisiae S288c]	853169	ENO1; YGR254 W	metabolic process;regulation of biological process	cytoplasm;cytosol;membrane;mitochondrion;vacu ole	catalytic activity;metal ion binding;protein binding	0.711	1.326	1.86
P53206	putative cysteine synthase [OS=Saccharomyces cerevisiae S288c]	852895	MCY1	metabolic process		catalytic activity	0.817	1.555	1.90
Q07651	SUR7 family protein FMP45 [OS=Saccharomyces cerevisiae S288c]	851304	FMP45; YDL222C	cell differentiation;cell organization and biogenesis	membrane;mitochondrion		3.125	6.017	1.93
Q12466	tricalbin-1 [OS=Saccharomyces cerevisiae S288c]	854253	TCB1; YOR086C	cell organization and biogenesis;regulation of biological process	endoplasmic reticulum;membrane;mitochondrion	metal ion binding;protein binding	0.191	0.369	1.93
P32939	GTP-binding protein ypt7 [OS=Saccharomyces cerevisiae S288c]	855012	YPT7; YML001 W	cell communication;cell organization and biogenesis;metabolic process;regulation of biological process;response to stimulus;transport	cytosol;endosome;membrane;mitochondrion;vacu ole	catalytic activity;nucleotide binding;protein binding	0.585	1.154	1.97
P35180	mitochondrial import receptor subunit Tom20 [OS=Saccharomyces cerevisiae S288c]	852973	TOM20; YGR082 W	cell organization and biogenesis;transport	membrane;mitochondrion	protein binding;transporte r activity	0.701	1.424	2.03
Q02863	ubiquitin carboxyl-terminal hydrolase 16 [OS=Saccharomyces cerevisiae S288c]	856032	UBP16; YPL072 W	metabolic process	membrane;mitochondrion	catalytic activity	0.072	0.15	2.08
P38909	cytochrome c mitochondrial import factor CYC2 [OS=Saccharomyces cerevisiae S288c]	854202	CYC2; YOR037 W	cell organization and biogenesis;metabolic process	membrane;mitochondrion	catalytic activity	0.093	0.194	2.09
P21306	ATP synthase subunit epsilon, mitochondrial [OS=Saccharomyces cerevisiae S288c]	855857	ATP15; YPL271 W	metabolic process;transport	membrane;mitochondrion	catalytic activity;transporte r activity	2.162	4.623	2.14
P05626	ATP synthase subunit 4, mitochondrial [OS=Saccharomyces cerevisiae S288c]	856027	ATP4; YPL078C	cell organization and biogenesis;metabolic process;transport	membrane;mitochondrion	catalytic activity;transporte r activity	3.642	7.799	2.14
Q04472	Mitochondrial inner membrane i-AAA protease supercomplex subunit MGR3 [OS=Saccharomyces cerevisiae S288c]	855142	MGR3; YMR115 W	metabolic process	membrane;mitochondrion	protein binding	0.15	0.322	2.15
Q01574	acetyl-coenzyme A synthetase 1 [OS=Saccharomyces cerevisiae S288c]	851245	ACS1; YAL054C	cell organization and biogenesis;metabolic process	cytoplasm;cytosol;endoplasmic reticulum;mitochondrion;nucleus	catalytic activity;nucleotide binding	0.624	1.482	2.38
P40515	Mitochondria fission 1 protein [OS=Saccharomyces cerevisiae S288c]	854745	FIS1; YIL065C	cell death;cell organization and biogenesis;regulation of biological process	membrane;mitochondrion	protein binding	0.468	1.154	2.47
P00358	glyceraldehyde-3-phosphate dehydrogenase 2 [OS=Saccharomyces cerevisiae S288c]	853465	TDH2; YJR009C	cell death;metabolic process	cytoplasm;cytosol;membrane;mitochondrion;nucl eus	catalytic activity;nucleotide binding;protein binding	1.721	4.484	2.61
P04806	Hexokinase-1 [OS=Saccharomyces cerevisiae S288c]	850614	HXK1; YFR053C	cellular homeostasis;metabolic process;transport	cytoplasm;cytosol;mitochondrion	catalytic activity;nucleotide binding	0.269	0.743	2.76
P16547	Mitochondrial outer membrane protein OM45 [OS=Saccharomyces cerevisiae S288c]	854670	OM45; YIL136W		membrane;mitochondrion		274.42 3	830.76 4	3.03
Q2V2P9	Uncharacterized protein YDR119W-A [OS=Saccharomyces cerevisiae S288c]	379997 0	COX26; YDR119 W-A		membrane;mitochondrion		1.154	3.642	3.16
P39722	Mitochondrial Rho GTPase 1 [OS=Saccharomyces cerevisiae S288c]	851249	GEM1; YAL048C	cell organization and biogenesis;regulation of biological process;response	membrane;mitochondrion	catalytic activity;metal ion binding;nucleotide	0.059	0.189	3.20

				to stimulus;transport		binding			
P53925	Uncharacterized vacuolar membrane protein YNL115C [OS=Saccharomyces cerevisiae S288c]	855608	YNL115C ; YNL115C		membrane;vacuole		0.072	0.233	3.24
P34231	Uncharacterized protein YKL187C [OS=Saccharomyces cerevisiae S288c]	853648	FAT3; YKL187C; YKL187C	cell organization and biogenesis;transport	membrane;mitochondrion		0.093	0.304	3.27
P28737	Protein MSP1 [OS=Saccharomyces cerevisiae S288c]	852915	MSP1; YGR028 W	cell organization and biogenesis;metabolic process;regulation of biological process;response to stimulus;transport	membrane;mitochondrion;nucleus	catalytic activity;nucleotide binding;protein binding;RNA binding	0.122	0.413	3.39
P25087	Sterol 24-C-methyltransferase [OS=Saccharomyces cerevisiae S288c]	855003	ERG6; YML008C	metabolic process	endoplasmic reticulum;membrane;mitochondrion	catalytic activity	0.557	1.894	3.40
P00331	Alcohol dehydrogenase 2 [OS=Saccharomyces cerevisiae S288c]	855349	ADH2; YMR303 C	metabolic process	cytoplasm	catalytic activity;metal ion binding	2.594	9	3.47
Q06405	ATP synthase subunit f, mitochondrial [OS=Saccharomyces cerevisiae S288c]	851983	ATP17; YDR377 W	metabolic process;transport	membrane;mitochondrion	catalytic activity;transporte r activity	1.512	5.31	3.51
P30902	ATP synthase subunit d, mitochondrial [OS=Saccharomyces cerevisiae S288c]	853853	ATP7; YKL016C	metabolic process;transport	membrane;mitochondrion	catalytic activity;transporte r activity	5.105	18.307	3.59
P00942	Triosephosphate isomerase [OS=Saccharomyces cerevisiae S288c]	851620	TPI1; YDR050C	metabolic process	cytoplasm;cytosol;membrane;mitochondrion	catalytic activity	0.194	0.701	3.61
P00360	glyceraldehyde-3-phosphate dehydrogenase 1 [OS=Saccharomyces cerevisiae S288c]	853395	TDH1; YJL052W	metabolic process	cytoplasm;cytosol;membrane;mitochondrion	catalytic activity;nucleotide binding	0.823	3.062	3.72
P34251	Uncharacterized oxidoreductase YKL107W [OS=Saccharomyces cerevisiae S288c]	853753	YKL107 W; YKL107 W	metabolic process	membrane	catalytic activity	0.274	1.069	3.90
P54115	Magnesium-activated aldehyde dehydrogenase, cytosolic [OS=Saccharomyces cerevisiae S288c]	856044	ALD6; YPL061 W	metabolic process;response to stimulus	cytoplasm;cytosol;mitochondrion	catalytic activity	0.077	0.346	4.49
P14540	fructose-bisphosphate aldolase [OS=Saccharomyces cerevisiae S288c]	853805	FBA1; YKL060C	metabolic process	cytoplasm;cytosol;membrane;mitochondrion	catalytic activity;metal ion binding	0.292	1.448	4.96
Q03640	Tricalbin-3 [OS=Saccharomyces cerevisiae S288c]	854903	TCB3; YML072C	cell organization and biogenesis;regulation of biological process	endoplasmic reticulum;membrane;mitochondrion	metal ion binding;protein binding	0.032	0.168	5.25
Q12230	Sphingolipid long chain base-responsive protein LSP1 [OS=Saccharomyces cerevisiae S288c]	856103	LSP1; YPL004C	regulation of biological process;response to stimulus;transport	cytoplasm;membrane;mitochondrion		14.849	84.77	5.71
P28240	isocitrate lyase [OS=Saccharomyces cerevisiae S288c]	856794	ICL1; YER065C	metabolic process	cytoplasm;extracellular;vacuole	catalytic activity;metal ion binding	0.077	0.45	5.84
Q06089	Uncharacterized mitochondrial outer membrane protein YPR098C [OS=Saccharomyces cerevisiae S288c]	856213	YPR098C ; YPR098C		membrane;mitochondrion		0.778	4.623	5.94
Q02981	ABC1 family protein YPL109C, mitochondrial [OS=Saccharomyces cerevisiae S288c]	855995	YPL109C; YPL109C		mitochondrion		0.059	0.413	7.00
P10963	phosphoenolpyruvate carboxykinase [ATP] [OS=Saccharomyces cerevisiae S288c]	853972	PCK1; YKR097 W	metabolic process	cytosol	catalytic activity;nucleotide binding;protein binding	0.166	1.326	7.99
P38825	Protein TOM71 [OS=Saccharomyces cerevisiae S288c]	856517	TOM71; YHR117 W	transport	membrane;mitochondrion	protein binding;transporte r activity	0.061	0.805	13.2 0
P39518	Long-chain-fatty-acid--CoA ligase 2 [OS=Saccharomyces cerevisiae S288c]	856734	FAA2; YER015 W	metabolic process;transport	cytoplasm;mitochondrion	catalytic activity;nucleotide binding	0.055	0.802	14.5 8

Supplemental Table S5. The relative concentrations of proteins in mitochondria purified from WT or *ups1Δ* cells cultured without LCA. Mitochondria were purified from WT or *ups1Δ* cells recovered on day 4 of cell culturing without LCA. Mass spectrometry-based identification and quantitation of proteins recovered in purified mitochondria were performed as described in Materials and methods. The exponentially modified protein abundance index (emPAI) provided by the ″Proteome Discoverer″ software was used to calculate the relative abundance of mitochondrial proteins between specified datasets. Data on the relative concentrations of mitochondrial proteins are presented as means of 2 independent experiments.

Accession	Description	Entrez Gene ID	Gene ID	Biological Process	Cellular Component	Molecular Function	emPAI WT	emPAI *ups1*	Ratio *ups1*/WT
Q12165	ATP synthase subunit delta, mitochondrial	851560	ATP16	metabolic process; transport	membrane; mitochondrion	catalytic activity; transporter activity	4.623	0.334	0.07
P37267	Assembly factor cbp4 [OS=Saccharomyces cerevisiae S288c]		CBP4	cell organization and biogenesis	membrane;mitochondrion		7.111	0.52	0.07
P81449	ATP synthase subunit e, mitochondrial [OS=Saccharomyces cerevisiae S288c]	851922	TIM11; YDR322C-A	cell organization and biogenesis;metabolic process;transport	membrane;mitochondrion	catalytic activity;structural molecule activity;transporter activity	9	0.778	0.09
P19955	37S ribosomal protein YMR-31, mitochondrial [OS=Saccharomyces cerevisiae S288c]	850610	YMR31; YFR049W	cell organization and biogenesis;metabolic process	mitochondrion;ribosome	catalytic activity;protein binding;structural molecule activity	26.826	2.594	0.10
P00128	Cytochrome b-c1 complex subunit 7 [OS=Saccharomyces cerevisiae S288c]	852142	QCR7; YDR529C	cell organization and biogenesis;metabolic process;transport	membrane;mitochondrion	catalytic activity;transporter activity	34.938	3.642	0.10
P53170	[Pyruvate dehydrogenase (acetyl-transferring)] kinase 2, mitochondrial [OS=Saccharomyces cerevisiae S288c]	852821	PKP2; YGL059W	metabolic process;regulation of biological process	mitochondrion;organelle lumen	catalytic activity;nucleotide binding;protein binding	0.778	0.086	0.11
P32799	Cytochrome c oxidase subunit 6A, mitochondrial [OS=Saccharomyces cerevisiae S288c]	852684	COX13; YGL191W	cell organization and biogenesis;metabolic process;regulation of biological process;transport	membrane;mitochondrion	catalytic activity;enzyme regulator activity;transporter activity	18.953	2.162	0.11
P36141	Putative redox protein fmp46, mitochondrial [OS=Saccharomyces cerevisiae S288c]	853923	FMP46; YKR049C	metabolic process	mitochondrion	catalytic activity;protein binding	4.337	0.52	0.12
P53219	Abhydrolase domain-containing protein IMO32 [OS=Saccharomyces cerevisiae S288c]	852919	IMO32; YGR031W	metabolic process;transport	mitochondrion	catalytic activity	0.931	0.116	0.12
P47140	Altered inheritance rate of mitochondria protein 25 [OS=Saccharomyces cerevisiae S288c]	853563	AIM25; YJR100C	cell organization and biogenesis	membrane;mitochondrion	transporter activity	0.874	0.11	0.13
P17695	Glutaredoxin-2, mitochondrial [OS=Saccharomyces cerevisiae S288c]	852124	GRX2; YDR513W	cellular homeostasis;metabolic process;regulation of biological process;response to stimulus;transport	cytoplasm;cytosol;mitochondrion;nucleus	antioxidant activity;catalytic activity	1.848	0.233	0.13
Q04472	Mitochondrial inner membrane i-AAA protease supercomplex subunit MGR3 [OS=Saccharomyces cerevisiae S288c]	855142	MGR3; YMR115W	metabolic process	membrane;mitochondrion	protein binding	0.52	0.072	0.14
Q96VH5	MICOS complex subunit Mic10 [OS=Saccharomyces cerevisiae S288c]	850300	MOS1; MIC10; YCL057C-A	cell organization and biogenesis	membrane;mitochondrion		6.197	0.931	0.15
P53736	Uncharacterized protein YNR040W [OS=Saccharomyces cerevisiae S288c]	855776	YNR040W; YNR040W		mitochondrion		0.73	0.116	0.16
P00427	Cytochrome c oxidase subunit 6, mitochondrial [OS=Saccharomyces cerevisiae S288c]	856448	COX6; YHR051W	metabolic process;transport	membrane;mitochondrion	catalytic activity;metal ion binding;transporter activity	7.254	1.154	0.16
P02381	Ribosomal protein VAR1, mitochondrial [OS=Saccharomyces cerevisiae S288c]	854586	VAR1; Q0140	cell organization and biogenesis;metabolic process	mitochondrion;ribosome	structural molecule activity	0.688	0.11	0.16
P53723	UPF0674 endoplasmic reticulum membrane protein YNR021W [OS=Saccharomyces cerevisiae S288c]	855755	YNR021W; YNR021W		endoplasmic reticulum;membrane	metal ion binding	0.65	0.105	0.16
P38341	MICOS complex subunit MIC12 [OS=Saccharomyces cerevisiae S288c]	852566	AIM5; MIC12; YBR262C	cell organization and biogenesis	membrane;mitochondrion		1.783	0.292	0.16

Q12032	Altered inheritance of mitochondria protein 41, mitochondrial [OS=Saccharomyces cerevisiae S288c]	854390	AIM41; YOR215C		mitochondrion	catalytic activity	2.831	0.468	0.17
P00045	Cytochrome c iso-2 [OS=Saccharomyces cerevisiae S288c]	856672	CYC7; YEL039C	metabolic process;transport	mitochondrion	metal ion binding	1.512	0.259	0.17
P00447	Superoxide dismutase [Mn], mitochondrial [OS=Saccharomyces cerevisiae S288c]	856399	SOD2; YHR008C	metabolic process;response to stimulus	mitochondrion;organelle lumen	antioxidant activity;catalytic activity;metal ion binding	6.017	1.031	0.17
P40452	Cytochrome c oxidase assembly factor 1 [OS=Saccharomyces cerevisiae S288c]	854649	COA1; YIL157C	cell organization and biogenesis	membrane;mitochondrion	protein binding	1.31	0.233	0.18
P23369	54S ribosomal protein L25, mitochondrial [OS=Saccharomyces cerevisiae S288c]	852967	MRPL25; YGR076C	cell organization and biogenesis;metabolic process	mitochondrion;ribosome	structural molecule activity	1.31	0.233	0.18
P32266	Dynamin-like GTPase MGM1, mitochondrial [OS=Saccharomyces cerevisiae S288c]	854386	MGM1; YOR211C	cell organization and biogenesis	membrane;mitochondrion	catalytic activity;nucleotide binding;protein binding	0.448	0.086	0.19
P22353	54S ribosomal protein L8, mitochondrial [OS=Saccharomyces cerevisiae S288c]	853382	MRPL8; YJL063C	cell organization and biogenesis;metabolic process	mitochondrion;ribosome	structural molecule activity	0.848	0.166	0.20
P08525	Cytochrome b-c1 complex subunit 8 [OS=Saccharomyces cerevisiae S288c]	853273	QCR8; YJL166W	metabolic process;transport	membrane;mitochondrion	catalytic activity;transporter activity	2.981	0.585	0.20
P23833	Protein SCO1, mitochondrial [OS=Saccharomyces cerevisiae S288c]	852325	SCO1; YBR037C	cell organization and biogenesis;cellular homeostasis;response to stimulus;transport	membrane;mitochondrion	antioxidant activity;catalytic activity;metal ion binding	1.448	0.292	0.20
P40086	Cytochrome c oxidase assembly protein cox15 [OS=Saccharomyces cerevisiae S288c]	856884	COX15; YER141W	cell organization and biogenesis;metabolic process;transport	membrane;mitochondrion	catalytic activity;transporter activity	2.594	0.532	0.21
P25372	Thioredoxin-3, mitochondrial [OS=Saccharomyces cerevisiae S288c]	850444	TRX3; YCR083W	cellular homeostasis;metabolic process;regulation of biological process;response to stimulus;transport	cytoplasm;mitochondrion	catalytic activity	2.511	0.52	0.21
P28239	Inorganic pyrophosphatase, mitochondrial [OS=Saccharomyces cerevisiae S288c]	855309	PPA2; YMR267W	metabolic process	cytoplasm;mitochondrion	catalytic activity;metal ion binding	0.585	0.122	0.21
P18496	Mitochondrial ATPase complex subunit ATP10 [OS=Saccharomyces cerevisiae S288c]	851109	ATP10; YLR393W	cell organization and biogenesis	cytoplasm;membrane;mitochondrion	protein binding	1.239	0.259	0.21
P36528	54S ribosomal protein L17, mitochondrial [OS=Saccharomyces cerevisiae S288c]	855469	MRPL17; YNL252C	cell organization and biogenesis;metabolic process	mitochondrion;ribosome	structural molecule activity	0.52	0.11	0.21
P47039	Probable kynurenine--oxoglutarate transaminase BNA3 [OS=Saccharomyces cerevisiae S288c]	853386	BNA3; YJL060W	metabolic process;regulation of biological process	cytoplasm;mitochondrion	catalytic activity	0.468	0.101	0.22
P40165	NAD(P)H-hydrate epimerase [OS=Saccharomyces cerevisiae S288c]	855521	NNR1; YNL200C	metabolic process	cytoplasm;mitochondrion	catalytic activity;metal ion binding;nucleotide binding	2.162	0.468	0.22
P53305	Mitochondrial 37S ribosomal protein S27 [OS=Saccharomyces cerevisiae S288c]	853129	RSM27; YGR215W	cell organization and biogenesis;metabolic process	mitochondrion;ribosome	structural molecule activity	2.162	0.468	0.22
P02992	elongation factor Tu, mitochondrial [OS=Saccharomyces cerevisiae S288c]	854359	TUF1; YOR187W	cell organization and biogenesis;metabolic process	mitochondrion	catalytic activity;nucleotide binding;RNA binding	30.623	6.667	0.22
P11325	Leucine--tRNA ligase, mitochondrial [OS=Saccharomyces cerevisiae S288c]	851098	NAM2; YLR382C	cell organization and biogenesis;metabolic process;regulation of biological process	cytosol;mitochondrion;organelle lumen	catalytic activity;nucleotide binding;RNA binding	0.353	0.078	0.22
P25039	Elongation factor G, mitochondrial [OS=Saccharomyces cerevisiae S288c]	850758	MEF1; YLR069C	cell organization and biogenesis;metabolic process	cytosol;mitochondrion	catalytic activity;nucleotide binding;RNA binding	0.931	0.207	0.22
P40098	uncharacterized mitochondrial membrane protein FMP10 [OS=Saccharomyces cerevisiae S288c]	856931	FMP10; YER182W		membrane;mitochondrion		5.952	1.336	0.22
P36066	Protein MRG3-like [OS=Saccharomyces cerevisiae S288c]	853725	YKL133C; YKL133C	metabolic process	membrane;mitochondrion	protein binding	1.054	0.241	0.23
P12687	54S ribosomal protein L2, mitochondrial [OS=Saccharomyces cerevisiae S288c]	855727	MRP7; YNL005C	cell organization and biogenesis;metabolic process	mitochondrion;ribosome	catalytic activity;RNA binding;structural molecule activity	1.565	0.369	0.24
P39525	3-oxoacyl-[acyl-carrier-protein] synthase homolog [OS=Saccharomyces cerevisiae S288c]	856790	CEM1; YER061C	metabolic process	mitochondrion	catalytic activity	2.899	0.688	0.24

Q05892	MIOREX complex component 2 [OS=Saccharomyces cerevisiae S288c]	850997	YLR290C; COQ11; YLR290C	metabolic process	mitochondrion	catalytic activity	3.217	0.778	0.24
P46998	mitochondrial membrane protein FMP33 [OS=Saccharomyces cerevisiae S288c]	853279	FMP33; YJL161W		membrane;mitochondrion		1.371	0.334	0.24
P50088	Stationary phase gene 1 protein [OS=Saccharomyces cerevisiae S288c]	853151	SPG1; YGR236C		endoplasmic reticulum;membrane;mitochondrion		629.957	157.489	0.25
P48569	Uncharacterized protein YDL183C [OS=Saccharomyces cerevisiae S288c]	851345	YDL183C; YDL183C	transport			1.069	0.274	0.26
P19516	Cytochrome c oxidase assembly protein COX11, mitochondrial [OS=Saccharomyces cerevisiae S288c]	855971	COX11; YPL132W	cell organization and biogenesis;metabolic process	membrane;mitochondrion;ribosome	metal ion binding	1.069	0.274	0.26
P38885	Altered inheritance of mitochondria protein 46, mitochondrial [OS=Saccharomyces cerevisiae S288c]	856606	AIM46; YHR199C		mitochondrion	catalytic activity	2.728	0.73	0.27
P38175	37S ribosomal protein MRP21, mitochondrial [OS=Saccharomyces cerevisiae S288c]	852188	MRP21; YBL090W	cell organization and biogenesis;metabolic process	mitochondrion;ribosome	structural molecule activity	0.701	0.194	0.28
P53230	Phosphatidate cytidylyltransferase, mitochondrial [OS=Saccharomyces cerevisiae S288c]	852937	TAM41; YGR046W	metabolic process	membrane;mitochondrion;organelle lumen	catalytic activity	0.668	0.186	0.28
P14693	sorting assembly machinery 35 kDa subunit [OS=Saccharomyces cerevisiae S288c]	856483	SAM35; YHR083W	cell organization and biogenesis;transport	membrane;mitochondrion	protein binding	0.54	0.155	0.29
P48527	Tyrosine--tRNA ligase, mitochondrial [OS=Saccharomyces cerevisiae S288c]	856007	MSY1; YPL097W	metabolic process	cytoplasm;cytosol;mitochondrion;organelle lumen	catalytic activity;nucleotide binding;RNA binding	0.52	0.15	0.29
P25605	Acetolactate synthase small subunit, mitochondrial [OS=Saccharomyces cerevisiae S288c]	850348	ILV6; YCL009C	metabolic process;regulation of biological process	mitochondrion	catalytic activity;enzyme regulator activity	6.848	1.976	0.29
P38323	ATP-dependent clpX-like chaperone, mitochondrial [OS=Saccharomyces cerevisiae S288c]	852528	MCX1; YBR227C	metabolic process	membrane;mitochondrion;organelle lumen	catalytic activity;nucleotide binding	0.501	0.145	0.29
P53081	NGG1-interacting factor 3 [OS=Saccharomyces cerevisiae S288c]	852651	NIF3; YGL221C		cytoplasm;mitochondrion		0.501	0.145	0.29
P32332	Mitochondrial oxaloacetate transport protein [OS=Saccharomyces cerevisiae S288c]	853739	OAC1; YKL120W	metabolic process;transport	membrane;mitochondrion	structural molecule activity;transporter activity	4.995	1.448	0.29
P53311	Mitochondrial pyruvate carrier 3 [OS=Saccharomyces cerevisiae S288c]	853158	FMP43; MPC3; YGR243W	transport	membrane;mitochondrion	transporter activity	1.61	0.468	0.29
Q07500	External NADH-ubiquinone oxidoreductase 2, mitochondrial [OS=Saccharomyces cerevisiae S288c]	851474	NDE2; YDL085W	metabolic process	mitochondrion	catalytic activity;nucleotide binding	13.678	4.012	0.29
P38122	3-methyl-2-oxobutanoate hydroxymethyltransferase [OS=Saccharomyces cerevisiae S288c]	852474	ECM31; YBR176W	metabolic process	mitochondrion	catalytic activity	0.413	0.122	0.30
P54857	lipase 2 [OS=Saccharomyces cerevisiae S288c]	851628	TGL2; YDR058C	metabolic process;transport	mitochondrion	catalytic activity	0.369	0.11	0.30
Q01217	Protein ARG5,6, mitochondrial [OS=Saccharomyces cerevisiae S288c]	856800	ARG5,6; YER069W	metabolic process;regulation of biological process	cytoplasm;mitochondrion;organelle lumen	catalytic activity;nucleotide binding;protein binding	0.286	0.087	0.30
Q03246	37S ribosomal protein S17, mitochondrial [OS=Saccharomyces cerevisiae S288c]	855226	MRPS17; YMR188C	cell differentiation;cell organization and biogenesis;metabolic process	mitochondrion;ribosome	RNA binding;structural molecule activity	1.276	0.389	0.30
P22354	54S ribosomal protein L20, mitochondrial [OS=Saccharomyces cerevisiae S288c]	853960	MRPL20; YKR085C	cell organization and biogenesis;metabolic process	mitochondrion;ribosome	structural molecule activity	1.276	0.389	0.30
P38969	Pentamidine resistance factor, mitochondrial [OS=Saccharomyces cerevisiae S288c]	854440	PNT1; YOR266W	cell organization and biogenesis;response to stimulus	membrane;mitochondrion		0.848	0.259	0.31
P15424	ATP-dependent RNA helicase MSS116, mitochondrial [OS=Saccharomyces cerevisiae S288c]	851775	MSS116; YDR194C	metabolic process;regulation of biological process	mitochondrion;organelle lumen	catalytic activity;nucleotide binding;RNA binding	1.644	0.506	0.31

P54115	Magnesium-activated aldehyde dehydrogenase, cytosolic [OS=Saccharomyces cerevisiae S288c]	856044	ALD6; YPL061W	metabolic process;response to stimulus	cytoplasm;cytosol;mitochondrion	catalytic activity	0.25	0.077	0.31
P39677	Ribosome-releasing factor 2, mitochondrial [OS=Saccharomyces cerevisiae S288c]	853342	MEF2; YJL102W	cell organization and biogenesis;metabolic process	cytosol;mitochondrion	catalytic activity;nucleotide binding;RNA binding	0.179	0.056	0.31
P40513	Mitochondrial acidic protein MAM33 [OS=Saccharomyces cerevisiae S288c]	854740	MAM33; YIL070C	metabolic process;regulation of biological process	mitochondrion;organelle lumen	translation regulator activity	3.642	1.154	0.32
P32445	Single-stranded DNA-binding protein RIM1, mitochondrial [OS=Saccharomyces cerevisiae S288c]	850395	RIM1; YCR028C-A	cell organization and biogenesis;metabolic process;regulation of biological process	mitochondrion	DNA binding	3.642	1.154	0.32
P81451	ATP synthase subunit K, mitochondrial [OS=Saccharomyces cerevisiae S288c]	854077	ATP19; YOL077W-A	cell organization and biogenesis;metabolic process;transport	membrane;mitochondrion	catalytic activity;transporter activity	3.642	1.154	0.32
P40008	Protein FMP52, mitochondrial [OS=Saccharomyces cerevisiae S288c]	856721	FMP52; YER004W		endoplasmic reticulum;membrane;mitochondrion	catalytic activity	3.217	1.054	0.33
P33310	ATP-dependent permease MDL1, mitochondrial [OS=Saccharomyces cerevisiae S288c]	850885	MDL1; YLR188W	transport	membrane;mitochondrion	catalytic activity;nucleotide binding;transporter activity	0.833	0.274	0.33
P40471	NADPH-dependent 1-acyldihydroxyacetone phosphate reductase [OS=Saccharomyces cerevisiae S288c]	854682	AYR1; YIL124W	metabolic process	cytoplasm;endoplasmic reticulum;membrane;mitochondrion	catalytic activity	1.239	0.413	0.33
P12686	37S ribosomal protein MRP13, mitochondrial [OS=Saccharomyces cerevisiae S288c]	852975	MRP13; YGR084C	cell organization and biogenesis;metabolic process	mitochondrion;ribosome	structural molecule activity	1.239	0.413	0.33
P32898	Mitochondrial presequence protease [OS=Saccharomyces cerevisiae S288c]	852041	CYM1; YDR430C	metabolic process	mitochondrion	catalytic activity;metal ion binding	1.349	0.45	0.33
P10662	37S ribosomal protein MRP1, mitochondrial [OS=Saccharomyces cerevisiae S288c]	851948	MRP1; YDR347W	cell organization and biogenesis;metabolic process;response to stimulus	mitochondrion;ribosome	antioxidant activity;catalytic activity;metal ion binding;structural molecule activity	2.981	0.995	0.33
P36163	mitochondrial metalloendopeptidase OMA1 [OS=Saccharomyces cerevisiae S288c]	853962	OMA1; YKR087C	metabolic process	membrane;mitochondrion	catalytic activity;metal ion binding	0.73	0.245	0.34
P38797	Protein phosphatase 2C homolog 7, mitochondrial [OS=Saccharomyces cerevisiae S288c]	856475	PTC7; YHR076W	metabolic process	mitochondrion	catalytic activity;metal ion binding;protein binding	0.688	0.233	0.34
P53326	Uncharacterized protein YGR266W [OS=Saccharomyces cerevisiae S288c]	853182	YGR266W; YGR266W		membrane;mitochondrion		1.081	0.369	0.34
P00410	Cytochrome c oxidase subunit 2 [OS=Saccharomyces cerevisiae S288c]	854622	COX2; Q0250	metabolic process;transport	membrane;mitochondrion	catalytic activity;metal ion binding;protein binding;transporter activity	4.623	1.61	0.35
P28817	3-hydroxyisobutyryl-CoA hydrolase, mitochondrial [OS=Saccharomyces cerevisiae S288c]	851606	EHD3; YDR036C	metabolic process	mitochondrion	catalytic activity	1.395	0.487	0.35
P32860	NifU-like protein, mitochondrial [OS=Saccharomyces cerevisiae S288c]	853826	NFU1; YKL040C	cell organization and biogenesis;cellular homeostasis;metabolic process	mitochondrion;organelle lumen	metal ion binding	3.87	1.371	0.35
P18900	hexaprenyl pyrophosphate synthase, mitochondrial [OS=Saccharomyces cerevisiae S288c]	852288	COQ1; YBR003W	metabolic process	membrane;mitochondrion	catalytic activity;metal ion binding;protein binding	0.468	0.166	0.35
P21306	ATP synthase subunit epsilon, mitochondrial [OS=Saccharomyces cerevisiae S288c]	855857	ATP15; YPL271W	metabolic process;transport	membrane;mitochondrion	catalytic activity;transporter activity	2.162	0.778	0.36
P39006	Phosphatidylserine decarboxylase proenzyme 1, mitochondrial [OS=Saccharomyces cerevisiae S288c]	855552	PSD1; YNL169C	metabolic process;regulation of biological process	membrane;mitochondrion	catalytic activity	0.743	0.269	0.36
P36516	54S ribosomal protein L3, mitochondrial [OS=Saccharomyces cerevisiae S288c]	855039	MRPL3; YMR024W	cell organization and biogenesis;metabolic process;response to stimulus	mitochondrion;nucleus;ribosome	catalytic activity;RNA binding;structural molecule activity	0.743	0.269	0.36
P08067	cytochrome b-c1 complex subunit Rieske, mitochondrial [OS=Saccharomyces cerevisiae S288c]	856689	RIP1; YEL024W	metabolic process;transport	membrane;mitochondrion	catalytic activity;metal ion binding;transporter activity	55.234	20.544	0.37
P46681	D-lactate dehydrogenase [cytochrome] 2, mitochondrial [OS=Saccharomyces cerevisiae S288c]	851376	DLD2; YDL178W	metabolic process	mitochondrion;organelle lumen	catalytic activity;nucleotide binding;protein binding	1.829	0.682	0.37
Q02784	Monothiol glutaredoxin-5, mitochondrial [OS=Saccharomyces	856048	GRX5; YPL059	cell organization and biogenesis;cellular	mitochondrion;organelle lumen	catalytic activity;metal ion	1.783	0.668	0.37

	cerevisiae S288c]		W	homeostasis;metabolic process;regulation of biological process;response to stimulus		binding			
P53732	37S ribosomal protein S12, mitochondrial [OS=Saccharomyces cerevisiae S288c]	855772	MRPS12; YNR036C	cell organization and biogenesis;metabolic process	mitochondrion;ribosome	structural molecule activity	1.783	0.668	0.37
Q12298	uncharacterized ABC transporter ATP-binding protein YDR061W [OS=Saccharomyces cerevisiae S288c]	851633	YDR061W; YDR061W		mitochondrion	catalytic activity;nucleotide binding	0.512	0.194	0.38
Q03798	Altered inheritance of mitochondria protein 36, mitochondrial [OS=Saccharomyces cerevisiae S288c]	855189	AIM36; YMR157C		membrane;mitochondrion		1.637	0.624	0.38
Q04458	Fatty aldehyde dehydrogenase HFD1 [OS=Saccharomyces cerevisiae S288c]	855137	HFD1; YMR110C	metabolic process	endoplasmic reticulum;endosome;membrane;mitochondrion	catalytic activity	4.055	1.555	0.38
P53140	Protein RMD9, mitochondrial [OS=Saccharomyces cerevisiae S288c]	852771	RMD9; YGL107C	cell differentiation;metabolic process;regulation of biological process	membrane;mitochondrion	RNA binding	1.512	0.585	0.39
P40364	Mitochondrial peculiar membrane protein 1 [OS=Saccharomyces cerevisiae S288c]	853379	MPM1; YJL066C		membrane;mitochondrion		1.512	0.585	0.39
P40502	Altered inheritance of mitochondria protein 19, mitochondrial [OS=Saccharomyces cerevisiae S288c]	854722	AIM19; YIL087C		membrane;mitochondrion		1.512	0.585	0.39
P00830	ATP synthase subunit beta, mitochondrial [OS=Saccharomyces cerevisiae S288c]	853585	ATP2; YJR121W	metabolic process;transport	cytosol;membrane;mitochondrion	catalytic activity;nucleotide binding;protein binding;transporter activity	78.433	30.623	0.39
P21560	Protein CBP3, mitochondrial [OS=Saccharomyces cerevisiae S288c]	855886	CBP3; YPL215W	cell organization and biogenesis;regulation of biological process	membrane;mitochondrion;ribosome		1.424	0.557	0.39
P32904	54S ribosomal protein L6, mitochondrial [OS=Saccharomyces cerevisiae S288c]	856552	MRPL6; YHR147C	cell organization and biogenesis;metabolic process	mitochondrion;ribosome	RNA binding;structural molecule activity	1.371	0.54	0.39
P36775	Lon protease homolog, mitochondrial [OS=Saccharomyces cerevisiae S288c]	852259	PIM1; YBL022C	cell organization and biogenesis;metabolic process;regulation of biological process;response to stimulus	cytoplasm;mitochondrion;organelle lumen	catalytic activity;DNA binding;nucleotide binding;RNA binding	1.362	0.537	0.39
Q06892	NADH kinase POS5, mitochondrial [OS=Saccharomyces cerevisiae S288c]	855913	POS5; YPL188W	metabolic process;response to stimulus	mitochondrion;organelle lumen	catalytic activity;nucleotide binding	1.31	0.52	0.40
P53163	54S ribosomal protein L12, mitochondrial [OS=Saccharomyces cerevisiae S288c]	852811	MNP1; YGL068W	cell organization and biogenesis;metabolic process	mitochondrion;ribosome	RNA binding;structural molecule activity	1.31	0.52	0.40
P00175	Cytochrome b2, mitochondrial [OS=Saccharomyces cerevisiae S288c]	854950	CYB2; YML054C	metabolic process;transport	cytosol;membrane;mitochondrion;nucleus	catalytic activity;metal ion binding;nucleotide binding	1.239	0.496	0.40
P07253	cytochrome B pre-mRNA-processing protein 6 [OS=Saccharomyces cerevisiae S288c]	852417	CBP6; YBR120C	cell organization and biogenesis;metabolic process;regulation of biological process	mitochondrion;ribosome		4.179	1.683	0.40
Q02486	ARS-binding factor 2, mitochondrial [OS=Saccharomyces cerevisiae S288c]	855094	ABF2; YMR072W	cell organization and biogenesis;metabolic process;regulation of biological process	chromosome;mitochondrion;nucleus	DNA binding	69.17	27.943	0.40
P40961	prohibitin-1 [OS=Saccharomyces cerevisiae S288c]	853033	PHB1; YGR132C	cell organization and biogenesis;metabolic process;regulation of biological process	membrane;mitochondrion	protein binding	6.848	2.793	0.41
Q04599	54S ribosomal protein L1, mitochondrial [OS=Saccharomyces cerevisiae S288c]	851694	MRPL1; YDR116C	cell organization and biogenesis;metabolic process	mitochondrion;ribosome	RNA binding;structural molecule activity	1.069	0.438	0.41
P00927	Threonine dehydratase, mitochondrial [OS=Saccharomyces cerevisiae S288c]	856819	ILV1; YER086W	metabolic process	cytoplasm;mitochondrion	catalytic activity	3.125	1.285	0.41
P07246	Alcohol dehydrogenase 3, mitochondrial [OS=Saccharomyces cerevisiae S288c]	855107	ADH3; YMR083W	metabolic process	mitochondrion;organelle lumen	catalytic activity;metal ion binding	10.159	4.179	0.41
Q06678	54S ribosomal protein L35, mitochondrial [OS=Saccharomyces cerevisiae S288c]	851921	MRPL35; YDR322W	cell organization and biogenesis;metabolic process	mitochondrion;ribosome	structural molecule activity	1.031	0.425	0.41
P35180	mitochondrial import receptor subunit Tom20 [OS=Saccharomyces cerevisiae S288c]	852973	TOM20; YGR082W	cell organization and biogenesis;transport	membrane;mitochondrion	protein binding;transporter activity	1.031	0.425	0.41
P43617	Uncharacterized mitochondrial carrier YFR045W [OS=Saccharomyces cerevisiae S288c]	850606	YFR045W; YFR045W	metabolic process;transport	membrane;mitochondrion	structural molecule activity;transporter activity	0.995	0.413	0.42
P04037	cytochrome c oxidase subunit 4, mitochondrial [OS=Saccharomyces cerevisiae S288c]	852688	COX4; YGL187C	cell organization and biogenesis;metabolic process;transport	membrane;mitochondrion	catalytic activity;metal ion binding;transporte	42.288	17.738	0.42

						r activity			
P33416	Heat shock protein 78, mitochondrial [OS=Saccharomyces cerevisiae S288c]	851845	HSP78; YDR258C	cell organization and biogenesis;metabolic process;regulation of biological process;response to stimulus	mitochondrion;organelle lumen	catalytic activity;nucleotide binding;protein binding	7.929	3.359	0.42
Q08968	UPF0061 protein FMP40 [OS=Saccharomyces cerevisiae S288c]	855879	FMP40; YPL222W		mitochondrion	protein binding	2.162	0.917	0.42
P25573	Mitochondrial inner membrane i-AAA protease supercomplex subunit MGR1 [OS=Saccharomyces cerevisiae S288c]	850313	MGR1; YCL044C	metabolic process	membrane;mitochondrion	protein binding	1.649	0.701	0.43
P40053	Altered inheritance of mitochondria protein 9, mitochondrial [OS=Saccharomyces cerevisiae S288c]	856813	AIM9; YER080W		mitochondrion		4.555	1.938	0.43
Q06236	Mitochondrial inner membrane protein SHH4 [OS=Saccharomyces cerevisiae S288c]	850861	SHH4; YLR164W	metabolic process	membrane;mitochondrion	catalytic activity;metal ion binding	3.217	1.371	0.43
P36101	tRNA threonylcarbamoyladenosine dehydratase 2 [OS=Saccharomyces cerevisiae S288c]	853841	TCD2; YKL027W; YKL027W	metabolic process	cytosol;membrane;mitochondrion	catalytic activity;nucleotide binding	0.778	0.334	0.43
P32843	Mitochondrial escape protein 2 [OS=Saccharomyces cerevisiae S288c]	855348	YME2; YMR302C	cell organization and biogenesis;metabolic process	membrane;mitochondrion	nucleotide binding;RNA binding	5.158	2.227	0.43
P53220	Mitochondrial import inner membrane translocase subunit TIM21 [OS=Saccharomyces cerevisiae S288c]	852921	TIM21; YGR033C	cell organization and biogenesis;transport	membrane;mitochondrion	protein binding	0.719	0.311	0.43
P07256	Cytochrome b-c1 complex subunit 1, mitochondrial [OS=Saccharomyces cerevisiae S288c]	852235	COR1; YBL045C	metabolic process;transport	membrane;mitochondrion	catalytic activity;metal ion binding;protein binding;transporter activity	18.783	8.183	0.44
P40159	Uncharacterized protein YNL208W [OS=Saccharomyces cerevisiae S288c]	855513	YNL208W; YNL208W		membrane;mitochondrion;ribosome		0.668	0.292	0.44
P40012	protoporphyrinogen oxidase [OS=Saccharomyces cerevisiae S288c]	856733	HEM14; YER014W	metabolic process	membrane;mitochondrion	catalytic activity	2.162	0.947	0.44
P40581	peroxiredoxin HYR1 [OS=Saccharomyces cerevisiae S288c]	854855	HYR1; YIR037W	metabolic process;response to stimulus	cytoplasm;cytosol;mitochondrion;organelle lumen	antioxidant activity;catalytic activity	0.585	0.259	0.44
P40530	[Pyruvate dehydrogenase (acetyl-transferring)] kinase 1, mitochondrial [OS=Saccharomyces cerevisiae S288c]	854769	PKP1; YIL042C	metabolic process	mitochondrion;organelle lumen	catalytic activity;nucleotide binding;protein binding	0.551	0.245	0.44
P36520	54S ribosomal protein L10, mitochondrial [OS=Saccharomyces cerevisiae S288c]	855436	MRPL10; YNL284C	cell organization and biogenesis;metabolic process	mitochondrion;ribosome	structural molecule activity	0.551	0.245	0.44
P38152	Tricarboxylate transport protein [OS=Saccharomyces cerevisiae S288c]	852594	CTP1; YBR291C	metabolic process;transport	membrane;mitochondrion	structural molecule activity;transporter activity	0.551	0.245	0.44
P39522	Dihydroxy-acid dehydratase, mitochondrial [OS=Saccharomyces cerevisiae S288c]	853473	ILV3; YJR016C	metabolic process	mitochondrion	catalytic activity;metal ion binding	29.539	13.175	0.45
P10507	mitochondrial-processing peptidase subunit beta [OS=Saccharomyces cerevisiae S288c]	850860	MAS1; YLR163C	metabolic process;transport	mitochondrion;organelle lumen	catalytic activity;metal ion binding;protein binding	1.371	0.616	0.45
P53312	Succinyl-CoA ligase [ADP-forming] subunit beta, mitochondrial [OS=Saccharomyces cerevisiae S288c]	853159	LSC2; YGR244C	metabolic process	mitochondrion	catalytic activity;nucleotide binding	20.017	9	0.45
Q06567	ABC1 family protein MCP2 [OS=Saccharomyces cerevisiae S288c]	850955	MCP2; YLR253W	cell organization and biogenesis	membrane;mitochondrion		0.484	0.218	0.45
P38702	mitochondrial carrier protein LEU5 [OS=Saccharomyces cerevisiae S288c]	856391	LEU5; YHR002W	metabolic process;transport	membrane;mitochondrion	structural molecule activity;transporter activity	0.468	0.212	0.45
P34224	Uncharacterized protein YBL059W [OS=Saccharomyces cerevisiae S288c]	852221	YBL059W; YBL059W		membrane;mitochondrion		0.468	0.212	0.45
P27929	37S ribosomal protein NAM9, mitochondrial [OS=Saccharomyces cerevisiae S288c]	855585	NAM9; YNL137C	cell organization and biogenesis;metabolic process;regulation of biological process	mitochondrion;ribosome	RNA binding;structural molecule activity	1.062	0.484	0.46
P32839	Mitochondrial chaperone BCS1 [OS=Saccharomyces cerevisiae S288c]	851981	BCS1; YDR375C	cell organization and biogenesis;metabolic process;response to stimulus;transport	cytosol;membrane;mitochondrion	catalytic activity;nucleotide binding;transporter activity	0.425	0.194	0.46

Q01976	ADP-ribose pyrophosphatase [OS=Saccharomyces cerevisiae S288c]	852408	YSA1; YBR111C	metabolic process	cytoplasm;mitochondrion;nucleus	catalytic activity;metal ion binding	0.425	0.194	0.46
Q04487	Mitochondrial inner membrane protein SHH3 [OS=Saccharomyces cerevisiae S288c]	855145	SHH3; YMR118C	metabolic process	membrane;mitochondrion	catalytic activity;metal ion binding	0.425	0.194	0.46
Q12428	Probable 2-methylcitrate dehydratase [OS=Saccharomyces cerevisiae S288c]	856108	PDH1; YPR002W	metabolic process	cytoplasm;membrane;mitochondrion	catalytic activity	18.11	8.306	0.46
Q04172	Sensitive to high expression protein 9, mitochondrial [OS=Saccharomyces cerevisiae S288c]	852002	SHE9; YDR393W	cell organization and biogenesis	membrane;mitochondrion		0.374	0.172	0.46
P25348	54S ribosomal protein L32, mitochondrial [OS=Saccharomyces cerevisiae S288c]	850359	MRPL32; YCR003W	cell organization and biogenesis;metabolic process	membrane;mitochondrion;ribosome	RNA binding;structural molecule activity	0.389	0.179	0.46
Q03713	Respiratory supercomplex factor 1, mitochondrial [OS=Saccharomyces cerevisiae S288c]	854978	RCF1; YML030W	cell organization and biogenesis	membrane;mitochondrion		2.162	0.995	0.46
P17558	37S ribosomal protein PET123, mitochondrial [OS=Saccharomyces cerevisiae S288c]	854329	PET123; YOR158W	cell organization and biogenesis;metabolic process	mitochondrion;ribosome	structural molecule activity	1.448	0.668	0.46
Q06630	Mitochondrial homologous recombination protein 1 [OS=Saccharomyces cerevisiae S288c]	851890	MHR1; YDR296W	cell organization and biogenesis;metabolic process;regulation of biological process;response to stimulus	mitochondrion;nucleus	catalytic activity;DNA binding;structural molecule activity	0.359	0.166	0.46
P48360	Probable NADPH:adrenodoxin oxidoreductase, mitochondrial [OS=Saccharomyces cerevisiae S288c]	851982	ARH1; YDR376W	cellular homeostasis;metabolic process;transport	cytoplasm;membrane;mitochondrion;organelle lumen	catalytic activity	0.346	0.16	0.46
P36523	54S ribosomal protein L15, mitochondrial [OS=Saccharomyces cerevisiae S288c]	851022	MRPL15; YLR312W-A	cell organization and biogenesis;metabolic process	mitochondrion;ribosome	catalytic activity;RNA binding;structural molecule activity	0.334	0.155	0.46
P38523	GrpE protein homolog, mitochondrial [OS=Saccharomyces cerevisiae S288c]	854407	MGE1; YOR232W	metabolic process;regulation of biological process;transport	membrane;mitochondrion;organelle lumen	enzyme regulator activity;nucleotide binding;protein binding	1.336	0.624	0.47
P25270	rRNA methyltransferase 1, mitochondrial [OS=Saccharomyces cerevisiae S288c]	854376	MRM1; YOR201C	metabolic process	mitochondrion	catalytic activity;RNA binding	0.274	0.129	0.47
P39722	Mitochondrial Rho GTPase 1 [OS=Saccharomyces cerevisiae S288c]	851249	GEM1; YAL048C	cell organization and biogenesis;regulation of biological process;response to stimulus;transport	membrane;mitochondrion	catalytic activity;metal ion binding;nucleotide binding	0.259	0.122	0.47
P00445	Superoxide dismutase [Cu-Zn] [OS=Saccharomyces cerevisiae S288c]	853568	SOD1; YJR104C	cell organization and biogenesis;cellular homeostasis;metabolic process;regulation of biological process;response to stimulus	cytoplasm;cytosol;mitochondrion;nucleus	antioxidant activity;catalytic activity;metal ion binding;protein binding	1.848	0.874	0.47
O14467	multiprotein-bridging factor 1 [OS=Saccharomyces cerevisiae S288c]	854474	MBF1; YOR298C-A	metabolic process;regulation of biological process	cytoplasm;mitochondrion;nucleus	DNA binding	1.848	0.874	0.47
P49367	Homoaconitase, mitochondrial [OS=Saccharomyces cerevisiae S288c]	851820	LYS4; YDR234W	metabolic process	mitochondrion	catalytic activity;metal ion binding;protein binding	1.54	0.73	0.47
P36046	Mitochondrial intermembrane space import and assembly protein 40 [OS=Saccharomyces cerevisiae S288c]	853639	MIA40; YKL195W	cellular component movement;metabolic process;response to stimulus;transport	membrane;mitochondrion	catalytic activity;protein binding;transporter activity	3.262	1.555	0.48
P53881	54S ribosomal protein L22, mitochondrial [OS=Saccharomyces cerevisiae S288c]	855544	MRPL22; YNL177C	cell organization and biogenesis;metabolic process	mitochondrion;ribosome	RNA binding;structural molecule activity	1.154	0.551	0.48
P51998	54S ribosomal protein yml6, mitochondrial [OS=Saccharomyces cerevisiae S288c]	854983	YML6; YML025C	cell organization and biogenesis;metabolic process	mitochondrion;ribosome	RNA binding;structural molecule activity	1.154	0.551	0.48
P19262	Dihydrolipoyllysine-residue succinyltransferase component of 2-oxoglutarate dehydrogenase complex, mitochondrial [OS=Saccharomyces cerevisiae S288c]	851726	KGD2; YDR148C	cell organization and biogenesis;metabolic process	mitochondrion	catalytic activity;protein binding	6.406	3.062	0.48
P33311	ATP-dependent permease MDL2, mitochondrial [OS=Saccharomyces cerevisiae S288c]	855858	MDL2; YPL270W	response to stimulus;transport	membrane;mitochondrion	catalytic activity;nucleotide binding;transporter activity	1.116	0.535	0.48
P00401	Cytochrome c oxidase subunit 1 [OS=Saccharomyces cerevisiae S288c]	854598	COX1; Q0045	metabolic process;transport	membrane;mitochondrion	catalytic activity;metal ion binding;transporter activity	1.61	0.778	0.48
Q08818	Meiotic sister-chromatid recombination protein 6, mitochondrial [OS=Saccharomyces cerevisiae S288c]	854536	MSC6; YOR354C	metabolic process	mitochondrion;organelle lumen	RNA binding	1.047	0.506	0.48
P49095	Glycine dehydrogenase (Decarboxylating), mitochondrial	855227	GCV2; YMR18	metabolic process	cytosol;mitochondrion	catalytic activity	2.433	1.184	0.49

	[OS=Saccharomyces cerevisiae S288c]		9W						
P37298	Succinate dehydrogenase [ubiquinone] cytochrome b small subunit, mitochondrial [OS=Saccharomyces cerevisiae S288c]	851758	SDH4; YDR178W	metabolic process;transport	membrane;mitochondrion	catalytic activity;metal ion binding	3.642	1.783	0.49
P32795	Mitochondrial inner membrane i-AAA protease supercomplex subunit YME1 [OS=Saccharomyces cerevisiae S288c]	856135	YME1; YPR024W	metabolic process;response to stimulus;transport	membrane;mitochondrion	catalytic activity;metal ion binding;nucleotide binding;protein binding	1.798	0.891	0.50
P08417	fumarate hydratase, mitochondrial [OS=Saccharomyces cerevisiae S288c]	855866	FUM1; YPL262W	cell organization and biogenesis;metabolic process	cytoplasm;cytosol;mitochondrion;organelle lumen	catalytic activity	13.874	6.88	0.50
P33421	Succinate dehydrogenase [ubiquinone] cytochrome b subunit, mitochondrial [OS=Saccharomyces cerevisiae S288c]	853716	SDH3; YKL141W	cell organization and biogenesis;metabolic process;transport	membrane;mitochondrion	catalytic activity;metal ion binding	1.276	0.638	0.50
P53969	Sorting assembly machinery 50 kDa subunit [OS=Saccharomyces cerevisiae S288c]	855705	SAM50; YNL026W	cell organization and biogenesis;transport	membrane;mitochondrion	protein binding;transporter activity	0.743	0.374	0.50
P25719	Peptidyl-prolyl cis-trans isomerase C, mitochondrial [OS=Saccharomyces cerevisiae S288c]	854897	CPR3; YML078W	cell death;metabolic process	mitochondrion;organelle lumen	catalytic activity	2.981	1.512	0.51
P40035	Mitochondrial phosphate carrier protein 2 [OS=Saccharomyces cerevisiae S288c]	856779	PIC2; YER053C	cellular homeostasis;metabolic process;transport	membrane;mitochondrion	structural molecule activity;transporter activity	1.637	0.833	0.51
Q08023	Protein FMP25, mitochondrial [OS=Saccharomyces cerevisiae S288c]	850766	FMP25; YLR077W	cell organization and biogenesis	membrane;mitochondrion		1.412	0.719	0.51
P47045	mitochondrial import inner membrane translocase subunit TIM54 [OS=Saccharomyces cerevisiae S288c]	853392	TIM54; YJL054W	cell organization and biogenesis;transport	cytosol;membrane;mitochondrion	transporter activity	0.655	0.334	0.51
P38127	mitochondrial carrier protein RIM2 [OS=Saccharomyces cerevisiae S288c]	852491	RIM2; YBR192W	cell organization and biogenesis;metabolic process;transport	membrane;mitochondrion	structural molecule activity;transporter activity	1.512	0.778	0.51
P32335	Protein MSS51, mitochondrial [OS=Saccharomyces cerevisiae S288c]	850900	MSS51; YLR203C	metabolic process;regulation of biological process	membrane;mitochondrion	protein binding;translation regulator activity	1.929	0.995	0.52
P53982	isocitrate dehydrogenase [NADP] [OS=Saccharomyces cerevisiae S288c]	855723	IDP3; YNL009W	metabolic process	cytoplasm;mitochondrion	catalytic activity;metal ion binding;nucleotide binding	2.652	1.371	0.52
P53889	uncharacterized mitochondrial hydrolase FMP41 [OS=Saccharomyces cerevisiae S288c]	855553	FMP41; YNL168C	metabolic process	mitochondrion	catalytic activity;metal ion binding	0.968	0.501	0.52
P25374	Cysteine desulfurase, mitochondrial [OS=Saccharomyces cerevisiae S288c]	850343	NFS1; YCL017C	cell organization and biogenesis;cellular homeostasis;metabolic process	mitochondrion;nucleus	catalytic activity;metal ion binding;protein binding	2.594	1.346	0.52
Q12289	mitochondrial carnitine carrier [OS=Saccharomyces cerevisiae S288c]	854267	CRC1; YOR100C	metabolic process;transport	membrane;mitochondrion	structural molecule activity;transporter activity	1.404	0.73	0.52
P37293	N-terminal acetyltransferase 2 [OS=Saccharomyces cerevisiae S288c]	853050	NAT2; YGR147C	metabolic process	cytoplasm;mitochondrion	catalytic activity	1.404	0.73	0.52
P36151	Uncharacterized protein YKR070W [OS=Saccharomyces cerevisiae S288c]	853944	YKR070W; YKR070W	metabolic process	mitochondrion		0.833	0.438	0.53
P16387	Pyruvate dehydrogenase E1 component subunit alpha, mitochondrial [OS=Saccharomyces cerevisiae S288c]	856925	PDA1; YER178W	cell growth;metabolic process	mitochondrion;organelle lumen	catalytic activity	21.638	11.496	0.53
P43567	alanine--glyoxylate aminotransferase 1 [OS=Saccharomyces cerevisiae S288c]	850514	AGX1; YFL030W	metabolic process	cytosol;mitochondrion	catalytic activity	2.162	1.154	0.53
P01097	ATPase inhibitor, mitochondrial [OS=Saccharomyces cerevisiae S288c]	851347	INH1; YDL181W	metabolic process;regulation of biological process	mitochondrion	catalytic activity;enzyme regulator activity;protein binding	2.162	1.154	0.53
P32317	Protein AFG1 [OS=Saccharomyces cerevisiae S288c]	856658	AFG1; YEL052W	cell organization and biogenesis;metabolic process;response to stimulus;transport	membrane;mitochondrion	catalytic activity;nucleotide binding	1.326	0.711	0.54
Q12031	Mitochondrial 2-methylisocitrate lyase [OS=Saccharomyces cerevisiae S288c]	856114	ICL2; YPR006C	metabolic process	mitochondrion;organelle lumen	catalytic activity	5.158	2.793	0.54

P46367	Potassium-activated aldehyde dehydrogenase, mitochondrial [OS=Saccharomyces cerevisiae S288c]	854556	ALD4; YOR374W	metabolic process	mitochondrion;organelle lumen	catalytic activity	47.497	25.827	0.54
P35191	DnaJ homolog 1, mitochondrial [OS=Saccharomyces cerevisiae S288c]	850530	MDJ1; YFL016C	cell organization and biogenesis;metabolic process;regulation of biological process;response to stimulus	mitochondrion;organelle lumen	enzyme regulator activity;metal ion binding;nucleotide binding;protein binding	3.037	1.656	0.55
Q02883	N-acyl-phosphatidylethanolamine-hydrolyzing phospholipase D, mitochondrial [OS=Saccharomyces cerevisiae S288c]	856001	FMP30; YPL103C	metabolic process	membrane;mitochondrion	catalytic activity;metal ion binding	0.557	0.304	0.55
P39925	Mitochondrial respiratory chain complexes assembly protein AFG3 [OS=Saccharomyces cerevisiae S288c]	856737	AFG3; YER017C	cell organization and biogenesis;metabolic process;response to stimulus;transport	membrane;mitochondrion	catalytic activity;metal ion binding;nucleotide binding;protein binding	1.342	0.734	0.55
Q08822	Probable electron transfer flavoprotein-ubiquinone oxidoreductase, mitochondrial [OS=Saccharomyces cerevisiae S288c]	854538	CIR2; YOR356W	metabolic process;transport	membrane;mitochondrion	catalytic activity;metal ion binding;nucleotide binding	3.424	1.873	0.55
P38071	Enoyl-[acyl-carrier protein] reductase [NADPH, B-specific], mitochondrial [OS=Saccharomyces cerevisiae S288c]	852314	ETR1; YBR026C	metabolic process	mitochondrion;organelle lumen	catalytic activity;DNA binding;metal ion binding	9	4.926	0.55
P39987	Heat shock protein SSC3, mitochondrial [OS=Saccharomyces cerevisiae S288c]	856682	ECM10; YEL030W	cell organization and biogenesis;metabolic process;transport	mitochondrion	nucleotide binding;protein binding	2.162	1.198	0.55
P36525	54S ribosomal protein L24, mitochondrial [OS=Saccharomyces cerevisiae S288c]	855231	MRPL24; YMR193W	cell organization and biogenesis;metabolic process	mitochondrion;ribosome	RNA binding;structural molecule activity	1.512	0.848	0.56
Q05931	Heat shock protein SSQ1, mitochondrial [OS=Saccharomyces cerevisiae S288c]	851084	SSQ1; YLR369W	cell organization and biogenesis;metabolic process	mitochondrion;organelle lumen	nucleotide binding;protein binding	0.884	0.496	0.56
Q06668	Methyltransferase OMS1, mitochondrial [OS=Saccharomyces cerevisiae S288c]	851911	OMS1; YDR316W	metabolic process	membrane;mitochondrion	catalytic activity	0.693	0.389	0.56
P37299	cytochrome b-c1 complex subunit 10 [OS=Saccharomyces cerevisiae S288c]	856390	QCR10; YHR001W-A	metabolic process;transport	membrane;mitochondrion	catalytic activity;transporter activity	5.31	2.981	0.56
Q12029	Probable mitochondrial transport protein fsf1 [OS=Saccharomyces cerevisiae S288c]	854445	FSF1; YOR271C	transport	membrane;mitochondrion	transporter activity	2.36	1.336	0.57
Q12233	ATP synthase subunit g, mitochondrial [OS=Saccharomyces cerevisiae S288c]	856131	ATP20; YPR020W	cell organization and biogenesis;metabolic process;transport	membrane;mitochondrion	catalytic activity;transporter activity	1.371	0.778	0.57
P36147	Presequence translocated-associated motor subunit pam17, mitochondrial [OS=Saccharomyces cerevisiae S288c]	853939	PAM17; YKR065C	transport	membrane;mitochondrion		1.371	0.778	0.57
P37292	Serine hydroxymethyltransferase, mitochondrial [OS=Saccharomyces cerevisiae S288c]	852565	SHM1; YBR263W	metabolic process	mitochondrion	catalytic activity	9	5.136	0.57
P11914	Mitochondrial-processing peptidase subunit alpha [OS=Saccharomyces cerevisiae S288c]	856419	MAS2; YHR024C	metabolic process;transport	membrane;mitochondrion;organelle lumen	catalytic activity;metal ion binding;protein binding	1.291	0.738	0.57
P47052	succinate dehydrogenase [ubiquinone] flavoprotein subunit 2, mitochondrial [OS=Saccharomyces cerevisiae S288c]	853405	YJL045W; YJL045W	metabolic process;transport	membrane;mitochondrion	catalytic activity;nucleotide binding	4.712	2.695	0.57
P38771	Ribosome-recycling factor, mitochondrial [OS=Saccharomyces cerevisiae S288c]	856433	RRF1; YHR038W	cell organization and biogenesis;metabolic process	mitochondrion	RNA binding	1.254	0.719	0.57
Q04935	Cytochrome c oxidase protein 20, mitochondrial [OS=Saccharomyces cerevisiae S288c]	851817	COX20; YDR231C	cell organization and biogenesis;metabolic process	membrane;mitochondrion	protein binding	2.455	1.424	0.58
P04710	ADP,ATP carrier protein 1 [OS=Saccharomyces cerevisiae S288c]	855078	AAC1; YMR056C	metabolic process;transport	cytosol;membrane;mitochondrion	structural molecule activity;transporter activity	11.743	6.848	0.58
P36521	54S ribosomal protein L11, mitochondrial [OS=Saccharomyces cerevisiae S288c]	851325	MRPL11; YDL202W	cell organization and biogenesis;metabolic process	mitochondrion;ribosome	structural molecule activity	1.069	0.624	0.58
P23641	mitochondrial phosphate carrier protein [OS=Saccharomyces cerevisiae S288c]	853540	MIR1; YJR077C	metabolic process;transport	membrane;mitochondrion	protein binding;structural molecule activity;transporter activity	30.623	17.957	0.59
P43635	Citrate synthase 3, mitochondrial [OS=Saccharomyces cerevisiae S288c]	856107	CIT3; YPR001W	metabolic process	mitochondrion	catalytic activity	5.529	3.262	0.59
P32787	Mitochondrial genome maintenance protein MGM101 [OS=Saccharomyces cerevisiae	853609	MGM101; YJR144	cell organization and biogenesis;metabolic process;response to stimulus	chromosome;mitochondrion	DNA binding	3.281	1.976	0.60

	S288c]		W						
P32891	D-lactate dehydrogenase [cytochrome] 1, mitochondrial [OS=Saccharomyces cerevisiae S288c]	851380	DLD1; YDL174C	metabolic process;transport	membrane;mitochondrion	catalytic activity;nucleotide binding	18.684	11.253	0.60
P00431	cytochrome c peroxidase, mitochondrial [OS=Saccharomyces cerevisiae S288c]	853940	CCP1; YKR066C	metabolic process;response to stimulus	mitochondrion;organelle lumen	antioxidant activity;catalytic activity;metal ion binding;protein binding	1.424	0.859	0.60
Q02950	37S ribosomal protein MRP51, mitochondrial [OS=Saccharomyces cerevisiae S288c]	855985	MRP51; YPL118W	cell organization and biogenesis;metabolic process	mitochondrion;ribosome	structural molecule activity	0.668	0.407	0.61
Q12480	Probable electron transfer flavoprotein subunit alpha, mitochondrial [OS=Saccharomyces cerevisiae S288c]	856112	AIM45; YPR004C	metabolic process;transport	mitochondrion;organelle lumen	catalytic activity;nucleotide binding	2.981	1.818	0.61
P07806	Valine--tRNA ligase, mitochondrial [OS=Saccharomyces cerevisiae S288c]	852986	VAS1; YGR094W	metabolic process;regulation of biological process	cytoplasm;cytosol;mitochondrion	catalytic activity;nucleotide binding	0.616	0.377	0.61
P32331	Carrier protein YMC1, mitochondrial [OS=Saccharomyces cerevisiae S288c]	856171	YMC1; YPR058W	metabolic process;transport	membrane;mitochondrion;vacuole	structural molecule activity;transporter activity	1.581	0.968	0.61
P32387	54S ribosomal protein L41, mitochondrial [OS=Saccharomyces cerevisiae S288c]	852014	MRP20; YDR405W	cell organization and biogenesis;metabolic process	mitochondrion;ribosome	nucleotide binding;protein binding;RNA binding;structural molecule activity	1.581	0.968	0.61
Q08179	Mitochondrial distribution and morphology protein 38 [OS=Saccharomyces cerevisiae S288c]	854130	MDM38; YOL027C	cell organization and biogenesis;cellular homeostasis;regulation of biological process;transport	membrane;mitochondrion		6.079	3.732	0.61
P43616	Cys-Gly metallodipeptidase dug1 [OS=Saccharomyces cerevisiae S288c]	850605	DUG1; YFR044C	metabolic process	cytoplasm;mitochondrion;ribosome	catalytic activity;metal ion binding	1.154	0.711	0.62
P05626	ATP synthase subunit 4, mitochondrial [OS=Saccharomyces cerevisiae S288c]	856027	ATP4; YPL078C	cell organization and biogenesis;metabolic process;transport	membrane;mitochondrion	catalytic activity;transporter activity	4.995	3.084	0.62
P38172	MIOREX complex component 3 [OS=Saccharomyces cerevisiae S288c]	852181	YBL095W; MRX3; YBL095W		membrane;mitochondrion		0.501	0.311	0.62
P27697	Atypical kinase COQ8, mitochondrial [OS=Saccharomyces cerevisiae S288c]	852758	COQ8; YGL119W	metabolic process	membrane;mitochondrion;organelle lumen	catalytic activity;nucleotide binding	0.453	0.283	0.62
Q3E824	Uncharacterized protein YOR020W-A [OS=Saccharomyces cerevisiae S288c]	1466480	YOR020W-A; YOR020W-A	metabolic process;transport	membrane;mitochondrion		5.813	3.642	0.63
P19414	Aconitate hydratase, mitochondrial [OS=Saccharomyces cerevisiae S288c]	851013	ACO1; YLR304C	cell organization and biogenesis;metabolic process;transport	cytoplasm;cytosol;mitochondrion;organelle lumen	catalytic activity;DNA binding;metal ion binding	48.619	30.623	0.63
P15179	Aspartate--tRNA ligase, mitochondrial [OS=Saccharomyces cerevisiae S288c]	856000	MSD1; YPL104W	metabolic process	cytoplasm;mitochondrion;organelle lumen	catalytic activity;nucleotide binding	0.379	0.239	0.63
P47127	Altered inheritance of mitochondria protein 24, mitochondrial [OS=Saccharomyces cerevisiae S288c]	853543	AIM24; YJR080C	cell organization and biogenesis	mitochondrion		2.857	1.807	0.63
P38072	Protein SCO2, mitochondrial [OS=Saccharomyces cerevisiae S288c]	852312	SCO2; YBR024W	cell organization and biogenesis;cellular homeostasis;metabolic process;transport	membrane;mitochondrion	antioxidant activity;catalytic activity;metal ion binding	1.154	0.73	0.63
Q05648	MIOREX complex component 10 [OS=Saccharomyces cerevisiae S288c]	851876	YDR282C; MRX10; YDR282C		membrane;mitochondrion		0.35	0.222	0.63
P32191	Glycerol-3-phosphate dehydrogenase, mitochondrial [OS=Saccharomyces cerevisiae S288c]	854651	GUT2; YIL155C	metabolic process	membrane;mitochondrion	catalytic activity	8.522	5.434	0.64
Q01532-1	Cysteine proteinase 1, mitochondrial [OS=Saccharomyces cerevisiae S288c]			metabolic process;regulation of biological process;response to stimulus	cytoplasm;mitochondrion	catalytic activity;DNA binding;RNA binding	0.28	0.179	0.64
P38705	Serine--tRNA ligase, mitochondrial [OS=Saccharomyces cerevisiae S288c]	856402	DIA4; YHR011W	metabolic process	cytoplasm;mitochondrion;organelle lumen	catalytic activity;nucleotide binding	0.233	0.15	0.64
P07236	Threonine--tRNA ligase, mitochondrial [OS=Saccharomyces cerevisiae S288c]	853640	MST1; YKL194C	metabolic process	cytoplasm;mitochondrion;organelle lumen	catalytic activity;nucleotide binding;RNA binding	0.233	0.15	0.64
P42900	Sigma-like sequence protein 1,	8508	SLS1;	cell organization and	membrane;mitochondrion	protein binding	0.194	0.125	0.64

	mitochondrial [OS=Saccharomyces cerevisiae S288c]	30	YLR139C	biogenesis;metabolic process					
P53166	ATP-dependent RNA helicase mrh4, mitochondrial [OS=Saccharomyces cerevisiae S288c]	852816	MRH4; YGL064C	cell organization and biogenesis;metabolic process	mitochondrion;organelle lumen	catalytic activity;nucleotide binding;RNA binding	0.225	0.145	0.64
P25349	Flavoprotein-like protein YCP4 [OS=Saccharomyces cerevisiae S288c]	850360	YCP4; YCR004C	metabolic process;regulation of biological process	cytoplasm;membrane;mitochondrion	catalytic activity;nucleotide binding;protein binding	9	5.813	0.65
P38088	Glycine--tRNA ligase 1, mitochondrial [OS=Saccharomyces cerevisiae S288c]			metabolic process	cytoplasm;mitochondrion;organelle lumen	catalytic activity;nucleotide binding;protein binding	0.616	0.399	0.65
P08466	mitochondrial nuclease [OS=Saccharomyces cerevisiae S288c]	853222	NUC1; YJL208C	cell death;metabolic process	membrane;mitochondrion;nucleus	catalytic activity;metal ion binding	1.637	1.069	0.65
P07275	Delta-1-pyrroline-5-carboxylate dehydrogenase, mitochondrial [OS=Saccharomyces cerevisiae S288c]	856432	PUT2; YHR037W	metabolic process	membrane;mitochondrion;organelle lumen	catalytic activity	6.055	3.977	0.66
Q04728	Arginine biosynthesis bifunctional protein ArgJ, mitochondrial [OS=Saccharomyces cerevisiae S288c]	855084	ARG7; YMR062C	metabolic process	mitochondrion;organelle lumen	catalytic activity	0.63	0.417	0.66
Q06143	mitochondrial dicarboxylate transporter [OS=Saccharomyces cerevisiae S288c]	851063	DIC1; YLR348C	metabolic process;transport	membrane;mitochondrion	structural molecule activity;transporter activity	1.404	0.931	0.66
P40047	Aldehyde dehydrogenase 5, mitochondrial [OS=Saccharomyces cerevisiae S288c]	856804	ALD5; YER073W	metabolic process	mitochondrion;organelle lumen	catalytic activity	2.384	1.581	0.66
Q07349	MIOREX complex component 9 [OS=Saccharomyces cerevisiae S288c]	851535	YDL027C; MRX9; YDL027C		endoplasmic reticulum;membrane;mitochondrion		1.913	1.276	0.67
P38910	10 kDa heat shock protein, mitochondrial [OS=Saccharomyces cerevisiae S288c]	854185	HSP10; YOR020C	cell organization and biogenesis;metabolic process;response to stimulus;transport	cytoplasm;mitochondrion;organelle lumen	metal ion binding;nucleotide binding;protein binding	3.217	2.162	0.67
P21954	Isocitrate dehydrogenase [NADP], mitochondrial [OS=Saccharomyces cerevisiae S288c]	851493	IDP1; YDL066W	metabolic process	mitochondrion	catalytic activity;metal ion binding;nucleotide binding;protein binding	38.811	26.123	0.67
P38325	Mitochondrial outer membrane protein OM14 [OS=Saccharomyces cerevisiae S288c]	852531	OM14; YBR230C	transport	membrane;mitochondrion		6.197	4.179	0.67
P28834	Isocitrate dehydrogenase [NAD] subunit 1, mitochondrial [OS=Saccharomyces cerevisiae S288c]	855691	IDH1; YNL037C	metabolic process;transport	cytosol;mitochondrion;organelle lumen	catalytic activity;metal ion binding;nucleotide binding;protein binding;RNA binding	15.238	10.288	0.68
P50085	Prohibitin-2 [OS=Saccharomyces cerevisiae S288c]	853146	PHB2; YGR231C	cell organization and biogenesis;metabolic process;regulation of biological process	membrane;mitochondrion	protein binding	4.926	3.329	0.68
Q12482	Mitochondrial aspartate-glutamate transporter AGC1 [OS=Saccharomyces cerevisiae S288c]	856132	AGC1; YPR021C	metabolic process;transport	membrane;mitochondrion	structural molecule activity;transporter activity	0.348	0.238	0.68
P16622	Ferrochelatase, mitochondrial [OS=Saccharomyces cerevisiae S288c]	854347	HEM15; YOR176W	metabolic process	membrane;mitochondrion	catalytic activity	6.305	4.337	0.69
Q02776	Mitochondrial import inner membrane translocase subunit TIM50 [OS=Saccharomyces cerevisiae S288c]	856042	TIM50; YPL063W	cell organization and biogenesis;metabolic process;transport	membrane;mitochondrion	catalytic activity;protein binding;transporter activity	9	6.197	0.69
P36013	NAD-dependent malic enzyme, mitochondrial [OS=Saccharomyces cerevisiae S288c]	853839	MAE1; YKL029C	metabolic process	mitochondrion;organelle lumen	catalytic activity;metal ion binding;nucleotide binding	1.985	1.371	0.69
P00560	Phosphoglycerate kinase [OS=Saccharomyces cerevisiae S288c]	850370	PGK1; YCR012W	metabolic process	cytoplasm;membrane;mitochondrion	catalytic activity;nucleotide binding	6.356	4.412	0.69
Q03430	37S ribosomal protein RSM28, mitochondrial [OS=Saccharomyces cerevisiae S288c]	852105	RSM28; YDR494W	cell organization and biogenesis;metabolic process	mitochondrion;ribosome	structural molecule activity	1.462	1.015	0.69
P42940	Probable electron transfer flavoprotein subunit beta [OS=Saccharomyces cerevisiae S288c]	853121	CIR1; YGR207C	metabolic process;transport	mitochondrion;organelle lumen		4.623	3.217	0.70
P38300	Inner membrane mitoribosome receptor MBA1, mitochondrial [OS=Saccharomyces cerevisiae S288c]	852483	MBA1; YBR185C	cell organization and biogenesis;regulation of biological process	membrane;mitochondrion		0.585	0.413	0.71

Q03104	Meiotic sister chromatid recombination protein 1 [OS=Saccharomyces cerevisiae S288c]	854911	MSC1; YML128C	metabolic process	endoplasmic reticulum;membrane;mitochondrion		2.415	1.712	0.71
P48015	Aminomethyltransferase, mitochondrial [OS=Saccharomyces cerevisiae S288c]	851582	GCV1; YDR019C	metabolic process	mitochondrion	catalytic activity;protein binding	9	6.406	0.71
P17505	Malate dehydrogenase, mitochondrial [OS=Saccharomyces cerevisiae S288c]	853777	MDH1; YKL085W	metabolic process	mitochondrion;organelle lumen	catalytic activity;protein binding;RNA binding	56.797	40.596	0.71
P32340	Rotenone-insensitive NADH-ubiquinone oxidoreductase, mitochondrial [OS=Saccharomyces cerevisiae S288c]	854919	NDI1; YML120C	metabolic process;regulation of biological process	membrane;mitochondrion;organelle lumen	catalytic activity;nucleotide binding	24.929	17.874	0.72
P0CS90	Heat shock protein SSC1, mitochondrial [OS=Saccharomyces cerevisiae S288c]	853503	SSC1; YJR045C	cell organization and biogenesis;metabolic process;regulation of biological process;transport	membrane;mitochondrion;nucleus;organelle lumen	catalytic activity;enzyme regulator activity;nucleotide binding;protein binding	48.482	34.938	0.72
P04807	Hexokinase-2 [OS=Saccharomyces cerevisiae S288c]	852639	HXK2; YGL253W	cell communication;cell organization and biogenesis;cellular homeostasis;metabolic process;regulation of biological process;response to stimulus;transport	cytosol;mitochondrion;nucleus	catalytic activity;nucleotide binding	0.359	0.259	0.72
P09440	C-1-tetrahydrofolate synthase, mitochondrial [OS=Saccharomyces cerevisiae S288c]	852378	MIS1; YBR084W	metabolic process	cytosol;mitochondrion	catalytic activity;nucleotide binding	1.049	0.762	0.73
P39726	Glycine cleavage system H protein, mitochondrial [OS=Saccharomyces cerevisiae S288c]	851254	GCV3; YAL044C	metabolic process	mitochondrion	catalytic activity	4.995	3.642	0.73
P54783	D-arabinono-1,4-lactone oxidase [OS=Saccharomyces cerevisiae S288c]	854888	ALO1; YML086C	metabolic process;response to stimulus	membrane;mitochondrion	catalytic activity;nucleotide binding	3.299	2.415	0.73
P39518	Long-chain-fatty-acid--CoA ligase 2 [OS=Saccharomyces cerevisiae S288c]	856734	FAA2; YER015W	metabolic process;transport	cytoplasm;mitochondrion	catalytic activity;nucleotide binding	0.619	0.455	0.74
P52893	probable alanine aminotransferase, mitochondrial [OS=Saccharomyces cerevisiae S288c]	850778	ALT1; YLR089C	metabolic process	mitochondrion;organelle lumen	catalytic activity	1.102	0.812	0.74
P32796-1	Carnitine O-acetyltransferase, mitochondrial [OS=Saccharomyces cerevisiae S288c]	854965	CAT2; YML042W	metabolic process;transport	membrane;mitochondrion	catalytic activity	13.816	10.189	0.74
Q99297	Mitochondrial 2-oxodicarboxylate carrier 2 [OS=Saccharomyces cerevisiae S288c]	854397	ODC2; YOR222W	metabolic process;transport	membrane;mitochondrion	structural molecule activity;transporter activity	1.054	0.778	0.74
P28625	Protein YIM1 [OS=Saccharomyces cerevisiae S288c]	855183	YIM1; YMR152W	metabolic process;response to stimulus	cytoplasm;endoplasmic reticulum;mitochondrion	catalytic activity;metal ion binding	0.833	0.624	0.75
P23180	Probable oxidoreductase AIM17 [OS=Saccharomyces cerevisiae S288c]	856365	AIM17; YHL021C	cell organization and biogenesis;metabolic process	mitochondrion	catalytic activity;metal ion binding	4.623	3.467	0.75
P36534	54S ribosomal protein L40, mitochondrial [OS=Saccharomyces cerevisiae S288c]	855930	MRPL40; YPL173W	cell organization and biogenesis;metabolic process	mitochondrion;ribosome	protein binding;structural molecule activity	0.778	0.585	0.75
P33759	37S ribosomal protein S5, mitochondrial [OS=Saccharomyces cerevisiae S288c]	852553	MRPS5; YBR251W	cell organization and biogenesis;metabolic process	mitochondrion;ribosome	RNA binding;structural molecule activity	0.778	0.585	0.75
P50087	MICOS subunit MIC26 [OS=Saccharomyces cerevisiae S288c]	853150	MOS2; MIC26; YGR235C	cell organization and biogenesis	membrane;mitochondrion		1.683	1.276	0.76
P32902	37S ribosomal protein MRP4, mitochondrial [OS=Saccharomyces cerevisiae S288c]	856384	MRP4; YHL004W	cell organization and biogenesis;metabolic process	mitochondrion;ribosome	structural molecule activity	0.616	0.468	0.76
P07257	Cytochrome b-c1 complex subunit 2, mitochondrial [OS=Saccharomyces cerevisiae S288c]	856321	QCR2; YPR191W	metabolic process;transport	membrane;mitochondrion	catalytic activity;metal ion binding;protein binding;transporter activity	36.751	27.943	0.76
P32473	Pyruvate dehydrogenase E1 component subunit beta, mitochondrial [OS=Saccharomyces cerevisiae S288c]	852522	PDB1; YBR221C	metabolic process	mitochondrion;organelle lumen	catalytic activity	9	6.848	0.76
P54003	Protein SUR7 [OS=Saccharomyces cerevisiae S288c]	854953	SUR7; YML052W	cell differentiation;transport	membrane;mitochondrion		1.512	1.154	0.76
P53598	Succinyl-CoA ligase [ADP-forming] subunit alpha, mitochondrial [OS=Saccharomyces cerevisiae S288c]	854310	LSC1; YOR142W	metabolic process	cytosol;mitochondrion	catalytic activity;nucleotide binding	11.743	9	0.77
Q02981	ABC1 family protein YPL109C, mitochondrial [OS=Saccharomyces cerevisiae S288c]	855995	YPL109C; YPL109C		mitochondrion		0.334	0.259	0.78

P23644	Mitochondrial import receptor subunit TOM40 [OS=Saccharomyces cerevisiae S288c]	855243	TOM40; YMR203W	transport	cytosol;membrane;mitochondrion	protein binding;transporter activity	1.154	0.896	0.78
Q00711	Succinate dehydrogenase [ubiquinone] flavoprotein subunit, mitochondrial [OS=Saccharomyces cerevisiae S288c]	853709	SDH1; YKL148C	metabolic process;transport	membrane;mitochondrion	catalytic activity;nucleotide binding;protein binding	12.242	9.578	0.78
P16451	Pyruvate dehydrogenase complex protein X component, mitochondrial [OS=Saccharomyces cerevisiae S288c]	853107	PDX1; YGR193C	metabolic process	mitochondrion;organelle lumen	catalytic activity;structural molecule activity	2.594	2.03	0.78
P38891	Branched-chain-amino-acid aminotransferase, mitochondrial [OS=Saccharomyces cerevisiae S288c]	856615	BAT1; YHR208W	metabolic process;regulation of biological process;response to stimulus	mitochondrion;organelle lumen	catalytic activity	9	7.111	0.79
P07213	Mitochondrial import receptor subunit TOM70 [OS=Saccharomyces cerevisiae S288c]	855602	TOM70; YNL121C	cell organization and biogenesis;transport	membrane;mitochondrion	protein binding;transporter activity	3.642	2.888	0.79
P32611	54S ribosomal protein RML2, mitochondrial [OS=Saccharomyces cerevisiae S288c]	856660	RML2; YEL050C	cell organization and biogenesis;metabolic process	mitochondrion;ribosome	catalytic activity;RNA binding;structural molecule activity	0.638	0.509	0.80
P40215	External NADH-ubiquinone oxidoreductase 1, mitochondrial [OS=Saccharomyces cerevisiae S288c]	855176	NDE1; YMR145C	metabolic process	mitochondrion	catalytic activity;nucleotide binding	5.469	4.367	0.80
Q08223	Altered inheritance of mitochondria protein 39, mitochondrial [OS=Saccharomyces cerevisiae S288c]	854103	AIM39; YOL053W		cytosol;membrane;mitochondrion		0.585	0.468	0.80
P32453	Protein ATP11, mitochondrial [OS=Saccharomyces cerevisiae S288c]	855401	ATP11; YNL315C	cell organization and biogenesis	mitochondrion	protein binding	1.239	0.995	0.80
Q03976	37S ribosomal protein S24, mitochondrial [OS=Saccharomyces cerevisiae S288c]	851755	RSM24; YDR175C	cell organization and biogenesis;metabolic process	mitochondrion;ribosome	structural molecule activity	0.905	0.738	0.82
P30902	ATP synthase subunit d, mitochondrial [OS=Saccharomyces cerevisiae S288c]	853853	ATP7; YKL016C	metabolic process;transport	membrane;mitochondrion	catalytic activity;transporter activity	5.105	4.179	0.82
P38825	Protein TOM71 [OS=Saccharomyces cerevisiae S288c]	856517	TOM71; YHR117W	transport	membrane;mitochondrion	protein binding;transporter activity	0.512	0.425	0.83
P07143	Cytochrome c1, heme protein, mitochondrial [OS=Saccharomyces cerevisiae S288c]	854231	CYT1; YOR065W	metabolic process;transport	cytosol;membrane;mitochondrion	metal ion binding	23.245	19.309	0.83
Q12166	2-isopropylmalate synthase 2, mitochondrial [OS=Saccharomyces cerevisiae S288c]	854275	LEU9; YOR108W	metabolic process	mitochondrion	catalytic activity;protein binding	2.472	2.065	0.84
P36112	MICOS complex subunit Mic60 [OS=Saccharomyces cerevisiae S288c]	853886	FCJ1; MIC60; YKR016W	cell organization and biogenesis;transport	membrane;mitochondrion		15.379	12.895	0.84
P53266	Cytochrome oxidase assembly protein SHY1 [OS=Saccharomyces cerevisiae S288c]	853009	SHY1; YGR112W	cell organization and biogenesis	membrane;mitochondrion	protein binding	1.154	0.978	0.85
Q03028	Mitochondrial 2-oxodicarboxylate carrier 1 [OS=Saccharomyces cerevisiae S288c]	855969	ODC1; YPL134C	metabolic process;transport	membrane;mitochondrion	structural molecule activity;transporter activity	5.813	4.995	0.86
P40341	Mitochondrial respiratory chain complexes assembly protein YTA12 [OS=Saccharomyces cerevisiae S288c]	855114	YTA12; YMR089C	cell organization and biogenesis;metabolic process;response to stimulus;transport	cytoplasm;membrane;mitochondrion	catalytic activity;metal ion binding;nucleotide binding;protein binding	1.371	1.184	0.86
P36060	NADH-cytochrome b5 reductase 2 [OS=Saccharomyces cerevisiae S288c]	853707	MCR1; YKL150W	metabolic process;response to stimulus	membrane;mitochondrion	catalytic activity;protein binding	15.238	13.384	0.88
P00359	Glyceraldehyde-3-phosphate dehydrogenase 3 [OS=Saccharomyces cerevisiae S288c]	853106	TDH3; YGR192C	cell death;metabolic process	cytoplasm;cytosol;membrane;mitochondrion;nucleus	catalytic activity;nucleotide binding;RNA binding	7.185	6.406	0.89
P38077	ATP synthase subunit gamma, mitochondrial [OS=Saccharomyces cerevisiae S288c]	852327	ATP3; YBR039W	metabolic process;transport	membrane;mitochondrion	catalytic activity;transporter activity	12.689	11.328	0.89
P18239	ADP,ATP carrier protein 2 [OS=Saccharomyces cerevisiae S288c]	852250	PET9; YBL030C	cell death;metabolic process;transport	membrane;mitochondrion	structural molecule activity;transporter activity	110.034	110.034	1.00
P06168	ketol-acid reductoisomerase, mitochondrial [OS=Saccharomyces cerevisiae S288c]	851069	ILV5; YLR355C	cell organization and biogenesis;metabolic process	mitochondrion	catalytic activity;DNA binding;metal ion binding	12.895	12.895	1.00
P07342	Acetolactate synthase catalytic subunit, mitochondrial [OS=Saccharomyces cerevisiae S288c]	855135	ILV2; YMR108W	metabolic process	mitochondrion	catalytic activity;metal ion binding;nucleotide binding	4.223	4.223	1.00

P09457	ATP synthase subunit 5, mitochondrial [OS=Saccharomyces cerevisiae S288c]	851892	ATP5; YDR298C	metabolic process;transport	membrane;mitochondrion	catalytic activity;transporter activity	9	9	1.00
P00358	glyceraldehyde-3-phosphate dehydrogenase 2 [OS=Saccharomyces cerevisiae S288c]	853465	TDH2; YJR009C	cell death;metabolic process	cytoplasm;cytosol;membrane;mitochondrion;nucleus	catalytic activity;nucleotide binding;protein binding	3.962	3.962	1.00
P15108	ATP-dependent molecular chaperone HSC82 [OS=Saccharomyces cerevisiae S288c]	855224	HSC82; YMR186W	cell organization and biogenesis;metabolic process;response to stimulus	cytoplasm;membrane;mitochondrion	catalytic activity;nucleotide binding;protein binding	1.081	1.081	1.00
Q04013	Citrate/oxoglutarate carrier protein [OS=Saccharomyces cerevisiae S288c]	855282	YHM2; YMR241W	cell organization and biogenesis;metabolic process;transport	membrane;mitochondrion	DNA binding;structural molecule activity;transporter activity	3.467	3.467	1.00
P32897	Mitochondrial import inner membrane translocase subunit tim23 [OS=Saccharomyces cerevisiae S288c]	855751	TIM23; YNR017W	transport	membrane;mitochondrion	protein binding;transporter activity	2.981	2.981	1.00
P00044	Cytochrome c iso-1 [OS=Saccharomyces cerevisiae S288c]	853507	CYC1; YJR048W	metabolic process;transport	mitochondrion	metal ion binding;protein binding	9	9	1.00
P40185	Protein mmf1, mitochondrial [OS=Saccharomyces cerevisiae S288c]	854760	MMF1; YIL051C	cell organization and biogenesis;metabolic process	mitochondrion;organelle lumen		5.31	5.31	1.00
P53721	Respiratory supercomplex factor 2, mitochondrial [OS=Saccharomyces cerevisiae S288c]	855752	RCF2; YNR018W	cell organization and biogenesis	membrane;mitochondrion		5.579	5.579	1.00
P00424	Cytochrome c oxidase polypeptide 5A, mitochondrial [OS=Saccharomyces cerevisiae S288c]	855675	COX5A; YNL052W	metabolic process;transport	membrane;mitochondrion	catalytic activity;transporter activity	2.511	2.511	1.00
P40582	Glutathione S-transferase 1 [OS=Saccharomyces cerevisiae S288c]	854856	GTT1; YIR038C	metabolic process	endoplasmic reticulum;membrane;mitochondrion;nucleus	antioxidant activity;catalytic activity;protein binding;RNA binding	1.683	1.683	1.00
P40508	Uncharacterized protein YIL077C [OS=Saccharomyces cerevisiae S288c]	854733	YIL077C; YIL077C		mitochondrion		0.896	0.896	1.00
Q02204	54S ribosomal protein L13, mitochondrial [OS=Saccharomyces cerevisiae S288c]	853875	MRPL13; YKR006C	cell organization and biogenesis;metabolic process	mitochondrion;ribosome	structural molecule activity	1.054	1.054	1.00
P22136	ATPase expression protein 2, mitochondrial [OS=Saccharomyces cerevisiae S288c]	855325	AEP2; YMR282C	metabolic process;regulation of biological process	mitochondrion	RNA binding	0.292	0.292	1.00
P50945	MICOS complex subunit MIC27 [OS=Saccharomyces cerevisiae S288c]	855623	AIM37; MIC27; YNL100W	cell organization and biogenesis	membrane;mitochondrion		0.968	0.968	1.00
Q06493	LETM1 domain-containing protein YLH47, mitochondrial [OS=Saccharomyces cerevisiae S288c]	856243	YLH47; YPR125W	cell organization and biogenesis;transport	membrane;mitochondrion		0.61	0.61	1.00
P42847	37S ribosomal protein S18, mitochondrial [OS=Saccharomyces cerevisiae S288c]	855410	MRPS18; YNL306W	cell organization and biogenesis;metabolic process	mitochondrion;ribosome	RNA binding;structural molecule activity	0.54	0.54	1.00
P21771	37S ribosomal protein S28, mitochondrial [OS=Saccharomyces cerevisiae S288c]	851937	MRPS28; YDR337W	cell organization and biogenesis;metabolic process	mitochondrion;ribosome	RNA binding;structural molecule activity	0.438	0.438	1.00
Q03201	37S ribosomal protein S10, mitochondrial [OS=Saccharomyces cerevisiae S288c]	851611	RSM10; YDR041W	cell organization and biogenesis;metabolic process	mitochondrion;ribosome	structural molecule activity	0.701	0.701	1.00
Q07914	mitochondrial import inner membrane translocase subunit TIM14 [OS=Saccharomyces cerevisiae S288c]	850694	PAM18; YLR008C	regulation of biological process;transport	membrane;mitochondrion	enzyme regulator activity;protein binding;transporter activity	0.778	0.778	1.00
P25345	Asparagine--tRNA ligase, mitochondrial [OS=Saccharomyces cerevisiae S288c]	850388	SLM5; YCR024C	metabolic process	cytoplasm;mitochondrion;organelle lumen	catalytic activity;nucleotide binding	0.172	0.172	1.00
P21375	Fumarate reductase 2 [OS=Saccharomyces cerevisiae S288c]	853510	OSM1; YJR051W	metabolic process	endoplasmic reticulum;mitochondrion	catalytic activity;nucleotide binding	0.25	0.25	1.00
P25038	Translation initiation factor IF-2, mitochondrial [OS=Saccharomyces cerevisiae S288c]	854135	IFM1; YOL023W	cell organization and biogenesis;metabolic process	mitochondrion;nucleus	catalytic activity;nucleotide binding;RNA binding	0.11	0.11	1.00
Q06005	Octanoyltransferase, mitochondrial [OS=Saccharomyces cerevisiae S288c]	850940	LIP2; YLR239C	metabolic process	cytoplasm;mitochondrion	catalytic activity	0.245	0.245	1.00
P47015	Altered inheritance of mitochondria protein 23, mitochondrial [OS=Saccharomyces cerevisiae S288c]	853310	AIM23; YJL131C	metabolic process	mitochondrion	RNA binding	0.35	0.35	1.00

P34222	Peptidyl-tRNA hydrolase 2 [OS=Saccharomyces cerevisiae S288c]	852223	PTH2; YBL057C	regulation of biological process	cytoplasm;cytosol;membrane;mitochondrion	catalytic activity;protein binding	0.425	0.425	1.00
P04803	Tryptophan--tRNA ligase, mitochondrial [OS=Saccharomyces cerevisiae S288c]	851861	MSW1; YDR268W	metabolic process	cytoplasm;mitochondrion;organelle lumen	catalytic activity;nucleotide binding	0.292	0.292	1.00
P22438	Methionine--tRNA ligase, mitochondrial [OS=Saccharomyces cerevisiae S288c]	853081	MSM1; YGR171C	metabolic process	cytoplasm;cytosol;mitochondrion;organelle lumen	catalytic activity;nucleotide binding	0.136	0.136	1.00
P53724	54S ribosomal protein L50, mitochondrial [OS=Saccharomyces cerevisiae S288c]	855756	MRPL50; YNR022C	cell organization and biogenesis;metabolic process	mitochondrion;ribosome	structural molecule activity	0.585	0.585	1.00
Q07560	Cardiolipin synthase (CMP-forming) [OS=Saccharomyces cerevisiae S288c]	851413	CRD1; YDL142C	cell organization and biogenesis;cellular homeostasis;metabolic process	membrane;mitochondrion	catalytic activity	0.166	0.166	1.00
P49954	probable hydrolase NIT3 [OS=Saccharomyces cerevisiae S288c]	851065	NIT3; YLR351C	metabolic process	cytoplasm;mitochondrion	catalytic activity	0.129	0.129	1.00
Q05867	Uncharacterized protein YLR283W, mitochondrial [OS=Saccharomyces cerevisiae S288c]	850988	YLR283W; YLR283W		endoplasmic reticulum;membrane;mitochondrion		0.11	0.11	1.00
Q07534	Solute carrier family 25 member 38 homolog [OS=Saccharomyces cerevisiae S288c]	851439	YDL119C; HEM25; YDL119C	metabolic process;transport	membrane;mitochondrion	structural molecule activity;transporter activity	0.245	0.245	1.00
P32378	4-hydroxybenzoate polyprenyltransferase, mitochondrial [OS=Saccharomyces cerevisiae S288c]	855778	COQ2; YNR041C	metabolic process;transport	membrane;mitochondrion	catalytic activity	0.292	0.292	1.00
Q07821	Iron-sulfur assembly protein 1 [OS=Saccharomyces cerevisiae S288c]	850632	ISA1; YLL027W	cell organization and biogenesis;metabolic process	mitochondrion;organelle lumen	metal ion binding;structural molecule activity	0.136	0.136	1.00
P41911	Glycerol-3-phosphate dehydrogenase [NAD(+)] 2, mitochondrial [OS=Saccharomyces cerevisiae S288c]	854095	GPD2; YOL059W	metabolic process	cytoplasm;cytosol;mitochondrion	catalytic activity;nucleotide binding;protein binding	0.089	0.089	1.00
Q06405	ATP synthase subunit f, mitochondrial [OS=Saccharomyces cerevisiae S288c]	851983	ATP17; YDR377W	metabolic process;transport	membrane;mitochondrion	catalytic activity;transporter activity	1.512	1.512	1.00
P38169	Kynurenine 3-monooxygenase [OS=Saccharomyces cerevisiae S288c]	852179	BNA4; YBL098W	metabolic process	membrane;mitochondrion	catalytic activity;nucleotide binding	0.172	0.172	1.00
P08425	phenylalanine--tRNA ligase, mitochondrial [OS=Saccharomyces cerevisiae S288c]	856160	MSF1; YPR047W	metabolic process	cytoplasm;mitochondrion;organelle lumen	catalytic activity;metal ion binding;nucleotide binding;RNA binding	0.16	0.16	1.00
P32463	Acyl carrier protein, mitochondrial [OS=Saccharomyces cerevisiae S288c]	853642	ACP1; YKL192C	metabolic process;transport	mitochondrion		0.259	0.259	1.00
P38816	thioredoxin reductase 2, mitochondrial [OS=Saccharomyces cerevisiae S288c]	856506	TRR2; YHR106W	cellular homeostasis;metabolic process;regulation of biological process;response to stimulus	cytoplasm;mitochondrion	antioxidant activity;catalytic activity	0.136	0.136	1.00
Q06510	Lysophosphatidylcholine acyltransferase [OS=Saccharomyces cerevisiae S288c]	856262	TAZ1; YPR140W	cell organization and biogenesis;metabolic process;transport	membrane;mitochondrion	catalytic activity	0.093	0.093	1.00
P04039	Cytochrome c oxidase polypeptide VIII, mitochondrial [OS=Saccharomyces cerevisiae S288c]	851111	COX8; YLR395C	metabolic process;transport	membrane;mitochondrion	catalytic activity;transporter activity	0.778	0.778	1.00
P10174	cytochrome c oxidase subunit 7 [OS=Saccharomyces cerevisiae S288c]	855298	COX7; YMR256C	metabolic process;transport	membrane;mitochondrion	catalytic activity;transporter activity	1.154	1.154	1.00
Q05779	Ubiquinone biosynthesis protein COQ9, mitochondrial [OS=Saccharomyces cerevisiae S288c]	850898	COQ9; YLR201C	metabolic process	membrane;mitochondrion		0.166	0.166	1.00
P25578	CDP-diacylglycerol--glycerol-3-phosphate 3-phosphatidyltransferase [OS=Saccharomyces cerevisiae S288c]	850352	PGS1; YCL004W	metabolic process	mitochondrion	catalytic activity;nucleotide binding	0.083	0.083	1.00
P07251	ATP synthase subunit alpha, mitochondrial [OS=Saccharomyces cerevisiae S288c]	852177	ATP1; YBL099W	metabolic process;transport	cytosol;membrane;mitochondrion	catalytic activity;nucleotide binding;transporter activity	34.938	37.312	1.07
P32316	acetyl-CoA hydrolase [OS=Saccharomyces cerevisiae S288c]	852266	ACH1; YBL015W	metabolic process	cytoplasm;cytosol;mitochondrion	catalytic activity	15.298	16.475	1.08
P32454	Aminopeptidase 2, mitochondrial [OS=Saccharomyces cerevisiae S288c]	853699	APE2; YKL157W	metabolic process	cytoplasm;extracellular;membrane;mitochondrion;nucleus	catalytic activity;metal ion binding	0.973	1.049	1.08
P40495	Homoisocitrate dehydrogenase, mitochondrial [OS=Saccharomyces cerevisiae S288c]	854714	LYS12; YIL094C	metabolic process	mitochondrion	catalytic activity;metal ion binding;nucleotide binding	4.109	4.623	1.13

Q01163	37S ribosomal protein S23, mitochondrial [OS=Saccharomyces cerevisiae S288c]	852748	RSM23; YGL129C	cell organization and biogenesis;metabolic process	mitochondrion;ribosome	catalytic activity;nucleotide binding;RNA binding;structural molecule activity	0.983	1.11	1.13
P04840	Mitochondrial outer membrane protein porin 1 [OS=Saccharomyces cerevisiae S288c]	855669	POR1; YNL055C	cell death;cell organization and biogenesis;cellular homeostasis;regulation of biological process;transport	cytoplasm;membrane;mitochondrion	transporter activity	15.238	17.33	1.14
P06208-1	2-isopropylmalate synthase [OS=Saccharomyces cerevisiae S288c]	855619	LEU4; YNL104C	metabolic process	cytoplasm;mitochondrion	catalytic activity;protein binding	5.236	6.017	1.15
P34227	Mitochondrial peroxiredoxin PRX1 [OS=Saccharomyces cerevisiae S288c]	852215	PRX1; YBL064C	cellular homeostasis;metabolic process;regulation of biological process;response to stimulus	mitochondrion	antioxidant activity;catalytic activity	5.158	5.952	1.15
P53292	37S ribosomal protein S35, mitochondrial [OS=Saccharomyces cerevisiae S288c]	853075	MRPS35; YGR165W	cell organization and biogenesis;metabolic process	mitochondrion;ribosome	structural molecule activity	1.512	1.754	1.16
P00360	glyceraldehyde-3-phosphate dehydrogenase 1 [OS=Saccharomyces cerevisiae S288c]	853395	TDH1; YJL052W	metabolic process	cytoplasm;cytosol;membrane;mitochondrion	catalytic activity;nucleotide binding	1.721	2.008	1.17
P53206	putative cysteine synthase [OS=Saccharomyces cerevisiae S288c]	852895	YGR012W; YGR012W	metabolic process	mitochondrion	catalytic activity	0.817	0.978	1.20
P20967	2-oxoglutarate dehydrogenase, mitochondrial [OS=Saccharomyces cerevisiae S288c]	854681	KGD1; YIL125W	metabolic process	cytosol;mitochondrion;organelle lumen	catalytic activity	11.426	13.785	1.21
P39952	Mitochondrial inner membrane protein OXA1 [OS=Saccharomyces cerevisiae S288c]	856898	OXA1; YER154W	cell organization and biogenesis;transport	membrane;mitochondrion	transporter activity	1.371	1.738	1.27
P28241	Isocitrate dehydrogenase [NAD] subunit 2, mitochondrial [OS=Saccharomyces cerevisiae S288c]	854303	IDH2; YOR136W	metabolic process	mitochondrion;organelle lumen	catalytic activity;metal ion binding;nucleotide binding;protein binding;RNA binding	3.642	4.78	1.31
P38988	Mitochondrial GTP/GDP carrier protein 1 [OS=Saccharomyces cerevisiae S288c]	851329	GGC1; YDL198C	cell organization and biogenesis;cellular homeostasis;metabolic process;transport	membrane;mitochondrion	structural molecule activity;transporter activity	3.16	4.179	1.32
P25087	Sterol 24-C-methyltransferase [OS=Saccharomyces cerevisiae S288c]	855003	ERG6; YML008C	metabolic process	endoplasmic reticulum;membrane;mitochondrion	catalytic activity	1.424	1.894	1.33
P12695	Dihydrolipoyllysine-residue acetyltransferase component of pyruvate dehydrogenase complex, mitochondrial [OS=Saccharomyces cerevisiae S288c]	855653	LAT1; YNL071W	metabolic process	mitochondrion;organelle lumen	catalytic activity	6.43	9	1.40
P36517	54S ribosomal protein L4, mitochondrial [OS=Saccharomyces cerevisiae S288c]	851160	MRPL4; YLR439W	cell organization and biogenesis;metabolic process	mitochondrion;ribosome	structural molecule activity	0.369	0.52	1.41
P40416	Iron-sulfur clusters transporter ATM1, mitochondrial [OS=Saccharomyces cerevisiae S288c]	855347	ATM1; YMR301C	cellular homeostasis;metabolic process;transport	membrane;mitochondrion	catalytic activity;nucleotide binding;transporter activity	0.453	0.645	1.42
Q12374	Nuclear control of ATPase protein 2 [OS=Saccharomyces cerevisiae S288c]	856278	NCA2; YPR155C	metabolic process	membrane;mitochondrion		0.468	0.668	1.43
P32939	GTP-binding protein ypt7 [OS=Saccharomyces cerevisiae S288c]	855012	YPT7; YML001W	cell communication;cell organization and biogenesis;metabolic process;regulation of biological process;response to stimulus;transport	cytosol;endosome;membrane;mitochondrion;vacuole	catalytic activity;nucleotide binding;protein binding	0.585	0.848	1.45
Q07651	SUR7 family protein FMP45 [OS=Saccharomyces cerevisiae S288c]	851304	FMP45; YDL222C	cell differentiation;cell organization and biogenesis	membrane;mitochondrion		4.878	7.377	1.51
P33303	Succinate/fumarate mitochondrial transporter [OS=Saccharomyces cerevisiae S288c]	853558	SFC1; YJR095W	metabolic process;transport	membrane;mitochondrion	structural molecule activity;transporter activity	9	13.678	1.52
P30624	Long-chain-fatty-acid--CoA ligase 1 [OS=Saccharomyces cerevisiae S288c]	854495	FAA1; YOR317W	metabolic process;transport	endoplasmic reticulum;membrane;mitochondrion	catalytic activity;nucleotide binding	4.549	7.072	1.55
P00890	citrate synthase, mitochondrial [OS=Saccharomyces cerevisiae S288c]	855732	CIT1; YNR001C	metabolic process;transport	cytosol;mitochondrion;organelle lumen	catalytic activity	9	14.086	1.57
P34231	Uncharacterized protein YKL187C [OS=Saccharomyces cerevisiae S288c]	853648	FAT3; YKL187C; YKL187C	cell organization and biogenesis;transport	membrane;mitochondrion		0.194	0.304	1.57
Q01852	Mitochondrial import inner membrane translocase subunit TIM44 [OS=Saccharomyces cerevisiae S288c]	854790	TIM44; YIL022W	transport	membrane;mitochondrion	nucleotide binding;protein binding	3.758	5.898	1.57

Q06485	Autophagy-related protein 33 [OS=Saccharomyces cerevisiae S288c]	851070	ATG33; YLR356W	cell communication;metabolic process;response to stimulus	membrane;mitochondrion		1.371	2.162	1.58
Q12251	Uncharacterized mitochondrial carrier YPR011C [OS=Saccharomyces cerevisiae S288c]	856121	YPR011C; YPR011C	metabolic process;transport	membrane;mitochondrion	structural molecule activity;transporter activity	0.245	0.389	1.59
P50110	Sorting assembly machinery 37 kDa subunit [OS=Saccharomyces cerevisiae S288c]	855082	SAM37; YMR060C	cell organization and biogenesis;transport	membrane;mitochondrion	protein binding	0.245	0.389	1.59
P41735	5-demethoxyubiquinone hydroxylase, mitochondrial [OS=Saccharomyces cerevisiae S288c]	854292	CAT5; YOR125C	metabolic process	membrane;mitochondrion	catalytic activity;metal ion binding	0.334	0.54	1.62
Q12375	mitochondrial ornithine transporter 1 [OS=Saccharomyces cerevisiae S288c]	854297	ORT1; YOR130C	metabolic process;transport	membrane;mitochondrion	structural molecule activity;transporter activity	0.334	0.54	1.62
Q03327	Mitochondrial fusion and transport protein Ugo1 [OS=Saccharomyces cerevisiae S288c]	852081	UGO1; YDR470C	cell organization and biogenesis;metabolic process	membrane;mitochondrion	protein binding;structural molecule activity	0.425	0.701	1.65
P00942	Triosephosphate isomerase [OS=Saccharomyces cerevisiae S288c]	851620	TPI1; YDR050C	metabolic process	cytoplasm;cytosol;membrane;mitochondrion	catalytic activity	0.425	0.701	1.65
P38297	Mitofusin FZO1 [OS=Saccharomyces cerevisiae S288c]	852477	FZO1; YBR179C	cell organization and biogenesis	membrane;mitochondrion	catalytic activity;nucleotide binding;protein binding	0.142	0.248	1.75
P39002	long-chain-fatty-acid--CoA ligase 3 [OS=Saccharomyces cerevisiae S288c]	854808	FAA3; YIL009W	metabolic process		catalytic activity;nucleotide binding	0.334	0.585	1.75
P21801	Succinate dehydrogenase [ubiquinone] iron-sulfur subunit, mitochondrial [OS=Saccharomyces cerevisiae S288c]	850685	SDH2; YLL041C	metabolic process;transport	membrane;mitochondrion	catalytic activity;metal ion binding	18.684	32.839	1.76
P19882	heat shock protein 60, mitochondrial [OS=Saccharomyces cerevisiae S288c]	850963	HSP60; YLR259C	cell organization and biogenesis;metabolic process;regulation of biological process;transport	cytoplasm;cytosol;membrane;mitochondrion;organelle lumen	catalytic activity;DNA binding;nucleotide binding;protein binding	22.357	39.296	1.76
P39515	mitochondrial import inner membrane translocase subunit Tim17 [OS=Saccharomyces cerevisiae S288c]	853298	TIM17; YJL143W	cell organization and biogenesis;transport	membrane;mitochondrion	protein binding;transporter activity	0.778	1.371	1.76
P09624	Dihydrolipoyl dehydrogenase, mitochondrial [OS=Saccharomyces cerevisiae S288c]	850527	LPD1; YFL018C	cellular homeostasis;metabolic process;regulation of biological process	mitochondrion;organelle lumen	catalytic activity;nucleotide binding	21.539	43.367	2.01
Q00402	Nuclear migration protein NUM1 [OS=Saccharomyces cerevisiae S288c]	851727	NUM1; YDR150W	cell organization and biogenesis;cellular component movement;metabolic process;regulation of biological process;transport	endoplasmic reticulum;mitochondrion	catalytic activity;motor activity;protein binding	0.025	0.052	2.08
Q02863	ubiquitin carboxyl-terminal hydrolase 16 [OS=Saccharomyces cerevisiae S288c]	856032	UBP16; YPL072W	metabolic process	membrane;mitochondrion	catalytic activity	0.072	0.15	2.08
P40015	inositol phosphosphingolipids phospholipase C [OS=Saccharomyces cerevisiae S288c]	856739	ISC1; YER019W	metabolic process;response to stimulus	endoplasmic reticulum;membrane;mitochondrion	catalytic activity;metal ion binding	0.101	0.212	2.10
P38271	Protein OPY1 [OS=Saccharomyces cerevisiae S288c]	852426	OPY1; YBR129C		cytoplasm;mitochondrion		0.129	0.274	2.12
P38884	Altered inheritance of mitochondria protein 18, mitochondrial [OS=Saccharomyces cerevisiae S288c]	856605	AIM18; YHR198C		mitochondrion	catalytic activity	0.145	0.311	2.14
Q08926	ULP1-interacting protein 4 [OS=Saccharomyces cerevisiae S288c]	855916	UIP4; YPL186C		endoplasmic reticulum;membrane;mitochondrion;nucleus		0.136	0.292	2.15
P41921	glutathione reductase [OS=Saccharomyces cerevisiae S288c]	856014	GLR1; YPL091W	cellular homeostasis;metabolic process;regulation of biological process;response to stimulus	cytoplasm;cytosol;mitochondrion;nucleus	antioxidant activity;catalytic activity;nucleotide binding	0.155	0.334	2.15
P16547	Mitochondrial outer membrane protein OM45 [OS=Saccharomyces cerevisiae S288c]	854670	OM45; YIL136W		membrane;mitochondrion		250.189	574.44	2.30
P28737	Protein MSP1 [OS=Saccharomyces cerevisiae S288c]	852915	MSP1; YGR028W	cell organization and biogenesis;metabolic process;regulation of biological process;response to stimulus;transport	membrane;mitochondrion;nucleus	catalytic activity;nucleotide binding;protein binding;RNA binding	0.259	0.778	3.00
P40496	37S ribosomal protein S25, mitochondrial [OS=Saccharomyces cerevisiae S288c]	854715	RSM25; YIL093C	cell organization and biogenesis;metabolic process	mitochondrion;ribosome	structural molecule activity	0.292	0.896	3.07
Q2V2P9	Uncharacterized protein YDR119W-A [OS=Saccharomyces cerevisiae S288c]	3799970	COX26; YDR119W-A		membrane;mitochondrion		1.154	3.642	3.16

P53925	Uncharacterized vacuolar membrane protein YNL115C [OS=Saccharomyces cerevisiae S288c]	855608	YNL115C; YNL115C		membrane;vacuole		0.072	0.233	3.24
P32792	UPF0744 protein YSC83 [OS=Saccharomyces cerevisiae S288c]	856410	YSC83; YHR017W		membrane;mitochondrion		0.11	0.52	4.73
P49334	Mitochondrial import receptor subunit tom22 [OS=Saccharomyces cerevisiae S288c]	855592	TOM22; YNL131W	cell organization and biogenesis;transport	membrane;mitochondrion	protein binding;transporter activity	0.292	1.783	6.11
Q08245	protein ZEO1 [OS=Saccharomyces cerevisiae S288c]	854040	ZEO1; YOL109W	cell organization and biogenesis	membrane;mitochondrion	protein binding	1.512	11.589	7.66

Supplemental Table S6. The relative concentrations of proteins in mitochondria purified from WT or *ups1Δ* cells cultured without LCA. Mitochondria were purified from WT or *ups1Δ* cells recovered on day 7 of cell culturing without LCA. Mass spectrometry-based identification and quantitation of proteins recovered in purified mitochondria were performed as described in Materials and methods. The exponentially modified protein abundance index (emPAI) provided by the ″Proteome Discoverer″ software was used to calculate the relative abundance of mitochondrial proteins between specified datasets. Data on the relative concentrations of mitochondrial proteins are presented as means of 2 independent experiments.

Accession	Description	Entrez Gene ID	Gene ID	Biological Process	Cellular Component	Molecular Function	emPAI WT	emPAI *ups1*	Ratio *ups1* / WT
P36527	54S ribosomal protein L28, mitochondrial	852073	MRPL28	cell organization and biogenesis; metabolic process	mitochondrion; ribosome	structural molecule activity	4.337	0.233	0.05
Q04935	Cytochrome c oxidase protein 20, mitochondrial [OS=Saccharomyces cerevisiae S288c]	851817	COX20; YDR231C	cell organization and biogenesis;metabolic process	membrane;mitochondrion	protein binding	1.894	0.194	0.10
P08525	Cytochrome b-c1 complex subunit 8 [OS=Saccharomyces cerevisiae S288c]	853273	QCR8; YJL166W	metabolic process;transport	membrane;mitochondrion	catalytic activity;transporter activity	5.31	0.585	0.11
P38341	MICOS complex subunit MIC12 [OS=Saccharomyces cerevisiae S288c]	852566	AIM5; MIC12; YBR262C	cell organization and biogenesis	membrane;mitochondrion		2.594	0.292	0.11
P07253	cytochrome B pre-mRNA-processing protein 6 [OS=Saccharomyces cerevisiae S288c]	852417	CBP6; YBR120C	cell organization and biogenesis;metabolic process;regulation of biological process	mitochondrion;ribosome		3.394	0.389	0.11
P36141	Putative redox protein fmp46, mitochondrial [OS=Saccharomyces cerevisiae S288c]	853923	FMP46; YKR049C	metabolic process	mitochondrion	catalytic activity;protein binding	4.337	0.52	0.12
P53219	Abhydrolase domain-containing protein IMO32 [OS=Saccharomyces cerevisiae S288c]	852919	IMO32; YGR031W	metabolic process;transport	mitochondrion	catalytic activity	0.73	0.116	0.16
P00045	Cytochrome c iso-2 [OS=Saccharomyces cerevisiae S288c]	856672	CYC7; YEL039C	metabolic process;transport	mitochondrion	metal ion binding	1.512	0.259	0.17
Q12032	Altered inheritance of mitochondria protein 41, mitochondrial [OS=Saccharomyces cerevisiae S288c]	854390	AIM41; YOR215C		mitochondrion	catalytic activity	1.154	0.212	0.18
P32799	Cytochrome c oxidase subunit 6A, mitochondrial [OS=Saccharomyces cerevisiae S288c]	852684	COX13; YGL191W	cell organization and biogenesis;metabolic process;regulation of biological process;transport	membrane;mitochondrion	catalytic activity;enzyme regulator activity;transporter activity	11.589	2.162	0.19
P38175	37S ribosomal protein MRP21, mitochondrial [OS=Saccharomyces cerevisiae S288c]	852188	MRP21; YBL090W	cell organization and biogenesis;metabolic process	mitochondrion;ribosome	structural molecule activity	1.031	0.194	0.19
P53305	Mitochondrial 37S ribosomal protein S27 [OS=Saccharomyces cerevisiae S288c]	853129	RSM27; YGR215W	cell organization and biogenesis;metabolic process	mitochondrion;ribosome	structural molecule activity	2.162	0.468	0.22
P54115	Magnesium-activated aldehyde dehydrogenase, cytosolic [OS=Saccharomyces cerevisiae S288c]	856044	ALD6; YPL061W	metabolic process;response to stimulus	cytoplasm;cytosol;mitochondrion	catalytic activity	0.346	0.077	0.22
P22438	Methionine--tRNA ligase, mitochondrial [OS=Saccharomyces cerevisiae S288c]	853081	MSM1; YGR171C	metabolic process	cytoplasm;cytosol;mitochondrion;organelle lumen	catalytic activity;nucleotide binding	0.292	0.066	0.23
P18496	Mitochondrial ATPase complex subunit ATP10 [OS=Saccharomyces cerevisiae S288c]	851109	ATP10; YLR393W	cell organization and biogenesis	cytoplasm;membrane;mitochondrion	protein binding	1.818	0.413	0.23
P18900	hexaprenyl pyrophosphate synthase, mitochondrial [OS=Saccharomyces cerevisiae S288c]	852288	COQ1; YBR003W	metabolic process	membrane;mitochondrion	catalytic activity;metal ion binding;protein binding	0.711	0.166	0.23
P48360	Probable NADPH:adrenodoxin oxidoreductase, mitochondrial [OS=Saccharomyces cerevisiae S288c]	851982	ARH1; YDR376W	cellular homeostasis;metabolic process;transport	cytoplasm;membrane;mitochondrion;organelle lumen	catalytic activity	0.682	0.16	0.23
P81449	ATP synthase subunit e, mitochondrial [OS=Saccharomyces cerevisiae S288c]	851922	TIM11; YDR322C-A	cell organization and biogenesis;metabolic process;transport	membrane;mitochondrion	catalytic activity;structural molecule activity;transporter activity	9	2.162	0.24
Q02784	Monothiol glutaredoxin-5, mitochondrial [OS=Saccharomyces cerevisiae S288c]	856048	GRX5; YPL059W	cell organization and biogenesis;cellular homeostasis;metabolic process;regulation of biological process;response to stimulus	mitochondrion;organelle lumen	catalytic activity;metal ion binding	2.594	0.668	0.26
Q6Q560	Protein isd11 [OS=Saccharomyces cerevisiae S288c]	856774	ISD11; YER048W-A	cell organization and biogenesis;metabolic process	mitochondrion;organelle lumen	catalytic activity;protein binding	2.594	0.668	0.26
Q08223	Altered inheritance of mitochondria protein 39, mitochondrial	854103	AIM39; YOL053		cytosol;membrane;mitochondrion		0.995	0.259	0.26

	[OS=Saccharomyces cerevisiae S288c]		W						
P40581	peroxiredoxin HYR1 [OS=Saccharomyces cerevisiae S288c]	854855	HYR1; YIR037W	metabolic process;response to stimulus	cytoplasm;cytosol;mitochondrion;organelle lumen	antioxidant activity;catalytic activity	0.995	0.259	0.26
P36163	mitochondrial metalloendopeptidase OMA1 [OS=Saccharomyces cerevisiae S288c]	853962	OMA1; YKR087C	metabolic process	membrane;mitochondrion	catalytic activity;metal ion binding	0.931	0.245	0.26
P47140	Altered inheritance rate of mitochondria protein 25 [OS=Saccharomyces cerevisiae S288c]	853563	AIM25; YJR100C	cell organization and biogenesis	membrane;mitochondrion	transporter activity	0.874	0.233	0.27
P53875	54S ribosomal protein L19, mitochondrial [OS=Saccharomyces cerevisiae S288c]	855536	MRPL19; YNL185C	cell organization and biogenesis;metabolic process	mitochondrion;ribosome	RNA binding;structural molecule activity	0.874	0.233	0.27
P40858	54S ribosomal protein L49, mitochondrial [OS=Saccharomyces cerevisiae S288c]	853349	MRPL49; YJL096W	cell organization and biogenesis;metabolic process	mitochondrion;ribosome	RNA binding;structural molecule activity	0.778	0.212	0.27
P40165	NAD(P)H-hydrate epimerase [OS=Saccharomyces cerevisiae S288c]	855521	YNL200C; YNL200C	metabolic process	cytoplasm;mitochondrion	catalytic activity;metal ion binding;nucleotide binding	1.61	0.468	0.29
P38271	Protein OPY1 [OS=Saccharomyces cerevisiae S288c]	852426	OPY1; YBR129C		cytoplasm;mitochondrion		0.438	0.129	0.29
P21560	Protein CBP3, mitochondrial [OS=Saccharomyces cerevisiae S288c]	855886	CBP3; YPL215W	cell organization and biogenesis;regulation of biological process	membrane;mitochondrion;ribosome		1.031	0.304	0.29
Q06236	Mitochondrial inner membrane protein SHH4 [OS=Saccharomyces cerevisiae S288c]	850861	SHH4; YLR164W	metabolic process	membrane;mitochondrion	catalytic activity;metal ion binding	4.623	1.371	0.30
P38705	Serine--tRNA ligase, mitochondrial [OS=Saccharomyces cerevisiae S288c]	856402	DIA4; YHR011W	metabolic process	cytoplasm;mitochondrion;organelle lumen	catalytic activity;nucleotide binding	0.233	0.072	0.31
Q02863	ubiquitin carboxyl-terminal hydrolase 16 [OS=Saccharomyces cerevisiae S288c]	856032	UBP16; YPL072W	metabolic process	membrane;mitochondrion	catalytic activity	0.233	0.072	0.31
P39722	Mitochondrial Rho GTPase 1 [OS=Saccharomyces cerevisiae S288c]	851249	GEM1; YAL048C	cell organization and biogenesis;regulation of biological process;response to stimulus;transport	membrane;mitochondrion	catalytic activity;metal ion binding;nucleotide binding	0.189	0.059	0.31
Q01519	Cytochrome c oxidase subunit 6b [OS=Saccharomyces cerevisiae S288c]	850727	COX12; YLR038C	cell organization and biogenesis;metabolic process;transport	mitochondrion	catalytic activity;transporter activity	3.642	1.154	0.32
P42847	37S ribosomal protein S18, mitochondrial [OS=Saccharomyces cerevisiae S288c]	855410	MRPS18; YNL306W	cell organization and biogenesis;metabolic process	mitochondrion;ribosome	RNA binding;structural molecule activity	1.054	0.334	0.32
Q03653	protein EFR3 [OS=Saccharomyces cerevisiae S288c]	855252	EFR3; YMR212C		membrane;mitochondrion		0.148	0.047	0.32
P11914	Mitochondrial-processing peptidase subunit alpha [OS=Saccharomyces cerevisiae S288c]	856419	MAS2; YHR024C	metabolic process;transport	membrane;mitochondrion;organelle lumen	catalytic activity;metal ion binding;protein binding	2.311	0.738	0.32
Q03798	Altered inheritance of mitochondria protein 36, mitochondrial [OS=Saccharomyces cerevisiae S288c]	855189	AIM36; YMR157C		membrane;mitochondrion		1.336	0.438	0.33
P14908	Mitochondrial transcription factor 1 [OS=Saccharomyces cerevisiae S288c]	855268	MTF1; YMR228W	metabolic process;regulation of biological process	mitochondrion;organelle lumen	catalytic activity;DNA binding;RNA binding	0.778	0.259	0.33
P10834	protein PET54 [OS=Saccharomyces cerevisiae S288c]	853137	PET54; YGR222W	metabolic process;regulation of biological process	membrane;mitochondrion;organelle lumen	nucleotide binding;RNA binding;translation regulator activity	0.688	0.233	0.34
Q12349	ATP synthase subunit H, mitochondrial [OS=Saccharomyces cerevisiae S288c]	851002	ATP14; YLR295C	cell organization and biogenesis;metabolic process;transport	membrane;mitochondrion	catalytic activity;transporter activity	2.728	0.931	0.34
Q05648	MIOREX complex component 10 [OS=Saccharomyces cerevisiae S288c]	851876	YDR282C; MRX10; YDR282C		membrane;mitochondrion		0.65	0.222	0.34
P38088	Glycine--tRNA ligase 1, mitochondrial [OS=Saccharomyces cerevisiae S288c]		GRS1	metabolic process	cytoplasm;mitochondrion;organelle lumen	catalytic activity;nucleotide binding;protein binding	0.616	0.212	0.34
P53166	ATP-dependent RNA helicase mrh4, mitochondrial [OS=Saccharomyces cerevisiae S288c]	852816	MRH4; YGL064C	cell organization and biogenesis;metabolic process	mitochondrion;organelle lumen	catalytic activity;nucleotide binding;RNA binding	1.412	0.501	0.35
P36516	54S ribosomal protein L3, mitochondrial [OS=Saccharomyces cerevisiae S288c]	855039	MRPL3; YMR024W	cell organization and biogenesis;metabolic process;response to stimulus	mitochondrion;nucleus;ribosome	catalytic activity;RNA binding;structural molecule activity	1.043	0.374	0.36
P38120	37S ribosomal protein S9, mitochondrial [OS=Saccharomyces	852443	MRPS9;	cell organization and biogenesis;metabolic process	mitochondrion;ribosome	RNA binding;structural	3.437	1.254	0.36

	cerevisiae S288c]		YBR146W			molecule activity			
P38885	Altered inheritance of mitochondria protein 46, mitochondrial [OS=Saccharomyces cerevisiae S288c]	856606	AIM46; YHR199C		mitochondrion	catalytic activity	1.994	0.73	0.37
P48527	Tyrosine--tRNA ligase, mitochondrial [OS=Saccharomyces cerevisiae S288c]	856007	MSY1; YPL097W	metabolic process	cytoplasm;cytosol;mitochondrion;organelle lumen	catalytic activity;nucleotide binding;RNA binding	0.63	0.233	0.37
P38323	ATP-dependent clpX-like chaperone, mitochondrial [OS=Saccharomyces cerevisiae S288c]	852528	MCX1; YBR227C	metabolic process	membrane;mitochondrion;organelle lumen	catalytic activity;nucleotide binding	0.607	0.225	0.37
P00044	Cytochrome c iso-1 [OS=Saccharomyces cerevisiae S288c]	853507	CYC1; YJR048W	metabolic process;transport	mitochondrion	metal ion binding;protein binding	12.335	4.623	0.37
P40035	Mitochondrial phosphate carrier protein 2 [OS=Saccharomyces cerevisiae S288c]	856779	PIC2; YER053C	cellular homeostasis;metabolic process;transport	membrane;mitochondrion	structural molecule activity;transporter activity	2.793	1.069	0.38
P32453	Protein ATP11, mitochondrial [OS=Saccharomyces cerevisiae S288c]	855401	ATP11; YNL315C	cell organization and biogenesis	mitochondrion	protein binding	1.512	0.585	0.39
P38127	mitochondrial carrier protein RIM2 [OS=Saccharomyces cerevisiae S288c]	852491	RIM2; YBR192W	cell organization and biogenesis;metabolic process;transport	membrane;mitochondrion	structural molecule activity;transporter activity	1.512	0.585	0.39
Q01163	37S ribosomal protein S23, mitochondrial [OS=Saccharomyces cerevisiae S288c]	852748	RSM23; YGL129C	cell organization and biogenesis;metabolic process	mitochondrion;ribosome	catalytic activity;nucleotide binding;RNA binding;structural molecule activity	4.367	1.707	0.39
P40452	Cytochrome c oxidase assembly factor 1 [OS=Saccharomyces cerevisiae S288c]	854649	COA1; YIL157C	cell organization and biogenesis	membrane;mitochondrion	protein binding	1.31	0.52	0.40
P43594	MICOS complex subunit MIC19 [OS=Saccharomyces cerevisiae S288c]	850563	AIM13; MIC19; YFR011C	cell organization and biogenesis	cytoplasm;membrane;mitochondrion		1.31	0.52	0.40
P47015	Altered inheritance of mitochondria protein 23, mitochondrial [OS=Saccharomyces cerevisiae S288c]	853310	AIM23; YJL131C	metabolic process	mitochondrion	RNA binding	1.228	0.492	0.40
P36517	54S ribosomal protein L4, mitochondrial [OS=Saccharomyces cerevisiae S288c]	851160	MRPL4; YLR439W	cell organization and biogenesis;metabolic process	mitochondrion;ribosome	structural molecule activity	2.162	0.874	0.40
P40496	37S ribosomal protein S25, mitochondrial [OS=Saccharomyces cerevisiae S288c]	854715	RSM25; YIL093C	cell organization and biogenesis;metabolic process	mitochondrion;ribosome	structural molecule activity	1.154	0.468	0.41
P23833	Protein SCO1, mitochondrial [OS=Saccharomyces cerevisiae S288c]	852325	SCO1; YBR037C	cell organization and biogenesis;cellular homeostasis;response to stimulus;transport	membrane;mitochondrion	antioxidant activity;catalytic activity;metal ion binding	1.154	0.468	0.41
P48569	Uncharacterized protein YDL183C [OS=Saccharomyces cerevisiae S288c]	851345	YDL183C; YDL183C	transport			1.069	0.438	0.41
P36066	Protein MRG3-like [OS=Saccharomyces cerevisiae S288c]	853725	YKL133C; YKL133C	metabolic process	membrane;mitochondrion	protein binding	1.054	0.433	0.41
P25573	Mitochondrial inner membrane i-AAA protease supercomplex subunit MGR1 [OS=Saccharomyces cerevisiae S288c]	850313	MGR1; YCL044C	metabolic process	membrane;mitochondrion	protein binding	1.031	0.425	0.41
P35180	mitochondrial import receptor subunit Tom20 [OS=Saccharomyces cerevisiae S288c]	852973	TOM20; YGR082W	cell organization and biogenesis;transport	membrane;mitochondrion	protein binding;transporter activity	1.031	0.425	0.41
Q02608	37S ribosomal protein S16, mitochondrial [OS=Saccharomyces cerevisiae S288c]	856094	MRPS16; YPL013C	cell organization and biogenesis;metabolic process	mitochondrion;ribosome	structural molecule activity	0.931	0.389	0.42
P46998	mitochondrial membrane protein FMP33 [OS=Saccharomyces cerevisiae S288c]	853279	FMP33; YJL161W		membrane;mitochondrion		0.778	0.334	0.43
P22354	54S ribosomal protein L20, mitochondrial [OS=Saccharomyces cerevisiae S288c]	853960	MRPL20; YKR085C	cell organization and biogenesis;metabolic process	mitochondrion;ribosome	structural molecule activity	2.162	0.931	0.43
Q12305	Thiosulfate sulfurtransferase RDL1, mitochondrial [OS=Saccharomyces cerevisiae S288c]	854459	RDL1; YOR285W		endoplasmic reticulum;membrane;mitochondrion	catalytic activity	0.668	0.292	0.44
P14063	54S ribosomal protein L31, mitochondrial [OS=Saccharomyces cerevisiae S288c]	853720	MRPL31; YKL138C	cell organization and biogenesis;metabolic process	mitochondrion;ribosome	structural molecule activity	0.668	0.292	0.44
P40416	Iron-sulfur clusters transporter ATM1, mitochondrial [OS=Saccharomyces cerevisiae S288c]	855347	ATM1; YMR301C	cellular homeostasis;metabolic process;transport	membrane;mitochondrion	catalytic activity;nucleotide binding;transporter activity	0.645	0.283	0.44
P42844	Mitochondrial protein import protein ZIM17 [OS=Saccharomyces cerevisiae	855406	ZIM17; YNL310	cell organization and biogenesis;metabolic	membrane;mitochondrion;organelle lumen	metal ion binding;protein	0.585	0.259	0.44

	S288c]		C	process;regulation of biological process;response to stimulus;transport		binding			
P36528	54S ribosomal protein L17, mitochondrial [OS=Saccharomyces cerevisiae S288c]	855469	MRPL17; YNL252C	cell organization and biogenesis;metabolic process	mitochondrion;ribosome	structural molecule activity	0.52	0.233	0.45
Q12487	54S ribosomal protein L23, mitochondrial [OS=Saccharomyces cerevisiae S288c]	854321	MRPL23; YOR150W	cell organization and biogenesis;metabolic process	mitochondrion;ribosome;vacuole	RNA binding;structural molecule activity	0.52	0.233	0.45
P34224	Uncharacterized protein YBL059W [OS=Saccharomyces cerevisiae S288c]	852221	YBL059W; YBL059W		membrane;mitochondrion		0.468	0.212	0.45
P42900	Sigma-like sequence protein 1, mitochondrial [OS=Saccharomyces cerevisiae S288c]	850830	SLS1; YLR139C	cell organization and biogenesis;metabolic process	membrane;mitochondrion	protein binding	0.425	0.194	0.46
P32839	Mitochondrial chaperone BCS1 [OS=Saccharomyces cerevisiae S288c]	851981	BCS1; YDR375C	cell organization and biogenesis;metabolic process;response to stimulus;transport	cytosol;membrane;mitochondrion	catalytic activity;nucleotide binding;transporter activity	0.425	0.194	0.46
Q08970	Mitochondrial metal transporter 2 [OS=Saccharomyces cerevisiae S288c]	855877	MMT2; YPL224C	cellular homeostasis;regulation of biological process;response to stimulus;transport	membrane;mitochondrion	transporter activity	0.407	0.186	0.46
P32266	Dynamin-like GTPase MGM1, mitochondrial [OS=Saccharomyces cerevisiae S288c]	854386	MGM1; YOR211C	cell organization and biogenesis	membrane;mitochondrion	catalytic activity;nucleotide binding;protein binding	0.389	0.179	0.46
P03879	Intron-encoded RNA maturase bI4 [OS=Saccharomyces cerevisiae S288c]	854582	BI4; Q0120	metabolic process	membrane;mitochondrion	catalytic activity;RNA binding	0.389	0.179	0.46
Q05779	Ubiquinone biosynthesis protein COQ9, mitochondrial [OS=Saccharomyces cerevisiae S288c]	850898	COQ9; YLR201C	metabolic process	membrane;mitochondrion		0.359	0.166	0.46
P36523	54S ribosomal protein L15, mitochondrial [OS=Saccharomyces cerevisiae S288c]	851022	MRPL15; YLR312W-A	cell organization and biogenesis;metabolic process	mitochondrion;ribosome	catalytic activity;RNA binding;structural molecule activity	0.334	0.155	0.46
P81450	ATP synthase subunit J, mitochondrial [OS=Saccharomyces cerevisiae S288c]	854893	ATP18; YML081C-A	cell organization and biogenesis;metabolic process;transport	membrane;mitochondrion	catalytic activity;transporter activity	4.623	2.162	0.47
P25270	rRNA methyltransferase 1, mitochondrial [OS=Saccharomyces cerevisiae S288c]	854376	MRM1; YOR201C	metabolic process	mitochondrion	catalytic activity;RNA binding	0.274	0.129	0.47
P38122	3-methyl-2-oxobutanoate hydroxymethyltransferase [OS=Saccharomyces cerevisiae S288c]	852474	ECM31; YBR176W	metabolic process	mitochondrion	catalytic activity	0.259	0.122	0.47
Q04689	Altered inheritance of mitochondria protein 32 [OS=Saccharomyces cerevisiae S288c]	854955	AIM32; YML050W				0.233	0.11	0.47
P43122	tRNA N6-adenosine threonylcarbamoyltransferase, mitochondrial [OS=Saccharomyces cerevisiae S288c]	851454	QRI7; YDL104C	cell organization and biogenesis;metabolic process	mitochondrion	catalytic activity;metal ion binding	0.212	0.101	0.48
P38909	cytochrome c mitochondrial import factor CYC2 [OS=Saccharomyces cerevisiae S288c]	854202	CYC2; YOR037W	cell organization and biogenesis;metabolic process	membrane;mitochondrion	catalytic activity	0.194	0.093	0.48
P25626	54S ribosomal protein IMG1, mitochondrial [OS=Saccharomyces cerevisiae S288c]	850413	IMG1; YCR046C	cell organization and biogenesis;metabolic process	mitochondrion;ribosome	structural molecule activity	3.924	1.894	0.48
Q08818	Meiotic sister-chromatid recombination protein 6, mitochondrial [OS=Saccharomyces cerevisiae S288c]	854536	MSC6; YOR354C	metabolic process	mitochondrion;organelle lumen	RNA binding	1.047	0.506	0.48
Q12204	Probable phospholipase YOR022C, mitochondrial [OS=Saccharomyces cerevisiae S288c]	854187	YOR022C; YOR022C	metabolic process	mitochondrion	catalytic activity;metal ion binding	0.11	0.054	0.49
P38771	Ribosome-recycling factor, mitochondrial [OS=Saccharomyces cerevisiae S288c]	856433	RRF1; YHR038W	cell organization and biogenesis;metabolic process	mitochondrion	RNA binding	1.955	0.968	0.50
P38969	Pentamidine resistance factor, mitochondrial [OS=Saccharomyces cerevisiae S288c]	854440	PNT1; YOR266W	cell organization and biogenesis;response to stimulus	membrane;mitochondrion		0.711	0.359	0.50
Q06405	ATP synthase subunit f, mitochondrial [OS=Saccharomyces cerevisiae S288c]	851983	ATP17; YDR377W	metabolic process;transport	membrane;mitochondrion	catalytic activity;transporter activity	2.981	1.512	0.51
Q08023	Protein FMP25, mitochondrial [OS=Saccharomyces cerevisiae S288c]	850766	FMP25; YLR077W	cell organization and biogenesis	membrane;mitochondrion		1.412	0.719	0.51
P25372	Thioredoxin-3, mitochondrial [OS=Saccharomyces cerevisiae S288c]	850444	TRX3; YCR083W	cellular homeostasis;metabolic process;regulation of biological process;response to stimulus;transport	cytoplasm;mitochondrion	catalytic activity	2.511	1.31	0.52
P12686	37S ribosomal protein MRP13, mitochondrial [OS=Saccharomyces cerevisiae S288c]	852975	MRP13; YGR084C	cell organization and biogenesis;metabolic process	mitochondrion;ribosome	structural molecule activity	0.778	0.413	0.53

P36046	Mitochondrial intermembrane space import and assembly protein 40 [OS=Saccharomyces cerevisiae S288c]	853639	MIA40; YKL195W	cellular component movement;metabolic process;response to stimulus;transport	membrane;mitochondrion	catalytic activity;protein binding;transporter activity	2.914	1.555	0.53
P53266	Cytochrome oxidase assembly protein SHY1 [OS=Saccharomyces cerevisiae S288c]	853009	SHY1; YGR112W	cell organization and biogenesis	membrane;mitochondrion	protein binding	1.783	0.978	0.55
P39006	Phosphatidylserine decarboxylase proenzyme 1, mitochondrial [OS=Saccharomyces cerevisiae S288c]	855552	PSD1; YNL169C	metabolic process;regulation of biological process	membrane;mitochondrion	catalytic activity	0.487	0.269	0.55
P37267	Assembly factor cbp4 [OS=Saccharomyces cerevisiae S288c]		CBP4	cell organization and biogenesis	membrane;mitochondrion		3.329	1.848	0.56
P17558	37S ribosomal protein PET123, mitochondrial [OS=Saccharomyces cerevisiae S288c]	854329	PET123; YOR158W	cell organization and biogenesis;metabolic process	mitochondrion;ribosome	structural molecule activity	2.594	1.448	0.56
P37299	cytochrome b-c1 complex subunit 10 [OS=Saccharomyces cerevisiae S288c]	856390	QCR10; YHR001W-A	metabolic process;transport	membrane;mitochondrion	catalytic activity;transporter activity	5.31	2.981	0.56
P19955	37S ribosomal protein YMR-31, mitochondrial [OS=Saccharomyces cerevisiae S288c]	850610	YMR31; YFR049W	cell organization and biogenesis;metabolic process	mitochondrion;ribosome	catalytic activity;protein binding;structural molecule activity	15.681	9	0.57
P53732	37S ribosomal protein S12, mitochondrial [OS=Saccharomyces cerevisiae S288c]	855772	MRPS12; YNR036C	cell organization and biogenesis;metabolic process	mitochondrion;ribosome	structural molecule activity	1.154	0.668	0.58
Q04599	54S ribosomal protein L1, mitochondrial [OS=Saccharomyces cerevisiae S288c]	851694	MRPL1; YDR116C	cell organization and biogenesis;metabolic process	mitochondrion;ribosome	RNA binding;structural molecule activity	1.069	0.624	0.58
P53724	54S ribosomal protein L50, mitochondrial [OS=Saccharomyces cerevisiae S288c]	855756	MRPL50; YNR022C	cell organization and biogenesis;metabolic process	mitochondrion;ribosome	structural molecule activity	0.995	0.585	0.59
Q3E824	Uncharacterized protein YOR020W-A [OS=Saccharomyces cerevisiae S288c]	1466480	YOR020W-A; YOR020W-A	metabolic process;transport	membrane;mitochondrion		3.642	2.162	0.59
P17695	Glutaredoxin-2, mitochondrial [OS=Saccharomyces cerevisiae S288c]	852124	GRX2; YDR513W	cellular homeostasis;metabolic process;regulation of biological process;response to stimulus;transport	cytoplasm;cytosol;mitochondrion;nucleus	antioxidant activity;catalytic activity	0.874	0.52	0.59
P40086	Cytochrome c oxidase assembly protein cox15 [OS=Saccharomyces cerevisiae S288c]	856884	COX15; YER141W	cell organization and biogenesis;metabolic process;transport	membrane;mitochondrion	catalytic activity;transporter activity	2.594	1.555	0.60
Q03020	iron sulfur cluster assembly protein 1, mitochondrial [OS=Saccharomyces cerevisiae S288c]	855968	ISU1; YPL135W	cell organization and biogenesis;cellular homeostasis;metabolic process	cytoplasm;mitochondrion;organelle lumen	catalytic activity;metal ion binding;protein binding	0.778	0.468	0.60
Q03799	37S ribosomal protein S8, mitochondrial [OS=Saccharomyces cerevisiae S288c]	855190	MRPS8; YMR158W	cell organization and biogenesis;metabolic process	mitochondrion;ribosome	structural molecule activity	0.701	0.425	0.61
Q12375	mitochondrial ornithine transporter 1 [OS=Saccharomyces cerevisiae S288c]	854297	ORT1; YOR130C	metabolic process;transport	membrane;mitochondrion	structural molecule activity;transporter activity	0.54	0.334	0.62
P14693	sorting assembly machinery 35 kDa subunit [OS=Saccharomyces cerevisiae S288c]	856483	SAM35; YHR083W	cell organization and biogenesis;transport	membrane;mitochondrion	protein binding	0.54	0.334	0.62
P00431	cytochrome c peroxidase, mitochondrial [OS=Saccharomyces cerevisiae S288c]	853940	CCP1; YKR066C	metabolic process;response to stimulus	mitochondrion;organelle lumen	antioxidant activity;catalytic activity;metal ion binding;protein binding	1.649	1.031	0.63
P49954	probable hydrolase NIT3 [OS=Saccharomyces cerevisiae S288c]	851065	NIT3; YLR351C	metabolic process	cytoplasm;mitochondrion	catalytic activity	0.438	0.274	0.63
P32792	UPF0744 protein YSC83 [OS=Saccharomyces cerevisiae S288c]	856410	YSC83; YHR017W		membrane;mitochondrion		0.369	0.233	0.63
P37293	N-terminal acetyltransferase 2 [OS=Saccharomyces cerevisiae S288c]	853050	NAT2; YGR147C	metabolic process	cytoplasm;mitochondrion	catalytic activity	1.154	0.73	0.63
Q08686	Thiosulfate sulfurtransferase TUM1 [OS=Saccharomyces cerevisiae S288c]	854425	TUM1; YOR251C	metabolic process	cytoplasm;mitochondrion	catalytic activity;protein binding	0.334	0.212	0.63
P40015	inositol phosphosphingolipids phospholipase C [OS=Saccharomyces cerevisiae S288c]	856739	ISC1; YER019W	metabolic process;response to stimulus	endoplasmic reticulum;membrane;mitochondrion	catalytic activity;metal ion binding	0.334	0.212	0.63
Q02883	N-acyl-phosphatidylethanolamine-hydrolyzing phospholipase D, mitochondrial [OS=Saccharomyces cerevisiae S288c]	856001	FMP30; YPL103C	metabolic process	membrane;mitochondrion	catalytic activity;metal ion binding	0.304	0.194	0.64
Q01532-1	Cysteine proteinase 1, mitochondrial [OS=Saccharomyces cerevisiae S288c]		MCY1	metabolic process;regulation of biological process;response to stimulus	cytoplasm;mitochondrion	catalytic activity;DNA binding;RNA binding	0.28	0.179	0.64
Q03557	Glutamyl-tRNA(Gln) amidotransferase subunit A,	855338	HER2; YMR29	cell organization and biogenesis;metabolic process	mitochondrion	catalytic activity;nucleotide	0.259	0.166	0.64

	mitochondrial [OS=Saccharomyces cerevisiae S288c]		3C			binding			
P07236	Threonine--tRNA ligase, mitochondrial [OS=Saccharomyces cerevisiae S288c]	853640	MST1; YKL194C	metabolic process	cytoplasm;mitochondrion;organelle lumen	catalytic activity;nucleotide binding;RNA binding	0.233	0.15	0.64
P40159	Uncharacterized protein YNL208W [OS=Saccharomyces cerevisiae S288c]	855513	YNL208W; YNL208W		membrane;mitochondrion;ribosome		1.783	1.154	0.65
P40008	Protein FMP52, mitochondrial [OS=Saccharomyces cerevisiae S288c]	856721	FMP52; YER004W		endoplasmic reticulum;membrane;mitochondrion	catalytic activity	2.652	1.738	0.66
Q03430	37S ribosomal protein RSM28, mitochondrial [OS=Saccharomyces cerevisiae S288c]	852105	RSM28; YDR494W	cell organization and biogenesis;metabolic process	mitochondrion;ribosome	structural molecule activity	3.062	2.008	0.66
Q06668	Methyltransferase OMS1, mitochondrial [OS=Saccharomyces cerevisiae S288c]	851911	OMS1; YDR316W	metabolic process	membrane;mitochondrion	catalytic activity	0.585	0.389	0.66
P23369	54S ribosomal protein L25, mitochondrial [OS=Saccharomyces cerevisiae S288c]	852967	MRPL25; YGR076C	cell organization and biogenesis;metabolic process	mitochondrion;ribosome	structural molecule activity	1.31	0.874	0.67
P32860	NifU-like protein, mitochondrial [OS=Saccharomyces cerevisiae S288c]	853826	NFU1; YKL040C	cell organization and biogenesis;cellular homeostasis;metabolic process	mitochondrion;organelle lumen	metal ion binding	3.87	2.652	0.69
Q05892	MIOREX complex component 2 [OS=Saccharomyces cerevisiae S288c]	850997	YLR290C; COQ11; YLR290C	metabolic process	mitochondrion	catalytic activity	3.87	2.652	0.69
P50087	MICOS subunit MIC26 [OS=Saccharomyces cerevisiae S288c]	853150	MOS2; MIC26; YGR235C	cell organization and biogenesis	membrane;mitochondrion		0.931	0.638	0.69
P38172	MIOREX complex component 3 [OS=Saccharomyces cerevisiae S288c]	852181	YBL095W; MRX3; YBL095W		membrane;mitochondrion		0.719	0.501	0.70
P28737	Protein MSP1 [OS=Saccharomyces cerevisiae S288c]	852915	MSP1; YGR028W	cell organization and biogenesis;metabolic process;regulation of biological process;response to stimulus;transport	membrane;mitochondrion;nucleus	catalytic activity;nucleotide binding;protein binding;RNA binding	0.585	0.413	0.71
Q12251	Uncharacterized mitochondrial carrier YPR011C [OS=Saccharomyces cerevisiae S288c]	856121	YPR011C; YPR011C	metabolic process;transport	membrane;mitochondrion	structural molecule activity;transporter activity	0.551	0.389	0.71
P38152	Tricarboxylate transport protein [OS=Saccharomyces cerevisiae S288c]	852594	CTP1; YBR291C	metabolic process;transport	membrane;mitochondrion	structural molecule activity;transporter activity	0.551	0.389	0.71
P02381	Ribosomal protein VAR1, mitochondrial [OS=Saccharomyces cerevisiae S288c]	854586	VAR1; Q0140	cell organization and biogenesis;metabolic process	mitochondrion;ribosome	structural molecule activity	0.52	0.369	0.71
P38702	mitochondrial carrier protein LEU5 [OS=Saccharomyces cerevisiae S288c]	856391	LEU5; YHR002W	metabolic process;transport	membrane;mitochondrion	structural molecule activity;transporter activity	0.468	0.334	0.71
P53230	Phosphatidate cytidylyltransferase, mitochondrial [OS=Saccharomyces cerevisiae S288c]	852937	TAM41; YGR046W	metabolic process	membrane;mitochondrion;organelle lumen	catalytic activity	0.407	0.292	0.72
P54003	Protein SUR7 [OS=Saccharomyces cerevisiae S288c]	854953	SUR7; YML052W	cell differentiation;transport	membrane;mitochondrion		1.154	0.848	0.73
Q12283	Malonyl CoA-acyl carrier protein transacylase, mitochondrial [OS=Saccharomyces cerevisiae S288c]	854396	MCT1; YOR221C	metabolic process	mitochondrion	catalytic activity	0.896	0.668	0.75
P36521	54S ribosomal protein L11, mitochondrial [OS=Saccharomyces cerevisiae S288c]	851325	MRPL11; YDL202W	cell organization and biogenesis;metabolic process	mitochondrion;ribosome	structural molecule activity	0.833	0.624	0.75
P43617	Uncharacterized mitochondrial carrier YFR045W [OS=Saccharomyces cerevisiae S288c]	850606	YFR045W; YFR045W	metabolic process;transport	membrane;mitochondrion	structural molecule activity;transporter activity	0.778	0.585	0.75
P36534	54S ribosomal protein L40, mitochondrial [OS=Saccharomyces cerevisiae S288c]	855930	MRPL40; YPL173W	cell organization and biogenesis;metabolic process	mitochondrion;ribosome	protein binding;structural molecule activity	0.778	0.585	0.75
P38300	Inner membrane mitoribosome receptor MBA1, mitochondrial [OS=Saccharomyces cerevisiae S288c]	852483	MBA1; YBR185C	cell organization and biogenesis;regulation of biological process	membrane;mitochondrion		0.778	0.585	0.75
P38072	Protein SCO2, mitochondrial [OS=Saccharomyces cerevisiae S288c]	852312	SCO2; YBR024W	cell organization and biogenesis;cellular homeostasis;metabolic process;transport	membrane;mitochondrion	antioxidant activity;catalytic activity;metal ion binding	0.73	0.551	0.75

P15179	Aspartate--tRNA ligase, mitochondrial [OS=Saccharomyces cerevisiae S288c]	856000	MSD1; YPL104W	metabolic process	cytoplasm;mitochondrion;organelle lumen	catalytic activity;nucleotide binding	0.708	0.535	0.76
P00427	Cytochrome c oxidase subunit 6, mitochondrial [OS=Saccharomyces cerevisiae S288c]	856448	COX6; YHR051W	metabolic process;transport	membrane;mitochondrion	catalytic activity;metal ion binding;transporter activity	2.831	2.162	0.76
Q05931	Heat shock protein SSQ1, mitochondrial [OS=Saccharomyces cerevisiae S288c]	851084	SSQ1; YLR369W	cell organization and biogenesis;metabolic process	mitochondrion;organelle lumen	nucleotide binding;protein binding	0.884	0.679	0.77
P53326	Uncharacterized protein YGR266W [OS=Saccharomyces cerevisiae S288c]	853182	YGR266W; YGR266W		membrane;mitochondrion		0.778	0.602	0.77
P40508	Uncharacterized protein YIL077C [OS=Saccharomyces cerevisiae S288c]	854733	YIL077C; YIL077C		mitochondrion		1.154	0.896	0.78
P25038	Translation initiation factor IF-2, mitochondrial [OS=Saccharomyces cerevisiae S288c]	854135	IFM1; YOL023W	cell organization and biogenesis;metabolic process	mitochondrion;nucleus	catalytic activity;nucleotide binding;RNA binding	0.299	0.233	0.78
Q01217	Protein ARG5,6, mitochondrial [OS=Saccharomyces cerevisiae S288c]	856800	ARG5,6; YER069W	metabolic process;regulation of biological process	cytoplasm;mitochondrion;organelle lumen	catalytic activity;nucleotide binding;protein binding	0.233	0.182	0.78
Q07349	MIOREX complex component 9 [OS=Saccharomyces cerevisiae S288c]	851535	YDL027C; MRX9; YDL027C		endoplasmic reticulum;membrane;mitochondrion		2.433	1.913	0.79
P40012	protoporphyrinogen oxidase [OS=Saccharomyces cerevisiae S288c]	856733	HEM14; YER014W	metabolic process	membrane;mitochondrion	catalytic activity	1.198	0.947	0.79
P36101	tRNA threonylcarbamoyladenosine dehydratase 2 [OS=Saccharomyces cerevisiae S288c]	853841	TCD2; YKL027W; YKL027W	metabolic process	cytosol;membrane;mitochondrion	catalytic activity;nucleotide binding	0.778	0.616	0.79
Q00055	Glycerol-3-phosphate dehydrogenase [NAD(+)] 1 [OS=Saccharomyces cerevisiae S288c]	851539	GPD1; YDL022W	cell organization and biogenesis;cellular homeostasis;metabolic process;transport	cytoplasm;cytosol;mitochondrion;nucleus	catalytic activity;nucleotide binding;protein binding	0.738	0.585	0.79
Q08968	UPF0061 protein FMP40 [OS=Saccharomyces cerevisiae S288c]	855879	FMP40; YPL222W		mitochondrion	protein binding	2.162	1.721	0.80
P39525	3-oxoacyl-[acyl-carrier-protein] synthase homolog [OS=Saccharomyces cerevisiae S288c]	856790	CEM1; YER061C	metabolic process	mitochondrion	catalytic activity	1.081	0.874	0.81
P00950	Phosphoglycerate mutase 1 [OS=Saccharomyces cerevisiae S288c]	853705	GPM1; YKL152C	metabolic process;regulation of biological process	cytoplasm;cytosol;membrane;mitochondrion	catalytic activity	2.981	2.415	0.81
P39925	Mitochondrial respiratory chain complexes assembly protein AFG3 [OS=Saccharomyces cerevisiae S288c]	856737	AFG3; YER017C	cell organization and biogenesis;metabolic process;response to stimulus;transport	membrane;mitochondrion	catalytic activity;metal ion binding;nucleotide binding;protein binding	1.015	0.823	0.81
P38297	Mitofusin FZO1 [OS=Saccharomyces cerevisiae S288c]	852477	FZO1; YBR179C	cell organization and biogenesis	membrane;mitochondrion	catalytic activity;nucleotide binding;protein binding	0.304	0.248	0.82
P51998	54S ribosomal protein yml6, mitochondrial [OS=Saccharomyces cerevisiae S288c]	854983	YML6; YML025C	cell organization and biogenesis;metabolic process	mitochondrion;ribosome	RNA binding;structural molecule activity	1.404	1.154	0.82
Q04728	Arginine biosynthesis bifunctional protein ArgJ, mitochondrial [OS=Saccharomyces cerevisiae S288c]	855084	ARG7; YMR062C	metabolic process	mitochondrion;organelle lumen	catalytic activity	0.63	0.52	0.83
P39987	Heat shock protein SSC3, mitochondrial [OS=Saccharomyces cerevisiae S288c]	856682	ECM10; YEL030W	cell organization and biogenesis;metabolic process;transport	mitochondrion	nucleotide binding;protein binding	1.976	1.637	0.83
P40053	Altered inheritance of mitochondria protein 9, mitochondrial [OS=Saccharomyces cerevisiae S288c]	856813	AIM9; YER080W		mitochondrion		4.036	3.348	0.83
P32332	Mitochondrial oxaloacetate transport protein [OS=Saccharomyces cerevisiae S288c]	853739	OAC1; YKL120W	metabolic process;transport	membrane;mitochondrion	structural molecule activity;transporter activity	2.594	2.162	0.83
P53881	54S ribosomal protein L22, mitochondrial [OS=Saccharomyces cerevisiae S288c]	855544	MRPL22; YNL177C	cell organization and biogenesis;metabolic process	mitochondrion;ribosome	RNA binding;structural molecule activity	1.683	1.404	0.83
P32795	Mitochondrial inner membrane i-AAA protease supercomplex subunit YME1 [OS=Saccharomyces cerevisiae S288c]	856135	YME1; YPR024W	metabolic process;response to stimulus;transport	membrane;mitochondrion	catalytic activity;metal ion binding;nucleotide binding;protein binding	1.3	1.085	0.83
Q12029	Probable mitochondrial transport protein fsf1 [OS=Saccharomyces cerevisiae S288c]	854445	FSF1; YOR271C	transport	membrane;mitochondrion	transporter activity	2.793	2.36	0.84

P15424	ATP-dependent RNA helicase MSS116, mitochondrial [OS=Saccharomyces cerevisiae S288c]	851775	MSS116; YDR194C	metabolic process;regulation of biological process	mitochondrion;organelle lumen	catalytic activity;nucleotide binding;RNA binding	1.783	1.512	0.85
P47052	succinate dehydrogenase [ubiquinone] flavoprotein subunit 2, mitochondrial [OS=Saccharomyces cerevisiae S288c]	853405	YJL045W; YJL045W	metabolic process;transport	membrane;mitochondrion	catalytic activity;nucleotide binding	4.043	3.453	0.85
P25605	Acetolactate synthase small subunit, mitochondrial [OS=Saccharomyces cerevisiae S288c]	850348	ILV6; YCL009C	metabolic process;regulation of biological process	mitochondrion	catalytic activity;enzyme regulator activity	10.288	9	0.87
P53598	Succinyl-CoA ligase [ADP-forming] subunit alpha, mitochondrial [OS=Saccharomyces cerevisiae S288c]	854310	LSC1; YOR142W	metabolic process	cytosol;mitochondrion	catalytic activity;nucleotide binding	13.384	11.743	0.88
P33311	ATP-dependent permease MDL2, mitochondrial [OS=Saccharomyces cerevisiae S288c]	855858	MDL2; YPL270W	response to stimulus;transport	membrane;mitochondrion	catalytic activity;nucleotide binding;transporter activity	1.006	0.901	0.90
P53140	Protein RMD9, mitochondrial [OS=Saccharomyces cerevisiae S288c]	852771	RMD9; YGL107C	cell differentiation;metabolic process;regulation of biological process	membrane;mitochondrion	RNA binding	1.154	1.047	0.91
P32843	Mitochondrial escape protein 2 [OS=Saccharomyces cerevisiae S288c]	855348	YME2; YMR302C	cell organization and biogenesis;metabolic process	membrane;mitochondrion	nucleotide binding;RNA binding	3.458	3.281	0.95
P00927	Threonine dehydratase, mitochondrial [OS=Saccharomyces cerevisiae S288c]	856819	ILV1; YER086W	metabolic process	cytoplasm;mitochondrion	catalytic activity	2.257	2.257	1.00
P07143	Cytochrome c1, heme protein, mitochondrial [OS=Saccharomyces cerevisiae S288c]	854231	CYT1; YOR065W	metabolic process;transport	cytosol;membrane;mitochondrion	metal ion binding	13.251	13.251	1.00
P38071	Enoyl-[acyl-carrier protein] reductase [NADPH, B-specific], mitochondrial [OS=Saccharomyces cerevisiae S288c]	852314	ETR1; YBR026C	metabolic process	mitochondrion;organelle lumen	catalytic activity;DNA binding;metal ion binding	9	9	1.00
P43567	alanine--glyoxylate aminotransferase 1 [OS=Saccharomyces cerevisiae S288c]	850514	AGX1; YFL030W	metabolic process	cytosol;mitochondrion	catalytic activity	2.162	2.162	1.00
P07806	Valine--tRNA ligase, mitochondrial [OS=Saccharomyces cerevisiae S288c]	852986	VAS1; YGR094W	metabolic process;regulation of biological process	cytoplasm;cytosol;mitochondrion	catalytic activity;nucleotide binding	0.668	0.668	1.00
P25374	Cysteine desulfurase, mitochondrial [OS=Saccharomyces cerevisiae S288c]	850343	NFS1; YCL017C	cell organization and biogenesis;cellular homeostasis;metabolic process	mitochondrion;nucleus	catalytic activity;metal ion binding;protein binding	2.3	2.3	1.00
P32787	Mitochondrial genome maintenance protein MGM101 [OS=Saccharomyces cerevisiae S288c]	853609	MGM101; YJR144W	cell organization and biogenesis;metabolic process;response to stimulus	chromosome;mitochondrion	DNA binding	3.281	3.281	1.00
P40098	uncharacterized mitochondrial membrane protein FMP10 [OS=Saccharomyces cerevisiae S288c]	856931	FMP10; YER182W		membrane;mitochondrion		4.456	4.456	1.00
P32897	Mitochondrial import inner membrane translocase subunit tim23 [OS=Saccharomyces cerevisiae S288c]	855751	TIM23; YNR017W	transport	membrane;mitochondrion	protein binding;transporter activity	4.012	4.012	1.00
Q06892	NADH kinase POS5, mitochondrial [OS=Saccharomyces cerevisiae S288c]	855913	POS5; YPL188W	metabolic process;response to stimulus	mitochondrion;organelle lumen	catalytic activity;nucleotide binding	1.565	1.565	1.00
P32317	Protein AFG1 [OS=Saccharomyces cerevisiae S288c]	856658	AFG1; YEL052W	cell organization and biogenesis;metabolic process;response to stimulus;transport	membrane;mitochondrion	catalytic activity;nucleotide binding	1.154	1.154	1.00
P46681	D-lactate dehydrogenase [cytochrome] 2, mitochondrial [OS=Saccharomyces cerevisiae S288c]	851376	DLD2; YDL178W	metabolic process	mitochondrion;organelle lumen	catalytic activity;nucleotide binding;protein binding	1.264	1.264	1.00
P00410	Cytochrome c oxidase subunit 2 [OS=Saccharomyces cerevisiae S288c]	854622	COX2; Q0250	metabolic process;transport	membrane;mitochondrion	catalytic activity;metal ion binding;protein binding;transporter activity	3.642	3.642	1.00
P53721	Respiratory supercomplex factor 2, mitochondrial [OS=Saccharomyces cerevisiae S288c]	855752	RCF2; YNR018W	cell organization and biogenesis	membrane;mitochondrion		7.111	7.111	1.00
P40513	Mitochondrial acidic protein MAM33 [OS=Saccharomyces cerevisiae S288c]	854740	MAM33; YIL070C	metabolic process;regulation of biological process	mitochondrion;organelle lumen	translation regulator activity	2.831	2.831	1.00
P12687	54S ribosomal protein L2, mitochondrial [OS=Saccharomyces cerevisiae S288c]	855727	MRP7; YNL005C	cell organization and biogenesis;metabolic process	mitochondrion;ribosome	catalytic activity;RNA binding;structural molecule activity	1.848	1.848	1.00
P40364	Mitochondrial peculiar membrane protein 1 [OS=Saccharomyces cerevisiae S288c]	853379	MPM1; YJL066C		membrane;mitochondrion		1.512	1.512	1.00
Q06567	ABC1 family protein MCP2 [OS=Saccharomyces cerevisiae S288c]	850955	YLR253W; MCP2; YLR253W	cell organization and biogenesis	membrane;mitochondrion		0.389	0.389	1.00

P36151	Uncharacterized protein YKR070W [OS=Saccharomyces cerevisiae S288c]	853944	YKR070W; YKR070W	metabolic process	mitochondrion		1.336	1.336	1.00
P38523	GrpE protein homolog, mitochondrial [OS=Saccharomyces cerevisiae S288c]	854407	MGE1; YOR232W	metabolic process;regulation of biological process;transport	membrane;mitochondrion;organelle lumen	enzyme regulator activity;nucleotide binding;protein binding	0.833	0.833	1.00
P28817	3-hydroxyisobutyryl-CoA hydrolase, mitochondrial [OS=Saccharomyces cerevisiae S288c]	851606	EHD3; YDR036C	metabolic process	mitochondrion	catalytic activity	0.743	0.743	1.00
P36520	54S ribosomal protein L10, mitochondrial [OS=Saccharomyces cerevisiae S288c]	855436	MRPL10; YNL284C	cell organization and biogenesis;metabolic process	mitochondrion;ribosome	structural molecule activity	0.73	0.73	1.00
P28625	Protein YIM1 [OS=Saccharomyces cerevisiae S288c]	855183	YIM1; YMR152W	metabolic process;response to stimulus	cytoplasm;endoplasmic reticulum;mitochondrion	catalytic activity;metal ion binding	1.069	1.069	1.00
P32904	54S ribosomal protein L6, mitochondrial [OS=Saccharomyces cerevisiae S288c]	856552	MRPL6; YHR147C	cell organization and biogenesis;metabolic process	mitochondrion;ribosome	RNA binding;structural molecule activity	1.054	1.054	1.00
P53969	Sorting assembly machinery 50 kDa subunit [OS=Saccharomyces cerevisiae S288c]	855705	SAM50; YNL026W	cell organization and biogenesis;transport	membrane;mitochondrion	protein binding;transporter activity	0.61	0.61	1.00
P53736	Uncharacterized protein YNR040W [OS=Saccharomyces cerevisiae S288c]	855776	YNR040W; YNR040W		mitochondrion		0.73	0.73	1.00
P38797	Protein phosphatase 2C homolog 7, mitochondrial [OS=Saccharomyces cerevisiae S288c]	856475	PTC7; YHR076W	metabolic process	mitochondrion	catalytic activity;metal ion binding;protein binding	0.874	0.874	1.00
P00401	Cytochrome c oxidase subunit 1 [OS=Saccharomyces cerevisiae S288c]	854598	COX1; Q0045	metabolic process;transport	membrane;mitochondrion	catalytic activity;metal ion binding;transporter activity	1.61	1.61	1.00
P32611	54S ribosomal protein RML2, mitochondrial [OS=Saccharomyces cerevisiae S288c]	856660	RML2; YEL050C	cell organization and biogenesis;metabolic process	mitochondrion;ribosome	catalytic activity;RNA binding;structural molecule activity	0.638	0.638	1.00
P40502	Altered inheritance of mitochondria protein 19, mitochondrial [OS=Saccharomyces cerevisiae S288c]	854722	AIM19; YIL087C		membrane;mitochondrion		1.512	1.512	1.00
Q02204	54S ribosomal protein L13, mitochondrial [OS=Saccharomyces cerevisiae S288c]	853875	MRPL13; YKR006C	cell organization and biogenesis;metabolic process	mitochondrion;ribosome	structural molecule activity	0.778	0.778	1.00
P50945	MICOS complex subunit MIC27 [OS=Saccharomyces cerevisiae S288c]	855623	AIM37; MIC27; YNL100W	cell organization and biogenesis	membrane;mitochondrion		0.968	0.968	1.00
Q03327	Mitochondrial fusion and transport protein Ugo1 [OS=Saccharomyces cerevisiae S288c]	852081	UGO1; YDR470C	cell organization and biogenesis;metabolic process	membrane;mitochondrion	protein binding;structural molecule activity	0.557	0.557	1.00
Q12233	ATP synthase subunit g, mitochondrial [OS=Saccharomyces cerevisiae S288c]	856131	ATP20; YPR020W	cell organization and biogenesis;metabolic process;transport	membrane;mitochondrion	catalytic activity;transporter activity	1.371	1.371	1.00
P32387	54S ribosomal protein L41, mitochondrial [OS=Saccharomyces cerevisiae S288c]	852014	MRP20; YDR405W	cell organization and biogenesis;metabolic process	mitochondrion;ribosome	nucleotide binding;protein binding;RNA binding;structural molecule activity	0.719	0.719	1.00
P36147	Presequence translocated-associated motor subunit pam17, mitochondrial [OS=Saccharomyces cerevisiae S288c]	853939	PAM17; YKR065C	transport	membrane;mitochondrion		1.371	1.371	1.00
P27697	Atypical kinase COQ8, mitochondrial [OS=Saccharomyces cerevisiae S288c]	852758	COQ8; YGL119W	metabolic process	membrane;mitochondrion;organelle lumen	catalytic activity;nucleotide binding	0.365	0.365	1.00
Q96VH5	MICOS complex subunit Mic10 [OS=Saccharomyces cerevisiae S288c]	850300	MOS1; MIC10; YCL057C-A	cell organization and biogenesis	membrane;mitochondrion		1.683	1.683	1.00
P35996	54S ribosomal protein L38, mitochondrial [OS=Saccharomyces cerevisiae S288c]	853684	MRPL38; YKL170W	cell organization and biogenesis;metabolic process	mitochondrion;ribosome	RNA binding;structural molecule activity	1.154	1.154	1.00
P53311	Mitochondrial pyruvate carrier 3 [OS=Saccharomyces cerevisiae S288c]	853158	FMP43; MPC3; YGR243W	transport	membrane;mitochondrion	transporter activity	1.154	1.154	1.00
Q03246	37S ribosomal protein S17, mitochondrial [OS=Saccharomyces cerevisiae S288c]	855226	MRPS17; YMR188C	cell differentiation;cell organization and biogenesis;metabolic process	mitochondrion;ribosome	RNA binding;structural molecule activity	0.931	0.931	1.00
Q04172	Sensitive to high expression protein 9, mitochondrial [OS=Saccharomyces cerevisiae S288c]	852002	SHE9; YDR393W	cell organization and biogenesis	membrane;mitochondrion		0.269	0.269	1.00

P41911	Glycerol-3-phosphate dehydrogenase [NAD(+)] 2, mitochondrial [OS=Saccharomyces cerevisiae S288c]	854095	GPD2; YOL059W	metabolic process	cytoplasm;cytosol;mitochondrion	catalytic activity;nucleotide binding;protein binding	0.292	0.292	1.00
P36519	54S ribosomal protein L7, mitochondrial [OS=Saccharomyces cerevisiae S288c]	851823	MRPL7; YDR237W	cell organization and biogenesis;metabolic process	mitochondrion;ribosome	RNA binding;structural molecule activity	0.468	0.468	1.00
P33421	Succinate dehydrogenase [ubiquinone] cytochrome b subunit, mitochondrial [OS=Saccharomyces cerevisiae S288c]	853716	SDH3; YKL141W	cell organization and biogenesis;metabolic process;transport	membrane;mitochondrion	catalytic activity;metal ion binding	1.276	1.276	1.00
P33759	37S ribosomal protein S5, mitochondrial [OS=Saccharomyces cerevisiae S288c]	852553	MRPS5; YBR251W	cell organization and biogenesis;metabolic process	mitochondrion;ribosome	RNA binding;structural molecule activity	0.413	0.413	1.00
P36526	54S ribosomal protein L27, mitochondrial [OS=Saccharomyces cerevisiae S288c]	852585	MRPL27; YBR282W	cell organization and biogenesis;metabolic process	mitochondrion;ribosome	structural molecule activity	1.512	1.512	1.00
Q99297	Mitochondrial 2-oxodicarboxylate carrier 2 [OS=Saccharomyces cerevisiae S288c]	854397	ODC2; YOR222W	metabolic process;transport	membrane;mitochondrion	structural molecule activity;transporter activity	0.778	0.778	1.00
Q12298	uncharacterized ABC transporter ATP-binding protein YDR061W [OS=Saccharomyces cerevisiae S288c]	851633	YDR061W; YDR061W		mitochondrion	catalytic activity;nucleotide binding	0.266	0.266	1.00
P21306	ATP synthase subunit epsilon, mitochondrial [OS=Saccharomyces cerevisiae S288c]	855857	ATP15; YPL271W	metabolic process;transport	membrane;mitochondrion	catalytic activity;transporter activity	2.162	2.162	1.00
P18409	Mitochondrial distribution and morphology protein 10 [OS=Saccharomyces cerevisiae S288c]	851223	MDM10; YAL010C	cell organization and biogenesis;transport	membrane;mitochondrion	protein binding	0.16	0.16	1.00
P25345	Asparagine--tRNA ligase, mitochondrial [OS=Saccharomyces cerevisiae S288c]	850388	SLM5; YCR024C	metabolic process	cytoplasm;mitochondrion;organelle lumen	catalytic activity;nucleotide binding	0.172	0.172	1.00
P25348	54S ribosomal protein L32, mitochondrial [OS=Saccharomyces cerevisiae S288c]	850359	MRPL32; YCR003W	cell organization and biogenesis;metabolic process	membrane;mitochondrion;ribosome	RNA binding;structural molecule activity	0.389	0.389	1.00
P34222	Peptidyl-tRNA hydrolase 2 [OS=Saccharomyces cerevisiae S288c]	852223	PTH2; YBL057C	regulation of biological process	cytoplasm;cytosol;membrane;mitochondrion	catalytic activity;protein binding	0.425	0.425	1.00
Q06630	Mitochondrial homologous recombination protein 1 [OS=Saccharomyces cerevisiae S288c]	851890	MHR1; YDR296W	cell organization and biogenesis;metabolic process;regulation of biological process;response to stimulus	mitochondrion;nucleus	catalytic activity;DNA binding;structural molecule activity	0.359	0.359	1.00
Q06510	Lysophosphatidylcholine acyltransferase [OS=Saccharomyces cerevisiae S288c]	856262	TAZ1; YPR140W	cell organization and biogenesis;metabolic process;transport	membrane;mitochondrion	catalytic activity	0.194	0.194	1.00
P38860	GTPase MTG2, mitochondrial [OS=Saccharomyces cerevisiae S288c]	856573	MTG2; YHR168W	metabolic process;transport	membrane;mitochondrion	catalytic activity;metal ion binding;nucleotide binding;transporter activity	0.07	0.07	1.00
Q07914	mitochondrial import inner membrane translocase subunit TIM14 [OS=Saccharomyces cerevisiae S288c]	850694	PAM18; YLR008C	regulation of biological process;transport	membrane;mitochondrion	enzyme regulator activity;protein binding;transporter activity	0.778	0.778	1.00
P47150	37S ribosomal protein S7, mitochondrial [OS=Saccharomyces cerevisiae S288c]	853578	RSM7; YJR113C	cell organization and biogenesis;metabolic process	mitochondrion;ribosome	RNA binding;structural molecule activity	0.334	0.334	1.00
P32606	putative mitochondrial translation system component PET127 [OS=Saccharomyces cerevisiae S288c]	854182	PET127; YOR017W	cell organization and biogenesis;metabolic process;regulation of biological process	mitochondrion		0.099	0.099	1.00
Q05867	Uncharacterized protein YLR283W, mitochondrial [OS=Saccharomyces cerevisiae S288c]	850988	YLR283W; YLR283W		endoplasmic reticulum;membrane;mitochondrion		0.11	0.11	1.00
P38720	6-phosphogluconate dehydrogenase, decarboxylating 1 [OS=Saccharomyces cerevisiae S288c]	856589	GND1; YHR183W	metabolic process;response to stimulus	cytoplasm;mitochondrion	catalytic activity	0.15	0.15	1.00
Q07821	Iron-sulfur assembly protein 1 [OS=Saccharomyces cerevisiae S288c]	850632	ISA1; YLL027W	cell organization and biogenesis;metabolic process	mitochondrion;organelle lumen	metal ion binding;structural molecule activity	0.136	0.136	1.00
P10849	mitochondrial transcription factor 2 [OS=Saccharomyces cerevisiae S288c]	851517	MTF2; YDL044C	metabolic process	mitochondrion;organelle lumen	protein binding;RNA binding	0.08	0.08	1.00
P39112	Exoribonuclease II, mitochondrial [OS=Saccharomyces cerevisiae S288c]	855331	DSS1; YMR287C	metabolic process	mitochondrion;organelle lumen	catalytic activity;RNA binding	0.087	0.087	1.00
P09950	5-aminolevulinate synthase, mitochondrial [OS=Saccharomyces cerevisiae S288c]	851818	HEM1; YDR232W	metabolic process	mitochondrion;organelle lumen	catalytic activity	0.086	0.086	1.00
P07263-1	Histidine--tRNA ligase, mitochondrial [OS=Saccharomyces cerevisiae S288c]	856145	HTS1; YPR033C	cell organization and biogenesis;metabolic process	cytoplasm;mitochondrion	catalytic activity;nucleotide binding;RNA binding	0.064	0.064	1.00

P38816	thioredoxin reductase 2, mitochondrial [OS=Saccharomyces cerevisiae S288c]	856506	TRR2; YHR106W	cellular homeostasis;metabolic process;regulation of biological process;response to stimulus	cytoplasm;mitochondrion	antioxidant activity;catalytic activity	0.136	0.136	1.00
P10174	cytochrome c oxidase subunit 7 [OS=Saccharomyces cerevisiae S288c]	855298	COX7; YMR256C	metabolic process;transport	membrane;mitochondrion	catalytic activity;transporter activity	1.154	1.154	1.00
P32463	Acyl carrier protein, mitochondrial [OS=Saccharomyces cerevisiae S288c]	853642	ACP1; YKL192C	metabolic process;transport	mitochondrion		0.259	0.259	1.00
P04039	Cytochrome c oxidase polypeptide VIII, mitochondrial [OS=Saccharomyces cerevisiae S288c]	851111	COX8; YLR395C	metabolic process;transport	membrane;mitochondrion	catalytic activity;transporter activity	0.778	0.778	1.00
P34231	Uncharacterized protein YKL187C [OS=Saccharomyces cerevisiae S288c]	853648	FAT3; YKL187C; YKL187C	cell organization and biogenesis;transport	membrane;mitochondrion		0.093	0.093	1.00
P25578	CDP-diacylglycerol--glycerol-3-phosphate 3-phosphatidyltransferase [OS=Saccharomyces cerevisiae S288c]	850352	PGS1; YCL004W	metabolic process	mitochondrion	catalytic activity;nucleotide binding	0.083	0.083	1.00
P39103	Cytochrome c oxidase assembly protein COX14 [OS=Saccharomyces cerevisiae S288c]	854910	COX14; YML129C	cell organization and biogenesis;regulation of biological process	membrane;mitochondrion;organelle lumen	protein binding	0.585	0.585	1.00
P27882	Mitochondrial FAD-linked sulfhydryl oxidase erv1 [OS=Saccharomyces cerevisiae S288c]	852916	ERV1; YGR029W	cellular homeostasis;metabolic process;response to stimulus;transport	mitochondrion	catalytic activity;protein binding	0.212	0.212	1.00
P53320	Mitochondrial carrier protein MTM1 [OS=Saccharomyces cerevisiae S288c]	853173	MTM1; YGR257C	metabolic process;transport	membrane;mitochondrion	structural molecule activity	0.11	0.11	1.00
Q08622	Genetic interactor of prohibitins 3, mitochondrial [OS=Saccharomyces cerevisiae S288c]	854380	GEP3; YOR205C	metabolic process;transport	cytosol;membrane;mitochondrion	catalytic activity;nucleotide binding;transporter activity	0.059	0.059	1.00
P30624	Long-chain-fatty-acid--CoA ligase 1 [OS=Saccharomyces cerevisiae S288c]	854495	FAA1; YOR317W	metabolic process;transport	endoplasmic reticulum;membrane;mitochondrion	catalytic activity;nucleotide binding	5.874	6.252	1.06
Q12031	Mitochondrial 2-methylisocitrate lyase [OS=Saccharomyces cerevisiae S288c]	856114	ICL2; YPR006C	metabolic process	mitochondrion;organelle lumen	catalytic activity	3.833	4.135	1.08
P47127	Altered inheritance of mitochondria protein 24, mitochondrial [OS=Saccharomyces cerevisiae S288c]	853543	AIM24; YJR080C	cell organization and biogenesis	mitochondrion		2.857	3.175	1.11
P27929	37S ribosomal protein NAM9, mitochondrial [OS=Saccharomyces cerevisiae S288c]	855585	NAM9; YNL137C	cell organization and biogenesis;metabolic process;regulation of biological process	mitochondrion;ribosome	RNA binding;structural molecule activity	1.202	1.352	1.12
P50085	Prohibitin-2 [OS=Saccharomyces cerevisiae S288c]	853146	PHB2; YGR231C	cell organization and biogenesis;metabolic process;regulation of biological process	membrane;mitochondrion	protein binding	3.806	4.337	1.14
P09457	ATP synthase subunit 5, mitochondrial [OS=Saccharomyces cerevisiae S288c]	851892	ATP5; YDR298C	metabolic process;transport	membrane;mitochondrion	catalytic activity;transporter activity	10.365	11.915	1.15
P38988	Mitochondrial GTP/GDP carrier protein 1 [OS=Saccharomyces cerevisiae S288c]	851329	GGC1; YDL198C	cell organization and biogenesis;cellular homeostasis;metabolic process;transport	membrane;mitochondrion	structural molecule activity;transporter activity	3.16	3.642	1.15
P40961	prohibitin-1 [OS=Saccharomyces cerevisiae S288c]	853033	PHB1; YGR132C	cell organization and biogenesis;metabolic process;regulation of biological process	membrane;mitochondrion	protein binding	5.158	5.952	1.15
P53206	putative cysteine synthase [OS=Saccharomyces cerevisiae S288c]	852895	YGR012W; YGR012W	metabolic process	mitochondrion	catalytic activity	1.346	1.555	1.16
P53292	37S ribosomal protein S35, mitochondrial [OS=Saccharomyces cerevisiae S288c]	853075	MRPS35; YGR165W	cell organization and biogenesis;metabolic process	mitochondrion;ribosome	structural molecule activity	1.512	1.754	1.16
Q12482	Mitochondrial aspartate-glutamate transporter AGC1 [OS=Saccharomyces cerevisiae S288c]	856132	AGC1; YPR021C	metabolic process;transport	membrane;mitochondrion	structural molecule activity;transporter activity	0.348	0.407	1.17
P32898	Mitochondrial presequence protease [OS=Saccharomyces cerevisiae S288c]	852041	CYM1; YDR430C	metabolic process	mitochondrion	catalytic activity;metal ion binding	0.812	0.951	1.17
P53170	[Pyruvate dehydrogenase (acetyl-transferring)] kinase 2, mitochondrial [OS=Saccharomyces cerevisiae S288c]	852821	PKP2; YGL059W	metabolic process;regulation of biological process	mitochondrion;organelle lumen	catalytic activity;nucleotide binding;protein binding	0.778	0.931	1.20
P00560	Phosphoglycerate kinase [OS=Saccharomyces cerevisiae S288c]	850370	PGK1; YCR012W	metabolic process	cytoplasm;membrane;mitochondrion	catalytic activity;nucleotide binding	4.843	5.813	1.20
Q02981	ABC1 family protein YPL109C, mitochondrial [OS=Saccharomyces cerevisiae S288c]	855995	YPL109C; YPL109C		mitochondrion		0.413	0.496	1.20
P35191	DnaJ homolog 1, mitochondrial [OS=Saccharomyces cerevisiae S288c]	850530	MDJ1; YFL016C	cell organization and biogenesis;metabolic process;regulation of biological process;response to stimulus	mitochondrion;organelle lumen	enzyme regulator activity;metal ion binding;nucleotide binding;protein	2.765	3.329	1.20

						binding			
P33310	ATP-dependent permease MDL1, mitochondrial [OS=Saccharomyces cerevisiae S288c]	850885	MDL1; YLR188W	transport	membrane;mitochondrion	catalytic activity;nucleotide binding;transporter activity	0.438	0.528	1.21
Q06493	LETM1 domain-containing protein YLH47, mitochondrial [OS=Saccharomyces cerevisiae S288c]	856243	YLH47; YPR125W	cell organization and biogenesis;transport	membrane;mitochondrion		0.61	0.743	1.22
P10662	37S ribosomal protein MRP1, mitochondrial [OS=Saccharomyces cerevisiae S288c]	851948	MRP1; YDR347W	cell organization and biogenesis;metabolic process;response to stimulus	mitochondrion;ribosome	antioxidant activity;catalytic activity;metal ion binding;structural molecule activity	1.239	1.512	1.22
P11325	Leucine--tRNA ligase, mitochondrial [OS=Saccharomyces cerevisiae S288c]	851098	NAM2; YLR382C	cell organization and biogenesis;metabolic process;regulation of biological process	cytosol;mitochondrion;organelle lumen	catalytic activity;nucleotide binding;RNA binding	0.208	0.254	1.22
P08466	mitochondrial nuclease [OS=Saccharomyces cerevisiae S288c]	853222	NUC1; YJL208C	cell death;metabolic process	membrane;mitochondrion;nucleus	catalytic activity;metal ion binding	1.336	1.637	1.23
Q07651	SUR7 family protein FMP45 [OS=Saccharomyces cerevisiae S288c]	851304	FMP45; YDL222C	cell differentiation;cell organization and biogenesis	membrane;mitochondrion		6.017	7.377	1.23
P53982	isocitrate dehydrogenase [NADP] [OS=Saccharomyces cerevisiae S288c]	855723	IDP3; YNL009W	metabolic process	cytoplasm;mitochondrion	catalytic activity;metal ion binding;nucleotide binding	1.943	2.398	1.23
Q04458	Fatty aldehyde dehydrogenase HFD1 [OS=Saccharomyces cerevisiae S288c]	855137	HFD1; YMR110C	metabolic process	endoplasmic reticulum;endosome;membrane;mitochondrion	catalytic activity	3.642	4.505	1.24
Q06143	mitochondrial dicarboxylate transporter [OS=Saccharomyces cerevisiae S288c]	851063	DIC1; YLR348C	metabolic process;transport	membrane;mitochondrion	structural molecule activity;transporter activity	0.931	1.154	1.24
P32331	Carrier protein YMC1, mitochondrial [OS=Saccharomyces cerevisiae S288c]	856171	YMC1; YPR058W	metabolic process;transport	membrane;mitochondrion;vacuole	structural molecule activity;transporter activity	1.254	1.581	1.26
P40185	Protein mmf1, mitochondrial [OS=Saccharomyces cerevisiae S288c]	854760	MMF1; YIL051C	cell organization and biogenesis;metabolic process	mitochondrion;organelle lumen		6.943	9	1.30
P36112	MICOS complex subunit Mic60 [OS=Saccharomyces cerevisiae S288c]	853886	FCJ1; MIC60; YKR016W	cell organization and biogenesis;transport	membrane;mitochondrion		15.379	19.962	1.30
P21375	Fumarate reductase 2 [OS=Saccharomyces cerevisiae S288c]	853510	OSM1; YJR051W	metabolic process	endoplasmic reticulum;mitochondrion	catalytic activity;nucleotide binding	0.346	0.45	1.30
P04710	ADP,ATP carrier protein 1 [OS=Saccharomyces cerevisiae S288c]	855078	AAC1; YMR056C	metabolic process;transport	cytosol;membrane;mitochondrion	structural molecule activity;transporter activity	10.288	13.384	1.30
P38825	Protein TOM71 [OS=Saccharomyces cerevisiae S288c]	856517	TOM71; YHR117W	transport	membrane;mitochondrion	protein binding;transporter activity	0.701	0.914	1.30
Q01852	Mitochondrial import inner membrane translocase subunit TIM44 [OS=Saccharomyces cerevisiae S288c]	854790	TIM44; YIL022W	transport	membrane;mitochondrion	nucleotide binding;protein binding	4.125	5.404	1.31
P36525	54S ribosomal protein L24, mitochondrial [OS=Saccharomyces cerevisiae S288c]	855231	MRPL24; YMR193W	cell organization and biogenesis;metabolic process	mitochondrion;ribosome	RNA binding;structural molecule activity	1.154	1.512	1.31
P00128	Cytochrome b-c1 complex subunit 7 [OS=Saccharomyces cerevisiae S288c]	852142	QCR7; YDR529C	cell organization and biogenesis;metabolic process;transport	membrane;mitochondrion	catalytic activity;transporter activity	11.915	15.681	1.32
Q02776	Mitochondrial import inner membrane translocase subunit TIM50 [OS=Saccharomyces cerevisiae S288c]	856042	TIM50; YPL063W	cell organization and biogenesis;metabolic process;transport	membrane;mitochondrion	catalytic activity;protein binding;transporter activity	7.483	9.857	1.32
P21801	Succinate dehydrogenase [ubiquinone] iron-sulfur subunit, mitochondrial [OS=Saccharomyces cerevisiae S288c]	850685	SDH2; YLL041C	metabolic process;transport	membrane;mitochondrion	catalytic activity;metal ion binding	18.684	24.809	1.33
P53220	Mitochondrial import inner membrane translocase subunit TIM21 [OS=Saccharomyces cerevisiae S288c]	852921	TIM21; YGR033C	cell organization and biogenesis;transport	membrane;mitochondrion	protein binding	0.719	0.968	1.35
Q01574	acetyl-coenzyme A synthetase 1 [OS=Saccharomyces cerevisiae S288c]	851245	ACS1; YAL054C	cell organization and biogenesis;metabolic process	cytoplasm;cytosol;endoplasmic reticulum;mitochondrion;nucleus	catalytic activity;nucleotide binding	3.281	4.456	1.36
P00445	Superoxide dismutase [Cu-Zn] [OS=Saccharomyces cerevisiae S288c]	853568	SOD1; YJR104C	cell organization and biogenesis;cellular homeostasis;metabolic process;regulation of biological process;response to stimulus	cytoplasm;cytosol;mitochondrion;nucleus	antioxidant activity;catalytic activity;metal ion binding;protein binding	1.848	2.511	1.36
P43635	Citrate synthase 3, mitochondrial [OS=Saccharomyces cerevisiae S288c]	856107	CIT3; YPR001W	metabolic process	mitochondrion	catalytic activity	3.642	4.995	1.37
P38169	Kynurenine 3-monooxygenase [OS=Saccharomyces cerevisiae S288c]	852179	BNA4; YBL098	metabolic process	membrane;mitochondrion	catalytic activity;nucleotide	0.269	0.374	1.39

			W			binding			
P48015	Aminomethyltransferase, mitochondrial [OS=Saccharomyces cerevisiae S288c]	851582	GCV1; YDR019C	metabolic process	mitochondrion	catalytic activity;protein binding	8.047	11.217	1.39
P22136	ATPase expression protein 2, mitochondrial [OS=Saccharomyces cerevisiae S288c]	855325	AEP2; YMR282C	metabolic process;regulation of biological process	mitochondrion	RNA binding	0.359	0.506	1.41
P42940	Probable electron transfer flavoprotein subunit beta [OS=Saccharomyces cerevisiae S288c]	853121	CIR1; YGR207C	metabolic process;transport	mitochondrion;organelle lumen		3.87	5.494	1.42
P19516	Cytochrome c oxidase assembly protein COX11, mitochondrial [OS=Saccharomyces cerevisiae S288c]	855971	COX11; YPL132W	cell organization and biogenesis;metabolic process	membrane;mitochondrion;ribosome	metal ion binding	0.438	0.624	1.42
P32902	37S ribosomal protein MRP4, mitochondrial [OS=Saccharomyces cerevisiae S288c]	856384	MRP4; YHL004W	cell organization and biogenesis;metabolic process	mitochondrion;ribosome	structural molecule activity	0.957	1.371	1.43
P53889	uncharacterized mitochondrial hydrolase FMP41 [OS=Saccharomyces cerevisiae S288c]	855553	FMP41; YNL168C	metabolic process	mitochondrion	catalytic activity;metal ion binding	0.501	0.719	1.44
P31334	54S ribosomal protein L9, mitochondrial [OS=Saccharomyces cerevisiae S288c]	853135	MRPL9; YGR220C	cell organization and biogenesis;metabolic process	mitochondrion;ribosome	structural molecule activity	0.501	0.719	1.44
P40215	External NADH-ubiquinone oxidoreductase 1, mitochondrial [OS=Saccharomyces cerevisiae S288c]	855176	NDE1; YMR145C	metabolic process	mitochondrion	catalytic activity;nucleotide binding	5.078	7.297	1.44
P30902	ATP synthase subunit d, mitochondrial [OS=Saccharomyces cerevisiae S288c]	853853	ATP7; YKL016C	metabolic process;transport	membrane;mitochondrion	catalytic activity;transporter activity	6.197	9	1.45
P00175	Cytochrome b2, mitochondrial [OS=Saccharomyces cerevisiae S288c]	854950	CYB2; YML054C	metabolic process;transport	cytosol;membrane;mitochondrion;nucleus	catalytic activity;metal ion binding;nucleotide binding	0.679	0.995	1.47
Q12428	Probable 2-methylcitrate dehydratase [OS=Saccharomyces cerevisiae S288c]	856108	PDH1; YPR002W	metabolic process	cytoplasm;membrane;mitochondrion	catalytic activity	12.335	18.11	1.47
P32335	Protein MSS51, mitochondrial [OS=Saccharomyces cerevisiae S288c]	850900	MSS51; YLR203C	metabolic process;regulation of biological process	membrane;mitochondrion	protein binding;translation regulator activity	1.154	1.712	1.48
P23641	mitochondrial phosphate carrier protein [OS=Saccharomyces cerevisiae S288c]	853540	MIR1; YJR077C	metabolic process;transport	membrane;mitochondrion	protein binding;structural molecule activity;transporter activity	20.544	30.623	1.49
P25719	Peptidyl-prolyl cis-trans isomerase C, mitochondrial [OS=Saccharomyces cerevisiae S288c]	854897	CPR3; YML078W	cell death;metabolic process	mitochondrion;organelle lumen	catalytic activity	2.415	3.642	1.51
P00447	Superoxide dismutase [Mn], mitochondrial [OS=Saccharomyces cerevisiae S288c]	856399	SOD2; YHR008C	metabolic process;response to stimulus	mitochondrion;organelle lumen	antioxidant activity;catalytic activity;metal ion binding	3.924	6.017	1.53
P04037	cytochrome c oxidase subunit 4, mitochondrial [OS=Saccharomyces cerevisiae S288c]	852688	COX4; YGL187C	cell organization and biogenesis;metabolic process;transport	membrane;mitochondrion	catalytic activity;metal ion binding;transporter activity	27.48	42.288	1.54
Q07500	External NADH-ubiquinone oxidoreductase 2, mitochondrial [OS=Saccharomyces cerevisiae S288c]	851474	NDE2; YDL085W	metabolic process	mitochondrion	catalytic activity;nucleotide binding	6.356	9.798	1.54
P36531	54S ribosomal protein L36, mitochondrial [OS=Saccharomyces cerevisiae S288c]	852419	MRPL36; YBR122C	cell organization and biogenesis;metabolic process	mitochondrion;ribosome	structural molecule activity	1.154	1.783	1.55
P08417	fumarate hydratase, mitochondrial [OS=Saccharomyces cerevisiae S288c]	855866	FUM1; YPL262W	cell organization and biogenesis;metabolic process	cytoplasm;cytosol;mitochondrion;organelle lumen	catalytic activity	8.237	12.738	1.55
Q06678	54S ribosomal protein L35, mitochondrial [OS=Saccharomyces cerevisiae S288c]	851921	MRPL35; YDR322W	cell organization and biogenesis;metabolic process	mitochondrion;ribosome	structural molecule activity	1.219	1.894	1.55
Q12466	tricalbin-1 [OS=Saccharomyces cerevisiae S288c]	854253	TCB1; YOR086C	cell organization and biogenesis;regulation of biological process	endoplasmic reticulum;membrane;mitochondrion	metal ion binding;protein binding	0.52	0.81	1.56
P40051	Intermediate cleaving peptidase 55 [OS=Saccharomyces cerevisiae S288c]	856811	ICP55; YER078C	metabolic process;regulation of biological process	membrane;mitochondrion;nucleus	catalytic activity;metal ion binding	0.16	0.25	1.56
P43616	Cys-Gly metallodipeptidase dug1 [OS=Saccharomyces cerevisiae S288c]	850605	DUG1; YFR044C	metabolic process	cytoplasm;mitochondrion;ribosome	catalytic activity;metal ion binding	0.848	1.326	1.56
P29704	squalene synthase [OS=Saccharomyces cerevisiae S288c]	856597	ERG9; YHR190W	metabolic process	endoplasmic reticulum;membrane;mitochondrion	catalytic activity	0.222	0.35	1.58
Q06485	Autophagy-related protein 33 [OS=Saccharomyces cerevisiae S288c]	851070	ATG33; YLR356W	cell communication;metabolic process;response to stimulus	membrane;mitochondrion		1.371	2.162	1.58
P50088	Stationary phase gene 1 protein [OS=Saccharomyces cerevisiae S288c]	853151	SPG1; YGR236C		endoplasmic reticulum;membrane;mitochondrion		157.489	250.189	1.59
P47045	mitochondrial import inner membrane translocase subunit TIM54 [OS=Saccharomyces cerevisiae	853392	TIM54; YJL054W	cell organization and biogenesis;transport	cytosol;membrane;mitochondrion	transporter activity	0.655	1.054	1.61

	S288c]								
P40047	Aldehyde dehydrogenase 5, mitochondrial [OS=Saccharomyces cerevisiae S288c]	856804	ALD5; YER073W	metabolic process	mitochondrion;organelle lumen	catalytic activity	1.955	3.146	1.61
P54783	D-arabinono-1,4-lactone oxidase [OS=Saccharomyces cerevisiae S288c]	854888	ALO1; YML086C	metabolic process;response to stimulus	membrane;mitochondrion	catalytic activity;nucleotide binding	3.299	5.31	1.61
P38884	Altered inheritance of mitochondria protein 18, mitochondrial [OS=Saccharomyces cerevisiae S288c]	856605	AIM18; YHR198C		mitochondrion	catalytic activity	0.311	0.501	1.61
P40341	Mitochondrial respiratory chain complexes assembly protein YTA12 [OS=Saccharomyces cerevisiae S288c]	855114	YTA12; YMR089C	cell organization and biogenesis;metabolic process;response to stimulus;transport	cytoplasm;membrane;mitochondrion	catalytic activity;metal ion binding;nucleotide binding;protein binding	1.184	1.913	1.62
P41735	5-demethoxyubiquinone hydroxylase, mitochondrial [OS=Saccharomyces cerevisiae S288c]	854292	CAT5; YOR125C	metabolic process	membrane;mitochondrion	catalytic activity;metal ion binding	0.334	0.54	1.62
P07246	Alcohol dehydrogenase 3, mitochondrial [OS=Saccharomyces cerevisiae S288c]	855107	ADH3; YMR083W	metabolic process	mitochondrion;organelle lumen	catalytic activity;metal ion binding	7.031	11.452	1.63
P16622	Ferrochelatase, mitochondrial [OS=Saccharomyces cerevisiae S288c]	854347	HEM15; YOR176W	metabolic process	membrane;mitochondrion	catalytic activity	4.337	7.111	1.64
P00925	Enolase 2 [OS=Saccharomyces cerevisiae S288c]	856579	ENO2; YHR174W	metabolic process;regulation of biological process	cytoplasm;cytosol;membrane;mitochondrion;vacuole	catalytic activity;metal ion binding;protein binding	1.395	2.29	1.64
P32454	Aminopeptidase 2, mitochondrial [OS=Saccharomyces cerevisiae S288c]	853699	APE2; YKL157W	metabolic process	cytoplasm;extracellular;membrane;mitochondrion;nucleus	catalytic activity;metal ion binding	1.209	1.988	1.64
Q03201	37S ribosomal protein S10, mitochondrial [OS=Saccharomyces cerevisiae S288c]	851611	RSM10; YDR041W	cell organization and biogenesis;metabolic process	mitochondrion;ribosome	structural molecule activity	0.425	0.701	1.65
P40515	Mitochondria fission 1 protein [OS=Saccharomyces cerevisiae S288c]	854745	FIS1; YIL065C	cell death;cell organization and biogenesis;regulation of biological process	membrane;mitochondrion	protein binding	0.468	0.778	1.66
P39522	Dihydroxy-acid dehydratase, mitochondrial [OS=Saccharomyces cerevisiae S288c]	853473	ILV3; YJR016C	metabolic process	mitochondrion	catalytic activity;metal ion binding	16.475	27.48	1.67
Q08822	Probable electron transfer flavoprotein-ubiquinone oxidoreductase, mitochondrial [OS=Saccharomyces cerevisiae S288c]	854538	CIR2; YOR356W	metabolic process;transport	membrane;mitochondrion	catalytic activity;metal ion binding;nucleotide binding	2.162	3.642	1.68
P40530	[Pyruvate dehydrogenase (acetyl-transferring)] kinase 1, mitochondrial [OS=Saccharomyces cerevisiae S288c]	854769	PKP1; YIL042C	metabolic process	mitochondrion;organelle lumen	catalytic activity;nucleotide binding;protein binding	0.551	0.931	1.69
Q02486	ARS-binding factor 2, mitochondrial [OS=Saccharomyces cerevisiae S288c]	855094	ABF2; YMR072W	cell organization and biogenesis;metabolic process;regulation of biological process	chromosome;mitochondrion;nucleus	DNA binding	69.17	118.378	1.71
Q12374	Nuclear control of ATPase protein 2 [OS=Saccharomyces cerevisiae S288c]	856278	NCA2; YPR155C	metabolic process	membrane;mitochondrion		0.668	1.154	1.73
P49095	Glycine dehydrogenase (Decarboxylating), mitochondrial [OS=Saccharomyces cerevisiae S288c]	855227	GCV2; YMR189W	metabolic process	cytosol;mitochondrion	catalytic activity	1.575	2.728	1.73
P07275	Delta-1-pyrroline-5-carboxylate dehydrogenase, mitochondrial [OS=Saccharomyces cerevisiae S288c]	856432	PUT2; YHR037W	metabolic process	membrane;mitochondrion;organelle lumen	catalytic activity	5.579	9.723	1.74
P39515	mitochondrial import inner membrane translocase subunit Tim17 [OS=Saccharomyces cerevisiae S288c]	853298	TIM17; YJL143W	cell organization and biogenesis;transport	membrane;mitochondrion	protein binding;transporter activity	0.778	1.371	1.76
P16451	Pyruvate dehydrogenase complex protein X component, mitochondrial [OS=Saccharomyces cerevisiae S288c]	853107	PDX1; YGR193C	metabolic process	mitochondrion;organelle lumen	catalytic activity;structural molecule activity	2.3	4.055	1.76
Q12289	mitochondrial carnitine carrier [OS=Saccharomyces cerevisiae S288c]	854267	CRC1; YOR100C	metabolic process;transport	membrane;mitochondrion	structural molecule activity;transporter activity	0.931	1.683	1.81
Q12480	Probable electron transfer flavoprotein subunit alpha, mitochondrial [OS=Saccharomyces cerevisiae S288c]	856112	AIM45; YPR004C	metabolic process;transport	mitochondrion;organelle lumen	catalytic activity;nucleotide binding	2.548	4.623	1.81
P37292	Serine hydroxymethyltransferase, mitochondrial [OS=Saccharomyces cerevisiae S288c]	852565	SHM1; YBR263W	metabolic process	mitochondrion	catalytic activity	7.697	14.199	1.84
Q04013	Citrate/oxoglutarate carrier protein [OS=Saccharomyces cerevisiae S288c]	855282	YHM2; YMR241W	cell organization and biogenesis;metabolic process;transport	membrane;mitochondrion	DNA binding;structural molecule activity;transporter activity	2.162	4.012	1.86
Q03976	37S ribosomal protein S24, mitochondrial [OS=Saccharomyces cerevisiae S288c]	851755	RSM24; YDR175C	cell organization and biogenesis;metabolic process	mitochondrion;ribosome	structural molecule activity	0.585	1.089	1.86

P05626	ATP synthase subunit 4, mitochondrial [OS=Saccharomyces cerevisiae S288c]	856027	ATP4; YPL078C	cell organization and biogenesis;metabolic process;transport	membrane;mitochondrion	catalytic activity;transporter activity	3.084	5.813	1.88
Q06089	Uncharacterized mitochondrial outer membrane protein YPR098C [OS=Saccharomyces cerevisiae S288c]	856213	YPR098C; YPR098C		membrane;mitochondrion		4.623	9	1.95
P25087	Sterol 24-C-methyltransferase [OS=Saccharomyces cerevisiae S288c]	855003	ERG6; YML008C	metabolic process	endoplasmic reticulum;membrane;mitochondrion	catalytic activity	1.424	2.775	1.95
P32473	Pyruvate dehydrogenase E1 component subunit beta, mitochondrial [OS=Saccharomyces cerevisiae S288c]	852522	PDB1; YBR221C	metabolic process	mitochondrion;organelle lumen	catalytic activity	6.848	13.384	1.95
Q00711	Succinate dehydrogenase [ubiquinone] flavoprotein subunit, mitochondrial [OS=Saccharomyces cerevisiae S288c]	853709	SDH1; YKL148C	metabolic process;transport	membrane;mitochondrion	catalytic activity;nucleotide binding;protein binding	8.454	16.535	1.96
P07213	Mitochondrial import receptor subunit TOM70 [OS=Saccharomyces cerevisiae S288c]	855602	TOM70; YNL121C	cell organization and biogenesis;transport	membrane;mitochondrion	protein binding;transporter activity	2.665	5.236	1.96
P23644	Mitochondrial import receptor subunit TOM40 [OS=Saccharomyces cerevisiae S288c]	855243	TOM40; YMR203W	transport	cytosol;membrane;mitochondrion	protein binding;transporter activity	0.896	1.783	1.99
P07342	Acetolactate synthase catalytic subunit, mitochondrial [OS=Saccharomyces cerevisiae S288c]	855135	ILV2; YMR108W	metabolic process	mitochondrion	catalytic activity;metal ion binding;nucleotide binding	4.223	8.427	2.00
Q02950	37S ribosomal protein MRP51, mitochondrial [OS=Saccharomyces cerevisiae S288c]	855985	MRP51; YPL118W	cell organization and biogenesis;metabolic process	mitochondrion;ribosome	structural molecule activity	0.668	1.346	2.01
P38910	10 kDa heat shock protein, mitochondrial [OS=Saccharomyces cerevisiae S288c]	854185	HSP10; YOR020C	cell organization and biogenesis;metabolic process;response to stimulus;transport	cytoplasm;mitochondrion;organelle lumen	metal ion binding;nucleotide binding;protein binding	3.217	6.499	2.02
P48526	Isoleucine--tRNA ligase, mitochondrial [OS=Saccharomyces cerevisiae S288c]	856067	ISM1; YPL040C	cell organization and biogenesis;metabolic process;regulation of biological process	cytoplasm;cytosol;mitochondrion;organelle lumen	catalytic activity;nucleotide binding	0.04	0.081	2.03
P21954	Isocitrate dehydrogenase [NADP], mitochondrial [OS=Saccharomyces cerevisiae S288c]	851493	IDP1; YDL066W	metabolic process	mitochondrion	catalytic activity;metal ion binding;nucleotide binding;protein binding	33.145	67.129	2.03
P32445	Single-stranded DNA-binding protein RIM1, mitochondrial [OS=Saccharomyces cerevisiae S288c]	850395	RIM1; YCR028C-A	cell organization and biogenesis;metabolic process;regulation of biological process	mitochondrion	DNA binding	1.783	3.642	2.04
Q00402	Nuclear migration protein NUM1 [OS=Saccharomyces cerevisiae S288c]	851727	NUM1; YDR150W	cell organization and biogenesis;cellular component movement;metabolic process;regulation of biological process;transport	endoplasmic reticulum;mitochondrion	catalytic activity;motor activity;protein binding	0.065	0.134	2.06
P10507	mitochondrial-processing peptidase subunit beta [OS=Saccharomyces cerevisiae S288c]	850860	MAS1; YLR163C	metabolic process;transport	mitochondrion;organelle lumen	catalytic activity;metal ion binding;protein binding	0.778	1.61	2.07
P43603	LAS seventeen-binding protein 3 [OS=Saccharomyces cerevisiae S288c]	850580	LSB3; YFR024C-A	cell organization and biogenesis;regulation of biological process	cytoplasm;mitochondrion	protein binding	0.089	0.186	2.09
Q08179	Mitochondrial distribution and morphology protein 38 [OS=Saccharomyces cerevisiae S288c]	854130	MDM38; YOL027C	cell organization and biogenesis;cellular homeostasis;regulation of biological process;transport	membrane;mitochondrion		3.732	7.913	2.12
P39965	probable proline--tRNA ligase, mitochondrial [OS=Saccharomyces cerevisiae S288c]	856820	AIM10; YER087W	metabolic process	cytoplasm;mitochondrion	catalytic activity;nucleotide binding;RNA binding	0.125	0.266	2.13
P21771	37S ribosomal protein S28, mitochondrial [OS=Saccharomyces cerevisiae S288c]	851937	MRPS28; YDR337W	cell organization and biogenesis;metabolic process	mitochondrion;ribosome	RNA binding;structural molecule activity	0.624	1.336	2.14
P28241	Isocitrate dehydrogenase [NAD] subunit 2, mitochondrial [OS=Saccharomyces cerevisiae S288c]	854303	IDH2; YOR136W	metabolic process	mitochondrion;organelle lumen	catalytic activity;metal ion binding;nucleotide binding;protein binding;RNA binding	4.179	9	2.15
P53212	Probable transcriptional regulatory protein HAH1 [OS=Saccharomyces cerevisiae S288c]	852904	YGR021W; YGR021W		mitochondrion		0.166	0.359	2.16
P47039	Probable kynurenine--oxoglutarate transaminase BNA3 [OS=Saccharomyces cerevisiae S288c]	853386	BNA3; YJL060W	metabolic process;regulation of biological process	cytoplasm;mitochondrion	catalytic activity	0.212	0.468	2.21
P09440	C-1-tetrahydrofolate synthase, mitochondrial [OS=Saccharomyces cerevisiae S288c]	852378	MIS1; YBR084W	metabolic process	cytosol;mitochondrion	catalytic activity;nucleotide binding	0.9	1.988	2.21

P49334	Mitochondrial import receptor subunit tom22 [OS=Saccharomyces cerevisiae S288c]	855592	TOM22; YNL131W	cell organization and biogenesis;transport	membrane;mitochondrion	protein binding;transporter activity	0.292	0.668	2.29
P33416	Heat shock protein 78, mitochondrial [OS=Saccharomyces cerevisiae S288c]	851845	HSP78; YDR258C	cell organization and biogenesis;metabolic process;regulation of biological process;response to stimulus	mitochondrion;organelle lumen	catalytic activity;nucleotide binding;protein binding	4.264	9.784	2.29
P36775	Lon protease homolog, mitochondrial [OS=Saccharomyces cerevisiae S288c]	852259	PIM1; YBL022C	cell organization and biogenesis;metabolic process;regulation of biological process;response to stimulus	cytoplasm;mitochondrion;organelle lumen	catalytic activity;DNA binding;nucleotide binding;RNA binding	0.685	1.59	2.32
P23301	Eukaryotic translation initiation factor 5A-1 [OS=Saccharomyces cerevisiae S288c]	856677	HYP2; YEL034W	metabolic process;regulation of biological process	cytoplasm;mitochondrion;ribosome	protein binding;RNA binding	0.334	0.778	2.33
P25349	Flavoprotein-like protein YCP4 [OS=Saccharomyces cerevisiae S288c]	850360	YCP4; YCR004C	metabolic process;regulation of biological process	cytoplasm;membrane;mitochondrion	catalytic activity;nucleotide binding;protein binding	5.813	13.678	2.35
P00830	ATP synthase subunit beta, mitochondrial [OS=Saccharomyces cerevisiae S288c]	853585	ATP2; YJR121W	metabolic process;transport	cytosol;membrane;mitochondrion	catalytic activity;nucleotide binding;protein binding;transporter activity	38.811	91.612	2.36
P52893	probable alanine aminotransferase, mitochondrial [OS=Saccharomyces cerevisiae S288c]	850778	ALT1; YLR089C	metabolic process	mitochondrion;organelle lumen	catalytic activity	0.951	2.282	2.40
Q03028	Mitochondrial 2-oxodicarboxylate carrier 1 [OS=Saccharomyces cerevisiae S288c]	855969	ODC1; YPL134C	metabolic process;transport	membrane;mitochondrion	structural molecule activity;transporter activity	4.275	10.365	2.42
P36013	NAD-dependent malic enzyme, mitochondrial [OS=Saccharomyces cerevisiae S288c]	853839	MAE1; YKL029C	metabolic process	mitochondrion;organelle lumen	catalytic activity;metal ion binding;nucleotide binding	1.512	3.732	2.47
P37298	Succinate dehydrogenase [ubiquinone] cytochrome b small subunit, mitochondrial [OS=Saccharomyces cerevisiae S288c]	851758	SDH4; YDR178W	metabolic process;transport	membrane;mitochondrion	catalytic activity;metal ion binding	3.642	9	2.47
P07256	Cytochrome b-c1 complex subunit 1, mitochondrial [OS=Saccharomyces cerevisiae S288c]	852235	COR1; YBL045C	metabolic process;transport	membrane;mitochondrion	catalytic activity;metal ion binding;protein binding;transporter activity	9.89	24.551	2.48
P38891	Branched-chain-amino-acid aminotransferase, mitochondrial [OS=Saccharomyces cerevisiae S288c]	856615	BAT1; YHR208W	metabolic process;regulation of biological process;response to stimulus	mitochondrion;organelle lumen	catalytic activity	7.111	17.738	2.49
P28834	Isocitrate dehydrogenase [NAD] subunit 1, mitochondrial [OS=Saccharomyces cerevisiae S288c]	855691	IDH1; YNL037C	metabolic process;transport	cytosol;mitochondrion;organelle lumen	catalytic activity;metal ion binding;nucleotide binding;protein binding;RNA binding	7.859	19.691	2.51
P39952	Mitochondrial inner membrane protein OXA1 [OS=Saccharomyces cerevisiae S288c]	856898	OXA1; YER154W	cell organization and biogenesis;transport	membrane;mitochondrion	transporter activity	1.054	2.652	2.52
P00424	Cytochrome c oxidase polypeptide 5A, mitochondrial [OS=Saccharomyces cerevisiae S288c]	855675	COX5A; YNL052W	metabolic process;transport	membrane;mitochondrion	catalytic activity;transporter activity	1.31	3.329	2.54
P16387	Pyruvate dehydrogenase E1 component subunit alpha, mitochondrial [OS=Saccharomyces cerevisiae S288c]	856925	PDA1; YER178W	cell growth;metabolic process	mitochondrion;organelle lumen	catalytic activity	12.46	31.819	2.55
Q12166	2-isopropylmalate synthase 2, mitochondrial [OS=Saccharomyces cerevisiae S288c]	854275	LEU9; YOR108W	metabolic process	mitochondrion	catalytic activity;protein binding	1.707	4.367	2.56
P08067	cytochrome b-c1 complex subunit Rieske, mitochondrial [OS=Saccharomyces cerevisiae S288c]	856689	RIP1; YEL024W	metabolic process;transport	membrane;mitochondrion	catalytic activity;metal ion binding;transporter activity	25.102	67.129	2.67
P41921	glutathione reductase [OS=Saccharomyces cerevisiae S288c]	856014	GLR1; YPL091W	cellular homeostasis;metabolic process;regulation of biological process;response to stimulus	cytoplasm;cytosol;mitochondrion;nucleus	antioxidant activity;catalytic activity;nucleotide binding	0.241	0.655	2.72
P19262	Dihydrolipoyllysine-residue succinyltransferase component of 2-oxoglutarate dehydrogenase complex, mitochondrial [OS=Saccharomyces cerevisiae S288c]	851726	KGD2; YDR148C	cell organization and biogenesis;metabolic process	mitochondrion	catalytic activity;protein binding	5.7	15.496	2.72
P02992	elongation factor Tu, mitochondrial [OS=Saccharomyces cerevisiae S288c]	854359	TUF1; YOR187W	cell organization and biogenesis;metabolic process	mitochondrion	catalytic activity;nucleotide binding;RNA binding	12.043	33.551	2.79
P32891	D-lactate dehydrogenase [cytochrome] 1, mitochondrial [OS=Saccharomyces cerevisiae S288c]	851380	DLD1; YDL174C	metabolic process;transport	membrane;mitochondrion	catalytic activity;nucleotide binding	11.253	32.839	2.92
P38325	Mitochondrial outer membrane protein OM14 [OS=Saccharomyces cerevisiae S288c]	852531	OM14; YBR230C	transport	membrane;mitochondrion		6.197	18.307	2.95

Q12230	Sphingolipid long chain base-responsive protein LSP1 [OS=Saccharomyces cerevisiae S288c]	856103	LSP1; YPL004C	regulation of biological process;response to stimulus;transport	cytoplasm;membrane;mitochondrion		24.119	72.564	3.01
P39726	Glycine cleavage system H protein, mitochondrial [OS=Saccharomyces cerevisiae S288c]	851254	GCV3; YAL044C	metabolic process	mitochondrion	catalytic activity	6.743	20.544	3.05
P38077	ATP synthase subunit gamma, mitochondrial [OS=Saccharomyces cerevisiae S288c]	852327	ATP3; YBR039W	metabolic process;transport	membrane;mitochondrion	catalytic activity;transporter activity	9	27.48	3.05
P48237	Mitochondrial group I intron splicing factor CCM1 [OS=Saccharomyces cerevisiae S288c]	853053	CCM1; YGR150C	cell organization and biogenesis;metabolic process	mitochondrion	RNA binding	0.044	0.136	3.09
P32191	Glycerol-3-phosphate dehydrogenase, mitochondrial [OS=Saccharomyces cerevisiae S288c]	854651	GUT2; YIL155C	metabolic process	membrane;mitochondrion	catalytic activity	6.097	18.855	3.09
P40495	Homoisocitrate dehydrogenase, mitochondrial [OS=Saccharomyces cerevisiae S288c]	854714	LYS12; YIL094C	metabolic process	mitochondrion	catalytic activity;metal ion binding;nucleotide binding	3.217	10.007	3.11
P49367	Homoaconitase, mitochondrial [OS=Saccharomyces cerevisiae S288c]	851820	LYS4; YDR234W	metabolic process	mitochondrion	catalytic activity;metal ion binding;protein binding	0.638	1.994	3.13
Q2V2P9	Uncharacterized protein YDR119W-A [OS=Saccharomyces cerevisiae S288c]	3799970	COX26; YDR119W-A		membrane;mitochondrion		1.154	3.642	3.16
P81451	ATP synthase subunit K, mitochondrial [OS=Saccharomyces cerevisiae S288c]	854077	ATP19; YOL077W-A	cell organization and biogenesis;metabolic process;transport	membrane;mitochondrion	catalytic activity;transporter activity	1.154	3.642	3.16
P38756	tRNA threonylcarbamoyladenosine dehydratase 1 [OS=Saccharomyces cerevisiae S288c]	856392	TCD1; YHR003C; YHR003C	metabolic process	cytosol;membrane;mitochondrion	catalytic activity;nucleotide binding	0.093	0.304	3.27
P34227	Mitochondrial peroxiredoxin PRX1 [OS=Saccharomyces cerevisiae S288c]	852215	PRX1; YBL064C	cellular homeostasis;metabolic process;regulation of biological process;response to stimulus	mitochondrion	antioxidant activity;catalytic activity	5.158	17.33	3.36
Q12165	ATP synthase subunit delta, mitochondrial [OS=Saccharomyces cerevisiae S288c]	851560	ATP16; YDL004W	metabolic process;transport	membrane;mitochondrion	catalytic activity;transporter activity	1.371	4.623	3.37
P04803	Tryptophan--tRNA ligase, mitochondrial [OS=Saccharomyces cerevisiae S288c]	851861	MSW1; YDR268W	metabolic process	cytoplasm;mitochondrion;organelle lumen	catalytic activity;nucleotide binding	0.136	0.468	3.44
P53163	54S ribosomal protein L12, mitochondrial [OS=Saccharomyces cerevisiae S288c]	852811	MNP1; YGL068W	cell organization and biogenesis;metabolic process	mitochondrion;ribosome	RNA binding;structural molecule activity	0.52	1.848	3.55
P36060	NADH-cytochrome b5 reductase 2 [OS=Saccharomyces cerevisiae S288c]	853707	MCR1; YKL150W	metabolic process;response to stimulus	membrane;mitochondrion	catalytic activity;protein binding	10.288	36.927	3.59
P12695	Dihydrolipoyllysine-residue acetyltransferase component of pyruvate dehydrogenase complex, mitochondrial [OS=Saccharomyces cerevisiae S288c]	855653	LAT1; YNL071W	metabolic process	mitochondrion;organelle lumen	catalytic activity	5.898	21.638	3.67
P32340	Rotenone-insensitive NADH-ubiquinone oxidoreductase, mitochondrial [OS=Saccharomyces cerevisiae S288c]	854919	NDI1; YML120C	metabolic process;regulation of biological process	membrane;mitochondrion;organelle lumen	catalytic activity;nucleotide binding	16.433	61.102	3.72
P23180	Probable oxidoreductase AIM17 [OS=Saccharomyces cerevisiae S288c]	856365	AIM17; YHL021C	cell organization and biogenesis;metabolic process	mitochondrion	catalytic activity;metal ion binding	2.981	11.589	3.89
P07257	Cytochrome b-c1 complex subunit 2, mitochondrial [OS=Saccharomyces cerevisiae S288c]	856321	QCR2; YPR191W	metabolic process;transport	membrane;mitochondrion	catalytic activity;metal ion binding;protein binding;transporter activity	21.19	82.768	3.91
P04840	Mitochondrial outer membrane protein porin 1 [OS=Saccharomyces cerevisiae S288c]	855669	POR1; YNL055C	cell death;cell organization and biogenesis;cellular homeostasis;regulation of biological process;transport	cytoplasm;membrane;mitochondrion	transporter activity	15.238	60.585	3.98
P14540	fructose-bisphosphate aldolase [OS=Saccharomyces cerevisiae S288c]	853805	FBA1; YKL060C	metabolic process	cytoplasm;cytosol;membrane;mitochondrion	catalytic activity;metal ion binding	0.896	3.642	4.06
Q08245	protein ZEO1 [OS=Saccharomyces cerevisiae S288c]	854040	ZEO1; YOL109W	cell organization and biogenesis	membrane;mitochondrion	protein binding	2.162	9	4.16
P00360	glyceraldehyde-3-phosphate dehydrogenase 1 [OS=Saccharomyces cerevisiae S288c]	853395	TDH1; YJL052W	metabolic process	cytoplasm;cytosol;membrane;mitochondrion	catalytic activity;nucleotide binding	1.721	7.185	4.17
P19414	Aconitate hydratase, mitochondrial [OS=Saccharomyces cerevisiae S288c]	851013	ACO1; YLR304C	cell organization and biogenesis;metabolic process;transport	cytoplasm;cytosol;mitochondrion;organelle lumen	catalytic activity;DNA binding;metal ion binding	24.885	109.53	4.40
P00890	citrate synthase, mitochondrial [OS=Saccharomyces cerevisiae S288c]	855732	CIT1; YNR001C	metabolic process;transport	cytosol;mitochondrion;organelle lumen	catalytic activity	8.211	36.276	4.42
P00358	glyceraldehyde-3-phosphate dehydrogenase 2 [OS=Saccharomyces cerevisiae S288c]	853465	TDH2; YJR009C	cell death;metabolic process	cytoplasm;cytosol;membrane;mitochondrion;nucleus	catalytic activity;nucleotide binding;protein binding	3.489	15.496	4.44

P00942	Triosephosphate isomerase [OS=Saccharomyces cerevisiae S288c]	851620	TPI1; YDR050C	metabolic process	cytoplasm;cytosol;membrane;mitochondrion	catalytic activity	0.425	1.894	4.46
P07251	ATP synthase subunit alpha, mitochondrial [OS=Saccharomyces cerevisiae S288c]	852177	ATP1; YBL099W	metabolic process;transport	cytosol;membrane;mitochondrion	catalytic activity;nucleotide binding;transporter activity	26.826	120.153	4.48
P25039	Elongation factor G, mitochondrial [OS=Saccharomyces cerevisiae S288c]	850758	MEF1; YLR069C	cell organization and biogenesis;metabolic process	cytosol;mitochondrion	catalytic activity;nucleotide binding;RNA binding	0.151	0.677	4.48
P39518	Long-chain-fatty-acid--CoA ligase 2 [OS=Saccharomyces cerevisiae S288c]	856734	FAA2; YER015W	metabolic process;transport	cytoplasm;mitochondrion	catalytic activity;nucleotide binding	0.535	2.427	4.54
P06208-1	2-isopropylmalate synthase [OS=Saccharomyces cerevisiae S288c]	855619	LEU4; YNL104C	metabolic process	cytoplasm;mitochondrion	catalytic activity;protein binding	4.223	19.309	4.57
P53252	sphingolipid long chain base-responsive protein PIL1 [OS=Saccharomyces cerevisiae S288c]	852977	PIL1; YGR086C	cell organization and biogenesis;regulation of biological process;response to stimulus;transport	cytoplasm;membrane;mitochondrion	protein binding	10.45	49.802	4.77
P06168	ketol-acid reductoisomerase, mitochondrial [OS=Saccharomyces cerevisiae S288c]	851069	ILV5; YLR355C	cell organization and biogenesis;metabolic process	mitochondrion	catalytic activity;DNA binding;metal ion binding	9	42.94	4.77
P32939	GTP-binding protein ypt7 [OS=Saccharomyces cerevisiae S288c]	855012	YPT7; YML001W	cell communication;cell organization and biogenesis;metabolic process;regulation of biological process;response to stimulus;transport	cytosol;endosome;membrane;mitochondrion;vacuole	catalytic activity;nucleotide binding;protein binding	0.166	0.848	5.11
P09624	Dihydrolipoyl dehydrogenase, mitochondrial [OS=Saccharomyces cerevisiae S288c]	850527	LPD1; YFL018C	cellular homeostasis;metabolic process;regulation of biological process	mitochondrion;organelle lumen	catalytic activity;nucleotide binding	21.539	113.505	5.27
P53312	Succinyl-CoA ligase [ADP-forming] subunit beta, mitochondrial [OS=Saccharomyces cerevisiae S288c]	853159	LSC2; YGR244C	metabolic process	mitochondrion	catalytic activity;nucleotide binding	7.003	37.075	5.29
P33303	Succinate/fumarate mitochondrial transporter [OS=Saccharomyces cerevisiae S288c]	853558	SFC1; YJR095W	metabolic process;transport	membrane;mitochondrion	structural molecule activity;transporter activity	6.743	39.842	5.91
P17505	Malate dehydrogenase, mitochondrial [OS=Saccharomyces cerevisiae S288c]	853777	MDH1; YKL085W	metabolic process	mitochondrion;organelle lumen	catalytic activity;protein binding;RNA binding	28.936	172.02	5.94
P12709	glucose-6-phosphate isomerase [OS=Saccharomyces cerevisiae S288c]	852495	PGI1; YBR196C	metabolic process	cytoplasm;membrane;mitochondrion	catalytic activity	0.166	0.995	5.99
P20967	2-oxoglutarate dehydrogenase, mitochondrial [OS=Saccharomyces cerevisiae S288c]	854681	KGD1; YIL125W	metabolic process	cytosol;mitochondrion;organelle lumen	catalytic activity	8.168	55.848	6.84
P16547	Mitochondrial outer membrane protein OM45 [OS=Saccharomyces cerevisiae S288c]	854670	OM45; YIL136W		membrane;mitochondrion		300.995	2088.296	6.94
P19882	heat shock protein 60, mitochondrial [OS=Saccharomyces cerevisiae S288c]	850963	HSP60; YLR259C	cell organization and biogenesis;metabolic process;regulation of biological process;transport	cytoplasm;cytosol;membrane;mitochondrion;organelle lumen	catalytic activity;DNA binding;nucleotide binding;protein binding	13.384	93.12	6.96
P46367	Potassium-activated aldehyde dehydrogenase, mitochondrial [OS=Saccharomyces cerevisiae S288c]	854556	ALD4; YOR374W	metabolic process	mitochondrion;organelle lumen	catalytic activity	24.119	219.22	9.09
P0CS90	Heat shock protein SSC1, mitochondrial [OS=Saccharomyces cerevisiae S288c]	853503	SSC1; YJR045C	cell organization and biogenesis;metabolic process;regulation of biological process;transport	membrane;mitochondrion;nucleus;organelle lumen	catalytic activity;enzyme regulator activity;nucleotide binding;protein binding	26.826	243.844	9.09
P32316	acetyl-CoA hydrolase [OS=Saccharomyces cerevisiae S288c]	852266	ACH1; YBL015W	metabolic process	cytoplasm;cytosol;mitochondrion	catalytic activity	10.498	99	9.43
P18239	ADP,ATP carrier protein 2 [OS=Saccharomyces cerevisiae S288c]	852250	PET9; YBL030C	cell death;metabolic process;transport	membrane;mitochondrion	structural molecule activity;transporter activity	47.064	591.553	12.57

Supplemental Table S7. The relative concentrations of proteins in mitochondria purified from *ups1Δ* cells cultured with or without LCA. Mitochondria were purified from *ups1Δ* cells recovered on day 2 of cell culturing with or without LCA. Mass spectrometry-based identification and quantitation of proteins recovered in purified mitochondria were performed as described in Materials and methods. The exponentially modified protein abundance index (emPAI) provided by the ″Proteome Discoverer″ software was used to calculate the relative abundance of mitochondrial proteins between specified datasets. Data on the relative concentrations of mitochondrial proteins are presented as means of 2 independent experiments.

Accession	Description	Entrez Gene ID	Gene ID	Biological Process	Cellular Component	Molecular Function	emPAI *ups1*	emPAI *ups1* + LCA	Ratio *ups1* + LCA/ *ups1*
P00175	Cytochrome b2, mitochondrial [	854950	CYB2	metabolic process; transport	cytosol; membrane; mitochondrion; nucleus	catalytic activity; metal ion binding; nucleotide binding	1.371	0.059	0.04
P16451	Pyruvate dehydrogenase complex protein X component, mitochondrial [OS=Saccharomyces cerevisiae S288c]	853107	PDX1; YGR193C	metabolic process	mitochondrion;organelle lumen	catalytic activity;structural molecule activity	1.555	0.089	0.06
P43635	Citrate synthase 3, mitochondrial [OS=Saccharomyces cerevisiae S288c]	856107	CIT3; YPR001W	metabolic process	mitochondrion	catalytic activity	2.914	0.186	0.06
P40513	Mitochondrial acidic protein MAM33 [OS=Saccharomyces cerevisiae S288c]	854740	MAM33; YIL070C	metabolic process;regulation of biological process	mitochondrion;organelle lumen	translation regulator activity	2.831	0.212	0.07
P53140	Protein RMD9, mitochondrial [OS=Saccharomyces cerevisiae S288c]	852771	RMD9; YGL107C	cell differentiation;metabolic process;regulation of biological process	membrane;mitochondrion	RNA binding	0.668	0.053	0.08
P81449	ATP synthase subunit e, mitochondrial [OS=Saccharomyces cerevisiae S288c]	851922	TIM11; YDR322C-A	cell organization and biogenesis;metabolic process;transport	membrane;mitochondrion	catalytic activity;structural molecule activity;transporter activity	9	0.778	0.09
P00360	glyceraldehyde-3-phosphate dehydrogenase 1 [OS=Saccharomyces cerevisiae S288c]	853395	TDH1; YJL052W	metabolic process	cytoplasm;cytosol;membrane;mitochondrion	catalytic activity;nucleotide binding	3.062	0.35	0.11
P16547	Mitochondrial outer membrane protein OM45 [OS=Saccharomyces cerevisiae S288c]	854670	OM45; YIL136W		membrane;mitochondrion		830.764	99	0.12
P32891	D-lactate dehydrogenase [cytochrome] 1, mitochondrial [OS=Saccharomyces cerevisiae S288c]	851380	DLD1; YDL174C	metabolic process;transport	membrane;mitochondrion	catalytic activity;nucleotide binding	11.253	1.412	0.13
P43567	alanine--glyoxylate aminotransferase 1 [OS=Saccharomyces cerevisiae S288c]	850514	AGX1; YFL030W	metabolic process	cytosol;mitochondrion	catalytic activity	1.61	0.212	0.13
P00431	cytochrome c peroxidase, mitochondrial [OS=Saccharomyces cerevisiae S288c]	853940	CCP1; YKR066C	metabolic process;response to stimulus	mitochondrion;organelle lumen	antioxidant activity;catalytic activity;metal ion binding;protein binding	0.701	0.093	0.13
P32787	Mitochondrial genome maintenance protein MGM101 [OS=Saccharomyces cerevisiae S288c]	853609	MGM101; YJR144W	cell organization and biogenesis;metabolic process;response to stimulus	chromosome;mitochondrion	DNA binding	3.281	0.438	0.13
Q02981	ABC1 family protein YPL109C, mitochondrial [OS=Saccharomyces cerevisiae S288c]	855995	YPL109C; YPL109C		mitochondrion		0.413	0.059	0.14
P32939	GTP-binding protein ypt7 [OS=Saccharomyces cerevisiae S288c]	855012	YPT7; YML001W	cell communication;cell organization and biogenesis;metabolic process;regulation of biological process;response to stimulus;transport	cytosol;endosome;membrane;mitochondrion;vacuole	catalytic activity;nucleotide binding;protein binding	1.154	0.166	0.14
P00927	Threonine dehydratase, mitochondrial [OS=Saccharomyces cerevisiae S288c]	856819	ILV1; YER086W	metabolic process	cytoplasm;mitochondrion	catalytic activity	1.285	0.194	0.15
Q02486	ARS-binding factor 2, mitochondrial [OS=Saccharomyces cerevisiae S288c]	855094	ABF2; YMR072W	cell organization and biogenesis;metabolic process;regulation of biological process	chromosome;mitochondrion;nucleus	DNA binding	48.239	7.377	0.15
P36521	54S ribosomal protein L11, mitochondrial [OS=Saccharomyces cerevisiae S288c]	851325	MRPL11; YDL202W	cell organization and biogenesis;metabolic process	mitochondrion;ribosome	structural molecule activity	0.833	0.129	0.15

P38891	Branched-chain-amino-acid aminotransferase, mitochondrial [OS=Saccharomyces cerevisiae S288c]	856615	BAT1; YHR208W	metabolic process;regulation of biological process;response to stimulus	mitochondrion;organelle lumen	catalytic activity	10.103	1.565	0.15
P49095	Glycine dehydrogenase (Decarboxylating), mitochondrial [OS=Saccharomyces cerevisiae S288c]	855227	GCV2; YMR189W	metabolic process	cytosol;mitochondrion	catalytic activity	1.471	0.228	0.15
P15424	ATP-dependent RNA helicase MSS116, mitochondrial [OS=Saccharomyces cerevisiae S288c]	851775	MSS116; YDR194C	metabolic process;regulation of biological process	mitochondrion;organelle lumen	catalytic activity;nucleotide binding;RNA binding	0.668	0.108	0.16
Q00711	Succinate dehydrogenase [ubiquinone] flavoprotein subunit, mitochondrial [OS=Saccharomyces cerevisiae S288c]	853709	SDH1; YKL148C	metabolic process;transport	membrane;mitochondrion	catalytic activity;nucleotide binding;protein binding	12.242	2.075	0.17
P53969	Sorting assembly machinery 50 kDa subunit [OS=Saccharomyces cerevisiae S288c]	855705	SAM50; YNL026W	cell organization and biogenesis;transport	membrane;mitochondrion	protein binding;transporter activity	0.487	0.083	0.17
P39006	Phosphatidylserine decarboxylase proenzyme 1, mitochondrial [OS=Saccharomyces cerevisiae S288c]	855552	PSD1; YNL169C	metabolic process;regulation of biological process	membrane;mitochondrion	catalytic activity	0.487	0.083	0.17
P04807	Hexokinase-2 [OS=Saccharomyces cerevisiae S288c]	852639	HXK2; YGL253W	cell communication;cell organization and biogenesis;cellular homeostasis;metabolic process;regulation of biological process;response to stimulus;transport	cytosol;mitochondrion;nucleus	catalytic activity;nucleotide binding	0.468	0.08	0.17
P32897	Mitochondrial import inner membrane translocase subunit tim23 [OS=Saccharomyces cerevisiae S288c]	855751	TIM23; YNR017W	transport	membrane;mitochondrion	protein binding;transporter activity	1.512	0.259	0.17
P39522	Dihydroxy-acid dehydratase, mitochondrial [OS=Saccharomyces cerevisiae S288c]	853473	ILV3; YJR016C	metabolic process	mitochondrion	catalytic activity;metal ion binding	14.199	2.511	0.18
P32340	Rotenone-insensitive NADH-ubiquinone oxidoreductase, mitochondrial [OS=Saccharomyces cerevisiae S288c]	854919	NDI1; YML120C	metabolic process;regulation of biological process	membrane;mitochondrion;organelle lumen	catalytic activity;nucleotide binding	29.392	5.21	0.18
P53311	Mitochondrial pyruvate carrier 3 [OS=Saccharomyces cerevisiae S288c]	853158	FMP43; MPC3; YGR243W	transport	membrane;mitochondrion	transporter activity	1.154	0.212	0.18
P20967	2-oxoglutarate dehydrogenase, mitochondrial [OS=Saccharomyces cerevisiae S288c]	854681	KGD1; YIL125W	metabolic process	cytosol;mitochondrion;organelle lumen	catalytic activity	22.843	4.212	0.18
P04037	cytochrome c oxidase subunit 4, mitochondrial [OS=Saccharomyces cerevisiae S288c]	852688	COX4; YGL187C	cell organization and biogenesis;metabolic process;transport	membrane;mitochondrion	catalytic activity;metal ion binding;transporter activity	17.738	3.329	0.19
P36013	NAD-dependent malic enzyme, mitochondrial [OS=Saccharomyces cerevisiae S288c]	853839	MAE1; YKL029C	metabolic process	mitochondrion;organelle lumen	catalytic activity;metal ion binding;nucleotide binding	2.162	0.413	0.19
P36060	NADH-cytochrome b5 reductase 2 [OS=Saccharomyces cerevisiae S288c]	853707	MCR1; YKL150W	metabolic process;response to stimulus	membrane;mitochondrion	catalytic activity;protein binding	19.691	3.833	0.19
P08525	Cytochrome b-c1 complex subunit 8 [OS=Saccharomyces cerevisiae S288c]	853273	QCR8; YJL166W	metabolic process;transport	membrane;mitochondrion	catalytic activity;transporter activity	2.981	0.585	0.20
P52893	probable alanine aminotransferase, mitochondrial [OS=Saccharomyces cerevisiae S288c]	850778	ALT1; YLR089C	metabolic process	mitochondrion;organelle lumen	catalytic activity	1.264	0.25	0.20
P21954	Isocitrate dehydrogenase [NADP], mitochondrial [OS=Saccharomyces cerevisiae S288c]	851493	IDP1; YDL066W	metabolic process	mitochondrion	catalytic activity;metal ion binding;nucleotide binding;protein binding	45.416	9	0.20
P28834	Isocitrate dehydrogenase [NAD] subunit 1, mitochondrial [OS=Saccharomyces cerevisiae S288c]	855691	IDH1; YNL037C	metabolic process;transport	cytosol;mitochondrion;organelle lumen	catalytic activity;metal ion binding;nucleotide binding;protein binding;RNA binding	11.743	2.36	0.20
P49367	Homoaconitase, mitochondrial [OS=Saccharomyces cerevisiae S288c]	851820	LYS4; YDR234W	metabolic process	mitochondrion	catalytic activity;metal ion binding;protein binding	1.154	0.245	0.21
P00427	Cytochrome c oxidase subunit 6, mitochondrial [OS=Saccharomyces cerevisiae S288c]	856448	COX6; YHR051W	metabolic process;transport	membrane;mitochondrion	catalytic activity;metal ion binding;transporter activity	3.642	0.778	0.21
P53312	Succinyl-CoA ligase [ADP-forming] subunit beta, mitochondrial [OS=Saccharomyces cerevisiae S288c]	853159	LSC2; YGR244C	metabolic process	mitochondrion	catalytic activity;nucleotide binding	10.602	2.282	0.22
P07251	ATP synthase subunit alpha, mitochondrial [OS=Saccharomyces cerevisiae S288c]	852177	ATP1; YBL099W	metabolic process;transport	cytosol;membrane;mitochondrion	catalytic activity;nucleotide binding;transporte	58.948	12.769	0.22

						r activity			
P00128	Cytochrome b-c1 complex subunit 7 [OS=Saccharomyces cerevisiae S288c]	852142	QCR7; YDR529C	cell organization and biogenesis;metabolic process;transport	membrane;mitochondrion	catalytic activity;transporter activity	11.915	2.594	0.22
P0CS90	Heat shock protein SSC1, mitochondrial [OS=Saccharomyces cerevisiae S288c]	853503	SSC1; YJR045C	cell organization and biogenesis;metabolic process;regulation of biological process;transport	membrane;mitochondrion;nucleus;organelle lumen	catalytic activity;enzyme regulator activity;nucleotide binding;protein binding	67.129	14.647	0.22
Q02883	N-acyl-phosphatidylethanolamine-hydrolyzing phospholipase D, mitochondrial [OS=Saccharomyces cerevisiae S288c]	856001	FMP30; YPL103C	metabolic process	membrane;mitochondrion	catalytic activity;metal ion binding	0.425	0.093	0.22
P38969	Pentamidine resistance factor, mitochondrial [OS=Saccharomyces cerevisiae S288c]	854440	PNT1; YOR266W	cell organization and biogenesis;response to stimulus	membrane;mitochondrion		0.359	0.08	0.22
P38088	Glycine--tRNA ligase 1, mitochondrial [OS=Saccharomyces cerevisiae S288c]		GRS1	metabolic process	cytoplasm;mitochondrion;organelle lumen	catalytic activity;nucleotide binding;protein binding	0.212	0.049	0.23
P00560	Phosphoglycerate kinase [OS=Saccharomyces cerevisiae S288c]	850370	PGK1; YCR012W	metabolic process	cytoplasm;membrane;mitochondrion	catalytic activity;nucleotide binding	3.642	0.848	0.23
P46681	D-lactate dehydrogenase [cytochrome] 2, mitochondrial [OS=Saccharomyces cerevisiae S288c]	851376	DLD2; YDL178W	metabolic process	mitochondrion;organelle lumen	catalytic activity;nucleotide binding;protein binding	0.682	0.16	0.23
P32796-1	Carnitine O-acetyltransferase, mitochondrial [OS=Saccharomyces cerevisiae S288c]	854965	CAT2; YML042W	metabolic process;transport	membrane;mitochondrion	catalytic activity	9.578	2.252	0.24
P36112	MICOS complex subunit Mic60 [OS=Saccharomyces cerevisiae S288c]	853886	FCJ1; MIC60; YKR016W	cell organization and biogenesis;transport	membrane;mitochondrion		12.895	3.047	0.24
P02992	elongation factor Tu, mitochondrial [OS=Saccharomyces cerevisiae S288c]	854359	TUF1; YOR187W	cell organization and biogenesis;metabolic process	mitochondrion	catalytic activity;nucleotide binding;RNA binding	9	2.162	0.24
P39952	Mitochondrial inner membrane protein OXA1 [OS=Saccharomyces cerevisiae S288c]	856898	OXA1; YER154W	cell organization and biogenesis;transport	membrane;mitochondrion	transporter activity	1.371	0.334	0.24
P54783	D-arabinono-1,4-lactone oxidase [OS=Saccharomyces cerevisiae S288c]	854888	ALO1; YML086C	metabolic process;response to stimulus	membrane;mitochondrion	catalytic activity;nucleotide binding	5.31	1.326	0.25
P32316	acetyl-CoA hydrolase [OS=Saccharomyces cerevisiae S288c]	852266	ACH1; YBL015W	metabolic process	cytoplasm;cytosol;mitochondrion	catalytic activity	20.544	5.136	0.25
Q02784	Monothiol glutaredoxin-5, mitochondrial [OS=Saccharomyces cerevisiae S288c]	856048	GRX5; YPL059W	cell organization and biogenesis;cellular homeostasis;metabolic process;regulation of biological process;response to stimulus	mitochondrion;organelle lumen	catalytic activity;metal ion binding	1.154	0.292	0.25
P32317	Protein AFG1 [OS=Saccharomyces cerevisiae S288c]	856658	AFG1; YEL052W	cell organization and biogenesis;metabolic process;response to stimulus;transport	membrane;mitochondrion	catalytic activity;nucleotide binding	0.995	0.259	0.26
P40502	Altered inheritance of mitochondria protein 19, mitochondrial [OS=Saccharomyces cerevisiae S288c]	854722	AIM19; YIL087C		membrane;mitochondrion		0.995	0.259	0.26
P53206	putative cysteine synthase [OS=Saccharomyces cerevisiae S288c]	852895	MCY1; YGR012W; YGR012W	metabolic process		catalytic activity	1.555	0.407	0.26
P12695	Dihydrolipoyllysine-residue acetyltransferase component of pyruvate dehydrogenase complex, mitochondrial [OS=Saccharomyces cerevisiae S288c]	855653	LAT1; YNL071W	metabolic process	mitochondrion;organelle lumen	catalytic activity	10.602	2.808	0.26
P36517	54S ribosomal protein L4, mitochondrial [OS=Saccharomyces cerevisiae S288c]	851160	MRPL4; YLR439W	cell organization and biogenesis;metabolic process	mitochondrion;ribosome	structural molecule activity	0.874	0.233	0.27
P10507	mitochondrial-processing peptidase subunit beta [OS=Saccharomyces cerevisiae S288c]	850860	MAS1; YLR163C	metabolic process;transport	mitochondrion;organelle lumen	catalytic activity;metal ion binding;protein binding	0.778	0.212	0.27
P07246	Alcohol dehydrogenase 3, mitochondrial [OS=Saccharomyces cerevisiae S288c]	855107	ADH3; YMR083W	metabolic process	mitochondrion;organelle lumen	catalytic activity;metal ion binding	11.452	3.16	0.28
P34222	Peptidyl-tRNA hydrolase 2 [OS=Saccharomyces cerevisiae S288c]	852223	PTH2; YBL057C	regulation of biological process	cytoplasm;cytosol;membrane;mitochondrion	catalytic activity;protein binding	0.701	0.194	0.28
Q05931	Heat shock protein SSQ1, mitochondrial [OS=Saccharomyces cerevisiae S288c]	851084	SSQ1; YLR369W	cell organization and biogenesis;metabolic process	mitochondrion;organelle lumen	nucleotide binding;protein binding	0.679	0.189	0.28
P30902	ATP synthase subunit d, mitochondrial [OS=Saccharomyces	853853	ATP7; YKL016	metabolic process;transport	membrane;mitochondrion	catalytic activity;transporte	18.307	5.105	0.28

	cerevisiae S288c]		C			r activity			
P09624	Dihydrolipoyl dehydrogenase, mitochondrial [OS=Saccharomyces cerevisiae S288c]	850527	LPD1; YFL018C	cellular homeostasis;metabolic process;regulation of biological process	mitochondrion;organelle lumen	catalytic activity;nucleotide binding	43.367	12.111	0.28
P37267	Assembly factor cbp4 [OS=Saccharomyces cerevisiae S288c]		CBP4	cell organization and biogenesis	membrane;mitochondrion		1.848	0.52	0.28
P27929	37S ribosomal protein NAM9, mitochondrial [OS=Saccharomyces cerevisiae S288c]	855585	NAM9; YNL137C	cell organization and biogenesis;metabolic process;regulation of biological process	mitochondrion;ribosome	RNA binding;structural molecule activity	1.062	0.301	0.28
P28241	Isocitrate dehydrogenase [NAD] subunit 2, mitochondrial [OS=Saccharomyces cerevisiae S288c]	854303	IDH2; YOR136W	metabolic process	mitochondrion;organelle lumen	catalytic activity;metal ion binding;nucleotide binding;protein binding;RNA binding	7.031	1.994	0.28
P37299	cytochrome b-c1 complex subunit 10 [OS=Saccharomyces cerevisiae S288c]	856390	QCR10; YHR001W-A	metabolic process;transport	membrane;mitochondrion	catalytic activity;transporter activity	5.31	1.512	0.28
P19414	Aconitate hydratase, mitochondrial [OS=Saccharomyces cerevisiae S288c]	851013	ACO1; YLR304C	cell organization and biogenesis;metabolic process;transport	cytoplasm;cytosol;mitochondrion;organelle lumen	catalytic activity;DNA binding;metal ion binding	43.893	12.503	0.28
P23180	Probable oxidoreductase AIM17 [OS=Saccharomyces cerevisiae S288c]	856365	AIM17; YHL021C	cell organization and biogenesis;metabolic process	mitochondrion	catalytic activity;metal ion binding	3.467	0.995	0.29
P14693	sorting assembly machinery 35 kDa subunit [OS=Saccharomyces cerevisiae S288c]	856483	SAM35; YHR083W	cell organization and biogenesis;transport	membrane;mitochondrion	protein binding	0.54	0.155	0.29
P41735	5-demethoxyubiquinone hydroxylase, mitochondrial [OS=Saccharomyces cerevisiae S288c]	854292	CAT5; YOR125C	metabolic process	membrane;mitochondrion	catalytic activity;metal ion binding	0.54	0.155	0.29
Q12375	mitochondrial ornithine transporter 1 [OS=Saccharomyces cerevisiae S288c]	854297	ORT1; YOR130C	metabolic process;transport	membrane;mitochondrion	structural molecule activity;transporter activity	0.54	0.155	0.29
Q12031	Mitochondrial 2-methylisocitrate lyase [OS=Saccharomyces cerevisiae S288c]	856114	ICL2; YPR006C	metabolic process	mitochondrion;organelle lumen	catalytic activity	3.281	0.947	0.29
P32387	54S ribosomal protein L41, mitochondrial [OS=Saccharomyces cerevisiae S288c]	852014	MRP20; YDR405W	cell organization and biogenesis;metabolic process	mitochondrion;ribosome	nucleotide binding;protein binding;RNA binding;structural molecule activity	0.501	0.145	0.29
P47127	Altered inheritance of mitochondria protein 24, mitochondrial [OS=Saccharomyces cerevisiae S288c]	853543	AIM24; YJR080C	cell organization and biogenesis	mitochondrion		2.562	0.743	0.29
P00401	Cytochrome c oxidase subunit 1 [OS=Saccharomyces cerevisiae S288c]	854598	COX1; Q0045	metabolic process;transport	membrane;mitochondrion	catalytic activity;metal ion binding;transporter activity	1.61	0.468	0.29
P43617	Uncharacterized mitochondrial carrier YFR045W [OS=Saccharomyces cerevisiae S288c]	850606	YFR045W; YFR045W	metabolic process;transport	membrane;mitochondrion	structural molecule activity;transporter activity	0.413	0.122	0.30
P32792	UPF0744 protein YSC83 [OS=Saccharomyces cerevisiae S288c]	856410	YSC83; YHR017W		membrane;mitochondrion		0.369	0.11	0.30
P53736	Uncharacterized protein YNR040W [OS=Saccharomyces cerevisiae S288c]	855776	YNR040W; YNR040W		mitochondrion		0.389	0.116	0.30
P35180	mitochondrial import receptor subunit Tom20 [OS=Saccharomyces cerevisiae S288c]	852973	TOM20; YGR082W	cell organization and biogenesis;transport	membrane;mitochondrion	protein binding;transporter activity	1.424	0.425	0.30
P07275	Delta-1-pyrroline-5-carboxylate dehydrogenase, mitochondrial [OS=Saccharomyces cerevisiae S288c]	856432	PUT2; YHR037W	metabolic process	membrane;mitochondrion;organelle lumen	catalytic activity	4.337	1.31	0.30
P38702	mitochondrial carrier protein LEU5 [OS=Saccharomyces cerevisiae S288c]	856391	LEU5; YHR002W	metabolic process;transport	membrane;mitochondrion	structural molecule activity;transporter activity	0.334	0.101	0.30
P34231	Uncharacterized protein YKL187C [OS=Saccharomyces cerevisiae S288c]	853648	FAT3; YKL187C; YKL187C	cell organization and biogenesis;transport	membrane;mitochondrion		0.304	0.093	0.31
P48360	Probable NADPH:adrenodoxin oxidoreductase, mitochondrial [OS=Saccharomyces cerevisiae S288c]	851982	ARH1; YDR376W	cellular homeostasis;metabolic process;transport	cytoplasm;membrane;mitochondrion;organelle lumen	catalytic activity	0.25	0.077	0.31
Q04172	Sensitive to high expression protein 9, mitochondrial	852002	SHE9; YDR39	cell organization and biogenesis	membrane;mitochondrion		0.269	0.083	0.31

	[OS=Saccharomyces cerevisiae S288c]		3W						
P36775	Lon protease homolog, mitochondrial [OS=Saccharomyces cerevisiae S288c]	852259	PIM1; YBL022C	cell organization and biogenesis;metabolic process;regulation of biological process;response to stimulus	cytoplasm;mitochondrion;organelle lumen	catalytic activity;DNA binding;nucleotide binding;RNA binding	0.537	0.166	0.31
P40495	Homoisocitrate dehydrogenase, mitochondrial [OS=Saccharomyces cerevisiae S288c]	854714	LYS12; YIL094C	metabolic process	mitochondrion	catalytic activity;metal ion binding;nucleotide binding	5.19	1.61	0.31
Q05892	MIOREX complex component 2 [OS=Saccharomyces cerevisiae S288c]	850997	YLR290C; COQ11; YLR290C	metabolic process	mitochondrion	catalytic activity	1.738	0.54	0.31
P36525	54S ribosomal protein L24, mitochondrial [OS=Saccharomyces cerevisiae S288c]	855231	MRPL24; YMR193W	cell organization and biogenesis;metabolic process	mitochondrion;ribosome	RNA binding;structural molecule activity	1.154	0.359	0.31
Q04728	Arginine biosynthesis bifunctional protein ArgJ, mitochondrial [OS=Saccharomyces cerevisiae S288c]	855084	ARG7; YMR062C	metabolic process	mitochondrion;organelle lumen	catalytic activity	0.748	0.233	0.31
Q06567	ABC1 family protein MCP2 [OS=Saccharomyces cerevisiae S288c]	850955	MCP2; YLR253W	cell organization and biogenesis	membrane;mitochondrion		0.218	0.068	0.31
P39722	Mitochondrial Rho GTPase 1 [OS=Saccharomyces cerevisiae S288c]	851249	GEM1; YAL048C	cell organization and biogenesis;regulation of biological process;response to stimulus;transport	membrane;mitochondrion	catalytic activity;metal ion binding;nucleotide binding	0.189	0.059	0.31
P16622	Ferrochelatase, mitochondrial [OS=Saccharomyces cerevisiae S288c]	854347	HEM15; YOR176W	metabolic process	membrane;mitochondrion	catalytic activity	8.006	2.511	0.31
P38071	Enoyl-[acyl-carrier protein] reductase [NADPH, B-specific], mitochondrial [OS=Saccharomyces cerevisiae S288c]	852314	ETR1; YBR026C	metabolic process	mitochondrion;organelle lumen	catalytic activity;DNA binding;metal ion binding	8.006	2.511	0.31
P32898	Mitochondrial presequence protease [OS=Saccharomyces cerevisiae S288c]	852041	CYM1; YDR430C	metabolic process	mitochondrion	catalytic activity;metal ion binding	0.505	0.16	0.32
P19955	37S ribosomal protein YMR-31, mitochondrial [OS=Saccharomyces cerevisiae S288c]	850610	YMR31; YFR049W	cell organization and biogenesis;metabolic process	mitochondrion;ribosome	catalytic activity;protein binding;structural molecule activity	3.642	1.154	0.32
Q01519	Cytochrome c oxidase subunit 6b [OS=Saccharomyces cerevisiae S288c]	850727	COX12; YLR038C	cell organization and biogenesis;metabolic process;transport	mitochondrion	catalytic activity;transporter activity	3.642	1.154	0.32
Q3E824	Uncharacterized protein YOR020W-A [OS=Saccharomyces cerevisiae S288c]	1466480	YOR020W-A; YOR020W-A	metabolic process;transport	membrane;mitochondrion		3.642	1.154	0.32
Q2V2P9	Uncharacterized protein YDR119W-A [OS=Saccharomyces cerevisiae S288c]	3799970	COX26; YDR119W-A		membrane;mitochondrion		3.642	1.154	0.32
P38771	Ribosome-recycling factor, mitochondrial [OS=Saccharomyces cerevisiae S288c]	856433	RRF1; YHR038W	cell organization and biogenesis;metabolic process	mitochondrion	RNA binding	1.581	0.501	0.32
P19882	heat shock protein 60, mitochondrial [OS=Saccharomyces cerevisiae S288c]	850963	HSP60; YLR259C	cell organization and biogenesis;metabolic process;regulation of biological process;transport	cytoplasm;cytosol;membrane;mitochondrion;organelle lumen	catalytic activity;DNA binding;nucleotide binding;protein binding	36.927	11.743	0.32
P36151	Uncharacterized protein YKR070W [OS=Saccharomyces cerevisiae S288c]	853944	YKR070W; YKR070W	metabolic process	mitochondrion		0.833	0.274	0.33
P08417	fumarate hydratase, mitochondrial [OS=Saccharomyces cerevisiae S288c]	855866	FUM1; YPL262W	cell organization and biogenesis;metabolic process	cytoplasm;cytosol;mitochondrion;organelle lumen	catalytic activity	6.88	2.29	0.33
P53292	37S ribosomal protein S35, mitochondrial [OS=Saccharomyces cerevisiae S288c]	853075	MRPS35; YGR165W	cell organization and biogenesis;metabolic process	mitochondrion;ribosome	structural molecule activity	1.754	0.585	0.33
P16387	Pyruvate dehydrogenase E1 component subunit alpha, mitochondrial [OS=Saccharomyces cerevisiae S288c]	856925	PDA1; YER178W	cell growth;metabolic process	mitochondrion;organelle lumen	catalytic activity	13.497	4.52	0.33
P47052	succinate dehydrogenase [ubiquinone] flavoprotein subunit 2, mitochondrial [OS=Saccharomyces cerevisiae S288c]	853405	YJL045W; YJL045W	metabolic process;transport	membrane;mitochondrion	catalytic activity;nucleotide binding	2.932	0.983	0.34
P51998	54S ribosomal protein yml6, mitochondrial [OS=Saccharomyces cerevisiae S288c]	854983	YML6; YML025C	cell organization and biogenesis;metabolic process	mitochondrion;ribosome	RNA binding;structural molecule activity	0.73	0.245	0.34
P38885	Altered inheritance of mitochondria protein 46, mitochondrial [OS=Saccharomyces cerevisiae S288c]	856606	AIM46; YHR199C		mitochondrion	catalytic activity	1.154	0.389	0.34

Q03430	37S ribosomal protein RSM28, mitochondrial [OS=Saccharomyces cerevisiae S288c]	852105	RSM28; YDR494W	cell organization and biogenesis;metabolic process	mitochondrion;ribosome	structural molecule activity	0.65	0.222	0.34
P25719	Peptidyl-prolyl cis-trans isomerase C, mitochondrial [OS=Saccharomyces cerevisiae S288c]	854897	CPR3; YML078W	cell death;metabolic process	mitochondrion;organelle lumen	catalytic activity	2.415	0.848	0.35
P32191	Glycerol-3-phosphate dehydrogenase, mitochondrial [OS=Saccharomyces cerevisiae S288c]	854651	GUT2; YIL155C	metabolic process	membrane;mitochondrion	catalytic activity	6.827	2.403	0.35
P00924	Enolase 1 [OS=Saccharomyces cerevisiae S288c]	853169	ENO1; YGR254W	metabolic process;regulation of biological process	cytoplasm;cytosol;membrane;mitochondrion;vacuole	catalytic activity;metal ion binding;protein binding	1.326	0.468	0.35
P32843	Mitochondrial escape protein 2 [OS=Saccharomyces cerevisiae S288c]	855348	YME2; YMR302C	cell organization and biogenesis;metabolic process	membrane;mitochondrion	nucleotide binding;RNA binding	2.36	0.833	0.35
P42940	Probable electron transfer flavoprotein subunit beta [OS=Saccharomyces cerevisiae S288c]	853121	CIR1; YGR207C	metabolic process;transport	mitochondrion;organelle lumen		3.87	1.371	0.35
P04840	Mitochondrial outer membrane protein porin 1 [OS=Saccharomyces cerevisiae S288c]	855669	POR1; YNL055C	cell death;cell organization and biogenesis;cellular homeostasis;regulation of biological process;transport	cytoplasm;membrane;mitochondrion	transporter activity	25.367	9	0.35
P07213	Mitochondrial import receptor subunit TOM70 [OS=Saccharomyces cerevisiae S288c]	855602	TOM70; YNL121C	cell organization and biogenesis;transport	membrane;mitochondrion	protein binding;transporter activity	2.257	0.805	0.36
P39726	Glycine cleavage system H protein, mitochondrial [OS=Saccharomyces cerevisiae S288c]	851254	GCV3; YAL044C	metabolic process	mitochondrion	catalytic activity	4.995	1.783	0.36
P07806	Valine--tRNA ligase, mitochondrial [OS=Saccharomyces cerevisiae S288c]	852986	VAS1; YGR094W	metabolic process;regulation of biological process	cytoplasm;cytosol;mitochondrion	catalytic activity;nucleotide binding	0.377	0.136	0.36
P09440	C-1-tetrahydrofolate synthase, mitochondrial [OS=Saccharomyces cerevisiae S288c]	852378	MIS1; YBR084W	metabolic process	cytosol;mitochondrion	catalytic activity;nucleotide binding	0.829	0.302	0.36
Q12298	uncharacterized ABC transporter ATP-binding protein YDR061W [OS=Saccharomyces cerevisiae S288c]	851633	YDR061W; YDR061W		mitochondrion	catalytic activity;nucleotide binding	0.343	0.125	0.36
P34227	Mitochondrial peroxiredoxin PRX1 [OS=Saccharomyces cerevisiae S288c]	852215	PRX1; YBL064C	cellular homeostasis;metabolic process;regulation of biological process;response to stimulus	mitochondrion	antioxidant activity;catalytic activity	4.456	1.637	0.37
P07143	Cytochrome c1, heme protein, mitochondrial [OS=Saccharomyces cerevisiae S288c]	854231	CYT1; YOR065W	metabolic process;transport	cytosol;membrane;mitochondrion	metal ion binding	13.251	4.878	0.37
P23644	Mitochondrial import receptor subunit TOM40 [OS=Saccharomyces cerevisiae S288c]	855243	TOM40; YMR203W	transport	cytosol;membrane;mitochondrion	protein binding;transporter activity	1.783	0.668	0.37
P06208-1	2-isopropylmalate synthase [OS=Saccharomyces cerevisiae S288c]	855619	LEU4; YNL104C	metabolic process	cytoplasm;mitochondrion	catalytic activity;protein binding	6.017	2.257	0.38
Q01852	Mitochondrial import inner membrane translocase subunit TIM44 [OS=Saccharomyces cerevisiae S288c]	854790	TIM44; YIL022W	transport	membrane;mitochondrion	nucleotide binding;protein binding	5.404	2.047	0.38
Q12029	Probable mitochondrial transport protein fsf1 [OS=Saccharomyces cerevisiae S288c]	854445	FSF1; YOR271C	transport	membrane;mitochondrion	transporter activity	1.637	0.624	0.38
P33311	ATP-dependent permease MDL2, mitochondrial [OS=Saccharomyces cerevisiae S288c]	855858	MDL2; YPL270W	response to stimulus;transport	membrane;mitochondrion	catalytic activity;nucleotide binding;transporter activity	0.802	0.307	0.38
P25374	Cysteine desulfurase, mitochondrial [OS=Saccharomyces cerevisiae S288c]	850343	NFS1; YCL017C	cell organization and biogenesis;cellular homeostasis;metabolic process	mitochondrion;nucleus	catalytic activity;metal ion binding;protein binding	1.346	0.532	0.40
P50945	MICOS complex subunit MIC27 [OS=Saccharomyces cerevisiae S288c]	855623	AIM37; MIC27; YNL100W	cell organization and biogenesis	membrane;mitochondrion		1.254	0.501	0.40
P33416	Heat shock protein 78, mitochondrial [OS=Saccharomyces cerevisiae S288c]	851845	HSP78; YDR258C	cell organization and biogenesis;metabolic process;regulation of biological process;response to stimulus	mitochondrion;organelle lumen	catalytic activity;nucleotide binding;protein binding	2.609	1.049	0.40
P32335	Protein MSS51, mitochondrial [OS=Saccharomyces cerevisiae S288c]	850900	MSS51; YLR203C	metabolic process;regulation of biological process	membrane;mitochondrion	protein binding;translation regulator activity	1.154	0.468	0.41
P01097	ATPase inhibitor, mitochondrial [OS=Saccharomyces cerevisiae S288c]	851347	INH1; YDL181W	metabolic process;regulation of biological process	mitochondrion	catalytic activity;enzyme regulator activity;protein binding	1.154	0.468	0.41
P17505	Malate dehydrogenase, mitochondrial [OS=Saccharomyces cerevisiae S288c]	853777	MDH1; YKL085W	metabolic process	mitochondrion;organelle lumen	catalytic activity;protein binding;RNA	56.797	23.041	0.41

						binding			
P37292	Serine hydroxymethyltransferase, mitochondrial [OS=Saccharomyces cerevisiae S288c]	852565	SHM1; YBR263W	metabolic process	mitochondrion	catalytic activity	5.579	2.275	0.41
P19262	Dihydrolipoyllysine-residue succinyltransferase component of 2-oxoglutarate dehydrogenase complex, mitochondrial [OS=Saccharomyces cerevisiae S288c]	851726	KGD2; YDR148C	cell organization and biogenesis;metabolic process	mitochondrion	catalytic activity;protein binding	5.7	2.325	0.41
Q01163	37S ribosomal protein S23, mitochondrial [OS=Saccharomyces cerevisiae S288c]	852748	RSM23; YGL129C	cell organization and biogenesis;metabolic process	mitochondrion;ribosome	catalytic activity;nucleotide binding;RNA binding;structural molecule activity	1.11	0.453	0.41
Q03798	Altered inheritance of mitochondria protein 36, mitochondrial [OS=Saccharomyces cerevisiae S288c]	855189	AIM36; YMR157C		membrane;mitochondrion		1.069	0.438	0.41
P46367	Potassium-activated aldehyde dehydrogenase, mitochondrial [OS=Saccharomyces cerevisiae S288c]	854556	ALD4; YOR374W	metabolic process	mitochondrion;organelle lumen	catalytic activity	50.795	21.022	0.41
P50087	MICOS subunit MIC26 [OS=Saccharomyces cerevisiae S288c]	853150	MOS2; MIC26; YGR235C	cell organization and biogenesis	membrane;mitochondrion		0.931	0.389	0.42
P32473	Pyruvate dehydrogenase E1 component subunit beta, mitochondrial [OS=Saccharomyces cerevisiae S288c]	852522	PDB1; YBR221C	metabolic process	mitochondrion;organelle lumen	catalytic activity	9	3.833	0.43
P38825	Protein TOM71 [OS=Saccharomyces cerevisiae S288c]	856517	TOM71; YHR117W	transport	membrane;mitochondrion	protein binding;transporter activity	0.805	0.343	0.43
Q06485	Autophagy-related protein 33 [OS=Saccharomyces cerevisiae S288c]	851070	ATG33; YLR356W	cell communication;metabolic process;response to stimulus	membrane;mitochondrion		0.778	0.334	0.43
P39515	mitochondrial import inner membrane translocase subunit Tim17 [OS=Saccharomyces cerevisiae S288c]	853298	TIM17; YJL143W	cell organization and biogenesis;transport	membrane;mitochondrion	protein binding;transporter activity	0.778	0.334	0.43
P53889	uncharacterized mitochondrial hydrolase FMP41 [OS=Saccharomyces cerevisiae S288c]	855553	FMP41; YNL168C	metabolic process	mitochondrion	catalytic activity;metal ion binding	0.719	0.311	0.43
P53220	Mitochondrial import inner membrane translocase subunit TIM21 [OS=Saccharomyces cerevisiae S288c]	852921	TIM21; YGR033C	cell organization and biogenesis;transport	membrane;mitochondrion	protein binding	0.719	0.311	0.43
P17558	37S ribosomal protein PET123, mitochondrial [OS=Saccharomyces cerevisiae S288c]	854329	PET123; YOR158W	cell organization and biogenesis;metabolic process	mitochondrion;ribosome	structural molecule activity	0.668	0.292	0.44
P53732	37S ribosomal protein S12, mitochondrial [OS=Saccharomyces cerevisiae S288c]	855772	MRPS12; YNR036C	cell organization and biogenesis;metabolic process	mitochondrion;ribosome	structural molecule activity	0.668	0.292	0.44
P28817	3-hydroxyisobutyryl-CoA hydrolase, mitochondrial [OS=Saccharomyces cerevisiae S288c]	851606	EHD3; YDR036C	metabolic process	mitochondrion	catalytic activity	0.61	0.269	0.44
P12686	37S ribosomal protein MRP13, mitochondrial [OS=Saccharomyces cerevisiae S288c]	852975	MRP13; YGR084C	cell organization and biogenesis;metabolic process	mitochondrion;ribosome	structural molecule activity	0.585	0.259	0.44
P32453	Protein ATP11, mitochondrial [OS=Saccharomyces cerevisiae S288c]	855401	ATP11; YNL315C	cell organization and biogenesis	mitochondrion	protein binding	0.585	0.259	0.44
P23641	mitochondrial phosphate carrier protein [OS=Saccharomyces cerevisiae S288c]	853540	MIR1; YJR077C	metabolic process;transport	membrane;mitochondrion	protein binding;structural molecule activity;transporter activity	26.826	11.915	0.44
P53881	54S ribosomal protein L22, mitochondrial [OS=Saccharomyces cerevisiae S288c]	855544	MRPL22; YNL177C	cell organization and biogenesis;metabolic process	mitochondrion;ribosome	RNA binding;structural molecule activity	0.551	0.245	0.44
P32860	NifU-like protein, mitochondrial [OS=Saccharomyces cerevisiae S288c]	853826	NFU1; YKL040C	cell organization and biogenesis;cellular homeostasis;metabolic process	mitochondrion;organelle lumen	metal ion binding	1.738	0.778	0.45
P25372	Thioredoxin-3, mitochondrial [OS=Saccharomyces cerevisiae S288c]	850444	TRX3; YCR083W	cellular homeostasis;metabolic process;regulation of biological process;response to stimulus;transport	cytoplasm;mitochondrion	catalytic activity	0.52	0.233	0.45
P22136	ATPase expression protein 2, mitochondrial [OS=Saccharomyces cerevisiae S288c]	855325	AEP2; YMR282C	metabolic process;regulation of biological process	mitochondrion	RNA binding	0.506	0.227	0.45
P40341	Mitochondrial respiratory chain complexes assembly protein YTA12	855114	YTA12; YMR08	cell organization and biogenesis;metabolic	cytoplasm;membrane;mitochondrion	catalytic activity;metal ion	1.575	0.707	0.45

	[OS=Saccharomyces cerevisiae S288c]		9C	process;response to stimulus;transport		binding;nucleotide binding;protein binding			
P35191	DnaJ homolog 1, mitochondrial [OS=Saccharomyces cerevisiae S288c]	850530	MDJ1; YFL016C	cell organization and biogenesis;metabolic process;regulation of biological process;response to stimulus	mitochondrion;organelle lumen	enzyme regulator activity;metal ion binding;nucleotide binding;protein binding	1.656	0.748	0.45
P40416	Iron-sulfur clusters transporter ATM1, mitochondrial [OS=Saccharomyces cerevisiae S288c]	855347	ATM1; YMR301C	cellular homeostasis;metabolic process;transport	membrane;mitochondrion	catalytic activity;nucleotide binding;transporter activity	0.453	0.205	0.45
P33310	ATP-dependent permease MDL1, mitochondrial [OS=Saccharomyces cerevisiae S288c]	850885	MDL1; YLR188W	transport	membrane;mitochondrion	catalytic activity;nucleotide binding;transporter activity	0.438	0.199	0.45
Q03327	Mitochondrial fusion and transport protein Ugo1 [OS=Saccharomyces cerevisiae S288c]	852081	UGO1; YDR470C	cell organization and biogenesis;metabolic process	membrane;mitochondrion	protein binding;structural molecule activity	0.425	0.194	0.46
P53230	Phosphatidate cytidylyltransferase, mitochondrial [OS=Saccharomyces cerevisiae S288c]	852937	TAM41; YGR046W	metabolic process	membrane;mitochondrion;organelle lumen	catalytic activity	0.407	0.186	0.46
P40053	Altered inheritance of mitochondria protein 9, mitochondrial [OS=Saccharomyces cerevisiae S288c]	856813	AIM9; YER080W		mitochondrion		1.938	0.891	0.46
Q03246	37S ribosomal protein S17, mitochondrial [OS=Saccharomyces cerevisiae S288c]	855226	MRPS17; YMR188C	cell differentiation;cell organization and biogenesis;metabolic process	mitochondrion;ribosome	RNA binding;structural molecule activity	0.389	0.179	0.46
P25039	Elongation factor G, mitochondrial [OS=Saccharomyces cerevisiae S288c]	850758	MEF1; YLR069C	cell organization and biogenesis;metabolic process	cytosol;mitochondrion	catalytic activity;nucleotide binding;RNA binding	0.326	0.151	0.46
P41921	glutathione reductase [OS=Saccharomyces cerevisiae S288c]	856014	GLR1; YPL091W	cellular homeostasis;metabolic process;regulation of biological process;response to stimulus	cytoplasm;cytosol;mitochondrion;nucleus	antioxidant activity;catalytic activity;nucleotide binding	0.334	0.155	0.46
Q04472	Mitochondrial inner membrane i-AAA protease supercomplex subunit MGR3 [OS=Saccharomyces cerevisiae S288c]	855142	MGR3; YMR115W	metabolic process	membrane;mitochondrion	protein binding	0.322	0.15	0.47
P05626	ATP synthase subunit 4, mitochondrial [OS=Saccharomyces cerevisiae S288c]	856027	ATP4; YPL078C	cell organization and biogenesis;metabolic process;transport	membrane;mitochondrion	catalytic activity;transporter activity	7.799	3.642	0.47
P38910	10 kDa heat shock protein, mitochondrial [OS=Saccharomyces cerevisiae S288c]	854185	HSP10; YOR020C	cell organization and biogenesis;metabolic process;response to stimulus;transport	cytoplasm;mitochondrion;organelle lumen	metal ion binding;nucleotide binding;protein binding	4.623	2.162	0.47
P21306	ATP synthase subunit epsilon, mitochondrial [OS=Saccharomyces cerevisiae S288c]	855857	ATP15; YPL271W	metabolic process;transport	membrane;mitochondrion	catalytic activity;transporter activity	4.623	2.162	0.47
P48015	Aminomethyltransferase, mitochondrial [OS=Saccharomyces cerevisiae S288c]	851582	GCV1; YDR019C	metabolic process	mitochondrion	catalytic activity;protein binding	5.7	2.675	0.47
P12687	54S ribosomal protein L2, mitochondrial [OS=Saccharomyces cerevisiae S288c]	855727	MRP7; YNL005C	cell organization and biogenesis;metabolic process	mitochondrion;ribosome	catalytic activity;RNA binding;structural molecule activity	0.233	0.11	0.47
Q05648	MIOREX complex component 10 [OS=Saccharomyces cerevisiae S288c]	851876	MRX10; YDR282C		membrane;mitochondrion		0.222	0.105	0.47
P36163	mitochondrial metalloendopeptidase OMA1 [OS=Saccharomyces cerevisiae S288c]	853962	OMA1; YKR087C	metabolic process	membrane;mitochondrion	catalytic activity;metal ion binding	0.245	0.116	0.47
P50110	Sorting assembly machinery 37 kDa subunit [OS=Saccharomyces cerevisiae S288c]	855082	SAM37; YMR060C	cell organization and biogenesis;transport	membrane;mitochondrion	protein binding	0.245	0.116	0.47
P40047	Aldehyde dehydrogenase 5, mitochondrial [OS=Saccharomyces cerevisiae S288c]	856804	ALD5; YER073W	metabolic process	mitochondrion;organelle lumen	catalytic activity	1.762	0.84	0.48
Q06143	mitochondrial dicarboxylate transporter [OS=Saccharomyces cerevisiae S288c]	851063	DIC1; YLR348C	metabolic process;transport	membrane;mitochondrion	structural molecule activity;transporter activity	1.154	0.551	0.48
P25605	Acetolactate synthase small subunit, mitochondrial [OS=Saccharomyces cerevisiae S288c]	850348	ILV6; YCL009C	metabolic process;regulation of biological process	mitochondrion	catalytic activity;enzyme regulator activity	2.793	1.336	0.48
P32839	Mitochondrial chaperone BCS1 [OS=Saccharomyces cerevisiae S288c]	851981	BCS1; YDR375C	cell organization and biogenesis;metabolic process;response to stimulus;transport	cytosol;membrane;mitochondrion	catalytic activity;nucleotide binding;transporter activity	0.194	0.093	0.48
P38909	cytochrome c mitochondrial import factor CYC2 [OS=Saccharomyces cerevisiae S288c]	854202	CYC2; YOR037W	cell organization and biogenesis;metabolic process	membrane;mitochondrion	catalytic activity	0.194	0.093	0.48

P03879	Intron-encoded RNA maturase bI4 [OS=Saccharomyces cerevisiae S288c]	854582	BI4; Q0120	metabolic process	membrane;mitochondrion	catalytic activity;RNA binding	0.179	0.086	0.48
Q07500	External NADH-ubiquinone oxidoreductase 2, mitochondrial [OS=Saccharomyces cerevisiae S288c]	851474	NDE2; YDL085W	metabolic process	mitochondrion	catalytic activity;nucleotide binding	4.012	1.929	0.48
P25345	Asparagine--tRNA ligase, mitochondrial [OS=Saccharomyces cerevisiae S288c]	850388	SLM5; YCR024C	metabolic process	cytoplasm;mitochondrion;organelle lumen	catalytic activity;nucleotide binding	0.172	0.083	0.48
Q07349	MIOREX complex component 9 [OS=Saccharomyces cerevisiae S288c]	851535	YDL027C; MRX9; YDL027C		endoplasmic reticulum;membrane;mitochondrion		1.913	0.931	0.49
P40215	External NADH-ubiquinone oxidoreductase 1, mitochondrial [OS=Saccharomyces cerevisiae S288c]	855176	NDE1; YMR145C	metabolic process	mitochondrion	catalytic activity;nucleotide binding	5.078	2.472	0.49
P38988	Mitochondrial GTP/GDP carrier protein 1 [OS=Saccharomyces cerevisiae S288c]	851329	GGC1; YDL198C	cell organization and biogenesis;cellular homeostasis;metabolic process;transport	membrane;mitochondrion	structural molecule activity;transporter activity	4.78	2.34	0.49
P09457	ATP synthase subunit 5, mitochondrial [OS=Saccharomyces cerevisiae S288c]	851892	ATP5; YDR298C	metabolic process;transport	membrane;mitochondrion	catalytic activity;transporter activity	13.678	6.743	0.49
P53598	Succinyl-CoA ligase [ADP-forming] subunit alpha, mitochondrial [OS=Saccharomyces cerevisiae S288c]	854310	LSC1; YOR142W	metabolic process	cytosol;mitochondrion	catalytic activity;nucleotide binding	10.288	5.158	0.50
Q08822	Probable electron transfer flavoprotein-ubiquinone oxidoreductase, mitochondrial [OS=Saccharomyces cerevisiae S288c]	854538	CIR2; YOR356W	metabolic process;transport	membrane;mitochondrion	catalytic activity;metal ion binding;nucleotide binding	2.481	1.26	0.51
P06168	ketol-acid reductoisomerase, mitochondrial [OS=Saccharomyces cerevisiae S288c]	851069	ILV5; YLR355C	cell organization and biogenesis;metabolic process	mitochondrion	catalytic activity;DNA binding;metal ion binding	9.857	5.105	0.52
Q03028	Mitochondrial 2-oxodicarboxylate carrier 1 [OS=Saccharomyces cerevisiae S288c]	855969	ODC1; YPL134C	metabolic process;transport	membrane;mitochondrion	structural molecule activity;transporter activity	4.995	2.594	0.52
Q07651	SUR7 family protein FMP45 [OS=Saccharomyces cerevisiae S288c]	851304	FMP45; YDL222C	cell differentiation;cell organization and biogenesis	membrane;mitochondrion		6.017	3.125	0.52
P07257	Cytochrome b-c1 complex subunit 2, mitochondrial [OS=Saccharomyces cerevisiae S288c]	856321	QCR2; YPR191W	metabolic process;transport	membrane;mitochondrion	catalytic activity;metal ion binding;protein binding;transporter activity	33.551	17.588	0.52
P40035	Mitochondrial phosphate carrier protein 2 [OS=Saccharomyces cerevisiae S288c]	856779	PIC2; YER053C	cellular homeostasis;metabolic process;transport	membrane;mitochondrion	structural molecule activity;transporter activity	0.833	0.438	0.53
P00044	Cytochrome c iso-1 [OS=Saccharomyces cerevisiae S288c]	853507	CYC1; YJR048W	metabolic process;transport	mitochondrion	metal ion binding;protein binding	12.335	6.499	0.53
P07342	Acetolactate synthase catalytic subunit, mitochondrial [OS=Saccharomyces cerevisiae S288c]	855135	ILV2; YMR108W	metabolic process	mitochondrion	catalytic activity;metal ion binding;nucleotide binding	4.223	2.257	0.53
P25573	Mitochondrial inner membrane i-AAA protease supercomplex subunit MGR1 [OS=Saccharomyces cerevisiae S288c]	850313	MGR1; YCL044C	metabolic process	membrane;mitochondrion	protein binding	1.031	0.557	0.54
P08466	mitochondrial nuclease [OS=Saccharomyces cerevisiae S288c]	853222	NUC1; YJL208C	cell death;metabolic process	membrane;mitochondrion;nucleus	catalytic activity;metal ion binding	1.976	1.069	0.54
P08067	cytochrome b-c1 complex subunit Rieske, mitochondrial [OS=Saccharomyces cerevisiae S288c]	856689	RIP1; YEL024W	metabolic process;transport	membrane;mitochondrion	catalytic activity;metal ion binding;transporter activity	25.102	13.678	0.54
Q06493	LETM1 domain-containing protein YLH47, mitochondrial [OS=Saccharomyces cerevisiae S288c]	856243	YLH47; YPR125W	cell organization and biogenesis;transport	membrane;mitochondrion		0.487	0.269	0.55
Q96VH5	MICOS complex subunit Mic10 [OS=Saccharomyces cerevisiae S288c]	850300	MOS1; MIC10; YCL057C-A	cell organization and biogenesis	membrane;mitochondrion		1.683	0.931	0.55
P39925	Mitochondrial respiratory chain complexes assembly protein AFG3 [OS=Saccharomyces cerevisiae S288c]	856737	AFG3; YER017C	cell organization and biogenesis;metabolic process;response to stimulus;transport	membrane;mitochondrion	catalytic activity;metal ion binding;nucleotide binding;protein binding	1.015	0.569	0.56
Q06405	ATP synthase subunit f, mitochondrial [OS=Saccharomyces cerevisiae S288c]	851983	ATP17; YDR377W	metabolic process;transport	membrane;mitochondrion	catalytic activity;transporter activity	5.31	2.981	0.56

P04710	ADP,ATP carrier protein 1 [OS=Saccharomyces cerevisiae S288c]	855078	AAC1; YMR056C	metabolic process;transport	cytosol;membrane;mitochondrion	structural molecule activity;transporter activity	7.859	4.456	0.57
Q12233	ATP synthase subunit g, mitochondrial [OS=Saccharomyces cerevisiae S288c]	856131	ATP20; YPR020W	cell organization and biogenesis;metabolic process;transport	membrane;mitochondrion	catalytic activity;transporter activity	1.371	0.778	0.57
P36147	Presequence translocated-associated motor subunit pam17, mitochondrial [OS=Saccharomyces cerevisiae S288c]	853939	PAM17; YKR065C	transport	membrane;mitochondrion		1.371	0.778	0.57
P33303	Succinate/fumarate mitochondrial transporter [OS=Saccharomyces cerevisiae S288c]	853558	SFC1; YJR095W	metabolic process;transport	membrane;mitochondrion	structural molecule activity;transporter activity	15.681	9	0.57
P00830	ATP synthase subunit beta, mitochondrial [OS=Saccharomyces cerevisiae S288c]	853585	ATP2; YJR121W	metabolic process;transport	cytosol;membrane;mitochondrion	catalytic activity;nucleotide binding;protein binding;transporter activity	41.987	24.119	0.57
Q03713	Respiratory supercomplex factor 1, mitochondrial [OS=Saccharomyces cerevisiae S288c]	854978	RCF1; YML030W	cell organization and biogenesis	membrane;mitochondrion		0.995	0.585	0.59
P00890	citrate synthase, mitochondrial [OS=Saccharomyces cerevisiae S288c]	855732	CIT1; YNR001C	metabolic process;transport	cytosol;mitochondrion;organelle lumen	catalytic activity	21.758	12.895	0.59
P40008	Protein FMP52, mitochondrial [OS=Saccharomyces cerevisiae S288c]	856721	FMP52; YER004W		endoplasmic reticulum;membrane;mitochondrion	catalytic activity	1.738	1.054	0.61
P53721	Respiratory supercomplex factor 2, mitochondrial [OS=Saccharomyces cerevisiae S288c]	855752	RCF2; YNR018W	cell organization and biogenesis	membrane;mitochondrion		7.111	4.337	0.61
P07253	cytochrome B pre-mRNA-processing protein 6 [OS=Saccharomyces cerevisiae S288c]	852417	CBP6; YBR120C	cell organization and biogenesis;metabolic process;regulation of biological process	mitochondrion;ribosome		2.728	1.683	0.62
P47045	mitochondrial import inner membrane translocase subunit TIM54 [OS=Saccharomyces cerevisiae S288c]	853392	TIM54; YJL054W	cell organization and biogenesis;transport	cytosol;membrane;mitochondrion	transporter activity	0.54	0.334	0.62
P36534	54S ribosomal protein L40, mitochondrial [OS=Saccharomyces cerevisiae S288c]	855930	MRPL40; YPL173W	cell organization and biogenesis;metabolic process	mitochondrion;ribosome	protein binding;structural molecule activity	0.413	0.259	0.63
P33759	37S ribosomal protein S5, mitochondrial [OS=Saccharomyces cerevisiae S288c]	852553	MRPS5; YBR251W	cell organization and biogenesis;metabolic process	mitochondrion;ribosome	RNA binding;structural molecule activity	0.413	0.259	0.63
Q08179	Mitochondrial distribution and morphology protein 38 [OS=Saccharomyces cerevisiae S288c]	854130	MDM38; YOL027C	cell organization and biogenesis;cellular homeostasis;regulation of biological process;transport	membrane;mitochondrion		3.732	2.35	0.63
P38152	Tricarboxylate transport protein [OS=Saccharomyces cerevisiae S288c]	852594	CTP1; YBR291C	metabolic process;transport	membrane;mitochondrion	structural molecule activity;transporter activity	0.389	0.245	0.63
P39525	3-oxoacyl-[acyl-carrier-protein] synthase homolog [OS=Saccharomyces cerevisiae S288c]	856790	CEM1; YER061C	metabolic process	mitochondrion	catalytic activity	0.369	0.233	0.63
P38797	Protein phosphatase 2C homolog 7, mitochondrial [OS=Saccharomyces cerevisiae S288c]	856475	PTC7; YHR076W	metabolic process	mitochondrion	catalytic activity;metal ion binding;protein binding	0.369	0.233	0.63
P40012	protoporphyrinogen oxidase [OS=Saccharomyces cerevisiae S288c]	856733	HEM14; YER014W	metabolic process	membrane;mitochondrion	catalytic activity	0.833	0.528	0.63
P36101	tRNA threonylcarbamoyladenosine dehydratase 2 [OS=Saccharomyces cerevisiae S288c]	853841	TCD2; YKL027W; YKL027W	metabolic process	cytosol;membrane;mitochondrion	catalytic activity;nucleotide binding	0.334	0.212	0.63
Q04013	Citrate/oxoglutarate carrier protein [OS=Saccharomyces cerevisiae S288c]	855282	YHM2; YMR241W	cell organization and biogenesis;metabolic process;transport	membrane;mitochondrion	DNA binding;structural molecule activity;transporter activity	4.012	2.548	0.64
P40961	prohibitin-1 [OS=Saccharomyces cerevisiae S288c]	853033	PHB1; YGR132C	cell organization and biogenesis;metabolic process;regulation of biological process	membrane;mitochondrion	protein binding	5.158	3.281	0.64
P38077	ATP synthase subunit gamma, mitochondrial [OS=Saccharomyces cerevisiae S288c]	852327	ATP3; YBR039W	metabolic process;transport	membrane;mitochondrion	catalytic activity;transporter activity	15.876	10.103	0.64
Q06510	Lysophosphatidylcholine acyltransferase [OS=Saccharomyces cerevisiae S288c]	856262	TAZ1; YPR140W	cell organization and biogenesis;metabolic process;transport	membrane;mitochondrion	catalytic activity	0.304	0.194	0.64
Q01574	acetyl-coenzyme A synthetase 1 [OS=Saccharomyces cerevisiae S288c]	851245	ACS1; YAL054C	cell organization and biogenesis;metabolic process	cytoplasm;cytosol;endoplasmic reticulum;mitochondrion;nucleus	catalytic activity;nucleotide binding	1.482	0.947	0.64

P36516	54S ribosomal protein L3, mitochondrial [OS=Saccharomyces cerevisiae S288c]	855039	MRPL3; YMR024W	cell organization and biogenesis;metabolic process;response to stimulus	mitochondrion;nucleus;ribosome	catalytic activity;RNA binding;structural molecule activity	0.269	0.172	0.64
P25349	Flavoprotein-like protein YCP4 [OS=Saccharomyces cerevisiae S288c]	850360	YCP4; YCR004C	metabolic process;regulation of biological process	cytoplasm;membrane;mitochondrion	catalytic activity;nucleotide binding;protein binding	7.799	4.995	0.64
Q12166	2-isopropylmalate synthase 2, mitochondrial [OS=Saccharomyces cerevisiae S288c]	854275	LEU9; YOR108W	metabolic process	mitochondrion	catalytic activity;protein binding	1.707	1.11	0.65
P36046	Mitochondrial intermembrane space import and assembly protein 40 [OS=Saccharomyces cerevisiae S288c]	853639	MIA40; YKL195W	cellular component movement;metabolic process;response to stimulus;transport	membrane;mitochondrion	catalytic activity;protein binding;transporter activity	0.817	0.532	0.65
Q02776	Mitochondrial import inner membrane translocase subunit TIM50 [OS=Saccharomyces cerevisiae S288c]	856042	TIM50; YPL063W	cell organization and biogenesis;metabolic process;transport	membrane;mitochondrion	catalytic activity;protein binding;transporter activity	4.179	2.728	0.65
P40098	uncharacterized mitochondrial membrane protein FMP10 [OS=Saccharomyces cerevisiae S288c]	856931	FMP10; YER182W		membrane;mitochondrion		1.637	1.069	0.65
P18239	ADP,ATP carrier protein 2 [OS=Saccharomyces cerevisiae S288c]	852250	PET9; YBL030C	cell death;metabolic process;transport	membrane;mitochondrion	structural molecule activity;transporter activity	122.285	80.113	0.66
P32795	Mitochondrial inner membrane i-AAA protease supercomplex subunit YME1 [OS=Saccharomyces cerevisiae S288c]	856135	YME1; YPR024W	metabolic process;response to stimulus;transport	membrane;mitochondrion	catalytic activity;metal ion binding;nucleotide binding;protein binding	1.085	0.714	0.66
Q12480	Probable electron transfer flavoprotein subunit alpha, mitochondrial [OS=Saccharomyces cerevisiae S288c]	856112	AIM45; YPR004C	metabolic process;transport	mitochondrion;organelle lumen	catalytic activity;nucleotide binding	1.512	0.995	0.66
P39518	Long-chain-fatty-acid--CoA ligase 2 [OS=Saccharomyces cerevisiae S288c]	856734	FAA2; YER015W	metabolic process;transport	cytoplasm;mitochondrion	catalytic activity;nucleotide binding	0.802	0.535	0.67
P53163	54S ribosomal protein L12, mitochondrial [OS=Saccharomyces cerevisiae S288c]	852811	MNP1; YGL068W	cell organization and biogenesis;metabolic process	mitochondrion;ribosome	RNA binding;structural molecule activity	1.31	0.874	0.67
Q12374	Nuclear control of ATPase protein 2 [OS=Saccharomyces cerevisiae S288c]	856278	NCA2; YPR155C	metabolic process	membrane;mitochondrion		0.565	0.377	0.67
P32332	Mitochondrial oxaloacetate transport protein [OS=Saccharomyces cerevisiae S288c]	853739	OAC1; YKL120W	metabolic process;transport	membrane;mitochondrion	structural molecule activity;transporter activity	2.162	1.448	0.67
Q06236	Mitochondrial inner membrane protein SHH4 [OS=Saccharomyces cerevisiae S288c]	850861	SHH4; YLR164W	metabolic process	membrane;mitochondrion	catalytic activity;metal ion binding	3.217	2.162	0.67
P21560	Protein CBP3, mitochondrial [OS=Saccharomyces cerevisiae S288c]	855886	CBP3; YPL215W	cell organization and biogenesis;regulation of biological process	membrane;mitochondrion;ribosome		1.031	0.701	0.68
P37298	Succinate dehydrogenase [ubiquinone] cytochrome b small subunit, mitochondrial [OS=Saccharomyces cerevisiae S288c]	851758	SDH4; YDR178W	metabolic process;transport	membrane;mitochondrion	catalytic activity;metal ion binding	2.594	1.783	0.69
P32904	54S ribosomal protein L6, mitochondrial [OS=Saccharomyces cerevisiae S288c]	856552	MRPL6; YHR147C	cell organization and biogenesis;metabolic process	mitochondrion;ribosome	RNA binding;structural molecule activity	0.778	0.54	0.69
P23833	Protein SCO1, mitochondrial [OS=Saccharomyces cerevisiae S288c]	852325	SCO1; YBR037C	cell organization and biogenesis;cellular homeostasis;response to stimulus;transport	membrane;mitochondrion	antioxidant activity;catalytic activity;metal ion binding	0.668	0.468	0.70
P53266	Cytochrome oxidase assembly protein SHY1 [OS=Saccharomyces cerevisiae S288c]	853009	SHY1; YGR112W	cell organization and biogenesis	membrane;mitochondrion	protein binding	1.154	0.817	0.71
Q12428	Probable 2-methylcitrate dehydratase [OS=Saccharomyces cerevisiae S288c]	856108	PDH1; YPR002W	metabolic process	cytoplasm;membrane;mitochondrion	catalytic activity	7.058	5.043	0.71
P36066	Protein MRG3-like [OS=Saccharomyces cerevisiae S288c]	853725	YKL133C; YKL133C	metabolic process	membrane;mitochondrion	protein binding	0.334	0.241	0.72
P53326	Uncharacterized protein YGR266W [OS=Saccharomyces cerevisiae S288c]	853182	YGR266W; YGR266W		membrane;mitochondrion		0.602	0.442	0.73
P54003	Protein SUR7 [OS=Saccharomyces cerevisiae S288c]	854953	SUR7; YML052W	cell differentiation;transport	membrane;mitochondrion		1.154	0.848	0.73
Q08023	Protein FMP25, mitochondrial [OS=Saccharomyces cerevisiae S288c]	850766	FMP25; YLR077W	cell organization and biogenesis	membrane;mitochondrion		0.968	0.719	0.74
P21801	Succinate dehydrogenase [ubiquinone] iron-sulfur subunit, mitochondrial [OS=Saccharomyces	850685	SDH2; YLL041C	metabolic process;transport	membrane;mitochondrion	catalytic activity;metal ion binding	18.684	14.013	0.75

	cerevisiae S288c]								
P40185	Protein mmf1, mitochondrial [OS=Saccharomyces cerevisiae S288c]	854760	MMF1; YIL051C	cell organization and biogenesis;metabolic process	mitochondrion;organelle lumen		5.31	4.012	0.76
P32799	Cytochrome c oxidase subunit 6A, mitochondrial [OS=Saccharomyces cerevisiae S288c]	852684	COX13; YGL191W	cell organization and biogenesis;metabolic process;regulation of biological process;transport	membrane;mitochondrion	catalytic activity;enzyme regulator activity;transporter activity	5.31	4.012	0.76
P50085	Prohibitin-2 [OS=Saccharomyces cerevisiae S288c]	853146	PHB2; YGR231C	cell organization and biogenesis;metabolic process;regulation of biological process	membrane;mitochondrion	protein binding	4.337	3.329	0.77
Q08968	UPF0061 protein FMP40 [OS=Saccharomyces cerevisiae S288c]	855879	FMP40; YPL222W		mitochondrion	protein binding	0.917	0.823	0.90
P07256	Cytochrome b-c1 complex subunit 1, mitochondrial [OS=Saccharomyces cerevisiae S288c]	852235	COR1; YBL045C	metabolic process;transport	membrane;mitochondrion	catalytic activity;metal ion binding;protein binding;transporter activity	9.89	9.89	1.00
P50088	Stationary phase gene 1 protein [OS=Saccharomyces cerevisiae S288c]	853151	SPG1; YGR236C		endoplasmic reticulum;membrane;mitochondrion		157.489	157.489	1.00
P00410	Cytochrome c oxidase subunit 2 [OS=Saccharomyces cerevisiae S288c]	854622	COX2; Q0250	metabolic process;transport	membrane;mitochondrion	catalytic activity;metal ion binding;protein binding;transporter activity	2.831	2.831	1.00
P38325	Mitochondrial outer membrane protein OM14 [OS=Saccharomyces cerevisiae S288c]	852531	OM14; YBR230C	transport	membrane;mitochondrion		9	9	1.00
P40508	Uncharacterized protein YIL077C [OS=Saccharomyces cerevisiae S288c]	854733	YIL077C; YIL077C		mitochondrion		0.668	0.668	1.00
P38072	Protein SCO2, mitochondrial [OS=Saccharomyces cerevisiae S288c]	852312	SCO2; YBR024W	cell organization and biogenesis;cellular homeostasis;metabolic process;transport	membrane;mitochondrion	antioxidant activity;catalytic activity;metal ion binding	0.73	0.73	1.00
Q06892	NADH kinase POS5, mitochondrial [OS=Saccharomyces cerevisiae S288c]	855913	POS5; YPL188W	metabolic process;response to stimulus	mitochondrion;organelle lumen	catalytic activity;nucleotide binding	0.369	0.369	1.00
P38127	mitochondrial carrier protein RIM2 [OS=Saccharomyces cerevisiae S288c]	852491	RIM2; YBR192W	cell organization and biogenesis;metabolic process;transport	membrane;mitochondrion	structural molecule activity;transporter activity	0.585	0.585	1.00
P32445	Single-stranded DNA-binding protein RIM1, mitochondrial [OS=Saccharomyces cerevisiae S288c]	850395	RIM1; YCR028C-A	cell organization and biogenesis;metabolic process;regulation of biological process	mitochondrion	DNA binding	1.154	1.154	1.00
P32331	Carrier protein YMC1, mitochondrial [OS=Saccharomyces cerevisiae S288c]	856171	YMC1; YPR058W	metabolic process;transport	membrane;mitochondrion;vacuole	structural molecule activity;transporter activity	0.501	0.501	1.00
Q12165	ATP synthase subunit delta, mitochondrial [OS=Saccharomyces cerevisiae S288c]	851560	ATP16; YDL004W	metabolic process;transport	membrane;mitochondrion	catalytic activity;transporter activity	1.371	1.371	1.00
P33421	Succinate dehydrogenase [ubiquinone] cytochrome b subunit, mitochondrial [OS=Saccharomyces cerevisiae S288c]	853716	SDH3; YKL141W	cell organization and biogenesis;metabolic process;transport	membrane;mitochondrion	catalytic activity;metal ion binding	0.931	0.931	1.00
Q06668	Methyltransferase OMS1, mitochondrial [OS=Saccharomyces cerevisiae S288c]	851911	OMS1; YDR316W	metabolic process	membrane;mitochondrion	catalytic activity	0.301	0.301	1.00
P17695	Glutaredoxin-2, mitochondrial [OS=Saccharomyces cerevisiae S288c]	852124	GRX2; YDR513W	cellular homeostasis;metabolic process;regulation of biological process;response to stimulus;transport	cytoplasm;cytosol;mitochondrion;nucleus	antioxidant activity;catalytic activity	0.52	0.52	1.00
P34224	Uncharacterized protein YBL059W [OS=Saccharomyces cerevisiae S288c]	852221	YBL059W; YBL059W		membrane;mitochondrion		0.468	0.468	1.00
Q99297	Mitochondrial 2-oxodicarboxylate carrier 2 [OS=Saccharomyces cerevisiae S288c]	854397	ODC2; YOR222W	metabolic process;transport	membrane;mitochondrion	structural molecule activity;transporter activity	0.54	0.54	1.00
Q08223	Altered inheritance of mitochondria protein 39, mitochondrial [OS=Saccharomyces cerevisiae S288c]	854103	AIM39; YOL053W		cytosol;membrane;mitochondrion		0.259	0.259	1.00
P32266	Dynamin-like GTPase MGM1, mitochondrial [OS=Saccharomyces cerevisiae S288c]	854386	MGM1; YOR211C	cell organization and biogenesis	membrane;mitochondrion	catalytic activity;nucleotide binding;protein binding	0.086	0.086	1.00
P19516	Cytochrome c oxidase assembly protein COX11, mitochondrial [OS=Saccharomyces cerevisiae S288c]	855971	COX11; YPL132W	cell organization and biogenesis;metabolic process	membrane;mitochondrion;ribosome	metal ion binding	0.274	0.274	1.00

P18496	Mitochondrial ATPase complex subunit ATP10 [OS=Saccharomyces cerevisiae S288c]	851109	ATP10; YLR393W	cell organization and biogenesis	cytoplasm;membrane;mitochondrion	protein binding	0.413	0.413	1.00
P28737	Protein MSP1 [OS=Saccharomyces cerevisiae S288c]	852915	MSP1; YGR028W	cell organization and biogenesis;metabolic process;regulation of biological process;response to stimulus;transport	membrane;mitochondrion;nucleus	catalytic activity;nucleotide binding;protein binding;RNA binding	0.413	0.413	1.00
P00127	Cytochrome b-c1 complex subunit 6 [OS=Saccharomyces cerevisiae S288c]	850593	QCR6; YFR033C	metabolic process;transport	membrane;mitochondrion	catalytic activity;transporter activity	0.334	0.334	1.00
P43594	MICOS complex subunit MIC19 [OS=Saccharomyces cerevisiae S288c]	850563	AIM13; MIC19; YFR011C	cell organization and biogenesis	cytoplasm;membrane;mitochondrion		0.52	0.52	1.00
P36528	54S ribosomal protein L17, mitochondrial [OS=Saccharomyces cerevisiae S288c]	855469	MRPL17; YNL252C	cell organization and biogenesis;metabolic process	mitochondrion;ribosome	structural molecule activity	0.11	0.11	1.00
P81450	ATP synthase subunit J, mitochondrial [OS=Saccharomyces cerevisiae S288c]	854893	ATP18; YML081C-A	cell organization and biogenesis;metabolic process;transport	membrane;mitochondrion	catalytic activity;transporter activity	2.162	2.162	1.00
Q3E6R5	uncharacterized mitochondrial outer membrane protein YDR381C-A [OS=Saccharomyces cerevisiae S288c]	851989	YDR381C-A; YDR381C-A		membrane;mitochondrion		0.389	0.389	1.00
P32463	Acyl carrier protein, mitochondrial [OS=Saccharomyces cerevisiae S288c]	853642	ACP1; YKL192C	metabolic process;transport	mitochondrion		0.259	0.259	1.00
P54857	lipase 2 [OS=Saccharomyces cerevisiae S288c]	851628	TGL2; YDR058C	metabolic process;transport	mitochondrion	catalytic activity	0.233	0.233	1.00
Q05779	Ubiquinone biosynthesis protein COQ9, mitochondrial [OS=Saccharomyces cerevisiae S288c]	850898	COQ9; YLR201C	metabolic process	membrane;mitochondrion		0.166	0.166	1.00
P47150	37S ribosomal protein S7, mitochondrial [OS=Saccharomyces cerevisiae S288c]	853578	RSM7; YJR113C	cell organization and biogenesis;metabolic process	mitochondrion;ribosome	RNA binding;structural molecule activity	0.101	0.101	1.00
P87275	Altered inheritance of mitochondria protein 11 [OS=Saccharomyces cerevisiae S288c]	856829	AIM11; YER093C-A		membrane;mitochondrion		0.389	0.389	1.00
P22289	cytochrome b-c1 complex subunit 9 [OS=Saccharomyces cerevisiae S288c]	853095	QCR9; YGR183C	cell organization and biogenesis;metabolic process;transport	membrane;mitochondrion	catalytic activity;transporter activity	1.154	1.154	1.00
P25038	Translation initiation factor IF-2, mitochondrial [OS=Saccharomyces cerevisiae S288c]	854135	IFM1; YOL023W	cell organization and biogenesis;metabolic process	mitochondrion;nucleus	catalytic activity;nucleotide binding;RNA binding	0.054	0.054	1.00
Q06630	Mitochondrial homologous recombination protein 1 [OS=Saccharomyces cerevisiae S288c]	851890	MHR1; YDR296W	cell organization and biogenesis;metabolic process;regulation of biological process;response to stimulus	mitochondrion;nucleus	catalytic activity;DNA binding;structural molecule activity	0.166	0.166	1.00
P00045	Cytochrome c iso-2 [OS=Saccharomyces cerevisiae S288c]	856672	CYC7; YEL039C	metabolic process;transport	mitochondrion	metal ion binding	0.259	0.259	1.00
P04039	Cytochrome c oxidase polypeptide VIII, mitochondrial [OS=Saccharomyces cerevisiae S288c]	851111	COX8; YLR395C	metabolic process;transport	membrane;mitochondrion	catalytic activity;transporter activity	0.778	0.778	1.00
P40581	peroxiredoxin HYR1 [OS=Saccharomyces cerevisiae S288c]	854855	HYR1; YIR037W	metabolic process;response to stimulus	cytoplasm;cytosol;mitochondrion;organelle lumen	antioxidant activity;catalytic activity	0.259	0.259	1.00
P07236	Threonine--tRNA ligase, mitochondrial [OS=Saccharomyces cerevisiae S288c]	853640	MST1; YKL194C	metabolic process	cytoplasm;mitochondrion;organelle lumen	catalytic activity;nucleotide binding;RNA binding	0.072	0.072	1.00
P40086	Cytochrome c oxidase assembly protein cox15 [OS=Saccharomyces cerevisiae S288c]	856884	COX15; YER141W	cell organization and biogenesis;metabolic process;transport	membrane;mitochondrion	catalytic activity;transporter activity	0.668	0.817	1.22
Q12289	mitochondrial carnitine carrier [OS=Saccharomyces cerevisiae S288c]	854267	CRC1; YOR100C	metabolic process;transport	membrane;mitochondrion	structural molecule activity;transporter activity	0.931	1.154	1.24
P38523	GrpE protein homolog, mitochondrial [OS=Saccharomyces cerevisiae S288c]	854407	MGE1; YOR232W	metabolic process;regulation of biological process;transport	membrane;mitochondrion;organelle lumen	enzyme regulator activity;nucleotide binding;protein binding	0.624	0.833	1.33
Q04935	Cytochrome c oxidase protein 20, mitochondrial [OS=Saccharomyces cerevisiae S288c]	851817	COX20; YDR231C	cell organization and biogenesis;metabolic process	membrane;mitochondrion	protein binding	1.031	1.424	1.38
P27697	Atypical kinase COQ8, mitochondrial [OS=Saccharomyces cerevisiae S288c]	852758	COQ8; YGL119W	metabolic process	membrane;mitochondrion;organelle lumen	catalytic activity;nucleotide binding	0.133	0.205	1.54
P40496	37S ribosomal protein S25, mitochondrial [OS=Saccharomyces cerevisiae S288c]	854715	RSM25; YIL093C	cell organization and biogenesis;metabolic process	mitochondrion;ribosome	structural molecule activity	0.292	0.468	1.60
Q06089	Uncharacterized mitochondrial outer membrane protein YPR098C [OS=Saccharomyces cerevisiae	856213	YPR098C; YPR098		membrane;mitochondrion		4.623	9	1.95

	S288c]		C						
P21771	37S ribosomal protein S28, mitochondrial [OS=Saccharomyces cerevisiae S288c]	851937	MRPS28; YDR337W	cell organization and biogenesis;metabolic process	mitochondrion;ribosome	RNA binding;structural molecule activity	0.129	0.274	2.12
P32378	4-hydroxybenzoate polyprenyltransferase, mitochondrial [OS=Saccharomyces cerevisiae S288c]	855778	COQ2; YNR041C	metabolic process;transport	membrane;mitochondrion	catalytic activity	0.136	0.292	2.15
P42847	37S ribosomal protein S18, mitochondrial [OS=Saccharomyces cerevisiae S288c]	855410	MRPS18; YNL306W	cell organization and biogenesis;metabolic process	mitochondrion;ribosome	RNA binding;structural molecule activity	0.155	0.334	2.15
Q08970	Mitochondrial metal transporter 2 [OS=Saccharomyces cerevisiae S288c]	855877	MMT2; YPL224C	cellular homeostasis;regulation of biological process;response to stimulus;transport	membrane;mitochondrion	transporter activity	0.186	0.407	2.19
P42949	Mitochondrial import inner membrane translocase subunit TIM16 [OS=Saccharomyces cerevisiae S288c]	853340	PAM16; YJL104W	transport	membrane;mitochondrion	protein binding	0.233	0.52	2.23
P46998	mitochondrial membrane protein FMP33 [OS=Saccharomyces cerevisiae S288c]	853279	FMP33; YJL161W		membrane;mitochondrion		0.334	0.778	2.33
Q12032	Altered inheritance of mitochondria protein 41, mitochondrial [OS=Saccharomyces cerevisiae S288c]	854390	AIM41; YOR215C		mitochondrion	catalytic activity	0.468	1.154	2.47
P00447	Superoxide dismutase [Mn], mitochondrial [OS=Saccharomyces cerevisiae S288c]	856399	SOD2; YHR008C	metabolic process;response to stimulus	mitochondrion;organelle lumen	antioxidant activity;catalytic activity;metal ion binding	1.894	4.878	2.58

Supplemental Table S8. The relative concentrations of proteins in mitochondria purified from *ups1Δ* cells cultured with or without LCA. Mitochondria were purified from *ups1Δ* cells recovered on day 4 of cell culturing with or without LCA. Mass spectrometry-based identification and quantitation of proteins recovered in purified mitochondria were performed as described in Materials and methods. The exponentially modified protein abundance index (emPAI) provided by the ″Proteome Discoverer″ software was used to calculate the relative abundance of mitochondrial proteins between specified datasets. Data on the relative concentrations of mitochondrial proteins are presented as means of 2 independent experiments.

Accession	Description	Entrez Gene ID	Gene ID	Biological Process	Cellular Component	Molecular Function	emPAI *ups1*	emPAI *ups1* + LCA	Ratio *ups1* + LCA / *ups1*
P50088	Stationary phase gene 1 protein	853151	SPG1		membrane; mitochondrion		157.489	38.811	0.25
P21771	37S ribosomal protein S28, mitochondrial	851937	MRPS28	cell organization and biogenesis; metabolic process	mitochondrion; ribosome	RNA binding; structural molecule activity	0.438	0.129	0.29
Q2V2P9	Uncharacterized protein YDR119W-A [OS=Saccharomyces cerevisiae S288c]	3799970	COX26; YDR119W-A		membrane;mitochondrion		3.642	1.154	0.32
P32387	54S ribosomal protein L41, mitochondrial [OS=Saccharomyces cerevisiae S288c]	852014	MRP20; YDR405W	cell organization and biogenesis;metabolic process	mitochondrion;ribosome	nucleotide binding;protein binding;RNA binding;structural molecule activity	0.968	0.311	0.32
Q08245	protein ZEO1 [OS=Saccharomyces cerevisiae S288c]	854040	ZEO1; YOL109W	cell organization and biogenesis	membrane;mitochondrion	protein binding	11.589	4.012	0.35
P00424	Cytochrome c oxidase polypeptide 5A, mitochondrial [OS=Saccharomyces cerevisiae S288c]	855675	COX5A; YNL052W	metabolic process;transport	membrane;mitochondrion	catalytic activity;transporter activity	2.511	0.874	0.35
P00044	Cytochrome c iso-1 [OS=Saccharomyces cerevisiae S288c]	853507	CYC1; YJR048W	metabolic process;transport	mitochondrion	metal ion binding;protein binding	9	3.217	0.36
Q06485	Autophagy-related protein 33 [OS=Saccharomyces cerevisiae S288c]	851070	ATG33; YLR356W	cell communication;metabolic process;response to stimulus	membrane;mitochondrion		2.162	0.778	0.36
P49334	Mitochondrial import receptor subunit tom22 [OS=Saccharomyces cerevisiae S288c]	855592	TOM22; YNL131W	cell organization and biogenesis;transport	membrane;mitochondrion	protein binding;transporter activity	1.783	0.668	0.37
Q12359	Ammonia transport outward protein 3 [OS=Saccharomyces cerevisiae S288c]	851992	ATO3; YDR384C	transport	membrane;mitochondrion	transporter activity	1.512	0.585	0.39
P01097	ATPase inhibitor, mitochondrial [OS=Saccharomyces cerevisiae S288c]	851347	INH1; YDL181W	metabolic process;regulation of biological process	mitochondrion	catalytic activity;enzyme regulator activity;protein binding	1.154	0.468	0.41
Q07914	mitochondrial import inner membrane translocase subunit TIM14 [OS=Saccharomyces cerevisiae S288c]	850694	PAM18; YLR008C	regulation of biological process;transport	membrane;mitochondrion	enzyme regulator activity;protein binding;transporter activity	0.778	0.334	0.43
P36147	Presequence translocated-associated motor subunit pam17, mitochondrial [OS=Saccharomyces cerevisiae S288c]	853939	PAM17; YKR065C	transport	membrane;mitochondrion		0.778	0.334	0.43
P33759	37S ribosomal protein S5, mitochondrial [OS=Saccharomyces cerevisiae S288c]	852553	MRPS5; YBR251W	cell organization and biogenesis;metabolic process	mitochondrion;ribosome	RNA binding;structural molecule activity	0.585	0.259	0.44
P51998	54S ribosomal protein yml6, mitochondrial [OS=Saccharomyces cerevisiae S288c]	854983	YML6; YML025C	cell organization and biogenesis;metabolic process	mitochondrion;ribosome	RNA binding;structural molecule activity	0.551	0.245	0.44
P36141	Putative redox protein fmp46, mitochondrial [OS=Saccharomyces cerevisiae S288c]	853923	FMP46; YKR049C	metabolic process	mitochondrion	catalytic activity;protein binding	0.52	0.233	0.45
P22354	54S ribosomal protein L20, mitochondrial [OS=Saccharomyces cerevisiae S288c]	853960	MRPL20; YKR085C	cell organization and biogenesis;metabolic process	mitochondrion;ribosome	structural molecule activity	0.389	0.179	0.46
P25613	Accumulation of dyads protein 2 [OS=Saccharomyces cerevisiae S288c]	850368	ADY2; YCR010C	transport	membrane;mitochondrion;vacuole	transporter activity	9	4.179	0.46
P04803	Tryptophan--tRNA ligase, mitochondrial [OS=Saccharomyces cerevisiae	851861	MSW1; YDR268W	metabolic process	cytoplasm;mitochondrion;organelle lumen	catalytic activity;nucleotide binding	0.292	0.136	0.47

	S288c]								
P53220	Mitochondrial import inner membrane translocase subunit TIM21 [OS=Saccharomyces cerevisiae S288c]	852921	TIM21; YGR033C	cell organization and biogenesis;transport	membrane;mitochondrion	protein binding	0.311	0.145	0.47
P48569	Uncharacterized protein YDL183C [OS=Saccharomyces cerevisiae S288c]	851345	YDL183C; YDL183C	transport			0.274	0.129	0.47
P48527	Tyrosine--tRNA ligase, mitochondrial [OS=Saccharomyces cerevisiae S288c]	856007	MSY1; YPL097W	metabolic process	cytoplasm;cytosol;mitochondrion;organelle lumen	catalytic activity;nucleotide binding;RNA binding	0.15	0.072	0.48
Q02863	ubiquitin carboxyl-terminal hydrolase 16 [OS=Saccharomyces cerevisiae S288c]	856032	UBP16; YPL072W	metabolic process	membrane;mitochondrion	catalytic activity	0.15	0.072	0.48
Q04172	Sensitive to high expression protein 9, mitochondrial [OS=Saccharomyces cerevisiae S288c]	852002	SHE9; YDR393W	cell organization and biogenesis	membrane;mitochondrion		0.172	0.083	0.48
P33303	Succinate/fumarate mitochondrial transporter [OS=Saccharomyces cerevisiae S288c]	853558	SFC1; YJR095W	metabolic process;transport	membrane;mitochondrion	structural molecule activity;transporter activity	13.678	6.743	0.49
P09624	Dihydrolipoyl dehydrogenase, mitochondrial [OS=Saccharomyces cerevisiae S288c]	850527	LPD1; YFL018C	cellular homeostasis;metabolic process;regulation of biological process	mitochondrion;organelle lumen	catalytic activity;nucleotide binding	43.367	21.539	0.50
P00360	glyceraldehyde-3-phosphate dehydrogenase 1 [OS=Saccharomyces cerevisiae S288c]	853395	TDH1; YJL052W	metabolic process	cytoplasm;cytosol;membrane;mitochondrion	catalytic activity;nucleotide binding	2.008	1.015	0.51
P28737	Protein MSP1 [OS=Saccharomyces cerevisiae S288c]	852915	MSP1; YGR028W	cell organization and biogenesis;metabolic process;regulation of biological process;response to stimulus;transport	membrane;mitochondrion;nucleus	catalytic activity;nucleotide binding;protein binding;RNA binding	0.778	0.413	0.53
P38072	Protein SCO2, mitochondrial [OS=Saccharomyces cerevisiae S288c]	852312	SCO2; YBR024W	cell organization and biogenesis;cellular homeostasis;metabolic process;transport	membrane;mitochondrion	antioxidant activity;catalytic activity;metal ion binding	0.73	0.389	0.53
P39525	3-oxoacyl-[acyl-carrier-protein] synthase homolog [OS=Saccharomyces cerevisiae S288c]	856790	CEM1; YER061C	metabolic process	mitochondrion	catalytic activity	0.688	0.369	0.54
P12695	Dihydrolipoyllysine-residue acetyltransferase component of pyruvate dehydrogenase complex, mitochondrial [OS=Saccharomyces cerevisiae S288c]	855653	LAT1; YNL071W	metabolic process	mitochondrion;organelle lumen	catalytic activity	9	4.946	0.55
P40582	Glutathione S-transferase 1 [OS=Saccharomyces cerevisiae S288c]	854856	GTT1; YIR038C	metabolic process	endoplasmic reticulum;membrane;mitochondrion;nucleus	antioxidant activity;catalytic activity;protein binding;RNA binding	1.683	0.931	0.55
P12709	glucose-6-phosphate isomerase [OS=Saccharomyces cerevisiae S288c]	852495	PGI1; YBR196C	metabolic process	cytoplasm;membrane;mitochondrion	catalytic activity	0.468	0.259	0.55
P40185	Protein mmf1, mitochondrial [OS=Saccharomyces cerevisiae S288c]	854760	MMF1; YIL051C	cell organization and biogenesis;metabolic process	mitochondrion;organelle lumen		5.31	2.981	0.56
P10662	37S ribosomal protein MRP1, mitochondrial [OS=Saccharomyces cerevisiae S288c]	851948	MRP1; YDR347W	cell organization and biogenesis;metabolic process;response to stimulus	mitochondrion;ribosome	antioxidant activity;catalytic activity;metal ion binding;structural molecule activity	0.995	0.585	0.59
Q03713	Respiratory supercomplex factor 1, mitochondrial [OS=Saccharomyces cerevisiae S288c]	854978	RCF1; YML030W	cell organization and biogenesis	membrane;mitochondrion		0.995	0.585	0.59
Q3E824	Uncharacterized protein YOR020W-A [OS=Saccharomyces cerevisiae S288c]	1466480	YOR020W-A; YOR020W-A	metabolic process;transport	membrane;mitochondrion		3.642	2.162	0.59
P00445	Superoxide dismutase [Cu-Zn] [OS=Saccharomyces cerevisiae S288c]	853568	SOD1; YJR104C	cell organization and biogenesis;cellular homeostasis;metabolic process;regulation of biological process;response to stimulus	cytoplasm;cytosol;mitochondrion;nucleus	antioxidant activity;catalytic activity;metal ion binding;protein binding	0.874	0.52	0.59
P00950	Phosphoglycerate mutase 1 [OS=Saccharomyces cerevisiae S288c]	853705	GPM1; YKL152C	metabolic process;regulation of biological process	cytoplasm;cytosol;membrane;mitochondrion	catalytic activity	1.929	1.154	0.60
P00942	Triosephosphate isomerase [OS=Saccharomyces cerevisiae S288c]	851620	TPI1; YDR050C	metabolic process	cytoplasm;cytosol;membrane;mitochondrion	catalytic activity	0.701	0.425	0.61

P33421	Succinate dehydrogenase [ubiquinone] cytochrome b subunit, mitochondrial [OS=Saccharomyces cerevisiae S288c]	853716	SDH3; YKL141W	cell organization and biogenesis;metabolic process;transport	membrane;mitochondrion	catalytic activity;metal ion binding	0.638	0.389	0.61
P40364	Mitochondrial peculiar membrane protein 1 [OS=Saccharomyces cerevisiae S288c]	853379	MPM1; YJL066C		membrane;mitochondrion		0.585	0.359	0.61
P41735	5-demethoxyubiquinone hydroxylase, mitochondrial [OS=Saccharomyces cerevisiae S288c]	854292	CAT5; YOR125C	metabolic process	membrane;mitochondrion	catalytic activity;metal ion binding	0.54	0.334	0.62
P42847	37S ribosomal protein S18, mitochondrial [OS=Saccharomyces cerevisiae S288c]	855410	MRPS18; YNL306W	cell organization and biogenesis;metabolic process	mitochondrion;ribosome	RNA binding;structural molecule activity	0.54	0.334	0.62
Q04599	54S ribosomal protein L1, mitochondrial [OS=Saccharomyces cerevisiae S288c]	851694	MRPL1; YDR116C	cell organization and biogenesis;metabolic process	mitochondrion;ribosome	RNA binding;structural molecule activity	0.438	0.274	0.63
Q12251	Uncharacterized mitochondrial carrier YPR011C [OS=Saccharomyces cerevisiae S288c]	856121	YPR011C; YPR011C	metabolic process;transport	membrane;mitochondrion	structural molecule activity;transporter activity	0.389	0.245	0.63
P12687	54S ribosomal protein L2, mitochondrial [OS=Saccharomyces cerevisiae S288c]	855727	MRP7; YNL005C	cell organization and biogenesis;metabolic process	mitochondrion;ribosome	catalytic activity;RNA binding;structural molecule activity	0.369	0.233	0.63
P00925	Enolase 2 [OS=Saccharomyces cerevisiae S288c]	856579	ENO2; YHR174W	metabolic process;regulation of biological process	cytoplasm;cytosol;membrane;mitochondrion;vacuole	catalytic activity;metal ion binding;protein binding	1.395	0.887	0.64
P15108	ATP-dependent molecular chaperone HSC82 [OS=Saccharomyces cerevisiae S288c]	855224	HSC82; YMR186W	cell organization and biogenesis;metabolic process;response to stimulus	cytoplasm;membrane;mitochondrion	catalytic activity;nucleotide binding;protein binding	1.081	0.688	0.64
P39006	Phosphatidylserine decarboxylase proenzyme 1, mitochondrial [OS=Saccharomyces cerevisiae S288c]	855552	PSD1; YNL169C	metabolic process;regulation of biological process	membrane;mitochondrion	catalytic activity	0.269	0.172	0.64
P38969	Pentamidine resistance factor, mitochondrial [OS=Saccharomyces cerevisiae S288c]	854440	PNT1; YOR266W	cell organization and biogenesis;response to stimulus	membrane;mitochondrion		0.259	0.166	0.64
P04807	Hexokinase-2 [OS=Saccharomyces cerevisiae S288c]	852639	HXK2; YGL253W	cell communication;cell organization and biogenesis;cellular homeostasis;metabolic process;regulation of biological process;response to stimulus;transport	cytosol;mitochondrion;nucleus	catalytic activity;nucleotide binding	0.259	0.166	0.64
P25087	Sterol 24-C-methyltransferase [OS=Saccharomyces cerevisiae S288c]	855003	ERG6; YML008C	metabolic process	endoplasmic reticulum;membrane;mitochondrion	catalytic activity	1.894	1.219	0.64
P16451	Pyruvate dehydrogenase complex protein X component, mitochondrial [OS=Saccharomyces cerevisiae S288c]	853107	PDX1; YGR193C	metabolic process	mitochondrion;organelle lumen	catalytic activity;structural molecule activity	2.03	1.346	0.66
P07251	ATP synthase subunit alpha, mitochondrial [OS=Saccharomyces cerevisiae S288c]	852177	ATP1; YBL099W	metabolic process;transport	cytosol;membrane;mitochondrion	catalytic activity;nucleotide binding;transporter activity	37.312	25.102	0.67
P00358	glyceraldehyde-3-phosphate dehydrogenase 2 [OS=Saccharomyces cerevisiae S288c]	853465	TDH2; YJR009C	cell death;metabolic process	cytoplasm;cytosol;membrane;mitochondrion;nucleus	catalytic activity;nucleotide binding;protein binding	3.962	2.675	0.68
P38088	Glycine--tRNA ligase 1, mitochondrial [OS=Saccharomyces cerevisiae S288c]			metabolic process	cytoplasm;mitochondrion;organelle lumen	catalytic activity;nucleotide binding;protein binding	0.399	0.271	0.68
P07143	Cytochrome c1, heme protein, mitochondrial [OS=Saccharomyces cerevisiae S288c]	854231	CYT1; YOR065W	metabolic process;transport	cytosol;membrane;mitochondrion	metal ion binding	19.309	13.251	0.69
P08417	fumarate hydratase, mitochondrial [OS=Saccharomyces cerevisiae S288c]	855866	FUM1; YPL262W	cell organization and biogenesis;metabolic process	cytoplasm;cytosol;mitochondrion;organelle lumen	catalytic activity	6.88	4.736	0.69
P52893	probable alanine aminotransferase, mitochondrial [OS=Saccharomyces cerevisiae S288c]	850778	ALT1; YLR089C	metabolic process	mitochondrion;organelle lumen	catalytic activity	0.812	0.562	0.69

P38891	Branched-chain-amino-acid aminotransferase, mitochondrial [OS=Saccharomyces cerevisiae S288c]	856615	BAT1; YHR208W	metabolic process;regulation of biological process;response to stimulus	mitochondrion;organelle lumen	catalytic activity	7.111	4.926	0.69
Q99297	Mitochondrial 2-oxodicarboxylate carrier 2 [OS=Saccharomyces cerevisiae S288c]	854397	ODC2; YOR222W	metabolic process;transport	membrane;mitochondrion	structural molecule activity;transporter activity	0.778	0.54	0.69
P06168	ketol-acid reductoisomerase, mitochondrial [OS=Saccharomyces cerevisiae S288c]	851069	ILV5; YLR355C	cell organization and biogenesis;metabolic process	mitochondrion	catalytic activity;DNA binding;metal ion binding	12.895	9	0.70
P00359	Glyceraldehyde-3-phosphate dehydrogenase 3 [OS=Saccharomyces cerevisiae S288c]	853106	TDH3; YGR192C	cell death;metabolic process	cytoplasm;cytosol;membrane;mitochondrion;nucleus	catalytic activity;nucleotide binding;RNA binding	6.406	4.484	0.70
P40416	Iron-sulfur clusters transporter ATM1, mitochondrial [OS=Saccharomyces cerevisiae S288c]	855347	ATM1; YMR301C	cellular homeostasis;metabolic process;transport	membrane;mitochondrion	catalytic activity;nucleotide binding;transporter activity	0.645	0.453	0.70
P43635	Citrate synthase 3, mitochondrial [OS=Saccharomyces cerevisiae S288c]	856107	CIT3; YPR001W	metabolic process	mitochondrion	catalytic activity	3.262	2.3	0.71
Q06892	NADH kinase POS5, mitochondrial [OS=Saccharomyces cerevisiae S288c]	855913	POS5; YPL188W	metabolic process;response to stimulus	mitochondrion;organelle lumen	catalytic activity;nucleotide binding	0.52	0.369	0.71
P32792	UPF0744 protein YSC83 [OS=Saccharomyces cerevisiae S288c]	856410	YSC83; YHR017W		membrane;mitochondrion		0.52	0.369	0.71
P39726	Glycine cleavage system H protein, mitochondrial [OS=Saccharomyces cerevisiae S288c]	851254	GCV3; YAL044C	metabolic process	mitochondrion	catalytic activity	3.642	2.594	0.71
Q04438	Stationary phase protein 4 [OS=Saccharomyces cerevisiae S288c]	855134	SPG4; YMR107W				3.642	2.594	0.71
Q12428	Probable 2-methylcitrate dehydratase [OS=Saccharomyces cerevisiae S288c]	856108	PDH1; YPR002W	metabolic process	cytoplasm;membrane;mitochondrion	catalytic activity	8.306	5.978	0.72
Q04935	Cytochrome c oxidase protein 20, mitochondrial [OS=Saccharomyces cerevisiae S288c]	851817	COX20; YDR231C	cell organization and biogenesis;metabolic process	membrane;mitochondrion	protein binding	1.424	1.031	0.72
P18239	ADP,ATP carrier protein 2 [OS=Saccharomyces cerevisiae S288c]	852250	PET9; YBL030C	cell death;metabolic process;transport	membrane;mitochondrion	structural molecule activity;transporter activity	110.034	80.113	0.73
P50087	MICOS subunit MIC26 [OS=Saccharomyces cerevisiae S288c]	853150	MOS2; MIC26; YGR235C	cell organization and biogenesis	membrane;mitochondrion		1.276	0.931	0.73
Q12230	Sphingolipid long chain base-responsive protein LSP1 [OS=Saccharomyces cerevisiae S288c]	856103	LSP1; YPL004C	regulation of biological process;response to stimulus;transport	cytoplasm;membrane;mitochondrion		53.117	38.811	0.73
Q00402	Nuclear migration protein NUM1 [OS=Saccharomyces cerevisiae S288c]	851727	NUM1; YDR150W	cell organization and biogenesis;cellular component movement;metabolic process;regulation of biological process;transport	endoplasmic reticulum;mitochondrion	catalytic activity;motor activity;protein binding	0.052	0.038	0.73
P54003	Protein SUR7 [OS=Saccharomyces cerevisiae S288c]	854953	SUR7; YML052W	cell differentiation;transport	membrane;mitochondrion		1.154	0.848	0.73
Q04013	Citrate/oxoglutarate carrier protein [OS=Saccharomyces cerevisiae S288c]	855282	YHM2; YMR241W	cell organization and biogenesis;metabolic process;transport	membrane;mitochondrion	DNA binding;structural molecule activity;transporter activity	3.467	2.548	0.73
Q02204	54S ribosomal protein L13, mitochondrial [OS=Saccharomyces cerevisiae S288c]	853875	MRPL13; YKR006C	cell organization and biogenesis;metabolic process	mitochondrion;ribosome	structural molecule activity	1.054	0.778	0.74
P50945	MICOS complex subunit MIC27 [OS=Saccharomyces cerevisiae S288c]	855623	AIM37; MIC27; YNL100W	cell organization and biogenesis	membrane;mitochondrion		0.968	0.719	0.74
P14540	fructose-bisphosphate aldolase [OS=Saccharomyces cerevisiae S288c]	853805	FBA1; YKL060C	metabolic process	cytoplasm;cytosol;membrane;mitochondrion	catalytic activity;metal ion binding	0.896	0.668	0.75
P40508	Uncharacterized protein YIL077C [OS=Saccharomyces cerevisiae S288c]	854733	YIL077C; YIL077C		mitochondrion		0.896	0.668	0.75
P40496	37S ribosomal protein S25, mitochondrial [OS=Saccharomyces cerevisiae S288c]	854715	RSM25; YIL093C	cell organization and biogenesis;metabolic process	mitochondrion;ribosome	structural molecule activity	0.896	0.668	0.75

Q12166	2-isopropylmalate synthase 2, mitochondrial [OS=Saccharomyces cerevisiae S288c]	854275	LEU9; YOR108W	metabolic process	mitochondrion	catalytic activity;protein binding	2.065	1.543	0.75
P00560	Phosphoglycerate kinase [OS=Saccharomyces cerevisiae S288c]	850370	PGK1; YCR012W	metabolic process	cytoplasm;membrane;mitochondrion	catalytic activity;nucleotide binding	4.412	3.299	0.75
P34227	Mitochondrial peroxiredoxin PRX1 [OS=Saccharomyces cerevisiae S288c]	852215	PRX1; YBL064C	cellular homeostasis;metabolic process;regulation of biological process;response to stimulus	mitochondrion	antioxidant activity;catalytic activity	5.952	4.456	0.75
P06208-1	2-isopropylmalate synthase [OS=Saccharomyces cerevisiae S288c]	855619	LEU4; YNL104C	metabolic process	cytoplasm;mitochondrion	catalytic activity;protein binding	6.017	4.541	0.75
P38885	Altered inheritance of mitochondria protein 46, mitochondrial [OS=Saccharomyces cerevisiae S288c]	856606	AIM46; YHR199C		mitochondrion	catalytic activity	0.73	0.551	0.75
P21801	Succinate dehydrogenase [ubiquinone] iron-sulfur subunit, mitochondrial [OS=Saccharomyces cerevisiae S288c]	850685	SDH2; YLL041C	metabolic process;transport	membrane;mitochondrion	catalytic activity;metal ion binding	32.839	24.809	0.76
P23641	mitochondrial phosphate carrier protein [OS=Saccharomyces cerevisiae S288c]	853540	MIR1; YJR077C	metabolic process;transport	membrane;mitochondrion	protein binding;structural molecule activity;transporter activity	17.957	13.678	0.76
P28241	Isocitrate dehydrogenase [NAD] subunit 2, mitochondrial [OS=Saccharomyces cerevisiae S288c]	854303	IDH2; YOR136W	metabolic process	mitochondrion;organelle lumen	catalytic activity;metal ion binding;nucleotide binding;protein binding;RNA binding	4.78	3.642	0.76
P28834	Isocitrate dehydrogenase [NAD] subunit 1, mitochondrial [OS=Saccharomyces cerevisiae S288c]	855691	IDH1; YNL037C	metabolic process;transport	cytosol;mitochondrion;organelle lumen	catalytic activity;metal ion binding;nucleotide binding;protein binding;RNA binding	10.288	7.859	0.76
P32611	54S ribosomal protein RML2, mitochondrial [OS=Saccharomyces cerevisiae S288c]	856660	RML2; YEL050C	cell organization and biogenesis;metabolic process	mitochondrion;ribosome	catalytic activity;RNA binding;structural molecule activity	0.509	0.389	0.76
Q08223	Altered inheritance of mitochondria protein 39, mitochondrial [OS=Saccharomyces cerevisiae S288c]	854103	AIM39; YOL053W		cytosol;membrane;mitochondrion		0.468	0.359	0.77
P32796-1	Carnitine O-acetyltransferase, mitochondrial [OS=Saccharomyces cerevisiae S288c]	854965	CAT2; YML042W	metabolic process;transport	membrane;mitochondrion	catalytic activity	10.189	7.938	0.78
P19882	heat shock protein 60, mitochondrial [OS=Saccharomyces cerevisiae S288c]	850963	HSP60; YLR259C	cell organization and biogenesis;metabolic process;regulation of biological process;transport	cytoplasm;cytosol;membrane;mitochondrion;organelle lumen	catalytic activity;DNA binding;nucleotide binding;protein binding	39.296	30.623	0.78
P32453	Protein ATP11, mitochondrial [OS=Saccharomyces cerevisiae S288c]	855401	ATP11; YNL315C	cell organization and biogenesis	mitochondrion	protein binding	0.995	0.778	0.78
P38297	Mitofusin FZO1 [OS=Saccharomyces cerevisiae S288c]	852477	FZO1; YBR179C	cell organization and biogenesis	membrane;mitochondrion	catalytic activity;nucleotide binding;protein binding	0.248	0.194	0.78
Q06143	mitochondrial dicarboxylate transporter [OS=Saccharomyces cerevisiae S288c]	851063	DIC1; YLR348C	metabolic process;transport	membrane;mitochondrion	structural molecule activity;transporter activity	0.931	0.73	0.78
P49095	Glycine dehydrogenase (Decarboxylating), mitochondrial [OS=Saccharomyces cerevisiae S288c]	855227	GCV2; YMR189W	metabolic process	cytosol;mitochondrion	catalytic activity	1.184	0.931	0.79
P40495	Homoisocitrate dehydrogenase, mitochondrial [OS=Saccharomyces cerevisiae S288c]	854714	LYS12; YIL094C	metabolic process	mitochondrion	catalytic activity;metal ion binding;nucleotide binding	4.623	3.642	0.79
P35191	DnaJ homolog 1, mitochondrial [OS=Saccharomyces cerevisiae S288c]	850530	MDJ1; YFL016C	cell organization and biogenesis;metabolic process;regulation of biological process;response to stimulus	mitochondrion;organelle lumen	enzyme regulator activity;metal ion binding;nucleoti	1.656	1.31	0.79

						de binding;protein binding			
P11914	Mitochondrial-processing peptidase subunit alpha [OS=Saccharomyces cerevisiae S288c]	856419	MAS2; YHR024C	metabolic process;transport	membrane;mitochondrion;organelle lumen	catalytic activity;metal ion binding;protein binding	0.738	0.585	0.79
Q03976	37S ribosomal protein S24, mitochondrial [OS=Saccharomyces cerevisiae S288c]	851755	RSM24; YDR175C	cell organization and biogenesis;metabolic process	mitochondrion;ribosome	structural molecule activity	0.738	0.585	0.79
P32891	D-lactate dehydrogenase [cytochrome] 1, mitochondrial [OS=Saccharomyces cerevisiae S288c]	851380	DLD1; YDL174C	metabolic process;transport	membrane;mitochondrion	catalytic activity;nucleotide binding	11.253	9	0.80
Q03430	37S ribosomal protein RSM28, mitochondrial [OS=Saccharomyces cerevisiae S288c]	852105	RSM28; YDR494W	cell organization and biogenesis;metabolic process	mitochondrion;ribosome	structural molecule activity	1.015	0.823	0.81
Q07651	SUR7 family protein FMP45 [OS=Saccharomyces cerevisiae S288c]	851304	FMP45; YDL222C	cell differentiation;cell organization and biogenesis	membrane;mitochondrion		7.377	6.017	0.82
P00431	cytochrome c peroxidase, mitochondrial [OS=Saccharomyces cerevisiae S288c]	853940	CCP1; YKR066C	metabolic process;response to stimulus	mitochondrion;organelle lumen	antioxidant activity;catalytic activity;metal ion binding;protein binding	0.859	0.701	0.82
P42940	Probable electron transfer flavoprotein subunit beta [OS=Saccharomyces cerevisiae S288c]	853121	CIR1; YGR207C	metabolic process;transport	mitochondrion;organelle lumen		3.217	2.652	0.82
P16547	Mitochondrial outer membrane protein OM45 [OS=Saccharomyces cerevisiae S288c]	854670	OM45; YIL136W		membrane;mitochondrion		574.44	477.63	0.83
P00175	Cytochrome b2, mitochondrial [OS=Saccharomyces cerevisiae S288c]	854950	CYB2; YML054C	metabolic process;transport	cytosol;membrane;mitochondrion;nucleus	catalytic activity;metal ion binding;nucleotide binding	0.496	0.413	0.83
P00330	Alcohol dehydrogenase 1 [OS=Saccharomyces cerevisiae S288c]	854068	ADH1; YOL086C	metabolic process	cytoplasm;membrane	catalytic activity;metal ion binding	2.36	1.976	0.84
P00890	citrate synthase, mitochondrial [OS=Saccharomyces cerevisiae S288c]	855732	CIT1; YNR001C	metabolic process;transport	cytosol;mitochondrion;organelle lumen	catalytic activity	14.086	11.798	0.84
P32191	Glycerol-3-phosphate dehydrogenase, mitochondrial [OS=Saccharomyces cerevisiae S288c]	854651	GUT2; YIL155C	metabolic process	membrane;mitochondrion	catalytic activity	5.434	4.555	0.84
Q12031	Mitochondrial 2-methylisocitrate lyase [OS=Saccharomyces cerevisiae S288c]	856114	ICL2; YPR006C	metabolic process	mitochondrion;organelle lumen	catalytic activity	2.793	2.36	0.84
Q12374	Nuclear control of ATPase protein 2 [OS=Saccharomyces cerevisiae S288c]	856278	NCA2; YPR155C	metabolic process	membrane;mitochondrion		0.668	0.565	0.85
Q08818	Meiotic sister-chromatid recombination protein 6, mitochondrial [OS=Saccharomyces cerevisiae S288c]	854536	MSC6; YOR354C	metabolic process	mitochondrion;organelle lumen	RNA binding	0.506	0.431	0.85
P32335	Protein MSS51, mitochondrial [OS=Saccharomyces cerevisiae S288c]	850900	MSS51; YLR203C	metabolic process;regulation of biological process	membrane;mitochondrion	protein binding;translation regulator activity	0.995	0.848	0.85
Q03028	Mitochondrial 2-oxodicarboxylate carrier 1 [OS=Saccharomyces cerevisiae S288c]	855969	ODC1; YPL134C	metabolic process;transport	membrane;mitochondrion	structural molecule activity;transporter activity	4.995	4.275	0.86
P25374	Cysteine desulfurase, mitochondrial [OS=Saccharomyces cerevisiae S288c]	850343	NFS1; YCL017C	cell organization and biogenesis;cellular homeostasis;metabolic process	mitochondrion;nucleus	catalytic activity;metal ion binding;protein binding	1.346	1.154	0.86
Q07349	MIOREX complex component 9 [OS=Saccharomyces cerevisiae S288c]	851535	YDL027C; MRX9; YDL027C		endoplasmic reticulum;membrane;mitochondrion		1.276	1.096	0.86
P39522	Dihydroxy-acid dehydratase, mitochondrial [OS=Saccharomyces cerevisiae S288c]	853473	ILV3; YJR016C	metabolic process	mitochondrion	catalytic activity;metal ion binding	13.175	11.328	0.86
P23180	Probable oxidoreductase AIM17 [OS=Saccharomyces cerevisiae S288c]	856365	AIM17; YHL021C	cell organization and biogenesis;metabolic process	mitochondrion	catalytic activity;metal ion binding	3.467	2.981	0.86
P53292	37S ribosomal protein S35, mitochondrial [OS=Saccharomyces cerevisiae S288c]	853075	MRPS35; YGR165W	cell organization and biogenesis;metabolic process	mitochondrion;ribosome	structural molecule activity	1.754	1.512	0.86

P32473	Pyruvate dehydrogenase E1 component subunit beta, mitochondrial [OS=Saccharomyces cerevisiae S288c]	852522	PDB1; YBR221C	metabolic process	mitochondrion;organelle lumen	catalytic activity	6.848	5.952	0.87
P16622	Ferrochelatase, mitochondrial [OS=Saccharomyces cerevisiae S288c]	854347	HEM15; YOR176W	metabolic process	membrane;mitochondrion	catalytic activity	4.337	3.806	0.88
Q00711	Succinate dehydrogenase [ubiquinone] flavoprotein subunit, mitochondrial [OS=Saccharomyces cerevisiae S288c]	853709	SDH1; YKL148C	metabolic process;transport	membrane;mitochondrion	catalytic activity;nucleotide binding;protein binding	9.578	8.454	0.88
Q01163	37S ribosomal protein S23, mitochondrial [OS=Saccharomyces cerevisiae S288c]	852748	RSM23; YGL129C	cell organization and biogenesis;metabolic process	mitochondrion;ribosome	catalytic activity;nucleotide binding;RNA binding;structural molecule activity	1.11	0.983	0.89
P07806	Valine--tRNA ligase, mitochondrial [OS=Saccharomyces cerevisiae S288c]	852986	VAS1; YGR094W	metabolic process;regulation of biological process	cytoplasm;cytosol;mitochondrion	catalytic activity;nucleotide binding	0.377	0.334	0.89
Q12466	tricalbin-1 [OS=Saccharomyces cerevisiae S288c]	854253	TCB1; YOR086C	cell organization and biogenesis;regulation of biological process	endoplasmic reticulum;membrane;mitochondrion	metal ion binding;protein binding	0.468	0.417	0.89
P17505	Malate dehydrogenase, mitochondrial [OS=Saccharomyces cerevisiae S288c]	853777	MDH1; YKL085W	metabolic process	mitochondrion;organelle lumen	catalytic activity;protein binding;RNA binding	40.596	36.276	0.89
P19414	Aconitate hydratase, mitochondrial [OS=Saccharomyces cerevisiae S288c]	851013	ACO1; YLR304C	cell organization and biogenesis;metabolic process;transport	cytoplasm;cytosol;mitochondrion;organelle lumen	catalytic activity;DNA binding;metal ion binding	30.623	27.61	0.90
P33416	Heat shock protein 78, mitochondrial [OS=Saccharomyces cerevisiae S288c]	851845	HSP78; YDR258C	cell organization and biogenesis;metabolic process;regulation of biological process;response to stimulus	mitochondrion;organelle lumen	catalytic activity;nucleotide binding;protein binding	3.359	3.042	0.91
Q07500	External NADH-ubiquinone oxidoreductase 2, mitochondrial [OS=Saccharomyces cerevisiae S288c]	851474	NDE2; YDL085W	metabolic process	mitochondrion	catalytic activity;nucleotide binding	4.012	3.642	0.91
P36112	MICOS complex subunit Mic60 [OS=Saccharomyces cerevisiae S288c]	853886	FCJ1; MIC60; YKR016W	cell organization and biogenesis;transport	membrane;mitochondrion		12.895	11.798	0.91
P32340	Rotenone-insensitive NADH-ubiquinone oxidoreductase, mitochondrial [OS=Saccharomyces cerevisiae S288c]	854919	NDI1; YML120C	metabolic process;regulation of biological process	membrane;mitochondrion;organelle lumen	catalytic activity;nucleotide binding	17.874	16.433	0.92
P53312	Succinyl-CoA ligase [ADP-forming] subunit beta, mitochondrial [OS=Saccharomyces cerevisiae S288c]	853159	LSC2; YGR244C	metabolic process	mitochondrion	catalytic activity;nucleotide binding	9	8.284	0.92
P07213	Mitochondrial import receptor subunit TOM70 [OS=Saccharomyces cerevisiae S288c]	855602	TOM70; YNL121C	cell organization and biogenesis;transport	membrane;mitochondrion	protein binding;transporter activity	2.888	2.665	0.92
P00830	ATP synthase subunit beta, mitochondrial [OS=Saccharomyces cerevisiae S288c]	853585	ATP2; YJR121W	metabolic process;transport	cytosol;membrane;mitochondrion	catalytic activity;nucleotide binding;protein binding;transporter activity	30.623	28.286	0.92
P40341	Mitochondrial respiratory chain complexes assembly protein YTA12 [OS=Saccharomyces cerevisiae S288c]	855114	YTA12; YMR089C	cell organization and biogenesis;metabolic process;response to stimulus;transport	cytoplasm;membrane;mitochondrion	catalytic activity;metal ion binding;nucleotide binding;protein binding	1.184	1.096	0.93
P46367	Potassium-activated aldehyde dehydrogenase, mitochondrial [OS=Saccharomyces cerevisiae S288c]	854556	ALD4; YOR374W	metabolic process	mitochondrion;organelle lumen	catalytic activity	25.827	24.119	0.93
P30624	Long-chain-fatty-acid--CoA ligase 1 [OS=Saccharomyces cerevisiae S288c]	854495	FAA1; YOR317W	metabolic process;transport	endoplasmic reticulum;membrane;mitochondrion	catalytic activity;nucleotide binding	7.072	7.072	1.00
P07342	Acetolactate synthase catalytic subunit, mitochondrial [OS=Saccharomyces cerevisiae S288c]	855135	ILV2; YMR108W	metabolic process	mitochondrion	catalytic activity;metal ion binding;nucleotide binding	4.223	4.223	1.00
P08067	cytochrome b-c1 complex subunit Rieske, mitochondrial [OS=Saccharomyces cerevisiae S288c]	856689	RIP1; YEL024W	metabolic process;transport	membrane;mitochondrion	catalytic activity;metal ion binding;transporter activity	20.544	20.544	1.00

Q02486	ARS-binding factor 2, mitochondrial [OS=Saccharomyces cerevisiae S288c]	855094	ABF2; YMR072W	cell organization and biogenesis;metabolic process;regulation of biological process	chromosome;mitochondrion;nucleus	DNA binding	27.943	27.943	1.00
P04037	cytochrome c oxidase subunit 4, mitochondrial [OS=Saccharomyces cerevisiae S288c]	852688	COX4; YGL187C	cell organization and biogenesis;metabolic process;transport	membrane;mitochondrion	catalytic activity;metal ion binding;transporter activity	17.738	17.738	1.00
P50085	Prohibitin-2 [OS=Saccharomyces cerevisiae S288c]	853146	PHB2; YGR231C	cell organization and biogenesis;metabolic process;regulation of biological process	membrane;mitochondrion	protein binding	3.329	3.329	1.00
P07246	Alcohol dehydrogenase 3, mitochondrial [OS=Saccharomyces cerevisiae S288c]	855107	ADH3; YMR083W	metabolic process	mitochondrion;organelle lumen	catalytic activity;metal ion binding	4.179	4.179	1.00
P32795	Mitochondrial inner membrane i-AAA protease supercomplex subunit YME1 [OS=Saccharomyces cerevisiae S288c]	856135	YME1; YPR024W	metabolic process;response to stimulus;transport	membrane;mitochondrion	catalytic activity;metal ion binding;nucleotide binding;protein binding	0.891	0.891	1.00
P38988	Mitochondrial GTP/GDP carrier protein 1 [OS=Saccharomyces cerevisiae S288c]	851329	GGC1; YDL198C	cell organization and biogenesis;cellular homeostasis;metabolic process;transport	membrane;mitochondrion	structural molecule activity;transporter activity	4.179	4.179	1.00
Q12480	Probable electron transfer flavoprotein subunit alpha, mitochondrial [OS=Saccharomyces cerevisiae S288c]	856112	AIM45; YPR004C	metabolic process;transport	mitochondrion;organelle lumen	catalytic activity;nucleotide binding	1.818	1.818	1.00
P40961	prohibitin-1 [OS=Saccharomyces cerevisiae S288c]	853033	PHB1; YGR132C	cell organization and biogenesis;metabolic process;regulation of biological process	membrane;mitochondrion	protein binding	2.793	2.793	1.00
P36046	Mitochondrial intermembrane space import and assembly protein 40 [OS=Saccharomyces cerevisiae S288c]	853639	MIA40; YKL195W	cellular component movement;metabolic process;response to stimulus;transport	membrane;mitochondrion	catalytic activity;protein binding;transporter activity	1.555	1.555	1.00
P53140	Protein RMD9, mitochondrial [OS=Saccharomyces cerevisiae S288c]	852771	RMD9; YGL107C	cell differentiation;metabolic process;regulation of biological process	membrane;mitochondrion	RNA binding	0.585	0.585	1.00
P53721	Respiratory supercomplex factor 2, mitochondrial [OS=Saccharomyces cerevisiae S288c]	855752	RCF2; YNR018W	cell organization and biogenesis	membrane;mitochondrion		5.579	5.579	1.00
P32897	Mitochondrial import inner membrane translocase subunit tim23 [OS=Saccharomyces cerevisiae S288c]	855751	TIM23; YNR017W	transport	membrane;mitochondrion	protein binding;transporter activity	2.981	2.981	1.00
P43616	Cys-Gly metallodipeptidase dug1 [OS=Saccharomyces cerevisiae S288c]	850605	DUG1; YFR044C	metabolic process	cytoplasm;mitochondrion;ribosome	catalytic activity;metal ion binding	0.711	0.711	1.00
P00410	Cytochrome c oxidase subunit 2 [OS=Saccharomyces cerevisiae S288c]	854622	COX2; Q0250	metabolic process;transport	membrane;mitochondrion	catalytic activity;metal ion binding;protein binding;transporter activity	1.61	1.61	1.00
P32331	Carrier protein YMC1, mitochondrial [OS=Saccharomyces cerevisiae S288c]	856171	YMC1; YPR058W	metabolic process;transport	membrane;mitochondrion;vacuole	structural molecule activity;transporter activity	0.968	0.968	1.00
P38325	Mitochondrial outer membrane protein OM14 [OS=Saccharomyces cerevisiae S288c]	852531	OM14; YBR230C	transport	membrane;mitochondrion		4.179	4.179	1.00
P53881	54S ribosomal protein L22, mitochondrial [OS=Saccharomyces cerevisiae S288c]	855544	MRPL22; YNL177C	cell organization and biogenesis;metabolic process	mitochondrion;ribosome	RNA binding;structural molecule activity	0.551	0.551	1.00
P23644	Mitochondrial import receptor subunit TOM40 [OS=Saccharomyces cerevisiae S288c]	855243	TOM40; YMR203W	transport	cytosol;membrane;mitochondrion	protein binding;transporter activity	0.896	0.896	1.00
P10507	mitochondrial-processing peptidase subunit beta [OS=Saccharomyces cerevisiae S288c]	850860	MAS1; YLR163C	metabolic process;transport	mitochondrion;organelle lumen	catalytic activity;metal ion binding;protein binding	0.616	0.616	1.00
Q06493	LETM1 domain-containing protein YLH47, mitochondrial [OS=Saccharomyces cerevisiae S288c]	856243	YLH47; YPR125W	cell organization and biogenesis;transport	membrane;mitochondrion		0.61	0.61	1.00
Q04728	Arginine biosynthesis bifunctional protein ArgJ, mitochondrial [OS=Saccharomyces cerevisiae S288c]	855084	ARG7; YMR062C	metabolic process	mitochondrion;organelle lumen	catalytic activity	0.417	0.417	1.00

P53969	Sorting assembly machinery 50 kDa subunit [OS=Saccharomyces cerevisiae S288c]	855705	SAM50; YNL026W	cell organization and biogenesis;transport	membrane;mitochondrion	protein binding;transporter activity	0.374	0.374	1.00
P25573	Mitochondrial inner membrane i-AAA protease supercomplex subunit MGR1 [OS=Saccharomyces cerevisiae S288c]	850313	MGR1; YCL044C	metabolic process	membrane;mitochondrion	protein binding	0.701	0.701	1.00
P22136	ATPase expression protein 2, mitochondrial [OS=Saccharomyces cerevisiae S288c]	855325	AEP2; YMR282C	metabolic process;regulation of biological process	mitochondrion	RNA binding	0.292	0.292	1.00
P38910	10 kDa heat shock protein, mitochondrial [OS=Saccharomyces cerevisiae S288c]	854185	HSP10; YOR020C	cell organization and biogenesis;metabolic process;response to stimulus;transport	cytoplasm;mitochondrion;organelle lumen	metal ion binding;nucleotide binding;protein binding	2.162	2.162	1.00
P38523	GrpE protein homolog, mitochondrial [OS=Saccharomyces cerevisiae S288c]	854407	MGE1; YOR232W	metabolic process;regulation of biological process;transport	membrane;mitochondrion;organelle lumen	enzyme regulator activity;nucleotide binding;protein binding	0.624	0.624	1.00
P19955	37S ribosomal protein YMR-31, mitochondrial [OS=Saccharomyces cerevisiae S288c]	850610	YMR31; YFR049W	cell organization and biogenesis;metabolic process	mitochondrion;ribosome	catalytic activity;protein binding;structural molecule activity	2.594	2.594	1.00
Q03798	Altered inheritance of mitochondria protein 36, mitochondrial [OS=Saccharomyces cerevisiae S288c]	855189	AIM36; YMR157C		membrane;mitochondrion		0.624	0.624	1.00
Q12482	Mitochondrial aspartate-glutamate transporter AGC1 [OS=Saccharomyces cerevisiae S288c]	856132	AGC1; YPR021C	metabolic process;transport	membrane;mitochondrion	structural molecule activity;transporter activity	0.238	0.238	1.00
P41921	glutathione reductase [OS=Saccharomyces cerevisiae S288c]	856014	GLR1; YPL091W	cellular homeostasis;metabolic process;regulation of biological process;response to stimulus	cytoplasm;cytosol;mitochondrion;nucleus	antioxidant activity;catalytic activity;nucleotide binding	0.334	0.334	1.00
P53889	uncharacterized mitochondrial hydrolase FMP41 [OS=Saccharomyces cerevisiae S288c]	855553	FMP41; YNL168C	metabolic process	mitochondrion	catalytic activity;metal ion binding	0.501	0.501	1.00
O14467	multiprotein-bridging factor 1 [OS=Saccharomyces cerevisiae S288c]	854474	MBF1; YOR298C-A	metabolic process;regulation of biological process	cytoplasm;mitochondrion;nucleus	DNA binding	0.874	0.874	1.00
P37299	cytochrome b-c1 complex subunit 10 [OS=Saccharomyces cerevisiae S288c]	856390	QCR10; YHR001W-A	metabolic process;transport	membrane;mitochondrion	catalytic activity;transporter activity	2.981	2.981	1.00
Q03327	Mitochondrial fusion and transport protein Ugo1 [OS=Saccharomyces cerevisiae S288c]	852081	UGO1; YDR470C	cell organization and biogenesis;metabolic process	membrane;mitochondrion	protein binding;structural molecule activity	0.701	0.701	1.00
P36151	Uncharacterized protein YKR070W [OS=Saccharomyces cerevisiae S288c]	853944	YKR070W; YKR070W	metabolic process	mitochondrion		0.438	0.438	1.00
P32902	37S ribosomal protein MRP4, mitochondrial [OS=Saccharomyces cerevisiae S288c]	856384	MRP4; YHL004W	cell organization and biogenesis;metabolic process	mitochondrion;ribosome	structural molecule activity	0.468	0.468	1.00
Q02883	N-acyl-phosphatidylethanolamine-hydrolyzing phospholipase D, mitochondrial [OS=Saccharomyces cerevisiae S288c]	856001	FMP30; YPL103C	metabolic process	membrane;mitochondrion	catalytic activity;metal ion binding	0.304	0.304	1.00
P17558	37S ribosomal protein PET123, mitochondrial [OS=Saccharomyces cerevisiae S288c]	854329	PET123; YOR158W	cell organization and biogenesis;metabolic process	mitochondrion;ribosome	structural molecule activity	0.668	0.668	1.00
P47045	mitochondrial import inner membrane translocase subunit TIM54 [OS=Saccharomyces cerevisiae S288c]	853392	TIM54; YJL054W	cell organization and biogenesis;transport	cytosol;membrane;mitochondrion	transporter activity	0.334	0.334	1.00
P00401	Cytochrome c oxidase subunit 1 [OS=Saccharomyces cerevisiae S288c]	854598	COX1; Q0045	metabolic process;transport	membrane;mitochondrion	catalytic activity;metal ion binding;transporter activity	0.778	0.778	1.00
P38705	Serine--tRNA ligase, mitochondrial [OS=Saccharomyces cerevisiae S288c]	856402	DIA4; YHR011W	metabolic process	cytoplasm;mitochondrion;organelle lumen	catalytic activity;nucleotide binding	0.15	0.15	1.00
P25038	Translation initiation factor IF-2, mitochondrial [OS=Saccharomyces cerevisiae S288c]	854135	IFM1; YOL023W	cell organization and biogenesis;metabolic process	mitochondrion;nucleus	catalytic activity;nucleotide binding;RNA binding	0.11	0.11	1.00

P32839	Mitochondrial chaperone BCS1 [OS=Saccharomyces cerevisiae S288c]	851981	BCS1; YDR375C	cell organization and biogenesis;metabolic process;response to stimulus;transport	cytosol;membrane;mitochondrion	catalytic activity;nucleotide binding;transporter activity	0.194	0.194	1.00
P38714	Arginine--tRNA ligase, mitochondrial [OS=Saccharomyces cerevisiae S288c]	856491	MSR1; YHR091C	cell organization and biogenesis;metabolic process	cytoplasm;mitochondrion;organelle lumen	catalytic activity;nucleotide binding	0.055	0.055	1.00
Q12375	mitochondrial ornithine transporter 1 [OS=Saccharomyces cerevisiae S288c]	854297	ORT1; YOR130C	metabolic process;transport	membrane;mitochondrion	structural molecule activity;transporter activity	0.54	0.54	1.00
P38169	Kynurenine 3-monooxygenase [OS=Saccharomyces cerevisiae S288c]	852179	BNA4; YBL098W	metabolic process	membrane;mitochondrion	catalytic activity;nucleotide binding	0.172	0.172	1.00
P34222	Peptidyl-tRNA hydrolase 2 [OS=Saccharomyces cerevisiae S288c]	852223	PTH2; YBL057C	regulation of biological process	cytoplasm;cytosol;membrane;mitochondrion	catalytic activity;protein binding	0.425	0.425	1.00
P53732	37S ribosomal protein S12, mitochondrial [OS=Saccharomyces cerevisiae S288c]	855772	MRPS12; YNR036C	cell organization and biogenesis;metabolic process	mitochondrion;ribosome	structural molecule activity	0.668	0.668	1.00
P40502	Altered inheritance of mitochondria protein 19, mitochondrial [OS=Saccharomyces cerevisiae S288c]	854722	AIM19; YIL087C		membrane;mitochondrion		0.585	0.585	1.00
P40165	NAD(P)H-hydrate epimerase [OS=Saccharomyces cerevisiae S288c]	855521	YNL200C; YNL200C	metabolic process	cytoplasm;mitochondrion	catalytic activity;metal ion binding;nucleotide binding	0.468	0.468	1.00
P38172	MIOREX complex component 3 [OS=Saccharomyces cerevisiae S288c]	852181	YBL095W; MRX3; YBL095W		membrane;mitochondrion		0.311	0.311	1.00
P39515	mitochondrial import inner membrane translocase subunit Tim17 [OS=Saccharomyces cerevisiae S288c]	853298	TIM17; YJL143W	cell organization and biogenesis;transport	membrane;mitochondrion	protein binding;transporter activity	1.371	1.371	1.00
P40159	Uncharacterized protein YNL208W [OS=Saccharomyces cerevisiae S288c]	855513	YNL208W; YNL208W		membrane;mitochondrion;ribosome		0.292	0.292	1.00
P19516	Cytochrome c oxidase assembly protein COX11, mitochondrial [OS=Saccharomyces cerevisiae S288c]	855971	COX11; YPL132W	cell organization and biogenesis;metabolic process	membrane;mitochondrion;ribosome	metal ion binding	0.274	0.274	1.00
P48360	Probable NADPH:adrenodoxin oxidoreductase, mitochondrial [OS=Saccharomyces cerevisiae S288c]	851982	ARH1; YDR376W	cellular homeostasis;metabolic process;transport	cytoplasm;membrane;mitochondrion;organelle lumen	catalytic activity	0.16	0.16	1.00
Q05648	MIOREX complex component 10 [OS=Saccharomyces cerevisiae S288c]	851876	YDR282C; MRX10; YDR282C		membrane;mitochondrion		0.222	0.222	1.00
P40530	[Pyruvate dehydrogenase (acetyl-transferring)] kinase 1, mitochondrial [OS=Saccharomyces cerevisiae S288c]	854769	PKP1; YIL042C	metabolic process	mitochondrion;organelle lumen	catalytic activity;nucleotide binding;protein binding	0.245	0.245	1.00
P22438	Methionine--tRNA ligase, mitochondrial [OS=Saccharomyces cerevisiae S288c]	853081	MSM1; YGR171C	metabolic process	cytoplasm;cytosol;mitochondrion;organelle lumen	catalytic activity;nucleotide binding	0.136	0.136	1.00
Q01976	ADP-ribose pyrophosphatase [OS=Saccharomyces cerevisiae S288c]	852408	YSA1; YBR111C	metabolic process	cytoplasm;mitochondrion;nucleus	catalytic activity;metal ion binding	0.194	0.194	1.00
Q01532-1	Cysteine proteinase 1, mitochondrial [OS=Saccharomyces cerevisiae S288c]			metabolic process;regulation of biological process;response to stimulus	cytoplasm;mitochondrion	catalytic activity;DNA binding;RNA binding	0.179	0.179	1.00
P25372	Thioredoxin-3, mitochondrial [OS=Saccharomyces cerevisiae S288c]	850444	TRX3; YCR083W	cellular homeostasis;metabolic process;regulation of biological process;response to stimulus;transport	cytoplasm;mitochondrion	catalytic activity	0.52	0.52	1.00
P21306	ATP synthase subunit epsilon, mitochondrial [OS=Saccharomyces cerevisiae S288c]	855857	ATP15; YPL271W	metabolic process;transport	membrane;mitochondrion	catalytic activity;transporter activity	0.778	0.778	1.00
Q06405	ATP synthase subunit f, mitochondrial [OS=Saccharomyces cerevisiae S288c]	851983	ATP17; YDR377W	metabolic process;transport	membrane;mitochondrion	catalytic activity;transporter activity	1.512	1.512	1.00
P46998	mitochondrial membrane protein FMP33 [OS=Saccharomyces cerevisiae S288c]	853279	FMP33; YJL161W		membrane;mitochondrion		0.334	0.334	1.00

P32463	Acyl carrier protein, mitochondrial [OS=Saccharomyces cerevisiae S288c]	853642	ACP1; YKL192C	metabolic process;transport	mitochondrion		0.259	0.259	1.00
Q07821	Iron-sulfur assembly protein 1 [OS=Saccharomyces cerevisiae S288c]	850632	ISA1; YLL027W	cell organization and biogenesis;metabolic process	mitochondrion;organelle lumen	metal ion binding;structural molecule activity	0.136	0.136	1.00
P25345	Asparagine--tRNA ligase, mitochondrial [OS=Saccharomyces cerevisiae S288c]	850388	SLM5; YCR024C	metabolic process	cytoplasm;mitochondrion;organelle lumen	catalytic activity;nucleotide binding	0.172	0.172	1.00
P08525	Cytochrome b-c1 complex subunit 8 [OS=Saccharomyces cerevisiae S288c]	853273	QCR8; YJL166W	metabolic process;transport	membrane;mitochondrion	catalytic activity;transporter activity	0.585	0.585	1.00
P34224	Uncharacterized protein YBL059W [OS=Saccharomyces cerevisiae S288c]	852221	YBL059W; YBL059W		membrane;mitochondrion		0.212	0.212	1.00
P38271	Protein OPY1 [OS=Saccharomyces cerevisiae S288c]	852426	OPY1; YBR129C		cytoplasm;mitochondrion		0.274	0.274	1.00
P40015	inositol phosphosphingolipids phospholipase C [OS=Saccharomyces cerevisiae S288c]	856739	ISC1; YER019W	metabolic process;response to stimulus	endoplasmic reticulum;membrane;mitochondrion	catalytic activity;metal ion binding	0.212	0.212	1.00
Q3E776	Uncharacterized protein YBR255C-A [OS=Saccharomyces cerevisiae S288c]	852558	YBR255C-A; YBR255C-A		membrane		0.389	0.389	1.00
P36163	mitochondrial metalloendopeptidase OMA1 [OS=Saccharomyces cerevisiae S288c]	853962	OMA1; YKR087C	metabolic process	membrane;mitochondrion	catalytic activity;metal ion binding	0.245	0.245	1.00
P38122	3-methyl-2-oxobutanoate hydroxymethyltransferase [OS=Saccharomyces cerevisiae S288c]	852474	ECM31; YBR176W	metabolic process	mitochondrion	catalytic activity	0.122	0.122	1.00
P32378	4-hydroxybenzoate polyprenyltransferase, mitochondrial [OS=Saccharomyces cerevisiae S288c]	855778	COQ2; YNR041C	metabolic process;transport	membrane;mitochondrion	catalytic activity	0.292	0.292	1.00
P18900	hexaprenyl pyrophosphate synthase, mitochondrial [OS=Saccharomyces cerevisiae S288c]	852288	COQ1; YBR003W	metabolic process	membrane;mitochondrion	catalytic activity;metal ion binding;protein binding	0.166	0.166	1.00
Q05779	Ubiquinone biosynthesis protein COQ9, mitochondrial [OS=Saccharomyces cerevisiae S288c]	850898	COQ9; YLR201C	metabolic process	membrane;mitochondrion		0.166	0.166	1.00
P54115	Magnesium-activated aldehyde dehydrogenase, cytosolic [OS=Saccharomyces cerevisiae S288c]	856044	ALD6; YPL061W	metabolic process;response to stimulus	cytoplasm;cytosol;mitochondrion	catalytic activity	0.077	0.077	1.00
P25348	54S ribosomal protein L32, mitochondrial [OS=Saccharomyces cerevisiae S288c]	850359	MRPL32; YCR003W	cell organization and biogenesis;metabolic process	membrane;mitochondrion;ribosome	RNA binding;structural molecule activity	0.179	0.179	1.00
P38341	MICOS complex subunit MIC12 [OS=Saccharomyces cerevisiae S288c]	852566	AIM5; MIC12; YBR262C	cell organization and biogenesis	membrane;mitochondrion		0.292	0.292	1.00
P10174	cytochrome c oxidase subunit 7 [OS=Saccharomyces cerevisiae S288c]	855298	COX7; YMR256C	metabolic process;transport	membrane;mitochondrion	catalytic activity;transporter activity	1.154	1.154	1.00
P04039	Cytochrome c oxidase polypeptide VIII, mitochondrial [OS=Saccharomyces cerevisiae S288c]	851111	COX8; YLR395C	metabolic process;transport	membrane;mitochondrion	catalytic activity;transporter activity	0.778	0.778	1.00
P53219	Abhydrolase domain-containing protein IMO32 [OS=Saccharomyces cerevisiae S288c]	852919	IMO32; YGR031W	metabolic process;transport	mitochondrion	catalytic activity	0.116	0.116	1.00
P00045	Cytochrome c iso-2 [OS=Saccharomyces cerevisiae S288c]	856672	CYC7; YEL039C	metabolic process;transport	mitochondrion	metal ion binding	0.259	0.259	1.00
P49954	probable hydrolase NIT3 [OS=Saccharomyces cerevisiae S288c]	851065	NIT3; YLR351C	metabolic process	cytoplasm;mitochondrion	catalytic activity	0.129	0.129	1.00
P36064	COX assembly mitochondrial protein [OS=Saccharomyces cerevisiae S288c]	853721	CMC1; YKL137W	cell organization and biogenesis	membrane;mitochondrion	metal ion binding	0.389	0.389	1.00
P38152	Tricarboxylate transport protein [OS=Saccharomyces cerevisiae S288c]	852594	CTP1; YBR291C	metabolic process;transport	membrane;mitochondrion	structural molecule activity;transporter activity	0.245	0.245	1.00
P53724	54S ribosomal protein L50, mitochondrial [OS=Saccharomyces cerevisiae	855756	MRPL50; YNR022C	cell organization and biogenesis;metabolic process	mitochondrion;ribosome	structural molecule activity	0.585	0.585	1.00

	S288c]								
Q06698	Putative ATP-dependent RNA helicase YLR419W [OS=Saccharomyces cerevisiae S288c]	851137	YLR419W; YLR419W	metabolic process	cytoplasm;mitochondrion;nucleus	catalytic activity;nucleotide binding;protein binding;RNA binding	0.027	0.027	1.00
P25578	CDP-diacylglycerol--glycerol-3-phosphate 3-phosphatidyltransferase [OS=Saccharomyces cerevisiae S288c]	850352	PGS1; YCL004W	metabolic process	mitochondrion	catalytic activity;nucleotide binding	0.083	0.083	1.00
Q04487	Mitochondrial inner membrane protein SHH3 [OS=Saccharomyces cerevisiae S288c]	855145	SHH3; YMR118C	metabolic process	membrane;mitochondrion	catalytic activity;metal ion binding	0.194	0.194	1.00
P20967	2-oxoglutarate dehydrogenase, mitochondrial [OS=Saccharomyces cerevisiae S288c]	854681	KGD1; YIL125W	metabolic process	cytosol;mitochondrion;organelle lumen	catalytic activity	13.785	14.441	1.05
P32843	Mitochondrial escape protein 2 [OS=Saccharomyces cerevisiae S288c]	855348	YME2; YMR302C	cell organization and biogenesis;metabolic process	membrane;mitochondrion	nucleotide binding;RNA binding	2.227	2.36	1.06
P16387	Pyruvate dehydrogenase E1 component subunit alpha, mitochondrial [OS=Saccharomyces cerevisiae S288c]	856925	PDA1; YER178W	cell growth;metabolic process	mitochondrion;organelle lumen	catalytic activity	11.496	12.46	1.08
P09440	C-1-tetrahydrofolate synthase, mitochondrial [OS=Saccharomyces cerevisiae S288c]	852378	MIS1; YBR084W	metabolic process	cytosol;mitochondrion	catalytic activity;nucleotide binding	0.762	0.829	1.09
Q01852	Mitochondrial import inner membrane translocase subunit TIM44 [OS=Saccharomyces cerevisiae S288c]	854790	TIM44; YIL022W	transport	membrane;mitochondrion	nucleotide binding;protein binding	5.898	6.43	1.09
P07275	Delta-1-pyrroline-5-carboxylate dehydrogenase, mitochondrial [OS=Saccharomyces cerevisiae S288c]	856432	PUT2; YHR037W	metabolic process	membrane;mitochondrion;organelle lumen	catalytic activity	3.977	4.337	1.09
P07257	Cytochrome b-c1 complex subunit 2, mitochondrial [OS=Saccharomyces cerevisiae S288c]	856321	QCR2; YPR191W	metabolic process;transport	membrane;mitochondrion	catalytic activity;metal ion binding;protein binding;transporter activity	27.943	30.623	1.10
P36013	NAD-dependent malic enzyme, mitochondrial [OS=Saccharomyces cerevisiae S288c]	853839	MAE1; YKL029C	metabolic process	mitochondrion;organelle lumen	catalytic activity;metal ion binding;nucleotide binding	1.371	1.512	1.10
P00927	Threonine dehydratase, mitochondrial [OS=Saccharomyces cerevisiae S288c]	856819	ILV1; YER086W	metabolic process	cytoplasm;mitochondrion	catalytic activity	1.285	1.424	1.11
P38077	ATP synthase subunit gamma, mitochondrial [OS=Saccharomyces cerevisiae S288c]	852327	ATP3; YBR039W	metabolic process;transport	membrane;mitochondrion	catalytic activity;transporter activity	11.328	12.689	1.12
P39925	Mitochondrial respiratory chain complexes assembly protein AFG3 [OS=Saccharomyces cerevisiae S288c]	856737	AFG3; YER017C	cell organization and biogenesis;metabolic process;response to stimulus;transport	membrane;mitochondrion	catalytic activity;metal ion binding;nucleotide binding;protein binding	0.734	0.823	1.12
P48015	Aminomethyltransferase, mitochondrial [OS=Saccharomyces cerevisiae S288c]	851582	GCV1; YDR019C	metabolic process	mitochondrion	catalytic activity;protein binding	6.406	7.185	1.12
P32898	Mitochondrial presequence protease [OS=Saccharomyces cerevisiae S288c]	852041	CYM1; YDR430C	metabolic process	mitochondrion	catalytic activity;metal ion binding	0.45	0.505	1.12
P47127	Altered inheritance of mitochondria protein 24, mitochondrial [OS=Saccharomyces cerevisiae S288c]	853543	AIM24; YJR080C	cell organization and biogenesis	mitochondrion		1.807	2.039	1.13
P38071	Enoyl-[acyl-carrier protein] reductase [NADPH, B-specific], mitochondrial [OS=Saccharomyces cerevisiae S288c]	852314	ETR1; YBR026C	metabolic process	mitochondrion;organelle lumen	catalytic activity;DNA binding;metal ion binding	4.926	5.579	1.13
P0CS90	Heat shock protein SSC1, mitochondrial [OS=Saccharomyces cerevisiae S288c]	853503	SSC1; YJR045C	cell organization and biogenesis;metabolic process;regulation of biological process;transport	membrane;mitochondrion;nucleus;organelle lumen	catalytic activity;enzyme regulator activity;nucleotide binding;protein binding	34.938	39.842	1.14

P09457	ATP synthase subunit 5, mitochondrial [OS=Saccharomyces cerevisiae S288c]	851892	ATP5; YDR298C	metabolic process;transport	membrane;mitochondrion	catalytic activity;transporter activity	9	10.365	1.15
Q08822	Probable electron transfer flavoprotein-ubiquinone oxidoreductase, mitochondrial [OS=Saccharomyces cerevisiae S288c]	854538	CIR2; YOR356W	metabolic process;transport	membrane;mitochondrion	catalytic activity;metal ion binding;nucleotide binding	1.873	2.162	1.15
Q08179	Mitochondrial distribution and morphology protein 38 [OS=Saccharomyces cerevisiae S288c]	854130	MDM38; YOL027C	cell organization and biogenesis;cellular homeostasis;regulation of biological process;transport	membrane;mitochondrion		3.732	4.309	1.15
P33311	ATP-dependent permease MDL2, mitochondrial [OS=Saccharomyces cerevisiae S288c]	855858	MDL2; YPL270W	response to stimulus;transport	membrane;mitochondrion	catalytic activity;nucleotide binding;transporter activity	0.535	0.619	1.16
P40215	External NADH-ubiquinone oxidoreductase 1, mitochondrial [OS=Saccharomyces cerevisiae S288c]	855176	NDE1; YMR145C	metabolic process	mitochondrion	catalytic activity;nucleotide binding	4.367	5.078	1.16
Q08023	Protein FMP25, mitochondrial [OS=Saccharomyces cerevisiae S288c]	850766	FMP25; YLR077W	cell organization and biogenesis	membrane;mitochondrion		0.719	0.84	1.17
P21954	Isocitrate dehydrogenase [NADP], mitochondrial [OS=Saccharomyces cerevisiae S288c]	851493	IDP1; YDL066W	metabolic process	mitochondrion	catalytic activity;metal ion binding;nucleotide binding;protein binding	26.123	30.623	1.17
P37292	Serine hydroxymethyltransferase, mitochondrial [OS=Saccharomyces cerevisiae S288c]	852565	SHM1; YBR263W	metabolic process	mitochondrion	catalytic activity	5.136	6.055	1.18
P53206	putative cysteine synthase [OS=Saccharomyces cerevisiae S288c]	852895	YGR012W; YGR012W	metabolic process	mitochondrion	catalytic activity	0.978	1.154	1.18
P43567	alanine--glyoxylate aminotransferase 1 [OS=Saccharomyces cerevisiae S288c]	850514	AGX1; YFL030W	metabolic process	cytosol;mitochondrion	catalytic activity	1.154	1.371	1.19
P00924	Enolase 1 [OS=Saccharomyces cerevisiae S288c]	853169	ENO1; YGR254W	metabolic process;regulation of biological process	cytoplasm;cytosol;membrane;mitochondrion;vacuole	catalytic activity;metal ion binding;protein binding	0.711	0.848	1.19
P32317	Protein AFG1 [OS=Saccharomyces cerevisiae S288c]	856658	AFG1; YEL052W	cell organization and biogenesis;metabolic process;response to stimulus;transport	membrane;mitochondrion	catalytic activity;nucleotide binding	0.711	0.848	1.19
P32787	Mitochondrial genome maintenance protein MGM101 [OS=Saccharomyces cerevisiae S288c]	853609	MGM101; YJR144W	cell organization and biogenesis;metabolic process;response to stimulus	chromosome;mitochondrion	DNA binding	1.976	2.36	1.19
P30902	ATP synthase subunit d, mitochondrial [OS=Saccharomyces cerevisiae S288c]	853853	ATP7; YKL016C	metabolic process;transport	membrane;mitochondrion	catalytic activity;transporter activity	4.179	5.105	1.22
P54783	D-arabinono-1,4-lactone oxidase [OS=Saccharomyces cerevisiae S288c]	854888	ALO1; YML086C	metabolic process;response to stimulus	membrane;mitochondrion	catalytic activity;nucleotide binding	2.415	2.981	1.23
P40047	Aldehyde dehydrogenase 5, mitochondrial [OS=Saccharomyces cerevisiae S288c]	856804	ALD5; YER073W	metabolic process	mitochondrion;organelle lumen	catalytic activity	1.581	1.955	1.24
P40053	Altered inheritance of mitochondria protein 9, mitochondrial [OS=Saccharomyces cerevisiae S288c]	856813	AIM9; YER080W		mitochondrion		1.938	2.403	1.24
Q06668	Methyltransferase OMS1, mitochondrial [OS=Saccharomyces cerevisiae S288c]	851911	OMS1; YDR316W	metabolic process	membrane;mitochondrion	catalytic activity	0.389	0.484	1.24
P08466	mitochondrial nuclease [OS=Saccharomyces cerevisiae S288c]	853222	NUC1; YJL208C	cell death;metabolic process	membrane;mitochondrion;nucleus	catalytic activity;metal ion binding	1.069	1.336	1.25
P04806	Hexokinase-1 [OS=Saccharomyces cerevisiae S288c]	850614	HXK1; YFR053C	cellular homeostasis;metabolic process;transport	cytoplasm;cytosol;mitochondrion	catalytic activity;nucleotide binding	0.487	0.61	1.25
P32860	NifU-like protein, mitochondrial [OS=Saccharomyces cerevisiae S288c]	853826	NFU1; YKL040C	cell organization and biogenesis;cellular homeostasis;metabolic process	mitochondrion;organelle lumen	metal ion binding	1.371	1.738	1.27
P49367	Homoaconitase, mitochondrial [OS=Saccharomyces cerevisiae S288c]	851820	LYS4; YDR234W	metabolic process	mitochondrion	catalytic activity;metal ion binding;protein	0.73	0.931	1.28

						binding			
P37293	N-terminal acetyltransferase 2 [OS=Saccharomyces cerevisiae S288c]	853050	NAT2; YGR147C	metabolic process	cytoplasm;mitochondrion	catalytic activity	0.73	0.931	1.28
P36775	Lon protease homolog, mitochondrial [OS=Saccharomyces cerevisiae S288c]	852259	PIM1; YBL022C	cell organization and biogenesis;metabolic process;regulation of biological process;response to stimulus	cytoplasm;mitochondrion;organelle lumen	catalytic activity;DNA binding;nucleotide binding;RNA binding	0.537	0.685	1.28
P25719	Peptidyl-prolyl cis-trans isomerase C, mitochondrial [OS=Saccharomyces cerevisiae S288c]	854897	CPR3; YML078W	cell death;metabolic process	mitochondrion;organelle lumen	catalytic activity	1.512	1.929	1.28
P38127	mitochondrial carrier protein RIM2 [OS=Saccharomyces cerevisiae S288c]	852491	RIM2; YBR192W	cell organization and biogenesis;metabolic process;transport	membrane;mitochondrion	structural molecule activity;transporter activity	0.778	0.995	1.28
Q02981	ABC1 family protein YPL109C, mitochondrial [OS=Saccharomyces cerevisiae S288c]	855995	YPL109C; YPL109C		mitochondrion		0.259	0.334	1.29
P04840	Mitochondrial outer membrane protein porin 1 [OS=Saccharomyces cerevisiae S288c]	855669	POR1; YNL055C	cell death;cell organization and biogenesis;cellular homeostasis;regulation of biological process;transport	cytoplasm;membrane;mitochondrion	transporter activity	17.33	22.357	1.29
P33310	ATP-dependent permease MDL1, mitochondrial [OS=Saccharomyces cerevisiae S288c]	850885	MDL1; YLR188W	transport	membrane;mitochondrion	catalytic activity;nucleotide binding;transporter activity	0.274	0.354	1.29
P36060	NADH-cytochrome b5 reductase 2 [OS=Saccharomyces cerevisiae S288c]	853707	MCR1; YKL150W	metabolic process;response to stimulus	membrane;mitochondrion	catalytic activity;protein binding	13.384	17.33	1.29
P53598	Succinyl-CoA ligase [ADP-forming] subunit alpha, mitochondrial [OS=Saccharomyces cerevisiae S288c]	854310	LSC1; YOR142W	metabolic process	cytosol;mitochondrion	catalytic activity;nucleotide binding	9	11.743	1.30
P04710	ADP,ATP carrier protein 1 [OS=Saccharomyces cerevisiae S288c]	855078	AAC1; YMR056C	metabolic process;transport	cytosol;membrane;mitochondrion	structural molecule activity;transporter activity	6.848	9	1.31
P32454	Aminopeptidase 2, mitochondrial [OS=Saccharomyces cerevisiae S288c]	853699	APE2; YKL157W	metabolic process	cytoplasm;extracellular;membrane;mitochondrion;nucleus	catalytic activity;metal ion binding	1.049	1.383	1.32
P15424	ATP-dependent RNA helicase MSS116, mitochondrial [OS=Saccharomyces cerevisiae S288c]	851775	MSS116; YDR194C	metabolic process;regulation of biological process	mitochondrion;organelle lumen	catalytic activity;nucleotide binding;RNA binding	0.506	0.668	1.32
P07256	Cytochrome b-c1 complex subunit 1, mitochondrial [OS=Saccharomyces cerevisiae S288c]	852235	COR1; YBL045C	metabolic process;transport	membrane;mitochondrion	catalytic activity;metal ion binding;protein binding;transporter activity	8.183	10.86	1.33
P36534	54S ribosomal protein L40, mitochondrial [OS=Saccharomyces cerevisiae S288c]	855930	MRPL40; YPL173W	cell organization and biogenesis;metabolic process	mitochondrion;ribosome	protein binding;structural molecule activity	0.585	0.778	1.33
P28625	Protein YIM1 [OS=Saccharomyces cerevisiae S288c]	855183	YIM1; YMR152W	metabolic process;response to stimulus	cytoplasm;endoplasmic reticulum;mitochondrion	catalytic activity;metal ion binding	0.624	0.833	1.33
P36521	54S ribosomal protein L11, mitochondrial [OS=Saccharomyces cerevisiae S288c]	851325	MRPL11; YDL202W	cell organization and biogenesis;metabolic process	mitochondrion;ribosome	structural molecule activity	0.624	0.833	1.33
P02992	elongation factor Tu, mitochondrial [OS=Saccharomyces cerevisiae S288c]	854359	TUF1; YOR187W	cell organization and biogenesis;metabolic process	mitochondrion	catalytic activity;nucleotide binding;RNA binding	6.667	9	1.35
P32939	GTP-binding protein ypt7 [OS=Saccharomyces cerevisiae S288c]	855012	YPT7; YML001W	cell communication;cell organization and biogenesis;metabolic process;regulation of biological process;response to stimulus;transport	cytosol;endosome;membrane;mitochondrion;vacuole	catalytic activity;nucleotide binding;protein binding	0.848	1.154	1.36
P36525	54S ribosomal protein L24, mitochondrial [OS=Saccharomyces cerevisiae S288c]	855231	MRPL24; YMR193W	cell organization and biogenesis;metabolic process	mitochondrion;ribosome	RNA binding;structural molecule activity	0.848	1.154	1.36
P39987	Heat shock protein SSC3, mitochondrial [OS=Saccharomyces cerevisiae S288c]	856682	ECM10; YEL030W	cell organization and biogenesis;metabolic process;transport	mitochondrion	nucleotide binding;protein binding	1.198	1.637	1.37
Q05931	Heat shock protein SSQ1, mitochondrial [OS=Saccharomyces cerevisiae S288c]	851084	SSQ1; YLR369W	cell organization and biogenesis;metabolic process	mitochondrion;organelle lumen	nucleotide binding;protein binding	0.496	0.679	1.37

Q12298	uncharacterized ABC transporter ATP-binding protein YDR061W [OS=Saccharomyces cerevisiae S288c]	851633	YDR061W; YDR061W		mitochondrion	catalytic activity;nucleotide binding	0.194	0.266	1.37
P53266	Cytochrome oxidase assembly protein SHY1 [OS=Saccharomyces cerevisiae S288c]	853009	SHY1; YGR112W	cell organization and biogenesis	membrane;mitochondrion	protein binding	0.978	1.346	1.38
P32799	Cytochrome c oxidase subunit 6A, mitochondrial [OS=Saccharomyces cerevisiae S288c]	852684	COX13; YGL191W	cell organization and biogenesis;metabolic process;regulation of biological process;transport	membrane;mitochondrion	catalytic activity;enzyme regulator activity;transporter activity	2.162	2.981	1.38
Q06567	ABC1 family protein MCP2 [OS=Saccharomyces cerevisiae S288c]	850955	YLR253W; MCP2; YLR253W	cell organization and biogenesis	membrane;mitochondrion		0.218	0.301	1.38
P21375	Fumarate reductase 2 [OS=Saccharomyces cerevisiae S288c]	853510	OSM1; YJR051W	metabolic process	endoplasmic reticulum;mitochondrion	catalytic activity;nucleotide binding	0.25	0.346	1.38
P36066	Protein MRG3-like [OS=Saccharomyces cerevisiae S288c]	853725	YKL133C; YKL133C	metabolic process	membrane;mitochondrion	protein binding	0.241	0.334	1.39
P47052	succinate dehydrogenase [ubiquinone] flavoprotein subunit 2, mitochondrial [OS=Saccharomyces cerevisiae S288c]	853405	YJL045W; YJL045W	metabolic process;transport	membrane;mitochondrion	catalytic activity;nucleotide binding	2.695	3.739	1.39
P34231	Uncharacterized protein YKL187C [OS=Saccharomyces cerevisiae S288c]	853648	FAT3; YKL187C; YKL187C	cell organization and biogenesis;transport	membrane;mitochondrion		0.304	0.425	1.40
P36101	tRNA threonylcarbamoyladenosine dehydratase 2 [OS=Saccharomyces cerevisiae S288c]	853841	TCD2; YKL027W; YKL027W	metabolic process	cytosol;membrane;mitochondrion	catalytic activity;nucleotide binding	0.334	0.468	1.40
P47015	Altered inheritance of mitochondria protein 23, mitochondrial [OS=Saccharomyces cerevisiae S288c]	853310	AIM23; YJL131C	metabolic process	mitochondrion	RNA binding	0.35	0.492	1.41
P53326	Uncharacterized protein YGR266W [OS=Saccharomyces cerevisiae S288c]	853182	YGR266W; YGR266W		membrane;mitochondrion		0.369	0.52	1.41
P40012	protoporphyrinogen oxidase [OS=Saccharomyces cerevisiae S288c]	856733	HEM14; YER014W	metabolic process	membrane;mitochondrion	catalytic activity	0.947	1.336	1.41
P25605	Acetolactate synthase small subunit, mitochondrial [OS=Saccharomyces cerevisiae S288c]	850348	ILV6; YCL009C	metabolic process;regulation of biological process	mitochondrion	catalytic activity;enzyme regulator activity	1.976	2.793	1.41
P50110	Sorting assembly machinery 37 kDa subunit [OS=Saccharomyces cerevisiae S288c]	855082	SAM37; YMR060C	cell organization and biogenesis;transport	membrane;mitochondrion	protein binding	0.389	0.551	1.42
P43617	Uncharacterized mitochondrial carrier YFR045W [OS=Saccharomyces cerevisiae S288c]	850606	YFR045W; YFR045W	metabolic process;transport	membrane;mitochondrion	structural molecule activity;transporter activity	0.413	0.585	1.42
P38300	Inner membrane mitoribosome receptor MBA1, mitochondrial [OS=Saccharomyces cerevisiae S288c]	852483	MBA1; YBR185C	cell organization and biogenesis;regulation of biological process	membrane;mitochondrion		0.413	0.585	1.42
P38825	Protein TOM71 [OS=Saccharomyces cerevisiae S288c]	856517	TOM71; YHR117W	transport	membrane;mitochondrion	protein binding;transporter activity	0.425	0.604	1.42
P32904	54S ribosomal protein L6, mitochondrial [OS=Saccharomyces cerevisiae S288c]	856552	MRPL6; YHR147C	cell organization and biogenesis;metabolic process	mitochondrion;ribosome	RNA binding;structural molecule activity	0.54	0.778	1.44
Q02776	Mitochondrial import inner membrane translocase subunit TIM50 [OS=Saccharomyces cerevisiae S288c]	856042	TIM50; YPL063W	cell organization and biogenesis;metabolic process;transport	membrane;mitochondrion	catalytic activity;protein binding;transporter activity	6.197	9	1.45
P37298	Succinate dehydrogenase [ubiquinone] cytochrome b small subunit, mitochondrial [OS=Saccharomyces cerevisiae S288c]	851758	SDH4; YDR178W	metabolic process;transport	membrane;mitochondrion	catalytic activity;metal ion binding	1.783	2.594	1.45
P19262	Dihydrolipoyllysine-residue succinyltransferase component of 2-oxoglutarate dehydrogenase complex, mitochondrial [OS=Saccharomyces cerevisiae S288c]	851726	KGD2; YDR148C	cell organization and biogenesis;metabolic process	mitochondrion	catalytic activity;protein binding	3.062	4.484	1.46
Q03201	37S ribosomal protein S10, mitochondrial [OS=Saccharomyces cerevisiae	851611	RSM10; YDR041W	cell organization and biogenesis;metabolic process	mitochondrion;ribosome	structural molecule activity	0.701	1.031	1.47

	S288c]								
Q12029	Probable mitochondrial transport protein fsf1 [OS=Saccharomyces cerevisiae S288c]	85444 5	FSF1; YOR271 C	transport	membrane;mitochondrion	transporter activity	1.336	1.976	1.48
P32332	Mitochondrial oxaloacetate transport protein [OS=Saccharomyces cerevisiae S288c]	85373 9	OAC1; YKL120 W	metabolic process;transport	membrane;mitochondrion	structural molecule activity;transpor ter activity	1.448	2.162	1.49
P32266	Dynamin-like GTPase MGM1, mitochondrial [OS=Saccharomyces cerevisiae S288c]	85438 6	MGM1; YOR211 C	cell organization and biogenesis	membrane;mitochondrion	catalytic activity;nucleoti de binding;protein binding	0.086	0.131	1.52
P28817	3-hydroxyisobutyryl-CoA hydrolase, mitochondrial [OS=Saccharomyces cerevisiae S288c]	85160 6	EHD3; YDR036 C	metabolic process	mitochondrion	catalytic activity	0.487	0.743	1.53
P39952	Mitochondrial inner membrane protein OXA1 [OS=Saccharomyces cerevisiae S288c]	85689 8	OXA1; YER154 W	cell organization and biogenesis;transport	membrane;mitochondrion	transporter activity	1.738	2.652	1.53
P11325	Leucine--tRNA ligase, mitochondrial [OS=Saccharomyces cerevisiae S288c]	85109 8	NAM2; YLR382C	cell organization and biogenesis;metabolic process;regulation of biological process	cytosol;mitochondrion;organelle lumen	catalytic activity;nucleoti de binding;RNA binding	0.078	0.12	1.54
P21560	Protein CBP3, mitochondrial [OS=Saccharomyces cerevisiae S288c]	85588 6	CBP3; YPL215 W	cell organization and biogenesis;regulation of biological process	membrane;mitochondrion;ribosome		0.557	0.859	1.54
P32445	Single-stranded DNA-binding protein RIM1, mitochondrial [OS=Saccharomyces cerevisiae S288c]	85039 5	RIM1; YCR028C -A	cell organization and biogenesis;metabolic process;regulation of biological process	mitochondrion	DNA binding	1.154	1.783	1.55
P25349	Flavoprotein-like protein YCP4 [OS=Saccharomyces cerevisiae S288c]	85036 0	YCP4; YCR004C	metabolic process;regulation of biological process	cytoplasm;membrane;mitochondrion	catalytic activity;nucleoti de binding;protein binding	5.813	9	1.55
P53166	ATP-dependent RNA helicase mrh4, mitochondrial [OS=Saccharomyces cerevisiae S288c]	85281 6	MRH4; YGL064C	cell organization and biogenesis;metabolic process	mitochondrion;organelle lumen	catalytic activity;nucleoti de binding;RNA binding	0.145	0.225	1.55
P42900	Sigma-like sequence protein 1, mitochondrial [OS=Saccharomyces cerevisiae S288c]	85083 0	SLS1; YLR139C	cell organization and biogenesis;metabolic process	membrane;mitochondrion	protein binding	0.125	0.194	1.55
P07236	Threonine--tRNA ligase, mitochondrial [OS=Saccharomyces cerevisiae S288c]	85364 0	MST1; YKL194C	metabolic process	cytoplasm;mitochondrion;organelle lumen	catalytic activity;nucleoti de binding;RNA binding	0.15	0.233	1.55
P39518	Long-chain-fatty-acid--CoA ligase 2 [OS=Saccharomyces cerevisiae S288c]	85673 4	FAA2; YER015 W	metabolic process;transport	cytoplasm;mitochondrion	catalytic activity;nucleoti de binding	0.455	0.708	1.56
P25039	Elongation factor G, mitochondrial [OS=Saccharomyces cerevisiae S288c]	85075 8	MEF1; YLR069C	cell organization and biogenesis;metabolic process	cytosol;mitochondrion	catalytic activity;nucleoti de binding;RNA binding	0.207	0.326	1.57
P38702	mitochondrial carrier protein LEU5 [OS=Saccharomyces cerevisiae S288c]	85639 1	LEU5; YHR002 W	metabolic process;transport	membrane;mitochondrion	structural molecule activity;transpor ter activity	0.212	0.334	1.58
Q06236	Mitochondrial inner membrane protein SHH4 [OS=Saccharomyces cerevisiae S288c]	85086 1	SHH4; YLR164 W	metabolic process	membrane;mitochondrion	catalytic activity;metal ion binding	1.371	2.162	1.58
P38797	Protein phosphatase 2C homolog 7, mitochondrial [OS=Saccharomyces cerevisiae S288c]	85647 5	PTC7; YHR076 W	metabolic process	mitochondrion	catalytic activity;metal ion binding;protein binding	0.233	0.369	1.58
P15179	Aspartate--tRNA ligase, mitochondrial [OS=Saccharomyces cerevisiae S288c]	85600 0	MSD1; YPL104 W	metabolic process	cytoplasm;mitochondrion;organelle lumen	catalytic activity;nucleoti de binding	0.239	0.379	1.59
P36520	54S ribosomal protein L10, mitochondrial [OS=Saccharomyces cerevisiae S288c]	85543 6	MRPL10; YNL284C	cell organization and biogenesis;metabolic process	mitochondrion;ribosome	structural molecule activity	0.245	0.389	1.59
Q08968	UPF0061 protein FMP40 [OS=Saccharomyces cerevisiae S288c]	85587 9	FMP40; YPL222 W		mitochondrion	protein binding	0.917	1.462	1.59
P18496	Mitochondrial ATPase complex subunit ATP10 [OS=Saccharomyces cerevisiae S288c]	85110 9	ATP10; YLR393 W	cell organization and biogenesis	cytoplasm;membrane;mitochondrion	protein binding	0.259	0.413	1.59
P27697	Atypical kinase COQ8, mitochondrial [OS=Saccharomyces cerevisiae S288c]	85275 8	COQ8; YGL119 W	metabolic process	membrane;mitochondrion;organelle lumen	catalytic activity;nucleoti de binding	0.283	0.453	1.60

P40035	Mitochondrial phosphate carrier protein 2 [OS=Saccharomyces cerevisiae S288c]	856779	PIC2; YER053C	cellular homeostasis;metabolic process;transport	membrane;mitochondrion	structural molecule activity;transporter activity	0.833	1.336	1.60
P46681	D-lactate dehydrogenase [cytochrome] 2, mitochondrial [OS=Saccharomyces cerevisiae S288c]	851376	DLD2; YDL178W	metabolic process	mitochondrion;organelle lumen	catalytic activity;nucleotide binding;protein binding	0.682	1.102	1.62
P05626	ATP synthase subunit 4, mitochondrial [OS=Saccharomyces cerevisiae S288c]	856027	ATP4; YPL078C	cell organization and biogenesis;metabolic process;transport	membrane;mitochondrion	catalytic activity;transporter activity	3.084	4.995	1.62
Q03246	37S ribosomal protein S17, mitochondrial [OS=Saccharomyces cerevisiae S288c]	855226	MRPS17; YMR188C	cell differentiation;cell organization and biogenesis;metabolic process	mitochondrion;ribosome	RNA binding;structural molecule activity	0.389	0.638	1.64
Q06678	54S ribosomal protein L35, mitochondrial [OS=Saccharomyces cerevisiae S288c]	851921	MRPL35; YDR322W	cell organization and biogenesis;metabolic process	mitochondrion;ribosome	structural molecule activity	0.425	0.701	1.65
P53311	Mitochondrial pyruvate carrier 3 [OS=Saccharomyces cerevisiae S288c]	853158	FMP43; MPC3; YGR243W	transport	membrane;mitochondrion	transporter activity	0.468	0.778	1.66
Q12032	Altered inheritance of mitochondria protein 41, mitochondrial [OS=Saccharomyces cerevisiae S288c]	854390	AIM41; YOR215C		mitochondrion	catalytic activity	0.468	0.778	1.66
P27929	37S ribosomal protein NAM9, mitochondrial [OS=Saccharomyces cerevisiae S288c]	855585	NAM9; YNL137C	cell organization and biogenesis;metabolic process;regulation of biological process	mitochondrion;ribosome	RNA binding;structural molecule activity	0.484	0.808	1.67
P36517	54S ribosomal protein L4, mitochondrial [OS=Saccharomyces cerevisiae S288c]	851160	MRPL4; YLR439W	cell organization and biogenesis;metabolic process	mitochondrion;ribosome	structural molecule activity	0.52	0.874	1.68
P37267	Assembly factor cbp4 [OS=Saccharomyces cerevisiae S288c]		CBP4	cell organization and biogenesis	membrane;mitochondrion		0.52	0.874	1.68
Q02784	Monothiol glutaredoxin-5, mitochondrial [OS=Saccharomyces cerevisiae S288c]	856048	GRX5; YPL059W	cell organization and biogenesis;cellular homeostasis;metabolic process;regulation of biological process;response to stimulus	mitochondrion;organelle lumen	catalytic activity;metal ion binding	0.668	1.154	1.73
P06169	pyruvate decarboxylase isozyme 1 [OS=Saccharomyces cerevisiae S288c]	850733	PDC1; YLR044C	metabolic process	cytoplasm;cytosol;nucleus	catalytic activity;metal ion binding	1.043	1.807	1.73
P38771	Ribosome-recycling factor, mitochondrial [OS=Saccharomyces cerevisiae S288c]	856433	RRF1; YHR038W	cell organization and biogenesis;metabolic process	mitochondrion	RNA binding	0.719	1.254	1.74
Q05892	MIOREX complex component 2 [OS=Saccharomyces cerevisiae S288c]	850997	YLR290C; COQ11; YLR290C	metabolic process	mitochondrion	catalytic activity	0.778	1.371	1.76
Q12233	ATP synthase subunit g, mitochondrial [OS=Saccharomyces cerevisiae S288c]	856131	ATP20; YPR020W	cell organization and biogenesis;metabolic process;transport	membrane;mitochondrion	catalytic activity;transporter activity	0.778	1.371	1.76
P81449	ATP synthase subunit e, mitochondrial [OS=Saccharomyces cerevisiae S288c]	851922	TIM11; YDR322C-A	cell organization and biogenesis;metabolic process;transport	membrane;mitochondrion	catalytic activity;structural molecule activity;transporter activity	0.778	1.371	1.76
P40098	uncharacterized mitochondrial membrane protein FMP10 [OS=Saccharomyces cerevisiae S288c]	856931	FMP10; YER182W		membrane;mitochondrion		1.336	2.36	1.77
Q96VH5	MICOS complex subunit Mic10 [OS=Saccharomyces cerevisiae S288c]	850300	MOS1; MIC10; YCL057C-A	cell organization and biogenesis	membrane;mitochondrion		0.931	1.683	1.81
P36516	54S ribosomal protein L3, mitochondrial [OS=Saccharomyces cerevisiae S288c]	855039	MRPL3; YMR024W	cell organization and biogenesis;metabolic process;response to stimulus	mitochondrion;nucleus;ribosome	catalytic activity;RNA binding;structural molecule activity	0.269	0.487	1.81
P00128	Cytochrome b-c1 complex subunit 7 [OS=Saccharomyces cerevisiae S288c]	852142	QCR7; YDR529C	cell organization and biogenesis;metabolic process;transport	membrane;mitochondrion	catalytic activity;transporter activity	3.642	6.743	1.85
P00427	Cytochrome c oxidase subunit 6, mitochondrial [OS=Saccharomyces cerevisiae S288c]	856448	COX6; YHR051W	metabolic process;transport	membrane;mitochondrion	catalytic activity;metal ion binding;transporter activity	1.154	2.162	1.87
P12686	37S ribosomal protein MRP13, mitochondrial [OS=Saccharomyces cerevisiae S288c]	852975	MRP13; YGR084C	cell organization and biogenesis;metabolic process	mitochondrion;ribosome	structural molecule activity	0.413	0.778	1.88

Q01574	acetyl-coenzyme A synthetase 1 [OS=Saccharomyces cerevisiae S288c]	851245	ACS1; YAL054C	cell organization and biogenesis;metabolic process	cytoplasm;cytosol;endoplasmic reticulum;mitochondrion;nucleus	catalytic activity;nucleotide binding	1.482	2.793	1.88
Q12289	mitochondrial carnitine carrier [OS=Saccharomyces cerevisiae S288c]	854267	CRC1; YOR100C	metabolic process;transport	membrane;mitochondrion	structural molecule activity;transporter activity	0.73	1.404	1.92
Q03104	Meiotic sister chromatid recombination protein 1 [OS=Saccharomyces cerevisiae S288c]	854911	MSC1; YML128C	metabolic process	endoplasmic reticulum;membrane;mitochondrion		1.712	3.299	1.93
Q02950	37S ribosomal protein MRP51, mitochondrial [OS=Saccharomyces cerevisiae S288c]	855985	MRP51; YPL118W	cell organization and biogenesis;metabolic process	mitochondrion;ribosome	structural molecule activity	0.407	0.817	2.01
P40008	Protein FMP52, mitochondrial [OS=Saccharomyces cerevisiae S288c]	856721	FMP52; YER004W		endoplasmic reticulum;membrane;mitochondrion	catalytic activity	1.054	2.162	2.05
P53170	[Pyruvate dehydrogenase (acetyl-transferring)] kinase 2, mitochondrial [OS=Saccharomyces cerevisiae S288c]	852821	PKP2; YGL059W	metabolic process;regulation of biological process	mitochondrion;organelle lumen	catalytic activity;nucleotide binding;protein binding	0.086	0.179	2.08
Q06510	Lysophosphatidylcholine acyltransferase [OS=Saccharomyces cerevisiae S288c]	856262	TAZ1; YPR140W	cell organization and biogenesis;metabolic process;transport	membrane;mitochondrion	catalytic activity	0.093	0.194	2.09
P38909	cytochrome c mitochondrial import factor CYC2 [OS=Saccharomyces cerevisiae S288c]	854202	CYC2; YOR037W	cell organization and biogenesis;metabolic process	membrane;mitochondrion	catalytic activity	0.093	0.194	2.09
P41911	Glycerol-3-phosphate dehydrogenase [NAD(+)] 2, mitochondrial [OS=Saccharomyces cerevisiae S288c]	854095	GPD2; YOL059W	metabolic process	cytoplasm;cytosol;mitochondrion	catalytic activity;nucleotide binding;protein binding	0.089	0.186	2.09
P53736	Uncharacterized protein YNR040W [OS=Saccharomyces cerevisiae S288c]	855776	YNR040W; YNR040W		mitochondrion		0.116	0.245	2.11
P36528	54S ribosomal protein L17, mitochondrial [OS=Saccharomyces cerevisiae S288c]	855469	MRPL17; YNL252C	cell organization and biogenesis;metabolic process	mitochondrion;ribosome	structural molecule activity	0.11	0.233	2.12
P02381	Ribosomal protein VAR1, mitochondrial [OS=Saccharomyces cerevisiae S288c]	854586	VAR1; Q0140	cell organization and biogenesis;metabolic process	mitochondrion;ribosome	structural molecule activity	0.11	0.233	2.12
P54857	lipase 2 [OS=Saccharomyces cerevisiae S288c]	851628	TGL2; YDR058C	metabolic process;transport	mitochondrion	catalytic activity	0.11	0.233	2.12
P39722	Mitochondrial Rho GTPase 1 [OS=Saccharomyces cerevisiae S288c]	851249	GEM1; YAL048C	cell organization and biogenesis;regulation of biological process;response to stimulus;transport	membrane;mitochondrion	catalytic activity;metal ion binding;nucleotide binding	0.122	0.259	2.12
P36139	protein PET10 [OS=Saccharomyces cerevisiae S288c]	853920	PET10; YKR046C	metabolic process	membrane		0.122	0.259	2.12
P09201	fructose-1,6-bisphosphatase [OS=Saccharomyces cerevisiae S288c]	851092	FBP1; YLR377C	metabolic process	cytosol	catalytic activity;metal ion binding	0.129	0.274	2.12
P25270	rRNA methyltransferase 1, mitochondrial [OS=Saccharomyces cerevisiae S288c]	854376	MRM1; YOR201C	metabolic process	mitochondrion	catalytic activity;RNA binding	0.129	0.274	2.12
P53982	isocitrate dehydrogenase [NADP] [OS=Saccharomyces cerevisiae S288c]	855723	IDP3; YNL009W	metabolic process	cytoplasm;mitochondrion	catalytic activity;metal ion binding;nucleotide binding	1.371	2.924	2.13
P38323	ATP-dependent clpX-like chaperone, mitochondrial [OS=Saccharomyces cerevisiae S288c]	852528	MCX1; YBR227C	metabolic process	membrane;mitochondrion;organelle lumen	catalytic activity;nucleotide binding	0.145	0.311	2.14
P38816	thioredoxin reductase 2, mitochondrial [OS=Saccharomyces cerevisiae S288c]	856506	TRR2; YHR106W	cellular homeostasis;metabolic process;regulation of biological process;response to stimulus	cytoplasm;mitochondrion	antioxidant activity;catalytic activity	0.136	0.292	2.15
Q06630	Mitochondrial homologous recombination protein 1 [OS=Saccharomyces cerevisiae S288c]	851890	MHR1; YDR296W	cell organization and biogenesis;metabolic process;regulation of biological process;response to stimulus	mitochondrion;nucleus	catalytic activity;DNA binding;structural molecule activity	0.166	0.359	2.16
Q07560	Cardiolipin synthase (CMP-forming) [OS=Saccharomyces cerevisiae S288c]	851413	CRD1; YDL142C	cell organization and biogenesis;cellular homeostasis;metabolic process	membrane;mitochondrion	catalytic activity	0.166	0.359	2.16
P40086	Cytochrome c oxidase assembly protein cox15 [OS=Saccharomyces cerevisiae S288c]	856884	COX15; YER141W	cell organization and biogenesis;metabolic process;transport	membrane;mitochondrion	catalytic activity;transporter activity	0.532	1.154	2.17

P53230	Phosphatidate cytidylyltransferase, mitochondrial [OS=Saccharomyces cerevisiae S288c]	852937	TAM41; YGR046W	metabolic process	membrane;mitochondrion;organelle lumen	catalytic activity	0.186	0.407	2.19
P23369	54S ribosomal protein L25, mitochondrial [OS=Saccharomyces cerevisiae S288c]	852967	MRPL25; YGR076C	cell organization and biogenesis;metabolic process	mitochondrion;ribosome	structural molecule activity	0.233	0.52	2.23
P40452	Cytochrome c oxidase assembly factor 1 [OS=Saccharomyces cerevisiae S288c]	854649	COA1; YIL157C	cell organization and biogenesis	membrane;mitochondrion	protein binding	0.233	0.52	2.23
P17709	Glucokinase-1 [OS=Saccharomyces cerevisiae S288c]	850317	GLK1; YCL040W	cellular homeostasis;metabolic process;transport	cytosol;membrane	catalytic activity;nucleotide binding	0.259	0.585	2.26
P23833	Protein SCO1, mitochondrial [OS=Saccharomyces cerevisiae S288c]	852325	SCO1; YBR037C	cell organization and biogenesis;cellular homeostasis;response to stimulus;transport	membrane;mitochondrion	antioxidant activity;catalytic activity;metal ion binding	0.292	0.668	2.29
Q12165	ATP synthase subunit delta, mitochondrial [OS=Saccharomyces cerevisiae S288c]	851560	ATP16; YDL004W	metabolic process;transport	membrane;mitochondrion	catalytic activity;transporter activity	0.334	0.778	2.33
P00447	Superoxide dismutase [Mn], mitochondrial [OS=Saccharomyces cerevisiae S288c]	856399	SOD2; YHR008C	metabolic process;response to stimulus	mitochondrion;organelle lumen	antioxidant activity;catalytic activity;metal ion binding	1.031	2.455	2.38
P35180	mitochondrial import receptor subunit Tom20 [OS=Saccharomyces cerevisiae S288c]	852973	TOM20; YGR082W	cell organization and biogenesis;transport	membrane;mitochondrion	protein binding;transporter activity	0.425	1.031	2.43
P40513	Mitochondrial acidic protein MAM33 [OS=Saccharomyces cerevisiae S288c]	854740	MAM33; YIL070C	metabolic process;regulation of biological process	mitochondrion;organelle lumen	translation regulator activity	1.154	2.831	2.45
P53163	54S ribosomal protein L12, mitochondrial [OS=Saccharomyces cerevisiae S288c]	852811	MNP1; YGL068W	cell organization and biogenesis;metabolic process	mitochondrion;ribosome	RNA binding;structural molecule activity	0.52	1.31	2.52
Q01217	Protein ARG5,6, mitochondrial [OS=Saccharomyces cerevisiae S288c]	856800	ARG5,6; YER069W	metabolic process;regulation of biological process	cytoplasm;mitochondrion;organelle lumen	catalytic activity;nucleotide binding;protein binding	0.087	0.233	2.68
P81451	ATP synthase subunit K, mitochondrial [OS=Saccharomyces cerevisiae S288c]	854077	ATP19; YOL077W-A	cell organization and biogenesis;metabolic process;transport	membrane;mitochondrion	catalytic activity;transporter activity	1.154	3.642	3.16
P39677	Ribosome-releasing factor 2, mitochondrial [OS=Saccharomyces cerevisiae S288c]	853342	MEF2; YJL102W	cell organization and biogenesis;metabolic process	cytosol;mitochondrion	catalytic activity;nucleotide binding;RNA binding	0.056	0.179	3.20
Q04472	Mitochondrial inner membrane i-AAA protease supercomplex subunit MGR3 [OS=Saccharomyces cerevisiae S288c]	855142	MGR3; YMR115W	metabolic process	membrane;mitochondrion	protein binding	0.072	0.233	3.24
P38756	tRNA threonylcarbamoyladenosine dehydratase 1 [OS=Saccharomyces cerevisiae S288c]	856392	TCD1; YHR003C; YHR003C	metabolic process	cytosol;membrane;mitochondrion	catalytic activity;nucleotide binding	0.093	0.304	3.27
P47039	Probable kynurenine--oxoglutarate transaminase BNA3 [OS=Saccharomyces cerevisiae S288c]	853386	BNA3; YJL060W	metabolic process;regulation of biological process	cytoplasm;mitochondrion	catalytic activity	0.101	0.334	3.31
P47140	Altered inheritance rate of mitochondria protein 25 [OS=Saccharomyces cerevisiae S288c]	853563	AIM25; YJR100C	cell organization and biogenesis	membrane;mitochondrion	transporter activity	0.11	0.369	3.35
P14693	sorting assembly machinery 35 kDa subunit [OS=Saccharomyces cerevisiae S288c]	856483	SAM35; YHR083W	cell organization and biogenesis;transport	membrane;mitochondrion	protein binding	0.155	0.54	3.48
P22353	54S ribosomal protein L8, mitochondrial [OS=Saccharomyces cerevisiae S288c]	853382	MRPL8; YJL063C	cell organization and biogenesis;metabolic process	mitochondrion;ribosome	structural molecule activity	0.166	0.585	3.52
P17695	Glutaredoxin-2, mitochondrial [OS=Saccharomyces cerevisiae S288c]	852124	GRX2; YDR513W	cellular homeostasis;metabolic process;regulation of biological process;response to stimulus;transport	cytoplasm;cytosol;mitochondrion;nucleus	antioxidant activity;catalytic activity	0.233	0.874	3.75
P39994	Putative 2-hydroxyacyl-CoA lyase [OS=Saccharomyces cerevisiae S288c]	856694	YEL020C; YEL020C		cytoplasm	catalytic activity;metal ion binding	0.083	0.374	4.51

Supplemental Table S9. The relative concentrations of proteins in mitochondria purified from *ups1Δ* cells cultured with or without LCA. Mitochondria were purified from *ups1Δ* cells recovered on day 7 of cell culturing with or without LCA. Mass spectrometry-based identification and quantitation of proteins recovered in purified mitochondria were performed as described in Materials and methods. The exponentially modified protein abundance index (emPAI) provided by the ″Proteome Discoverer″ software was used to calculate the relative abundance of mitochondrial proteins between specified datasets. Data on the relative concentrations of mitochondrial proteins are presented as means of 2 independent experiments.

Accession	Description	Entrez Gene ID	Gene ID	Biological Process	Cellular Component	Molecular Function	emPAI *ups1*	emPAI *ups1* + LCA	Ratio *ups1* + LCA / *ups1*
Q12374	Nuclear control of ATPase protein 2	856278	NCA2	metabolic process	membrane; mitochondrion		1.154	0.066	0.06
P50088	Stationary phase gene 1 protein [OS=Saccharomyces cerevisiae S288c]	853151	SPG1; YGR236C		endoplasmic reticulum;membrane;mitochondrion		250.19	14.849	0.06
P00128	Cytochrome b-c1 complex subunit 7 [OS=Saccharomyces cerevisiae S288c]	852142	QCR7; YDR529C	cell organization and biogenesis;metabolic process;transport	membrane;mitochondrion	catalytic activity;transporter activity	15.681	1.154	0.07
P37298	Succinate dehydrogenase [ubiquinone] cytochrome b small subunit, mitochondrial [OS=Saccharomyces cerevisiae S288c]	851758	SDH4; YDR178W	metabolic process;transport	membrane;mitochondrion	catalytic activity;metal ion binding	9	0.668	0.07
P16547	Mitochondrial outer membrane protein OM45 [OS=Saccharomyces cerevisiae S288c]	854670	OM45; YIL136W		membrane;mitochondrion		2088.3	189.546	0.09
P18239	ADP,ATP carrier protein 2 [OS=Saccharomyces cerevisiae S288c]	852250	PET9; YBL030C	cell death;metabolic process;transport	membrane;mitochondrion	structural molecule activity;transporter activity	591.55	64.793	0.11
P39952	Mitochondrial inner membrane protein OXA1 [OS=Saccharomyces cerevisiae S288c]	856898	OXA1; YER154W	cell organization and biogenesis;transport	membrane;mitochondrion	transporter activity	2.652	0.334	0.13
P12687	54S ribosomal protein L2, mitochondrial [OS=Saccharomyces cerevisiae S288c]	855727	MRP7; YNL005C	cell organization and biogenesis;metabolic process	mitochondrion;ribosome	catalytic activity;RNA binding;structural molecule activity	1.848	0.233	0.13
P39726	Glycine cleavage system H protein, mitochondrial [OS=Saccharomyces cerevisiae S288c]	851254	GCV3; YAL044C	metabolic process	mitochondrion	catalytic activity	20.544	2.594	0.13
P33421	Succinate dehydrogenase [ubiquinone] cytochrome b subunit, mitochondrial [OS=Saccharomyces cerevisiae S288c]	853716	SDH3; YKL141W	cell organization and biogenesis;metabolic process;transport	membrane;mitochondrion	catalytic activity;metal ion binding	1.276	0.179	0.14
P32897	Mitochondrial import inner membrane translocase subunit tim23 [OS=Saccharomyces cerevisiae S288c]	855751	TIM23; YNR017W	transport	membrane;mitochondrion	protein binding;transporter activity	4.012	0.585	0.15
P32340	Rotenone-insensitive NADH-ubiquinone oxidoreductase, mitochondrial [OS=Saccharomyces cerevisiae S288c]	854919	NDI1; YML120C	metabolic process;regulation of biological process	membrane;mitochondrion;organelle lumen	catalytic activity;nucleotide binding	61.102	9	0.15
P00424	Cytochrome c oxidase polypeptide 5A, mitochondrial [OS=Saccharomyces cerevisiae S288c]	855675	COX5A; YNL052W	metabolic process;transport	membrane;mitochondrion	catalytic activity;transporter activity	3.329	0.52	0.16
Q12165	ATP synthase subunit delta, mitochondrial [OS=Saccharomyces cerevisiae S288c]	851560	ATP16; YDL004W	metabolic process;transport	membrane;mitochondrion	catalytic activity;transporter activity	4.623	0.778	0.17
P07251	ATP synthase subunit alpha, mitochondrial [OS=Saccharomyces cerevisiae S288c]	852177	ATP1; YBL099W	metabolic process;transport	cytosol;membrane;mitochondrion	catalytic activity;nucleotide binding;transporter activity	120.15	20.544	0.17
P36526	54S ribosomal protein L27, mitochondrial [OS=Saccharomyces cerevisiae S288c]	852585	MRPL27; YBR282W	cell organization and biogenesis;metabolic process	mitochondrion;ribosome	structural molecule activity	1.512	0.259	0.17
P27697	Atypical kinase COQ8, mitochondrial [OS=Saccharomyces cerevisiae S288c]	852758	COQ8; YGL119W	metabolic process	membrane;mitochondrion;organelle lumen	catalytic activity;nucleotide binding	0.365	0.064	0.18
P46367	Potassium-activated aldehyde dehydrogenase, mitochondrial [OS=Saccharomyces cerevisiae S288c]	854556	ALD4; YOR374W	metabolic process	mitochondrion;organelle lumen	catalytic activity	219.22	38.811	0.18
P32316	acetyl-CoA hydrolase [OS=Saccharomyces cerevisiae S288c]	852266	ACH1; YBL015W	metabolic process	cytoplasm;cytosol;mitochondrion	catalytic activity	99	17.738	0.18
P53311	Mitochondrial pyruvate carrier 3 [OS=Saccharomyces cerevisiae S288c]	853158	FMP43; MPC3; YGR243W	transport	membrane;mitochondrion	transporter activity	1.154	0.212	0.18
P20967	2-oxoglutarate dehydrogenase, mitochondrial [OS=Saccharomyces cerevisiae S288c]	854681	KGD1; YIL125W	metabolic process	cytosol;mitochondrion;organelle lumen	catalytic activity	55.848	10.392	0.19

P53312	Succinyl-CoA ligase [ADP-forming] subunit beta, mitochondrial [OS=Saccharomyces cerevisiae S288c]	853159	LSC2; YGR244C	metabolic process	mitochondrion	catalytic activity;nucleotide binding	37.075	7.003	0.19
P06168	ketol-acid reductoisomerase, mitochondrial [OS=Saccharomyces cerevisiae S288c]	851069	ILV5; YLR355C	cell organization and biogenesis;metabolic process	mitochondrion	catalytic activity;DNA binding;metal ion binding	42.94	8.211	0.19
P07213	Mitochondrial import receptor subunit TOM70 [OS=Saccharomyces cerevisiae S288c]	855602	TOM70; YNL121C	cell organization and biogenesis;transport	membrane;mitochondrion	protein binding;transporter activity	5.236	1.031	0.20
P32891	D-lactate dehydrogenase [cytochrome] 1, mitochondrial [OS=Saccharomyces cerevisiae S288c]	851380	DLD1; YDL174C	metabolic process;transport	membrane;mitochondrion	catalytic activity;nucleotide binding	32.839	6.627	0.20
P21771	37S ribosomal protein S28, mitochondrial [OS=Saccharomyces cerevisiae S288c]	851937	MRPS28; YDR337W	cell organization and biogenesis;metabolic process	mitochondrion;ribosome	RNA binding;structural molecule activity	1.336	0.274	0.21
P38910	10 kDa heat shock protein, mitochondrial [OS=Saccharomyces cerevisiae S288c]	854185	HSP10; YOR020C	cell organization and biogenesis;metabolic process;response to stimulus;transport	cytoplasm;mitochondrion;organelle lumen	metal ion binding;nucleotide binding;protein binding	6.499	1.371	0.21
P15179	Aspartate--tRNA ligase, mitochondrial [OS=Saccharomyces cerevisiae S288c]	856000	MSD1; YPL104W	metabolic process	cytoplasm;mitochondrion;organelle lumen	catalytic activity;nucleotide binding	0.535	0.113	0.21
P47039	Probable kynurenine--oxoglutarate transaminase BNA3 [OS=Saccharomyces cerevisiae S288c]	853386	BNA3; YJL060W	metabolic process;regulation of biological process	cytoplasm;mitochondrion	catalytic activity	0.468	0.101	0.22
P09624	Dihydrolipoyl dehydrogenase, mitochondrial [OS=Saccharomyces cerevisiae S288c]	850527	LPD1; YFL018C	cellular homeostasis;metabolic process;regulation of biological process	mitochondrion;organelle lumen	catalytic activity;nucleotide binding	113.51	24.809	0.22
P34227	Mitochondrial peroxiredoxin PRX1 [OS=Saccharomyces cerevisiae S288c]	852215	PRX1; YBL064C	cellular homeostasis;metabolic process;regulation of biological process;response to stimulus	mitochondrion	antioxidant activity;catalytic activity	17.33	3.833	0.22
P33303	Succinate/fumarate mitochondrial transporter [OS=Saccharomyces cerevisiae S288c]	853558	SFC1; YJR095W	metabolic process;transport	membrane;mitochondrion	structural molecule activity;transporter activity	39.842	9	0.23
P47045	mitochondrial import inner membrane translocase subunit TIM54 [OS=Saccharomyces cerevisiae S288c]	853392	TIM54; YJL054W	cell organization and biogenesis;transport	cytosol;membrane;mitochondrion	transporter activity	1.054	0.241	0.23
Q06892	NADH kinase POS5, mitochondrial [OS=Saccharomyces cerevisiae S288c]	855913	POS5; YPL188W	metabolic process;response to stimulus	mitochondrion;organelle lumen	catalytic activity;nucleotide binding	1.565	0.369	0.24
P41921	glutathione reductase [OS=Saccharomyces cerevisiae S288c]	856014	GLR1; YPL091W	cellular homeostasis;metabolic process;regulation of biological process;response to stimulus	cytoplasm;cytosol;mitochondrion;nucleus	antioxidant activity;catalytic activity;nucleotide binding	0.655	0.155	0.24
P19414	Aconitate hydratase, mitochondrial [OS=Saccharomyces cerevisiae S288c]	851013	ACO1; YLR304C	cell organization and biogenesis;metabolic process;transport	cytoplasm;cytosol;mitochondrion;organelle lumen	catalytic activity;DNA binding;metal ion binding	109.53	26.213	0.24
P30902	ATP synthase subunit d, mitochondrial [OS=Saccharomyces cerevisiae S288c]	853853	ATP7; YKL016C	metabolic process;transport	membrane;mitochondrion	catalytic activity;transporter activity	9	2.162	0.24
P02992	elongation factor Tu, mitochondrial [OS=Saccharomyces cerevisiae S288c]	854359	TUF1; YOR187W	cell organization and biogenesis;metabolic process	mitochondrion	catalytic activity;nucleotide binding;RNA binding	33.551	8.152	0.24
Q06236	Mitochondrial inner membrane protein SHH4 [OS=Saccharomyces cerevisiae S288c]	850861	SHH4; YLR164W	metabolic process	membrane;mitochondrion	catalytic activity;metal ion binding	1.371	0.334	0.24
P38120	37S ribosomal protein S9, mitochondrial [OS=Saccharomyces cerevisiae S288c]	852443	MRPS9; YBR146W	cell organization and biogenesis;metabolic process	mitochondrion;ribosome	RNA binding;structural molecule activity	1.254	0.311	0.25
P12695	Dihydrolipoyllysine-residue acetyltransferase component of pyruvate dehydrogenase complex, mitochondrial [OS=Saccharomyces cerevisiae S288c]	855653	LAT1; YNL071W	metabolic process	mitochondrion;organelle lumen	catalytic activity	21.638	5.404	0.25
Q03028	Mitochondrial 2-oxodicarboxylate carrier 1 [OS=Saccharomyces cerevisiae S288c]	855969	ODC1; YPL134C	metabolic process;transport	membrane;mitochondrion	structural molecule activity;transporter activity	10.365	2.594	0.25
P32191	Glycerol-3-phosphate dehydrogenase, mitochondrial [OS=Saccharomyces cerevisiae S288c]	854651	GUT2; YIL155C	metabolic process	membrane;mitochondrion	catalytic activity	18.855	4.834	0.26
P37292	Serine hydroxymethyltransferase, mitochondrial [OS=Saccharomyces cerevisiae S288c]	852565	SHM1; YBR263W	metabolic process	mitochondrion	catalytic activity	14.199	3.642	0.26
P23180	Probable oxidoreductase AIM17 [OS=Saccharomyces cerevisiae S288c]	856365	AIM17; YHL021C	cell organization and biogenesis;metabolic process	mitochondrion	catalytic activity;metal ion binding	11.589	2.981	0.26
P40530	[Pyruvate dehydrogenase (acetyl-transferring)] kinase 1, mitochondrial [OS=Saccharomyces cerevisiae S288c]	854769	PKP1; YIL042C	metabolic process	mitochondrion;organelle lumen	catalytic activity;nucleotide binding;protein binding	0.931	0.245	0.26
P17505	Malate dehydrogenase, mitochondrial [OS=Saccharomyces cerevisiae S288c]	853777	MDH1; YKL085W	metabolic process	mitochondrion;organelle lumen	catalytic activity;protein binding;RNA binding	172.02	45.416	0.26

P19882	heat shock protein 60, mitochondrial [OS=Saccharomyces cerevisiae S288c]	850963	HSP60; YLR259C	cell organization and biogenesis;metabolic process;regulation of biological process;transport	cytoplasm;cytosol;membrane;mitochondrion;organelle lumen	catalytic activity;DNA binding;nucleotide binding;protein binding	93.12	25.367	0.27
P10662	37S ribosomal protein MRP1, mitochondrial [OS=Saccharomyces cerevisiae S288c]	851948	MRP1; YDR347W	cell organization and biogenesis;metabolic process;response to stimulus	mitochondrion;ribosome	antioxidant activity;catalytic activity;metal ion binding;structural molecule activity	1.512	0.413	0.27
P32335	Protein MSS51, mitochondrial [OS=Saccharomyces cerevisiae S288c]	850900	MSS51; YLR203C	metabolic process;regulation of biological process	membrane;mitochondrion	protein binding;translation regulator activity	1.712	0.468	0.27
P49367	Homoaconitase, mitochondrial [OS=Saccharomyces cerevisiae S288c]	851820	LYS4; YDR234W	metabolic process	mitochondrion	catalytic activity;metal ion binding;protein binding	1.994	0.551	0.28
Q03201	37S ribosomal protein S10, mitochondrial [OS=Saccharomyces cerevisiae S288c]	851611	RSM10; YDR041W	cell organization and biogenesis;metabolic process	mitochondrion;ribosome	structural molecule activity	0.701	0.194	0.28
Q12230	Sphingolipid long chain base-responsive protein LSP1 [OS=Saccharomyces cerevisiae S288c]	856103	LSP1; YPL004C	regulation of biological process;response to stimulus;transport	cytoplasm;membrane;mitochondrion		72.564	20.544	0.28
Q02486	ARS-binding factor 2, mitochondrial [OS=Saccharomyces cerevisiae S288c]	855094	ABF2; YMR072W	cell organization and biogenesis;metabolic process;regulation of biological process	chromosome;mitochondrion;nucleus	DNA binding	118.38	33.551	0.28
P04840	Mitochondrial outer membrane protein porin 1 [OS=Saccharomyces cerevisiae S288c]	855669	POR1; YNL055C	cell death;cell organization and biogenesis;cellular homeostasis;regulation of biological process;transport	cytoplasm;membrane;mitochondrion	transporter activity	60.585	17.33	0.29
P27929	37S ribosomal protein NAM9, mitochondrial [OS=Saccharomyces cerevisiae S288c]	855585	NAM9; YNL137C	cell organization and biogenesis;metabolic process;regulation of biological process	mitochondrion;ribosome	RNA binding;structural molecule activity	1.352	0.389	0.29
P38172	MIOREX complex component 3 [OS=Saccharomyces cerevisiae S288c]	852181	YBL095W; MRX3; YBL095W		membrane;mitochondrion		0.501	0.145	0.29
P38884	Altered inheritance of mitochondria protein 18, mitochondrial [OS=Saccharomyces cerevisiae S288c]	856605	AIM18; YHR198C		mitochondrion	catalytic activity	0.501	0.145	0.29
P36519	54S ribosomal protein L7, mitochondrial [OS=Saccharomyces cerevisiae S288c]	851823	MRPL7; YDR237W	cell organization and biogenesis;metabolic process	mitochondrion;ribosome	RNA binding;structural molecule activity	0.468	0.136	0.29
P04803	Tryptophan--tRNA ligase, mitochondrial [OS=Saccharomyces cerevisiae S288c]	851861	MSW1; YDR268W	metabolic process	cytoplasm;mitochondrion;organelle lumen	catalytic activity;nucleotide binding	0.468	0.136	0.29
Q03976	37S ribosomal protein S24, mitochondrial [OS=Saccharomyces cerevisiae S288c]	851755	RSM24; YDR175C	cell organization and biogenesis;metabolic process	mitochondrion;ribosome	structural molecule activity	1.089	0.318	0.29
P0CS90	Heat shock protein SSC1, mitochondrial [OS=Saccharomyces cerevisiae S288c]	853503	SSC1; YJR045C	cell organization and biogenesis;metabolic process;regulation of biological process;transport	membrane;mitochondrion;nucleus;organelle lumen	catalytic activity;enzyme regulator activity;nucleotide binding;protein binding	243.84	71.629	0.29
Q06678	54S ribosomal protein L35, mitochondrial [OS=Saccharomyces cerevisiae S288c]	851921	MRPL35; YDR322W	cell organization and biogenesis;metabolic process	mitochondrion;ribosome	structural molecule activity	1.894	0.557	0.29
Q12251	Uncharacterized mitochondrial carrier YPR011C [OS=Saccharomyces cerevisiae S288c]	856121	YPR011C; YPR011C	metabolic process;transport	membrane;mitochondrion	structural molecule activity;transporter activity	0.389	0.116	0.30
P38152	Tricarboxylate transport protein [OS=Saccharomyces cerevisiae S288c]	852594	CTP1; YBR291C	metabolic process;transport	membrane;mitochondrion	structural molecule activity;transporter activity	0.389	0.116	0.30
P53170	[Pyruvate dehydrogenase (acetyl-transferring)] kinase 2, mitochondrial [OS=Saccharomyces cerevisiae S288c]	852821	PKP2; YGL059W	metabolic process;regulation of biological process	mitochondrion;organelle lumen	catalytic activity;nucleotide binding;protein binding	0.931	0.28	0.30
Q02950	37S ribosomal protein MRP51, mitochondrial [OS=Saccharomyces cerevisiae S288c]	855985	MRP51; YPL118W	cell organization and biogenesis;metabolic process	mitochondrion;ribosome	structural molecule activity	1.346	0.407	0.30
P25039	Elongation factor G, mitochondrial [OS=Saccharomyces cerevisiae S288c]	850758	MEF1; YLR069C	cell organization and biogenesis;metabolic process	cytosol;mitochondrion	catalytic activity;nucleotide binding;RNA binding	0.677	0.207	0.31
P21954	Isocitrate dehydrogenase [NADP], mitochondrial [OS=Saccharomyces cerevisiae S288c]	851493	IDP1; YDL066W	metabolic process	mitochondrion	catalytic activity;metal ion binding;nucleotide binding;protein binding	67.129	20.544	0.31
P07257	Cytochrome b-c1 complex subunit 2, mitochondrial [OS=Saccharomyces cerevisiae S288c]	856321	QCR2; YPR191W	metabolic process;transport	membrane;mitochondrion	catalytic activity;metal ion binding;protein binding;transporter activity	82.768	25.49	0.31

Q04172	Sensitive to high expression protein 9, mitochondrial [OS=Saccharomyces cerevisiae S288c]	852002	SHE9; YDR393W	cell organization and biogenesis	membrane;mitochondrion		0.269	0.083	0.31
P25605	Acetolactate synthase small subunit, mitochondrial [OS=Saccharomyces cerevisiae S288c]	850348	ILV6; YCL009C	metabolic process;regulation of biological process	mitochondrion	catalytic activity;enzyme regulator activity	9	2.793	0.31
P33416	Heat shock protein 78, mitochondrial [OS=Saccharomyces cerevisiae S288c]	851845	HSP78; YDR258C	cell organization and biogenesis;metabolic process;regulation of biological process;response to stimulus	mitochondrion;organelle lumen	catalytic activity;nucleotide binding;protein binding	9.784	3.042	0.31
P00447	Superoxide dismutase [Mn], mitochondrial [OS=Saccharomyces cerevisiae S288c]	856399	SOD2; YHR008C	metabolic process;response to stimulus	mitochondrion;organelle lumen	antioxidant activity;catalytic activity;metal ion binding	6.017	1.894	0.31
P36060	NADH-cytochrome b5 reductase 2 [OS=Saccharomyces cerevisiae S288c]	853707	MCR1; YKL150W	metabolic process;response to stimulus	membrane;mitochondrion	catalytic activity;protein binding	36.927	11.743	0.32
P50945	MICOS complex subunit MIC27 [OS=Saccharomyces cerevisiae S288c]	855623	AIM37; MIC27; YNL100W	cell organization and biogenesis	membrane;mitochondrion		0.968	0.311	0.32
P53220	Mitochondrial import inner membrane translocase subunit TIM21 [OS=Saccharomyces cerevisiae S288c]	852921	TIM21; YGR033C	cell organization and biogenesis;transport	membrane;mitochondrion	protein binding	0.968	0.311	0.32
P40495	Homoisocitrate dehydrogenase, mitochondrial [OS=Saccharomyces cerevisiae S288c]	854714	LYS12; YIL094C	metabolic process	mitochondrion	catalytic activity;metal ion binding;nucleotide binding	10.007	3.217	0.32
Q08179	Mitochondrial distribution and morphology protein 38 [OS=Saccharomyces cerevisiae S288c]	854130	MDM38; YOL027C	cell organization and biogenesis;cellular homeostasis;regulation of biological process;transport	membrane;mitochondrion		7.913	2.548	0.32
Q03430	37S ribosomal protein RSM28, mitochondrial [OS=Saccharomyces cerevisiae S288c]	852105	RSM28; YDR494W	cell organization and biogenesis;metabolic process	mitochondrion;ribosome	structural molecule activity	2.008	0.65	0.32
Q00711	Succinate dehydrogenase [ubiquinone] flavoprotein subunit, mitochondrial [OS=Saccharomyces cerevisiae S288c]	853709	SDH1; YKL148C	metabolic process;transport	membrane;mitochondrion	catalytic activity;nucleotide binding;protein binding	16.535	5.381	0.33
P36151	Uncharacterized protein YKR070W [OS=Saccharomyces cerevisiae S288c]	853944	YKR070W; YKR070W	metabolic process	mitochondrion		1.336	0.438	0.33
P38523	GrpE protein homolog, mitochondrial [OS=Saccharomyces cerevisiae S288c]	854407	MGE1; YOR232W	metabolic process;regulation of biological process;transport	membrane;mitochondrion;organelle lumen	enzyme regulator activity;nucleotide binding;protein binding	0.833	0.274	0.33
P36013	NAD-dependent malic enzyme, mitochondrial [OS=Saccharomyces cerevisiae S288c]	853839	MAE1; YKL029C	metabolic process	mitochondrion;organelle lumen	catalytic activity;metal ion binding;nucleotide binding	3.732	1.239	0.33
P16387	Pyruvate dehydrogenase E1 component subunit alpha, mitochondrial [OS=Saccharomyces cerevisiae S288c]	856925	PDA1; YER178W	cell growth;metabolic process	mitochondrion;organelle lumen	catalytic activity	31.819	10.602	0.33
P06208-1	2-isopropylmalate synthase [OS=Saccharomyces cerevisiae S288c]	855619	LEU4; YNL104C	metabolic process	cytoplasm;mitochondrion	catalytic activity;protein binding	19.309	6.444	0.33
P36520	54S ribosomal protein L10, mitochondrial [OS=Saccharomyces cerevisiae S288c]	855436	MRPL10; YNL284C	cell organization and biogenesis;metabolic process	mitochondrion;ribosome	structural molecule activity	0.73	0.245	0.34
P04037	cytochrome c oxidase subunit 4, mitochondrial [OS=Saccharomyces cerevisiae S288c]	852688	COX4; YGL187C	cell organization and biogenesis;metabolic process;transport	membrane;mitochondrion	catalytic activity;metal ion binding;transporter activity	42.288	14.199	0.34
P51998	54S ribosomal protein yml6, mitochondrial [OS=Saccharomyces cerevisiae S288c]	854983	YML6; YML025C	cell organization and biogenesis;metabolic process	mitochondrion;ribosome	RNA binding;structural molecule activity	1.154	0.389	0.34
P32902	37S ribosomal protein MRP4, mitochondrial [OS=Saccharomyces cerevisiae S288c]	856384	MRP4; YHL004W	cell organization and biogenesis;metabolic process	mitochondrion;ribosome	structural molecule activity	1.371	0.468	0.34
P38891	Branched-chain-amino-acid aminotransferase, mitochondrial [OS=Saccharomyces cerevisiae S288c]	856615	BAT1; YHR208W	metabolic process;regulation of biological process;response to stimulus	mitochondrion;organelle lumen	catalytic activity	17.738	6.305	0.36
P00427	Cytochrome c oxidase subunit 6, mitochondrial [OS=Saccharomyces cerevisiae S288c]	856448	COX6; YHR051W	metabolic process;transport	membrane;mitochondrion	catalytic activity;metal ion binding;transporter activity	2.162	0.778	0.36
P81449	ATP synthase subunit e, mitochondrial [OS=Saccharomyces cerevisiae S288c]	851922	TIM11; YDR322C-A	cell organization and biogenesis;metabolic process;transport	membrane;mitochondrion	catalytic activity;structural molecule activity;transporter activity	2.162	0.778	0.36
P40098	uncharacterized mitochondrial membrane protein FMP10 [OS=Saccharomyces cerevisiae S288c]	856931	FMP10; YER182W		membrane;mitochondrion		4.456	1.637	0.37
P38077	ATP synthase subunit gamma, mitochondrial [OS=Saccharomyces cerevisiae S288c]	852327	ATP3; YBR039W	metabolic process;transport	membrane;mitochondrion	catalytic activity;transporter activity	27.48	10.103	0.37

P25626	54S ribosomal protein IMG1, mitochondrial [OS=Saccharomyces cerevisiae S288c]	850413	IMG1; YCR046C	cell organization and biogenesis;metabolic process	mitochondrion;ribosome	structural molecule activity	1.894	0.701	0.37
P08067	cytochrome b-c1 complex subunit Rieske, mitochondrial [OS=Saccharomyces cerevisiae S288c]	856689	RIP1; YEL024W	metabolic process;transport	membrane;mitochondrion	catalytic activity;metal ion binding;transporter activity	67.129	25.102	0.37
P23644	Mitochondrial import receptor subunit TOM40 [OS=Saccharomyces cerevisiae S288c]	855243	TOM40; YMR203W	transport	cytosol;membrane;mitochondrion	protein binding;transporter activity	1.783	0.668	0.37
Q02981	ABC1 family protein YPL109C, mitochondrial [OS=Saccharomyces cerevisiae S288c]	855995	YPL109C; YPL109C		mitochondrion		0.496	0.189	0.38
P16451	Pyruvate dehydrogenase complex protein X component, mitochondrial [OS=Saccharomyces cerevisiae S288c]	853107	PDX1; YGR193C	metabolic process	mitochondrion;organelle lumen	catalytic activity;structural molecule activity	4.055	1.555	0.38
P40502	Altered inheritance of mitochondria protein 19, mitochondrial [OS=Saccharomyces cerevisiae S288c]	854722	AIM19; YIL087C		membrane;mitochondrion		1.512	0.585	0.39
P53881	54S ribosomal protein L22, mitochondrial [OS=Saccharomyces cerevisiae S288c]	855544	MRPL22; YNL177C	cell organization and biogenesis;metabolic process	mitochondrion;ribosome	RNA binding;structural molecule activity	1.404	0.551	0.39
Q12480	Probable electron transfer flavoprotein subunit alpha, mitochondrial [OS=Saccharomyces cerevisiae S288c]	856112	AIM45; YPR004C	metabolic process;transport	mitochondrion;organelle lumen	catalytic activity;nucleotide binding	4.623	1.818	0.39
P25372	Thioredoxin-3, mitochondrial [OS=Saccharomyces cerevisiae S288c]	850444	TRX3; YCR083W	cellular homeostasis;metabolic process;regulation of biological process;response to stimulus;transport	cytoplasm;mitochondrion	catalytic activity	1.31	0.52	0.40
Q01574	acetyl-coenzyme A synthetase 1 [OS=Saccharomyces cerevisiae S288c]	851245	ACS1; YAL054C	cell organization and biogenesis;metabolic process	cytoplasm;cytosol;endoplasmic reticulum;mitochondrion;nucleus	catalytic activity;nucleotide binding	4.456	1.801	0.40
P16622	Ferrochelatase, mitochondrial [OS=Saccharomyces cerevisiae S288c]	854347	HEM15; YOR176W	metabolic process	membrane;mitochondrion	catalytic activity	7.111	2.899	0.41
P40035	Mitochondrial phosphate carrier protein 2 [OS=Saccharomyces cerevisiae S288c]	856779	PIC2; YER053C	cellular homeostasis;metabolic process;transport	membrane;mitochondrion	structural molecule activity;transporter activity	1.069	0.438	0.41
P00431	cytochrome c peroxidase, mitochondrial [OS=Saccharomyces cerevisiae S288c]	853940	CCP1; YKR066C	metabolic process;response to stimulus	mitochondrion;organelle lumen	antioxidant activity;catalytic activity;metal ion binding;protein binding	1.031	0.425	0.41
P52893	probable alanine aminotransferase, mitochondrial [OS=Saccharomyces cerevisiae S288c]	850778	ALT1; YLR089C	metabolic process	mitochondrion;organelle lumen	catalytic activity	2.282	0.951	0.42
P07246	Alcohol dehydrogenase 3, mitochondrial [OS=Saccharomyces cerevisiae S288c]	855107	ADH3; YMR083W	metabolic process	mitochondrion;organelle lumen	catalytic activity;metal ion binding	11.452	4.78	0.42
Q03246	37S ribosomal protein S17, mitochondrial [OS=Saccharomyces cerevisiae S288c]	855226	MRPS17; YMR188C	cell differentiation;cell organization and biogenesis;metabolic process	mitochondrion;ribosome	RNA binding;structural molecule activity	0.931	0.389	0.42
Q12349	ATP synthase subunit H, mitochondrial [OS=Saccharomyces cerevisiae S288c]	851002	ATP14; YLR295C	cell organization and biogenesis;metabolic process;transport	membrane;mitochondrion	catalytic activity;transporter activity	0.931	0.389	0.42
Q12428	Probable 2-methylcitrate dehydratase [OS=Saccharomyces cerevisiae S288c]	856108	PDH1; YPR002W	metabolic process	cytoplasm;membrane;mitochondrion	catalytic activity	18.11	7.66	0.42
P00890	citrate synthase, mitochondrial [OS=Saccharomyces cerevisiae S288c]	855732	CIT1; YNR001C	metabolic process;transport	cytosol;mitochondrion;organelle lumen	catalytic activity	36.276	15.379	0.42
Q07914	mitochondrial import inner membrane translocase subunit TIM14 [OS=Saccharomyces cerevisiae S288c]	850694	PAM18; YLR008C	regulation of biological process;transport	membrane;mitochondrion	enzyme regulator activity;protein binding;transporter activity	0.778	0.334	0.43
P11914	Mitochondrial-processing peptidase subunit alpha [OS=Saccharomyces cerevisiae S288c]	856419	MAS2; YHR024C	metabolic process;transport	membrane;mitochondrion;organelle lumen	catalytic activity;metal ion binding;protein binding	0.738	0.318	0.43
P31334	54S ribosomal protein L9, mitochondrial [OS=Saccharomyces cerevisiae S288c]	853135	MRPL9; YGR220C	cell organization and biogenesis;metabolic process	mitochondrion;ribosome	structural molecule activity	0.719	0.311	0.43
Q12289	mitochondrial carnitine carrier [OS=Saccharomyces cerevisiae S288c]	854267	CRC1; YOR100C	metabolic process;transport	membrane;mitochondrion	structural molecule activity;transporter activity	1.683	0.73	0.43
P49095	Glycine dehydrogenase (Decarboxylating), mitochondrial [OS=Saccharomyces cerevisiae S288c]	855227	GCV2; YMR189W	metabolic process	cytosol;mitochondrion	catalytic activity	2.728	1.184	0.43
P40215	External NADH-ubiquinone oxidoreductase 1, mitochondrial [OS=Saccharomyces cerevisiae S288c]	855176	NDE1; YMR145C	metabolic process	mitochondrion	catalytic activity;nucleotide binding	7.297	3.184	0.44
Q12283	Malonyl CoA-acyl carrier protein transacylase, mitochondrial [OS=Saccharomyces cerevisiae S288c]	854396	MCT1; YOR221C	metabolic process	mitochondrion	catalytic activity	0.668	0.292	0.44

P19516	Cytochrome c oxidase assembly protein COX11, mitochondrial [OS=Saccharomyces cerevisiae S288c]	855971	COX11; YPL132W	cell organization and biogenesis;metabolic process	membrane;mitochondrion;ribosome	metal ion binding	0.624	0.274	0.44
Q01163	37S ribosomal protein S23, mitochondrial [OS=Saccharomyces cerevisiae S288c]	852748	RSM23; YGL129C	cell organization and biogenesis;metabolic process	mitochondrion;ribosome	catalytic activity;nucleotide binding;RNA binding;structural molecule activity	1.707	0.751	0.44
P53969	Sorting assembly machinery 50 kDa subunit [OS=Saccharomyces cerevisiae S288c]	855705	SAM50; YNL026W	cell organization and biogenesis;transport	membrane;mitochondrion	protein binding;transporter activity	0.61	0.269	0.44
P43617	Uncharacterized mitochondrial carrier YFR045W [OS=Saccharomyces cerevisiae S288c]	850606	YFR045W; YFR045W	metabolic process;transport	membrane;mitochondrion	structural molecule activity;transporter activity	0.585	0.259	0.44
P05626	ATP synthase subunit 4, mitochondrial [OS=Saccharomyces cerevisiae S288c]	856027	ATP4; YPL078C	cell organization and biogenesis;metabolic process;transport	membrane;mitochondrion	catalytic activity;transporter activity	5.813	2.594	0.45
P23641	mitochondrial phosphate carrier protein [OS=Saccharomyces cerevisiae S288c]	853540	MIR1; YJR077C	metabolic process;transport	membrane;mitochondrion	protein binding;structural molecule activity;transporter activity	30.623	13.678	0.45
P17695	Glutaredoxin-2, mitochondrial [OS=Saccharomyces cerevisiae S288c]	852124	GRX2; YDR513W	cellular homeostasis;metabolic process;regulation of biological process;response to stimulus;transport	cytoplasm;cytosol;mitochondrion;nucleus	antioxidant activity;catalytic activity	0.52	0.233	0.45
P43594	MICOS complex subunit MIC19 [OS=Saccharomyces cerevisiae S288c]	850563	AIM13; MIC19; YFR011C	cell organization and biogenesis	cytoplasm;membrane;mitochondrion		0.52	0.233	0.45
P09440	C-1-tetrahydrofolate synthase, mitochondrial [OS=Saccharomyces cerevisiae S288c]	852378	MIS1; YBR084W	metabolic process	cytosol;mitochondrion	catalytic activity;nucleotide binding	1.988	0.9	0.45
P40165	NAD(P)H-hydrate epimerase [OS=Saccharomyces cerevisiae S288c]	855521	YNL200C; YNL200C	metabolic process	cytoplasm;mitochondrion	catalytic activity;metal ion binding;nucleotide binding	0.468	0.212	0.45
Q03020	iron sulfur cluster assembly protein 1, mitochondrial [OS=Saccharomyces cerevisiae S288c]	855968	ISU1; YPL135W	cell organization and biogenesis;cellular homeostasis;metabolic process	cytoplasm;mitochondrion;organelle lumen	catalytic activity;metal ion binding;protein binding	0.468	0.212	0.45
P28834	Isocitrate dehydrogenase [NAD] subunit 1, mitochondrial [OS=Saccharomyces cerevisiae S288c]	855691	IDH1; YNL037C	metabolic process;transport	cytosol;mitochondrion;organelle lumen	catalytic activity;metal ion binding;nucleotide binding;protein binding;RNA binding	19.691	9	0.46
P38169	Kynurenine 3-monooxygenase [OS=Saccharomyces cerevisiae S288c]	852179	BNA4; YBL098W	metabolic process	membrane;mitochondrion	catalytic activity;nucleotide binding	0.374	0.172	0.46
P25348	54S ribosomal protein L32, mitochondrial [OS=Saccharomyces cerevisiae S288c]	850359	MRPL32; YCR003W	cell organization and biogenesis;metabolic process	membrane;mitochondrion;ribosome	RNA binding;structural molecule activity	0.389	0.179	0.46
P53212	Probable transcriptional regulatory protein HAH1 [OS=Saccharomyces cerevisiae S288c]	852904	YGR021W; YGR021W		mitochondrion		0.359	0.166	0.46
P07342	Acetolactate synthase catalytic subunit, mitochondrial [OS=Saccharomyces cerevisiae S288c]	855135	ILV2; YMR108W	metabolic process	mitochondrion	catalytic activity;metal ion binding;nucleotide binding	8.427	3.924	0.47
Q12298	uncharacterized ABC transporter ATP-binding protein YDR061W [OS=Saccharomyces cerevisiae S288c]	851633	YDR061W; YDR061W		mitochondrion	catalytic activity;nucleotide binding	0.266	0.125	0.47
P40416	Iron-sulfur clusters transporter ATM1, mitochondrial [OS=Saccharomyces cerevisiae S288c]	855347	ATM1; YMR301C	cellular homeostasis;metabolic process;transport	membrane;mitochondrion	catalytic activity;nucleotide binding;transporter activity	0.283	0.133	0.47
P49954	probable hydrolase NIT3 [OS=Saccharomyces cerevisiae S288c]	851065	NIT3; YLR351C	metabolic process	cytoplasm;mitochondrion	catalytic activity	0.274	0.129	0.47
P14908	Mitochondrial transcription factor 1 [OS=Saccharomyces cerevisiae S288c]	855268	MTF1; YMR228W	metabolic process;regulation of biological process	mitochondrion;organelle lumen	catalytic activity;DNA binding;RNA binding	0.259	0.122	0.47
P10834	protein PET54 [OS=Saccharomyces cerevisiae S288c]	853137	PET54; YGR222W	metabolic process;regulation of biological process	membrane;mitochondrion;organelle lumen	nucleotide binding;RNA binding;translation regulator activity	0.233	0.11	0.47
P36528	54S ribosomal protein L17, mitochondrial [OS=Saccharomyces cerevisiae S288c]	855469	MRPL17; YNL252C	cell organization and biogenesis;metabolic process	mitochondrion;ribosome	structural molecule activity	0.233	0.11	0.47
P11325	Leucine--tRNA ligase, mitochondrial [OS=Saccharomyces cerevisiae S288c]	851098	NAM2; YLR382C	cell organization and biogenesis;metabolic process;regulation of biological process	cytosol;mitochondrion;organelle lumen	catalytic activity;nucleotide binding;RNA binding	0.254	0.12	0.47

Q12166	2-isopropylmalate synthase 2, mitochondrial [OS=Saccharomyces cerevisiae S288c]	854275	LEU9; YOR108W	metabolic process	mitochondrion	catalytic activity;protein binding	4.367	2.065	0.47
P53163	54S ribosomal protein L12, mitochondrial [OS=Saccharomyces cerevisiae S288c]	852811	MNP1; YGL068W	cell organization and biogenesis;metabolic process	mitochondrion;ribosome	RNA binding;structural molecule activity	1.848	0.874	0.47
Q06143	mitochondrial dicarboxylate transporter [OS=Saccharomyces cerevisiae S288c]	851063	DIC1; YLR348C	metabolic process;transport	membrane;mitochondrion	structural molecule activity;transporter activity	1.154	0.551	0.48
Q12031	Mitochondrial 2-methylisocitrate lyase [OS=Saccharomyces cerevisiae S288c]	856114	ICL2; YPR006C	metabolic process	mitochondrion;organelle lumen	catalytic activity	4.135	1.976	0.48
Q08970	Mitochondrial metal transporter 2 [OS=Saccharomyces cerevisiae S288c]	855877	MMT2; YPL224C	cellular homeostasis;regulation of biological process;response to stimulus;transport	membrane;mitochondrion	transporter activity	0.186	0.089	0.48
P32839	Mitochondrial chaperone BCS1 [OS=Saccharomyces cerevisiae S288c]	851981	BCS1; YDR375C	cell organization and biogenesis;metabolic process;response to stimulus;transport	cytosol;membrane;mitochondrion	catalytic activity;nucleotide binding;transporter activity	0.194	0.093	0.48
Q06510	Lysophosphatidylcholine acyltransferase [OS=Saccharomyces cerevisiae S288c]	856262	TAZ1; YPR140W	cell organization and biogenesis;metabolic process;transport	membrane;mitochondrion	catalytic activity	0.194	0.093	0.48
P07236	Threonine--tRNA ligase, mitochondrial [OS=Saccharomyces cerevisiae S288c]	853640	MST1; YKL194C	metabolic process	cytoplasm;mitochondrion;organelle lumen	catalytic activity;nucleotide binding;RNA binding	0.15	0.072	0.48
P42940	Probable electron transfer flavoprotein subunit beta [OS=Saccharomyces cerevisiae S288c]	853121	CIR1; YGR207C	metabolic process;transport	mitochondrion;organelle lumen		5.494	2.652	0.48
P00401	Cytochrome c oxidase subunit 1 [OS=Saccharomyces cerevisiae S288c]	854598	COX1; Q0045	metabolic process;transport	membrane;mitochondrion	catalytic activity;metal ion binding;transporter activity	1.61	0.778	0.48
P07256	Cytochrome b-c1 complex subunit 1, mitochondrial [OS=Saccharomyces cerevisiae S288c]	852235	COR1; YBL045C	metabolic process;transport	membrane;mitochondrion	catalytic activity;metal ion binding;protein binding;transporter activity	24.551	11.915	0.49
Q07349	MIOREX complex component 9 [OS=Saccharomyces cerevisiae S288c]	851535	YDL027C; MRX9; YDL027C		endoplasmic reticulum;membrane;mitochondrion		1.913	0.931	0.49
P21801	Succinate dehydrogenase [ubiquinone] iron-sulfur subunit, mitochondrial [OS=Saccharomyces cerevisiae S288c]	850685	SDH2; YLL041C	metabolic process;transport	membrane;mitochondrion	catalytic activity;metal ion binding	24.809	12.111	0.49
P32445	Single-stranded DNA-binding protein RIM1, mitochondrial [OS=Saccharomyces cerevisiae S288c]	850395	RIM1; YCR028C-A	cell organization and biogenesis;metabolic process;regulation of biological process	mitochondrion	DNA binding	3.642	1.783	0.49
P08417	fumarate hydratase, mitochondrial [OS=Saccharomyces cerevisiae S288c]	855866	FUM1; YPL262W	cell organization and biogenesis;metabolic process	cytoplasm;cytosol;mitochondrion;organelle lumen	catalytic activity	12.738	6.279	0.49
P48526	Isoleucine--tRNA ligase, mitochondrial [OS=Saccharomyces cerevisiae S288c]	856067	ISM1; YPL040C	cell organization and biogenesis;metabolic process;regulation of biological process	cytoplasm;cytosol;mitochondrion;organelle lumen	catalytic activity;nucleotide binding	0.081	0.04	0.49
P53326	Uncharacterized protein YGR266W [OS=Saccharomyces cerevisiae S288c]	853182	YGR266W; YGR266W		membrane;mitochondrion		0.602	0.299	0.50
P35191	DnaJ homolog 1, mitochondrial [OS=Saccharomyces cerevisiae S288c]	850530	MDJ1; YFL016C	cell organization and biogenesis;metabolic process;regulation of biological process;response to stimulus	mitochondrion;organelle lumen	enzyme regulator activity;metal ion binding;nucleotide binding;protein binding	3.329	1.656	0.50
P00175	Cytochrome b2, mitochondrial [OS=Saccharomyces cerevisiae S288c]	854950	CYB2; YML054C	metabolic process;transport	cytosol;membrane;mitochondrion;nucleus	catalytic activity;metal ion binding;nucleotide binding	0.995	0.496	0.50
P15424	ATP-dependent RNA helicase MSS116, mitochondrial [OS=Saccharomyces cerevisiae S288c]	851775	MSS116; YDR194C	metabolic process;regulation of biological process	mitochondrion;organelle lumen	catalytic activity;nucleotide binding;RNA binding	1.512	0.756	0.50
P40047	Aldehyde dehydrogenase 5, mitochondrial [OS=Saccharomyces cerevisiae S288c]	856804	ALD5; YER073W	metabolic process	mitochondrion;organelle lumen	catalytic activity	3.146	1.581	0.50
P54783	D-arabinono-1,4-lactone oxidase [OS=Saccharomyces cerevisiae S288c]	854888	ALO1; YML086C	metabolic process;response to stimulus	membrane;mitochondrion	catalytic activity;nucleotide binding	5.31	2.687	0.51
P08466	mitochondrial nuclease [OS=Saccharomyces cerevisiae S288c]	853222	NUC1; YJL208C	cell death;metabolic process	membrane;mitochondrion;nucleus	catalytic activity;metal ion binding	1.637	0.833	0.51
Q04458	Fatty aldehyde dehydrogenase HFD1 [OS=Saccharomyces cerevisiae S288c]	855137	HFD1; YMR110C	metabolic process	endoplasmic reticulum;endosome;membrane;mitochondrion	catalytic activity	4.505	2.3	0.51
P32473	Pyruvate dehydrogenase E1 component subunit beta, mitochondrial [OS=Saccharomyces	852522	PDB1; YBR221C	metabolic process	mitochondrion;organelle lumen	catalytic activity	13.384	6.848	0.51

	cerevisiae S288c]								
P04710	ADP,ATP carrier protein 1 [OS=Saccharomyces cerevisiae S288c]	85507 8	AAC1; YMR05 6C	metabolic process;transport	cytosol;membrane;mitochondrion	structural molecule activity;transporter activity	13.3 84	6.848	0.51
Q06089	Uncharacterized mitochondrial outer membrane protein YPR098C [OS=Saccharomyces cerevisiae S288c]	85621 3	YPR098 C; YPR098 C		membrane;mitochondrion		9	4.623	0.51
Q08822	Probable electron transfer flavoprotein-ubiquinone oxidoreductase, mitochondrial [OS=Saccharomyces cerevisiae S288c]	85453 8	CIR2; YOR35 6W	metabolic process;transport	membrane;mitochondrion	catalytic activity;metal ion binding;nucleotide binding	3.64 2	1.873	0.51
P39522	Dihydroxy-acid dehydratase, mitochondrial [OS=Saccharomyces cerevisiae S288c]	85347 3	ILV3; YJR016 C	metabolic process	mitochondrion	catalytic activity;metal ion binding	27.4 8	14.19 9	0.52
P19262	Dihydrolipoyllysine-residue succinyltransferase component of 2-oxoglutarate dehydrogenase complex, mitochondrial [OS=Saccharomyces cerevisiae S288c]	85172 6	KGD2; YDR14 8C	cell organization and biogenesis;metabolic process	mitochondrion	catalytic activity;protein binding	15.4 96	8.047	0.52
P43635	Citrate synthase 3, mitochondrial [OS=Saccharomyces cerevisiae S288c]	85610 7	CIT3; YPR001 W	metabolic process	mitochondrion	catalytic activity	4.99 5	2.594	0.52
P00445	Superoxide dismutase [Cu-Zn] [OS=Saccharomyces cerevisiae S288c]	85356 8	SOD1; YJR104 C	cell organization and biogenesis;cellular homeostasis;metabolic process;regulation of biological process;response to stimulus	cytoplasm;cytosol;mitochondrion;nucl eus	antioxidant activity;catalytic activity;metal ion binding;protein binding	2.51 1	1.31	0.52
P25719	Peptidyl-prolyl cis-trans isomerase C, mitochondrial [OS=Saccharomyces cerevisiae S288c]	85489 7	CPR3; YML07 8W	cell death;metabolic process	mitochondrion;organelle lumen	catalytic activity	3.64 2	1.929	0.53
P28241	Isocitrate dehydrogenase [NAD] subunit 2, mitochondrial [OS=Saccharomyces cerevisiae S288c]	85430 3	IDH2; YOR13 6W	metabolic process	mitochondrion;organelle lumen	catalytic activity;metal ion binding;nucleotide binding;protein binding;RNA binding	9	4.78	0.53
P37293	N-terminal acetyltransferase 2 [OS=Saccharomyces cerevisiae S288c]	85305 0	NAT2; YGR14 7C	metabolic process	cytoplasm;mitochondrion	catalytic activity	0.73	0.389	0.53
P32332	Mitochondrial oxaloacetate transport protein [OS=Saccharomyces cerevisiae S288c]	85373 9	OAC1; YKL120 W	metabolic process;transport	membrane;mitochondrion	structural molecule activity;transporter activity	2.16 2	1.154	0.53
P43616	Cys-Gly metallodipeptidase dug1 [OS=Saccharomyces cerevisiae S288c]	85060 5	DUG1; YFR044 C	metabolic process	cytoplasm;mitochondrion;ribosome	catalytic activity;metal ion binding	1.32 6	0.711	0.54
Q07500	External NADH-ubiquinone oxidoreductase 2, mitochondrial [OS=Saccharomyces cerevisiae S288c]	85147 4	NDE2; YDL085 W	metabolic process	mitochondrion	catalytic activity;nucleotide binding	9.79 8	5.31	0.54
P53266	Cytochrome oxidase assembly protein SHY1 [OS=Saccharomyces cerevisiae S288c]	85300 9	SHY1; YGR11 2W	cell organization and biogenesis	membrane;mitochondrion	protein binding	0.97 8	0.532	0.54
Q96VH5	MICOS complex subunit Mic10 [OS=Saccharomyces cerevisiae S288c]	85030 0	MOS1; MIC10; YCL057 C-A	cell organization and biogenesis	membrane;mitochondrion		1.68 3	0.931	0.55
P19955	37S ribosomal protein YMR-31, mitochondrial [OS=Saccharomyces cerevisiae S288c]	85061 0	YMR31 ; YFR049 W	cell organization and biogenesis;metabolic process	mitochondrion;ribosome	catalytic activity;protein binding;structural molecule activity	9	4.995	0.56
P38825	Protein TOM71 [OS=Saccharomyces cerevisiae S288c]	85651 7	TOM71 ; YHR11 7W	transport	membrane;mitochondrion	protein binding;transporter activity	0.91 4	0.512	0.56
P36525	54S ribosomal protein L24, mitochondrial [OS=Saccharomyces cerevisiae S288c]	85523 1	MRPL2 4; YMR19 3W	cell organization and biogenesis;metabolic process	mitochondrion;ribosome	RNA binding;structural molecule activity	1.51 2	0.848	0.56
P40364	Mitochondrial peculiar membrane protein 1 [OS=Saccharomyces cerevisiae S288c]	85337 9	MPM1; YJL066 C		membrane;mitochondrion		1.51 2	0.848	0.56
P36147	Presequence translocated-associated motor subunit pam17, mitochondrial [OS=Saccharomyces cerevisiae S288c]	85393 9	PAM17 ; YKR065 C	transport	membrane;mitochondrion		1.37 1	0.778	0.57
P39515	mitochondrial import inner membrane translocase subunit Tim17 [OS=Saccharomyces cerevisiae S288c]	85329 8	TIM17; YJL143 W	cell organization and biogenesis;transport	membrane;mitochondrion	protein binding;transporter activity	1.37 1	0.778	0.57
Q12233	ATP synthase subunit g, mitochondrial [OS=Saccharomyces cerevisiae S288c]	85613 1	ATP20; YPR020 W	cell organization and biogenesis;metabolic process;transport	membrane;mitochondrion	catalytic activity;transporter activity	1.37 1	0.778	0.57
P25349	Flavoprotein-like protein YCP4 [OS=Saccharomyces cerevisiae S288c]	85036 0	YCP4; YCR004 C	metabolic process;regulation of biological process	cytoplasm;membrane;mitochondrion	catalytic activity;nucleotide binding;protein binding	13.6 78	7.799	0.57
P40341	Mitochondrial respiratory chain complexes assembly protein YTA12 [OS=Saccharomyces cerevisiae S288c]	85511 4	YTA12; YMR08 9C	cell organization and biogenesis;metabolic process;response to stimulus;transport	cytoplasm;membrane;mitochondrion	catalytic activity;metal ion binding;nucleotide binding;protein	1.91 3	1.096	0.57

						binding			
P22136	ATPase expression protein 2, mitochondrial [OS=Saccharomyces cerevisiae S288c]	855325	AEP2; YMR282C	metabolic process;regulation of biological process	mitochondrion	RNA binding	0.506	0.292	0.58
Q08818	Meiotic sister-chromatid recombination protein 6, mitochondrial [OS=Saccharomyces cerevisiae S288c]	854536	MSC6; YOR354C	metabolic process	mitochondrion;organelle lumen	RNA binding	0.506	0.292	0.58
P00830	ATP synthase subunit beta, mitochondrial [OS=Saccharomyces cerevisiae S288c]	853585	ATP2; YJR121W	metabolic process;transport	cytosol;membrane;mitochondrion	catalytic activity;nucleotide binding;protein binding;transporter activity	91.612	53.117	0.58
Q00402	Nuclear migration protein NUM1 [OS=Saccharomyces cerevisiae S288c]	851727	NUM1; YDR150W	cell organization and biogenesis;cellular component movement;metabolic process;regulation of biological process;transport	endoplasmic reticulum;mitochondrion	catalytic activity;motor activity;protein binding	0.134	0.078	0.58
P40185	Protein mmf1, mitochondrial [OS=Saccharomyces cerevisiae S288c]	854760	MMF1; YIL051C	cell organization and biogenesis;metabolic process	mitochondrion;organelle lumen		9	5.31	0.59
P32898	Mitochondrial presequence protease [OS=Saccharomyces cerevisiae S288c]	852041	CYM1; YDR430C	metabolic process	mitochondrion	catalytic activity;metal ion binding	0.951	0.562	0.59
P36112	MICOS complex subunit Mic60 [OS=Saccharomyces cerevisiae S288c]	853886	FCJ1; MIC60; YKR016W	cell organization and biogenesis;transport	membrane;mitochondrion		19.962	11.798	0.59
P33311	ATP-dependent permease MDL2, mitochondrial [OS=Saccharomyces cerevisiae S288c]	855858	MDL2; YPL270W	response to stimulus;transport	membrane;mitochondrion	catalytic activity;nucleotide binding;transporter activity	0.901	0.535	0.59
P10507	mitochondrial-processing peptidase subunit beta [OS=Saccharomyces cerevisiae S288c]	850860	MAS1; YLR163C	metabolic process;transport	mitochondrion;organelle lumen	catalytic activity;metal ion binding;protein binding	1.61	0.957	0.59
P53721	Respiratory supercomplex factor 2, mitochondrial [OS=Saccharomyces cerevisiae S288c]	855752	RCF2; YNR018W	cell organization and biogenesis	membrane;mitochondrion		7.111	4.337	0.61
P32317	Protein AFG1 [OS=Saccharomyces cerevisiae S288c]	856658	AFG1; YEL052W	cell organization and biogenesis;metabolic process;response to stimulus;transport	membrane;mitochondrion	catalytic activity;nucleotide binding	1.154	0.711	0.62
P41735	5-demethoxyubiquinone hydroxylase, mitochondrial [OS=Saccharomyces cerevisiae S288c]	854292	CAT5; YOR125C	metabolic process	membrane;mitochondrion	catalytic activity;metal ion binding	0.54	0.334	0.62
P53292	37S ribosomal protein S35, mitochondrial [OS=Saccharomyces cerevisiae S288c]	853075	MRPS35; YGR165W	cell organization and biogenesis;metabolic process	mitochondrion;ribosome	structural molecule activity	1.754	1.089	0.62
P07275	Delta-1-pyrroline-5-carboxylate dehydrogenase, mitochondrial [OS=Saccharomyces cerevisiae S288c]	856432	PUT2; YHR037W	metabolic process	membrane;mitochondrion;organelle lumen	catalytic activity	9.723	6.055	0.62
P40053	Altered inheritance of mitochondria protein 9, mitochondrial [OS=Saccharomyces cerevisiae S288c]	856813	AIM9; YER080W		mitochondrion		3.348	2.086	0.62
P53206	putative cysteine synthase [OS=Saccharomyces cerevisiae S288c]	852895	YGR012W; YGR012W	metabolic process		catalytic activity	1.555	0.978	0.63
Q06485	Autophagy-related protein 33 [OS=Saccharomyces cerevisiae S288c]	851070	ATG33; YLR356W	cell communication;metabolic process;response to stimulus	membrane;mitochondrion		2.162	1.371	0.63
P29704	squalene synthase [OS=Saccharomyces cerevisiae S288c]	856597	ERG9; YHR190W	metabolic process	endoplasmic reticulum;membrane;mitochondrion	catalytic activity	0.35	0.222	0.63
P41911	Glycerol-3-phosphate dehydrogenase [NAD(+)] 2, mitochondrial [OS=Saccharomyces cerevisiae S288c]	854095	GPD2; YOL059W	metabolic process	cytoplasm;cytosol;mitochondrion	catalytic activity;nucleotide binding;protein binding	0.292	0.186	0.64
P53140	Protein RMD9, mitochondrial [OS=Saccharomyces cerevisiae S288c]	852771	RMD9; YGL107C	cell differentiation;metabolic process;regulation of biological process	membrane;mitochondrion	RNA binding	1.047	0.668	0.64
P21560	Protein CBP3, mitochondrial [OS=Saccharomyces cerevisiae S288c]	855886	CBP3; YPL215W	cell organization and biogenesis;regulation of biological process	membrane;mitochondrion;ribosome		0.304	0.194	0.64
P39006	Phosphatidylserine decarboxylase proenzyme 1, mitochondrial [OS=Saccharomyces cerevisiae S288c]	855552	PSD1; YNL169C	metabolic process;regulation of biological process	membrane;mitochondrion	catalytic activity	0.269	0.172	0.64
P32843	Mitochondrial escape protein 2 [OS=Saccharomyces cerevisiae S288c]	855348	YME2; YMR302C	cell organization and biogenesis;metabolic process	membrane;mitochondrion	nucleotide binding;RNA binding	3.281	2.099	0.64
P47127	Altered inheritance of mitochondria protein 24, mitochondrial [OS=Saccharomyces cerevisiae S288c]	853543	AIM24; YJR080C	cell organization and biogenesis	mitochondrion		3.175	2.039	0.64
P48527	Tyrosine--tRNA ligase, mitochondrial [OS=Saccharomyces cerevisiae S288c]	856007	MSY1; YPL097W	metabolic process	cytoplasm;cytosol;mitochondrion;organelle lumen	catalytic activity;nucleotide binding;RNA binding	0.233	0.15	0.64

P42900	Sigma-like sequence protein 1, mitochondrial [OS=Saccharomyces cerevisiae S288c]	850830	SLS1; YLR139C	cell organization and biogenesis;metabolic process	membrane;mitochondrion	protein binding	0.194	0.125	0.64
P36775	Lon protease homolog, mitochondrial [OS=Saccharomyces cerevisiae S288c]	852259	PIM1; YBL022C	cell organization and biogenesis;metabolic process;regulation of biological process;response to stimulus	cytoplasm;mitochondrion;organelle lumen	catalytic activity;DNA binding;nucleotide binding;RNA binding	1.59	1.026	0.65
P47052	succinate dehydrogenase [ubiquinone] flavoprotein subunit 2, mitochondrial [OS=Saccharomyces cerevisiae S288c]	853405	YJL045W; YJL045W	metabolic process;transport	membrane;mitochondrion	catalytic activity;nucleotide binding	3.453	2.262	0.66
Q05892	MIOREX complex component 2 [OS=Saccharomyces cerevisiae S288c]	850997	YLR290C; COQ11; YLR290C	metabolic process	mitochondrion	catalytic activity	2.652	1.738	0.66
Q06493	LETM1 domain-containing protein YLH47, mitochondrial [OS=Saccharomyces cerevisiae S288c]	856243	YLH47; YPR125W	cell organization and biogenesis;transport	membrane;mitochondrion		0.743	0.487	0.66
P28817	3-hydroxyisobutyryl-CoA hydrolase, mitochondrial [OS=Saccharomyces cerevisiae S288c]	851606	EHD3; YDR036C	metabolic process	mitochondrion	catalytic activity	0.743	0.487	0.66
P32795	Mitochondrial inner membrane i-AAA protease supercomplex subunit YME1 [OS=Saccharomyces cerevisiae S288c]	856135	YME1; YPR024W	metabolic process;response to stimulus;transport	membrane;mitochondrion	catalytic activity;metal ion binding;nucleotide binding;protein binding	1.085	0.714	0.66
P40012	protoporphyrinogen oxidase [OS=Saccharomyces cerevisiae S288c]	856733	HEM14; YER014W	metabolic process	membrane;mitochondrion	catalytic activity	0.947	0.624	0.66
P25087	Sterol 24-C-methyltransferase [OS=Saccharomyces cerevisiae S288c]	855003	ERG6; YML008C	metabolic process	endoplasmic reticulum;membrane;mitochondrion	catalytic activity	2.775	1.894	0.68
P22354	54S ribosomal protein L20, mitochondrial [OS=Saccharomyces cerevisiae S288c]	853960	MRPL20; YKR085C	cell organization and biogenesis;metabolic process	mitochondrion;ribosome	structural molecule activity	0.931	0.638	0.69
Q12029	Probable mitochondrial transport protein fsf1 [OS=Saccharomyces cerevisiae S288c]	854445	FSF1; YOR271C	transport	membrane;mitochondrion	transporter activity	2.36	1.637	0.69
Q99297	Mitochondrial 2-oxodicarboxylate carrier 2 [OS=Saccharomyces cerevisiae S288c]	854397	ODC2; YOR222W	metabolic process;transport	membrane;mitochondrion	structural molecule activity;transporter activity	0.778	0.54	0.69
P00044	Cytochrome c iso-1 [OS=Saccharomyces cerevisiae S288c]	853507	CYC1; YJR048W	metabolic process;transport	mitochondrion	metal ion binding;protein binding	4.623	3.217	0.70
P53889	uncharacterized mitochondrial hydrolase FMP41 [OS=Saccharomyces cerevisiae S288c]	855553	FMP41; YNL168C	metabolic process	mitochondrion	catalytic activity;metal ion binding	0.719	0.501	0.70
P32387	54S ribosomal protein L41, mitochondrial [OS=Saccharomyces cerevisiae S288c]	852014	MRP20; YDR405W	cell organization and biogenesis;metabolic process	mitochondrion;ribosome	nucleotide binding;protein binding;RNA binding;structural molecule activity	0.719	0.501	0.70
P38071	Enoyl-[acyl-carrier protein] reductase [NADPH, B-specific], mitochondrial [OS=Saccharomyces cerevisiae S288c]	852314	ETR1; YBR026C	metabolic process	mitochondrion;organelle lumen	catalytic activity;DNA binding;metal ion binding	9	6.305	0.70
P07806	Valine--tRNA ligase, mitochondrial [OS=Saccharomyces cerevisiae S288c]	852986	VAS1; YGR094W	metabolic process;regulation of biological process	cytoplasm;cytosol;mitochondrion	catalytic activity;nucleotide binding	0.668	0.468	0.70
P36521	54S ribosomal protein L11, mitochondrial [OS=Saccharomyces cerevisiae S288c]	851325	MRPL11; YDL202W	cell organization and biogenesis;metabolic process	mitochondrion;ribosome	structural molecule activity	0.624	0.438	0.70
P38325	Mitochondrial outer membrane protein OM14 [OS=Saccharomyces cerevisiae S288c]	852531	OM14; YBR230C	transport	membrane;mitochondrion		18.307	12.895	0.70
P38300	Inner membrane mitoribosome receptor MBA1, mitochondrial [OS=Saccharomyces cerevisiae S288c]	852483	MBA1; YBR185C	cell organization and biogenesis;regulation of biological process	membrane;mitochondrion		0.585	0.413	0.71
P38072	Protein SCO2, mitochondrial [OS=Saccharomyces cerevisiae S288c]	852312	SCO2; YBR024W	cell organization and biogenesis;cellular homeostasis;metabolic process;transport	membrane;mitochondrion	antioxidant activity;catalytic activity;metal ion binding	0.551	0.389	0.71
P48015	Aminomethyltransferase, mitochondrial [OS=Saccharomyces cerevisiae S288c]	851582	GCV1; YDR019C	metabolic process	mitochondrion	catalytic activity;protein binding	11.217	8.047	0.72
P36516	54S ribosomal protein L3, mitochondrial [OS=Saccharomyces cerevisiae S288c]	855039	MRPL3; YMR024W	cell organization and biogenesis;metabolic process;response to stimulus	mitochondrion;nucleus;ribosome	catalytic activity;RNA binding;structural molecule activity	0.374	0.269	0.72
P39965	probable proline--tRNA ligase, mitochondrial [OS=Saccharomyces cerevisiae S288c]	856820	AIM10; YER087W	metabolic process	cytoplasm;mitochondrion	catalytic activity;nucleotide binding;RNA binding	0.266	0.194	0.73
P25038	Translation initiation factor IF-2, mitochondrial [OS=Saccharomyces	854135	IFM1; YOL023	cell organization and biogenesis;metabolic process	mitochondrion;nucleus	catalytic activity;nucleotide	0.233	0.17	0.73

	cerevisiae S288c]		W			binding;RNA binding			
Q01217	Protein ARG5,6, mitochondrial [OS=Saccharomyces cerevisiae S288c]	856800	ARG5,6; YER069W	metabolic process;regulation of biological process	cytoplasm;mitochondrion;organelle lumen	catalytic activity;nucleotide binding;protein binding	0.182	0.134	0.74
P32904	54S ribosomal protein L6, mitochondrial [OS=Saccharomyces cerevisiae S288c]	856552	MRPL6; YHR147C	cell organization and biogenesis;metabolic process	mitochondrion;ribosome	RNA binding;structural molecule activity	1.054	0.778	0.74
P40086	Cytochrome c oxidase assembly protein cox15 [OS=Saccharomyces cerevisiae S288c]	856884	COX15; YER141W	cell organization and biogenesis;metabolic process;transport	membrane;mitochondrion	catalytic activity;transporter activity	1.555	1.154	0.74
P43567	alanine--glyoxylate aminotransferase 1 [OS=Saccharomyces cerevisiae S288c]	850514	AGX1; YFL030W	metabolic process	cytosol;mitochondrion	catalytic activity	2.162	1.61	0.74
P40508	Uncharacterized protein YIL077C [OS=Saccharomyces cerevisiae S288c]	854733	YIL077C; YIL077C		mitochondrion		0.896	0.668	0.75
P40961	prohibitin-1 [OS=Saccharomyces cerevisiae S288c]	853033	PHB1; YGR132C	cell organization and biogenesis;metabolic process;regulation of biological process	membrane;mitochondrion	protein binding	5.952	4.456	0.75
P38885	Altered inheritance of mitochondria protein 46, mitochondrial [OS=Saccharomyces cerevisiae S288c]	856606	AIM46; YHR199C		mitochondrion	catalytic activity	0.73	0.551	0.75
P36101	tRNA threonylcarbamoyladenosine dehydratase 2 [OS=Saccharomyces cerevisiae S288c]	853841	TCD2; YKL027W; YKL027W	metabolic process	cytosol;membrane;mitochondrion	catalytic activity;nucleotide binding	0.616	0.468	0.76
Q03327	Mitochondrial fusion and transport protein Ugo1 [OS=Saccharomyces cerevisiae S288c]	852081	UGO1; YDR470C	cell organization and biogenesis;metabolic process	membrane;mitochondrion	protein binding;structural molecule activity	0.557	0.425	0.76
P21375	Fumarate reductase 2 [OS=Saccharomyces cerevisiae S288c]	853510	OSM1; YJR051W	metabolic process	endoplasmic reticulum;mitochondrion	catalytic activity;nucleotide binding	0.45	0.346	0.77
P36066	Protein MRG3-like [OS=Saccharomyces cerevisiae S288c]	853725	YKL133C; YKL133C	metabolic process	membrane;mitochondrion	protein binding	0.433	0.334	0.77
Q06567	ABC1 family protein MCP2 [OS=Saccharomyces cerevisiae S288c]	850955	YLR253W; MCP2; YLR253W	cell organization and biogenesis	membrane;mitochondrion		0.389	0.301	0.77
P25374	Cysteine desulfurase, mitochondrial [OS=Saccharomyces cerevisiae S288c]	850343	NFS1; YCL017C	cell organization and biogenesis;cellular homeostasis;metabolic process	mitochondrion;nucleus	catalytic activity;metal ion binding;protein binding	2.3	1.783	0.78
P39525	3-oxoacyl-[acyl-carrier-protein] synthase homolog [OS=Saccharomyces cerevisiae S288c]	856790	CEM1; YER061C	metabolic process	mitochondrion	catalytic activity	0.874	0.688	0.79
P40008	Protein FMP52, mitochondrial [OS=Saccharomyces cerevisiae S288c]	856721	FMP52; YER004W		endoplasmic reticulum;membrane;mitochondrion	catalytic activity	1.738	1.371	0.79
P39925	Mitochondrial respiratory chain complexes assembly protein AFG3 [OS=Saccharomyces cerevisiae S288c]	856737	AFG3; YER017C	cell organization and biogenesis;metabolic process;response to stimulus;transport	membrane;mitochondrion	catalytic activity;metal ion binding;nucleotide binding;protein binding	0.823	0.65	0.79
P32331	Carrier protein YMC1, mitochondrial [OS=Saccharomyces cerevisiae S288c]	856171	YMC1; YPR058W	metabolic process;transport	membrane;mitochondrion;vacuole	structural molecule activity;transporter activity	1.581	1.254	0.79
P17558	37S ribosomal protein PET123, mitochondrial [OS=Saccharomyces cerevisiae S288c]	854329	PET123; YOR158W	cell organization and biogenesis;metabolic process	mitochondrion;ribosome	structural molecule activity	1.448	1.154	0.80
P32860	NifU-like protein, mitochondrial [OS=Saccharomyces cerevisiae S288c]	853826	NFU1; YKL040C	cell organization and biogenesis;cellular homeostasis;metabolic process	mitochondrion;organelle lumen	metal ion binding	2.652	2.162	0.82
Q07651	SUR7 family protein FMP45 [OS=Saccharomyces cerevisiae S288c]	851304	FMP45; YDL222C	cell differentiation;cell organization and biogenesis	membrane;mitochondrion		7.377	6.017	0.82
P07143	Cytochrome c1, heme protein, mitochondrial [OS=Saccharomyces cerevisiae S288c]	854231	CYT1; YOR065W	metabolic process;transport	cytosol;membrane;mitochondrion	metal ion binding	13.251	10.938	0.83
Q03104	Meiotic sister chromatid recombination protein 1 [OS=Saccharomyces cerevisiae S288c]	854911	MSC1; YML128C	metabolic process	endoplasmic reticulum;membrane;mitochondrion		5.31	4.412	0.83
Q01852	Mitochondrial import inner membrane translocase subunit TIM44 [OS=Saccharomyces cerevisiae S288c]	854790	TIM44; YIL022W	transport	membrane;mitochondrion	nucleotide binding;protein binding	5.404	4.52	0.84
P00927	Threonine dehydratase, mitochondrial [OS=Saccharomyces cerevisiae S288c]	856819	ILV1; YER086W	metabolic process	cytoplasm;mitochondrion	catalytic activity	2.257	1.894	0.84
Q04013	Citrate/oxoglutarate carrier protein [OS=Saccharomyces cerevisiae S288c]	855282	YHM2; YMR241W	cell organization and biogenesis;metabolic process;transport	membrane;mitochondrion	DNA binding;structural molecule	4.012	3.467	0.86

						activity;transporter activity			
P38988	Mitochondrial GTP/GDP carrier protein 1 [OS=Saccharomyces cerevisiae S288c]	851329	GGC1; YDL198C	cell organization and biogenesis;cellular homeostasis;metabolic process;transport	membrane;mitochondrion	structural molecule activity;transporter activity	3.642	3.16	0.87
P09457	ATP synthase subunit 5, mitochondrial [OS=Saccharomyces cerevisiae S288c]	851892	ATP5; YDR298C	metabolic process;transport	membrane;mitochondrion	catalytic activity;transporter activity	11.915	10.365	0.87
P46681	D-lactate dehydrogenase [cytochrome] 2, mitochondrial [OS=Saccharomyces cerevisiae S288c]	851376	DLD2; YDL178W	metabolic process	mitochondrion;organelle lumen	catalytic activity;nucleotide binding;protein binding	1.264	1.102	0.87
P50085	Prohibitin-2 [OS=Saccharomyces cerevisiae S288c]	853146	PHB2; YGR231C	cell organization and biogenesis;metabolic process;regulation of biological process	membrane;mitochondrion	protein binding	4.337	3.806	0.88
Q02776	Mitochondrial import inner membrane translocase subunit TIM50 [OS=Saccharomyces cerevisiae S288c]	856042	TIM50; YPL063W	cell organization and biogenesis;metabolic process;transport	membrane;mitochondrion	catalytic activity;protein binding;transporter activity	9.857	9	0.91
Q08968	UPF0061 protein FMP40 [OS=Saccharomyces cerevisiae S288c]	855879	FMP40; YPL222W		mitochondrion	protein binding	1.721	1.588	0.92
P53598	Succinyl-CoA ligase [ADP-forming] subunit alpha, mitochondrial [OS=Saccharomyces cerevisiae S288c]	854310	LSC1; YOR142W	metabolic process	cytosol;mitochondrion	catalytic activity;nucleotide binding	11.743	11.743	1.00
P00410	Cytochrome c oxidase subunit 2 [OS=Saccharomyces cerevisiae S288c]	854622	COX2; Q0250	metabolic process;transport	membrane;mitochondrion	catalytic activity;metal ion binding;protein binding;transporter activity	3.642	3.642	1.00
P40513	Mitochondrial acidic protein MAM33 [OS=Saccharomyces cerevisiae S288c]	854740	MAM33; YIL070C	metabolic process;regulation of biological process	mitochondrion;organelle lumen	translation regulator activity	2.831	2.831	1.00
Q08023	Protein FMP25, mitochondrial [OS=Saccharomyces cerevisiae S288c]	850766	FMP25; YLR077W	cell organization and biogenesis	membrane;mitochondrion		0.719	0.719	1.00
Q04728	Arginine biosynthesis bifunctional protein ArgJ, mitochondrial [OS=Saccharomyces cerevisiae S288c]	855084	ARG7; YMR062C	metabolic process	mitochondrion;organelle lumen	catalytic activity	0.52	0.52	1.00
P38797	Protein phosphatase 2C homolog 7, mitochondrial [OS=Saccharomyces cerevisiae S288c]	856475	PTC7; YHR076W	metabolic process	mitochondrion	catalytic activity;metal ion binding;protein binding	0.874	0.874	1.00
P36517	54S ribosomal protein L4, mitochondrial [OS=Saccharomyces cerevisiae S288c]	851160	MRPL4; YLR439W	cell organization and biogenesis;metabolic process	mitochondrion;ribosome	structural molecule activity	0.874	0.874	1.00
P38079	protein YRO2 [OS=Saccharomyces cerevisiae S288c]	852343	YRO2; YBR054W	transport	endoplasmic reticulum;membrane;mitochondrion	transporter activity	1.424	1.424	1.00
P32611	54S ribosomal protein RML2, mitochondrial [OS=Saccharomyces cerevisiae S288c]	856660	RML2; YEL050C	cell organization and biogenesis;metabolic process	mitochondrion;ribosome	catalytic activity;RNA binding;structural molecule activity	0.638	0.638	1.00
Q06668	Methyltransferase OMS1, mitochondrial [OS=Saccharomyces cerevisiae S288c]	851911	OMS1; YDR316W	metabolic process	membrane;mitochondrion	catalytic activity	0.389	0.389	1.00
P32453	Protein ATP11, mitochondrial [OS=Saccharomyces cerevisiae S288c]	855401	ATP11; YNL315C	cell organization and biogenesis	mitochondrion	protein binding	0.585	0.585	1.00
P35180	mitochondrial import receptor subunit Tom20 [OS=Saccharomyces cerevisiae S288c]	852973	TOM20; YGR082W	cell organization and biogenesis;transport	membrane;mitochondrion	protein binding;transporter activity	0.425	0.425	1.00
Q3E824	Uncharacterized protein YOR020W-A [OS=Saccharomyces cerevisiae S288c]	1466480	YOR020W-A; YOR020W-A	metabolic process;transport	membrane;mitochondrion		2.162	2.162	1.00
P37299	cytochrome b-c1 complex subunit 10 [OS=Saccharomyces cerevisiae S288c]	856390	QCR10; YHR001W-A	metabolic process;transport	membrane;mitochondrion	catalytic activity;transporter activity	2.981	2.981	1.00
P49334	Mitochondrial import receptor subunit tom22 [OS=Saccharomyces cerevisiae S288c]	855592	TOM22; YNL131W	cell organization and biogenesis;transport	membrane;mitochondrion	protein binding;transporter activity	0.668	0.668	1.00
P39112	Exoribonuclease II, mitochondrial [OS=Saccharomyces cerevisiae S288c]	855331	DSS1; YMR287C	metabolic process	mitochondrion;organelle lumen	catalytic activity;RNA binding	0.087	0.087	1.00
P14693	sorting assembly machinery 35 kDa subunit [OS=Saccharomyces cerevisiae S288c]	856483	SAM35; YHR083W	cell organization and biogenesis;transport	membrane;mitochondrion	protein binding	0.334	0.334	1.00
P25345	Asparagine--tRNA ligase, mitochondrial [OS=Saccharomyces cerevisiae S288c]	850388	SLM5; YCR024C	metabolic process	cytoplasm;mitochondrion;organelle lumen	catalytic activity;nucleotide binding	0.172	0.172	1.00
P34222	Peptidyl-tRNA hydrolase 2 [OS=Saccharomyces cerevisiae S288c]	852223	PTH2; YBL057C	regulation of biological process	cytoplasm;cytosol;membrane;mitochondrion	catalytic activity;protein binding	0.425	0.425	1.00

P28737	Protein MSP1 [OS=Saccharomyces cerevisiae S288c]	852915	MSP1; YGR028W	cell organization and biogenesis;metabolic process;regulation of biological process;response to stimulus;transport	membrane;mitochondrion;nucleus	catalytic activity;nucleotide binding;protein binding;RNA binding	0.413	0.413	1.00
P21306	ATP synthase subunit epsilon, mitochondrial [OS=Saccharomyces cerevisiae S288c]	855857	ATP15; YPL271W	metabolic process;transport	membrane;mitochondrion	catalytic activity;transporter activity	2.162	2.162	1.00
Q01532-1	Cysteine proteinase 1, mitochondrial [OS=Saccharomyces cerevisiae S288c]		MCY1	metabolic process;regulation of biological process;response to stimulus	cytoplasm;mitochondrion	catalytic activity;DNA binding;RNA binding	0.179	0.179	1.00
Q03557	Glutamyl-tRNA(Gln) amidotransferase subunit A, mitochondrial [OS=Saccharomyces cerevisiae S288c]	855338	HER2; YMR293C	cell organization and biogenesis;metabolic process	mitochondrion	catalytic activity;nucleotide binding	0.166	0.166	1.00
P53732	37S ribosomal protein S12, mitochondrial [OS=Saccharomyces cerevisiae S288c]	855772	MRPS12; YNR036C	cell organization and biogenesis;metabolic process	mitochondrion;ribosome	structural molecule activity	0.668	0.668	1.00
P32792	UPF0744 protein YSC83 [OS=Saccharomyces cerevisiae S288c]	856410	YSC83; YHR017W		membrane;mitochondrion		0.233	0.233	1.00
P23833	Protein SCO1, mitochondrial [OS=Saccharomyces cerevisiae S288c]	852325	SCO1; YBR037C	cell organization and biogenesis;cellular homeostasis;response to stimulus;transport	membrane;mitochondrion	antioxidant activity;catalytic activity;metal ion binding	0.468	0.468	1.00
Q05648	MIOREX complex component 10 [OS=Saccharomyces cerevisiae S288c]	851876	YDR282C; MRX10; YDR282C		membrane;mitochondrion		0.222	0.222	1.00
P40496	37S ribosomal protein S25, mitochondrial [OS=Saccharomyces cerevisiae S288c]	854715	RSM25; YIL093C	cell organization and biogenesis;metabolic process	mitochondrion;ribosome	structural molecule activity	0.468	0.468	1.00
P50087	MICOS subunit MIC26 [OS=Saccharomyces cerevisiae S288c]	853150	MOS2; MIC26; YGR235C	cell organization and biogenesis	membrane;mitochondrion		0.638	0.638	1.00
P33759	37S ribosomal protein S5, mitochondrial [OS=Saccharomyces cerevisiae S288c]	852553	MRPS5; YBR251W	cell organization and biogenesis;metabolic process	mitochondrion;ribosome	RNA binding;structural molecule activity	0.413	0.413	1.00
P18496	Mitochondrial ATPase complex subunit ATP10 [OS=Saccharomyces cerevisiae S288c]	851109	ATP10; YLR393W	cell organization and biogenesis	cytoplasm;membrane;mitochondrion	protein binding	0.413	0.413	1.00
P18900	hexaprenyl pyrophosphate synthase, mitochondrial [OS=Saccharomyces cerevisiae S288c]	852288	COQ1; YBR003W	metabolic process	membrane;mitochondrion	catalytic activity;metal ion binding;protein binding	0.166	0.166	1.00
Q07821	Iron-sulfur assembly protein 1 [OS=Saccharomyces cerevisiae S288c]	850632	ISA1; YLL027W	cell organization and biogenesis;metabolic process	mitochondrion;organelle lumen	metal ion binding;structural molecule activity	0.136	0.136	1.00
Q06630	Mitochondrial homologous recombination protein 1 [OS=Saccharomyces cerevisiae S288c]	851890	MHR1; YDR296W	cell organization and biogenesis;metabolic process;regulation of biological process;response to stimulus	mitochondrion;nucleus	catalytic activity;DNA binding;structural molecule activity	0.359	0.359	1.00
P32606	putative mitochondrial translation system component PET127 [OS=Saccharomyces cerevisiae S288c]	854182	PET127; YOR017W	cell organization and biogenesis;metabolic process;regulation of biological process	mitochondrion		0.099	0.099	1.00
P08525	Cytochrome b-c1 complex subunit 8 [OS=Saccharomyces cerevisiae S288c]	853273	QCR8; YJL166W	metabolic process;transport	membrane;mitochondrion	catalytic activity;transporter activity	0.585	0.585	1.00
Q02608	37S ribosomal protein S16, mitochondrial [OS=Saccharomyces cerevisiae S288c]	856094	MRPS16; YPL013C	cell organization and biogenesis;metabolic process	mitochondrion;ribosome	structural molecule activity	0.389	0.389	1.00
P40581	peroxiredoxin HYR1 [OS=Saccharomyces cerevisiae S288c]	854855	HYR1; YIR037W	metabolic process;response to stimulus	cytoplasm;cytosol;mitochondrion;organelle lumen	antioxidant activity;catalytic activity	0.259	0.259	1.00
P38122	3-methyl-2-oxobutanoate hydroxymethyltransferase [OS=Saccharomyces cerevisiae S288c]	852474	ECM31; YBR176W	metabolic process	mitochondrion	catalytic activity	0.122	0.122	1.00
P32463	Acyl carrier protein, mitochondrial [OS=Saccharomyces cerevisiae S288c]	853642	ACP1; YKL192C	metabolic process;transport	mitochondrion		0.259	0.259	1.00
P04039	Cytochrome c oxidase polypeptide VIII, mitochondrial [OS=Saccharomyces cerevisiae S288c]	851111	COX8; YLR395C	metabolic process;transport	membrane;mitochondrion	catalytic activity;transporter activity	0.778	0.778	1.00
Q05779	Ubiquinone biosynthesis protein COQ9, mitochondrial [OS=Saccharomyces cerevisiae S288c]	850898	COQ9; YLR201C	metabolic process	membrane;mitochondrion		0.166	0.166	1.00
P03879	Intron-encoded RNA maturase bI4 [OS=Saccharomyces cerevisiae S288c]	854582	BI4; Q0120	metabolic process	membrane;mitochondrion	catalytic activity;RNA binding	0.179	0.179	1.00
P46998	mitochondrial membrane protein FMP33 [OS=Saccharomyces cerevisiae S288c]	853279	FMP33; YJL161W		membrane;mitochondrion		0.334	0.334	1.00
Q12487	54S ribosomal protein L23, mitochondrial [OS=Saccharomyces cerevisiae S288c]	854321	MRPL23; YOR15	cell organization and biogenesis;metabolic process	mitochondrion;ribosome;vacuole	RNA binding;structural molecule activity	0.233	0.233	1.00

			0W						
Q04689	Altered inheritance of mitochondria protein 32 [OS=Saccharomyces cerevisiae S288c]	854955	AIM32; YML050W				0.11	0.11	1.00
P07253	cytochrome B pre-mRNA-processing protein 6 [OS=Saccharomyces cerevisiae S288c]	852417	CBP6; YBR120C	cell organization and biogenesis;metabolic process;regulation of biological process	mitochondrion;ribosome		0.389	0.389	1.00
P00045	Cytochrome c iso-2 [OS=Saccharomyces cerevisiae S288c]	856672	CYC7; YEL039C	metabolic process;transport	mitochondrion	metal ion binding	0.259	0.259	1.00
P53724	54S ribosomal protein L50, mitochondrial [OS=Saccharomyces cerevisiae S288c]	855756	MRPL50; YNR022C	cell organization and biogenesis;metabolic process	mitochondrion;ribosome	structural molecule activity	0.585	0.585	1.00
P27882	Mitochondrial FAD-linked sulfhydryl oxidase erv1 [OS=Saccharomyces cerevisiae S288c]	852916	ERV1; YGR029W	cellular homeostasis;metabolic process;response to stimulus;transport	mitochondrion	catalytic activity;protein binding	0.212	0.212	1.00
P36046	Mitochondrial intermembrane space import and assembly protein 40 [OS=Saccharomyces cerevisiae S288c]	853639	MIA40; YKL195W	cellular component movement;metabolic process;response to stimulus;transport	membrane;mitochondrion	catalytic activity;protein binding;transporter activity	1.555	1.783	1.15
P32787	Mitochondrial genome maintenance protein MGM101 [OS=Saccharomyces cerevisiae S288c]	853609	MGM101; YJR144W	cell organization and biogenesis;metabolic process;response to stimulus	chromosome;mitochondrion	DNA binding	3.281	3.833	1.17
P39987	Heat shock protein SSC3, mitochondrial [OS=Saccharomyces cerevisiae S288c]	856682	ECM10; YEL030W	cell organization and biogenesis;metabolic process;transport	mitochondrion	nucleotide binding;protein binding	1.637	1.976	1.21
P38297	Mitofusin FZO1 [OS=Saccharomyces cerevisiae S288c]	852477	FZO1; YBR179C	cell organization and biogenesis	membrane;mitochondrion	catalytic activity;nucleotide binding;protein binding	0.248	0.304	1.23
P32266	Dynamin-like GTPase MGM1, mitochondrial [OS=Saccharomyces cerevisiae S288c]	854386	MGM1; YOR211C	cell organization and biogenesis	membrane;mitochondrion	catalytic activity;nucleotide binding;protein binding	0.179	0.228	1.27
P38088	Glycine--tRNA ligase 1, mitochondrial [OS=Saccharomyces cerevisiae S288c]		GRS1	metabolic process	cytoplasm;mitochondrion;organelle lumen	catalytic activity;nucleotide binding;protein binding	0.212	0.271	1.28
P38771	Ribosome-recycling factor, mitochondrial [OS=Saccharomyces cerevisiae S288c]	856433	RRF1; YHR038W	cell organization and biogenesis;metabolic process	mitochondrion	RNA binding	0.968	1.254	1.30
Q05931	Heat shock protein SSQ1, mitochondrial [OS=Saccharomyces cerevisiae S288c]	851084	SSQ1; YLR369W	cell organization and biogenesis;metabolic process	mitochondrion;organelle lumen	nucleotide binding;protein binding	0.679	0.884	1.30
P25573	Mitochondrial inner membrane i-AAA protease supercomplex subunit MGR1 [OS=Saccharomyces cerevisiae S288c]	850313	MGR1; YCL044C	metabolic process	membrane;mitochondrion	protein binding	0.425	0.557	1.31
P36534	54S ribosomal protein L40, mitochondrial [OS=Saccharomyces cerevisiae S288c]	855930	MRPL40; YPL173W	cell organization and biogenesis;metabolic process	mitochondrion;ribosome	protein binding;structural molecule activity	0.585	0.778	1.33
P33310	ATP-dependent permease MDL1, mitochondrial [OS=Saccharomyces cerevisiae S288c]	850885	MDL1; YLR188W	transport	membrane;mitochondrion	catalytic activity;nucleotide binding;transporter activity	0.528	0.725	1.37
P32799	Cytochrome c oxidase subunit 6A, mitochondrial [OS=Saccharomyces cerevisiae S288c]	852684	COX13; YGL191W	cell organization and biogenesis;metabolic process;regulation of biological process;transport	membrane;mitochondrion	catalytic activity;enzyme regulator activity;transporter activity	2.162	2.981	1.38
P38323	ATP-dependent clpX-like chaperone, mitochondrial [OS=Saccharomyces cerevisiae S288c]	852528	MCX1; YBR227C	metabolic process	membrane;mitochondrion;organelle lumen	catalytic activity;nucleotide binding	0.225	0.311	1.38
Q08223	Altered inheritance of mitochondria protein 39, mitochondrial [OS=Saccharomyces cerevisiae S288c]	854103	AIM39; YOL053W		cytosol;membrane;mitochondrion		0.259	0.359	1.39
P53230	Phosphatidate cytidylyltransferase, mitochondrial [OS=Saccharomyces cerevisiae S288c]	852937	TAM41; YGR046W	metabolic process	membrane;mitochondrion;organelle lumen	catalytic activity	0.292	0.407	1.39
P48360	Probable NADPH:adrenodoxin oxidoreductase, mitochondrial [OS=Saccharomyces cerevisiae S288c]	851982	ARH1; YDR376W	cellular homeostasis;metabolic process;transport	cytoplasm;membrane;mitochondrion;organelle lumen	catalytic activity	0.16	0.25	1.56
P47140	Altered inheritance rate of mitochondria protein 25 [OS=Saccharomyces cerevisiae S288c]	853563	AIM25; YJR100C	cell organization and biogenesis	membrane;mitochondrion	transporter activity	0.233	0.369	1.58
P36163	mitochondrial metalloendopeptidase OMA1 [OS=Saccharomyces cerevisiae S288c]	853962	OMA1; YKR087C	metabolic process	membrane;mitochondrion	catalytic activity;metal ion binding	0.245	0.389	1.59
P42847	37S ribosomal protein S18, mitochondrial [OS=Saccharomyces cerevisiae S288c]	855410	MRPS18; YNL306W	cell organization and biogenesis;metabolic process	mitochondrion;ribosome	RNA binding;structural molecule activity	0.334	0.54	1.62
Q03798	Altered inheritance of mitochondria protein 36, mitochondrial [OS=Saccharomyces cerevisiae S288c]	855189	AIM36; YMR157C		membrane;mitochondrion		0.438	0.833	1.90

Q12204	Probable phospholipase YOR022C, mitochondrial [OS=Saccharomyces cerevisiae S288c]	854187	YOR022C; YOR022C	metabolic process	mitochondrion	catalytic activity;metal ion binding	0.054	0.11	2.04
P22438	Methionine--tRNA ligase, mitochondrial [OS=Saccharomyces cerevisiae S288c]	853081	MSM1; YGR171C	metabolic process	cytoplasm;cytosol;mitochondrion;organelle lumen	catalytic activity;nucleotide binding	0.066	0.136	2.06
P38127	mitochondrial carrier protein RIM2 [OS=Saccharomyces cerevisiae S288c]	852491	RIM2; YBR192W	cell organization and biogenesis;metabolic process;transport	membrane;mitochondrion	structural molecule activity;transporter activity	0.585	1.239	2.12
P25270	rRNA methyltransferase 1, mitochondrial [OS=Saccharomyces cerevisiae S288c]	854376	MRM1; YOR201C	metabolic process	mitochondrion	catalytic activity;RNA binding	0.129	0.274	2.12
P38816	thioredoxin reductase 2, mitochondrial [OS=Saccharomyces cerevisiae S288c]	856506	TRR2; YHR106W	cellular homeostasis;metabolic process;regulation of biological process;response to stimulus	cytoplasm;mitochondrion	antioxidant activity;catalytic activity	0.136	0.292	2.15
P39722	Mitochondrial Rho GTPase 1 [OS=Saccharomyces cerevisiae S288c]	851249	GEM1; YAL048C	cell organization and biogenesis;regulation of biological process;response to stimulus;transport	membrane;mitochondrion	catalytic activity;metal ion binding;nucleotide binding	0.059	0.189	3.20
Q04935	Cytochrome c oxidase protein 20, mitochondrial [OS=Saccharomyces cerevisiae S288c]	851817	COX20; YDR231C	cell organization and biogenesis;metabolic process	membrane;mitochondrion	protein binding	0.194	0.701	3.61
P38705	Serine--tRNA ligase, mitochondrial [OS=Saccharomyces cerevisiae S288c]	856402	DIA4; YHR011W	metabolic process	cytoplasm;mitochondrion;organelle lumen	catalytic activity;nucleotide binding	0.072	0.322	4.47

Supplemental Table S10. The relative concentrations of proteins in mitochondria purified from WT or *ups2Δ* cells cultured without LCA. Mitochondria were purified from WT or *ups2Δ* cells recovered on day 2 of cell culturing without LCA. Mass spectrometry-based identification and quantitation of proteins recovered in purified mitochondria were performed as described in Materials and methods. The exponentially modified protein abundance index (emPAI) provided by the ″Proteome Discoverer″ software was used to calculate the relative abundance of mitochondrial proteins between specified datasets. Data on the relative concentrations of mitochondrial proteins are presented as means of 2 independent experiments.

Accession	Description	Entrez Gene ID	Gene ID	Biological Process	Cellular Component	Molecular Function	emPAI WT	emPAI *ups2*	Ratio *ups2* / WT
P00431	cytochrome c peroxidase, mitochondrial [OS=Saccharomyces cerevisiae S288c]	853940	CCP1; YKR066C	metabolic process;response to stimulus	mitochondrion;organelle lumen	antioxidant activity;catalytic activity;metal ion binding;protein binding	2.162	0.093	0.04
P39522	Dihydroxy-acid dehydratase, mitochondrial [OS=Saccharomyces cerevisiae S288c]	853473	ILV3; YJR016C	metabolic process	mitochondrion	catalytic activity;metal ion binding	39.37	2.054	0.05
P25605	Acetolactate synthase small subunit, mitochondrial [OS=Saccharomyces cerevisiae S288c]	850348	ILV6; YCL009C	metabolic process;regulation of biological process	mitochondrion	catalytic activity;enzyme regulator activity	11.743	0.624	0.05
Q06678	54S ribosomal protein L35, mitochondrial [OS=Saccharomyces cerevisiae S288c]	851921	MRPL35; YDR322W	cell organization and biogenesis;metabolic process	mitochondrion;ribosome	structural molecule activity	1.424	0.093	0.07
P19955	37S ribosomal protein YMR-31, mitochondrial [OS=Saccharomyces cerevisiae S288c]	850610	YMR31; YFR049W	cell organization and biogenesis;metabolic process	mitochondrion;ribosome	catalytic activity;protein binding;structural molecule activity	15.681	1.154	0.07
P53219	Abhydrolase domain-containing protein IMO32 [OS=Saccharomyces cerevisiae S288c]	852919	IMO32; YGR031W	metabolic process;transport	mitochondrion	catalytic activity	1.404	0.116	0.08
P81449	ATP synthase subunit e, mitochondrial [OS=Saccharomyces cerevisiae S288c]	851922	TIM11; YDR322C-A	cell organization and biogenesis;metabolic process;transport	membrane;mitochondrion	catalytic activity;structural molecule activity;transporter activity	9	0.778	0.09
P11914	Mitochondrial-processing peptidase subunit alpha [OS=Saccharomyces cerevisiae S288c]	856419	MAS2; YHR024C	metabolic process;transport	membrane;mitochondrion;organelle lumen	catalytic activity;metal ion binding;protein binding	2.311	0.202	0.09
Q06892	NADH kinase POS5, mitochondrial [OS=Saccharomyces cerevisiae S288c]	855913	POS5; YPL188W	metabolic process;response to stimulus	mitochondrion;organelle lumen	catalytic activity;nucleotide binding	2.511	0.233	0.09
P39676	Flavohemoprotein [OS=Saccharomyces cerevisiae S288c]	853149	YHB1; YGR234W	metabolic process;response to stimulus	cytoplasm;cytosol;mitochondrion;nucleus;organelle lumen	catalytic activity;metal ion binding;protein binding	4.179	0.389	0.09
P37267	Assembly factor cbp4 [OS=Saccharomyces cerevisiae S288c]		CBP4	cell organization and biogenesis	membrane;mitochondrion		5.579	0.52	0.09
P25039	Elongation factor G, mitochondrial [OS=Saccharomyces cerevisiae S288c]	850758	MEF1; YLR069C	cell organization and biogenesis;metabolic process	cytosol;mitochondrion	catalytic activity;nucleotide binding;RNA binding	1.024	0.099	0.10
P32445	Single-stranded DNA-binding protein RIM1, mitochondrial [OS=Saccharomyces cerevisiae S288c]	850395	RIM1; YCR028C-A	cell organization and biogenesis;metabolic process;regulation of biological process	mitochondrion	DNA binding	6.743	0.668	0.10
P38088	Glycine--tRNA ligase 1, mitochondrial [OS=Saccharomyces cerevisiae S288c]		GRS1	metabolic process	cytoplasm;mitochondrion;organelle lumen	catalytic activity;nucleotide binding;protein binding	0.468	0.049	0.10
Q02486	ARS-binding factor 2, mitochondrial [OS=Saccharomyces cerevisiae S288c]	855094	ABF2; YMR072W	cell organization and biogenesis;metabolic process;regulation of biological process	chromosome;mitochondrion;nucleus	DNA binding	69.17	7.377	0.11
P08525	Cytochrome b-c1 complex subunit 8 [OS=Saccharomyces cerevisiae S288c]	853273	QCR8; YJL166W	metabolic process;transport	membrane;mitochondrion	catalytic activity;transporter activity	5.31	0.585	0.11
P33416	Heat shock protein 78, mitochondrial [OS=Saccharomyces cerevisiae S288c]	851845	HSP78; YDR258C	cell organization and biogenesis;metabolic process;regulation of biological process;response to stimulus	mitochondrion;organelle lumen	catalytic activity;nucleotide binding;protein binding	12.525	1.383	0.11
P23180	Probable oxidoreductase AIM17 [OS=Saccharomyces cerevisiae S288c]	856365	AIM17; YHL021C	cell organization and biogenesis;metabolic process	mitochondrion	catalytic activity;metal ion binding	9	0.995	0.11

Q02784	Monothiol glutaredoxin-5, mitochondrial [OS=Saccharomyces cerevisiae S288c]	856048	GRX5; YPL059W	cell organization and biogenesis;cellular homeostasis;metabolic process;regulation of biological process;response to stimulus	mitochondrion;organelle lumen	catalytic activity;metal ion binding	2.594	0.292	0.11
P00128	Cytochrome b-c1 complex subunit 7 [OS=Saccharomyces cerevisiae S288c]	852142	QCR7; YDR529C	cell organization and biogenesis;metabolic process;transport	membrane;mitochondrion	catalytic activity;transporter activity	15.681	1.783	0.11
P38771	Ribosome-recycling factor, mitochondrial [OS=Saccharomyces cerevisiae S288c]	856433	RRF1; YHR038W	cell organization and biogenesis;metabolic process	mitochondrion	RNA binding	1.254	0.145	0.12
P32898	Mitochondrial presequence protease [OS=Saccharomyces cerevisiae S288c]	852041	CYM1; YDR430C	metabolic process	mitochondrion	catalytic activity;metal ion binding	2.162	0.25	0.12
Q12031	Mitochondrial 2-methylisocitrate lyase [OS=Saccharomyces cerevisiae S288c]	856114	ICL2; YPR006C	metabolic process	mitochondrion;organelle lumen	catalytic activity	7.859	0.947	0.12
P06168	ketol-acid reductoisomerase, mitochondrial [OS=Saccharomyces cerevisiae S288c]	851069	ILV5; YLR355C	cell organization and biogenesis;metabolic process	mitochondrion	catalytic activity;DNA binding;metal ion binding	28.126	3.394	0.12
P21954	Isocitrate dehydrogenase [NADP], mitochondrial [OS=Saccharomyces cerevisiae S288c]	851493	IDP1; YDL066W	metabolic process	mitochondrion	catalytic activity;metal ion binding;nucleotide binding;protein binding	35.869	4.412	0.12
P40530	[Pyruvate dehydrogenase (acetyl-transferring)] kinase 1, mitochondrial [OS=Saccharomyces cerevisiae S288c]	854769	PKP1; YIL042C	metabolic process	mitochondrion;organelle lumen	catalytic activity;nucleotide binding;protein binding	0.931	0.116	0.12
P10834	protein PET54 [OS=Saccharomyces cerevisiae S288c]	853137	PET54; YGR222W	metabolic process;regulation of biological process	membrane;mitochondrion;organelle lumen	nucleotide binding;RNA binding;translation regulator activity	0.874	0.11	0.13
P00927	Threonine dehydratase, mitochondrial [OS=Saccharomyces cerevisiae S288c]	856819	ILV1; YER086W	metabolic process	cytoplasm;mitochondrion	catalytic activity	3.375	0.425	0.13
P12687	54S ribosomal protein L2, mitochondrial [OS=Saccharomyces cerevisiae S288c]	855727	MRP7; YNL005C	cell organization and biogenesis;metabolic process	mitochondrion;ribosome	catalytic activity;RNA binding;structural molecule activity	1.848	0.233	0.13
P39726	Glycine cleavage system H protein, mitochondrial [OS=Saccharomyces cerevisiae S288c]	851254	GCV3; YAL044C	metabolic process	mitochondrion	catalytic activity	9	1.154	0.13
P49095	Glycine dehydrogenase (Decarboxylating), mitochondrial [OS=Saccharomyces cerevisiae S288c]	855227	GCV2; YMR189W	metabolic process	cytosol;mitochondrion	catalytic activity	2.884	0.389	0.13
Q01163	37S ribosomal protein S23, mitochondrial [OS=Saccharomyces cerevisiae S288c]	852748	RSM23; YGL129C	cell organization and biogenesis;metabolic process	mitochondrion;ribosome	catalytic activity;nucleotide binding;RNA binding;structural molecule activity	2.695	0.365	0.14
Q03201	37S ribosomal protein S10, mitochondrial [OS=Saccharomyces cerevisiae S288c]	851611	RSM10; YDR041W	cell organization and biogenesis;metabolic process	mitochondrion;ribosome	structural molecule activity	1.424	0.194	0.14
P36151	Uncharacterized protein YKR070W [OS=Saccharomyces cerevisiae S288c]	853944	YKR070W; YKR070W	metabolic process	mitochondrion		1.976	0.274	0.14
P38323	ATP-dependent clpX-like chaperone, mitochondrial [OS=Saccharomyces cerevisiae S288c]	852528	MCX1; YBR227C	metabolic process	membrane;mitochondrion;organelle lumen	catalytic activity;nucleotide binding	0.501	0.07	0.14
P36060	NADH-cytochrome b5 reductase 2 [OS=Saccharomyces cerevisiae S288c]	853707	MCR1; YKL150W	metabolic process;response to stimulus	membrane;mitochondrion	catalytic activity;protein binding	19.691	2.793	0.14
P18496	Mitochondrial ATPase complex subunit ATP10 [OS=Saccharomyces cerevisiae S288c]	851109	ATP10; YLR393W	cell organization and biogenesis	cytoplasm;membrane;mitochondrion	protein binding	1.818	0.259	0.14
P15424	ATP-dependent RNA helicase MSS116, mitochondrial [OS=Saccharomyces cerevisiae S288c]	851775	MSS116; YDR194C	metabolic process;regulation of biological process	mitochondrion;organelle lumen	catalytic activity;nucleotide binding;RNA binding	1.154	0.166	0.14
P42847	37S ribosomal protein S18, mitochondrial [OS=Saccharomyces cerevisiae S288c]	855410	MRPS18; YNL306W	cell organization and biogenesis;metabolic process	mitochondrion;ribosome	RNA binding;structural molecule activity	1.054	0.155	0.15
P40961	prohibitin-1 [OS=Saccharomyces cerevisiae S288c]	853033	PHB1; YGR132C	cell organization and biogenesis;metabolic process;regulation of biological	membrane;mitochondrion	protein binding	9	1.336	0.15

				process					
P36517	54S ribosomal protein L4, mitochondrial [OS=Saccharomyces cerevisiae S288c]	851160	MRPL4; YLR439W	cell organization and biogenesis;metabolic process	mitochondrion;ribosome	structural molecule activity	1.565	0.233	0.15
P19414	Aconitate hydratase, mitochondrial [OS=Saccharomyces cerevisiae S288c]	851013	ACO1; YLR304C	cell organization and biogenesis;metabolic process;transport	cytoplasm;cytosol;mitochondrion;organelle lumen	catalytic activity;DNA binding;metal ion binding	66.002	10.053	0.15
P46998	mitochondrial membrane protein FMP33 [OS=Saccharomyces cerevisiae S288c]	853279	FMP33; YJL161W		membrane;mitochondrion		2.162	0.334	0.15
P28834	Isocitrate dehydrogenase [NAD] subunit 1, mitochondrial [OS=Saccharomyces cerevisiae S288c]	855691	IDH1; YNL037C	metabolic process;transport	cytosol;mitochondrion;organelle lumen	catalytic activity;metal ion binding;nucleotide binding;protein binding;RNA binding	15.238	2.36	0.15
P32799	Cytochrome c oxidase subunit 6A, mitochondrial [OS=Saccharomyces cerevisiae S288c]	852684	COX13; YGL191W	cell organization and biogenesis;metabolic process;regulation of biological process;transport	membrane;mitochondrion	catalytic activity;enzyme regulator activity;transporter activity	18.953	2.981	0.16
P38891	Branched-chain-amino-acid aminotransferase, mitochondrial [OS=Saccharomyces cerevisiae S288c]	856615	BAT1; YHR208W	metabolic process;regulation of biological process;response to stimulus	mitochondrion;organelle lumen	catalytic activity	15.876	2.511	0.16
P00447	Superoxide dismutase [Mn], mitochondrial [OS=Saccharomyces cerevisiae S288c]	856399	SOD2; YHR008C	metabolic process;response to stimulus	mitochondrion;organelle lumen	antioxidant activity;catalytic activity;metal ion binding	9	1.424	0.16
P08067	cytochrome b-c1 complex subunit Rieske, mitochondrial [OS=Saccharomyces cerevisiae S288c]	856689	RIP1; YEL024W	metabolic process;transport	membrane;mitochondrion	catalytic activity;metal ion binding;transporter activity	55.234	9	0.16
P04037	cytochrome c oxidase subunit 4, mitochondrial [OS=Saccharomyces cerevisiae S288c]	852688	COX4; YGL187C	cell organization and biogenesis;metabolic process;transport	membrane;mitochondrion	catalytic activity;metal ion binding;transporter activity	34.112	5.579	0.16
P36516	54S ribosomal protein L3, mitochondrial [OS=Saccharomyces cerevisiae S288c]	855039	MRPL3; YMR024W	cell organization and biogenesis;metabolic process;response to stimulus	mitochondrion;nucleus;ribosome	catalytic activity;RNA binding;structural molecule activity	1.043	0.172	0.16
Q00711	Succinate dehydrogenase [ubiquinone] flavoprotein subunit, mitochondrial [OS=Saccharomyces cerevisiae S288c]	853709	SDH1; YKL148C	metabolic process;transport	membrane;mitochondrion	catalytic activity;nucleotide binding;protein binding	18.619	3.072	0.16
P02992	elongation factor Tu, mitochondrial [OS=Saccharomyces cerevisiae S288c]	854359	TUF1; YOR187W	cell organization and biogenesis;metabolic process	mitochondrion	catalytic activity;nucleotide binding;RNA binding	23.245	3.924	0.17
P39006	Phosphatidylserine decarboxylase proenzyme 1, mitochondrial [OS=Saccharomyces cerevisiae S288c]	855552	PSD1; YNL169C	metabolic process;regulation of biological process	membrane;mitochondrion	catalytic activity	0.487	0.083	0.17
P53312	Succinyl-CoA ligase [ADP-forming] subunit beta, mitochondrial [OS=Saccharomyces cerevisiae S288c]	853159	LSC2; YGR244C	metabolic process	mitochondrion	catalytic activity;nucleotide binding	20.017	3.417	0.17
P12686	37S ribosomal protein MRP13, mitochondrial [OS=Saccharomyces cerevisiae S288c]	852975	MRP13; YGR084C	cell organization and biogenesis;metabolic process	mitochondrion;ribosome	structural molecule activity	1.512	0.259	0.17
P36534	54S ribosomal protein L40, mitochondrial [OS=Saccharomyces cerevisiae S288c]	855930	MRPL40; YPL173W	cell organization and biogenesis;metabolic process	mitochondrion;ribosome	protein binding;structural molecule activity	1.512	0.259	0.17
P32316	acetyl-CoA hydrolase [OS=Saccharomyces cerevisiae S288c]	852266	ACH1; YBL015W	metabolic process	cytoplasm;cytosol;mitochondrion	catalytic activity	29.539	5.136	0.17
P38885	Altered inheritance of mitochondria protein 46, mitochondrial [OS=Saccharomyces cerevisiae S288c]	856606	AIM46; YHR199C	mitochondrion	catalytic activity	1.404	0.245	0.17	
P00830	ATP synthase subunit beta, mitochondrial [OS=Saccharomyces cerevisiae S288c]	853585	ATP2; YJR121W	metabolic process;transport	cytosol;membrane;mitochondrion	catalytic activity;nucleotide binding;protein binding;transporter activity	99	17.478	0.18
P53140	Protein RMD9, mitochondrial [OS=Saccharomyces cerevisiae S288c]	852771	RMD9; YGL107C	cell differentiation;metabolic process;regulation of biological process	membrane;mitochondrion	RNA binding	1.268	0.227	0.18
P32902	37S ribosomal protein MRP4, mitochondrial	856384	MRP4; YHL004	cell organization and biogenesis;metabolic process	mitochondrion;ribosome	structural molecule activity	1.154	0.212	0.18

	[OS=Saccharomyces cerevisiae S288c]		W						
P08417	fumarate hydratase, mitochondrial [OS=Saccharomyces cerevisiae S288c]	855866	FUM1; YPL262W	cell organization and biogenesis;metabolic process	cytoplasm;cytosol;mitochondrion;organelle lumen	catalytic activity	13.874	2.562	0.18
P38523	GrpE protein homolog, mitochondrial [OS=Saccharomyces cerevisiae S288c]	854407	MGE1; YOR232W	metabolic process;regulation of biological process;transport	membrane;mitochondrion;organelle lumen	enzyme regulator activity;nucleotide binding;protein binding	2.36	0.438	0.19
P32787	Mitochondrial genome maintenance protein MGM101 [OS=Saccharomyces cerevisiae S288c]	853609	MGM101; YJR144W	cell organization and biogenesis;metabolic process;response to stimulus	chromosome;mitochondrion	DNA binding	4.456	0.833	0.19
P43567	alanine--glyoxylate aminotransferase 1 [OS=Saccharomyces cerevisiae S288c]	850514	AGX1; YFL030W	metabolic process	cytosol;mitochondrion	catalytic activity	2.481	0.468	0.19
P10507	mitochondrial-processing peptidase subunit beta [OS=Saccharomyces cerevisiae S288c]	850860	MAS1; YLR163C	metabolic process;transport	mitochondrion;organelle lumen	catalytic activity;metal ion binding;protein binding	2.481	0.468	0.19
P47052	succinate dehydrogenase [ubiquinone] flavoprotein subunit 2, mitochondrial [OS=Saccharomyces cerevisiae S288c]	853405	YJL045W; YJL045W	metabolic process;transport	membrane;mitochondrion	catalytic activity;nucleotide binding	5.884	1.11	0.19
Q05931	Heat shock protein SSQ1, mitochondrial [OS=Saccharomyces cerevisiae S288c]	851084	SSQ1; YLR369W	cell organization and biogenesis;metabolic process	mitochondrion;organelle lumen	nucleotide binding;protein binding	0.995	0.189	0.19
P06208-1	2-isopropylmalate synthase [OS=Saccharomyces cerevisiae S288c]	855619	LEU4; YNL104C	metabolic process	cytoplasm;mitochondrion	catalytic activity;protein binding	9	1.728	0.19
P53881	54S ribosomal protein L22, mitochondrial [OS=Saccharomyces cerevisiae S288c]	855544	MRPL22; YNL177C	cell organization and biogenesis;metabolic process	mitochondrion;ribosome	RNA binding;structural molecule activity	1.994	0.389	0.20
P25719	Peptidyl-prolyl cis-trans isomerase C, mitochondrial [OS=Saccharomyces cerevisiae S288c]	854897	CPR3; YML078W	cell death;metabolic process	mitochondrion;organelle lumen	catalytic activity	2.981	0.585	0.20
P39525	3-oxoacyl-[acyl-carrier-protein] synthase homolog [OS=Saccharomyces cerevisiae S288c]	856790	CEM1; YER061C	metabolic process	mitochondrion	catalytic activity	1.848	0.369	0.20
P25372	Thioredoxin-3, mitochondrial [OS=Saccharomyces cerevisiae S288c]	850444	TRX3; YCR083W	cellular homeostasis;metabolic process;regulation of biological process;response to stimulus;transport	cytoplasm;mitochondrion	catalytic activity	2.511	0.52	0.21
P38122	3-methyl-2-oxobutanoate hydroxymethyltransferase [OS=Saccharomyces cerevisiae S288c]	852474	ECM31; YBR176W	metabolic process	mitochondrion	catalytic activity	0.585	0.122	0.21
P36520	54S ribosomal protein L10, mitochondrial [OS=Saccharomyces cerevisiae S288c]	855436	MRPL10; YNL284C	cell organization and biogenesis;metabolic process	mitochondrion;ribosome	structural molecule activity	0.551	0.116	0.21
P36013	NAD-dependent malic enzyme, mitochondrial [OS=Saccharomyces cerevisiae S288c]	853839	MAE1; YKL029C	metabolic process	mitochondrion;organelle lumen	catalytic activity;metal ion binding;nucleotide binding	2.35	0.496	0.21
P36528	54S ribosomal protein L17, mitochondrial [OS=Saccharomyces cerevisiae S288c]	855469	MRPL17; YNL252C	cell organization and biogenesis;metabolic process	mitochondrion;ribosome	structural molecule activity	0.52	0.11	0.21
Q07500	External NADH-ubiquinone oxidoreductase 2, mitochondrial [OS=Saccharomyces cerevisiae S288c]	851474	NDE2; YDL085W	metabolic process	mitochondrion	catalytic activity;nucleotide binding	12.594	2.687	0.21
P40513	Mitochondrial acidic protein MAM33 [OS=Saccharomyces cerevisiae S288c]	854740	MAM33; YIL070C	metabolic process;regulation of biological process	mitochondrion;organelle lumen	translation regulator activity	3.642	0.778	0.21
P32839	Mitochondrial chaperone BCS1 [OS=Saccharomyces cerevisiae S288c]	851981	BCS1; YDR375C	cell organization and biogenesis;metabolic process;response to stimulus;transport	cytosol;membrane;mitochondrion	catalytic activity;nucleotide binding;transporter activity	0.425	0.093	0.22
P43635	Citrate synthase 3, mitochondrial [OS=Saccharomyces cerevisiae S288c]	856107	CIT3; YPR001W	metabolic process	mitochondrion	catalytic activity	6.11	1.346	0.22
P32891	D-lactate dehydrogenase [cytochrome] 1, mitochondrial [OS=Saccharomyces cerevisiae S288c]	851380	DLD1; YDL174C	metabolic process;transport	membrane;mitochondrion	catalytic activity;nucleotide binding	13.03	2.875	0.22
P28817	3-hydroxyisobutyryl-CoA hydrolase, mitochondrial [OS=Saccharomyces cerevisiae S288c]	851606	EHD3; YDR036C	metabolic process	mitochondrion	catalytic activity	1.212	0.269	0.22

Q96VH5	MICOS complex subunit Mic10 [OS=Saccharomyces cerevisiae S288c]	850300	MOS1; MIC10; YCL057C-A	cell organization and biogenesis	membrane;mitochondrion		4.179	0.931	0.22
Q03976	37S ribosomal protein S24, mitochondrial [OS=Saccharomyces cerevisiae S288c]	851755	RSM24; YDR175C	cell organization and biogenesis;metabolic process	mitochondrion;ribosome	structural molecule activity	0.905	0.202	0.22
P07246	Alcohol dehydrogenase 3, mitochondrial [OS=Saccharomyces cerevisiae S288c]	855107	ADH3; YMR083W	metabolic process	mitochondrion;organelle lumen	catalytic activity;metal ion binding	10.159	2.34	0.23
P37298	Succinate dehydrogenase [ubiquinone] cytochrome b small subunit, mitochondrial [OS=Saccharomyces cerevisiae S288c]	851758	SDH4; YDR178W	metabolic process;transport	membrane;mitochondrion	catalytic activity;metal ion binding	4.995	1.154	0.23
P16387	Pyruvate dehydrogenase E1 component subunit alpha, mitochondrial [OS=Saccharomyces cerevisiae S288c]	856925	PDA1; YER178W	cell growth;metabolic process	mitochondrion;organelle lumen	catalytic activity	25.264	5.898	0.23
P34227	Mitochondrial peroxiredoxin PRX1 [OS=Saccharomyces cerevisiae S288c]	852215	PRX1; YBL064C	cellular homeostasis;metabolic process;regulation of biological process;response to stimulus	mitochondrion	antioxidant activity;catalytic activity	6.848	1.637	0.24
P52893	probable alanine aminotransferase, mitochondrial [OS=Saccharomyces cerevisiae S288c]	850778	ALT1; YLR089C	metabolic process	mitochondrion;organelle lumen	catalytic activity	1.438	0.346	0.24
P16451	Pyruvate dehydrogenase complex protein X component, mitochondrial [OS=Saccharomyces cerevisiae S288c]	853107	PDX1; YGR193C	metabolic process	mitochondrion;organelle lumen	catalytic activity;structural molecule activity	4.055	0.978	0.24
P37292	Serine hydroxymethyltransferase, mitochondrial [OS=Saccharomyces cerevisiae S288c]	852565	SHM1; YBR263W	metabolic process	mitochondrion	catalytic activity	12.219	3.037	0.25
P32340	Rotenone-insensitive NADH-ubiquinone oxidoreductase, mitochondrial [OS=Saccharomyces cerevisiae S288c]	854919	NDI1; YML120C	metabolic process;regulation of biological process	membrane;mitochondrion;organelle lumen	catalytic activity;nucleotide binding	22.95	5.723	0.25
P32860	NifU-like protein, mitochondrial [OS=Saccharomyces cerevisiae S288c]	853826	NFU1; YKL040C	cell organization and biogenesis;cellular homeostasis;metabolic process	mitochondrion;organelle lumen	metal ion binding	5.494	1.371	0.25
P00427	Cytochrome c oxidase subunit 6, mitochondrial [OS=Saccharomyces cerevisiae S288c]	856448	COX6; YHR051W	metabolic process;transport	membrane;mitochondrion	catalytic activity;metal ion binding;transporter activity	4.623	1.154	0.25
Q02950	37S ribosomal protein MRP51, mitochondrial [OS=Saccharomyces cerevisiae S288c]	855985	MRP51; YPL118W	cell organization and biogenesis;metabolic process	mitochondrion;ribosome	structural molecule activity	1.154	0.292	0.25
P53732	37S ribosomal protein S12, mitochondrial [OS=Saccharomyces cerevisiae S288c]	855772	MRPS12; YNR036C	cell organization and biogenesis;metabolic process	mitochondrion;ribosome	structural molecule activity	1.154	0.292	0.25
P19516	Cytochrome c oxidase assembly protein COX11, mitochondrial [OS=Saccharomyces cerevisiae S288c]	855971	COX11; YPL132W	cell organization and biogenesis;metabolic process	membrane;mitochondrion;ribosome	metal ion binding	1.069	0.274	0.26
P36521	54S ribosomal protein L11, mitochondrial [OS=Saccharomyces cerevisiae S288c]	851325	MRPL11; YDL202W	cell organization and biogenesis;metabolic process	mitochondrion;ribosome	structural molecule activity	1.069	0.274	0.26
P40502	Altered inheritance of mitochondria protein 19, mitochondrial [OS=Saccharomyces cerevisiae S288c]	854722	AIM19; YIL087C		membrane;mitochondrion		0.995	0.259	0.26
P38300	Inner membrane mitoribosome receptor MBA1, mitochondrial [OS=Saccharomyces cerevisiae S288c]	852483	MBA1; YBR185C	cell organization and biogenesis;regulation of biological process	membrane;mitochondrion		0.995	0.259	0.26
Q08968	UPF0061 protein FMP40 [OS=Saccharomyces cerevisiae S288c]	855879	FMP40; YPL222W	mitochondrion	protein binding	2.495	0.65	0.26	
P10662	37S ribosomal protein MRP1, mitochondrial [OS=Saccharomyces cerevisiae S288c]	851948	MRP1; YDR347W	cell organization and biogenesis;metabolic process;response to stimulus	mitochondrion;ribosome	antioxidant activity;catalytic activity;metal ion binding;structural molecule activity	2.981	0.778	0.26
P23833	Protein SCO1, mitochondrial [OS=Saccharomyces cerevisiae S288c]	852325	SCO1; YBR037C	cell organization and biogenesis;cellular homeostasis;response to	membrane;mitochondrion	antioxidant activity;catalytic activity;metal ion	1.783	0.468	0.26

				stimulus;transport		binding			
P51998	54S ribosomal protein yml6, mitochondrial [OS=Saccharomyces cerevisiae S288c]	854983	YML6; YML025C	cell organization and biogenesis;metabolic process	mitochondrion;ribosome	RNA binding;structural molecule activity	0.931	0.245	0.26
Q12029	Probable mitochondrial transport protein fsf1 [OS=Saccharomyces cerevisiae S288c]	854445	FSF1; YOR271C	transport	membrane;mitochondrion	transporter activity	2.36	0.624	0.26
P38797	Protein phosphatase 2C homolog 7, mitochondrial [OS=Saccharomyces cerevisiae S288c]	856475	PTC7; YHR076W	metabolic process	mitochondrion	catalytic activity;metal ion binding;protein binding	0.874	0.233	0.27
P32191	Glycerol-3-phosphate dehydrogenase, mitochondrial [OS=Saccharomyces cerevisiae S288c]	854651	GUT2; YIL155C	metabolic process	membrane;mitochondrion	catalytic activity	9.502	2.574	0.27
P32453	Protein ATP11, mitochondrial [OS=Saccharomyces cerevisiae S288c]	855401	ATP11; YNL315C	cell organization and biogenesis	mitochondrion	protein binding	1.512	0.413	0.27
P46367	Potassium-activated aldehyde dehydrogenase, mitochondrial [OS=Saccharomyces cerevisiae S288c]	854556	ALD4; YOR374W	metabolic process	mitochondrion;organelle lumen	catalytic activity	58.078	15.927	0.27
P35180	mitochondrial import receptor subunit Tom20 [OS=Saccharomyces cerevisiae S288c]	852973	TOM20; YGR082W	cell organization and biogenesis;transport	membrane;mitochondrion	protein binding;transporter activity	0.701	0.194	0.28
P40053	Altered inheritance of mitochondria protein 9, mitochondrial [OS=Saccharomyces cerevisiae S288c]	856813	AIM9; YER080W		mitochondrion		4.289	1.19	0.28
P33303	Succinate/fumarate mitochondrial transporter [OS=Saccharomyces cerevisiae S288c]	853558	SFC1; YJR095W	metabolic process;transport	membrane;mitochondrion	structural molecule activity;transporter activity	17.957	4.995	0.28
Q04728	Arginine biosynthesis bifunctional protein ArgJ, mitochondrial [OS=Saccharomyces cerevisiae S288c]	855084	ARG7; YMR062C	metabolic process	mitochondrion;organelle lumen	catalytic activity	1.154	0.322	0.28
P00445	Superoxide dismutase [Cu-Zn] [OS=Saccharomyces cerevisiae S288c]	853568	SOD1; YJR104C	cell organization and biogenesis;cellular homeostasis;metabolic process;regulation of biological process;response to stimulus	cytoplasm;cytosol;mitochondrion;nucleus	antioxidant activity;catalytic activity;metal ion binding;protein binding	1.848	0.52	0.28
P07275	Delta-1-pyrroline-5-carboxylate dehydrogenase, mitochondrial [OS=Saccharomyces cerevisiae S288c]	856432	PUT2; YHR037W	metabolic process	membrane;mitochondrion;organelle lumen	catalytic activity	6.565	1.848	0.28
P42940	Probable electron transfer flavoprotein subunit beta [OS=Saccharomyces cerevisiae S288c]	853121	CIR1; YGR207C	metabolic process;transport	mitochondrion;organelle lumen		7.66	2.162	0.28
P32939	GTP-binding protein ypt7 [OS=Saccharomyces cerevisiae S288c]	855012	YPT7; YML001W	cell communication;cell organization and biogenesis;metabolic process;regulation of biological process;response to stimulus;transport	cytosol;endosome;membrane;mitochondrion;vacuole	catalytic activity;nucleotide binding;protein binding	0.585	0.166	0.28
Q06630	Mitochondrial homologous recombination protein 1 [OS=Saccharomyces cerevisiae S288c]	851890	MHR1; YDR296W	cell organization and biogenesis;metabolic process;regulation of biological process;response to stimulus	mitochondrion;nucleus	catalytic activity;DNA binding;structural molecule activity	0.585	0.166	0.28
Q12480	Probable electron transfer flavoprotein subunit alpha, mitochondrial [OS=Saccharomyces cerevisiae S288c]	856112	AIM45; YPR004C	metabolic process;transport	mitochondrion;organelle lumen	catalytic activity;nucleotide binding	3.467	0.995	0.29
P41735	5-demethoxyubiquinone hydroxylase, mitochondrial [OS=Saccharomyces cerevisiae S288c]	854292	CAT5; YOR125C	metabolic process	membrane;mitochondrion	catalytic activity;metal ion binding	0.54	0.155	0.29
P07806	Valine--tRNA ligase, mitochondrial [OS=Saccharomyces cerevisiae S288c]	852986	VAS1; YGR094W	metabolic process;regulation of biological process	cytoplasm;cytosol;mitochondrion	catalytic activity;nucleotide binding	0.722	0.212	0.29
P36775	Lon protease homolog, mitochondrial [OS=Saccharomyces cerevisiae S288c]	852259	PIM1; YBL022C	cell organization and biogenesis;metabolic process;regulation of biological process;response to stimulus	cytoplasm;mitochondrion;organelle lumen	catalytic activity;DNA binding;nucleotide binding;RNA binding	1.222	0.359	0.29
P07256	Cytochrome b-c1 complex subunit 1, mitochondrial [OS=Saccharomyces cerevisiae S288c]	852235	COR1; YBL045C	metabolic process;transport	membrane;mitochondrion	catalytic activity;metal ion binding;protein binding;transporter activity	18.783	5.529	0.29
P38910	10 kDa heat shock protein, mitochondrial [OS=Saccharomyces cerevisiae	854185	HSP10; YOR020C	cell organization and biogenesis;metabolic process;response to stimulus;transport	cytoplasm;mitochondrion;organelle lumen	metal ion binding;nucleotide binding;protein	4.623	1.371	0.30

	S288c]					binding			
Q12166	2-isopropylmalate synthase 2, mitochondrial [OS=Saccharomyces cerevisiae S288c]	854275	LEU9; YOR108W	metabolic process	mitochondrion	catalytic activity;protein binding	3.739	1.11	0.30
P16547	Mitochondrial outer membrane protein OM45 [OS=Saccharomyces cerevisiae S288c]	854670	OM45; YIL136W		membrane;mitochondrion		274.423	82.176	0.30
P19262	Dihydrolipoyllysine-residue succinyltransferase component of 2-oxoglutarate dehydrogenase complex, mitochondrial [OS=Saccharomyces cerevisiae S288c]	851726	KGD2; YDR148C	cell organization and biogenesis;metabolic process	mitochondrion	catalytic activity;protein binding	5.7	1.721	0.30
P36525	54S ribosomal protein L24, mitochondrial [OS=Saccharomyces cerevisiae S288c]	855231	MRPL24; YMR193W	cell organization and biogenesis;metabolic process	mitochondrion;ribosome	RNA binding;structural molecule activity	1.929	0.585	0.30
P22354	54S ribosomal protein L20, mitochondrial [OS=Saccharomyces cerevisiae S288c]	853960	MRPL20; YKR085C	cell organization and biogenesis;metabolic process	mitochondrion;ribosome	structural molecule activity	1.276	0.389	0.30
Q12428	Probable 2-methylcitrate dehydratase [OS=Saccharomyces cerevisiae S288c]	856108	PDH1; YPR002W	metabolic process	cytoplasm;membrane;mitochondrion	catalytic activity	19.535	5.978	0.31
P40047	Aldehyde dehydrogenase 5, mitochondrial [OS=Saccharomyces cerevisiae S288c]	856804	ALD5; YER073W	metabolic process	mitochondrion;organelle lumen	catalytic activity	3.146	0.968	0.31
P38169	Kynurenine 3-monooxygenase [OS=Saccharomyces cerevisiae S288c]	852179	BNA4; YBL098W	metabolic process	membrane;mitochondrion	catalytic activity;nucleotide binding	0.269	0.083	0.31
P48015	Aminomethyltransferase, mitochondrial [OS=Saccharomyces cerevisiae S288c]	851582	GCV1; YDR019C	metabolic process	mitochondrion	catalytic activity;protein binding	11.217	3.489	0.31
P41921	glutathione reductase [OS=Saccharomyces cerevisiae S288c]	856014	GLR1; YPL091W	cellular homeostasis;metabolic process;regulation of biological process;response to stimulus	cytoplasm;cytosol;mitochondrion;nucleus	antioxidant activity;catalytic activity;nucleotide binding	0.241	0.075	0.31
Q06567	ABC1 family protein MCP2 [OS=Saccharomyces cerevisiae S288c]	850955	YLR253W; MCP2; YLR253W	cell organization and biogenesis	membrane;mitochondrion		0.218	0.068	0.31
P35191	DnaJ homolog 1, mitochondrial [OS=Saccharomyces cerevisiae S288c]	850530	MDJ1; YFL016C	cell organization and biogenesis;metabolic process;regulation of biological process;response to stimulus	mitochondrion;organelle lumen	enzyme regulator activity;metal ion binding;nucleotide binding;protein binding	4.722	1.477	0.31
P25374	Cysteine desulfurase, mitochondrial [OS=Saccharomyces cerevisiae S288c]	850343	NFS1; YCL017C	cell organization and biogenesis;cellular homeostasis;metabolic process	mitochondrion;nucleus	catalytic activity;metal ion binding;protein binding	2.594	0.817	0.31
P09624	Dihydrolipoyl dehydrogenase, mitochondrial [OS=Saccharomyces cerevisiae S288c]	850527	LPD1; YFL018C	cellular homeostasis;metabolic process;regulation of biological process	mitochondrion;organelle lumen	catalytic activity;nucleotide binding	30.623	9.701	0.32
P38172	MIOREX complex component 3 [OS=Saccharomyces cerevisiae S288c]	852181	YBL095W; MRX3; YBL095W		membrane;mitochondrion		0.968	0.311	0.32
P17558	37S ribosomal protein PET123, mitochondrial [OS=Saccharomyces cerevisiae S288c]	854329	PET123; YOR158W	cell organization and biogenesis;metabolic process	mitochondrion;ribosome	structural molecule activity	1.448	0.468	0.32
P27929	37S ribosomal protein NAM9, mitochondrial [OS=Saccharomyces cerevisiae S288c]	855585	NAM9; YNL137C	cell organization and biogenesis;metabolic process;regulation of biological process	mitochondrion;ribosome	RNA binding;structural molecule activity	0.931	0.301	0.32
P17505	Malate dehydrogenase, mitochondrial [OS=Saccharomyces cerevisiae S288c]	853777	MDH1; YKL085W	metabolic process	mitochondrion;organelle lumen	catalytic activity;protein binding;RNA binding	99	32.405	0.33
Q05892	MIOREX complex component 2 [OS=Saccharomyces cerevisiae S288c]	850997	YLR290C; COQ11; YLR290C	metabolic process	mitochondrion	catalytic activity	3.217	1.054	0.33
P21771	37S ribosomal protein S28, mitochondrial [OS=Saccharomyces cerevisiae S288c]	851937	MRPS28; YDR337W	cell organization and biogenesis;metabolic process	mitochondrion;ribosome	RNA binding;structural molecule activity	0.833	0.274	0.33
P30902	ATP synthase subunit d, mitochondrial [OS=Saccharomyces cerevisiae	853853	ATP7; YKL016C	metabolic process;transport	membrane;mitochondrion	catalytic activity;transporter activity	5.105	1.683	0.33

	S288c]								
P43617	Uncharacterized mitochondrial carrier YFR045W [OS=Saccharomyces cerevisiae S288c]	850606	YFR045W; YFR045W	metabolic process;transport	membrane;mitochondrion	structural molecule activity;transporter activity	0.778	0.259	0.33
P53736	Uncharacterized protein YNR040W [OS=Saccharomyces cerevisiae S288c]	855776	YNR040W; YNR040W	mitochondrion		1.154	0.389	0.34	
P53326	Uncharacterized protein YGR266W [OS=Saccharomyces cerevisiae S288c]	853182	YGR266W; YGR266W	membrane;mitochondrion		1.081	0.369	0.34	
P0CS90	Heat shock protein SSC1, mitochondrial [OS=Saccharomyces cerevisiae S288c]	853503	SSC1; YJR045C	cell organization and biogenesis;metabolic process;regulation of biological process;transport	membrane;mitochondrion;nucleus;organelle lumen	catalytic activity;enzyme regulator activity;nucleotide binding;protein binding	51.75	17.957	0.35
P36046	Mitochondrial intermembrane space import and assembly protein 40 [OS=Saccharomyces cerevisiae S288c]	853639	MIA40; YKL195W	cellular component movement;metabolic process;response to stimulus;transport	membrane;mitochondrion	catalytic activity;protein binding;transporter activity	2.3	0.817	0.36
Q06485	Autophagy-related protein 33 [OS=Saccharomyces cerevisiae S288c]	851070	ATG33; YLR356W	cell communication;metabolic process;response to stimulus	membrane;mitochondrion		2.162	0.778	0.36
P21306	ATP synthase subunit epsilon, mitochondrial [OS=Saccharomyces cerevisiae S288c]	855857	ATP15; YPL271W	metabolic process;transport	membrane;mitochondrion	catalytic activity;transporter activity	2.162	0.778	0.36
P21801	Succinate dehydrogenase [ubiquinone] iron-sulfur subunit, mitochondrial [OS=Saccharomyces cerevisiae S288c]	850685	SDH2; YLL041C	metabolic process;transport	membrane;mitochondrion	catalytic activity;metal ion binding	32.839	12.111	0.37
Q3E824	Uncharacterized protein YOR020W-A [OS=Saccharomyces cerevisiae S288c]	1466480	YOR020W-A; YOR020W-A	metabolic process;transport	membrane;mitochondrion		5.813	2.162	0.37
P32473	Pyruvate dehydrogenase E1 component subunit beta, mitochondrial [OS=Saccharomyces cerevisiae S288c]	852522	PDB1; YBR221C	metabolic process	mitochondrion;organelle lumen	catalytic activity	10.288	3.833	0.37
P46681	D-lactate dehydrogenase [cytochrome] 2, mitochondrial [OS=Saccharomyces cerevisiae S288c]	851376	DLD2; YDL178W	metabolic process	mitochondrion;organelle lumen	catalytic activity;nucleotide binding;protein binding	1.829	0.682	0.37
P53266	Cytochrome oxidase assembly protein SHY1 [OS=Saccharomyces cerevisiae S288c]	853009	SHY1; YGR112W	cell organization and biogenesis	membrane;mitochondrion	protein binding	1.783	0.668	0.37
P00044	Cytochrome c iso-1 [OS=Saccharomyces cerevisiae S288c]	853507	CYC1; YJR048W	metabolic process;transport	mitochondrion	metal ion binding;protein binding	12.335	4.623	0.37
Q12374	Nuclear control of ATPase protein 2 [OS=Saccharomyces cerevisiae S288c]	856278	NCA2; YPR155C	metabolic process	membrane;mitochondrion		0.565	0.212	0.38
P20967	2-oxoglutarate dehydrogenase, mitochondrial [OS=Saccharomyces cerevisiae S288c]	854681	KGD1; YIL125W	metabolic process	cytosol;mitochondrion;organelle lumen	catalytic activity	13.785	5.201	0.38
P28241	Isocitrate dehydrogenase [NAD] subunit 2, mitochondrial [OS=Saccharomyces cerevisiae S288c]	854303	IDH2; YOR136W	metabolic process	mitochondrion;organelle lumen	catalytic activity;metal ion binding;nucleotide binding;protein binding;RNA binding	6.197	2.34	0.38
P09440	C-1-tetrahydrofolate synthase, mitochondrial [OS=Saccharomyces cerevisiae S288c]	852378	MIS1; YBR084W	metabolic process	cytosol;mitochondrion	catalytic activity;nucleotide binding	1.668	0.633	0.38
P40495	Homoisocitrate dehydrogenase, mitochondrial [OS=Saccharomyces cerevisiae S288c]	854714	LYS12; YIL094C	metabolic process	mitochondrion	catalytic activity;metal ion binding;nucleotide binding	6.499	2.481	0.38
P07143	Cytochrome c1, heme protein, mitochondrial [OS=Saccharomyces cerevisiae S288c]	854231	CYT1; YOR065W	metabolic process;transport	cytosol;membrane;mitochondrion	metal ion binding	19.309	7.377	0.38
P23641	mitochondrial phosphate carrier protein [OS=Saccharomyces cerevisiae S288c]	853540	MIR1; YJR077C	metabolic process;transport	membrane;mitochondrion	protein binding;structural molecule activity;transporter activity	26.826	10.365	0.39
P16622	Ferrochelatase, mitochondrial [OS=Saccharomyces cerevisiae S288c]	854347	HEM15; YOR176W	metabolic process	membrane;mitochondrion	catalytic activity	5.579	2.162	0.39

P07251	ATP synthase subunit alpha, mitochondrial [OS=Saccharomyces cerevisiae S288c]	852177	ATP1; YBL099W	metabolic process;transport	cytosol;membrane;mitochondrion	catalytic activity;nucleotide binding;transporter activity	34.938	13.678	0.39
P50088	Stationary phase gene 1 protein [OS=Saccharomyces cerevisiae S288c]	853151	SPG1; YGR236C	endoplasmic reticulum;membrane;mitochondrion		250.189	99	0.40	
P17695	Glutaredoxin-2, mitochondrial [OS=Saccharomyces cerevisiae S288c]	852124	GRX2; YDR513W	cellular homeostasis;metabolic process;regulation of biological process;response to stimulus;transport	cytoplasm;cytosol;mitochondrion;nucleus	antioxidant activity;catalytic activity	1.31	0.52	0.40
P53889	uncharacterized mitochondrial hydrolase FMP41 [OS=Saccharomyces cerevisiae S288c]	855553	FMP41; YNL168C	metabolic process	mitochondrion	catalytic activity;metal ion binding	1.254	0.501	0.40
P50945	MICOS complex subunit MIC27 [OS=Saccharomyces cerevisiae S288c]	855623	AIM37; MIC27; YNL100W	cell organization and biogenesis	membrane;mitochondrion		1.254	0.501	0.40
P32317	Protein AFG1 [OS=Saccharomyces cerevisiae S288c]	856658	AFG1; YEL052W	cell organization and biogenesis;metabolic process;response to stimulus;transport	membrane;mitochondrion	catalytic activity;nucleotide binding	1.154	0.468	0.41
P23644	Mitochondrial import receptor subunit TOM40 [OS=Saccharomyces cerevisiae S288c]	855243	TOM40; YMR203W	transport	cytosol;membrane;mitochondrion	protein binding;transporter activity	1.154	0.468	0.41
P32331	Carrier protein YMC1, mitochondrial [OS=Saccharomyces cerevisiae S288c]	856171	YMC1; YPR058W	metabolic process;transport	membrane;mitochondrion;vacuole	structural molecule activity;transporter activity	2.384	0.968	0.41
P32795	Mitochondrial inner membrane i-AAA protease supercomplex subunit YME1 [OS=Saccharomyces cerevisiae S288c]	856135	YME1; YPR024W	metabolic process;response to stimulus;transport	membrane;mitochondrion	catalytic activity;metal ion binding;nucleotide binding;protein binding	1.938	0.8	0.41
P33759	37S ribosomal protein S5, mitochondrial [OS=Saccharomyces cerevisiae S288c]	852553	MRPS5; YBR251W	cell organization and biogenesis;metabolic process	mitochondrion;ribosome	RNA binding;structural molecule activity	0.995	0.413	0.42
P37293	N-terminal acetyltransferase 2 [OS=Saccharomyces cerevisiae S288c]	853050	NAT2; YGR147C	metabolic process	cytoplasm;mitochondrion	catalytic activity	0.931	0.389	0.42
Q03246	37S ribosomal protein S17, mitochondrial [OS=Saccharomyces cerevisiae S288c]	855226	MRPS17; YMR188C	cell differentiation;cell organization and biogenesis;metabolic process	mitochondrion;ribosome	RNA binding;structural molecule activity	0.931	0.389	0.42
Q07914	mitochondrial import inner membrane translocase subunit TIM14 [OS=Saccharomyces cerevisiae S288c]	850694	PAM18; YLR008C	regulation of biological process;transport	membrane;mitochondrion	enzyme regulator activity;protein binding;transporter activity	0.778	0.334	0.43
P39515	mitochondrial import inner membrane translocase subunit Tim17 [OS=Saccharomyces cerevisiae S288c]	853298	TIM17; YJL143W	cell organization and biogenesis;transport	membrane;mitochondrion	protein binding;transporter activity	0.778	0.334	0.43
P49367	Homoaconitase, mitochondrial [OS=Saccharomyces cerevisiae S288c]	851820	LYS4; YDR234W	metabolic process	mitochondrion	catalytic activity;metal ion binding;protein binding	1.276	0.551	0.43
P40341	Mitochondrial respiratory chain complexes assembly protein YTA12 [OS=Saccharomyces cerevisiae S288c]	855114	YTA12; YMR089C	cell organization and biogenesis;metabolic process;response to stimulus;transport	cytoplasm;membrane;mitochondrion	catalytic activity;metal ion binding;nucleotide binding;protein binding	1.795	0.778	0.43
Q04013	Citrate/oxoglutarate carrier protein [OS=Saccharomyces cerevisiae S288c]	855282	YHM2; YMR241W	cell organization and biogenesis;metabolic process;transport	membrane;mitochondrion	DNA binding;structural molecule activity;transporter activity	3.467	1.512	0.44
P36112	MICOS complex subunit Mic60 [OS=Saccharomyces cerevisiae S288c]	853886	FCJ1; MIC60; YKR016W	cell organization and biogenesis;transport	membrane;mitochondrion		12.895	5.629	0.44
P47127	Altered inheritance of mitochondria protein 24, mitochondrial [OS=Saccharomyces cerevisiae S288c]	853543	AIM24; YJR080C	cell organization and biogenesis	mitochondrion		3.175	1.395	0.44
P40185	Protein mmf1, mitochondrial [OS=Saccharomyces cerevisiae S288c]	854760	MMF1; YIL051C	cell organization and biogenesis;metabolic process	mitochondrion;organelle lumen		9	4.012	0.45
P39952	Mitochondrial inner membrane protein OXA1 [OS=Saccharomyces cerevisiae S288c]	856898	OXA1; YER154W	cell organization and biogenesis;transport	membrane;mitochondrion	transporter activity	1.738	0.778	0.45
P42949	Mitochondrial import inner membrane translocase subunit TIM16 [OS=Saccharomyces cerevisiae S288c]	853340	PAM16; YJL104W	transport	membrane;mitochondrion	protein binding	0.52	0.233	0.45
P40098	uncharacterized mitochondrial membrane protein FMP10 [OS=Saccharomyces cerevisiae	856931	FMP10; YER182W		membrane;mitochondrion		2.36	1.069	0.45

	S288c]								
Q04172	Sensitive to high expression protein 9, mitochondrial [OS=Saccharomyces cerevisiae S288c]	852002	SHE9; YDR393W	cell organization and biogenesis	membrane;mitochondrion		0.374	0.172	0.46
P22353	54S ribosomal protein L8, mitochondrial [OS=Saccharomyces cerevisiae S288c]	853382	MRPL8; YJL063C	cell organization and biogenesis;metabolic process	mitochondrion;ribosome	structural molecule activity	0.359	0.166	0.46
P39925	Mitochondrial respiratory chain complexes assembly protein AFG3 [OS=Saccharomyces cerevisiae S288c]	856737	AFG3; YER017C	cell organization and biogenesis;metabolic process;response to stimulus;transport	membrane;mitochondrion	catalytic activity;metal ion binding;nucleotide binding;protein binding	1.228	0.569	0.46
P38884	Altered inheritance of mitochondria protein 18, mitochondrial [OS=Saccharomyces cerevisiae S288c]	856605	AIM18; YHR198C	mitochondrion	catalytic activity	0.311	0.145	0.47	
Q06493	LETM1 domain-containing protein YLH47, mitochondrial [OS=Saccharomyces cerevisiae S288c]	856243	YLH47; YPR125W	cell organization and biogenesis;transport	membrane;mitochondrion		1.043	0.487	0.47
P38071	Enoyl-[acyl-carrier protein] reductase [NADPH, B-specific], mitochondrial [OS=Saccharomyces cerevisiae S288c]	852314	ETR1; YBR026C	metabolic process	mitochondrion;organelle lumen	catalytic activity;DNA binding;metal ion binding	7.111	3.329	0.47
P32335	Protein MSS51, mitochondrial [OS=Saccharomyces cerevisiae S288c]	850900	MSS51; YLR203C	metabolic process;regulation of biological process	membrane;mitochondrion	protein binding;translation regulator activity	1.512	0.711	0.47
Q01532-1	Cysteine proteinase 1, mitochondrial [OS=Saccharomyces cerevisiae S288c]		MCY1	metabolic process;regulation of biological process;response to stimulus	cytoplasm;mitochondrion	catalytic activity;DNA binding;RNA binding	0.179	0.086	0.48
P21375	Fumarate reductase 2 [OS=Saccharomyces cerevisiae S288c]	853510	OSM1; YJR051W	metabolic process	endoplasmic reticulum;mitochondrion	catalytic activity;nucleotide binding	0.16	0.077	0.48
Q12032	Altered inheritance of mitochondria protein 41, mitochondrial [OS=Saccharomyces cerevisiae S288c]	854390	AIM41; YOR215C	mitochondrion	catalytic activity	1.61	0.778	0.48	
Q07349	MIOREX complex component 9 [OS=Saccharomyces cerevisiae S288c]	851535	YDL027C; MRX9; YDL027C		endoplasmic reticulum;membrane;mitochondrion		1.913	0.931	0.49
P40008	Protein FMP52, mitochondrial [OS=Saccharomyces cerevisiae S288c]	856721	FMP52; YER004W		endoplasmic reticulum;membrane;mitochondrion	catalytic activity	2.162	1.054	0.49
P53292	37S ribosomal protein S35, mitochondrial [OS=Saccharomyces cerevisiae S288c]	853075	MRPS35; YGR165W	cell organization and biogenesis;metabolic process	mitochondrion;ribosome	structural molecule activity	1.512	0.738	0.49
Q08822	Probable electron transfer flavoprotein-ubiquinone oxidoreductase, mitochondrial [OS=Saccharomyces cerevisiae S288c]	854538	CIR2; YOR356W	metabolic process;transport	membrane;mitochondrion	catalytic activity;metal ion binding;nucleotide binding	3.019	1.488	0.49
P19882	heat shock protein 60, mitochondrial [OS=Saccharomyces cerevisiae S288c]	850963	HSP60; YLR259C	cell organization and biogenesis;metabolic process;regulation of biological process;transport	cytoplasm;cytosol;membrane;mitochondrion;organelle lumen	catalytic activity;DNA binding;nucleotide binding;protein binding	23.816	11.743	0.49
P33421	Succinate dehydrogenase [ubiquinone] cytochrome b subunit, mitochondrial [OS=Saccharomyces cerevisiae S288c]	853716	SDH3; YKL141W	cell organization and biogenesis;metabolic process;transport	membrane;mitochondrion	catalytic activity;metal ion binding	1.276	0.638	0.50
Q02776	Mitochondrial import inner membrane translocase subunit TIM50 [OS=Saccharomyces cerevisiae S288c]	856042	TIM50; YPL063W	cell organization and biogenesis;metabolic process;transport	membrane;mitochondrion	catalytic activity;protein binding;transporter activity	7.483	3.771	0.50
P43616	Cys-Gly metallodipeptidase dug1 [OS=Saccharomyces cerevisiae S288c]	850605	DUG1; YFR044C	metabolic process	cytoplasm;mitochondrion;ribosome	catalytic activity;metal ion binding	1.154	0.585	0.51
P00950	Phosphoglycerate mutase 1 [OS=Saccharomyces cerevisiae S288c]	853705	GPM1; YKL152C	metabolic process;regulation of biological process	cytoplasm;cytosol;membrane;mitochondrion	catalytic activity	2.981	1.512	0.51
P07342	Acetolactate synthase catalytic subunit, mitochondrial [OS=Saccharomyces cerevisiae S288c]	855135	ILV2; YMR108W	metabolic process	mitochondrion	catalytic activity;metal ion binding;nucleotide binding	5.236	2.665	0.51
P50085	Prohibitin-2 [OS=Saccharomyces cerevisiae S288c]	853146	PHB2; YGR231C	cell organization and biogenesis;metabolic process;regulation of biological process	membrane;mitochondrion	protein binding	4.926	2.511	0.51

P36066	Protein MRG3-like [OS=Saccharomyces cerevisiae S288c]	853725	YKL133C; YKL133C	metabolic process	membrane;mitochondrion	protein binding	0.655	0.334	0.51
P47045	mitochondrial import inner membrane translocase subunit TIM54 [OS=Saccharomyces cerevisiae S288c]	853392	TIM54; YJL054W	cell organization and biogenesis;transport	cytosol;membrane;mitochondrion	transporter activity	0.655	0.334	0.51
P38072	Protein SCO2, mitochondrial [OS=Saccharomyces cerevisiae S288c]	852312	SCO2; YBR024W	cell organization and biogenesis;cellular homeostasis;metabolic process;transport	membrane;mitochondrion	antioxidant activity;catalytic activity;metal ion binding	1.404	0.73	0.52
P04803	Tryptophan--tRNA ligase, mitochondrial [OS=Saccharomyces cerevisiae S288c]	851861	MSW1; YDR268W	metabolic process	cytoplasm;mitochondrion;organelle lumen	catalytic activity;nucleotide binding	0.896	0.468	0.52
P07257	Cytochrome b-c1 complex subunit 2, mitochondrial [OS=Saccharomyces cerevisiae S288c]	856321	QCR2; YPR191W	metabolic process;transport	membrane;mitochondrion	catalytic activity;metal ion binding;protein binding;transporter activity	33.551	17.588	0.52
P32843	Mitochondrial escape protein 2 [OS=Saccharomyces cerevisiae S288c]	855348	YME2; YMR302C	cell organization and biogenesis;metabolic process	membrane;mitochondrion	nucleotide binding;RNA binding	3.112	1.637	0.53
P12695	Dihydrolipoyllysine-residue acetyltransferase component of pyruvate dehydrogenase complex, mitochondrial [OS=Saccharomyces cerevisiae S288c]	855653	LAT1; YNL071W	metabolic process	mitochondrion;organelle lumen	catalytic activity	7.003	3.758	0.54
P36101	tRNA threonylcarbamoyladenosine dehydratase 2 [OS=Saccharomyces cerevisiae S288c]	853841	TCD2; YKL027W; YKL027W	metabolic process	cytosol;membrane;mitochondrion	catalytic activity;nucleotide binding	0.616	0.334	0.54
Q03028	Mitochondrial 2-oxodicarboxylate carrier 1 [OS=Saccharomyces cerevisiae S288c]	855969	ODC1; YPL134C	metabolic process;transport	membrane;mitochondrion	structural molecule activity;transporter activity	7.799	4.275	0.55
P38705	Serine--tRNA ligase, mitochondrial [OS=Saccharomyces cerevisiae S288c]	856402	DIA4; YHR011W	metabolic process	cytoplasm;mitochondrion;organelle lumen	catalytic activity;nucleotide binding	0.417	0.233	0.56
P37299	cytochrome b-c1 complex subunit 10 [OS=Saccharomyces cerevisiae S288c]	856390	QCR10; YHR001W-A	metabolic process;transport	membrane;mitochondrion	catalytic activity;transporter activity	5.31	2.981	0.56
P32904	54S ribosomal protein L6, mitochondrial [OS=Saccharomyces cerevisiae S288c]	856552	MRPL6; YHR147C	cell organization and biogenesis;metabolic process	mitochondrion;ribosome	RNA binding;structural molecule activity	1.371	0.778	0.57
Q12233	ATP synthase subunit g, mitochondrial [OS=Saccharomyces cerevisiae S288c]	856131	ATP20; YPR020W	cell organization and biogenesis;metabolic process;transport	membrane;mitochondrion	catalytic activity;transporter activity	1.371	0.778	0.57
P36147	Presequence translocated-associated motor subunit pam17, mitochondrial [OS=Saccharomyces cerevisiae S288c]	853939	PAM17; YKR065C	transport	membrane;mitochondrion		1.371	0.778	0.57
P53598	Succinyl-CoA ligase [ADP-forming] subunit alpha, mitochondrial [OS=Saccharomyces cerevisiae S288c]	854310	LSC1; YOR142W	metabolic process	cytosol;mitochondrion	catalytic activity;nucleotide binding	9	5.158	0.57
P32387	54S ribosomal protein L41, mitochondrial [OS=Saccharomyces cerevisiae S288c]	852014	MRP20; YDR405W	cell organization and biogenesis;metabolic process	mitochondrion;ribosome	nucleotide binding;protein binding;RNA binding;structural molecule activity	1.254	0.719	0.57
P33311	ATP-dependent permease MDL2, mitochondrial [OS=Saccharomyces cerevisiae S288c]	855858	MDL2; YPL270W	response to stimulus;transport	membrane;mitochondrion	catalytic activity;nucleotide binding;transporter activity	1.233	0.708	0.57
P04710	ADP,ATP carrier protein 1 [OS=Saccharomyces cerevisiae S288c]	855078	AAC1; YMR056C	metabolic process;transport	cytosol;membrane;mitochondrion	structural molecule activity;transporter activity	10.288	5.952	0.58
Q12482	Mitochondrial aspartate-glutamate transporter AGC1 [OS=Saccharomyces cerevisiae S288c]	856132	AGC1; YPR021C	metabolic process;transport	membrane;mitochondrion	structural molecule activity;transporter activity	0.407	0.238	0.58
P40035	Mitochondrial phosphate carrier protein 2 [OS=Saccharomyces cerevisiae S288c]	856779	PIC2; YER053C	cellular homeostasis;metabolic process;transport	membrane;mitochondrion	structural molecule activity;transporter activity	2.793	1.637	0.59
P04840	Mitochondrial outer membrane protein porin 1 [OS=Saccharomyces cerevisiae S288c]	855669	POR1; YNL055C	cell death;cell organization and biogenesis;cellular homeostasis;regulation of biological process;transport	cytoplasm;membrane;mitochondrion	transporter activity	15.238	9	0.59

P00410	Cytochrome c oxidase subunit 2 [OS=Saccharomyces cerevisiae S288c]	854622	COX2; Q0250	metabolic process;transport	membrane;mitochondrion	catalytic activity;metal ion binding;protein binding;transporter activity	3.642	2.162	0.59
Q08179	Mitochondrial distribution and morphology protein 38 [OS=Saccharomyces cerevisiae S288c]	854130	MDM38; YOL027C	cell organization and biogenesis;cellular homeostasis;regulation of biological process;transport	membrane;mitochondrion		5.683	3.467	0.61
Q03798	Altered inheritance of mitochondria protein 36, mitochondrial [OS=Saccharomyces cerevisiae S288c]	855189	AIM36; YMR157C	membrane;mitochondrion		1.336	0.833	0.62	
Q12298	uncharacterized ABC transporter ATP-binding protein YDR061W [OS=Saccharomyces cerevisiae S288c]	851633	YDR061W; YDR061W		mitochondrion	catalytic activity;nucleotide binding	0.425	0.266	0.63
P32332	Mitochondrial oxaloacetate transport protein [OS=Saccharomyces cerevisiae S288c]	853739	OAC1; YKL120W	metabolic process;transport	membrane;mitochondrion	structural molecule activity;transporter activity	5.813	3.642	0.63
P38152	Tricarboxylate transport protein [OS=Saccharomyces cerevisiae S288c]	852594	CTP1; YBR291C	metabolic process;transport	membrane;mitochondrion	structural molecule activity;transporter activity	0.389	0.245	0.63
Q06143	mitochondrial dicarboxylate transporter [OS=Saccharomyces cerevisiae S288c]	851063	DIC1; YLR348C	metabolic process;transport	membrane;mitochondrion	structural molecule activity;transporter activity	1.154	0.73	0.63
Q05648	MIOREX complex component 10 [OS=Saccharomyces cerevisiae S288c]	851876	YDR282C; MRX10; YDR282C		membrane;mitochondrion		0.35	0.222	0.63
P38702	mitochondrial carrier protein LEU5 [OS=Saccharomyces cerevisiae S288c]	856391	LEU5; YHR002W	metabolic process;transport	membrane;mitochondrion	structural molecule activity;transporter activity	0.334	0.212	0.63
P40012	protoporphyrinogen oxidase [OS=Saccharomyces cerevisiae S288c]	856733	HEM14; YER014W	metabolic process	membrane;mitochondrion	catalytic activity	1.482	0.947	0.64
P32266	Dynamin-like GTPase MGM1, mitochondrial [OS=Saccharomyces cerevisiae S288c]	854386	MGM1; YOR211C	cell organization and biogenesis	membrane;mitochondrion	catalytic activity;nucleotide binding;protein binding	0.28	0.179	0.64
P38969	Pentamidine resistance factor, mitochondrial [OS=Saccharomyces cerevisiae S288c]	854440	PNT1; YOR266W	cell organization and biogenesis;response to stimulus	membrane;mitochondrion		0.259	0.166	0.64
P07236	Threonine--tRNA ligase, mitochondrial [OS=Saccharomyces cerevisiae S288c]	853640	MST1; YKL194C	metabolic process	cytoplasm;mitochondrion;organelle lumen	catalytic activity;nucleotide binding;RNA binding	0.233	0.15	0.64
P09457	ATP synthase subunit 5, mitochondrial [OS=Saccharomyces cerevisiae S288c]	851892	ATP5; YDR298C	metabolic process;transport	membrane;mitochondrion	catalytic activity;transporter activity	9	5.813	0.65
P54783	D-arabinono-1,4-lactone oxidase [OS=Saccharomyces cerevisiae S288c]	854888	ALO1; YML086C	metabolic process;response to stimulus	membrane;mitochondrion	catalytic activity;nucleotide binding	3.299	2.162	0.66
Q08223	Altered inheritance of mitochondria protein 39, mitochondrial [OS=Saccharomyces cerevisiae S288c]	854103	AIM39; YOL053W	cytosol;membrane;mitochondrion		0.711	0.468	0.66	
P07213	Mitochondrial import receptor subunit TOM70 [OS=Saccharomyces cerevisiae S288c]	855602	TOM70; YNL121C	cell organization and biogenesis;transport	membrane;mitochondrion	protein binding;transporter activity	1.728	1.154	0.67
P53311	Mitochondrial pyruvate carrier 3 [OS=Saccharomyces cerevisiae S288c]	853158	FMP43; MPC3; YGR243W	transport	membrane;mitochondrion	transporter activity	1.154	0.778	0.67
P00359	Glyceraldehyde-3-phosphate dehydrogenase 3 [OS=Saccharomyces cerevisiae S288c]	853106	TDH3; YGR192C	cell death;metabolic process	cytoplasm;cytosol;membrane;mitochondrion;nucleus	catalytic activity;nucleotide binding;RNA binding	3.962	2.675	0.68
P38325	Mitochondrial outer membrane protein OM14 [OS=Saccharomyces cerevisiae S288c]	852531	OM14; YBR230C	transport	membrane;mitochondrion		9	6.197	0.69
P54003	Protein SUR7 [OS=Saccharomyces cerevisiae S288c]	854953	SUR7; YML052W	cell differentiation;transport	membrane;mitochondrion		0.848	0.585	0.69
Q99297	Mitochondrial 2-oxodicarboxylate carrier 2 [OS=Saccharomyces cerevisiae S288c]	854397	ODC2; YOR222W	metabolic process;transport	membrane;mitochondrion	structural molecule activity;transporter activity	0.778	0.54	0.69

P53220	Mitochondrial import inner membrane translocase subunit TIM21 [OS=Saccharomyces cerevisiae S288c]	852921	TIM21; YGR033C	cell organization and biogenesis;transport	membrane;mitochondrion	protein binding	0.719	0.501	0.70
Q12251	Uncharacterized mitochondrial carrier YPR011C [OS=Saccharomyces cerevisiae S288c]	856121	YPR011C; YPR011C	metabolic process;transport	membrane;mitochondrion	structural molecule activity;transporter activity	0.551	0.389	0.71
P36163	mitochondrial metalloendopeptidase OMA1 [OS=Saccharomyces cerevisiae S288c]	853962	OMA1; YKR087C	metabolic process	membrane;mitochondrion	catalytic activity;metal ion binding	0.551	0.389	0.71
P00424	Cytochrome c oxidase polypeptide 5A, mitochondrial [OS=Saccharomyces cerevisiae S288c]	855675	COX5A; YNL052W	metabolic process;transport	membrane;mitochondrion	catalytic activity;transporter activity	1.848	1.31	0.71
Q03327	Mitochondrial fusion and transport protein Ugo1 [OS=Saccharomyces cerevisiae S288c]	852081	UGO1; YDR470C	cell organization and biogenesis;metabolic process	membrane;mitochondrion	protein binding;structural molecule activity	0.425	0.304	0.72
Q02883	N-acyl-phosphatidylethanolamine-hydrolyzing phospholipase D, mitochondrial [OS=Saccharomyces cerevisiae S288c]	856001	FMP30; YPL103C	metabolic process	membrane;mitochondrion	catalytic activity;metal ion binding	0.425	0.304	0.72
P53969	Sorting assembly machinery 50 kDa subunit [OS=Saccharomyces cerevisiae S288c]	855705	SAM50; YNL026W	cell organization and biogenesis;transport	membrane;mitochondrion	protein binding;transporter activity	0.374	0.269	0.72
P40215	External NADH-ubiquinone oxidoreductase 1, mitochondrial [OS=Saccharomyces cerevisiae S288c]	855176	NDE1; YMR145C	metabolic process	mitochondrion	catalytic activity;nucleotide binding	5.469	4.043	0.74
P38988	Mitochondrial GTP/GDP carrier protein 1 [OS=Saccharomyces cerevisiae S288c]	851329	GGC1; YDL198C	cell organization and biogenesis;cellular homeostasis;metabolic process;transport	membrane;mitochondrion	structural molecule activity;transporter activity	3.642	2.728	0.75
Q04935	Cytochrome c oxidase protein 20, mitochondrial [OS=Saccharomyces cerevisiae S288c]	851817	COX20; YDR231C	cell organization and biogenesis;metabolic process	membrane;mitochondrion	protein binding	1.894	1.424	0.75
Q04458	Fatty aldehyde dehydrogenase HFD1 [OS=Saccharomyces cerevisiae S288c]	855137	HFD1; YMR110C	metabolic process	endoplasmic reticulum;endosome;membrane;mitochondrion	catalytic activity	1.783	1.346	0.75
P25087	Sterol 24-C-methyltransferase [OS=Saccharomyces cerevisiae S288c]	855003	ERG6; YML008C	metabolic process	endoplasmic reticulum;membrane;mitochondrion	catalytic activity	0.557	0.425	0.76
Q08970	Mitochondrial metal transporter 2 [OS=Saccharomyces cerevisiae S288c]	855877	MMT2; YPL224C	cellular homeostasis;regulation of biological process;response to stimulus;transport	membrane;mitochondrion	transporter activity	0.532	0.407	0.77
P53721	Respiratory supercomplex factor 2, mitochondrial [OS=Saccharomyces cerevisiae S288c]	855752	RCF2; YNR018W	cell organization and biogenesis	membrane;mitochondrion		7.111	5.579	0.78
P07253	cytochrome B pre-mRNA-processing protein 6 [OS=Saccharomyces cerevisiae S288c]	852417	CBP6; YBR120C	cell organization and biogenesis;metabolic process;regulation of biological process	mitochondrion;ribosome		2.728	2.162	0.79
P38077	ATP synthase subunit gamma, mitochondrial [OS=Saccharomyces cerevisiae S288c]	852327	ATP3; YBR039W	metabolic process;transport	membrane;mitochondrion	catalytic activity;transporter activity	11.328	9	0.79
P40508	Uncharacterized protein YIL077C [OS=Saccharomyces cerevisiae S288c]	854733	YIL077C; YIL077C		mitochondrion		1.448	1.154	0.80
P38127	mitochondrial carrier protein RIM2 [OS=Saccharomyces cerevisiae S288c]	852491	RIM2; YBR192W	cell organization and biogenesis;metabolic process;transport	membrane;mitochondrion	structural molecule activity;transporter activity	1.239	0.995	0.80
P27697	Atypical kinase COQ8, mitochondrial [OS=Saccharomyces cerevisiae S288c]	852758	COQ8; YGL119W	metabolic process	membrane;mitochondrion;organelle lumen	catalytic activity;nucleotide binding	0.453	0.365	0.81
P18239	ADP,ATP carrier protein 2 [OS=Saccharomyces cerevisiae S288c]	852250	PET9; YBL030C	cell death;metabolic process;transport	membrane;mitochondrion	structural molecule activity;transporter activity	135.887	110.034	0.81
P53206	putative cysteine synthase [OS=Saccharomyces cerevisiae S288c]	852895	YGR012W; YGR012W	metabolic process		catalytic activity	0.817	0.668	0.82
P33310	ATP-dependent permease MDL1, mitochondrial [OS=Saccharomyces cerevisiae S288c]	850885	MDL1; YLR188W	transport	membrane;mitochondrion	catalytic activity;nucleotide binding;transporter activity	0.624	0.528	0.85
P05626	ATP synthase subunit 4, mitochondrial	856027	ATP4; YPL078	cell organization and biogenesis;metabolic	membrane;mitochondrion	catalytic activity;transport	3.642	3.084	0.85

	[OS=Saccharomyces cerevisiae S288c]		C	process;transport		er activity			
P25349	Flavoprotein-like protein YCP4 [OS=Saccharomyces cerevisiae S288c]	850360	YCP4; YCR004C	metabolic process;regulation of biological process	cytoplasm;membrane;mitochondrion	catalytic activity;nucleotide binding;protein binding	6.743	5.813	0.86
P40086	Cytochrome c oxidase assembly protein cox15 [OS=Saccharomyces cerevisiae S288c]	856884	COX15; YER141W	cell organization and biogenesis;metabolic process;transport	membrane;mitochondrion	catalytic activity;transporter activity	1.555	1.346	0.87
Q01852	Mitochondrial import inner membrane translocase subunit TIM44 [OS=Saccharomyces cerevisiae S288c]	854790	TIM44; YIL022W	transport	membrane;mitochondrion	nucleotide binding;protein binding	4.125	3.758	0.91
P00890	citrate synthase, mitochondrial [OS=Saccharomyces cerevisiae S288c]	855732	CIT1; YNR001C	metabolic process;transport	cytosol;mitochondrion;organelle lumen	catalytic activity	11.798	10.788	0.91
Q08023	Protein FMP25, mitochondrial [OS=Saccharomyces cerevisiae S288c]	850766	FMP25; YLR077W	cell organization and biogenesis	membrane;mitochondrion		0.968	0.968	1.00
P25573	Mitochondrial inner membrane i-AAA protease supercomplex subunit MGR1 [OS=Saccharomyces cerevisiae S288c]	850313	MGR1; YCL044C	metabolic process	membrane;mitochondrion	protein binding	0.701	0.701	1.00
P08466	mitochondrial nuclease [OS=Saccharomyces cerevisiae S288c]	853222	NUC1; YJL208C	cell death;metabolic process	membrane;mitochondrion;nucleus	catalytic activity;metal ion binding	1.336	1.336	1.00
P00360	glyceraldehyde-3-phosphate dehydrogenase 1 [OS=Saccharomyces cerevisiae S288c]	853395	TDH1; YJL052W	metabolic process	cytoplasm;cytosol;membrane;mitochondrion	catalytic activity;nucleotide binding	0.823	0.823	1.00
Q01574	acetyl-coenzyme A synthetase 1 [OS=Saccharomyces cerevisiae S288c]	851245	ACS1; YAL054C	cell organization and biogenesis;metabolic process	cytoplasm;cytosol;endoplasmic reticulum;mitochondrion;nucleus	catalytic activity;nucleotide binding	0.624	0.624	1.00
Q06668	Methyltransferase OMS1, mitochondrial [OS=Saccharomyces cerevisiae S288c]	851911	OMS1; YDR316W	metabolic process	membrane;mitochondrion	catalytic activity	0.484	0.484	1.00
Q12375	mitochondrial ornithine transporter 1 [OS=Saccharomyces cerevisiae S288c]	854297	ORT1; YOR130C	metabolic process;transport	membrane;mitochondrion	structural molecule activity;transporter activity	0.54	0.54	1.00
P53163	54S ribosomal protein L12, mitochondrial [OS=Saccharomyces cerevisiae S288c]	852811	MNP1; YGL068W	cell organization and biogenesis;metabolic process	mitochondrion;ribosome	RNA binding;structural molecule activity	1.31	1.31	1.00
P40496	37S ribosomal protein S25, mitochondrial [OS=Saccharomyces cerevisiae S288c]	854715	RSM25; YIL093C	cell organization and biogenesis;metabolic process	mitochondrion;ribosome	structural molecule activity	0.468	0.468	1.00
P39533	Homocitrate dehydratase, mitochondrial [OS=Saccharomyces cerevisiae S288c]	853230	ACO2; YJL200C	metabolic process	mitochondrion	catalytic activity;metal ion binding	0.17	0.17	1.00
P34224	Uncharacterized protein YBL059W [OS=Saccharomyces cerevisiae S288c]	852221	YBL059W; YBL059W		membrane;mitochondrion		0.468	0.468	1.00
P34222	Peptidyl-tRNA hydrolase 2 [OS=Saccharomyces cerevisiae S288c]	852223	PTH2; YBL057C	regulation of biological process	cytoplasm;cytosol;membrane;mitochondrion	catalytic activity;protein binding	0.425	0.425	1.00
Q2V2P9	Uncharacterized protein YDR119W-A [OS=Saccharomyces cerevisiae S288c]	3799970	COX26; YDR119W-A		membrane;mitochondrion		1.154	1.154	1.00
Q06510	Lysophosphatidylcholine acyltransferase [OS=Saccharomyces cerevisiae S288c]	856262	TAZ1; YPR140W	cell organization and biogenesis;metabolic process;transport	membrane;mitochondrion	catalytic activity	0.194	0.194	1.00
P54857	lipase 2 [OS=Saccharomyces cerevisiae S288c]	851628	TGL2; YDR058C	metabolic process;transport	mitochondrion	catalytic activity	0.369	0.369	1.00
P32463	Acyl carrier protein, mitochondrial [OS=Saccharomyces cerevisiae S288c]	853642	ACP1; YKL192C	metabolic process;transport	mitochondrion		0.259	0.259	1.00
Q05867	Uncharacterized protein YLR283W, mitochondrial [OS=Saccharomyces cerevisiae S288c]	850988	YLR283W; YLR283W	endoplasmic reticulum;membrane;mitochondrion		0.11	0.11	1.00	
P40581	peroxiredoxin HYR1 [OS=Saccharomyces cerevisiae S288c]	854855	HYR1; YIR037W	metabolic process;response to stimulus	cytoplasm;cytosol;mitochondrion;organelle lumen	antioxidant activity;catalytic activity	0.259	0.259	1.00
Q12328	Mitochondrial import inner membrane translocase subunit TIM22 [OS=Saccharomyces cerevisiae S288c]	851309	TIM22; YDL217C	cell organization and biogenesis;transport	cytosol;membrane;mitochondrion	protein binding;transporter activity	0.233	0.233	1.00
P04039	Cytochrome c oxidase polypeptide VIII, mitochondrial	851111	COX8; YLR395	metabolic process;transport	membrane;mitochondrion	catalytic activity;transport	0.778	0.778	1.00

	[OS=Saccharomyces cerevisiae S288c]		C			er activity			
P38816	thioredoxin reductase 2, mitochondrial [OS=Saccharomyces cerevisiae S288c]	856506	TRR2; YHR106W	cellular homeostasis;metabolic process;regulation of biological process;response to stimulus	cytoplasm;mitochondrion	antioxidant activity;catalytic activity	0.136	0.136	1.00
P14693	sorting assembly machinery 35 kDa subunit [OS=Saccharomyces cerevisiae S288c]	856483	SAM35; YHR083W	cell organization and biogenesis;transport	membrane;mitochondrion	protein binding	0.334	0.334	1.00
Q02863	ubiquitin carboxyl-terminal hydrolase 16 [OS=Saccharomyces cerevisiae S288c]	856032	UBP16; YPL072W	metabolic process	membrane;mitochondrion	catalytic activity	0.072	0.072	1.00
P00358	glyceraldehyde-3-phosphate dehydrogenase 2 [OS=Saccharomyces cerevisiae S288c]	853465	TDH2; YJR009C	cell death;metabolic process	cytoplasm;cytosol;membrane;mitochondrion;nucleus	catalytic activity;nucleotide binding;protein binding	1.721	2.008	1.17
P21560	Protein CBP3, mitochondrial [OS=Saccharomyces cerevisiae S288c]	855886	CBP3; YPL215W	cell organization and biogenesis;regulation of biological process	membrane;mitochondrion;ribosome		1.031	1.219	1.18
P08539	Guanine nucleotide-binding protein alpha-1 subunit [OS=Saccharomyces cerevisiae S288c]	856394	GPA1; YHR005C	cell organization and biogenesis;cellular component movement;regulation of biological process;response to stimulus;transport	cytosol;endosome;membrane	catalytic activity;metal ion binding;nucleotide binding;protein binding;signal transducer activity	0.682	0.812	1.19
P22136	ATPase expression protein 2, mitochondrial [OS=Saccharomyces cerevisiae S288c]	855325	AEP2; YMR282C	metabolic process;regulation of biological process	mitochondrion	RNA binding	0.359	0.431	1.20
P40416	Iron-sulfur clusters transporter ATM1, mitochondrial [OS=Saccharomyces cerevisiae S288c]	855347	ATM1; YMR301C	cellular homeostasis;metabolic process;transport	membrane;mitochondrion	catalytic activity;nucleotide binding;transporter activity	0.453	0.546	1.21
P53982	isocitrate dehydrogenase [NADP] [OS=Saccharomyces cerevisiae S288c]	855723	IDP3; YNL009W	metabolic process	cytoplasm;mitochondrion	catalytic activity;metal ion binding;nucleotide binding	1.943	2.398	1.23
P00560	Phosphoglycerate kinase [OS=Saccharomyces cerevisiae S288c]	850370	PGK1; YCR012W	metabolic process	cytoplasm;membrane;mitochondrion	catalytic activity;nucleotide binding	2.415	2.981	1.23
Q07651	SUR7 family protein FMP45 [OS=Saccharomyces cerevisiae S288c]	851304	FMP45; YDL222C	cell differentiation;cell organization and biogenesis	membrane;mitochondrion		3.125	3.924	1.26
P10823	Guanine nucleotide-binding protein alpha-2 subunit [OS=Saccharomyces cerevisiae S288c]	856741	GPA2; YER020W	cell differentiation;cell growth;cell organization and biogenesis;cellular homeostasis;regulation of biological process;response to stimulus	cytosol;membrane;mitochondrion	catalytic activity;metal ion binding;nucleotide binding;protein binding;signal transducer activity	0.978	1.346	1.38
P53230	Phosphatidate cytidylyltransferase, mitochondrial [OS=Saccharomyces cerevisiae S288c]	852937	TAM41; YGR046W	metabolic process	membrane;mitochondrion;organelle lumen	catalytic activity	0.292	0.407	1.39
P50087	MICOS subunit MIC26 [OS=Saccharomyces cerevisiae S288c]	853150	MOS2; MIC26; YGR235C	cell organization and biogenesis	membrane;mitochondrion		0.638	0.931	1.46
P32792	UPF0744 protein YSC83 [OS=Saccharomyces cerevisiae S288c]	856410	YSC83; YHR017W	membrane;mitochondrion		0.233	0.369	1.58	
P32378	4-hydroxybenzoate polyprenyltransferase, mitochondrial [OS=Saccharomyces cerevisiae S288c]	855778	COQ2; YNR041C	metabolic process;transport	membrane;mitochondrion	catalytic activity	0.292	0.468	1.60
Q12289	mitochondrial carnitine carrier [OS=Saccharomyces cerevisiae S288c]	854267	CRC1; YOR100C	metabolic process;transport	membrane;mitochondrion	structural molecule activity;transporter activity	0.931	1.683	1.81
Q08245	protein ZEO1 [OS=Saccharomyces cerevisiae S288c]	854040	ZEO1; YOL109W	cell organization and biogenesis	membrane;mitochondrion	protein binding	2.162	4.012	1.86
Q12230	Sphingolipid long chain base-responsive protein LSP1 [OS=Saccharomyces cerevisiae S288c]	856103	LSP1; YPL004C	regulation of biological process;response to stimulus;transport	cytoplasm;membrane;mitochondrion		14.849	28.286	1.90
Q12466	tricalbin-1 [OS=Saccharomyces cerevisiae S288c]	854253	TCB1; YOR086C	cell organization and biogenesis;regulation of biological process	endoplasmic reticulum;membrane;mitochondrion	metal ion binding;protein binding	0.191	0.369	1.93
Q06405	ATP synthase subunit f, mitochondrial [OS=Saccharomyces cerevisiae S288c]	851983	ATP17; YDR377W	metabolic process;transport	membrane;mitochondrion	catalytic activity;transporter activity	1.512	2.981	1.97
P38909	cytochrome c mitochondrial import factor CYC2	854202	CYC2; YOR037	cell organization and biogenesis;metabolic process	membrane;mitochondrion	catalytic activity	0.093	0.194	2.09

	[OS=Saccharomyces cerevisiae S288c]		W						
Q04472	Mitochondrial inner membrane i-AAA protease supercomplex subunit MGR3 [OS=Saccharomyces cerevisiae S288c]	855142	MGR3; YMR115W	metabolic process	membrane;mitochondrion	protein binding	0.15	0.322	2.15
P29311	Protein BMH1 [OS=Saccharomyces cerevisiae S288c]	856924	BMH1; YER177W	cell differentiation;cell growth;cell organization and biogenesis;metabolic process;regulation of biological process;response to stimulus	cytoplasm;membrane;nucleus	DNA binding;protein binding	0.136	0.292	2.15
Q00873	cytochrome c1 heme lyase [OS=Saccharomyces cerevisiae S288c]	853775	CYT2; YKL087C	cell organization and biogenesis;metabolic process	membrane;mitochondrion	catalytic activity;metal ion binding	0.166	0.359	2.16
P38626	NADH-cytochrome b5 reductase 1 [OS=Saccharomyces cerevisiae S288c]	854768	CBR1; YIL043C	metabolic process	endoplasmic reticulum;membrane;mitochondrion;nucleus	catalytic activity	0.334	0.778	2.33
P16603	NADPH--cytochrome P450 reductase [OS=Saccharomyces cerevisiae S288c]	856438	NCP1; YHR042W	metabolic process	cytoplasm;cytosol;endoplasmic reticulum;membrane;mitochondrion	catalytic activity;metal ion binding;nucleotide binding	0.194	0.604	3.11
Q02981	ABC1 family protein YPL109C, mitochondrial [OS=Saccharomyces cerevisiae S288c]	855995	YPL109C; YPL109C	mitochondrion		0.059	0.259	4.39	
P54115	Magnesium-activated aldehyde dehydrogenase, cytosolic [OS=Saccharomyces cerevisiae S288c]	856044	ALD6; YPL061W	metabolic process;response to stimulus	cytoplasm;cytosol;mitochondrion	catalytic activity	0.077	0.346	4.49
P34231	Uncharacterized protein YKL187C [OS=Saccharomyces cerevisiae S288c]	853648	FAT3; YKL187C; YKL187C	cell organization and biogenesis;transport	membrane;mitochondrion		0.093	0.425	4.57
P28737	Protein MSP1 [OS=Saccharomyces cerevisiae S288c]	852915	MSP1; YGR028W	cell organization and biogenesis;metabolic process;regulation of biological process;response to stimulus;transport	membrane;mitochondrion;nucleus	catalytic activity;nucleotide binding;protein binding;RNA binding	0.122	0.585	4.80
P38825	Protein TOM71 [OS=Saccharomyces cerevisiae S288c]	856517	TOM71; YHR117W	transport	membrane;mitochondrion	protein binding;transporter activity	0.061	0.512	8.39
P39518	Long-chain-fatty-acid--CoA ligase 2 [OS=Saccharomyces cerevisiae S288c]	856734	FAA2; YER015W	metabolic process;transport	cytoplasm;mitochondrion	catalytic activity;nucleotide binding	0.055	0.619	11.25
Q03640	Tricalbin-3 [OS=Saccharomyces cerevisiae S288c]	854903	TCB3; YML072C	cell organization and biogenesis;regulation of biological process	endoplasmic reticulum;membrane;mitochondrion	metal ion binding;protein binding	0.032	0.365	11.41

Supplemental Table S11. The relative concentrations of proteins in mitochondria purified from WT or *ups2Δ* cells cultured without LCA. Mitochondria were purified from WT or *ups2Δ* cells recovered on day 4 of cell culturing without LCA. Mass spectrometry-based identification and quantitation of proteins recovered in purified mitochondria were performed as described in Materials and methods. The exponentially modified protein abundance index (emPAI) provided by the ″Proteome Discoverer″ software was used to calculate the relative abundance of mitochondrial proteins between specified datasets. Data on the relative concentrations of mitochondrial proteins are presented as means of 2 independent experiments.

Accession	Description	Entrez Gene ID	Gene ID	Biological Process	Cellular Component	Molecular Function	emPAI WT	emPAI *ups2*	Ratio *ups2* / WT
P37267	Assembly factor cbp4 [OS=Saccharomyces cerevisiae S288c]		CBP4	cell organization and biogenesis	membrane;mitochondrion		7.111	0.233	0.03
P81449	ATP synthase subunit e, mitochondrial [OS=Saccharomyces cerevisiae S288c]	851922	TIM11; YDR322C-A	cell organization and biogenesis;metabolic process;transport	membrane;mitochondrion	catalytic activity;structural molecule activity;transporter activity	9	0.334	0.04
P07253	cytochrome B pre-mRNA-processing protein 6 [OS=Saccharomyces cerevisiae S288c]	852417	CBP6; YBR120C	cell organization and biogenesis;metabolic process;regulation of biological process	mitochondrion;ribosome		4.179	0.179	0.04
P19955	37S ribosomal protein YMR-31, mitochondrial [OS=Saccharomyces cerevisiae S288c]	850610	YMR31; YFR049W	cell organization and biogenesis;metabolic process	mitochondrion;ribosome	catalytic activity;protein binding;structural molecule activity	26.826	1.154	0.04
P00128	Cytochrome b-c1 complex subunit 7 [OS=Saccharomyces cerevisiae S288c]	852142	QCR7; YDR529C	cell organization and biogenesis;metabolic process;transport	membrane;mitochondrion	catalytic activity;transporter activity	34.938	1.783	0.05
P32799	Cytochrome c oxidase subunit 6A, mitochondrial [OS=Saccharomyces cerevisiae S288c]	852684	COX13; YGL191W	cell organization and biogenesis;metabolic process;regulation of biological process;transport	membrane;mitochondrion	catalytic activity;enzyme regulator activity;transporter activity	18.953	0.995	0.05
P43594	MICOS complex subunit MIC19 [OS=Saccharomyces cerevisiae S288c]	850563	AIM13; MIC19; YFR011C	cell organization and biogenesis	cytoplasm;membrane;mitochondrion		4.337	0.233	0.05
Q12165	ATP synthase subunit delta, mitochondrial [OS=Saccharomyces cerevisiae S288c]	851560	ATP16; YDL004W	metabolic process;transport	membrane;mitochondrion	catalytic activity;transporter activity	4.623	0.334	0.07
P38885	Altered inheritance of mitochondria protein 46, mitochondrial [OS=Saccharomyces cerevisiae S288c]	856606	AIM46; YHR199C		mitochondrion	catalytic activity	2.728	0.245	0.09
P40165	NAD(P)H-hydrate epimerase [OS=Saccharomyces cerevisiae S288c]	855521	YNL200C; YNL200C	metabolic process	cytoplasm;mitochondrion	catalytic activity;metal ion binding;nucleotide binding	2.162	0.212	0.10
P04037	cytochrome c oxidase subunit 4, mitochondrial [OS=Saccharomyces cerevisiae S288c]	852688	COX4; YGL187C	cell organization and biogenesis;metabolic process;transport	membrane;mitochondrion	catalytic activity;metal ion binding;transporter activity	42.288	4.337	0.10
P35736	Uncharacterized protein YKL050C [OS=Saccharomyces cerevisiae S288c]	853816	YKL050C; YKL050C	cell organization and biogenesis	membrane		0.374	0.04	0.11
P00427	Cytochrome c oxidase subunit 6, mitochondrial [OS=Saccharomyces cerevisiae S288c]	856448	COX6; YHR051W	metabolic process;transport	membrane;mitochondrion	catalytic activity;metal ion binding;transporter activity	7.254	0.778	0.11
P36141	Putative redox protein fmp46, mitochondrial [OS=Saccharomyces cerevisiae S288c]	853923	FMP46; YKR049C	metabolic process	mitochondrion	catalytic activity;protein binding	4.337	0.52	0.12
Q96VH5	MICOS complex subunit Mic10 [OS=Saccharomyces cerevisiae S288c]	850300	MOS1; MIC10; YCL057C-A	cell organization and biogenesis	membrane;mitochondrion		6.197	0.931	0.15
P00175	Cytochrome b2, mitochondrial [OS=Saccharomyces cerevisiae S288c]	854950	CYB2; YML054C	metabolic process;transport	cytosol;membrane;mitochondrion;nucleus	catalytic activity;metal ion binding;nucleotide binding	1.239	0.189	0.15
P38341	MICOS complex subunit MIC12 [OS=Saccharomyces cerevisiae S288c]	852566	AIM5; MIC12; YBR262C	cell organization and biogenesis	membrane;mitochondrion		1.783	0.292	0.16

Q05892	MIOREX complex component 2 [OS=Saccharomyces cerevisiae S288c]	850997	YLR290C; COQ11; YLR290C	metabolic process	mitochondrion	catalytic activity	3.217	0.54	0.17
P40502	Altered inheritance of mitochondria protein 19, mitochondrial [OS=Saccharomyces cerevisiae S288c]	854722	AIM19; YIL087C		membrane;mitochondrion		1.512	0.259	0.17
P39525	3-oxoacyl-[acyl-carrier-protein] synthase homolog [OS=Saccharomyces cerevisiae S288c]	856790	CEM1; YER061C	metabolic process	mitochondrion	catalytic activity	2.899	0.52	0.18
P22353	54S ribosomal protein L8, mitochondrial [OS=Saccharomyces cerevisiae S288c]	853382	MRPL8; YJL063C	cell organization and biogenesis;metabolic process	mitochondrion;ribosome	structural molecule activity	0.848	0.166	0.20
P08525	Cytochrome b-c1 complex subunit 8 [OS=Saccharomyces cerevisiae S288c]	853273	QCR8; YJL166W	metabolic process;transport	membrane;mitochondrion	catalytic activity;transporter activity	2.981	0.585	0.20
P02992	elongation factor Tu, mitochondrial [OS=Saccharomyces cerevisiae S288c]	854359	TUF1; YOR187W	cell organization and biogenesis;metabolic process	mitochondrion	catalytic activity;nucleotide binding;RNA binding	30.623	6.017	0.20
P08067	cytochrome b-c1 complex subunit Rieske, mitochondrial [OS=Saccharomyces cerevisiae S288c]	856689	RIP1; YEL024W	metabolic process;transport	membrane;mitochondrion	catalytic activity;metal ion binding;transporter activity	55.234	11.115	0.20
P38523	GrpE protein homolog, mitochondrial [OS=Saccharomyces cerevisiae S288c]	854407	MGE1; YOR232W	metabolic process;regulation of biological process;transport	membrane;mitochondrion;organelle lumen	enzyme regulator activity;nucleotide binding;protein binding	1.336	0.274	0.21
P00424	Cytochrome c oxidase polypeptide 5A, mitochondrial [OS=Saccharomyces cerevisiae S288c]	855675	COX5A; YNL052W	metabolic process;transport	membrane;mitochondrion	catalytic activity;transporter activity	2.511	0.52	0.21
P25372	Thioredoxin-3, mitochondrial [OS=Saccharomyces cerevisiae S288c]	850444	TRX3; YCR083W	cellular homeostasis;metabolic process;regulation of biological process;response to stimulus;transport	cytoplasm;mitochondrion	catalytic activity	2.511	0.52	0.21
Q04689	Altered inheritance of mitochondria protein 32 [OS=Saccharomyces cerevisiae S288c]	854955	AIM32; YML050W				0.52	0.11	0.21
P53305	Mitochondrial 37S ribosomal protein S27 [OS=Saccharomyces cerevisiae S288c]	853129	RSM27; YGR215W	cell organization and biogenesis;metabolic process	mitochondrion;ribosome	structural molecule activity	2.162	0.468	0.22
Q08970	Mitochondrial metal transporter 2 [OS=Saccharomyces cerevisiae S288c]	855877	MMT2; YPL224C	cellular homeostasis;regulation of biological process;response to stimulus;transport	membrane;mitochondrion	transporter activity	0.407	0.089	0.22
Q02608	37S ribosomal protein S16, mitochondrial [OS=Saccharomyces cerevisiae S288c]	856094	MRPS16; YPL013C	cell organization and biogenesis;metabolic process	mitochondrion;ribosome	structural molecule activity	1.683	0.389	0.23
P10834	protein PET54 [OS=Saccharomyces cerevisiae S288c]	853137	PET54; YGR222W	metabolic process;regulation of biological process	membrane;mitochondrion;organelle lumen	nucleotide binding;RNA binding;translation regulator activity	1.565	0.369	0.24
Q07500	External NADH-ubiquinone oxidoreductase 2, mitochondrial [OS=Saccharomyces cerevisiae S288c]	851474	NDE2; YDL085W	metabolic process	mitochondrion	catalytic activity;nucleotide binding	13.678	3.299	0.24
Q06236	Mitochondrial inner membrane protein SHH4 [OS=Saccharomyces cerevisiae S288c]	850861	SHH4; YLR164W	metabolic process	membrane;mitochondrion	catalytic activity;metal ion binding	3.217	0.778	0.24
P00127	Cytochrome b-c1 complex subunit 6 [OS=Saccharomyces cerevisiae S288c]	850593	QCR6; YFR033C	metabolic process;transport	membrane;mitochondrion	catalytic activity;transporter activity	1.371	0.334	0.24
Q12428	Probable 2-methylcitrate dehydratase [OS=Saccharomyces cerevisiae S288c]	856108	PDH1; YPR002W	metabolic process	cytoplasm;membrane;mitochondrion	catalytic activity	18.11	4.623	0.26
P48569	Uncharacterized protein YDL183C [OS=Saccharomyces cerevisiae S288c]	851345	YDL183C; YDL183C	transport	mitochondrion		1.069	0.274	0.26
P19516	Cytochrome c oxidase assembly protein COX11, mitochondrial [OS=Saccharomyces cerevisiae S288c]	855971	COX11; YPL132W	cell organization and biogenesis;metabolic process	membrane;mitochondrion;ribosome	metal ion binding	1.069	0.274	0.26
P25573	Mitochondrial inner membrane i-AAA protease supercomplex subunit MGR1 [OS=Saccharomyces cerevisiae S288c]	850313	MGR1; YCL044C	metabolic process	membrane;mitochondrion	protein binding	1.649	0.425	0.26
P53219	Abhydrolase domain-containing protein IMO32 [OS=Saccharomyces cerevisiae S288c]	852919	IMO32; YGR031W	metabolic process;transport	mitochondrion	catalytic activity	0.931	0.245	0.26

Q03798	Altered inheritance of mitochondria protein 36, mitochondrial [OS=Saccharomyces cerevisiae S288c]	855189	AIM36; YMR157C		membrane;mitochondrion		1.637	0.438	0.27
P53326	Uncharacterized protein YGR266W [OS=Saccharomyces cerevisiae S288c]	853182	YGR266W; YGR266W		membrane;mitochondrion		1.081	0.299	0.28
Q03201	37S ribosomal protein S10, mitochondrial [OS=Saccharomyces cerevisiae S288c]	851611	RSM10; YDR041W	cell organization and biogenesis;metabolic process	mitochondrion;ribosome	structural molecule activity	0.701	0.194	0.28
P38175	37S ribosomal protein MRP21, mitochondrial [OS=Saccharomyces cerevisiae S288c]	852188	MRP21; YBL090W	cell organization and biogenesis;metabolic process	mitochondrion;ribosome	structural molecule activity	0.701	0.194	0.28
P00445	Superoxide dismutase [Cu-Zn] [OS=Saccharomyces cerevisiae S288c]	853568	SOD1; YJR104C	cell organization and biogenesis;cellular homeostasis;metabolic process;regulation of biological process;response to stimulus	cytoplasm;cytosol;mitochondrion	antioxidant activity;catalytic activity;metal ion binding;protein binding	1.848	0.52	0.28
Q06892	NADH kinase POS5, mitochondrial [OS=Saccharomyces cerevisiae S288c]	855913	POS5; YPL188W	metabolic process;response to stimulus	mitochondrion;organelle lumen	catalytic activity;nucleotide binding	1.31	0.369	0.28
P25719	Peptidyl-prolyl cis-trans isomerase C, mitochondrial [OS=Saccharomyces cerevisiae S288c]	854897	CPR3; YML078W	cell death;metabolic process	mitochondrion;organelle lumen	catalytic activity	2.981	0.848	0.28
P43567	alanine--glyoxylate aminotransferase 1 [OS=Saccharomyces cerevisiae S288c]	850514	AGX1; YFL030W	metabolic process	cytosol;mitochondrion	catalytic activity	2.162	0.616	0.28
Q04935	Cytochrome c oxidase protein 20, mitochondrial [OS=Saccharomyces cerevisiae S288c]	851817	COX20; YDR231C	cell organization and biogenesis;metabolic process	membrane;mitochondrion	protein binding	2.455	0.701	0.29
P14693	sorting assembly machinery 35 kDa subunit [OS=Saccharomyces cerevisiae S288c]	856483	SAM35; YHR083W	cell organization and biogenesis;transport	membrane;mitochondrion	protein binding	0.54	0.155	0.29
P42847	37S ribosomal protein S18, mitochondrial [OS=Saccharomyces cerevisiae S288c]	855410	MRPS18; YNL306W	cell organization and biogenesis;metabolic process	mitochondrion;ribosome	RNA binding;structural molecule activity	0.54	0.155	0.29
Q04472	Mitochondrial inner membrane i-AAA protease supercomplex subunit MGR3 [OS=Saccharomyces cerevisiae S288c]	855142	MGR3; YMR115W	metabolic process	membrane;mitochondrion	protein binding	0.52	0.15	0.29
P25605	Acetolactate synthase small subunit, mitochondrial [OS=Saccharomyces cerevisiae S288c]	850348	ILV6; YCL009C	metabolic process;regulation of biological process	mitochondrion	catalytic activity;enzyme regulator activity	6.848	1.976	0.29
P00401	Cytochrome c oxidase subunit 1 [OS=Saccharomyces cerevisiae S288c]	854598	COX1; Q0045	metabolic process;transport	membrane;mitochondrion	catalytic activity;metal ion binding;transporter activity	1.61	0.468	0.29
P00927	Threonine dehydratase, mitochondrial [OS=Saccharomyces cerevisiae S288c]	856819	ILV1; YER086W	metabolic process	cytoplasm;mitochondrion	catalytic activity	3.125	0.914	0.29
Q06678	54S ribosomal protein L35, mitochondrial [OS=Saccharomyces cerevisiae S288c]	851921	MRPL35; YDR322W	cell organization and biogenesis;metabolic process	mitochondrion;ribosome	structural molecule activity	1.031	0.304	0.29
P38122	3-methyl-2-oxobutanoate hydroxymethyltransferase [OS=Saccharomyces cerevisiae S288c]	852474	ECM31; YBR176W	metabolic process	mitochondrion	catalytic activity	0.413	0.122	0.30
P21954	Isocitrate dehydrogenase [NADP], mitochondrial [OS=Saccharomyces cerevisiae S288c]	851493	IDP1; YDL066W	metabolic process	mitochondrion	catalytic activity;metal ion binding;nucleotide binding;protein binding	38.811	11.589	0.30
P38120	37S ribosomal protein S9, mitochondrial [OS=Saccharomyces cerevisiae S288c]	852443	MRPS9; YBR146W	cell organization and biogenesis;metabolic process	mitochondrion;ribosome	RNA binding;structural molecule activity	2.384	0.719	0.30
Q01217	Protein ARG5,6, mitochondrial [OS=Saccharomyces cerevisiae S288c]	856800	ARG5,6; YER069W	metabolic process;regulation of biological process	cytoplasm;mitochondrion;organelle lumen	catalytic activity;nucleotide binding;protein binding	0.286	0.087	0.30
Q03246	37S ribosomal protein S17, mitochondrial [OS=Saccharomyces cerevisiae	855226	MRPS17; YMR188C	cell differentiation;cell organization and biogenesis;metabolic process	mitochondrion;ribosome	RNA binding;structural molecule	1.276	0.389	0.30

	S288c]					activity			
P22354	54S ribosomal protein L20, mitochondrial [OS=Saccharomyces cerevisiae S288c]	853960	MRPL20; YKR085C	cell organization and biogenesis;metabolic process	mitochondrion;ribosome	structural molecule activity	1.276	0.389	0.30
P09950	5-aminolevulinate synthase, mitochondrial [OS=Saccharomyces cerevisiae S288c]	851818	HEM1; YDR232W	metabolic process	mitochondrion;organelle lumen	catalytic activity	0.28	0.086	0.31
P00830	ATP synthase subunit beta, mitochondrial [OS=Saccharomyces cerevisiae S288c]	853585	ATP2; YJR121W	metabolic process;transport	cytosol;membrane;mitochondrion	catalytic activity;nucleotide binding;protein binding;transporter activity	78.433	24.119	0.31
P07236	Threonine--tRNA ligase, mitochondrial [OS=Saccharomyces cerevisiae S288c]	853640	MST1; YKL194C	metabolic process	cytoplasm;mitochondrion;organelle lumen	catalytic activity;nucleotide binding;RNA binding	0.233	0.072	0.31
P32445	Single-stranded DNA-binding protein RIM1, mitochondrial [OS=Saccharomyces cerevisiae S288c]	850395	RIM1; YCR028C-A	cell organization and biogenesis;metabolic process;regulation of biological process	mitochondrion	DNA binding	3.642	1.154	0.32
P37298	Succinate dehydrogenase [ubiquinone] cytochrome b small subunit, mitochondrial [OS=Saccharomyces cerevisiae S288c]	851758	SDH4; YDR178W	metabolic process;transport	membrane;mitochondrion	catalytic activity;metal ion binding	3.642	1.154	0.32
P81451	ATP synthase subunit K, mitochondrial [OS=Saccharomyces cerevisiae S288c]	854077	ATP19; YOL077W-A	cell organization and biogenesis;metabolic process;transport	membrane;mitochondrion	catalytic activity;transporter activity	3.642	1.154	0.32
P32387	54S ribosomal protein L41, mitochondrial [OS=Saccharomyces cerevisiae S288c]	852014	MRP20; YDR405W	cell organization and biogenesis;metabolic process	mitochondrion;ribosome	nucleotide binding;protein binding;RNA binding;structural molecule activity	1.581	0.501	0.32
Q99297	Mitochondrial 2-oxodicarboxylate carrier 2 [OS=Saccharomyces cerevisiae S288c]	854397	ODC2; YOR222W	metabolic process;transport	membrane;mitochondrion	structural molecule activity;transporter activity	1.054	0.334	0.32
P23833	Protein SCO1, mitochondrial [OS=Saccharomyces cerevisiae S288c]	852325	SCO1; YBR037C	cell organization and biogenesis;cellular homeostasis;response to stimulus;transport	membrane;mitochondrion	antioxidant activity;catalytic activity;metal ion binding	1.448	0.468	0.32
Q12283	Malonyl CoA-acyl carrier protein transacylase, mitochondrial [OS=Saccharomyces cerevisiae S288c]	854396	MCT1; YOR221C	metabolic process	mitochondrion	catalytic activity	0.896	0.292	0.33
P40008	Protein FMP52, mitochondrial [OS=Saccharomyces cerevisiae S288c]	856721	FMP52; YER004W		endoplasmic reticulum;membrane;mitochondrion	catalytic activity	3.217	1.054	0.33
P39522	Dihydroxy-acid dehydratase, mitochondrial [OS=Saccharomyces cerevisiae S288c]	853473	ILV3; YJR016C	metabolic process	mitochondrion	catalytic activity;metal ion binding	29.539	9.723	0.33
P12686	37S ribosomal protein MRP13, mitochondrial [OS=Saccharomyces cerevisiae S288c]	852975	MRP13; YGR084C	cell organization and biogenesis;metabolic process	mitochondrion;ribosome	structural molecule activity	1.239	0.413	0.33
P10662	37S ribosomal protein MRP1, mitochondrial [OS=Saccharomyces cerevisiae S288c]	851948	MRP1; YDR347W	cell organization and biogenesis;metabolic process;response to stimulus	mitochondrion;ribosome	antioxidant activity;catalytic activity;metal ion binding;structural molecule activity	2.981	0.995	0.33
P02381	Ribosomal protein VAR1, mitochondrial [OS=Saccharomyces cerevisiae S288c]	854586	VAR1; Q0140	cell organization and biogenesis;metabolic process	mitochondrion;ribosome	structural molecule activity	0.688	0.233	0.34
P11325	Leucine--tRNA ligase, mitochondrial [OS=Saccharomyces cerevisiae S288c]	851098	NAM2; YLR382C	cell organization and biogenesis;metabolic process;regulation of biological process	cytosol;mitochondrion;organelle lumen	catalytic activity;nucleotide binding;RNA binding	0.353	0.12	0.34
P25349	Flavoprotein-like protein YCP4 [OS=Saccharomyces cerevisiae S288c]	850360	YCP4; YCR004C	metabolic process;regulation of biological process	cytoplasm;membrane;mitochondrion	catalytic activity;nucleotide binding;protein binding	9	3.084	0.34
P40961	prohibitin-1 [OS=Saccharomyces cerevisiae S288c]	853033	PHB1; YGR132C	cell organization and biogenesis;metabolic process;regulation of biological process	membrane;mitochondrion	protein binding	6.848	2.36	0.34
P00410	Cytochrome c oxidase subunit 2 [OS=Saccharomyces cerevisiae S288c]	854622	COX2; Q0250	metabolic process;transport	membrane;mitochondrion	catalytic activity;metal ion binding;protei	4.623	1.61	0.35

						n binding;transporter activity			
Q02883	N-acyl-phosphatidylethanolamine-hydrolyzing phospholipase D, mitochondrial [OS=Saccharomyces cerevisiae S288c]	856001	FMP30; YPL103C	metabolic process	membrane;mitochondrion	catalytic activity;metal ion binding	0.557	0.194	0.35
P32860	NifU-like protein, mitochondrial [OS=Saccharomyces cerevisiae S288c]	853826	NFU1; YKL040C	cell organization and biogenesis;cellular homeostasis;metabolic process	mitochondrion;organelle lumen	metal ion binding	3.87	1.371	0.35
P18900	hexaprenyl pyrophosphate synthase, mitochondrial [OS=Saccharomyces cerevisiae S288c]	852288	COQ1; YBR003W	metabolic process	membrane;mitochondrion	catalytic activity;metal ion binding;protein binding	0.468	0.166	0.35
P39726	Glycine cleavage system H protein, mitochondrial [OS=Saccharomyces cerevisiae S288c]	851254	GCV3; YAL044C	metabolic process	mitochondrion	catalytic activity	4.995	1.783	0.36
P00044	Cytochrome c iso-1 [OS=Saccharomyces cerevisiae S288c]	853507	CYC1; YJR048W	metabolic process;transport	mitochondrion	metal ion binding;protein binding	9	3.217	0.36
P49367	Homoaconitase, mitochondrial [OS=Saccharomyces cerevisiae S288c]	851820	LYS4; YDR234W	metabolic process	mitochondrion	catalytic activity;metal ion binding;protein binding	1.54	0.551	0.36
P21306	ATP synthase subunit epsilon, mitochondrial [OS=Saccharomyces cerevisiae S288c]	855857	ATP15; YPL271W	metabolic process;transport	membrane;mitochondrion	catalytic activity;transporter activity	2.162	0.778	0.36
P53170	[Pyruvate dehydrogenase (acetyl-transferring)] kinase 2, mitochondrial [OS=Saccharomyces cerevisiae S288c]	852821	PKP2; YGL059W	metabolic process;regulation of biological process	mitochondrion;organelle lumen	catalytic activity;nucleotide binding;protein binding	0.778	0.28	0.36
Q3E824	Uncharacterized protein YOR020W-A [OS=Saccharomyces cerevisiae S288c]	1466480	YOR020W-A; YOR020W-A	metabolic process;transport	membrane;mitochondrion		5.813	2.162	0.37
Q02784	Monothiol glutaredoxin-5, mitochondrial [OS=Saccharomyces cerevisiae S288c]	856048	GRX5; YPL059W	cell organization and biogenesis;cellular homeostasis;metabolic process;regulation of biological process;response to stimulus	mitochondrion;organelle lumen	catalytic activity;metal ion binding	1.783	0.668	0.37
Q6Q560	Protein isd11 [OS=Saccharomyces cerevisiae S288c]	856774	ISD11; YER048W-A	cell organization and biogenesis;metabolic process	mitochondrion;organelle lumen	catalytic activity;protein binding	1.783	0.668	0.37
P08417	fumarate hydratase, mitochondrial [OS=Saccharomyces cerevisiae S288c]	855866	FUM1; YPL262W	cell organization and biogenesis;metabolic process	cytoplasm;cytosol;mitochondrion;organelle lumen	catalytic activity	13.874	5.21	0.38
P25374	Cysteine desulfurase, mitochondrial [OS=Saccharomyces cerevisiae S288c]	850343	NFS1; YCL017C	cell organization and biogenesis;cellular homeostasis;metabolic process	mitochondrion;nucleus	catalytic activity;metal ion binding;protein binding	2.594	0.978	0.38
Q12298	uncharacterized ABC transporter ATP-binding protein YDR061W [OS=Saccharomyces cerevisiae S288c]	851633	YDR061W; YDR061W		mitochondrion	catalytic activity;nucleotide binding	0.512	0.194	0.38
P32891	D-lactate dehydrogenase [cytochrome] 1, mitochondrial [OS=Saccharomyces cerevisiae S288c]	851380	DLD1; YDL174C	metabolic process;transport	membrane;mitochondrion	catalytic activity;nucleotide binding	18.684	7.161	0.38
P33416	Heat shock protein 78, mitochondrial [OS=Saccharomyces cerevisiae S288c]	851845	HSP78; YDR258C	cell organization and biogenesis;metabolic process;regulation of biological process;response to stimulus	mitochondrion;organelle lumen	catalytic activity;nucleotide binding;protein binding	7.929	3.042	0.38
P36525	54S ribosomal protein L24, mitochondrial [OS=Saccharomyces cerevisiae S288c]	855231	MRPL24; YMR193W	cell organization and biogenesis;metabolic process	mitochondrion;ribosome	RNA binding;structural molecule activity	1.512	0.585	0.39
P54003	Protein SUR7 [OS=Saccharomyces cerevisiae S288c]	854953	SUR7; YML052W	cell differentiation;transport	membrane;mitochondrion		1.512	0.585	0.39
P00431	cytochrome c peroxidase, mitochondrial [OS=Saccharomyces cerevisiae S288c]	853940	CCP1; YKR066C	metabolic process;response to stimulus	mitochondrion;organelle lumen	antioxidant activity;catalytic activity;metal ion binding;protein binding	1.424	0.557	0.39
P21560	Protein CBP3, mitochondrial [OS=Saccharomyces cerevisiae S288c]	855886	CBP3; YPL215W	cell organization and biogenesis;regulation of biological process	membrane;mitochondrion;ribosome		1.424	0.557	0.39
P37293	N-terminal acetyltransferase 2 [OS=Saccharomyces cerevisiae S288c]	853050	NAT2; YGR147C	metabolic process	cytoplasm;mitochondrion	catalytic activity	1.404	0.551	0.39

P40053	Altered inheritance of mitochondria protein 9, mitochondrial [OS=Saccharomyces cerevisiae S288c]	856813	AIM9; YER080W		mitochondrion		4.555	1.798	0.39
P53163	54S ribosomal protein L12, mitochondrial [OS=Saccharomyces cerevisiae S288c]	852811	MNP1; YGL068W	cell organization and biogenesis;metabolic process	mitochondrion;ribosome	RNA binding;structural molecule activity	1.31	0.52	0.40
P47052	succinate dehydrogenase [ubiquinone] flavoprotein subunit 2, mitochondrial [OS=Saccharomyces cerevisiae S288c]	853405	YJL045W; YJL045W	metabolic process;transport	membrane;mitochondrion	catalytic activity;nucleotide binding	4.712	1.88	0.40
Q01574	acetyl-coenzyme A synthetase 1 [OS=Saccharomyces cerevisiae S288c]	851245	ACS1; YAL054C	cell organization and biogenesis;metabolic process	cytoplasm;cytosol;endoplasmic reticulum;mitochondrion;nucleus	catalytic activity;nucleotide binding	2.36	0.947	0.40
P40185	Protein mmf1, mitochondrial [OS=Saccharomyces cerevisiae S288c]	854760	MMF1; YIL051C	cell organization and biogenesis;metabolic process	mitochondrion;organelle lumen		5.31	2.162	0.41
P00447	Superoxide dismutase [Mn], mitochondrial [OS=Saccharomyces cerevisiae S288c]	856399	SOD2; YHR008C	metabolic process;response to stimulus	mitochondrion;organelle lumen	antioxidant activity;catalytic activity;metal ion binding	6.017	2.455	0.41
Q04599	54S ribosomal protein L1, mitochondrial [OS=Saccharomyces cerevisiae S288c]	851694	MRPL1; YDR116C	cell organization and biogenesis;metabolic process	mitochondrion;ribosome	RNA binding;structural molecule activity	1.069	0.438	0.41
P36066	Protein MRG3-like [OS=Saccharomyces cerevisiae S288c]	853725	YKL133C; YKL133C	metabolic process	membrane;mitochondrion	protein binding	1.054	0.433	0.41
P07246	Alcohol dehydrogenase 3, mitochondrial [OS=Saccharomyces cerevisiae S288c]	855107	ADH3; YMR083W	metabolic process	mitochondrion;organelle lumen	catalytic activity;metal ion binding	10.159	4.179	0.41
P35180	mitochondrial import receptor subunit Tom20 [OS=Saccharomyces cerevisiae S288c]	852973	TOM20; YGR082W	cell organization and biogenesis;transport	membrane;mitochondrion	protein binding;transporter activity	1.031	0.425	0.41
Q03799	37S ribosomal protein S8, mitochondrial [OS=Saccharomyces cerevisiae S288c]	855190	MRPS8; YMR158W	cell organization and biogenesis;metabolic process	mitochondrion;ribosome	structural molecule activity	1.031	0.425	0.41
P46367	Potassium-activated aldehyde dehydrogenase, mitochondrial [OS=Saccharomyces cerevisiae S288c]	854556	ALD4; YOR374W	metabolic process	mitochondrion;organelle lumen	catalytic activity	47.497	19.62	0.41
P43617	Uncharacterized mitochondrial carrier YFR045W [OS=Saccharomyces cerevisiae S288c]	850606	YFR045W; YFR045W	metabolic process;transport	membrane;mitochondrion	structural molecule activity;transporter activity	0.995	0.413	0.42
P32796-1	Carnitine O-acetyltransferase, mitochondrial [OS=Saccharomyces cerevisiae S288c]	854965	CAT2; YML042W	metabolic process;transport	membrane;mitochondrion	catalytic activity	13.816	5.749	0.42
P36101	tRNA threonylcarbamoyladenosine dehydratase 2 [OS=Saccharomyces cerevisiae S288c]	853841	TCD2; YKL027W; YKL027W	metabolic process	cytosol;membrane;mitochondrion	catalytic activity;nucleotide binding	0.778	0.334	0.43
Q07914	mitochondrial import inner membrane translocase subunit TIM14 [OS=Saccharomyces cerevisiae S288c]	850694	PAM18; YLR008C	regulation of biological process;transport	membrane;mitochondrion	enzyme regulator activity;protein binding;transporter activity	0.778	0.334	0.43
P05626	ATP synthase subunit 4, mitochondrial [OS=Saccharomyces cerevisiae S288c]	856027	ATP4; YPL078C	cell organization and biogenesis;metabolic process;transport	membrane;mitochondrion	catalytic activity;transporter activity	4.995	2.162	0.43
P40159	Uncharacterized protein YNL208W [OS=Saccharomyces cerevisiae S288c]	855513	YNL208W; YNL208W		membrane;mitochondrion;ribosome		0.668	0.292	0.44
P36531	54S ribosomal protein L36, mitochondrial [OS=Saccharomyces cerevisiae S288c]	852419	MRPL36; YBR122C	cell organization and biogenesis;metabolic process	mitochondrion;ribosome	structural molecule activity	0.668	0.292	0.44
Q04458	Fatty aldehyde dehydrogenase HFD1 [OS=Saccharomyces cerevisiae S288c]	855137	HFD1; YMR110C	metabolic process	endoplasmic reticulum;endosome;membrane;mitochondrion	catalytic activity	4.055	1.783	0.44
P38325	Mitochondrial outer membrane protein OM14 [OS=Saccharomyces cerevisiae S288c]	852531	OM14; YBR230C	transport	membrane;mitochondrion		6.197	2.728	0.44
P32317	Protein AFG1 [OS=Saccharomyces cerevisiae S288c]	856658	AFG1; YEL052W	cell organization and biogenesis;metabolic process;response to stimulus;transport	membrane;mitochondrion	catalytic activity;nucleotide binding	1.326	0.585	0.44
P53140	Protein RMD9, mitochondrial [OS=Saccharomyces cerevisiae S288c]	852771	RMD9; YGL107C	cell differentiation;metabolic process;regulation of biological process	membrane;mitochondrion	RNA binding	1.512	0.668	0.44

P40513	Mitochondrial acidic protein MAM33 [OS=Saccharomyces cerevisiae S288c]	854740	MAM33; YIL070C	metabolic process;regulation of biological process	mitochondrion;organelle lumen	translation regulator activity	3.642	1.61	0.44
P40581	peroxiredoxin HYR1 [OS=Saccharomyces cerevisiae S288c]	854855	HYR1; YIR037W	metabolic process;response to stimulus	cytoplasm;cytosol;mitochondrion;organelle lumen	antioxidant activity;catalytic activity	0.585	0.259	0.44
P40530	[Pyruvate dehydrogenase (acetyl-transferring)] kinase 1, mitochondrial [OS=Saccharomyces cerevisiae S288c]	854769	PKP1; YIL042C	metabolic process	mitochondrion;organelle lumen	catalytic activity;nucleotide binding;protein binding	0.551	0.245	0.44
P40086	Cytochrome c oxidase assembly protein cox15 [OS=Saccharomyces cerevisiae S288c]	856884	COX15; YER141W	cell organization and biogenesis;metabolic process;transport	membrane;mitochondrion	catalytic activity;transporter activity	2.594	1.154	0.44
P23641	mitochondrial phosphate carrier protein [OS=Saccharomyces cerevisiae S288c]	853540	MIR1; YJR077C	metabolic process;transport	membrane;mitochondrion	protein binding;structural molecule activity;transporter activity	30.623	13.678	0.45
P10507	mitochondrial-processing peptidase subunit beta [OS=Saccharomyces cerevisiae S288c]	850860	MAS1; YLR163C	metabolic process;transport	mitochondrion;organelle lumen	catalytic activity;metal ion binding;protein binding	1.371	0.616	0.45
P53312	Succinyl-CoA ligase [ADP-forming] subunit beta, mitochondrial [OS=Saccharomyces cerevisiae S288c]	853159	LSC2; YGR244C	metabolic process	mitochondrion	catalytic activity;nucleotide binding	20.017	9	0.45
P40858	54S ribosomal protein L49, mitochondrial [OS=Saccharomyces cerevisiae S288c]	853349	MRPL49; YJL096W	cell organization and biogenesis;metabolic process	mitochondrion;ribosome	RNA binding;structural molecule activity	0.468	0.212	0.45
P34224	Uncharacterized protein YBL059W [OS=Saccharomyces cerevisiae S288c]	852221	YBL059W; YBL059W		membrane;mitochondrion		0.468	0.212	0.45
Q03020	iron sulfur cluster assembly protein 1, mitochondrial [OS=Saccharomyces cerevisiae S288c]	855968	ISU1; YPL135W	cell organization and biogenesis;cellular homeostasis;metabolic process	cytoplasm;mitochondrion;organelle lumen	catalytic activity;metal ion binding;protein binding	0.468	0.212	0.45
P32839	Mitochondrial chaperone BCS1 [OS=Saccharomyces cerevisiae S288c]	851981	BCS1; YDR375C	cell organization and biogenesis;metabolic process;response to stimulus;transport	cytosol;membrane;mitochondrion	catalytic activity;nucleotide binding;transporter activity	0.425	0.194	0.46
Q01976	ADP-ribose pyrophosphatase [OS=Saccharomyces cerevisiae S288c]	852408	YSA1; YBR111C	metabolic process	cytoplasm;mitochondrion;nucleus	catalytic activity;metal ion binding	0.425	0.194	0.46
P34222	Peptidyl-tRNA hydrolase 2 [OS=Saccharomyces cerevisiae S288c]	852223	PTH2; YBL057C	regulation of biological process	cytoplasm;cytosol;membrane;mitochondrion	catalytic activity;protein binding	0.425	0.194	0.46
Q04487	Mitochondrial inner membrane protein SHH3 [OS=Saccharomyces cerevisiae S288c]	855145	SHH3; YMR118C	metabolic process	membrane;mitochondrion	catalytic activity;metal ion binding	0.425	0.194	0.46
P36523	54S ribosomal protein L15, mitochondrial [OS=Saccharomyces cerevisiae S288c]	851022	MRPL15; YLR312W-A	cell organization and biogenesis;metabolic process	mitochondrion;ribosome	catalytic activity;RNA binding;structural molecule activity	0.334	0.155	0.46
P36519	54S ribosomal protein L7, mitochondrial [OS=Saccharomyces cerevisiae S288c]	851823	MRPL7; YDR237W	cell organization and biogenesis;metabolic process	mitochondrion;ribosome	RNA binding;structural molecule activity	0.292	0.136	0.47
Q05931	Heat shock protein SSQ1, mitochondrial [OS=Saccharomyces cerevisiae S288c]	851084	SSQ1; YLR369W	cell organization and biogenesis;metabolic process	mitochondrion;organelle lumen	nucleotide binding;protein binding	0.884	0.413	0.47
P00560	Phosphoglycerate kinase [OS=Saccharomyces cerevisiae S288c]	850370	PGK1; YCR012W	metabolic process	cytoplasm;membrane;mitochondrion	catalytic activity;nucleotide binding	6.356	2.981	0.47
P40098	uncharacterized mitochondrial membrane protein FMP10 [OS=Saccharomyces cerevisiae S288c]	856931	FMP10; YER182W		membrane;mitochondrion		5.952	2.793	0.47
Q08968	UPF0061 protein FMP40 [OS=Saccharomyces cerevisiae S288c]	855879	FMP40; YPL222W		mitochondrion	protein binding	2.162	1.015	0.47
P07143	Cytochrome c1, heme protein, mitochondrial [OS=Saccharomyces cerevisiae S288c]	854231	CYT1; YOR065W	metabolic process;transport	cytosol;membrane;mitochondrion	metal ion binding	23.245	10.938	0.47
P39722	Mitochondrial Rho GTPase 1 [OS=Saccharomyces cerevisiae S288c]	851249	GEM1; YAL048C	cell organization and biogenesis;regulation of biological process;response to stimulus;transport	membrane;mitochondrion	catalytic activity;metal ion binding;nucleotide binding	0.259	0.122	0.47
P18496	Mitochondrial ATPase complex subunit ATP10	851109	ATP10; YLR393W	cell organization and biogenesis	cytoplasm;membrane;mitochondrion	protein binding	1.239	0.585	0.47

	[OS=Saccharomyces cerevisiae S288c]								
P29704	squalene synthase [OS=Saccharomyces cerevisiae S288c]	856597	ERG9; YHR190W	metabolic process	endoplasmic reticulum;membrane;mitochondrion	catalytic activity	0.222	0.105	0.47
P43122	tRNA N6-adenosine threonylcarbamoyltransferase, mitochondrial [OS=Saccharomyces cerevisiae S288c]	851454	QRI7; YDL104C	cell organization and biogenesis;metabolic process	mitochondrion	catalytic activity;metal ion binding	0.212	0.101	0.48
P36046	Mitochondrial intermembrane space import and assembly protein 40 [OS=Saccharomyces cerevisiae S288c]	853639	MIA40; YKL195W	cellular component movement;metabolic process;response to stimulus;transport	membrane;mitochondrion	catalytic activity;protein binding;transporter activity	3.262	1.555	0.48
P38072	Protein SCO2, mitochondrial [OS=Saccharomyces cerevisiae S288c]	852312	SCO2; YBR024W	cell organization and biogenesis;cellular homeostasis;metabolic process;transport	membrane;mitochondrion	antioxidant activity;catalytic activity;metal ion binding	1.154	0.551	0.48
P53311	Mitochondrial pyruvate carrier 3 [OS=Saccharomyces cerevisiae S288c]	853158	FMP43; MPC3; YGR243W	transport	membrane;mitochondrion	transporter activity	1.61	0.778	0.48
Q08818	Meiotic sister-chromatid recombination protein 6, mitochondrial [OS=Saccharomyces cerevisiae S288c]	854536	MSC6; YOR354C	metabolic process	mitochondrion;organelle lumen	RNA binding	1.047	0.506	0.48
P39925	Mitochondrial respiratory chain complexes assembly protein AFG3 [OS=Saccharomyces cerevisiae S288c]	856737	AFG3; YER017C	cell organization and biogenesis;metabolic process;response to stimulus;transport	membrane;mitochondrion	catalytic activity;metal ion binding;nucleotide binding;protein binding	1.342	0.65	0.48
Q02486	ARS-binding factor 2, mitochondrial [OS=Saccharomyces cerevisiae S288c]	855094	ABF2; YMR072W	cell organization and biogenesis;metabolic process;regulation of biological process	chromosome;mitochondrion	DNA binding	69.17	33.551	0.49
P25039	Elongation factor G, mitochondrial [OS=Saccharomyces cerevisiae S288c]	850758	MEF1; YLR069C	cell organization and biogenesis;metabolic process	cytosol;mitochondrion	catalytic activity;nucleotide binding;RNA binding	0.931	0.456	0.49
P32473	Pyruvate dehydrogenase E1 component subunit beta, mitochondrial [OS=Saccharomyces cerevisiae S288c]	852522	PDB1; YBR221C	metabolic process	mitochondrion;organelle lumen	catalytic activity	9	4.456	0.50
P32787	Mitochondrial genome maintenance protein MGM101 [OS=Saccharomyces cerevisiae S288c]	853609	MGM101; YJR144W	cell organization and biogenesis;metabolic process;response to stimulus	chromosome;mitochondrion	DNA binding	3.281	1.637	0.50
P33421	Succinate dehydrogenase [ubiquinone] cytochrome b subunit, mitochondrial [OS=Saccharomyces cerevisiae S288c]	853716	SDH3; YKL141W	cell organization and biogenesis;metabolic process;transport	membrane;mitochondrion	catalytic activity;metal ion binding	1.276	0.638	0.50
P32898	Mitochondrial presequence protease [OS=Saccharomyces cerevisiae S288c]	852041	CYM1; YDR430C	metabolic process	mitochondrion	catalytic activity;metal ion binding	1.349	0.682	0.51
P00358	glyceraldehyde-3-phosphate dehydrogenase 2 [OS=Saccharomyces cerevisiae S288c]	853465	TDH2; YJR009C	cell death;metabolic process	cytoplasm;cytosol;membrane;mitochondrion;nucleus	catalytic activity;nucleotide binding;protein binding	3.962	2.008	0.51
P53598	Succinyl-CoA ligase [ADP-forming] subunit alpha, mitochondrial [OS=Saccharomyces cerevisiae S288c]	854310	LSC1; YOR142W	metabolic process	cytosol;mitochondrion	catalytic activity;nucleotide binding	11.743	5.952	0.51
Q12480	Probable electron transfer flavoprotein subunit alpha, mitochondrial [OS=Saccharomyces cerevisiae S288c]	856112	AIM45; YPR004C	metabolic process;transport	mitochondrion;organelle lumen	catalytic activity;nucleotide binding	2.981	1.512	0.51
P32266	Dynamin-like GTPase MGM1, mitochondrial [OS=Saccharomyces cerevisiae S288c]	854386	MGM1; YOR211C	cell organization and biogenesis	membrane;mitochondrion	catalytic activity;nucleotide binding;protein binding	0.448	0.228	0.51
P50085	Prohibitin-2 [OS=Saccharomyces cerevisiae S288c]	853146	PHB2; YGR231C	cell organization and biogenesis;metabolic process;regulation of biological process	membrane;mitochondrion	protein binding	4.926	2.511	0.51
P32843	Mitochondrial escape protein 2 [OS=Saccharomyces cerevisiae S288c]	855348	YME2; YMR302C	cell organization and biogenesis;metabolic process	membrane;mitochondrion	nucleotide binding;RNA binding	5.158	2.643	0.51
Q02776	Mitochondrial import inner membrane translocase subunit TIM50 [OS=Saccharomyces cerevisiae S288c]	856042	TIM50; YPL063W	cell organization and biogenesis;metabolic process;transport	membrane;mitochondrion	catalytic activity;protein binding;transporter activity	9	4.623	0.51
P38127	mitochondrial carrier protein RIM2 [OS=Saccharomyces	852491	RIM2; YBR192W	cell organization and biogenesis;metabolic process;transport	membrane;mitochondrion	structural molecule	1.512	0.778	0.51

	cerevisiae S288c]					activity;transporter activity			
P32335	Protein MSS51, mitochondrial [OS=Saccharomyces cerevisiae S288c]	850900	MSS51; YLR203C	metabolic process;regulation of biological process	membrane;mitochondrion	protein binding;translation regulator activity	1.929	0.995	0.52
P53889	uncharacterized mitochondrial hydrolase FMP41 [OS=Saccharomyces cerevisiae S288c]	855553	FMP41; YNL168C	metabolic process	mitochondrion	catalytic activity;metal ion binding	0.968	0.501	0.52
P46681	D-lactate dehydrogenase [cytochrome] 2, mitochondrial [OS=Saccharomyces cerevisiae S288c]	851376	DLD2; YDL178W	metabolic process	mitochondrion;organelle lumen	catalytic activity;nucleotide binding;protein binding	1.829	0.951	0.52
P36151	Uncharacterized protein YKR070W [OS=Saccharomyces cerevisiae S288c]	853944	YKR070W ; YKR070W	metabolic process	mitochondrion		0.833	0.438	0.53
P40495	Homoisocitrate dehydrogenase, mitochondrial [OS=Saccharomyces cerevisiae S288c]	854714	LYS12; YIL094C	metabolic process	mitochondrion	catalytic activity;metal ion binding;nucleotide binding	4.109	2.162	0.53
P07256	Cytochrome b-c1 complex subunit 1, mitochondrial [OS=Saccharomyces cerevisiae S288c]	852235	COR1; YBL045C	metabolic process;transport	membrane;mitochondrion	catalytic activity;metal ion binding;protein binding;transporter activity	18.783	9.89	0.53
P43635	Citrate synthase 3, mitochondrial [OS=Saccharomyces cerevisiae S288c]	856107	CIT3; YPR001W	metabolic process	mitochondrion	catalytic activity	5.529	2.914	0.53
P36534	54S ribosomal protein L40, mitochondrial [OS=Saccharomyces cerevisiae S288c]	855930	MRPL40; YPL173W	cell organization and biogenesis;metabolic process	mitochondrion;ribosome	protein binding;structural molecule activity	0.778	0.413	0.53
P33759	37S ribosomal protein S5, mitochondrial [OS=Saccharomyces cerevisiae S288c]	852553	MRPS5; YBR251W	cell organization and biogenesis;metabolic process	mitochondrion;ribosome	RNA binding;structural molecule activity	0.778	0.413	0.53
P36163	mitochondrial metalloendopeptidase OMA1 [OS=Saccharomyces cerevisiae S288c]	853962	OMA1; YKR087C	metabolic process	membrane;mitochondrion	catalytic activity;metal ion binding	0.73	0.389	0.53
P32191	Glycerol-3-phosphate dehydrogenase, mitochondrial [OS=Saccharomyces cerevisiae S288c]	854651	GUT2; YIL155C	metabolic process	membrane;mitochondrion	catalytic activity	8.522	4.555	0.53
P38797	Protein phosphatase 2C homolog 7, mitochondrial [OS=Saccharomyces cerevisiae S288c]	856475	PTC7; YHR076W	metabolic process	mitochondrion	catalytic activity;metal ion binding;protein binding	0.688	0.369	0.54
Q12031	Mitochondrial 2-methylisocitrate lyase [OS=Saccharomyces cerevisiae S288c]	856114	ICL2; YPR006C	metabolic process	mitochondrion;organelle lumen	catalytic activity	5.158	2.793	0.54
P38088	metabolic process	cytoplasm;mitochondrion;organelle lumen	catalytic activity;nucleotide binding;protein binding	0.616	0.334	0.54			
P32902	37S ribosomal protein MRP4, mitochondrial [OS=Saccharomyces cerevisiae S288c]	856384	MRP4; YHL004W	cell organization and biogenesis;metabolic process	mitochondrion;ribosome	structural molecule activity	0.616	0.334	0.54
P32316	acetyl-CoA hydrolase [OS=Saccharomyces cerevisiae S288c]	852266	ACH1; YBL015W	metabolic process	cytoplasm;cytosol;mitochondrion	catalytic activity	15.298	8.326	0.54
P35191	DnaJ homolog 1, mitochondrial [OS=Saccharomyces cerevisiae S288c]	850530	MDJ1; YFL016C	cell organization and biogenesis;metabolic process;regulation of biological process;response to stimulus	mitochondrion;organelle lumen	enzyme regulator activity;metal ion binding;nucleotide binding;protein binding	3.037	1.656	0.55
P38071	Enoyl-[acyl-carrier protein] reductase [NADPH, B-specific], mitochondrial [OS=Saccharomyces cerevisiae S288c]	852314	ETR1; YBR026C	metabolic process	mitochondrion;organelle lumen	catalytic activity;DNA binding;metal ion binding	9	4.926	0.55
P32795	Mitochondrial inner membrane i-AAA protease supercomplex subunit YME1 [OS=Saccharomyces cerevisiae S288c]	856135	YME1; YPR024W	metabolic process;response to stimulus;transport	membrane;mitochondrion	catalytic activity;metal ion binding;nucleotide binding;protein binding	1.798	0.986	0.55
P00359	Glyceraldehyde-3-phosphate dehydrogenase 3	853106	TDH3; YGR192C	cell death;metabolic process	cytoplasm;cytosol;membrane;mitochondrion;nucleus	catalytic activity;nucleo	7.185	3.962	0.55

	[OS=Saccharomyces cerevisiae S288c]					tide binding;RNA binding			
P40582	Glutathione S-transferase 1 [OS=Saccharomyces cerevisiae S288c]	854856	GTT1; YIR038C	metabolic process	endoplasmic reticulum;membrane;mitochondrion;nucleus	antioxidant activity;catalytic activity;protein binding;RNA binding	1.683	0.931	0.55
P50087	MICOS subunit MIC26 [OS=Saccharomyces cerevisiae S288c]	853150	MOS2; MIC26; YGR235C	cell organization and biogenesis	membrane;mitochondrion		1.683	0.931	0.55
P39987	Heat shock protein SSC3, mitochondrial [OS=Saccharomyces cerevisiae S288c]	856682	ECM10; YEL030W	cell organization and biogenesis;metabolic process;transport	mitochondrion	nucleotide binding;protein binding	2.162	1.198	0.55
P15108	ATP-dependent molecular chaperone HSC82 [OS=Saccharomyces cerevisiae S288c]	855224	HSC82; YMR186W	cell organization and biogenesis;metabolic process;response to stimulus	cytoplasm;membrane;mitochondrion	catalytic activity;nucleotide binding;protein binding	1.081	0.602	0.56
P40364	Mitochondrial peculiar membrane protein 1 [OS=Saccharomyces cerevisiae S288c]	853379	MPM1; YJL066C		membrane;mitochondrion		1.512	0.848	0.56
P37299	cytochrome b-c1 complex subunit 10 [OS=Saccharomyces cerevisiae S288c]	856390	QCR10; YHR001W-A	metabolic process;transport	membrane;mitochondrion	catalytic activity;transporter activity	5.31	2.981	0.56
Q12233	ATP synthase subunit g, mitochondrial [OS=Saccharomyces cerevisiae S288c]	856131	ATP20; YPR020W	cell organization and biogenesis;metabolic process;transport	membrane;mitochondrion	catalytic activity;transporter activity	1.371	0.778	0.57
P36147	Presequence translocated-associated motor subunit pam17, mitochondrial [OS=Saccharomyces cerevisiae S288c]	853939	PAM17; YKR065C	transport	membrane;mitochondrion		1.371	0.778	0.57
Q06485	Autophagy-related protein 33 [OS=Saccharomyces cerevisiae S288c]	851070	ATG33; YLR356W	cell communication;metabolic process;response to stimulus	membrane;mitochondrion		1.371	0.778	0.57
P17505	Malate dehydrogenase, mitochondrial [OS=Saccharomyces cerevisiae S288c]	853777	MDH1; YKL085W	metabolic process	mitochondrion;organelle lumen	catalytic activity;protein binding;RNA binding	56.797	32.405	0.57
P42940	Probable electron transfer flavoprotein subunit beta [OS=Saccharomyces cerevisiae S288c]	853121	CIR1; YGR207C	metabolic process;transport	mitochondrion;organelle lumen		4.623	2.652	0.57
P16387	Pyruvate dehydrogenase E1 component subunit alpha, mitochondrial [OS=Saccharomyces cerevisiae S288c]	856925	PDA1; YER178W	cell growth;metabolic process	mitochondrion;organelle lumen	catalytic activity	21.638	12.46	0.58
P36775	Lon protease homolog, mitochondrial [OS=Saccharomyces cerevisiae S288c]	852259	PIM1; YBL022C	cell organization and biogenesis;metabolic process;regulation of biological process;response to stimulus	cytoplasm;mitochondrion;organelle lumen	catalytic activity;DNA binding;nucleotide binding;RNA binding	1.362	0.792	0.58
P04710	ADP,ATP carrier protein 1 [OS=Saccharomyces cerevisiae S288c]	855078	AAC1; YMR056C	metabolic process;transport	cytosol;membrane;mitochondrion	structural molecule activity;transporter activity	11.743	6.848	0.58
P28834	Isocitrate dehydrogenase [NAD] subunit 1, mitochondrial [OS=Saccharomyces cerevisiae S288c]	855691	IDH1; YNL037C	metabolic process;transport	cytosol;mitochondrion;organelle lumen	catalytic activity;metal ion binding;nucleotide binding;protein binding;RNA binding	15.238	9	0.59
P36527	54S ribosomal protein L28, mitochondrial [OS=Saccharomyces cerevisiae S288c]	852073	MRPL28; YDR462W	cell organization and biogenesis;metabolic process	mitochondrion;ribosome	structural molecule activity	0.874	0.52	0.59
P16622	Ferrochelatase, mitochondrial [OS=Saccharomyces cerevisiae S288c]	854347	HEM15; YOR176W	metabolic process	membrane;mitochondrion	catalytic activity	6.305	3.806	0.60
P40012	protoporphyrinogen oxidase [OS=Saccharomyces cerevisiae S288c]	856733	HEM14; YER014W	metabolic process	membrane;mitochondrion	catalytic activity	2.162	1.336	0.62
P32453	Protein ATP11, mitochondrial [OS=Saccharomyces cerevisiae S288c]	855401	ATP11; YNL315C	cell organization and biogenesis	mitochondrion	protein binding	1.239	0.778	0.63
P40215	External NADH-ubiquinone oxidoreductase 1, mitochondrial [OS=Saccharomyces cerevisiae S288c]	855176	NDE1; YMR145C	metabolic process	mitochondrion	catalytic activity;nucleotide binding	5.469	3.453	0.63
Q08822	Probable electron transfer flavoprotein-ubiquinone oxidoreductase, mitochondrial	854538	CIR2; YOR356W	metabolic process;transport	membrane;mitochondrion	catalytic activity;metal ion	3.424	2.162	0.63

	[OS=Saccharomyces cerevisiae S288c]					binding;nucleo tide binding			
P54857	lipase 2 [OS=Saccharomyces cerevisiae S288c]	85162 8	TGL2; YDR058C	metabolic process;transport	mitochondrion	catalytic activity	0.369	0.233	0.63
P51998	54S ribosomal protein yml6, mitochondrial [OS=Saccharomyces cerevisiae S288c]	85498 3	YML6; YML025C	cell organization and biogenesis;metabolic process	mitochondrion;ribosome	RNA binding;structu ral molecule activity	1.154	0.73	0.63
P33310	ATP-dependent permease MDL1, mitochondrial [OS=Saccharomyces cerevisiae S288c]	85088 5	MDL1; YLR188W	transport	membrane;mitochondrion	catalytic activity;nucleo tide binding;transp orter activity	0.833	0.528	0.63
Q05648	MIOREX complex component 10 [OS=Saccharomyces cerevisiae S288c]	85187 6	YDR282C; MRX10; YDR282C		membrane;mitochondrion		0.35	0.222	0.63
P47015	Altered inheritance of mitochondria protein 23, mitochondrial [OS=Saccharomyces cerevisiae S288c]	85331 0	AIM23; YJL131C	metabolic process	mitochondrion	RNA binding	0.35	0.222	0.63
P28817	3-hydroxyisobutyryl-CoA hydrolase, mitochondrial [OS=Saccharomyces cerevisiae S288c]	85160 6	EHD3; YDR036C	metabolic process	mitochondrion	catalytic activity	1.395	0.887	0.64
Q01532-1	Cysteine proteinase 1, mitochondrial [OS=Saccharomyces cerevisiae S288c]		MCY1	metabolic process;regulation of biological process;response to stimulus	cytoplasm;mitochondrion	catalytic activity;DNA binding;RNA binding	0.28	0.179	0.64
P03879	Intron-encoded RNA maturase bI4 [OS=Saccharomyces cerevisiae S288c]	85458 2	BI4; Q0120	metabolic process	membrane;mitochondrion	catalytic activity;RNA binding	0.28	0.179	0.64
P17967	Protein disulfide-isomerase [OS=Saccharomyces cerevisiae S288c]	85031 4	PDI1; YCL043C	cellular homeostasis;metabolic process;regulation of biological process;response to stimulus	endoplasmic reticulum;organelle lumen	catalytic activity;protein binding	1.326	0.848	0.64
P10849	mitochondrial transcription factor 2 [OS=Saccharomyces cerevisiae S288c]	85151 7	MTF2; YDL044C	metabolic process	mitochondrion;organelle lumen	protein binding;RNA binding	0.259	0.166	0.64
Q03557	Glutamyl-tRNA(Gln) amidotransferase subunit A, mitochondrial [OS=Saccharomyces cerevisiae S288c]	85533 8	HER2; YMR293C	cell organization and biogenesis;metabolic process	mitochondrion	catalytic activity;nucleo tide binding	0.259	0.166	0.64
P38705	Serine--tRNA ligase, mitochondrial [OS=Saccharomyces cerevisiae S288c]	85640 2	DIA4; YHR011 W	metabolic process	cytoplasm;mitochondrion;organelle lumen	catalytic activity;nucleo tide binding	0.233	0.15	0.64
P23180	Probable oxidoreductase AIM17 [OS=Saccharomyces cerevisiae S288c]	85636 5	AIM17; YHL021C	cell organization and biogenesis;metabolic process	mitochondrion	catalytic activity;metal ion binding	4.623	2.981	0.64
P53732	37S ribosomal protein S12, mitochondrial [OS=Saccharomyces cerevisiae S288c]	85577 2	MRPS12; YNR036C	cell organization and biogenesis;metabolic process	mitochondrion;ribosome	structural molecule activity	1.783	1.154	0.65
P39677	Ribosome-releasing factor 2, mitochondrial [OS=Saccharomyces cerevisiae S288c]	85334 2	MEF2; YJL102W	cell organization and biogenesis;metabolic process	cytosol;mitochondrion	catalytic activity;nucleo tide binding;RNA binding	0.179	0.116	0.65
P39006	Phosphatidylserine decarboxylase proenzyme 1, mitochondrial [OS=Saccharomyces cerevisiae S288c]	85555 2	PSD1; YNL169C	metabolic process;regulation of biological process	membrane;mitochondrion	catalytic activity	0.743	0.487	0.66
P53969	Sorting assembly machinery 50 kDa subunit [OS=Saccharomyces cerevisiae S288c]	85570 5	SAM50; YNL026W	cell organization and biogenesis;transport	membrane;mitochondrion	protein binding;transp orter activity	0.743	0.487	0.66
Q04728	Arginine biosynthesis bifunctional protein ArgJ, mitochondrial [OS=Saccharomyces cerevisiae S288c]	85508 4	ARG7; YMR062C	metabolic process	mitochondrion;organelle lumen	catalytic activity	0.63	0.417	0.66
Q06143	mitochondrial dicarboxylate transporter [OS=Saccharomyces cerevisiae S288c]	85106 3	DIC1; YLR348C	metabolic process;transport	membrane;mitochondrion	structural molecule activity;transp orter activity	1.404	0.931	0.66
P23369	54S ribosomal protein L25, mitochondrial [OS=Saccharomyces cerevisiae S288c]	85296 7	MRPL25; YGR076C	cell organization and biogenesis;metabolic process	mitochondrion;ribosome	structural molecule activity	1.31	0.874	0.67
P38910	10 kDa heat shock protein, mitochondrial [OS=Saccharomyces cerevisiae S288c]	85418 5	HSP10; YOR020C	cell organization and biogenesis;metabolic process;response to stimulus;transport	cytoplasm;mitochondrion;organelle lumen	metal ion binding;nucleo tide binding;protei n binding	3.217	2.162	0.67
P07806	Valine--tRNA ligase, mitochondrial [OS=Saccharomyces cerevisiae S288c]	85298 6	VAS1; YGR094 W	metabolic process;regulation of biological process	cytoplasm;cytosol;mitochondrion	catalytic activity;nucleo tide binding	0.616	0.422	0.69

P16451	Pyruvate dehydrogenase complex protein X component, mitochondrial [OS=Saccharomyces cerevisiae S288c]	853107	PDX1; YGR193C	metabolic process	mitochondrion;organelle lumen	catalytic activity;structural molecule activity	2.594	1.783	0.69
P38969	Pentamidine resistance factor, mitochondrial [OS=Saccharomyces cerevisiae S288c]	854440	PNT1; YOR266W	cell organization and biogenesis;response to stimulus	membrane;mitochondrion		0.848	0.585	0.69
P49095	Glycine dehydrogenase (Decarboxylating), mitochondrial [OS=Saccharomyces cerevisiae S288c]	855227	GCV2; YMR189W	metabolic process	cytosol;mitochondrion	catalytic activity	2.433	1.683	0.69
P07257	Cytochrome b-c1 complex subunit 2, mitochondrial [OS=Saccharomyces cerevisiae S288c]	856321	QCR2; YPR191W	metabolic process;transport	membrane;mitochondrion	catalytic activity;metal ion binding;protein binding;transporter activity	36.751	25.49	0.69
Q06668	Methyltransferase OMS1, mitochondrial [OS=Saccharomyces cerevisiae S288c]	851911	OMS1; YDR316W	metabolic process	membrane;mitochondrion	catalytic activity	0.693	0.484	0.70
P19262	Dihydrolipoyllysine-residue succinyltransferase component of 2-oxoglutarate dehydrogenase complex, mitochondrial [OS=Saccharomyces cerevisiae S288c]	851726	KGD2; YDR148C	cell organization and biogenesis;metabolic process	mitochondrion	catalytic activity;protein binding	6.406	4.484	0.70
P38891	Branched-chain-amino-acid aminotransferase, mitochondrial [OS=Saccharomyces cerevisiae S288c]	856615	BAT1; YHR208W	metabolic process;regulation of biological process;response to stimulus	mitochondrion;organelle lumen	catalytic activity	9	6.305	0.70
P11914	Mitochondrial-processing peptidase subunit alpha [OS=Saccharomyces cerevisiae S288c]	856419	MAS2; YHR024C	metabolic process;transport	membrane;mitochondrion;organelle lumen	catalytic activity;metal ion binding;protein binding	1.291	0.905	0.70
P38152	Tricarboxylate transport protein [OS=Saccharomyces cerevisiae S288c]	852594	CTP1; YBR291C	metabolic process;transport	membrane;mitochondrion	structural molecule activity;transporter activity	0.551	0.389	0.71
Q08179	Mitochondrial distribution and morphology protein 38 [OS=Saccharomyces cerevisiae S288c]	854130	MDM38; YOL027C	cell organization and biogenesis;cellular homeostasis;regulation of biological process;transport	membrane;mitochondrion		6.079	4.309	0.71
P38077	ATP synthase subunit gamma, mitochondrial [OS=Saccharomyces cerevisiae S288c]	852327	ATP3; YBR039W	metabolic process;transport	membrane;mitochondrion	catalytic activity;transporter activity	12.689	9	0.71
P36528	54S ribosomal protein L17, mitochondrial [OS=Saccharomyces cerevisiae S288c]	855469	MRPL17; YNL252C	cell organization and biogenesis;metabolic process	mitochondrion;ribosome	structural molecule activity	0.52	0.369	0.71
P48015	Aminomethyltransferase, mitochondrial [OS=Saccharomyces cerevisiae S288c]	851582	GCV1; YDR019C	metabolic process	mitochondrion	catalytic activity;protein binding	9	6.406	0.71
P38702	mitochondrial carrier protein LEU5 [OS=Saccharomyces cerevisiae S288c]	856391	LEU5; YHR002W	metabolic process;transport	membrane;mitochondrion	structural molecule activity;transporter activity	0.468	0.334	0.71
Q04172	Sensitive to high expression protein 9, mitochondrial [OS=Saccharomyces cerevisiae S288c]	852002	SHE9; YDR393W	cell organization and biogenesis	membrane;mitochondrion		0.374	0.269	0.72
P04807	Hexokinase-2 [OS=Saccharomyces cerevisiae S288c]	852639	HXK2; YGL253W	cell communication;cell organization and biogenesis;cellular homeostasis;metabolic process;regulation of biological process;response to stimulus;transport	cytosol;mitochondrion	catalytic activity;nucleotide binding	0.359	0.259	0.72
P32897	Mitochondrial import inner membrane translocase subunit tim23 [OS=Saccharomyces cerevisiae S288c]	855751	TIM23; YNR017W	transport	membrane;mitochondrion	protein binding;transporter activity	2.981	2.162	0.73
P37292	Serine hydroxymethyltransferase, mitochondrial [OS=Saccharomyces cerevisiae S288c]	852565	SHM1; YBR263W	metabolic process	mitochondrion	catalytic activity	9	6.565	0.73
P07213	Mitochondrial import receptor subunit TOM70 [OS=Saccharomyces cerevisiae S288c]	855602	TOM70; YNL121C	cell organization and biogenesis;transport	membrane;mitochondrion	protein binding;transporter activity	3.642	2.665	0.73
P54783	D-arabinono-1,4-lactone oxidase [OS=Saccharomyces cerevisiae S288c]	854888	ALO1; YML086C	metabolic process;response to stimulus	membrane;mitochondrion	catalytic activity;nucleotide binding	3.299	2.415	0.73
P43616	Cys-Gly metallodipeptidase dug1 [OS=Saccharomyces cerevisiae S288c]	850605	DUG1; YFR044C	metabolic process	cytoplasm;mitochondrion;ribosome	catalytic activity;metal ion binding	1.154	0.848	0.73

Q02204	54S ribosomal protein L13, mitochondrial [OS=Saccharomyces cerevisiae S288c]	853875	MRPL13; YKR006C	cell organization and biogenesis;metabolic process	mitochondrion;ribosome	structural molecule activity	1.054	0.778	0.74
P40047	Aldehyde dehydrogenase 5, mitochondrial [OS=Saccharomyces cerevisiae S288c]	856804	ALD5; YER073W	metabolic process	mitochondrion;organelle lumen	catalytic activity	2.384	1.762	0.74
P50945	MICOS complex subunit MIC27 [OS=Saccharomyces cerevisiae S288c]	855623	AIM37; MIC27; YNL100W	cell organization and biogenesis	membrane;mitochondrion		0.968	0.719	0.74
P28625	Protein YIM1 [OS=Saccharomyces cerevisiae S288c]	855183	YIM1; YMR152W	metabolic process;response to stimulus	cytoplasm;endoplasmic reticulum;mitochondrion	catalytic activity;metal ion binding	0.833	0.624	0.75
P53736	Uncharacterized protein YNR040W [OS=Saccharomyces cerevisiae S288c]	855776	YNR040W; YNR040W		mitochondrion		0.73	0.551	0.75
P36013	NAD-dependent malic enzyme, mitochondrial [OS=Saccharomyces cerevisiae S288c]	853839	MAE1; YKL029C	metabolic process	mitochondrion;organelle lumen	catalytic activity;metal ion binding;nucleotide binding	1.985	1.512	0.76
P36112	MICOS complex subunit Mic60 [OS=Saccharomyces cerevisiae S288c]	853886	FCJ1; MIC60; YKR016W	cell organization and biogenesis;transport	membrane;mitochondrion		15.379	11.798	0.77
P07251	ATP synthase subunit alpha, mitochondrial [OS=Saccharomyces cerevisiae S288c]	852177	ATP1; YBL099W	metabolic process;transport	cytosol;membrane;mitochondrion	catalytic activity;nucleotide binding;transporter activity	34.938	26.826	0.77
P32904	54S ribosomal protein L6, mitochondrial [OS=Saccharomyces cerevisiae S288c]	856552	MRPL6; YHR147C	cell organization and biogenesis;metabolic process	mitochondrion;ribosome	RNA binding;structural molecule activity	1.371	1.054	0.77
P0CS90	Heat shock protein SSC1, mitochondrial [OS=Saccharomyces cerevisiae S288c]	853503	SSC1; YJR045C	cell organization and biogenesis;metabolic process;regulation of biological process;transport	membrane;mitochondrion;nucleus;organelle lumen	catalytic activity;enzyme regulator activity;nucleotide binding;protein binding	48.482	37.312	0.77
P36060	NADH-cytochrome b5 reductase 2 [OS=Saccharomyces cerevisiae S288c]	853707	MCR1; YKL150W	metabolic process;response to stimulus	membrane;mitochondrion	catalytic activity;protein binding	15.238	11.743	0.77
P38771	Ribosome-recycling factor, mitochondrial [OS=Saccharomyces cerevisiae S288c]	856433	RRF1; YHR038W	cell organization and biogenesis;metabolic process	mitochondrion	RNA binding	1.254	0.968	0.77
P23644	Mitochondrial import receptor subunit TOM40 [OS=Saccharomyces cerevisiae S288c]	855243	TOM40; YMR203W	transport	cytosol;membrane;mitochondrion	protein binding;transporter activity	1.154	0.896	0.78
P36521	54S ribosomal protein L11, mitochondrial [OS=Saccharomyces cerevisiae S288c]	851325	MRPL11; YDL202W	cell organization and biogenesis;metabolic process	mitochondrion;ribosome	structural molecule activity	1.069	0.833	0.78
Q00711	Succinate dehydrogenase [ubiquinone] flavoprotein subunit, mitochondrial [OS=Saccharomyces cerevisiae S288c]	853709	SDH1; YKL148C	metabolic process;transport	membrane;mitochondrion	catalytic activity;nucleotide binding;protein binding	12.242	9.578	0.78
Q08023	Protein FMP25, mitochondrial [OS=Saccharomyces cerevisiae S288c]	850766	FMP25; YLR077W	cell organization and biogenesis	membrane;mitochondrion		1.412	1.106	0.78
P48526	Isoleucine--tRNA ligase, mitochondrial [OS=Saccharomyces cerevisiae S288c]	856067	ISM1; YPL040C	cell organization and biogenesis;metabolic process;regulation of biological process	cytoplasm;cytosol;mitochondrion;organelle lumen	catalytic activity;nucleotide binding	0.215	0.169	0.79
P32611	54S ribosomal protein RML2, mitochondrial [OS=Saccharomyces cerevisiae S288c]	856660	RML2; YEL050C	cell organization and biogenesis;metabolic process	mitochondrion;ribosome	catalytic activity;RNA binding;structural molecule activity	0.638	0.509	0.80
Q06493	LETM1 domain-containing protein YLH47, mitochondrial [OS=Saccharomyces cerevisiae S288c]	856243	YLH47; YPR125W	cell organization and biogenesis;transport	membrane;mitochondrion		0.61	0.487	0.80
Q08223	Altered inheritance of mitochondria protein 39, mitochondrial [OS=Saccharomyces cerevisiae S288c]	854103	AIM39; YOL053W		cytosol;membrane;mitochondrion		0.585	0.468	0.80
P48527	Tyrosine--tRNA ligase, mitochondrial [OS=Saccharomyces cerevisiae S288c]	856007	MSY1; YPL097W	metabolic process	cytoplasm;cytosol;mitochondrion;organelle lumen	catalytic activity;nucleotide binding;RNA binding	0.52	0.417	0.80
P38323	ATP-dependent clpX-like chaperone, mitochondrial [OS=Saccharomyces cerevisiae	852528	MCX1; YBR227C	metabolic process	membrane;mitochondrion;organelle lumen	catalytic activity;nucleotide binding	0.501	0.403	0.80

	S288c]								
P53881	54S ribosomal protein L22, mitochondrial [OS=Saccharomyces cerevisiae S288c]	855544	MRPL22; YNL177C	cell organization and biogenesis;metabolic process	mitochondrion;ribosome	RNA binding;structural molecule activity	1.154	0.931	0.81
P33311	ATP-dependent permease MDL2, mitochondrial [OS=Saccharomyces cerevisiae S288c]	855858	MDL2; YPL270W	response to stimulus;transport	membrane;mitochondrion	catalytic activity;nucleotide binding;transporter activity	1.116	0.901	0.81
Q03976	37S ribosomal protein S24, mitochondrial [OS=Saccharomyces cerevisiae S288c]	851755	RSM24; YDR175C	cell organization and biogenesis;metabolic process	mitochondrion;ribosome	structural molecule activity	0.905	0.738	0.82
P08466	mitochondrial nuclease [OS=Saccharomyces cerevisiae S288c]	853222	NUC1; YJL208C	cell death;metabolic process	membrane;mitochondrion;nucleus	catalytic activity;metal ion binding	1.637	1.336	0.82
P30902	ATP synthase subunit d, mitochondrial [OS=Saccharomyces cerevisiae S288c]	853853	ATP7; YKL016C	metabolic process;transport	membrane;mitochondrion	catalytic activity;transporter activity	5.105	4.179	0.82
P36516	54S ribosomal protein L3, mitochondrial [OS=Saccharomyces cerevisiae S288c]	855039	MRPL3; YMR024W	cell organization and biogenesis;metabolic process;response to stimulus	mitochondrion;nucleus;ribosome	catalytic activity;RNA binding;structural molecule activity	0.743	0.61	0.82
P47045	mitochondrial import inner membrane translocase subunit TIM54 [OS=Saccharomyces cerevisiae S288c]	853392	TIM54; YJL054W	cell organization and biogenesis;transport	cytosol;membrane;mitochondrion	transporter activity	0.655	0.54	0.82
Q12166	2-isopropylmalate synthase 2, mitochondrial [OS=Saccharomyces cerevisiae S288c]	854275	LEU9; YOR108W	metabolic process	mitochondrion	catalytic activity;protein binding	2.472	2.065	0.84
P06168	ketol-acid reductoisomerase, mitochondrial [OS=Saccharomyces cerevisiae S288c]	851069	ILV5; YLR355C	cell organization and biogenesis;metabolic process	mitochondrion	catalytic activity;DNA binding;metal ion binding	12.895	10.788	0.84
P15424	ATP-dependent RNA helicase MSS116, mitochondrial [OS=Saccharomyces cerevisiae S288c]	851775	MSS116; YDR194C	metabolic process;regulation of biological process	mitochondrion;organelle lumen	catalytic activity;nucleotide binding;RNA binding	1.644	1.387	0.84
P07275	Delta-1-pyrroline-5-carboxylate dehydrogenase, mitochondrial [OS=Saccharomyces cerevisiae S288c]	856432	PUT2; YHR037W	metabolic process	membrane;mitochondrion;organelle lumen	catalytic activity	6.055	5.136	0.85
P00360	glyceraldehyde-3-phosphate dehydrogenase 1 [OS=Saccharomyces cerevisiae S288c]	853395	TDH1; YJL052W	metabolic process	cytoplasm;cytosol;membrane;mitochondrion	catalytic activity;nucleotide binding	1.721	1.462	0.85
P09440	C-1-tetrahydrofolate synthase, mitochondrial [OS=Saccharomyces cerevisiae S288c]	852378	MIS1; YBR084W	metabolic process	cytosol;mitochondrion	catalytic activity;nucleotide binding	1.049	0.9	0.86
P09457	ATP synthase subunit 5, mitochondrial [OS=Saccharomyces cerevisiae S288c]	851892	ATP5; YDR298C	metabolic process;transport	membrane;mitochondrion	catalytic activity;transporter activity	9	7.799	0.87
P04840	Mitochondrial outer membrane protein porin 1 [OS=Saccharomyces cerevisiae S288c]	855669	POR1; YNL055C	cell death;cell organization and biogenesis;cellular homeostasis;regulation of biological process;transport	cytoplasm;membrane;mitochondrion	transporter activity	15.238	13.384	0.88
P40341	Mitochondrial respiratory chain complexes assembly protein YTA12 [OS=Saccharomyces cerevisiae S288c]	855114	YTA12; YMR089C	cell organization and biogenesis;metabolic process;response to stimulus;transport	cytoplasm;membrane;mitochondrion	catalytic activity;metal ion binding;nucleotide binding;protein binding	1.371	1.276	0.93
P06208-1	2-isopropylmalate synthase [OS=Saccharomyces cerevisiae S288c]	855619	LEU4; YNL104C	metabolic process	cytoplasm;mitochondrion	catalytic activity;protein binding	5.236	4.878	0.93
P19414	Aconitate hydratase, mitochondrial [OS=Saccharomyces cerevisiae S288c]	851013	ACO1; YLR304C	cell organization and biogenesis;metabolic process;transport	cytoplasm;cytosol;mitochondrion;organelle lumen	catalytic activity;DNA binding;metal ion binding	48.619	46.197	0.95
P07342	Acetolactate synthase catalytic subunit, mitochondrial [OS=Saccharomyces cerevisiae S288c]	855135	ILV2; YMR108W	metabolic process	mitochondrion	catalytic activity;metal ion binding;nucleotide binding	4.223	4.223	1.00
P47127	Altered inheritance of mitochondria protein 24, mitochondrial [OS=Saccharomyces cerevisiae S288c]	853543	AIM24; YJR080C	cell organization and biogenesis	mitochondrion		2.857	2.857	1.00
P34227	Mitochondrial peroxiredoxin PRX1 [OS=Saccharomyces cerevisiae S288c]	852215	PRX1; YBL064C	cellular homeostasis;metabolic process;regulation of biological process;response to stimulus	mitochondrion	antioxidant activity;catalytic activity	5.158	5.158	1.00

Q03104	Meiotic sister chromatid recombination protein 1 [OS=Saccharomyces cerevisiae S288c]	854911	MSC1; YML128C	metabolic process	endoplasmic reticulum;membrane;mitochondrion		2.415	2.415	1.00
Q03028	Mitochondrial 2-oxodicarboxylate carrier 1 [OS=Saccharomyces cerevisiae S288c]	855969	ODC1; YPL134C	metabolic process;transport	membrane;mitochondrion	structural molecule activity;transporter activity	5.813	5.813	1.00
P52893	probable alanine aminotransferase, mitochondrial [OS=Saccharomyces cerevisiae S288c]	850778	ALT1; YLR089C	metabolic process	mitochondrion;organelle lumen	catalytic activity	1.102	1.102	1.00
P38988	Mitochondrial GTP/GDP carrier protein 1 [OS=Saccharomyces cerevisiae S288c]	851329	GGC1; YDL198C	cell organization and biogenesis;cellular homeostasis;metabolic process;transport	membrane;mitochondrion	structural molecule activity;transporter activity	3.16	3.16	1.00
Q12029	Probable mitochondrial transport protein fsf1 [OS=Saccharomyces cerevisiae S288c]	854445	FSF1; YOR271C	transport	membrane;mitochondrion	transporter activity	2.36	2.36	1.00
Q07349	MIOREX complex component 9 [OS=Saccharomyces cerevisiae S288c]	851535	YDL027C; MRX9; YDL027C		endoplasmic reticulum;membrane;mitochondrion		1.913	1.913	1.00
P39518	Long-chain-fatty-acid--CoA ligase 2 [OS=Saccharomyces cerevisiae S288c]	856734	FAA2; YER015W	metabolic process;transport	cytoplasm;mitochondrion	catalytic activity;nucleotide binding	0.619	0.619	1.00
P32331	Carrier protein YMC1, mitochondrial [OS=Saccharomyces cerevisiae S288c]	856171	YMC1; YPR058W	metabolic process;transport	membrane;mitochondrion;vacuole	structural molecule activity;transporter activity	1.581	1.581	1.00
P38825	Protein TOM71 [OS=Saccharomyces cerevisiae S288c]	856517	TOM71; YHR117W	transport	membrane;mitochondrion	protein binding;transporter activity	0.512	0.512	1.00
P40508	Uncharacterized protein YIL077C [OS=Saccharomyces cerevisiae S288c]	854733	YIL077C; YIL077C		mitochondrion		0.896	0.896	1.00
P15179	Aspartate--tRNA ligase, mitochondrial [OS=Saccharomyces cerevisiae S288c]	856000	MSD1; YPL104W	metabolic process	cytoplasm;mitochondrion;organelle lumen	catalytic activity;nucleotide binding	0.379	0.379	1.00
P00950	Phosphoglycerate mutase 1 [OS=Saccharomyces cerevisiae S288c]	853705	GPM1; YKL152C	metabolic process;regulation of biological process	cytoplasm;cytosol;membrane;mitochondrion	catalytic activity	1.929	1.929	1.00
P40416	Iron-sulfur clusters transporter ATM1, mitochondrial [OS=Saccharomyces cerevisiae S288c]	855347	ATM1; YMR301C	cellular homeostasis;metabolic process;transport	membrane;mitochondrion	catalytic activity;nucleotide binding;transporter activity	0.453	0.453	1.00
P38626	NADH-cytochrome b5 reductase 1 [OS=Saccharomyces cerevisiae S288c]	854768	CBR1; YIL043C	metabolic process	endoplasmic reticulum;membrane;mitochondrion;nucleus	catalytic activity	0.957	0.957	1.00
P53230	Phosphatidate cytidylyltransferase, mitochondrial [OS=Saccharomyces cerevisiae S288c]	852937	TAM41; YGR046W	metabolic process	membrane;mitochondrion;organelle lumen	catalytic activity	0.668	0.668	1.00
P27697	Atypical kinase COQ8, mitochondrial [OS=Saccharomyces cerevisiae S288c]	852758	COQ8; YGL119W	metabolic process	membrane;mitochondrion;organelle lumen	catalytic activity;nucleotide binding	0.453	0.453	1.00
P47039	Probable kynurenine--oxoglutarate transaminase BNA3 [OS=Saccharomyces cerevisiae S288c]	853386	BNA3; YJL060W	metabolic process;regulation of biological process	cytoplasm;mitochondrion	catalytic activity	0.468	0.468	1.00
P25626	54S ribosomal protein IMG1, mitochondrial [OS=Saccharomyces cerevisiae S288c]	850413	IMG1; YCR046C	cell organization and biogenesis;metabolic process	mitochondrion;ribosome	structural molecule activity	1.031	1.031	1.00
P53220	Mitochondrial import inner membrane translocase subunit TIM21 [OS=Saccharomyces cerevisiae S288c]	852921	TIM21; YGR033C	cell organization and biogenesis;transport	membrane;mitochondrion	protein binding	0.719	0.719	1.00
P32939	GTP-binding protein ypt7 [OS=Saccharomyces cerevisiae S288c]	855012	YPT7; YML001W	cell communication;cell organization and biogenesis;metabolic process;regulation of biological process;response to stimulus;transport	cytosol;endosome;membrane;mitochondrion;vacuole	catalytic activity;nucleotide binding;protein binding	0.585	0.585	1.00
P48360	Probable NADPH:adrenodoxin oxidoreductase, mitochondrial [OS=Saccharomyces cerevisiae S288c]	851982	ARH1; YDR376W	cellular homeostasis;metabolic process;transport	cytoplasm;membrane;mitochondrion;organelle lumen	catalytic activity	0.346	0.346	1.00
P25270	rRNA methyltransferase 1, mitochondrial [OS=Saccharomyces cerevisiae S288c]	854376	MRM1; YOR201C	metabolic process	mitochondrion	catalytic activity;RNA binding	0.274	0.274	1.00
P38172	MIOREX complex component 3 [OS=Saccharomyces cerevisiae S288c]	852181	YBL095W; MRX3; YBL095W		membrane;mitochondrion		0.501	0.501	1.00
P09201	fructose-1,6-bisphosphatase [OS=Saccharomyces cerevisiae S288c]	851092	FBP1; YLR377C	metabolic process	cytosol	catalytic activity;metal ion binding	0.274	0.274	1.00

Q06005	Octanoyltransferase, mitochondrial [OS=Saccharomyces cerevisiae S288c]	850940	LIP2; YLR239C	metabolic process	cytoplasm;mitochondrion	catalytic activity	0.245	0.245	1.00
Q06630	Mitochondrial homologous recombination protein 1 [OS=Saccharomyces cerevisiae S288c]	851890	MHR1; YDR296W	cell organization and biogenesis;metabolic process;regulation of biological process;response to stimulus	mitochondrion	catalytic activity;DNA binding;structural molecule activity	0.359	0.359	1.00
P18409	Mitochondrial distribution and morphology protein 10 [OS=Saccharomyces cerevisiae S288c]	851223	MDM10; YAL010C	cell organization and biogenesis;transport	membrane;mitochondrion	protein binding	0.16	0.16	1.00
P32378	4-hydroxybenzoate polyprenyltransferase, mitochondrial [OS=Saccharomyces cerevisiae S288c]	855778	COQ2; YNR041C	metabolic process;transport	membrane;mitochondrion	catalytic activity	0.292	0.292	1.00
P32493	ATPase expression protein 1, mitochondrial [OS=Saccharomyces cerevisiae S288c]	855086	AEP1; YMR064W	regulation of biological process	mitochondrion	translation regulator activity	0.15	0.15	1.00
Q07821	Iron-sulfur assembly protein 1 [OS=Saccharomyces cerevisiae S288c]	850632	ISA1; YLL027W	cell organization and biogenesis;metabolic process	mitochondrion;organelle lumen	metal ion binding;structural molecule activity	0.136	0.136	1.00
Q00402	Nuclear migration protein NUM1 [OS=Saccharomyces cerevisiae S288c]	851727	NUM1; YDR150W	cell organization and biogenesis;cellular component movement;metabolic process;regulation of biological process;transport	endoplasmic reticulum;mitochondrion	catalytic activity;motor activity;protein binding	0.025	0.025	1.00
P34231	Uncharacterized protein YKL187C [OS=Saccharomyces cerevisiae S288c]	853648	FAT3; YKL187C; YKL187C	cell organization and biogenesis;transport	membrane;mitochondrion		0.194	0.194	1.00
P39515	mitochondrial import inner membrane translocase subunit Tim17 [OS=Saccharomyces cerevisiae S288c]	853298	TIM17; YJL143W	cell organization and biogenesis;transport	membrane;mitochondrion	protein binding;transporter activity	0.778	0.778	1.00
P38884	Altered inheritance of mitochondria protein 18, mitochondrial [OS=Saccharomyces cerevisiae S288c]	856605	AIM18; YHR198C		mitochondrion	catalytic activity	0.145	0.145	1.00
Q2V2P9	Uncharacterized protein YDR119W-A [OS=Saccharomyces cerevisiae S288c]	3799970	COX26; YDR119W-A		membrane;mitochondrion		1.154	1.154	1.00
P32463	Acyl carrier protein, mitochondrial [OS=Saccharomyces cerevisiae S288c]	853642	ACP1; YKL192C	metabolic process;transport	mitochondrion		0.259	0.259	1.00
P47158	Putative transferase CAF17, mitochondrial [OS=Saccharomyces cerevisiae S288c]	853586	IBA57; YJR122W	cell organization and biogenesis;metabolic process	mitochondrion;organelle lumen	catalytic activity	0.075	0.075	1.00
P40051	Intermediate cleaving peptidase 55 [OS=Saccharomyces cerevisiae S288c]	856811	ICP55; YER078C	metabolic process;regulation of biological process	membrane;mitochondrion	catalytic activity;metal ion binding	0.077	0.077	1.00
P38816	thioredoxin reductase 2, mitochondrial [OS=Saccharomyces cerevisiae S288c]	856506	TRR2; YHR106W	cellular homeostasis;metabolic process;regulation of biological process;response to stimulus	cytoplasm;mitochondrion	antioxidant activity;catalytic activity	0.136	0.136	1.00
Q06510	Lysophosphatidylcholine acyltransferase [OS=Saccharomyces cerevisiae S288c]	856262	TAZ1; YPR140W	cell organization and biogenesis;metabolic process;transport	membrane;mitochondrion	catalytic activity	0.093	0.093	1.00
P04039	Cytochrome c oxidase polypeptide VIII, mitochondrial [OS=Saccharomyces cerevisiae S288c]	851111	COX8; YLR395C	metabolic process;transport	membrane;mitochondrion	catalytic activity;transporter activity	0.778	0.778	1.00
P10174	cytochrome c oxidase subunit 7 [OS=Saccharomyces cerevisiae S288c]	855298	COX7; YMR256C	metabolic process;transport	membrane;mitochondrion	catalytic activity;transporter activity	1.154	1.154	1.00
P13433	DNA-directed RNA polymerase, mitochondrial [OS=Saccharomyces cerevisiae S288c]	850507	RPO41; YFL036W	cell organization and biogenesis;metabolic process	cytoplasm;mitochondrion;organelle lumen	catalytic activity;DNA binding	0.056	0.056	1.00
Q05779	Ubiquinone biosynthesis protein COQ9, mitochondrial [OS=Saccharomyces cerevisiae S288c]	850898	COQ9; YLR201C	metabolic process	membrane;mitochondrion		0.166	0.166	1.00
Q02863	ubiquitin carboxyl-terminal hydrolase 16 [OS=Saccharomyces cerevisiae S288c]	856032	UBP16; YPL072W	metabolic process	membrane;mitochondrion	catalytic activity	0.072	0.072	1.00
P04801	Threonine--tRNA ligase, cytoplasmic [OS=Saccharomyces cerevisiae S288c]	854732	THS1; YIL078W	metabolic process	cytoplasm;mitochondrion	catalytic activity;nucleotide binding;RNA binding	0.05	0.05	1.00
P09624	Dihydrolipoyl dehydrogenase, mitochondrial [OS=Saccharomyces cerevisiae S288c]	850527	LPD1; YFL018C	cellular homeostasis;metabolic process;regulation of biological process	mitochondrion;organelle lumen	catalytic activity;nucleotide binding	21.539	23.119	1.07

P16547	Mitochondrial outer membrane protein OM45 [OS=Saccharomyces cerevisiae S288c]	854670	OM45; YIL136W		membrane;mitochondrion		250.189	274.423	1.10
Q12466	tricalbin-1 [OS=Saccharomyces cerevisiae S288c]	854253	TCB1; YOR086C	cell organization and biogenesis;regulation of biological process	endoplasmic reticulum;membrane;mitochondrion	metal ion binding;protein binding	0.417	0.468	1.12
P32332	Mitochondrial oxaloacetate transport protein [OS=Saccharomyces cerevisiae S288c]	853739	OAC1; YKL120W	metabolic process;transport	membrane;mitochondrion	structural molecule activity;transporter activity	4.995	5.813	1.16
Q12482	Mitochondrial aspartate-glutamate transporter AGC1 [OS=Saccharomyces cerevisiae S288c]	856132	AGC1; YPR021C	metabolic process;transport	membrane;mitochondrion	structural molecule activity;transporter activity	0.348	0.407	1.17
P00924	Enolase 1 [OS=Saccharomyces cerevisiae S288c]	853169	ENO1; YGR254W	metabolic process;regulation of biological process	cytoplasm;cytosol;membrane;mitochondrion;vacuole	catalytic activity;metal ion binding;protein binding	0.848	0.995	1.17
P12695	Dihydrolipoyllysine-residue acetyltransferase component of pyruvate dehydrogenase complex, mitochondrial [OS=Saccharomyces cerevisiae S288c]	855653	LAT1; YNL071W	metabolic process	mitochondrion;organelle lumen	catalytic activity	6.43	7.62	1.19
P40035	Mitochondrial phosphate carrier protein 2 [OS=Saccharomyces cerevisiae S288c]	856779	PIC2; YER053C	cellular homeostasis;metabolic process;transport	membrane;mitochondrion	structural molecule activity;transporter activity	1.637	1.976	1.21
Q12374	Nuclear control of ATPase protein 2 [OS=Saccharomyces cerevisiae S288c]	856278	NCA2; YPR155C	metabolic process	membrane;mitochondrion		0.468	0.565	1.21
Q07651	SUR7 family protein FMP45 [OS=Saccharomyces cerevisiae S288c]	851304	FMP45; YDL222C	cell differentiation;cell organization and biogenesis	membrane;mitochondrion		4.878	6.017	1.23
Q01852	Mitochondrial import inner membrane translocase subunit TIM44 [OS=Saccharomyces cerevisiae S288c]	854790	TIM44; YIL022W	transport	membrane;mitochondrion	nucleotide binding;protein binding	3.758	4.946	1.32
P33303	Succinate/fumarate mitochondrial transporter [OS=Saccharomyces cerevisiae S288c]	853558	SFC1; YJR095W	metabolic process;transport	membrane;mitochondrion	structural molecule activity;transporter activity	9	11.915	1.32
P25087	Sterol 24-C-methyltransferase [OS=Saccharomyces cerevisiae S288c]	855003	ERG6; YML008C	metabolic process	endoplasmic reticulum;membrane;mitochondrion	catalytic activity	1.424	1.894	1.33
Q04013	Citrate/oxoglutarate carrier protein [OS=Saccharomyces cerevisiae S288c]	855282	YHM2; YMR241W	cell organization and biogenesis;metabolic process;transport	membrane;mitochondrion	DNA binding;structural molecule activity;transporter activity	3.467	4.623	1.33
P53292	37S ribosomal protein S35, mitochondrial [OS=Saccharomyces cerevisiae S288c]	853075	MRPS35; YGR165W	cell organization and biogenesis;metabolic process	mitochondrion;ribosome	structural molecule activity	1.512	2.02	1.34
P53266	Cytochrome oxidase assembly protein SHY1 [OS=Saccharomyces cerevisiae S288c]	853009	SHY1; YGR112W	cell organization and biogenesis	membrane;mitochondrion	protein binding	1.154	1.555	1.35
P42900	Sigma-like sequence protein 1, mitochondrial [OS=Saccharomyces cerevisiae S288c]	850830	SLS1; YLR139C	cell organization and biogenesis;metabolic process	membrane;mitochondrion	protein binding	0.194	0.266	1.37
P54115	Magnesium-activated aldehyde dehydrogenase, cytosolic [OS=Saccharomyces cerevisiae S288c]	856044	ALD6; YPL061W	metabolic process;response to stimulus	cytoplasm;cytosol;mitochondrion	catalytic activity	0.25	0.346	1.38
P20967	2-oxoglutarate dehydrogenase, mitochondrial [OS=Saccharomyces cerevisiae S288c]	854681	KGD1; YIL125W	metabolic process	cytosol;mitochondrion;organelle lumen	catalytic activity	11.426	15.843	1.39
P53206	putative cysteine synthase [OS=Saccharomyces cerevisiae S288c]	852895	YGR012W; YGR012W	metabolic process		catalytic activity	0.817	1.154	1.41
Q12289	mitochondrial carnitine carrier [OS=Saccharomyces cerevisiae S288c]	854267	CRC1; YOR100C	metabolic process;transport	membrane;mitochondrion	structural molecule activity;transporter activity	1.404	1.994	1.42
P21771	37S ribosomal protein S28, mitochondrial [OS=Saccharomyces cerevisiae S288c]	851937	MRPS28; YDR337W	cell organization and biogenesis;metabolic process	mitochondrion;ribosome	RNA binding;structural molecule activity	0.438	0.624	1.42
Q06567	ABC1 family protein MCP2 [OS=Saccharomyces cerevisiae S288c]	850955	YLR253W; MCP2; YLR253W	cell organization and biogenesis	membrane;mitochondrion		0.484	0.693	1.43
P00890	citrate synthase, mitochondrial [OS=Saccharomyces cerevisiae S288c]	855732	CIT1; YNR001C	metabolic process;transport	cytosol;mitochondrion;organelle lumen	catalytic activity	9	12.895	1.43

P17558	37S ribosomal protein PET123, mitochondrial [OS=Saccharomyces cerevisiae S288c]	854329	PET123; YOR158W	cell organization and biogenesis;metabolic process	mitochondrion;ribosome	structural molecule activity	1.448	2.162	1.49
P28241	Isocitrate dehydrogenase [NAD] subunit 2, mitochondrial [OS=Saccharomyces cerevisiae S288c]	854303	IDH2; YOR136W	metabolic process	mitochondrion;organelle lumen	catalytic activity;metal ion binding;nucleotide binding;protein binding;RNA binding	3.642	5.449	1.50
P18239	ADP,ATP carrier protein 2 [OS=Saccharomyces cerevisiae S288c]	852250	PET9; YBL030C	cell death;metabolic process;transport	membrane;mitochondrion	structural molecule activity;transporter activity	110.034	167.761	1.52
P21801	Succinate dehydrogenase [ubiquinone] iron-sulfur subunit, mitochondrial [OS=Saccharomyces cerevisiae S288c]	850685	SDH2; YLL041C	metabolic process;transport	membrane;mitochondrion	catalytic activity;metal ion binding	18.684	28.552	1.53
P24720	Protein MNE1 [OS=Saccharomyces cerevisiae S288c]	854532	MNE1; YOR350C	metabolic process	cytoplasm;mitochondrion;organelle lumen		0.125	0.194	1.55
P22438	Methionine--tRNA ligase, mitochondrial [OS=Saccharomyces cerevisiae S288c]	853081	MSM1; YGR171C	metabolic process	cytoplasm;cytosol;mitochondrion;organelle lumen	catalytic activity;nucleotide binding	0.136	0.212	1.56
P53721	Respiratory supercomplex factor 2, mitochondrial [OS=Saccharomyces cerevisiae S288c]	855752	RCF2; YNR018W	cell organization and biogenesis	membrane;mitochondrion		5.579	9	1.61
P14540	fructose-bisphosphate aldolase [OS=Saccharomyces cerevisiae S288c]	853805	FBA1; YKL060C	metabolic process	cytoplasm;cytosol;membrane;mitochondrion	catalytic activity;metal ion binding	0.896	1.448	1.62
Q12375	mitochondrial ornithine transporter 1 [OS=Saccharomyces cerevisiae S288c]	854297	ORT1; YOR130C	metabolic process;transport	membrane;mitochondrion	structural molecule activity;transporter activity	0.334	0.54	1.62
P00942	Triosephosphate isomerase [OS=Saccharomyces cerevisiae S288c]	851620	TPI1; YDR050C	metabolic process	cytoplasm;cytosol;membrane;mitochondrion	catalytic activity	0.425	0.701	1.65
P36526	54S ribosomal protein L27, mitochondrial [OS=Saccharomyces cerevisiae S288c]	852585	MRPL27; YBR282W	cell organization and biogenesis;metabolic process	mitochondrion;ribosome	structural molecule activity	0.585	0.995	1.70
P40033	37S ribosomal protein rsm18, mitochondrial [OS=Saccharomyces cerevisiae S288c]	856776	RSM18; YER050C	cell organization and biogenesis;metabolic process	mitochondrion;ribosome	structural molecule activity	0.585	0.995	1.70
Q02981	ABC1 family protein YPL109C, mitochondrial [OS=Saccharomyces cerevisiae S288c]	855995	YPL109C; YPL109C		mitochondrion		0.334	0.585	1.75
P27929	37S ribosomal protein NAM9, mitochondrial [OS=Saccharomyces cerevisiae S288c]	855585	NAM9; YNL137C	cell organization and biogenesis;metabolic process;regulation of biological process	mitochondrion;ribosome	RNA binding;structural molecule activity	1.062	1.865	1.76
P19882	heat shock protein 60, mitochondrial [OS=Saccharomyces cerevisiae S288c]	850963	HSP60; YLR259C	cell organization and biogenesis;metabolic process;regulation of biological process;transport	cytoplasm;cytosol;membrane;mitochondrion;organelle lumen	catalytic activity;DNA binding;nucleotide binding;protein binding	22.357	39.296	1.76
P16603	NADPH--cytochrome P450 reductase [OS=Saccharomyces cerevisiae S288c]	856438	NCP1; YHR042W	metabolic process	cytoplasm;cytosol;endoplasmic reticulum;membrane;mitochondrion	catalytic activity;metal ion binding;nucleotide binding	0.343	0.604	1.76
P21375	Fumarate reductase 2 [OS=Saccharomyces cerevisiae S288c]	853510	OSM1; YJR051W	metabolic process	endoplasmic reticulum;mitochondrion	catalytic activity;nucleotide binding	0.25	0.45	1.80
P12687	54S ribosomal protein L2, mitochondrial [OS=Saccharomyces cerevisiae S288c]	855727	MRP7; YNL005C	cell organization and biogenesis;metabolic process	mitochondrion;ribosome	catalytic activity;RNA binding;structural molecule activity	1.565	2.899	1.85
P48237	Mitochondrial group I intron splicing factor CCM1 [OS=Saccharomyces cerevisiae S288c]	853053	CCM1; YGR150C	cell organization and biogenesis;metabolic process	mitochondrion	RNA binding	0.044	0.089	2.02
Q01802	Aspartate aminotransferase, mitochondrial [OS=Saccharomyces cerevisiae S288c]	853755	AAT1; YKL106W	metabolic process	mitochondrion;organelle lumen	catalytic activity;protein binding	0.075	0.155	2.07
P41911	Glycerol-3-phosphate dehydrogenase [NAD(+)] 2, mitochondrial [OS=Saccharomyces cerevisiae S288c]	854095	GPD2; YOL059W	metabolic process	cytoplasm;cytosol;mitochondrion	catalytic activity;nucleotide binding;protein binding	0.089	0.186	2.09

Q01163	37S ribosomal protein S23, mitochondrial [OS=Saccharomyces cerevisiae S288c]	852748	RSM23; YGL129C	cell organization and biogenesis;metabolic process	mitochondrion;ribosome	catalytic activity;nucleotide binding;RNA binding;structural molecule activity	0.983	2.065	2.10
P32792	UPF0744 protein YSC83 [OS=Saccharomyces cerevisiae S288c]	856410	YSC83; YHR017W		membrane;mitochondrion		0.11	0.233	2.12
P49954	probable hydrolase NIT3 [OS=Saccharomyces cerevisiae S288c]	851065	NIT3; YLR351C	metabolic process	cytoplasm;mitochondrion	catalytic activity	0.129	0.274	2.12
P38297	Mitofusin FZO1 [OS=Saccharomyces cerevisiae S288c]	852477	FZO1; YBR179C	cell organization and biogenesis	membrane;mitochondrion	catalytic activity;nucleotide binding;protein binding	0.142	0.304	2.14
Q08926	ULP1-interacting protein 4 [OS=Saccharomyces cerevisiae S288c]	855916	UIP4; YPL186C		endoplasmic reticulum;membrane;mitochondrion;nucleus		0.136	0.292	2.15
P41921	glutathione reductase [OS=Saccharomyces cerevisiae S288c]	856014	GLR1; YPL091W	cellular homeostasis;metabolic process;regulation of biological process;response to stimulus	cytoplasm;cytosol;mitochondrion;nucleus	antioxidant activity;catalytic activity;nucleotide binding	0.155	0.334	2.15
P12709	glucose-6-phosphate isomerase [OS=Saccharomyces cerevisiae S288c]	852495	PGI1; YBR196C	metabolic process	cytoplasm;membrane;mitochondrion	catalytic activity	0.166	0.359	2.16
P53166	ATP-dependent RNA helicase mrh4, mitochondrial [OS=Saccharomyces cerevisiae S288c]	852816	MRH4; YGL064C	cell organization and biogenesis;metabolic process	mitochondrion;organelle lumen	catalytic activity;nucleotide binding;RNA binding	0.225	0.501	2.23
P50110	Sorting assembly machinery 37 kDa subunit [OS=Saccharomyces cerevisiae S288c]	855082	SAM37; YMR060C	cell organization and biogenesis;transport	membrane;mitochondrion	protein binding	0.245	0.551	2.25
P22136	ATPase expression protein 2, mitochondrial [OS=Saccharomyces cerevisiae S288c]	855325	AEP2; YMR282C	metabolic process;regulation of biological process	mitochondrion	RNA binding	0.292	0.668	2.29
P04803	Tryptophan--tRNA ligase, mitochondrial [OS=Saccharomyces cerevisiae S288c]	851861	MSW1; YDR268W	metabolic process	cytoplasm;mitochondrion;organelle lumen	catalytic activity;nucleotide binding	0.292	0.668	2.29
P40496	37S ribosomal protein S25, mitochondrial [OS=Saccharomyces cerevisiae S288c]	854715	RSM25; YIL093C	cell organization and biogenesis;metabolic process	mitochondrion;ribosome	structural molecule activity	0.292	0.668	2.29
Q03430	37S ribosomal protein RSM28, mitochondrial [OS=Saccharomyces cerevisiae S288c]	852105	RSM28; YDR494W	cell organization and biogenesis;metabolic process	mitochondrion;ribosome	structural molecule activity	1.462	3.489	2.39
P36520	54S ribosomal protein L10, mitochondrial [OS=Saccharomyces cerevisiae S288c]	855436	MRPL10; YNL284C	cell organization and biogenesis;metabolic process	mitochondrion;ribosome	structural molecule activity	0.551	1.404	2.55
Q03653	protein EFR3 [OS=Saccharomyces cerevisiae S288c]	855252	EFR3; YMR212C		membrane;mitochondrion		0.096	0.259	2.70
P39952	Mitochondrial inner membrane protein OXA1 [OS=Saccharomyces cerevisiae S288c]	856898	OXA1; YER154W	cell organization and biogenesis;transport	membrane;mitochondrion	transporter activity	1.371	3.87	2.82
P38169	Kynurenine 3-monooxygenase [OS=Saccharomyces cerevisiae S288c]	852179	BNA4; YBL098W	metabolic process	membrane;mitochondrion	catalytic activity;nucleotide binding	0.172	0.487	2.83
Q03640	Tricalbin-3 [OS=Saccharomyces cerevisiae S288c]	854903	TCB3; YML072C	cell organization and biogenesis;regulation of biological process	endoplasmic reticulum;membrane;mitochondrion	metal ion binding;protein binding	0.283	0.806	2.85
P36517	54S ribosomal protein L4, mitochondrial [OS=Saccharomyces cerevisiae S288c]	851160	MRPL4; YLR439W	cell organization and biogenesis;metabolic process	mitochondrion;ribosome	structural molecule activity	0.369	1.081	2.93
Q12251	Uncharacterized mitochondrial carrier YPR011C [OS=Saccharomyces cerevisiae S288c]	856121	YPR011C; YPR011C	metabolic process;transport	membrane;mitochondrion	structural molecule activity;transporter activity	0.245	0.73	2.98
P28737	Protein MSP1 [OS=Saccharomyces cerevisiae S288c]	852915	MSP1; YGR028W	cell organization and biogenesis;metabolic process;regulation of biological process;response to stimulus;transport	membrane;mitochondrion;nucleus	catalytic activity;nucleotide binding;protein binding;RNA binding	0.259	0.778	3.00
P40015	inositol phosphosphingolipids phospholipase C [OS=Saccharomyces cerevisiae S288c]	856739	ISC1; YER019W	metabolic process;response to stimulus	endoplasmic reticulum;membrane;mitochondrion	catalytic activity;metal ion binding	0.101	0.334	3.31
Q03327	Mitochondrial fusion and transport protein Ugo1 [OS=Saccharomyces cerevisiae S288c]	852081	UGO1; YDR470C	cell organization and biogenesis;metabolic process	membrane;mitochondrion	protein binding;structural molecule activity	0.425	1.424	3.35

P25038	Translation initiation factor IF-2, mitochondrial [OS=Saccharomyces cerevisiae S288c]	854135	IFM1; YOL023W	cell organization and biogenesis;metabolic process	mitochondrion;nucleus	catalytic activity;nucleotide binding;RNA binding	0.11	0.369	3.35
Q12117	Protein MRH1 [OS=Saccharomyces cerevisiae S288c]	851597	MRH1; YDR033W	transport	endoplasmic reticulum;membrane;mitochondrion	transporter activity	2.162	7.254	3.36
P49334	Mitochondrial import receptor subunit tom22 [OS=Saccharomyces cerevisiae S288c]	855592	TOM22; YNL131W	cell organization and biogenesis;transport	membrane;mitochondrion	protein binding;transporter activity	0.292	1.154	3.95
P25578	CDP-diacylglycerol--glycerol-3-phosphate 3-phosphatidyltransferase [OS=Saccharomyces cerevisiae S288c]	850352	PGS1; YCL004W	metabolic process	mitochondrion	catalytic activity;nucleotide binding	0.083	0.374	4.51

Supplemental Table S12. The relative concentrations of proteins in mitochondria purified from WT or *ups2Δ* cells cultured without LCA. Mitochondria were purified from WT or *ups2Δ* cells recovered on day 7 of cell culturing without LCA. Mass spectrometry-based identification and quantitation of proteins recovered in purified mitochondria were performed as described in Materials and methods. The exponentially modified protein abundance index (emPAI) provided by the ″Proteome Discoverer″ software was used to calculate the relative abundance of mitochondrial proteins between specified datasets. Data on the relative concentrations of mitochondrial proteins are presented as means of 2 independent experiments.

Accession	Description	Entrez Gene ID	Gene ID	Biological Process	Cellular Component	Molecular Function	emPAI WT	emPAI *ups2*	Ratio *ups2* / WT
P38120	37S ribosomal protein S9, mitochondrial [OS=Saccharomyces cerevisiae S288c]	852443	MRPS9; YBR146W	cell organization and biogenesis;metabolic process	mitochondrion;ribosome	RNA binding;structural molecule activity	3.437	0.145	0.04
P12687	54S ribosomal protein L2, mitochondrial [OS=Saccharomyces cerevisiae S288c]	855727	MRP7; YNL005C	cell organization and biogenesis;metabolic process	mitochondrion;ribosome	catalytic activity;RNA binding;structural molecule activity	1.848	0.11	0.06
P08525	Cytochrome b-c1 complex subunit 8 [OS=Saccharomyces cerevisiae S288c]	853273	QCR8; YJL166W	metabolic process;transport	membrane;mitochondrion	catalytic activity;transporter activity	5.31	0.585	0.11
P48527	Tyrosine--tRNA ligase, mitochondrial [OS=Saccharomyces cerevisiae S288c]	856007	MSY1; YPL097W	metabolic process	cytoplasm;cytosol;mitochondrion;organelle lumen	catalytic activity;nucleotide binding;RNA binding	0.63	0.072	0.11
Q03430	37S ribosomal protein RSM28, mitochondrial [OS=Saccharomyces cerevisiae S288c]	852105	RSM28; YDR494W	cell organization and biogenesis;metabolic process	mitochondrion;ribosome	structural molecule activity	3.062	0.35	0.11
P36141	Putative redox protein fmp46, mitochondrial [OS=Saccharomyces cerevisiae S288c]	853923	FMP46; YKR049C	metabolic process	mitochondrion	catalytic activity;protein binding	4.337	0.52	0.12
P38885	Altered inheritance of mitochondria protein 46, mitochondrial [OS=Saccharomyces cerevisiae S288c]	856606	AIM46; YHR199C	mitochondrion		catalytic activity	1.994	0.245	0.12
P38797	Protein phosphatase 2C homolog 7, mitochondrial [OS=Saccharomyces cerevisiae S288c]	856475	PTC7; YHR076W	metabolic process	mitochondrion	catalytic activity;metal ion binding;protein binding	0.874	0.11	0.13
P25605	Acetolactate synthase small subunit, mitochondrial [OS=Saccharomyces cerevisiae S288c]	850348	ILV6; YCL009C	metabolic process;regulation of biological process	mitochondrion	catalytic activity;enzyme regulator activity	10.288	1.336	0.13
P40165	NAD(P)H-hydrate epimerase [OS=Saccharomyces cerevisiae S288c]	855521	YNL200C; YNL200C	metabolic process	cytoplasm;mitochondrion	catalytic activity;metal ion binding;nucleotide binding	1.61	0.212	0.13
Q12349	ATP synthase subunit H, mitochondrial [OS=Saccharomyces cerevisiae S288c]	851002	ATP14; YLR295C	cell organization and biogenesis;metabolic process;transport	membrane;mitochondrion	catalytic activity;transporter activity	2.728	0.389	0.14
P42847	37S ribosomal protein S18, mitochondrial [OS=Saccharomyces cerevisiae S288c]	855410	MRPS18; YNL306W	cell organization and biogenesis;metabolic process	mitochondrion;ribosome	RNA binding;structural molecule activity	1.054	0.155	0.15
P00128	Cytochrome b-c1 complex subunit 7 [OS=Saccharomyces cerevisiae S288c]	852142	QCR7; YDR529C	cell organization and biogenesis;metabolic process;transport	membrane;mitochondrion	catalytic activity;transporter activity	11.915	1.783	0.15
P81449	ATP synthase subunit e, mitochondrial [OS=Saccharomyces cerevisiae S288c]	851922	TIM11; YDR322C-A	cell organization and biogenesis;metabolic process;transport	membrane;mitochondrion	catalytic activity;structural molecule activity;transporter activity	9	1.371	0.15
P36521	54S ribosomal protein L11, mitochondrial [OS=Saccharomyces cerevisiae S288c]	851325	MRPL11; YDL202W	cell organization and biogenesis;metabolic process	mitochondrion;ribosome	structural molecule activity	0.833	0.129	0.15
P37267	Assembly factor cbp4 [OS=Saccharomyces cerevisiae S288c]		CBP4	cell organization and biogenesis	membrane;mitochondrion		3.329	0.52	0.16
P14908	Mitochondrial transcription factor 1	855268	MTF1; YMR228W	metabolic process;regulation of	mitochondrion;organelle lumen	catalytic activity;DNA	0.778	0.122	0.16

	[OS=Saccharomyces cerevisiae S288c]			biological process		binding;RNA binding			
Q02486	ARS-binding factor 2, mitochondrial [OS=Saccharomyces cerevisiae S288c]	855094	ABF2; YMR072W	cell organization and biogenesis;metabolic process;regulation of biological process	chromosome;mitochondrion;nucleus	DNA binding	69.17	10.938	0.16
P36520	54S ribosomal protein L10, mitochondrial [OS=Saccharomyces cerevisiae S288c]	855436	MRPL10; YNL284C	cell organization and biogenesis;metabolic process	mitochondrion;ribosome	structural molecule activity	0.73	0.116	0.16
P40159	Uncharacterized protein YNL208W [OS=Saccharomyces cerevisiae S288c]	855513	YNL208W; YNL208W	membrane;mitochondrion;ribosome			1.783	0.292	0.16
P19955	37S ribosomal protein YMR-31, mitochondrial [OS=Saccharomyces cerevisiae S288c]	850610	YMR31; YFR049W	cell organization and biogenesis;metabolic process	mitochondrion;ribosome	catalytic activity;protein binding;structural molecule activity	15.681	2.594	0.17
P36517	54S ribosomal protein L4, mitochondrial [OS=Saccharomyces cerevisiae S288c]	851160	MRPL4; YLR439W	cell organization and biogenesis;metabolic process	mitochondrion;ribosome	structural molecule activity	2.162	0.369	0.17
P40502	Altered inheritance of mitochondria protein 19, mitochondrial [OS=Saccharomyces cerevisiae S288c]	854722	AIM19; YIL087C	membrane;mitochondrion			1.512	0.259	0.17
P00045	Cytochrome c iso-2 [OS=Saccharomyces cerevisiae S288c]	856672	CYC7; YEL039C	metabolic process;transport	mitochondrion	metal ion binding	1.512	0.259	0.17
P36526	54S ribosomal protein L27, mitochondrial [OS=Saccharomyces cerevisiae S288c]	852585	MRPL27; YBR282W	cell organization and biogenesis;metabolic process	mitochondrion;ribosome	structural molecule activity	1.512	0.259	0.17
Q01163	37S ribosomal protein S23, mitochondrial [OS=Saccharomyces cerevisiae S288c]	852748	RSM23; YGL129C	cell organization and biogenesis;metabolic process	mitochondrion;ribosome	catalytic activity;nucleotide binding;RNA binding;structural molecule activity	4.367	0.751	0.17
P51998	54S ribosomal protein yml6, mitochondrial [OS=Saccharomyces cerevisiae S288c]	854983	YML6; YML025C	cell organization and biogenesis;metabolic process	mitochondrion;ribosome	RNA binding;structural molecule activity	1.404	0.245	0.17
P32799	Cytochrome c oxidase subunit 6A, mitochondrial [OS=Saccharomyces cerevisiae S288c]	852684	COX13; YGL191W	cell organization and biogenesis;metabolic process;regulation of biological process;transport	membrane;mitochondrion	catalytic activity;enzyme regulator activity;transporter activity	11.589	2.162	0.19
P07253	cytochrome B pre-mRNA-processing protein 6 [OS=Saccharomyces cerevisiae S288c]	852417	CBP6; YBR120C	cell organization and biogenesis;metabolic process;regulation of biological process	mitochondrion;ribosome		3.394	0.638	0.19
P35180	mitochondrial import receptor subunit Tom20 [OS=Saccharomyces cerevisiae S288c]	852973	TOM20; YGR082W	cell organization and biogenesis;transport	membrane;mitochondrion	protein binding;transporter activity	1.031	0.194	0.19
P11914	Mitochondrial-processing peptidase subunit alpha [OS=Saccharomyces cerevisiae S288c]	856419	MAS2; YHR024C	metabolic process;transport	membrane;mitochondrion;organelle lumen	catalytic activity;metal ion binding;protein binding	2.311	0.445	0.19
P22353	54S ribosomal protein L8, mitochondrial [OS=Saccharomyces cerevisiae S288c]	853382	MRPL8; YJL063C	cell organization and biogenesis;metabolic process	mitochondrion;ribosome	structural molecule activity	0.848	0.166	0.20
P21771	37S ribosomal protein S28, mitochondrial [OS=Saccharomyces cerevisiae S288c]	851937	MRPS28; YDR337W	cell organization and biogenesis;metabolic process	mitochondrion;ribosome	RNA binding;structural molecule activity	0.624	0.129	0.21
P02381	Ribosomal protein VAR1, mitochondrial [OS=Saccharomyces cerevisiae S288c]	854586	VAR1; Q0140	cell organization and biogenesis;metabolic process	mitochondrion;ribosome	structural molecule activity	0.52	0.11	0.21
Q08818	Meiotic sister-chromatid recombination protein 6, mitochondrial [OS=Saccharomyces cerevisiae S288c]	854536	MSC6; YOR354C	metabolic process	mitochondrion;organelle lumen	RNA binding	1.047	0.227	0.22
P39676	Flavohemoprotein [OS=Saccharomyces cerevisiae S288c]	853149	YHB1; YGR234W	metabolic process;response to stimulus	cytoplasm;cytosol;mitochondrion;nucleus;organelle lumen	catalytic activity;metal ion binding;protein binding	4.179	0.931	0.22
P53881	54S ribosomal protein L22, mitochondrial [OS=Saccharomyces cerevisiae S288c]	855544	MRPL22; YNL177C	cell organization and biogenesis;metabolic process	mitochondrion;ribosome	RNA binding;structural molecule activity	1.683	0.389	0.23

P28817	3-hydroxyisobutyryl-CoA hydrolase, mitochondrial [OS=Saccharomyces cerevisiae S288c]	851606	EHD3; YDR036C	metabolic process	mitochondrion	catalytic activity	0.743	0.172	0.23
Q04728	Arginine biosynthesis bifunctional protein ArgJ, mitochondrial [OS=Saccharomyces cerevisiae S288c]	855084	ARG7; YMR062C	metabolic process	mitochondrion;organelle lumen	catalytic activity	0.63	0.15	0.24
P38323	ATP-dependent clpX-like chaperone, mitochondrial [OS=Saccharomyces cerevisiae S288c]	852528	MCX1; YBR227C	metabolic process	membrane;mitochondrion;organelle lumen	catalytic activity;nucleotide binding	0.607	0.145	0.24
P32897	Mitochondrial import inner membrane translocase subunit tim23 [OS=Saccharomyces cerevisiae S288c]	855751	TIM23; YNR017W	transport	membrane;mitochondrion	protein binding;transporter activity	4.012	0.995	0.25
P38088	Glycine--tRNA ligase 1, mitochondrial [OS=Saccharomyces cerevisiae S288c]		GRS1	metabolic process	cytoplasm;mitochondrion;organelle lumen	catalytic activity;nucleotide binding;protein binding	0.616	0.155	0.25
P23833	Protein SCO1, mitochondrial [OS=Saccharomyces cerevisiae S288c]	852325	SCO1; YBR037C	cell organization and biogenesis;cellular homeostasis;response to stimulus;transport	membrane;mitochondrion	antioxidant activity;catalytic activity;metal ion binding	1.154	0.292	0.25
P38771	Ribosome-recycling factor, mitochondrial [OS=Saccharomyces cerevisiae S288c]	856433	RRF1; YHR038W	cell organization and biogenesis;metabolic process	mitochondrion	RNA binding	1.955	0.501	0.26
P17558	37S ribosomal protein PET123, mitochondrial [OS=Saccharomyces cerevisiae S288c]	854329	PET123; YOR158W	cell organization and biogenesis;metabolic process	mitochondrion;ribosome	structural molecule activity	2.594	0.668	0.26
P40581	peroxiredoxin HYR1 [OS=Saccharomyces cerevisiae S288c]	854855	HYR1; YIR037W	metabolic process;response to stimulus	cytoplasm;cytosol;mitochondrion;organelle lumen	antioxidant activity;catalytic activity	0.995	0.259	0.26
P17695	Glutaredoxin-2, mitochondrial [OS=Saccharomyces cerevisiae S288c]	852124	GRX2; YDR513W	cellular homeostasis;metabolic process;regulation of biological process;response to stimulus;transport	cytoplasm;cytosol;mitochondrion;nucleus	antioxidant activity;catalytic activity	0.874	0.233	0.27
P00927	Threonine dehydratase, mitochondrial [OS=Saccharomyces cerevisiae S288c]	856819	ILV1; YER086W	metabolic process	cytoplasm;mitochondrion	catalytic activity	2.257	0.604	0.27
P32860	NifU-like protein, mitochondrial [OS=Saccharomyces cerevisiae S288c]	853826	NFU1; YKL040C	cell organization and biogenesis;cellular homeostasis;metabolic process	mitochondrion;organelle lumen	metal ion binding	3.87	1.054	0.27
Q05892	MIOREX complex component 2 [OS=Saccharomyces cerevisiae S288c]	850997	YLR290C; COQ11; YLR290C	metabolic process	mitochondrion	catalytic activity	3.87	1.054	0.27
P40513	Mitochondrial acidic protein MAM33 [OS=Saccharomyces cerevisiae S288c]	854740	MAM33; YIL070C	metabolic process;regulation of biological process	mitochondrion;organelle lumen	translation regulator activity	2.831	0.778	0.27
P00427	Cytochrome c oxidase subunit 6, mitochondrial [OS=Saccharomyces cerevisiae S288c]	856448	COX6; YHR051W	metabolic process;transport	membrane;mitochondrion	catalytic activity;metal ion binding;transporter activity	2.831	0.778	0.27
P00445	Superoxide dismutase [Cu-Zn] [OS=Saccharomyces cerevisiae S288c]	853568	SOD1; YJR104C	cell organization and biogenesis;cellular homeostasis;metabolic process;regulation of biological process;response to stimulus	cytoplasm;cytosol;mitochondrion;nucleus	antioxidant activity;catalytic activity;metal ion binding;protein binding	1.848	0.52	0.28
P42900	Sigma-like sequence protein 1, mitochondrial [OS=Saccharomyces cerevisiae S288c]	850830	SLS1; YLR139C	cell organization and biogenesis;metabolic process	membrane;mitochondrion	protein binding	0.425	0.125	0.29
P40582	Glutathione S-transferase 1 [OS=Saccharomyces cerevisiae S288c]	854856	GTT1; YIR038C	metabolic process	endoplasmic reticulum;membrane;mitochondrion;nucleus	antioxidant activity;catalytic activity;protein binding;RNA binding	2.162	0.638	0.30
P41911	Glycerol-3-phosphate dehydrogenase [NAD(+)] 2, mitochondrial [OS=Saccharomyces cerevisiae S288c]	854095	GPD2; YOL059W	metabolic process	cytoplasm;cytosol;mitochondrion	catalytic activity;nucleotide binding;protein binding	0.292	0.089	0.30
P38169	Kynurenine 3-monooxygenase [OS=Saccharomyces	852179	BNA4; YBL098W	metabolic process	membrane;mitochondrion	catalytic activity;nucleotide binding	0.269	0.083	0.31

	cerevisiae S288c]								
Q03557	Glutamyl-tRNA(Gln) amidotransferase subunit A, mitochondrial [OS=Saccharomyces cerevisiae S288c]	855338	HER2; YMR293C	cell organization and biogenesis;metabolic process	mitochondrion	catalytic activity;nucleotide binding	0.259	0.08	0.31
Q02863	ubiquitin carboxyl-terminal hydrolase 16 [OS=Saccharomyces cerevisiae S288c]	856032	UBP16; YPL072W	metabolic process	membrane;mitochondrion	catalytic activity	0.233	0.072	0.31
P37298	Succinate dehydrogenase [ubiquinone] cytochrome b small subunit, mitochondrial [OS=Saccharomyces cerevisiae S288c]	851758	SDH4; YDR178W	metabolic process;transport	membrane;mitochondrion	catalytic activity;metal ion binding	3.642	1.154	0.32
P36066	Protein MRG3-like [OS=Saccharomyces cerevisiae S288c]	853725	YKL133C; YKL133C	metabolic process	membrane;mitochondrion	protein binding	1.054	0.334	0.32
P50945	MICOS complex subunit MIC27 [OS=Saccharomyces cerevisiae S288c]	855623	AIM37; MIC27; YNL100W	cell organization and biogenesis	membrane;mitochondrion		0.968	0.311	0.32
P27929	37S ribosomal protein NAM9, mitochondrial [OS=Saccharomyces cerevisiae S288c]	855585	NAM9; YNL137C	cell organization and biogenesis;metabolic process;regulation of biological process	mitochondrion;ribosome	RNA binding;structural molecule activity	1.202	0.389	0.32
P32787	Mitochondrial genome maintenance protein MGM101 [OS=Saccharomyces cerevisiae S288c]	853609	MGM101; YJR144W	cell organization and biogenesis;metabolic process;response to stimulus	chromosome;mitochondrion	DNA binding	3.281	1.069	0.33
P04037	cytochrome c oxidase subunit 4, mitochondrial [OS=Saccharomyces cerevisiae S288c]	852688	COX4; YGL187C	cell organization and biogenesis;metabolic process;transport	membrane;mitochondrion	catalytic activity;metal ion binding;transporter activity	27.48	9	0.33
P36151	Uncharacterized protein YKR070W [OS=Saccharomyces cerevisiae S288c]	853944	YKR070W; YKR070W	metabolic process	mitochondrion		1.336	0.438	0.33
P15424	ATP-dependent RNA helicase MSS116, mitochondrial [OS=Saccharomyces cerevisiae S288c]	851775	MSS116; YDR194C	metabolic process;regulation of biological process	mitochondrion;organelle lumen	catalytic activity;nucleotide binding;RNA binding	1.783	0.585	0.33
Q06892	NADH kinase POS5, mitochondrial [OS=Saccharomyces cerevisiae S288c]	855913	POS5; YPL188W	metabolic process;response to stimulus	mitochondrion;organelle lumen	catalytic activity;nucleotide binding	1.565	0.52	0.33
P12686	37S ribosomal protein MRP13, mitochondrial [OS=Saccharomyces cerevisiae S288c]	852975	MRP13; YGR084C	cell organization and biogenesis;metabolic process	mitochondrion;ribosome	structural molecule activity	0.778	0.259	0.33
P38300	Inner membrane mitoribosome receptor MBA1, mitochondrial [OS=Saccharomyces cerevisiae S288c]	852483	MBA1; YBR185C	cell organization and biogenesis;regulation of biological process	membrane;mitochondrion		0.778	0.259	0.33
P39525	3-oxoacyl-[acyl-carrier-protein] synthase homolog [OS=Saccharomyces cerevisiae S288c]	856790	CEM1; YER061C	metabolic process	mitochondrion	catalytic activity	1.081	0.369	0.34
Q06678	54S ribosomal protein L35, mitochondrial [OS=Saccharomyces cerevisiae S288c]	851921	MRPL35; YDR322W	cell organization and biogenesis;metabolic process	mitochondrion;ribosome	structural molecule activity	1.219	0.425	0.35
P32902	37S ribosomal protein MRP4, mitochondrial [OS=Saccharomyces cerevisiae S288c]	856384	MRP4; YHL004W	cell organization and biogenesis;metabolic process	mitochondrion;ribosome	structural molecule activity	0.957	0.334	0.35
P21954	Isocitrate dehydrogenase [NADP], mitochondrial [OS=Saccharomyces cerevisiae S288c]	851493	IDP1; YDL066W	metabolic process	mitochondrion	catalytic activity;metal ion binding;nucleotide binding;protein binding	33.145	11.589	0.35
P25719	Peptidyl-prolyl cis-trans isomerase C, mitochondrial [OS=Saccharomyces cerevisiae S288c]	854897	CPR3; YML078W	cell death;metabolic process	mitochondrion;organelle lumen	catalytic activity	2.415	0.848	0.35
P25374	Cysteine desulfurase, mitochondrial [OS=Saccharomyces cerevisiae S288c]	850343	NFS1; YCL017C	cell organization and biogenesis;cellular homeostasis;metabolic process	mitochondrion;nucleus	catalytic activity;metal ion binding;protein binding	2.3	0.817	0.36

P36516	54S ribosomal protein L3, mitochondrial [OS=Saccharomyces cerevisiae S288c]	855039	MRPL3; YMR024W	cell organization and biogenesis;metabolic process;response to stimulus	mitochondrion;nucleus;ribosome	catalytic activity;RNA binding;structural molecule activity	1.043	0.374	0.36
P43567	alanine--glyoxylate aminotransferase 1 [OS=Saccharomyces cerevisiae S288c]	850514	AGX1; YFL030W	metabolic process	cytosol;mitochondrion	catalytic activity	2.162	0.778	0.36
P21306	ATP synthase subunit epsilon, mitochondrial [OS=Saccharomyces cerevisiae S288c]	855857	ATP15; YPL271W	metabolic process;transport	membrane;mitochondrion	catalytic activity;transporter activity	2.162	0.778	0.36
P02992	elongation factor Tu, mitochondrial [OS=Saccharomyces cerevisiae S288c]	854359	TUF1; YOR187W	cell organization and biogenesis;metabolic process	mitochondrion	catalytic activity;nucleotide binding;RNA binding	12.043	4.38	0.36
P38969	Pentamidine resistance factor, mitochondrial [OS=Saccharomyces cerevisiae S288c]	854440	PNT1; YOR266W	cell organization and biogenesis;response to stimulus	membrane;mitochondrion		0.711	0.259	0.36
P18900	hexaprenyl pyrophosphate synthase, mitochondrial [OS=Saccharomyces cerevisiae S288c]	852288	COQ1; YBR003W	metabolic process	membrane;mitochondrion	catalytic activity;metal ion binding;protein binding	0.711	0.259	0.36
P32898	Mitochondrial presequence protease [OS=Saccharomyces cerevisiae S288c]	852041	CYM1; YDR430C	metabolic process	mitochondrion	catalytic activity;metal ion binding	0.812	0.297	0.37
P25038	Translation initiation factor IF-2, mitochondrial [OS=Saccharomyces cerevisiae S288c]	854135	IFM1; YOL023W	cell organization and biogenesis;metabolic process	mitochondrion;nucleus	catalytic activity;nucleotide binding;RNA binding	0.299	0.11	0.37
P33416	Heat shock protein 78, mitochondrial [OS=Saccharomyces cerevisiae S288c]	851845	HSP78; YDR258C	cell organization and biogenesis;metabolic process;regulation of biological process;response to stimulus	mitochondrion;organelle lumen	catalytic activity;nucleotide binding;protein binding	4.264	1.569	0.37
Q04935	Cytochrome c oxidase protein 20, mitochondrial [OS=Saccharomyces cerevisiae S288c]	851817	COX20; YDR231C	cell organization and biogenesis;metabolic process	membrane;mitochondrion	protein binding	1.894	0.701	0.37
P32445	Single-stranded DNA-binding protein RIM1, mitochondrial [OS=Saccharomyces cerevisiae S288c]	850395	RIM1; YCR028C-A	cell organization and biogenesis;metabolic process;regulation of biological process	mitochondrion	DNA binding	1.783	0.668	0.37
P00044	Cytochrome c iso-1 [OS=Saccharomyces cerevisiae S288c]	853507	CYC1; YJR048W	metabolic process;transport	mitochondrion	metal ion binding;protein binding	12.335	4.623	0.37
P07806	Valine--tRNA ligase, mitochondrial [OS=Saccharomyces cerevisiae S288c]	852986	VAS1; YGR094W	metabolic process;regulation of biological process	cytoplasm;cytosol;mitochondrion	catalytic activity;nucleotide binding	0.668	0.251	0.38
Q08968	UPF0061 protein FMP40 [OS=Saccharomyces cerevisiae S288c]	855879	FMP40; YPL222W	mitochondrion		protein binding	2.162	0.823	0.38
P40053	Altered inheritance of mitochondria protein 9, mitochondrial [OS=Saccharomyces cerevisiae S288c]	856813	AIM9; YER080W	mitochondrion			4.036	1.537	0.38
P53326	Uncharacterized protein YGR266W [OS=Saccharomyces cerevisiae S288c]	853182	YGR266W; YGR266W	membrane;mitochondrion			0.778	0.299	0.38
P39726	Glycine cleavage system H protein, mitochondrial [OS=Saccharomyces cerevisiae S288c]	851254	GCV3; YAL044C	metabolic process	mitochondrion	catalytic activity	6.743	2.594	0.38
P08417	fumarate hydratase, mitochondrial [OS=Saccharomyces cerevisiae S288c]	855866	FUM1; YPL262W	cell organization and biogenesis;metabolic process	cytoplasm;cytosol;mitochondrion;organelle lumen	catalytic activity	8.237	3.175	0.39
P32453	Protein ATP11, mitochondrial [OS=Saccharomyces cerevisiae S288c]	855401	ATP11; YNL315C	cell organization and biogenesis	mitochondrion	protein binding	1.512	0.585	0.39
Q03713	Respiratory supercomplex factor 1, mitochondrial [OS=Saccharomyces cerevisiae S288c]	854978	RCF1; YML030W	cell organization and biogenesis	membrane;mitochondrion		1.512	0.585	0.39
P50088	Stationary phase gene 1 protein [OS=Saccharomyces cerevisiae S288c]	853151	SPG1; YGR236C	endoplasmic reticulum;membrane;mitochondrion			157.489	62.096	0.39

P37292	Serine hydroxymethyltransferase, mitochondrial [OS=Saccharomyces cerevisiae S288c]	852565	SHM1; YBR263W	metabolic process	mitochondrion	catalytic activity	7.697	3.037	0.39
P40452	Cytochrome c oxidase assembly factor 1 [OS=Saccharomyces cerevisiae S288c]	854649	COA1; YIL157C	cell organization and biogenesis	membrane;mitochondrion	protein binding	1.31	0.52	0.40
P39522	Dihydroxy-acid dehydratase, mitochondrial [OS=Saccharomyces cerevisiae S288c]	853473	ILV3; YJR016C	metabolic process	mitochondrion	catalytic activity;metal ion binding	16.475	6.565	0.40
P40496	37S ribosomal protein S25, mitochondrial [OS=Saccharomyces cerevisiae S288c]	854715	RSM25; YIL093C	cell organization and biogenesis;metabolic process	mitochondrion;ribosome	structural molecule activity	1.154	0.468	0.41
P53311	Mitochondrial pyruvate carrier 3 [OS=Saccharomyces cerevisiae S288c]	853158	FMP43; MPC3; YGR243W	transport	membrane;mitochondrion	transporter activity	1.154	0.468	0.41
Q12032	Altered inheritance of mitochondria protein 41, mitochondrial [OS=Saccharomyces cerevisiae S288c]	854390	AIM41; YOR215C	mitochondrion		catalytic activity	1.154	0.468	0.41
P48569	Uncharacterized protein YDL183C [OS=Saccharomyces cerevisiae S288c]	851345	YDL183C; YDL183C	transport			1.069	0.438	0.41
P36163	mitochondrial metalloendopeptidase OMA1 [OS=Saccharomyces cerevisiae S288c]	853962	OMA1; YKR087C	metabolic process	membrane;mitochondrion	catalytic activity;metal ion binding	0.931	0.389	0.42
Q03246	37S ribosomal protein S17, mitochondrial [OS=Saccharomyces cerevisiae S288c]	855226	MRPS17; YMR188C	cell differentiation;cell organization and biogenesis;metabolic process	mitochondrion;ribosome	RNA binding;structural molecule activity	0.931	0.389	0.42
P00431	cytochrome c peroxidase, mitochondrial [OS=Saccharomyces cerevisiae S288c]	853940	CCP1; YKR066C	metabolic process;response to stimulus	mitochondrion;organelle lumen	antioxidant activity;catalytic activity;metal ion binding;protein binding	1.649	0.701	0.43
P16451	Pyruvate dehydrogenase complex protein X component, mitochondrial [OS=Saccharomyces cerevisiae S288c]	853107	PDX1; YGR193C	metabolic process	mitochondrion;organelle lumen	catalytic activity;structural molecule activity	2.3	0.978	0.43
Q02204	54S ribosomal protein L13, mitochondrial [OS=Saccharomyces cerevisiae S288c]	853875	MRPL13; YKR006C	cell organization and biogenesis;metabolic process	mitochondrion;ribosome	structural molecule activity	0.778	0.334	0.43
P46998	mitochondrial membrane protein FMP33 [OS=Saccharomyces cerevisiae S288c]	853279	FMP33; YJL161W	membrane;mitochondrion			0.778	0.334	0.43
Q07914	mitochondrial import inner membrane translocase subunit TIM14 [OS=Saccharomyces cerevisiae S288c]	850694	PAM18; YLR008C	regulation of biological process;transport	membrane;mitochondrion	enzyme regulator activity;protein binding;transporter activity	0.778	0.334	0.43
P40185	Protein mmf1, mitochondrial [OS=Saccharomyces cerevisiae S288c]	854760	MMF1; YIL051C	cell organization and biogenesis;metabolic process	mitochondrion;organelle lumen		6.943	2.981	0.43
P38172	MIOREX complex component 3 [OS=Saccharomyces cerevisiae S288c]	852181	YBL095W; MRX3; YBL095W	membrane;mitochondrion			0.719	0.311	0.43
P53220	Mitochondrial import inner membrane translocase subunit TIM21 [OS=Saccharomyces cerevisiae S288c]	852921	TIM21; YGR033C	cell organization and biogenesis;transport	membrane;mitochondrion	protein binding	0.719	0.311	0.43
P15179	Aspartate--tRNA ligase, mitochondrial [OS=Saccharomyces cerevisiae S288c]	856000	MSD1; YPL104W	metabolic process	cytoplasm;mitochondrion;organelle lumen	catalytic activity;nucleotide binding	0.708	0.307	0.43
P30902	ATP synthase subunit d, mitochondrial [OS=Saccharomyces cerevisiae S288c]	853853	ATP7; YKL016C	metabolic process;transport	membrane;mitochondrion	catalytic activity;transporter activity	6.197	2.728	0.44
P46681	D-lactate dehydrogenase [cytochrome] 2,	851376	DLD2; YDL178W	metabolic process	mitochondrion;organelle lumen	catalytic activity;nucleoti	1.264	0.562	0.44

	mitochondrial [OS=Saccharomyces cerevisiae S288c]					de binding;protein binding			
P38152	Tricarboxylate transport protein [OS=Saccharomyces cerevisiae S288c]	852594	CTP1; YBR291C	metabolic process;transport	membrane;mitochondrion	structural molecule activity;transporter activity	0.551	0.245	0.44
P36528	54S ribosomal protein L17, mitochondrial [OS=Saccharomyces cerevisiae S288c]	855469	MRPL17; YNL252C	cell organization and biogenesis;metabolic process	mitochondrion;ribosome	structural molecule activity	0.52	0.233	0.45
Q07349	MIOREX complex component 9 [OS=Saccharomyces cerevisiae S288c]	851535	YDL027C; MRX9; YDL027C	endoplasmic reticulum;membrane;mitochondrion			2.433	1.096	0.45
P32839	Mitochondrial chaperone BCS1 [OS=Saccharomyces cerevisiae S288c]	851981	BCS1; YDR375C	cell organization and biogenesis;metabolic process;response to stimulus;transport	cytosol;membrane;mitochondrion	catalytic activity;nucleotide binding;transporter activity	0.425	0.194	0.46
Q01976	ADP-ribose pyrophosphatase [OS=Saccharomyces cerevisiae S288c]	852408	YSA1; YBR111C	metabolic process	cytoplasm;mitochondrion;nucleus	catalytic activity;metal ion binding	0.425	0.194	0.46
P03879	Intron-encoded RNA maturase bI4 [OS=Saccharomyces cerevisiae S288c]	854582	BI4; Q0120	metabolic process	membrane;mitochondrion	catalytic activity;RNA binding	0.389	0.179	0.46
P54115	Magnesium-activated aldehyde dehydrogenase, cytosolic [OS=Saccharomyces cerevisiae S288c]	856044	ALD6; YPL061W	metabolic process;response to stimulus	cytoplasm;cytosol;mitochondrion	catalytic activity	0.346	0.16	0.46
P36523	54S ribosomal protein L15, mitochondrial [OS=Saccharomyces cerevisiae S288c]	851022	MRPL15; YLR312W-A	cell organization and biogenesis;metabolic process	mitochondrion;ribosome	catalytic activity;RNA binding;structural molecule activity	0.334	0.155	0.46
P38297	Mitofusin FZO1 [OS=Saccharomyces cerevisiae S288c]	852477	FZO1; YBR179C	cell organization and biogenesis	membrane;mitochondrion	catalytic activity;nucleotide binding;protein binding	0.304	0.142	0.47
Q05931	Heat shock protein SSQ1, mitochondrial [OS=Saccharomyces cerevisiae S288c]	851084	SSQ1; YLR369W	cell organization and biogenesis;metabolic process	mitochondrion;organelle lumen	nucleotide binding;protein binding	0.884	0.413	0.47
Q06089	Uncharacterized mitochondrial outer membrane protein YPR098C [OS=Saccharomyces cerevisiae S288c]	856213	YPR098C; YPR098C	membrane;mitochondrion			4.623	2.162	0.47
Q08223	Altered inheritance of mitochondria protein 39, mitochondrial [OS=Saccharomyces cerevisiae S288c]	854103	AIM39; YOL053W	cytosol;membrane;mitochondrion			0.995	0.468	0.47
P10662	37S ribosomal protein MRP1, mitochondrial [OS=Saccharomyces cerevisiae S288c]	851948	MRP1; YDR347W	cell organization and biogenesis;metabolic process;response to stimulus	mitochondrion;ribosome	antioxidant activity;catalytic activity;metal ion binding;structural molecule activity	1.239	0.585	0.47
P52893	probable alanine aminotransferase, mitochondrial [OS=Saccharomyces cerevisiae S288c]	850778	ALT1; YLR089C	metabolic process	mitochondrion;organelle lumen	catalytic activity	0.951	0.45	0.47
P35191	DnaJ homolog 1, mitochondrial [OS=Saccharomyces cerevisiae S288c]	850530	MDJ1; YFL016C	cell organization and biogenesis;metabolic process;regulation of biological process;response to stimulus	mitochondrion;organelle lumen	enzyme regulator activity;metal ion binding;nucleotide binding;protein binding	2.765	1.31	0.47
P36008	Elongation factor 1-gamma 2 [OS=Saccharomyces cerevisiae S288c]	853781	TEF4; YKL081W	metabolic process;regulation of biological process	cytoplasm;mitochondrion;nucleus;ribosome	catalytic activity;protein binding;RNA binding	0.202	0.096	0.48
Q12029	Probable mitochondrial transport protein fsf1 [OS=Saccharomyces cerevisiae S288c]	854445	FSF1; YOR271C	transport	membrane;mitochondrion	transporter activity	2.793	1.336	0.48
P18409	Mitochondrial distribution and morphology protein 10 [OS=Saccharomyces cerevisiae S288c]	851223	MDM10; YAL010C	cell organization and biogenesis;transport	membrane;mitochondrion	protein binding	0.16	0.077	0.48

P00447	Superoxide dismutase [Mn], mitochondrial [OS=Saccharomyces cerevisiae S288c]	856399	SOD2; YHR008C	metabolic process;response to stimulus	mitochondrion;organelle lumen	antioxidant activity;catalytic activity;metal ion binding	3.924	1.894	0.48
P00401	Cytochrome c oxidase subunit 1 [OS=Saccharomyces cerevisiae S288c]	854598	COX1; Q0045	metabolic process;transport	membrane;mitochondrion	catalytic activity;metal ion binding;transporter activity	1.61	0.778	0.48
Q12480	Probable electron transfer flavoprotein subunit alpha, mitochondrial [OS=Saccharomyces cerevisiae S288c]	856112	AIM45; YPR004C	metabolic process;transport	mitochondrion;organelle lumen	catalytic activity;nucleotide binding	2.548	1.239	0.49
P38714	Arginine--tRNA ligase, mitochondrial [OS=Saccharomyces cerevisiae S288c]	856491	MSR1; YHR091C	cell organization and biogenesis;metabolic process	cytoplasm;mitochondrion;organelle lumen	catalytic activity;nucleotide binding	0.113	0.055	0.49
P28834	Isocitrate dehydrogenase [NAD] subunit 1, mitochondrial [OS=Saccharomyces cerevisiae S288c]	855691	IDH1; YNL037C	metabolic process;transport	cytosol;mitochondrion;organelle lumen	catalytic activity;metal ion binding;nucleotide binding;protein binding;RNA binding	7.859	3.833	0.49
P38825	Protein TOM71 [OS=Saccharomyces cerevisiae S288c]	856517	TOM71; YHR117W	transport	membrane;mitochondrion	protein binding;transporter activity	0.701	0.343	0.49
P43635	Citrate synthase 3, mitochondrial [OS=Saccharomyces cerevisiae S288c]	856107	CIT3; YPR001W	metabolic process	mitochondrion	catalytic activity	3.642	1.783	0.49
P07275	Delta-1-pyrroline-5-carboxylate dehydrogenase, mitochondrial [OS=Saccharomyces cerevisiae S288c]	856432	PUT2; YHR037W	metabolic process	membrane;mitochondrion;organelle lumen	catalytic activity	5.579	2.765	0.50
P32891	D-lactate dehydrogenase [cytochrome] 1, mitochondrial [OS=Saccharomyces cerevisiae S288c]	851380	DLD1; YDL174C	metabolic process;transport	membrane;mitochondrion	catalytic activity;nucleotide binding	11.253	5.661	0.50
P53140	Protein RMD9, mitochondrial [OS=Saccharomyces cerevisiae S288c]	852771	RMD9; YGL107C	cell differentiation;metabolic process;regulation of biological process	membrane;mitochondrion	RNA binding	1.154	0.585	0.51
P32317	Protein AFG1 [OS=Saccharomyces cerevisiae S288c]	856658	AFG1; YEL052W	cell organization and biogenesis;metabolic process;response to stimulus;transport	membrane;mitochondrion	catalytic activity;nucleotide binding	1.154	0.585	0.51
P36525	54S ribosomal protein L24, mitochondrial [OS=Saccharomyces cerevisiae S288c]	855231	MRPL24; YMR193W	cell organization and biogenesis;metabolic process	mitochondrion;ribosome	RNA binding;structural molecule activity	1.154	0.585	0.51
P47052	succinate dehydrogenase [ubiquinone] flavoprotein subunit 2, mitochondrial [OS=Saccharomyces cerevisiae S288c]	853405	YJL045W; YJL045W	metabolic process;transport	membrane;mitochondrion	catalytic activity;nucleotide binding	4.043	2.065	0.51
Q12031	Mitochondrial 2-methylisocitrate lyase [OS=Saccharomyces cerevisiae S288c]	856114	ICL2; YPR006C	metabolic process	mitochondrion;organelle lumen	catalytic activity	3.833	1.976	0.52
P40008	Protein FMP52, mitochondrial [OS=Saccharomyces cerevisiae S288c]	856721	FMP52; YER004W	endoplasmic reticulum;membrane;mitochondrion		catalytic activity	2.652	1.371	0.52
P40086	Cytochrome c oxidase assembly protein cox15 [OS=Saccharomyces cerevisiae S288c]	856884	COX15; YER141W	cell organization and biogenesis;metabolic process;transport	membrane;mitochondrion	catalytic activity;transporter activity	2.594	1.346	0.52
P40098	uncharacterized mitochondrial membrane protein FMP10 [OS=Saccharomyces cerevisiae S288c]	856931	FMP10; YER182W	membrane;mitochondrion			4.456	2.36	0.53
P43617	Uncharacterized mitochondrial carrier YFR045W [OS=Saccharomyces cerevisiae S288c]	850606	YFR045W; YFR045W	metabolic process;transport	membrane;mitochondrion	structural molecule activity;transporter activity	0.778	0.413	0.53
P38891	Branched-chain-amino-acid aminotransferase, mitochondrial [OS=Saccharomyces	856615	BAT1; YHR208W	metabolic process;regulation of biological process;response to	mitochondrion;organelle lumen	catalytic activity	7.111	3.806	0.54

	cerevisiae S288c]			stimulus					
P10834	protein PET54 [OS=Saccharomyces cerevisiae S288c]	853137	PET54; YGR222W	metabolic process;regulation of biological process	membrane;mitochondrion;organelle lumen	nucleotide binding;RNA binding;translation regulator activity	0.688	0.369	0.54
P19262	Dihydrolipoyllysine-residue succinyltransferase component of 2-oxoglutarate dehydrogenase complex, mitochondrial [OS=Saccharomyces cerevisiae S288c]	851726	KGD2; YDR148C	cell organization and biogenesis;metabolic process	mitochondrion	catalytic activity;protein binding	5.7	3.062	0.54
Q05648	MIOREX complex component 10 [OS=Saccharomyces cerevisiae S288c]	851876	YDR282C; MRX10; YDR282C	membrane;mitochondrion			0.65	0.35	0.54
P34227	Mitochondrial peroxiredoxin PRX1 [OS=Saccharomyces cerevisiae S288c]	852215	PRX1; YBL064C	cellular homeostasis;metabolic process;regulation of biological process;response to stimulus	mitochondrion	antioxidant activity;catalytic activity	5.158	2.793	0.54
P40961	prohibitin-1 [OS=Saccharomyces cerevisiae S288c]	853033	PHB1; YGR132C	cell organization and biogenesis;metabolic process;regulation of biological process	membrane;mitochondrion	protein binding	5.158	2.793	0.54
P39006	Phosphatidylserine decarboxylase proenzyme 1, mitochondrial [OS=Saccharomyces cerevisiae S288c]	855552	PSD1; YNL169C	metabolic process;regulation of biological process	membrane;mitochondrion	catalytic activity	0.487	0.269	0.55
Q96VH5	MICOS complex subunit Mic10 [OS=Saccharomyces cerevisiae S288c]	850300	MOS1; MIC10; YCL057C-A	cell organization and biogenesis	membrane;mitochondrion		1.683	0.931	0.55
P42940	Probable electron transfer flavoprotein subunit beta [OS=Saccharomyces cerevisiae S288c]	853121	CIR1; YGR207C	metabolic process;transport	mitochondrion;organelle lumen		3.87	2.162	0.56
Q04472	Mitochondrial inner membrane i-AAA protease supercomplex subunit MGR3 [OS=Saccharomyces cerevisiae S288c]	855142	MGR3; YMR115W	metabolic process	membrane;mitochondrion	protein binding	0.417	0.233	0.56
Q06567	ABC1 family protein MCP2 [OS=Saccharomyces cerevisiae S288c]	850955	YLR253W; MCP2; YLR253W	cell organization and biogenesis	membrane;mitochondrion		0.389	0.218	0.56
P09457	ATP synthase subunit 5, mitochondrial [OS=Saccharomyces cerevisiae S288c]	851892	ATP5; YDR298C	metabolic process;transport	membrane;mitochondrion	catalytic activity;transporter activity	10.365	5.813	0.56
P37299	cytochrome b-c1 complex subunit 10 [OS=Saccharomyces cerevisiae S288c]	856390	QCR10; YHR001W-A	metabolic process;transport	membrane;mitochondrion	catalytic activity;transporter activity	5.31	2.981	0.56
Q12374	Nuclear control of ATPase protein 2 [OS=Saccharomyces cerevisiae S288c]	856278	NCA2; YPR155C	metabolic process	membrane;mitochondrion		0.668	0.377	0.56
P40047	Aldehyde dehydrogenase 5, mitochondrial [OS=Saccharomyces cerevisiae S288c]	856804	ALD5; YER073W	metabolic process	mitochondrion;organelle lumen	catalytic activity	1.955	1.106	0.57
Q12233	ATP synthase subunit g, mitochondrial [OS=Saccharomyces cerevisiae S288c]	856131	ATP20; YPR020W	cell organization and biogenesis;metabolic process;transport	membrane;mitochondrion	catalytic activity;transporter activity	1.371	0.778	0.57
P36147	Presequence translocated-associated motor subunit pam17, mitochondrial [OS=Saccharomyces cerevisiae S288c]	853939	PAM17; YKR065C	transport	membrane;mitochondrion		1.371	0.778	0.57
Q12428	Probable 2-methylcitrate dehydratase [OS=Saccharomyces cerevisiae S288c]	856108	PDH1; YPR002W	metabolic process	cytoplasm;membrane;mitochondrion	catalytic activity	12.335	7.058	0.57
P53732	37S ribosomal protein S12, mitochondrial [OS=Saccharomyces cerevisiae S288c]	855772	MRPS12; YNR036C	cell organization and biogenesis;metabolic process	mitochondrion;ribosome	structural molecule activity	1.154	0.668	0.58

P36775	Lon protease homolog, mitochondrial [OS=Saccharomyces cerevisiae S288c]	852259	PIM1; YBL022C	cell organization and biogenesis;metabolic process;regulation of biological process;response to stimulus	cytoplasm;mitochondrion;organelle lumen	catalytic activity;DNA binding;nucleotide binding;RNA binding	0.685	0.402	0.59
P53598	Succinyl-CoA ligase [ADP-forming] subunit alpha, mitochondrial [OS=Saccharomyces cerevisiae S288c]	854310	LSC1; YOR142W	metabolic process	cytosol;mitochondrion	catalytic activity;nucleotide binding	13.384	7.859	0.59
Q06143	mitochondrial dicarboxylate transporter [OS=Saccharomyces cerevisiae S288c]	851063	DIC1; YLR348C	metabolic process;transport	membrane;mitochondrion	structural molecule activity;transporter activity	0.931	0.551	0.59
P00410	Cytochrome c oxidase subunit 2 [OS=Saccharomyces cerevisiae S288c]	854622	COX2; Q0250	metabolic process;transport	membrane;mitochondrion	catalytic activity;metal ion binding;protein binding;transporter activity	3.642	2.162	0.59
P07246	Alcohol dehydrogenase 3, mitochondrial [OS=Saccharomyces cerevisiae S288c]	855107	ADH3; YMR083W	metabolic process	mitochondrion;organelle lumen	catalytic activity;metal ion binding	7.031	4.179	0.59
Q08023	Protein FMP25, mitochondrial [OS=Saccharomyces cerevisiae S288c]	850766	FMP25; YLR077W	cell organization and biogenesis	membrane;mitochondrion		1.412	0.84	0.59
P53292	37S ribosomal protein S35, mitochondrial [OS=Saccharomyces cerevisiae S288c]	853075	MRPS35; YGR165W	cell organization and biogenesis;metabolic process	mitochondrion;ribosome	structural molecule activity	1.512	0.905	0.60
P09624	Dihydrolipoyl dehydrogenase, mitochondrial [OS=Saccharomyces cerevisiae S288c]	850527	LPD1; YFL018C	cellular homeostasis;metabolic process;regulation of biological process	mitochondrion;organelle lumen	catalytic activity;nucleotide binding	21.539	13.03	0.60
P32843	Mitochondrial escape protein 2 [OS=Saccharomyces cerevisiae S288c]	855348	YME2; YMR302C	cell organization and biogenesis;metabolic process	membrane;mitochondrion	nucleotide binding;RNA binding	3.458	2.099	0.61
Q02950	37S ribosomal protein MRP51, mitochondrial [OS=Saccharomyces cerevisiae S288c]	855985	MRP51; YPL118W	cell organization and biogenesis;metabolic process	mitochondrion;ribosome	structural molecule activity	0.668	0.407	0.61
P32611	54S ribosomal protein RML2, mitochondrial [OS=Saccharomyces cerevisiae S288c]	856660	RML2; YEL050C	cell organization and biogenesis;metabolic process	mitochondrion;ribosome	catalytic activity;RNA binding;structural molecule activity	0.638	0.389	0.61
P23180	Probable oxidoreductase AIM17 [OS=Saccharomyces cerevisiae S288c]	856365	AIM17; YHL021C	cell organization and biogenesis;metabolic process	mitochondrion	catalytic activity;metal ion binding	2.981	1.818	0.61
P33311	ATP-dependent permease MDL2, mitochondrial [OS=Saccharomyces cerevisiae S288c]	855858	MDL2; YPL270W	response to stimulus;transport	membrane;mitochondrion	catalytic activity;nucleotide binding;transporter activity	1.006	0.619	0.62
Q12375	mitochondrial ornithine transporter 1 [OS=Saccharomyces cerevisiae S288c]	854297	ORT1; YOR130C	metabolic process;transport	membrane;mitochondrion	structural molecule activity;transporter activity	0.54	0.334	0.62
P14693	sorting assembly machinery 35 kDa subunit [OS=Saccharomyces cerevisiae S288c]	856483	SAM35; YHR083W	cell organization and biogenesis;transport	membrane;mitochondrion	protein binding	0.54	0.334	0.62
P38071	Enoyl-[acyl-carrier protein] reductase [NADPH, B-specific], mitochondrial [OS=Saccharomyces cerevisiae S288c]	852314	ETR1; YBR026C	metabolic process	mitochondrion;organelle lumen	catalytic activity;DNA binding;metal ion binding	9	5.579	0.62
P53889	uncharacterized mitochondrial hydrolase FMP41 [OS=Saccharomyces cerevisiae S288c]	855553	FMP41; YNL168C	metabolic process	mitochondrion	catalytic activity;metal ion binding	0.501	0.311	0.62
Q03798	Altered inheritance of mitochondria protein 36, mitochondrial [OS=Saccharomyces cerevisiae S288c]	855189	AIM36; YMR157C	membrane;mitochondrion			1.336	0.833	0.62
P19516	Cytochrome c oxidase assembly protein COX11, mitochondrial [OS=Saccharomyces cerevisiae S288c]	855971	COX11; YPL132W	cell organization and biogenesis;metabolic process	membrane;mitochondrion;ribosome	metal ion binding	0.438	0.274	0.63

P38271	Protein OPY1 [OS=Saccharomyces cerevisiae S288c]	852426	OPY1; YBR129C	cytoplasm;mitochondrion			0.438	0.274	0.63
P48015	Aminomethyltransferase, mitochondrial [OS=Saccharomyces cerevisiae S288c]	851582	GCV1; YDR019C	metabolic process	mitochondrion	catalytic activity;protein binding	8.047	5.062	0.63
Q07500	External NADH-ubiquinone oxidoreductase 2, mitochondrial [OS=Saccharomyces cerevisiae S288c]	851474	NDE2; YDL085W	metabolic process	mitochondrion	catalytic activity;nucleotide binding	6.356	4.012	0.63
P32792	UPF0744 protein YSC83 [OS=Saccharomyces cerevisiae S288c]	856410	YSC83; YHR017W	membrane;mitochondrion			0.369	0.233	0.63
P47127	Altered inheritance of mitochondria protein 24, mitochondrial [OS=Saccharomyces cerevisiae S288c]	853543	AIM24; YJR080C	cell organization and biogenesis	mitochondrion		2.857	1.807	0.63
P37293	N-terminal acetyltransferase 2 [OS=Saccharomyces cerevisiae S288c]	853050	NAT2; YGR147C	metabolic process	cytoplasm;mitochondrion	catalytic activity	1.154	0.73	0.63
P40015	inositol phosphosphingolipids phospholipase C [OS=Saccharomyces cerevisiae S288c]	856739	ISC1; YER019W	metabolic process;response to stimulus	endoplasmic reticulum;membrane;mitochondrion	catalytic activity;metal ion binding	0.334	0.212	0.63
Q00711	Succinate dehydrogenase [ubiquinone] flavoprotein subunit, mitochondrial [OS=Saccharomyces cerevisiae S288c]	853709	SDH1; YKL148C	metabolic process;transport	membrane;mitochondrion	catalytic activity;nucleotide binding;protein binding	8.454	5.381	0.64
Q01532-1	Cysteine proteinase 1, mitochondrial [OS=Saccharomyces cerevisiae S288c]		MCY1	metabolic process;regulation of biological process;response to stimulus	cytoplasm;mitochondrion	catalytic activity;DNA binding;RNA binding	0.28	0.179	0.64
P41921	glutathione reductase [OS=Saccharomyces cerevisiae S288c]	856014	GLR1; YPL091W	cellular homeostasis;metabolic process;regulation of biological process;response to stimulus	cytoplasm;cytosol;mitochondrion	antioxidant activity;catalytic activity;nucleotide binding	0.241	0.155	0.64
P38705	Serine--tRNA ligase, mitochondrial [OS=Saccharomyces cerevisiae S288c]	856402	DIA4; YHR011W	metabolic process	cytoplasm;mitochondrion;organelle lumen	catalytic activity;nucleotide binding	0.233	0.15	0.64
P07236	Threonine--tRNA ligase, mitochondrial [OS=Saccharomyces cerevisiae S288c]	853640	MST1; YKL194C	metabolic process	cytoplasm;mitochondrion;organelle lumen	catalytic activity;nucleotide binding;RNA binding	0.233	0.15	0.64
P53266	Cytochrome oxidase assembly protein SHY1 [OS=Saccharomyces cerevisiae S288c]	853009	SHY1; YGR112W	cell organization and biogenesis	membrane;mitochondrion	protein binding	1.783	1.154	0.65
P32340	Rotenone-insensitive NADH-ubiquinone oxidoreductase, mitochondrial [OS=Saccharomyces cerevisiae S288c]	854919	NDI1; YML120C	metabolic process;regulation of biological process	membrane;mitochondrion;organelle lumen	catalytic activity;nucleotide binding	16.433	10.721	0.65
P36013	NAD-dependent malic enzyme, mitochondrial [OS=Saccharomyces cerevisiae S288c]	853839	MAE1; YKL029C	metabolic process	mitochondrion;organelle lumen	catalytic activity;metal ion binding;nucleotide binding	1.512	0.995	0.66
P38127	mitochondrial carrier protein RIM2 [OS=Saccharomyces cerevisiae S288c]	852491	RIM2; YBR192W	cell organization and biogenesis;metabolic process;transport	membrane;mitochondrion	structural molecule activity;transporter activity	1.512	0.995	0.66
Q06668	Methyltransferase OMS1, mitochondrial [OS=Saccharomyces cerevisiae S288c]	851911	OMS1; YDR316W	metabolic process	membrane;mitochondrion	catalytic activity	0.585	0.389	0.66
P04710	ADP,ATP carrier protein 1 [OS=Saccharomyces cerevisiae S288c]	855078	AAC1; YMR056C	metabolic process;transport	cytosol;membrane;mitochondrion	structural molecule activity;transporter activity	10.288	6.848	0.67
P23641	mitochondrial phosphate carrier protein [OS=Saccharomyces cerevisiae S288c]	853540	MIR1; YJR077C	metabolic process;transport	membrane;mitochondrion	protein binding;structural molecule activity;transporter activity	20.544	13.678	0.67
P08067	cytochrome b-c1 complex subunit Rieske, mitochondrial	856689	RIP1; YEL024W	metabolic process;transport	membrane;mitochondrion	catalytic activity;metal ion	25.102	16.783	0.67

	[OS=Saccharomyces cerevisiae S288c]					binding;transpo rter activity			
P38910	10 kDa heat shock protein, mitochondrial [OS=Saccharomyces cerevisiae S288c]	85418 5	HSP10; YOR020C	cell organization and biogenesis;metabolic process;response to stimulus;transport	cytoplasm;mitochondrion;organelle lumen	metal ion binding;nucleoti de binding;protein binding	3.217	2.162	0.67
P00830	ATP synthase subunit beta, mitochondrial [OS=Saccharomyces cerevisiae S288c]	85358 5	ATP2; YJR121W	metabolic process;transport	cytosol;membrane;mitochondrion	catalytic activity;nucleoti de binding;protein binding;transpo rter activity	38.81 1	26.12 3	0.67
P21560	Protein CBP3, mitochondrial [OS=Saccharomyces cerevisiae S288c]	85588 6	CBP3; YPL215W	cell organization and biogenesis;regulation of biological process	membrane;mitochondrion;ribosome		1.031	0.701	0.68
P15108	ATP-dependent molecular chaperone HSC82 [OS=Saccharomyces cerevisiae S288c]	85522 4	HSC82; YMR186W	cell organization and biogenesis;metabolic process;response to stimulus	cytoplasm;membrane;mitochondrion	catalytic activity;nucleoti de binding;protein binding	1.565	1.081	0.69
P49095	Glycine dehydrogenase (Decarboxylating), mitochondrial [OS=Saccharomyces cerevisiae S288c]	85522 7	GCV2; YMR189W	metabolic process	cytosol;mitochondrion	catalytic activity	1.575	1.096	0.70
P36046	Mitochondrial intermembrane space import and assembly protein 40 [OS=Saccharomyces cerevisiae S288c]	85363 9	MIA40; YKL195W	cellular component movement;metabolic process;response to stimulus;transport	membrane;mitochondrion	catalytic activity;protein binding;transpo rter activity	2.914	2.03	0.70
P32387	54S ribosomal protein L41, mitochondrial [OS=Saccharomyces cerevisiae S288c]	85201 4	MRP20; YDR405W	cell organization and biogenesis;metabolic process	mitochondrion;ribosome	nucleotide binding;protein binding;RNA binding;structur al molecule activity	0.719	0.501	0.70
P32191	Glycerol-3-phosphate dehydrogenase, mitochondrial [OS=Saccharomyces cerevisiae S288c]	85465 1	GUT2; YIL155C	metabolic process	membrane;mitochondrion	catalytic activity	6.097	4.289	0.70
Q12251	Uncharacterized mitochondrial carrier YPR011C [OS=Saccharomyces cerevisiae S288c]	85612 1	YPR011C; YPR011C	metabolic process;transport	membrane;mitochondrion	structural molecule activity;transpo rter activity	0.551	0.389	0.71
P40035	Mitochondrial phosphate carrier protein 2 [OS=Saccharomyces cerevisiae S288c]	85677 9	PIC2; YER053C	cellular homeostasis;metabolic process;transport	membrane;mitochondrion	structural molecule activity;transpo rter activity	2.793	1.976	0.71
Q04458	Fatty aldehyde dehydrogenase HFD1 [OS=Saccharomyces cerevisiae S288c]	85513 7	HFD1; YMR110C	metabolic process	endoplasmic reticulum;endosome;membrane;mitochondri on	catalytic activity	3.642	2.594	0.71
P38702	mitochondrial carrier protein LEU5 [OS=Saccharomyces cerevisiae S288c]	85639 1	LEU5; YHR002W	metabolic process;transport	membrane;mitochondrion	structural molecule activity;transpo rter activity	0.468	0.334	0.71
Q08970	Mitochondrial metal transporter 2 [OS=Saccharomyces cerevisiae S288c]	85587 7	MMT2; YPL224C	cellular homeostasis;regulation of biological process;response to stimulus;transport	membrane;mitochondrion	transporter activity	0.407	0.292	0.72
P39925	Mitochondrial respiratory chain complexes assembly protein AFG3 [OS=Saccharomyces cerevisiae S288c]	85673 7	AFG3; YER017C	cell organization and biogenesis;metabolic process;response to stimulus;transport	membrane;mitochondrion	catalytic activity;metal ion binding;nucleoti de binding;protein binding	1.015	0.734	0.72
P33421	Succinate dehydrogenase [ubiquinone] cytochrome b subunit, mitochondrial [OS=Saccharomyces cerevisiae S288c]	85371 6	SDH3; YKL141W	cell organization and biogenesis;metabolic process;transport	membrane;mitochondrion	catalytic activity;metal ion binding	1.276	0.931	0.73
Q12166	2-isopropylmalate synthase 2, mitochondrial [OS=Saccharomyces cerevisiae S288c]	85427 5	LEU9; YOR108W	metabolic process	mitochondrion	catalytic activity;protein binding	1.707	1.246	0.73
P19414	Aconitate hydratase, mitochondrial [OS=Saccharomyces cerevisiae S288c]	85101 3	ACO1; YLR304C	cell organization and biogenesis;metabolic process;transport	cytoplasm;cytosol;mitochondrion;organelle lumen	catalytic activity;DNA binding;metal ion binding	24.88 5	18.16 9	0.73

P40215	External NADH-ubiquinone oxidoreductase 1, mitochondrial [OS=Saccharomyces cerevisiae S288c]	855176	NDE1; YMR145C	metabolic process	mitochondrion	catalytic activity;nucleotide binding	5.078	3.739	0.74
P32904	54S ribosomal protein L6, mitochondrial [OS=Saccharomyces cerevisiae S288c]	856552	MRPL6; YHR147C	cell organization and biogenesis;metabolic process	mitochondrion;ribosome	RNA binding;structural molecule activity	1.054	0.778	0.74
P06208-1	2-isopropylmalate synthase [OS=Saccharomyces cerevisiae S288c]	855619	LEU4; YNL104C	metabolic process	cytoplasm;mitochondrion	catalytic activity;protein binding	4.223	3.125	0.74
P23644	Mitochondrial import receptor subunit TOM40 [OS=Saccharomyces cerevisiae S288c]	855243	TOM40; YMR203W	transport	cytosol;membrane;mitochondrion	protein binding;transporter activity	0.896	0.668	0.75
P38523	GrpE protein homolog, mitochondrial [OS=Saccharomyces cerevisiae S288c]	854407	MGE1; YOR232W	metabolic process;regulation of biological process;transport	membrane;mitochondrion;organelle lumen	enzyme regulator activity;nucleotide binding;protein binding	0.833	0.624	0.75
P07256	Cytochrome b-c1 complex subunit 1, mitochondrial [OS=Saccharomyces cerevisiae S288c]	852235	COR1; YBL045C	metabolic process;transport	membrane;mitochondrion	catalytic activity;metal ion binding;protein binding;transporter activity	9.89	7.432	0.75
P36534	54S ribosomal protein L40, mitochondrial [OS=Saccharomyces cerevisiae S288c]	855930	MRPL40; YPL173W	cell organization and biogenesis;metabolic process	mitochondrion;ribosome	protein binding;structural molecule activity	0.778	0.585	0.75
P32473	Pyruvate dehydrogenase E1 component subunit beta, mitochondrial [OS=Saccharomyces cerevisiae S288c]	852522	PDB1; YBR221C	metabolic process	mitochondrion;organelle lumen	catalytic activity	6.848	5.158	0.75
P53736	Uncharacterized protein YNR040W [OS=Saccharomyces cerevisiae S288c]	855776	YNR040W; YNR040W	mitochondrion			0.73	0.551	0.75
P50085	Prohibitin-2 [OS=Saccharomyces cerevisiae S288c]	853146	PHB2; YGR231C	cell organization and biogenesis;metabolic process;regulation of biological process	membrane;mitochondrion	protein binding	3.806	2.899	0.76
P07251	ATP synthase subunit alpha, mitochondrial [OS=Saccharomyces cerevisiae S288c]	852177	ATP1; YBL099W	metabolic process;transport	cytosol;membrane;mitochondrion	catalytic activity;nucleotide binding;transporter activity	26.826	20.544	0.77
P36112	MICOS complex subunit Mic60 [OS=Saccharomyces cerevisiae S288c]	853886	FCJ1; MIC60; YKR016W	cell organization and biogenesis;transport	membrane;mitochondrion		15.379	11.798	0.77
P40508	Uncharacterized protein YIL077C [OS=Saccharomyces cerevisiae S288c]	854733	YIL077C; YIL077C	mitochondrion			1.154	0.896	0.78
Q01574	acetyl-coenzyme A synthetase 1 [OS=Saccharomyces cerevisiae S288c]	851245	ACS1; YAL054C	cell organization and biogenesis;metabolic process	cytoplasm;cytosol;endoplasmic reticulum;mitochondrion;nucleus	catalytic activity;nucleotide binding	3.281	2.57	0.78
P40012	protoporphyrinogen oxidase [OS=Saccharomyces cerevisiae S288c]	856733	HEM14; YER014W	metabolic process	membrane;mitochondrion	catalytic activity	1.198	0.947	0.79
P36101	tRNA threonylcarbamoyladenosine dehydratase 2 [OS=Saccharomyces cerevisiae S288c]	853841	TCD2; YKL027W; YKL027W	metabolic process	cytosol;membrane;mitochondrion	catalytic activity;nucleotide binding	0.778	0.616	0.79
P10507	mitochondrial-processing peptidase subunit beta [OS=Saccharomyces cerevisiae S288c]	850860	MAS1; YLR163C	metabolic process;transport	mitochondrion;organelle lumen	catalytic activity;metal ion binding;protein binding	0.778	0.616	0.79
P53969	Sorting assembly machinery 50 kDa subunit [OS=Saccharomyces cerevisiae S288c]	855705	SAM50; YNL026W	cell organization and biogenesis;transport	membrane;mitochondrion	protein binding;transporter activity	0.61	0.487	0.80
Q02981	ABC1 family protein YPL109C, mitochondrial [OS=Saccharomyces cerevisiae S288c]	855995	YPL109C; YPL109C	mitochondrion			0.413	0.334	0.81
P46367	Potassium-activated aldehyde dehydrogenase, mitochondrial	854556	ALD4; YOR374W	metabolic process	mitochondrion;organelle lumen	catalytic activity	24.119	19.62	0.81

	[OS=Saccharomyces cerevisiae S288c]								
P47045	mitochondrial import inner membrane translocase subunit TIM54 [OS=Saccharomyces cerevisiae S288c]	853392	TIM54; YJL054W	cell organization and biogenesis;transport	cytosol;membrane;mitochondrion	transporter activity	0.655	0.54	0.82
P25573	Mitochondrial inner membrane i-AAA protease supercomplex subunit MGR1 [OS=Saccharomyces cerevisiae S288c]	850313	MGR1; YCL044C	metabolic process	membrane;mitochondrion	protein binding	1.031	0.859	0.83
P53312	Succinyl-CoA ligase [ADP-forming] subunit beta, mitochondrial [OS=Saccharomyces cerevisiae S288c]	853159	LSC2; YGR244C	metabolic process	mitochondrion	catalytic activity;nucleotide binding	7.003	5.898	0.84
P40416	Iron-sulfur clusters transporter ATM1, mitochondrial [OS=Saccharomyces cerevisiae S288c]	855347	ATM1; YMR301C	cellular homeostasis;metabolic process;transport	membrane;mitochondrion	catalytic activity;nucleotide binding;transporter activity	0.645	0.546	0.85
P00360	glyceraldehyde-3-phosphate dehydrogenase 1 [OS=Saccharomyces cerevisiae S288c]	853395	TDH1; YJL052W	metabolic process	cytoplasm;cytosol;membrane;mitochondrion	catalytic activity;nucleotide binding	1.721	1.462	0.85
P53206	putative cysteine synthase [OS=Saccharomyces cerevisiae S288c]	852895	YGR012W; YGR012W	metabolic process		catalytic activity	1.346	1.154	0.86
P07342	Acetolactate synthase catalytic subunit, mitochondrial [OS=Saccharomyces cerevisiae S288c]	855135	ILV2; YMR108W	metabolic process	mitochondrion	catalytic activity;metal ion binding;nucleotide binding	4.223	3.642	0.86
P21801	Succinate dehydrogenase [ubiquinone] iron-sulfur subunit, mitochondrial [OS=Saccharomyces cerevisiae S288c]	850685	SDH2; YLL041C	metabolic process;transport	membrane;mitochondrion	catalytic activity;metal ion binding	18.684	16.191	0.87
P54783	D-arabinono-1,4-lactone oxidase [OS=Saccharomyces cerevisiae S288c]	854888	ALO1; YML086C	metabolic process;response to stimulus	membrane;mitochondrion	catalytic activity;nucleotide binding	3.299	2.981	0.90
P06168	ketol-acid reductoisomerase, mitochondrial [OS=Saccharomyces cerevisiae S288c]	851069	ILV5; YLR355C	cell organization and biogenesis;metabolic process	mitochondrion	catalytic activity;DNA binding;metal ion binding	9	8.211	0.91
P12695	Dihydrolipoyllysine-residue acetyltransferase component of pyruvate dehydrogenase complex, mitochondrial [OS=Saccharomyces cerevisiae S288c]	855653	LAT1; YNL071W	metabolic process	mitochondrion;organelle lumen	catalytic activity	5.898	5.404	0.92
P16387	Pyruvate dehydrogenase E1 component subunit alpha, mitochondrial [OS=Saccharomyces cerevisiae S288c]	856925	PDA1; YER178W	cell growth;metabolic process	mitochondrion;organelle lumen	catalytic activity	12.46	11.496	0.92
P0CS90	Heat shock protein SSC1, mitochondrial [OS=Saccharomyces cerevisiae S288c]	853503	SSC1; YJR045C	cell organization and biogenesis;metabolic process;regulation of biological process;transport	membrane;mitochondrion;nucleus;organelle lumen	catalytic activity;enzyme regulator activity;nucleotide binding;protein binding	26.826	25.102	0.94
P07257	Cytochrome b-c1 complex subunit 2, mitochondrial [OS=Saccharomyces cerevisiae S288c]	856321	QCR2; YPR191W	metabolic process;transport	membrane;mitochondrion	catalytic activity;metal ion binding;protein binding;transporter activity	21.19	21.19	1.00
P00560	Phosphoglycerate kinase [OS=Saccharomyces cerevisiae S288c]	850370	PGK1; YCR012W	metabolic process	cytoplasm;membrane;mitochondrion	catalytic activity;nucleotide binding	4.843	4.843	1.00
Q01852	Mitochondrial import inner membrane translocase subunit TIM44 [OS=Saccharomyces cerevisiae S288c]	854790	TIM44; YIL022W	transport	membrane;mitochondrion	nucleotide binding;protein binding	4.125	4.125	1.00
P36060	NADH-cytochrome b5 reductase 2 [OS=Saccharomyces cerevisiae S288c]	853707	MCR1; YKL150W	metabolic process;response to stimulus	membrane;mitochondrion	catalytic activity;protein binding	10.288	10.288	1.00

P28241	Isocitrate dehydrogenase [NAD] subunit 2, mitochondrial [OS=Saccharomyces cerevisiae S288c]	854303	IDH2; YOR136W	metabolic process	mitochondrion;organelle lumen	catalytic activity;metal ion binding;nucleotide binding;protein binding;RNA binding	4.179	4.179	1.00
Q07651	SUR7 family protein FMP45 [OS=Saccharomyces cerevisiae S288c]	851304	FMP45; YDL222C	cell differentiation;cell organization and biogenesis	membrane;mitochondrion		6.017	6.017	1.00
P09440	C-1-tetrahydrofolate synthase, mitochondrial [OS=Saccharomyces cerevisiae S288c]	852378	MIS1; YBR084W	metabolic process	cytosol;mitochondrion	catalytic activity;nucleotide binding	0.9	0.9	1.00
P36148	glycerol-3-phosphate O-acyltransferase 2 [OS=Saccharomyces cerevisiae S288c]	853941	GPT2; YKR067W	metabolic process	cytoplasm;endoplasmic reticulum;membrane	catalytic activity	1.116	1.116	1.00
P32335	Protein MSS51, mitochondrial [OS=Saccharomyces cerevisiae S288c]	850900	MSS51; YLR203C	metabolic process;regulation of biological process	membrane;mitochondrion	protein binding;translation regulator activity	1.154	1.154	1.00
P32332	Mitochondrial oxaloacetate transport protein [OS=Saccharomyces cerevisiae S288c]	853739	OAC1; YKL120W	metabolic process;transport	membrane;mitochondrion	structural molecule activity;transporter activity	2.594	2.594	1.00
P53721	Respiratory supercomplex factor 2, mitochondrial [OS=Saccharomyces cerevisiae S288c]	855752	RCF2; YNR018W	cell organization and biogenesis	membrane;mitochondrion		7.111	7.111	1.00
P38325	Mitochondrial outer membrane protein OM14 [OS=Saccharomyces cerevisiae S288c]	852531	OM14; YBR230C	transport	membrane;mitochondrion		6.197	6.197	1.00
P38626	NADH-cytochrome b5 reductase 1 [OS=Saccharomyces cerevisiae S288c]	854768	CBR1; YIL043C	metabolic process	endoplasmic reticulum;membrane;mitochondrion;nucleus	catalytic activity	1.61	1.61	1.00
P40364	Mitochondrial peculiar membrane protein 1 [OS=Saccharomyces cerevisiae S288c]	853379	MPM1; YJL066C	membrane;mitochondrion			1.512	1.512	1.00
P32266	Dynamin-like GTPase MGM1, mitochondrial [OS=Saccharomyces cerevisiae S288c]	854386	MGM1; YOR211C	cell organization and biogenesis	membrane;mitochondrion	catalytic activity;nucleotide binding;protein binding	0.389	0.389	1.00
P32331	Carrier protein YMC1, mitochondrial [OS=Saccharomyces cerevisiae S288c]	856171	YMC1; YPR058W	metabolic process;transport	membrane;mitochondrion;vacuole	structural molecule activity;transporter activity	1.254	1.254	1.00
Q06493	LETM1 domain-containing protein YLH47, mitochondrial [OS=Saccharomyces cerevisiae S288c]	856243	YLH47; YPR125W	cell organization and biogenesis;transport	membrane;mitochondrion		0.61	0.61	1.00
P38072	Protein SCO2, mitochondrial [OS=Saccharomyces cerevisiae S288c]	852312	SCO2; YBR024W	cell organization and biogenesis;cellular homeostasis;metabolic process;transport	membrane;mitochondrion	antioxidant activity;catalytic activity;metal ion binding	0.73	0.73	1.00
P21375	Fumarate reductase 2 [OS=Saccharomyces cerevisiae S288c]	853510	OSM1; YJR051W	metabolic process	endoplasmic reticulum;mitochondrion	catalytic activity;nucleotide binding	0.346	0.346	1.00
Q03976	37S ribosomal protein S24, mitochondrial [OS=Saccharomyces cerevisiae S288c]	851755	RSM24; YDR175C	cell organization and biogenesis;metabolic process	mitochondrion;ribosome	structural molecule activity	0.585	0.585	1.00
P27697	Atypical kinase COQ8, mitochondrial [OS=Saccharomyces cerevisiae S288c]	852758	COQ8; YGL119W	metabolic process	membrane;mitochondrion;organelle lumen	catalytic activity;nucleotide binding	0.365	0.365	1.00
Q99297	Mitochondrial 2-oxodicarboxylate carrier 2 [OS=Saccharomyces cerevisiae S288c]	854397	ODC2; YOR222W	metabolic process;transport	membrane;mitochondrion	structural molecule activity;transporter activity	0.778	0.778	1.00
Q06485	Autophagy-related protein 33 [OS=Saccharomyces cerevisiae S288c]	851070	ATG33; YLR356W	cell communication;metabolic process;response to stimulus	membrane;mitochondrion		1.371	1.371	1.00
P50087	MICOS subunit MIC26 [OS=Saccharomyces cerevisiae S288c]	853150	MOS2; MIC26; YGR235C	cell organization and biogenesis	membrane;mitochondrion		0.931	0.931	1.00
Q12298	uncharacterized ABC transporter ATP-binding protein YDR061W [OS=Saccharomyces	851633	YDR061W; YDR061W	mitochondrion		catalytic activity;nucleotide binding	0.266	0.266	1.00

	cerevisiae S288c]								
Q02883	N-acyl-phosphatidylethanolamine-hydrolyzing phospholipase D, mitochondrial [OS=Saccharomyces cerevisiae S288c]	856001	FMP30; YPL103C	metabolic process	membrane;mitochondrion	catalytic activity;metal ion binding	0.304	0.304	1.00
P34224	Uncharacterized protein YBL059W [OS=Saccharomyces cerevisiae S288c]	852221	YBL059W; YBL059W	membrane;mitochondrion			0.468	0.468	1.00
P34222	Peptidyl-tRNA hydrolase 2 [OS=Saccharomyces cerevisiae S288c]	852223	PTH2; YBL057C	regulation of biological process	cytoplasm;cytosol;membrane;mitochondrion	catalytic activity;protein binding	0.425	0.425	1.00
P38122	3-methyl-2-oxobutanoate hydroxymethyltransferase [OS=Saccharomyces cerevisiae S288c]	852474	ECM31; YBR176W	metabolic process	mitochondrion	catalytic activity	0.259	0.259	1.00
Q06510	Lysophosphatidylcholine acyltransferase [OS=Saccharomyces cerevisiae S288c]	856262	TAZ1; YPR140W	cell organization and biogenesis;metabolic process;transport	membrane;mitochondrion	catalytic activity	0.194	0.194	1.00
Q06405	ATP synthase subunit f, mitochondrial [OS=Saccharomyces cerevisiae S288c]	851983	ATP17; YDR377W	metabolic process;transport	membrane;mitochondrion	catalytic activity;transporter activity	2.981	2.981	1.00
Q3E6R5	uncharacterized mitochondrial outer membrane protein YDR381C-A [OS=Saccharomyces cerevisiae S288c]	851989	YDR381C-A; YDR381C-A	membrane;mitochondrion			0.389	0.389	1.00
Q05867	Uncharacterized protein YLR283W, mitochondrial [OS=Saccharomyces cerevisiae S288c]	850988	YLR283W; YLR283W	endoplasmic reticulum;membrane;mitochondrion			0.11	0.11	1.00
Q2V2P9	Uncharacterized protein YDR119W-A [OS=Saccharomyces cerevisiae S288c]	3799970	COX26; YDR119W-A	membrane;mitochondrion			1.154	1.154	1.00
P48526	Isoleucine--tRNA ligase, mitochondrial [OS=Saccharomyces cerevisiae S288c]	856067	ISM1; YPL040C	cell organization and biogenesis;metabolic process;regulation of biological process	cytoplasm;cytosol;mitochondrion;organelle lumen	catalytic activity;nucleotide binding	0.04	0.04	1.00
P32378	4-hydroxybenzoate polyprenyltransferase, mitochondrial [OS=Saccharomyces cerevisiae S288c]	855778	COQ2; YNR041C	metabolic process;transport	membrane;mitochondrion	catalytic activity	0.292	0.292	1.00
P38909	cytochrome c mitochondrial import factor CYC2 [OS=Saccharomyces cerevisiae S288c]	854202	CYC2; YOR037W	cell organization and biogenesis;metabolic process	membrane;mitochondrion	catalytic activity	0.194	0.194	1.00
P36064	COX assembly mitochondrial protein [OS=Saccharomyces cerevisiae S288c]	853721	CMC1; YKL137W	cell organization and biogenesis	membrane;mitochondrion	metal ion binding	0.389	0.389	1.00
P32463	Acyl carrier protein, mitochondrial [OS=Saccharomyces cerevisiae S288c]	853642	ACP1; YKL192C	metabolic process;transport	mitochondrion		0.259	0.259	1.00
P04039	Cytochrome c oxidase polypeptide VIII, mitochondrial [OS=Saccharomyces cerevisiae S288c]	851111	COX8; YLR395C	metabolic process;transport	membrane;mitochondrion	catalytic activity;transporter activity	0.778	0.778	1.00
P39103	Cytochrome c oxidase assembly protein COX14 [OS=Saccharomyces cerevisiae S288c]	854910	COX14; YML129C	cell organization and biogenesis;regulation of biological process	membrane;mitochondrion;organelle lumen	protein binding	0.585	0.585	1.00
Q00873	cytochrome c1 heme lyase [OS=Saccharomyces cerevisiae S288c]	853775	CYT2; YKL087C	cell organization and biogenesis;metabolic process	membrane;mitochondrion	catalytic activity;metal ion binding	0.359	0.359	1.00
P53320	Mitochondrial carrier protein MTM1 [OS=Saccharomyces cerevisiae S288c]	853173	MTM1; YGR257C	metabolic process;transport	membrane;mitochondrion	structural molecule activity	0.11	0.11	1.00
P40341	Mitochondrial respiratory chain complexes assembly protein YTA12 [OS=Saccharomyces cerevisiae S288c]	855114	YTA12; YMR089C	cell organization and biogenesis;metabolic process;response to stimulus;transport	cytoplasm;membrane;mitochondrion	catalytic activity;metal ion binding;nucleotide binding;protein binding	1.184	1.276	1.08
P32795	Mitochondrial inner membrane i-AAA	856135	YME1; YPR024W	metabolic process;response to	membrane;mitochondrion	catalytic activity;metal	1.3	1.415	1.09

	protease supercomplex subunit YME1 [OS=Saccharomyces cerevisiae S288c]			stimulus;transport		ion binding;nucleotide binding;protein binding			
P16547	Mitochondrial outer membrane protein OM45 [OS=Saccharomyces cerevisiae S288c]	854670	OM45; YIL136W	membrane;mitochondrion			300.995	330.131	1.10
Q12466	tricalbin-1 [OS=Saccharomyces cerevisiae S288c]	854253	TCB1; YOR086C	cell organization and biogenesis;regulation of biological process	endoplasmic reticulum;membrane;mitochondrion	metal ion binding;protein binding	0.52	0.574	1.10
P17505	Malate dehydrogenase, mitochondrial [OS=Saccharomyces cerevisiae S288c]	853777	MDH1; YKL085W	metabolic process	mitochondrion;organelle lumen	catalytic activity;protein binding;RNA binding	28.936	32.405	1.12
P16622	Ferrochelatase, mitochondrial [OS=Saccharomyces cerevisiae S288c]	854347	HEM15; YOR176W	metabolic process	membrane;mitochondrion	catalytic activity	4.337	4.926	1.14
P49367	Homoaconitase, mitochondrial [OS=Saccharomyces cerevisiae S288c]	851820	LYS4; YDR234W	metabolic process	mitochondrion	catalytic activity;metal ion binding;protein binding	0.638	0.73	1.14
Q08822	Probable electron transfer flavoprotein-ubiquinone oxidoreductase, mitochondrial [OS=Saccharomyces cerevisiae S288c]	854538	CIR2; YOR356W	metabolic process;transport	membrane;mitochondrion	catalytic activity;metal ion binding;nucleotide binding	2.162	2.481	1.15
P25087	Sterol 24-C-methyltransferase [OS=Saccharomyces cerevisiae S288c]	855003	ERG6; YML008C	metabolic process	endoplasmic reticulum;membrane;mitochondrion	catalytic activity	1.424	1.649	1.16
Q12482	Mitochondrial aspartate-glutamate transporter AGC1 [OS=Saccharomyces cerevisiae S288c]	856132	AGC1; YPR021C	metabolic process;transport	membrane;mitochondrion	structural molecule activity;transporter activity	0.348	0.407	1.17
P07213	Mitochondrial import receptor subunit TOM70 [OS=Saccharomyces cerevisiae S288c]	855602	TOM70; YNL121C	cell organization and biogenesis;transport	membrane;mitochondrion	protein binding;transporter activity	2.665	3.125	1.17
Q00402	Nuclear migration protein NUM1 [OS=Saccharomyces cerevisiae S288c]	851727	NUM1; YDR150W	cell organization and biogenesis;cellular component movement;metabolic process;regulation of biological process;transport	endoplasmic reticulum;mitochondrion	catalytic activity;motor activity;protein binding	0.065	0.078	1.20
P33310	ATP-dependent permease MDL1, mitochondrial [OS=Saccharomyces cerevisiae S288c]	850885	MDL1; YLR188W	transport	membrane;mitochondrion	catalytic activity;nucleotide binding;transporter activity	0.438	0.528	1.21
P07143	Cytochrome c1, heme protein, mitochondrial [OS=Saccharomyces cerevisiae S288c]	854231	CYT1; YOR065W	metabolic process;transport	cytosol;membrane;mitochondrion	metal ion binding	13.251	16.013	1.21
P20967	2-oxoglutarate dehydrogenase, mitochondrial [OS=Saccharomyces cerevisiae S288c]	854681	KGD1; YIL125W	metabolic process	cytosol;mitochondrion;organelle lumen	catalytic activity	8.168	9.908	1.21
P00358	glyceraldehyde-3-phosphate dehydrogenase 2 [OS=Saccharomyces cerevisiae S288c]	853465	TDH2; YJR009C	cell death;metabolic process	cytoplasm;cytosol;membrane;mitochondrion;nucleus	catalytic activity;nucleotide binding;protein binding	3.489	4.484	1.29
P04840	Mitochondrial outer membrane protein porin 1 [OS=Saccharomyces cerevisiae S288c]	855669	POR1; YNL055C	cell death;cell organization and biogenesis;cellular homeostasis;regulation of biological process;transport	cytoplasm;membrane;mitochondrion	transporter activity	15.238	19.691	1.29
P53230	Phosphatidate cytidylyltransferase, mitochondrial [OS=Saccharomyces cerevisiae S288c]	852937	TAM41; YGR046W	metabolic process	membrane;mitochondrion;organelle lumen	catalytic activity	0.407	0.532	1.31
Q02776	Mitochondrial import inner membrane translocase subunit TIM50 [OS=Saccharomyces cerevisiae S288c]	856042	TIM50; YPL063W	cell organization and biogenesis;metabolic process;transport	membrane;mitochondrion	catalytic activity;protein binding;transporter activity	7.483	9.857	1.32
P28737	Protein MSP1 [OS=Saccharomyces	852915	MSP1; YGR028W	cell organization and biogenesis;metabolic	membrane;mitochondrion;nucleus	catalytic activity;nucleoti	0.585	0.778	1.33

	cerevisiae S288c]			process;regulation of biological process;response to stimulus;transport		de binding;protein binding;RNA binding			
P33303	Succinate/fumarate mitochondrial transporter [OS=Saccharomyces cerevisiae S288c]	853558	SFC1; YJR095W	metabolic process;transport	membrane;mitochondrion	structural molecule activity;transporter activity	6.743	9	1.33
P39722	Mitochondrial Rho GTPase 1 [OS=Saccharomyces cerevisiae S288c]	851249	GEM1; YAL048C	cell organization and biogenesis;regulation of biological process;response to stimulus;transport	membrane;mitochondrion	catalytic activity;metal ion binding;nucleotide binding	0.189	0.259	1.37
P25039	Elongation factor G, mitochondrial [OS=Saccharomyces cerevisiae S288c]	850758	MEF1; YLR069C	cell organization and biogenesis;metabolic process	cytosol;mitochondrion	catalytic activity;nucleotide binding;RNA binding	0.151	0.207	1.37
Q04013	Citrate/oxoglutarate carrier protein [OS=Saccharomyces cerevisiae S288c]	855282	YHM2; YMR241W	cell organization and biogenesis;metabolic process;transport	membrane;mitochondrion	DNA binding;structural molecule activity;transporter activity	2.162	2.981	1.38
P05626	ATP synthase subunit 4, mitochondrial [OS=Saccharomyces cerevisiae S288c]	856027	ATP4; YPL078C	cell organization and biogenesis;metabolic process;transport	membrane;mitochondrion	catalytic activity;transporter activity	3.084	4.275	1.39
Q04172	Sensitive to high expression protein 9, mitochondrial [OS=Saccharomyces cerevisiae S288c]	852002	SHE9; YDR393W	cell organization and biogenesis	membrane;mitochondrion		0.269	0.374	1.39
P38077	ATP synthase subunit gamma, mitochondrial [OS=Saccharomyces cerevisiae S288c]	852327	ATP3; YBR039W	metabolic process;transport	membrane;mitochondrion	catalytic activity;transporter activity	9	12.689	1.41
P00424	Cytochrome c oxidase polypeptide 5A, mitochondrial [OS=Saccharomyces cerevisiae S288c]	855675	COX5A; YNL052W	metabolic process;transport	membrane;mitochondrion	catalytic activity;transporter activity	1.31	1.848	1.41
P33759	37S ribosomal protein S5, mitochondrial [OS=Saccharomyces cerevisiae S288c]	852553	MRPS5; YBR251W	cell organization and biogenesis;metabolic process	mitochondrion;ribosome	RNA binding;structural molecule activity	0.413	0.585	1.42
Q08179	Mitochondrial distribution and morphology protein 38 [OS=Saccharomyces cerevisiae S288c]	854130	MDM38; YOL027C	cell organization and biogenesis;cellular homeostasis;regulation of biological process;transport	membrane;mitochondrion		3.732	5.31	1.42
P40495	Homoisocitrate dehydrogenase, mitochondrial [OS=Saccharomyces cerevisiae S288c]	854714	LYS12; YIL094C	metabolic process	mitochondrion	catalytic activity;metal ion binding;nucleotide binding	3.217	4.623	1.44
P08466	mitochondrial nuclease [OS=Saccharomyces cerevisiae S288c]	853222	NUC1; YJL208C	cell death;metabolic process	membrane;mitochondrion;nucleus	catalytic activity;metal ion binding	1.336	1.976	1.48
P00950	Phosphoglycerate mutase 1 [OS=Saccharomyces cerevisiae S288c]	853705	GPM1; YKL152C	metabolic process;regulation of biological process	cytoplasm;cytosol;membrane;mitochondrion	catalytic activity	2.981	4.412	1.48
P39518	Long-chain-fatty-acid--CoA ligase 2 [OS=Saccharomyces cerevisiae S288c]	856734	FAA2; YER015W	metabolic process;transport	cytoplasm;mitochondrion	catalytic activity;nucleotide binding	0.535	0.802	1.50
P25349	Flavoprotein-like protein YCP4 [OS=Saccharomyces cerevisiae S288c]	850360	YCP4; YCR004C	metabolic process;regulation of biological process	cytoplasm;membrane;mitochondrion	catalytic activity;nucleotide binding;protein binding	5.813	9	1.55
P16603	NADPH--cytochrome P450 reductase [OS=Saccharomyces cerevisiae S288c]	856438	NCP1; YHR042W	metabolic process	cytoplasm;cytosol;endoplasmic reticulum;membrane;mitochondrion	catalytic activity;metal ion binding;nucleotide binding	0.512	0.805	1.57
P29704	squalene synthase [OS=Saccharomyces cerevisiae S288c]	856597	ERG9; YHR190W	metabolic process	endoplasmic reticulum;membrane;mitochondrion	catalytic activity	0.222	0.35	1.58
Q03028	Mitochondrial 2-oxodicarboxylate carrier 1 [OS=Saccharomyces cerevisiae S288c]	855969	ODC1; YPL134C	metabolic process;transport	membrane;mitochondrion	structural molecule activity;transporter activity	4.275	6.743	1.58
Q99190	Very-long-chain enoyl-CoA reductase [OS=Saccharomyces cerevisiae S288c]	851547	TSC13; YDL015C	metabolic process	cytoplasm;endoplasmic reticulum;membrane;mitochondrion	catalytic activity;protein binding	0.311	0.501	1.61
P14540	fructose-bisphosphate aldolase [OS=Saccharomyces cerevisiae S288c]	853805	FBA1; YKL060C	metabolic process	cytoplasm;cytosol;membrane;mitochondrion	catalytic activity;metal ion binding	0.896	1.448	1.62

P22136	ATPase expression protein 2, mitochondrial [OS=Saccharomyces cerevisiae S288c]	855325	AEP2; YMR282C	metabolic process;regulation of biological process	mitochondrion	RNA binding	0.359	0.585	1.63
P00942	Triosephosphate isomerase [OS=Saccharomyces cerevisiae S288c]	851620	TPI1; YDR050C	metabolic process	cytoplasm;cytosol;membrane;mitochondrion	catalytic activity	0.425	0.701	1.65
Q05050	Eisosome protein 1 [OS=Saccharomyces cerevisiae S288c]	855047	EIS1; YMR031C	cell organization and biogenesis;metabolic process;transport	cytoplasm;extracellular;membrane;mitochondrion		0.661	1.099	1.66
P53163	54S ribosomal protein L12, mitochondrial [OS=Saccharomyces cerevisiae S288c]	852811	MNP1; YGL068W	cell organization and biogenesis;metabolic process	mitochondrion;ribosome	RNA binding;structural molecule activity	0.52	0.874	1.68
P00890	citrate synthase, mitochondrial [OS=Saccharomyces cerevisiae S288c]	855732	CIT1; YNR001C	metabolic process;transport	cytosol;mitochondrion;organelle lumen	catalytic activity	8.211	14.086	1.72
P38988	Mitochondrial GTP/GDP carrier protein 1 [OS=Saccharomyces cerevisiae S288c]	851329	GGC1; YDL198C	cell organization and biogenesis;cellular homeostasis;metabolic process;transport	membrane;mitochondrion	structural molecule activity;transporter activity	3.16	5.449	1.72
P39515	mitochondrial import inner membrane translocase subunit Tim17 [OS=Saccharomyces cerevisiae S288c]	853298	TIM17; YJL143W	cell organization and biogenesis;transport	membrane;mitochondrion	protein binding;transporter activity	0.778	1.371	1.76
Q12289	mitochondrial carnitine carrier [OS=Saccharomyces cerevisiae S288c]	854267	CRC1; YOR100C	metabolic process;transport	membrane;mitochondrion	structural molecule activity;transporter activity	0.931	1.683	1.81
P15992	heat shock protein 26 [OS=Saccharomyces cerevisiae S288c]	852364	HSP26; YBR072W	metabolic process;response to stimulus	cytoplasm;mitochondrion;nucleus	protein binding;RNA binding	4.878	9	1.85
Q03327	Mitochondrial fusion and transport protein Ugo1 [OS=Saccharomyces cerevisiae S288c]	852081	UGO1; YDR470C	cell organization and biogenesis;metabolic process	membrane;mitochondrion	protein binding;structural molecule activity	0.557	1.031	1.85
P18239	ADP,ATP carrier protein 2 [OS=Saccharomyces cerevisiae S288c]	852250	PET9; YBL030C	cell death;metabolic process;transport	membrane;mitochondrion	structural molecule activity;transporter activity	47.064	89.063	1.89
P25613	Accumulation of dyads protein 2 [OS=Saccharomyces cerevisiae S288c]	850368	ADY2; YCR010C	transport	membrane;mitochondrion	transporter activity	9	18.307	2.03
P53252	sphingolipid long chain base-responsive protein PIL1 [OS=Saccharomyces cerevisiae S288c]	852977	PIL1; YGR086C	cell organization and biogenesis;regulation of biological process;response to stimulus;transport	cytoplasm;membrane;mitochondrion	protein binding	10.45	21.539	2.06
P34231	Uncharacterized protein YKL187C [OS=Saccharomyces cerevisiae S288c]	853648	FAT3; YKL187C; YKL187C	cell organization and biogenesis;transport	membrane;mitochondrion		0.093	0.194	2.09
P09368	Proline dehydrogenase, mitochondrial [OS=Saccharomyces cerevisiae S288c]	850833	PUT1; YLR142W	metabolic process	mitochondrion;organelle lumen	catalytic activity	0.093	0.194	2.09
P43603	LAS seventeen-binding protein 3 [OS=Saccharomyces cerevisiae S288c]	850580	LSB3; YFR024C-A	cell organization and biogenesis;regulation of biological process	cytoplasm;mitochondrion	protein binding	0.089	0.186	2.09
P32939	GTP-binding protein ypt7 [OS=Saccharomyces cerevisiae S288c]	855012	YPT7; YML001W	cell communication;cell organization and biogenesis;metabolic process;regulation of biological process;response to stimulus;transport	cytosol;endosome;membrane;mitochondrion;vacuole	catalytic activity;nucleotide binding;protein binding	0.166	0.359	2.16
P54857	lipase 2 [OS=Saccharomyces cerevisiae S288c]	851628	TGL2; YDR058C	metabolic process;transport	mitochondrion	catalytic activity	0.233	0.52	2.23
P49334	Mitochondrial import receptor subunit tom22 [OS=Saccharomyces cerevisiae S288c]	855592	TOM22; YNL131W	cell organization and biogenesis;transport	membrane;mitochondrion	protein binding;transporter activity	0.292	0.668	2.29
P19882	heat shock protein 60, mitochondrial [OS=Saccharomyces cerevisiae S288c]	850963	HSP60; YLR259C	cell organization and biogenesis;metabolic process;regulation of biological process;transport	cytoplasm;cytosol;membrane;mitochondrion;organelle lumen	catalytic activity;DNA binding;nucleotide binding;protein binding	13.384	32.598	2.44
Q08245	protein ZEO1 [OS=Saccharomyces cerevisiae S288c]	854040	ZEO1; YOL109W	cell organization and biogenesis	membrane;mitochondrion	protein binding	2.162	5.31	2.46
Q12230	Sphingolipid long chain base-responsive protein LSP1 [OS=Saccharomyces	856103	LSP1; YPL004C	regulation of biological process;response to stimulus;transport	cytoplasm;membrane;mitochondrion		24.119	62.096	2.57

	cerevisiae S288c]								
P12709	glucose-6-phosphate isomerase [OS=Saccharomyces cerevisiae S288c]	852495	PGI1; YBR196C	metabolic process	cytoplasm;membrane;mitochondrion	catalytic activity	0.166	0.468	2.82
P39952	Mitochondrial inner membrane protein OXA1 [OS=Saccharomyces cerevisiae S288c]	856898	OXA1; YER154W	cell organization and biogenesis;transport	membrane;mitochondrion	transporter activity	1.054	3.217	3.05
P50110	Sorting assembly machinery 37 kDa subunit [OS=Saccharomyces cerevisiae S288c]	855082	SAM37; YMR060C	cell organization and biogenesis;transport	membrane;mitochondrion	protein binding	0.116	0.389	3.35
P04803	Tryptophan--tRNA ligase, mitochondrial [OS=Saccharomyces cerevisiae S288c]	851861	MSW1; YDR268W	metabolic process	cytoplasm;mitochondrion;organelle lumen	catalytic activity;nucleotide binding	0.136	0.468	3.44
Q00955	Acetyl-CoA carboxylase [OS=Saccharomyces cerevisiae S288c]	855750	ACC1; YNR016C	cell organization and biogenesis;metabolic process;transport	cytoplasm;cytosol;endoplasmic reticulum;membrane;mitochondrion	catalytic activity;metal ion binding;nucleotide binding	0.032	0.271	8.47

Supplemental Table S13. The relative concentrations of proteins in mitochondria purified from *ups2Δ* cells cultured with or without LCA. Mitochondria were purified from *ups2Δ* cells recovered on day 2 of cell culturing with or without LCA. Mass spectrometry-based identification and quantitation of proteins recovered in purified mitochondria were performed as described in Materials and methods. The exponentially modified protein abundance index (emPAI) provided by the ″Proteome Discoverer″ software was used to calculate the relative abundance of mitochondrial proteins between specified datasets. Data on the relative concentrations of mitochondrial proteins are presented as means of 2 independent experiments.

Accession	Description	Entrez Gene ID	Gene ID	Biological Process	Cellular Component	Molecular Function	emPAI *ups2*	emPAI *ups2* + LCA	Ratio *ups2* + LCA / *ups2*
P40513	Mitochondrial acidic protein MAM33 [OS=Saccharomyces cerevisiae S288c]	854740	MAM33; YIL070C	metabolic process;regulation of biological process	mitochondrion;organelle lumen	translation regulator activity	0.778	0.212	0.27
P17558	37S ribosomal protein PET123, mitochondrial [OS=Saccharomyces cerevisiae S288c]	854329	PET123; YOR158W	cell organization and biogenesis;metabolic process	mitochondrion;ribosome	structural molecule activity	0.468	0.136	0.29
P46681	D-lactate dehydrogenase [cytochrome] 2, mitochondrial [OS=Saccharomyces cerevisiae S288c]	851376	DLD2; YDL178W	metabolic process	mitochondrion;organelle lumen	catalytic activity;nucleotide binding;protein binding	0.682	0.25	0.37
P32387	54S ribosomal protein L41, mitochondrial [OS=Saccharomyces cerevisiae S288c]	852014	MRP20; YDR405W	cell organization and biogenesis;metabolic process	mitochondrion;ribosome	nucleotide binding;protein binding;RNA binding;structural molecule activity	0.719	0.311	0.43
P25372	Thioredoxin-3, mitochondrial [OS=Saccharomyces cerevisiae S288c]	850444	TRX3; YCR083W	cellular homeostasis;metabolic process;regulation of biological process;response to stimulus;transport	cytoplasm;mitochondrion	catalytic activity	0.52	0.233	0.45
P00445	Superoxide dismutase [Cu-Zn] [OS=Saccharomyces cerevisiae S288c]	853568	SOD1; YJR104C	cell organization and biogenesis;cellular homeostasis;metabolic process;regulation of biological process;response to stimulus	cytoplasm;cytosol;mitochondrion;nucleus	antioxidant activity;catalytic activity;metal ion binding;protein binding	0.52	0.233	0.45
P14693	sorting assembly machinery 35 kDa subunit [OS=Saccharomyces cerevisiae S288c]	856483	SAM35; YHR083W	cell organization and biogenesis;transport	membrane;mitochondrion	protein binding	0.334	0.155	0.46
Q12298	uncharacterized ABC transporter ATP-binding protein YDR061W [OS=Saccharomyces cerevisiae S288c]	851633	YDR061W; YDR061W		mitochondrion	catalytic activity;nucleotide binding	0.266	0.125	0.47
P21771	37S ribosomal protein S28, mitochondrial [OS=Saccharomyces cerevisiae S288c]	851937	MRPS28; YDR337W	cell organization and biogenesis;metabolic process	mitochondrion;ribosome	RNA binding;structural molecule activity	0.274	0.129	0.47
P38797	Protein phosphatase 2C homolog 7, mitochondrial [OS=Saccharomyces cerevisiae S288c]	856475	PTC7; YHR076W	metabolic process	mitochondrion	catalytic activity;metal ion binding;protein binding	0.233	0.11	0.47
P36517	54S ribosomal protein L4, mitochondrial [OS=Saccharomyces cerevisiae S288c]	851160	MRPL4; YLR439W	cell organization and biogenesis;metabolic process	mitochondrion;ribosome	structural molecule activity	0.233	0.11	0.47
P51998	54S ribosomal protein yml6, mitochondrial [OS=Saccharomyces cerevisiae S288c]	854983	YML6; YML025C	cell organization and biogenesis;metabolic process	mitochondrion;ribosome	RNA binding;structural molecule activity	0.245	0.116	0.47
P53140	Protein RMD9, mitochondrial [OS=Saccharomyces cerevisiae S288c]	852771	RMD9; YGL107C	cell differentiation;metabolic process;regulation of biological process	membrane;mitochondrion	RNA binding	0.227	0.108	0.48
P07236	Threonine--tRNA ligase, mitochondrial [OS=Saccharomyces cerevisiae S288c]	853640	MST1; YKL194C	metabolic process	cytoplasm;mitochondrion;organelle lumen	catalytic activity;nucleotide binding;RNA binding	0.15	0.072	0.48
P32332	Mitochondrial oxaloacetate transport protein [OS=Saccharomyces cerevisiae S288c]	853739	OAC1; YKL120W	metabolic process;transport	membrane;mitochondrion	structural molecule activity;transporter activity	3.642	1.783	0.49
P07275	Delta-1-pyrroline-5-carboxylate dehydrogenase, mitochondrial [OS=Saccharomyces cerevisiae S288c]	856432	PUT2; YHR037W	metabolic process	membrane;mitochondrion;organelle lumen	catalytic activity	1.848	1.009	0.55
P00950	Phosphoglycerate mutase 1 [OS=Saccharomyces cerevisiae S288c]	853705	GPM1; YKL152C	metabolic process;regulation of biological process	cytoplasm;cytosol;membrane;mitochondrion	catalytic activity	1.512	0.848	0.56
P32860	NifU-like protein, mitochondrial [OS=Saccharomyces cerevisiae S288c]	853826	NFU1; YKL040C	cell organization and biogenesis;cellular homeostasis;metabolic process	mitochondrion;organelle lumen	metal ion binding	1.371	0.778	0.57
P43635	Citrate synthase 3, mitochondrial [OS=Saccharomyces cerevisiae S288c]	856107	CIT3; YPR001W	metabolic process	mitochondrion	catalytic activity	1.346	0.817	0.61
P40086	Cytochrome c oxidase assembly protein cox15 [OS=Saccharomyces	856884	COX15; YER141	cell organization and biogenesis;metabolic	membrane;mitochondrion	catalytic activity;transporter	1.346	0.817	0.61

	cerevisiae S288c]		W	process;transport		activity			
P36525	54S ribosomal protein L24, mitochondrial [OS=Saccharomyces cerevisiae S288c]	855231	MRPL24; YMR193W	cell organization and biogenesis;metabolic process	mitochondrion;ribosome	RNA binding;structural molecule activity	0.585	0.359	0.61
P39525	3-oxoacyl-[acyl-carrier-protein] synthase homolog [OS=Saccharomyces cerevisiae S288c]	856790	CEM1; YER061C	metabolic process	mitochondrion	catalytic activity	0.369	0.233	0.63
P32898	Mitochondrial presequence protease [OS=Saccharomyces cerevisiae S288c]	852041	CYM1; YDR430C	metabolic process	mitochondrion	catalytic activity;metal ion binding	0.25	0.16	0.64
P07806	Valine--tRNA ligase, mitochondrial [OS=Saccharomyces cerevisiae S288c]	852986	VAS1; YGR094W	metabolic process;regulation of biological process	cytoplasm;cytosol;mitochondrion	catalytic activity;nucleotide binding	0.212	0.136	0.64
Q05931	Heat shock protein SSQ1, mitochondrial [OS=Saccharomyces cerevisiae S288c]	851084	SSQ1; YLR369W	cell organization and biogenesis;metabolic process	mitochondrion;organelle lumen	nucleotide binding;protein binding	0.189	0.122	0.65
P38071	Enoyl-[acyl-carrier protein] reductase [NADPH, B-specific], mitochondrial [OS=Saccharomyces cerevisiae S288c]	852314	ETR1; YBR026C	metabolic process	mitochondrion;organelle lumen	catalytic activity;DNA binding;metal ion binding	3.329	2.162	0.65
P15424	ATP-dependent RNA helicase MSS116, mitochondrial [OS=Saccharomyces cerevisiae S288c]	851775	MSS116; YDR194C	metabolic process;regulation of biological process	mitochondrion;organelle lumen	catalytic activity;nucleotide binding;RNA binding	0.166	0.108	0.65
P25374	Cysteine desulfurase, mitochondrial [OS=Saccharomyces cerevisiae S288c]	850343	NFS1; YCL017C	cell organization and biogenesis;cellular homeostasis;metabolic process	mitochondrion;nucleus	catalytic activity;metal ion binding;protein binding	0.817	0.532	0.65
P38825	Protein TOM71 [OS=Saccharomyces cerevisiae S288c]	856517	TOM71; YHR117W	transport	membrane;mitochondrion	protein binding;transporter activity	0.512	0.343	0.67
P33310	ATP-dependent permease MDL1, mitochondrial [OS=Saccharomyces cerevisiae S288c]	850885	MDL1; YLR188W	transport	membrane;mitochondrion	catalytic activity;nucleotide binding;transporter activity	0.528	0.354	0.67
P38325	Mitochondrial outer membrane protein OM14 [OS=Saccharomyces cerevisiae S288c]	852531	OM14; YBR230C	transport	membrane;mitochondrion		6.197	4.179	0.67
P32904	54S ribosomal protein L6, mitochondrial [OS=Saccharomyces cerevisiae S288c]	856552	MRPL6; YHR147C	cell organization and biogenesis;metabolic process	mitochondrion;ribosome	RNA binding;structural molecule activity	0.778	0.54	0.69
P33416	Heat shock protein 78, mitochondrial [OS=Saccharomyces cerevisiae S288c]	851845	HSP78; YDR258C	cell organization and biogenesis;metabolic process;regulation of biological process;response to stimulus	mitochondrion;organelle lumen	catalytic activity;nucleotide binding;protein binding	1.383	0.973	0.70
P16603	NADPH--cytochrome P450 reductase [OS=Saccharomyces cerevisiae S288c]	856438	NCP1; YHR042W	metabolic process	cytoplasm;cytosol;endoplasmic reticulum;membrane;mitochondrion	catalytic activity;metal ion binding;nucleotide binding	0.604	0.425	0.70
P49367	Homoaconitase, mitochondrial [OS=Saccharomyces cerevisiae S288c]	851820	LYS4; YDR234W	metabolic process	mitochondrion	catalytic activity;metal ion binding;protein binding	0.551	0.389	0.71
P43567	alanine--glyoxylate aminotransferase 1 [OS=Saccharomyces cerevisiae S288c]	850514	AGX1; YFL030W	metabolic process	cytosol;mitochondrion	catalytic activity	0.468	0.334	0.71
P10507	mitochondrial-processing peptidase subunit beta [OS=Saccharomyces cerevisiae S288c]	850860	MAS1; YLR163C	metabolic process;transport	mitochondrion;organelle lumen	catalytic activity;metal ion binding;protein binding	0.468	0.334	0.71
Q08970	Mitochondrial metal transporter 2 [OS=Saccharomyces cerevisiae S288c]	855877	MMT2; YPL224C	cellular homeostasis;regulation of biological process;response to stimulus;transport	membrane;mitochondrion	transporter activity	0.407	0.292	0.72
P00447	Superoxide dismutase [Mn], mitochondrial [OS=Saccharomyces cerevisiae S288c]	856399	SOD2; YHR008C	metabolic process;response to stimulus	mitochondrion;organelle lumen	antioxidant activity;catalytic activity;metal ion binding	1.424	1.031	0.72
P09440	C-1-tetrahydrofolate synthase, mitochondrial [OS=Saccharomyces cerevisiae S288c]	852378	MIS1; YBR084W	metabolic process	cytosol;mitochondrion	catalytic activity;nucleotide binding	0.633	0.459	0.73
P32799	Cytochrome c oxidase subunit 6A, mitochondrial [OS=Saccharomyces cerevisiae S288c]	852684	COX13; YGL191W	cell organization and biogenesis;metabolic process;regulation of biological process;transport	membrane;mitochondrion	catalytic activity;enzyme regulator activity;transporter activity	2.981	2.162	0.73
Q04458	Fatty aldehyde dehydrogenase HFD1 [OS=Saccharomyces cerevisiae S288c]	855137	HFD1; YMR110C	metabolic process	endoplasmic reticulum;endosome;membrane;mitochondrion	catalytic activity	1.346	0.978	0.73
P53312	Succinyl-CoA ligase [ADP-forming] subunit beta, mitochondrial [OS=Saccharomyces cerevisiae S288c]	853159	LSC2; YGR244C	metabolic process	mitochondrion	catalytic activity;nucleotide binding	3.417	2.535	0.74
P40185	Protein mmf1, mitochondrial [OS=Saccharomyces cerevisiae S288c]	854760	MMF1; YIL051C	cell organization and biogenesis;metabolic process	mitochondrion;organelle lumen		4.012	2.981	0.74
P00410	Cytochrome c oxidase subunit 2 [OS=Saccharomyces cerevisiae S288c]	854622	COX2; Q0250	metabolic process;transport	membrane;mitochondrion	catalytic activity;metal ion binding;protein	2.162	1.61	0.74

						binding;transporter activity			
P40053	Altered inheritance of mitochondria protein 9, mitochondrial [OS=Saccharomyces cerevisiae S288c]	856813	AIM9; YER080W		mitochondrion		1.19	0.891	0.75
Q03798	Altered inheritance of mitochondria protein 36, mitochondrial [OS=Saccharomyces cerevisiae S288c]	855189	AIM36; YMR157C		membrane;mitochondrion		0.833	0.624	0.75
P10662	37S ribosomal protein MRP1, mitochondrial [OS=Saccharomyces cerevisiae S288c]	851948	MRP1; YDR347W	cell organization and biogenesis;metabolic process;response to stimulus	mitochondrion;ribosome	antioxidant activity;catalytic activity;metal ion binding;structural molecule activity	0.778	0.585	0.75
Q06143	mitochondrial dicarboxylate transporter [OS=Saccharomyces cerevisiae S288c]	851063	DIC1; YLR348C	metabolic process;transport	membrane;mitochondrion	structural molecule activity;transporter activity	0.73	0.551	0.75
P00890	citrate synthase, mitochondrial [OS=Saccharomyces cerevisiae S288c]	855732	CIT1; YNR001C	metabolic process;transport	cytosol;mitochondrion;organelle lumen	catalytic activity	10.788	8.211	0.76
P19882	heat shock protein 60, mitochondrial [OS=Saccharomyces cerevisiae S288c]	850963	HSP60; YLR259C	cell organization and biogenesis;metabolic process;regulation of biological process;transport	cytoplasm;cytosol;membrane;mitochondrion;organelle lumen	catalytic activity;DNA binding;nucleotide binding;protein binding	11.743	9	0.77
P27697	Atypical kinase COQ8, mitochondrial [OS=Saccharomyces cerevisiae S288c]	852758	COQ8; YGL119W	metabolic process	membrane;mitochondrion;organelle lumen	catalytic activity;nucleotide binding	0.365	0.283	0.78
Q01163	37S ribosomal protein S23, mitochondrial [OS=Saccharomyces cerevisiae S288c]	852748	RSM23; YGL129C	cell organization and biogenesis;metabolic process	mitochondrion;ribosome	catalytic activity;nucleotide binding;RNA binding;structural molecule activity	0.365	0.283	0.78
P07253	cytochrome B pre-mRNA-processing protein 6 [OS=Saccharomyces cerevisiae S288c]	852417	CBP6; YBR120C	cell organization and biogenesis;metabolic process;regulation of biological process	mitochondrion;ribosome		2.162	1.683	0.78
Q12480	Probable electron transfer flavoprotein subunit alpha, mitochondrial [OS=Saccharomyces cerevisiae S288c]	856112	AIM45; YPR004C	metabolic process;transport	mitochondrion;organelle lumen	catalytic activity;nucleotide binding	0.995	0.778	0.78
P53292	37S ribosomal protein S35, mitochondrial [OS=Saccharomyces cerevisiae S288c]	853075	MRPS35; YGR165W	cell organization and biogenesis;metabolic process	mitochondrion;ribosome	structural molecule activity	0.738	0.585	0.79
P0CS90	Heat shock protein SSC1, mitochondrial [OS=Saccharomyces cerevisiae S288c]	853503	SSC1; YJR045C	cell organization and biogenesis;metabolic process;regulation of biological process;transport	membrane;mitochondrion;nucleus;organelle lumen	catalytic activity;enzyme regulator activity;nucleotide binding;protein binding	17.957	14.647	0.82
P40035	Mitochondrial phosphate carrier protein 2 [OS=Saccharomyces cerevisiae S288c]	856779	PIC2; YER053C	cellular homeostasis;metabolic process;transport	membrane;mitochondrion	structural molecule activity;transporter activity	1.637	1.336	0.82
P22136	ATPase expression protein 2, mitochondrial [OS=Saccharomyces cerevisiae S288c]	855325	AEP2; YMR282C	metabolic process;regulation of biological process	mitochondrion	RNA binding	0.431	0.359	0.83
P32473	Pyruvate dehydrogenase E1 component subunit beta, mitochondrial [OS=Saccharomyces cerevisiae S288c]	852522	PDB1; YBR221C	metabolic process	mitochondrion;organelle lumen	catalytic activity	3.833	3.281	0.86
P33311	ATP-dependent permease MDL2, mitochondrial [OS=Saccharomyces cerevisiae S288c]	855858	MDL2; YPL270W	response to stimulus;transport	membrane;mitochondrion	catalytic activity;nucleotide binding;transporter activity	0.708	0.619	0.87
P48015	Aminomethyltransferase, mitochondrial [OS=Saccharomyces cerevisiae S288c]	851582	GCV1; YDR019C	metabolic process	mitochondrion	catalytic activity;protein binding	3.489	3.062	0.88
Q12031	Mitochondrial 2-methylisocitrate lyase [OS=Saccharomyces cerevisiae S288c]	856114	ICL2; YPR006C	metabolic process	mitochondrion;organelle lumen	catalytic activity	0.947	0.833	0.88
P36775	Lon protease homolog, mitochondrial [OS=Saccharomyces cerevisiae S288c]	852259	PIM1; YBL022C	cell organization and biogenesis;metabolic process;regulation of biological process;response to stimulus	cytoplasm;mitochondrion;organelle lumen	catalytic activity;DNA binding;nucleotide binding;RNA binding	0.359	0.318	0.89
P35191	DnaJ homolog 1, mitochondrial [OS=Saccharomyces cerevisiae S288c]	850530	MDJ1; YFL016C	cell organization and biogenesis;metabolic process;regulation of biological process;response to stimulus	mitochondrion;organelle lumen	enzyme regulator activity;metal ion binding;nucleotide binding;protein binding	1.477	1.31	0.89
P54783	D-arabinono-1,4-lactone oxidase [OS=Saccharomyces cerevisiae S288c]	854888	ALO1; YML086C	metabolic process;response to stimulus	membrane;mitochondrion	catalytic activity;nucleotide binding	2.162	1.929	0.89
P17505	Malate dehydrogenase, mitochondrial [OS=Saccharomyces cerevisiae S288c]	853777	MDH1; YKL085W	metabolic process	mitochondrion;organelle lumen	catalytic activity;protein binding;RNA binding	32.405	28.936	0.89
P37292	Serine hydroxymethyltransferase, mitochondrial [OS=Saccharomyces cerevisiae S288c]	852565	SHM1; YBR263W	metabolic process	mitochondrion	catalytic activity	3.037	2.765	0.91

P16387	Pyruvate dehydrogenase E1 component subunit alpha, mitochondrial [OS=Saccharomyces cerevisiae S288c]	856925	PDA1; YER178W	cell growth;metabolic process	mitochondrion;organelle lumen	catalytic activity	5.898	5.404	0.92
Q12428	Probable 2-methylcitrate dehydratase [OS=Saccharomyces cerevisiae S288c]	856108	PDH1; YPR002W	metabolic process	cytoplasm;membrane;mitochondrion	catalytic activity	5.978	5.494	0.92
P07342	Acetolactate synthase catalytic subunit, mitochondrial [OS=Saccharomyces cerevisiae S288c]	855135	ILV2; YMR108W	metabolic process	mitochondrion	catalytic activity;metal ion binding;nucleotide binding	2.665	2.455	0.92
P07251	ATP synthase subunit alpha, mitochondrial [OS=Saccharomyces cerevisiae S288c]	852177	ATP1; YBL099W	metabolic process;transport	cytosol;membrane;mitochondrion	catalytic activity;nucleotide binding;transporter activity	13.678	13.678	1.00
Q08179	Mitochondrial distribution and morphology protein 38 [OS=Saccharomyces cerevisiae S288c]	854130	MDM38; YOL027C	cell organization and biogenesis;cellular homeostasis;regulation of biological process;transport	membrane;mitochondrion		3.467	3.467	1.00
P02992	elongation factor Tu, mitochondrial [OS=Saccharomyces cerevisiae S288c]	854359	TUF1; YOR187W	cell organization and biogenesis;metabolic process	mitochondrion	catalytic activity;nucleotide binding;RNA binding	3.924	3.924	1.00
P25349	Flavoprotein-like protein YCP4 [OS=Saccharomyces cerevisiae S288c]	850360	YCP4; YCR004C	metabolic process;regulation of biological process	cytoplasm;membrane;mitochondrion	catalytic activity;nucleotide binding;protein binding	5.813	5.813	1.00
P00359	Glyceraldehyde-3-phosphate dehydrogenase 3 [OS=Saccharomyces cerevisiae S288c]	853106	TDH3; YGR192C	cell death;metabolic process	cytoplasm;cytosol;membrane;mitochondrion;nucleus	catalytic activity;nucleotide binding;RNA binding	2.675	2.675	1.00
Q03028	Mitochondrial 2-oxodicarboxylate carrier 1 [OS=Saccharomyces cerevisiae S288c]	855969	ODC1; YPL134C	metabolic process;transport	membrane;mitochondrion	structural molecule activity;transporter activity	4.275	4.275	1.00
P32795	Mitochondrial inner membrane i-AAA protease supercomplex subunit YME1 [OS=Saccharomyces cerevisiae S288c]	856135	YME1; YPR024W	metabolic process;response to stimulus;transport	membrane;mitochondrion	catalytic activity;metal ion binding;nucleotide binding;protein binding	0.8	0.8	1.00
P28834	Isocitrate dehydrogenase [NAD] subunit 1, mitochondrial [OS=Saccharomyces cerevisiae S288c]	855691	IDH1; YNL037C	metabolic process;transport	cytosol;mitochondrion;organelle lumen	catalytic activity;metal ion binding;nucleotide binding;protein binding;RNA binding	2.36	2.36	1.00
P04037	cytochrome c oxidase subunit 4, mitochondrial [OS=Saccharomyces cerevisiae S288c]	852688	COX4; YGL187C	cell organization and biogenesis;metabolic process;transport	membrane;mitochondrion	catalytic activity;metal ion binding;transporter activity	5.579	5.579	1.00
Q08968	UPF0061 protein FMP40 [OS=Saccharomyces cerevisiae S288c]	855879	FMP40; YPL222W		mitochondrion	protein binding	0.65	0.65	1.00
P53721	Respiratory supercomplex factor 2, mitochondrial [OS=Saccharomyces cerevisiae S288c]	855752	RCF2; YNR018W	cell organization and biogenesis	membrane;mitochondrion		5.579	5.579	1.00
P08466	mitochondrial nuclease [OS=Saccharomyces cerevisiae S288c]	853222	NUC1; YJL208C	cell death;metabolic process	membrane;mitochondrion	catalytic activity;metal ion binding	1.336	1.336	1.00
P40508	Uncharacterized protein YIL077C [OS=Saccharomyces cerevisiae S288c]	854733	YIL077C; YIL077C		mitochondrion		1.154	1.154	1.00
P00360	glyceraldehyde-3-phosphate dehydrogenase 1 [OS=Saccharomyces cerevisiae S288c]	853395	TDH1; YJL052W	metabolic process	cytoplasm;cytosol;membrane;mitochondrion	catalytic activity;nucleotide binding	0.823	0.823	1.00
P16451	Pyruvate dehydrogenase complex protein X component, mitochondrial [OS=Saccharomyces cerevisiae S288c]	853107	PDX1; YGR193C	metabolic process	mitochondrion;organelle lumen	catalytic activity;structural molecule activity	0.978	0.978	1.00
P39726	Glycine cleavage system H protein, mitochondrial [OS=Saccharomyces cerevisiae S288c]	851254	GCV3; YAL044C	metabolic process	mitochondrion	catalytic activity	1.154	1.154	1.00
Q06668	Methyltransferase OMS1, mitochondrial [OS=Saccharomyces cerevisiae S288c]	851911	OMS1; YDR316W	metabolic process	membrane;mitochondrion	catalytic activity	0.484	0.484	1.00
P25573	Mitochondrial inner membrane i-AAA protease supercomplex subunit MGR1 [OS=Saccharomyces cerevisiae S288c]	850313	MGR1; YCL044C	metabolic process	membrane;mitochondrion	protein binding	0.701	0.701	1.00
P40416	Iron-sulfur clusters transporter ATM1, mitochondrial [OS=Saccharomyces cerevisiae S288c]	855347	ATM1; YMR301C	cellular homeostasis;metabolic process;transport	membrane;mitochondrion	catalytic activity;nucleotide binding;transporter activity	0.546	0.546	1.00
P42940	Probable electron transfer flavoprotein subunit beta [OS=Saccharomyces cerevisiae S288c]	853121	CIR1; YGR207C	metabolic process;transport	mitochondrion;organelle lumen		2.162	2.162	1.00
P25605	Acetolactate synthase small subunit, mitochondrial [OS=Saccharomyces cerevisiae S288c]	850348	ILV6; YCL009C	metabolic process;regulation of biological process	mitochondrion	catalytic activity;enzyme regulator activity	0.624	0.624	1.00
P38127	mitochondrial carrier protein RIM2 [OS=Saccharomyces cerevisiae S288c]	852491	RIM2; YBR192W	cell organization and biogenesis;metabolic process;transport	membrane;mitochondrion	structural molecule activity;transporter activity	0.995	0.995	1.00

P38626	NADH-cytochrome b5 reductase 1 [OS=Saccharomyces cerevisiae S288c]	854768	CBR1; YIL043C	metabolic process	endoplasmic reticulum;membrane;mitochondrion;nucleus	catalytic activity	0.778	0.778	1.00
P38072	Protein SCO2, mitochondrial [OS=Saccharomyces cerevisiae S288c]	852312	SCO2; YBR024W	cell organization and biogenesis;cellular homeostasis;metabolic process;transport	membrane;mitochondrion	antioxidant activity;catalytic activity;metal ion binding	0.73	0.73	1.00
P40008	Protein FMP52, mitochondrial [OS=Saccharomyces cerevisiae S288c]	856721	FMP52; YER004W		endoplasmic reticulum;membrane;mitochondrion	catalytic activity	1.054	1.054	1.00
P32331	Carrier protein YMC1, mitochondrial [OS=Saccharomyces cerevisiae S288c]	856171	YMC1; YPR058W	metabolic process;transport	membrane;mitochondrion;vacuole	structural molecule activity;transporter activity	0.968	0.968	1.00
Q04472	Mitochondrial inner membrane i-AAA protease supercomplex subunit MGR3 [OS=Saccharomyces cerevisiae S288c]	855142	MGR3; YMR115W	metabolic process	membrane;mitochondrion	protein binding	0.322	0.322	1.00
P38079	protein YRO2 [OS=Saccharomyces cerevisiae S288c]	852343	YRO2; YBR054W	transport	endoplasmic reticulum;membrane;mitochondrion	transporter activity	1.424	1.424	1.00
P32787	Mitochondrial genome maintenance protein MGM101 [OS=Saccharomyces cerevisiae S288c]	853609	MGM101; YJR144W	cell organization and biogenesis;metabolic process;response to stimulus	chromosome;mitochondrion	DNA binding	0.833	0.833	1.00
Q04935	Cytochrome c oxidase protein 20, mitochondrial [OS=Saccharomyces cerevisiae S288c]	851817	COX20; YDR231C	cell organization and biogenesis;metabolic process	membrane;mitochondrion	protein binding	1.424	1.424	1.00
P50087	MICOS subunit MIC26 [OS=Saccharomyces cerevisiae S288c]	853150	MOS2; MIC26; YGR235C	cell organization and biogenesis	membrane;mitochondrion		0.931	0.931	1.00
P54115	Magnesium-activated aldehyde dehydrogenase, cytosolic [OS=Saccharomyces cerevisiae S288c]	856044	ALD6; YPL061W	metabolic process;response to stimulus	cytoplasm;cytosol;mitochondrion	catalytic activity	0.346	0.346	1.00
P28737	Protein MSP1 [OS=Saccharomyces cerevisiae S288c]	852915	MSP1; YGR028W	cell organization and biogenesis;metabolic process;regulation of biological process;response to stimulus;transport	membrane;mitochondrion;nucleus	catalytic activity;nucleotide binding;protein binding;RNA binding	0.585	0.585	1.00
Q05892	MIOREX complex component 2 [OS=Saccharomyces cerevisiae S288c]	850997	YLR290C; COQ11; YLR290C	metabolic process	mitochondrion	catalytic activity	1.054	1.054	1.00
Q3E824	Uncharacterized protein YOR020W-A [OS=Saccharomyces cerevisiae S288c]	1466480	YOR020W-A; YOR020W-A	metabolic process;transport	membrane;mitochondrion		2.162	2.162	1.00
P36151	Uncharacterized protein YKR070W [OS=Saccharomyces cerevisiae S288c]	853944	YKR070W; YKR070W	metabolic process	mitochondrion		0.274	0.274	1.00
Q04438	Stationary phase protein 4 [OS=Saccharomyces cerevisiae S288c]	855134	SPG4; YMR107W				1.783	1.783	1.00
Q03246	37S ribosomal protein S17, mitochondrial [OS=Saccharomyces cerevisiae S288c]	855226	MRPS17; YMR188C	cell differentiation;cell organization and biogenesis;metabolic process	mitochondrion;ribosome	RNA binding;structural molecule activity	0.389	0.389	1.00
P38910	10 kDa heat shock protein, mitochondrial [OS=Saccharomyces cerevisiae S288c]	854185	HSP10; YOR020C	cell organization and biogenesis;metabolic process;response to stimulus;transport	cytoplasm;mitochondrion;organelle lumen	metal ion binding;nucleotide binding;protein binding	1.371	1.371	1.00
P32792	UPF0744 protein YSC83 [OS=Saccharomyces cerevisiae S288c]	856410	YSC83; YHR017W		membrane;mitochondrion		0.369	0.369	1.00
P25719	Peptidyl-prolyl cis-trans isomerase C, mitochondrial [OS=Saccharomyces cerevisiae S288c]	854897	CPR3; YML078W	cell death;metabolic process	mitochondrion;organelle lumen	catalytic activity	0.585	0.585	1.00
P36163	mitochondrial metalloendopeptidase OMA1 [OS=Saccharomyces cerevisiae S288c]	853962	OMA1; YKR087C	metabolic process	membrane;mitochondrion	catalytic activity;metal ion binding	0.389	0.389	1.00
P53230	Phosphatidate cytidylyltransferase, mitochondrial [OS=Saccharomyces cerevisiae S288c]	852937	TAM41; YGR046W	metabolic process	membrane;mitochondrion;organelle lumen	catalytic activity	0.407	0.407	1.00
P34231	Uncharacterized protein YKL187C [OS=Saccharomyces cerevisiae S288c]	853648	FAT3; YKL187C; YKL187C	cell organization and biogenesis;transport	membrane;mitochondrion		0.425	0.425	1.00
Q96VH5	MICOS complex subunit Mic10 [OS=Saccharomyces cerevisiae S288c]	850300	MOS1; MIC10; YCL057C-A	cell organization and biogenesis	membrane;mitochondrion		0.931	0.931	1.00
Q12374	Nuclear control of ATPase protein 2 [OS=Saccharomyces cerevisiae S288c]	856278	NCA2; YPR155C	metabolic process	membrane;mitochondrion		0.212	0.212	1.00
P17695	Glutaredoxin-2, mitochondrial [OS=Saccharomyces cerevisiae S288c]	852124	GRX2; YDR513W	cellular homeostasis;metabolic process;regulation of biological process;response to	cytoplasm;cytosol;mitochondrion	antioxidant activity;catalytic activity	0.52	0.52	1.00

				stimulus;transport					
Q12375	mitochondrial ornithine transporter 1 [OS=Saccharomyces cerevisiae S288c]	854297	ORT1; YOR130C	metabolic process;transport	membrane;mitochondrion	structural molecule activity;transporter activity	0.54	0.54	1.00
P28817	3-hydroxyisobutyryl-CoA hydrolase, mitochondrial [OS=Saccharomyces cerevisiae S288c]	851606	EHD3; YDR036C	metabolic process	mitochondrion	catalytic activity	0.269	0.269	1.00
P50110	Sorting assembly machinery 37 kDa subunit [OS=Saccharomyces cerevisiae S288c]	855082	SAM37; YMR060C	cell organization and biogenesis;transport	membrane;mitochondrion	protein binding	0.245	0.245	1.00
P40496	37S ribosomal protein S25, mitochondrial [OS=Saccharomyces cerevisiae S288c]	854715	RSM25; YIL093C	cell organization and biogenesis;metabolic process	mitochondrion;ribosome	structural molecule activity	0.468	0.468	1.00
P34222	Peptidyl-tRNA hydrolase 2 [OS=Saccharomyces cerevisiae S288c]	852223	PTH2; YBL057C	regulation of biological process	cytoplasm;cytosol;membrane;mitochondrion	catalytic activity;protein binding	0.425	0.425	1.00
Q05648	MIOREX complex component 10 [OS=Saccharomyces cerevisiae S288c]	851876	YDR282C; MRX10; YDR282C		membrane;mitochondrion		0.222	0.222	1.00
P38152	Tricarboxylate transport protein [OS=Saccharomyces cerevisiae S288c]	852594	CTP1; YBR291C	metabolic process;transport	membrane;mitochondrion	structural molecule activity;transporter activity	0.245	0.245	1.00
P25039	Elongation factor G, mitochondrial [OS=Saccharomyces cerevisiae S288c]	850758	MEF1; YLR069C	cell organization and biogenesis;metabolic process	cytosol;mitochondrion	catalytic activity;nucleotide binding;RNA binding	0.099	0.099	1.00
P34224	Uncharacterized protein YBL059W [OS=Saccharomyces cerevisiae S288c]	852221	YBL059W; YBL059W		membrane;mitochondrion		0.468	0.468	1.00
P12686	37S ribosomal protein MRP13, mitochondrial [OS=Saccharomyces cerevisiae S288c]	852975	MRP13; YGR084C	cell organization and biogenesis;metabolic process	mitochondrion;ribosome	structural molecule activity	0.259	0.259	1.00
P36147	Presequence translocated-associated motor subunit pam17, mitochondrial [OS=Saccharomyces cerevisiae S288c]	853939	PAM17; YKR065C	transport	membrane;mitochondrion		0.778	0.778	1.00
P53220	Mitochondrial import inner membrane translocase subunit TIM21 [OS=Saccharomyces cerevisiae S288c]	852921	TIM21; YGR033C	cell organization and biogenesis;transport	membrane;mitochondrion	protein binding	0.501	0.501	1.00
P38172	MIOREX complex component 3 [OS=Saccharomyces cerevisiae S288c]	852181	YBL095W; MRX3; YBL095W		membrane;mitochondrion		0.311	0.311	1.00
P38702	mitochondrial carrier protein LEU5 [OS=Saccharomyces cerevisiae S288c]	856391	LEU5; YHR002W	metabolic process;transport	membrane;mitochondrion	structural molecule activity;transporter activity	0.212	0.212	1.00
P11914	Mitochondrial-processing peptidase subunit alpha [OS=Saccharomyces cerevisiae S288c]	856419	MAS2; YHR024C	metabolic process;transport	membrane;mitochondrion;organelle lumen	catalytic activity;metal ion binding;protein binding	0.202	0.202	1.00
P38088	Glycine--tRNA ligase 1, mitochondrial [OS=Saccharomyces cerevisiae S288c]		GRS1	metabolic process	cytoplasm;mitochondrion;organelle lumen	catalytic activity;nucleotide binding;protein binding	0.049	0.049	1.00
Q06510	Lysophosphatidylcholine acyltransferase [OS=Saccharomyces cerevisiae S288c]	856262	TAZ1; YPR140W	cell organization and biogenesis;metabolic process;transport	membrane;mitochondrion	catalytic activity	0.194	0.194	1.00
P22353	54S ribosomal protein L8, mitochondrial [OS=Saccharomyces cerevisiae S288c]	853382	MRPL8; YJL063C	cell organization and biogenesis;metabolic process	mitochondrion;ribosome	structural molecule activity	0.166	0.166	1.00
Q12251	Uncharacterized mitochondrial carrier YPR011C [OS=Saccharomyces cerevisiae S288c]	856121	YPR011C; YPR011C	metabolic process;transport	membrane;mitochondrion	structural molecule activity;transporter activity	0.389	0.389	1.00
P00127	Cytochrome b-c1 complex subunit 6 [OS=Saccharomyces cerevisiae S288c]	850593	QCR6; YFR033C	metabolic process;transport	membrane;mitochondrion	catalytic activity;transporter activity	0.334	0.334	1.00
Q12233	ATP synthase subunit g, mitochondrial [OS=Saccharomyces cerevisiae S288c]	856131	ATP20; YPR020W	cell organization and biogenesis;metabolic process;transport	membrane;mitochondrion	catalytic activity;transporter activity	0.778	0.778	1.00
Q02950	37S ribosomal protein MRP51, mitochondrial [OS=Saccharomyces cerevisiae S288c]	855985	MRP51; YPL118W	cell organization and biogenesis;metabolic process	mitochondrion;ribosome	structural molecule activity	0.292	0.292	1.00
Q2V2P9	Uncharacterized protein YDR119W-A [OS=Saccharomyces cerevisiae S288c]	3799970	COX26; YDR119W-A		membrane;mitochondrion		1.154	1.154	1.00
Q05867	Uncharacterized protein YLR283W, mitochondrial [OS=Saccharomyces cerevisiae S288c]	850988	YLR283W; YLR283W		endoplasmic reticulum;membrane;mitochondrion		0.11	0.11	1.00

P19516	Cytochrome c oxidase assembly protein COX11, mitochondrial [OS=Saccharomyces cerevisiae S288c]	855971	COX11; YPL132W	cell organization and biogenesis;metabolic process	membrane;mitochondrion;ribosome	metal ion binding	0.274	0.274	1.00
P38323	ATP-dependent clpX-like chaperone, mitochondrial [OS=Saccharomyces cerevisiae S288c]	852528	MCX1; YBR227C	metabolic process	membrane;mitochondrion;organelle lumen	catalytic activity;nucleotide binding	0.07	0.07	1.00
P40530	[Pyruvate dehydrogenase (acetyl-transferring)] kinase 1, mitochondrial [OS=Saccharomyces cerevisiae S288c]	854769	PKP1; YIL042C	metabolic process	mitochondrion;organelle lumen	catalytic activity;nucleotide binding;protein binding	0.116	0.116	1.00
P40581	peroxiredoxin HYR1 [OS=Saccharomyces cerevisiae S288c]	854855	HYR1; YIR037W	metabolic process;response to stimulus	cytoplasm;cytosol;mitochondrion;organelle lumen	antioxidant activity;catalytic activity	0.259	0.259	1.00
P36528	54S ribosomal protein L17, mitochondrial [OS=Saccharomyces cerevisiae S288c]	855469	MRPL17; YNL252C	cell organization and biogenesis;metabolic process	mitochondrion;ribosome	structural molecule activity	0.11	0.11	1.00
P53732	37S ribosomal protein S12, mitochondrial [OS=Saccharomyces cerevisiae S288c]	855772	MRPS12; YNR036C	cell organization and biogenesis;metabolic process	mitochondrion;ribosome	structural molecule activity	0.292	0.292	1.00
Q01532-1	Cysteine proteinase 1, mitochondrial [OS=Saccharomyces cerevisiae S288c]		MCY1	metabolic process;regulation of biological process;response to stimulus	cytoplasm;mitochondrion	catalytic activity;DNA binding;RNA binding	0.086	0.086	1.00
P41735	5-demethoxyubiquinone hydroxylase, mitochondrial [OS=Saccharomyces cerevisiae S288c]	854292	CAT5; YOR125C	metabolic process	membrane;mitochondrion	catalytic activity;metal ion binding	0.155	0.155	1.00
Q12328	Mitochondrial import inner membrane translocase subunit TIM22 [OS=Saccharomyces cerevisiae S288c]	851309	TIM22; YDL217C	cell organization and biogenesis;transport	cytosol;membrane;mitochondrion	protein binding;transporter activity	0.233	0.233	1.00
P40502	Altered inheritance of mitochondria protein 19, mitochondrial [OS=Saccharomyces cerevisiae S288c]	854722	AIM19; YIL087C		membrane;mitochondrion		0.259	0.259	1.00
P32463	Acyl carrier protein, mitochondrial [OS=Saccharomyces cerevisiae S288c]	853642	ACP1; YKL192C	metabolic process;transport	mitochondrion		0.259	0.259	1.00
P38816	thioredoxin reductase 2, mitochondrial [OS=Saccharomyces cerevisiae S288c]	856506	TRR2; YHR106W	cellular homeostasis;metabolic process;regulation of biological process;response to stimulus	cytoplasm;mitochondrion	antioxidant activity;catalytic activity	0.136	0.136	1.00
P43557	Protein fmp32, mitochondrial [OS=Saccharomyces cerevisiae S288c]	850498	FMP32; YFL046W	cell organization and biogenesis	membrane;mitochondrion		0.179	0.179	1.00
P38122	3-methyl-2-oxobutanoate hydroxymethyltransferase [OS=Saccharomyces cerevisiae S288c]	852474	ECM31; YBR176W	metabolic process	mitochondrion	catalytic activity	0.122	0.122	1.00
P14832	peptidyl-prolyl cis-trans isomerase [OS=Saccharomyces cerevisiae S288c]	851733	CPR1; YDR155C	cell differentiation;cell organization and biogenesis;metabolic process;regulation of biological process;transport	cytoplasm;mitochondrion;nucleus	catalytic activity;RNA binding	0.233	0.233	1.00
P04039	Cytochrome c oxidase polypeptide VIII, mitochondrial [OS=Saccharomyces cerevisiae S288c]	851111	COX8; YLR395C	metabolic process;transport	membrane;mitochondrion	catalytic activity;transporter activity	0.778	0.778	1.00
Q02784	Monothiol glutaredoxin-5, mitochondrial [OS=Saccharomyces cerevisiae S288c]	856048	GRX5; YPL059W	cell organization and biogenesis;cellular homeostasis;metabolic process;regulation of biological process;response to stimulus	mitochondrion;organelle lumen	catalytic activity;metal ion binding	0.292	0.292	1.00
Q06630	Mitochondrial homologous recombination protein 1 [OS=Saccharomyces cerevisiae S288c]	851890	MHR1; YDR296W	cell organization and biogenesis;metabolic process;regulation of biological process;response to stimulus	mitochondrion;nucleus	catalytic activity;DNA binding;structural molecule activity	0.166	0.166	1.00
P36520	54S ribosomal protein L10, mitochondrial [OS=Saccharomyces cerevisiae S288c]	855436	MRPL10; YNL284C	cell organization and biogenesis;metabolic process	mitochondrion;ribosome	structural molecule activity	0.116	0.116	1.00
Q03976	37S ribosomal protein S24, mitochondrial [OS=Saccharomyces cerevisiae S288c]	851755	RSM24; YDR175C	cell organization and biogenesis;metabolic process	mitochondrion;ribosome	structural molecule activity	0.202	0.202	1.00
P38909	cytochrome c mitochondrial import factor CYC2 [OS=Saccharomyces cerevisiae S288c]	854202	CYC2; YOR037W	cell organization and biogenesis;metabolic process	membrane;mitochondrion	catalytic activity	0.194	0.194	1.00
Q02863	ubiquitin carboxyl-terminal hydrolase 16 [OS=Saccharomyces cerevisiae S288c]	856032	UBP16; YPL072W	metabolic process	membrane;mitochondrion	catalytic activity	0.072	0.072	1.00
P00431	cytochrome c peroxidase, mitochondrial [OS=Saccharomyces cerevisiae S288c]	853940	CCP1; YKR066C	metabolic process;response to stimulus	mitochondrion;organelle lumen	antioxidant activity;catalytic activity;metal ion binding;protein binding	0.093	0.093	1.00
P10834	protein PET54 [OS=Saccharomyces cerevisiae S288c]	853137	PET54; YGR222W	metabolic process;regulation of biological process	membrane;mitochondrion;organelle lumen	nucleotide binding;RNA binding;translation regulator activity	0.11	0.11	1.00
P46367	Potassium-activated aldehyde dehydrogenase, mitochondrial [OS=Saccharomyces cerevisiae S288c]	854556	ALD4; YOR374W	metabolic process	mitochondrion;organelle lumen	catalytic activity	15.927	17.078	1.07
P40215	External NADH-ubiquinone oxidoreductase 1, mitochondrial	855176	NDE1; YMR14	metabolic process	mitochondrion	catalytic activity;nucleotide	4.043	4.367	1.08

	[OS=Saccharomyces cerevisiae S288c]		5C			binding			
P20967	2-oxoglutarate dehydrogenase, mitochondrial [OS=Saccharomyces cerevisiae S288c]	854681	KGD1; YIL125W	metabolic process	cytosol;mitochondrion;organelle lumen	catalytic activity	5.201	5.764	1.11
Q07500	External NADH-ubiquinone oxidoreductase 2, mitochondrial [OS=Saccharomyces cerevisiae S288c]	851474	NDE2; YDL085W	metabolic process	mitochondrion	catalytic activity;nucleotide binding	2.687	2.981	1.11
P38077	ATP synthase subunit gamma, mitochondrial [OS=Saccharomyces cerevisiae S288c]	852327	ATP3; YBR039W	metabolic process;transport	membrane;mitochondrion	catalytic activity;transporter activity	9	10.103	1.12
P40012	protoporphyrinogen oxidase [OS=Saccharomyces cerevisiae S288c]	856733	HEM14; YER014W	metabolic process	membrane;mitochondrion	catalytic activity	0.947	1.069	1.13
P32843	Mitochondrial escape protein 2 [OS=Saccharomyces cerevisiae S288c]	855348	YME2; YMR302C	cell organization and biogenesis;metabolic process	membrane;mitochondrion	nucleotide binding;RNA binding	1.637	1.859	1.14
P40495	Homoisocitrate dehydrogenase, mitochondrial [OS=Saccharomyces cerevisiae S288c]	854714	LYS12; YIL094C	metabolic process	mitochondrion	catalytic activity;metal ion binding;nucleotide binding	2.481	2.831	1.14
P47127	Altered inheritance of mitochondria protein 24, mitochondrial [OS=Saccharomyces cerevisiae S288c]	853543	AIM24; YJR080C	cell organization and biogenesis	mitochondrion		1.395	1.593	1.14
P40047	Aldehyde dehydrogenase 5, mitochondrial [OS=Saccharomyces cerevisiae S288c]	856804	ALD5; YER073W	metabolic process	mitochondrion;organelle lumen	catalytic activity	0.968	1.106	1.14
P23641	mitochondrial phosphate carrier protein [OS=Saccharomyces cerevisiae S288c]	853540	MIR1; YJR077C	metabolic process;transport	membrane;mitochondrion	protein binding;structural molecule activity;transporter activity	10.365	11.915	1.15
P53598	Succinyl-CoA ligase [ADP-forming] subunit alpha, mitochondrial [OS=Saccharomyces cerevisiae S288c]	854310	LSC1; YOR142W	metabolic process	cytosol;mitochondrion	catalytic activity;nucleotide binding	5.158	5.952	1.15
P38891	Branched-chain-amino-acid aminotransferase, mitochondrial [OS=Saccharomyces cerevisiae S288c]	856615	BAT1; YHR208W	metabolic process;regulation of biological process;response to stimulus	mitochondrion;organelle lumen	catalytic activity	2.511	2.899	1.15
P50085	Prohibitin-2 [OS=Saccharomyces cerevisiae S288c]	853146	PHB2; YGR231C	cell organization and biogenesis;metabolic process;regulation of biological process	membrane;mitochondrion	protein binding	2.511	2.899	1.15
P21801	Succinate dehydrogenase [ubiquinone] iron-sulfur subunit, mitochondrial [OS=Saccharomyces cerevisiae S288c]	850685	SDH2; YLL041C	metabolic process;transport	membrane;mitochondrion	catalytic activity;metal ion binding	12.111	14.013	1.16
P38988	Mitochondrial GTP/GDP carrier protein 1 [OS=Saccharomyces cerevisiae S288c]	851329	GGC1; YDL198C	cell organization and biogenesis;cellular homeostasis;metabolic process;transport	membrane;mitochondrion	structural molecule activity;transporter activity	2.728	3.16	1.16
P33303	Succinate/fumarate mitochondrial transporter [OS=Saccharomyces cerevisiae S288c]	853558	SFC1; YJR095W	metabolic process;transport	membrane;mitochondrion	structural molecule activity;transporter activity	4.995	5.813	1.16
P07246	Alcohol dehydrogenase 3, mitochondrial [OS=Saccharomyces cerevisiae S288c]	855107	ADH3; YMR083W	metabolic process	mitochondrion;organelle lumen	catalytic activity;metal ion binding	2.34	2.728	1.17
P32796-1	Carnitine O-acetyltransferase, mitochondrial [OS=Saccharomyces cerevisiae S288c]	854965	CAT2; YML042W	metabolic process;transport	membrane;mitochondrion	catalytic activity	2.44	2.849	1.17
P00830	ATP synthase subunit beta, mitochondrial [OS=Saccharomyces cerevisiae S288c]	853585	ATP2; YJR121W	metabolic process;transport	cytosol;membrane;mitochondrion	catalytic activity;nucleotide binding;protein binding;transporter activity	17.478	20.544	1.18
P53326	Uncharacterized protein YGR266W [OS=Saccharomyces cerevisiae S288c]	853182	YGR266W; YGR266W		membrane;mitochondrion		0.369	0.442	1.20
P32191	Glycerol-3-phosphate dehydrogenase, mitochondrial [OS=Saccharomyces cerevisiae S288c]	854651	GUT2; YIL155C	metabolic process	membrane;mitochondrion	catalytic activity	2.574	3.14	1.22
Q02486	ARS-binding factor 2, mitochondrial [OS=Saccharomyces cerevisiae S288c]	855094	ABF2; YMR072W	cell organization and biogenesis;metabolic process;regulation of biological process	chromosome;mitochondrion;nucleus	DNA binding	7.377	9	1.22
P00560	Phosphoglycerate kinase [OS=Saccharomyces cerevisiae S288c]	850370	PGK1; YCR012W	metabolic process	cytoplasm;membrane;mitochondrion	catalytic activity;nucleotide binding	2.981	3.642	1.22
P39522	Dihydroxy-acid dehydratase, mitochondrial [OS=Saccharomyces cerevisiae S288c]	853473	ILV3; YJR016C	metabolic process	mitochondrion	catalytic activity;metal ion binding	2.054	2.511	1.22
P53206	putative cysteine synthase [OS=Saccharomyces cerevisiae S288c]	852895	YGR012W; YGR012W	metabolic process		catalytic activity	0.668	0.817	1.22
Q12482	Mitochondrial aspartate-glutamate transporter AGC1 [OS=Saccharomyces cerevisiae S288c]	856132	AGC1; YPR021C	metabolic process;transport	membrane;mitochondrion	structural molecule activity;transporter activity	0.238	0.292	1.23

Q00711	Succinate dehydrogenase [ubiquinone] flavoprotein subunit, mitochondrial [OS=Saccharomyces cerevisiae S288c]	853709	SDH1; YKL148C	metabolic process;transport	membrane;mitochondrion	catalytic activity;nucleotide binding;protein binding	3.072	3.819	1.24
Q08223	Altered inheritance of mitochondria protein 39, mitochondrial [OS=Saccharomyces cerevisiae S288c]	854103	AIM39; YOL053W		cytosol;membrane;mitochondrion		0.468	0.585	1.25
Q00402	Nuclear migration protein NUM1 [OS=Saccharomyces cerevisiae S288c]	851727	NUM1; YDR150W	cell organization and biogenesis;cellular component movement;metabolic process;regulation of biological process;transport	endoplasmic reticulum;mitochondrion	catalytic activity;motor activity;protein binding	0.052	0.065	1.25
Q12166	2-isopropylmalate synthase 2, mitochondrial [OS=Saccharomyces cerevisiae S288c]	854275	LEU9; YOR108W	metabolic process	mitochondrion	catalytic activity;protein binding	1.11	1.39	1.25
Q06493	LETM1 domain-containing protein YLH47, mitochondrial [OS=Saccharomyces cerevisiae S288c]	856243	YLH47; YPR125W	cell organization and biogenesis;transport	membrane;mitochondrion		0.487	0.61	1.25
Q12117	Protein MRH1 [OS=Saccharomyces cerevisiae S288c]	851597	MRH1; YDR033W	transport	endoplasmic reticulum;membrane;mitochondrion	transporter activity	2.831	3.642	1.29
P27929	37S ribosomal protein NAM9, mitochondrial [OS=Saccharomyces cerevisiae S288c]	855585	NAM9; YNL137C	cell organization and biogenesis;metabolic process;regulation of biological process	mitochondrion;ribosome	RNA binding;structural molecule activity	0.301	0.389	1.29
Q08023	Protein FMP25, mitochondrial [OS=Saccharomyces cerevisiae S288c]	850766	FMP25; YLR077W	cell organization and biogenesis	membrane;mitochondrion		0.968	1.254	1.30
P39518	Long-chain-fatty-acid--CoA ligase 2 [OS=Saccharomyces cerevisiae S288c]	856734	FAA2; YER015W	metabolic process;transport	cytoplasm;mitochondrion	catalytic activity;nucleotide binding	0.619	0.802	1.30
P47045	mitochondrial import inner membrane translocase subunit TIM54 [OS=Saccharomyces cerevisiae S288c]	853392	TIM54; YJL054W	cell organization and biogenesis;transport	cytosol;membrane;mitochondrion	transporter activity	0.334	0.433	1.30
P52893	probable alanine aminotransferase, mitochondrial [OS=Saccharomyces cerevisiae S288c]	850778	ALT1; YLR089C	metabolic process	mitochondrion;organelle lumen	catalytic activity	0.346	0.45	1.30
P32891	D-lactate dehydrogenase [cytochrome] 1, mitochondrial [OS=Saccharomyces cerevisiae S288c]	851380	DLD1; YDL174C	metabolic process;transport	membrane;mitochondrion	catalytic activity;nucleotide binding	2.875	3.748	1.30
P04840	Mitochondrial outer membrane protein porin 1 [OS=Saccharomyces cerevisiae S288c]	855669	POR1; YNL055C	cell death;cell organization and biogenesis;cellular homeostasis;regulation of biological process;transport	cytoplasm;membrane;mitochondrion	transporter activity	9	11.743	1.30
P12695	Dihydrolipoyllysine-residue acetyltransferase component of pyruvate dehydrogenase complex, mitochondrial [OS=Saccharomyces cerevisiae S288c]	855653	LAT1; YNL071W	metabolic process	mitochondrion;organelle lumen	catalytic activity	3.758	4.946	1.32
P09624	Dihydrolipoyl dehydrogenase, mitochondrial [OS=Saccharomyces cerevisiae S288c]	850527	LPD1; YFL018C	cellular homeostasis;metabolic process;regulation of biological process	mitochondrion;organelle lumen	catalytic activity;nucleotide binding	9.701	13.03	1.34
P07213	Mitochondrial import receptor subunit TOM70 [OS=Saccharomyces cerevisiae S288c]	855602	TOM70; YNL121C	cell organization and biogenesis;transport	membrane;mitochondrion	protein binding;transporter activity	1.154	1.572	1.36
P36013	NAD-dependent malic enzyme, mitochondrial [OS=Saccharomyces cerevisiae S288c]	853839	MAE1; YKL029C	metabolic process	mitochondrion;organelle lumen	catalytic activity;metal ion binding;nucleotide binding	0.496	0.679	1.37
Q07349	MIOREX complex component 9 [OS=Saccharomyces cerevisiae S288c]	851535	YDL027C; MRX9; YDL027C		endoplasmic reticulum;membrane;mitochondrion		0.931	1.276	1.37
P08417	fumarate hydratase, mitochondrial [OS=Saccharomyces cerevisiae S288c]	855866	FUM1; YPL262W	cell organization and biogenesis;metabolic process	cytoplasm;cytosol;mitochondrion;organelle lumen	catalytic activity	2.562	3.52	1.37
P05626	ATP synthase subunit 4, mitochondrial [OS=Saccharomyces cerevisiae S288c]	856027	ATP4; YPL078C	cell organization and biogenesis;metabolic process;transport	membrane;mitochondrion	catalytic activity;transporter activity	3.084	4.275	1.39
P47052	succinate dehydrogenase [ubiquinone] flavoprotein subunit 2, mitochondrial [OS=Saccharomyces cerevisiae S288c]	853405	YJL045W; YJL045W	metabolic process;transport	membrane;mitochondrion	catalytic activity;nucleotide binding	1.11	1.543	1.39
P53969	Sorting assembly machinery 50 kDa subunit [OS=Saccharomyces cerevisiae S288c]	855705	SAM50; YNL026W	cell organization and biogenesis;transport	membrane;mitochondrion	protein binding;transporter activity	0.269	0.374	1.39
Q12289	mitochondrial carnitine carrier [OS=Saccharomyces cerevisiae S288c]	854267	CRC1; YOR100C	metabolic process;transport	membrane;mitochondrion	structural molecule activity;transporter activity	1.683	2.34	1.39
P00427	Cytochrome c oxidase subunit 6, mitochondrial [OS=Saccharomyces cerevisiae S288c]	856448	COX6; YHR051W	metabolic process;transport	membrane;mitochondrion	catalytic activity;metal ion binding;transporter activity	1.154	1.61	1.40
Q02883	N-acyl-phosphatidylethanolamine-hydrolyzing phospholipase D, mitochondrial [OS=Saccharomyces cerevisiae S288c]	856001	FMP30; YPL103C	metabolic process	membrane;mitochondrion	catalytic activity;metal ion binding	0.304	0.425	1.40

P32335	Protein MSS51, mitochondrial [OS=Saccharomyces cerevisiae S288c]	850900	MSS51; YLR203C	metabolic process;regulation of biological process	membrane;mitochondrion	protein binding;translation regulator activity	0.711	0.995	1.40
P00044	Cytochrome c iso-1 [OS=Saccharomyces cerevisiae S288c]	853507	CYC1; YJR048W	metabolic process;transport	mitochondrion	metal ion binding;protein binding	4.623	6.499	1.41
P40341	Mitochondrial respiratory chain complexes assembly protein YTA12 [OS=Saccharomyces cerevisiae S288c]	855114	YTA12; YMR089C	cell organization and biogenesis;metabolic process;response to stimulus;transport	cytoplasm;membrane;mitochondrion	catalytic activity;metal ion binding;nucleotide binding;protein binding	0.778	1.096	1.41
P54857	lipase 2 [OS=Saccharomyces cerevisiae S288c]	851628	TGL2; YDR058C	metabolic process;transport	mitochondrion	catalytic activity	0.369	0.52	1.41
P00424	Cytochrome c oxidase polypeptide 5A, mitochondrial [OS=Saccharomyces cerevisiae S288c]	855675	COX5A; YNL052W	metabolic process;transport	membrane;mitochondrion	catalytic activity;transporter activity	1.31	1.848	1.41
P53163	54S ribosomal protein L12, mitochondrial [OS=Saccharomyces cerevisiae S288c]	852811	MNP1; YGL068W	cell organization and biogenesis;metabolic process	mitochondrion;ribosome	RNA binding;structural molecule activity	1.31	1.848	1.41
P36046	Mitochondrial intermembrane space import and assembly protein 40 [OS=Saccharomyces cerevisiae S288c]	853639	MIA40; YKL195W	cellular component movement;metabolic process;response to stimulus;transport	membrane;mitochondrion	catalytic activity;protein binding;transporter activity	0.817	1.154	1.41
P32453	Protein ATP11, mitochondrial [OS=Saccharomyces cerevisiae S288c]	855401	ATP11; YNL315C	cell organization and biogenesis	mitochondrion	protein binding	0.413	0.585	1.42
P33759	37S ribosomal protein S5, mitochondrial [OS=Saccharomyces cerevisiae S288c]	852553	MRPS5; YBR251W	cell organization and biogenesis;metabolic process	mitochondrion;ribosome	RNA binding;structural molecule activity	0.413	0.585	1.42
P38523	GrpE protein homolog, mitochondrial [OS=Saccharomyces cerevisiae S288c]	854407	MGE1; YOR232W	metabolic process;regulation of biological process;transport	membrane;mitochondrion;organelle lumen	enzyme regulator activity;nucleotide binding;protein binding	0.438	0.624	1.42
P23644	Mitochondrial import receptor subunit TOM40 [OS=Saccharomyces cerevisiae S288c]	855243	TOM40; YMR203W	transport	cytosol;membrane;mitochondrion	protein binding;transporter activity	0.468	0.668	1.43
P23833	Protein SCO1, mitochondrial [OS=Saccharomyces cerevisiae S288c]	852325	SCO1; YBR037C	cell organization and biogenesis;cellular homeostasis;response to stimulus;transport	membrane;mitochondrion	antioxidant activity;catalytic activity;metal ion binding	0.468	0.668	1.43
P32378	4-hydroxybenzoate polyprenyltransferase, mitochondrial [OS=Saccharomyces cerevisiae S288c]	855778	COQ2; YNR041C	metabolic process;transport	membrane;mitochondrion	catalytic activity	0.468	0.668	1.43
P53889	uncharacterized mitochondrial hydrolase FMP41 [OS=Saccharomyces cerevisiae S288c]	855553	FMP41; YNL168C	metabolic process	mitochondrion	catalytic activity;metal ion binding	0.501	0.719	1.44
Q01852	Mitochondrial import inner membrane translocase subunit TIM44 [OS=Saccharomyces cerevisiae S288c]	854790	TIM44; YIL022W	transport	membrane;mitochondrion	nucleotide binding;protein binding	3.758	5.404	1.44
P32340	Rotenone-insensitive NADH-ubiquinone oxidoreductase, mitochondrial [OS=Saccharomyces cerevisiae S288c]	854919	NDI1; YML120C	metabolic process;regulation of biological process	membrane;mitochondrion;organelle lumen	catalytic activity;nucleotide binding	5.723	8.237	1.44
Q99297	Mitochondrial 2-oxodicarboxylate carrier 2 [OS=Saccharomyces cerevisiae S288c]	854397	ODC2; YOR222W	metabolic process;transport	membrane;mitochondrion	structural molecule activity;transporter activity	0.54	0.778	1.44
P39925	Mitochondrial respiratory chain complexes assembly protein AFG3 [OS=Saccharomyces cerevisiae S288c]	856737	AFG3; YER017C	cell organization and biogenesis;metabolic process;response to stimulus;transport	membrane;mitochondrion	catalytic activity;metal ion binding;nucleotide binding;protein binding	0.569	0.823	1.45
P07257	Cytochrome b-c1 complex subunit 2, mitochondrial [OS=Saccharomyces cerevisiae S288c]	856321	QCR2; YPR191W	metabolic process;transport	membrane;mitochondrion	catalytic activity;metal ion binding;protein binding;transporter activity	17.588	25.49	1.45
P00128	Cytochrome b-c1 complex subunit 7 [OS=Saccharomyces cerevisiae S288c]	852142	QCR7; YDR529C	cell organization and biogenesis;metabolic process;transport	membrane;mitochondrion	catalytic activity;transporter activity	1.783	2.594	1.45
P07256	Cytochrome b-c1 complex subunit 1, mitochondrial [OS=Saccharomyces cerevisiae S288c]	852235	COR1; YBL045C	metabolic process;transport	membrane;mitochondrion	catalytic activity;metal ion binding;protein binding;transporter activity	5.529	8.183	1.48
P53311	Mitochondrial pyruvate carrier 3 [OS=Saccharomyces cerevisiae S288c]	853158	FMP43; MPC3; YGR243W	transport	membrane;mitochondrion	transporter activity	0.778	1.154	1.48
Q02776	Mitochondrial import inner membrane translocase subunit TIM50 [OS=Saccharomyces cerevisiae S288c]	856042	TIM50; YPL063W	cell organization and biogenesis;metabolic process;transport	membrane;mitochondrion	catalytic activity;protein binding;transporter activity	3.771	5.629	1.49
P04710	ADP,ATP carrier protein 1 [OS=Saccharomyces cerevisiae S288c]	855078	AAC1; YMR056C	metabolic process;transport	cytosol;membrane;mitochondrion	structural molecule activity;transporter activity	5.952	9	1.51
P23180	Probable oxidoreductase AIM17 [OS=Saccharomyces cerevisiae S288c]	856365	AIM17; YHL021C	cell organization and biogenesis;metabolic process	mitochondrion	catalytic activity;metal ion binding	0.995	1.512	1.52

P18239	ADP,ATP carrier protein 2 [OS=Saccharomyces cerevisiae S288c]	852250	PET9; YBL030C	cell death;metabolic process;transport	membrane;mitochondrion	structural molecule activity;transporter activity	110.034	167.761	1.52
P16622	Ferrochelatase, mitochondrial [OS=Saccharomyces cerevisiae S288c]	854347	HEM15; YOR176W	metabolic process	membrane;mitochondrion	catalytic activity	2.162	3.329	1.54
P37298	Succinate dehydrogenase [ubiquinone] cytochrome b small subunit, mitochondrial [OS=Saccharomyces cerevisiae S288c]	851758	SDH4; YDR178W	metabolic process;transport	membrane;mitochondrion	catalytic activity;metal ion binding	1.154	1.783	1.55
P21560	Protein CBP3, mitochondrial [OS=Saccharomyces cerevisiae S288c]	855886	CBP3; YPL215W	cell organization and biogenesis;regulation of biological process	membrane;mitochondrion;ribosome		1.219	1.894	1.55
Q12466	tricalbin-1 [OS=Saccharomyces cerevisiae S288c]	854253	TCB1; YOR086C	cell organization and biogenesis;regulation of biological process	endoplasmic reticulum;membrane;mitochondrion	metal ion binding;protein binding	0.369	0.574	1.56
P28241	Isocitrate dehydrogenase [NAD] subunit 2, mitochondrial [OS=Saccharomyces cerevisiae S288c]	854303	IDH2; YOR136W	metabolic process	mitochondrion;organelle lumen	catalytic activity;metal ion binding;nucleotide binding;protein binding;RNA binding	2.34	3.642	1.56
P36516	54S ribosomal protein L3, mitochondrial [OS=Saccharomyces cerevisiae S288c]	855039	MRPL3; YMR024W	cell organization and biogenesis;metabolic process;response to stimulus	mitochondrion;nucleus;ribosome	catalytic activity;RNA binding;structural molecule activity	0.172	0.269	1.56
P32266	Dynamin-like GTPase MGM1, mitochondrial [OS=Saccharomyces cerevisiae S288c]	854386	MGM1; YOR211C	cell organization and biogenesis	membrane;mitochondrion	catalytic activity;nucleotide binding;protein binding	0.179	0.28	1.56
P29704	squalene synthase [OS=Saccharomyces cerevisiae S288c]	856597	ERG9; YHR190W	metabolic process	endoplasmic reticulum;membrane;mitochondrion	catalytic activity	0.222	0.35	1.58
Q06892	NADH kinase POS5, mitochondrial [OS=Saccharomyces cerevisiae S288c]	855913	POS5; YPL188W	metabolic process;response to stimulus	mitochondrion;organelle lumen	catalytic activity;nucleotide binding	0.233	0.369	1.58
P38885	Altered inheritance of mitochondria protein 46, mitochondrial [OS=Saccharomyces cerevisiae S288c]	856606	AIM46; YHR199C		mitochondrion	catalytic activity	0.245	0.389	1.59
Q02981	ABC1 family protein YPL109C, mitochondrial [OS=Saccharomyces cerevisiae S288c]	855995	YPL109C; YPL109C		mitochondrion		0.259	0.413	1.59
P43617	Uncharacterized mitochondrial carrier YFR045W [OS=Saccharomyces cerevisiae S288c]	850606	YFR045W; YFR045W	metabolic process;transport	membrane;mitochondrion	structural molecule activity;transporter activity	0.259	0.413	1.59
P36534	54S ribosomal protein L40, mitochondrial [OS=Saccharomyces cerevisiae S288c]	855930	MRPL40; YPL173W	cell organization and biogenesis;metabolic process	mitochondrion;ribosome	protein binding;structural molecule activity	0.259	0.413	1.59
P38300	Inner membrane mitoribosome receptor MBA1, mitochondrial [OS=Saccharomyces cerevisiae S288c]	852483	MBA1; YBR185C	cell organization and biogenesis;regulation of biological process	membrane;mitochondrion		0.259	0.413	1.59
P38271	Protein OPY1 [OS=Saccharomyces cerevisiae S288c]	852426	OPY1; YBR129C		cytoplasm;mitochondrion		0.274	0.438	1.60
P36521	54S ribosomal protein L11, mitochondrial [OS=Saccharomyces cerevisiae S288c]	851325	MRPL11; YDL202W	cell organization and biogenesis;metabolic process	mitochondrion;ribosome	structural molecule activity	0.274	0.438	1.60
Q00873	cytochrome c1 heme lyase [OS=Saccharomyces cerevisiae S288c]	853775	CYT2; YKL087C	cell organization and biogenesis;metabolic process	membrane;mitochondrion	catalytic activity;metal ion binding	0.359	0.585	1.63
P49095	Glycine dehydrogenase (Decarboxylating), mitochondrial [OS=Saccharomyces cerevisiae S288c]	855227	GCV2; YMR189W	metabolic process	cytosol;mitochondrion	catalytic activity	0.389	0.638	1.64
P00927	Threonine dehydratase, mitochondrial [OS=Saccharomyces cerevisiae S288c]	856819	ILV1; YER086W	metabolic process	cytoplasm;mitochondrion	catalytic activity	0.425	0.701	1.65
Q08822	Probable electron transfer flavoprotein-ubiquinone oxidoreductase, mitochondrial [OS=Saccharomyces cerevisiae S288c]	854538	CIR2; YOR356W	metabolic process;transport	membrane;mitochondrion	catalytic activity;metal ion binding;nucleotide binding	1.488	2.481	1.67
P06208-1	2-isopropylmalate synthase [OS=Saccharomyces cerevisiae S288c]	855619	LEU4; YNL104C	metabolic process	cytoplasm;mitochondrion	catalytic activity;protein binding	1.728	2.888	1.67
P34227	Mitochondrial peroxiredoxin PRX1 [OS=Saccharomyces cerevisiae S288c]	852215	PRX1; YBL064C	cellular homeostasis;metabolic process;regulation of biological process;response to stimulus	mitochondrion	antioxidant activity;catalytic activity	1.637	2.793	1.71
P19414	Aconitate hydratase, mitochondrial [OS=Saccharomyces cerevisiae S288c]	851013	ACO1; YLR304C	cell organization and biogenesis;metabolic process;transport	cytoplasm;cytosol;mitochondrion;organelle lumen	catalytic activity;DNA binding;metal ion binding	10.053	17.233	1.71
P32445	Single-stranded DNA-binding protein RIM1, mitochondrial [OS=Saccharomyces cerevisiae S288c]	850395	RIM1; YCR028C-A	cell organization and biogenesis;metabolic process;regulation of biological process	mitochondrion	DNA binding	0.668	1.154	1.73

P30624	Long-chain-fatty-acid--CoA ligase 1 [OS=Saccharomyces cerevisiae S288c]	854495	FAA1; YOR317W	metabolic process;transport	endoplasmic reticulum;membrane;mitochondrion	catalytic activity;nucleotide binding	2.427	4.259	1.75
Q06485	Autophagy-related protein 33 [OS=Saccharomyces cerevisiae S288c]	851070	ATG33; YLR356W	cell communication;metabolic process;response to stimulus	membrane;mitochondrion		0.778	1.371	1.76
P81449	ATP synthase subunit e, mitochondrial [OS=Saccharomyces cerevisiae S288c]	851922	TIM11; YDR322C-A	cell organization and biogenesis;metabolic process;transport	membrane;mitochondrion	catalytic activity;structural molecule activity;transporter activity	0.778	1.371	1.76
P19262	Dihydrolipoyllysine-residue succinyltransferase component of 2-oxoglutarate dehydrogenase complex, mitochondrial [OS=Saccharomyces cerevisiae S288c]	851726	KGD2; YDR148C	cell organization and biogenesis;metabolic process	mitochondrion	catalytic activity;protein binding	1.721	3.062	1.78
P37299	cytochrome b-c1 complex subunit 10 [OS=Saccharomyces cerevisiae S288c]	856390	QCR10; YHR001W-A	metabolic process;transport	membrane;mitochondrion	catalytic activity;transporter activity	2.981	5.31	1.78
Q06405	ATP synthase subunit f, mitochondrial [OS=Saccharomyces cerevisiae S288c]	851983	ATP17; YDR377W	metabolic process;transport	membrane;mitochondrion	catalytic activity;transporter activity	2.981	5.31	1.78
P09457	ATP synthase subunit 5, mitochondrial [OS=Saccharomyces cerevisiae S288c]	851892	ATP5; YDR298C	metabolic process;transport	membrane;mitochondrion	catalytic activity;transporter activity	5.813	10.365	1.78
P32317	Protein AFG1 [OS=Saccharomyces cerevisiae S288c]	856658	AFG1; YEL052W	cell organization and biogenesis;metabolic process;response to stimulus;transport	membrane;mitochondrion	catalytic activity;nucleotide binding	0.468	0.848	1.81
P36101	tRNA threonylcarbamoyladenosine dehydratase 2 [OS=Saccharomyces cerevisiae S288c]	853841	TCD2; YKL027W; YKL027W	metabolic process	cytosol;membrane;mitochondrion	catalytic activity;nucleotide binding	0.334	0.616	1.84
P37293	N-terminal acetyltransferase 2 [OS=Saccharomyces cerevisiae S288c]	853050	NAT2; YGR147C	metabolic process	cytoplasm;mitochondrion	catalytic activity	0.389	0.73	1.88
P53736	Uncharacterized protein YNR040W [OS=Saccharomyces cerevisiae S288c]	855776	YNR040W; YNR040W		mitochondrion		0.389	0.73	1.88
P16547	Mitochondrial outer membrane protein OM45 [OS=Saccharomyces cerevisiae S288c]	854670	OM45; YIL136W		membrane;mitochondrion		82.176	157.489	1.92
P36112	MICOS complex subunit Mic60 [OS=Saccharomyces cerevisiae S288c]	853886	FCJ1; MIC60; YKR016W	cell organization and biogenesis;transport	membrane;mitochondrion		5.629	10.788	1.92
P50945	MICOS complex subunit MIC27 [OS=Saccharomyces cerevisiae S288c]	855623	AIM37; MIC27; YNL100W	cell organization and biogenesis	membrane;mitochondrion		0.501	0.968	1.93
P36066	Protein MRG3-like [OS=Saccharomyces cerevisiae S288c]	853725	YKL133C; YKL133C	metabolic process	membrane;mitochondrion	protein binding	0.334	0.655	1.96
Q04013	Citrate/oxoglutarate carrier protein [OS=Saccharomyces cerevisiae S288c]	855282	YHM2; YMR241W	cell organization and biogenesis;metabolic process;transport	membrane;mitochondrion	DNA binding;structural molecule activity;transporter activity	1.512	2.981	1.97
P33421	Succinate dehydrogenase [ubiquinone] cytochrome b subunit, mitochondrial [OS=Saccharomyces cerevisiae S288c]	853716	SDH3; YKL141W	cell organization and biogenesis;metabolic process;transport	membrane;mitochondrion	catalytic activity;metal ion binding	0.638	1.276	2.00
P06168	ketol-acid reductoisomerase, mitochondrial [OS=Saccharomyces cerevisiae S288c]	851069	ILV5; YLR355C	cell organization and biogenesis;metabolic process	mitochondrion	catalytic activity;DNA binding;metal ion binding	3.394	6.814	2.01
P53266	Cytochrome oxidase assembly protein SHY1 [OS=Saccharomyces cerevisiae S288c]	853009	SHY1; YGR112W	cell organization and biogenesis	membrane;mitochondrion	protein binding	0.668	1.346	2.01
P41921	glutathione reductase [OS=Saccharomyces cerevisiae S288c]	856014	GLR1; YPL091W	cellular homeostasis;metabolic process;regulation of biological process;response to stimulus	cytoplasm;cytosol;mitochondrion	antioxidant activity;catalytic activity;nucleotide binding	0.075	0.155	2.07
Q12032	Altered inheritance of mitochondria protein 41, mitochondrial [OS=Saccharomyces cerevisiae S288c]	854390	AIM41; YOR215C		mitochondrion	catalytic activity	0.778	1.61	2.07
P38169	Kynurenine 3-monooxygenase [OS=Saccharomyces cerevisiae S288c]	852179	BNA4; YBL098W	metabolic process	membrane;mitochondrion	catalytic activity;nucleotide binding	0.083	0.172	2.07
P21375	Fumarate reductase 2 [OS=Saccharomyces cerevisiae S288c]	853510	OSM1; YJR051W	metabolic process	endoplasmic reticulum;mitochondrion	catalytic activity;nucleotide binding	0.077	0.16	2.08
P40961	prohibitin-1 [OS=Saccharomyces cerevisiae S288c]	853033	PHB1; YGR132C	cell organization and biogenesis;metabolic process;regulation of biological process	membrane;mitochondrion	protein binding	1.336	2.793	2.09

P40015	inositol phosphosphingolipids phospholipase C [OS=Saccharomyces cerevisiae S288c]	856739	ISC1; YER019W	metabolic process;response to stimulus	endoplasmic reticulum;membrane;mitochondrion	catalytic activity;metal ion binding	0.101	0.212	2.10
P38771	Ribosome-recycling factor, mitochondrial [OS=Saccharomyces cerevisiae S288c]	856433	RRF1; YHR038W	cell organization and biogenesis;metabolic process	mitochondrion	RNA binding	0.145	0.311	2.14
P38884	Altered inheritance of mitochondria protein 18, mitochondrial [OS=Saccharomyces cerevisiae S288c]	856605	AIM18; YHR198C		mitochondrion	catalytic activity	0.145	0.311	2.14
P32939	GTP-binding protein ypt7 [OS=Saccharomyces cerevisiae S288c]	855012	YPT7; YML001W	cell communication;cell organization and biogenesis;metabolic process;regulation of biological process;response to stimulus;transport	cytosol;endosome;membrane;mitochondrion;vacuole	catalytic activity;nucleotide binding;protein binding	0.166	0.359	2.16
P07143	Cytochrome c1, heme protein, mitochondrial [OS=Saccharomyces cerevisiae S288c]	854231	CYT1; YOR065W	metabolic process;transport	cytosol;membrane;mitochondrion	metal ion binding	7.377	16.013	2.17
Q04172	Sensitive to high expression protein 9, mitochondrial [OS=Saccharomyces cerevisiae S288c]	852002	SHE9; YDR393W	cell organization and biogenesis	membrane;mitochondrion		0.172	0.374	2.17
P35180	mitochondrial import receptor subunit Tom20 [OS=Saccharomyces cerevisiae S288c]	852973	TOM20; YGR082W	cell organization and biogenesis;transport	membrane;mitochondrion	protein binding;transporter activity	0.194	0.425	2.19
P19955	37S ribosomal protein YMR-31, mitochondrial [OS=Saccharomyces cerevisiae S288c]	850610	YMR31; YFR049W	cell organization and biogenesis;metabolic process	mitochondrion;ribosome	catalytic activity;protein binding;structural molecule activity	1.154	2.594	2.25
P18496	Mitochondrial ATPase complex subunit ATP10 [OS=Saccharomyces cerevisiae S288c]	851109	ATP10; YLR393W	cell organization and biogenesis	cytoplasm;membrane;mitochondrion	protein binding	0.259	0.585	2.26
P28625	Protein YIM1 [OS=Saccharomyces cerevisiae S288c]	855183	YIM1; YMR152W	metabolic process;response to stimulus	cytoplasm;endoplasmic reticulum;mitochondrion	catalytic activity;metal ion binding	0.274	0.624	2.28
P08067	cytochrome b-c1 complex subunit Rieske, mitochondrial [OS=Saccharomyces cerevisiae S288c]	856689	RIP1; YEL024W	metabolic process;transport	membrane;mitochondrion	catalytic activity;metal ion binding;transporter activity	9	20.544	2.28
Q07651	SUR7 family protein FMP45 [OS=Saccharomyces cerevisiae S288c]	851304	FMP45; YDL222C	cell differentiation;cell organization and biogenesis	membrane;mitochondrion		3.924	9	2.29
Q03327	Mitochondrial fusion and transport protein Ugo1 [OS=Saccharomyces cerevisiae S288c]	852081	UGO1; YDR470C	cell organization and biogenesis;metabolic process	membrane;mitochondrion	protein binding;structural molecule activity	0.304	0.701	2.31
P46998	mitochondrial membrane protein FMP33 [OS=Saccharomyces cerevisiae S288c]	853279	FMP33; YJL161W		membrane;mitochondrion		0.334	0.778	2.33
P36060	NADH-cytochrome b5 reductase 2 [OS=Saccharomyces cerevisiae S288c]	853707	MCR1; YKL150W	metabolic process;response to stimulus	membrane;mitochondrion	catalytic activity;protein binding	2.793	6.848	2.45
P37267	Assembly factor cbp4 [OS=Saccharomyces cerevisiae S288c]		CBP4	cell organization and biogenesis	membrane;mitochondrion		0.52	1.31	2.52
P40098	uncharacterized mitochondrial membrane protein FMP10 [OS=Saccharomyces cerevisiae S288c]	856931	FMP10; YER182W		membrane;mitochondrion		1.069	2.793	2.61
Q12029	Probable mitochondrial transport protein fsf1 [OS=Saccharomyces cerevisiae S288c]	854445	FSF1; YOR271C	transport	membrane;mitochondrion	transporter activity	0.624	1.637	2.62
P21306	ATP synthase subunit epsilon, mitochondrial [OS=Saccharomyces cerevisiae S288c]	855857	ATP15; YPL271W	metabolic process;transport	membrane;mitochondrion	catalytic activity;transporter activity	0.778	2.162	2.78
P38969	Pentamidine resistance factor, mitochondrial [OS=Saccharomyces cerevisiae S288c]	854440	PNT1; YOR266W	cell organization and biogenesis;response to stimulus	membrane;mitochondrion		0.166	0.468	2.82
P21954	Isocitrate dehydrogenase [NADP], mitochondrial [OS=Saccharomyces cerevisiae S288c]	851493	IDP1; YDL066W	metabolic process	mitochondrion	catalytic activity;metal ion binding;nucleotide binding;protein binding	4.412	12.594	2.85
Q06567	ABC1 family protein MCP2 [OS=Saccharomyces cerevisiae S288c]	850955	YLR253W; MCP2; YLR253W	cell organization and biogenesis	membrane;mitochondrion		0.068	0.218	3.21
P39006	Phosphatidylserine decarboxylase proenzyme 1, mitochondrial [OS=Saccharomyces cerevisiae S288c]	855552	PSD1; YNL169C	metabolic process;regulation of biological process	membrane;mitochondrion	catalytic activity	0.083	0.269	3.24
P32839	Mitochondrial chaperone BCS1 [OS=Saccharomyces cerevisiae S288c]	851981	BCS1; YDR375C	cell organization and biogenesis;metabolic process;response to stimulus;transport	cytosol;membrane;mitochondrion	catalytic activity;nucleotide binding;transporter activity	0.093	0.304	3.27
Q06678	54S ribosomal protein L35, mitochondrial [OS=Saccharomyces cerevisiae S288c]	851921	MRPL35; YDR322W	cell organization and biogenesis;metabolic process	mitochondrion;ribosome	structural molecule activity	0.093	0.304	3.27
P42949	Mitochondrial import inner membrane translocase subunit TIM16 [OS=Saccharomyces cerevisiae S288c]	853340	PAM16; YJL104W	transport	membrane;mitochondrion	protein binding	0.233	0.874	3.75

P39515	mitochondrial import inner membrane translocase subunit Tim17 [OS=Saccharomyces cerevisiae S288c]	853298	TIM17; YJL143W	cell organization and biogenesis;transport	membrane;mitochondrion	protein binding;transporter activity	0.334	1.371	4.10
P39952	Mitochondrial inner membrane protein OXA1 [OS=Saccharomyces cerevisiae S288c]	856898	OXA1; YER154W	cell organization and biogenesis;transport	membrane;mitochondrion	transporter activity	0.778	3.217	4.13
P25087	Sterol 24-C-methyltransferase [OS=Saccharomyces cerevisiae S288c]	855003	ERG6; YML008C	metabolic process	endoplasmic reticulum;membrane;mitochondrion	catalytic activity	0.425	1.894	4.46
Q01574	acetyl-coenzyme A synthetase 1 [OS=Saccharomyces cerevisiae S288c]	851245	ACS1; YAL054C	cell organization and biogenesis;metabolic process	cytoplasm;cytosol;endoplasmic reticulum;mitochondrion;nucleus	catalytic activity;nucleotide binding	0.624	2.793	4.48
P08525	Cytochrome b-c1 complex subunit 8 [OS=Saccharomyces cerevisiae S288c]	853273	QCR8; YJL166W	metabolic process;transport	membrane;mitochondrion	catalytic activity;transporter activity	0.585	2.981	5.10
P30902	ATP synthase subunit d, mitochondrial [OS=Saccharomyces cerevisiae S288c]	853853	ATP7; YKL016C	metabolic process;transport	membrane;mitochondrion	catalytic activity;transporter activity	1.683	9	5.35
Q07914	mitochondrial import inner membrane translocase subunit TIM14 [OS=Saccharomyces cerevisiae S288c]	850694	PAM18; YLR008C	regulation of biological process;transport	membrane;mitochondrion	enzyme regulator activity;protein binding;transporter activity	0.334	3.217	9.63

Supplemental Table S14. The relative concentrations of proteins in mitochondria purified from *ups2Δ* cells cultured with or without LCA. Mitochondria were purified from *ups2Δ* cells recovered on day 4 of cell culturing with or without LCA. Mass spectrometry-based identification and quantitation of proteins recovered in purified mitochondria were performed as described in Materials and methods. The exponentially modified protein abundance index (emPAI) provided by the ″Proteome Discoverer″ software was used to calculate the relative abundance of mitochondrial proteins between specified datasets. Data on the relative concentrations of mitochondrial proteins are presented as means of 2 independent experiments.

Accession	Description	Entrez Gene ID	Gene ID	Biological Process	Cellular Component	Molecular Function	emPAI *ups2*	emPAI *ups2* + LCA	RATIO *ups2* + LCA / *ups2*
P25578	CDP-diacylglycerol--glycerol-3-phosphate 3-phosphatidyltransferase [OS=Saccharomyces cerevisiae S288c]	850352	PGS1; YCL004W	metabolic process	mitochondrion	catalytic activity;nucleotide binding	0.374	0.083	0.22
P36526	54S ribosomal protein L27, mitochondrial [OS=Saccharomyces cerevisiae S288c]	852585	MRPL27; YBR282W	cell organization and biogenesis;metabolic process	mitochondrion;ribosome	structural molecule activity	0.995	0.259	0.26
P24720	Protein MNE1 [OS=Saccharomyces cerevisiae S288c]	854532	MNE1; YOR350C	metabolic process	cytoplasm;mitochondrion;organelle lumen		0.194	0.061	0.31
Q01574	acetyl-coenzyme A synthetase 1 [OS=Saccharomyces cerevisiae S288c]	851245	ACS1; YAL054C	cell organization and biogenesis;metabolic process	cytoplasm;cytosol;endoplasmic reticulum;mitochondrion;nucleus	catalytic activity;nucleotide binding	0.947	0.354	0.37
P36527	54S ribosomal protein L28, mitochondrial [OS=Saccharomyces cerevisiae S288c]	852073	MRPL28; YDR462W	cell organization and biogenesis;metabolic process	mitochondrion;ribosome	structural molecule activity	0.52	0.233	0.45
P12687	54S ribosomal protein L2, mitochondrial [OS=Saccharomyces cerevisiae S288c]	855727	MRP7; YNL005C	cell organization and biogenesis;metabolic process	mitochondrion;ribosome	catalytic activity;RNA binding;structural molecule activity	2.899	1.31	0.45
Q03799	37S ribosomal protein S8, mitochondrial [OS=Saccharomyces cerevisiae S288c]	855190	MRPS8; YMR158W	cell organization and biogenesis;metabolic process	mitochondrion;ribosome	structural molecule activity	0.425	0.194	0.46
Q06630	Mitochondrial homologous recombination protein 1 [OS=Saccharomyces cerevisiae S288c]	851890	MHR1; YDR296W	cell organization and biogenesis;metabolic process;regulation of biological process;response to stimulus	mitochondrion;nucleus	catalytic activity;DNA binding;structural molecule activity	0.359	0.166	0.46
P54115	Magnesium-activated aldehyde dehydrogenase, cytosolic [OS=Saccharomyces cerevisiae S288c]	856044	ALD6; YPL061W	metabolic process;response to stimulus	cytoplasm;cytosol;mitochondrion	catalytic activity	0.346	0.16	0.46
P32378	4-hydroxybenzoate polyprenyltransferase, mitochondrial [OS=Saccharomyces cerevisiae S288c]	855778	COQ2; YNR041C	metabolic process;transport	membrane;mitochondrion	catalytic activity	0.292	0.136	0.47
P49954	probable hydrolase NIT3 [OS=Saccharomyces cerevisiae S288c]	851065	NIT3; YLR351C	metabolic process	cytoplasm;mitochondrion	catalytic activity	0.274	0.129	0.47
P38746	Obg-like ATPase homolog [OS=Saccharomyces cerevisiae S288c]	856372	YLF2; YHL014C		mitochondrion	nucleotide binding	0.194	0.093	0.48
P38705	Serine--tRNA ligase, mitochondrial [OS=Saccharomyces cerevisiae S288c]	856402	DIA4; YHR011W	metabolic process	cytoplasm;mitochondrion;organelle lumen	catalytic activity;nucleotide binding	0.15	0.072	0.48
P36056	37S ribosomal protein S22, mitochondrial [OS=Saccharomyces cerevisiae S288c]	853701	RSM22; YKL155C	cell organization and biogenesis;metabolic process	mitochondrion;ribosome	catalytic activity;structural molecule activity	0.125	0.061	0.49
P15108	ATP-dependent molecular chaperone HSC82 [OS=Saccharomyces cerevisiae S288c]	855224	HSC82; YMR186W	cell organization and biogenesis;metabolic process;response to stimulus	cytoplasm;membrane;mitochondrion	catalytic activity;nucleotide binding;protein binding	0.602	0.299	0.50
Q08223	Altered inheritance of mitochondria protein 39, mitochondrial [OS=Saccharomyces cerevisiae S288c]	854103	AIM39; YOL053W		cytosol;membrane;mitochondrion		0.468	0.259	0.55
P39112	Exoribonuclease II, mitochondrial [OS=Saccharomyces cerevisiae S288c]	855331	DSS1; YMR287C	metabolic process	mitochondrion;organelle lumen	catalytic activity;RNA binding	0.233	0.134	0.58
Q02486	ARS-binding factor 2, mitochondrial [OS=Saccharomyces cerevisiae S288c]	855094	ABF2; YMR072W	cell organization and biogenesis;metabolic process;regulation of biological process	chromosome;mitochondrion;nucleus	DNA binding	33.551	19.309	0.58
Q03430	37S ribosomal protein RSM28, mitochondrial [OS=Saccharomyces cerevisiae S288c]	852105	RSM28; YDR494W	cell organization and biogenesis;metabolic process	mitochondrion;ribosome	structural molecule activity	3.489	2.008	0.58

P49334	Mitochondrial import receptor subunit tom22 [OS=Saccharomyces cerevisiae S288c]	855592	TOM22; YNL131W	cell organization and biogenesis;transport	membrane;mitochondrion	protein binding;transporter activity	1.154	0.668	0.58
P53732	37S ribosomal protein S12, mitochondrial [OS=Saccharomyces cerevisiae S288c]	855772	MRPS12; YNR036C	cell organization and biogenesis;metabolic process	mitochondrion;ribosome	structural molecule activity	1.154	0.668	0.58
P40033	37S ribosomal protein rsm18, mitochondrial [OS=Saccharomyces cerevisiae S288c]	856776	RSM18; YER050C	cell organization and biogenesis;metabolic process	mitochondrion;ribosome	structural molecule activity	0.995	0.585	0.59
P23369	54S ribosomal protein L25, mitochondrial [OS=Saccharomyces cerevisiae S288c]	852967	MRPL25; YGR076C	cell organization and biogenesis;metabolic process	mitochondrion;ribosome	structural molecule activity	0.874	0.52	0.59
P33421	Succinate dehydrogenase [ubiquinone] cytochrome b subunit, mitochondrial [OS=Saccharomyces cerevisiae S288c]	853716	SDH3; YKL141W	cell organization and biogenesis;metabolic process;transport	membrane;mitochondrion	catalytic activity;metal ion binding	0.638	0.389	0.61
P32939	GTP-binding protein ypt7 [OS=Saccharomyces cerevisiae S288c]	855012	YPT7; YML001W	cell communication;cell organization and biogenesis;metabolic process;regulation of biological process;response to stimulus;transport	cytosol;endosome;membrane;mitochondrion;vacuole	catalytic activity;nucleotide binding;protein binding	0.585	0.359	0.61
P27697	Atypical kinase COQ8, mitochondrial [OS=Saccharomyces cerevisiae S288c]	852758	COQ8; YGL119W	metabolic process	membrane;mitochondrion;organelle lumen	catalytic activity;nucleotide binding	0.453	0.283	0.62
Q04599	54S ribosomal protein L1, mitochondrial [OS=Saccharomyces cerevisiae S288c]	851694	MRPL1; YDR116C	cell organization and biogenesis;metabolic process	mitochondrion;ribosome	RNA binding;structural molecule activity	0.438	0.274	0.63
P38152	Tricarboxylate transport protein [OS=Saccharomyces cerevisiae S288c]	852594	CTP1; YBR291C	metabolic process;transport	membrane;mitochondrion	structural molecule activity;transporter activity	0.389	0.245	0.63
P36528	54S ribosomal protein L17, mitochondrial [OS=Saccharomyces cerevisiae S288c]	855469	MRPL17; YNL252C	cell organization and biogenesis;metabolic process	mitochondrion;ribosome	structural molecule activity	0.369	0.233	0.63
P38910	10 kDa heat shock protein, mitochondrial [OS=Saccharomyces cerevisiae S288c]	854185	HSP10; YOR020C	cell organization and biogenesis;metabolic process;response to stimulus;transport	cytoplasm;mitochondrion;organelle lumen	metal ion binding;nucleotide binding;protein binding	2.162	1.371	0.63
P38702	mitochondrial carrier protein LEU5 [OS=Saccharomyces cerevisiae S288c]	856391	LEU5; YHR002W	metabolic process;transport	membrane;mitochondrion	structural molecule activity;transporter activity	0.334	0.212	0.63
P40458	Autophagy-related protein 32 [OS=Saccharomyces cerevisiae S288c]	854660	ATG32; YIL146C	metabolic process	membrane;mitochondrion;vacuole	protein binding	0.318	0.202	0.64
P22438	Methionine--tRNA ligase, mitochondrial [OS=Saccharomyces cerevisiae S288c]	853081	MSM1; YGR171C	metabolic process	cytoplasm;cytosol;mitochondrion;organelle lumen	catalytic activity;nucleotide binding	0.212	0.136	0.64
P25626	54S ribosomal protein IMG1, mitochondrial [OS=Saccharomyces cerevisiae S288c]	850413	IMG1; YCR046C	cell organization and biogenesis;metabolic process	mitochondrion;ribosome	structural molecule activity	1.031	0.701	0.68
P40364	Mitochondrial peculiar membrane protein 1 [OS=Saccharomyces cerevisiae S288c]	853379	MPM1; YJL066C		membrane;mitochondrion		0.848	0.585	0.69
P04803	Tryptophan--tRNA ligase, mitochondrial [OS=Saccharomyces cerevisiae S288c]	851861	MSW1; YDR268W	metabolic process	cytoplasm;mitochondrion;organelle lumen	catalytic activity;nucleotide binding	0.668	0.468	0.70
Q12289	mitochondrial carnitine carrier [OS=Saccharomyces cerevisiae S288c]	854267	CRC1; YOR100C	metabolic process;transport	membrane;mitochondrion	structural molecule activity;transporter activity	1.994	1.404	0.70
Q08245	protein ZEO1 [OS=Saccharomyces cerevisiae S288c]	854040	ZEO1; YOL109W	cell organization and biogenesis	membrane;mitochondrion	protein binding	2.981	2.162	0.73
P00431	cytochrome c peroxidase, mitochondrial [OS=Saccharomyces cerevisiae S288c]	853940	CCP1; YKR066C	metabolic process;response to stimulus	mitochondrion;organelle lumen	antioxidant activity;catalytic activity;metal ion binding;protein binding	0.557	0.425	0.76
P38169	Kynurenine 3-monooxygenase [OS=Saccharomyces cerevisiae S288c]	852179	BNA4; YBL098W	metabolic process	membrane;mitochondrion	catalytic activity;nucleotide binding	0.487	0.374	0.77
P38323	ATP-dependent clpX-like chaperone, mitochondrial [OS=Saccharomyces cerevisiae S288c]	852528	MCX1; YBR227C	metabolic process	membrane;mitochondrion;organelle lumen	catalytic activity;nucleotide binding	0.403	0.311	0.77
P53881	54S ribosomal protein L22, mitochondrial [OS=Saccharomyces cerevisiae S288c]	855544	MRPL22; YNL177C	cell organization and biogenesis;metabolic process	mitochondrion;ribosome	RNA binding;structural molecule activity	0.931	0.73	0.78
P36516	54S ribosomal protein L3, mitochondrial [OS=Saccharomyces cerevisiae S288c]	855039	MRPL3; YMR024W	cell organization and biogenesis;metabolic process;response to stimulus	mitochondrion;nucleus;ribosome	catalytic activity;RNA binding;structural molecule activity	0.61	0.487	0.80
P53166	ATP-dependent RNA helicase mrh4, mitochondrial [OS=Saccharomyces	852816	MRH4; YGL064	cell organization and biogenesis;metabolic process	mitochondrion;organelle lumen	catalytic activity;nucleotide	0.501	0.403	0.80

	cerevisiae S288c]		C			binding;RNA binding			
P36517	54S ribosomal protein L4, mitochondrial [OS=Saccharomyces cerevisiae S288c]	851160	MRPL4; YLR439W	cell organization and biogenesis;metabolic process	mitochondrion;ribosome	structural molecule activity	1.081	0.874	0.81
P25038	Translation initiation factor IF-2, mitochondrial [OS=Saccharomyces cerevisiae S288c]	854135	IFM1; YOL023W	cell organization and biogenesis;metabolic process	mitochondrion;nucleus	catalytic activity;nucleotide binding;RNA binding	0.369	0.299	0.81
P38088	Glycine--tRNA ligase 1, mitochondrial [OS=Saccharomyces cerevisiae S288c]		GRS1	metabolic process	cytoplasm;mitochondrion;organelle lumen	catalytic activity;nucleotide binding;protein binding	0.334	0.271	0.81
P38297	Mitofusin FZO1 [OS=Saccharomyces cerevisiae S288c]	852477	FZO1; YBR179C	cell organization and biogenesis	membrane;mitochondrion	catalytic activity;nucleotide binding;protein binding	0.304	0.248	0.82
P36520	54S ribosomal protein L10, mitochondrial [OS=Saccharomyces cerevisiae S288c]	855436	MRPL10; YNL284C	cell organization and biogenesis;metabolic process	mitochondrion;ribosome	structural molecule activity	1.404	1.154	0.82
P17558	37S ribosomal protein PET123, mitochondrial [OS=Saccharomyces cerevisiae S288c]	854329	PET123; YOR158W	cell organization and biogenesis;metabolic process	mitochondrion;ribosome	structural molecule activity	2.162	1.783	0.82
Q01163	37S ribosomal protein S23, mitochondrial [OS=Saccharomyces cerevisiae S288c]	852748	RSM23; YGL129C	cell organization and biogenesis;metabolic process	mitochondrion;ribosome	catalytic activity;nucleotide binding;RNA binding;structural molecule activity	2.065	1.707	0.83
P28817	3-hydroxyisobutyryl-CoA hydrolase, mitochondrial [OS=Saccharomyces cerevisiae S288c]	851606	EHD3; YDR036C	metabolic process	mitochondrion	catalytic activity	0.887	0.743	0.84
Q06567	ABC1 family protein MCP2 [OS=Saccharomyces cerevisiae S288c]	850955	YLR253W; MCP2; YLR253W	cell organization and biogenesis	membrane;mitochondrion		0.693	0.585	0.84
P40098	uncharacterized mitochondrial membrane protein FMP10 [OS=Saccharomyces cerevisiae S288c]	856931	FMP10; YER182W		membrane;mitochondrion		2.793	2.36	0.84
Q03327	Mitochondrial fusion and transport protein Ugo1 [OS=Saccharomyces cerevisiae S288c]	852081	UGO1; YDR470C	cell organization and biogenesis;metabolic process	membrane;mitochondrion	protein binding;structural molecule activity	1.424	1.219	0.86
P32332	Mitochondrial oxaloacetate transport protein [OS=Saccharomyces cerevisiae S288c]	853739	OAC1; YKL120W	metabolic process;transport	membrane;mitochondrion	structural molecule activity;transporter activity	5.813	4.995	0.86
P21801	Succinate dehydrogenase [ubiquinone] iron-sulfur subunit, mitochondrial [OS=Saccharomyces cerevisiae S288c]	850685	SDH2; YLL041C	metabolic process;transport	membrane;mitochondrion	catalytic activity;metal ion binding	28.552	24.809	0.87
P27929	37S ribosomal protein NAM9, mitochondrial [OS=Saccharomyces cerevisiae S288c]	855585	NAM9; YNL137C	cell organization and biogenesis;metabolic process;regulation of biological process	mitochondrion;ribosome	RNA binding;structural molecule activity	1.865	1.683	0.90
P15424	ATP-dependent RNA helicase MSS116, mitochondrial [OS=Saccharomyces cerevisiae S288c]	851775	MSS116; YDR194C	metabolic process;regulation of biological process	mitochondrion;organelle lumen	catalytic activity;nucleotide binding;RNA binding	1.387	1.268	0.91
Q12031	Mitochondrial 2-methylisocitrate lyase [OS=Saccharomyces cerevisiae S288c]	856114	ICL2; YPR006C	metabolic process	mitochondrion;organelle lumen	catalytic activity	2.793	2.793	1.00
P43635	Citrate synthase 3, mitochondrial [OS=Saccharomyces cerevisiae S288c]	856107	CIT3; YPR001W	metabolic process	mitochondrion	catalytic activity	2.914	2.914	1.00
P25087	Sterol 24-C-methyltransferase [OS=Saccharomyces cerevisiae S288c]	855003	ERG6; YML008C	metabolic process	endoplasmic reticulum;membrane;mitochondrion	catalytic activity	1.894	1.894	1.00
P39952	Mitochondrial inner membrane protein OXA1 [OS=Saccharomyces cerevisiae S288c]	856898	OXA1; YER154W	cell organization and biogenesis;transport	membrane;mitochondrion	transporter activity	3.87	3.87	1.00
Q04013	Citrate/oxoglutarate carrier protein [OS=Saccharomyces cerevisiae S288c]	855282	YHM2; YMR241W	cell organization and biogenesis;metabolic process;transport	membrane;mitochondrion	DNA binding;structural molecule activity;transporter activity	4.623	4.623	1.00
P53266	Cytochrome oxidase assembly protein SHY1 [OS=Saccharomyces cerevisiae S288c]	853009	SHY1; YGR112W	cell organization and biogenesis	membrane;mitochondrion	protein binding	1.555	1.555	1.00
Q12029	Probable mitochondrial transport protein fsf1 [OS=Saccharomyces cerevisiae S288c]	854445	FSF1; YOR271C	transport	membrane;mitochondrion	transporter activity	2.36	2.36	1.00
P53721	Respiratory supercomplex factor 2, mitochondrial [OS=Saccharomyces cerevisiae S288c]	855752	RCF2; YNR018W	cell organization and biogenesis	membrane;mitochondrion		9	9	1.00
P38079	protein YRO2 [OS=Saccharomyces cerevisiae S288c]	852343	YRO2; YBR054W	transport	endoplasmic reticulum;membrane;mitochondrion	transporter activity	3.125	3.125	1.00

Q06668	Methyltransferase OMS1, mitochondrial [OS=Saccharomyces cerevisiae S288c]	851911	OMS1; YDR316W	metabolic process	membrane;mitochondrion	catalytic activity	0.484	0.484	1.00
P00447	Superoxide dismutase [Mn], mitochondrial [OS=Saccharomyces cerevisiae S288c]	856399	SOD2; YHR008C	metabolic process;response to stimulus	mitochondrion;organelle lumen	antioxidant activity;catalytic activity;metal ion binding	2.455	2.455	1.00
P00044	Cytochrome c iso-1 [OS=Saccharomyces cerevisiae S288c]	853507	CYC1; YJR048W	metabolic process;transport	mitochondrion	metal ion binding;protein binding	3.217	3.217	1.00
P40508	Uncharacterized protein YIL077C [OS=Saccharomyces cerevisiae S288c]	854733	YIL077C; YIL077C		mitochondrion		0.896	0.896	1.00
P53969	Sorting assembly machinery 50 kDa subunit [OS=Saccharomyces cerevisiae S288c]	855705	SAM50; YNL026W	cell organization and biogenesis;transport	membrane;mitochondrion	protein binding;transporter activity	0.487	0.487	1.00
P32331	Carrier protein YMC1, mitochondrial [OS=Saccharomyces cerevisiae S288c]	856171	YMC1; YPR058W	metabolic process;transport	membrane;mitochondrion;vacuole	structural molecule activity;transporter activity	1.581	1.581	1.00
P53230	Phosphatidate cytidylyltransferase, mitochondrial [OS=Saccharomyces cerevisiae S288c]	852937	TAM41; YGR046W	metabolic process	membrane;mitochondrion;organelle lumen	catalytic activity	0.668	0.668	1.00
P00950	Phosphoglycerate mutase 1 [OS=Saccharomyces cerevisiae S288c]	853705	GPM1; YKL152C	metabolic process;regulation of biological process	cytoplasm;cytosol;membrane;mitochondrion	catalytic activity	1.929	1.929	1.00
Q06143	mitochondrial dicarboxylate transporter [OS=Saccharomyces cerevisiae S288c]	851063	DIC1; YLR348C	metabolic process;transport	membrane;mitochondrion	structural molecule activity;transporter activity	0.931	0.931	1.00
P40416	Iron-sulfur clusters transporter ATM1, mitochondrial [OS=Saccharomyces cerevisiae S288c]	855347	ATM1; YMR301C	cellular homeostasis;metabolic process;transport	membrane;mitochondrion	catalytic activity;nucleotide binding;transporter activity	0.453	0.453	1.00
P28737	Protein MSP1 [OS=Saccharomyces cerevisiae S288c]	852915	MSP1; YGR028W	cell organization and biogenesis;metabolic process;regulation of biological process;response to stimulus;transport	membrane;mitochondrion;nucleus	catalytic activity;nucleotide binding;protein binding;RNA binding	0.778	0.778	1.00
P47039	Probable kynurenine--oxoglutarate transaminase BNA3 [OS=Saccharomyces cerevisiae S288c]	853386	BNA3; YJL060W	metabolic process;regulation of biological process	cytoplasm;mitochondrion	catalytic activity	0.468	0.468	1.00
Q12251	Uncharacterized mitochondrial carrier YPR011C [OS=Saccharomyces cerevisiae S288c]	856121	YPR011C; YPR011C	metabolic process;transport	membrane;mitochondrion	structural molecule activity;transporter activity	0.73	0.73	1.00
P32387	54S ribosomal protein L41, mitochondrial [OS=Saccharomyces cerevisiae S288c]	852014	MRP20; YDR405W	cell organization and biogenesis;metabolic process	mitochondrion;ribosome	nucleotide binding;protein binding;RNA binding;structural molecule activity	0.501	0.501	1.00
P36101	tRNA threonylcarbamoyladenosine dehydratase 2 [OS=Saccharomyces cerevisiae S288c]	853841	TCD2; YKL027W; YKL027W	metabolic process	cytosol;membrane;mitochondrion	catalytic activity;nucleotide binding	0.334	0.334	1.00
P37299	cytochrome b-c1 complex subunit 10 [OS=Saccharomyces cerevisiae S288c]	856390	QCR10; YHR001W-A	metabolic process;transport	membrane;mitochondrion	catalytic activity;transporter activity	2.981	2.981	1.00
P53889	uncharacterized mitochondrial hydrolase FMP41 [OS=Saccharomyces cerevisiae S288c]	855553	FMP41; YNL168C	metabolic process	mitochondrion	catalytic activity;metal ion binding	0.501	0.501	1.00
Q04172	Sensitive to high expression protein 9, mitochondrial [OS=Saccharomyces cerevisiae S288c]	852002	SHE9; YDR393W	cell organization and biogenesis	membrane;mitochondrion		0.269	0.269	1.00
P21771	37S ribosomal protein S28, mitochondrial [OS=Saccharomyces cerevisiae S288c]	851937	MRPS28; YDR337W	cell organization and biogenesis;metabolic process	mitochondrion;ribosome	RNA binding;structural molecule activity	0.624	0.624	1.00
P50945	MICOS complex subunit MIC27 [OS=Saccharomyces cerevisiae S288c]	855623	AIM37; MIC27; YNL100W	cell organization and biogenesis	membrane;mitochondrion		0.719	0.719	1.00
P42900	Sigma-like sequence protein 1, mitochondrial [OS=Saccharomyces cerevisiae S288c]	850830	SLS1; YLR139C	cell organization and biogenesis;metabolic process	membrane;mitochondrion	protein binding	0.266	0.266	1.00
Q3E824	Uncharacterized protein YOR020W-A [OS=Saccharomyces cerevisiae S288c]	1466480	YOR020W-A; YOR020W-A	metabolic process;transport	membrane;mitochondrion		2.162	2.162	1.00
P36521	54S ribosomal protein L11, mitochondrial [OS=Saccharomyces cerevisiae S288c]	851325	MRPL11; YDL202W	cell organization and biogenesis;metabolic process	mitochondrion;ribosome	structural molecule activity	0.833	0.833	1.00

P18496	Mitochondrial ATPase complex subunit ATP10 [OS=Saccharomyces cerevisiae S288c]	851109	ATP10; YLR393W	cell organization and biogenesis	cytoplasm;membrane;mitochondrion	protein binding	0.585	0.585	1.00
Q06698	Putative ATP-dependent RNA helicase YLR419W [OS=Saccharomyces cerevisiae S288c]	851137	YLR419W; YLR419W	metabolic process	cytoplasm;mitochondrion;nucleus	catalytic activity;nucleotide binding;protein binding;RNA binding	0.11	0.11	1.00
Q12467	MIOREX complex component 4 [OS=Saccharomyces cerevisiae S288c]	855935	YPL168W; MRX4; YPL168W		membrane;mitochondrion		0.318	0.318	1.00
P32839	Mitochondrial chaperone BCS1 [OS=Saccharomyces cerevisiae S288c]	851981	BCS1; YDR375C	cell organization and biogenesis;metabolic process;response to stimulus;transport	cytosol;membrane;mitochondrion	catalytic activity;nucleotide binding;transporter activity	0.194	0.194	1.00
P32904	54S ribosomal protein L6, mitochondrial [OS=Saccharomyces cerevisiae S288c]	856552	MRPL6; YHR147C	cell organization and biogenesis;metabolic process	mitochondrion;ribosome	RNA binding;structural molecule activity	1.054	1.054	1.00
P10834	protein PET54 [OS=Saccharomyces cerevisiae S288c]	853137	PET54; YGR222W	metabolic process;regulation of biological process	membrane;mitochondrion;organelle lumen	nucleotide binding;RNA binding;translation regulator activity	0.369	0.369	1.00
Q12375	mitochondrial ornithine transporter 1 [OS=Saccharomyces cerevisiae S288c]	854297	ORT1; YOR130C	metabolic process;transport	membrane;mitochondrion	structural molecule activity;transporter activity	0.54	0.54	1.00
Q01532-1	Cysteine proteinase 1, mitochondrial [OS=Saccharomyces cerevisiae S288c]			metabolic process;regulation of biological process;response to stimulus	cytoplasm;mitochondrion	catalytic activity;DNA binding;RNA binding	0.179	0.179	1.00
P39677	Ribosome-releasing factor 2, mitochondrial [OS=Saccharomyces cerevisiae S288c]	853342	MEF2; YJL102W	cell organization and biogenesis;metabolic process	cytosol;mitochondrion	catalytic activity;nucleotide binding;RNA binding	0.116	0.116	1.00
P33759	37S ribosomal protein S5, mitochondrial [OS=Saccharomyces cerevisiae S288c]	852553	MRPS5; YBR251W	cell organization and biogenesis;metabolic process	mitochondrion;ribosome	RNA binding;structural molecule activity	0.413	0.413	1.00
P41911	Glycerol-3-phosphate dehydrogenase [NAD(+)] 2, mitochondrial [OS=Saccharomyces cerevisiae S288c]	854095	GPD2; YOL059W	metabolic process	cytoplasm;cytosol;mitochondrion	catalytic activity;nucleotide binding;protein binding	0.186	0.186	1.00
P40496	37S ribosomal protein S25, mitochondrial [OS=Saccharomyces cerevisiae S288c]	854715	RSM25; YIL093C	cell organization and biogenesis;metabolic process	mitochondrion;ribosome	structural molecule activity	0.668	0.668	1.00
P10849	mitochondrial transcription factor 2 [OS=Saccharomyces cerevisiae S288c]	851517	MTF2; YDL044C	metabolic process	mitochondrion;organelle lumen	protein binding;RNA binding	0.166	0.166	1.00
P40015	inositol phosphosphingolipids phospholipase C [OS=Saccharomyces cerevisiae S288c]	856739	ISC1; YER019W	metabolic process;response to stimulus	endoplasmic reticulum;membrane;mitochondrion	catalytic activity;metal ion binding	0.334	0.334	1.00
P48237	Mitochondrial group I intron splicing factor CCM1 [OS=Saccharomyces cerevisiae S288c]	853053	CCM1; YGR150C	cell organization and biogenesis;metabolic process	mitochondrion	RNA binding	0.089	0.089	1.00
P48525	Glutamate--tRNA ligase, mitochondrial [OS=Saccharomyces cerevisiae S288c]	854124	MSE1; YOL033W	cell organization and biogenesis;metabolic process	cytoplasm;mitochondrion;organelle lumen	catalytic activity;nucleotide binding;RNA binding	0.145	0.145	1.00
P40159	Uncharacterized protein YNL208W [OS=Saccharomyces cerevisiae S288c]	855513	YNL208W; YNL208W		membrane;mitochondrion;ribosome		0.292	0.292	1.00
P53219	Abhydrolase domain-containing protein IMO32 [OS=Saccharomyces cerevisiae S288c]	852919	IMO32; YGR031W	metabolic process;transport	mitochondrion	catalytic activity	0.245	0.245	1.00
P18409	Mitochondrial distribution and morphology protein 10 [OS=Saccharomyces cerevisiae S288c]	851223	MDM10; YAL010C	cell organization and biogenesis;transport	membrane;mitochondrion	protein binding	0.16	0.16	1.00
P36519	54S ribosomal protein L7, mitochondrial [OS=Saccharomyces cerevisiae S288c]	851823	MRPL7; YDR237W	cell organization and biogenesis;metabolic process	mitochondrion;ribosome	RNA binding;structural molecule activity	0.136	0.136	1.00
Q07821	Iron-sulfur assembly protein 1 [OS=Saccharomyces cerevisiae S288c]	850632	ISA1; YLL027W	cell organization and biogenesis;metabolic process	mitochondrion;organelle lumen	metal ion binding;structural molecule activity	0.136	0.136	1.00
Q05779	Ubiquinone biosynthesis protein COQ9, mitochondrial [OS=Saccharomyces cerevisiae S288c]	850898	COQ9; YLR201C	metabolic process	membrane;mitochondrion		0.166	0.166	1.00
P25372	Thioredoxin-3, mitochondrial [OS=Saccharomyces cerevisiae S288c]	850444	TRX3; YCR083W	cellular homeostasis;metabolic process;regulation of biological process;response to stimulus;transport	cytoplasm;mitochondrion	catalytic activity	0.52	0.52	1.00
Q12233	ATP synthase subunit g, mitochondrial [OS=Saccharomyces cerevisiae S288c]	856131	ATP20; YPR020W	cell organization and biogenesis;metabolic process;transport	membrane;mitochondrion	catalytic activity;transporter activity	0.778	0.778	1.00
P21306	ATP synthase subunit epsilon, mitochondrial [OS=Saccharomyces cerevisiae S288c]	855857	ATP15; YPL271W	metabolic process;transport	membrane;mitochondrion	catalytic activity;transporter activity	0.778	0.778	1.00

P19516	Cytochrome c oxidase assembly protein COX11, mitochondrial [OS=Saccharomyces cerevisiae S288c]	855971	COX11; YPL132W	cell organization and biogenesis;metabolic process	membrane;mitochondrion;ribosome	metal ion binding	0.274	0.274	1.00
Q06236	Mitochondrial inner membrane protein SHH4 [OS=Saccharomyces cerevisiae S288c]	850861	SHH4; YLR164W	metabolic process	membrane;mitochondrion	catalytic activity;metal ion binding	0.778	0.778	1.00
P39515	mitochondrial import inner membrane translocase subunit Tim17 [OS=Saccharomyces cerevisiae S288c]	853298	TIM17; YJL143W	cell organization and biogenesis;transport	membrane;mitochondrion	protein binding;transporter activity	0.778	0.778	1.00
P03875	Putative COX1/OXI3 intron 1 protein [OS=Saccharomyces cerevisiae S288c]	854593	AI1; Q0050	metabolic process;transport	mitochondrion	catalytic activity;metal ion binding;transporter activity	0.081	0.081	1.00
P36523	54S ribosomal protein L15, mitochondrial [OS=Saccharomyces cerevisiae S288c]	851022	MRPL15; YLR312W-A	cell organization and biogenesis;metabolic process	mitochondrion;ribosome	catalytic activity;RNA binding;structural molecule activity	0.155	0.155	1.00
P38909	cytochrome c mitochondrial import factor CYC2 [OS=Saccharomyces cerevisiae S288c]	854202	CYC2; YOR037W	cell organization and biogenesis;metabolic process	membrane;mitochondrion	catalytic activity	0.194	0.194	1.00
P08525	Cytochrome b-c1 complex subunit 8 [OS=Saccharomyces cerevisiae S288c]	853273	QCR8; YJL166W	metabolic process;transport	membrane;mitochondrion	catalytic activity;transporter activity	0.585	0.585	1.00
P32463	Acyl carrier protein, mitochondrial [OS=Saccharomyces cerevisiae S288c]	853642	ACP1; YKL192C	metabolic process;transport	mitochondrion		0.259	0.259	1.00
P25270	rRNA methyltransferase 1, mitochondrial [OS=Saccharomyces cerevisiae S288c]	854376	MRM1; YOR201C	metabolic process	mitochondrion	catalytic activity;RNA binding	0.274	0.274	1.00
P81451	ATP synthase subunit K, mitochondrial [OS=Saccharomyces cerevisiae S288c]	854077	ATP19; YOL077W-A	cell organization and biogenesis;metabolic process;transport	membrane;mitochondrion	catalytic activity;transporter activity	1.154	1.154	1.00
P53305	Mitochondrial 37S ribosomal protein S27 [OS=Saccharomyces cerevisiae S288c]	853129	RSM27; YGR215W	cell organization and biogenesis;metabolic process	mitochondrion;ribosome	structural molecule activity	0.468	0.468	1.00
P36531	54S ribosomal protein L36, mitochondrial [OS=Saccharomyces cerevisiae S288c]	852419	MRPL36; YBR122C	cell organization and biogenesis;metabolic process	mitochondrion;ribosome	structural molecule activity	0.292	0.292	1.00
P32493	ATPase expression protein 1, mitochondrial [OS=Saccharomyces cerevisiae S288c]	855086	AEP1; YMR064W	regulation of biological process	mitochondrion	translation regulator activity	0.15	0.15	1.00
P22354	54S ribosomal protein L20, mitochondrial [OS=Saccharomyces cerevisiae S288c]	853960	MRPL20; YKR085C	cell organization and biogenesis;metabolic process	mitochondrion;ribosome	structural molecule activity	0.389	0.389	1.00
Q2V2P9	Uncharacterized protein YDR119W-A [OS=Saccharomyces cerevisiae S288c]	3799970	COX26; YDR119W-A		membrane;mitochondrion		1.154	1.154	1.00
P03879	Intron-encoded RNA maturase bI4 [OS=Saccharomyces cerevisiae S288c]	854582	BI4; Q0120	metabolic process	membrane;mitochondrion	catalytic activity;RNA binding	0.179	0.179	1.00
P38341	MICOS complex subunit MIC12 [OS=Saccharomyces cerevisiae S288c]	852566	AIM5; MIC12; YBR262C	cell organization and biogenesis	membrane;mitochondrion		0.292	0.292	1.00
P38816	thioredoxin reductase 2, mitochondrial [OS=Saccharomyces cerevisiae S288c]	856506	TRR2; YHR106W	cellular homeostasis;metabolic process;regulation of biological process;response to stimulus	cytoplasm;mitochondrion	antioxidant activity;catalytic activity	0.136	0.136	1.00
Q03218	Mitochondrial metal transporter 1 [OS=Saccharomyces cerevisiae S288c]	855215	MMT1; YMR177W	cellular homeostasis;regulation of biological process;response to stimulus;transport	membrane;mitochondrion	transporter activity	0.096	0.096	1.00
P04039	Cytochrome c oxidase polypeptide VIII, mitochondrial [OS=Saccharomyces cerevisiae S288c]	851111	COX8; YLR395C	metabolic process;transport	membrane;mitochondrion	catalytic activity;transporter activity	0.778	0.778	1.00
Q04748	Protein SOV1, mitochondrial [OS=Saccharomyces cerevisiae S288c]	855088	SOV1; YMR066W		mitochondrion		0.041	0.041	1.00
Q04487	Mitochondrial inner membrane protein SHH3 [OS=Saccharomyces cerevisiae S288c]	855145	SHH3; YMR118C	metabolic process	membrane;mitochondrion	catalytic activity;metal ion binding	0.194	0.194	1.00
P40858	54S ribosomal protein L49, mitochondrial [OS=Saccharomyces cerevisiae S288c]	853349	MRPL49; YJL096W	cell organization and biogenesis;metabolic process	mitochondrion;ribosome	RNA binding;structural molecule activity	0.212	0.212	1.00
P10174	cytochrome c oxidase subunit 7 [OS=Saccharomyces cerevisiae S288c]	855298	COX7; YMR256C	metabolic process;transport	membrane;mitochondrion	catalytic activity;transporter activity	1.154	1.154	1.00
Q03640	Tricalbin-3 [OS=Saccharomyces cerevisiae S288c]	854903	TCB3; YML072C	cell organization and biogenesis;regulation of biological process	endoplasmic reticulum;membrane;mitochondrion	metal ion binding;protein binding	0.806	0.863	1.07
P00560	Phosphoglycerate kinase [OS=Saccharomyces cerevisiae S288c]	850370	PGK1; YCR012W	metabolic process	cytoplasm;membrane;mitochondrion	catalytic activity;nucleotide binding	2.981	3.299	1.11
P07806	Valine--tRNA ligase, mitochondrial [OS=Saccharomyces cerevisiae S288c]	852986	VAS1; YGR094W	metabolic process;regulation of biological process	cytoplasm;cytosol;mitochondrion	catalytic activity;nucleotide binding	0.422	0.468	1.11

P32191	Glycerol-3-phosphate dehydrogenase, mitochondrial [OS=Saccharomyces cerevisiae S288c]	854651	GUT2; YIL155C	metabolic process	membrane;mitochondrion	catalytic activity	4.555	5.127	1.13
Q07349	MIOREX complex component 9 [OS=Saccharomyces cerevisiae S288c]	851535	YDL027C; MRX9; YDL027C		endoplasmic reticulum;membrane;mitochondrion		1.913	2.162	1.13
P22136	ATPase expression protein 2, mitochondrial [OS=Saccharomyces cerevisiae S288c]	855325	AEP2; YMR282C	metabolic process;regulation of biological process	mitochondrion	RNA binding	0.668	0.756	1.13
Q08023	Protein FMP25, mitochondrial [OS=Saccharomyces cerevisiae S288c]	850766	FMP25; YLR077W	cell organization and biogenesis	membrane;mitochondrion		1.106	1.254	1.13
P39518	Long-chain-fatty-acid--CoA ligase 2 [OS=Saccharomyces cerevisiae S288c]	856734	FAA2; YER015W	metabolic process;transport	cytoplasm;mitochondrion	catalytic activity;nucleotide binding	0.619	0.708	1.14
P53292	37S ribosomal protein S35, mitochondrial [OS=Saccharomyces cerevisiae S288c]	853075	MRPS35; YGR165W	cell organization and biogenesis;metabolic process	mitochondrion;ribosome	structural molecule activity	2.02	2.311	1.14
Q12482	Mitochondrial aspartate-glutamate transporter AGC1 [OS=Saccharomyces cerevisiae S288c]	856132	AGC1; YPR021C	metabolic process;transport	membrane;mitochondrion	structural molecule activity;transporter activity	0.407	0.468	1.15
P46681	D-lactate dehydrogenase [cytochrome] 2, mitochondrial [OS=Saccharomyces cerevisiae S288c]	851376	DLD2; YDL178W	metabolic process	mitochondrion;organelle lumen	catalytic activity;nucleotide binding;protein binding	0.951	1.102	1.16
P40086	Cytochrome c oxidase assembly protein cox15 [OS=Saccharomyces cerevisiae S288c]	856884	COX15; YER141W	cell organization and biogenesis;metabolic process;transport	membrane;mitochondrion	catalytic activity;transporter activity	1.154	1.346	1.17
P43616	Cys-Gly metallodipeptidase dug1 [OS=Saccharomyces cerevisiae S288c]	850605	DUG1; YFR044C	metabolic process	cytoplasm;mitochondrion;ribosome	catalytic activity;metal ion binding	0.848	0.995	1.17
P15179	Aspartate--tRNA ligase, mitochondrial [OS=Saccharomyces cerevisiae S288c]	856000	MSD1; YPL104W	metabolic process	cytoplasm;mitochondrion;organelle lumen	catalytic activity;nucleotide binding	0.379	0.455	1.20
P11914	Mitochondrial-processing peptidase subunit alpha [OS=Saccharomyces cerevisiae S288c]	856419	MAS2; YHR024C	metabolic process;transport	membrane;mitochondrion;organelle lumen	catalytic activity;metal ion binding;protein binding	0.905	1.089	1.20
P38626	NADH-cytochrome b5 reductase 1 [OS=Saccharomyces cerevisiae S288c]	854768	CBR1; YIL043C	metabolic process	endoplasmic reticulum;membrane;mitochondrion;nucleus	catalytic activity	0.957	1.154	1.21
Q08968	UPF0061 protein FMP40 [OS=Saccharomyces cerevisiae S288c]	855879	FMP40; YPL222W		mitochondrion	protein binding	1.015	1.228	1.21
P47045	mitochondrial import inner membrane translocase subunit TIM54 [OS=Saccharomyces cerevisiae S288c]	853392	TIM54; YJL054W	cell organization and biogenesis;transport	cytosol;membrane;mitochondrion	transporter activity	0.54	0.655	1.21
P38969	Pentamidine resistance factor, mitochondrial [OS=Saccharomyces cerevisiae S288c]	854440	PNT1; YOR266W	cell organization and biogenesis;response to stimulus	membrane;mitochondrion		0.585	0.711	1.22
P40012	protoporphyrinogen oxidase [OS=Saccharomyces cerevisiae S288c]	856733	HEM14; YER014W	metabolic process	membrane;mitochondrion	catalytic activity	1.336	1.637	1.23
Q03976	37S ribosomal protein S24, mitochondrial [OS=Saccharomyces cerevisiae S288c]	851755	RSM24; YDR175C	cell organization and biogenesis;metabolic process	mitochondrion;ribosome	structural molecule activity	0.738	0.905	1.23
P40047	Aldehyde dehydrogenase 5, mitochondrial [OS=Saccharomyces cerevisiae S288c]	856804	ALD5; YER073W	metabolic process	mitochondrion;organelle lumen	catalytic activity	1.762	2.162	1.23
Q12117	Protein MRH1 [OS=Saccharomyces cerevisiae S288c]	851597	MRH1; YDR033W	transport	endoplasmic reticulum;membrane;mitochondrion	transporter activity	7.254	9	1.24
P48527	Tyrosine--tRNA ligase, mitochondrial [OS=Saccharomyces cerevisiae S288c]	856007	MSY1; YPL097W	metabolic process	cytoplasm;cytosol;mitochondrion;organelle lumen	catalytic activity;nucleotide binding;RNA binding	0.417	0.52	1.25
P36066	Protein MRG3-like [OS=Saccharomyces cerevisiae S288c]	853725	YKL133C; YKL133C	metabolic process	membrane;mitochondrion	protein binding	0.433	0.54	1.25
P21375	Fumarate reductase 2 [OS=Saccharomyces cerevisiae S288c]	853510	OSM1; YJR051W	metabolic process	endoplasmic reticulum;mitochondrion	catalytic activity;nucleotide binding	0.45	0.562	1.25
P09440	C-1-tetrahydrofolate synthase, mitochondrial [OS=Saccharomyces cerevisiae S288c]	852378	MIS1; YBR084W	metabolic process	cytosol;mitochondrion	catalytic activity;nucleotide binding	0.9	1.127	1.25
P39006	Phosphatidylserine decarboxylase proenzyme 1, mitochondrial [OS=Saccharomyces cerevisiae S288c]	855552	PSD1; YNL169C	metabolic process;regulation of biological process	membrane;mitochondrion	catalytic activity	0.487	0.61	1.25
P32611	54S ribosomal protein RML2, mitochondrial [OS=Saccharomyces cerevisiae S288c]	856660	RML2; YEL050C	cell organization and biogenesis;metabolic process	mitochondrion;ribosome	catalytic activity;RNA binding;structural	0.509	0.638	1.25

						molecule activity			
P10507	mitochondrial-processing peptidase subunit beta [OS=Saccharomyces cerevisiae S288c]	850860	MAS1; YLR163C	metabolic process;transport	mitochondrion;organelle lumen	catalytic activity;metal ion binding;protein binding	0.616	0.778	1.26
P48526	Isoleucine--tRNA ligase, mitochondrial [OS=Saccharomyces cerevisiae S288c]	856067	ISM1; YPL040C	cell organization and biogenesis;metabolic process;regulation of biological process	cytoplasm;cytosol;mitochondrion;organelle lumen	catalytic activity;nucleotide binding	0.169	0.215	1.27
P51998	54S ribosomal protein yml6, mitochondrial [OS=Saccharomyces cerevisiae S288c]	854983	YML6; YML025C	cell organization and biogenesis;metabolic process	mitochondrion;ribosome	RNA binding;structural molecule activity	0.73	0.931	1.28
P32453	Protein ATP11, mitochondrial [OS=Saccharomyces cerevisiae S288c]	855401	ATP11; YNL315C	cell organization and biogenesis	mitochondrion	protein binding	0.778	0.995	1.28
P49095	Glycine dehydrogenase (Decarboxylating), mitochondrial [OS=Saccharomyces cerevisiae S288c]	855227	GCV2; YMR189W	metabolic process	cytosol;mitochondrion	catalytic activity	1.683	2.162	1.28
P28241	Isocitrate dehydrogenase [NAD] subunit 2, mitochondrial [OS=Saccharomyces cerevisiae S288c]	854303	IDH2; YOR136W	metabolic process	mitochondrion;organelle lumen	catalytic activity;metal ion binding;nucleotide binding;protein binding;RNA binding	5.449	7.031	1.29
P38771	Ribosome-recycling factor, mitochondrial [OS=Saccharomyces cerevisiae S288c]	856433	RRF1; YHR038W	cell organization and biogenesis;metabolic process	mitochondrion	RNA binding	0.968	1.254	1.30
P48360	Probable NADPH:adrenodoxin oxidoreductase, mitochondrial [OS=Saccharomyces cerevisiae S288c]	851982	ARH1; YDR376W	cellular homeostasis;metabolic process;transport	cytoplasm;membrane;mitochondrion;organelle lumen	catalytic activity	0.346	0.45	1.30
P28834	Isocitrate dehydrogenase [NAD] subunit 1, mitochondrial [OS=Saccharomyces cerevisiae S288c]	855691	IDH1; YNL037C	metabolic process;transport	cytosol;mitochondrion;organelle lumen	catalytic activity;metal ion binding;nucleotide binding;protein binding;RNA binding	9	11.743	1.30
P04710	ADP,ATP carrier protein 1 [OS=Saccharomyces cerevisiae S288c]	855078	AAC1; YMR056C	metabolic process;transport	cytosol;membrane;mitochondrion	structural molecule activity;transporter activity	6.848	9	1.31
P39676	Flavohemoprotein [OS=Saccharomyces cerevisiae S288c]	853149	YHB1; YGR234W	metabolic process;response to stimulus	cytoplasm;cytosol;mitochondrion;organelle lumen	catalytic activity;metal ion binding;protein binding	0.551	0.73	1.32
P37293	N-terminal acetyltransferase 2 [OS=Saccharomyces cerevisiae S288c]	853050	NAT2; YGR147C	metabolic process	cytoplasm;mitochondrion	catalytic activity	0.551	0.73	1.32
P38072	Protein SCO2, mitochondrial [OS=Saccharomyces cerevisiae S288c]	852312	SCO2; YBR024W	cell organization and biogenesis;cellular homeostasis;metabolic process;transport	membrane;mitochondrion	antioxidant activity;catalytic activity;metal ion binding	0.551	0.73	1.32
P50110	Sorting assembly machinery 37 kDa subunit [OS=Saccharomyces cerevisiae S288c]	855082	SAM37; YMR060C	cell organization and biogenesis;transport	membrane;mitochondrion	protein binding	0.551	0.73	1.32
P09457	ATP synthase subunit 5, mitochondrial [OS=Saccharomyces cerevisiae S288c]	851892	ATP5; YDR298C	metabolic process;transport	membrane;mitochondrion	catalytic activity;transporter activity	7.799	10.365	1.33
Q02981	ABC1 family protein YPL109C, mitochondrial [OS=Saccharomyces cerevisiae S288c]	855995	YPL109C; YPL109C		mitochondrion		0.585	0.778	1.33
P40471	NADPH-dependent 1-acyldihydroxyacetone phosphate reductase [OS=Saccharomyces cerevisiae S288c]	854682	AYR1; YIL124W	metabolic process	cytoplasm;endoplasmic reticulum;membrane;mitochondrion	catalytic activity	0.585	0.778	1.33
P32335	Protein MSS51, mitochondrial [OS=Saccharomyces cerevisiae S288c]	850900	MSS51; YLR203C	metabolic process;regulation of biological process	membrane;mitochondrion	protein binding;translation regulator activity	0.995	1.326	1.33
P16603	NADPH--cytochrome P450 reductase [OS=Saccharomyces cerevisiae S288c]	856438	NCP1; YHR042W	metabolic process	cytoplasm;cytosol;endoplasmic reticulum;membrane;mitochondrion	catalytic activity;metal ion binding;nucleotide binding	0.604	0.805	1.33
Q03028	Mitochondrial 2-oxodicarboxylate carrier 1 [OS=Saccharomyces cerevisiae S288c]	855969	ODC1; YPL134C	metabolic process;transport	membrane;mitochondrion	structural molecule activity;transporter activity	5.813	7.799	1.34
P53206	putative cysteine synthase [OS=Saccharomyces cerevisiae S288c]	852895	YGR012W; YGR012W	metabolic process	mitochondrion	catalytic activity	1.154	1.555	1.35
P33311	ATP-dependent permease MDL2, mitochondrial [OS=Saccharomyces cerevisiae S288c]	855858	MDL2; YPL270W	response to stimulus;transport	membrane;mitochondrion	catalytic activity;nucleotide binding;transporter activity	0.901	1.233	1.37
P00175	Cytochrome b2, mitochondrial [OS=Saccharomyces cerevisiae	854950	CYB2; YML05	metabolic process;transport	cytosol;membrane;mitochondrion;nucleus	catalytic activity;metal ion	0.189	0.259	1.37

	S288c]		4C			binding;nucleotide binding			
P40582	Glutathione S-transferase 1 [OS=Saccharomyces cerevisiae S288c]	854856	GTT1; YIR038C	metabolic process	endoplasmic reticulum;membrane;mitochondrion;nucleus	antioxidant activity;catalytic activity;protein binding;RNA binding	0.931	1.276	1.37
P50087	MICOS subunit MIC26 [OS=Saccharomyces cerevisiae S288c]	853150	MOS2; MIC26; YGR235C	cell organization and biogenesis	membrane;mitochondrion		0.931	1.276	1.37
P33310	ATP-dependent permease MDL1, mitochondrial [OS=Saccharomyces cerevisiae S288c]	850885	MDL1; YLR188W	transport	membrane;mitochondrion	catalytic activity;nucleotide binding;transporter activity	0.528	0.725	1.37
P00360	glyceraldehyde-3-phosphate dehydrogenase 1 [OS=Saccharomyces cerevisiae S288c]	853395	TDH1; YJL052W	metabolic process	cytoplasm;cytosol;membrane;mitochondrion	catalytic activity;nucleotide binding	1.462	2.008	1.37
P25374	Cysteine desulfurase, mitochondrial [OS=Saccharomyces cerevisiae S288c]	850343	NFS1; YCL017C	cell organization and biogenesis;cellular homeostasis;metabolic process	mitochondrion;nucleus	catalytic activity;metal ion binding;protein binding	0.978	1.346	1.38
Q12374	Nuclear control of ATPase protein 2 [OS=Saccharomyces cerevisiae S288c]	856278	NCA2; YPR155C	metabolic process	membrane;mitochondrion		0.565	0.778	1.38
P53312	Succinyl-CoA ligase [ADP-forming] subunit beta, mitochondrial [OS=Saccharomyces cerevisiae S288c]	853159	LSC2; YGR244C	metabolic process	mitochondrion	catalytic activity;nucleotide binding	9	12.46	1.38
P53170	[Pyruvate dehydrogenase (acetyl-transferring)] kinase 2, mitochondrial [OS=Saccharomyces cerevisiae S288c]	852821	PKP2; YGL059W	metabolic process;regulation of biological process	mitochondrion;organelle lumen	catalytic activity;nucleotide binding;protein binding	0.28	0.389	1.39
P32898	Mitochondrial presequence protease [OS=Saccharomyces cerevisiae S288c]	852041	CYM1; YDR430C	metabolic process	mitochondrion	catalytic activity;metal ion binding	0.682	0.951	1.39
Q08822	Probable electron transfer flavoprotein-ubiquinone oxidoreductase, mitochondrial [OS=Saccharomyces cerevisiae S288c]	854538	CIR2; YOR356W	metabolic process;transport	membrane;mitochondrion	catalytic activity;metal ion binding;nucleotide binding	2.162	3.019	1.40
P32902	37S ribosomal protein MRP4, mitochondrial [OS=Saccharomyces cerevisiae S288c]	856384	MRP4; YHL004W	cell organization and biogenesis;metabolic process	mitochondrion;ribosome	structural molecule activity	0.334	0.468	1.40
P48015	Aminomethyltransferase, mitochondrial [OS=Saccharomyces cerevisiae S288c]	851582	GCV1; YDR019C	metabolic process	mitochondrion	catalytic activity;protein binding	6.406	9	1.40
P32454	Aminopeptidase 2, mitochondrial [OS=Saccharomyces cerevisiae S288c]	853699	APE2; YKL157W	metabolic process	cytoplasm;extracellular;membrane;mitochondrion;nucleus	catalytic activity;metal ion binding	1.668	2.346	1.41
Q06892	NADH kinase POS5, mitochondrial [OS=Saccharomyces cerevisiae S288c]	855913	POS5; YPL188W	metabolic process;response to stimulus	mitochondrion;organelle lumen	catalytic activity;nucleotide binding	0.369	0.52	1.41
Q02950	37S ribosomal protein MRP51, mitochondrial [OS=Saccharomyces cerevisiae S288c]	855985	MRP51; YPL118W	cell organization and biogenesis;metabolic process	mitochondrion;ribosome	structural molecule activity	0.817	1.154	1.41
P40035	Mitochondrial phosphate carrier protein 2 [OS=Saccharomyces cerevisiae S288c]	856779	PIC2; YER053C	cellular homeostasis;metabolic process;transport	membrane;mitochondrion	structural molecule activity;transporter activity	1.976	2.793	1.41
Q01852	Mitochondrial import inner membrane translocase subunit TIM44 [OS=Saccharomyces cerevisiae S288c]	854790	TIM44; YIL022W	transport	membrane;mitochondrion	nucleotide binding;protein binding	4.946	7.003	1.42
P36163	mitochondrial metalloendopeptidase OMA1 [OS=Saccharomyces cerevisiae S288c]	853962	OMA1; YKR087C	metabolic process	membrane;mitochondrion	catalytic activity;metal ion binding	0.389	0.551	1.42
P43617	Uncharacterized mitochondrial carrier YFR045W [OS=Saccharomyces cerevisiae S288c]	850606	YFR045W; YFR045W	metabolic process;transport	membrane;mitochondrion	structural molecule activity;transporter activity	0.413	0.585	1.42
P36534	54S ribosomal protein L40, mitochondrial [OS=Saccharomyces cerevisiae S288c]	855930	MRPL40; YPL173W	cell organization and biogenesis;metabolic process	mitochondrion;ribosome	protein binding;structural molecule activity	0.413	0.585	1.42
Q12166	2-isopropylmalate synthase 2, mitochondrial [OS=Saccharomyces cerevisiae S288c]	854275	LEU9; YOR108W	metabolic process	mitochondrion	catalytic activity;protein binding	2.065	2.932	1.42
Q03798	Altered inheritance of mitochondria protein 36, mitochondrial [OS=Saccharomyces cerevisiae S288c]	855189	AIM36; YMR157C		membrane;mitochondrion		0.438	0.624	1.42
P06168	ketol-acid reductoisomerase, mitochondrial [OS=Saccharomyces cerevisiae S288c]	851069	ILV5; YLR355C	cell organization and biogenesis;metabolic process	mitochondrion	catalytic activity;DNA binding;metal ion binding	10.788	15.379	1.43
P05626	ATP synthase subunit 4, mitochondrial [OS=Saccharomyces	856027	ATP4; YPL078	cell organization and biogenesis;metabolic	membrane;mitochondrion	catalytic activity;transporter	2.162	3.084	1.43

	cerevisiae S288c]		C	process;transport		activity			
P32795	Mitochondrial inner membrane i-AAA protease supercomplex subunit YME1 [OS=Saccharomyces cerevisiae S288c]	856135	YME1; YPR024W	metabolic process;response to stimulus;transport	membrane;mitochondrion	catalytic activity;metal ion binding;nucleotide binding;protein binding	0.986	1.415	1.44
P00359	Glyceraldehyde-3-phosphate dehydrogenase 3 [OS=Saccharomyces cerevisiae S288c]	853106	TDH3; YGR192C	cell death;metabolic process	cytoplasm;cytosol;membrane;mitochondrion;nucleus	catalytic activity;nucleotide binding;RNA binding	3.962	5.7	1.44
P08417	fumarate hydratase, mitochondrial [OS=Saccharomyces cerevisiae S288c]	855866	FUM1; YPL262W	cell organization and biogenesis;metabolic process	cytoplasm;cytosol;mitochondrion;organelle lumen	catalytic activity	5.21	7.532	1.45
P42940	Probable electron transfer flavoprotein subunit beta [OS=Saccharomyces cerevisiae S288c]	853121	CIR1; YGR207C	metabolic process;transport	mitochondrion;organelle lumen		2.652	3.87	1.46
P33416	Heat shock protein 78, mitochondrial [OS=Saccharomyces cerevisiae S288c]	851845	HSP78; YDR258C	cell organization and biogenesis;metabolic process;regulation of biological process;response to stimulus	mitochondrion;organelle lumen	catalytic activity;nucleotide binding;protein binding	3.042	4.466	1.47
P52893	probable alanine aminotransferase, mitochondrial [OS=Saccharomyces cerevisiae S288c]	850778	ALT1; YLR089C	metabolic process	mitochondrion;organelle lumen	catalytic activity	1.102	1.626	1.48
P36046	Mitochondrial intermembrane space import and assembly protein 40 [OS=Saccharomyces cerevisiae S288c]	853639	MIA40; YKL195W	cellular component movement;metabolic process;response to stimulus;transport	membrane;mitochondrion	catalytic activity;protein binding;transporter activity	1.555	2.3	1.48
P37292	Serine hydroxymethyltransferase, mitochondrial [OS=Saccharomyces cerevisiae S288c]	852565	SHM1; YBR263W	metabolic process	mitochondrion	catalytic activity	6.565	9.723	1.48
P53311	Mitochondrial pyruvate carrier 3 [OS=Saccharomyces cerevisiae S288c]	853158	FMP43; MPC3; YGR243W	transport	membrane;mitochondrion	transporter activity	0.778	1.154	1.48
P25039	Elongation factor G, mitochondrial [OS=Saccharomyces cerevisiae S288c]	850758	MEF1; YLR069C	cell organization and biogenesis;metabolic process	cytosol;mitochondrion	catalytic activity;nucleotide binding;RNA binding	0.456	0.677	1.48
P16387	Pyruvate dehydrogenase E1 component subunit alpha, mitochondrial [OS=Saccharomyces cerevisiae S288c]	856925	PDA1; YER178W	cell growth;metabolic process	mitochondrion;organelle lumen	catalytic activity	12.46	18.513	1.49
Q07651	SUR7 family protein FMP45 [OS=Saccharomyces cerevisiae S288c]	851304	FMP45; YDL222C	cell differentiation;cell organization and biogenesis	membrane;mitochondrion		6.017	9	1.50
P33303	Succinate/fumarate mitochondrial transporter [OS=Saccharomyces cerevisiae S288c]	853558	SFC1; YJR095W	metabolic process;transport	membrane;mitochondrion	structural molecule activity;transporter activity	11.915	17.957	1.51
Q04728	Arginine biosynthesis bifunctional protein ArgJ, mitochondrial [OS=Saccharomyces cerevisiae S288c]	855084	ARG7; YMR062C	metabolic process	mitochondrion;organelle lumen	catalytic activity	0.417	0.63	1.51
P06208-1	2-isopropylmalate synthase [OS=Saccharomyces cerevisiae S288c]	855619	LEU4; YNL104C	metabolic process	cytoplasm;mitochondrion	catalytic activity;protein binding	4.878	7.377	1.51
P38988	Mitochondrial GTP/GDP carrier protein 1 [OS=Saccharomyces cerevisiae S288c]	851329	GGC1; YDL198C	cell organization and biogenesis;cellular homeostasis;metabolic process;transport	membrane;mitochondrion	structural molecule activity;transporter activity	3.16	4.78	1.51
P10662	37S ribosomal protein MRP1, mitochondrial [OS=Saccharomyces cerevisiae S288c]	851948	MRP1; YDR347W	cell organization and biogenesis;metabolic process;response to stimulus	mitochondrion;ribosome	antioxidant activity;catalytic activity;metal ion binding;structural molecule activity	0.995	1.512	1.52
P00358	glyceraldehyde-3-phosphate dehydrogenase 2 [OS=Saccharomyces cerevisiae S288c]	853465	TDH2; YJR009C	cell death;metabolic process	cytoplasm;cytosol;membrane;mitochondrion;nucleus	catalytic activity;nucleotide binding;protein binding	2.008	3.062	1.52
Q01217	Protein ARG5,6, mitochondrial [OS=Saccharomyces cerevisiae S288c]	856800	ARG5,6; YER069W	metabolic process;regulation of biological process	cytoplasm;mitochondrion;organelle lumen	catalytic activity;nucleotide binding;protein binding	0.087	0.134	1.54
P36775	Lon protease homolog, mitochondrial [OS=Saccharomyces cerevisiae S288c]	852259	PIM1; YBL022C	cell organization and biogenesis;metabolic process;regulation of biological process;response to stimulus	cytoplasm;mitochondrion;organelle lumen	catalytic activity;DNA binding;nucleotide binding;RNA binding	0.792	1.222	1.54
P32445	Single-stranded DNA-binding protein RIM1, mitochondrial [OS=Saccharomyces cerevisiae S288c]	850395	RIM1; YCR028C-A	cell organization and biogenesis;metabolic process;regulation of biological process	mitochondrion	DNA binding	1.154	1.783	1.55
P37298	Succinate dehydrogenase [ubiquinone] cytochrome b small subunit, mitochondrial [OS=Saccharomyces cerevisiae S288c]	851758	SDH4; YDR178W	metabolic process;transport	membrane;mitochondrion	catalytic activity;metal ion binding	1.154	1.783	1.55
P39533	Homocitrate dehydratase,	85323	ACO2;	metabolic process	mitochondrion	catalytic	0.11	0.17	1.55

	mitochondrial [OS=Saccharomyces cerevisiae S288c]	0	YJL200C			activity;metal ion binding			
P39722	Mitochondrial Rho GTPase 1 [OS=Saccharomyces cerevisiae S288c]	851249	GEM1; YAL048C	cell organization and biogenesis;regulation of biological process;response to stimulus;transport	membrane;mitochondrion	catalytic activity;metal ion binding;nucleotide binding	0.122	0.189	1.55
P23180	Probable oxidoreductase AIM17 [OS=Saccharomyces cerevisiae S288c]	856365	AIM17; YHL021C	cell organization and biogenesis;metabolic process	mitochondrion	catalytic activity;metal ion binding	2.981	4.623	1.55
P36112	MICOS complex subunit Mic60 [OS=Saccharomyces cerevisiae S288c]	853886	FCJ1; MIC60; YKR016W	cell organization and biogenesis;transport	membrane;mitochondrion		11.798	18.307	1.55
P38756	tRNA threonylcarbamoyladenosine dehydratase 1 [OS=Saccharomyces cerevisiae S288c]	856392	TCD1; YHR003C; YHR003C	metabolic process	cytosol;membrane;mitochondrion	catalytic activity;nucleotide binding	0.194	0.304	1.57
Q05648	MIOREX complex component 10 [OS=Saccharomyces cerevisiae S288c]	851876	YDR282C; MRX10; YDR282C		membrane;mitochondrion		0.222	0.35	1.58
P02381	Ribosomal protein VAR1, mitochondrial [OS=Saccharomyces cerevisiae S288c]	854586	VAR1; Q0140	cell organization and biogenesis;metabolic process	mitochondrion;ribosome	structural molecule activity	0.233	0.369	1.58
P07256	Cytochrome b-c1 complex subunit 1, mitochondrial [OS=Saccharomyces cerevisiae S288c]	852235	COR1; YBL045C	metabolic process;transport	membrane;mitochondrion	catalytic activity;metal ion binding;protein binding;transporter activity	9.89	15.681	1.59
P40530	[Pyruvate dehydrogenase (acetyl-transferring)] kinase 1, mitochondrial [OS=Saccharomyces cerevisiae S288c]	854769	PKP1; YIL042C	metabolic process	mitochondrion;organelle lumen	catalytic activity;nucleotide binding;protein binding	0.245	0.389	1.59
P38127	mitochondrial carrier protein RIM2 [OS=Saccharomyces cerevisiae S288c]	852491	RIM2; YBR192W	cell organization and biogenesis;metabolic process;transport	membrane;mitochondrion	structural molecule activity;transporter activity	0.778	1.239	1.59
Q12466	tricalbin-1 [OS=Saccharomyces cerevisiae S288c]	854253	TCB1; YOR086C	cell organization and biogenesis;regulation of biological process	endoplasmic reticulum;membrane;mitochondrion	metal ion binding;protein binding	0.468	0.748	1.60
Q08926	ULP1-interacting protein 4 [OS=Saccharomyces cerevisiae S288c]	855916	UIP4; YPL186C		endoplasmic reticulum;membrane;mitochondrion;nucleus		0.292	0.468	1.60
Q12283	Malonyl CoA-acyl carrier protein transacylase, mitochondrial [OS=Saccharomyces cerevisiae S288c]	854396	MCT1; YOR221C	metabolic process	mitochondrion	catalytic activity	0.292	0.468	1.60
Q99297	Mitochondrial 2-oxodicarboxylate carrier 2 [OS=Saccharomyces cerevisiae S288c]	854397	ODC2; YOR222W	metabolic process;transport	membrane;mitochondrion	structural molecule activity;transporter activity	0.334	0.54	1.62
Q12106	MDM10-complementing protein 1 [OS=Saccharomyces cerevisiae S288c]	854403	YOR228C; MCP1; YOR228C	cell organization and biogenesis	membrane;mitochondrion	catalytic activity	0.359	0.585	1.63
P07342	Acetolactate synthase catalytic subunit, mitochondrial [OS=Saccharomyces cerevisiae S288c]	855135	ILV2; YMR108W	metabolic process	mitochondrion	catalytic activity;metal ion binding;nucleotide binding	4.223	6.897	1.63
P53982	isocitrate dehydrogenase [NADP] [OS=Saccharomyces cerevisiae S288c]	855723	IDP3; YNL009W	metabolic process	cytoplasm;mitochondrion	catalytic activity;metal ion binding;nucleotide binding	2.162	3.532	1.63
P16451	Pyruvate dehydrogenase complex protein X component, mitochondrial [OS=Saccharomyces cerevisiae S288c]	853107	PDX1; YGR193C	metabolic process	mitochondrion;organelle lumen	catalytic activity;structural molecule activity	1.783	2.914	1.63
Q03246	37S ribosomal protein S17, mitochondrial [OS=Saccharomyces cerevisiae S288c]	855226	MRPS17; YMR188C	cell differentiation;cell organization and biogenesis;metabolic process	mitochondrion;ribosome	RNA binding;structural molecule activity	0.389	0.638	1.64
Q05931	Heat shock protein SSQ1, mitochondrial [OS=Saccharomyces cerevisiae S288c]	851084	SSQ1; YLR369W	cell organization and biogenesis;metabolic process	mitochondrion;organelle lumen	nucleotide binding;protein binding	0.413	0.679	1.64
P39987	Heat shock protein SSC3, mitochondrial [OS=Saccharomyces cerevisiae S288c]	856682	ECM10; YEL030W	cell organization and biogenesis;metabolic process;transport	mitochondrion	nucleotide binding;protein binding	1.198	1.976	1.65
Q03104	Meiotic sister chromatid recombination protein 1 [OS=Saccharomyces cerevisiae S288c]	854911	MSC1; YML128C	metabolic process	endoplasmic reticulum;membrane;mitochondrion		2.415	4.012	1.66
P39525	3-oxoacyl-[acyl-carrier-protein] synthase homolog [OS=Saccharomyces cerevisiae S288c]	856790	CEM1; YER061C	metabolic process	mitochondrion	catalytic activity	0.52	0.874	1.68

P32843	Mitochondrial escape protein 2 [OS=Saccharomyces cerevisiae S288c]	855348	YME2; YMR302C	cell organization and biogenesis;metabolic process	membrane;mitochondrion	nucleotide binding;RNA binding	2.643	4.456	1.69
P00890	citrate synthase, mitochondrial [OS=Saccharomyces cerevisiae S288c]	855732	CIT1; YNR001C	metabolic process;transport	cytosol;mitochondrion;organelle lumen	catalytic activity	12.895	21.758	1.69
P53736	Uncharacterized protein YNR040W [OS=Saccharomyces cerevisiae S288c]	855776	YNR040W; YNR040W		mitochondrion		0.551	0.931	1.69
P18239	ADP,ATP carrier protein 2 [OS=Saccharomyces cerevisiae S288c]	852250	PET9; YBL030C	cell death;metabolic process;transport	membrane;mitochondrion	structural molecule activity;transporter activity	167.761	283.804	1.69
P00927	Threonine dehydratase, mitochondrial [OS=Saccharomyces cerevisiae S288c]	856819	ILV1; YER086W	metabolic process	cytoplasm;mitochondrion	catalytic activity	0.914	1.572	1.72
Q08179	Mitochondrial distribution and morphology protein 38 [OS=Saccharomyces cerevisiae S288c]	854130	MDM38; YOL027C	cell organization and biogenesis;cellular homeostasis;regulation of biological process;transport	membrane;mitochondrion		4.309	7.414	1.72
P50085	Prohibitin-2 [OS=Saccharomyces cerevisiae S288c]	853146	PHB2; YGR231C	cell organization and biogenesis;metabolic process;regulation of biological process	membrane;mitochondrion	protein binding	2.511	4.337	1.73
P53140	Protein RMD9, mitochondrial [OS=Saccharomyces cerevisiae S288c]	852771	RMD9; YGL107C	cell differentiation;metabolic process;regulation of biological process	membrane;mitochondrion	RNA binding	0.668	1.154	1.73
Q02784	Monothiol glutaredoxin-5, mitochondrial [OS=Saccharomyces cerevisiae S288c]	856048	GRX5; YPL059W	cell organization and biogenesis;cellular homeostasis;metabolic process;regulation of biological process;response to stimulus	mitochondrion;organelle lumen	catalytic activity;metal ion binding	0.668	1.154	1.73
Q6Q560	Protein isd11 [OS=Saccharomyces cerevisiae S288c]	856774	ISD11; YER048W-A	cell organization and biogenesis;metabolic process	mitochondrion;organelle lumen	catalytic activity;protein binding	0.668	1.154	1.73
P53598	Succinyl-CoA ligase [ADP-forming] subunit alpha, mitochondrial [OS=Saccharomyces cerevisiae S288c]	854310	LSC1; YOR142W	metabolic process	cytosol;mitochondrion	catalytic activity;nucleotide binding	5.952	10.288	1.73
P11325	Leucine--tRNA ligase, mitochondrial [OS=Saccharomyces cerevisiae S288c]	851098	NAM2; YLR382C	cell organization and biogenesis;metabolic process;regulation of biological process	cytosol;mitochondrion;organelle lumen	catalytic activity;nucleotide binding;RNA binding	0.12	0.208	1.73
P53326	Uncharacterized protein YGR266W [OS=Saccharomyces cerevisiae S288c]	853182	YGR266W; YGR266W		membrane;mitochondrion		0.299	0.52	1.74
P53220	Mitochondrial import inner membrane translocase subunit TIM21 [OS=Saccharomyces cerevisiae S288c]	852921	TIM21; YGR033C	cell organization and biogenesis;transport	membrane;mitochondrion	protein binding	0.719	1.254	1.74
P34227	Mitochondrial peroxiredoxin PRX1 [OS=Saccharomyces cerevisiae S288c]	852215	PRX1; YBL064C	cellular homeostasis;metabolic process;regulation of biological process;response to stimulus	mitochondrion	antioxidant activity;catalytic activity	5.158	9	1.74
P00410	Cytochrome c oxidase subunit 2 [OS=Saccharomyces cerevisiae S288c]	854622	COX2; Q0250	metabolic process;transport	membrane;mitochondrion	catalytic activity;metal ion binding;protein binding;transporter activity	1.61	2.831	1.76
Q02204	54S ribosomal protein L13, mitochondrial [OS=Saccharomyces cerevisiae S288c]	853875	MRPL13; YKR006C	cell organization and biogenesis;metabolic process	mitochondrion;ribosome	structural molecule activity	0.778	1.371	1.76
P36147	Presequence translocated-associated motor subunit pam17, mitochondrial [OS=Saccharomyces cerevisiae S288c]	853939	PAM17; YKR065C	transport	membrane;mitochondrion		0.778	1.371	1.76
P32473	Pyruvate dehydrogenase E1 component subunit beta, mitochondrial [OS=Saccharomyces cerevisiae S288c]	852522	PDB1; YBR221C	metabolic process	mitochondrion;organelle lumen	catalytic activity	4.456	7.859	1.76
P38077	ATP synthase subunit gamma, mitochondrial [OS=Saccharomyces cerevisiae S288c]	852327	ATP3; YBR039W	metabolic process;transport	membrane;mitochondrion	catalytic activity;transporter activity	9	15.876	1.76
Q12298	uncharacterized ABC transporter ATP-binding protein YDR061W [OS=Saccharomyces cerevisiae S288c]	851633	YDR061W; YDR061W		mitochondrion	catalytic activity;nucleotide binding	0.194	0.343	1.77
P12695	Dihydrolipoyllysine-residue acetyltransferase component of pyruvate dehydrogenase complex, mitochondrial [OS=Saccharomyces cerevisiae S288c]	855653	LAT1; YNL071W	metabolic process	mitochondrion;organelle lumen	catalytic activity	7.62	13.497	1.77
P30902	ATP synthase subunit d, mitochondrial [OS=Saccharomyces cerevisiae S288c]	853853	ATP7; YKL016C	metabolic process;transport	membrane;mitochondrion	catalytic activity;transporter activity	4.179	7.483	1.79
Q96VH5	MICOS complex subunit Mic10 [OS=Saccharomyces cerevisiae S288c]	850300	MOS1; MIC10; YCL057	cell organization and biogenesis	membrane;mitochondrion		0.931	1.683	1.81

			C-A						
Q06493	LETM1 domain-containing protein YLH47, mitochondrial [OS=Saccharomyces cerevisiae S288c]	856243	YLH47; YPR125W	cell organization and biogenesis;transport	membrane;mitochondrion		0.487	0.887	1.82
P47127	Altered inheritance of mitochondria protein 24, mitochondrial [OS=Saccharomyces cerevisiae S288c]	853543	AIM24; YJR080C	cell organization and biogenesis	mitochondrion		2.857	5.21	1.82
Q04458	Fatty aldehyde dehydrogenase HFD1 [OS=Saccharomyces cerevisiae S288c]	855137	HFD1; YMR110C	metabolic process	endoplasmic reticulum;endosome;membrane;mitochondrion	catalytic activity	1.783	3.262	1.83
P08067	cytochrome b-c1 complex subunit Rieske, mitochondrial [OS=Saccharomyces cerevisiae S288c]	856689	RIP1; YEL024W	metabolic process;transport	membrane;mitochondrion	catalytic activity;metal ion binding;transporter activity	11.115	20.544	1.85
P40185	Protein mmf1, mitochondrial [OS=Saccharomyces cerevisiae S288c]	854760	MMF1; YIL051C	cell organization and biogenesis;metabolic process	mitochondrion;organelle lumen		2.162	4.012	1.86
P40053	Altered inheritance of mitochondria protein 9, mitochondrial [OS=Saccharomyces cerevisiae S288c]	856813	AIM9; YER080W		mitochondrion		1.798	3.348	1.86
P38797	Protein phosphatase 2C homolog 7, mitochondrial [OS=Saccharomyces cerevisiae S288c]	856475	PTC7; YHR076W	metabolic process	mitochondrion	catalytic activity;metal ion binding;protein binding	0.369	0.688	1.86
Q08818	Meiotic sister-chromatid recombination protein 6, mitochondrial [OS=Saccharomyces cerevisiae S288c]	854536	MSC6; YOR354C	metabolic process	mitochondrion;organelle lumen	RNA binding	0.506	0.945	1.87
P32796-1	Carnitine O-acetyltransferase, mitochondrial [OS=Saccharomyces cerevisiae S288c]	854965	CAT2; YML042W	metabolic process;transport	membrane;mitochondrion	catalytic activity	5.749	10.835	1.88
P07257	Cytochrome b-c1 complex subunit 2, mitochondrial [OS=Saccharomyces cerevisiae S288c]	856321	QCR2; YPR191W	metabolic process;transport	membrane;mitochondrion	catalytic activity;metal ion binding;protein binding;transporter activity	25.49	48.239	1.89
P07275	Delta-1-pyrroline-5-carboxylate dehydrogenase, mitochondrial [OS=Saccharomyces cerevisiae S288c]	856432	PUT2; YHR037W	metabolic process	membrane;mitochondrion;organelle lumen	catalytic activity	5.136	9.723	1.89
P36151	Uncharacterized protein YKR070W [OS=Saccharomyces cerevisiae S288c]	853944	YKR070W; YKR070W	metabolic process	mitochondrion		0.438	0.833	1.90
P23833	Protein SCO1, mitochondrial [OS=Saccharomyces cerevisiae S288c]	852325	SCO1; YBR037C	cell organization and biogenesis;cellular homeostasis;response to stimulus;transport	membrane;mitochondrion	antioxidant activity;catalytic activity;metal ion binding	0.468	0.896	1.91
Q07500	External NADH-ubiquinone oxidoreductase 2, mitochondrial [OS=Saccharomyces cerevisiae S288c]	851474	NDE2; YDL085W	metabolic process	mitochondrion	catalytic activity;nucleotide binding	3.299	6.356	1.93
P38172	MIOREX complex component 3 [OS=Saccharomyces cerevisiae S288c]	852181	YBL095W; MRX3; YBL095W		membrane;mitochondrion		0.501	0.968	1.93
P32860	NifU-like protein, mitochondrial [OS=Saccharomyces cerevisiae S288c]	853826	NFU1; YKL040C	cell organization and biogenesis;cellular homeostasis;metabolic process	mitochondrion;organelle lumen	metal ion binding	1.371	2.652	1.93
Q00711	Succinate dehydrogenase [ubiquinone] flavoprotein subunit, mitochondrial [OS=Saccharomyces cerevisiae S288c]	853709	SDH1; YKL148C	metabolic process;transport	membrane;mitochondrion	catalytic activity;nucleotide binding;protein binding	9.578	18.619	1.94
P41921	glutathione reductase [OS=Saccharomyces cerevisiae S288c]	856014	GLR1; YPL091W	cellular homeostasis;metabolic process;regulation of biological process;response to stimulus	cytoplasm;cytosol;mitochondrion;nucleus	antioxidant activity;catalytic activity;nucleotide binding	0.334	0.655	1.96
P23641	mitochondrial phosphate carrier protein [OS=Saccharomyces cerevisiae S288c]	853540	MIR1; YJR077C	metabolic process;transport	membrane;mitochondrion	protein binding;structural molecule activity;transporter activity	13.678	26.826	1.96
P36013	NAD-dependent malic enzyme, mitochondrial [OS=Saccharomyces cerevisiae S288c]	853839	MAE1; YKL029C	metabolic process	mitochondrion;organelle lumen	catalytic activity;metal ion binding;nucleotide binding	1.512	2.981	1.97
P32317	Protein AFG1 [OS=Saccharomyces cerevisiae S288c]	856658	AFG1; YEL052W	cell organization and biogenesis;metabolic process;response to stimulus;transport	membrane;mitochondrion	catalytic activity;nucleotide binding	0.585	1.154	1.97
P36525	54S ribosomal protein L24, mitochondrial [OS=Saccharomyces cerevisiae S288c]	855231	MRPL24; YMR193W	cell organization and biogenesis;metabolic process	mitochondrion;ribosome	RNA binding;structural molecule activity	0.585	1.154	1.97
P47052	succinate dehydrogenase	85340	YJL045	metabolic process;transport	membrane;mitochondrion	catalytic	1.88	3.739	1.99

	[ubiquinone] flavoprotein subunit 2, mitochondrial [OS=Saccharomyces cerevisiae S288c]	5	W; YJL045 W			activity;nucleotide binding			
P23644	Mitochondrial import receptor subunit TOM40 [OS=Saccharomyces cerevisiae S288c]	855243	TOM40; YMR203W	transport	cytosol;membrane;mitochondrion	protein binding;transporter activity	0.896	1.783	1.99
P54783	D-arabinono-1,4-lactone oxidase [OS=Saccharomyces cerevisiae S288c]	854888	ALO1; YML086C	metabolic process;response to stimulus	membrane;mitochondrion	catalytic activity;nucleotide binding	2.415	4.843	2.01
P35191	DnaJ homolog 1, mitochondrial [OS=Saccharomyces cerevisiae S288c]	850530	MDJ1; YFL016C	cell organization and biogenesis;metabolic process;regulation of biological process;response to stimulus	mitochondrion;organelle lumen	enzyme regulator activity;metal ion binding;nucleotide binding;protein binding	1.656	3.329	2.01
P38891	Branched-chain-amino-acid aminotransferase, mitochondrial [OS=Saccharomyces cerevisiae S288c]	856615	BAT1; YHR208W	metabolic process;regulation of biological process;response to stimulus	mitochondrion;organelle lumen	catalytic activity	6.305	12.689	2.01
P38825	Protein TOM71 [OS=Saccharomyces cerevisiae S288c]	856517	TOM71; YHR117W	transport	membrane;mitochondrion	protein binding;transporter activity	0.512	1.031	2.01
Q04935	Cytochrome c oxidase protein 20, mitochondrial [OS=Saccharomyces cerevisiae S288c]	851817	COX20; YDR231C	cell organization and biogenesis;metabolic process	membrane;mitochondrion	protein binding	0.701	1.424	2.03
P38071	Enoyl-[acyl-carrier protein] reductase [NADPH, B-specific], mitochondrial [OS=Saccharomyces cerevisiae S288c]	852314	ETR1; YBR026C	metabolic process	mitochondrion;organelle lumen	catalytic activity;DNA binding;metal ion binding	4.926	10.103	2.05
P40008	Protein FMP52, mitochondrial [OS=Saccharomyces cerevisiae S288c]	856721	FMP52; YER004W		endoplasmic reticulum;membrane;mitochondrion	catalytic activity	1.054	2.162	2.05
Q12171	Mitochondrial distribution and morphology protein 32 [OS=Saccharomyces cerevisiae S288c]	854318	MDM32; YOR147W	cell organization and biogenesis;cellular homeostasis	membrane;mitochondrion		0.066	0.136	2.06
P32606	putative mitochondrial translation system component PET127 [OS=Saccharomyces cerevisiae S288c]	854182	PET127; YOR017W	cell organization and biogenesis;metabolic process;regulation of biological process	mitochondrion		0.048	0.099	2.06
P39925	Mitochondrial respiratory chain complexes assembly protein AFG3 [OS=Saccharomyces cerevisiae S288c]	856737	AFG3; YER017C	cell organization and biogenesis;metabolic process;response to stimulus;transport	membrane;mitochondrion	catalytic activity;metal ion binding;nucleotide binding;protein binding	0.65	1.342	2.06
P20967	2-oxoglutarate dehydrogenase, mitochondrial [OS=Saccharomyces cerevisiae S288c]	854681	KGD1; YIL125W	metabolic process	cytosol;mitochondrion;organelle lumen	catalytic activity	15.843	32.752	2.07
P00427	Cytochrome c oxidase subunit 6, mitochondrial [OS=Saccharomyces cerevisiae S288c]	856448	COX6; YHR051W	metabolic process;transport	membrane;mitochondrion	catalytic activity;metal ion binding;transporter activity	0.778	1.61	2.07
P03874	Cytochrome B pre-mRNA-processing protein 2 [OS=Saccharomyces cerevisiae S288c]	856347	CBP2; YHL038C	metabolic process	mitochondrion	RNA binding	0.068	0.141	2.07
P40051	Intermediate cleaving peptidase 55 [OS=Saccharomyces cerevisiae S288c]	856811	ICP55; YER078C	metabolic process;regulation of biological process	membrane;mitochondrion	catalytic activity;metal ion binding	0.077	0.16	2.08
P09950	5-aminolevulinate synthase, mitochondrial [OS=Saccharomyces cerevisiae S288c]	851818	HEM1; YDR232W	metabolic process	mitochondrion;organelle lumen	catalytic activity	0.086	0.179	2.08
Q02863	ubiquitin carboxyl-terminal hydrolase 16 [OS=Saccharomyces cerevisiae S288c]	856032	UBP16; YPL072W	metabolic process	membrane;mitochondrion	catalytic activity	0.072	0.15	2.08
P08466	mitochondrial nuclease [OS=Saccharomyces cerevisiae S288c]	853222	NUC1; YJL208C	cell death;metabolic process	membrane;mitochondrion;nucleus	catalytic activity;metal ion binding	1.336	2.793	2.09
P49367	Homoaconitase, mitochondrial [OS=Saccharomyces cerevisiae S288c]	851820	LYS4; YDR234W	metabolic process	mitochondrion	catalytic activity;metal ion binding;protein binding	0.551	1.154	2.09
P19882	heat shock protein 60, mitochondrial [OS=Saccharomyces cerevisiae S288c]	850963	HSP60; YLR259C	cell organization and biogenesis;metabolic process;regulation of biological process;transport	cytoplasm;cytosol;membrane;mitochondrion;organelle lumen	catalytic activity;DNA binding;nucleotide binding;protein binding	39.296	82.378	2.10
P38122	3-methyl-2-oxobutanoate hydroxymethyltransferase [OS=Saccharomyces cerevisiae S288c]	852474	ECM31; YBR176W	metabolic process	mitochondrion	catalytic activity	0.122	0.259	2.12
P07143	Cytochrome c1, heme protein, mitochondrial [OS=Saccharomyces cerevisiae S288c]	854231	CYT1; YOR065W	metabolic process;transport	cytosol;membrane;mitochondrion	metal ion binding	10.938	23.245	2.13
P28625	Protein YIM1 [OS=Saccharomyces cerevisiae S288c]	855183	YIM1; YMR152W	metabolic process;response to stimulus	cytoplasm;endoplasmic reticulum;mitochondrion	catalytic activity;metal ion binding	0.624	1.336	2.14
P38884	Altered inheritance of mitochondria protein 18, mitochondrial [OS=Saccharomyces cerevisiae	856605	AIM18; YHR198C		mitochondrion	catalytic activity	0.145	0.311	2.14

	S288c]								
Q04472	Mitochondrial inner membrane i-AAA protease supercomplex subunit MGR3 [OS=Saccharomyces cerevisiae S288c]	855142	MGR3; YMR115W	metabolic process	membrane;mitochondrion	protein binding	0.15	0.322	2.15
P09624	Dihydrolipoyl dehydrogenase, mitochondrial [OS=Saccharomyces cerevisiae S288c]	850527	LPD1; YFL018C	cellular homeostasis;metabolic process;regulation of biological process	mitochondrion;organelle lumen	catalytic activity;nucleotide binding	23.119	49.802	2.15
P42847	37S ribosomal protein S18, mitochondrial [OS=Saccharomyces cerevisiae S288c]	855410	MRPS18; YNL306W	cell organization and biogenesis;metabolic process	mitochondrion;ribosome	RNA binding;structural molecule activity	0.155	0.334	2.15
P14693	sorting assembly machinery 35 kDa subunit [OS=Saccharomyces cerevisiae S288c]	856483	SAM35; YHR083W	cell organization and biogenesis;transport	membrane;mitochondrion	protein binding	0.155	0.334	2.15
P32799	Cytochrome c oxidase subunit 6A, mitochondrial [OS=Saccharomyces cerevisiae S288c]	852684	COX13; YGL191W	cell organization and biogenesis;metabolic process;regulation of biological process;transport	membrane;mitochondrion	catalytic activity;enzyme regulator activity;transporter activity	0.995	2.162	2.17
P40961	prohibitin-1 [OS=Saccharomyces cerevisiae S288c]	853033	PHB1; YGR132C	cell organization and biogenesis;metabolic process;regulation of biological process	membrane;mitochondrion	protein binding	2.36	5.158	2.19
P34231	Uncharacterized protein YKL187C [OS=Saccharomyces cerevisiae S288c]	853648	FAT3; YKL187C; YKL187C	cell organization and biogenesis;transport	membrane;mitochondrion		0.194	0.425	2.19
Q01976	ADP-ribose pyrophosphatase [OS=Saccharomyces cerevisiae S288c]	852408	YSA1; YBR111C	metabolic process	cytoplasm;mitochondrion;nucleus	catalytic activity;metal ion binding	0.194	0.425	2.19
Q03201	37S ribosomal protein S10, mitochondrial [OS=Saccharomyces cerevisiae S288c]	851611	RSM10; YDR041W	cell organization and biogenesis;metabolic process	mitochondrion;ribosome	structural molecule activity	0.194	0.425	2.19
P34222	Peptidyl-tRNA hydrolase 2 [OS=Saccharomyces cerevisiae S288c]	852223	PTH2; YBL057C	regulation of biological process	cytoplasm;cytosol;membrane;mitochondrion	catalytic activity;protein binding	0.194	0.425	2.19
P07251	ATP synthase subunit alpha, mitochondrial [OS=Saccharomyces cerevisiae S288c]	852177	ATP1; YBL099W	metabolic process;transport	cytosol;membrane;mitochondrion	catalytic activity;nucleotide binding;transporter activity	26.826	58.948	2.20
P00830	ATP synthase subunit beta, mitochondrial [OS=Saccharomyces cerevisiae S288c]	853585	ATP2; YJR121W	metabolic process;transport	cytosol;membrane;mitochondrion	catalytic activity;nucleotide binding;protein binding;transporter activity	24.119	53.117	2.20
P40165	NAD(P)H-hydrate epimerase [OS=Saccharomyces cerevisiae S288c]	855521	YNL200C; YNL200C	metabolic process	cytoplasm;mitochondrion	catalytic activity;metal ion binding;nucleotide binding	0.212	0.468	2.21
P34224	Uncharacterized protein YBL059W [OS=Saccharomyces cerevisiae S288c]	852221	YBL059W; YBL059W		membrane;mitochondrion		0.212	0.468	2.21
P47015	Altered inheritance of mitochondria protein 23, mitochondrial [OS=Saccharomyces cerevisiae S288c]	853310	AIM23; YJL131C	metabolic process	mitochondrion	RNA binding	0.222	0.492	2.22
P32792	UPF0744 protein YSC83 [OS=Saccharomyces cerevisiae S288c]	856410	YSC83; YHR017W		membrane;mitochondrion		0.233	0.52	2.23
P54857	lipase 2 [OS=Saccharomyces cerevisiae S288c]	851628	TGL2; YDR058C	metabolic process;transport	mitochondrion	catalytic activity	0.233	0.52	2.23
P14832	peptidyl-prolyl cis-trans isomerase [OS=Saccharomyces cerevisiae S288c]	851733	CPR1; YDR155C	cell differentiation;cell organization and biogenesis;metabolic process;regulation of biological process;transport	cytoplasm;mitochondrion;nucleus	catalytic activity;RNA binding	0.233	0.52	2.23
P19955	37S ribosomal protein YMR-31, mitochondrial [OS=Saccharomyces cerevisiae S288c]	850610	YMR31; YFR049W	cell organization and biogenesis;metabolic process	mitochondrion;ribosome	catalytic activity;protein binding;structural molecule activity	1.154	2.594	2.25
P40502	Altered inheritance of mitochondria protein 19, mitochondrial [OS=Saccharomyces cerevisiae S288c]	854722	AIM19; YIL087C		membrane;mitochondrion		0.259	0.585	2.26
P40341	Mitochondrial respiratory chain complexes assembly protein YTA12 [OS=Saccharomyces cerevisiae S288c]	855114	YTA12; YMR089C	cell organization and biogenesis;metabolic process;response to stimulus;transport	cytoplasm;membrane;mitochondrion	catalytic activity;metal ion binding;nucleotide binding;protein binding	1.276	2.884	2.26
P40513	Mitochondrial acidic protein MAM33 [OS=Saccharomyces cerevisiae S288c]	854740	MAM33; YIL070C	metabolic process;regulation of biological process	mitochondrion;organelle lumen	translation regulator activity	1.61	3.642	2.26
P39522	Dihydroxy-acid dehydratase,	85347	ILV3;	metabolic process	mitochondrion	catalytic	9.723	22.10	2.27

	mitochondrial [OS=Saccharomyces cerevisiae S288c]	3	YJR016C			activity;metal ion binding		1	
P48569	Uncharacterized protein YDL183C [OS=Saccharomyces cerevisiae S288c]	851345	YDL183C; YDL183C	transport	mitochondrion		0.274	0.624	2.28
Q12480	Probable electron transfer flavoprotein subunit alpha, mitochondrial [OS=Saccharomyces cerevisiae S288c]	856112	AIM45; YPR004C	metabolic process;transport	mitochondrion;organelle lumen	catalytic activity;nucleotide binding	1.512	3.467	2.29
P0CS90	Heat shock protein SSC1, mitochondrial [OS=Saccharomyces cerevisiae S288c]	853503	SSC1; YJR045C	cell organization and biogenesis;metabolic process;regulation of biological process;transport	membrane;mitochondrion;nucleus;organelle lumen	catalytic activity;enzyme regulator activity;nucleotide binding;protein binding	37.312	86.992	2.33
P32787	Mitochondrial genome maintenance protein MGM101 [OS=Saccharomyces cerevisiae S288c]	853609	MGM101; YJR144W	cell organization and biogenesis;metabolic process;response to stimulus	chromosome;mitochondrion	DNA binding	1.637	3.833	2.34
P16622	Ferrochelatase, mitochondrial [OS=Saccharomyces cerevisiae S288c]	854347	HEM15; YOR176W	metabolic process	membrane;mitochondrion	catalytic activity	3.806	9	2.36
P19414	Aconitate hydratase, mitochondrial [OS=Saccharomyces cerevisiae S288c]	851013	ACO1; YLR304C	cell organization and biogenesis;metabolic process;transport	cytoplasm;cytosol;mitochondrion;organelle lumen	catalytic activity;DNA binding;metal ion binding	46.197	109.53	2.37
P12686	37S ribosomal protein MRP13, mitochondrial [OS=Saccharomyces cerevisiae S288c]	852975	MRP13; YGR084C	cell organization and biogenesis;metabolic process	mitochondrion;ribosome	structural molecule activity	0.413	0.995	2.41
P04840	Mitochondrial outer membrane protein porin 1 [OS=Saccharomyces cerevisiae S288c]	855669	POR1; YNL055C	cell death;cell organization and biogenesis;cellular homeostasis;regulation of biological process;transport	cytoplasm;membrane;mitochondrion	transporter activity	13.384	32.598	2.44
P17505	Malate dehydrogenase, mitochondrial [OS=Saccharomyces cerevisiae S288c]	853777	MDH1; YKL085W	metabolic process	mitochondrion;organelle lumen	catalytic activity;protein binding;RNA binding	32.405	79.309	2.45
P32897	Mitochondrial import inner membrane translocase subunit tim23 [OS=Saccharomyces cerevisiae S288c]	855751	TIM23; YNR017W	transport	membrane;mitochondrion	protein binding;transporter activity	2.162	5.31	2.46
P32266	Dynamin-like GTPase MGM1, mitochondrial [OS=Saccharomyces cerevisiae S288c]	854386	MGM1; YOR211C	cell organization and biogenesis	membrane;mitochondrion	catalytic activity;nucleotide binding;protein binding	0.228	0.572	2.51
P00424	Cytochrome c oxidase polypeptide 5A, mitochondrial [OS=Saccharomyces cerevisiae S288c]	855675	COX5A; YNL052W	metabolic process;transport	membrane;mitochondrion	catalytic activity;transporter activity	0.52	1.31	2.52
P53163	54S ribosomal protein L12, mitochondrial [OS=Saccharomyces cerevisiae S288c]	852811	MNP1; YGL068W	cell organization and biogenesis;metabolic process	mitochondrion;ribosome	RNA binding;structural molecule activity	0.52	1.31	2.52
P28321	monoglyceride lipase [OS=Saccharomyces cerevisiae S288c]	853768	YJU3; YKL094W	metabolic process;regulation of biological process	cytoplasm;endoplasmic reticulum;membrane;mitochondrion	catalytic activity	0.551	1.404	2.55
P07213	Mitochondrial import receptor subunit TOM70 [OS=Saccharomyces cerevisiae S288c]	855602	TOM70; YNL121C	cell organization and biogenesis;transport	membrane;mitochondrion	protein binding;transporter activity	2.665	6.897	2.59
Q00402	Nuclear migration protein NUM1 [OS=Saccharomyces cerevisiae S288c]	851727	NUM1; YDR150W	cell organization and biogenesis;cellular component movement;metabolic process;regulation of biological process;transport	endoplasmic reticulum;mitochondrion	catalytic activity;motor activity;protein binding	0.025	0.065	2.60
P40215	External NADH-ubiquinone oxidoreductase 1, mitochondrial [OS=Saccharomyces cerevisiae S288c]	855176	NDE1; YMR145C	metabolic process	mitochondrion	catalytic activity;nucleotide binding	3.453	9	2.61
P38120	37S ribosomal protein S9, mitochondrial [OS=Saccharomyces cerevisiae S288c]	852443	MRPS9; YBR146W	cell organization and biogenesis;metabolic process	mitochondrion;ribosome	RNA binding;structural molecule activity	0.719	1.955	2.72
P07246	Alcohol dehydrogenase 3, mitochondrial [OS=Saccharomyces cerevisiae S288c]	855107	ADH3; YMR083W	metabolic process	mitochondrion;organelle lumen	catalytic activity;metal ion binding	4.179	11.452	2.74
P36060	NADH-cytochrome b5 reductase 2 [OS=Saccharomyces cerevisiae S288c]	853707	MCR1; YKL150W	metabolic process;response to stimulus	membrane;mitochondrion	catalytic activity;protein binding	11.743	32.598	2.78
Q06485	Autophagy-related protein 33 [OS=Saccharomyces cerevisiae S288c]	851070	ATG33; YLR356W	cell communication;metabolic process;response to stimulus	membrane;mitochondrion		0.778	2.162	2.78
Q02776	Mitochondrial import inner membrane translocase subunit TIM50 [OS=Saccharomyces cerevisiae S288c]	856042	TIM50; YPL063W	cell organization and biogenesis;metabolic process;transport	membrane;mitochondrion	catalytic activity;protein binding;transporter activity	4.623	12.895	2.79
P39726	Glycine cleavage system H protein, mitochondrial [OS=Saccharomyces cerevisiae S288c]	851254	GCV3; YAL044C	metabolic process	mitochondrion	catalytic activity	1.783	4.995	2.80

P25719	Peptidyl-prolyl cis-trans isomerase C, mitochondrial [OS=Saccharomyces cerevisiae S288c]	854897	CPR3; YML078W	cell death;metabolic process	mitochondrion;organelle lumen	catalytic activity	0.848	2.415	2.85
P25573	Mitochondrial inner membrane i-AAA protease supercomplex subunit MGR1 [OS=Saccharomyces cerevisiae S288c]	850313	MGR1; YCL044C	metabolic process	membrane;mitochondrion	protein binding	0.425	1.219	2.87
Q02883	N-acyl-phosphatidylethanolamine-hydrolyzing phospholipase D, mitochondrial [OS=Saccharomyces cerevisiae S288c]	856001	FMP30; YPL103C	metabolic process	membrane;mitochondrion	catalytic activity;metal ion binding	0.194	0.557	2.87
Q12428	Probable 2-methylcitrate dehydratase [OS=Saccharomyces cerevisiae S288c]	856108	PDH1; YPR002W	metabolic process	cytoplasm;membrane;mitochondrion	catalytic activity	4.623	13.33	2.88
P30624	Long-chain-fatty-acid--CoA ligase 1 [OS=Saccharomyces cerevisiae S288c]	854495	FAA1; YOR317W	metabolic process;transport	endoplasmic reticulum;membrane;mitochondrion	catalytic activity;nucleotide binding	3.725	10.743	2.88
P32340	Rotenone-insensitive NADH-ubiquinone oxidoreductase, mitochondrial [OS=Saccharomyces cerevisiae S288c]	854919	NDI1; YML120C	metabolic process;regulation of biological process	membrane;mitochondrion;organelle lumen	catalytic activity;nucleotide binding	12.738	37.566	2.95
P21560	Protein CBP3, mitochondrial [OS=Saccharomyces cerevisiae S288c]	855886	CBP3; YPL215W	cell organization and biogenesis;regulation of biological process	membrane;mitochondrion;ribosome		0.557	1.649	2.96
P40495	Homoisocitrate dehydrogenase, mitochondrial [OS=Saccharomyces cerevisiae S288c]	854714	LYS12; YIL094C	metabolic process	mitochondrion	catalytic activity;metal ion binding;nucleotide binding	2.162	6.499	3.01
P53252	sphingolipid long chain base-responsive protein PIL1 [OS=Saccharomyces cerevisiae S288c]	852977	PIL1; YGR086C	cell organization and biogenesis;regulation of biological process;response to stimulus;transport	cytoplasm;membrane;mitochondrion	protein binding	24.809	75.27	3.03
P38523	GrpE protein homolog, mitochondrial [OS=Saccharomyces cerevisiae S288c]	854407	MGE1; YOR232W	metabolic process;regulation of biological process;transport	membrane;mitochondrion;organelle lumen	enzyme regulator activity;nucleotide binding;protein binding	0.274	0.833	3.04
P43567	alanine--glyoxylate aminotransferase 1 [OS=Saccharomyces cerevisiae S288c]	850514	AGX1; YFL030W	metabolic process	cytosol;mitochondrion	catalytic activity	0.616	1.873	3.04
P19262	Dihydrolipoyllysine-residue succinyltransferase component of 2-oxoglutarate dehydrogenase complex, mitochondrial [OS=Saccharomyces cerevisiae S288c]	851726	KGD2; YDR148C	cell organization and biogenesis;metabolic process	mitochondrion	catalytic activity;protein binding	4.484	13.925	3.11
P02992	elongation factor Tu, mitochondrial [OS=Saccharomyces cerevisiae S288c]	854359	TUF1; YOR187W	cell organization and biogenesis;metabolic process	mitochondrion	catalytic activity;nucleotide binding;RNA binding	6.017	19.309	3.21
Q05892	MIOREX complex component 2 [OS=Saccharomyces cerevisiae S288c]	850997	YLR290C; COQ11; YLR290C	metabolic process	mitochondrion	catalytic activity	0.54	1.738	3.22
P04037	cytochrome c oxidase subunit 4, mitochondrial [OS=Saccharomyces cerevisiae S288c]	852688	COX4; YGL187C	cell organization and biogenesis;metabolic process;transport	membrane;mitochondrion	catalytic activity;metal ion binding;transporter activity	4.337	14.199	3.27
P54003	Protein SUR7 [OS=Saccharomyces cerevisiae S288c]	854953	SUR7; YML052W	cell differentiation;transport	membrane;mitochondrion		0.585	1.929	3.30
P38325	Mitochondrial outer membrane protein OM14 [OS=Saccharomyces cerevisiae S288c]	852531	OM14; YBR230C	transport	membrane;mitochondrion		2.728	9	3.30
P35180	mitochondrial import receptor subunit Tom20 [OS=Saccharomyces cerevisiae S288c]	852973	TOM20; YGR082W	cell organization and biogenesis;transport	membrane;mitochondrion	protein binding;transporter activity	0.425	1.424	3.35
Q04689	Altered inheritance of mitochondria protein 32 [OS=Saccharomyces cerevisiae S288c]	854955	AIM32; YML050W				0.11	0.369	3.35
P32419	Malate dehydrogenase, peroxisomal [OS=Saccharomyces cerevisiae S288c]	851481	MDH3; YDL078C	metabolic process	organelle lumen	catalytic activity;nucleotide binding;RNA binding	2.162	7.254	3.36
P00401	Cytochrome c oxidase subunit 1 [OS=Saccharomyces cerevisiae S288c]	854598	COX1; Q0045	metabolic process;transport	membrane;mitochondrion	catalytic activity;metal ion binding;transporter activity	0.468	1.61	3.44
Q03557	Glutamyl-tRNA(Gln) amidotransferase subunit A, mitochondrial [OS=Saccharomyces cerevisiae S288c]	855338	HER2; YMR293C	cell organization and biogenesis;metabolic process	mitochondrion	catalytic activity;nucleotide binding	0.166	0.585	3.52
P32316	acetyl-CoA hydrolase [OS=Saccharomyces cerevisiae S288c]	852266	ACH1; YBL015W	metabolic process	cytoplasm;cytosol;mitochondrion	catalytic activity	8.326	29.539	3.55
P46367	Potassium-activated aldehyde dehydrogenase, mitochondrial [OS=Saccharomyces cerevisiae S288c]	854556	ALD4; YOR374W	metabolic process	mitochondrion;organelle lumen	catalytic activity	19.62	70.969	3.62

P00128	Cytochrome b-c1 complex subunit 7 [OS=Saccharomyces cerevisiae S288c]	852142	QCR7; YDR529C	cell organization and biogenesis;metabolic process;transport	membrane;mitochondrion	catalytic activity;transporter activity	1.783	6.743	3.78
P25349	Flavoprotein-like protein YCP4 [OS=Saccharomyces cerevisiae S288c]	850360	YCP4; YCR004C	metabolic process;regulation of biological process	cytoplasm;membrane;mitochondrion	catalytic activity;nucleotide binding;protein binding	3.084	11.915	3.86
P25605	Acetolactate synthase small subunit, mitochondrial [OS=Saccharomyces cerevisiae S288c]	850348	ILV6; YCL009C	metabolic process;regulation of biological process	mitochondrion	catalytic activity;enzyme regulator activity	1.976	7.859	3.98
P32891	D-lactate dehydrogenase [cytochrome] 1, mitochondrial [OS=Saccharomyces cerevisiae S288c]	851380	DLD1; YDL174C	metabolic process;transport	membrane;mitochondrion	catalytic activity;nucleotide binding	7.161	28.552	3.99
Q07914	mitochondrial import inner membrane translocase subunit TIM14 [OS=Saccharomyces cerevisiae S288c]	850694	PAM18; YLR008C	regulation of biological process;transport	membrane;mitochondrion	enzyme regulator activity;protein binding;transporter activity	0.334	1.371	4.10
Q12165	ATP synthase subunit delta, mitochondrial [OS=Saccharomyces cerevisiae S288c]	851560	ATP16; YDL004W	metabolic process;transport	membrane;mitochondrion	catalytic activity;transporter activity	0.334	1.371	4.10
P21954	Isocitrate dehydrogenase [NADP], mitochondrial [OS=Saccharomyces cerevisiae S288c]	851493	IDP1; YDL066W	metabolic process	mitochondrion	catalytic activity;metal ion binding;nucleotide binding;protein binding	11.589	49.119	4.24
P18900	hexaprenyl pyrophosphate synthase, mitochondrial [OS=Saccharomyces cerevisiae S288c]	852288	COQ1; YBR003W	metabolic process	membrane;mitochondrion	catalytic activity;metal ion binding;protein binding	0.166	0.711	4.28
Q06510	Lysophosphatidylcholine acyltransferase [OS=Saccharomyces cerevisiae S288c]	856262	TAZ1; YPR140W	cell organization and biogenesis;metabolic process;transport	membrane;mitochondrion	catalytic activity	0.093	0.425	4.57
P38885	Altered inheritance of mitochondria protein 46, mitochondrial [OS=Saccharomyces cerevisiae S288c]	856606	AIM46; YHR199C		mitochondrion	catalytic activity	0.245	1.154	4.71
Q06678	54S ribosomal protein L35, mitochondrial [OS=Saccharomyces cerevisiae S288c]	851921	MRPL35; YDR322W	cell organization and biogenesis;metabolic process	mitochondrion;ribosome	structural molecule activity	0.304	1.649	5.42
P07236	Threonine--tRNA ligase, mitochondrial [OS=Saccharomyces cerevisiae S288c]	853640	MST1; YKL194C	metabolic process	cytoplasm;mitochondrion;organelle lumen	catalytic activity;nucleotide binding;RNA binding	0.072	0.417	5.79
P81449	ATP synthase subunit e, mitochondrial [OS=Saccharomyces cerevisiae S288c]	851922	TIM11; YDR322C-A	cell organization and biogenesis;metabolic process;transport	membrane;mitochondrion	catalytic activity;structural molecule activity;transporter activity	0.334	2.162	6.47
P22353	54S ribosomal protein L8, mitochondrial [OS=Saccharomyces cerevisiae S288c]	853382	MRPL8; YJL063C	cell organization and biogenesis;metabolic process	mitochondrion;ribosome	structural molecule activity	0.166	1.154	6.95
P16547	Mitochondrial outer membrane protein OM45 [OS=Saccharomyces cerevisiae S288c]	854670	OM45; YIL136W		membrane;mitochondrion		274.423	2510.886	9.15

Supplemental Table S15. The relative concentrations of proteins in mitochondria purified from *ups2Δ* cells cultured with or without LCA. Mitochondria were purified from *ups2Δ* cells recovered on day 7 of cell culturing with or without LCA. Mass spectrometry-based identification and quantitation of proteins recovered in purified mitochondria were performed as described in Materials and methods. The exponentially modified protein abundance index (emPAI) provided by the ″Proteome Discoverer″ software was used to calculate the relative abundance of mitochondrial proteins between specified datasets. Data on the relative concentrations of mitochondrial proteins are presented as means of 2 independent experiments.

Accession	Description	Entrez Gene ID	Gene ID	Biological Process	Cellular Component	Molecular Function	emPAI *ups2*	emPAI *ups2* + LCA	Ratio *ups2* + LCA / *ups2*
Q01163	37S ribosomal protein S23, mitochondrial [OS=Saccharomyces cerevisiae S288c]	852748	RSM23; YGL129C	cell organization and biogenesis;metabolic process	mitochondrion;ribosome	catalytic activity;nucleotide binding;RNA binding;structural molecule activity	0.751	0.133	0.18
P00447	Superoxide dismutase [Mn], mitochondrial [OS=Saccharomyces cerevisiae S288c]	856399	SOD2; YHR008C	metabolic process;response to stimulus	mitochondrion;organelle lumen	antioxidant activity;catalytic activity;metal ion binding	1.894	0.701	0.37
Q12031	Mitochondrial 2-methylisocitrate lyase [OS=Saccharomyces cerevisiae S288c]	856114	ICL2; YPR006C	metabolic process	mitochondrion;organelle lumen	catalytic activity	1.976	0.833	0.42
P40035	Mitochondrial phosphate carrier protein 2 [OS=Saccharomyces cerevisiae S288c]	856779	PIC2; YER053C	cellular homeostasis;metabolic process;transport	membrane;mitochondrion	structural molecule activity;transporter activity	1.976	0.833	0.42
Q99297	Mitochondrial 2-oxodicarboxylate carrier 2 [OS=Saccharomyces cerevisiae S288c]	854397	ODC2; YOR222W	metabolic process;transport	membrane;mitochondrion	structural molecule activity;transporter activity	0.778	0.334	0.43
P32904	54S ribosomal protein L6, mitochondrial [OS=Saccharomyces cerevisiae S288c]	856552	MRPL6; YHR147C	cell organization and biogenesis;metabolic process	mitochondrion;ribosome	RNA binding;structural molecule activity	0.778	0.334	0.43
P17558	37S ribosomal protein PET123, mitochondrial [OS=Saccharomyces cerevisiae S288c]	854329	PET123; YOR158W	cell organization and biogenesis;metabolic process	mitochondrion;ribosome	structural molecule activity	0.668	0.292	0.44
P49334	Mitochondrial import receptor subunit tom22 [OS=Saccharomyces cerevisiae S288c]	855592	TOM22; YNL131W	cell organization and biogenesis;transport	membrane;mitochondrion	protein binding;transporter activity	0.668	0.292	0.44
P33759	37S ribosomal protein S5, mitochondrial [OS=Saccharomyces cerevisiae S288c]	852553	MRPS5; YBR251W	cell organization and biogenesis;metabolic process	mitochondrion;ribosome	RNA binding;structural molecule activity	0.585	0.259	0.44
P54857	lipase 2 [OS=Saccharomyces cerevisiae S288c]	851628	TGL2; YDR058C	metabolic process;transport	mitochondrion	catalytic activity	0.52	0.233	0.45
Q06892	NADH kinase POS5, mitochondrial [OS=Saccharomyces cerevisiae S288c]	855913	POS5; YPL188W	metabolic process;response to stimulus	mitochondrion;organelle lumen	catalytic activity;nucleotide binding	0.52	0.233	0.45
P36141	Putative redox protein fmp46, mitochondrial [OS=Saccharomyces cerevisiae S288c]	853923	FMP46; YKR049C	metabolic process	mitochondrion	catalytic activity;protein binding	0.52	0.233	0.45
P40452	Cytochrome c oxidase assembly factor 1 [OS=Saccharomyces cerevisiae S288c]	854649	COA1; YIL157C	cell organization and biogenesis	membrane;mitochondrion	protein binding	0.52	0.233	0.45
P34224	Uncharacterized protein YBL059W [OS=Saccharomyces cerevisiae S288c]	852221	YBL059W; YBL059W		membrane;mitochondrion		0.468	0.212	0.45
P36516	54S ribosomal protein L3, mitochondrial [OS=Saccharomyces cerevisiae S288c]	855039	MRPL3; YMR024W	cell organization and biogenesis;metabolic process;response to stimulus	mitochondrion;nucleus;ribosome	catalytic activity;RNA binding;structural molecule activity	0.374	0.172	0.46
P32611	54S ribosomal protein RML2, mitochondrial [OS=Saccharomyces cerevisiae S288c]	856660	RML2; YEL050C	cell organization and biogenesis;metabolic process	mitochondrion;ribosome	catalytic activity;RNA binding;structural molecule activity	0.389	0.179	0.46
Q12298	uncharacterized ABC transporter ATP-binding protein YDR061W [OS=Saccharomyces cerevisiae S288c]	851633	YDR061W; YDR061W		mitochondrion	catalytic activity;nucleotide binding	0.266	0.125	0.47
P38122	3-methyl-2-oxobutanoate hydroxymethyltransferase [OS=Saccharomyces cerevisiae S288c]	852474	ECM31; YBR176W	metabolic process	mitochondrion	catalytic activity	0.259	0.122	0.47
P36528	54S ribosomal protein L17, mitochondrial [OS=Saccharomyces cerevisiae S288c]	855469	MRPL17; YNL252C	cell organization and biogenesis;metabolic process	mitochondrion;ribosome	structural molecule activity	0.233	0.11	0.47
P51998	54S ribosomal protein yml6, mitochondrial [OS=Saccharomyces cerevisiae S288c]	854983	YML6; YML025C	cell organization and biogenesis;metabolic process	mitochondrion;ribosome	RNA binding;structural molecule activity	0.245	0.116	0.47
Q06510	Lysophosphatidylcholine acyltransferase [OS=Saccharomyces	856262	TAZ1; YPR140	cell organization and biogenesis;metabolic	membrane;mitochondrion	catalytic activity	0.194	0.093	0.48

	cerevisiae S288c]		W	process;transport					
P03879	Intron-encoded RNA maturase bI4 [OS=Saccharomyces cerevisiae S288c]	854582	BI4; Q0120	metabolic process	membrane;mitochondrion	catalytic activity;RNA binding	0.179	0.086	0.48
P40309	K(+)/H(+) antiporter 1 [OS=Saccharomyces cerevisiae S288c]	853351	KHA1; YJL094C	transport	endoplasmic reticulum;Golgi;membrane;mitochondrion	transporter activity	0.129	0.062	0.48
P25038	Translation initiation factor IF-2, mitochondrial [OS=Saccharomyces cerevisiae S288c]	854135	IFM1; YOL023W	cell organization and biogenesis;metabolic process	mitochondrion;nucleus	catalytic activity;nucleotide binding;RNA binding	0.11	0.054	0.49
P09440	C-1-tetrahydrofolate synthase, mitochondrial [OS=Saccharomyces cerevisiae S288c]	852378	MIS1; YBR084W	metabolic process	cytosol;mitochondrion	catalytic activity;nucleotide binding	0.9	0.459	0.51
Q03976	37S ribosomal protein S24, mitochondrial [OS=Saccharomyces cerevisiae S288c]	851755	RSM24; YDR175C	cell organization and biogenesis;metabolic process	mitochondrion;ribosome	structural molecule activity	0.585	0.318	0.54
P53969	Sorting assembly machinery 50 kDa subunit [OS=Saccharomyces cerevisiae S288c]	855705	SAM50; YNL026W	cell organization and biogenesis;transport	membrane;mitochondrion	protein binding;transporter activity	0.487	0.269	0.55
P27929	37S ribosomal protein NAM9, mitochondrial [OS=Saccharomyces cerevisiae S288c]	855585	NAM9; YNL137C	cell organization and biogenesis;metabolic process;regulation of biological process	mitochondrion;ribosome	RNA binding;structural molecule activity	0.389	0.218	0.56
P40364	Mitochondrial peculiar membrane protein 1 [OS=Saccharomyces cerevisiae S288c]	853379	MPM1; YJL066C		membrane;mitochondrion		1.512	0.848	0.56
P15179	Aspartate--tRNA ligase, mitochondrial [OS=Saccharomyces cerevisiae S288c]	856000	MSD1; YPL104W	metabolic process	cytoplasm;mitochondrion;organelle lumen	catalytic activity;nucleotide binding	0.307	0.174	0.57
P81449	ATP synthase subunit e, mitochondrial [OS=Saccharomyces cerevisiae S288c]	851922	TIM11; YDR322C-A	cell organization and biogenesis;metabolic process;transport	membrane;mitochondrion	catalytic activity;structural molecule activity;transporter activity	1.371	0.778	0.57
P38988	Mitochondrial GTP/GDP carrier protein 1 [OS=Saccharomyces cerevisiae S288c]	851329	GGC1; YDL198C	cell organization and biogenesis;cellular homeostasis;metabolic process;transport	membrane;mitochondrion	structural molecule activity;transporter activity	5.449	3.16	0.58
P39676	Flavohemoprotein [OS=Saccharomyces cerevisiae S288c]	853149	YHB1; YGR234W	metabolic process;response to stimulus	cytoplasm;cytosol;mitochondrion;nucleus;organelle lumen	catalytic activity;metal ion binding;protein binding	0.931	0.551	0.59
P04840	Mitochondrial outer membrane protein porin 1 [OS=Saccharomyces cerevisiae S288c]	855669	POR1; YNL055C	cell death;cell organization and biogenesis;cellular homeostasis;regulation of biological process;transport	cytoplasm;membrane;mitochondrion	transporter activity	19.691	11.743	0.60
P48015	Aminomethyltransferase, mitochondrial [OS=Saccharomyces cerevisiae S288c]	851582	GCV1; YDR019C	metabolic process	mitochondrion	catalytic activity;protein binding	5.062	3.062	0.60
P00431	cytochrome c peroxidase, mitochondrial [OS=Saccharomyces cerevisiae S288c]	853940	CCP1; YKR066C	metabolic process;response to stimulus	mitochondrion;organelle lumen	antioxidant activity;catalytic activity;metal ion binding;protein binding	0.701	0.425	0.61
P00942	Triosephosphate isomerase [OS=Saccharomyces cerevisiae S288c]	851620	TPI1; YDR050C	metabolic process	cytoplasm;cytosol;membrane;mitochondrion	catalytic activity	0.701	0.425	0.61
P40086	Cytochrome c oxidase assembly protein cox15 [OS=Saccharomyces cerevisiae S288c]	856884	COX15; YER141W	cell organization and biogenesis;metabolic process;transport	membrane;mitochondrion	catalytic activity;transporter activity	1.346	0.817	0.61
P43616	Cys-Gly metallodipeptidase dug1 [OS=Saccharomyces cerevisiae S288c]	850605	DUG1; YFR044C	metabolic process	cytoplasm;mitochondrion;ribosome	catalytic activity;metal ion binding	0.585	0.359	0.61
P36525	54S ribosomal protein L24, mitochondrial [OS=Saccharomyces cerevisiae S288c]	855231	MRPL24; YMR193W	cell organization and biogenesis;metabolic process	mitochondrion;ribosome	RNA binding;structural molecule activity	0.585	0.359	0.61
Q99190	Very-long-chain enoyl-CoA reductase [OS=Saccharomyces cerevisiae S288c]	851547	TSC13; YDL015C	metabolic process	cytoplasm;endoplasmic reticulum;membrane;mitochondrion	catalytic activity;protein binding	0.501	0.311	0.62
P36151	Uncharacterized protein YKR070W [OS=Saccharomyces cerevisiae S288c]	853944	YKR070W; YKR070W	metabolic process	mitochondrion		0.438	0.274	0.63
Q12480	Probable electron transfer flavoprotein subunit alpha, mitochondrial [OS=Saccharomyces cerevisiae S288c]	856112	AIM45; YPR004C	metabolic process;transport	mitochondrion;organelle lumen	catalytic activity;nucleotide binding	1.239	0.778	0.63
P32795	Mitochondrial inner membrane i-AAA protease supercomplex subunit YME1 [OS=Saccharomyces cerevisiae S288c]	856135	YME1; YPR024W	metabolic process;response to stimulus;transport	membrane;mitochondrion	catalytic activity;metal ion binding;nucleotide binding;protein binding	1.415	0.891	0.63
P38910	10 kDa heat shock protein, mitochondrial [OS=Saccharomyces cerevisiae S288c]	854185	HSP10; YOR020C	cell organization and biogenesis;metabolic process;response to stimulus;transport	cytoplasm;mitochondrion;organelle lumen	metal ion binding;nucleotide binding;protein binding	2.162	1.371	0.63

Q03430	37S ribosomal protein RSM28, mitochondrial [OS=Saccharomyces cerevisiae S288c]	852105	RSM28; YDR494W	cell organization and biogenesis;metabolic process	mitochondrion;ribosome	structural molecule activity	0.35	0.222	0.63
P32902	37S ribosomal protein MRP4, mitochondrial [OS=Saccharomyces cerevisiae S288c]	856384	MRP4; YHL004W	cell organization and biogenesis;metabolic process	mitochondrion;ribosome	structural molecule activity	0.334	0.212	0.63
P32473	Pyruvate dehydrogenase E1 component subunit beta, mitochondrial [OS=Saccharomyces cerevisiae S288c]	852522	PDB1; YBR221C	metabolic process	mitochondrion;organelle lumen	catalytic activity	5.158	3.281	0.64
P54783	D-arabinono-1,4-lactone oxidase [OS=Saccharomyces cerevisiae S288c]	854888	ALO1; YML086C	metabolic process;response to stimulus	membrane;mitochondrion	catalytic activity;nucleotide binding	2.981	1.929	0.65
P33310	ATP-dependent permease MDL1, mitochondrial [OS=Saccharomyces cerevisiae S288c]	850885	MDL1; YLR188W	transport	membrane;mitochondrion	catalytic activity;nucleotide binding;transporter activity	0.528	0.354	0.67
P39952	Mitochondrial inner membrane protein OXA1 [OS=Saccharomyces cerevisiae S288c]	856898	OXA1; YER154W	cell organization and biogenesis;transport	membrane;mitochondrion	transporter activity	3.217	2.162	0.67
P38325	Mitochondrial outer membrane protein OM14 [OS=Saccharomyces cerevisiae S288c]	852531	OM14; YBR230C	transport	membrane;mitochondrion		6.197	4.179	0.67
P00950	Phosphoglycerate mutase 1 [OS=Saccharomyces cerevisiae S288c]	853705	GPM1; YKL152C	metabolic process;regulation of biological process	cytoplasm;cytosol;membrane;mitochondrion	catalytic activity	4.412	2.981	0.68
P00560	Phosphoglycerate kinase [OS=Saccharomyces cerevisiae S288c]	850370	PGK1; YCR012W	metabolic process	cytoplasm;membrane;mitochondrion	catalytic activity;nucleotide binding	4.843	3.299	0.68
P00358	glyceraldehyde-3-phosphate dehydrogenase 2 [OS=Saccharomyces cerevisiae S288c]	853465	TDH2; YJR009C	cell death;metabolic process	cytoplasm;cytosol;membrane;mitochondrion	catalytic activity;nucleotide binding;protein binding	4.484	3.062	0.68
P33421	Succinate dehydrogenase [ubiquinone] cytochrome b subunit, mitochondrial [OS=Saccharomyces cerevisiae S288c]	853716	SDH3; YKL141W	cell organization and biogenesis;metabolic process;transport	membrane;mitochondrion	catalytic activity;metal ion binding	0.931	0.638	0.69
P32332	Mitochondrial oxaloacetate transport protein [OS=Saccharomyces cerevisiae S288c]	853739	OAC1; YKL120W	metabolic process;transport	membrane;mitochondrion	structural molecule activity;transporter activity	2.594	1.783	0.69
P39726	Glycine cleavage system H protein, mitochondrial [OS=Saccharomyces cerevisiae S288c]	851254	GCV3; YAL044C	metabolic process	mitochondrion	catalytic activity	2.594	1.783	0.69
P25719	Peptidyl-prolyl cis-trans isomerase C, mitochondrial [OS=Saccharomyces cerevisiae S288c]	854897	CPR3; YML078W	cell death;metabolic process	mitochondrion;organelle lumen	catalytic activity	0.848	0.585	0.69
Q08179	Mitochondrial distribution and morphology protein 38 [OS=Saccharomyces cerevisiae S288c]	854130	MDM38; YOL027C	cell organization and biogenesis;cellular homeostasis;regulation of biological process;transport	membrane;mitochondrion		5.31	3.732	0.70
P46367	Potassium-activated aldehyde dehydrogenase, mitochondrial [OS=Saccharomyces cerevisiae S288c]	854556	ALD4; YOR374W	metabolic process	mitochondrion;organelle lumen	catalytic activity	19.62	13.84	0.71
P36534	54S ribosomal protein L40, mitochondrial [OS=Saccharomyces cerevisiae S288c]	855930	MRPL40; YPL173W	cell organization and biogenesis;metabolic process	mitochondrion;ribosome	protein binding;structural molecule activity	0.585	0.413	0.71
P53736	Uncharacterized protein YNR040W [OS=Saccharomyces cerevisiae S288c]	855776	YNR040W; YNR040W		mitochondrion		0.551	0.389	0.71
P49095	Glycine dehydrogenase (Decarboxylating), mitochondrial [OS=Saccharomyces cerevisiae S288c]	855227	GCV2; YMR189W	metabolic process	cytosol;mitochondrion	catalytic activity	1.096	0.778	0.71
P17505	Malate dehydrogenase, mitochondrial [OS=Saccharomyces cerevisiae S288c]	853777	MDH1; YKL085W	metabolic process	mitochondrion;organelle lumen	catalytic activity;protein binding;RNA binding	32.405	23.041	0.71
P11914	Mitochondrial-processing peptidase subunit alpha [OS=Saccharomyces cerevisiae S288c]	856419	MAS2; YHR024C	metabolic process;transport	membrane;mitochondrion;organelle lumen	catalytic activity;metal ion binding;protein binding	0.445	0.318	0.71
Q04172	Sensitive to high expression protein 9, mitochondrial [OS=Saccharomyces cerevisiae S288c]	852002	SHE9; YDR393W	cell organization and biogenesis	membrane;mitochondrion		0.374	0.269	0.72
P32266	Dynamin-like GTPase MGM1, mitochondrial [OS=Saccharomyces cerevisiae S288c]	854386	MGM1; YOR211C	cell organization and biogenesis	membrane;mitochondrion	catalytic activity;nucleotide binding;protein binding	0.389	0.28	0.72
P40185	Protein mmf1, mitochondrial [OS=Saccharomyces cerevisiae S288c]	854760	MMF1; YIL051C	cell organization and biogenesis;metabolic process	mitochondrion;organelle lumen		2.981	2.162	0.73
P32191	Glycerol-3-phosphate dehydrogenase, mitochondrial [OS=Saccharomyces cerevisiae S288c]	854651	GUT2; YIL155C	metabolic process	membrane;mitochondrion	catalytic activity	4.289	3.14	0.73
P54003	Protein SUR7 [OS=Saccharomyces cerevisiae S288c]	854953	SUR7; YML052W	cell differentiation;transport	membrane;mitochondrion		1.154	0.848	0.73
P32860	NifU-like protein, mitochondrial [OS=Saccharomyces cerevisiae S288c]	853826	NFU1; YKL040	cell organization and biogenesis;cellular	mitochondrion;organelle lumen	metal ion binding	1.054	0.778	0.74

			C	homeostasis;metabolic process					
Q03028	Mitochondrial 2-oxodicarboxylate carrier 1 [OS=Saccharomyces cerevisiae S288c]	855969	ODC1; YPL134C	metabolic process;transport	membrane;mitochondrion	structural molecule activity;transporter activity	6.743	4.995	0.74
P00410	Cytochrome c oxidase subunit 2 [OS=Saccharomyces cerevisiae S288c]	854622	COX2; Q0250	metabolic process;transport	membrane;mitochondrion	catalytic activity;metal ion binding;protein binding;transporter activity	2.162	1.61	0.74
Q03798	Altered inheritance of mitochondria protein 36, mitochondrial [OS=Saccharomyces cerevisiae S288c]	855189	AIM36; YMR157C		membrane;mitochondrion		0.833	0.624	0.75
P06168	ketol-acid reductoisomerase, mitochondrial [OS=Saccharomyces cerevisiae S288c]	851069	ILV5; YLR355C	cell organization and biogenesis;metabolic process	mitochondrion	catalytic activity;DNA binding;metal ion binding	8.211	6.197	0.75
P37293	N-terminal acetyltransferase 2 [OS=Saccharomyces cerevisiae S288c]	853050	NAT2; YGR147C	metabolic process	cytoplasm;mitochondrion	catalytic activity	0.73	0.551	0.75
P28241	Isocitrate dehydrogenase [NAD] subunit 2, mitochondrial [OS=Saccharomyces cerevisiae S288c]	854303	IDH2; YOR136W	metabolic process	mitochondrion;organelle lumen	catalytic activity;metal ion binding;nucleotide binding;protein binding;RNA binding	4.179	3.16	0.76
P53598	Succinyl-CoA ligase [ADP-forming] subunit alpha, mitochondrial [OS=Saccharomyces cerevisiae S288c]	854310	LSC1; YOR142W	metabolic process	cytosol;mitochondrion	catalytic activity;nucleotide binding	7.859	5.952	0.76
P40047	Aldehyde dehydrogenase 5, mitochondrial [OS=Saccharomyces cerevisiae S288c]	856804	ALD5; YER073W	metabolic process	mitochondrion;organelle lumen	catalytic activity	1.106	0.84	0.76
P36101	tRNA threonylcarbamoyladenosine dehydratase 2 [OS=Saccharomyces cerevisiae S288c]	853841	TCD2; YKL027W; YKL027W	metabolic process	cytosol;membrane;mitochondrion	catalytic activity;nucleotide binding	0.616	0.468	0.76
P53230	Phosphatidate cytidylyltransferase, mitochondrial [OS=Saccharomyces cerevisiae S288c]	852937	TAM41; YGR046W	metabolic process	membrane;mitochondrion;organelle lumen	catalytic activity	0.532	0.407	0.77
P0CS90	Heat shock protein SSC1, mitochondrial [OS=Saccharomyces cerevisiae S288c]	853503	SSC1; YJR045C	cell organization and biogenesis;metabolic process;regulation of biological process;transport	membrane;mitochondrion;nucleus;organelle lumen	catalytic activity;enzyme regulator activity;nucleotide binding;protein binding	25.102	19.209	0.77
P35191	DnaJ homolog 1, mitochondrial [OS=Saccharomyces cerevisiae S288c]	850530	MDJ1; YFL016C	cell organization and biogenesis;metabolic process;regulation of biological process;response to stimulus	mitochondrion;organelle lumen	enzyme regulator activity;metal ion binding;nucleotide binding;protein binding	1.31	1.009	0.77
P32331	Carrier protein YMC1, mitochondrial [OS=Saccharomyces cerevisiae S288c]	856171	YMC1; YPR058W	metabolic process;transport	membrane;mitochondrion	structural molecule activity;transporter activity	1.254	0.968	0.77
P47127	Altered inheritance of mitochondria protein 24, mitochondrial [OS=Saccharomyces cerevisiae S288c]	853543	AIM24; YJR080C	cell organization and biogenesis	mitochondrion		1.807	1.395	0.77
P38071	Enoyl-[acyl-carrier protein] reductase [NADPH, B-specific], mitochondrial [OS=Saccharomyces cerevisiae S288c]	852314	ETR1; YBR026C	metabolic process	mitochondrion;organelle lumen	catalytic activity;DNA binding;metal ion binding	5.579	4.337	0.78
P38127	mitochondrial carrier protein RIM2 [OS=Saccharomyces cerevisiae S288c]	852491	RIM2; YBR192W	cell organization and biogenesis;metabolic process;transport	membrane;mitochondrion	structural molecule activity;transporter activity	0.995	0.778	0.78
P39522	Dihydroxy-acid dehydratase, mitochondrial [OS=Saccharomyces cerevisiae S288c]	853473	ILV3; YJR016C	metabolic process	mitochondrion	catalytic activity;metal ion binding	6.565	5.136	0.78
Q04458	Fatty aldehyde dehydrogenase HFD1 [OS=Saccharomyces cerevisiae S288c]	855137	HFD1; YMR110C	metabolic process	endoplasmic reticulum;endosome;membrane;mitochondrion	catalytic activity	2.594	2.03	0.78
P16387	Pyruvate dehydrogenase E1 component subunit alpha, mitochondrial [OS=Saccharomyces cerevisiae S288c]	856925	PDA1; YER178W	cell growth;metabolic process	mitochondrion;organelle lumen	catalytic activity	11.496	9	0.78
P40495	Homoisocitrate dehydrogenase, mitochondrial [OS=Saccharomyces cerevisiae S288c]	854714	LYS12; YIL094C	metabolic process	mitochondrion	catalytic activity;metal ion binding;nucleotide binding	4.623	3.642	0.79
Q08968	UPF0061 protein FMP40 [OS=Saccharomyces cerevisiae S288c]	855879	FMP40; YPL222W		mitochondrion	protein binding	0.823	0.65	0.79
P00359	Glyceraldehyde-3-phosphate dehydrogenase 3 [OS=Saccharomyces cerevisiae S288c]	853106	TDH3; YGR192C	cell death;metabolic process	cytoplasm;cytosol;membrane;mitochondrion	catalytic activity;nucleotide binding;RNA binding	6.406	5.062	0.79
P43567	alanine--glyoxylate aminotransferase 1 [OS=Saccharomyces cerevisiae S288c]	850514	AGX1; YFL030W	metabolic process	cytosol;mitochondrion	catalytic activity	0.778	0.616	0.79

P25605	Acetolactate synthase small subunit, mitochondrial [OS=Saccharomyces cerevisiae S288c]	850348	ILV6; YCL009C	metabolic process;regulation of biological process	mitochondrion	catalytic activity;enzyme regulator activity	1.336	1.069	0.80
P46681	D-lactate dehydrogenase [cytochrome] 2, mitochondrial [OS=Saccharomyces cerevisiae S288c]	851376	DLD2; YDL178W	metabolic process	mitochondrion;organelle lumen	catalytic activity;nucleotide binding;protein binding	0.562	0.45	0.80
P47045	mitochondrial import inner membrane translocase subunit TIM54 [OS=Saccharomyces cerevisiae S288c]	853392	TIM54; YJL054W	cell organization and biogenesis;transport	cytosol;membrane;mitochondrion	transporter activity	0.54	0.433	0.80
Q08822	Probable electron transfer flavoprotein-ubiquinone oxidoreductase, mitochondrial [OS=Saccharomyces cerevisiae S288c]	854538	CIR2; YOR356W	metabolic process;transport	membrane;mitochondrion	catalytic activity;metal ion binding;nucleotide binding	2.481	2.014	0.81
P08067	cytochrome b-c1 complex subunit Rieske, mitochondrial [OS=Saccharomyces cerevisiae S288c]	856689	RIP1; YEL024W	metabolic process;transport	membrane;mitochondrion	catalytic activity;metal ion binding;transporter activity	16.783	13.678	0.81
P53292	37S ribosomal protein S35, mitochondrial [OS=Saccharomyces cerevisiae S288c]	853075	MRPS35; YGR165W	cell organization and biogenesis;metabolic process	mitochondrion;ribosome	structural molecule activity	0.905	0.738	0.82
P19882	heat shock protein 60, mitochondrial [OS=Saccharomyces cerevisiae S288c]	850963	HSP60; YLR259C	cell organization and biogenesis;metabolic process;regulation of biological process;transport	cytoplasm;cytosol;membrane;mitochondrion;organelle lumen	catalytic activity;DNA binding;nucleotide binding;protein binding	32.598	27.014	0.83
Q03327	Mitochondrial fusion and transport protein Ugo1 [OS=Saccharomyces cerevisiae S288c]	852081	UGO1; YDR470C	cell organization and biogenesis;metabolic process	membrane;mitochondrion	protein binding;structural molecule activity	1.031	0.859	0.83
P12695	Dihydrolipoyllysine-residue acetyltransferase component of pyruvate dehydrogenase complex, mitochondrial [OS=Saccharomyces cerevisiae S288c]	855653	LAT1; YNL071W	metabolic process	mitochondrion;organelle lumen	catalytic activity	5.404	4.52	0.84
P40098	uncharacterized mitochondrial membrane protein FMP10 [OS=Saccharomyces cerevisiae S288c]	856931	FMP10; YER182W		membrane;mitochondrion		2.36	1.976	0.84
P00360	glyceraldehyde-3-phosphate dehydrogenase 1 [OS=Saccharomyces cerevisiae S288c]	853395	TDH1; YJL052W	metabolic process	cytoplasm;cytosol;membrane;mitochondrion	catalytic activity;nucleotide binding	1.462	1.228	0.84
Q12428	Probable 2-methylcitrate dehydratase [OS=Saccharomyces cerevisiae S288c]	856108	PDH1; YPR002W	metabolic process	cytoplasm;membrane;mitochondrion	catalytic activity	7.058	5.978	0.85
P53206	putative cysteine synthase [OS=Saccharomyces cerevisiae S288c]	852895	YGR012W; YGR012W	metabolic process	mitochondrion	catalytic activity	1.154	0.978	0.85
P07213	Mitochondrial import receptor subunit TOM70 [OS=Saccharomyces cerevisiae S288c]	855602	TOM70; YNL121C	cell organization and biogenesis;transport	membrane;mitochondrion	protein binding;transporter activity	3.125	2.665	0.85
P22136	ATPase expression protein 2, mitochondrial [OS=Saccharomyces cerevisiae S288c]	855325	AEP2; YMR282C	metabolic process;regulation of biological process	mitochondrion	RNA binding	0.585	0.506	0.86
P15424	ATP-dependent RNA helicase MSS116, mitochondrial [OS=Saccharomyces cerevisiae S288c]	851775	MSS116; YDR194C	metabolic process;regulation of biological process	mitochondrion;organelle lumen	catalytic activity;nucleotide binding;RNA binding	0.585	0.506	0.86
P33303	Succinate/fumarate mitochondrial transporter [OS=Saccharomyces cerevisiae S288c]	853558	SFC1; YJR095W	metabolic process;transport	membrane;mitochondrion	structural molecule activity;transporter activity	9	7.799	0.87
P43635	Citrate synthase 3, mitochondrial [OS=Saccharomyces cerevisiae S288c]	856107	CIT3; YPR001W	metabolic process	mitochondrion	catalytic activity	1.783	1.555	0.87
P49367	Homoaconitase, mitochondrial [OS=Saccharomyces cerevisiae S288c]	851820	LYS4; YDR234W	metabolic process	mitochondrion	catalytic activity;metal ion binding;protein binding	0.73	0.638	0.87
P32796-1	Carnitine O-acetyltransferase, mitochondrial [OS=Saccharomyces cerevisiae S288c]	854965	CAT2; YML042W	metabolic process;transport	membrane;mitochondrion	catalytic activity	6.552	5.749	0.88
P39518	Long-chain-fatty-acid--CoA ligase 2 [OS=Saccharomyces cerevisiae S288c]	856734	FAA2; YER015W	metabolic process;transport	cytoplasm;mitochondrion	catalytic activity;nucleotide binding	0.802	0.708	0.88
P36775	Lon protease homolog, mitochondrial [OS=Saccharomyces cerevisiae S288c]	852259	PIM1; YBL022C	cell organization and biogenesis;metabolic process;regulation of biological process;response to stimulus	cytoplasm;mitochondrion;organelle lumen	catalytic activity;DNA binding;nucleotide binding;RNA binding	0.402	0.359	0.89
P19414	Aconitate hydratase, mitochondrial [OS=Saccharomyces cerevisiae S288c]	851013	ACO1; YLR304C	cell organization and biogenesis;metabolic process;transport	cytoplasm;cytosol;mitochondrion;organelle lumen	catalytic activity;DNA binding;metal ion binding	18.169	16.343	0.90
Q07500	External NADH-ubiquinone oxidoreductase 2, mitochondrial [OS=Saccharomyces cerevisiae S288c]	851474	NDE2; YDL085W	metabolic process	mitochondrion	catalytic activity;nucleotide binding	4.012	3.642	0.91
P07275	Delta-1-pyrroline-5-carboxylate dehydrogenase, mitochondrial [OS=Saccharomyces cerevisiae S288c]	856432	PUT2; YHR037W	metabolic process	membrane;mitochondrion;organelle lumen	catalytic activity	2.765	2.511	0.91

P37292	Serine hydroxymethyltransferase, mitochondrial [OS=Saccharomyces cerevisiae S288c]	852565	SHM1; YBR263W	metabolic process	mitochondrion	catalytic activity	3.037	2.765	0.91
P00890	citrate synthase, mitochondrial [OS=Saccharomyces cerevisiae S288c]	855732	CIT1; YNR001C	metabolic process;transport	cytosol;mitochondrion;organelle lumen	catalytic activity	14.086	12.895	0.92
P53312	Succinyl-CoA ligase [ADP-forming] subunit beta, mitochondrial [OS=Saccharomyces cerevisiae S288c]	853159	LSC2; YGR244C	metabolic process	mitochondrion	catalytic activity;nucleotide binding	5.898	5.404	0.92
P32316	acetyl-CoA hydrolase [OS=Saccharomyces cerevisiae S288c]	852266	ACH1; YBL015W	metabolic process	cytoplasm;cytosol;mitochondrion	catalytic activity	8.326	7.697	0.92
P07342	Acetolactate synthase catalytic subunit, mitochondrial [OS=Saccharomyces cerevisiae S288c]	855135	ILV2; YMR108W	metabolic process	mitochondrion	catalytic activity;metal ion binding;nucleotide binding	3.642	3.375	0.93
Q00711	Succinate dehydrogenase [ubiquinone] flavoprotein subunit, mitochondrial [OS=Saccharomyces cerevisiae S288c]	853709	SDH1; YKL148C	metabolic process;transport	membrane;mitochondrion	catalytic activity;nucleotide binding;protein binding	5.381	5.032	0.94
P20967	2-oxoglutarate dehydrogenase, mitochondrial [OS=Saccharomyces cerevisiae S288c]	854681	KGD1; YIL125W	metabolic process	cytosol;mitochondrion;organelle lumen	catalytic activity	9.908	9.444	0.95
P16547	Mitochondrial outer membrane protein OM45 [OS=Saccharomyces cerevisiae S288c]	854670	OM45; YIL136W		membrane;mitochondrion		330.131	330.131	1.00
P00830	ATP synthase subunit beta, mitochondrial [OS=Saccharomyces cerevisiae S288c]	853585	ATP2; YJR121W	metabolic process;transport	cytosol;membrane;mitochondrion	catalytic activity;nucleotide binding;protein binding;transporter activity	26.123	26.123	1.00
Q12230	Sphingolipid long chain base-responsive protein LSP1 [OS=Saccharomyces cerevisiae S288c]	856103	LSP1; YPL004C	regulation of biological process;response to stimulus;transport	cytoplasm;membrane;mitochondrion		62.096	62.096	1.00
P23641	mitochondrial phosphate carrier protein [OS=Saccharomyces cerevisiae S288c]	853540	MIR1; YJR077C	metabolic process;transport	membrane;mitochondrion	protein binding;structural molecule activity;transporter activity	13.678	13.678	1.00
P38077	ATP synthase subunit gamma, mitochondrial [OS=Saccharomyces cerevisiae S288c]	852327	ATP3; YBR039W	metabolic process;transport	membrane;mitochondrion	catalytic activity;transporter activity	12.689	12.689	1.00
P07143	Cytochrome c1, heme protein, mitochondrial [OS=Saccharomyces cerevisiae S288c]	854231	CYT1; YOR065W	metabolic process;transport	cytosol;membrane;mitochondrion	metal ion binding	16.013	16.013	1.00
P06208-1	2-isopropylmalate synthase [OS=Saccharomyces cerevisiae S288c]	855619	LEU4; YNL104C	metabolic process	cytoplasm;mitochondrion	catalytic activity;protein binding	3.125	3.125	1.00
P25349	Flavoprotein-like protein YCP4 [OS=Saccharomyces cerevisiae S288c]	850360	YCP4; YCR004C	metabolic process;regulation of biological process	cytoplasm;membrane;mitochondrion	catalytic activity;nucleotide binding;protein binding	9	9	1.00
P36060	NADH-cytochrome b5 reductase 2 [OS=Saccharomyces cerevisiae S288c]	853707	MCR1; YKL150W	metabolic process;response to stimulus	membrane;mitochondrion	catalytic activity;protein binding	10.288	10.288	1.00
P47052	succinate dehydrogenase [ubiquinone] flavoprotein subunit 2, mitochondrial [OS=Saccharomyces cerevisiae S288c]	853405	YJL045W; YJL045W	metabolic process;transport	membrane;mitochondrion	catalytic activity;nucleotide binding	2.065	2.065	1.00
P28834	Isocitrate dehydrogenase [NAD] subunit 1, mitochondrial [OS=Saccharomyces cerevisiae S288c]	855691	IDH1; YNL037C	metabolic process;transport	cytosol;mitochondrion;organelle lumen	catalytic activity;metal ion binding;nucleotide binding;protein binding;RNA binding	3.833	3.833	1.00
P15108	ATP-dependent molecular chaperone HSC82 [OS=Saccharomyces cerevisiae S288c]	855224	HSC82; YMR186W	cell organization and biogenesis;metabolic process;response to stimulus	cytoplasm;membrane;mitochondrion	catalytic activity;nucleotide binding;protein binding	1.081	1.081	1.00
P04710	ADP,ATP carrier protein 1 [OS=Saccharomyces cerevisiae S288c]	855078	AAC1; YMR056C	metabolic process;transport	cytosol;membrane;mitochondrion	structural molecule activity;transporter activity	6.848	6.848	1.00
P07246	Alcohol dehydrogenase 3, mitochondrial [OS=Saccharomyces cerevisiae S288c]	855107	ADH3; YMR083W	metabolic process	mitochondrion;organelle lumen	catalytic activity;metal ion binding	4.179	4.179	1.00
P36046	Mitochondrial intermembrane space import and assembly protein 40 [OS=Saccharomyces cerevisiae S288c]	853639	MIA40; YKL195W	cellular component movement;metabolic process;response to stimulus;transport	membrane;mitochondrion	catalytic activity;protein binding;transporter activity	2.03	2.03	1.00
P04037	cytochrome c oxidase subunit 4, mitochondrial [OS=Saccharomyces cerevisiae S288c]	852688	COX4; YGL187C	cell organization and biogenesis;metabolic process;transport	membrane;mitochondrion	catalytic activity;metal ion binding;transporter activity	9	9	1.00
P40012	protoporphyrinogen oxidase [OS=Saccharomyces cerevisiae S288c]	856733	HEM14; YER014W	metabolic process	membrane;mitochondrion	catalytic activity	0.947	0.947	1.00
P40961	prohibitin-1 [OS=Saccharomyces cerevisiae S288c]	853033	PHB1; YGR132C	cell organization and biogenesis;metabolic process;regulation of biological	membrane;mitochondrion	protein binding	2.793	2.793	1.00

				process					
P50088	Stationary phase gene 1 protein [OS=Saccharomyces cerevisiae S288c]	853151	SPG1; YGR236C		endoplasmic reticulum;membrane;mitochondrion		62.096	62.096	1.00
P53140	Protein RMD9, mitochondrial [OS=Saccharomyces cerevisiae S288c]	852771	RMD9; YGL107C	cell differentiation;metabolic process;regulation of biological process	membrane;mitochondrion	RNA binding	0.585	0.585	1.00
P23180	Probable oxidoreductase AIM17 [OS=Saccharomyces cerevisiae S288c]	856365	AIM17; YHL021C	cell organization and biogenesis;metabolic process	mitochondrion	catalytic activity;metal ion binding	1.818	1.818	1.00
P53721	Respiratory supercomplex factor 2, mitochondrial [OS=Saccharomyces cerevisiae S288c]	855752	RCF2; YNR018W	cell organization and biogenesis	membrane;mitochondrion		7.111	7.111	1.00
Q12289	mitochondrial carnitine carrier [OS=Saccharomyces cerevisiae S288c]	854267	CRC1; YOR100C	metabolic process;transport	membrane;mitochondrion	structural molecule activity;transporter activity	1.683	1.683	1.00
P08466	mitochondrial nuclease [OS=Saccharomyces cerevisiae S288c]	853222	NUC1; YJL208C	cell death;metabolic process	membrane;mitochondrion;nucleus	catalytic activity;metal ion binding	1.976	1.976	1.00
P38626	NADH-cytochrome b5 reductase 1 [OS=Saccharomyces cerevisiae S288c]	854768	CBR1; YIL043C	metabolic process	endoplasmic reticulum;membrane;mitochondrion	catalytic activity	1.61	1.61	1.00
P25374	Cysteine desulfurase, mitochondrial [OS=Saccharomyces cerevisiae S288c]	850343	NFS1; YCL017C	cell organization and biogenesis;cellular homeostasis;metabolic process	mitochondrion;nucleus	catalytic activity;metal ion binding;protein binding	0.817	0.817	1.00
Q08245	protein ZEO1 [OS=Saccharomyces cerevisiae S288c]	854040	ZEO1; YOL109W	cell organization and biogenesis	membrane;mitochondrion	protein binding	5.31	5.31	1.00
P40416	Iron-sulfur clusters transporter ATM1, mitochondrial [OS=Saccharomyces cerevisiae S288c]	855347	ATM1; YMR301C	cellular homeostasis;metabolic process;transport	membrane;mitochondrion	catalytic activity;nucleotide binding;transporter activity	0.546	0.546	1.00
P32898	Mitochondrial presequence protease [OS=Saccharomyces cerevisiae S288c]	852041	CYM1; YDR430C	metabolic process	mitochondrion	catalytic activity;metal ion binding	0.297	0.297	1.00
Q06493	LETM1 domain-containing protein YLH47, mitochondrial [OS=Saccharomyces cerevisiae S288c]	856243	YLH47; YPR125W	cell organization and biogenesis;transport	membrane;mitochondrion		0.61	0.61	1.00
P40508	Uncharacterized protein YIL077C [OS=Saccharomyces cerevisiae S288c]	854733	YIL077C; YIL077C		mitochondrion		0.896	0.896	1.00
P40008	Protein FMP52, mitochondrial [OS=Saccharomyces cerevisiae S288c]	856721	FMP52; YER004W		endoplasmic reticulum;membrane;mitochondrion	catalytic activity	1.371	1.371	1.00
P32317	Protein AFG1 [OS=Saccharomyces cerevisiae S288c]	856658	AFG1; YEL052W	cell organization and biogenesis;metabolic process;response to stimulus;transport	membrane;mitochondrion	catalytic activity;nucleotide binding	0.585	0.585	1.00
P38072	Protein SCO2, mitochondrial [OS=Saccharomyces cerevisiae S288c]	852312	SCO2; YBR024W	cell organization and biogenesis;cellular homeostasis;metabolic process;transport	membrane;mitochondrion	antioxidant activity;catalytic activity;metal ion binding	0.73	0.73	1.00
P28737	Protein MSP1 [OS=Saccharomyces cerevisiae S288c]	852915	MSP1; YGR028W	cell organization and biogenesis;metabolic process;regulation of biological process;response to stimulus;transport	membrane;mitochondrion;nucleus	catalytic activity;nucleotide binding;protein binding;RNA binding	0.778	0.778	1.00
P10507	mitochondrial-processing peptidase subunit beta [OS=Saccharomyces cerevisiae S288c]	850860	MAS1; YLR163C	metabolic process;transport	mitochondrion;organelle lumen	catalytic activity;metal ion binding;protein binding	0.616	0.616	1.00
P32453	Protein ATP11, mitochondrial [OS=Saccharomyces cerevisiae S288c]	855401	ATP11; YNL315C	cell organization and biogenesis	mitochondrion	protein binding	0.585	0.585	1.00
P37298	Succinate dehydrogenase [ubiquinone] cytochrome b small subunit, mitochondrial [OS=Saccharomyces cerevisiae S288c]	851758	SDH4; YDR178W	metabolic process;transport	membrane;mitochondrion	catalytic activity;metal ion binding	1.154	1.154	1.00
P19955	37S ribosomal protein YMR-31, mitochondrial [OS=Saccharomyces cerevisiae S288c]	850610	YMR31; YFR049W	cell organization and biogenesis;metabolic process	mitochondrion;ribosome	catalytic activity;protein binding;structural molecule activity	2.594	2.594	1.00
Q08223	Altered inheritance of mitochondria protein 39, mitochondrial [OS=Saccharomyces cerevisiae S288c]	854103	AIM39; YOL053W		cytosol;membrane;mitochondrion		0.468	0.468	1.00
P38523	GrpE protein homolog, mitochondrial [OS=Saccharomyces cerevisiae S288c]	854407	MGE1; YOR232W	metabolic process;regulation of biological process;transport	membrane;mitochondrion;organelle lumen	enzyme regulator activity;nucleotide binding;protein binding	0.624	0.624	1.00
Q06567	ABC1 family protein MCP2 [OS=Saccharomyces cerevisiae S288c]	850955	YLR253W; MCP2; YLR253W	cell organization and biogenesis	membrane;mitochondrion		0.218	0.218	1.00
Q05931	Heat shock protein SSQ1, mitochondrial [OS=Saccharomyces cerevisiae S288c]	851084	SSQ1; YLR369W	cell organization and biogenesis;metabolic process	mitochondrion;organelle lumen	nucleotide binding;protein binding	0.413	0.413	1.00

Q05892	MIOREX complex component 2 [OS=Saccharomyces cerevisiae S288c]	850997	YLR290C; COQ11; YLR290C	metabolic process	mitochondrion	catalytic activity	1.054	1.054	1.00
P39722	Mitochondrial Rho GTPase 1 [OS=Saccharomyces cerevisiae S288c]	851249	GEM1; YAL048C	cell organization and biogenesis;regulation of biological process;response to stimulus;transport	membrane;mitochondrion	catalytic activity;metal ion binding;nucleotide binding	0.259	0.259	1.00
Q02950	37S ribosomal protein MRP51, mitochondrial [OS=Saccharomyces cerevisiae S288c]	855985	MRP51; YPL118W	cell organization and biogenesis;metabolic process	mitochondrion;ribosome	structural molecule activity	0.407	0.407	1.00
P40513	Mitochondrial acidic protein MAM33 [OS=Saccharomyces cerevisiae S288c]	854740	MAM33; YIL070C	metabolic process;regulation of biological process	mitochondrion;organelle lumen	translation regulator activity	0.778	0.778	1.00
P25039	Elongation factor G, mitochondrial [OS=Saccharomyces cerevisiae S288c]	850758	MEF1; YLR069C	cell organization and biogenesis;metabolic process	cytosol;mitochondrion	catalytic activity;nucleotide binding;RNA binding	0.207	0.207	1.00
P00401	Cytochrome c oxidase subunit 1 [OS=Saccharomyces cerevisiae S288c]	854598	COX1; Q0045	metabolic process;transport	membrane;mitochondrion	catalytic activity;metal ion binding;transporter activity	0.778	0.778	1.00
Q3E824	Uncharacterized protein YOR020W-A [OS=Saccharomyces cerevisiae S288c]	1466480	YOR020W-A; YOR020W-A	metabolic process;transport	membrane;mitochondrion		2.162	2.162	1.00
P43617	Uncharacterized mitochondrial carrier YFR045W [OS=Saccharomyces cerevisiae S288c]	850606	YFR045W; YFR045W	metabolic process;transport	membrane;mitochondrion	structural molecule activity;transporter activity	0.413	0.413	1.00
P39006	Phosphatidylserine decarboxylase proenzyme 1, mitochondrial [OS=Saccharomyces cerevisiae S288c]	855552	PSD1; YNL169C	metabolic process;regulation of biological process	membrane;mitochondrion	catalytic activity	0.269	0.269	1.00
P38702	mitochondrial carrier protein LEU5 [OS=Saccharomyces cerevisiae S288c]	856391	LEU5; YHR002W	metabolic process;transport	membrane;mitochondrion	structural molecule activity;transporter activity	0.334	0.334	1.00
P50087	MICOS subunit MIC26 [OS=Saccharomyces cerevisiae S288c]	853150	MOS2; MIC26; YGR235C	cell organization and biogenesis	membrane;mitochondrion		0.931	0.931	1.00
Q05648	MIOREX complex component 10 [OS=Saccharomyces cerevisiae S288c]	851876	YDR282C; MRX10; YDR282C		membrane;mitochondrion		0.35	0.35	1.00
Q02204	54S ribosomal protein L13, mitochondrial [OS=Saccharomyces cerevisiae S288c]	853875	MRPL13; YKR006C	cell organization and biogenesis;metabolic process	mitochondrion;ribosome	structural molecule activity	0.334	0.334	1.00
P53881	54S ribosomal protein L22, mitochondrial [OS=Saccharomyces cerevisiae S288c]	855544	MRPL22; YNL177C	cell organization and biogenesis;metabolic process	mitochondrion;ribosome	RNA binding;structural molecule activity	0.389	0.389	1.00
P32839	Mitochondrial chaperone BCS1 [OS=Saccharomyces cerevisiae S288c]	851981	BCS1; YDR375C	cell organization and biogenesis;metabolic process;response to stimulus;transport	cytosol;membrane;mitochondrion	catalytic activity;nucleotide binding;transporter activity	0.194	0.194	1.00
Q08970	Mitochondrial metal transporter 2 [OS=Saccharomyces cerevisiae S288c]	855877	MMT2; YPL224C	cellular homeostasis;regulation of biological process;response to stimulus;transport	membrane;mitochondrion	transporter activity	0.292	0.292	1.00
Q06485	Autophagy-related protein 33 [OS=Saccharomyces cerevisiae S288c]	851070	ATG33; YLR356W	cell communication;metabolic process;response to stimulus	membrane;mitochondrion		1.371	1.371	1.00
Q03246	37S ribosomal protein S17, mitochondrial [OS=Saccharomyces cerevisiae S288c]	855226	MRPS17; YMR188C	cell differentiation;cell organization and biogenesis;metabolic process	mitochondrion;ribosome	RNA binding;structural molecule activity	0.389	0.389	1.00
P38088	Glycine--tRNA ligase 1, mitochondrial [OS=Saccharomyces cerevisiae S288c]			metabolic process	cytoplasm;mitochondrion;organelle lumen	catalytic activity;nucleotide binding;protein binding	0.155	0.155	1.00
P32387	54S ribosomal protein L41, mitochondrial [OS=Saccharomyces cerevisiae S288c]	852014	MRP20; YDR405W	cell organization and biogenesis;metabolic process	mitochondrion;ribosome	nucleotide binding;protein binding;RNA binding;structural molecule activity	0.501	0.501	1.00
P53163	54S ribosomal protein L12, mitochondrial [OS=Saccharomyces cerevisiae S288c]	852811	MNP1; YGL068W	cell organization and biogenesis;metabolic process	mitochondrion;ribosome	RNA binding;structural molecule activity	0.874	0.874	1.00
P38152	Tricarboxylate transport protein [OS=Saccharomyces cerevisiae S288c]	852594	CTP1; YBR291C	metabolic process;transport	membrane;mitochondrion	structural molecule activity;transporter activity	0.245	0.245	1.00
P00427	Cytochrome c oxidase subunit 6, mitochondrial [OS=Saccharomyces cerevisiae S288c]	856448	COX6; YHR051W	metabolic process;transport	membrane;mitochondrion	catalytic activity;metal ion binding;transporter activity	0.778	0.778	1.00

P36163	mitochondrial metalloendopeptidase OMA1 [OS=Saccharomyces cerevisiae S288c]	853962	OMA1; YKR087C	metabolic process	membrane;mitochondrion	catalytic activity;metal ion binding	0.389	0.389	1.00
Q08818	Meiotic sister-chromatid recombination protein 6, mitochondrial [OS=Saccharomyces cerevisiae S288c]	854536	MSC6; YOR354C	metabolic process	mitochondrion;organelle lumen	RNA binding	0.227	0.227	1.00
Q96VH5	MICOS complex subunit Mic10 [OS=Saccharomyces cerevisiae S288c]	850300	MOS1; MIC10; YCL057C-A	cell organization and biogenesis	membrane;mitochondrion		0.931	0.931	1.00
P10834	protein PET54 [OS=Saccharomyces cerevisiae S288c]	853137	PET54; YGR222W	metabolic process;regulation of biological process	membrane;mitochondrion;organelle lumen	nucleotide binding;RNA binding;translation regulator activity	0.369	0.369	1.00
P39515	mitochondrial import inner membrane translocase subunit Tim17 [OS=Saccharomyces cerevisiae S288c]	853298	TIM17; YJL143W	cell organization and biogenesis;transport	membrane;mitochondrion	protein binding;transporter activity	1.371	1.371	1.00
P40496	37S ribosomal protein S25, mitochondrial [OS=Saccharomyces cerevisiae S288c]	854715	RSM25; YIL093C	cell organization and biogenesis;metabolic process	mitochondrion;ribosome	structural molecule activity	0.468	0.468	1.00
P18900	hexaprenyl pyrophosphate synthase, mitochondrial [OS=Saccharomyces cerevisiae S288c]	852288	COQ1; YBR003W	metabolic process	membrane;mitochondrion	catalytic activity;metal ion binding;protein binding	0.259	0.259	1.00
Q01532-1	Cysteine proteinase 1, mitochondrial [OS=Saccharomyces cerevisiae S288c]			metabolic process;regulation of biological process;response to stimulus	cytoplasm;mitochondrion	catalytic activity;DNA binding;RNA binding	0.179	0.179	1.00
P34222	Peptidyl-tRNA hydrolase 2 [OS=Saccharomyces cerevisiae S288c]	852223	PTH2; YBL057C	regulation of biological process	cytoplasm;cytosol;membrane;mitochondrion	catalytic activity;protein binding	0.425	0.425	1.00
Q06405	ATP synthase subunit f, mitochondrial [OS=Saccharomyces cerevisiae S288c]	851983	ATP17; YDR377W	metabolic process;transport	membrane;mitochondrion	catalytic activity;transporter activity	2.981	2.981	1.00
Q12251	Uncharacterized mitochondrial carrier YPR011C [OS=Saccharomyces cerevisiae S288c]	856121	YPR011C; YPR011C	metabolic process;transport	membrane;mitochondrion	structural molecule activity;transporter activity	0.389	0.389	1.00
P38271	Protein OPY1 [OS=Saccharomyces cerevisiae S288c]	852426	OPY1; YBR129C		cytoplasm;mitochondrion		0.274	0.274	1.00
P36147	Presequence translocated-associated motor subunit pam17, mitochondrial [OS=Saccharomyces cerevisiae S288c]	853939	PAM17; YKR065C	transport	membrane;mitochondrion		0.778	0.778	1.00
Q01976	ADP-ribose pyrophosphatase [OS=Saccharomyces cerevisiae S288c]	852408	YSA1; YBR111C	metabolic process	cytoplasm;mitochondrion;nucleus	catalytic activity;metal ion binding	0.194	0.194	1.00
P36517	54S ribosomal protein L4, mitochondrial [OS=Saccharomyces cerevisiae S288c]	851160	MRPL4; YLR439W	cell organization and biogenesis;metabolic process	mitochondrion;ribosome	structural molecule activity	0.369	0.369	1.00
Q05867	Uncharacterized protein YLR283W, mitochondrial [OS=Saccharomyces cerevisiae S288c]	850988	YLR283W; YLR283W		endoplasmic reticulum;membrane;mitochondrion		0.11	0.11	1.00
P00127	Cytochrome b-c1 complex subunit 6 [OS=Saccharomyces cerevisiae S288c]	850593	QCR6; YFR033C	metabolic process;transport	membrane;mitochondrion	catalytic activity;transporter activity	0.334	0.334	1.00
P02381	Ribosomal protein VAR1, mitochondrial [OS=Saccharomyces cerevisiae S288c]	854586	VAR1; Q0140	cell organization and biogenesis;metabolic process	mitochondrion;ribosome	structural molecule activity	0.11	0.11	1.00
P40581	peroxiredoxin HYR1 [OS=Saccharomyces cerevisiae S288c]	854855	HYR1; YIR037W	metabolic process;response to stimulus	cytoplasm;cytosol;mitochondrion;organelle lumen	antioxidant activity;catalytic activity	0.259	0.259	1.00
P30952	Malate synthase 1, glyoxysomal [OS=Saccharomyces cerevisiae S288c]	855606	MLS1; YNL117W	metabolic process	cytoplasm;cytosol;organelle lumen	catalytic activity;protein binding	0.145	0.145	1.00
P29704	squalene synthase [OS=Saccharomyces cerevisiae S288c]	856597	ERG9; YHR190W	metabolic process	endoplasmic reticulum;membrane;mitochondrion	catalytic activity	0.35	0.35	1.00
P32378	4-hydroxybenzoate polyprenyltransferase, mitochondrial [OS=Saccharomyces cerevisiae S288c]	855778	COQ2; YNR041C	metabolic process;transport	membrane;mitochondrion	catalytic activity	0.292	0.292	1.00
P14693	sorting assembly machinery 35 kDa subunit [OS=Saccharomyces cerevisiae S288c]	856483	SAM35; YHR083W	cell organization and biogenesis;transport	membrane;mitochondrion	protein binding	0.334	0.334	1.00
Q00873	cytochrome c1 heme lyase [OS=Saccharomyces cerevisiae S288c]	853775	CYT2; YKL087C	cell organization and biogenesis;metabolic process	membrane;mitochondrion	catalytic activity;metal ion binding	0.359	0.359	1.00
P46998	mitochondrial membrane protein FMP33 [OS=Saccharomyces cerevisiae S288c]	853279	FMP33; YJL161W		membrane;mitochondrion		0.334	0.334	1.00
P28817	3-hydroxyisobutyryl-CoA hydrolase, mitochondrial [OS=Saccharomyces cerevisiae S288c]	851606	EHD3; YDR036C	metabolic process	mitochondrion	catalytic activity	0.172	0.172	1.00
Q2V2P9	Uncharacterized protein YDR119W-A [OS=Saccharomyces cerevisiae S288c]	3799970	COX26; YDR119W-A		membrane;mitochondrion		1.154	1.154	1.00

P36520	54S ribosomal protein L10, mitochondrial [OS=Saccharomyces cerevisiae S288c]	855436	MRPL10; YNL284C	cell organization and biogenesis;metabolic process	mitochondrion;ribosome	structural molecule activity	0.116	0.116	1.00
P38300	Inner membrane mitoribosome receptor MBA1, mitochondrial [OS=Saccharomyces cerevisiae S288c]	852483	MBA1; YBR185C	cell organization and biogenesis;regulation of biological process	membrane;mitochondrion		0.259	0.259	1.00
P42847	37S ribosomal protein S18, mitochondrial [OS=Saccharomyces cerevisiae S288c]	855410	MRPS18; YNL306W	cell organization and biogenesis;metabolic process	mitochondrion;ribosome	RNA binding;structural molecule activity	0.155	0.155	1.00
P36526	54S ribosomal protein L27, mitochondrial [OS=Saccharomyces cerevisiae S288c]	852585	MRPL27; YBR282W	cell organization and biogenesis;metabolic process	mitochondrion;ribosome	structural molecule activity	0.259	0.259	1.00
P32463	Acyl carrier protein, mitochondrial [OS=Saccharomyces cerevisiae S288c]	853642	ACP1; YKL192C	metabolic process;transport	mitochondrion		0.259	0.259	1.00
P40165	NAD(P)H-hydrate epimerase [OS=Saccharomyces cerevisiae S288c]	855521	YNL200C; YNL200C	metabolic process	cytoplasm;mitochondrion	catalytic activity;metal ion binding;nucleotide binding	0.212	0.212	1.00
P36523	54S ribosomal protein L15, mitochondrial [OS=Saccharomyces cerevisiae S288c]	851022	MRPL15; YLR312W-A	cell organization and biogenesis;metabolic process	mitochondrion;ribosome	catalytic activity;RNA binding;structural molecule activity	0.155	0.155	1.00
P48526	Isoleucine--tRNA ligase, mitochondrial [OS=Saccharomyces cerevisiae S288c]	856067	ISM1; YPL040C	cell organization and biogenesis;metabolic process;regulation of biological process	cytoplasm;cytosol;mitochondrion;organelle lumen	catalytic activity;nucleotide binding	0.04	0.04	1.00
Q06089	Uncharacterized mitochondrial outer membrane protein YPR098C [OS=Saccharomyces cerevisiae S288c]	856213	YPR098C; YPR098C		membrane;mitochondrion		2.162	2.162	1.00
P53320	Mitochondrial carrier protein MTM1 [OS=Saccharomyces cerevisiae S288c]	853173	MTM1; YGR257C	metabolic process;transport	membrane;mitochondrion	structural molecule activity	0.11	0.11	1.00
P21306	ATP synthase subunit epsilon, mitochondrial [OS=Saccharomyces cerevisiae S288c]	855857	ATP15; YPL271W	metabolic process;transport	membrane;mitochondrion	catalytic activity;transporter activity	0.778	0.778	1.00
P38909	cytochrome c mitochondrial import factor CYC2 [OS=Saccharomyces cerevisiae S288c]	854202	CYC2; YOR037W	cell organization and biogenesis;metabolic process	membrane;mitochondrion	catalytic activity	0.194	0.194	1.00
P21771	37S ribosomal protein S28, mitochondrial [OS=Saccharomyces cerevisiae S288c]	851937	MRPS28; YDR337W	cell organization and biogenesis;metabolic process	mitochondrion;ribosome	RNA binding;structural molecule activity	0.129	0.129	1.00
P04039	Cytochrome c oxidase polypeptide VIII, mitochondrial [OS=Saccharomyces cerevisiae S288c]	851111	COX8; YLR395C	metabolic process;transport	membrane;mitochondrion	catalytic activity;transporter activity	0.778	0.778	1.00
Q04623	Protein ECM18 [OS=Saccharomyces cerevisiae S288c]	851703	ECM18; YDR125C	cell organization and biogenesis	mitochondrion	catalytic activity	0.08	0.08	1.00
P00045	Cytochrome c iso-2 [OS=Saccharomyces cerevisiae S288c]	856672	CYC7; YEL039C	metabolic process;transport	mitochondrion	metal ion binding	0.259	0.259	1.00
P38797	Protein phosphatase 2C homolog 7, mitochondrial [OS=Saccharomyces cerevisiae S288c]	856475	PTC7; YHR076W	metabolic process	mitochondrion	catalytic activity;metal ion binding;protein binding	0.11	0.11	1.00
P36064	COX assembly mitochondrial protein [OS=Saccharomyces cerevisiae S288c]	853721	CMC1; YKL137W	cell organization and biogenesis	membrane;mitochondrion	metal ion binding	0.389	0.389	1.00
Q02863	ubiquitin carboxyl-terminal hydrolase 16 [OS=Saccharomyces cerevisiae S288c]	856032	UBP16; YPL072W	metabolic process	membrane;mitochondrion	catalytic activity	0.072	0.072	1.00
Q3E776	Uncharacterized protein YBR255C-A [OS=Saccharomyces cerevisiae S288c]	852558	YBR255C-A; YBR255C-A		membrane		0.389	0.389	1.00
P39103	Cytochrome c oxidase assembly protein COX14 [OS=Saccharomyces cerevisiae S288c]	854910	COX14; YML129C	cell organization and biogenesis;regulation of biological process	membrane;mitochondrion;organelle lumen	protein binding	0.585	0.585	1.00
P12687	54S ribosomal protein L2, mitochondrial [OS=Saccharomyces cerevisiae S288c]	855727	MRP7; YNL005C	cell organization and biogenesis;metabolic process	mitochondrion;ribosome	catalytic activity;RNA binding;structural molecule activity	0.11	0.11	1.00
P36521	54S ribosomal protein L11, mitochondrial [OS=Saccharomyces cerevisiae S288c]	851325	MRPL11; YDL202W	cell organization and biogenesis;metabolic process	mitochondrion;ribosome	structural molecule activity	0.129	0.129	1.00
P08456	CDP-diacylglycerol--serine O-phosphatidyltransferase [OS=Saccharomyces cerevisiae S288c]	856748	CHO1; YER026C	metabolic process	endoplasmic reticulum;membrane;mitochondrion	catalytic activity;metal ion binding;nucleotide binding	0.212	0.212	1.00
P38120	37S ribosomal protein S9, mitochondrial [OS=Saccharomyces cerevisiae S288c]	852443	MRPS9; YBR146W	cell organization and biogenesis;metabolic process	mitochondrion;ribosome	RNA binding;structural molecule activity	0.145	0.145	1.00
P07259	Protein URA2 [OS=Saccharomyces cerevisiae S288c]	853311	URA2; YJL130	metabolic process;regulation of biological process	cytoplasm;mitochondrion	catalytic activity;metal ion	0.016	0.016	1.00

			C			binding;nucleotide binding			
P07251	ATP synthase subunit alpha, mitochondrial [OS=Saccharomyces cerevisiae S288c]	852177	ATP1; YBL099W	metabolic process;transport	cytosol;membrane;mitochondrion	catalytic activity;nucleotide binding;transporter activity	20.544	21.967	1.07
P40341	Mitochondrial respiratory chain complexes assembly protein YTA12 [OS=Saccharomyces cerevisiae S288c]	855114	YTA12; YMR089C	cell organization and biogenesis;metabolic process;response to stimulus;transport	cytoplasm;membrane;mitochondrion	catalytic activity;metal ion binding;nucleotide binding;protein binding	1.276	1.371	1.07
P40215	External NADH-ubiquinone oxidoreductase 1, mitochondrial [OS=Saccharomyces cerevisiae S288c]	855176	NDE1; YMR145C	metabolic process	mitochondrion	catalytic activity;nucleotide binding	3.739	4.043	1.08
P32454	Aminopeptidase 2, mitochondrial [OS=Saccharomyces cerevisiae S288c]	853699	APE2; YKL157W	metabolic process	cytoplasm;extracellular;membrane;mitochondrion	catalytic activity;metal ion binding	0.696	0.762	1.09
P07256	Cytochrome b-c1 complex subunit 1, mitochondrial [OS=Saccharomyces cerevisiae S288c]	852235	COR1; YBL045C	metabolic process;transport	membrane;mitochondrion	catalytic activity;metal ion binding;protein binding;transporter activity	7.432	8.183	1.10
P02992	elongation factor Tu, mitochondrial [OS=Saccharomyces cerevisiae S288c]	854359	TUF1; YOR187W	cell organization and biogenesis;metabolic process	mitochondrion	catalytic activity;nucleotide binding;RNA binding	4.38	4.878	1.11
Q12166	2-isopropylmalate synthase 2, mitochondrial [OS=Saccharomyces cerevisiae S288c]	854275	LEU9; YOR108W	metabolic process	mitochondrion	catalytic activity;protein binding	1.246	1.39	1.12
P36013	NAD-dependent malic enzyme, mitochondrial [OS=Saccharomyces cerevisiae S288c]	853839	MAE1; YKL029C	metabolic process	mitochondrion;organelle lumen	catalytic activity;metal ion binding;nucleotide binding	0.995	1.113	1.12
P19262	Dihydrolipoyllysine-residue succinyltransferase component of 2-oxoglutarate dehydrogenase complex, mitochondrial [OS=Saccharomyces cerevisiae S288c]	851726	KGD2; YDR148C	cell organization and biogenesis;metabolic process	mitochondrion	catalytic activity;protein binding	3.062	3.489	1.14
P38891	Branched-chain-amino-acid aminotransferase, mitochondrial [OS=Saccharomyces cerevisiae S288c]	856615	BAT1; YHR208W	metabolic process;regulation of biological process;response to stimulus	mitochondrion;organelle lumen	catalytic activity	3.806	4.337	1.14
Q12482	Mitochondrial aspartate-glutamate transporter AGC1 [OS=Saccharomyces cerevisiae S288c]	856132	AGC1; YPR021C	metabolic process;transport	membrane;mitochondrion	structural molecule activity;transporter activity	0.407	0.468	1.15
Q08023	Protein FMP25, mitochondrial [OS=Saccharomyces cerevisiae S288c]	850766	FMP25; YLR077W	cell organization and biogenesis	membrane;mitochondrion		0.84	0.968	1.15
P09624	Dihydrolipoyl dehydrogenase, mitochondrial [OS=Saccharomyces cerevisiae S288c]	850527	LPD1; YFL018C	cellular homeostasis;metabolic process;regulation of biological process	mitochondrion;organelle lumen	catalytic activity;nucleotide binding	13.03	15.065	1.16
Q04013	Citrate/oxoglutarate carrier protein [OS=Saccharomyces cerevisiae S288c]	855282	YHM2; YMR241W	cell organization and biogenesis;metabolic process;transport	membrane;mitochondrion	DNA binding;structural molecule activity;transporter activity	2.981	3.467	1.16
Q07349	MIOREX complex component 9 [OS=Saccharomyces cerevisiae S288c]	851535	YDL027C; MRX9; YDL027C		endoplasmic reticulum;membrane;mitochondrion		1.096	1.276	1.16
P40053	Altered inheritance of mitochondria protein 9, mitochondrial [OS=Saccharomyces cerevisiae S288c]	856813	AIM9; YER080W		mitochondrion		1.537	1.798	1.17
P16451	Pyruvate dehydrogenase complex protein X component, mitochondrial [OS=Saccharomyces cerevisiae S288c]	853107	PDX1; YGR193C	metabolic process	mitochondrion;organelle lumen	catalytic activity;structural molecule activity	0.978	1.154	1.18
P36112	MICOS complex subunit Mic60 [OS=Saccharomyces cerevisiae S288c]	853886	FCJ1; MIC60; YKR016W	cell organization and biogenesis;transport	membrane;mitochondrion		11.798	14.086	1.19
P25573	Mitochondrial inner membrane i-AAA protease supercomplex subunit MGR1 [OS=Saccharomyces cerevisiae S288c]	850313	MGR1; YCL044C	metabolic process	membrane;mitochondrion	protein binding	0.859	1.031	1.20
Q07651	SUR7 family protein FMP45 [OS=Saccharomyces cerevisiae S288c]	851304	FMP45; YDL222C	cell differentiation;cell organization and biogenesis	membrane;mitochondrion		6.017	7.377	1.23
P08417	fumarate hydratase, mitochondrial [OS=Saccharomyces cerevisiae S288c]	855866	FUM1; YPL262W	cell organization and biogenesis;metabolic process	cytoplasm;cytosol;mitochondrion;organelle lumen	catalytic activity	3.175	3.894	1.23
Q12374	Nuclear control of ATPase protein 2 [OS=Saccharomyces cerevisiae S288c]	856278	NCA2; YPR155C	metabolic process	membrane;mitochondrion		0.377	0.468	1.24
P52893	probable alanine aminotransferase, mitochondrial [OS=Saccharomyces cerevisiae S288c]	850778	ALT1; YLR089C	metabolic process	mitochondrion;organelle lumen	catalytic activity	0.45	0.562	1.25
P39925	Mitochondrial respiratory chain complexes assembly protein AFG3 [OS=Saccharomyces cerevisiae S288c]	856737	AFG3; YER017C	cell organization and biogenesis;metabolic process;response to stimulus;transport	membrane;mitochondrion	catalytic activity;metal ion binding;nucleotide binding;protein	0.734	0.917	1.25

						binding			
P32787	Mitochondrial genome maintenance protein MGM101 [OS=Saccharomyces cerevisiae S288c]	853609	MGM101; YJR144W	cell organization and biogenesis;metabolic process;response to stimulus	chromosome;mitochondrion	DNA binding	1.069	1.336	1.25
P33311	ATP-dependent permease MDL2, mitochondrial [OS=Saccharomyces cerevisiae S288c]	855858	MDL2; YPL270W	response to stimulus;transport	membrane;mitochondrion	catalytic activity;nucleotide binding;transporter activity	0.619	0.802	1.30
P36066	Protein MRG3-like [OS=Saccharomyces cerevisiae S288c]	853725	YKL133C; YKL133C	metabolic process	membrane;mitochondrion	protein binding	0.334	0.433	1.30
Q02776	Mitochondrial import inner membrane translocase subunit TIM50 [OS=Saccharomyces cerevisiae S288c]	856042	TIM50; YPL063W	cell organization and biogenesis;metabolic process;transport	membrane;mitochondrion	catalytic activity;protein binding;transporter activity	9.857	12.895	1.31
Q01852	Mitochondrial import inner membrane translocase subunit TIM44 [OS=Saccharomyces cerevisiae S288c]	854790	TIM44; YIL022W	transport	membrane;mitochondrion	nucleotide binding;protein binding	4.125	5.404	1.31
Q06678	54S ribosomal protein L35, mitochondrial [OS=Saccharomyces cerevisiae S288c]	851921	MRPL35; YDR322W	cell organization and biogenesis;metabolic process	mitochondrion;ribosome	structural molecule activity	0.425	0.557	1.31
P07257	Cytochrome b-c1 complex subunit 2, mitochondrial [OS=Saccharomyces cerevisiae S288c]	856321	QCR2; YPR191W	metabolic process;transport	membrane;mitochondrion	catalytic activity;metal ion binding;protein binding;transporter activity	21.19	27.943	1.32
P07806	Valine--tRNA ligase, mitochondrial [OS=Saccharomyces cerevisiae S288c]	852986	VAS1; YGR094W	metabolic process;regulation of biological process	cytoplasm;cytosol;mitochondrion	catalytic activity;nucleotide binding	0.251	0.334	1.33
P00927	Threonine dehydratase, mitochondrial [OS=Saccharomyces cerevisiae S288c]	856819	ILV1; YER086W	metabolic process	cytoplasm;mitochondrion	catalytic activity	0.604	0.805	1.33
P33416	Heat shock protein 78, mitochondrial [OS=Saccharomyces cerevisiae S288c]	851845	HSP78; YDR258C	cell organization and biogenesis;metabolic process;regulation of biological process;response to stimulus	mitochondrion;organelle lumen	catalytic activity;nucleotide binding;protein binding	1.569	2.103	1.34
P53266	Cytochrome oxidase assembly protein SHY1 [OS=Saccharomyces cerevisiae S288c]	853009	SHY1; YGR112W	cell organization and biogenesis	membrane;mitochondrion	protein binding	1.154	1.555	1.35
P00424	Cytochrome c oxidase polypeptide 5A, mitochondrial [OS=Saccharomyces cerevisiae S288c]	855675	COX5A; YNL052W	metabolic process;transport	membrane;mitochondrion	catalytic activity;transporter activity	1.848	2.511	1.36
Q00402	Nuclear migration protein NUM1 [OS=Saccharomyces cerevisiae S288c]	851727	NUM1; YDR150W	cell organization and biogenesis;cellular component movement;metabolic process;regulation of biological process;transport	endoplasmic reticulum;mitochondrion	catalytic activity;motor activity;protein binding	0.078	0.106	1.36
P34227	Mitochondrial peroxiredoxin PRX1 [OS=Saccharomyces cerevisiae S288c]	852215	PRX1; YBL064C	cellular homeostasis;metabolic process;regulation of biological process;response to stimulus	mitochondrion	antioxidant activity;catalytic activity	2.793	3.833	1.37
Q04472	Mitochondrial inner membrane i-AAA protease supercomplex subunit MGR3 [OS=Saccharomyces cerevisiae S288c]	855142	MGR3; YMR115W	metabolic process	membrane;mitochondrion	protein binding	0.233	0.322	1.38
P00044	Cytochrome c iso-1 [OS=Saccharomyces cerevisiae S288c]	853507	CYC1; YJR048W	metabolic process;transport	mitochondrion	metal ion binding;protein binding	4.623	6.499	1.41
P39525	3-oxoacyl-[acyl-carrier-protein] synthase homolog [OS=Saccharomyces cerevisiae S288c]	856790	CEM1; YER061C	metabolic process	mitochondrion	catalytic activity	0.369	0.52	1.41
P50110	Sorting assembly machinery 37 kDa subunit [OS=Saccharomyces cerevisiae S288c]	855082	SAM37; YMR060C	cell organization and biogenesis;transport	membrane;mitochondrion	protein binding	0.389	0.551	1.42
P48569	Uncharacterized protein YDL183C [OS=Saccharomyces cerevisiae S288c]	851345	YDL183C; YDL183C	transport	membrane;mitochondrion		0.438	0.624	1.42
P16603	NADPH--cytochrome P450 reductase [OS=Saccharomyces cerevisiae S288c]	856438	NCP1; YHR042W	metabolic process	cytoplasm;cytosol;endoplasmic reticulum;membrane;mitochondrion	catalytic activity;metal ion binding;nucleotide binding	0.805	1.154	1.43
Q12029	Probable mitochondrial transport protein fsf1 [OS=Saccharomyces cerevisiae S288c]	854445	FSF1; YOR271C	transport	membrane;mitochondrion	transporter activity	1.336	1.976	1.48
P42940	Probable electron transfer flavoprotein subunit beta [OS=Saccharomyces cerevisiae S288c]	853121	CIR1; YGR207C	metabolic process;transport	mitochondrion;organelle lumen		2.162	3.217	1.49
P50085	Prohibitin-2 [OS=Saccharomyces cerevisiae S288c]	853146	PHB2; YGR231C	cell organization and biogenesis;metabolic process;regulation of biological process	membrane;mitochondrion	protein binding	2.899	4.337	1.50
Q06668	Methyltransferase OMS1, mitochondrial [OS=Saccharomyces cerevisiae S288c]	851911	OMS1; YDR316W	metabolic process	membrane;mitochondrion	catalytic activity	0.389	0.585	1.50

P21801	Succinate dehydrogenase [ubiquinone] iron-sulfur subunit, mitochondrial [OS=Saccharomyces cerevisiae S288c]	850685	SDH2; YLL041C	metabolic process;transport	membrane;mitochondrion	catalytic activity;metal ion binding	16.191	24.809	1.53
P42900	Sigma-like sequence protein 1, mitochondrial [OS=Saccharomyces cerevisiae S288c]	850830	SLS1; YLR139C	cell organization and biogenesis;metabolic process	membrane;mitochondrion	protein binding	0.125	0.194	1.55
Q04728	Arginine biosynthesis bifunctional protein ArgJ, mitochondrial [OS=Saccharomyces cerevisiae S288c]	855084	ARG7; YMR062C	metabolic process	mitochondrion;organelle lumen	catalytic activity	0.15	0.233	1.55
P07236	Threonine--tRNA ligase, mitochondrial [OS=Saccharomyces cerevisiae S288c]	853640	MST1; YKL194C	metabolic process	cytoplasm;mitochondrion;organelle lumen	catalytic activity;nucleotide binding;RNA binding	0.15	0.233	1.55
P41921	glutathione reductase [OS=Saccharomyces cerevisiae S288c]	856014	GLR1; YPL091W	cellular homeostasis;metabolic process;regulation of biological process;response to stimulus	cytoplasm;cytosol;mitochondrion	antioxidant activity;catalytic activity;nucleotide binding	0.155	0.241	1.55
P54115	Magnesium-activated aldehyde dehydrogenase, cytosolic [OS=Saccharomyces cerevisiae S288c]	856044	ALD6; YPL061W	metabolic process;response to stimulus	cytoplasm;cytosol;mitochondrion	catalytic activity	0.16	0.25	1.56
P05626	ATP synthase subunit 4, mitochondrial [OS=Saccharomyces cerevisiae S288c]	856027	ATP4; YPL078C	cell organization and biogenesis;metabolic process;transport	membrane;mitochondrion	catalytic activity;transporter activity	4.275	6.743	1.58
P32792	UPF0744 protein YSC83 [OS=Saccharomyces cerevisiae S288c]	856410	YSC83; YHR017W		membrane;mitochondrion		0.233	0.369	1.58
P12686	37S ribosomal protein MRP13, mitochondrial [OS=Saccharomyces cerevisiae S288c]	852975	MRP13; YGR084C	cell organization and biogenesis;metabolic process	mitochondrion;ribosome	structural molecule activity	0.259	0.413	1.59
P53889	uncharacterized mitochondrial hydrolase FMP41 [OS=Saccharomyces cerevisiae S288c]	855553	FMP41; YNL168C	metabolic process	mitochondrion	catalytic activity;metal ion binding	0.311	0.501	1.61
P38172	MIOREX complex component 3 [OS=Saccharomyces cerevisiae S288c]	852181	YBL095W; MRX3; YBL095W		membrane;mitochondrion		0.311	0.501	1.61
P53220	Mitochondrial import inner membrane translocase subunit TIM21 [OS=Saccharomyces cerevisiae S288c]	852921	TIM21; YGR033C	cell organization and biogenesis;transport	membrane;mitochondrion	protein binding	0.311	0.501	1.61
Q12375	mitochondrial ornithine transporter 1 [OS=Saccharomyces cerevisiae S288c]	854297	ORT1; YOR130C	metabolic process;transport	membrane;mitochondrion	structural molecule activity;transporter activity	0.334	0.54	1.62
P16622	Ferrochelatase, mitochondrial [OS=Saccharomyces cerevisiae S288c]	854347	HEM15; YOR176W	metabolic process	membrane;mitochondrion	catalytic activity	4.926	8.006	1.63
P32939	GTP-binding protein ypt7 [OS=Saccharomyces cerevisiae S288c]	855012	YPT7; YML001W	cell communication;cell organization and biogenesis;metabolic process;regulation of biological process;response to stimulus;transport	cytosol;endosome;membrane;mitochondrion;vacuole	catalytic activity;nucleotide binding;protein binding	0.359	0.585	1.63
P32340	Rotenone-insensitive NADH-ubiquinone oxidoreductase, mitochondrial [OS=Saccharomyces cerevisiae S288c]	854919	NDI1; YML120C	metabolic process;regulation of biological process	membrane;mitochondrion;organelle lumen	catalytic activity;nucleotide binding	10.721	17.874	1.67
P32335	Protein MSS51, mitochondrial [OS=Saccharomyces cerevisiae S288c]	850900	MSS51; YLR203C	metabolic process;regulation of biological process	membrane;mitochondrion	protein binding;translation regulator activity	1.154	1.929	1.67
Q06143	mitochondrial dicarboxylate transporter [OS=Saccharomyces cerevisiae S288c]	851063	DIC1; YLR348C	metabolic process;transport	membrane;mitochondrion	structural molecule activity;transporter activity	0.551	0.931	1.69
P18239	ADP,ATP carrier protein 2 [OS=Saccharomyces cerevisiae S288c]	852250	PET9; YBL030C	cell death;metabolic process;transport	membrane;mitochondrion	structural molecule activity;transporter activity	89.063	150.991	1.70
Q03713	Respiratory supercomplex factor 1, mitochondrial [OS=Saccharomyces cerevisiae S288c]	854978	RCF1; YML030W	cell organization and biogenesis	membrane;mitochondrion		0.585	0.995	1.70
P23644	Mitochondrial import receptor subunit TOM40 [OS=Saccharomyces cerevisiae S288c]	855243	TOM40; YMR203W	transport	cytosol;membrane;mitochondrion	protein binding;transporter activity	0.668	1.154	1.73
P32445	Single-stranded DNA-binding protein RIM1, mitochondrial [OS=Saccharomyces cerevisiae S288c]	850395	RIM1; YCR028C-A	cell organization and biogenesis;metabolic process;regulation of biological process	mitochondrion	DNA binding	0.668	1.154	1.73
P32843	Mitochondrial escape protein 2 [OS=Saccharomyces cerevisiae S288c]	855348	YME2; YMR302C	cell organization and biogenesis;metabolic process	membrane;mitochondrion	nucleotide binding;RNA binding	2.099	3.642	1.74
P53326	Uncharacterized protein YGR266W [OS=Saccharomyces cerevisiae S288c]	853182	YGR266W; YGR266W		membrane;mitochondrion		0.299	0.52	1.74
P38825	Protein TOM71 [OS=Saccharomyces cerevisiae S288c]	856517	TOM71; YHR117W	transport	membrane;mitochondrion	protein binding;transporter activity	0.343	0.604	1.76

Q12233	ATP synthase subunit g, mitochondrial [OS=Saccharomyces cerevisiae S288c]	856131	ATP20; YPR020W	cell organization and biogenesis;metabolic process;transport	membrane;mitochondrion	catalytic activity;transporter activity	0.778	1.371	1.76
P27697	Atypical kinase COQ8, mitochondrial [OS=Saccharomyces cerevisiae S288c]	852758	COQ8; YGL119W	metabolic process	membrane;mitochondrion;organelle lumen	catalytic activity;nucleotide binding	0.365	0.645	1.77
P37299	cytochrome b-c1 complex subunit 10 [OS=Saccharomyces cerevisiae S288c]	856390	QCR10; YHR001W-A	metabolic process;transport	membrane;mitochondrion	catalytic activity;transporter activity	2.981	5.31	1.78
P09457	ATP synthase subunit 5, mitochondrial [OS=Saccharomyces cerevisiae S288c]	851892	ATP5; YDR298C	metabolic process;transport	membrane;mitochondrion	catalytic activity;transporter activity	5.813	10.365	1.78
Q02883	N-acyl-phosphatidylethanolamine-hydrolyzing phospholipase D, mitochondrial [OS=Saccharomyces cerevisiae S288c]	856001	FMP30; YPL103C	metabolic process	membrane;mitochondrion	catalytic activity;metal ion binding	0.304	0.557	1.83
P32891	D-lactate dehydrogenase [cytochrome] 1, mitochondrial [OS=Saccharomyces cerevisiae S288c]	851380	DLD1; YDL174C	metabolic process;transport	membrane;mitochondrion	catalytic activity;nucleotide binding	5.661	10.45	1.85
P25087	Sterol 24-C-methyltransferase [OS=Saccharomyces cerevisiae S288c]	855003	ERG6; YML008C	metabolic process	endoplasmic reticulum;membrane;mitochondrion	catalytic activity	1.649	3.125	1.90
P21954	Isocitrate dehydrogenase [NADP], mitochondrial [OS=Saccharomyces cerevisiae S288c]	851493	IDP1; YDL066W	metabolic process	mitochondrion	catalytic activity;metal ion binding;nucleotide binding;protein binding	11.589	22.263	1.92
P38771	Ribosome-recycling factor, mitochondrial [OS=Saccharomyces cerevisiae S288c]	856433	RRF1; YHR038W	cell organization and biogenesis;metabolic process	mitochondrion	RNA binding	0.501	0.968	1.93
P40471	NADPH-dependent 1-acyldihydroxyacetone phosphate reductase [OS=Saccharomyces cerevisiae S288c]	854682	AYR1; YIL124W	metabolic process	cytoplasm;endoplasmic reticulum;membrane;mitochondrion	catalytic activity	0.778	1.512	1.94
P21375	Fumarate reductase 2 [OS=Saccharomyces cerevisiae S288c]	853510	OSM1; YJR051W	metabolic process	endoplasmic reticulum;mitochondrion	catalytic activity;nucleotide binding	0.346	0.682	1.97
P07253	cytochrome B pre-mRNA-processing protein 6 [OS=Saccharomyces cerevisiae S288c]	852417	CBP6; YBR120C	cell organization and biogenesis;metabolic process;regulation of biological process	mitochondrion;ribosome		0.638	1.276	2.00
P00128	Cytochrome b-c1 complex subunit 7 [OS=Saccharomyces cerevisiae S288c]	852142	QCR7; YDR529C	cell organization and biogenesis;metabolic process;transport	membrane;mitochondrion	catalytic activity;transporter activity	1.783	3.642	2.04
P41911	Glycerol-3-phosphate dehydrogenase [NAD(+)] 2, mitochondrial [OS=Saccharomyces cerevisiae S288c]	854095	GPD2; YOL059W	metabolic process	cytoplasm;cytosol;mitochondrion	catalytic activity;nucleotide binding;protein binding	0.089	0.186	2.09
P10662	37S ribosomal protein MRP1, mitochondrial [OS=Saccharomyces cerevisiae S288c]	851948	MRP1; YDR347W	cell organization and biogenesis;metabolic process;response to stimulus	mitochondrion;ribosome	antioxidant activity;catalytic activity;metal ion binding;structural molecule activity	0.585	1.239	2.12
Q02486	ARS-binding factor 2, mitochondrial [OS=Saccharomyces cerevisiae S288c]	855094	ABF2; YMR072W	cell organization and biogenesis;metabolic process;regulation of biological process	chromosome;mitochondrion;nucleus	DNA binding	10.938	23.245	2.13
P38297	Mitofusin FZO1 [OS=Saccharomyces cerevisiae S288c]	852477	FZO1; YBR179C	cell organization and biogenesis	membrane;mitochondrion	catalytic activity;nucleotide binding;protein binding	0.142	0.304	2.14
Q08926	ULP1-interacting protein 4 [OS=Saccharomyces cerevisiae S288c]	855916	UIP4; YPL186C		endoplasmic reticulum;membrane;mitochondrion;nucleus		0.136	0.292	2.15
P43603	LAS seventeen-binding protein 3 [OS=Saccharomyces cerevisiae S288c]	850580	LSB3; YFR024C-A	cell organization and biogenesis;regulation of biological process	cytoplasm;mitochondrion	protein binding	0.186	0.407	2.19
P38079	protein YRO2 [OS=Saccharomyces cerevisiae S288c]	852343	YRO2; YBR054W	transport	endoplasmic reticulum;membrane;mitochondrion	transporter activity	1.424	3.125	2.19
P40015	inositol phosphosphingolipids phospholipase C [OS=Saccharomyces cerevisiae S288c]	856739	ISC1; YER019W	metabolic process;response to stimulus	endoplasmic reticulum;membrane;mitochondrion	catalytic activity;metal ion binding	0.212	0.468	2.21
P38969	Pentamidine resistance factor, mitochondrial [OS=Saccharomyces cerevisiae S288c]	854440	PNT1; YOR266W	cell organization and biogenesis;response to stimulus	membrane;mitochondrion		0.259	0.585	2.26
P40502	Altered inheritance of mitochondria protein 19, mitochondrial [OS=Saccharomyces cerevisiae S288c]	854722	AIM19; YIL087C		membrane;mitochondrion		0.259	0.585	2.26
P28625	Protein YIM1 [OS=Saccharomyces cerevisiae S288c]	855183	YIM1; YMR152W	metabolic process;response to stimulus	cytoplasm;endoplasmic reticulum;mitochondrion	catalytic activity;metal ion binding	0.274	0.624	2.28
P50945	MICOS complex subunit MIC27 [OS=Saccharomyces cerevisiae S288c]	855623	AIM37; MIC27; YNL100W	cell organization and biogenesis	membrane;mitochondrion		0.311	0.719	2.31
Q07914	mitochondrial import inner membrane translocase subunit TIM14 [OS=Saccharomyces cerevisiae S288c]	850694	PAM18; YLR008C	regulation of biological process;transport	membrane;mitochondrion	enzyme regulator activity;protein binding;transporter activity	0.334	0.778	2.33

P21560	Protein CBP3, mitochondrial [OS=Saccharomyces cerevisiae S288c]	855886	CBP3; YPL215W	cell organization and biogenesis;regulation of biological process	membrane;mitochondrion;ribosome		0.701	1.649	2.35
Q03104	Meiotic sister chromatid recombination protein 1 [OS=Saccharomyces cerevisiae S288c]	854911	MSC1; YML128C	metabolic process	endoplasmic reticulum;membrane;mitochondrion		2.687	6.356	2.37
Q12349	ATP synthase subunit H, mitochondrial [OS=Saccharomyces cerevisiae S288c]	851002	ATP14; YLR295C	cell organization and biogenesis;metabolic process;transport	membrane;mitochondrion	catalytic activity;transporter activity	0.389	0.931	2.39
P32799	Cytochrome c oxidase subunit 6A, mitochondrial [OS=Saccharomyces cerevisiae S288c]	852684	COX13; YGL191W	cell organization and biogenesis;metabolic process;regulation of biological process;transport	membrane;mitochondrion	catalytic activity;enzyme regulator activity;transporter activity	2.162	5.31	2.46
Q12032	Altered inheritance of mitochondria protein 41, mitochondrial [OS=Saccharomyces cerevisiae S288c]	854390	AIM41; YOR215C		mitochondrion	catalytic activity	0.468	1.154	2.47
P53311	Mitochondrial pyruvate carrier 3 [OS=Saccharomyces cerevisiae S288c]	853158	FMP43; MPC3; YGR243W	transport	membrane;mitochondrion	transporter activity	0.468	1.154	2.47
P08525	Cytochrome b-c1 complex subunit 8 [OS=Saccharomyces cerevisiae S288c]	853273	QCR8; YJL166W	metabolic process;transport	membrane;mitochondrion	catalytic activity;transporter activity	0.585	1.512	2.58
Q02981	ABC1 family protein YPL109C, mitochondrial [OS=Saccharomyces cerevisiae S288c]	855995	YPL109C; YPL109C		mitochondrion		0.334	0.884	2.65
Q04935	Cytochrome c oxidase protein 20, mitochondrial [OS=Saccharomyces cerevisiae S288c]	851817	COX20; YDR231C	cell organization and biogenesis;metabolic process	membrane;mitochondrion	protein binding	0.701	1.894	2.70
P38323	ATP-dependent clpX-like chaperone, mitochondrial [OS=Saccharomyces cerevisiae S288c]	852528	MCX1; YBR227C	metabolic process	membrane;mitochondrion;organelle lumen	catalytic activity;nucleotide binding	0.145	0.403	2.78
P34231	Uncharacterized protein YKL187C [OS=Saccharomyces cerevisiae S288c]	853648	FAT3; YKL187C; YKL187C	cell organization and biogenesis;transport	membrane;mitochondrion		0.194	0.557	2.87
P19516	Cytochrome c oxidase assembly protein COX11, mitochondrial [OS=Saccharomyces cerevisiae S288c]	855971	COX11; YPL132W	cell organization and biogenesis;metabolic process	membrane;mitochondrion;ribosome	metal ion binding	0.274	0.833	3.04
Q01574	acetyl-coenzyme A synthetase 1 [OS=Saccharomyces cerevisiae S288c]	851245	ACS1; YAL054C	cell organization and biogenesis;metabolic process	cytoplasm;cytosol;endoplasmic reticulum;mitochondrion;nucleus	catalytic activity;nucleotide binding	2.57	7.859	3.06
Q12276	HMG2-induced ER-remodeling protein 1 [OS=Saccharomyces cerevisiae S288c]	854402	HER1; YOR227W	cell organization and biogenesis	cytoplasm;mitochondrion;ribosome	protein binding	0.032	0.098	3.06
P23833	Protein SCO1, mitochondrial [OS=Saccharomyces cerevisiae S288c]	852325	SCO1; YBR037C	cell organization and biogenesis;cellular homeostasis;response to stimulus;transport	membrane;mitochondrion	antioxidant activity;catalytic activity;metal ion binding	0.292	0.896	3.07
P38169	Kynurenine 3-monooxygenase [OS=Saccharomyces cerevisiae S288c]	852179	BNA4; YBL098W	metabolic process	membrane;mitochondrion	catalytic activity;nucleotide binding	0.083	0.269	3.24
P53982	isocitrate dehydrogenase [NADP] [OS=Saccharomyces cerevisiae S288c]	855723	IDP3; YNL009W	metabolic process	cytoplasm;mitochondrion	catalytic activity;metal ion binding;nucleotide binding	1.943	6.499	3.34
P40582	Glutathione S-transferase 1 [OS=Saccharomyces cerevisiae S288c]	854856	GTT1; YIR038C	metabolic process	endoplasmic reticulum;membrane;mitochondrion	antioxidant activity;catalytic activity;protein binding;RNA binding	0.638	2.162	3.39
P38885	Altered inheritance of mitochondria protein 46, mitochondrial [OS=Saccharomyces cerevisiae S288c]	856606	AIM46; YHR199C		mitochondrion	catalytic activity	0.245	0.931	3.80
P30902	ATP synthase subunit d, mitochondrial [OS=Saccharomyces cerevisiae S288c]	853853	ATP7; YKL016C	metabolic process;transport	membrane;mitochondrion	catalytic activity;transporter activity	2.728	10.788	3.95
P32897	Mitochondrial import inner membrane translocase subunit tim23 [OS=Saccharomyces cerevisiae S288c]	855751	TIM23; YNR017W	transport	membrane;mitochondrion	protein binding;transporter activity	0.995	4.012	4.03
Q06698	Putative ATP-dependent RNA helicase YLR419W [OS=Saccharomyces cerevisiae S288c]	851137	YLR419W; YLR419W	metabolic process	cytoplasm;mitochondrion;nucleus	catalytic activity;nucleotide binding;protein binding;RNA binding	0.054	0.233	4.31
P37267	Assembly factor cbp4 [OS=Saccharomyces cerevisiae S288c]		CBP4	cell organization and biogenesis	membrane;mitochondrion		0.52	2.511	4.83
P35180	mitochondrial import receptor subunit Tom20 [OS=Saccharomyces cerevisiae S288c]	852973	TOM20; YGR082W	cell organization and biogenesis;transport	membrane;mitochondrion	protein binding;transporter activity	0.194	1.031	5.31
Q03653	protein EFR3 [OS=Saccharomyces cerevisiae S288c]	855252	EFR3; YMR212C		membrane;mitochondrion		0.047	0.259	5.51
P17695	Glutaredoxin-2, mitochondrial [OS=Saccharomyces cerevisiae S288c]	852124	GRX2; YDR513W	cellular homeostasis;metabolic process;regulation of biological process;response to	cytoplasm;cytosol;mitochondrion	antioxidant activity;catalytic activity	0.233	1.31	5.62

				stimulus;transport					
P36139	protein PET10 [OS=Saccharomyces cerevisiae S288c]	853920	PET10; YKR046C	metabolic process	membrane		0.122	0.995	8.16

Supplemental Table S16. Proteins that are downregulated by the *ups2Δ* mutation (but not by the *ups1Δ* mutation) in cells cultured without LCA are involved in various biological processes. Mitochondria were purified from cells recovered on day 2, 4 or 7 of cell culturing without LCA. Mass spectrometry-based identification and quantitation of proteins recovered in purified mitochondria were performed as described in Materials and methods. The exponentially modified protein abundance index (emPAI) provided by the ″Proteome Discoverer″ software was used to calculate the relative abundance of mitochondrial proteins between *ups2Δ* and WT. Data on the relative concentrations of mitochondrial proteins are presented as means of 2 independent experiments.

Day	Protein	Ratio *ups2*/WT	Biological process
2	Mrpl35	0.07	Ribosome assembly (mitochondrial ribosomal protein)
	Imo32	0.08	Unknown
	Tim11	0.09	ATP synthesis (a subunit of mitochondrial F1F0-ATPase)
	Yhb1	0.09	Stress response (a flavohemoglobin that plays role in oxidative and nitrosative stress responses)
	Abf2	0.11	mtDNA replication/recombination/repair (mitochondrial DNA-binding protein)
	Qcr8	0.11	Electron transport chain/aerobic respiration (subunit 8 of ubiquinol cytochrome-c reductase [Complex III])
	Qcr7	0.11	Electron transport chain/aerobic respiration (subunit 7 of ubiquinol cytochrome-c reductase [Complex III])
	Rrf1	0.12	Protein synthesis (mitochondrial ribosome recycling factor)
	Idp1	0.12	Amino acid synthesis or degradation (mitochondrial NADP-specific isocitrate dehydrogenase)
	Gcv3	0.13	Metabolite synthesis (H subunit of the glycine decarboxylase complex required for folate synthesis)
	Gcv2	0.13	Metabolite synthesis (P subunit of the glycine decarboxylase complex required for folate synthesis)
	Mcx1	0.14	Protein import/proteostasis (non-proteolytic ATPase of the AAA family)
	Mcr1	0.14	Lipid metabolism/homeostasis (NADH-cytochrome b5 reductase involved in ergosterol biosynthesis)
	Mss116	0.14	mRNA synthesis/processing (transcription elongation factor required for splicing of introns)
	Phb1	0.15	Proteostasis (subunit of the prohibitin complex that stabilizes newly synthesized proteins)
	Mrpl4	0.15	Ribosome assembly (a mitochondrial ribosomal protein)
	Aco1	0.15	TCA cycle (aconitase)
	Idh1	0.15	TCA cycle (subunit of mitochondrial NAD(+)-dependent isocitrate dehydrogenase)
	Bat1	0.16	Amino acid synthesis or degradation (mitochondrial branched-chain amino acid (BCAA) aminotransferase)
	Cox4	0.16	Electron transport chain/aerobic respiration (subunit IV of cytochrome c oxidase)
	Sdh1	0.16	TCA cycle/Electron transport chain/aerobic respiration (flavoprotein subunit of succinate dehydrogenase)
	Psd1	0.17	Lipid metabolism/homeostasis (phosphatidylserine decarboxylase that converts PS to PE)
	Lsc2	0.17	TCA cycle (β subunit of succinyl-CoA ligase involved in conversion of succinyl-CoA to succinate)
	Ach1	0.17	Carbohydrate metabolism (catalyzes CoA-SH transfer from succinyl-CoA to acetate)
	Aim46	0.17	Unknown
	Cpr1	0.18	Proteostasis (catalyzes the cis-trans isomerization of peptide bonds N-terminal to proline)
	Rmd9	0.18	Aerobic respiration (mitochondrial protein required for respiratory growth)
	Mrp4	0.18	Ribosome assembly (mitochondrial ribosomal protein)
	Fum1	0.18	TCA cycle (fumarase)
	Mgm101	0.19	mtDNA replication/recombination/repair (protein involved in mitochondrial DNA recombinational repair)
	Agx1	0.19	Amino acid synthesis or degradation (alanine:glyoxylate aminotransferase; catalyzes the synthesis of glycine from glyoxylate)
	YJL045W	0.19	Electron transport chain/aerobic respiration (minor succinate dehydrogenase isozyme; participates in oxidation of succinate and transfer of electrons to ubiquinone)
	Ssq1	0.19	Proteostasis (mitochondrial hsp70-type molecular chaperone; required for assembly of iron/sulfur clusters into proteins at a step after cluster synthesis)
	Leu4	0.19	Amino acid synthesis or degradation (2-isopropylmalate synthase; the main isozyme responsible for the first step in the leucine biosynthesis pathway)
	Cpr3	0.20	Proteostasis (catalyzes the cis-trans isomerization of peptide bonds N-terminal to proline)
	Mae1	0.21	Carbohydrate metabolism (mitochondrial malic enzyme; catalyzes the decarboxylation of malate to pyruvate, a key intermediate in gluconeogenesis and a precursor for synthesis of amino acids)
	Mam33	0.21	Protein synthesis (specific translational activator for the mitochondrial COX1 mRNA)
	Dld1	0.22	Carbohydrate metabolism (major D-lactate dehydrogenase oxidizing D-lactate to pyruvate)
	Ehd3	0.22	Carbohydrate metabolism (3-hydroxyisobutyryl-CoA hydrolase)
	Rsm24	0.22	Ribosome assembly (mitochondrial ribosomal protein)
	Adh3	0.23	Carbohydrate metabolism (mitochondrial alcohol dehydrogenase isozyme III)
	Sdh4	0.23	TCA cycle/Electron transport chain/aerobic respiration (membrane anchor subunit of succinate dehydrogenase)
	Pda1	0.23	Carbohydrate metabolism (E1 alpha subunit of the pyruvate dehydrogenase complex; catalyzes the direct oxidative decarboxylation of pyruvate to acetyl-CoA)
	Prx1	0.24	Redox homeostasis (mitochondrial peroxiredoxin with thioredoxin peroxidase activity)
	Alt1	0.24	Amino acid synthesis or degradation (alanine transaminase (glutamic pyruvic transaminase); involved in alanine biosynthesis and catabolism)
	Ndi1	0.25	Electron transport chain/aerobic respiration (NADH:ubiquinone oxidoreductase; transfers electrons from NADH to ubiquinone in respiratory chain)
	Cox6	0.25	Electron transport chain/aerobic respiration (Subunit VI of cytochrome c oxidase [Complex IV])
	Mrps12	0.25	Ribosome assembly (mitochondrial ribosomal protein)
	Mrpl11	0.26	Ribosome assembly (mitochondrial ribosomal protein)
	Aim19	0.26	Unknown
	Mba1	0.26	Protein synthesis (membrane-associated mitochondrial ribosome receptor; forms a complex with Mdm38p that may facilitate recruitment of mRNA-specific translational activators to ribosomes)
	Mrp1	0.26	Ribosome assembly (mitochondrial ribosomal protein)
	Yml6	0.26	Ribosome assembly (mitochondrial ribosomal protein)
	Fsf1	0.26	Unknown
	Gut2	0.27	Carbohydrate metabolism (E1 alpha subunit of the pyruvate dehydrogenase complex; catalyzes the direct oxidative decarboxylation of pyruvate to acetyl-CoA)
	Ald4	0.27	Carbohydrate metabolism (mitochondrial glycerol-3-phosphate dehydrogenase involved in glycerol degradation)
	Tom20	0.28	Protein import (component of the TOM (translocase of outer membrane) complex; responsible for recognition and initial import steps for all mitochondrially directed proteins)
	Sfc1	0.28	Metabolite transport (mitochondrial succinate-fumarate transporter; transports succinate into and fumarate out of the mitochondrion)
	Arg7	0.28	Amino acid synthesis or degradation (mitochondrial ornithine acetyltransferase; catalyzes the fifth step in arginine biosynthesis)
	Put2	0.28	Amino acid synthesis or degradation (delta-1-pyrroline-5-carboxylate dehydrogenase; mitochondrial protein involved in utilization of proline as sole nitrogen source)
	Cir1	0.28	Unknown
	Ypt7	0.28	Contact sites between mitochondria and vacuoles (Rab family GTPase)
	Cat5	0.29	Metabolite synthesis (is involved in ubiquinone synthesis)
	Vas1	0.29	Protein synthesis (valyl-tRNA synthetase that performs alanyl-tRNA aminoacylation by coupling valine to valyl-tRNA)
	Cor1	0.29	Electron transport chain/aerobic respiration (core subunit of the ubiquinol-cytochrome c reductase complex)
	Hsp10	0.30	Proteostasis (mitochondrial matrix co-chaperonin; inhibits the ATPase activity of Hsp60, a mitochondrial chaperonin)
	Om45	0.30	Unknown
	Kgd2	0.30	TCA cycle (dihydrolipoyl transsuccinylase; component of the mitochondrial α-ketoglutarate dehydrogenase complex, which catalyzes the oxidative decarboxylation of alpha-ketoglutarate to succinyl-
	Mrpl24	0.30	Ribosome assembly (mitochondrial ribosomal protein)
	Ald5	0.31	Carbohydrate metabolism (mitochondrial aldehyde dehydrogenase; involved in acetate formation)
	Bna4	0.31	Metabolite synthesis (kynurenine 3-monooxygenase; required for the de novo biosynthesis of NAD from tryptophan via kynurenine)
	Gcv1	0.31	Metabolite synthesis (T subunit of the glycine decarboxylase complex required for folate synthesis)
	Glr1	0.31	Redox homeostasis (glutathione oxidoreductase; converts oxidized glutathione to reduced glutathione)
	Mcp2	0.31	Lipid metabolism/homeostasis (integral membrane protein that localizes to the mitochondrial inner membrane; associates with mitochondrial ribosome)

	Nfs1	0.31	Protein synthesis (cysteine desulfurase; involved in thio-modification of mitochondrial and cytoplasmic tRNAs)
	Lpd1	0.32	Carbohydrate metabolism (dihydrolipoamide dehydrogenase; the lipoamide dehydrogenase component (E3) of the pyruvate dehydrogenase and 2-oxoglutarate dehydrogenase multi-enzyme complexe
	Mrx3	0.32	Unknown
	Nam9	0.32	Ribosome assembly (mitochondrial ribosomal protein)
	Mdh1	0.33	TCA cycle (mitochondrial malate dehydrogenase; catalyzes interconversion of malate and oxaloacetate)
	Coq11	0.33	Metabolite synthesis (is involved in ubiquinone synthesis)
	Atp7	0.33	ATP synthesis (a subunit of mitochondrial F1F0-ATPase)
	YFR045W	0.33	Unknown
	YGR266W	0.34	Unknown
	Ssc1	0.35	Protein import/proteostasis (Hsp70 family ATPase; involved in protein translocation and folding)
	Spg4	0.36	Unknown
	Atp15	0.36	ATP synthesis (a subunit of mitochondrial F1F0-ATPase)
	Sdh2	0.37	TCA cycle/Electron transport chain/aerobic respiration (iron-sulfur protein subunit of succinate dehydrogenase; couples the oxidation of succinate to the transfer of electrons to ubiquinone as part of t respiratory chain)
	YOR020W-A	0.37	Unknown
	Pdb1	0.37	Carbohydrate metabolism (E1 beta subunit of the pyruvate dehydrogenase complex)
	Shy1	0.37	Electron transport chain/aerobic respiration (mitochondrial inner membrane protein required for complex IV assembly)
	Cyc1	0.37	Electron transport chain/aerobic respiration (cytochrome c, isoform 1)
	Nca2	0.38	ATP synthesis (protein that regulates expression of Fo-F1 ATP synthase subunits)
	Kgd1	0.38	TCA cycle (subunit of the mitochondrial α-ketoglutarate dehydrogenase complex)
	Idh2	0.38	TCA cycle (subunit of mitochondrial NAD(+)-dependent isocitrate dehydrogenase)
	Mis1	0.38	Metabolite synthesis (mitochondrial C1-tetrahydrofolate synthase)
	Lys12	0.38	Amino acid synthesis or degradation (homo-isocitrate dehydrogenase; an NAD-linked mitochondrial enzyme required for the fourth step in the biosynthesis of lysine)
	Cyt1	0.38	Electron transport chain/aerobic respiration (Cytochrome c1)
	Mir1	0.39	Metabolite transport (mitochondrial phosphate carrier; imports inorganic phosphate into mitochondria)
	Hem15	0.39	Metabolite synthesis (Ferrochelatase involved in the biosynthesis of heme)
	Atp1	0.39	ATP synthesis (α subunit of the F1 sector of mitochondrial F1F0 ATP synthase)
	Spg1	0.40	Unknown
	Fmp41	0.40	Unknown
	Aim37	0.40	Contact sites/cristae maintenance (Component of the MICOS [Mitochondrial contact site and cristae organizing system] complex)
	Afg1	0.41	Protein import/proteostasis (non-proteolytic ATPase of the AAA family)
	Tom40	0.41	Protein import (component of the TOM (translocase of outer membrane) complex; responsible for recognition and initial import steps for all mitochondrially directed proteins)
	Yme1	0.41	Protein import/proteostasis (catalytic subunit of i-AAA protease complex)
	Pam18	0.43	Protein import/proteostasis (subunit of the PAM [Presequence translocase-associated motor] complex)
	Tim17	0.43	Protein import (essential component of the TIM23 complex; contributes to the architecture and function of the import channel)
	Lys4	0.43	Amino acid synthesis or degradation (homoaconitase; catalyzes the conversion of homocitrate to homoisocitrate, which is a step in the lysine biosynthesis pathway)
	Yta12	0.43	Protein import/proteostasis (mitochondrial inner membrane m-AAA protease component)
	Yhm2	0.44	Metabolite transport (citrate and oxoglutarate carrier protein; exports citrate from and imports oxoglutarate into the mitochondrion, causing net export of NADPH reducing equivalents)
	Fcj1	0.44	Contact sites/cristae maintenance (Component of the MICOS [Mitochondrial contact site and cristae organizing system] complex)
	Aim24	0.44	Contact sites/cristae maintenance (inner membrane protein that interacts with the MICOS [Mitochondrial contact site and cristae organizing system] complex and is required for its integrity)
	Mmf1	0.45	Amino acid synthesis or degradation (mitochondrial protein required for transamination of isoleucine)
	Oxa1	0.45	Protein import/proteostasis (mitochondrial inner membrane insertase; mediates the insertion of both mitochondrial- and nuclear-encoded proteins from the matrix into the inner membrane)
	Fmp10	0.45	Unknown
	She9	0.46	Contact sites/cristae maintenance (mitochondrial inner membrane protein required for the inner membrane organization and normal mitochondrial morphology)
	Mrpl8	0.46	Ribosome assembly (mitochondrial ribosomal protein)
	Afg3	0.46	Proteostasis (ATPase and metallopeptidase of the inner mitochondrial membrane; subunit of the m-AAA complex involved in assembly of mitochondrial membrane protein complexes, degradation of processing of proteins imported into mitochondria)
	Etr1	0.47	Lipid metabolism/homeostasis (2-enoyl thioester reductase; member of the medium chain dehydrogenase/reductase family; has a probable role in fatty acid synthesis)
	Mss51	0.47	Protein synthesis (specific translational activator for the mitochondrial COX1 mRNA)
	Ape2	0.48	Proteostasis (aminopeptidase yscII; may have a role in obtaining leucine from dipeptide substrates)
	Mcy1	0.48	Amino acid synthesis or degradation (putative cysteine synthase)
	Mrx9	0.49	Unknown
	Fmp52	0.49	Unknown
	Mrps35	0.49	Ribosome assembly (mitochondrial ribosomal protein)
	Cir2	0.49	Unknown
	Hsp60	0.49	Proteostasis (mitochondrial chaperonin; required for ATP-dependent folding of precursor polypeptides and complex assembly; prevents aggregation and mediates protein refolding after heat shock)
4	Aim13	0.05	Contact sites/cristae maintenance (Component of the MICOS [Mitochondrial contact site and cristae organizing system] complex)
	YNL200C	0.10	Metabolite synthesis (NADHX epimerase; catalyzes isomerization of (R)- and (S)-NADHX)
	Cox5A	0.21	Electron transport chain/aerobic respiration (subunit Va of cytochrome c oxidase)
	Aim32	0.21	Unknown
	Mmt2	0.22	Metabolite transport (putative metal transporter involved in mitochondrial iron accumulation)
	Mrps16	0.23	Ribosome assembly (mitochondrial ribosomal protein)
	Pet54	0.24	Protein synthesis (mitochondrial inner membrane protein; binds to the 5' UTR of the COX3 mRNA to activate its translation)
	Qcr6	0.24	Electron transport chain/aerobic respiration (subunit 6 of the ubiquinol cytochrome-c reductase complex)
	Rsm10	0.28	Ribosome assembly (mitochondrial ribosomal protein)
	Cpr3	0.28	Proteostasis (mitochondrial peptidyl-prolyl cis-trans isomerase (cyclophilin); catalyzes the cis-trans isomerization of peptide bonds N-terminal to proline residues)
	Agx1	0.28	Amino acid synthesis or degradation (alanine:glyoxylate aminotransferase (AGT); catalyzes the synthesis of glycine from glyoxylate)
	Cox20	0.29	Proteostasis (mitochondrial inner membrane protein; required for proteolytic processing of Cox2p and its assembly into cytochrome c oxidase)
	Mrps18	0.29	Ribosome assembly (mitochondrial ribosomal protein)
	Idp1	0.30	Amino acid synthesis or degradation (mitochondrial NADP-specific isocitrate dehydrogenase; catalyzes the oxidation of isocitrate to alpha-ketoglutarate; diverts alpha-ketoglutarate to glutamine synth
	Mrps9	0.30	Ribosome assembly (mitochondrial ribosomal protein)
	Hem1	0.31	Metabolite synthesis (5-aminolevulinate synthase; catalyzes the first step in the heme biosynthetic pathway)
	Mst1	0.31	Protein synthesis (mitochondrial threonyl-tRNA synthetase)
	Mrp20	0.32	Ribosome assembly (mitochondrial ribosomal protein)
	Odc2	0.32	Metabolite transport (mitochondrial inner membrane transporter; 2-oxodicarboxylate transporter, exports 2-oxoadipate and 2-oxoglutarate from the mitochondrial matrix to the cytosol)
	Mct1	0.33	Lipid metabolism/homeostasis (Predicted malonyl-CoA:ACP transferase; putative component of a type-II mitochondrial fatty acid synthase that produces intermediates for phospholipid remodeling)
	Ycp4	0.34	Unknown
	Fmp30	0.35	Lipid metabolism/homeostasis (is involved in maintaining normal cardiolipin levels)
	Gcv3	0.36	Metabolite synthesis (H subunit of the glycine decarboxylase complex required for folate synthesis)
	Cyc1	0.36	Electron transport chain/aerobic respiration (cytochrome c, isoform 1)
	Rfs1	0.37	Unknown
	YOR020W-A	0.37	Unknown
	Isd11	0.37	Metabolite synthesis (essential for the formation of the persulfide intermediate at the desulfurase active site during pyridoxal phosphate-dependent desulfuration of cysteine; required for mitochondrial
	Fum1	0.38	TCA cycle (fumarase)
	Nfs1	0.38	Protein synthesis (cysteine desulfurase; involved in thio-modification of mitochondrial and cytoplasmic tRNAs)
	Dld1	0.38	Carbohydrate metabolism (major D-lactate dehydrogenase oxidizing D-lactate to pyruvate)
	Mrpl24	0.39	Ribosome assembly (mitochondrial ribosomal protein)
	Ccp1	0.39	Redox homeostasis (mitochondrial cytochrome-c peroxidase; degrades reactive oxygen species in mitochondria, involved in the response to oxidative stress)
	Nat2	0.39	Unknown
	YJL045W	0.40	Electron transport chain/aerobic respiration (minor succinate dehydrogenase isozyme; participates in oxidation of succinate and transfer of electrons to ubiquinone)
	Acs1	0.40	Carbohydrate metabolism (Acetyl-coA synthetase isoform)

	Mmf1	0.41	Amino acid synthesis or degradation (mitochondrial protein required for transamination of isoleucine)
	Mrps8	0.41	Ribosome assembly (mitochondrial ribosomal protein)
	Ald4	0.41	Carbohydrate metabolism (mitochondrial glycerol-3-phosphate dehydrogenase involved in glycerol degradation)
	Cat2	0.42	Metabolite transport (carnitine acetyl-CoA transferase; transfers activated acetyl groups to carnitine to form acetylcarnitine which can be shuttled across membranes)
	Pam18	0.43	Protein import/proteostasis (subunit of the PAM [Presequence translocase-associated motor] complex)
	Atp4	0.43	ATP synthesis (subunit b of the F1 sector of mitochondrial F1F0 ATP synthase)
	Mrpl36	0.44	Ribosome assembly (mitochondrial ribosomal protein)
	Om14	0.44	Protein import (mitochondrial outer membrane protein that binds to the nascent chain-associated complex to dock cytosolic ribosomes at the mitochondrion and facilitate co-translational import of mit
	Afg1	0.44	Protein import/proteostasis (non-proteolytic ATPase of the AAA family)
	Mir1	0.45	Metabolite transport (mitochondrial phosphate carrier; imports inorganic phosphate into mitochondria)
	Mrpl49	0.45	Ribosome assembly (mitochondrial ribosomal protein)
	Isu1	0.45	Metabolite synthesis (performs a scaffolding function during assembly of iron-sulfur clusters)
	Pth2	0.46	Proteostasis (one of two mitochondrially-localized peptidyl-tRNA hydrolases; negatively regulates the ubiquitin-proteasome pathway via interactions with ubiquitin-like ubiquitin-associated proteins)
	Mrpl7	0.47	Ribosome assembly (mitochondrial ribosomal protein)
	Ssq1	0.47	Proteostasis (mitochondrial hsp70-type molecular chaperone; required for assembly of iron/sulfur clusters into proteins at a step after cluster synthesis)
	Pgk1	0.47	ATP synthesis (3-phosphoglycerate kinase; catalyzes transfer of high-energy phosphoryl groups from the acyl phosphate of 1,3-bisphosphoglycerate to ADP to produce ATP)
	Cyt1	0.47	Electron transport chain/aerobic respiration (Cytochrome c1)
	Erg9	0.47	Lipid metabolism/homeostasis (Farnesyl-diphosphate farnesyl transferase (squalene synthase); joins two farnesyl pyrophosphate moieties to form squalene in the sterol biosynthesis pathway)
	Qri7	0.48	Protein synthesis (highly conserved mitochondrial protein; essential for t6A modification of mitochondrial tRNAs that decode ANN codons)
	Sco2	0.48	Metabolite transport (is involved in delivery of copper to cytochrome c oxidase)
	Fmp43	0.48	Metabolite transport (highly conserved subunit of the mitochondrial pyruvate carrier)
	Afg3	0.48	Proteostasis (ATPase and metallopeptidase of the inner mitochondrial membrane; subunit of the m-AAA complex involved in assembly of mitochondrial membrane protein complexes, degradation of processing of proteins imported into mitochondria)
7	Mrp7	0.06	Ribosome assembly (mitochondrial ribosomal protein)
	Rsm28	0.11	Ribosome assembly (mitochondrial ribosomal protein)
	Ptc7	0.13	Metabolite synthesis (Type 2C serine/threonine protein phosphatase; activates coenzyme Q6 biosynthesis by dephosphorylation of demethoxy-Q6 hydroxylase Coq7p)
	Ilv6	0.13	Amino acid synthesis or degradation (regulatory subunit of acetolactate synthase; acetolactate synthase catalyzes the first step of branched-chain amino acid biosynthesis)
	Qcr7	0.15	Electron transport chain/aerobic respiration (subunit 7 of ubiquinol cytochrome-c reductase [Complex III])
	Mrpl11	0.15	Ribosome assembly (mitochondrial ribosomal protein)
	Cbp4	0.16	Proteostasis (mitochondrial protein required for assembly of cytochrome bc1 complex)
	Abf2	0.16	mtDNA replication/recombination/repair (mitochondrial DNA-binding protein)
	Mrpl10	0.16	Ribosome assembly (mitochondrial ribosomal protein)
	YNL208W	0.16	Unknown
	Ymr31	0.17	TCA cycle (subunit of the mitochondrial alpha-ketoglutarate dehydrogenase)
	Aim19	0.17	Unknown
	Mrpl27	0.17	Ribosome assembly (mitochondrial ribosomal protein)
	Yml6	0.17	Ribosome assembly (mitochondrial ribosomal protein)
	Mrpl8	0.20	Ribosome assembly (mitochondrial ribosomal protein)
	Mrps28	0.21	Ribosome assembly (mitochondrial ribosomal protein)
	Var1	0.21	Ribosome assembly (mitochondrial ribosomal protein)
	Yhb1	0.22	Stress response (a flavohemoglobin that plays role in oxidative and nitrosative stress responses)
	Mrpl22	0.23	Ribosome assembly (mitochondrial ribosomal protein)
	Ehd3	0.23	Carbohydrate metabolism (3-hydroxyisobutyryl-CoA hydrolase)
	Arg7	0.24	Amino acid synthesis or degradation (mitochondrial ornithine acetyltransferase; catalyzes the fifth step in arginine biosynthesis)
	Tim23	0.25	Protein import (essential component of the TIM23 complex; involved in protein import into mitochondrial matrix and inner membrane)
	Rrf1	0.26	Protein synthesis (mitochondrial ribosome recycling factor)
	Pet123	0.26	Ribosome assembly (mitochondrial ribosomal protein)
	Grx2	0.27	Redox homeostasis (glutaredoxin; thioltransferase, glutathione-dependent disulfide oxidoreductase involved in maintaining redox state of target proteins, also exhibits glutathione peroxidase activity)
	Ilv1	0.27	Amino acid synthesis or degradation (threonine deaminase, catalyzes first step in isoleucine biosynthesis)
	Nfu1	0.27	Proteostasis (protein involved in Fe-S cluster transfer to mitochondrial clients; protects [4Fe-4S] clusters from damage due to oxidative stress; acts along with Bol3 at a late step in the transfer of [4Fe- protein)
	Coq11	0.27	Metabolite synthesis (is involved in ubiquinone synthesis)
	Mam33	0.27	Protein synthesis (specific translational activator for the mitochondrial COX1 mRNA)
	Cox6	0.27	Electron transport chain/aerobic respiration (Subunit VI of cytochrome c oxidase [Complex IV])
	Sod1	0.28	Stress response (copper-zinc superoxide dismutase; detoxifies superoxide; phosphorylated by Dun1p, enters nucleus under oxidative stress to promote transcription of stress response genes)
	Gtt1	0.30	Redox homeostasis (glutathione S-transferase)
	Gpd2	0.30	Carbohydrate metabolism (NAD-dependent glycerol 3-phosphate dehydrogenase; is involved in glycerol biosynthesis)
	Bna4	0.31	Metabolite synthesis (kynurenine 3-monooxygenase; required for the de novo biosynthesis of NAD from tryptophan via kynurenine)
	Her2	0.31	Unknown
	Sdh4	0.32	TCA cycle/Electron transport chain/aerobic respiration (membrane anchor subunit of succinate dehydrogenase)
	Aim37	0.32	Contact sites/cristae maintenance (Component of the MICOS [Mitochondrial contact site and cristae organizing system] complex)
	Nam9	0.32	Ribosome assembly (mitochondrial ribosomal protein)
	Mgm101	0.33	mtDNA replication/recombination/repair (protein involved in mitochondrial DNA recombinational repair)
	Cox4	0.33	Electron transport chain/aerobic respiration (subunit IV of cytochrome c oxidase)
	YKR070W	0.33	Unknown
	Mss116	0.33	mRNA synthesis/processing (transcription elongation factor required for splicing of introns)
	Pos5	0.33	Metabolite synthesis (mitochondrial NADH kinase; phosphorylates NADH)
	Mrp13	0.33	Ribosome assembly (mitochondrial ribosomal protein)
	Mba1	0.33	Protein synthesis (membrane-associated mitochondrial ribosome receptor; forms a complex with Mdm38p that may facilitate recruitment of mRNA-specific translational activators to ribosomes)
	Cem1	0.34	Carbohydrate metabolism (mitochondrial beta-keto-acyl synthase; possible role in fatty acid synthesis)
	Mrpl35	0.35	Ribosome assembly (mitochondrial ribosomal protein)
	Mrp4	0.35	Ribosome assembly (mitochondrial ribosomal protein)
	Idp1	0.35	Amino acid synthesis or degradation (mitochondrial NADP-specific isocitrate dehydrogenase; catalyzes the oxidation of isocitrate to alpha-ketoglutarate; diverts alpha-ketoglutarate to glutamine synth
	Cpr3	0.35	Proteostasis (mitochondrial peptidyl-prolyl cis-trans isomerase (cyclophilin); catalyzes the cis-trans isomerization of peptide bonds N-terminal to proline residues)
	Nfs1	0.36	Protein synthesis (cysteine desulfurase; involved in thio-modification of mitochondrial and cytoplasmic tRNAs)
	Agx1	0.36	Amino acid synthesis or degradation (alanine:glyoxylate aminotransferase (AGT); catalyzes the synthesis of glycine from glyoxylate)
	Atp15	0.36	ATP synthesis (a subunit of mitochondrial F1F0-ATPase)
	Tuf1	0.36	Protein synthesis (Mitochondrial translation elongation factor Tu)
	Pnt1	0.36	Proteostasis (mitochondrial integral inner membrane protein; involved in membrane insertion of C-terminus of Cox2p)
	Cym1	0.37	Proteostasis (lysine-specific metalloprotease of the pitrilysin family; metalloprotease of the intermembrane space; degrades proteins and presequence peptides cleaved from imported proteins)
	Ifm1	0.37	Protein synthesis (mitochondrial translation initiation factor 2)
	Hsp78	0.37	Proteostasis (oligomeric mitochondrial matrix chaperone; able to prevent the aggregation of misfolded proteins as well as resolubilize protein aggregates)
	Rim1	0.37	mtDNA replication/recombination/repair (ssDNA-binding protein essential for mitochondrial genome maintenance; involved in mitochondrial DNA replication)
	Vas1	0.38	Protein synthesis (valyl-tRNA synthetase that performs alanyl-tRNA aminoacylation by coupling valine to valyl-tRNA)
	Fmp40	0.38	Unknown
	Aim9	0.38	Unknown
	YGR266W	0.38	Unknown
	Gcv3	0.38	Metabolite synthesis (H subunit of the glycine decarboxylase complex required for folate synthesis)
	Fum1	0.39	TCA cycle (fumarase)
	Rcf1	0.39	Proteostasis (cytochrome c oxidase subunit; required for assembly of the Complex III-Complex IV supercomplex, and for assembly of Cox13p and Rcf2p into cytochrome c oxidase)
	Spg1	0.39	Unknown

	Shm1	0.39	Metabolite synthesis (mitochondrial serine hydroxymethyltransferase; converts serine to glycine plus 5,10 methylenetetrahydrofolate; involved in generating precursors for purine, pyrimidine, amino a[illegible] generates serine)
	Ilv3	0.40	Metabolite synthesis (dihydroxyacid dehydratase; catalyzes third step in the common pathway leading to biosynthesis of branched-chain amino acids)
	Fmp43	0.41	Metabolite transport (highly conserved subunit of the mitochondrial pyruvate carrier)
	Mrps17	0.42	Ribosome assembly (mitochondrial ribosomal protein)
	Ccp1	0.43	Redox homeostasis (mitochondrial cytochrome-c peroxidase; degrades reactive oxygen species in mitochondria, involved in the response to oxidative stress)
	Pdx1	0.43	Carbohydrate metabolism (E3-binding protein of the mitochondrial pyruvate dehydrogenase complex)
	Mrpl13	0.43	Ribosome assembly (mitochondrial ribosomal protein)
	Pam18	0.43	Protein import/proteostasis (subunit of the PAM [Presequence translocase-associated motor] complex)
	Mmf1	0.43	Amino acid synthesis or degradation (mitochondrial protein required for transamination of isoleucine)
	Mrx3	0.43	Unknown
	Tim21	0.43	Protein import (component of the TIM23 complex; interacts with the Translocase of the Outer Mitochondrial membrane (TOM complex)
	Msd1	0.43	Protein synthesis (mitochondrial aspartyl-tRNA synthetase; required for acylation of aspartyl-tRNA)
	Atp7	0.44	ATP synthesis (a subunit of mitochondrial F1F0-ATPase)
	Dld2	0.44	Carbohydrate metabolism (D-2-hydroxyglutarate dehydrogenase, and minor D-lactate dehydrogenase; mitochondrial matrix protein that oxidizes D-2-hydroxyglutarate to α-ketoglutarate)
	Ctp1	0.44	Metabolite transport (mitochondrial inner membrane citrate transporter)
	Mrx9	0.45	Unknown
	Ysa1	0.46	Metabolite synthesis (nudix hydrolase family member with ADP-ribose pyrophosphatase activity; metabolizes O-acetyl-ADP-ribose to AMP and acetylated ribose 5'-phosphate)
	Fzo1	0.47	Mitochondrial fusion (mitofusin; integral membrane protein involved in mitochondrial outer membrane tethering and fusion)
	Ssq1	0.47	Proteostasis (mitochondrial hsp70-type molecular chaperone; required for assembly of iron/sulfur clusters into proteins at a step after cluster synthesis)
	YPR098C	0.47	Unknown
	Mrp1	0.47	Ribosome assembly (mitochondrial ribosomal protein)
	Alt1	0.47	Amino acid synthesis or degradation (alanine transaminase (glutamic pyruvic transaminase); involved in alanine biosynthesis and catabolism)
	Mdj1	0.47	Proteostasis (Co-chaperone that stimulates HSP70 protein Ssc1p ATPase activity; involved in protein folding/refolding in the mitochondrial matrix; required for proteolysis of misfolded proteins)
	Tef4	0.48	Protein synthesis (gamma subunit of translational elongation factor eEF1B)
	Fsf1	0.48	Unknown
	Mdm10	0.48	Metabolite transport (component of ERMES complex which acts as a molecular tether between the mitochondria and the ER, necessary for efficient phospholipid exchange between organelles and for[illegible]
	Sod2	0.48	Stress response (mitochondrial manganese superoxide dismutase; protects cells against oxygen toxicity and oxidative stress)
	Cox1	0.48	Electron transport chain/aerobic respiration (Subunit I of cytochrome c oxidase [Complex IV])
	Aim45	0.49	Unknown
	Msr1	0.49	Protein synthesis (mitochondrial arginyl-tRNA synthetase)
	Idh1	0.49	TCA cycle (subunit of mitochondrial NAD(+)-dependent isocitrate dehydrogenase)
	Tom71	0.49	Protein import (mitochondrial outer membrane protein; probable minor component of the TOM (translocase of outer membrane) complex responsible for recognition and import of mitochondrially di[illegible]
	Cit3	0.49	TCA cycle (dual specificity mitochondrial citrate and methylcitrate synthase; catalyzes the condensation of acetyl-CoA and oxaloacetate to form citrate)

Supplemental Table S17. Proteins that are upregulated by the *ups2Δ* mutation (but not by the *ups1Δ* mutation) in cells cultured without LCA are involved in various biological processes. Mitochondria were purified from cells recovered on day 2, 4 or 7 of cell culturing without LCA. Mass spectrometry-based identification and quantitation of proteins recovered in purified mitochondria were performed as described in Materials and methods. The exponentially modified protein abundance index (emPAI) provided by the ″Proteome Discoverer″ software was used to calculate the relative abundance of mitochondrial proteins between *ups2Δ* and WT. Data on the relative concentrations of mitochondrial proteins are presented as means of 2 independent experiments.

Day	Protein	Ratio *ups2*/WT	Biological process
2	Coq2	1.60	Metabolite synthesis (is involved in ubiquinone synthesis)
	Crc1	1.81	Metabolite transport (is required for carnitine-dependent transport of acetyl-CoA)
	Zeo1	1.86	Unknown
	Cyt2	2.16	Electron transport chain/aerobic respiration (is involved in maturation of cytochrome c1)
4	Pet9	1.52	Metabolite transport (major ADP/ATP carrier of the mitochondrial inner membrane; exchanges cytosolic ADP for mitochondrially synthesized ATP; also imports heme and ATP)
	Mne1	1.55	mRNA synthesis/processing (protein involved in splicing Group I aI5-beta intron from COX1 mRNA)
	Msm1	1.56	Protein synthesis (mitochondrial methionyl-tRNA synthetase)
	Rcf2	1.61	Electron transport chain/respiration (cytochrome c oxidase subunit; has a role in assembly of respiratory supercomplexes)
	Fba1	1.62	Carbohydrate metabolism (Fructose 1,6-bisphosphate aldolase; required for glycolysis and gluconeogenesis; catalyzes conversion of fructose 1,6 bisphosphate to glyceraldehyde-3-P and dihydroxyacetone-P; l oxidative stress)
	Mrpl27	1.70	Ribosome assembly (mitochondrial ribosomal protein)
	Rsm18	1.70	Ribosome assembly (mitochondrial ribosomal protein)
	YPL109C	1.75	Unknown
	Nam9	1.76	Ribosome assembly (mitochondrial ribosomal protein)
	Ncp1	1.76	Lipid metabolism (NADP-cytochrome P450 reductase; involved in ergosterol biosynthesis)
	Osm1	1.80	Metabolite synthesis (fumarate reductase with a role in oxidation of FAD)
	Mrp7	1.85	Ribosome assembly (mitochondrial ribosomal protein)
	Ccm1	2.02	mRNA synthesis/processing (mitochondrial 15S rRNA-binding protein; required for intron removal of COB and COX1 pre-mRNAs)
	Aat1	2.07	Amino acid metabolism (mitochondrial aspartate aminotransferase; catalyzes the conversion of oxaloacetate to aspartate in aspartate and asparagine biosynthesis)
	Gpd2	2.09	Carbohydrate metabolism (NAD-dependent glycerol 3-phosphate dehydrogenase; is involved in glycerol metabolism)
	Rsm23	2.10	Ribosome assembly (mitochondrial ribosomal protein)
	Nit3	2.12	Unknown
	Pgi1	2.16	Carbohydrate metabolism (phosphoglucose isomerase; catalyzes the interconversion of glucose-6-phosphate and fructose-6-phosphate in glycolysis and gluconeogenesis)
	Mrh4	2.23	Ribosome assembly (mitochondrial ATP-dependent RNA helicase of the DEAD-box family; required for assembly of the large subunit of mitochondrial ribosomes)
	Aep2	2.29	Protein synthesis (is involved in translation of the mitochondrial OLI1 mRNA)
	Msw1	2.29	Protein synthesis (mitochondrial tryptophanyl-tRNA synthetase)
	Rsm28	2.39	Ribosome assembly (mitochondrial ribosomal protein)
	Mrpl10	2.55	Ribosome assembly (mitochondrial ribosomal protein)
	Efr3	2.70	Unknown
	Oxa1	2.82	Protein import/proteostasis (mitochondrial inner membrane insertase; mediates the insertion of both mitochondrial- and nuclear-encoded proteins from the matrix into the inner membrane; also has a role in ins membrane)
	Bna4	2.83	Metabolite synthesis (kynurenine 3-monooxygenase; required for the de novo biosynthesis of NAD from tryptophan via kynurenine)
	Tcb3	2.85	Unknown
	Mrpl4	2.93	Ribosome assembly (mitochondrial ribosomal protein)
	Ifm1	3.35	Protein synthesis (Mitochondrial translation initiation factor 2)
	Mrh1	3.36	Unknown
	Pgs1	4.51	Lipid metabolism (Phosphatidylglycerolphosphate synthase; catalyzes the synthesis of phosphatidylglycerolphosphate from CDP-diacylglycerol and sn-glycerol 3-phosphate in the first committed and rate-lim
7	Ncp1	1.57	Lipid metabolism (NADP-cytochrome P450 reductase; involved in ergosterol biosynthesis)
	Tsc13	1.61	Lipid metabolism (enoyl reductase; catalyzes last step in each cycle of very long chain fatty acid elongation)
	Aep2	1.63	Protein synthesis (is involved in translation of the mitochondrial OLI1 mRNA)
	Eis1	1.66	Unknown
	Ggc1	1.72	Metabolite transport (mitochondrial GTP/GDP transporter)
	Hsp26	1.85	Proteostasis (small heat shock protein with chaperone activity; forms hollow, sphere-shaped oligomers that suppress unfolded proteins aggregation)
	Ugo1	1.85	Mitochondrial fusion (outer membrane component of the mitochondrial fusion machinery)
	Ady2	2.03	Metabolite transport (acetate transporter)
	Fat3	2.09	Metabolite transport (protein required for fatty acid uptake)
	Put1	2.09	Amino acid metabolism (proline oxidase; nuclear-encoded mitochondrial protein involved in utilization of proline as sole nitrogen source)
	Tgl2	2.23	Lipid metabolism (Triacylglycerol lipase that is localized to the mitochondria)
	Sam37	3.35	Proteostasis (component of the Sorting and Assembly Machinery (SAM) complex; the SAM complex is located in the mitochondrial outer membrane; binds precursors of beta-barrel proteins and facilitates the SAM complex stability)
	Acc1	8.47	Lipid metabolism (acetyl-CoA carboxylase, biotin containing enzyme; catalyzes carboxylation of acetyl-CoA to form malonyl-CoA; required for de novo biosynthesis of long-chain fatty acids)

Supplemental Table S18. Proteins that are downregulated by LCA only in *ups2Δ* cells (but not in WT or *ups1Δ* cells) are involved in several biological processes. Mitochondria were purified from cells recovered on day 2, 4 or 7 of cell culturing with LCA. Mass spectrometry-based identification and quantitation of proteins recovered in purified mitochondria were performed as described in Materials and methods. The exponentially modified protein abundance index (emPAI) provided by the ″Proteome Discoverer″ software was used to calculate the relative abundance of mitochondrial proteins between *ups2Δ* cells cultured with or without LCA. Data on the relative concentrations of mitochondrial proteins are presented as means of 2 independent experiments.

**Day**	**Protein**	**Ratio *ups2* + LCA/*ups2***	**Biological process**
2	Mrps28	0.47	Ribosome assembly (mitochondrial ribosomal protein)
	Ptc7	0.47	Metabolite synthesis (type 2C serine/threonine protein phosphatase; activates coenzyme Q6 biosynthesis by dephosphorylation of demethoxy-Q6 hydroxylase Coq7p)
	Mst1	0.48	Protein synthesis (mitochondrial threonyl-tRNA synthetase)
	Oac1	0.49	Metabolite transport (mitochondrial inner membrane transporter; transports oxaloacetate, sulfate, thiosulfate, and isopropylmalate)
4	Pgs1	0.22	Lipid metabolism (phosphatidylglycerolphosphate synthase; catalyzes the synthesis of phosphatidylglycerolphosphate from CDP-diacylglycerol and sn-glycerol 3-phosphate in the first committed and rate-l
	Mrpl27	0.26	Ribosome assembly (mitochondrial ribosomal protein)
	Mne1	0.31	mRNA synthesis/processing (protein involved in splicing Group I aI5-beta intron from COX1 mRNA)
	Acs1	0.37	Carbohydrate metabolism (acetyl-coA synthetase isoform)
	Mrpl28	0.45	Ribosome assembly (mitochondrial ribosomal protein)
	Mrp7	0.45	Ribosome assembly (mitochondrial ribosomal protein)
	Mrps8	0.46	Ribosome assembly (mitochondrial ribosomal protein)
	Mhr1	0.46	Ribosome assembly (mitochondrial ribosomal protein)
	Ald6	0.46	Carbohydrate metabolism (aldehyde dehydrogenase; required for conversion of acetaldehyde to acetate; locates to the mitochondrial outer surface upon oxidative stress)
	Coq2	0.47	Metabolite synthesis (Para hydroxybenzoate polyprenyl transferase; catalyzes the second step in ubiquinone [coenzyme Q] biosynthesis)
	Nit3	0.47	Unknown
	Ylf2	0.48	Unknown
	Dia4	0.48	Protein synthesis (mitochondrial seryl-tRNA synthetase that performs seryl-tRNA aminoacylation in the mitochondria by coupling serine to seryl-tRNA)
	Rsm22	0.49	Ribosome assembly (mitochondrial ribosomal protein)
7	Mrpl6	0.43	Ribosome assembly (mitochondrial ribosomal protein)
	Pet123	0.44	Ribosome assembly (mitochondrial ribosomal protein)
	Tom22	0.44	Protein import (Component of the TOM [Translocase of Outer Membrane] complex; responsible for initial import of mitochondrially directed proteins)
	Mrps5	0.44	Ribosome assembly (mitochondrial ribosomal protein)
	Tgl2	0.45	Lipid metabolism (triacylglycerol lipase that is localized to the mitochondria)
	Fmp46	0.45	Unknown
	Rml2	0.46	Ribosome assembly (mitochondrial ribosomal protein)
	Bi4	0.48	mRNA synthesis/processing (mitochondrial mRNA maturase; forms a complex with Nam2p to mediate splicing of the bI4 intron of the COB gene)
	Kha1	0.48	Metabolite transport (putative K+/H+ antiporter)
	Ifm1	0.49	Protein synthesis (mitochondrial translation initiation factor 2)

Supplemental Table S19. Proteins that are upregulated by LCA only in *ups2Δ* cells (but not in WT or *ups1Δ* cells) are involved in various biological processes. Mitochondria were purified from cells recovered on day 2, 4 or 7 of cell culturing with LCA. Mass spectrometry-based identification and quantitation of proteins recovered in purified mitochondria were performed as described in Materials and methods. The exponentially modified protein abundance index (emPAI) provided by the ″Proteome Discoverer″ software was used to calculate the relative abundance of mitochondrial proteins between *ups2Δ* cells cultured with or without LCA. Data on the relative concentrations of mitochondrial proteins are presented as means of 2 independent experiments.

Day	Protein	Ratio *ups2* + LCA/ *ups2*	Biological process
2	Aac1	1.51	Metabolite transport (mitochondrial inner membrane ADP/ATP translocator; exchanges cytosolic ADP for mitochondrially synthesized ATP)
	Aim17	1.52	Unknown
	Pet9	1.52	Metabolite transport (major ADP/ATP carrier of the mitochondrial inner membrane; exchanges cytosolic ADP for mitochondrially synthesized ATP; also imports heme and ATP)
	Hem15	1.54	Metabolite synthesis (ferrochelatase; a mitochondrial inner membrane protein, catalyzes insertion of ferrous iron into protoporphyrin IX, the eighth and final step in the heme biosynthetic pathway)
	Sdh4	1.55	TCA cycle **and** ETC/respiration (membrane anchor subunit of succinate dehydrogenase; involved in coupling the oxidation of succinate to the transfer of electrons to ubiquinone as part of the TCA cycle and t
	Cbp3	1.55	Proteostasis **and** protein synthesis (mitochondrial protein required for assembly of cytochrome bc1 complex; forms a complex with Cbp6p that binds to mt ribosomes near the polypeptide tunnel exit and prom Cbp3p-Cbp6p complex also interacts with newly synthesized cytochrome b (Cobp) and Cbp4p to promote assembly of Cobp into the cytochrome bc1 complex)
	Tcb1	1.56	Lipid metabolism (lipid-binding protein)
	Idh2	1.56	TCA cycle (subunit of mitochondrial NAD(+)-dependent isocitrate dehydrogenase; complex catalyzes the oxidation of isocitrate to alpha-ketoglutarate in the TCA cycle)
	Mrpl3	1.56	Ribosome assembly (mitochondrial ribosomal protein)
	Erg9	1.58	Lipid metabolism (farnesyl-diphosphate farnesyl transferase (squalene synthase); joins two farnesyl pyrophosphate moieties to form squalene in the sterol biosynthesis pathway)
	Pos5	1.58	Metabolite synthesis (mitochondrial NADH kinase; phosphorylates NADH; also phosphorylates NAD(+) with lower specificity)
	Aim46	1.59	Unknown
	YFR045 W	1.59	Unknown
	Mrpl40	1.59	Ribosome assembly (mitochondrial ribosomal protein)
	Mba1	1.59	Protein synthesis (membrane-associated mitochondrial ribosome receptor; forms a complex with Mdm38p that may facilitate recruitment of mRNA-specific translational activators to ribosomes)
	Opy1	1.60	Unknown
	Mrpl11	1.60	Ribosome assembly (mitochondrial ribosomal protein)
	Cyt2	1.63	Proteostasis **and** ETC/respiration (cytochrome c1 heme lyase; involved in maturation of cytochrome c1, which is a subunit of the mitochondrial ubiquinol-cytochrome-c reductase; links heme covalently to apo
	Gcv2	1.64	Amino acid metabolism (P subunit of the mitochondrial glycine decarboxylase complex)
	Ilv1	1.65	Amino acid metabolism (threonine deaminase, catalyzes first step in isoleucine biosynthesis)
	Cir2	1.67	Unknown
	Leu4	1.67	Amino acid metabolism (α-isopropylmalate synthase; the main isozyme responsible for the first step in the leucine biosynthesis pathway)
	Prx1	1.71	Redox homeostasis (mitochondrial peroxiredoxin with thioredoxin peroxidase activity; has a role in reduction of hydroperoxides)
	Aco1	1.71	TCA cycle (aconitase)
	Rim1	1.73	mtDNA replication/recombination/repair (ssDNA-binding protein essential for mitochondrial genome maintenance; involved in mitochondrial DNA replication)
	Faa1	1.75	Lipid metabolism (long chain fatty acyl-CoA synthetase; activates fatty acids with a preference for C12:0-C16:0 chain lengths)
	Atg33	1.76	Mitophagy (mitochondrial mitophagy-specific protein; required primarily for mitophagy induced at post-log phase)
	Tim11	1.76	ATP synthesis (subunit e of mitochondrial F1F0-ATPase)
	Qcr10	1.78	ETC/respiration (subunit of the ubiquinol-cytochrome c oxidoreductase complex; this complex comprises part of the mitochondrial respiratory chain)
	Atp17	1.78	ATP synthesis (subunit f of the F0 sector of mitochondrial F1F0 ATP synthase)
	Atp5	1.78	ATP synthesis (subunit 5 of the stator stalk of mitochondrial F1F0 ATP synthase)
	Afg1	1.81	Proteostasis (protein that may act as a chaperone for cytochrome c oxidase subunits; may act as a chaperone in the degradation of misfolded or unassembled cytochrome c oxidase subunits; member of the AA
	Nat2	1.88	Unknown
	YNR040 W	1.88	Unknown
	Om45	1.92	Unknown
	Fcj1	1.92	Contact sites/cristae maintenance (component of the MICOS [Mitochondrial contact site and cristae organizing system] complex; MICOS is a mitochondrial inner membrane complex that extends into the inter maintenance of crista junctions, inner membrane architecture, and formation of contact sites to the outer membrane)
	Aim37	1.93	Contact sites/cristae maintenance (component of the MICOS [Mitochondrial contact site and cristae organizing system] complex; MICOS is a mitochondrial inner membrane complex that extends into the inter maintenance of crista junctions, inner membrane architecture, and formation of contact sites to the outer membrane)
	YKL133 C	1.96	Unknown
	Yhm2	1.97	Metabolite transport (citrate and oxoglutarate carrier protein; exports citrate from and imports oxoglutarate into the mitochondrion, causing net export of NADPH reducing equivalents)
	Sdh3	2.00	TCA cycle **and** ETC/respiration **and** Protein import (subunit of succinate dehydrogenase and of TIM22 translocase; functions as cytochrome b subunit of succinate dehydrogenase, which couples oxidation of ubiquinone as part of the TCA cycle and the mitochondrial respiratory chain)
	Ilv5	2.01	Amino acid metabolism **and** mtDNA replication/recombination/repair (acetohydroxyacid reductoisomerase and mtDNA binding protein; involved in branched-chain amino acid biosynthesis and maintenance o mitochondrial nucleoids)
	Shy1	2.01	Proteostasis (mitochondrial inner membrane protein required for complex IV assembly; associates with complex IV assembly intermediates and complex III/complex IV supercomplexes)
	Bna4	2.07	Metabolite synthesis (kynurenine 3-monooxygenase; required for the de novo biosynthesis of NAD from tryptophan via kynurenine)
	Phb1	2.09	Proteostasis (subunit of the prohibitin complex (Phb1p-Phb2p); prohibitin is an inner mitochondrial membrane chaperone that stabilizes newly synthesized proteins)
	Isc1	2.10	Lipid metabolism (inositol phosphosphingolipid phospholipase C; hydrolyzes complex sphingolipids to produce ceramide)
	Aim18	2.14	Unknown
	Ypt7	2.16	Mitochondria/vacuoles contact site (Rab family GTPase; localizes to sites of contact between the vacuole and mitochondria)
	Cyt1	2.17	ETC/respiration (cytochrome c1; component of the mitochondrial respiratory chain)
	She9	2.17	Unknown
	Tom20	2.19	Protein import (component of the TOM (translocase of outer membrane) complex; responsible for recognition and initial import steps for all mitochondrially directed proteins; acts as a receptor for incoming p
	Ymr31	2.25	TCA cycle (subunit of the mitochondrial α-ketoglutarate dehydrogenase)
	Atp10	2.26	Proteostasis **and** ATP synthesis (assembly factor for the F0 sector of mitochondrial F1F0 ATP synthase)
	Yim1	2.28	Unknown
	Rip1	2.28	ETC/respiration (ubiquinol-cytochrome-c reductase; a Rieske iron-sulfur protein of the mitochondrial cytochrome bc1 complex; transfers electrons from ubiquinol to cytochrome c1 during respiration)
	Fmp45	2.29	Unknown
	Ugo1	2.31	Mitochondrial fusion (outer membrane component of the mitochondrial fusion machinery)
	Mcr1	2.45	Lipid metabolism (mitochondrial NADH-cytochrome b5 reductase; involved in ergosterol biosynthesis)
	Cbp4	2.52	Proteostasis **and** ETC/respiration (mitochondrial protein required for assembly of cytochrome bc1 complex; interacts with the Cbp3p-Cbp6p complex and newly synthesized cytochrome b (Cobp) to promote a complex)
	Fsf1	2.62	Unknown
	Atp15	2.78	ATP synthesis (epsilon subunit of the F1 sector of mitochondrial F1F0 ATP synthase)
	Idp1	2.85	Amino acid metabolism (mitochondrial NADP-specific isocitrate dehydrogenase; catalyzes the oxidation of isocitrate to α-ketoglutarate; not required for mitochondrial respiration; diverts α-ketoglutarate to glu
	Mcp2	3.21	Lipid metabolism (mitochondrial protein involved in lipid homeostasis; non-essential gene which interacts genetically with MDM10, and other members of the ERMES complex)
	Psd1	3.24	Lipid metabolism (phosphatidylserine decarboxylase of the mitochondrial inner membrane; converts phosphatidylserine to phosphatidylethanolamine)
	Bcs1	3.27	Proteostasis **and** ETC/respiration (chaperone required for Complex III assembly; member of the AAA ATPase family; also required for assembly of the Qcr10p subunit)
	Mrpl35	3.27	Ribosome assembly (mitochondrial ribosomal protein)

	Tim17	4.10	Protein import (essential component of the TIM23 complex; with Tim23p, contributes to the architecture and function of the import channel; may link the import motor to the core translocase of the inner mitoc
	Oxa1	4.13	Proteostasis (mitochondrial inner membrane insertase; mediates the insertion of both mitochondrial- and nuclear-encoded proteins from the matrix into the inner membrane; also has a role in insertion of carrier
	Qcr8	5.10	ETC/respiration (subunit 8 of ubiquinol cytochrome-c reductase (Complex III); Complex III is a component of the mitochondrial inner membrane electron transport chain)
	Atp7	5.35	ATP synthesis (subunit d of the stator stalk of mitochondrial F1F0 ATP synthase)
	Pam18	9.63	Protein import (subunit of the import motor (PAM complex); the PAM complex is a component of the Translocase of the Inner Mitochondrial membrane (TIM23 complex); essential J-protein co-chaperone tha import)
4	Arg7	1.51	Amino acid metabolism (mitochondrial ornithine acetyltransferase; catalyzes the fifth step in arginine biosynthesis)
	Leu4	1.51	Amino acid metabolism (α-isopropylmalate synthase; the main isozyme responsible for the first step in the leucine biosynthesis pathway)
	Ggc1	1.51	Metabolite transport (mitochondrial GTP/GDP transporter; has a role in mitochondrial iron transport)
	Mrp1	1.52	Ribosome assembly (mitochondrial ribosomal protein)
	Tdh2	1.52	Carbohydrate metabolism (glyceraldehyde-3-phosphate dehydrogenase, isozyme 2; involved in glycolysis and gluconeogenesis)
	Pim1	1.54	Proteostasis (ATP-dependent Lon protease; involved in degradation of misfolded proteins in mitochondria)
	Aco2	1.55	TCA cycle (mitochondrial aconitase isozyme; similarity to Aco1, an aconitase required for the TCA cycle)
	Mrx10	1.58	Ribosome assembly (mitochondrial ribosomal protein)
	Cor1	1.59	ETC/respiration (core subunit of the ubiquinol-cytochrome c reductase complex; the ubiquinol-cytochrome c reductase complex (bc1 complex) is a component of the mitochondrial inner membrane electron tra
	Rim2	1.59	Metabolite transport (mitochondrial pyrimidine nucleotide transporter; imports pyrimidine nucleoside triphosphates and exports pyrimidine nucleoside monophosphates)
	Tcb1	1.60	Lipid metabolism (lipid-binding protein)
	Uip4	1.60	Unknown
	Mct1	1.60	Lipid metabolism (malonyl-CoA:ACP transferase; component of a type-II mitochondrial fatty acid synthase that produces intermediates for phospholipid remodeling)
	Odc2	1.62	Metabolite transport (mitochondrial inner membrane transporter; 2-oxodicarboxylate transporter, exports 2-oxoadipate and 2-oxoglutarate from the mitochondrial matrix to the cytosol)
	Mcp1	1.63	Lipid metabolism (mitochondrial protein involved in lipid homeostasis; interacts genetically with MDM10 and other members of the ERMES complex)
	Pdx1	1.63	Carbohydrate metabolism (E3-binding protein of the mitochondrial pyruvate dehydrogenase complex)
	Ssq1	1.64	Proteostasis (mitochondrial hsp70-type molecular chaperone; required for assembly of iron/sulfur clusters into proteins at a step after cluster synthesis)
	Ecm10	1.65	Protein import **and** Proteostasis (heat shock protein of the Hsp70 family; plays a role in protein translocation)
	Cem1	1.68	Lipid metabolism (mitochondrial beta-keto-acyl synthase; possible role in fatty acid synthesis)
	Yme2	1.69	mtDNA replication/recombination/repair (integral inner mitochondrial membrane protein; role in maintaining mitochondrial nucleoid structure and number)
	Pet9	1.69	Metabolite transport (major ADP/ATP carrier of the mitochondrial inner membrane; exchanges cytosolic ADP for mitochondrially synthesized ATP; also imports heme and ATP)
	Ilv1	1.72	Amino acid metabolism (threonine deaminase, catalyzes first step in isoleucine biosynthesis)
	Mdm38	1.72	Protein synthesis (forms a complex with Mba1p to facilitate recruitment of mRNA-specific translational activators to ribosomes)
	Phb2	1.73	Proteostasis (subunit of the prohibitin complex (Phb1p-Phb2p); prohibitin is an inner mitochondrial membrane chaperone that stabilizes newly synthesized proteins)
	Rmd9	1.73	Protein synthesis (mutant phenotype and genetic interactions suggest a role in delivering mt mRNAs to ribosomes; located on matrix face of the inner membrane and loosely associated with mitoribosomes)
	Isd11	1.73	Metabolite synthesis (cysteine desulfurase activator; required for mitochondrial iron-sulfur cluster biosynthesis)
	Lsc1	1.73	TCA cycle (α subunit of succinyl-CoA ligase; succinyl-CoA ligase is a mitochondrial enzyme of the TCA cycle that catalyzes the nucleotide-dependent conversion of succinyl-CoA to succinate)
	YGR266 W	1.74	Unknown
	Cox2	1.76	ETC/respiration (subunit II of cytochrome c oxidase (Complex IV); Complex IV is the terminal member of the mitochondrial inner membrane electron transport chain)
	Mrpl13	1.76	Ribosome assembly (mitochondrial ribosomal protein)
	Pdb1	1.76	Carbohydrate metabolism (E1 beta subunit of the pyruvate dehydrogenase (PDH) complex)
	Atp3	1.76	ATP synthesis (gamma subunit of the F1 sector of mitochondrial F1F0 ATP synthase)
	YDR061 W	1.77	Unknown
	Lat1	1.77	Carbohydrate metabolism (dihydrolipoamide acetyltransferase component (E2) of the pyruvate dehydrogenase complex)
	Atp7	1.79	ATP synthesis (subunit d of the stator stalk of mitochondrial F1F0 ATP synthase)
	Aim24	1.82	Unknown
	Hfd1	1.83	Metabolite synthesis **and** Lipid metabolism (dehydrogenase involved in ubiquinone and sphingolipid metabolism; oxidizes 4-hydroxybenzaldehyde into 4-hydroxybenzoic acid in ubiquinone biosynthesis; con sphingosine 1-phosphate breakdown pathway)
	Rip1	1.85	ETC/respiration (ubiquinol-cytochrome-c reductase; a Rieske iron-sulfur protein of the mitochondrial cytochrome bc1 complex; transfers electrons from ubiquinol to cytochrome c1 during respiration)
	Aim9	1.86	Unknown
	Msc6	1.87	Protein synthesis (multicopy suppressor of HER2 involved in mitochondrial translation)
	Cat2	1.88	Metabolite transport (carnitine acetyl-CoA transferase; transfers activated acetyl groups to carnitine to form acetylcarnitine which can be shuttled across membranes)
	Qcr2	1.89	ETC/respiration (subunit 2 of ubiquinol cytochrome-c reductase (Complex III), a component of the mitochondrial inner membrane electron transport chain)
	Put2	1.89	Amino acid metabolism (delta-1-pyrroline-5-carboxylate dehydrogenase; nuclear-encoded mitochondrial protein involved in utilization of proline as sole nitrogen source)
	Nde2	1.93	ETC/respiration (mitochondrial external NADH dehydrogenase; catalyzes the oxidation of cytosolic NADH; provides the cytosolic NADH to the mitochondrial respiratory chain)
	Mrx3	1.93	Unknown
	Nfu1	1.93	Proteostasis (protein involved in Fe-S cluster transfer to mitochondrial clients)
	Sdh1	1.94	TCA cycle **and** ETC/respiration (flavoprotein subunit of succinate dehydrogenase; couples the oxidation of succinate to the transfer of electrons to ubiquinone as part of the TCA cycle and the mitochondrial re
	Mir1	1.96	Metabolite transport (mitochondrial phosphate carrier; imports inorganic phosphate into mitochondria)
	Mae1	1.97	Carbohydrate metabolism **and** Amino acid metabolism (mitochondrial malic enzyme; catalyzes the oxidative decarboxylation of malate to pyruvate, which is a key intermediate in sugar metabolism and a prec
	Afg1	1.97	Proteostasis (protein that may act as a chaperone for cytochrome c oxidase subunits; may act as a chaperone in the degradation of misfolded or unassembled cytochrome c oxidase subunits; member of the AA
	YJL045 W	1.99	ETC/respiration (minor succinate dehydrogenase isozyme; participates in oxidation of succinate and transfer of electrons to ubiquinone)
	Tom40	1.99	Protein import (component of the TOM (translocase of outer membrane) complex; responsible for recognition and initial import steps for all mitochondrially directed proteins)
	Alo1	2.01	Redox homeostasis (D-Arabinono-1,4-lactone oxidase; catalyzes the final step in biosynthesis of dehydro-D-arabinono-1,4-lactone, which is protective against oxidative stress)
	Bat1	2.01	Amino acid metabolism (mitochondrial branched-chain amino acid (BCAA) aminotransferase involved in BCAA biosynthesis)
	Tom71	2.01	Protein import (component of the TOM (translocase of outer membrane) complex responsible for recognition and import of mitochondrially directed proteins)
	Cox20	2.03	Proteostasis (mitochondrial inner membrane protein; required for proteolytic processing of Cox2p and its assembly into cytochrome c oxidase)
	Etr1	2.05	Lipid metabolism (mitochondrial 2-enoyl thioester reductase; member of the medium chain dehydrogenase/reductase family; is involved in fatty acid synthesis)
	Mdm32	2.06	mtDNA replication/recombination/repair (mitochondrial inner membrane protein required for normal mitochondrial morphology and stability of mtDNA)
	Pet127	2.06	mRNA synthesis/processing (protein with a role in 5'-end processing of mitochondrial RNAs)
	Afg3	2.06	Proteostasis (Mitochondrial inner membrane m-AAA protease component; mediates degradation of misfolded or unassembled proteins; also required for correct assembly of mitochondrial enzyme complexes)
	Kgd1	2.07	TCA cycle (subunit of the mitochondrial alpha-ketoglutarate dehydrogenase complex; catalyzes a key step in the tricarboxylic acid (TCA) cycle, the oxidative decarboxylation of alpha-ketoglutarate to form su
	Cbp2	2.07	mRNA synthesis/processing (required for splicing of the group I intron bI5 of the COB pre-mRNA; nuclear-encoded mitochondrial protein that binds to the RNA to promote splicing)
	Ubp16	2.08	Proteostasis (deubiquitinating enzyme anchored to the outer mitochondrial membrane)
	Nuc1	2.09	mtDNA replication/recombination/repair (major mitochondrial nuclease; roles in mitochondrial recombination, apoptosis and maintenance of polyploidy; involved in fragmentation of genomic DNA during PN
	Lys4	2.09	Amino acid metabolism (homoaconitase; catalyzes the conversion of homocitrate to homoisocitrate, which is a step in the lysine biosynthesis pathway)
	Hsp60	2.10	Proteostasis (tetradecameric mitochondrial chaperonin; required for ATP-dependent folding of precursor polypeptides and complex assembly; prevents aggregation and mediates protein refolding after heat sho
	Ecm31	2.12	Metabolite synthesis (ketopantoate hydroxymethyltransferase; required for pantothenic acid and acetyl-CoA biosynthesis)
	Cyt1	2.13	ETC/respiration (cytochrome c1; component of the mitochondrial respiratory chain)
	Cox13	2.17	ETC/respiration **and** Proteostasis (subunit VIa of cytochrome c oxidase; present in a subclass of cytochrome c oxidase complexes that may have a role in minimizing generation of reactive oxygen species; not but may modulate activity in response to ATP; required for assembly of Rcf2p into cytochrome c oxidase - cytochrome bc1 supercomplexes)
	Ysa1	2.19	Metabolite synthesis (nudix hydrolase family member with ADP-ribose pyrophosphatase activity; shown to metabolize O-acetyl-ADP-ribose to AMP and acetylated ribose 5'-phosphate)
	Rsm10	2.19	Ribosome assembly (mitochondrial ribosomal protein)
	Atp1	2.20	ATP synthesis (α subunit of the F1 sector of mitochondrial F1F0 ATP synthase)
	Atp2	2.20	ATP synthesis (β subunit of the F1 sector of mitochondrial F1F0 ATP synthase)
	YNL200 C	2.21	Metabolite synthesis (NADHX epimerase; catalyzes isomerization of (R)- and (S)-NADHX)
	YBL059 W	2.21	Unknown
	Ysc83	2.23	Unknown
	Cpr1	2.23	Proteostasis (cytoplasmic peptidyl-prolyl cis-trans isomerase (cyclophilin); catalyzes the cis-trans isomerization of peptide bonds N-terminal to proline residues)
	Aim19	2.26	Unknown
	Yta12	2.26	Proteostasis (mitochondrial inner membrane m-AAA protease component; mediates degradation of misfolded or unassembled proteins; also required for correct assembly of mitochondrial enzyme complexes)

	Ilv3	2.27	Amino acid metabolism (dihydroxyacid dehydratase; catalyzes third step in the common pathway leading to biosynthesis of branched-chain amino acids)
	YDL183 C	2.28	Metabolite transport (protein that may form an active mitochondrial K+/H+ exchanger system)
	Aim45	2.29	Unknown
	Ssc1	2.33	Protein import **and** Proteostasis (Hsp70 family ATPase; constituent of the import motor component of the Translocase of the Inner Mitochondrial membrane (TIM23 complex); involved in protein translocatio
	Mgm101	2.34	mtDNA replication/recombination/repair (Protein with a role in mitochondrial DNA recombinational repair; also involved in interstrand cross-link repair; component of the mitochondrial nucleoid, required for mitochondrial genome maintenance)
	Hem15	2.36	Metabolite synthesis (ferrochelatase; a mitochondrial inner membrane protein, catalyzes insertion of ferrous iron into protoporphyrin IX, the eighth and final step in the heme biosynthetic pathway)
	Por1	2.44	Metabolite transport (mitochondrial porin (voltage-dependent anion channel); outer membrane protein required for maintenance of mitochondrial osmotic stability and mitochondrial membrane permeability)
	Mdh1	2.45	TCA cycle (mitochondrial malate dehydrogenase; catalyzes interconversion of malate and oxaloacetate; involved in the tricarboxylic acid (TCA) cycle)
	Cox5A	2.52	ETC/respiration (Subunit Va of cytochrome c oxidase, the terminal member of the mitochondrial inner membrane electron transport chain)
	Yju3	2.55	Lipid metabolism (monoglyceride lipase)
	Tom70	2.59	Protein import (component of the TOM (translocase of outer membrane) complex; involved in the recognition and initial import steps for all mitochondrially directed proteins)
	Num1	2.60	Mitochondria/ER contact sites (component of the mitochondria-ER-cortex-anchor (MECA); required for the association of mitochondria with the cell cortex; interacts with Mdm36p to link the ER and mitocho
	Nde1	2.61	ETC/respiration (mitochondrial external NADH dehydrogenase; provides cytosolic NADH to the mitochondrial respiratory chain)
	Mrps9	2.72	Ribosome assembly (mitochondrial ribosomal protein)
	Adh3	2.74	Carbohydrate metabolism (mitochondrial NAD-dependent alcohol dehydrogenase involved in NADH oxidation)
	Mcr1	2.78	Lipid metabolism (mitochondrial NADH-cytochrome b5 reductase; involved in ergosterol biosynthesis)
	Atg33	2.78	Mitophagy (mitochondrial mitophagy-specific protein; required primarily for mitophagy induced at post-log phase)
	Tim50	2.79	Protein import (acts as receptor for the translocase of the inner mitochondrial membrane (TIM23) complex guiding incoming precursors from the TOM complex)
	Cpr3	2.85	Proteostasis (mitochondrial peptidyl-prolyl cis-trans isomerase (cyclophilin); catalyzes the cis-trans isomerization of peptide bonds N-terminal to proline residues)
	Mgr1	2.87	Proteostasis (subunit of the mitochondrial (mt) i-AAA protease supercomplex; i-AAA degrades misfolded mitochondrial proteins)
	Fmp30	2.87	Lipid metabolism (involved in maintaining normal cardiolipin levels)
	Pdh1	2.88	ETC/respiration (2-methylcitrate dehydratase; participates in respiration)
	Faa1	2.88	Lipid metabolism (long chain fatty acyl-CoA synthetase; activates fatty acids with a preference for C12:0-C16:0 chain lengths)
	Ndi1	2.95	ETC/respiration (NADH:ubiquinone oxidoreductase; transfers electrons from NADH to ubiquinone in respiratory chain)
	Lys12	3.01	Amino acid metabolism (homo-isocitrate dehydrogenase; an NAD-linked mitochondrial enzyme required for the fourth step in the biosynthesis of lysine)
	Pil1	3.03	Unknown
	Mge1	3.04	Proteostasis **and** Protein import (mitochondrial matrix co-chaperone; nucleotide release factor for Ssc1p in protein translocation and folding; also acts as co-chaperone for Ssq1p in folding of Fe-S cluster prote
	Agx1	3.04	Amino acid metabolism (alanine:glyoxylate aminotransferase (AGT); catalyzes the synthesis of glycine from glyoxylate)
	Tuf1	3.21	Protein synthesis **and** mtDNA replication/recombination/repair (multicopy suppressor of HER2 involved in mitochondrial translation)
	Cox4	3.27	ETC/respiration (subunit IV of cytochrome c oxidase; the terminal member of the mitochondrial inner membrane electron transport chain)
	Sur7	3.30	Unknown
	Aim32	3.35	Unknown
	Mdh3	3.36	Carbohydrate metabolism (malate dehydrogenase; catalyzes interconversion of malate and oxaloacetate; involved in the glyoxylate cycle)
	Cox1	3.44	ETC/respiration (subunit I of cytochrome c oxidase (Complex IV); Complex IV is the terminal member of the mitochondrial inner membrane electron transport chain)
	Her2	3.52	Protein synthesis (glutaminyl-tRNA synthase)
	Ach1	3.55	Carbohydrate metabolism (protein with CoA transferase activity; particularly for CoASH transfer from succinyl-CoA to acetate)
	Dld1	3.99	Carbohydrate metabolism (major mitochondrial D-lactate dehydrogenase; oxidizes D-lactate to pyruvate)
	Pam18	4.10	Protein import (subunit of the import motor (PAM complex); the PAM complex is a component of the Translocase of the Inner Mitochondrial membrane (TIM23 complex); essential J-protein co-chaperone tha import)
	Idp1	4.24	Amino acid metabolism (mitochondrial NADP-specific isocitrate dehydrogenase; catalyzes the oxidation of isocitrate to α-ketoglutarate; not required for mitochondrial respiration; diverts α-ketoglutarate to gl
	Aim46	4.71	Unknown
	Om45	9.15	Unknown
7	Sdh2	1.53	ETC/respiration **and** TCA cycle (iron-sulfur protein subunit of succinate dehydrogenase; the complex couples the oxidation of succinate to the transfer of electrons to ubiquinone as part of the TCA cycle and t
	Sls1	1.55	mRNA synthesis/processing (mitochondrial membrane protein; coordinates expression of mitochondrially-encoded genes)
	Arg7	1.55	Amino acid metabolism (mitochondrial ornithine acetyltransferase; catalyzes the fifth step in arginine biosynthesis)
	Mst1	1.55	Protein synthesis (mitochondrial threonyl-tRNA synthetase)
	Glr1	1.55	Redox homeostasis (glutathione oxidoreductase; converts oxidized glutathione to reduced glutathione)
	Ald6	1.56	Carbohydrate metabolism (aldehyde dehydrogenase; required for conversion of acetaldehyde to acetate; locates to the mitochondrial outer surface upon oxidative stress)
	Atp4	1.58	ATP synthesis (subunit b of the stator stalk of mitochondrial F1F0 ATP synthase)
	Ysc83	1.58	Unknown
	Mrp13	1.59	Ribosome assembly (mitochondrial ribosomal protein)
	Fmp41	1.61	Unknown
	Mrx3	1.61	Unknown
	Tim21	1.61	Protein import (component of the TIM23 complex; interacts with the Translocase of the Outer Mitochondrial membrane (TOM complex); may regulate the Translocase of the Inner Mitochondrial membrane (T
	Ort1	1.62	Metabolite transport **and** Amino acid metabolism (ornithine transporter of the mitochondrial inner membrane; exports ornithine from mitochondria as part of arginine biosynthesis)
	Hem15	1.63	Metabolite synthesis (ferrochelatase; a mitochondrial inner membrane protein, catalyzes insertion of ferrous iron into protoporphyrin IX, the eighth and final step in the heme biosynthetic pathway)
	Ypt7	1.63	Mitochondria/vacuoles contact site (Rab family GTPase; localizes to sites of contact between the vacuole and mitochondria)
	Ndi1	1.67	ETC/respiration (NADH:ubiquinone oxidoreductase; transfers electrons from NADH to ubiquinone in respiratory chain)
	Mss51	1.67	Protein synthesis **and** Proteostasis (specific translational activator for the mitochondrial COX1 mRNA; influences both COX1 mRNA translation and Cox1p assembly into cytochrome c oxidase)
	Dic1	1.69	Metabolite transport (mitochondrial dicarboxylate carrier; integral membrane protein, catalyzes a dicarboxylate-phosphate exchange across the inner mitochondrial membrane, transports cytoplasmic dicarboxy
	Pet9	1.70	Metabolite transport (major ADP/ATP carrier of the mitochondrial inner membrane; exchanges cytosolic ADP for mitochondrially synthesized ATP; also imports heme and ATP)
	Tom40	1.73	Protein import (component of the TOM (translocase of outer membrane) complex; responsible for recognition and initial import steps for all mitochondrially directed proteins)
	Rim1	1.73	mtDNA replication/recombination/repair (ssDNA-binding protein essential for mitochondrial genome maintenance; involved in mitochondrial DNA replication)
	Yme2	1.74	mtDNA replication/recombination/repair (role in maintaining mitochondrial nucleoid structure and number; mutants exhibit an increased rate of mitochondrial DNA escape)
	YGR266 W	1.74	Unknown
	Tom71	1.76	Protein import (component of the TOM (translocase of outer membrane) complex responsible for recognition and import of mitochondrially directed proteins)
	Atp20	1.76	ATP synthesis (subunit g of the mitochondrial F1F0 ATP synthase)
	Coq8	1.77	Metabolite synthesis (ATPase required for ubiquinone biosynthesis and respiratory growth; maintains levels of ubiquinone biosynthetic proteins; binds to ubiquinone biosynthesis intermediates)
	Qcr10	1.78	ETC/respiration (subunit of the ubiquinol-cytochrome c oxidoreductase complex; this complex comprises part of the mitochondrial respiratory chain)
	Atp5	1.78	ATP synthesis (subunit 5 of the stator stalk of mitochondrial F1F0 ATP synthase)
	Fmp30	1.83	Lipid metabolism (involved in maintaining normal cardiolipin levels)
	Dld1	1.85	Carbohydrate metabolism (major mitochondrial D-lactate dehydrogenase; oxidizes D-lactate to pyruvate)
	Erg6	1.90	Lipid metabolism (delta(24)-sterol C-methyltransferase; converts zymosterol to fecosterol in the ergosterol biosynthetic pathway by methylating position C-24)
	Idp1	1.92	Amino acid metabolism (mitochondrial NADP-specific isocitrate dehydrogenase; catalyzes the oxidation of isocitrate to α-ketoglutarate; not required for mitochondrial respiration; diverts α-ketoglutarate to gl
	Rrf1	1.93	Protein synthesis **and** ETC/respiration (mitochondrial ribosome recycling factor; essential for mitochondrial protein synthesis and for the maintenance of the respiratory function of mitochondria)
	Ayr1	1.94	Lipid metabolism (bifunctional triacylglycerol lipase and 1-acyl DHAP reductase; NADPH-dependent 1-acyl dihydroxyacetone phosphate reductase involved in phosphatidic acid biosynthesis)
	Osm1	1.97	Carbohydrate metabolism **and** Redox homeostasis (fumarate reductase, catalyzes the reduction of fumarate to succinate; required for the reoxidation of intracellular NADH under anaerobic conditions)
	Cbp6	2.00	Protein synthesis **and** Proteostasis (required for translation of the COB mRNA; forms a complex with Cbp3p that binds to mt ribosomes near the polypeptide tunnel exit and promotes efficient translation of the interacts with newly synthesized cytochrome b (Cobp) and Cbp4p to promote assembly of Cobp into the cytochrome bc1 complex)
	Qcr7	2.04	ETC/respiration (subunit 7 of ubiquinol cytochrome-c reductase (Complex III); Complex III is a component of the mitochondrial inner membrane electron transport chain)
	Gpd2	2.09	Carbohydrate metabolism (NAD-dependent glycerol 3-phosphate dehydrogenase involved in glycerol biosynthesis)
	Mrp1	2.12	Ribosome assembly (mitochondrial ribosomal protein)
	Abf2	2.13	mtDNA replication/recombination/repair (mitochondrial DNA-binding protein; involved in mitochondrial DNA replication and recombination)
	Fzo1	2.14	Mitochondrial fusion (mitofusin; integral membrane protein involved in mitochondrial outer membrane tethering and fusion)
	Uip4	2.15	Unknown
	Lsb3	2.19	Unknown
	Yro2	2.19	Unknown
	Isc1	2.21	Lipid metabolism (inositol phosphosphingolipid phospholipase C; hydrolyzes complex sphingolipids to produce ceramide)

	Pnt1	2.26	Proteostasis (mitochondrial integral inner membrane protein; involved in membrane insertion of C-terminus of Cox2p)
	Aim19	2.26	Unknown
	Yim1	2.28	Unknown
	Aim37	2.31	Contact sites/cristae maintenance (component of the MICOS [Mitochondrial contact site and cristae organizing system] complex; MICOS is a mitochondrial inner membrane complex that extends into the inter maintenance of crista junctions, inner membrane architecture, and formation of contact sites to the outer membrane)
	Pam18	2.33	Protein import (subunit of the import motor (PAM complex); the PAM complex is a component of the Translocase of the Inner Mitochondrial membrane (TIM23 complex); essential J-protein co-chaperone tha import)
	Cbp3	2.35	Proteostasis **and** protein synthesis (mitochondrial protein required for assembly of cytochrome bc1 complex; forms a complex with Cbp6p that binds to mt ribosomes near the polypeptide tunnel exit and prom Cbp3p-Cbp6p complex also interacts with newly synthesized cytochrome b (Cobp) and Cbp4p to promote assembly of Cobp into the cytochrome bc1 complex)
	Msc1	2.37	Unknown
	Atp14	2.39	ATP synthesis (subunit h of the F0 sector of mitochondrial F1F0 ATP synthase)
	Cox13	2.46	ETC/respiration **and** Proteostasis (subunit VIa of cytochrome c oxidase; present in a subclass of cytochrome c oxidase complexes that may have a role in minimizing generation of reactive oxygen species; not but may modulate activity in response to ATP; required for assembly of Rcf2p into cytochrome c oxidase - cytochrome bc1 supercomplexes)
	Fmp43	2.47	Metabolite transport (subunit of the mitochondrial pyruvate carrier (MPC); MPC localizes to the mitochondrial inner membrane and mediates pyruvate uptake)
	Qcr8	2.58	ETC/respiration (subunit 8 of ubiquinol cytochrome-c reductase (Complex III); Complex III is a component of the mitochondrial inner membrane electron transport chain)
	YPL109C	2.65	Unknown
	Mcx1	2.78	Proteostasis (non-proteolytic ATPase of the AAA family; stimulates incorporation of the pyridoxal phosphate cofactor into Hem1p (5-aminolevulinic acid synthase))
	Cox11	3.04	Proteostasis (protein required for delivery of copper to Cox1p)
	Acs1	3.06	Carbohydrate metabolism (acetyl-coA synthetase isoform)
	Her1	3.06	Unknown
	Sco1	3.07	Proteostasis **and** ETC/respiration (copper-binding protein of mitochondrial inner membrane; required for cytochrome c oxidase activity and respiration; may function to deliver copper to cytochrome c oxidase
	Bna4	3.24	Metabolite synthesis (kynurenine 3-monooxygenase; required for the de novo biosynthesis of NAD from tryptophan via kynurenine)
	Idp3	3.34	Amino acid metabolism (NADP-dependent isocitrate dehydrogenase; catalyzes oxidation of isocitrate to alpha-ketoglutarate with the formation of NADP(H+); involved in glutamate biosynthesis)
	Gtt1	3.39	Redox homeostasis (glutathione S-transferase; involved in glutathione-glutaredoxin redox reactions)
	Aim46	3.80	Unknown
	Atp7	3.95	ATP synthesis (subunit d of the stator stalk of mitochondrial F1F0 ATP synthase)
	Tim23	4.03	Protein import (essential component of the TIM23 [translocase of the inner mitochondrial membrane] complex; involved in protein import into mitochondrial matrix and inner membrane)
	YLR419 W	4.31	Unknown
	Cbp4	4.83	Proteostasis (required for assembly of cytochrome bc1 complex; interacts with the Cbp3p-Cbp6p complex and newly synthesized cytochrome b (Cobp) to promote assembly of Cobp into the cytochrome bc1 c
	Tom20	5.31	Protein import (component of the TOM (translocase of outer membrane) complex; responsible for recognition and initial import steps for all mitochondrially directed proteins; acts as a receptor for incoming p
	Efr3	5.51	Unknown
	Grx2	5.62	Redox homeostasis (glutaredoxin; thioltransferase, glutathione-dependent disulfide oxidoreductase involved in maintaining redox state of target proteins, also exhibits glutathione peroxidase activity)
	Pet10	8.16	Unknown
